# An integrative DNA barcoding framework of ladybird beetles (Coleoptera: Coccinellidae)

**DOI:** 10.1038/s41598-020-66874-1

**Published:** 2020-06-22

**Authors:** Weidong Huang, Xiufeng Xie, Lizhi Huo, Xinyue Liang, Xingmin Wang, Xiaosheng Chen

**Affiliations:** 1 Guangdong Key Laboratory for Innovative Development and Utilization of Forest Plant Germplasm; Department of Forest Protection, College of Forestry and Landscape Architecture, South China Agricultural University, Guangzhou, 510640 China; 2 Key Laboratory of Bio-Pesticide Innovation and Application, Guangdong Province; Engineering Technology Research Center of Agricultural Pest Biocontrol, Guangdong Province, Engineering Research Center of Biological Control, Ministry of Education, Guangzhou, 510640 China; 3 Guangdong Agriculture Industry Business Polytechnic College, Guangzhou, 510507 China; 4 Guangzhou Institute of Forestry and Landscape Architecture, Guangzhou, 510405 China

**Keywords:** Molecular biology, Zoology

## Abstract

Even though ladybirds are well known as economically important biological control agents, an integrative framework of DNA barcoding research was not available for the family so far. We designed and present a set of efficient mini-barcoding primers to recover full DNA barcoding sequences for Coccinellidae, even for specimens collected 40 years ago. Based on these mini-barcoding primers, we obtained 104 full DNA barcode sequences for 104 species of Coccinellidae, in which 101 barcodes were newly reported for the first time. We also downloaded 870 COI barcode sequences (658 bp) from GenBank and BOLD database, belonging to 108 species within 46 genera, to assess the optimum genetic distance threshold and compare four methods of species delimitation (GMYC, bPTP, BIN and ABGD) to determine the most accurate approach for the family. The results suggested the existence of a ‘barcode gap’ and that 3% is likely an appropriate genetic distance threshold to delimit species of Coccinellidae using DNA barcodes. Species delimitation analyses confirm ABGD as an accurate and efficient approach, more suitable than the other three methods. Our research provides an integrative framework for DNA barcoding and descriptions of new taxa in Coccinellidae. Our results enrich DNA barcoding public reference libraries, including data for Chinese coccinellids. This will facilitate taxonomic identification and biodiversity monitoring of ladybirds using metabarcoding.

## Introduction

As species are a fundamental biological category, accurately identifying them is an essential premise of biological studies. Tautz *et al*.^1^ proposed DNA sequences as a species identification system for the first time. Subsequently, the 5’ end of the mitochondrial cytochrome c oxidase subunit I gene (COI) has been suggested as a standardized DNA “barcode” for identifying species of all groups of animals, launching the DNA barcoding technology^2^. The success of barcode identification based upon genetic distances ultimately depended on differences between intra- and interspecific divergences^2–4^. DNA barcoding using the 658 bp 5’ region of mtCOI DNA sequence as a tool, has turned out as very efficient and reliable for identifying specimens of unknown origin and taxonomic status, and also for the identification of different developmental stages. The approximately 1600 base-pairs comprise a range of different functional domains showing heterogenous substitution patterns^5,6^. In addition to its utility for distinguishing known species, the COI region has also been found suitable for revealing cryptic species^7–11^, and biogeographic and phylogeographic patterns, and also species level phylogenetic relationships^12–14^.

The family Coccinellidae is placed in the superfamily Coccinelloidea within the Coleopteran suborder Polyphaga^15,16^. Coccinellids are well known as economically important biological control agents, but this family is ecologically and morphologically very diverse. It comprises about 490 genera and nearly 6000 described species worldwide^17^. Due to relatively small body size and similar elytral shapes and color patterns (particularly in the genera *Scymnus*, *Sasajiscymnus*, *Nephus*, and *Stethorus* with body lengths of 0.50 mm–2.00 mm), most of the species are very difficult to identify^18^. Therefore, using DNA barcode identification appears highly desirable. Moreover, the usefulness of DNA barcoding for distinguishing and diagnosing coccinellid species has been proved in previous studies^19,20^. Until now, there are 7,845 published records of coccinellid DNA barcodes from 46 countries and 37 institutions, comprising 529 BINs (Barcoding Index Numbers) in BOLD (Barcoding of Library Dataset) database system (http://www.barcodinglife.org). However, there is still a lack of reference data for such a large group of beetles. It is apparent that a comprehensive database is urgently required, and further barcoding is critical to improve the taxonomic resolution.

Collecting fresh specimens is expensive, time-consuming and often insufficient to ensure a wide coverage of species. Therefore, it is often necessary to use museum specimens for DNA extraction and target band amplification. However, insect material of natural history collections is often pinned without further preservation treatment^21^. The soft tissue soon dries out and decomposes, resulting in the fragmentation of DNA and negatively affecting the amplification success. The damaged DNA is broken down into small fragments of a few hundred base pairs^22^. Consequently, amplification with standard DNA barcoding primers for the 658 bp COI region is not possible. To solve this problem, Hajibabaei *et al*.^23^ and Meusnier *et al*.^24^ developed mini-barcode, which means using a region of about 100–150 bp to replaces the standard DNA barcoding procedure for identifying species. The mini-barcode approach proved successful for this purpose, even using museum specimens. In addition, Van Houdt *et al*.^25^ recovered full DNA barcodes by continuously amplifying mini-barcodes. As PCR amplification success rate is related to the length of the target band, the amplification of a sequence of about 200 bp is easier than amplifying standard DNA barcodes with 658 bp.

In the present study, we used Van Houdt’s^25^ approach to use universal mini-barcoding primer sets that allowed to reconstruct full DNA barcodes for Diptera, and also a new design with a set of universal primers for recovering the standard DNA barcodes for museum specimens of ladybird beetles. Our specific objectives are (1) develop a set of universal primers to increase the amplification efficiency for museum specimens of Coccinellidae; (2) contribute DNA barcodes for morphologically identified coccinellid species without previous DNA barcoding records. Moreover, we combined GenBank with BOLD published data to (3) screen out the optimum genetic distance threshold to the identification of ladybird or other beetles. As the genetic distance threshold is taxon specific^26^, the threshold for Coccinellidae was hitherto unknown. The choice of the analytical methods for DNA barcoding data and species delimitation methods is obviously important. Therefore we compared and analyzed Generalized Mixed Yule Coalescent (GMYC)^12^, Bayesian Poisson tree processes (bPTP)^27^, Automatic Barcode Gap Discovery (ABGD)^28^, Barcode Index Number (BIN)^29^ to (4) find out which approach is more accurate for delimiting species of Coccinellidae and suitable for describing new taxa.

## Results

### Amplification efficiency of mini-barcoding

**Figure 1:**
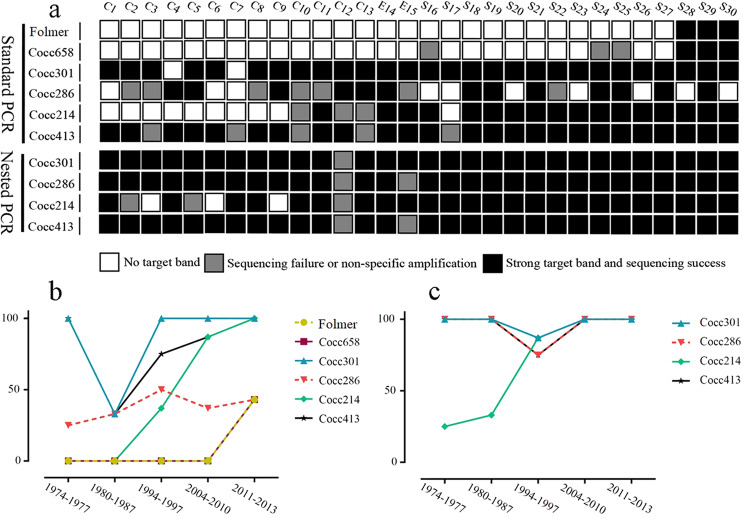
Amplification efficiency of new design primers. (**a**) Comparison amplification efficiency between standard polymerase chain reaction (PCR) and nested PCR for each primer pair; (**b**) standard PCR success rate of each pair of primers of different age classes; (**c**) nested PCR success rate of each pair of primers of different age classes.

The universal LCO1490/HCO2198 primer pair used to amplify the DNA barcoding region resulted in an extremely low success rate. Only the sequence of 3 specimens collected in 2013 could be amplified successfully. Similar results were obtained with our new design primer pair of WDF/XSR (Fig. 1a). The consequences of these PCR experiments are summarized in Fig. 1. For agarose electrophoretic images corresponding with the results see Supplementary Fig. S1.

We separately amplified four mini-barcoding fragments using both standard PCR and nested PCR methods. The PCR success rate was 93% and 83% for Cocc301 and Cocc413 markers using standard PCR, respectively, while PCR success rate was 97% and 93% for Cocc301 and Cocc413 markers, respectively, using nested PCR. The Amplification success rate of Cocc301 and Cocc413 in the nested PCR was slightly higher than in the standard PCR. However, the Cocc286 and Cocc214 markers resulted in a relatively low success rate of 43% and 57%, respectively, using standard PCR method, especially for samples collected before 1987 (Fig. 1b). Surprisingly, the PCR success rate with nested PCR was much higher, 93% for Cocc286 and 80% for Cocc214 markers (Fig. 1c). Overall, it is much more efficient to use mini-barcoding primers to obtain the target band, than to use LCO1490/HCO2198 and WDF/XSR.

### Screening the optimum genetic distance threshold of Coccinellidae

**Figure 2:**
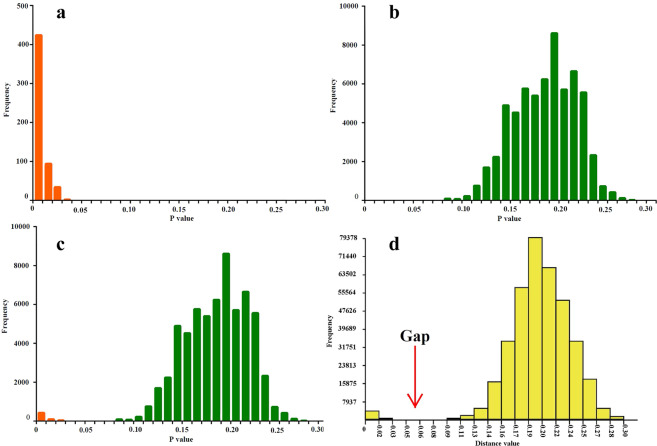
Distribution of pairwise genetic divergences estimated from DNA barcodes for the 870 aligned sequences of Coccinellidae based on the K2P model. (**a**) Intraspecific distances; (**b**) interspecific distances; (**c**) combined intra- with interspecific distances. (**d**) Histogram of pairwise K2P distances generated from ABGD online.

**Figure 3:**
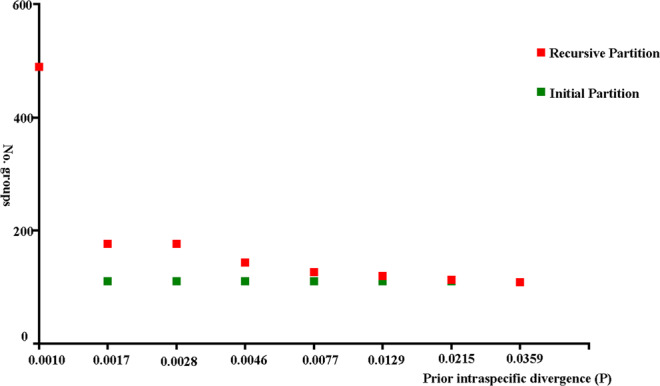
The automatic partition results of 870 aligned sequences of Coccinellidae with ABGD.

The complete dataset of 870 barcodes comprised 658 bp, which were belonging to 108 species within 46 genera. In total, 354 variable sites were identified, 340 of which were parsimony informative. Among these species, *Hippodamia notata* has the largest number of sequences, up to 20 barcode sequences, while *Mulasntina picata* has only one barcode sequence. The average barcode sequence is 8 for each species (Table S3). Intraspecific divergences ranged from 0 to 3.1% and the average intraspecific genetic distance was 0.6% (standard deviation is 0.007). A maximum intraspecific divergence of 3.1% was found in *Tytthaspis sedecimpunctata* (Supplementary Table S4; Fig. 2a). The minimum interspecific genetic divergence was 10% between *Scymnus ningshanensis* and *Scymnus sinuanodulus*, and the maximum interspecific genetic divergence was 29.1% between *Coleomegilla maculata* and *Stethorus punctillum*, as well as the average interspecific genetic distance was 20.7% (standard deviation is 0.031) (Supplementary Table S4; Fig. 2b). The results confirm that the interspecific divergence distance is distinctly higher than the intraspecific divergence distance, and that consequently a clear DNA barcodes gap is present (Fig. 2c). In addition, the results obtained with ABGD analysis are conform with the results based on MEGA (Fig. 2d). Both methods support the presence of DNA barcode gaps. In ABGD, both initial partition and recursive partition were employed to partition the dataset. Results showed that the initial partition is more stable, with 870 sequences divided into 110–121 putative species based on the different value of *P*. In contrast to this, the recursive partition displayed large undulation, and apparently overestimated the number of species (Fig. 3). The results obtained with MEGA and ABGD suggest that 3% is likely a suitable genetic distance threshold to delimit species of Coccinellidae using DNA barcodes.

### Species delimitation

Four species delimitation methods were employed to evaluate which one is most consistent with a morphology-based concept. Depending on the employed method, the number of putative species ranged from 110 to 223. ABGD analyses suggested the smallest numbers of species, whereas more putative species were obtained with GMYC, bPTP and BIN.

**Figure 4:**
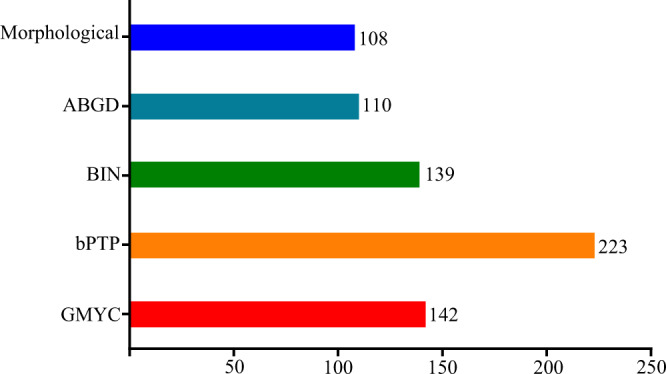
Compare the number of putative species between four species delimitation methods and morphological identification.

The ABDG analysis with a 1.3%–3.6% maximum intra-specific divergence yielded 110–121 putative species, which was close to the numbers of the species based on morphology (Fig. 4). In BOLD, 870 COI barcodes were assigned to 139 BINs, 114 BINs of them with one record, and 25 with two or more records. For instance, *Adalia bipunctata*, *Calvia quatuordecimguttata*, *Coccinella quinquepunctata*, *Psyllobora vigintimaculata* and *Microweisea misella*, each of them was assigned to three BINs, but *Scymnus camptodromus* to four. *Hippodamia caseyi* and *Hippodamia convergens* (BOLD: AAH3293) were the only case of two morphology-based species assigned to a single BIN.

Using GMYC with single-threshold calculations yielded 142 lineages, and very similar results with BIN. Surprisingly, the bPTP method yielded 223 putative species and the results twice the number of identified morphospecies. The bPTP species delimitation results were significantly more than GMYC, ABGD and BIN.

## Discussion

### Amplification efficiency of mini-barcoding

Apparently, the amplification success rate and efficiency of mini-barcoding were much higher compared to the full DNA barcode^23–25^. The observed pattern is consistent with DNA fragmentation models performed by Zimmermann *et al*.^22^, which predict a fast initial drop in average DNA fragment size in the first 5 years followed by a more gradual change. This also illustrates that DNA increasingly breaks down into smaller fragments with time^30^, and that small PCR fragments more easily amplify than relatively long PCR fragments^23,25^.

Nested PCR, a modification of standard PCR, has shown to be an extremely sensitive and specific method for amplifying target sequences^31^. In this study, we also employed this PCR strategy to amplify four mini-barcoding markers. Our results show that the amplification efficiency of nested PCR is obviously higher than that of standard PCR, especially for the Cocc286 and Cocc214 markers (Fig. 1a,c). The PCR success rate of Cocc286 was 93% and 80% for Cocc214 with the nested PCR method, but only 43% and 57% with standard PCR methods. In summary, the presented four mini-barcoding markers and protocols will make more coccinellid collection material suitable for use in a DNA barcoding context.

### The optimum genetic distance threshold for Coccinellidae

A characteristic of typical barcode data is the ‘barcode gap’ between intraspecific diversity and interspecific diversity^28^. However, this is not a general feature found in all groups. Zhou *et al*.^32^ pointed out the absence of the gap in Ephemeroptera and Trichoptera, due to species complexes with recent diversification according to their interpretation. Similarly, low interspecific divergences were observed in butterflies of the genus *Agrodiaetus*, resulting from a rapid divergence of species during a radiation accompanied by minimal divergence in mtDNA^33^. In our analyses, both methods confirmed the presence of a ‘barcode gap’ (Fig. 2c,d), indicating that DNA barcodes can be used to delineate coccinellid species. Greenstone *et al*.^19^, who analyzed the haplotype variation in North American agroecosystem ladybird beetles using DNA barcodes, also supported the general utility of this approach for distinguishing and diagnosing coccinellid species. However, no specific value is given in that study for a suitable genetic distance threshold for species delimitation in this family.

Even though several attempts have been made to establish a standard limit between intra- and interspecific divergence, none of them can be used for a wide range of groups. Due to variable population sizes and time of divergence in different species, defining such a threshold is a problematic issue and somewhat arbitrary^34^. In addition, using a fixed empirical threshold to delimit species may lead to overestimating species diversity in a genetically highly differentiated population^35^. Meyer and Paulay^36^ also found that the use of a single threshold is particularly problematic for closely related species in a comprehensive sampling. The Kimura 2 Parameter (K2P) genetic distance analysis showed that intraspecific divergences range from 0 to 3.1%, and the average intraspecific genetic distance was 0.6%. The ABGD analysis we conducted showed that the initial partition is more stable, with 870 sequences divided into 110–121 putative species based on different prior *P*. In contrast to this, the recursive partition showed a large undulation, and yielded an overestimation of species numbers. However, both approaches reached the same partition when the prior maximum divergence of *P* was 0.0359, dividing 870 sequences into 110 putative species. The reliability of the ABGD method was confirmed by Puillandre *et al*.^37^ and Ratnasingham and Hebert^29^, who pointed out that recursive partition is unstable and prone to excessive partitioning. Our results confirm that initial partition results are more stable and more consistent with morphospecies. Taking into consideration what we discussed above, we propose that 3% is a useful genetic distance threshold to delimitate ladybird beetles using DNA barcodes.

### Species delimitation

The rapidly increasing rate of extinction coupled with the magnitude of unknown biodiversity requires accurate species delimitation methods^29^. Given this situation, it is evident that new methods are needed for efficient biodiversity assessments and for in developing a sound species-level taxonomy^38^. Analysing DNA barcode sequences with varying techniques for cluster recognition provides an efficient approach for identifying putative species^39^. In the present study, GMYC and BIN methods yielded similar results (Fig. 4). BIN was developed by Ratnasingham and Hebert^29^, who pointed out that the taxonomic performance was similar to that of GMYC. Their analyses based on different datasets suggested that the taxonomic performance of these two methods was stronger than results obtained with ABGD. In contrast to this, our analyses suggest that ABGD is a more accurate method of species delimitation than BIN and GMYC. It is known that GMYC can lead to an overestimation of species numbers, whereas ABGD is regarded as a more conservative method, especially in groups where large species numbers are expected^38–41^. Unexpectedly, the bPTP approach yielded 223 OTUs, more than twice the number of species identified based on morphological characters. Similar observations were made by Song *et al*.^42^, who evaluated the potential of eight species delimitation methods (GMYC, bPTP, mPTP, BIN, ABGD, jMOTU, NJ, threshold clustering), using a superdiverse insect genus *Polypedilum* Kieffer (Diptera: Chironomidae). Their results indicated a conservative number of species with ABGD, whereas bPTP yielded a much higher operational taxonomic unit (OTU) count than the other methods.

GMYC has been developed to delimit species based on single-locus data, which has a strong theoretical basis. However, it typically generates more OTUs than other methods^13,43,44^. Although anchored in a solid phylogenetic framework, this method heavily depends on the correctness of the ultrametric gene tree. Errors in this framework underpinning the analysis affect the final results^38^. GMYC yielded more OTUs than species based on morphology in our analysis, which may be in fact a result of erroneous ultrametric tree reconstruction. Evaluations of the accuracy and efficiency of ABGD conducted by different researchers reached a consensus that ABGD is more conservative and faster than other methods^28,37,38,45^. This is confirmed by our results, where ABGD yielded 110 OTUs, a number very close to that of species defined based on morphology. Consequently, we recommend ABGD as the best method to delimit species of Coccinellidae. BINs in BOLD have been yielded results largely conform with traditional taxonomy in many groups of animals^46^. However, it apparently overestimated species numbers in our study, arguably due to the low intra-specific genetic distance threshold obtained for Coccinellidae with the Refined Single Linkage (RESL) method. Both BIN and ABGD use clustering algorithms to distinguish partitions in the genetic distance among a group of individuals, resulting in a final array of OTUs^38^. In contrast to BIN, ABGD infers an appropriate intracluster threshold with an automatic statistical approach, resulting in a partition close to the pattern of morphology-based species. bPTP neither requires an ultrametric input tree nor a sequence similarity threshold as input^27^. Instead it adds Bayesian support values to delimit species on the input tree. Higher Bayesian support value on a node indicates that all descendants of this node are likely species. In our analyses, bPTP yielded an astonishing overestimation of 223 OTUs, more than twice the number of morphology-based species. Similar results were also obtained by other authors^42,47^. Therefore, we conclude that bPTP is not an appropriate method to delimit species of Coccinellidae.

### Suggestions for species delimitation

As different analytical approaches have different theoretical foundations, it is recommendable to test a wide variety of methods of species delimitation, and to prefer patterns that are congruent across the results^38,48^. Furthermore, the comparison of different methods helps to understand their tendency to either split or merge clusters. The disadvantage of using several methods is the increased complexity of the investigation and resulting from this an increased amount of time and effort required for OTU delimitation^38^. We also suggest to apply different species delimitation methods, preferring delimitation patterns consistent across different approaches, even though the process is complicated and time-consuming. Sauer and Hausdorf^49^, who investigated the performance of different methods using the land snail genus *Xerocrassa* (Gastropoda: Hygromiidae) on Crete, suggested that all methods based on single-locus sequences are insufficient for delimiting species. They showed that single-gene species delimitation can be affected by several forms of bias, such as the presence of pseudogenes, incomplete lineage sorting, or introgression^44,50,51^. Consequently, the inclusion of at least one additional independent gene is required. Additionally, the use of morphological, geographical and ecological data is highly recommendable, resulting in an integrative framework to delimit species^28,37,52,53^. Apparently, decisions based on analyses of single-genes contain an interpretational risk. Nevertheless, they are indifferent if the outcome is viewed as a scaffold for taxonomy, rather than as the sole criterion for the description of new taxa^38,48^.

## Methods

### Taxon sampling and data collection

All fresh specimens were collected by the authors and deposited in the Insect Collection, Department of Entomology, South China Agricultural University (SCAU). Museum specimens used in this study were also obtained from the Insect Collection of SCAU. All of them were used with permission from the Insect Collection of SCAU.

A total of 104 species of Coccinellidae (see Supplementary Table S2) have been extracted and amplified to obtain their barcode sequences. We also used museum specimens to detect the amplification efficiency of a new set of mini-barcoding primers. The collecting information and date of museum specimens are presented in Supplementary Table S1. Specimens were identified based on morphological features using major taxonomic revisions and species description^54–61^. In addition to our own data, standard 658 bp DNA barcode sequences without stop codons were searched and downloaded from GenBank and BOLD. In total, 870 COI barcodes were included. The GenBank accession number and BOLD BINs are presented in Supplementary Table S3.

### DNA extraction, PCR amplification and sequencing

**Figure 5:**
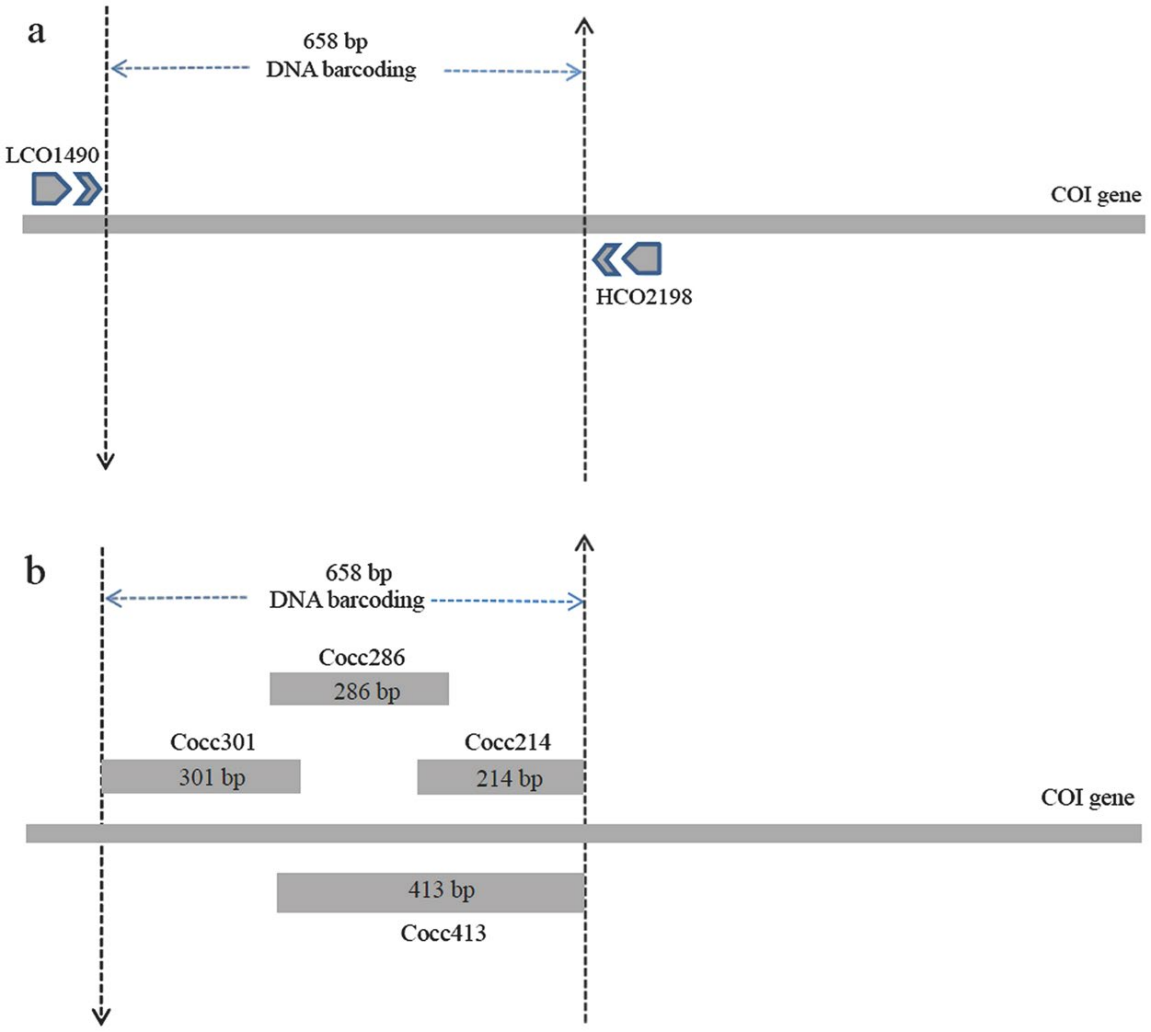
Schematic diagram of PCR amplification. (**a**) Standard DNA barcoding; (**b**) using mini barcodes to reconstruct the full DNA barcoding sequence.

Total genomic DNA was extracted from the thorax, legs or entire specimens, using the DNeasy Blood and Tissue Kit from TianGen (TianGen Biochemistry, Beijing, China), following the protocol provided by the manufacturer. We pretreated the museum specimens with 0.9% NaCl buffer before DNA as outlined in a previous study^62^. The standard COI barcoding primers LCO1490/HCO2198^63^ were compared to our set of universal primers for museum material of ladybird beetles. These specimens were collected between 1974 and 2013, pinned and stored at room temperature. The amplicon regions were coded Cocc301, Cocc286, Cocc214, Cocc413, with a length of 301 bp, 286 bp, 214 bp, 413 bp, respectively (Fig. 5). With these fragments the standard 658 bp barcode can be reconstructed through two (Cocc301 and Cocc413) or three (Cocc301, Cocc286 and Cocc214) overlapping amplicons (Table 1).

**Table 1:** Primer sets and corresponding information.

Marker Code	Primer	Sequence (5’-3’)	Amplicon size	References
Folmer	LCO1490	GGTCAACAAATCATAAAGATATTGG	658 bp	Folmer *et al*. 1994
	HCO2198	TAAACTTCAGGGTGACCAAAAAATCA		Folmer *et al*. 1994
Cocc658	WDF	TGTCAACWAATCATAAAGATATTGG	658 bp	this study
	XSR	CTTCAGGATGGCCAAAAAATCA		this study
Cocc301	WDF	TGTCAACWAATCATAAAGATATTGG	301 bp	this study
	Cocc301R	CCTGCYCCTCTTTCTACTAT		this study
Cocc286	Cocc286F	GCHTTCCCTCGWYTAAAYAATAT	286 bp	this study
	Cocc286R	GCTAAWACAGGGARAGAWAATAA		this study
Cocc214	Cocc214F	YTCYTCWATTTTAGGAGCWRTWAA	214 bp	this study
	XSR	CTTCAGGATGGCCAAAAAATCA		this study
Cocc413	Cocc413F	GCHTTCCCWCGWTTAAAYAA	413 bp	this study
	XSR	CTTCAGGATGGCCAAAAAATCA		this study

Polymerase chain reaction (PCR) amplifications were conducted in a 25 μL volumes containing 12 μL 2 × EasyTaq PCR SuperMix (TransGen Biotech, Beijing, China), 10 μL ultrapure water, 1 μL of each primer and 1 μL DNA template. PCR was performed with an initial denaturation step of 95 °C for 3 min followed by 40 cycles at 96 °C for 15 s, 50 °C for 30 s, 60 °C for 3 min and a final extension at 72 °C for 10 min. We also used the nested PCR to ensure a high success rate of PCR amplification. Nested PCR is a modification of standard PCR that uses two sets of primers in two separate PCR rounds to amplify the target band, where the product of the first round serves as the DNA template for the second round of PCR. The advantage is that nested PCR is more sensitive and specific than the standard PCR^64^. For nested PCR, as the first round of primers we used our own WDF/XSR. In the second round the PCR conditions were the same as in the first round. PCR products were electrophoresed in 1% agarose gel. DNA fragments were sequenced in both directions with sufficient overlap to ensure the accuracy of sequence data. All sequences were confirmed as the correct target fragments using BLAST (GenBank). Raw sequences were assembled and edited in Geneious 7.1.4^65^. The sequences were aligned using the MUSLE algorithm^66^ and checked for stop codons in MEGA 7^67^.

### Screening the optimum genetic distance threshold of Coccinellidae

We selected 870 standard COI barcode sequences (658 bp) from GenBank and BOLD. These sequences of 108 morphologically identified species were used for screening the optimum genetic distance threshold. The intraspecific and interspecific genetic distance matrix was calculated using MEGA 7 based on K2P model^68^. Then, ABGD analysis was implemented on the website (http://wwwabi.snv.jussieu.fr/public/abgd/abgdweb.html), using relative gap width (*X* = 1.5) and intraspecific divergence (*P*) values between 0.005 and 0.100 with the K2P model. All other settings were default.

### Molecular species delimitation

Initially, we used the GMYC method to delimit species. GMYC delimits distinct genetic clusters by optimizing the set of nodes defining the transitions between inter- and intraspecific processes^12^. The analysis was conducted using BEAST 1.8.0^69^ under a strict clock model and speciation with Yule process Tree model. The runs consisted of 70 million generations sampled every 5000 cycles. Convergence was assessed by ESS values. A burn-in with 25% was set to obtain an optimal consensus tree. We then used the obtained tree to analyse the data under the GMYC species delimitation approach in the software **R** with the package ‘splits’^70^ using the single-threshold method.

In a second step, we used the bPTP model to infer molecular delimitation clades. The bPTP model treats the number of substitutions between branching and speciation as independent events^29^. The bPTP analyses were conducted on the web server (http://species.h-its.org/ptp/) using rooted phylogenetic input tree constructed with RAxML 8.0^71^ using 500 rapid bootstrapping replicates and the GTR + I + G nucleotide substitution model. The following setting was employed: 500 000 MCMC generations, thinning interval of 100, and first 25% were discarded as burn-in.

The other two methods are ABGD and BIN. The ABGD detects a gap in the distribution of divergence that corresponds to differences between intra-specific and inter-specific distances firstly. When a gap exists, the method will work well for species delimitation^28^. The ABGD analyses were performed at the web server (http://wwwabi.snv.jussieu.fr/public/abgd/abgdweb.html). The following setting was used: relative gap width *X* = 1.5, K2P distance and intraspecific divergence (*P*) values range from 0.001 to 0.100, other parameter values employed defaults. BIN, which is widely used in the BOLD system, employs varied distance metrics to generate a neighbor-joining (NJ) tree and is established as a persistent registry for life OTUs^29^.

## Supplementary information

Supplementary information.

## Data Availability

All specimen data are accessible on BOLD (www.boldsystems.org). The data include collection locality, geographic coordinates, altitude, collector, one or more images, identifier and voucher depository. Sequences data are available on BOLD and include a detailed LIMS report, primer information and trace files, and sequences have been deposited also in GenBank (Accession Nos. MH608369 – MH611354).
