# Supplementary material for: An integrative DNA barcoding framework of ladybird beetles (Coleoptera: Coccinellidae)
Source: Sci Rep. 2020 Jun 22;10:10063. doi: 10.1038/s41598-020-66874-1 (PMC7308296; doi:10.1038/s41598-020-66874-1)
Supplement: Supplementary file 1 — Supplementary information. [file 41598_2020_66874_MOESM1_ESM.pdf]

# Supplementary Information

## An integrative DNA barcoding framework of ladybird beetles (Coleoptera: Coccinellidae)

Weidong Huang[1, 2], Xiufeng Xie[3], Lizhi Huo[4], Xinyue Liang[1, 2], Xingmin Wang[2], Xiaosheng Chen[1, 2 *]

[1]Guangdong Key Laboratory for Innovative Development and Utilization of Forest Plant Germplasm; Department of Forest Protection, College of Forestry and Landscape Architecture, South China Agricultural University, Guangzhou 510640, China.

[2]Key Laboratory of Bio-Pesticide Innovation and Application, Guangdong Province; Engineering Technology Research Center of Agricultural Pest Biocontrol, Guangdong Province; Engineering Research Center of Biological Control, Ministry of Education, Guangzhou 510640, China.

[3]Guangdong Agriculture Industry Business Polytechnic College, Guangzhou 510507, China.

[4]Guangzhou Institute of Forestry and Landscape Architecture, Guangzhou 510405, China.

* Correspondence: Xiaosheng Chen, email: xshchen@scau.edu.cn

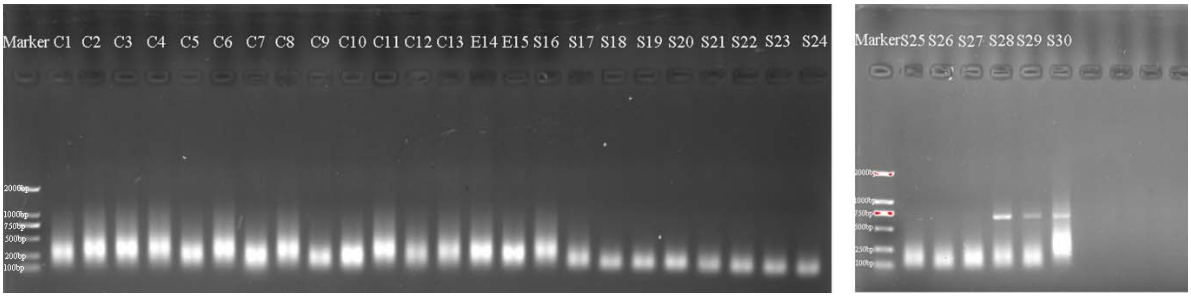


**Figure S1-1. Agarose electrophoretic images of the standard PCR using Folmer's primer pairs**. The grouping of gels was cropped from different gels with different exposures. In total, 30 specimens were detected in this study. A row of gel hole only contains 24 specimens and the other 6 specimens need to be detected by using another row of gel hole. The samples derive from the same experiment and that gels/blots were processed in parallel.

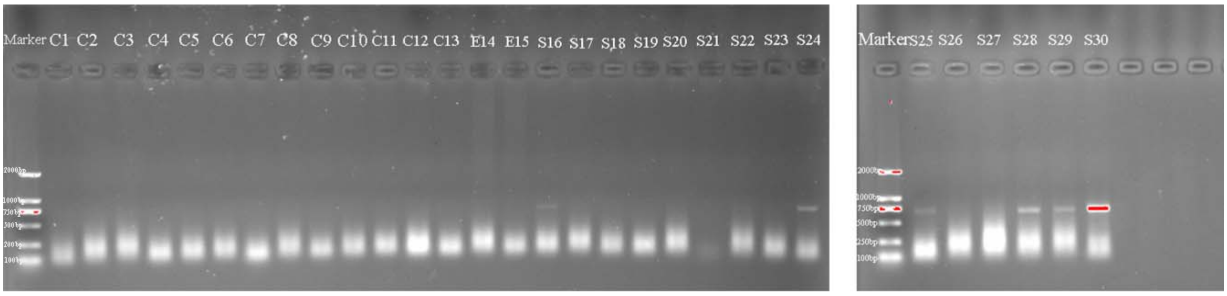


**Figure S1-2. Agarose electrophoretic images of the standard PCR using Cocc658 primer pairs.** The grouping of gels was cropped from different gels with different exposures. In total, 30 specimens were detected in this study. A row of gel hole only contains 24 specimens and the other 6 specimens need to be detected by using another row of gel hole. The samples derive from the same experiment and that gels/blots were processed in parallel.

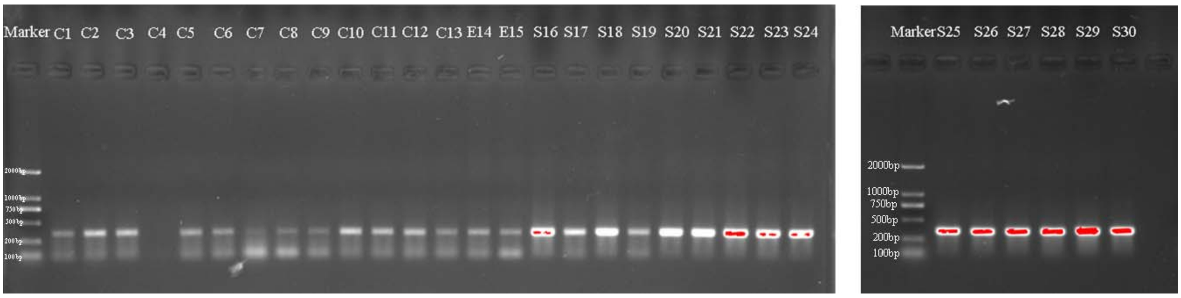


**Figure S1-3. Agarose electrophoretic images of the standard PCR using Cocc301 primer pairs**. The grouping of gels was cropped from different gels with different exposures. In total, 30 specimens were detected in this study. A row of gel hole only contains 24 specimens and

the other 6 specimens need to be detected by using another row of gel hole. The samples derive from the same experiment and that gels/blots were processed in parallel.

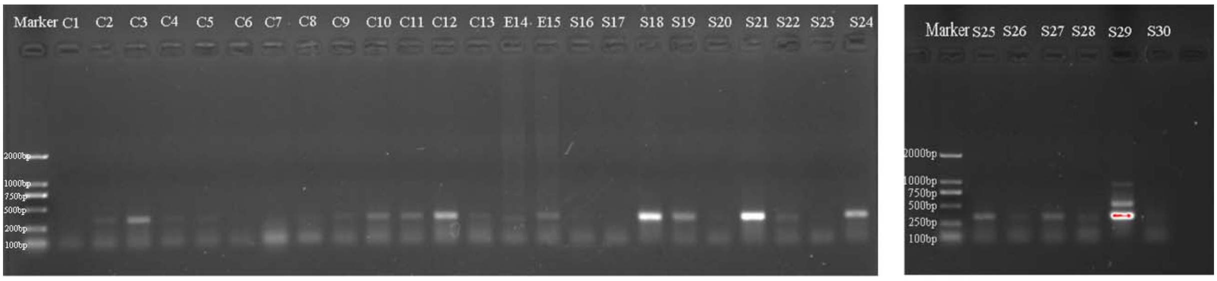


**Figure S1-4. Agarose electrophoretic images of the standard PCR using Cocc286 primer pairs**. The grouping of gels was cropped from different gels with different exposures. In total, 30 specimens were detected in this study. A row of gel hole only contains 24 specimens and the other 6 specimens need to be detected by using another row of gel hole. The samples derive from the same experiment and that gels/blots were processed in parallel.

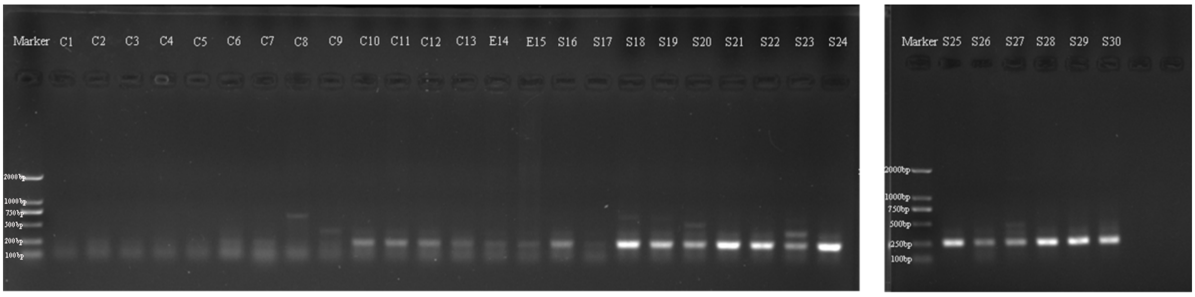


**Figure S1-5. Agarose electrophoretic images of the standard PCR using Cocc214 primer pairs**. The grouping of gels was cropped from different gels with different exposures. In total, 30 specimens were detected in this study. A row of gel hole only contains 24 specimens and the other 6 specimens need to be detected by using another row of gel hole. The samples derive from the same experiment and that gels/blots were processed in parallel.

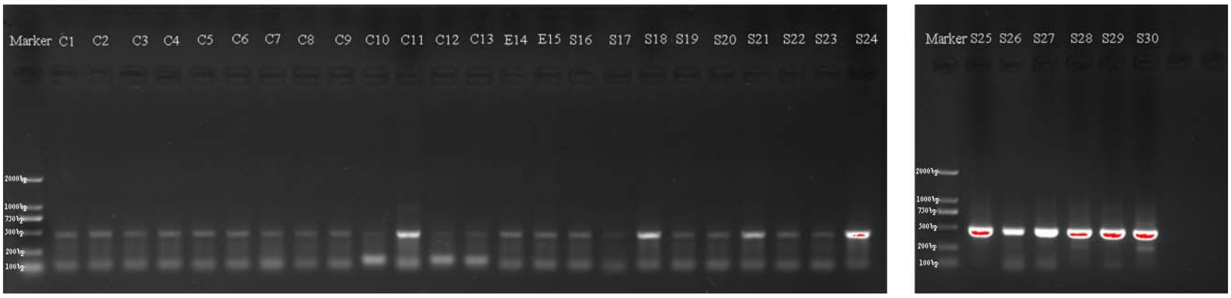


**Figure S1-6. Agarose electrophoretic images of the standard PCR using Cocc413 primer**

**pairs**. The grouping of gels was cropped from different gels with different exposures. In total, 30 specimens were detected in this study. A row of gel hole only contains 24 specimens and the other 6 specimens need to be detected by using another row of gel hole. The samples derive from the same experiment and that gels/blots were processed in parallel.

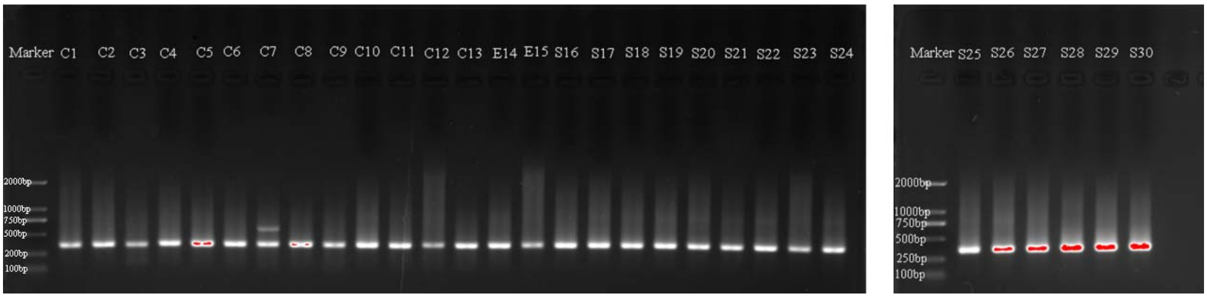


**Figure S1-7. Agarose electrophoretic images of the nested PCR using Cocc301 primer pairs.** The grouping of gels was cropped from different gels with different exposures. In total, 30 specimens were detected in this study. A row of gel hole only contains 24 specimens and the other 6 specimens need to be detected by using another row of gel hole. The samples derive from the same experiment and that gels/blots were processed in parallel.

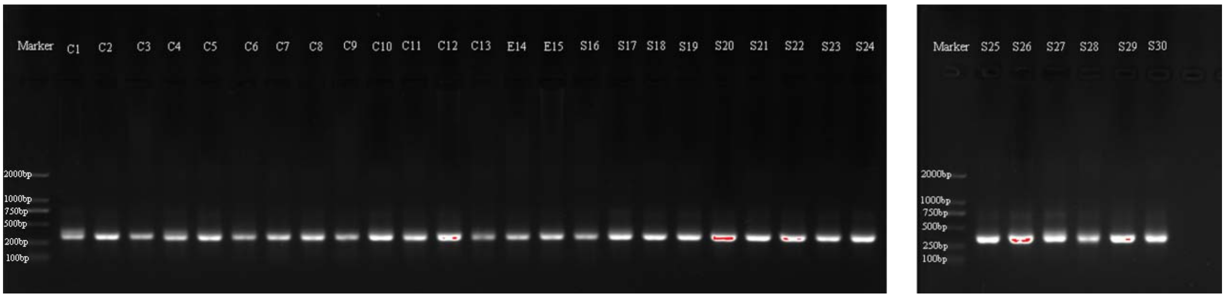


**Figure S1-8. Agarose electrophoretic images of the nested PCR using Cocc286 primer pairs**. The grouping of gels was cropped from different gels with different exposures. In total, 30 specimens were detected in this study. A row of gel hole only contains 24 specimens and the other 6 specimens need to be detected by using another row of gel hole. The samples derive from the same experiment and that gels/blots were processed in parallel.

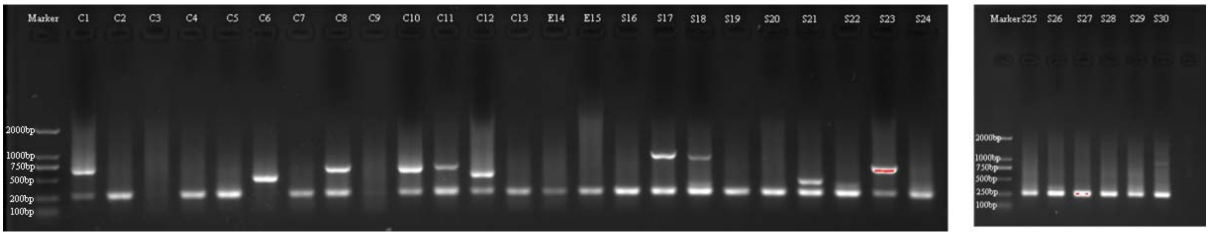


**Figure S1-9. Agarose electrophoretic images of the nested PCR using Cocc214 primer pairs.** The grouping of gels was cropped from different gels with different exposures. In total, 30 specimens were detected in this study. A row of gel hole only contains 24 specimens and the other 6 specimens need to be detected by using another row of gel hole. The samples derive from the same experiment and that gels/blots were processed in parallel.

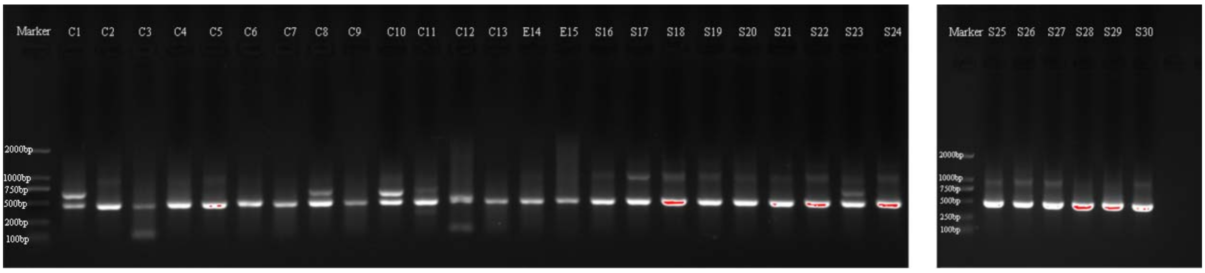


**Figure S1-10. Agarose electrophoretic images of the nested PCR using Cocc413 primer pairs**. The grouping of gels was cropped from different gels with different exposures. In total, 30 specimens were detected in this study. A row of gel hole only contains 24 specimens and the other 6 specimens need to be detected by using another row of gel hole. The samples derive from the same experiment and that gels/blots were processed in parallel.

## Supplementary Table S1 The infromation of museum specimens

| Number | Voucher ID | Genus | Species name | Date | Locality |
|---|---|---|---|---|---|
| 1 | SCAU (E) 60470 | *Coccinella* | *Coccinella septempunctata* | 1974 | China |
| 2 | SCAU (E) 60471 | *Coccinella* | *Coccinella septempunctata* | 1974 | China |
| 3 | SCAU (E) 60472 | *Coccinella* | *Coccinella septempunctata* | 1980 | China |
| 4 | SCAU (E) 60473 | *Coccinella* | *Coccinella septempunctata* | 1980 | China |
| 5 | SCAU (E) 60474 | *Harmonia* | *Harmonia axyridis* | 1977 | China |
| 6 | SCAU (E) 60475 | *Harmonia* | *Harmonia axyridis* | 1977 | China |
| 7 | SCAU (E) 60476 | *Harmonia* | *Harmonia axyridis* | 1987 | China |
| 8 | SCAU (E) 60477 | *Harmonia* | *Harmonia axyridis* | 1994 | China |
| 9 | SCAU (E) 60478 | *Harmonia* | *Harmonia axyridis* | 1994 | China |
| 10 | SCAU (E) 60479 | *Menochilus* | *Menochilus sexmaculatus* | 1995 | China |
| 11 | SCAU (E) 60480 | *Menochilus* | *Menochilus sexmaculatus* | 1995 | China |
| 12 | SCAU (E) 60481 | *Menochilus* | *Menochilus sexmaculatus* | 1996 | China |
| 13 | SCAU (E) 60482 | *Menochilus* | *Menochilus sexmaculatus* | 1996 | China |
| 14 | SCAU (E) 60483 | *Henosepilachna* | *Henosepilachna vigintioctopunctata* | 1996 | China |
| 15 | SCAU (E) 60484 | *Henosepilachna* | *Henosepilachna vigintioctopunctata* | 1997 | China |
| 16 | SCAU (E) 60485 | *Scymnus* | *Scymnus* (*Pullus*) *posticalis* | 2004 | China |
| 17 | SCAU (E) 60486 | *Scymnus* | *Scymnus* (*Pullus*) *posticalis* | 2004 | China |
| 18 | SCAU (E) 60487 | *Scymnus* | *Scymnus* (*Pullus*) *posticalis* | 2006 | China |
| 19 | SCAU (E) 60488 | *Scymnus* | *Scymnus* (*Pullus*) *posticalis* | 2006 | China |
| 20 | SCAU (E) 60489 | *Scymnus* | *Scymnus* (*Pullus*) *posticalis* | 2008 | China |
| 21 | SCAU (E) 60490 | *Scymnus* | *Scymnus* (*Pullus*) *posticalis* | 2008 | China |
| 22 | SCAU (E) 60491 | *Scymnus* | *Scymnus* (*Pullus*) *posticalis* | 2010 | China |
| 23 | SCAU (E) 60492 | *Scymnus* | *Scymnus* (*Pullus*) *posticalis* | 2010 | China |
| 24 | SCAU (E) 60493 | *Scymnus* | *Scymnus* (*Pullus*) *posticalis* | 2011 | China |
| 25 | SCAU (E) 60494 | *Scymnus* | *Scymnus* (*Pullus*) *posticalis* | 2011 | China |
| 26 | SCAU (E) 60495 | *Scymnus* | *Scymnus* (*Pullus*) *posticalis* | 2012 | China |
| 27 | SCAU (E) 60496 | *Scymnus* | *Scymnus* (*Pullus*) *posticalis* | 2012 | China |
| 28 | SCAU (E) 60497 | *Scymnus* | *Scymnus* (*Pullus*) *posticalis* | 2013 | China |
| 29 | SCAU (E) 60498 | *Scymnus* | *Scymnus* (*Pullus*) *posticalis* | 2013 | China |
| 30 | SCAU (E) 60499 | *Scymnus* | *Scymnus* (*Pullus*) *posticalis* | 2013 | China |

# Supplementary Table S2 The samples information of ladybird beetles

| Number | Code | Genus | Sub-genus | Species name | GenBank accession number | BOLD: BIN | Country | Province | Region |
|---|---|---|---|---|---|---|---|---|---|
| 1 | HW1 | *Horniolus* | | *Horniolus sonduongensis* Hoàng, 1979 | MK801972 | ADY2930 | China | Guangdong | Huizhou |
| 2 | HW2 | *Horniolus* | | *Horniolus hisamatsu* Miyatake, 1976 | MK801973 | ADY2929 | China | Guangdong | Guangzhou |
| 3 | SV1 | *Sasajiscymnus* | | *Sasajiscymnus curvatus* (Yu, 1999) | MK801984 | ADY3448 | China | Anhui | Anqin |
| 4 | SV2 | *Sasajiscymnus* | | *Sasajiscymnus seminigrinus* (Yu et Pang, 1993) | MK801986 | ADY3446 | China | Hainan | Wuzhishan |
| 5 | SV3 | *Sasajiscymnus* | | *Sasajiscymnus quinquepunctatus* (Weise, 1923) | MK801987 | ADY4043 | China | Anhui | Huangshan |
| 6 | SV4 | *Sasajiscymnus* | | *Sasajiscymnus bivalvis* (Yu, 1997) | MK801983 | ADY3447 | China | Anhui | Huangshan |
| 7 | SV5 | *Sasajiscymnus* | | *Sasajiscymnus disselasmatus* (Pang et Huang, 1986) | MK801985 | ADY3449 | China | Anhui | Luan |
| 8 | NG1 | *Nephaspis* | | *Nephaspis indus* Gordon, 1996 | MK801977 | ACO6393 | China | Taiwan | Taidong |
| 9 | AK1 | *Axinoscymnus* | | *Axinoscymnus cardilobus* Ren et Pang, 1992 | MK801967 | ADY4976 | China | Hainan | Sanya |
| 10 | AK2 | *Axinoscymnus* | | *Axinoscymnus* sp. | MK801968 | ADY6084 | China | Anhui | Chizhou |
| 11 | NM2 | *Nephus* | | *Nephus* sp. | MK801981 | ADY0188 | China | Anhui | Luan |
| 12 | NNM1 | *Nephus* | *Nephus* | *Nephus* (*Nephus*) *quadrimaculatus* (Herbst, 1783) | MK801980 | ADY5654 | China | Hebei | Zhangjiakou |
| 13 | NGF1 | *Nephus* | *Geminosipho* | *Nephus* (*Geminosipho*) *patagiatus* (Lewis, 1896) | MK801979 | ADY0302 | China | Guangxi | Fangchenggang |
| 14 | NGF2 | *Nephus* | *Geminosipho* | *Nephus* (*Geminosipho*) *koltzei* (Weise, 1887) | MK801978 | ADY0301 | China | Ningxia | Guyuan |
| 15 | KS1 | *Keiscymnus* | | *Keiscymnus* sp.1 | MK801974 | ADY0829 | China | Xizang | Linzhi |
| 16 | KS2 | *Keiscymnus* | | *Keiscymnus* sp.2 | MK801975 | ADY6078 | China | Guizhou | |
| 17 | SOC1 | *Scymnus* | *Orthoscymnus* | *Scymnus* (*Orthoscymnus*) *rhododendri* Canepari, 1997 | MK801995 | ADY2349 | China | Xizang | Rikaze |
| 18 | SOC2 | *Scymnus* | *Orthoscymnus* | *Scymnus* (*Orthoscymnus*) *paradoxus* Chen et Ren, 2016 | MK801994 | ADY3298 | China | Xizang | Rikaze |
| 19 | SPY1 | *Scymnus* | *Parapullus* | *Scymnus* (*Parapullus*) *secula* Yang, 1978 | MK801997 | ADY4953 | China | Taiwan | Jiayi |
| 20 | SPY2 | *Scymnus* | *Parapullus* | *Scymnus* (*Parapullus*) *alishanensis* Pang et Yu, 1993 | MK801996 | ADY5365 | China | Taiwan | Jiayi |
| 21 | SNS2 | *Scymnus* | *Neopullus* | *Scymnus* (*Neopullus*) *paralleus* Yu et Pang, 1994 | MK801992 | ACO8443 | China | Tianjin | Jixian |
| 22 | SNS3 | *Scymnus* | *Neopullus* | *Scymnus* (*Neopullus*) *babai* Sasaji, 1971 | MK801988 | ADY3021 | China | Zhejiang | Hangzhou |
| 23 | SNS4 | *Scymnus* | *Neopullus* | *Scymnus* (*Neopullus*) *fuscatus* Boheman, 1859 | MK801989 | ACV1539 | China | Fujian | Sanming |
| 24 | SNS5 | *Scymnus* | *Neopullus* | *Scymnus* (*Neopullus*) *lijiangensis* Yu, 2000 | MK801990 | ADY5388 | China | Yunnan | Kunming |
| 25 | SNS6 | *Scymnus* | *Neopullus* | *Scymnus* (*Neopullus*) *minisculus* Yu et Pang, 1993 | MK801991 | ADY1587 | China | Hunan | Zhuzhou |
| 26 | SNS7 | *Scymnus* | *Neopullus* | *Scymnus* (*Neopullus*) *thecacontus* Ren et Pang, 1993 | MK801993 | ADY4273 | China | Hubei | Shiyan |
| 27 | SSK1 | *Scymnus* | *Scymnus* | *Scymnus* (*Scymnus*) *nubilus* Mulsant, 1850 | MK802057 | ADY2169 | China | Taiwan | Pingdong |
| 28 | SSK2 | *Scymnus* | *Scymnus* | *Scymnus* (*Scymnus*) *crinitus* Fürsch, 1966 | MK802048 | ACD4625 | China | Yunnan | Dali |

## Supplementary Table S2 (continued)

| Number | Code | Genus | Sub-genus | Species name | GenBank accession number | BOLD: BIN | Country | Province | Region |
|---|---|---|---|---|---|---|---|---|---|
| 29 | SSK3 | *Scymnus* | *Scymnus* | *Scymnus* (*Scymnus*) *dolichonychus* Yu et Pang, 1994 | MK802049 | ADY5718 | China | Fujian | Sanming |
| 30 | SSK5 | *Scymnus* | *Scymnus* | *Scymnus* (*Scymnus*) *longmenicus* Pang, 1986 | MK802054 | ADY5390 | China | Fujian | Yongan |
| 31 | SSK6 | *Scymnus* | *Scymnus* | *Scymnus* (*Scymnus*) *folchinii* Canepari, 1979 | MK802051 | ADV3437 | China | Anhui | Huangshan |
| 32 | SSK7 | *Scymnus* | *Scymnus* | *Scymnus* (*Scymnus*) *caiyanghensis* Chen, Wang et Ren, 2013 | MK802046 | ADY3010 | China | Yunnan | Xishuangbanna |
| 33 | SSK8 | *Scymnus* | *Scymnus* | *Scymnus* (*Scymnus*) *sufflavus* Canepari, 1997 | MK802061 | ADY4586 | China | Yunnan | Kunming |
| 34 | SSK9 | *Scymnus* | *Scymnus* | *Scymnus* (*Scymnus*) *trisulicus* Canepari, 1997 | MK802062 | ADY4270 | China | Xizang | Shannan |
| 35 | SSK10 | *Scymnus* | *Scymnus* | *Scymnus* (*Scymnus*) *elagatisophyllus* Kuznetsov et Ren, 1991 | MK802050 | ADY5719 | China | Yunnan | Jinghong |
| 36 | SSK11 | *Scymnus* | *Scymnus* | *Scymnus* (*Scymnus*) *scapanulus* Pang et Huang, 1986 | MK802060 | ADY5391 | China | Shanxi | Yuncheng |
| 37 | SSK12 | *Scymnus* | *Scymnus* | *Scymnus* (*Scymnus*) *kabakovi* Hoàng, 1982 | MK802053 | ADY0861 | China | Yunan | Gongshan |
| 38 | SSK13 | *Scymnus* | *Scymnus* | *Scymnus* (*Scymnus*) *pinguis* Yu, 1999 | MK802059 | ADY3757 | China | Hunan | Zhuzhou |
| 39 | SSK14 | *Scymnus* | *Scymnus* | *Scymnus* (*Scymnus*) *manipulus* Fürsch et Kreissl, 1967 | MK802055 | ADY1586 | China | Xinjiang | Akesu |
| 40 | SSK15 | *Scymnus* | *Scymnus* | *Scymnus* (*Scymnus*) *paracrinitus* Yu, 2000 | MK802058 | ADY4622 | China | Yunnan | Kunming |
| 41 | SSK16 | *Scymnus* | *Scymnus* | *Scymnus* (*Scymnus*) *acidotus* Pang et Huang, 1985 | MK802044 | ADY5364 | China | Yunnan | Xishuangbanna |
| 42 | SSK17 | *Scymnus* | *Scymnus* | *Scymnus* (*Scymnus*) *bifurcatus* Yu, 1995 | MK802045 | ADY2766 | China | Yunnan | Honghe |
| 43 | SSK19 | *Scymnus* | *Scymnus* | *Scymnus* (*Scymnus*) *comosus* Chen, Wang et Ren, 2013 | MK802047 | ADY5721 | China | Yunnan | Puer |
| 44 | SSK20 | *Scymnus* | *Scymnus* | *Scymnus* (*Scymnus*) *inderihensis* Mulsant, 1850 | MK802052 | ADY0868 | China | Shanxi | Xinzhou |
| 45 | SSK23 | *Scymnus* | *Scymnus* | *Scymnus* (*Scymnus*) *notidanus* Pang et Huang, 1985 | MK802056 | ADY4307 | China | Guangdong | Qingyuan |
| 46 | SPM1 | *Scymnus* | *Pullus* | *Scymnus* (*Pullus*) *podoides* Yu et Pang, 1992 | MK802028 | ADX7375 | China | Anhui | Huangshan |
| 47 | SPM2 | *Scymnus* | *Pullus* | *Scymnus* (*Pullus*) *nephrospilus* Ren et Pang, 1995 | MK802023 | ADY1590 | China | Anhui | Chizhou |
| 48 | SPM3 | *Scymnus* | *Pullus* | *Scymnus* (*Pullus*) *trimaculatus* Yu et Pang, 1994 | MK802042 | ADY4271 | China | Hunan | Zhuzhou |
| 49 | SPM6 | *Scymnus* | *Pullus* | *Scymnus* (*Pullus*) *rhamphiatus* Pang et Huang, 1985 | MK802030 | ADX8949 | China | Hunan | Zhuzhou |
| 50 | SPM7 | *Scymnus* | *Pullus* | *Scymnus* (*Pullus*) *klinosiphonicus* Ren et Pang, 1995 | MK802017 | ADY4790 | China | Hunan | Zhuzhou |
| 51 | SPM8 | *Scymnus* | *Pullus* | *Scymnus* (*Pullus*) *oestocraerus* Pang et Huang, 1985 | MK802024 | ADY3754 | China | Xizang | Linzhi |
| 52 | SPM9 | *Scymnus* | *Pullus* | *Scymnus* (*Pullus*) *godavariensis* Miyatake, 1985 | MK802011 | ADY2136 | China | Xizang | Linzhi |
| 53 | SPM10 | *Scymnus* | *Pullus* | *Scymnus* (*Pullus*) *hingstoni* Kapur, 1963 | MK802014 | ADY4370 | China | Xizang | Linzhi |
| 54 | SPM11 | *Scymnus* | *Pullus* | *Scymnus* (*Pullus*) *scalpratus* Yu, 1993 | MK802032 | ADX8950 | China | Taiwan | Pingdong |
| 55 | SPM12 | *Scymnus* | *Pullus* | *Scymnus* (*Pullus*) *fujianensis* Pang et Gordon, 1986 | MK802010 | ADI4742 | China | Yunnan | Nujiang |
| 56 | SPM13 | *Scymnus* | *Pullus* | *Scymnus* (*Pullus*) *subvillosus* (Goeze, 1777) | MK802037 | ADY3039 | China | Xinjiang | Aletai |
| 57 | SPM14 | *Scymnus* | *Pullus* | *Scymnus* (*Pullus*) *martensi* Canepari, 1997 | MK802020 | ADX8894 | China | Xizang | Shannan |
| 58 | SPM16 | *Scymnus* | *Pullus* | *Scymnus* (*Pullus*) *ambonoidea* Pang, 1988 | MK801999 | ADY5021 | China | Yunnan | Jinghong |
| 59 | SPM18 | *Scymnus* | *Pullus* | *Scymnus* (*Pullus*) *eminulus* Chen et Ren, 2015 | MK802018 | ADY5389 | China | Yunnan | Puer |
| 60 | SPM19 | *Scymnus* | *Pullus* | *Scymnus* (*Pullus*) *accamptus* Pang et Pu, 1990 | MK801998 | ADY2588 | China | Yunnan | Puer |

## Supplementary Table S2 (continued)

| Number | Code | Genus | Sub-genus | Species name | GenBank accession number | BOLD: BIN | Country | Province | Region |
|---|---|---|---|---|---|---|---|---|---|
| 61 | SPM20 | *Scymnus* | *Pullus* | *Scymnus* (*Pullus*) *sodalis* (Weise, 1923) | MK802036 | ADJ0520 | China | Yunnan | Honghe |
| 62 | SPM22 | *Scymnus* | *Pullus* | *Scymnus* (*Pullus*) *posticalis* Sicard, 1912 | MK802029 | ADY3097 | China | Yunnan | Honghe |
| 63 | SPM23 | *Scymnus* | *Pullus* | *Scymnus* (*Pullus*) *japonicus* Weise, 1879 | MK802015 | ADY2135 | China | Yunnan | Xishuangbanna |
| 64 | SPM24 | *Scymnus* | *Pullus* | *Scymnus* (*Pullus*) *facetus* Canepari, 1986 | MK802008 | ADY5717 | China | Xizang | Linzhi |
| 65 | SPM27 | *Scymnus* | *Pullus* | *Scymnus* (*Pullus*) *bourdilloni* Kapur, 1955 | MK802001 | ADY6057 | China | Xizang | Linzhi |
| 66 | SPM28 | *Scymnus* | *Pullus* | *Scymnus* (*Pullus*) *centralis* Kamiya, 1965 | MK802002 | ADY5052 | China | Yunnan | Jinghong |
| 67 | SPM29 | *Scymnus* | *Pullus* | *Scymnus* (*Pullus*) *scrobiculatus* Yu, 1993 | MK802033 | ADX9626 | China | Yunnan | Xishuangbanna |
| 68 | SPM30 | *Scymnus* | *Pullus* | *Scymnus* (*Pullus*) *tegminalis* Hoàng, 1985 | MK802040 | ADY2323 | China | Yunnan | Xishuangbanna |
| 69 | SPM31 | *Scymnus* | *Pullus* | *Scymnus* (*Pullus*) *shirozui* Kamiya, 1965 | MK802034 | ADY5294 | China | Yunnan | Xishuangbanna |
| 70 | SPM35 | *Scymnus* | *Pullus* | *Scymnus* (*Pullus*) *mastigoides* Ren et Pang, 1995 | MK802021 | ADY1585 | China | Fujian | Yongan |
| 71 | SPM36 | *Scymnus* | *Pullus* | *Scymnus* (*Pullus*) *shixingicus* Yu et Pang, 1992 | MK802035 | ADY5293 | China | Fujian | Jiangle |
| 72 | SPM39 | *Scymnus* | *Pullus* | *Scymnus* (*Pullus*) *takasago* Kamiya, 1965 | MK802039 | ADY1967 | China | Taiwan | Yilan |
| 73 | SPM40 | *Scymnus* | *Pullus* | *Scymnus* (*Pullus*) *cryphaconicus* Ren et Pang, 1994 | MK802005 | ADX9300 | China | Shaanxi | Hanzhong |
| 74 | SPM42 | *Scymnus* | *Pullus* | *Scymnus* (*Pullus*) *fanjingicus* Ren et Pang, 1995 | MK802009 | ADY0003 | China | Taiwan | Jiayi |
| 75 | SPM43 | *Scymnus* | *Pullus* | *Scymnus* (*Pullus*) *contemtus* (Weise, 1923) | MK802004 | ADX9302 | China | Taiwan | Gaoxiong |
| 76 | SPM45 | *Scymnus* | *Pullus* | *Scymnus* (*Pullus*) *heptaspilicus* Ren et Pang, 1995 | MK802012 | ADY4369 | China | Hunan | Zhuzhou |
| 77 | SPM46 | *Scymnus* | *Pullus* | *Scymnus* (*Pullus*) *penicilliformis* Yu, 1999 | MK802027 | ADY3756 | China | Shanxi | Yuncheng |
| 78 | SPM47 | *Scymnus* | *Pullus* | *Scymnus* (*Pullus*) *auritus* (Thunberg, 1795) | MK802000 | ADY4784 | China | Anhui | Chizhou |
| 79 | SPM48 | *Scymnus* | *Pullus* | *Scymnus* (*Pullus*) *exocorycus* Pang et Huang, 1986 | MK802007 | ADY5720 | China | Hunan | Zhuzhou |
| 80 | SPM50 | *Scymnus* | *Pullus* | *Scymnus* (*Pullus*) *lonchiatus* Pang et Huang, 1985 | MK802019 | ADX9339 | China | Sichuan | Leibo |
| 81 | SPM51 | *Scymnus* | *Pullus* | *Scymnus* (*Pullus*) *heyuanus* Yu, 2000 | MK802013 | ADY4371 | China | Anhui | Anqin |
| 82 | SPM52 | *Scymnus* | *Pullus* | *Scymnus* (*Pullus*) *takabayashii* (Ohta, 1929) | MK802038 | ADY1966 | China | Guizhou | |
| 83 | SPM53 | *Scymnus* | *Pullus* | *Scymnus* (*Pullus*) *cladocerus* Ren et Pang, 1995 | MK802003 | ADY5020 | China | Hunan | Zhuzhou |
| 84 | SPM54 | *Scymnus* | *Pullus* | *Scymnus* (*Pullus*) *dactylicus* Pang, 1988 | MK802006 | ADX9301 | China | Guangdong | Qingyuan |
| 85 | SPM56 | *Scymnus* | *Pullus* | *Scymnus* (*Pullus*) *paraperdere* Yu et Pang, 1993 | MK802026 | ADY3755 | China | Yunnan | Puer |
| 86 | SPM57 | *Scymnus* | *Pullus* | *Scymnus* (*Pullus*) *tympanus* Yu et Pang, 1992 | MK802043 | ADY4272 | China | Yunnan | Xishuangbanna |
| 87 | SPM58 | *Scymnus* | *Pullus* | *Scymnus* (*Pullus*) *pangi* Fürsch, 1989 | MK802025 | ADY3753 | China | Guangdong | Qingyuan |
| 88 | SPM59 | *Scymnus* | *Pullus* | *Scymnus* (*Pullus*) *rhombicus* Yu et Pang, 1993 | MK802031 | ADY1695 | China | Hunan | Chenzhou |
| 89 | SPM60 | *Scymnus* | *Pullus* | *Scymnus* (*Pullus*) *kaguyahime* Kamiya, 1961 | MK802016 | ADY5051 | China | Taiwan | Pingdong |
| 90 | SPM61 | *Scymnus* | *Pullus* | *Scymnus* (*Pullus*) *nankunicus* Pang, 1986 | MK802022 | ADY1589 | China | Hunan | Zhuzhou |
| 91 | SPM65 | *Scymnus* | *Pullus* | *Scymnus* (*Pullus*) *toxosiphonius* Pang et Huang, 1986 | MK802041 | ADY4876 | China | Fujian | Quanzhou |
| 92 | HCW1 | *Serangium* | | *Serangium japonicum* Chapin, 1940 | MK802063 | ADY3178 | China | Guangdong | Guangzhou |

## Supplementary Table S2 (continued)

| Number | Code | Genus | Sub-genus | Species name | GenBank accession number | BOLD: BIN | Country | Province | Region |
|---|---|---|---|---|---|---|---|---|---|
| 93 | HCW2 | *Rodolia* | | *Rodolia pumila* Weise, 1892 | MK801982 | ADY3453 | China | Guangdong | Guangzhou |
| 94 | HCW3 | *Menochilus* | | *Menochilus sexmaculatus* (Fabricius, 1781) | MK801976 | ACM3340 | China | Guangdong | Guangzhou |
| 95 | HCW4 | *Henosepilachna* | | *Henosepilachna vigintioctopunctata* (Fabricius, 1775) | MK801971 | AAI6802 | China | Guangdong | Guangzhou |
| 96 | HCW5 | *Cryptolaemus* | | *Cryptolaemus montrouzieri* Mulsant, 1853 | MK801969 | AAD6040 | China | Guangdong | Guangzhou |
| 97 | HCW6 | *Afissula* | | *Afissula expansa* (Dieke, 1947) | MK801966 | ADY0707 | China | Yunnan | Honghe |
| 98 | HCW7 | *Epilachna* | | *Epilachna plicata* Weise, 1889 | MK801970 | ADY3643 | China | Shaanxi | Baoji |
| 99 | HCW8 | *Aspidimerus* | | *Aspidimerus matsumurai* Sasaji, 1968 | MK802064 | ADY5411 | China | Yunnan | Nanping |
| 100 | HCW9 | *Aspidimerus* | | *Aspidimerus zhenkangicus* Huo et Ren, 2013 | MK802065 | ADY1008 | China | Yunnan | Mengdui |
| 101 | HCW10 | *Cryptogonus* | | *Cryptogonus angusticarinatus* Sasaji, 1968 | MK802066 | ADY4034 | China | Taiwan | Taidong |
| 102 | HCW11 | *Cryptogonus* | | *Cryptogonus lijiangiensis* Pang et Mao, 1979 | MK802067 | ADY2676 | China | Yunnan | Diqing |
| 103 | HCW12 | *Cryptogonus* | | *Cryptogonus nepalensis* Bielawski, 1972 | MK802068 | ADY3233 | China | Yunnan | Kunming |
| 104 | HCW13 | *Cryptogonus* | | *Cryptogonus ohtai* Sasaji, 1968 | MK802069 | ADY4256 | China | Taiwan | Taidong |

**Supplementary Table S3  List of standard DNA barcodes 658 bp downloaded from GenBank and BOLD**

| Subfamily | Genus | Species name | Code | GenBank accession number | BOLD BIN | Collection country |
|---|---|---|---|---|---|---|
| Chilocorinae | *Brumoides* | *Brumoides suturalis* | 1 | KY831199 | BOLD:AAP7925 | Pakistan |
| Chilocorinae | *Brumoides* | *Brumoides suturalis* | 2 | KY844818 | BOLD:AAP7925 | Pakistan |
| Chilocorinae | *Brumus* | *Brumus quadripustulatus* | 3 | KJ963252 | BOLD:AAP7229 | Finland |
| Chilocorinae | *Brumus* | *Brumus quadripustulatus* | 4 | JF889511 | BOLD:AAP7229 | Germany |
| Chilocorinae | *Brumus* | *Brumus quadripustulatus* | 5 | KM439470 | BOLD:AAP7229 | Germany |
| Chilocorinae | *Brumus* | *Brumus quadripustulatus* | 6 | KM451511 | BOLD:AAP7229 | Germany |
| Chilocorinae | *Brumus* | *Brumus quadripustulatus* | 7 | KM450715 | BOLD:AAP7229 | Germany |
| Chilocorinae | *Brumus* | *Brumus quadripustulatus* | 8 | KM440963 | BOLD:AAP7229 | Germany |
| Chilocorinae | *Brumus* | *Brumus quadripustulatus* | 9 | KM450884 | BOLD:AAP7229 | Germany |
| Chilocorinae | *Brumus* | *Brumus quadripustulatus* | 10 | KM450902 | BOLD:AAP7229 | Germany |
| Chilocorinae | *Brumus* | *Brumus quadripustulatus* | 11 | KM449849 | BOLD:AAP7229 | Germany |
| Chilocorinae | *Brumus* | *Brumus quadripustulatus* | 12 | KU917901 | BOLD:AAP7229 | Germany |
| Chilocorinae | *Brumus* | *Brumus quadripustulatus* | 13 | KU916167 | BOLD:AAP7229 | Germany |
| Chilocorinae | *Brumus* | *Brumus quadripustulatus* | 14 | KU908778 | BOLD:AAP7229 | Germany |
| Chilocorinae | *Brumus* | *Brumus quadripustulatus* | 15 | KU915297 | BOLD:AAP7229 | Germany |
| Chilocorinae | *Brumus* | *Brumus quadripustulatus* | 16 | KM441111 | BOLD:AAP7229 | Germany |
| Chilocorinae | *Brumus* | *Brumus quadripustulatus* | 17 | KM444220 | BOLD:AAP7229 | Belgium |
| Chilocorinae | *Chilocorus* | *Chilocorus bipustulatus* | 18 | KJ964331 | BOLD:ACD0938 | Finland |
| Chilocorinae | *Chilocorus* | *Chilocorus bipustulatus* | 19 | KM441967 | BOLD:ACD0938 | Germany |
| Chilocorinae | *Chilocorus* | *Chilocorus bipustulatus* | 20 | KU906200 | BOLD:ACD0938 | Germany |
| Chilocorinae | *Chilocorus* | *Chilocorus bipustulatus* | 21 | KU918089 | BOLD:ACD0938 | Germany |
| Chilocorinae | *Chilocorus* | *Chilocorus bipustulatus* | 22 | KU908307 | BOLD:ACD0938 | Germany |
| Chilocorinae | *Chilocorus* | *Chilocorus bipustulatus* | 23 | KU916640 | BOLD:ACD0938 | Germany |
| Chilocorinae | *Chilocorus* | *Chilocorus bipustulatus* | 24 | KU913478 | BOLD:ACD0938 | Germany |
| Chilocorinae | *Chilocorus* | *Chilocorus nigritus* | 25 | KY835267 | BOLD:ABX2096 | Pakistan |
| Chilocorinae | *Chilocorus* | *Chilocorus nigritus* | 26 | KY847513 | BOLD:ABX2096 | Pakistan |
| Chilocorinae | *Chilocorus* | *Chilocorus nigritus* | 27 | KY842829 | BOLD:ABX2096 | Pakistan |
| Chilocorinae | *Chilocorus* | *Chilocorus nigritus* | 28 | KY831562 | BOLD:ABX2096 | Pakistan |

**Supplementary Table S3 (continued)**

| Subfamily | Genus | Species name | Code | GenBank accession number | BOLD BIN | Collection country |
|---|---|---|---|---|---|---|
| Chilocorinae | *Chilocorus* | *Chilocorus nigritus* | 29 | KX051943 | BOLD:ABX2096 | French Polynesia |
| Chilocorinae | *Chilocorus* | *Chilocorus nigritus* | 30 | KX051942 | BOLD:ABX2096 | French Polynesia |
| Chilocorinae | *Chilocorus* | *Chilocorus renipustulatus* | 31 | KJ964366 | BOLD:AAO1521 | Finland |
| Chilocorinae | *Chilocorus* | *Chilocorus renipustulatus* | 32 | KJ965157 | BOLD:AAO1521 | Finland |
| Chilocorinae | *Chilocorus* | *Chilocorus renipustulatus* | 33 | KJ962647 | BOLD:AAO1521 | Finland |
| Chilocorinae | *Chilocorus* | *Chilocorus renipustulatus* | 34 | KM442999 | BOLD:AAO1521 | Germany |
| Chilocorinae | *Chilocorus* | *Chilocorus renipustulatus* | 35 | KM444958 | BOLD:AAO1521 | Germany |
| Chilocorinae | *Chilocorus* | *Chilocorus renipustulatus* | 36 | KM452404 | BOLD:AAO1521 | Germany |
| Chilocorinae | *Chilocorus* | *Chilocorus renipustulatus* | 37 | KM450481 | BOLD:AAO1521 | Germany |
| Chilocorinae | *Chilocorus* | *Chilocorus renipustulatus* | 38 | KM439352 | BOLD:AAO1521 | Germany |
| Chilocorinae | *Chilocorus* | *Chilocorus renipustulatus* | 39 | KU912888 | BOLD:AAO1521 | Germany |
| Chilocorinae | *Chilocorus* | *Chilocorus renipustulatus* | 40 | KU915376 | BOLD:AAO1521 | Germany |
| Chilocorinae | *Chilocorus* | *Chilocorus renipustulatus* | 41 | KU909412 | BOLD:AAO1521 | Germany |
| Chilocorinae | *Chilocorus* | *Chilocorus renipustulatus* | 42 | KU917290 | BOLD:AAO1521 | Germany |
| Chilocorinae | *Chilocorus* | *Chilocorus stigma* | 43 | | BOLD:AAH3301 | Canada |
| Chilocorinae | *Chilocorus* | *Chilocorus stigma* | 44 | JF887492 | BOLD:AAH3301 | Canada |
| Chilocorinae | *Chilocorus* | *Chilocorus stigma* | 45 | KM843029 | BOLD:ACA6340 | Canada |
| Chilocorinae | *Chilocorus* | *Chilocorus stigma* | 46 | KM841886 | BOLD:AAH3301 | Canada |
| Chilocorinae | *Chilocorus* | *Chilocorus stigma* | 47 | KR484556 | BOLD:AAH3301 | Canada |
| Chilocorinae | *Exochomus* | *Exochomus nigromaculatus* | 48 | KM452108 | BOLD:ABA7750 | Germany |
| Chilocorinae | *Exochomus* | *Exochomus nigromaculatus* | 49 | KM451145 | BOLD:ABA7750 | Germany |
| Chilocorinae | *Platynaspis* | *Platynaspis luteorubra* | 50 | KM439449 | BOLD:ABU7107 | Germany |
| Chilocorinae | *Platynaspis* | *Platynaspis luteorubra* | 51 | KM449676 | BOLD:ABU7107 | Germany |
| Chilocorinae | *Platynaspis* | *Platynaspis luteorubra* | 52 | KM446263 | BOLD:ABU7107 | Germany |
| Chilocorinae | *Platynaspis* | *Platynaspis luteorubra* | 53 | KM441829 | BOLD:ABU7107 | Germany |
| Chilocorinae | *Platynaspis* | *Platynaspis luteorubra* | 54 | KM451017 | BOLD:ABU7107 | Germany |
| Chilocorinae | *Platynaspis* | *Platynaspis luteorubra* | 55 | KM451632 | BOLD:ABU7107 | Germany |
| Chilocorinae | *Platynaspis* | *Platynaspis luteorubra* | 56 | KU910757 | BOLD:ABU7107 | Germany |
| Chilocorinae | *Platynaspis* | *Platynaspis luteorubra* | 57 | KU908960 | BOLD:ABU7107 | Germany |
| Chilocorinae | *Platynaspis* | *Platynaspis luteorubra* | 58 | KU913036 | BOLD:ABU7107 | Germany |
| Chilocorinae | *Platynaspis* | *Platynaspis luteorubra* | 59 | KU916864 | BOLD:ABU7107 | Germany |

## Supplementary Table S3 (continued)

| Subfamily | Genus | Species name | Code | GenBank accession number | BOLD BIN | Collection country |
|---|---|---|---|---|---|---|
| Chilocorinae | *Platynaspis* | *Platynaspis luteorubra* | 60 | KU915137 | BOLD:ABU7107 | Germany |
| Chilocorinae | *Platynaspis* | *Platynaspis luteorubra* | 61 | KU911082 | BOLD:ABU7107 | Germany |
| Coccidulinae | *Coccidula* | *Coccidula rufa* | 62 | HM909060 | BOLD:AAJ4046 | Finland |
| Coccidulinae | *Coccidula* | *Coccidula rufa* | 63 | KJ963897 | BOLD:AAJ4046 | Finland |
| Coccidulinae | *Coccidula* | *Coccidula rufa* | 64 | KJ966050 | BOLD:AAJ4046 | Finland |
| Coccidulinae | *Coccidula* | *Coccidula rufa* | 65 | KJ964921 | BOLD:AAJ4046 | Finland |
| Coccidulinae | *Coccidula* | *Coccidula rufa* | 66 | HQ954057 | BOLD:AAJ4046 | Germany |
| Coccidulinae | *Coccidula* | *Coccidula rufa* | 67 | HQ954058 | BOLD:AAJ4046 | Germany |
| Coccidulinae | *Coccidula* | *Coccidula rufa* | 68 | KM440165 | BOLD:AAJ4046 | Germany |
| Coccidulinae | *Coccidula* | *Coccidula rufa* | 69 | KM451183 | BOLD:AAJ4046 | Germany |
| Coccidulinae | *Coccidula* | *Coccidula rufa* | 70 | KM449395 | BOLD:AAJ4046 | Germany |
| Coccidulinae | *Coccidula* | *Coccidula rufa* | 71 | KM448057 | BOLD:AAJ4046 | Germany |
| Coccidulinae | *Coccidula* | *Coccidula rufa* | 72 | KM450503 | BOLD:AAJ4046 | Germany |
| Coccidulinae | *Coccidula* | *Coccidula rufa* | 73 | KU916012 | BOLD:AAJ4046 | Germany |
| Coccidulinae | *Coccidula* | *Coccidula rufa* | 74 | KU913652 | BOLD:AAJ4046 | Germany |
| Coccidulinae | *Coccidula* | *Coccidula rufa* | 75 | KU913006 | BOLD:AAJ4046 | Germany |
| Coccidulinae | *Coccidula* | *Coccidula rufa* | 76 | KU916642 | BOLD:AAJ4046 | Germany |
| Coccidulinae | *Coccidula* | *Coccidula scutellata* | 77 | HQ559234 | BOLD:AAM0291 | Finland |
| Coccidulinae | *Coccidula* | *Coccidula scutellata* | 78 | KJ965128 | BOLD:AAM0291 | Finland |
| Coccidulinae | *Coccidula* | *Coccidula scutellata* | 79 | KJ966202 | BOLD:AAM0291 | Finland |
| Coccidulinae | *Coccidula* | *Coccidula scutellata* | 80 | KJ964995 | BOLD:AAM0291 | Finland |
| Coccidulinae | *Coccidula* | *Coccidula scutellata* | 81 | KJ967069 | BOLD:AAM0291 | Finland |
| Coccidulinae | *Coccidula* | *Coccidula scutellata* | 82 | JF889801 | BOLD:AAM0291 | Germany |
| Coccidulinae | *Coccidula* | *Coccidula scutellata* | 83 | KM450555 | BOLD:AAM0291 | Germany |
| Coccidulinae | *Coccidula* | *Coccidula scutellata* | 84 | KM443382 | BOLD:AAM0291 | Germany |
| Coccidulinae | *Rhyzobius* | *Rhyzobius chrysomeloides* | 85 | HQ953831 | BOLD:AAO1169 | Belgium |
| Coccidulinae | *Rhyzobius* | *Rhyzobius chrysomeloides* | 86 | KM449740 | BOLD:AAO1169 | Germany |
| Coccidulinae | *Rhyzobius* | *Rhyzobius chrysomeloides* | 87 | KM444065 | BOLD:AAO1169 | Germany |
| Coccidulinae | *Rhyzobius* | *Rhyzobius chrysomeloides* | 88 | KU916517 | BOLD:AAO1169 | Germany |
| Coccidulinae | *Rhyzobius* | *Rhyzobius chrysomeloides* | 89 | KU914541 | BOLD:AAO1169 | Germany |
| Coccidulinae | *Rhyzobius* | *Rhyzobius chrysomeloides* | 90 | KU907902 | BOLD:AAO1169 | Germany |

## Supplementary Table S3 (continued)

| Subfamily | Genus | Species name | Code | GenBank accession number | BOLD BIN | Collection country |
|---|---|---|---|---|---|---|
| Coccidulinae | *Rhyzobius* | *Rhyzobius chrysomeloides* | 91 | KU908172 | BOLD:AAO1169 | Germany |
| Coccidulinae | *Rhyzobius* | *Rhyzobius chrysomeloides* | 92 | KU916728 | BOLD:AAO1169 | Germany |
| Coccidulinae | *Rhyzobius* | *Rhyzobius chrysomeloides* | 93 | KU914339 | BOLD:AAO1169 | Germany |
| Coccidulinae | *Rhyzobius* | *Rhyzobius chrysomeloides* | 94 | KU911679 | BOLD:AAO1169 | Germany |
| Coccidulinae | *Rhyzobius* | *Rhyzobius chrysomeloides* | 95 | KU917845 | BOLD:AAO1169 | Germany |
| Coccidulinae | *Rhyzobius* | *Rhyzobius litura* | 96 | KU919344 | BOLD:AAZ4378 | Germany |
| Coccidulinae | *Rhyzobius* | *Rhyzobius litura* | 97 | KU915832 | BOLD:AAZ4378 | Germany |
| Coccinellinae | *Adalia* | *Adalia bipunctata* | 98 | JN290468 | BOLD:AAH3288 | Canada |
| Coccinellinae | *Adalia* | *Adalia bipunctata* | 99 | JF888334 | BOLD:ACE3479 | Canada |
| Coccinellinae | *Adalia* | *Adalia bipunctata* | 100 | KJ963219 | BOLD:AAH3316 | Finland |
| Coccinellinae | *Adalia* | *Adalia bipunctata* | 101 | KJ964535 | BOLD:AAH3316 | Finland |
| Coccinellinae | *Adalia* | *Adalia bipunctata* | 102 | KJ964290 | BOLD:AAH3316 | Finland |
| Coccinellinae | *Adalia* | *Adalia bipunctata* | 103 | KM442127 | BOLD:AAH3316 | Germany |
| Coccinellinae | *Adalia* | *Adalia bipunctata* | 104 | KM439180 | BOLD:AAH3316 | Germany |
| Coccinellinae | *Adalia* | *Adalia bipunctata* | 105 | KM450179 | BOLD:ACE3479 | Germany |
| Coccinellinae | *Adalia* | *Adalia bipunctata* | 106 | KM441776 | BOLD:AAH3316 | Iran |
| Coccinellinae | *Adalia* | *Adalia bipunctata* | 107 | KM449309 | BOLD:AAH3316 | Iran |
| Coccinellinae | *Adalia* | *Adalia bipunctata* | 108 | KM439489 | BOLD:AAH3316 | Iran |
| Coccinellinae | *Adalia* | *Adalia bipunctata* | 109 | KM444477 | BOLD:AAH3316 | Iran |
| Coccinellinae | *Adalia* | *Adalia decempunctata* | 110 | KJ966482 | BOLD:AAJ5695 | Finland |
| Coccinellinae | *Adalia* | *Adalia decempunctata* | 111 | KJ961723 | BOLD:AAJ5695 | Sweden |
| Coccinellinae | *Adalia* | *Adalia decempunctata* | 112 | KJ965563 | BOLD:AAJ5695 | Sweden |
| Coccinellinae | *Adalia* | *Adalia decempunctata* | 113 | KM449519 | BOLD:AAJ5695 | Germany |
| Coccinellinae | *Adalia* | *Adalia decempunctata* | 114 | KM443613 | BOLD:AAJ5695 | Germany |
| Coccinellinae | *Adalia* | *Adalia decempunctata* | 115 | KM445658 | BOLD:AAJ5695 | Germany |
| Coccinellinae | *Adalia* | *Adalia decempunctata* | 116 | KM444763 | BOLD:AAJ5695 | Germany |
| Coccinellinae | *Adalia* | *Adalia decempunctata* | 117 | KM443708 | BOLD:AAJ5695 | Germany |
| Coccinellinae | *Adalia* | *Adalia decempunctata* | 118 | KU910455 | BOLD:AAJ5695 | Germany |
| Coccinellinae | *Adalia* | *Adalia decempunctata* | 119 | KU914508 | BOLD:AAJ5695 | Germany |
| Coccinellinae | *Adalia* | *Adalia decempunctata* | 120 | KU907424 | BOLD:AAJ5695 | Germany |
| Coccinellinae | *Adalia* | *Adalia decempunctata* | 121 | KU917463 | BOLD:AAJ5695 | Germany |

## Supplementary Table S3 (continued)

| Subfamily | Genus | Species name | Code | GenBank accession number | BOLD BIN | Collection country |
|---|---|---|---|---|---|---|
| Coccinellinae | *Adalia* | *Adalia decempunctata* | 122 | KU918545 | BOLD:AAJ5695 | Germany |
| Coccinellinae | *Anatis* | *Anatis labiculata* | 123 | JN290517 | BOLD:AAF3857 | Canada |
| Coccinellinae | *Anatis* | *Anatis labiculata* | 124 | JN290520 | BOLD:AAF3857 | Canada |
| Coccinellinae | *Anatis* | *Anatis labiculata* | 125 | KR489303 | BOLD:AAF3857 | Canada |
| Coccinellinae | *Anatis* | *Anatis labiculata* | 126 | KR482454 | BOLD:AAF3857 | Canada |
| Coccinellinae | *Anatis* | *Anatis labiculata* | 127 | KM849275 | BOLD:AAF3857 | Canada |
| Coccinellinae | *Anatis* | *Anatis mali* | 128 | HM411934 | BOLD:AAH3318 | Canada |
| Coccinellinae | *Anatis* | *Anatis mali* | 129 | HQ551583 | BOLD:AAH3318 | Canada |
| Coccinellinae | *Anatis* | *Anatis mali* | 130 | KM841891 | BOLD:AAH3318 | Canada |
| Coccinellinae | *Anatis* | *Anatis mali* | 131 | KU874125 | BOLD:AAH3318 | Canada |
| Coccinellinae | *Anatis* | *Anatis ocellata* | 132 | KJ964254 | BOLD:ABW4603 | Finland |
| Coccinellinae | *Anatis* | *Anatis ocellata* | 133 | KJ962284 | BOLD:ABW4603 | Finland |
| Coccinellinae | *Anatis* | *Anatis ocellata* | 134 | KJ963933 | BOLD:ABW4603 | Finland |
| Coccinellinae | *Anatis* | *Anatis ocellata* | 135 | KM451872 | BOLD:ABW4603 | Germany |
| Coccinellinae | *Anatis* | *Anatis ocellata* | 136 | KM449972 | BOLD:ABW4603 | Belgium |
| Coccinellinae | *Anatis* | *Anatis ocellata* | 137 | KM446379 | BOLD:ABW4603 | Germany |
| Coccinellinae | *Anatis* | *Anatis ocellata* | 138 | KM446888 | BOLD:ABW4603 | Germany |
| Coccinellinae | *Anatis* | *Anatis ocellata* | 139 | KM439523 | BOLD:ABW4603 | Germany |
| Coccinellinae | *Anatis* | *Anatis ocellata* | 140 | KU907798 | BOLD:ABW4603 | Germany |
| Coccinellinae | *Anatis* | *Anatis ocellata* | 141 | KU918775 | BOLD:ABW4603 | Germany |
| Coccinellinae | *Anatis* | *Anatis ocellata* | 142 | KU910382 | BOLD:ABW4603 | Germany |
| Coccinellinae | *Anatis* | *Anatis ocellata* | 143 | KU913592 | BOLD:ABW4603 | Germany |
| Coccinellinae | *Anatis* | *Anatis ocellata* | 144 | KU912390 | BOLD:ABW4603 | Germany |
| Coccinellinae | *Anatis* | *Anatis ocellata* | 145 | KU916646 | BOLD:ABW4603 | Germany |
| Coccinellinae | *Anatis* | *Anatis ocellata* | 146 | KU908130 | BOLD:ABW4603 | Germany |
| Coccinellinae | *Anisosticta* | *Anisosticta bitriangularis* | 147 | GU679565 | BOLD:AAH3287 | Canada |
| Coccinellinae | *Anisosticta* | *Anisosticta bitriangularis* | 148 | KJ203950 | BOLD:AAH3287 | Canada |
| Coccinellinae | *Anisosticta* | *Anisosticta bitriangularis* | 149 | KJ203715 | BOLD:AAH3287 | Canada |
| Coccinellinae | *Anisosticta* | *Anisosticta bitriangularis* | 150 | KJ202751 | BOLD:AAH3287 | Canada |
| Coccinellinae | *Anisosticta* | *Anisosticta bitriangularis* | 151 | KJ203739 | BOLD:AAH3287 | Canada |
| Coccinellinae | *Anisosticta* | *Anisosticta bitriangularis* | 152 | KR486462 | BOLD:AAH3287 | Canada |

## Supplementary Table S3 (continued)

| Subfamily | Genus | Species name | Code | GenBank accession number | BOLD BIN | Collection country |
|---|---|---|---|---|---|---|
| Coccinellinae | *Anisosticta* | *Anisosticta bitriangularis* | 153 | HQ989876 | BOLD:AAH3287 | Canada |
| Coccinellinae | *Anisosticta* | *Anisosticta bitriangularis* | 154 | KJ203709 | BOLD:AAH3287 | Canada |
| Coccinellinae | *Anisosticta* | *Anisosticta bitriangularis* | 155 | KJ204025 | BOLD:AAH3287 | Canada |
| Coccinellinae | *Anisosticta* | *Anisosticta bitriangularis* | 156 | KM843162 | BOLD:AAH3287 | Canada |
| Coccinellinae | *Anisosticta* | *Anisosticta bitriangularis* | 157 | JN265286 | BOLD:AAH3287 | Canada |
| Coccinellinae | *Anisosticta* | *Anisosticta bitriangularis* | 158 | KU874130 | BOLD:AAH3287 | United States |
| Coccinellinae | *Anisosticta* | *Anisosticta novemdecimpunctata* | 159 | KJ967187 | BOLD:AAZ4374 | Finland |
| Coccinellinae | *Anisosticta* | *Anisosticta novemdecimpunctata* | 160 | KJ961873 | BOLD:AAZ4374 | Finland |
| Coccinellinae | *Anisosticta* | *Anisosticta novemdecimpunctata* | 161 | KJ962106 | BOLD:AAZ4374 | Finland |
| Coccinellinae | *Anisosticta* | *Anisosticta novemdecimpunctata* | 162 | KJ966548 | BOLD:AAZ4374 | Finland |
| Coccinellinae | *Anisosticta* | *Anisosticta novemdecimpunctata* | 163 | KJ965639 | BOLD:AAZ4374 | Finland |
| Coccinellinae | *Anisosticta* | *Anisosticta novemdecimpunctata* | 164 | KM450655 | BOLD:AAZ4374 | Germany |
| Coccinellinae | *Anisosticta* | *Anisosticta novemdecimpunctata* | 165 | KM450614 | BOLD:AAZ4374 | Germany |
| Coccinellinae | *Anisosticta* | *Anisosticta novemdecimpunctata* | 166 | KM445688 | BOLD:AAZ4374 | Germany |
| Coccinellinae | *Anisosticta* | *Anisosticta novemdecimpunctata* | 167 | KM451409 | BOLD:AAZ4374 | Germany |
| Coccinellinae | *Anisosticta* | *Anisosticta novemdecimpunctata* | 168 | KU906502 | BOLD:AAZ4374 | Germany |
| Coccinellinae | *Anisosticta* | *Anisosticta novemdecimpunctata* | 169 | KU909006 | BOLD:AAZ4374 | Germany |
| Coccinellinae | *Anisosticta* | *Anisosticta novemdecimpunctata* | 170 | KU906170 | BOLD:AAZ4374 | Germany |
| Coccinellinae | *Anisosticta* | *Anisosticta novemdecimpunctata* | 171 | KU916822 | BOLD:AAZ4374 | Germany |
| Coccinellinae | *Anisosticta* | *Anisosticta novemdecimpunctata* | 172 | KU911310 | BOLD:AAZ4374 | Germany |
| Coccinellinae | *Anisosticta* | *Anisosticta novemdecimpunctata* | 173 | KU909892 | BOLD:AAZ4374 | Germany |
| Coccinellinae | *Anisosticta* | *Anisosticta novemdecimpunctata* | 174 | KU907678 | BOLD:AAZ4374 | Germany |
| Coccinellinae | *Aphidecta* | *Aphidecta obliterata* | 175 | HM909101 | BOLD:AAK2029 | Finland |
| Coccinellinae | *Aphidecta* | *Aphidecta obliterata* | 176 | KJ966619 | BOLD:AAK2029 | Finland |
| Coccinellinae | *Aphidecta* | *Aphidecta obliterata* | 177 | KJ966356 | BOLD:AAK2029 | Finland |
| Coccinellinae | *Aphidecta* | *Aphidecta obliterata* | 178 | KJ963033 | BOLD:AAK2029 | Finland |
| Coccinellinae | *Aphidecta* | *Aphidecta obliterata* | 179 | JF889849 | BOLD:AAK2029 | Germany |
| Coccinellinae | *Aphidecta* | *Aphidecta obliterata* | 180 | KM443018 | BOLD:AAK2029 | Germany |
| Coccinellinae | *Aphidecta* | *Aphidecta obliterata* | 181 | KM452624 | BOLD:AAK2029 | Germany |
| Coccinellinae | *Aphidecta* | *Aphidecta obliterata* | 182 | KM446232 | BOLD:AAK2029 | Germany |
| Coccinellinae | *Aphidecta* | *Aphidecta obliterata* | 183 | KM448426 | BOLD:AAK2029 | Germany |

**Supplementary Table S3 (continued)**

| Subfamily | Genus | Species name | Code | GenBank accession number | BOLD BIN | Collection country |
|---|---|---|---|---|---|---|
| Coccinellinae | *Aphidecta* | *Aphidecta obliterata* | 184 | KM451253 | BOLD:AAK2029 | Germany |
| Coccinellinae | *Aphidecta* | *Aphidecta obliterata* | 185 | KM443587 | BOLD:AAK2029 | Germany |
| Coccinellinae | *Aphidecta* | *Aphidecta obliterata* | 186 | KM443156 | BOLD:AAK2029 | Germany |
| Coccinellinae | *Calvia* | *Calvia decemguttata* | 187 | JF889620 | BOLD:AAO0118 | Germany |
| Coccinellinae | *Calvia* | *Calvia decemguttata* | 188 | KM446285 | BOLD:AAO0118 | Germany |
| Coccinellinae | *Calvia* | *Calvia decemguttata* | 189 | KM444412 | BOLD:AAO0118 | Germany |
| Coccinellinae | *Calvia* | *Calvia decemguttata* | 190 | KM441002 | BOLD:AAO0118 | Germany |
| Coccinellinae | *Calvia* | *Calvia decemguttata* | 191 | KM439339 | BOLD:AAO0118 | Germany |
| Coccinellinae | *Calvia* | *Calvia decemguttata* | 192 | KM447884 | BOLD:AAO0118 | Germany |
| Coccinellinae | *Calvia* | *Calvia decemguttata* | 193 | KM444073 | BOLD:AAO0118 | Germany |
| Coccinellinae | *Calvia* | *Calvia decemguttata* | 194 | KM448469 | BOLD:AAO0118 | Germany |
| Coccinellinae | *Calvia* | *Calvia decemguttata* | 195 | KU915776 | BOLD:ACS2677 | Germany |
| Coccinellinae | *Calvia* | *Calvia decemguttata* | 196 | KU913681 | BOLD:AAO0118 | Germany |
| Coccinellinae | *Calvia* | *Calvia decemguttata* | 197 | KU918567 | BOLD:AAO0118 | Germany |
| Coccinellinae | *Calvia* | *Calvia decemguttata* | 198 | KU913668 | BOLD:AAO0118 | Germany |
| Coccinellinae | *Calvia* | *Calvia decemguttata* | 199 | KU907102 | BOLD:ACS2677 | Germany |
| Coccinellinae | *Calvia* | *Calvia decemguttata* | 200 | KU909985 | BOLD:ACS2677 | Germany |
| Coccinellinae | *Calvia* | *Calvia quatuordecimguttata* | 201 | JF888336 | BOLD:AAH3295 | Canada |
| Coccinellinae | *Calvia* | *Calvia quatuordecimguttata* | 202 | JF888337 | BOLD:AAH3295 | Canada |
| Coccinellinae | *Calvia* | *Calvia quatuordecimguttata* | 203 | KJ962673 | BOLD:ABY9024 | Finland |
| Coccinellinae | *Calvia* | *Calvia quatuordecimguttata* | 204 | KM449347 | BOLD:ABY9024 | Germany |
| Coccinellinae | *Calvia* | *Calvia quatuordecimguttata* | 205 | KM439644 | BOLD:ABY9024 | Germany |
| Coccinellinae | *Calvia* | *Calvia quatuordecimguttata* | 206 | KU906531 | BOLD:AAN3361 | Germany |
| Coccinellinae | *Calvia* | *Calvia quatuordecimguttata* | 207 | KU907607 | BOLD:AAN3361 | Germany |
| Coccinellinae | *Calvia* | *Calvia quatuordecimguttata* | 208 | KU911885 | BOLD:ABY9024 | Germany |
| Coccinellinae | *Calvia* | *Calvia quatuordecimguttata* | 209 | KU911831 | BOLD:AAN3361 | Germany |
| Coccinellinae | *Calvia* | *Calvia quatuordecimguttata* | 210 | KM843504 | BOLD:AAH3295 | Canada |
| Coccinellinae | *Calvia* | *Calvia quatuordecimguttata* | 211 | KM850960 | BOLD:AAH3295 | Canada |
| Coccinellinae | *Calvia* | *Calvia quatuordecimguttata* | 212 | KU874521 | BOLD:AAH3295 | United States |
| Coccinellinae | *Calvia* | *Calvia quatuordecimguttata* | 213 | KU874522 | BOLD:AAH3295 | United States |
| Coccinellinae | *Calvia* | *Calvia quatuordecimguttata* | 214 | JN290458 | BOLD:AAH3295 | Canada |

**Supplementary Table S3 (continued)**

| Subfamily | Genus | Species name | Code | GenBank accession number | BOLD BIN | Collection country |
|---|---|---|---|---|---|---|
| Coccinellinae | *Calvia* | *Calvia quatuordecimguttata* | 215 | JF888335 | BOLD:AAH3295 | Canada |
| Coccinellinae | *Calvia* | *Calvia quatuordecimguttata* | 216 | KJ965619 | BOLD:AAN3361 | Finland |
| Coccinellinae | *Calvia* | *Calvia quatuordecimguttata* | 217 | KU914685 | BOLD:ABY9024 | Germany |
| Coccinellinae | *Calvia* | *Calvia quatuordecimguttata* | 218 | KU912785 | BOLD:ABY9024 | Germany |
| Coccinellinae | *Ceratomegilla* | *Ceratomegilla ulkei* | 219 | KJ202950 | BOLD:AAH3291 | Canada |
| Coccinellinae | *Ceratomegilla* | *Ceratomegilla ulkei* | 220 | KJ202743 | BOLD:AAH3291 | Canada |
| Coccinellinae | *Ceratomegilla* | *Ceratomegilla ulkei* | 221 | KJ202768 | BOLD:AAH3291 | Canada |
| Coccinellinae | *Ceratomegilla* | *Ceratomegilla ulkei* | 222 | HM432924 | BOLD:AAH3289 | Canada |
| Coccinellinae | *Ceratomegilla* | *Ceratomegilla ulkei* | 223 | HM432925 | BOLD:AAH3289 | Canada |
| Coccinellinae | *Cheilomenes* | *Cheilomenes sexmaculata* | 224 | KM207085 | BOLD:ACM3340 | China |
| Coccinellinae | *Coccinella* | *Coccinella hieroglyphica* | 225 | KJ966585 | BOLD:ACE2719 | Finland |
| Coccinellinae | *Coccinella* | *Coccinella hieroglyphica* | 226 | KM448934 | BOLD:ABU6461 | Germany |
| Coccinellinae | *Coccinella* | *Coccinella hieroglyphica* | 227 | KU911785 | BOLD:ACE2719 | Germany |
| Coccinellinae | *Coccinella* | *Coccinella magnifica* | 228 | KM439619 | BOLD:ACA7861 | Germany |
| Coccinellinae | *Coccinella* | *Coccinella magnifica* | 229 | KM449697 | BOLD:ACA7861 | Germany |
| Coccinellinae | *Coccinella* | *Coccinella magnifica* | 230 | KU916547 | BOLD:ACA7861 | Germany |
| Coccinellinae | *Coccinella* | *Coccinella magnifica* | 231 | KU916434 | BOLD:ACA7861 | Germany |
| Coccinellinae | *Coccinella* | *Coccinella quinquepunctata* | 232 | KJ964993 | BOLD:ACK9088 | Finland |
| Coccinellinae | *Coccinella* | *Coccinella quinquepunctata* | 233 | KM445875 | BOLD:ABZ1369 | Finland |
| Coccinellinae | *Coccinella* | *Coccinella quinquepunctata* | 234 | KM446568 | BOLD:ABZ1369 | Germany |
| Coccinellinae | *Coccinella* | *Coccinella quinquepunctata* | 235 | KU909371 | BOLD:ABU6461 | Germany |
| Coccinellinae | *Coccinella* | *Coccinella quinquepunctata* | 236 | KU907168 | BOLD:ABU6461 | Germany |
| Coccinellinae | *Coccinella* | *Coccinella quinquepunctata* | 237 | KU911642 | BOLD:ABU6461 | Germany |
| Coccinellinae | *Coccinella* | *Coccinella quinquepunctata* | 238 | KU918783 | BOLD:ABU6461 | Germany |
| Coccinellinae | *Coccinella* | *Coccinella quinquepunctata* | 239 | KU914152 | BOLD:ABU6461 | Italy |
| Coccinellinae | *Coccinella* | *Coccinella quinquepunctata* | 240 | KU910692 | BOLD:ABU6461 | Germany |
| Coccinellinae | *Coccinella* | *Coccinella septempunctata* | 241 | | BOLD:AAA8933 | United States |
| Coccinellinae | *Coccinella* | *Coccinella septempunctata* | 242 | | BOLD:AAA8933 | United States |
| Coccinellinae | *Coccinella* | *Coccinella septempunctata* | 243 | | BOLD:AAA8933 | United States |
| Coccinellinae | *Coccinella* | *Coccinella septempunctata* | 244 | | BOLD:AAA8933 | United States |
| Coccinellinae | *Coccinella* | *Coccinella septempunctata* | 245 | | BOLD:AAA8933 | United States |

**Supplementary Table S3 (continued)**

| Subfamily | Genus | Species name | Code | GenBank accession number | BOLD BIN | Collection country |
|---|---|---|---|---|---|---|
| Coccinellinae | *Coccinella* | *Coccinella septempunctata* | 246 | JN290459 | BOLD:AAA8933 | Canada |
| Coccinellinae | *Coccinella* | *Coccinella septempunctata* | 247 | JF888320 | BOLD:AAA8933 | Canada |
| Coccinellinae | *Coccinella* | *Coccinella septempunctata* | 248 | JF888321 | BOLD:AAA8933 | Canada |
| Coccinellinae | *Coccinella* | *Coccinella septempunctata* | 249 | JF888322 | BOLD:AAA8933 | Canada |
| Coccinellinae | *Coccinella* | *Coccinella septempunctata* | 250 | JF888323 | BOLD:AAA8933 | Canada |
| Coccinellinae | *Coccinella* | *Coccinella septempunctata* | 251 | KJ962275 | BOLD:AAA8933 | Finland |
| Coccinellinae | *Coccinella* | *Coccinella septempunctata* | 252 | KJ961952 | BOLD:AAA8933 | Finland |
| Coccinellinae | *Coccinella* | *Coccinella septempunctata* | 253 | KM442471 | BOLD:AAA8933 | Germany |
| Coccinellinae | *Coccinella* | *Coccinella septempunctata* | 254 | KM439459 | BOLD:AAA8933 | Germany |
| Coccinellinae | *Coccinella* | *Coccinella septempunctata* | 255 | KM442483 | BOLD:AAA8933 | Germany |
| Coccinellinae | *Coccinella* | *Coccinella septempunctata* | 256 | KM440740 | BOLD:AAA8933 | Germany |
| Coccinellinae | *Coccinella* | *Coccinella transversalis* | 257 | | BOLD:AAH3297 | Australia |
| Coccinellinae | *Coccinella* | *Coccinella transversalis* | 258 | KY838208 | BOLD:AAH3297 | Pakistan |
| Coccinellinae | *Coccinella* | *Coccinella transversalis* | 259 | | BOLD:AAH3297 | Australia |
| Coccinellinae | *Coccinella* | *Coccinella transversalis* | 260 | KX052277 | BOLD:AAH3297 | French Polynesia |
| Coccinellinae | *Coccinella* | *Coccinella transversalis* | 261 | KX052278 | BOLD:AAH3297 | French Polynesia |
| Coccinellinae | *Coccinella* | *Coccinella transversalis* | 262 | KX052280 | BOLD:AAH3297 | French Polynesia |
| Coccinellinae | *Coccinella* | *Coccinella transversalis* | 263 | KX052281 | BOLD:AAH3297 | French Polynesia |
| Coccinellinae | *Coccinella* | *Coccinella transversalis* | 264 | KX052279 | BOLD:AAH3297 | French Polynesia |
| Coccinellinae | *Coccinella* | *Coccinella transversoguttata* | 265 | KU874657 | BOLD:AAH3315 | United States |
| Coccinellinae | *Coccinella* | *Coccinella transversoguttata* | 266 | KU874658 | BOLD:AAH3315 | United States |
| Coccinellinae | *Coccinella* | *Coccinella transversoguttata* | 267 | KU374414 | BOLD:AAH3315 | Greenland |
| Coccinellinae | *Coccinella* | *Coccinella transversoguttata* | 268 | KU374604 | BOLD:AAH3315 | Greenland |
| Coccinellinae | *Coccinella* | *Coccinella transversoguttata* | 269 | KU374407 | BOLD:AAH3315 | Greenland |
| Coccinellinae | *Coccinella* | *Coccinella transversoguttata* | 270 | KU373350 | BOLD:AAH3315 | Greenland |
| Coccinellinae | *Coccinella* | *Coccinella transversoguttata* | 271 | KU374561 | BOLD:AAH3315 | Greenland |
| Coccinellinae | *Coccinella* | *Coccinella trifasciata* | 272 | HM411861 | BOLD:AAE2547 | Canada |
| Coccinellinae | *Coccinella* | *Coccinella trifasciata* | 273 | KJ962029 | BOLD:ACI3513 | Finland |
| Coccinellinae | *Coccinella* | *Coccinella trifasciata* | 274 | KR482491 | BOLD:AAE2547 | Canada |
| Coccinellinae | *Coccinella* | *Coccinella trifasciata* | 275 | KR481746 | BOLD:AAE2547 | Canada |
| Coccinellinae | *Coccinella* | *Coccinella trifasciata* | 276 | KM845070 | BOLD:AAE2547 | Canada |

**Supplementary Table S3 (continued)**

| Subfamily | Genus | Species name | Code | GenBank accession number | BOLD BIN | Collection country |
|---|---|---|---|---|---|---|
| Coccinellinae | *Coccinella* | *Coccinella trifasciata* | 277 | KM844996 | BOLD:AAE2547 | Canada |
| Coccinellinae | *Coccinella* | *Coccinella trifasciata* | 278 | KM842213 | BOLD:AAE2547 | Canada |
| Coccinellinae | *Coccinella* | *Coccinella trifasciata* | 279 | KU874660 | BOLD:AAE2547 | United States |
| Coccinellinae | *Coccinella* | *Coccinella trifasciata* | 280 | | BOLD:AAE2547 | Canada |
| Coccinellinae | *Coccinella* | *Coccinella trifasciata* | 281 | HQ551599 | BOLD:AAE2547 | Canada |
| Coccinellinae | *Coccinella* | *Coccinella undecimpunctata* | 282 | KJ963757 | BOLD:AAP7476 | Finland |
| Coccinellinae | *Coccinella* | *Coccinella undecimpunctata* | 283 | KJ964536 | BOLD:AAP7476 | Russia |
| Coccinellinae | *Coccinella* | *Coccinella undecimpunctata* | 284 | KJ965061 | BOLD:AAP7476 | Finland |
| Coccinellinae | *Coccinella* | *Coccinella undecimpunctata* | 285 | KJ965514 | BOLD:AAP7476 | Sweden |
| Coccinellinae | *Coccinella* | *Coccinella undecimpunctata* | 286 | KJ965686 | BOLD:AAP7476 | Finland |
| Coccinellinae | *Coccinella* | *Coccinella undecimpunctata* | 287 | JF889781 | BOLD:AAP7476 | Germany |
| Coccinellinae | *Coccinella* | *Coccinella undecimpunctata* | 288 | JF889782 | BOLD:AAP7476 | Germany |
| Coccinellinae | *Coccinella* | *Coccinella undecimpunctata* | 289 | KM443366 | BOLD:AAP7476 | Germany |
| Coccinellinae | *Coccinella* | *Coccinella undecimpunctata* | 290 | KM451106 | BOLD:AAP7476 | Germany |
| Coccinellinae | *Coccinella* | *Coccinella undecimpunctata* | 291 | KM452280 | BOLD:AAP7476 | Germany |
| Coccinellinae | *Coccinula* | *Coccinula quatuordecimpustulata* | 292 | KJ962244 | BOLD:AAJ4056 | Finland |
| Coccinellinae | *Coccinula* | *Coccinula quatuordecimpustulata* | 293 | KJ964358 | BOLD:AAJ4056 | Finland |
| Coccinellinae | *Coccinula* | *Coccinula quatuordecimpustulata* | 294 | KJ961753 | BOLD:AAJ4056 | Estonia |
| Coccinellinae | *Coccinula* | *Coccinula quatuordecimpustulata* | 295 | KJ963201 | BOLD:AAJ4056 | Finland |
| Coccinellinae | *Coccinula* | *Coccinula quatuordecimpustulata* | 296 | HQ953944 | BOLD:AAJ4056 | Germany |
| Coccinellinae | *Coccinula* | *Coccinula quatuordecimpustulata* | 297 | HQ953945 | BOLD:AAJ4056 | Germany |
| Coccinellinae | *Coccinula* | *Coccinula quatuordecimpustulata* | 298 | KM439174 | BOLD:AAJ4056 | Germany |
| Coccinellinae | *Coccinula* | *Coccinula quatuordecimpustulata* | 299 | KM446373 | BOLD:AAJ4056 | Germany |
| Coccinellinae | *Coccinula* | *Coccinula quatuordecimpustulata* | 300 | KM451941 | BOLD:AAJ4056 | Germany |
| Coccinellinae | *Coccinula* | *Coccinula quatuordecimpustulata* | 301 | KM442011 | BOLD:AAJ4056 | Germany |
| Coccinellinae | *Coccinula* | *Coccinula quatuordecimpustulata* | 302 | KM442070 | BOLD:AAJ4056 | Germany |
| Coccinellinae | *Coccinula* | *Coccinula quatuordecimpustulata* | 303 | KM452155 | BOLD:AAJ4056 | Germany |
| Coccinellinae | *Coccinula* | *Coccinula quatuordecimpustulata* | 304 | KM440045 | BOLD:AAJ4056 | Germany |
| Coccinellinae | *Coccinula* | *Coccinula quatuordecimpustulata* | 305 | KM442499 | BOLD:AAJ4056 | Germany |
| Coccinellinae | *Coccinula* | *Coccinula quatuordecimpustulata* | 306 | KU911323 | BOLD:AAJ4056 | Germany |
| Coccinellinae | *Coccinula* | *Coccinula quatuordecimpustulata* | 307 | KU918804 | BOLD:AAJ4056 | Germany |

## Supplementary Table S3 (continued)

| Subfamily | Genus | Species name | Code | GenBank accession number | BOLD BIN | Collection country |
|---|---|---|---|---|---|---|
| Coccinellinae | *Coccinula* | *Coccinula quatuordecimpustulata* | 308 | KU913339 | BOLD:AAJ4056 | Germany |
| Coccinellinae | *Coccinula* | *Coccinula quatuordecimpustulata* | 309 | KU918472 | BOLD:AAJ4056 | Germany |
| Coccinellinae | *Coelophora* | *Coelophora inaequalis* | 310 | | BOLD:AAH3296 | Australia |
| Coccinellinae | *Coelophora* | *Coelophora inaequalis* | 311 | | BOLD:AAH3296 | Australia |
| Coccinellinae | *Coelophora* | *Coelophora inaequalis* | 312 | | BOLD:AAH3296 | Australia |
| Coccinellinae | *Coelophora* | *Coelophora inaequalis* | 313 | | BOLD:AAH3296 | Australia |
| Coccinellinae | *Coelophora* | *Coelophora inaequalis* | 314 | | BOLD:AAH3296 | Australia |
| Coccinellinae | *Coelophora* | *Coelophora inaequalis* | 315 | | BOLD:AAH3296 | Australia |
| Coccinellinae | *Coelophora* | *Coelophora inaequalis* | 316 | | BOLD:AAH3296 | Australia |
| Coccinellinae | *Coelophora* | *Coelophora inaequalis* | 317 | | BOLD:AAH3296 | Australia |
| Coccinellinae | *Coelophora* | *Coelophora inaequalis* | 318 | | BOLD:AAH3296 | Australia |
| Coccinellinae | *Coelophora* | *Coelophora inaequalis* | 319 | KX052318 | BOLD:AAH3296 | French Polynesia |
| Coccinellinae | *Coelophora* | *Coelophora inaequalis* | 320 | KX052317 | BOLD:AAH3296 | French Polynesia |
| Coccinellinae | *Coelophora* | *Coelophora inaequalis* | 321 | KX052322 | BOLD:AAH3296 | French Polynesia |
| Coccinellinae | *Coelophora* | *Coelophora inaequalis* | 322 | KX052316 | BOLD:AAH3296 | French Polynesia |
| Coccinellinae | *Coelophora* | *Coelophora inaequalis* | 323 | | BOLD:AAH3296 | Australia |
| Coccinellinae | *Coelophora* | *Coelophora inaequalis* | 324 | | BOLD:AAH3296 | Australia |
| Coccinellinae | *Coelophora* | *Coelophora inaequalis* | 325 | | BOLD:AAH3296 | Australia |
| Coccinellinae | *Coelophora* | *Coelophora inaequalis* | 326 | | BOLD:AAH3296 | Australia |
| Coccinellinae | *Coleomegilla* | *Coleomegilla maculata* | 327 | | BOLD:AAD7604 | United States |
| Coccinellinae | *Coleomegilla* | *Coleomegilla maculata* | 328 | HM905915 | BOLD:AAD7604 | Canada |
| Coccinellinae | *Coleomegilla* | *Coleomegilla maculata* | 329 | KR490674 | BOLD:AAD7604 | Canada |
| Coccinellinae | *Coleomegilla* | *Coleomegilla maculata* | 330 | KR486191 | BOLD:AAD7604 | Canada |
| Coccinellinae | *Coleomegilla* | *Coleomegilla maculata* | 331 | KR483156 | BOLD:AAD7604 | Canada |
| Coccinellinae | *Coleomegilla* | *Coleomegilla maculata* | 332 | JF296236 | BOLD:AAD7604 | |
| Coccinellinae | *Coleomegilla* | *Coleomegilla maculata* | 333 | KR481048 | BOLD:AAD7604 | Canada |
| Coccinellinae | *Coleomegilla* | *Coleomegilla maculata* | 334 | KR489878 | BOLD:AAD7604 | Canada |
| Coccinellinae | *Coleomegilla* | *Coleomegilla maculata* | 335 | KR486311 | BOLD:AAD7604 | Canada |
| Coccinellinae | *Coleomegilla* | *Coleomegilla maculata* | 336 | JF296232 | BOLD:AAD7604 | |
| Coccinellinae | *Coleomegilla* | *Coleomegilla maculata* | 337 | JF296231 | BOLD:AAD7604 | |
| Coccinellinae | *Coleomegilla* | *Coleomegilla maculata* | 338 | JF296227 | BOLD:AAD7604 | |

**Supplementary Table S3 (continued)**

| Subfamily | Genus | Species name | Code | GenBank accession number | BOLD BIN | Collection country |
|---|---|---|---|---|---|---|
| Coccinellinae | *Coleomegilla* | *Coleomegilla maculata* | 339 | KR488977 | BOLD:AAD7604 | Canada |
| Coccinellinae | *Cycloneda* | *Cycloneda sanguinea* | 340 | | BOLD:AAN6125 | United States |
| Coccinellinae | *Cycloneda* | *Cycloneda sanguinea* | 341 | | BOLD:AAN6125 | United States |
| Coccinellinae | *Cycloneda* | *Cycloneda sanguinea* | 342 | | BOLD:AAN6125 | United States |
| Coccinellinae | *Cycloneda* | *Cycloneda sanguinea* | 343 | | BOLD:AAN6125 | United States |
| Coccinellinae | *Cycloneda* | *Cycloneda sanguinea* | 344 | | BOLD:AAN6125 | United States |
| Coccinellinae | *Halyzia* | *Halyzia sedecimguttata* | 345 | KJ965538 | BOLD:ABW2151 | Finland |
| Coccinellinae | *Halyzia* | *Halyzia sedecimguttata* | 346 | KJ967096 | BOLD:ABW2152 | Finland |
| Coccinellinae | *Halyzia* | *Halyzia sedecimguttata* | 347 | KJ966736 | BOLD:ABW2152 | Finland |
| Coccinellinae | *Halyzia* | *Halyzia sedecimguttata* | 348 | KJ963428 | BOLD:ABW2151 | Finland |
| Coccinellinae | *Halyzia* | *Halyzia sedecimguttata* | 349 | KM449446 | BOLD:ABW2152 | Germany |
| Coccinellinae | *Halyzia* | *Halyzia sedecimguttata* | 350 | KM445222 | BOLD:ABW2151 | Germany |
| Coccinellinae | *Halyzia* | *Halyzia sedecimguttata* | 351 | KU916872 | BOLD:ABW2151 | Germany |
| Coccinellinae | *Halyzia* | *Halyzia sedecimguttata* | 352 | KU907306 | BOLD:ABW2151 | Germany |
| Coccinellinae | *Halyzia* | *Halyzia sedecimguttata* | 353 | KU917225 | BOLD:ABW2151 | Germany |
| Coccinellinae | *Harmonia* | *Harmonia axyridis* | 354 | | BOLD:AAB5640 | United States |
| Coccinellinae | *Harmonia* | *Harmonia axyridis* | 355 | | BOLD:AAB5640 | United States |
| Coccinellinae | *Harmonia* | *Harmonia axyridis* | 356 | | BOLD:AAB5640 | United States |
| Coccinellinae | *Harmonia* | *Harmonia axyridis* | 357 | | BOLD:AAB5640 | United States |
| Coccinellinae | *Harmonia* | *Harmonia axyridis* | 358 | HM411832 | BOLD:AAB5640 | Canada |
| Coccinellinae | *Harmonia* | *Harmonia axyridis* | 359 | HQ953582 | BOLD:AAB5640 | Belgium |
| Coccinellinae | *Harmonia* | *Harmonia axyridis* | 360 | KM448492 | BOLD:AAB5640 | Germany |
| Coccinellinae | *Harmonia* | *Harmonia axyridis* | 361 | KM450721 | BOLD:AAB5640 | Germany |
| Coccinellinae | *Harmonia* | *Harmonia axyridis* | 362 | KC135950 | BOLD:AAB5640 | South Korea |
| Coccinellinae | *Harmonia* | *Harmonia axyridis* | 363 | KU908539 | BOLD:AAB5640 | Germany |
| Coccinellinae | *Harmonia* | *Harmonia axyridis* | 364 | KU908868 | BOLD:AAB5640 | Germany |
| Coccinellinae | *Harmonia* | *Harmonia conformis* | 365 | | BOLD:AAH3302 | Australia |
| Coccinellinae | *Harmonia* | *Harmonia conformis* | 366 | | BOLD:AAH3302 | Australia |
| Coccinellinae | *Harmonia* | *Harmonia conformis* | 367 | | BOLD:AAH3302 | Australia |
| Coccinellinae | *Harmonia* | *Harmonia conformis* | 368 | | BOLD:AAH3302 | Australia |
| Coccinellinae | *Harmonia* | *Harmonia conformis* | 369 | | BOLD:AAH3302 | Australia |

## Supplementary Table S3 (continued)

| Subfamily | Genus | Species name | Code | GenBank accession number | BOLD BIN | Collection country |
|---|---|---|---|---|---|---|
| Coccinellinae | *Harmonia* | *Harmonia conformis* | 370 | | BOLD:AAH3302 | Australia |
| Coccinellinae | *Harmonia* | *Harmonia conformis* | 371 | | BOLD:AAH3302 | Australia |
| Coccinellinae | *Harmonia* | *Harmonia conformis* | 372 | | BOLD:AAH3302 | Australia |
| Coccinellinae | *Harmonia* | *Harmonia conformis* | 373 | | BOLD:AAH3302 | Australia |
| Coccinellinae | *Harmonia* | *Harmonia conformis* | 374 | | BOLD:AAH3302 | Australia |
| Coccinellinae | *Harmonia* | *Harmonia conformis* | 375 | | BOLD:AAH3302 | Australia |
| Coccinellinae | *Harmonia* | *Harmonia quadripunctata* | 376 | KM447142 | BOLD:ACD0984 | Slovenia |
| Coccinellinae | *Harmonia* | *Harmonia quadripunctata* | 377 | KM446380 | BOLD:ACD0984 | Germany |
| Coccinellinae | *Harmonia* | *Harmonia quadripunctata* | 378 | KU914822 | BOLD:ACD0984 | Germany |
| Coccinellinae | *Harmonia* | *Harmonia quadripunctata* | 379 | KU909968 | BOLD:ACD0984 | Germany |
| Coccinellinae | *Harmonia* | *Harmonia quadripunctata* | 380 | KU917400 | BOLD:ACD0984 | Germany |
| Coccinellinae | *Harmonia* | *Harmonia quadripunctata* | 381 | KU915124 | BOLD:ACD0984 | Germany |
| Coccinellinae | *Harmonia* | *Harmonia quadripunctata* | 382 | KU906663 | BOLD:ACD0984 | Germany |
| Coccinellinae | *Hippodamia* | *Hippodamia alpina* | 383 | KM448865 | BOLD:ABA4696 | Italy |
| Coccinellinae | *Hippodamia* | *Hippodamia alpina* | 384 | KM447516 | BOLD:ABA4696 | Italy |
| Coccinellinae | *Hippodamia* | *Hippodamia alpina* | 385 | KM444555 | BOLD:ACC4209 | Italy |
| Coccinellinae | *Hippodamia* | *Hippodamia alpina* | 386 | KM448660 | BOLD:ACC4209 | Italy |
| Coccinellinae | *Hippodamia* | *Hippodamia alpina* | 387 | KM443353 | BOLD:ACC4209 | Italy |
| Coccinellinae | *Hippodamia* | *Hippodamia alpina* | 388 | KM442826 | BOLD:ABA4696 | Italy |
| Coccinellinae | *Hippodamia* | *Hippodamia arctica* | 389 | KU875200 | BOLD:ACD0847 | United States |
| Coccinellinae | *Hippodamia* | *Hippodamia arctica* | 390 | KU875199 | BOLD:ACD0847 | United States |
| Coccinellinae | *Hippodamia* | *Hippodamia caseyi* | 391 | | BOLD:AAH3293 | Canada |
| Coccinellinae | *Hippodamia* | *Hippodamia caseyi* | 392 | | BOLD:AAH3293 | Canada |
| Coccinellinae | *Hippodamia* | *Hippodamia convergens* | 393 | | BOLD:AAH3293 | United States |
| Coccinellinae | *Hippodamia* | *Hippodamia convergens* | 394 | | BOLD:AAH3293 | United States |
| Coccinellinae | *Hippodamia* | *Hippodamia convergens* | 395 | | BOLD:AAH3293 | United States |
| Coccinellinae | *Hippodamia* | *Hippodamia convergens* | 396 | | BOLD:AAH3293 | United States |
| Coccinellinae | *Hippodamia* | *Hippodamia convergens* | 397 | | BOLD:AAH3293 | United States |
| Coccinellinae | *Hippodamia* | *Hippodamia convergens* | 398 | | BOLD:AAH3293 | United States |
| Coccinellinae | *Hippodamia* | *Hippodamia convergens* | 399 | JF888328 | BOLD:AAH3293 | Canada |
| Coccinellinae | *Hippodamia* | *Hippodamia convergens* | 400 | | BOLD:AAH3293 | United States |

## Supplementary Table S3 (continued)

| Subfamily | Genus | Species name | Code | GenBank accession number | BOLD BIN | Collection country |
|---|---|---|---|---|---|---|
| Coccinellinae | *Hippodamia* | *Hippodamia convergens* | 401 | | BOLD:AAH3293 | United States |
| Coccinellinae | *Hippodamia* | *Hippodamia convergens* | 402 | | BOLD:AAH3293 | United States |
| Coccinellinae | *Hippodamia* | *Hippodamia convergens* | 403 | | BOLD:AAH3293 | United States |
| Coccinellinae | *Hippodamia* | *Hippodamia convergens* | 404 | KJ204174 | BOLD:AAH3293 | Canada |
| Coccinellinae | *Hippodamia* | *Hippodamia convergens* | 405 | JF888327 | BOLD:AAH3293 | Canada |
| Coccinellinae | *Hippodamia* | *Hippodamia convergens* | 406 | JF888331 | BOLD:AAH3293 | Canada |
| Coccinellinae | *Hippodamia* | *Hippodamia glacialis* | 407 | | BOLD:AAH3305 | Canada |
| Coccinellinae | *Hippodamia* | *Hippodamia glacialis* | 408 | | BOLD:AAH3305 | Canada |
| Coccinellinae | *Hippodamia* | *Hippodamia glacialis* | 409 | KM846002 | BOLD:AAH3305 | Canada |
| Coccinellinae | *Hippodamia* | *Hippodamia moesta* | 410 | | BOLD:AAH3293 | Canada |
| Coccinellinae | *Hippodamia* | *Hippodamia moesta* | 411 | | BOLD:AAH3293 | Canada |
| Coccinellinae | *Hippodamia* | *Hippodamia notata* | 412 | KJ963275 | BOLD:AAX1230 | Finland |
| Coccinellinae | *Hippodamia* | *Hippodamia notata* | 413 | KJ967163 | BOLD:ABW2193 | Estonia |
| Coccinellinae | *Hippodamia* | *Hippodamia notata* | 414 | KJ967325 | BOLD:AAX1230 | Finland |
| Coccinellinae | *Hippodamia* | *Hippodamia notata* | 415 | HQ948244 | BOLD:AAX1230 | Germany |
| Coccinellinae | *Hippodamia* | *Hippodamia notata* | 416 | KM450459 | BOLD:AAX1230 | Austria |
| Coccinellinae | *Hippodamia* | *Hippodamia notata* | 417 | KM443996 | BOLD:AAX1230 | Czech Republic |
| Coccinellinae | *Hippodamia* | *Hippodamia notata* | 418 | KM445732 | BOLD:ABW2193 | Germany |
| Coccinellinae | *Hippodamia* | *Hippodamia notata* | 419 | KM443863 | BOLD:ABW2193 | France |
| Coccinellinae | *Hippodamia* | *Hippodamia notata* | 420 | KM446925 | BOLD:AAX1230 | France |
| Coccinellinae | *Hippodamia* | *Hippodamia notata* | 421 | KM451621 | BOLD:ABW2193 | France |
| Coccinellinae | *Hippodamia* | *Hippodamia notata* | 422 | KM446767 | BOLD:AAX1230 | Italy |
| Coccinellinae | *Hippodamia* | *Hippodamia notata* | 423 | KU906476 | BOLD:AAX1230 | Austria |
| Coccinellinae | *Hippodamia* | *Hippodamia notata* | 424 | KU908037 | BOLD:AAX1230 | Austria |
| Coccinellinae | *Hippodamia* | *Hippodamia notata* | 425 | KU919551 | BOLD:AAX1230 | Austria |
| Coccinellinae | *Hippodamia* | *Hippodamia notata* | 426 | KU906550 | BOLD:AAX1230 | Austria |
| Coccinellinae | *Hippodamia* | *Hippodamia notata* | 427 | KU917762 | BOLD:AAX1230 | Italy |
| Coccinellinae | *Hippodamia* | *Hippodamia notata* | 428 | KU912748 | BOLD:AAX1230 | Germany |
| Coccinellinae | *Hippodamia* | *Hippodamia notata* | 429 | KU911912 | BOLD:ABW2193 | Germany |
| Coccinellinae | *Hippodamia* | *Hippodamia notata* | 430 | KJ964706 | BOLD:AAX1230 | Finland |
| Coccinellinae | *Hippodamia* | *Hippodamia notata* | 431 | KM442003 | BOLD:ABW2193 | France |

## Supplementary Table S3 (continued)

| Subfamily | Genus | Species name | Code | GenBank accession number | BOLD BIN | Collection country |
|---|---|---|---|---|---|---|
| Coccinellinae | *Hippodamia* | *Hippodamia parenthesis* | 432 | JF296241 | BOLD:AAB8013 | |
| Coccinellinae | *Hippodamia* | *Hippodamia parenthesis* | 433 | JF296239 | BOLD:AAB8013 | |
| Coccinellinae | *Hippodamia* | *Hippodamia parenthesis* | 434 | KR483115 | BOLD:AAB8013 | Canada |
| Coccinellinae | *Hippodamia* | *Hippodamia parenthesis* | 435 | KR491238 | BOLD:AAB8013 | Canada |
| Coccinellinae | *Hippodamia* | *Hippodamia parenthesis* | 436 | KM850346 | BOLD:AAB8013 | Canada |
| Coccinellinae | *Hippodamia* | *Hippodamia parenthesis* | 437 | KM849091 | BOLD:AAB8013 | Canada |
| Coccinellinae | *Hippodamia* | *Hippodamia parenthesis* | 438 | KM845578 | BOLD:AAB8013 | Canada |
| Coccinellinae | *Hippodamia* | *Hippodamia parenthesis* | 439 | KM844470 | BOLD:AAB8013 | Canada |
| Coccinellinae | *Hippodamia* | *Hippodamia parenthesis* | 440 | KM847284 | BOLD:AAB8013 | Canada |
| Coccinellinae | *Hippodamia* | *Hippodamia parenthesis* | 441 | KM847712 | BOLD:AAB8013 | Canada |
| Coccinellinae | *Hippodamia* | *Hippodamia parenthesis* | 442 | KM847898 | BOLD:AAB8013 | Canada |
| Coccinellinae | *Hippodamia* | *Hippodamia quinquesignata* | 443 | KU875207 | BOLD:AAH3293 | United States |
| Coccinellinae | *Hippodamia* | *Hippodamia quinquesignata* | 444 | KU875209 | BOLD:AAH3293 | United States |
| Coccinellinae | *Hippodamia* | *Hippodamia quinquesignata* | 445 | KU875208 | BOLD:AAH3293 | United States |
| Coccinellinae | *Hippodamia* | *Hippodamia tredecimpunctata* | 446 | KJ965246 | BOLD:ABW5064 | Finland |
| Coccinellinae | *Hippodamia* | *Hippodamia tredecimpunctata* | 447 | KJ966401 | BOLD:ABW5064 | Finland |
| Coccinellinae | *Hippodamia* | *Hippodamia tredecimpunctata* | 448 | KJ964732 | BOLD:ABW5064 | Finland |
| Coccinellinae | *Hippodamia* | *Hippodamia tredecimpunctata* | 449 | KJ965869 | BOLD:ABW5064 | Finland |
| Coccinellinae | *Hippodamia* | *Hippodamia tredecimpunctata* | 450 | KU914839 | BOLD:ABW5064 | Germany |
| Coccinellinae | *Hippodamia* | *Hippodamia tredecimpunctata* | 451 | KU914324 | BOLD:ABW5064 | Germany |
| Coccinellinae | *Hippodamia* | *Hippodamia tredecimpunctata* | 452 | KU916942 | BOLD:ABW5064 | Germany |
| Coccinellinae | *Hippodamia* | *Hippodamia tredecimpunctata* | 453 | KU915679 | BOLD:ABW5064 | Germany |
| Coccinellinae | *Hippodamia* | *Hippodamia tredecimpunctata* | 454 | KU916754 | BOLD:ABW5064 | Germany |
| Coccinellinae | *Hippodamia* | *Hippodamia tredecimpunctata* | 455 | KU918987 | BOLD:ABW5064 | Germany |
| Coccinellinae | *Hippodamia* | *Hippodamia tredecimpunctata* | 456 | KU914579 | BOLD:ABW5064 | Germany |
| Coccinellinae | *Hippodamia* | *Hippodamia tredecimpunctata* | 457 | KM850065 | BOLD:AAX1229 | Canada |
| Coccinellinae | *Hippodamia* | *Hippodamia tredecimpunctata* | 458 | KM846466 | BOLD:AAX1229 | Canada |
| Coccinellinae | *Hippodamia* | *Hippodamia tredecimpunctata* | 459 | KU875210 | BOLD:AAX1229 | United States |
| Coccinellinae | *Hippodamia* | *Hippodamia undecimnotata* | 460 | HQ953691 | BOLD:AAO0563 | Germany |
| Coccinellinae | *Hippodamia* | *Hippodamia undecimnotata* | 461 | KM449355 | BOLD:AAO0563 | Germany |
| Coccinellinae | *Hippodamia* | *Hippodamia undecimnotata* | 462 | KM439233 | BOLD:AAO0563 | Germany |

## Supplementary Table S3 (continued)

| Subfamily | Genus | Species name | Code | GenBank accession number | BOLD BIN | Collection country |
|---|---|---|---|---|---|---|
| Coccinellinae | *Hippodamia* | *Hippodamia undecimnotata* | 463 | KM439752 | BOLD:AAO0563 | Germany |
| Coccinellinae | *Hippodamia* | *Hippodamia undecimnotata* | 464 | KM445695 | BOLD:AAO0563 | Germany |
| Coccinellinae | *Hippodamia* | *Hippodamia undecimnotata* | 465 | KU906140 | BOLD:AAO0563 | Germany |
| Coccinellinae | *Hippodamia* | *Hippodamia undecimnotata* | 466 | KU917933 | BOLD:AAO0563 | Germany |
| Coccinellinae | *Hippodamia* | *Hippodamia variegata* | 467 | KJ964021 | BOLD:AAH3299 | Finland |
| Coccinellinae | *Hippodamia* | *Hippodamia variegata* | 468 | KM449469 | BOLD:AAH3299 | Slovenia |
| Coccinellinae | *Hippodamia* | *Hippodamia variegata* | 469 | KM445423 | BOLD:AAH3299 | Slovenia |
| Coccinellinae | *Hippodamia* | *Hippodamia variegata* | 470 | KM439665 | BOLD:AAH3299 | Slovenia |
| Coccinellinae | *Hippodamia* | *Hippodamia variegata* | 471 | KU918664 | BOLD:AAH3299 | Germany |
| Coccinellinae | *Hippodamia* | *Hippodamia variegata* | 472 | KY839421 | BOLD:AAH3299 | Pakistan |
| Coccinellinae | *Hippodamia* | *Hippodamia variegata* | 473 | KJ965564 | BOLD:AAH3299 | Finland |
| Coccinellinae | *Illeis* | *Illeis galbula* | 474 |  | BOLD:AAF2558 | Australia |
| Coccinellinae | *Illeis* | *Illeis galbula* | 475 |  | BOLD:AAF2558 | Australia |
| Coccinellinae | *Illeis* | *Illeis galbula* | 476 |  | BOLD:AAF2558 | Australia |
| Coccinellinae | *Macronaemia* | *Macronaemia episcopalis* | 477 | JF888135 | BOLD:AAP7833 | Canada |
| Coccinellinae | *Macronaemia* | *Macronaemia episcopalis* | 478 | KU875442 | BOLD:AAP7833 | United States |
| Coccinellinae | *Mulsantina* | *Mulsantina hudsonica* | 479 | HQ989923 | BOLD:AAI3237 | Canada |
| Coccinellinae | *Mulsantina* | *Mulsantina hudsonica* | 480 | KM842879 | BOLD:AAI3237 | Canada |
| Coccinellinae | *Mulsantina* | *Mulsantina hudsonica* | 481 | KM842783 | BOLD:AAI3237 | Canada |
| Coccinellinae | *Mulsantina* | *Mulsantina hudsonica* | 482 | KM846626 | BOLD:AAI3237 | Canada |
| Coccinellinae | *Mulsantina* | *Mulsantina hudsonica* | 483 | KM843477 | BOLD:AAI3237 | Canada |
| Coccinellinae | *Mulsantina* | *Mulsantina picta* | 484 | KM844401 | BOLD:AAM7729 | Canada |
| Coccinellinae | *Myrrha* | *Myrrha octodecimguttata* | 485 | KJ965232 | BOLD:AAX2942 | Finland |
| Coccinellinae | *Myrrha* | *Myrrha octodecimguttata* | 486 | KJ964427 | BOLD:AAX2942 | Finland |
| Coccinellinae | *Myrrha* | *Myrrha octodecimguttata* | 487 | KM450857 | BOLD:AAX2942 |  |
| Coccinellinae | *Myrrha* | *Myrrha octodecimguttata* | 488 | KM443105 | BOLD:ACF5104 | Germany |
| Coccinellinae | *Myrrha* | *Myrrha octodecimguttata* | 489 | KM448744 | BOLD:ACF5104 | Germany |
| Coccinellinae | *Myrrha* | *Myrrha octodecimguttata* | 490 | KM439936 | BOLD:AAX2942 | Germany |
| Coccinellinae | *Myrrha* | *Myrrha octodecimguttata* | 491 | KM448818 | BOLD:ACF5104 | Germany |
| Coccinellinae | *Myrrha* | *Myrrha octodecimguttata* | 492 | KM451571 | BOLD:ACF5104 | Germany |
| Coccinellinae | *Myrrha* | *Myrrha octodecimguttata* | 493 | KM448808 | BOLD:AAX2942 | Germany |

## Supplementary Table S3 (continued)

| Subfamily | Genus | Species name | Code | GenBank accession number | BOLD BIN | Collection country |
|---|---|---|---|---|---|---|
| Coccinellinae | *Myrrha* | *Myrrha octodecimguttata* | 494 | KU907354 | BOLD:AAX2942 | Germany |
| Coccinellinae | *Myrrha* | *Myrrha octodecimguttata* | 495 | KU912849 | BOLD:AAX2942 | Germany |
| Coccinellinae | *Myrrha* | *Myrrha octodecimguttata* | 496 | KU917551 | BOLD:AAX2942 | Germany |
| Coccinellinae | *Myrrha* | *Myrrha octodecimguttata* | 497 | KU916543 | BOLD:AAX2942 | Germany |
| Coccinellinae | *Myrrha* | *Myrrha octodecimguttata* | 498 | KU919438 | BOLD:AAX2942 | Germany |
| Coccinellinae | *Myrrha* | *Myrrha octodecimguttata* | 499 | KU917191 | BOLD:AAX2942 | Germany |
| Coccinellinae | *Myrrha* | *Myrrha octodecimguttata* | 500 | KU913763 | BOLD:ACF5104 | Germany |
| Coccinellinae | *Myrrha* | *Myrrha octodecimguttata* | 501 | KU915301 | BOLD:AAX2942 | Germany |
| Coccinellinae | *Myrrha* | *Myrrha octodecimguttata* | 502 | KU906233 | BOLD:AAX2942 | Germany |
| Coccinellinae | *Myzia* | *Myzia oblongoguttata* | 503 | KJ966701 | BOLD:AAP7387 | Finland |
| Coccinellinae | *Myzia* | *Myzia oblongoguttata* | 504 | KJ963781 | BOLD:AAP7387 | Finland |
| Coccinellinae | *Myzia* | *Myzia oblongoguttata* | 505 | JF889602 | BOLD:AAP7387 | Germany |
| Coccinellinae | *Myzia* | *Myzia oblongoguttata* | 506 | KM450947 | BOLD:AAP7387 | Germany |
| Coccinellinae | *Myzia* | *Myzia oblongoguttata* | 507 | KM451601 | BOLD:AAP7387 | Germany |
| Coccinellinae | *Myzia* | *Myzia oblongoguttata* | 508 | KM449651 | BOLD:AAP7387 | Slovenia |
| Coccinellinae | *Myzia* | *Myzia oblongoguttata* | 509 | KU909388 | BOLD:AAP7387 | Germany |
| Coccinellinae | *Myzia* | *Myzia oblongoguttata* | 510 | KU907054 | BOLD:AAP7387 | Germany |
| Coccinellinae | *Myzia* | *Myzia oblongoguttata* | 511 | KU915391 | BOLD:AAP7387 | Germany |
| Coccinellinae | *Myzia* | *Myzia oblongoguttata* | 512 | KU913844 | BOLD:AAP7387 | Germany |
| Coccinellinae | *Myzia* | *Myzia oblongoguttata* | 513 | KU908480 | BOLD:AAP7387 | Germany |
| Coccinellinae | *Myzia* | *Myzia oblongoguttata* | 514 | KU907848 | BOLD:AAP7387 | Germany |
| Coccinellinae | *Myzia* | *Myzia oblongoguttata* | 515 | KU912826 | BOLD:AAP7387 | Germany |
| Coccinellinae | *Myzia* | *Myzia oblongoguttata* | 516 | KU907893 | BOLD:AAP7387 | Germany |
| Coccinellinae | *Myzia* | *Myzia pullata* | 517 | | BOLD:AAH3314 | United States |
| Coccinellinae | *Myzia* | *Myzia pullata* | 518 | | BOLD:AAH3314 | United States |
| Coccinellinae | *Myzia* | *Myzia pullata* | 519 | | BOLD:ACA6005 | United States |
| Coccinellinae | *Neoharmonia* | *Neoharmonia venusta* | 520 | | BOLD:ABX0180 | United States |
| Coccinellinae | *Neoharmonia* | *Neoharmonia venusta* | 521 | | BOLD:ABX0180 | United States |
| Coccinellinae | *Oenopia* | *Oenopia conglobata* | 522 | KM446481 | BOLD:ACC8437 | Germany |
| Coccinellinae | *Oenopia* | *Oenopia conglobata* | 523 | KM440180 | BOLD:ACC8437 | Germany |
| Coccinellinae | *Oenopia* | *Oenopia conglobata* | 524 | KM444810 | BOLD:ACC8437 | Germany |

## Supplementary Table S3 (continued)

| Subfamily | Genus | Species name | Code | GenBank accession number | BOLD BIN | Collection country |
|---|---|---|---|---|---|---|
| Coccinellinae | *Olla* | *Olla vnigrum* | 525 | | BOLD:AAH3312 | United States |
| Coccinellinae | *Olla* | *Olla vnigrum* | 526 | | BOLD:AAH3312 | United States |
| Coccinellinae | *Olla* | *Olla vnigrum* | 527 | | BOLD:AAH3312 | United States |
| Coccinellinae | *Olla* | *Olla vnigrum* | 528 | | BOLD:AAH3312 | United States |
| Coccinellinae | *Olla* | *Olla vnigrum* | 529 | KX054244 | BOLD:AAH3312 | French Polynesia |
| Coccinellinae | *Olla* | *Olla vnigrum* | 530 | KX054243 | BOLD:AAH3312 | French Polynesia |
| Coccinellinae | *Olla* | *Olla vnigrum* | 531 | KX054245 | BOLD:AAH3312 | French Polynesia |
| Coccinellinae | *Propylaea* | *Propylaea quatuordecimpunctata* | 532 | KJ964908 | BOLD:AAF6935 | Finland |
| Coccinellinae | *Propylaea* | *Propylaea quatuordecimpunctata* | 533 | KJ963270 | BOLD:AAF6935 | Finland |
| Coccinellinae | *Propylaea* | *Propylaea quatuordecimpunctata* | 534 | KJ962089 | BOLD:AAF6935 | Estonia |
| Coccinellinae | *Propylaea* | *Propylaea quatuordecimpunctata* | 535 | KJ966239 | BOLD:AAF6935 | Finland |
| Coccinellinae | *Propylaea* | *Propylaea quatuordecimpunctata* | 536 | KJ962666 | BOLD:AAF6935 | Finland |
| Coccinellinae | *Propylaea* | *Propylaea quatuordecimpunctata* | 537 | KR483681 | BOLD:AAF6935 | Canada |
| Coccinellinae | *Propylaea* | *Propylaea quatuordecimpunctata* | 538 | KM445931 | BOLD:AAF6935 | Germany |
| Coccinellinae | *Propylaea* | *Propylaea quatuordecimpunctata* | 539 | KM445167 | BOLD:AAF6935 | Germany |
| Coccinellinae | *Propylaea* | *Propylaea quatuordecimpunctata* | 540 | KM445576 | BOLD:AAF6935 | Germany |
| Coccinellinae | *Propylaea* | *Propylaea quatuordecimpunctata* | 541 | KM440758 | BOLD:AAF6935 | Germany |
| Coccinellinae | *Propylaea* | *Propylaea quatuordecimpunctata* | 542 | KM442421 | BOLD:AAF6935 | Germany |
| Coccinellinae | *Propylaea* | *Propylaea quatuordecimpunctata* | 543 | KM439327 | BOLD:AAF6935 | Germany |
| Coccinellinae | *Propylaea* | *Propylaea quatuordecimpunctata* | 544 | KM441123 | BOLD:AAF6935 | Germany |
| Coccinellinae | *Propylaea* | *Propylaea quatuordecimpunctata* | 545 | KU916806 | BOLD:AAF6935 | Germany |
| Coccinellinae | *Propylaea* | *Propylaea quatuordecimpunctata* | 546 | KU907632 | BOLD:AAF6935 | Germany |
| Coccinellinae | *Propylaea* | *Propylaea quatuordecimpunctata* | 547 | KU914040 | BOLD:AAF6935 | Germany |
| Coccinellinae | *Propylaea* | *Propylaea quatuordecimpunctata* | 548 | KU906647 | BOLD:AAF6935 | Germany |
| Coccinellinae | *Propylaea* | *Propylaea quatuordecimpunctata* | 549 | KU916339 | BOLD:AAF6935 | Germany |
| Coccinellinae | *Psyllobora* | *Psyllobora renifer* | 550 | | BOLD:ABX4030 | United States |
| Coccinellinae | *Psyllobora* | *Psyllobora renifer* | 551 | KM846812 | BOLD:AAU2688 | Canada |
| Coccinellinae | *Psyllobora* | *Psyllobora renifer* | 552 | KM846457 | BOLD:AAU2688 | Canada |
| Coccinellinae | *Psyllobora* | *Psyllobora renifer* | 553 | KM843128 | BOLD:AAU2688 | Canada |
| Coccinellinae | *Psyllobora* | *Psyllobora renifer* | 554 | KM849587 | BOLD:AAU2688 | Canada |
| Coccinellinae | *Psyllobora* | *Psyllobora renifer* | 555 | | BOLD:AAU2688 | United States |

## Supplementary Table S3 (continued)

| Subfamily | Genus | Species name | Code | GenBank accession number | BOLD BIN | Collection country |
|---|---|---|---|---|---|---|
| Coccinellinae | *Psyllobora* | *Psyllobora vigintiduopunctata* | 556 | KJ965785 | BOLD:AAO1034 | Sweden |
| Coccinellinae | *Psyllobora* | *Psyllobora vigintiduopunctata* | 557 | HQ953406 | BOLD:AAO1034 | Germany |
| Coccinellinae | *Psyllobora* | *Psyllobora vigintiduopunctata* | 558 | KM441270 | BOLD:AAO1034 | Germany |
| Coccinellinae | *Psyllobora* | *Psyllobora vigintiduopunctata* | 559 | KM449439 | BOLD:AAO1034 | Germany |
| Coccinellinae | *Psyllobora* | *Psyllobora vigintiduopunctata* | 560 | KU914298 | BOLD:AAO1034 | Germany |
| Coccinellinae | *Psyllobora* | *Psyllobora vigintiduopunctata* | 561 | KU914082 | BOLD:AAO1034 | Germany |
| Coccinellinae | *Psyllobora* | *Psyllobora vigintiduopunctata* | 562 | KJ967506 | BOLD:AAO1034 | Finland |
| Coccinellinae | *Psyllobora* | *Psyllobora vigintiduopunctata* | 563 | KJ965216 | BOLD:AAO1034 | Finland |
| Coccinellinae | *Psyllobora* | *Psyllobora vigintiduopunctata* | 564 | KJ963164 | BOLD:AAO1034 | Finland |
| Coccinellinae | *Psyllobora* | *Psyllobora vigintiduopunctata* | 565 | HQ954459 | BOLD:AAO1034 | Germany |
| Coccinellinae | *Psyllobora* | *Psyllobora vigintiduopunctata* | 566 | KM442148 | BOLD:AAO1034 | Germany |
| Coccinellinae | *Psyllobora* | *Psyllobora vigintiduopunctata* | 567 | KM444472 | BOLD:AAO1034 | Germany |
| Coccinellinae | *Psyllobora* | *Psyllobora vigintiduopunctata* | 568 | KM442027 | BOLD:AAO1034 | Germany |
| Coccinellinae | *Psyllobora* | *Psyllobora vigintiduopunctata* | 569 | KM439403 | BOLD:AAO1034 | Germany |
| Coccinellinae | *Psyllobora* | *Psyllobora vigintiduopunctata* | 570 | KM441422 | BOLD:AAO1034 | Germany |
| Coccinellinae | *Psyllobora* | *Psyllobora vigintiduopunctata* | 571 | KM448526 | BOLD:ACI8392 | France |
| Coccinellinae | *Psyllobora* | *Psyllobora vigintiduopunctata* | 572 | KU916076 | BOLD:AAO1034 | Germany |
| Coccinellinae | *Psyllobora* | *Psyllobora vigintimaculata* | 573 | JF887760 | BOLD:AAH3320 | Canada |
| Coccinellinae | *Psyllobora* | *Psyllobora vigintimaculata* | 574 | HQ551576 | BOLD:AAM7657 | Canada |
| Coccinellinae | *Psyllobora* | *Psyllobora vigintimaculata* | 575 | KR130523 | BOLD:ACC9145 | Canada |
| Coccinellinae | *Psyllobora* | *Psyllobora vigintimaculata* | 576 | KR119905 | BOLD:ACC9145 | Canada |
| Coccinellinae | *Tytthaspis* | *Tytthaspis sedecimpunctata* | 577 | KJ966079 | BOLD:AAN9355 | Sweden |
| Coccinellinae | *Tytthaspis* | *Tytthaspis sedecimpunctata* | 578 | KJ964456 | BOLD:AAN9355 | Sweden |
| Coccinellinae | *Tytthaspis* | *Tytthaspis sedecimpunctata* | 579 | HQ953877 | BOLD:AAN9355 | Germany |
| Coccinellinae | *Tytthaspis* | *Tytthaspis sedecimpunctata* | 580 | HQ953878 | BOLD:AAN9355 | Germany |
| Coccinellinae | *Tytthaspis* | *Tytthaspis sedecimpunctata* | 581 | KM446576 | BOLD:AAN9355 | Germany |
| Coccinellinae | *Tytthaspis* | *Tytthaspis sedecimpunctata* | 582 | KM443042 | BOLD:AAN9355 | Germany |
| Coccinellinae | *Tytthaspis* | *Tytthaspis sedecimpunctata* | 583 | KM444030 | BOLD:AAN9355 | Germany |
| Coccinellinae | *Tytthaspis* | *Tytthaspis sedecimpunctata* | 584 | KM439347 | BOLD:AAN9355 | Germany |
| Coccinellinae | *Tytthaspis* | *Tytthaspis sedecimpunctata* | 585 | KM448756 | BOLD:AAN9355 | Germany |
| Coccinellinae | *Tytthaspis* | *Tytthaspis sedecimpunctata* | 586 | KM451457 | BOLD:AAN9355 | Germany |

## Supplementary Table S3 (continued)

| Subfamily | Genus | Species name | Code | GenBank accession number | BOLD BIN | Collection country |
|---|---|---|---|---|---|---|
| Coccinellinae | *Tytthaspis* | *Tytthaspis sedecimpunctata* | 587 | KU907601 | BOLD:AAN9355 | Germany |
| Coccinellinae | *Tytthaspis* | *Tytthaspis sedecimpunctata* | 588 | KU908174 | BOLD:AAN9355 | Germany |
| Coccinellinae | *Tytthaspis* | *Tytthaspis sedecimpunctata* | 589 | KU906879 | BOLD:AAN9355 | Germany |
| Coccinellinae | *Tytthaspis* | *Tytthaspis sedecimpunctata* | 590 | KU909976 | BOLD:AAN9355 | Germany |
| Coccinellinae | *Tytthaspis* | *Tytthaspis sedecimpunctata* | 591 | KU908637 | BOLD:AAN9355 | Germany |
| Coccinellinae | *Tytthaspis* | *Tytthaspis sedecimpunctata* | 592 | KU910585 | BOLD:AAN9355 | Germany |
| Coccinellinae | *Tytthaspis* | *Tytthaspis sedecimpunctata* | 593 | KU911025 | BOLD:AAN9355 | Germany |
| Coccinellinae | *Tytthaspis* | *Tytthaspis sedecimpunctata* | 594 | KU919593 | BOLD:AAN9355 | Germany |
| Coccinellinae | *Tytthaspis* | *Tytthaspis sedecimpunctata* | 595 | KU913719 | BOLD:AAN9355 | Germany |
| Coccinellinae | *Vibidia* | *Vibidia duodecimguttata* | 596 | KM450433 | BOLD:ACD6271 | Germany |
| Coccinellinae | *Vibidia* | *Vibidia duodecimguttata* | 597 | KM446364 | BOLD:ACD6271 | Germany |
| Coccinellinae | *Vibidia* | *Vibidia duodecimguttata* | 598 | KM447545 | BOLD:ACD6271 | Germany |
| Coccinellinae | *Vibidia* | *Vibidia duodecimguttata* | 599 | KU912901 | BOLD:ACD6271 | Germany |
| Coccinellinae | *Vibidia* | *Vibidia duodecimguttata* | 600 | KU917348 | BOLD:ACD6271 | Germany |
| Coccinellinae | *Vibidia* | *Vibidia duodecimguttata* | 601 | KU913175 | BOLD:ACD6271 | Germany |
| Coccinellinae | *Vibidia* | *Vibidia duodecimguttata* | 602 | KU912225 | BOLD:ACD6271 | Germany |
| Coccinellinae | *Vibidia* | *Vibidia duodecimguttata* | 603 | KU909233 | BOLD:ACD6271 | Germany |
| Epilachninae | *Cynegetis* | *Cynegetis impunctata* | 604 | KJ963646 | BOLD:ACF2139 | Finland |
| Epilachninae | *Cynegetis* | *Cynegetis impunctata* | 605 | KM440148 | BOLD:ACF2139 | Germany |
| Epilachninae | *Cynegetis* | *Cynegetis impunctata* | 606 | KU908367 | BOLD:ACF2139 | Germany |
| Epilachninae | *Cynegetis* | *Cynegetis impunctata* | 607 | KU917582 | BOLD:ACF2139 | Germany |
| Epilachninae | *Cynegetis* | *Cynegetis impunctata* | 608 | KU913127 | BOLD:ACF2139 | Germany |
| Epilachninae | *Cynegetis* | *Cynegetis impunctata* | 609 | KU906930 | BOLD:ACF2139 | Germany |
| Epilachninae | *Cynegetis* | *Cynegetis impunctata* | 610 | KU913194 | BOLD:ACF2139 | Germany |
| Epilachninae | *Cynegetis* | *Cynegetis impunctata* | 611 | KU906808 | BOLD:ACF2139 | Germany |
| Epilachninae | *Cynegetis* | *Cynegetis impunctata* | 612 | KU906884 | BOLD:ACF2139 | Germany |
| Epilachninae | *Cynegetis* | *Cynegetis impunctata* | 613 | KU911545 | BOLD:ACF2139 | Germany |
| Epilachninae | *Cynegetis* | *Cynegetis impunctata* | 614 | KU908528 | BOLD:ACF2139 | Germany |
| Epilachninae | *Henosepilachna* | *Henosepilachna argus* | 615 | HQ953992 | BOLD:AAO4570 | Germany |
| Epilachninae | *Henosepilachna* | *Henosepilachna argus* | 616 | KM448639 | BOLD:AAO4570 | Germany |
| Epilachninae | *Henosepilachna* | *Henosepilachna argus* | 617 | KU912822 | BOLD:AAO4570 | Germany |

**Supplementary Table S3 (continued)**

| Subfamily | Genus | Species name | Code | GenBank accession number | BOLD BIN | Collection country |
|---|---|---|---|---|---|---|
| Epilachninae | *Henosepilachna* | *Henosepilachna argus* | 618 | KU910456 | BOLD:AAO4570 | Germany |
| Epilachninae | *Subcoccinella* | *Subcoccinella vigintiquatuorpunctata* | 619 | KM447888 | BOLD:ABA4526 | Germany |
| Epilachninae | *Subcoccinella* | *Subcoccinella vigintiquatuorpunctata* | 620 | KM440383 | BOLD:ABA4526 | Germany |
| Epilachninae | *Subcoccinella* | *Subcoccinella vigintiquatuorpunctata* | 621 | KM450224 | BOLD:ABA4526 | Germany |
| Epilachninae | *Subcoccinella* | *Subcoccinella vigintiquatuorpunctata* | 622 | KM448719 | BOLD:ABA4526 | Germany |
| Epilachninae | *Subcoccinella* | *Subcoccinella vigintiquatuorpunctata* | 623 | KM442853 | BOLD:ABA4526 | Germany |
| Epilachninae | *Subcoccinella* | *Subcoccinella vigintiquatuorpunctata* | 624 | KU917924 | BOLD:ABA4526 | Germany |
| Epilachninae | *Subcoccinella* | *Subcoccinella vigintiquatuorpunctata* | 625 | KU912178 | BOLD:ABA4526 | Germany |
| Epilachninae | *Subcoccinella* | *Subcoccinella vigintiquatuorpunctata* | 626 | KU907367 | BOLD:ABA4526 | Germany |
| Epilachninae | *Subcoccinella* | *Subcoccinella vigintiquatuorpunctata* | 627 | KU913524 | BOLD:ABA4526 | Germany |
| Epilachninae | *Subcoccinella* | *Subcoccinella vigintiquatuorpunctata* | 628 | KU913867 | BOLD:ABA4526 | Italy |
| Epilachninae | *Subcoccinella* | *Subcoccinella vigintiquatuorpunctata* | 629 | KU918684 | BOLD:ABA4526 | Italy |
| Epilachninae | *Subcoccinella* | *Subcoccinella vigintiquatuorpunctata* | 630 | KU917285 | BOLD:ABA4526 | Germany |
| Epilachninae | *Subcoccinella* | *Subcoccinella vigintiquatuorpunctata* | 631 | KU907135 | BOLD:ABA4526 | Germany |
| Scymninae | *Brachiacantha* | *Brachiacantha rotunda* | 632 | HM411988 | BOLD:AAH3319 | Canada |
| Scymninae | *Brachiacantha* | *Brachiacantha rotunda* | 633 | HM411989 | BOLD:AAH3319 | Canada |
| Scymninae | *Brachiacantha* | *Brachiacantha rotunda* | 634 | HQ551591 | BOLD:ACK1868 | Canada |
| Scymninae | *Brachiacantha* | *Brachiacantha rotunda* | 635 | HQ551640 | BOLD:AAH3319 | Canada |
| Scymninae | *Brachiacantha* | *Brachiacantha rotunda* | 636 | KR486866 | BOLD:ACK1868 | Canada |
| Scymninae | *Brachiacantha* | *Brachiacantha ursina* | 637 | | BOLD:ABA6356 | Canada |
| Scymninae | *Brachiacantha* | *Brachiacantha ursina* | 638 | KR479918 | BOLD:ABA6356 | Canada |
| Scymninae | *Clitostethus* | *Clitostethus arcuatus* | 639 | HQ953841 | BOLD:AAN9969 | Germany |
| Scymninae | *Clitostethus* | *Clitostethus arcuatus* | 640 | KM447251 | BOLD:AAN9969 | Germany |
| Scymninae | *Clitostethus* | *Clitostethus arcuatus* | 641 | KU911667 | BOLD:AAN9969 | Germany |
| Scymninae | *Clitostethus* | *Clitostethus arcuatus* | 642 | KU906388 | BOLD:AAN9969 | Germany |
| Scymninae | *Clitostethus* | *Clitostethus arcuatus* | 643 | KU917488 | BOLD:AAN9969 | Germany |
| Scymninae | *Didion* | *Didion punctatum* | 644 | KM843344 | BOLD:AAG4502 | Canada |
| Scymninae | *Didion* | *Didion punctatum* | 645 | HM432949 | BOLD:AAG4502 | Canada |
| Scymninae | *Didion* | *Didion punctatum* | 646 | JN279155 | BOLD:AAG4502 | Canada |
| Scymninae | *Diomus* | *Diomus terminatus* | 647 | | BOLD:AAN5910 | United States |
| Scymninae | *Diomus* | *Diomus terminatus* | 648 | KR483709 | BOLD:AAN5910 | Canada |

## Supplementary Table S3 (continued)

| Subfamily | Genus | Species name | Code | GenBank accession number | BOLD BIN | Collection country |
|---|---|---|---|---|---|---|
| Scymninae | *Hyperaspis* | *Hyperaspis binotata* | 649 | KR486395 | BOLD:AAN6147 | Canada |
| Scymninae | *Hyperaspis* | *Hyperaspis binotata* | 650 | KT705895 | BOLD:AAN6147 | Canada |
| Scymninae | *Hyperaspis* | *Hyperaspis campestris* | 651 | KM443952 | BOLD:ACC6750 | Belgium |
| Scymninae | *Hyperaspis* | *Hyperaspis campestris* | 652 | KU908096 | BOLD:ACC6750 | Germany |
| Scymninae | *Hyperaspis* | *Hyperaspis campestris* | 653 | KU913071 | BOLD:ACC6750 | Germany |
| Scymninae | *Hyperaspis* | *Hyperaspis lateralis* | 654 | KR486355 | BOLD:ACU7795 | Canada |
| Scymninae | *Hyperaspis* | *Hyperaspis lateralis* | 655 | KR483378 | BOLD:ACW2336 | Canada |
| Scymninae | *Hyperaspis* | *Hyperaspis lateralis* | 656 | KR489108 | BOLD:ACA7487 | Canada |
| Scymninae | *Hyperaspis* | *Hyperaspis lateralis* | 657 | KR487276 | BOLD:ACU7795 | Canada |
| Scymninae | *Hyperaspis* | *Hyperaspis oregona* | 658 | KM842010 | BOLD:ACC5893 | Canada |
| Scymninae | *Hyperaspis* | *Hyperaspis oregona* | 659 | KM848775 | BOLD:ACC5893 | Canada |
| Scymninae | *Hyperaspis* | *Hyperaspis proba* | 660 | KJ444321 | BOLD:ACA7425 | Canada |
| Scymninae | *Hyperaspis* | *Hyperaspis proba* | 661 | KJ445209 | BOLD:ACA7425 | Canada |
| Scymninae | *Hyperaspis* | *Hyperaspis reppensis* | 662 | KU907915 | BOLD:ACZ0893 | Germany |
| Scymninae | *Hyperaspis* | *Hyperaspis reppensis* | 663 | KU912574 | BOLD:ACZ0893 | Germany |
| Scymninae | *Hyperaspis* | *Hyperaspis undulata* | 664 | JN290355 | BOLD:AAH3292 | Canada |
| Scymninae | *Hyperaspis* | *Hyperaspis undulata* | 665 | KR482317 | BOLD:AAH3292 | Canada |
| Scymninae | *Hyperaspis* | *Hyperaspis undulata* | 666 | KR480997 | BOLD:AAH3292 | Canada |
| Scymninae | *Hyperaspis* | *Hyperaspis undulata* | 667 | JN279154 | BOLD:AAH3292 | Canada |
| Scymninae | *Nephus* | *Nephus quadrimaculatus* | 668 | KM441460 | BOLD:ACC4840 | Germany |
| Scymninae | *Nephus* | *Nephus quadrimaculatus* | 669 | KM441053 | BOLD:ACC4840 | Germany |
| Scymninae | *Nephus* | *Nephus quadrimaculatus* | 670 | KM445209 | BOLD:ACC4840 | Germany |
| Scymninae | *Nephus* | *Nephus quadrimaculatus* | 671 | KM450038 | BOLD:ACC4840 | Germany |
| Scymninae | *Nephus* | *Nephus quadrimaculatus* | 672 | KM447573 | BOLD:ACC4840 | Germany |
| Scymninae | *Nephus* | *Nephus quadrimaculatus* | 673 | KU908501 | BOLD:ACC4840 | Germany |
| Scymninae | *Nephus* | *Nephus redtenbacheri* | 674 | KJ965701 | BOLD:ABW4198 | Finland |
| Scymninae | *Nephus* | *Nephus redtenbacheri* | 675 | KM451158 | BOLD:ABW4198 | Austria |
| Scymninae | *Nephus* | *Nephus redtenbacheri* | 676 | KM440946 | BOLD:ABW4198 | Germany |
| Scymninae | *Nephus* | *Nephus redtenbacheri* | 677 | KM443786 | BOLD:ABW4198 | Germany |
| Scymninae | *Nephus* | *Nephus redtenbacheri* | 678 | KM451612 | BOLD:ABW4198 | Germany |
| Scymninae | *Nephus* | *Nephus redtenbacheri* | 679 | KU919339 | BOLD:ABW4198 | Germany |

## Supplementary Table S3 (continued)

| Subfamily | Genus | Species name | Code | GenBank accession number | BOLD BIN | Collection country |
|---|---|---|---|---|---|---|
| Scymninae | *Nephus* | *Nephus redtenbacheri* | 680 | KU918203 | BOLD:ABW4198 | Germany |
| Scymninae | *Nephus* | *Nephus redtenbacheri* | 681 | KU916398 | BOLD:ABW4198 | Germany |
| Scymninae | *Scymnus* | *Scymnus abietis* | 682 | KM448896 | BOLD:AAJ7337 | Germany |
| Scymninae | *Scymnus* | *Scymnus abietis* | 683 | KM445210 | BOLD:AAJ7337 | Germany |
| Scymninae | *Scymnus* | *Scymnus abietis* | 684 | KM440635 | BOLD:AAJ7337 | Germany |
| Scymninae | *Scymnus* | *Scymnus abietis* | 685 | KM447172 | BOLD:AAJ7337 | Germany |
| Scymninae | *Scymnus* | *Scymnus abietis* | 686 | KM446018 | BOLD:AAJ7337 | Austria |
| Scymninae | *Scymnus* | *Scymnus abietis* | 687 | KM446867 | BOLD:AAJ7337 | Austria |
| Scymninae | *Scymnus* | *Scymnus abietis* | 688 | KU914118 | BOLD:AAJ7337 | Germany |
| Scymninae | *Scymnus* | *Scymnus abietis* | 689 | KU914831 | BOLD:AAJ7337 | Germany |
| Scymninae | *Scymnus* | *Scymnus apetzi* | 690 | HQ953922 | BOLD:AAO3236 | Germany |
| Scymninae | *Scymnus* | *Scymnus apetzi* | 691 | HQ953923 | BOLD:AAO3236 | Germany |
| Scymninae | *Scymnus* | *Scymnus apetzi* | 692 | KM439621 | BOLD:AAO3236 | Germany |
| Scymninae | *Scymnus* | *Scymnus apetzi* | 693 | KM441259 | BOLD:AAO3236 | Germany |
| Scymninae | *Scymnus* | *Scymnus apetzi* | 694 | KM445528 | BOLD:AAO3236 | Germany |
| Scymninae | *Scymnus* | *Scymnus apetzi* | 695 | KM444007 | BOLD:AAO3236 | Germany |
| Scymninae | *Scymnus* | *Scymnus apetzi* | 696 | KM449572 | BOLD:AAO3236 | Germany |
| Scymninae | *Scymnus* | *Scymnus apetzi* | 697 | KM444720 | BOLD:AAO3236 | Germany |
| Scymninae | *Scymnus* | *Scymnus auritus* | 698 | HQ953832 | BOLD:AAO0044 | Belgium |
| Scymninae | *Scymnus* | *Scymnus auritus* | 699 | KM451681 | BOLD:AAO0044 | Germany |
| Scymninae | *Scymnus* | *Scymnus auritus* | 700 | KU908345 | BOLD:AAO0044 | Germany |
| Scymninae | *Scymnus* | *Scymnus auritus* | 701 | KU907994 | BOLD:AAO0044 | Germany |
| Scymninae | *Scymnus* | *Scymnus auritus* | 702 | | BOLD:AAO0044 | Germany |
| Scymninae | *Scymnus* | *Scymnus auritus* | 703 | | BOLD:AAO0044 | China |
| Scymninae | *Scymnus* | *Scymnus camptodromus* | 704 | | BOLD:ADB4729 | China |
| Scymninae | *Scymnus* | *Scymnus camptodromus* | 705 | | BOLD:ADB4623 | China |
| Scymninae | *Scymnus* | *Scymnus camptodromus* | 706 | | BOLD:ADB4624 | China |
| Scymninae | *Scymnus* | *Scymnus camptodromus* | 707 | | BOLD:ADB4624 | China |
| Scymninae | *Scymnus* | *Scymnus camptodromus* | 708 | | BOLD:ADB4624 | China |
| Scymninae | *Scymnus* | *Scymnus camptodromus* | 709 | | BOLD:ADB4624 | China |
| Scymninae | *Scymnus* | *Scymnus camptodromus* | 710 | | BOLD:ADB4624 | China |

## Supplementary Table S3 (continued)

| Subfamily | Genus | Species name | Code | GenBank accession number | BOLD BIN | Collection country |
|---|---|---|---|---|---|---|
| Scymninae | *Scymnus* | *Scymnus camptodromus* | 711 | | BOLD:ADB7467 | China |
| Scymninae | *Scymnus* | *Scymnus camptodromus* | 712 | | BOLD:ADB7467 | China |
| Scymninae | *Scymnus* | *Scymnus camptodromus* | 713 | | BOLD:ADB4623 | China |
| Scymninae | *Scymnus* | *Scymnus camptodromus* | 714 | | BOLD:ADB4729 | China |
| Scymninae | *Scymnus* | *Scymnus camptodromus* | 715 | | BOLD:ADB7467 | China |
| Scymninae | *Scymnus* | *Scymnus camptodromus* | 716 | | BOLD:ADB4623 | China |
| Scymninae | *Scymnus* | *Scymnus camptodromus* | 717 | | BOLD:ADB7467 | China |
| Scymninae | *Scymnus* | *Scymnus camptodromus* | 718 | | BOLD:ADB4623 | China |
| Scymninae | *Scymnus* | *Scymnus camptodromus* | 719 | | BOLD:ADB4624 | China |
| Scymninae | *Scymnus* | *Scymnus caurinus* | 720 | KR488821 | BOLD:ACL3086 | Canada |
| Scymninae | *Scymnus* | *Scymnus caurinus* | 721 | KR490680 | BOLD:ACL3086 | Canada |
| Scymninae | *Scymnus* | *Scymnus caurinus* | 722 | KR481353 | BOLD:ACL3086 | Canada |
| Scymninae | *Scymnus* | *Scymnus ferrugatus* | 723 | JF889715 | BOLD:AAP7197 | Germany |
| Scymninae | *Scymnus* | *Scymnus ferrugatus* | 724 | KM439508 | BOLD:AAP7197 | Germany |
| Scymninae | *Scymnus* | *Scymnus ferrugatus* | 725 | KM446678 | BOLD:AAP7197 | Germany |
| Scymninae | *Scymnus* | *Scymnus ferrugatus* | 726 | KM448093 | BOLD:AAP7197 | Germany |
| Scymninae | *Scymnus* | *Scymnus ferrugatus* | 727 | KU911648 | BOLD:AAP7197 | Germany |
| Scymninae | *Scymnus* | *Scymnus ferrugatus* | 728 | KU913590 | BOLD:AAP7197 | Germany |
| Scymninae | *Scymnus* | *Scymnus ferrugatus* | 729 | KU907073 | BOLD:AAP7197 | Germany |
| Scymninae | *Scymnus* | *Scymnus ferrugatus* | 730 | KU906312 | BOLD:AAP7197 | Germany |
| Scymninae | *Scymnus* | *Scymnus ferrugatus* | 731 | KU918430 | BOLD:AAP7197 | Germany |
| Scymninae | *Scymnus* | *Scymnus ferrugatus* | 732 | KU916215 | BOLD:AAP7197 | Germany |
| Scymninae | *Scymnus* | *Scymnus ferrugatus* | 733 | KU908446 | BOLD:AAP7197 | Germany |
| Scymninae | *Scymnus* | *Scymnus frontalis* | 734 | KJ966166 | BOLD:ACC1327 | Finland |
| Scymninae | *Scymnus* | *Scymnus frontalis* | 735 | KM446164 | BOLD:ACC1327 | Czech Republic |
| Scymninae | *Scymnus* | *Scymnus frontalis* | 736 | KU907730 | BOLD:ACC1327 | Germany |
| Scymninae | *Scymnus* | *Scymnus frontalis* | 737 | KU917801 | BOLD:ACC1327 | Germany |
| Scymninae | *Scymnus* | *Scymnus frontalis* | 738 | KU912068 | BOLD:ACC1327 | Germany |
| Scymninae | *Scymnus* | *Scymnus frontalis* | 739 | KU908070 | BOLD:ACC1327 | Germany |
| Scymninae | *Scymnus* | *Scymnus frontalis* | 740 | KU908034 | BOLD:ACC1327 | Germany |
| Scymninae | *Scymnus* | *Scymnus frontalis* | 741 | KU907750 | BOLD:ACC1327 | Germany |

## Supplementary Table S3 (continued)

| Subfamily | Genus | Species name | Code | GenBank accession number | BOLD BIN | Collection country |
|---|---|---|---|---|---|---|
| Scymninae | *Scymnus* | *Scymnus frontalis* | 742 | KU918248 | BOLD:ACC1327 | Germany |
| Scymninae | *Scymnus* | *Scymnus frontalis* | 743 | KU911364 | BOLD:ACC1327 | Germany |
| Scymninae | *Scymnus* | *Scymnus frontalis* | 744 | KU910093 | BOLD:ACC1327 | Germany |
| Scymninae | *Scymnus* | *Scymnus frontalis* | 745 | KU909265 | BOLD:ACC1327 | Czech Republic |
| Scymninae | *Scymnus* | *Scymnus frontalis* | 746 | KU906273 | BOLD:ACC1327 | Germany |
| Scymninae | *Scymnus* | *Scymnus haemorrhoidalis* | 747 | KJ966591 | BOLD:AAV8990 | Finland |
| Scymninae | *Scymnus* | *Scymnus haemorrhoidalis* | 748 | KJ964428 | BOLD:AAV8990 | Finland |
| Scymninae | *Scymnus* | *Scymnus haemorrhoidalis* | 749 | JN279151 | BOLD:AAV8990 | Germany |
| Scymninae | *Scymnus* | *Scymnus haemorrhoidalis* | 750 | KM439241 | BOLD:AAV8990 | Germany |
| Scymninae | *Scymnus* | *Scymnus haemorrhoidalis* | 751 | KM440387 | BOLD:AAV8990 | Germany |
| Scymninae | *Scymnus* | *Scymnus haemorrhoidalis* | 752 | KM445762 | BOLD:AAV8990 | Slovenia |
| Scymninae | *Scymnus* | *Scymnus haemorrhoidalis* | 753 | KU912538 | BOLD:AAV8990 | Germany |
| Scymninae | *Scymnus* | *Scymnus haemorrhoidalis* | 754 | KU916592 | BOLD:AAV8990 | Germany |
| Scymninae | *Scymnus* | *Scymnus haemorrhoidalis* | 755 | KU908863 | BOLD:AAV8990 | Germany |
| Scymninae | *Scymnus* | *Scymnus haemorrhoidalis* | 756 | KU912453 | BOLD:AAV8990 | Germany |
| Scymninae | *Scymnus* | *Scymnus haemorrhoidalis* | 757 | KU906576 | BOLD:AAV8990 | Germany |
| Scymninae | *Scymnus* | *Scymnus haemorrhoidalis* | 758 | KU910003 | BOLD:AAV8990 | Germany |
| Scymninae | *Scymnus* | *Scymnus interruptus* | 759 | KM450488 | BOLD:ACD0241 | Germany |
| Scymninae | *Scymnus* | *Scymnus interruptus* | 760 | KM440972 | BOLD:ACD0241 | Germany |
| Scymninae | *Scymnus* | *Scymnus interruptus* | 761 | KM446534 | BOLD:ACD0241 | Germany |
| Scymninae | *Scymnus* | *Scymnus interruptus* | 762 | KM444904 | BOLD:ACD0241 | Germany |
| Scymninae | *Scymnus* | *Scymnus interruptus* | 763 | KM447601 | BOLD:ACD0241 | Germany |
| Scymninae | *Scymnus* | *Scymnus interruptus* | 764 | KU907875 | BOLD:ACD0241 | Germany |
| Scymninae | *Scymnus* | *Scymnus interruptus* | 765 | KU910606 | BOLD:ACD0241 | Germany |
| Scymninae | *Scymnus* | *Scymnus interruptus* | 766 | KU918122 | BOLD:ACD0241 | Germany |
| Scymninae | *Scymnus* | *Scymnus interruptus* | 767 | KU906399 | BOLD:ACD0241 | Germany |
| Scymninae | *Scymnus* | *Scymnus interruptus* | 768 | KU909516 | BOLD:ACD0241 | Germany |
| Scymninae | *Scymnus* | *Scymnus interruptus* | 769 | KU907182 | BOLD:ACD0241 | Germany |
| Scymninae | *Scymnus* | *Scymnus loewii* | 770 | | BOLD:AAM7630 | United States |
| Scymninae | *Scymnus* | *Scymnus loewii* | 771 | | BOLD:AAM7630 | United States |
| Scymninae | *Scymnus* | *Scymnus loewii* | 772 | | BOLD:ADA9577 | United States |

## Supplementary Table S3 (continued)

| Subfamily | Genus | Species name | Code | GenBank accession number | BOLD BIN | Collection country |
|---|---|---|---|---|---|---|
| Scymninae | *Scymnus* | *Scymnus loewii* | 773 | | BOLD:ADA9577 | United States |
| Scymninae | *Scymnus* | *Scymnus loewii* | 774 | | BOLD:AAM7630 | United States |
| Scymninae | *Scymnus* | *Scymnus mimulus* | 775 | HQ954283 | BOLD:AAO4399 | Germany |
| Scymninae | *Scymnus* | *Scymnus mimulus* | 776 | KM440453 | BOLD:AAO4399 | Germany |
| Scymninae | *Scymnus* | *Scymnus mimulus* | 777 | KU918638 | BOLD:AAO4399 | Germany |
| Scymninae | *Scymnus* | *Scymnus mimulus* | 778 | KU914087 | BOLD:AAO4399 | Germany |
| Scymninae | *Scymnus* | *Scymnus mitior* | 779 | | BOLD:AAH3306 | Australia |
| Scymninae | *Scymnus* | *Scymnus mitior* | 780 | | BOLD:AAH3306 | Australia |
| Scymninae | *Scymnus* | *Scymnus nebulosus* | 781 | | BOLD:ABX2482 | United States |
| Scymninae | *Scymnus* | *Scymnus nebulosus* | 782 | KM850302 | BOLD:ABX2482 | Canada |
| Scymninae | *Scymnus* | *Scymnus nebulosus* | 783 | | BOLD:ABX2482 | United States |
| Scymninae | *Scymnus* | *Scymnus nigrinus* | 784 | KJ964392 | BOLD:ACC4579 | Finland |
| Scymninae | *Scymnus* | *Scymnus nigrinus* | 785 | KJ965464 | BOLD:ACC4579 | Finland |
| Scymninae | *Scymnus* | *Scymnus nigrinus* | 786 | KJ964304 | BOLD:ACC4579 | Finland |
| Scymninae | *Scymnus* | *Scymnus nigrinus* | 787 | KU918863 | BOLD:ACC4579 | Germany |
| Scymninae | *Scymnus* | *Scymnus ningshanensis* | 788 | | BOLD:ADB3647 | China |
| Scymninae | *Scymnus* | *Scymnus ningshanensis* | 789 | | BOLD:ADB3647 | China |
| Scymninae | *Scymnus* | *Scymnus ningshanensis* | 790 | | BOLD:ADB3647 | China |
| Scymninae | *Scymnus* | *Scymnus ningshanensis* | 791 | | BOLD:ADB3647 | China |
| Scymninae | *Scymnus* | *Scymnus ningshanensis* | 792 | | BOLD:ADB3647 | China |
| Scymninae | *Scymnus* | *Scymnus ningshanensis* | 793 | | BOLD:ADB3647 | China |
| Scymninae | *Scymnus* | *Scymnus ningshanensis* | 794 | | BOLD:ADB3647 | China |
| Scymninae | *Scymnus* | *Scymnus ningshanensis* | 795 | | BOLD:ADB3647 | China |
| Scymninae | *Scymnus* | *Scymnus nubilus* | 796 | | BOLD:AAP7968 | South Africa |
| Scymninae | *Scymnus* | *Scymnus nubilus* | 797 | | BOLD:AAP7968 | South Africa |
| Scymninae | *Scymnus* | *Scymnus nubilus* | 798 | KY842188 | BOLD:AAP7968 | Pakistan |
| Scymninae | *Scymnus* | *Scymnus nubilus* | 799 | KY829911 | BOLD:AAP7968 | Pakistan |
| Scymninae | *Scymnus* | *Scymnus nubilus* | 800 | KY839685 | BOLD:AAP7968 | Pakistan |
| Scymninae | *Scymnus* | *Scymnus nubilus* | 801 | KY829676 | BOLD:AAP7968 | Pakistan |
| Scymninae | *Scymnus* | *Scymnus nubilus* | 802 | KY844086 | BOLD:AAP7968 | Pakistan |
| Scymninae | *Scymnus* | *Scymnus nubilus* | 803 | KY833044 | BOLD:AAP7968 | Pakistan |

## Supplementary Table S3 (continued)

| Subfamily | Genus | Species name | Code | GenBank accession number | BOLD BIN | Collection country |
|---|---|---|---|---|---|---|
| Scymninae | *Scymnus* | *Scymnus nubilus* | 804 | KY836984 | BOLD:AAP7968 | Pakistan |
| Scymninae | *Scymnus* | *Scymnus nubilus* | 805 | KY830126 | BOLD:AAP7968 | Pakistan |
| Scymninae | *Scymnus* | *Scymnus nubilus* | 806 | KY839222 | BOLD:AAP7968 | Pakistan |
| Scymninae | *Scymnus* | *Scymnus nubilus* | 807 | KY834475 | BOLD:AAP7968 | Pakistan |
| Scymninae | *Scymnus* | *Scymnus nubilus* | 808 | KY835852 | BOLD:AAP7968 | Pakistan |
| Scymninae | *Scymnus* | *Scymnus nubilus* | 809 | KY846946 | BOLD:AAP7968 | Pakistan |
| Scymninae | *Scymnus* | *Scymnus nubilus* | 810 | KY846248 | BOLD:AAP7968 | Pakistan |
| Scymninae | *Scymnus* | *Scymnus pallipediformis* | 811 | KM451761 | BOLD:ACG3470 | Germany |
| Scymninae | *Scymnus* | *Scymnus pallipediformis* | 812 | KM448063 | BOLD:ACJ7446 | Germany |
| Scymninae | *Scymnus* | *Scymnus rubromaculatus* | 813 | HQ953816 | BOLD:AAN9250 | Germany |
| Scymninae | *Scymnus* | *Scymnus rubromaculatus* | 814 | HQ953858 | BOLD:AAN9250 | Germany |
| Scymninae | *Scymnus* | *Scymnus rubromaculatus* | 815 | HQ953889 | BOLD:AAN9250 | Germany |
| Scymninae | *Scymnus* | *Scymnus rubromaculatus* | 816 | KM439645 | BOLD:AAN9250 | Germany |
| Scymninae | *Scymnus* | *Scymnus rubromaculatus* | 817 | KM449986 | BOLD:AAN9250 | Germany |
| Scymninae | *Scymnus* | *Scymnus rubromaculatus* | 818 | KM450706 | BOLD:AAN9250 | Germany |
| Scymninae | *Scymnus* | *Scymnus rubromaculatus* | 819 | KU911134 | BOLD:AAN9250 | Germany |
| Scymninae | *Scymnus* | *Scymnus rubromaculatus* | 820 | KU909493 | BOLD:AAN9250 | Germany |
| Scymninae | *Scymnus* | *Scymnus rubromaculatus* | 821 | KU906947 | BOLD:AAN9250 | Germany |
| Scymninae | *Scymnus* | *Scymnus rubromaculatus* | 822 | KU909193 | BOLD:AAN9250 | Germany |
| Scymninae | *Scymnus* | *Scymnus rubromaculatus* | 823 | KU909972 | BOLD:AAN9250 | Germany |
| Scymninae | *Scymnus* | *Scymnus rubromaculatus* | 824 | KU910150 | BOLD:AAN9250 | Germany |
| Scymninae | *Scymnus* | *Scymnus rubromaculatus* | 825 | KM449518 | BOLD:AAN9250 | Germany |
| Scymninae | *Scymnus* | *Scymnus rubromaculatus* | 826 | KU907233 | BOLD:AAN9250 | Germany |
| Scymninae | *Scymnus* | *Scymnus rubromaculatus* | 827 | KU910496 | BOLD:AAN9250 | Germany |
| Scymninae | *Scymnus* | *Scymnus sinuanodulus* | 828 | | BOLD:ADB4579 | China |
| Scymninae | *Scymnus* | *Scymnus sinuanodulus* | 829 | | BOLD:ADB4579 | China |
| Scymninae | *Scymnus* | *Scymnus sinuanodulus* | 830 | | BOLD:ADB4579 | China |
| Scymninae | *Scymnus* | *Scymnus sinuanodulus* | 831 | | BOLD:ADB4579 | China |
| Scymninae | *Scymnus* | *Scymnus suturalis* | 832 | KJ964091 | BOLD:AAP8242 | Finland |
| Scymninae | *Scymnus* | *Scymnus suturalis* | 833 | JF889798 | BOLD:AAP8242 | Germany |
| Scymninae | *Scymnus* | *Scymnus suturalis* | 834 | KM444345 | BOLD:AAP8242 | Germany |

## Supplementary Table S3 (continued)

| Subfamily | Genus | Species name | Code | GenBank accession number | BOLD BIN | Collection country |
|---|---|---|---|---|---|---|
| Scymninae | *Scymnus* | *Scymnus suturalis* | 835 | KM441163 | BOLD:AAP8242 | Germany |
| Scymninae | *Scymnus* | *Scymnus suturalis* | 836 | KU913777 | BOLD:AAP8242 | Germany |
| Scymninae | *Scymnus* | *Scymnus suturalis* | 837 | KU918484 | BOLD:AAP8242 | Germany |
| Scymninae | *Scymnus* | *Scymnus suturalis* | 838 | KU907827 | BOLD:AAP8242 | Germany |
| Scymninae | *Scymnus* | *Scymnus suturalis* | 839 | KU916113 | BOLD:AAP8242 | Germany |
| Scymninae | *Scymnus* | *Scymnus suturalis* | 840 | KU916618 | BOLD:AAP8242 | Germany |
| Scymninae | *Scymnus* | *Scymnus suturalis* | 841 | KU919415 | BOLD:AAP8242 | Germany |
| Scymninae | *Scymnus* | *Scymnus suturalis* | 842 | KU918418 | BOLD:AAP8242 | Germany |
| Scymninae | *Scymnus* | *Scymnus suturalis* | 843 | KU909212 | BOLD:AAP8242 | Germany |
| Scymninae | *Scymnus* | *Scymnus suturalis* | 844 | KU912139 | BOLD:AAP8242 | Germany |
| Scymninae | *Scymnus* | *Scymnus suturalis* | 845 | KU909504 | BOLD:AAP8242 | Germany |
| Scymninae | *Scymnus* | *Scymnus suturalis* | 846 | KU908915 | BOLD:AAP8242 | Germany |
| Scymninae | *Scymnus* | *Scymnus suturalis* | 847 | KU909264 | BOLD:AAP8242 | Germany |
| Scymninae | *Scymnus* | *Scymnus suturalis* | 848 | KR489329 | BOLD:AAP8242 | Canada |
| Scymninae | *Stethorus* | *Stethorus punctillum* | 849 | KJ963340 | BOLD:AAN6149 | Finland |
| Scymninae | *Stethorus* | *Stethorus punctillum* | 850 | HQ942792 | BOLD:AAN6149 | Canada |
| Scymninae | *Stethorus* | *Stethorus punctillum* | 851 | JF889549 | BOLD:AAN6149 | Germany |
| Scymninae | *Stethorus* | *Stethorus punctillum* | 852 | KM446280 | BOLD:AAN6149 | Germany |
| Scymninae | *Stethorus* | *Stethorus punctillum* | 853 | KM450770 | BOLD:AAN6149 | Germany |
| Scymninae | *Stethorus* | *Stethorus punctillum* | 854 | KM448697 | BOLD:AAN6149 | Germany |
| Scymninae | *Stethorus* | *Stethorus punctillum* | 855 | KM439429 | BOLD:AAN6149 | Germany |
| Scymninae | *Stethorus* | *Stethorus punctillum* | 856 | KU912091 | BOLD:AAN6149 | Germany |
| Scymninae | *Stethorus* | *Stethorus punctillum* | 857 | KU915596 | BOLD:AAN6149 | Germany |
| Scymninae | *Stethorus* | *Stethorus punctillum* | 858 | KU910744 | BOLD:AAN6149 | Germany |
| Scymninae | *Stethorus* | *Stethorus punctillum* | 859 | KU914673 | BOLD:AAN6149 | Germany |
| Scymninae | *Stethorus* | *Stethorus punctillum* | 860 | KU916463 | BOLD:AAN6149 | Germany |
| Sticholotidinae | *Microweisea* | *Microweisea misella* | 861 | MF636637 | BOLD:ACU7801 | Canada |
| Sticholotidinae | *Microweisea* | *Microweisea misella* | 862 | MF638214 | BOLD:ACU9431 | Canada |
| Sticholotidinae | *Microweisea* | *Microweisea misella* | 863 | KR482303 | BOLD:ACU8237 | Canada |
| Sticholotidinae | *Microweisea* | *Microweisea misella* | 864 | | BOLD:ACU8237 | Canada |
| Sticholotidinae | *Microweisea* | *Microweisea misella* | 865 | | BOLD:ACU8237 | Canada |

**Supplementary Table S3 (continued)**

| Subfamily | Genus | Species name | Code | GenBank accession number | BOLD BIN | Collection country |
|---|---|---|---|---|---|---|
| Sticholotidinae | *Microweisea* | *Microweisea misella* | 866 | | BOLD:ACU8237 | Canada |
| Sticholotidinae | *Microweisea* | *Microweisea misella* | 867 | | BOLD:ACU8237 | Canada |
| Sticholotidinae | *Coccidophilus* | *Coccidophilus marginatus* | 868 | MG059043 | BOLD:ACP9704 | Canada |
| Sticholotidinae | *Coccidophilus* | *Coccidophilus marginatus* | 869 | KR480795 | BOLD:ACP9704 | Canada |
| Sticholotidinae | *Coccidophilus* | *Coccidophilus marginatus* | 870 | MG059627 | BOLD:ACC9999 | Canada |

# Supplementary Table S4 The intra-specific and inter-specific genetic distance of Coccinellidae

| | | |
|---|---|---|
| Scymnus_suturalis_832 | Scymnus_suturalis_841 | 0.000 |
| Scymnus_suturalis_835 | Scymnus_suturalis_841 | 0.000 |
| Scymnus_suturalis_836 | Scymnus_suturalis_841 | 0.000 |
| Scymnus_suturalis_839 | Scymnus_suturalis_841 | 0.000 |
| Scymnus_suturalis_832 | Scymnus_suturalis_842 | 0.000 |
| Scymnus_suturalis_835 | Scymnus_suturalis_842 | 0.000 |
| Scymnus_suturalis_836 | Scymnus_suturalis_842 | 0.000 |
| Scymnus_suturalis_839 | Scymnus_suturalis_842 | 0.000 |
| Scymnus_suturalis_841 | Scymnus_suturalis_842 | 0.000 |
| Scymnus_suturalis_832 | Scymnus_suturalis_844 | 0.000 |
| Scymnus_suturalis_835 | Scymnus_suturalis_844 | 0.000 |
| Scymnus_suturalis_836 | Scymnus_suturalis_844 | 0.000 |
| Scymnus_suturalis_839 | Scymnus_suturalis_844 | 0.000 |
| Scymnus_suturalis_841 | Scymnus_suturalis_844 | 0.000 |
| Scymnus_suturalis_842 | Scymnus_suturalis_844 | 0.000 |
| Scymnus_suturalis_832 | Scymnus_suturalis_846 | 0.000 |
| Scymnus_suturalis_835 | Scymnus_suturalis_846 | 0.000 |
| Scymnus_suturalis_836 | Scymnus_suturalis_846 | 0.000 |
| Scymnus_suturalis_839 | Scymnus_suturalis_846 | 0.000 |
| Scymnus_suturalis_841 | Scymnus_suturalis_846 | 0.000 |
| Scymnus_suturalis_842 | Scymnus_suturalis_846 | 0.000 |
| Scymnus_suturalis_844 | Scymnus_suturalis_846 | 0.000 |
| Scymnus_suturalis_832 | Scymnus_suturalis_847 | 0.000 |
| Scymnus_suturalis_835 | Scymnus_suturalis_847 | 0.000 |
| Scymnus_suturalis_836 | Scymnus_suturalis_847 | 0.000 |
| Scymnus_suturalis_839 | Scymnus_suturalis_847 | 0.000 |
| Scymnus_suturalis_841 | Scymnus_suturalis_847 | 0.000 |
| Scymnus_suturalis_842 | Scymnus_suturalis_847 | 0.000 |
| Scymnus_suturalis_844 | Scymnus_suturalis_847 | 0.000 |
| Scymnus_suturalis_846 | Scymnus_suturalis_847 | 0.000 |
| Scymnus_suturalis_832 | Scymnus_suturalis_848 | 0.000 |
| Scymnus_suturalis_835 | Scymnus_suturalis_848 | 0.000 |
| Scymnus_suturalis_836 | Scymnus_suturalis_848 | 0.000 |
| Scymnus_suturalis_839 | Scymnus_suturalis_848 | 0.000 |
| Scymnus_suturalis_841 | Scymnus_suturalis_848 | 0.000 |
| Scymnus_suturalis_842 | Scymnus_suturalis_848 | 0.000 |
| Scymnus_suturalis_844 | Scymnus_suturalis_848 | 0.000 |
| Scymnus_suturalis_846 | Scymnus_suturalis_848 | 0.000 |
| Scymnus_suturalis_847 | Scymnus_suturalis_848 | 0.000 |
| Stethorus_punctillum_854 | Stethorus_punctillum_856 | 0.000 |
| Stethorus_punctillum_851 | Stethorus_punctillum_858 | 0.000 |
| Subcoccinella_vigintiquatuorpunctata_620 | Subcoccinella_vigintiquatuorpunctata_621 | 0.000 |
| Subcoccinella_vigintiquatuorpunctata_620 | Subcoccinella_vigintiquatuorpunctata_628 | 0.000 |
| Subcoccinella_vigintiquatuorpunctata_621 | Subcoccinella_vigintiquatuorpunctata_628 | 0.000 |
| Subcoccinella_vigintiquatuorpunctata_620 | Subcoccinella_vigintiquatuorpunctata_629 | 0.000 |
| Subcoccinella_vigintiquatuorpunctata_621 | Subcoccinella_vigintiquatuorpunctata_629 | 0.000 |
| Subcoccinella_vigintiquatuorpunctata_628 | Subcoccinella_vigintiquatuorpunctata_629 | 0.000 |
| Subcoccinella_vigintiquatuorpunctata_620 | Subcoccinella_vigintiquatuorpunctata_631 | 0.000 |
| Subcoccinella_vigintiquatuorpunctata_621 | Subcoccinella_vigintiquatuorpunctata_631 | 0.000 |
| Subcoccinella_vigintiquatuorpunctata_628 | Subcoccinella_vigintiquatuorpunctata_631 | 0.000 |
| Subcoccinella_vigintiquatuorpunctata_629 | Subcoccinella_vigintiquatuorpunctata_631 | 0.000 |
| Tytthaspis_sedecimpunctata_577 | Tytthaspis_sedecimpunctata_578 | 0.000 |
| Tytthaspis_sedecimpunctata_577 | Tytthaspis_sedecimpunctata_579 | 0.000 |
| Tytthaspis_sedecimpunctata_578 | Tytthaspis_sedecimpunctata_579 | 0.000 |
| Tytthaspis_sedecimpunctata_577 | Tytthaspis_sedecimpunctata_581 | 0.000 |
| Tytthaspis_sedecimpunctata_578 | Tytthaspis_sedecimpunctata_581 | 0.000 |

Supplementary Table S4 (continued)

| | | |
|---|---|---|
| Tytthaspis_sedecimpunctata_579 | Tytthaspis_sedecimpunctata_581 | 0.000 |
| Tytthaspis_sedecimpunctata_577 | Tytthaspis_sedecimpunctata_582 | 0.000 |
| Tytthaspis_sedecimpunctata_578 | Tytthaspis_sedecimpunctata_582 | 0.000 |
| Tytthaspis_sedecimpunctata_579 | Tytthaspis_sedecimpunctata_582 | 0.000 |
| Tytthaspis_sedecimpunctata_581 | Tytthaspis_sedecimpunctata_582 | 0.000 |
| Tytthaspis_sedecimpunctata_577 | Tytthaspis_sedecimpunctata_590 | 0.000 |
| Tytthaspis_sedecimpunctata_578 | Tytthaspis_sedecimpunctata_590 | 0.000 |
| Tytthaspis_sedecimpunctata_579 | Tytthaspis_sedecimpunctata_590 | 0.000 |
| Tytthaspis_sedecimpunctata_581 | Tytthaspis_sedecimpunctata_590 | 0.000 |
| Tytthaspis_sedecimpunctata_582 | Tytthaspis_sedecimpunctata_590 | 0.000 |
| Tytthaspis_sedecimpunctata_577 | Tytthaspis_sedecimpunctata_594 | 0.000 |
| Tytthaspis_sedecimpunctata_578 | Tytthaspis_sedecimpunctata_594 | 0.000 |
| Tytthaspis_sedecimpunctata_579 | Tytthaspis_sedecimpunctata_594 | 0.000 |
| Tytthaspis_sedecimpunctata_581 | Tytthaspis_sedecimpunctata_594 | 0.000 |
| Tytthaspis_sedecimpunctata_582 | Tytthaspis_sedecimpunctata_594 | 0.000 |
| Tytthaspis_sedecimpunctata_590 | Tytthaspis_sedecimpunctata_594 | 0.000 |
| Tytthaspis_sedecimpunctata_577 | Tytthaspis_sedecimpunctata_595 | 0.000 |
| Tytthaspis_sedecimpunctata_578 | Tytthaspis_sedecimpunctata_595 | 0.000 |
| Tytthaspis_sedecimpunctata_579 | Tytthaspis_sedecimpunctata_595 | 0.000 |
| Tytthaspis_sedecimpunctata_581 | Tytthaspis_sedecimpunctata_595 | 0.000 |
| Tytthaspis_sedecimpunctata_582 | Tytthaspis_sedecimpunctata_595 | 0.000 |
| Tytthaspis_sedecimpunctata_590 | Tytthaspis_sedecimpunctata_595 | 0.000 |
| Tytthaspis_sedecimpunctata_594 | Tytthaspis_sedecimpunctata_595 | 0.000 |
| Vibidia_duodecimguttata_597 | Vibidia_duodecimguttata_600 | 0.000 |
| Vibidia_duodecimguttata_599 | Vibidia_duodecimguttata_602 | 0.000 |
| Scymnus_rubromaculatus_813 | Scymnus_rubromaculatus_822 | 0.000 |
| Scymnus_rubromaculatus_815 | Scymnus_rubromaculatus_822 | 0.000 |
| Scymnus_rubromaculatus_816 | Scymnus_rubromaculatus_822 | 0.000 |
| Scymnus_rubromaculatus_817 | Scymnus_rubromaculatus_822 | 0.000 |
| Scymnus_rubromaculatus_818 | Scymnus_rubromaculatus_822 | 0.000 |
| Scymnus_rubromaculatus_819 | Scymnus_rubromaculatus_822 | 0.000 |
| Scymnus_rubromaculatus_820 | Scymnus_rubromaculatus_822 | 0.000 |
| Scymnus_rubromaculatus_821 | Scymnus_rubromaculatus_822 | 0.000 |
| Scymnus_rubromaculatus_813 | Scymnus_rubromaculatus_823 | 0.000 |
| Scymnus_rubromaculatus_815 | Scymnus_rubromaculatus_823 | 0.000 |
| Scymnus_rubromaculatus_816 | Scymnus_rubromaculatus_823 | 0.000 |
| Scymnus_rubromaculatus_817 | Scymnus_rubromaculatus_823 | 0.000 |
| Scymnus_rubromaculatus_818 | Scymnus_rubromaculatus_823 | 0.000 |
| Scymnus_rubromaculatus_819 | Scymnus_rubromaculatus_823 | 0.000 |
| Scymnus_rubromaculatus_820 | Scymnus_rubromaculatus_823 | 0.000 |
| Scymnus_rubromaculatus_821 | Scymnus_rubromaculatus_823 | 0.000 |
| Scymnus_rubromaculatus_822 | Scymnus_rubromaculatus_823 | 0.000 |
| Scymnus_rubromaculatus_813 | Scymnus_rubromaculatus_824 | 0.000 |
| Scymnus_rubromaculatus_815 | Scymnus_rubromaculatus_824 | 0.000 |
| Scymnus_rubromaculatus_816 | Scymnus_rubromaculatus_824 | 0.000 |
| Scymnus_rubromaculatus_817 | Scymnus_rubromaculatus_824 | 0.000 |
| Scymnus_rubromaculatus_818 | Scymnus_rubromaculatus_824 | 0.000 |
| Scymnus_rubromaculatus_819 | Scymnus_rubromaculatus_824 | 0.000 |
| Scymnus_rubromaculatus_820 | Scymnus_rubromaculatus_824 | 0.000 |
| Scymnus_rubromaculatus_821 | Scymnus_rubromaculatus_824 | 0.000 |
| Scymnus_rubromaculatus_822 | Scymnus_rubromaculatus_824 | 0.000 |
| Scymnus_rubromaculatus_823 | Scymnus_rubromaculatus_824 | 0.000 |
| Scymnus_rubromaculatus_813 | Scymnus_rubromaculatus_825 | 0.000 |
| Scymnus_rubromaculatus_815 | Scymnus_rubromaculatus_825 | 0.000 |
| Scymnus_rubromaculatus_816 | Scymnus_rubromaculatus_825 | 0.000 |
| Scymnus_rubromaculatus_817 | Scymnus_rubromaculatus_825 | 0.000 |

| | | |
|---|---|---|
| Scymnus_rubromaculatus_818 | Scymnus_rubromaculatus_825 | 0.000 |
| Scymnus_rubromaculatus_819 | Scymnus_rubromaculatus_825 | 0.000 |
| Scymnus_rubromaculatus_820 | Scymnus_rubromaculatus_825 | 0.000 |
| Scymnus_rubromaculatus_821 | Scymnus_rubromaculatus_825 | 0.000 |
| Scymnus_rubromaculatus_822 | Scymnus_rubromaculatus_825 | 0.000 |
| Scymnus_rubromaculatus_823 | Scymnus_rubromaculatus_825 | 0.000 |
| Scymnus_rubromaculatus_824 | Scymnus_rubromaculatus_825 | 0.000 |
| Scymnus_rubromaculatus_813 | Scymnus_rubromaculatus_826 | 0.000 |
| Scymnus_rubromaculatus_815 | Scymnus_rubromaculatus_826 | 0.000 |
| Scymnus_rubromaculatus_816 | Scymnus_rubromaculatus_826 | 0.000 |
| Scymnus_rubromaculatus_817 | Scymnus_rubromaculatus_826 | 0.000 |
| Scymnus_rubromaculatus_818 | Scymnus_rubromaculatus_826 | 0.000 |
| Scymnus_rubromaculatus_819 | Scymnus_rubromaculatus_826 | 0.000 |
| Scymnus_rubromaculatus_820 | Scymnus_rubromaculatus_826 | 0.000 |
| Scymnus_rubromaculatus_821 | Scymnus_rubromaculatus_826 | 0.000 |
| Scymnus_rubromaculatus_822 | Scymnus_rubromaculatus_826 | 0.000 |
| Scymnus_rubromaculatus_823 | Scymnus_rubromaculatus_826 | 0.000 |
| Scymnus_rubromaculatus_824 | Scymnus_rubromaculatus_826 | 0.000 |
| Scymnus_rubromaculatus_825 | Scymnus_rubromaculatus_826 | 0.000 |
| Scymnus_rubromaculatus_813 | Scymnus_rubromaculatus_827 | 0.000 |
| Scymnus_rubromaculatus_815 | Scymnus_rubromaculatus_827 | 0.000 |
| Scymnus_rubromaculatus_816 | Scymnus_rubromaculatus_827 | 0.000 |
| Scymnus_rubromaculatus_817 | Scymnus_rubromaculatus_827 | 0.000 |
| Scymnus_rubromaculatus_818 | Scymnus_rubromaculatus_827 | 0.000 |
| Scymnus_rubromaculatus_819 | Scymnus_rubromaculatus_827 | 0.000 |
| Scymnus_rubromaculatus_820 | Scymnus_rubromaculatus_827 | 0.000 |
| Scymnus_rubromaculatus_821 | Scymnus_rubromaculatus_827 | 0.000 |
| Scymnus_rubromaculatus_822 | Scymnus_rubromaculatus_827 | 0.000 |
| Scymnus_rubromaculatus_823 | Scymnus_rubromaculatus_827 | 0.000 |
| Scymnus_rubromaculatus_824 | Scymnus_rubromaculatus_827 | 0.000 |
| Scymnus_rubromaculatus_825 | Scymnus_rubromaculatus_827 | 0.000 |
| Scymnus_rubromaculatus_826 | Scymnus_rubromaculatus_827 | 0.000 |
| Scymnus_suturalis_832 | Scymnus_suturalis_835 | 0.000 |
| Scymnus_suturalis_832 | Scymnus_suturalis_836 | 0.000 |
| Scymnus_suturalis_835 | Scymnus_suturalis_836 | 0.000 |
| Scymnus_suturalis_832 | Scymnus_suturalis_839 | 0.000 |
| Scymnus_suturalis_835 | Scymnus_suturalis_839 | 0.000 |
| Scymnus_suturalis_836 | Scymnus_suturalis_839 | 0.000 |
| Scymnus_suturalis_838 | Scymnus_suturalis_841 | 0.002 |
| Scymnus_suturalis_840 | Scymnus_suturalis_841 | 0.002 |
| Scymnus_suturalis_838 | Scymnus_suturalis_842 | 0.002 |
| Scymnus_suturalis_840 | Scymnus_suturalis_842 | 0.002 |
| Scymnus_suturalis_838 | Scymnus_suturalis_844 | 0.002 |
| Scymnus_suturalis_840 | Scymnus_suturalis_844 | 0.002 |
| Scymnus_suturalis_838 | Scymnus_suturalis_846 | 0.002 |
| Scymnus_suturalis_840 | Scymnus_suturalis_846 | 0.002 |
| Scymnus_suturalis_838 | Scymnus_suturalis_847 | 0.002 |
| Scymnus_suturalis_840 | Scymnus_suturalis_847 | 0.002 |
| Scymnus_suturalis_838 | Scymnus_suturalis_848 | 0.002 |
| Scymnus_suturalis_840 | Scymnus_suturalis_848 | 0.002 |
| Subcoccinella_vigintiquatuorpunctata_620 | Subcoccinella_vigintiquatuorpunctata_623 | 0.002 |
| Subcoccinella_vigintiquatuorpunctata_621 | Subcoccinella_vigintiquatuorpunctata_623 | 0.002 |
| Subcoccinella_vigintiquatuorpunctata_623 | Subcoccinella_vigintiquatuorpunctata_628 | 0.002 |
| Subcoccinella_vigintiquatuorpunctata_623 | Subcoccinella_vigintiquatuorpunctata_629 | 0.002 |
| Subcoccinella_vigintiquatuorpunctata_623 | Subcoccinella_vigintiquatuorpunctata_631 | 0.002 |
| Tytthaspis_sedecimpunctata_577 | Tytthaspis_sedecimpunctata_580 | 0.002 |

| | | |
|---|---|---|
| Tytthaspis_sedecimpunctata_578 | Tytthaspis_sedecimpunctata_580 | 0.002 |
| Tytthaspis_sedecimpunctata_579 | Tytthaspis_sedecimpunctata_580 | 0.002 |
| Tytthaspis_sedecimpunctata_580 | Tytthaspis_sedecimpunctata_581 | 0.002 |
| Tytthaspis_sedecimpunctata_580 | Tytthaspis_sedecimpunctata_582 | 0.002 |
| Tytthaspis_sedecimpunctata_580 | Tytthaspis_sedecimpunctata_590 | 0.002 |
| Tytthaspis_sedecimpunctata_580 | Tytthaspis_sedecimpunctata_594 | 0.002 |
| Tytthaspis_sedecimpunctata_580 | Tytthaspis_sedecimpunctata_595 | 0.002 |
| Scymnus_suturalis_832 | Scymnus_suturalis_838 | 0.002 |
| Scymnus_suturalis_835 | Scymnus_suturalis_838 | 0.002 |
| Scymnus_suturalis_836 | Scymnus_suturalis_838 | 0.002 |
| Scymnus_suturalis_838 | Scymnus_suturalis_839 | 0.002 |
| Scymnus_suturalis_832 | Scymnus_suturalis_840 | 0.002 |
| Scymnus_suturalis_835 | Scymnus_suturalis_840 | 0.002 |
| Scymnus_suturalis_836 | Scymnus_suturalis_840 | 0.002 |
| Scymnus_suturalis_839 | Scymnus_suturalis_840 | 0.002 |
| Scymnus_suturalis_834 | Scymnus_suturalis_841 | 0.002 |
| Scymnus_suturalis_834 | Scymnus_suturalis_842 | 0.002 |
| Scymnus_suturalis_832 | Scymnus_suturalis_843 | 0.002 |
| Scymnus_suturalis_835 | Scymnus_suturalis_843 | 0.002 |
| Scymnus_suturalis_836 | Scymnus_suturalis_843 | 0.002 |
| Scymnus_suturalis_839 | Scymnus_suturalis_843 | 0.002 |
| Scymnus_suturalis_841 | Scymnus_suturalis_843 | 0.002 |
| Scymnus_suturalis_842 | Scymnus_suturalis_843 | 0.002 |
| Scymnus_suturalis_834 | Scymnus_suturalis_844 | 0.002 |
| Scymnus_suturalis_843 | Scymnus_suturalis_844 | 0.002 |
| Scymnus_suturalis_832 | Scymnus_suturalis_845 | 0.002 |
| Scymnus_suturalis_835 | Scymnus_suturalis_845 | 0.002 |
| Scymnus_suturalis_836 | Scymnus_suturalis_845 | 0.002 |
| Scymnus_suturalis_839 | Scymnus_suturalis_845 | 0.002 |
| Scymnus_suturalis_841 | Scymnus_suturalis_845 | 0.002 |
| Scymnus_suturalis_842 | Scymnus_suturalis_845 | 0.002 |
| Scymnus_suturalis_844 | Scymnus_suturalis_845 | 0.002 |
| Scymnus_suturalis_834 | Scymnus_suturalis_846 | 0.002 |
| Scymnus_suturalis_843 | Scymnus_suturalis_846 | 0.002 |
| Scymnus_suturalis_845 | Scymnus_suturalis_846 | 0.002 |
| Scymnus_suturalis_834 | Scymnus_suturalis_847 | 0.002 |
| Scymnus_suturalis_843 | Scymnus_suturalis_847 | 0.002 |
| Scymnus_suturalis_845 | Scymnus_suturalis_847 | 0.002 |
| Scymnus_suturalis_834 | Scymnus_suturalis_848 | 0.002 |
| Scymnus_suturalis_843 | Scymnus_suturalis_848 | 0.002 |
| Scymnus_suturalis_845 | Scymnus_suturalis_848 | 0.002 |
| Stethorus_punctillum_853 | Stethorus_punctillum_855 | 0.002 |
| Subcoccinella_vigintiquatuorpunctata_620 | Subcoccinella_vigintiquatuorpunctata_626 | 0.002 |
| Subcoccinella_vigintiquatuorpunctata_621 | Subcoccinella_vigintiquatuorpunctata_626 | 0.002 |
| Subcoccinella_vigintiquatuorpunctata_626 | Subcoccinella_vigintiquatuorpunctata_628 | 0.002 |
| Subcoccinella_vigintiquatuorpunctata_626 | Subcoccinella_vigintiquatuorpunctata_629 | 0.002 |
| Subcoccinella_vigintiquatuorpunctata_626 | Subcoccinella_vigintiquatuorpunctata_631 | 0.002 |
| Tytthaspis_sedecimpunctata_586 | Tytthaspis_sedecimpunctata_588 | 0.002 |
| Tytthaspis_sedecimpunctata_577 | Tytthaspis_sedecimpunctata_592 | 0.002 |
| Tytthaspis_sedecimpunctata_578 | Tytthaspis_sedecimpunctata_592 | 0.002 |
| Tytthaspis_sedecimpunctata_579 | Tytthaspis_sedecimpunctata_592 | 0.002 |
| Tytthaspis_sedecimpunctata_581 | Tytthaspis_sedecimpunctata_592 | 0.002 |
| Tytthaspis_sedecimpunctata_582 | Tytthaspis_sedecimpunctata_592 | 0.002 |
| Tytthaspis_sedecimpunctata_590 | Tytthaspis_sedecimpunctata_592 | 0.002 |
| Tytthaspis_sedecimpunctata_592 | Tytthaspis_sedecimpunctata_594 | 0.002 |
| Tytthaspis_sedecimpunctata_592 | Tytthaspis_sedecimpunctata_595 | 0.002 |

| | | |
|---|---|---|
| Vibidia_duodecimguttata_599 | Vibidia_duodecimguttata_601 | 0.002 |
| Vibidia_duodecimguttata_601 | Vibidia_duodecimguttata_602 | 0.002 |
| Scymnus_rubromaculatus_814 | Scymnus_rubromaculatus_822 | 0.002 |
| Scymnus_rubromaculatus_814 | Scymnus_rubromaculatus_823 | 0.002 |
| Scymnus_rubromaculatus_814 | Scymnus_rubromaculatus_824 | 0.002 |
| Scymnus_rubromaculatus_814 | Scymnus_rubromaculatus_825 | 0.002 |
| Scymnus_rubromaculatus_814 | Scymnus_rubromaculatus_826 | 0.002 |
| Scymnus_rubromaculatus_814 | Scymnus_rubromaculatus_827 | 0.002 |
| Scymnus_suturalis_832 | Scymnus_suturalis_834 | 0.002 |
| Scymnus_suturalis_834 | Scymnus_suturalis_835 | 0.002 |
| Scymnus_suturalis_834 | Scymnus_suturalis_836 | 0.002 |
| Scymnus_suturalis_834 | Scymnus_suturalis_839 | 0.002 |
| Scymnus_sinuanodulus_829 | Scymnus_sinuanodulus_831 | 0.002 |
| Scymnus_sinuanodulus_828 | Scymnus_sinuanodulus_829 | 0.002 |
| Scymnus_suturalis_838 | Scymnus_suturalis_843 | 0.003 |
| Scymnus_suturalis_840 | Scymnus_suturalis_843 | 0.003 |
| Scymnus_suturalis_838 | Scymnus_suturalis_845 | 0.003 |
| Scymnus_suturalis_840 | Scymnus_suturalis_845 | 0.003 |
| Subcoccinella_vigintiquatuorpunctata_623 | Subcoccinella_vigintiquatuorpunctata_626 | 0.003 |
| Tytthaspis_sedecimpunctata_580 | Tytthaspis_sedecimpunctata_592 | 0.003 |
| Scymnus_suturalis_834 | Scymnus_suturalis_838 | 0.003 |
| Scymnus_suturalis_834 | Scymnus_suturalis_840 | 0.003 |
| Scymnus_suturalis_838 | Scymnus_suturalis_840 | 0.003 |
| Scymnus_suturalis_837 | Scymnus_suturalis_841 | 0.003 |
| Scymnus_suturalis_837 | Scymnus_suturalis_842 | 0.003 |
| Scymnus_suturalis_834 | Scymnus_suturalis_843 | 0.003 |
| Scymnus_suturalis_837 | Scymnus_suturalis_844 | 0.003 |
| Scymnus_suturalis_834 | Scymnus_suturalis_845 | 0.003 |
| Scymnus_suturalis_843 | Scymnus_suturalis_845 | 0.003 |
| Scymnus_suturalis_837 | Scymnus_suturalis_846 | 0.003 |
| Scymnus_suturalis_837 | Scymnus_suturalis_847 | 0.003 |
| Scymnus_suturalis_837 | Scymnus_suturalis_848 | 0.003 |
| Stethorus_punctillum_854 | Stethorus_punctillum_860 | 0.003 |
| Stethorus_punctillum_856 | Stethorus_punctillum_860 | 0.003 |
| Subcoccinella_vigintiquatuorpunctata_620 | Subcoccinella_vigintiquatuorpunctata_627 | 0.003 |
| Subcoccinella_vigintiquatuorpunctata_621 | Subcoccinella_vigintiquatuorpunctata_627 | 0.003 |
| Subcoccinella_vigintiquatuorpunctata_627 | Subcoccinella_vigintiquatuorpunctata_628 | 0.003 |
| Subcoccinella_vigintiquatuorpunctata_627 | Subcoccinella_vigintiquatuorpunctata_629 | 0.003 |
| Subcoccinella_vigintiquatuorpunctata_620 | Subcoccinella_vigintiquatuorpunctata_630 | 0.003 |
| Subcoccinella_vigintiquatuorpunctata_621 | Subcoccinella_vigintiquatuorpunctata_630 | 0.003 |
| Subcoccinella_vigintiquatuorpunctata_627 | Subcoccinella_vigintiquatuorpunctata_630 | 0.003 |
| Subcoccinella_vigintiquatuorpunctata_628 | Subcoccinella_vigintiquatuorpunctata_630 | 0.003 |
| Subcoccinella_vigintiquatuorpunctata_629 | Subcoccinella_vigintiquatuorpunctata_630 | 0.003 |
| Subcoccinella_vigintiquatuorpunctata_627 | Subcoccinella_vigintiquatuorpunctata_631 | 0.003 |
| Subcoccinella_vigintiquatuorpunctata_630 | Subcoccinella_vigintiquatuorpunctata_631 | 0.003 |
| Tytthaspis_sedecimpunctata_585 | Tytthaspis_sedecimpunctata_592 | 0.003 |
| Tytthaspis_sedecimpunctata_577 | Tytthaspis_sedecimpunctata_593 | 0.003 |
| Tytthaspis_sedecimpunctata_578 | Tytthaspis_sedecimpunctata_593 | 0.003 |
| Tytthaspis_sedecimpunctata_579 | Tytthaspis_sedecimpunctata_593 | 0.003 |
| Tytthaspis_sedecimpunctata_581 | Tytthaspis_sedecimpunctata_593 | 0.003 |
| Tytthaspis_sedecimpunctata_582 | Tytthaspis_sedecimpunctata_593 | 0.003 |
| Tytthaspis_sedecimpunctata_590 | Tytthaspis_sedecimpunctata_593 | 0.003 |
| Tytthaspis_sedecimpunctata_593 | Tytthaspis_sedecimpunctata_594 | 0.003 |
| Tytthaspis_sedecimpunctata_593 | Tytthaspis_sedecimpunctata_595 | 0.003 |
| Vibidia_duodecimguttata_599 | Vibidia_duodecimguttata_603 | 0.003 |
| Vibidia_duodecimguttata_602 | Vibidia_duodecimguttata_603 | 0.003 |

| | | |
|---|---|---|
| Scymnus_suturalis_832 | Scymnus_suturalis_837 | 0.003 |
| Scymnus_suturalis_835 | Scymnus_suturalis_837 | 0.003 |
| Scymnus_suturalis_836 | Scymnus_suturalis_837 | 0.003 |
| Scymnus_suturalis_837 | Scymnus_suturalis_839 | 0.003 |
| Scymnus_sinuanodulus_828 | Scymnus_sinuanodulus_831 | 0.003 |
| Subcoccinella_vigintiquatuorpunctata_623 | Subcoccinella_vigintiquatuorpunctata_627 | 0.005 |
| Subcoccinella_vigintiquatuorpunctata_623 | Subcoccinella_vigintiquatuorpunctata_630 | 0.005 |
| Tytthaspis_sedecimpunctata_588 | Tytthaspis_sedecimpunctata_589 | 0.005 |
| Tytthaspis_sedecimpunctata_580 | Tytthaspis_sedecimpunctata_593 | 0.005 |
| Vibidia_duodecimguttata_596 | Vibidia_duodecimguttata_597 | 0.005 |
| Vibidia_duodecimguttata_596 | Vibidia_duodecimguttata_600 | 0.005 |
| Scymnus_suturalis_837 | Scymnus_suturalis_838 | 0.005 |
| Scymnus_suturalis_837 | Scymnus_suturalis_840 | 0.005 |
| Scymnus_suturalis_837 | Scymnus_suturalis_843 | 0.005 |
| Scymnus_suturalis_837 | Scymnus_suturalis_845 | 0.005 |
| Subcoccinella_vigintiquatuorpunctata_620 | Subcoccinella_vigintiquatuorpunctata_622 | 0.005 |
| Subcoccinella_vigintiquatuorpunctata_621 | Subcoccinella_vigintiquatuorpunctata_622 | 0.005 |
| Subcoccinella_vigintiquatuorpunctata_622 | Subcoccinella_vigintiquatuorpunctata_627 | 0.005 |
| Subcoccinella_vigintiquatuorpunctata_626 | Subcoccinella_vigintiquatuorpunctata_627 | 0.005 |
| Subcoccinella_vigintiquatuorpunctata_622 | Subcoccinella_vigintiquatuorpunctata_628 | 0.005 |
| Subcoccinella_vigintiquatuorpunctata_622 | Subcoccinella_vigintiquatuorpunctata_629 | 0.005 |
| Subcoccinella_vigintiquatuorpunctata_622 | Subcoccinella_vigintiquatuorpunctata_630 | 0.005 |
| Subcoccinella_vigintiquatuorpunctata_626 | Subcoccinella_vigintiquatuorpunctata_630 | 0.005 |
| Subcoccinella_vigintiquatuorpunctata_622 | Subcoccinella_vigintiquatuorpunctata_631 | 0.005 |
| Tytthaspis_sedecimpunctata_577 | Tytthaspis_sedecimpunctata_583 | 0.005 |
| Tytthaspis_sedecimpunctata_578 | Tytthaspis_sedecimpunctata_583 | 0.005 |
| Tytthaspis_sedecimpunctata_579 | Tytthaspis_sedecimpunctata_583 | 0.005 |
| Tytthaspis_sedecimpunctata_581 | Tytthaspis_sedecimpunctata_583 | 0.005 |
| Tytthaspis_sedecimpunctata_582 | Tytthaspis_sedecimpunctata_583 | 0.005 |
| Tytthaspis_sedecimpunctata_577 | Tytthaspis_sedecimpunctata_585 | 0.005 |
| Tytthaspis_sedecimpunctata_578 | Tytthaspis_sedecimpunctata_585 | 0.005 |
| Tytthaspis_sedecimpunctata_579 | Tytthaspis_sedecimpunctata_585 | 0.005 |
| Tytthaspis_sedecimpunctata_581 | Tytthaspis_sedecimpunctata_585 | 0.005 |
| Tytthaspis_sedecimpunctata_582 | Tytthaspis_sedecimpunctata_585 | 0.005 |
| Tytthaspis_sedecimpunctata_587 | Tytthaspis_sedecimpunctata_588 | 0.005 |
| Tytthaspis_sedecimpunctata_583 | Tytthaspis_sedecimpunctata_590 | 0.005 |
| Tytthaspis_sedecimpunctata_585 | Tytthaspis_sedecimpunctata_590 | 0.005 |
| Tytthaspis_sedecimpunctata_592 | Tytthaspis_sedecimpunctata_593 | 0.005 |
| Tytthaspis_sedecimpunctata_583 | Tytthaspis_sedecimpunctata_594 | 0.005 |
| Tytthaspis_sedecimpunctata_585 | Tytthaspis_sedecimpunctata_594 | 0.005 |
| Tytthaspis_sedecimpunctata_583 | Tytthaspis_sedecimpunctata_595 | 0.005 |
| Tytthaspis_sedecimpunctata_585 | Tytthaspis_sedecimpunctata_595 | 0.005 |
| Vibidia_duodecimguttata_596 | Vibidia_duodecimguttata_598 | 0.005 |
| Vibidia_duodecimguttata_601 | Vibidia_duodecimguttata_603 | 0.005 |
| Scymnus_suturalis_834 | Scymnus_suturalis_837 | 0.005 |
| Subcoccinella_vigintiquatuorpunctata_619 | Subcoccinella_vigintiquatuorpunctata_620 | 0.005 |
| Subcoccinella_vigintiquatuorpunctata_619 | Subcoccinella_vigintiquatuorpunctata_621 | 0.005 |
| Subcoccinella_vigintiquatuorpunctata_619 | Subcoccinella_vigintiquatuorpunctata_628 | 0.005 |
| Subcoccinella_vigintiquatuorpunctata_619 | Subcoccinella_vigintiquatuorpunctata_629 | 0.005 |
| Subcoccinella_vigintiquatuorpunctata_619 | Subcoccinella_vigintiquatuorpunctata_631 | 0.005 |
| Stethorus_punctillum_855 | Stethorus_punctillum_859 | 0.006 |
| Subcoccinella_vigintiquatuorpunctata_622 | Subcoccinella_vigintiquatuorpunctata_623 | 0.006 |
| Tytthaspis_sedecimpunctata_580 | Tytthaspis_sedecimpunctata_583 | 0.006 |
| Tytthaspis_sedecimpunctata_580 | Tytthaspis_sedecimpunctata_585 | 0.006 |
| Tytthaspis_sedecimpunctata_586 | Tytthaspis_sedecimpunctata_589 | 0.006 |
| Vibidia_duodecimguttata_597 | Vibidia_duodecimguttata_598 | 0.006 |

Supplementary Table S4 (continued)

| | | |
|---|---|---|
| Vibidia_duodecimguttata_598 | Vibidia_duodecimguttata_600 | 0.006 |
| Stethorus_punctillum_850 | Stethorus_punctillum_854 | 0.006 |
| Stethorus_punctillum_850 | Stethorus_punctillum_856 | 0.006 |
| Subcoccinella_vigintiquatuorpunctata_624 | Subcoccinella_vigintiquatuorpunctata_625 | 0.006 |
| Subcoccinella_vigintiquatuorpunctata_622 | Subcoccinella_vigintiquatuorpunctata_626 | 0.006 |
| Tytthaspis_sedecimpunctata_586 | Tytthaspis_sedecimpunctata_587 | 0.006 |
| Tytthaspis_sedecimpunctata_583 | Tytthaspis_sedecimpunctata_592 | 0.006 |
| Subcoccinella_vigintiquatuorpunctata_619 | Subcoccinella_vigintiquatuorpunctata_623 | 0.006 |
| Subcoccinella_vigintiquatuorpunctata_619 | Subcoccinella_vigintiquatuorpunctata_626 | 0.006 |
| Stethorus_punctillum_851 | Stethorus_punctillum_855 | 0.008 |
| Stethorus_punctillum_855 | Stethorus_punctillum_858 | 0.008 |
| Stethorus_punctillum_853 | Stethorus_punctillum_859 | 0.008 |
| Tytthaspis_sedecimpunctata_577 | Tytthaspis_sedecimpunctata_584 | 0.008 |
| Tytthaspis_sedecimpunctata_578 | Tytthaspis_sedecimpunctata_584 | 0.008 |
| Tytthaspis_sedecimpunctata_579 | Tytthaspis_sedecimpunctata_584 | 0.008 |
| Tytthaspis_sedecimpunctata_581 | Tytthaspis_sedecimpunctata_584 | 0.008 |
| Tytthaspis_sedecimpunctata_582 | Tytthaspis_sedecimpunctata_584 | 0.008 |
| Tytthaspis_sedecimpunctata_584 | Tytthaspis_sedecimpunctata_590 | 0.008 |
| Tytthaspis_sedecimpunctata_577 | Tytthaspis_sedecimpunctata_591 | 0.008 |
| Tytthaspis_sedecimpunctata_578 | Tytthaspis_sedecimpunctata_591 | 0.008 |
| Tytthaspis_sedecimpunctata_579 | Tytthaspis_sedecimpunctata_591 | 0.008 |
| Tytthaspis_sedecimpunctata_581 | Tytthaspis_sedecimpunctata_591 | 0.008 |
| Tytthaspis_sedecimpunctata_582 | Tytthaspis_sedecimpunctata_591 | 0.008 |
| Tytthaspis_sedecimpunctata_590 | Tytthaspis_sedecimpunctata_591 | 0.008 |
| Tytthaspis_sedecimpunctata_584 | Tytthaspis_sedecimpunctata_594 | 0.008 |
| Tytthaspis_sedecimpunctata_591 | Tytthaspis_sedecimpunctata_594 | 0.008 |
| Tytthaspis_sedecimpunctata_584 | Tytthaspis_sedecimpunctata_595 | 0.008 |
| Tytthaspis_sedecimpunctata_591 | Tytthaspis_sedecimpunctata_595 | 0.008 |
| Stethorus_punctillum_849 | Stethorus_punctillum_850 | 0.008 |
| Stethorus_punctillum_849 | Stethorus_punctillum_854 | 0.008 |
| Stethorus_punctillum_852 | Stethorus_punctillum_854 | 0.008 |
| Stethorus_punctillum_849 | Stethorus_punctillum_856 | 0.008 |
| Stethorus_punctillum_852 | Stethorus_punctillum_856 | 0.008 |
| Stethorus_punctillum_851 | Stethorus_punctillum_859 | 0.008 |
| Stethorus_punctillum_858 | Stethorus_punctillum_859 | 0.008 |
| Subcoccinella_vigintiquatuorpunctata_620 | Subcoccinella_vigintiquatuorpunctata_624 | 0.008 |
| Subcoccinella_vigintiquatuorpunctata_621 | Subcoccinella_vigintiquatuorpunctata_624 | 0.008 |
| Subcoccinella_vigintiquatuorpunctata_620 | Subcoccinella_vigintiquatuorpunctata_625 | 0.008 |
| Subcoccinella_vigintiquatuorpunctata_621 | Subcoccinella_vigintiquatuorpunctata_625 | 0.008 |
| Subcoccinella_vigintiquatuorpunctata_624 | Subcoccinella_vigintiquatuorpunctata_627 | 0.008 |
| Subcoccinella_vigintiquatuorpunctata_625 | Subcoccinella_vigintiquatuorpunctata_627 | 0.008 |
| Subcoccinella_vigintiquatuorpunctata_624 | Subcoccinella_vigintiquatuorpunctata_628 | 0.008 |
| Subcoccinella_vigintiquatuorpunctata_625 | Subcoccinella_vigintiquatuorpunctata_628 | 0.008 |
| Subcoccinella_vigintiquatuorpunctata_624 | Subcoccinella_vigintiquatuorpunctata_629 | 0.008 |
| Subcoccinella_vigintiquatuorpunctata_625 | Subcoccinella_vigintiquatuorpunctata_629 | 0.008 |
| Subcoccinella_vigintiquatuorpunctata_624 | Subcoccinella_vigintiquatuorpunctata_630 | 0.008 |
| Subcoccinella_vigintiquatuorpunctata_625 | Subcoccinella_vigintiquatuorpunctata_630 | 0.008 |
| Subcoccinella_vigintiquatuorpunctata_624 | Subcoccinella_vigintiquatuorpunctata_631 | 0.008 |
| Subcoccinella_vigintiquatuorpunctata_625 | Subcoccinella_vigintiquatuorpunctata_631 | 0.008 |
| Tytthaspis_sedecimpunctata_583 | Tytthaspis_sedecimpunctata_593 | 0.008 |
| Tytthaspis_sedecimpunctata_585 | Tytthaspis_sedecimpunctata_593 | 0.008 |
| Vibidia_duodecimguttata_598 | Vibidia_duodecimguttata_599 | 0.008 |
| Vibidia_duodecimguttata_598 | Vibidia_duodecimguttata_602 | 0.008 |
| Subcoccinella_vigintiquatuorpunctata_619 | Subcoccinella_vigintiquatuorpunctata_627 | 0.008 |
| Subcoccinella_vigintiquatuorpunctata_619 | Subcoccinella_vigintiquatuorpunctata_630 | 0.008 |
| Scymnus_sinuanodulus_829 | Scymnus_sinuanodulus_830 | 0.008 |

| | | |
|---|---|---|
| Stethorus_punctillum_855 | Stethorus_punctillum_857 | 0.009 |
| Tytthaspis_sedecimpunctata_580 | Tytthaspis_sedecimpunctata_584 | 0.009 |
| Tytthaspis_sedecimpunctata_580 | Tytthaspis_sedecimpunctata_591 | 0.009 |
| Scymnus_suturalis_833 | Scymnus_suturalis_841 | 0.009 |
| Scymnus_suturalis_833 | Scymnus_suturalis_842 | 0.009 |
| Scymnus_suturalis_833 | Scymnus_suturalis_844 | 0.009 |
| Scymnus_suturalis_833 | Scymnus_suturalis_846 | 0.009 |
| Scymnus_suturalis_833 | Scymnus_suturalis_847 | 0.009 |
| Scymnus_suturalis_833 | Scymnus_suturalis_848 | 0.009 |
| Stethorus_punctillum_849 | Stethorus_punctillum_851 | 0.009 |
| Stethorus_punctillum_851 | Stethorus_punctillum_853 | 0.009 |
| Stethorus_punctillum_849 | Stethorus_punctillum_858 | 0.009 |
| Stethorus_punctillum_853 | Stethorus_punctillum_858 | 0.009 |
| Subcoccinella_vigintiquatuorpunctata_623 | Subcoccinella_vigintiquatuorpunctata_624 | 0.009 |
| Subcoccinella_vigintiquatuorpunctata_623 | Subcoccinella_vigintiquatuorpunctata_625 | 0.009 |
| Tytthaspis_sedecimpunctata_587 | Tytthaspis_sedecimpunctata_589 | 0.009 |
| Tytthaspis_sedecimpunctata_584 | Tytthaspis_sedecimpunctata_592 | 0.009 |
| Tytthaspis_sedecimpunctata_591 | Tytthaspis_sedecimpunctata_592 | 0.009 |
| Scymnus_suturalis_832 | Scymnus_suturalis_833 | 0.009 |
| Scymnus_suturalis_833 | Scymnus_suturalis_835 | 0.009 |
| Scymnus_suturalis_833 | Scymnus_suturalis_836 | 0.009 |
| Scymnus_suturalis_833 | Scymnus_suturalis_837 | 0.009 |
| Scymnus_suturalis_833 | Scymnus_suturalis_839 | 0.009 |
| Stethorus_punctillum_849 | Stethorus_punctillum_852 | 0.009 |
| Stethorus_punctillum_850 | Stethorus_punctillum_860 | 0.009 |
| Subcoccinella_vigintiquatuorpunctata_622 | Subcoccinella_vigintiquatuorpunctata_624 | 0.009 |
| Subcoccinella_vigintiquatuorpunctata_622 | Subcoccinella_vigintiquatuorpunctata_625 | 0.009 |
| Subcoccinella_vigintiquatuorpunctata_624 | Subcoccinella_vigintiquatuorpunctata_626 | 0.009 |
| Subcoccinella_vigintiquatuorpunctata_625 | Subcoccinella_vigintiquatuorpunctata_626 | 0.009 |
| Tytthaspis_sedecimpunctata_583 | Tytthaspis_sedecimpunctata_585 | 0.009 |
| Vibidia_duodecimguttata_598 | Vibidia_duodecimguttata_601 | 0.009 |
| Subcoccinella_vigintiquatuorpunctata_619 | Subcoccinella_vigintiquatuorpunctata_622 | 0.009 |
| Stethorus_punctillum_850 | Stethorus_punctillum_853 | 0.011 |
| Stethorus_punctillum_853 | Stethorus_punctillum_854 | 0.011 |
| Stethorus_punctillum_849 | Stethorus_punctillum_855 | 0.011 |
| Stethorus_punctillum_853 | Stethorus_punctillum_856 | 0.011 |
| Stethorus_punctillum_853 | Stethorus_punctillum_857 | 0.011 |
| Scymnus_suturalis_833 | Scymnus_suturalis_838 | 0.011 |
| Scymnus_suturalis_833 | Scymnus_suturalis_840 | 0.011 |
| Scymnus_suturalis_833 | Scymnus_suturalis_843 | 0.011 |
| Scymnus_suturalis_833 | Scymnus_suturalis_845 | 0.011 |
| Stethorus_punctillum_850 | Stethorus_punctillum_851 | 0.011 |
| Stethorus_punctillum_851 | Stethorus_punctillum_854 | 0.011 |
| Stethorus_punctillum_851 | Stethorus_punctillum_856 | 0.011 |
| Stethorus_punctillum_850 | Stethorus_punctillum_858 | 0.011 |
| Stethorus_punctillum_854 | Stethorus_punctillum_858 | 0.011 |
| Stethorus_punctillum_856 | Stethorus_punctillum_858 | 0.011 |
| Stethorus_punctillum_849 | Stethorus_punctillum_859 | 0.011 |
| Stethorus_punctillum_857 | Stethorus_punctillum_859 | 0.011 |
| Tytthaspis_sedecimpunctata_584 | Tytthaspis_sedecimpunctata_593 | 0.011 |
| Tytthaspis_sedecimpunctata_591 | Tytthaspis_sedecimpunctata_593 | 0.011 |
| Scymnus_suturalis_833 | Scymnus_suturalis_834 | 0.011 |
| Stethorus_punctillum_850 | Stethorus_punctillum_852 | 0.011 |
| Stethorus_punctillum_849 | Stethorus_punctillum_860 | 0.011 |
| Stethorus_punctillum_852 | Stethorus_punctillum_860 | 0.011 |
| Vibidia_duodecimguttata_598 | Vibidia_duodecimguttata_603 | 0.011 |

| | | |
|---|---|---|
| Stethorus_punctillum_849 | Stethorus_punctillum_853 | 0.012 |
| Stethorus_punctillum_850 | Stethorus_punctillum_855 | 0.012 |
| Stethorus_punctillum_854 | Stethorus_punctillum_855 | 0.012 |
| Stethorus_punctillum_855 | Stethorus_punctillum_856 | 0.012 |
| Stethorus_punctillum_851 | Stethorus_punctillum_852 | 0.012 |
| Stethorus_punctillum_851 | Stethorus_punctillum_857 | 0.012 |
| Stethorus_punctillum_852 | Stethorus_punctillum_858 | 0.012 |
| Stethorus_punctillum_857 | Stethorus_punctillum_858 | 0.012 |
| Stethorus_punctillum_850 | Stethorus_punctillum_859 | 0.012 |
| Stethorus_punctillum_854 | Stethorus_punctillum_859 | 0.012 |
| Stethorus_punctillum_856 | Stethorus_punctillum_859 | 0.012 |
| Tytthaspis_sedecimpunctata_583 | Tytthaspis_sedecimpunctata_584 | 0.012 |
| Tytthaspis_sedecimpunctata_584 | Tytthaspis_sedecimpunctata_585 | 0.012 |
| Tytthaspis_sedecimpunctata_583 | Tytthaspis_sedecimpunctata_591 | 0.012 |
| Tytthaspis_sedecimpunctata_585 | Tytthaspis_sedecimpunctata_591 | 0.012 |
| Vibidia_duodecimguttata_597 | Vibidia_duodecimguttata_599 | 0.012 |
| Vibidia_duodecimguttata_599 | Vibidia_duodecimguttata_600 | 0.012 |
| Vibidia_duodecimguttata_597 | Vibidia_duodecimguttata_602 | 0.012 |
| Vibidia_duodecimguttata_600 | Vibidia_duodecimguttata_602 | 0.012 |
| Vibidia_duodecimguttata_597 | Vibidia_duodecimguttata_603 | 0.012 |
| Vibidia_duodecimguttata_600 | Vibidia_duodecimguttata_603 | 0.012 |
| Scymnus_sinuanodulus_830 | Scymnus_sinuanodulus_831 | 0.012 |
| Vibidia_duodecimguttata_596 | Vibidia_duodecimguttata_599 | 0.012 |
| Vibidia_duodecimguttata_596 | Vibidia_duodecimguttata_602 | 0.012 |
| Vibidia_duodecimguttata_596 | Vibidia_duodecimguttata_603 | 0.012 |
| Subcoccinella_vigintiquatuorpunctata_619 | Subcoccinella_vigintiquatuorpunctata_624 | 0.012 |
| Subcoccinella_vigintiquatuorpunctata_619 | Subcoccinella_vigintiquatuorpunctata_625 | 0.012 |
| Stethorus_punctillum_852 | Stethorus_punctillum_855 | 0.014 |
| Stethorus_punctillum_853 | Stethorus_punctillum_860 | 0.014 |
| Stethorus_punctillum_852 | Stethorus_punctillum_859 | 0.014 |
| Stethorus_punctillum_851 | Stethorus_punctillum_860 | 0.014 |
| Stethorus_punctillum_858 | Stethorus_punctillum_860 | 0.014 |
| Vibidia_duodecimguttata_597 | Vibidia_duodecimguttata_601 | 0.014 |
| Vibidia_duodecimguttata_600 | Vibidia_duodecimguttata_601 | 0.014 |
| Scymnus_sinuanodulus_828 | Scymnus_sinuanodulus_830 | 0.014 |
| Vibidia_duodecimguttata_596 | Vibidia_duodecimguttata_601 | 0.014 |
| Stethorus_punctillum_852 | Stethorus_punctillum_853 | 0.015 |
| Stethorus_punctillum_849 | Stethorus_punctillum_857 | 0.015 |
| Stethorus_punctillum_852 | Stethorus_punctillum_857 | 0.015 |
| Stethorus_punctillum_855 | Stethorus_punctillum_860 | 0.015 |
| Tytthaspis_sedecimpunctata_584 | Tytthaspis_sedecimpunctata_591 | 0.015 |
| Stethorus_punctillum_859 | Stethorus_punctillum_860 | 0.015 |
| Stethorus_punctillum_850 | Stethorus_punctillum_857 | 0.017 |
| Stethorus_punctillum_854 | Stethorus_punctillum_857 | 0.017 |
| Stethorus_punctillum_856 | Stethorus_punctillum_857 | 0.017 |
| Tytthaspis_sedecimpunctata_585 | Tytthaspis_sedecimpunctata_588 | 0.017 |
| Tytthaspis_sedecimpunctata_588 | Tytthaspis_sedecimpunctata_592 | 0.017 |
| Tytthaspis_sedecimpunctata_585 | Tytthaspis_sedecimpunctata_586 | 0.019 |
| Tytthaspis_sedecimpunctata_577 | Tytthaspis_sedecimpunctata_588 | 0.019 |
| Tytthaspis_sedecimpunctata_578 | Tytthaspis_sedecimpunctata_588 | 0.019 |
| Tytthaspis_sedecimpunctata_579 | Tytthaspis_sedecimpunctata_588 | 0.019 |
| Tytthaspis_sedecimpunctata_581 | Tytthaspis_sedecimpunctata_588 | 0.019 |
| Tytthaspis_sedecimpunctata_582 | Tytthaspis_sedecimpunctata_588 | 0.019 |
| Tytthaspis_sedecimpunctata_588 | Tytthaspis_sedecimpunctata_590 | 0.019 |
| Tytthaspis_sedecimpunctata_586 | Tytthaspis_sedecimpunctata_592 | 0.019 |
| Tytthaspis_sedecimpunctata_588 | Tytthaspis_sedecimpunctata_594 | 0.019 |

| | | |
|---|---|---|
| Tytthaspis_sedecimpunctata_588 | Tytthaspis_sedecimpunctata_595 | 0.019 |
| Stethorus_punctillum_857 | Stethorus_punctillum_860 | 0.020 |
| Tytthaspis_sedecimpunctata_580 | Tytthaspis_sedecimpunctata_588 | 0.020 |
| Tytthaspis_sedecimpunctata_585 | Tytthaspis_sedecimpunctata_589 | 0.020 |
| Tytthaspis_sedecimpunctata_589 | Tytthaspis_sedecimpunctata_592 | 0.020 |
| Tytthaspis_sedecimpunctata_577 | Tytthaspis_sedecimpunctata_586 | 0.020 |
| Tytthaspis_sedecimpunctata_578 | Tytthaspis_sedecimpunctata_586 | 0.020 |
| Tytthaspis_sedecimpunctata_579 | Tytthaspis_sedecimpunctata_586 | 0.020 |
| Tytthaspis_sedecimpunctata_581 | Tytthaspis_sedecimpunctata_586 | 0.020 |
| Tytthaspis_sedecimpunctata_582 | Tytthaspis_sedecimpunctata_586 | 0.020 |
| Tytthaspis_sedecimpunctata_583 | Tytthaspis_sedecimpunctata_588 | 0.020 |
| Tytthaspis_sedecimpunctata_586 | Tytthaspis_sedecimpunctata_590 | 0.020 |
| Tytthaspis_sedecimpunctata_586 | Tytthaspis_sedecimpunctata_594 | 0.020 |
| Tytthaspis_sedecimpunctata_586 | Tytthaspis_sedecimpunctata_595 | 0.020 |
| Tytthaspis_sedecimpunctata_580 | Tytthaspis_sedecimpunctata_586 | 0.022 |
| Tytthaspis_sedecimpunctata_577 | Tytthaspis_sedecimpunctata_589 | 0.022 |
| Tytthaspis_sedecimpunctata_578 | Tytthaspis_sedecimpunctata_589 | 0.022 |
| Tytthaspis_sedecimpunctata_579 | Tytthaspis_sedecimpunctata_589 | 0.022 |
| Tytthaspis_sedecimpunctata_581 | Tytthaspis_sedecimpunctata_589 | 0.022 |
| Tytthaspis_sedecimpunctata_582 | Tytthaspis_sedecimpunctata_589 | 0.022 |
| Tytthaspis_sedecimpunctata_589 | Tytthaspis_sedecimpunctata_590 | 0.022 |
| Tytthaspis_sedecimpunctata_589 | Tytthaspis_sedecimpunctata_594 | 0.022 |
| Tytthaspis_sedecimpunctata_589 | Tytthaspis_sedecimpunctata_595 | 0.022 |
| Tytthaspis_sedecimpunctata_583 | Tytthaspis_sedecimpunctata_586 | 0.022 |
| Tytthaspis_sedecimpunctata_585 | Tytthaspis_sedecimpunctata_587 | 0.022 |
| Tytthaspis_sedecimpunctata_587 | Tytthaspis_sedecimpunctata_592 | 0.022 |
| Tytthaspis_sedecimpunctata_588 | Tytthaspis_sedecimpunctata_593 | 0.022 |
| Tytthaspis_sedecimpunctata_580 | Tytthaspis_sedecimpunctata_589 | 0.023 |
| Tytthaspis_sedecimpunctata_583 | Tytthaspis_sedecimpunctata_589 | 0.023 |
| Tytthaspis_sedecimpunctata_577 | Tytthaspis_sedecimpunctata_587 | 0.023 |
| Tytthaspis_sedecimpunctata_578 | Tytthaspis_sedecimpunctata_587 | 0.023 |
| Tytthaspis_sedecimpunctata_579 | Tytthaspis_sedecimpunctata_587 | 0.023 |
| Tytthaspis_sedecimpunctata_581 | Tytthaspis_sedecimpunctata_587 | 0.023 |
| Tytthaspis_sedecimpunctata_582 | Tytthaspis_sedecimpunctata_587 | 0.023 |
| Tytthaspis_sedecimpunctata_587 | Tytthaspis_sedecimpunctata_590 | 0.023 |
| Tytthaspis_sedecimpunctata_586 | Tytthaspis_sedecimpunctata_593 | 0.023 |
| Tytthaspis_sedecimpunctata_587 | Tytthaspis_sedecimpunctata_594 | 0.023 |
| Tytthaspis_sedecimpunctata_587 | Tytthaspis_sedecimpunctata_595 | 0.023 |
| Tytthaspis_sedecimpunctata_580 | Tytthaspis_sedecimpunctata_587 | 0.025 |
| Tytthaspis_sedecimpunctata_589 | Tytthaspis_sedecimpunctata_593 | 0.025 |
| Tytthaspis_sedecimpunctata_583 | Tytthaspis_sedecimpunctata_587 | 0.025 |
| Tytthaspis_sedecimpunctata_584 | Tytthaspis_sedecimpunctata_588 | 0.026 |
| Tytthaspis_sedecimpunctata_588 | Tytthaspis_sedecimpunctata_591 | 0.026 |
| Tytthaspis_sedecimpunctata_587 | Tytthaspis_sedecimpunctata_593 | 0.026 |
| Tytthaspis_sedecimpunctata_584 | Tytthaspis_sedecimpunctata_586 | 0.028 |
| Tytthaspis_sedecimpunctata_586 | Tytthaspis_sedecimpunctata_591 | 0.028 |
| Tytthaspis_sedecimpunctata_584 | Tytthaspis_sedecimpunctata_589 | 0.030 |
| Tytthaspis_sedecimpunctata_589 | Tytthaspis_sedecimpunctata_591 | 0.030 |
| Tytthaspis_sedecimpunctata_584 | Tytthaspis_sedecimpunctata_587 | 0.031 |
| Tytthaspis_sedecimpunctata_587 | Tytthaspis_sedecimpunctata_591 | 0.031 |
| Scymnus_ningshanensis_789 | Scymnus_sinuanodulus_830 | 0.100 |
| Scymnus_ningshanensis_790 | Scymnus_sinuanodulus_830 | 0.100 |
| Scymnus_ningshanensis_788 | Scymnus_sinuanodulus_830 | 0.102 |
| Scymnus_ningshanensis_791 | Scymnus_sinuanodulus_830 | 0.102 |
| Scymnus_ningshanensis_792 | Scymnus_sinuanodulus_830 | 0.102 |
| Scymnus_ningshanensis_793 | Scymnus_sinuanodulus_830 | 0.102 |

| | | |
|---|---|---|
| Scymnus_ningshanensis_794 | Scymnus_sinuanodulus_830 | 0.102 |
| Scymnus_ningshanensis_795 | Scymnus_sinuanodulus_830 | 0.102 |
| Scymnus_ningshanensis_789 | Scymnus_sinuanodulus_829 | 0.102 |
| Scymnus_ningshanensis_790 | Scymnus_sinuanodulus_829 | 0.102 |
| Scymnus_camptodromus_715 | Scymnus_sinuanodulus_831 | 0.103 |
| Scymnus_camptodromus_715 | Scymnus_sinuanodulus_829 | 0.103 |
| Scymnus_ningshanensis_789 | Scymnus_sinuanodulus_831 | 0.104 |
| Scymnus_ningshanensis_790 | Scymnus_sinuanodulus_831 | 0.104 |
| Scymnus_ningshanensis_788 | Scymnus_sinuanodulus_829 | 0.104 |
| Scymnus_ningshanensis_791 | Scymnus_sinuanodulus_829 | 0.104 |
| Scymnus_ningshanensis_792 | Scymnus_sinuanodulus_829 | 0.104 |
| Scymnus_ningshanensis_793 | Scymnus_sinuanodulus_829 | 0.104 |
| Scymnus_ningshanensis_794 | Scymnus_sinuanodulus_829 | 0.104 |
| Scymnus_ningshanensis_795 | Scymnus_sinuanodulus_829 | 0.104 |
| Scymnus_camptodromus_717 | Scymnus_sinuanodulus_831 | 0.104 |
| Scymnus_camptodromus_717 | Scymnus_sinuanodulus_829 | 0.104 |
| Scymnus_camptodromus_715 | Scymnus_sinuanodulus_830 | 0.104 |
| Scymnus_ningshanensis_788 | Scymnus_sinuanodulus_831 | 0.105 |
| Scymnus_ningshanensis_791 | Scymnus_sinuanodulus_831 | 0.105 |
| Scymnus_ningshanensis_792 | Scymnus_sinuanodulus_831 | 0.105 |
| Scymnus_ningshanensis_793 | Scymnus_sinuanodulus_831 | 0.105 |
| Scymnus_ningshanensis_794 | Scymnus_sinuanodulus_831 | 0.105 |
| Scymnus_ningshanensis_795 | Scymnus_sinuanodulus_831 | 0.105 |
| Scymnus_camptodromus_717 | Scymnus_sinuanodulus_830 | 0.106 |
| Scymnus_camptodromus_711 | Scymnus_sinuanodulus_831 | 0.106 |
| Scymnus_camptodromus_706 | Scymnus_sinuanodulus_831 | 0.106 |
| Scymnus_camptodromus_711 | Scymnus_sinuanodulus_829 | 0.106 |
| Scymnus_camptodromus_706 | Scymnus_sinuanodulus_829 | 0.106 |
| Scymnus_interruptus_762 | Scymnus_rubromaculatus_822 | 0.107 |
| Scymnus_interruptus_762 | Scymnus_rubromaculatus_823 | 0.107 |
| Scymnus_interruptus_762 | Scymnus_rubromaculatus_824 | 0.107 |
| Scymnus_interruptus_762 | Scymnus_rubromaculatus_825 | 0.107 |
| Scymnus_interruptus_762 | Scymnus_rubromaculatus_826 | 0.107 |
| Scymnus_interruptus_762 | Scymnus_rubromaculatus_827 | 0.107 |
| Scymnus_camptodromus_711 | Scymnus_sinuanodulus_830 | 0.107 |
| Scymnus_camptodromus_706 | Scymnus_sinuanodulus_830 | 0.108 |
| Scymnus_camptodromus_715 | Scymnus_sinuanodulus_828 | 0.108 |
| Scymnus_ningshanensis_789 | Scymnus_sinuanodulus_828 | 0.109 |
| Scymnus_ningshanensis_790 | Scymnus_sinuanodulus_828 | 0.109 |
| Scymnus_camptodromus_712 | Scymnus_sinuanodulus_831 | 0.109 |
| Scymnus_camptodromus_712 | Scymnus_sinuanodulus_829 | 0.109 |
| Scymnus_camptodromus_717 | Scymnus_sinuanodulus_828 | 0.109 |
| Scymnus_interruptus_760 | Scymnus_rubromaculatus_822 | 0.110 |
| Scymnus_interruptus_761 | Scymnus_rubromaculatus_822 | 0.110 |
| Scymnus_interruptus_763 | Scymnus_rubromaculatus_822 | 0.110 |
| Scymnus_interruptus_764 | Scymnus_rubromaculatus_822 | 0.110 |
| Scymnus_interruptus_765 | Scymnus_rubromaculatus_822 | 0.110 |
| Scymnus_interruptus_766 | Scymnus_rubromaculatus_822 | 0.110 |
| Scymnus_interruptus_768 | Scymnus_rubromaculatus_822 | 0.110 |
| Scymnus_interruptus_760 | Scymnus_rubromaculatus_823 | 0.110 |
| Scymnus_interruptus_761 | Scymnus_rubromaculatus_823 | 0.110 |
| Scymnus_interruptus_763 | Scymnus_rubromaculatus_823 | 0.110 |
| Scymnus_interruptus_764 | Scymnus_rubromaculatus_823 | 0.110 |
| Scymnus_interruptus_765 | Scymnus_rubromaculatus_823 | 0.110 |
| Scymnus_interruptus_766 | Scymnus_rubromaculatus_823 | 0.110 |
| Scymnus_interruptus_768 | Scymnus_rubromaculatus_823 | 0.110 |

| | | |
|---|---|---|
| Scymnus_interruptus_760 | Scymnus_rubromaculatus_824 | 0.110 |
| Scymnus_interruptus_761 | Scymnus_rubromaculatus_824 | 0.110 |
| Scymnus_interruptus_763 | Scymnus_rubromaculatus_824 | 0.110 |
| Scymnus_interruptus_764 | Scymnus_rubromaculatus_824 | 0.110 |
| Scymnus_interruptus_765 | Scymnus_rubromaculatus_824 | 0.110 |
| Scymnus_interruptus_766 | Scymnus_rubromaculatus_824 | 0.110 |
| Scymnus_interruptus_768 | Scymnus_rubromaculatus_824 | 0.110 |
| Scymnus_interruptus_760 | Scymnus_rubromaculatus_825 | 0.110 |
| Scymnus_interruptus_761 | Scymnus_rubromaculatus_825 | 0.110 |
| Scymnus_interruptus_763 | Scymnus_rubromaculatus_825 | 0.110 |
| Scymnus_interruptus_764 | Scymnus_rubromaculatus_825 | 0.110 |
| Scymnus_interruptus_765 | Scymnus_rubromaculatus_825 | 0.110 |
| Scymnus_interruptus_766 | Scymnus_rubromaculatus_825 | 0.110 |
| Scymnus_interruptus_768 | Scymnus_rubromaculatus_825 | 0.110 |
| Scymnus_interruptus_760 | Scymnus_rubromaculatus_826 | 0.110 |
| Scymnus_interruptus_761 | Scymnus_rubromaculatus_826 | 0.110 |
| Scymnus_interruptus_763 | Scymnus_rubromaculatus_826 | 0.110 |
| Scymnus_interruptus_764 | Scymnus_rubromaculatus_826 | 0.110 |
| Scymnus_interruptus_765 | Scymnus_rubromaculatus_826 | 0.110 |
| Scymnus_interruptus_766 | Scymnus_rubromaculatus_826 | 0.110 |
| Scymnus_interruptus_768 | Scymnus_rubromaculatus_826 | 0.110 |
| Scymnus_interruptus_760 | Scymnus_rubromaculatus_827 | 0.110 |
| Scymnus_interruptus_761 | Scymnus_rubromaculatus_827 | 0.110 |
| Scymnus_interruptus_763 | Scymnus_rubromaculatus_827 | 0.110 |
| Scymnus_interruptus_764 | Scymnus_rubromaculatus_827 | 0.110 |
| Scymnus_interruptus_765 | Scymnus_rubromaculatus_827 | 0.110 |
| Scymnus_interruptus_766 | Scymnus_rubromaculatus_827 | 0.110 |
| Scymnus_interruptus_768 | Scymnus_rubromaculatus_827 | 0.110 |
| Scymnus_ningshanensis_788 | Scymnus_sinuanodulus_828 | 0.111 |
| Scymnus_ningshanensis_791 | Scymnus_sinuanodulus_828 | 0.111 |
| Scymnus_ningshanensis_792 | Scymnus_sinuanodulus_828 | 0.111 |
| Scymnus_ningshanensis_793 | Scymnus_sinuanodulus_828 | 0.111 |
| Scymnus_ningshanensis_794 | Scymnus_sinuanodulus_828 | 0.111 |
| Scymnus_ningshanensis_795 | Scymnus_sinuanodulus_828 | 0.111 |
| Scymnus_camptodromus_712 | Scymnus_sinuanodulus_830 | 0.111 |
| Scymnus_camptodromus_707 | Scymnus_sinuanodulus_831 | 0.111 |
| Scymnus_camptodromus_708 | Scymnus_sinuanodulus_831 | 0.111 |
| Scymnus_camptodromus_709 | Scymnus_sinuanodulus_831 | 0.111 |
| Scymnus_camptodromus_710 | Scymnus_sinuanodulus_831 | 0.111 |
| Scymnus_camptodromus_719 | Scymnus_sinuanodulus_831 | 0.111 |
| Scymnus_camptodromus_711 | Scymnus_sinuanodulus_828 | 0.111 |
| Scymnus_camptodromus_706 | Scymnus_sinuanodulus_828 | 0.111 |
| Scymnus_camptodromus_707 | Scymnus_sinuanodulus_829 | 0.111 |
| Scymnus_camptodromus_708 | Scymnus_sinuanodulus_829 | 0.111 |
| Scymnus_camptodromus_709 | Scymnus_sinuanodulus_829 | 0.111 |
| Scymnus_camptodromus_710 | Scymnus_sinuanodulus_829 | 0.111 |
| Scymnus_camptodromus_719 | Scymnus_sinuanodulus_829 | 0.111 |
| Scymnus_interruptus_759 | Scymnus_rubromaculatus_822 | 0.112 |
| Scymnus_interruptus_767 | Scymnus_rubromaculatus_822 | 0.112 |
| Scymnus_interruptus_769 | Scymnus_rubromaculatus_822 | 0.112 |
| Scymnus_interruptus_759 | Scymnus_rubromaculatus_823 | 0.112 |
| Scymnus_interruptus_767 | Scymnus_rubromaculatus_823 | 0.112 |
| Scymnus_interruptus_769 | Scymnus_rubromaculatus_823 | 0.112 |
| Scymnus_interruptus_759 | Scymnus_rubromaculatus_824 | 0.112 |
| Scymnus_interruptus_767 | Scymnus_rubromaculatus_824 | 0.112 |
| Scymnus_interruptus_769 | Scymnus_rubromaculatus_824 | 0.112 |

| | | |
|---|---|---|
| Scymnus_interruptus_759 | Scymnus_rubromaculatus_825 | 0.112 |
| Scymnus_interruptus_767 | Scymnus_rubromaculatus_825 | 0.112 |
| Scymnus_interruptus_769 | Scymnus_rubromaculatus_825 | 0.112 |
| Scymnus_interruptus_759 | Scymnus_rubromaculatus_826 | 0.112 |
| Scymnus_interruptus_767 | Scymnus_rubromaculatus_826 | 0.112 |
| Scymnus_interruptus_769 | Scymnus_rubromaculatus_826 | 0.112 |
| Scymnus_interruptus_759 | Scymnus_rubromaculatus_827 | 0.112 |
| Scymnus_interruptus_767 | Scymnus_rubromaculatus_827 | 0.112 |
| Scymnus_interruptus_769 | Scymnus_rubromaculatus_827 | 0.112 |
| Scymnus_camptodromus_707 | Scymnus_sinuanodulus_830 | 0.113 |
| Scymnus_camptodromus_708 | Scymnus_sinuanodulus_830 | 0.113 |
| Scymnus_camptodromus_709 | Scymnus_sinuanodulus_830 | 0.113 |
| Scymnus_camptodromus_710 | Scymnus_sinuanodulus_830 | 0.113 |
| Scymnus_camptodromus_719 | Scymnus_sinuanodulus_830 | 0.113 |
| Scymnus_camptodromus_718 | Scymnus_sinuanodulus_831 | 0.113 |
| Scymnus_camptodromus_718 | Scymnus_sinuanodulus_829 | 0.113 |
| Scymnus_camptodromus_712 | Scymnus_sinuanodulus_828 | 0.114 |
| Scymnus_camptodromus_705 | Scymnus_sinuanodulus_831 | 0.114 |
| Scymnus_camptodromus_705 | Scymnus_sinuanodulus_829 | 0.115 |
| Scymnus_camptodromus_718 | Scymnus_sinuanodulus_828 | 0.116 |
| Scymnus_camptodromus_705 | Scymnus_sinuanodulus_830 | 0.116 |
| Scymnus_camptodromus_714 | Scymnus_sinuanodulus_831 | 0.116 |
| Scymnus_camptodromus_707 | Scymnus_sinuanodulus_828 | 0.116 |
| Scymnus_camptodromus_708 | Scymnus_sinuanodulus_828 | 0.116 |
| Scymnus_camptodromus_709 | Scymnus_sinuanodulus_828 | 0.116 |
| Scymnus_camptodromus_710 | Scymnus_sinuanodulus_828 | 0.116 |
| Scymnus_camptodromus_719 | Scymnus_sinuanodulus_828 | 0.116 |
| Scymnus_camptodromus_718 | Scymnus_sinuanodulus_830 | 0.116 |
| Scymnus_camptodromus_713 | Scymnus_sinuanodulus_831 | 0.116 |
| Scymnus_camptodromus_716 | Scymnus_sinuanodulus_831 | 0.116 |
| Scymnus_camptodromus_714 | Scymnus_sinuanodulus_829 | 0.116 |
| Scymnus_camptodromus_713 | Scymnus_sinuanodulus_829 | 0.116 |
| Scymnus_camptodromus_716 | Scymnus_sinuanodulus_829 | 0.116 |
| Scymnus_camptodromus_714 | Scymnus_sinuanodulus_830 | 0.118 |
| Scymnus_camptodromus_713 | Scymnus_sinuanodulus_830 | 0.118 |
| Scymnus_camptodromus_716 | Scymnus_sinuanodulus_830 | 0.118 |
| Scymnus_camptodromus_704 | Scymnus_sinuanodulus_831 | 0.118 |
| Scymnus_camptodromus_704 | Scymnus_sinuanodulus_829 | 0.118 |
| Scymnus_camptodromus_705 | Scymnus_sinuanodulus_828 | 0.120 |
| Scymnus_camptodromus_704 | Scymnus_sinuanodulus_830 | 0.120 |
| Scymnus_camptodromus_715 | Scymnus_suturalis_843 | 0.120 |
| Scymnus_camptodromus_714 | Scymnus_sinuanodulus_828 | 0.121 |
| Scymnus_camptodromus_715 | Scymnus_suturalis_833 | 0.121 |
| Scymnus_camptodromus_713 | Scymnus_sinuanodulus_828 | 0.121 |
| Scymnus_camptodromus_716 | Scymnus_sinuanodulus_828 | 0.121 |
| Scymnus_camptodromus_715 | Scymnus_suturalis_841 | 0.122 |
| Scymnus_camptodromus_715 | Scymnus_suturalis_842 | 0.122 |
| Scymnus_camptodromus_715 | Scymnus_suturalis_844 | 0.122 |
| Scymnus_camptodromus_715 | Scymnus_suturalis_845 | 0.122 |
| Scymnus_camptodromus_715 | Scymnus_suturalis_846 | 0.122 |
| Scymnus_camptodromus_715 | Scymnus_suturalis_847 | 0.122 |
| Scymnus_camptodromus_715 | Scymnus_suturalis_848 | 0.122 |
| Scymnus_camptodromus_715 | Scymnus_suturalis_832 | 0.122 |
| Scymnus_camptodromus_715 | Scymnus_suturalis_835 | 0.122 |
| Scymnus_camptodromus_715 | Scymnus_suturalis_836 | 0.122 |
| Scymnus_camptodromus_715 | Scymnus_suturalis_837 | 0.122 |

| | | |
|---|---|---|
| Scymnus_camptodromus_715 | Scymnus_suturalis_839 | 0.122 |
| Anisosticta_bitriangularis_150 | Tytthaspis_sedecimpunctata_592 | 0.122 |
| Anisosticta_bitriangularis_150 | Tytthaspis_sedecimpunctata_580 | 0.122 |
| Scymnus_camptodromus_711 | Scymnus_suturalis_843 | 0.123 |
| Scymnus_camptodromus_717 | Scymnus_suturalis_843 | 0.123 |
| Scymnus_camptodromus_717 | Scymnus_suturalis_845 | 0.123 |
| Scymnus_camptodromus_706 | Scymnus_suturalis_843 | 0.123 |
| Scymnus_camptodromus_706 | Scymnus_suturalis_845 | 0.123 |
| Scymnus_camptodromus_704 | Scymnus_sinuanodulus_828 | 0.123 |
| Scymnus_camptodromus_715 | Scymnus_suturalis_838 | 0.123 |
| Scymnus_camptodromus_715 | Scymnus_suturalis_840 | 0.123 |
| Scymnus_camptodromus_715 | Scymnus_suturalis_834 | 0.123 |
| Anisosticta_bitriangularis_150 | Tytthaspis_sedecimpunctata_577 | 0.123 |
| Anisosticta_bitriangularis_150 | Tytthaspis_sedecimpunctata_578 | 0.123 |
| Anisosticta_bitriangularis_150 | Tytthaspis_sedecimpunctata_579 | 0.123 |
| Anisosticta_bitriangularis_150 | Tytthaspis_sedecimpunctata_581 | 0.123 |
| Anisosticta_bitriangularis_150 | Tytthaspis_sedecimpunctata_582 | 0.123 |
| Anisosticta_bitriangularis_150 | Tytthaspis_sedecimpunctata_590 | 0.123 |
| Anisosticta_bitriangularis_150 | Tytthaspis_sedecimpunctata_593 | 0.123 |
| Anisosticta_bitriangularis_150 | Tytthaspis_sedecimpunctata_594 | 0.123 |
| Anisosticta_bitriangularis_150 | Tytthaspis_sedecimpunctata_595 | 0.123 |
| Scymnus_camptodromus_711 | Scymnus_suturalis_833 | 0.124 |
| Scymnus_camptodromus_711 | Scymnus_suturalis_841 | 0.125 |
| Scymnus_camptodromus_711 | Scymnus_suturalis_842 | 0.125 |
| Scymnus_camptodromus_711 | Scymnus_suturalis_844 | 0.125 |
| Scymnus_camptodromus_711 | Scymnus_suturalis_845 | 0.125 |
| Scymnus_camptodromus_711 | Scymnus_suturalis_846 | 0.125 |
| Scymnus_camptodromus_711 | Scymnus_suturalis_847 | 0.125 |
| Scymnus_camptodromus_711 | Scymnus_suturalis_848 | 0.125 |
| Scymnus_camptodromus_711 | Scymnus_suturalis_832 | 0.125 |
| Scymnus_camptodromus_717 | Scymnus_suturalis_833 | 0.125 |
| Scymnus_camptodromus_711 | Scymnus_suturalis_835 | 0.125 |
| Scymnus_camptodromus_711 | Scymnus_suturalis_836 | 0.125 |
| Scymnus_camptodromus_711 | Scymnus_suturalis_837 | 0.125 |
| Scymnus_camptodromus_711 | Scymnus_suturalis_839 | 0.125 |
| Scymnus_camptodromus_717 | Scymnus_suturalis_841 | 0.125 |
| Scymnus_camptodromus_717 | Scymnus_suturalis_842 | 0.125 |
| Scymnus_camptodromus_717 | Scymnus_suturalis_844 | 0.125 |
| Scymnus_camptodromus_717 | Scymnus_suturalis_846 | 0.125 |
| Scymnus_camptodromus_717 | Scymnus_suturalis_847 | 0.125 |
| Scymnus_camptodromus_717 | Scymnus_suturalis_848 | 0.125 |
| Scymnus_camptodromus_717 | Scymnus_suturalis_832 | 0.125 |
| Scymnus_camptodromus_717 | Scymnus_suturalis_835 | 0.125 |
| Scymnus_camptodromus_717 | Scymnus_suturalis_836 | 0.125 |
| Scymnus_camptodromus_717 | Scymnus_suturalis_837 | 0.125 |
| Scymnus_camptodromus_717 | Scymnus_suturalis_839 | 0.125 |
| Scymnus_camptodromus_706 | Scymnus_suturalis_833 | 0.125 |
| Scymnus_camptodromus_706 | Scymnus_suturalis_840 | 0.125 |
| Scymnus_camptodromus_706 | Scymnus_suturalis_841 | 0.125 |
| Scymnus_camptodromus_706 | Scymnus_suturalis_842 | 0.125 |
| Scymnus_camptodromus_706 | Scymnus_suturalis_844 | 0.125 |
| Scymnus_camptodromus_706 | Scymnus_suturalis_846 | 0.125 |
| Scymnus_camptodromus_706 | Scymnus_suturalis_847 | 0.125 |
| Scymnus_camptodromus_706 | Scymnus_suturalis_848 | 0.125 |
| Scymnus_camptodromus_706 | Scymnus_suturalis_832 | 0.125 |
| Scymnus_camptodromus_706 | Scymnus_suturalis_835 | 0.125 |

| | | |
|---|---|---|
| Scymnus_camptodromus_706 | Scymnus_suturalis_836 | 0.125 |
| Scymnus_camptodromus_706 | Scymnus_suturalis_837 | 0.125 |
| Scymnus_camptodromus_706 | Scymnus_suturalis_839 | 0.125 |
| Anisosticta_bitriangularis_150 | Tytthaspis_sedecimpunctata_584 | 0.125 |
| Anisosticta_bitriangularis_150 | Tytthaspis_sedecimpunctata_583 | 0.125 |
| Anisosticta_bitriangularis_150 | Tytthaspis_sedecimpunctata_585 | 0.125 |
| Scymnus_camptodromus_707 | Scymnus_suturalis_843 | 0.126 |
| Scymnus_camptodromus_708 | Scymnus_suturalis_843 | 0.126 |
| Scymnus_camptodromus_709 | Scymnus_suturalis_843 | 0.126 |
| Scymnus_camptodromus_710 | Scymnus_suturalis_843 | 0.126 |
| Scymnus_camptodromus_719 | Scymnus_suturalis_843 | 0.126 |
| Scymnus_camptodromus_707 | Scymnus_suturalis_845 | 0.126 |
| Scymnus_camptodromus_708 | Scymnus_suturalis_845 | 0.126 |
| Scymnus_camptodromus_709 | Scymnus_suturalis_845 | 0.126 |
| Scymnus_camptodromus_710 | Scymnus_suturalis_845 | 0.126 |
| Scymnus_camptodromus_719 | Scymnus_suturalis_845 | 0.126 |
| Scymnus_camptodromus_711 | Scymnus_suturalis_838 | 0.126 |
| Scymnus_camptodromus_711 | Scymnus_suturalis_840 | 0.126 |
| Scymnus_camptodromus_711 | Scymnus_suturalis_834 | 0.126 |
| Scymnus_camptodromus_717 | Scymnus_suturalis_838 | 0.126 |
| Scymnus_camptodromus_717 | Scymnus_suturalis_840 | 0.126 |
| Scymnus_camptodromus_717 | Scymnus_suturalis_834 | 0.126 |
| Scymnus_camptodromus_706 | Scymnus_suturalis_838 | 0.127 |
| Scymnus_camptodromus_706 | Scymnus_suturalis_834 | 0.127 |
| Scymnus_camptodromus_712 | Scymnus_suturalis_843 | 0.128 |
| Scymnus_camptodromus_718 | Scymnus_suturalis_843 | 0.128 |
| Scymnus_camptodromus_718 | Scymnus_suturalis_845 | 0.128 |
| Scymnus_camptodromus_707 | Scymnus_suturalis_833 | 0.128 |
| Scymnus_camptodromus_708 | Scymnus_suturalis_833 | 0.128 |
| Scymnus_camptodromus_709 | Scymnus_suturalis_833 | 0.128 |
| Scymnus_camptodromus_710 | Scymnus_suturalis_833 | 0.128 |
| Scymnus_camptodromus_719 | Scymnus_suturalis_833 | 0.128 |
| Scymnus_camptodromus_707 | Scymnus_suturalis_840 | 0.128 |
| Scymnus_camptodromus_708 | Scymnus_suturalis_840 | 0.128 |
| Scymnus_camptodromus_709 | Scymnus_suturalis_840 | 0.128 |
| Scymnus_camptodromus_710 | Scymnus_suturalis_840 | 0.128 |
| Scymnus_camptodromus_719 | Scymnus_suturalis_840 | 0.128 |
| Scymnus_camptodromus_707 | Scymnus_suturalis_841 | 0.128 |
| Scymnus_camptodromus_708 | Scymnus_suturalis_841 | 0.128 |
| Scymnus_camptodromus_709 | Scymnus_suturalis_841 | 0.128 |
| Scymnus_camptodromus_710 | Scymnus_suturalis_841 | 0.128 |
| Scymnus_camptodromus_719 | Scymnus_suturalis_841 | 0.128 |
| Scymnus_camptodromus_707 | Scymnus_suturalis_842 | 0.128 |
| Scymnus_camptodromus_708 | Scymnus_suturalis_842 | 0.128 |
| Scymnus_camptodromus_709 | Scymnus_suturalis_842 | 0.128 |
| Scymnus_camptodromus_710 | Scymnus_suturalis_842 | 0.128 |
| Scymnus_camptodromus_719 | Scymnus_suturalis_842 | 0.128 |
| Scymnus_camptodromus_707 | Scymnus_suturalis_844 | 0.128 |
| Scymnus_camptodromus_708 | Scymnus_suturalis_844 | 0.128 |
| Scymnus_camptodromus_709 | Scymnus_suturalis_844 | 0.128 |
| Scymnus_camptodromus_710 | Scymnus_suturalis_844 | 0.128 |
| Scymnus_camptodromus_719 | Scymnus_suturalis_844 | 0.128 |
| Scymnus_camptodromus_707 | Scymnus_suturalis_846 | 0.128 |
| Scymnus_camptodromus_708 | Scymnus_suturalis_846 | 0.128 |
| Scymnus_camptodromus_709 | Scymnus_suturalis_846 | 0.128 |
| Scymnus_camptodromus_710 | Scymnus_suturalis_846 | 0.128 |

| | | |
|---|---|---|
| Scymnus_camptodromus_719 | Scymnus_suturalis_846 | 0.128 |
| Scymnus_camptodromus_707 | Scymnus_suturalis_847 | 0.128 |
| Scymnus_camptodromus_708 | Scymnus_suturalis_847 | 0.128 |
| Scymnus_camptodromus_709 | Scymnus_suturalis_847 | 0.128 |
| Scymnus_camptodromus_710 | Scymnus_suturalis_847 | 0.128 |
| Scymnus_camptodromus_719 | Scymnus_suturalis_847 | 0.128 |
| Scymnus_camptodromus_707 | Scymnus_suturalis_848 | 0.128 |
| Scymnus_camptodromus_708 | Scymnus_suturalis_848 | 0.128 |
| Scymnus_camptodromus_709 | Scymnus_suturalis_848 | 0.128 |
| Scymnus_camptodromus_710 | Scymnus_suturalis_848 | 0.128 |
| Scymnus_camptodromus_719 | Scymnus_suturalis_848 | 0.128 |
| Scymnus_camptodromus_707 | Scymnus_suturalis_832 | 0.128 |
| Scymnus_camptodromus_708 | Scymnus_suturalis_832 | 0.128 |
| Scymnus_camptodromus_709 | Scymnus_suturalis_832 | 0.128 |
| Scymnus_camptodromus_710 | Scymnus_suturalis_832 | 0.128 |
| Scymnus_camptodromus_719 | Scymnus_suturalis_832 | 0.128 |
| Scymnus_camptodromus_707 | Scymnus_suturalis_835 | 0.128 |
| Scymnus_camptodromus_708 | Scymnus_suturalis_835 | 0.128 |
| Scymnus_camptodromus_709 | Scymnus_suturalis_835 | 0.128 |
| Scymnus_camptodromus_710 | Scymnus_suturalis_835 | 0.128 |
| Scymnus_camptodromus_719 | Scymnus_suturalis_835 | 0.128 |
| Scymnus_camptodromus_707 | Scymnus_suturalis_836 | 0.128 |
| Scymnus_camptodromus_708 | Scymnus_suturalis_836 | 0.128 |
| Scymnus_camptodromus_709 | Scymnus_suturalis_836 | 0.128 |
| Scymnus_camptodromus_710 | Scymnus_suturalis_836 | 0.128 |
| Scymnus_camptodromus_719 | Scymnus_suturalis_836 | 0.128 |
| Scymnus_camptodromus_707 | Scymnus_suturalis_837 | 0.128 |
| Scymnus_camptodromus_708 | Scymnus_suturalis_837 | 0.128 |
| Scymnus_camptodromus_709 | Scymnus_suturalis_837 | 0.128 |
| Scymnus_camptodromus_710 | Scymnus_suturalis_837 | 0.128 |
| Scymnus_camptodromus_719 | Scymnus_suturalis_837 | 0.128 |
| Scymnus_camptodromus_707 | Scymnus_suturalis_839 | 0.128 |
| Scymnus_camptodromus_708 | Scymnus_suturalis_839 | 0.128 |
| Scymnus_camptodromus_709 | Scymnus_suturalis_839 | 0.128 |
| Scymnus_camptodromus_710 | Scymnus_suturalis_839 | 0.128 |
| Scymnus_camptodromus_719 | Scymnus_suturalis_839 | 0.128 |
| Anisosticta_bitriangularis_155 | Tytthaspis_sedecimpunctata_592 | 0.128 |
| Anisosticta_bitriangularis_155 | Tytthaspis_sedecimpunctata_580 | 0.128 |
| Anisosticta_bitriangularis_148 | Tytthaspis_sedecimpunctata_592 | 0.128 |
| Anisosticta_bitriangularis_148 | Tytthaspis_sedecimpunctata_580 | 0.128 |
| Scymnus_camptodromus_712 | Scymnus_suturalis_833 | 0.130 |
| Scymnus_camptodromus_718 | Scymnus_suturalis_833 | 0.130 |
| Scymnus_camptodromus_712 | Scymnus_suturalis_841 | 0.130 |
| Scymnus_camptodromus_718 | Scymnus_suturalis_841 | 0.130 |
| Scymnus_camptodromus_712 | Scymnus_suturalis_842 | 0.130 |
| Scymnus_camptodromus_718 | Scymnus_suturalis_842 | 0.130 |
| Scymnus_camptodromus_712 | Scymnus_suturalis_844 | 0.130 |
| Scymnus_camptodromus_718 | Scymnus_suturalis_844 | 0.130 |
| Scymnus_camptodromus_712 | Scymnus_suturalis_846 | 0.130 |
| Scymnus_camptodromus_718 | Scymnus_suturalis_846 | 0.130 |
| Scymnus_camptodromus_712 | Scymnus_suturalis_847 | 0.130 |
| Scymnus_camptodromus_718 | Scymnus_suturalis_847 | 0.130 |
| Scymnus_camptodromus_712 | Scymnus_suturalis_848 | 0.130 |
| Scymnus_camptodromus_718 | Scymnus_suturalis_848 | 0.130 |
| Scymnus_camptodromus_712 | Scymnus_suturalis_832 | 0.130 |
| Scymnus_camptodromus_718 | Scymnus_suturalis_832 | 0.130 |

| | | |
|---|---|---|
| Scymnus_camptodromus_712 | Scymnus_suturalis_835 | 0.130 |
| Scymnus_camptodromus_718 | Scymnus_suturalis_835 | 0.130 |
| Scymnus_camptodromus_712 | Scymnus_suturalis_836 | 0.130 |
| Scymnus_camptodromus_718 | Scymnus_suturalis_836 | 0.130 |
| Scymnus_camptodromus_712 | Scymnus_suturalis_837 | 0.130 |
| Scymnus_camptodromus_718 | Scymnus_suturalis_837 | 0.130 |
| Scymnus_camptodromus_707 | Scymnus_suturalis_838 | 0.130 |
| Scymnus_camptodromus_708 | Scymnus_suturalis_838 | 0.130 |
| Scymnus_camptodromus_709 | Scymnus_suturalis_838 | 0.130 |
| Scymnus_camptodromus_710 | Scymnus_suturalis_838 | 0.130 |
| Scymnus_camptodromus_719 | Scymnus_suturalis_838 | 0.130 |
| Scymnus_camptodromus_712 | Scymnus_suturalis_839 | 0.130 |
| Scymnus_camptodromus_718 | Scymnus_suturalis_839 | 0.130 |
| Scymnus_camptodromus_707 | Scymnus_suturalis_834 | 0.130 |
| Scymnus_camptodromus_708 | Scymnus_suturalis_834 | 0.130 |
| Scymnus_camptodromus_709 | Scymnus_suturalis_834 | 0.130 |
| Scymnus_camptodromus_710 | Scymnus_suturalis_834 | 0.130 |
| Scymnus_camptodromus_719 | Scymnus_suturalis_834 | 0.130 |
| Anisosticta_bitriangularis_155 | Tytthaspis_sedecimpunctata_577 | 0.130 |
| Anisosticta_bitriangularis_155 | Tytthaspis_sedecimpunctata_578 | 0.130 |
| Anisosticta_bitriangularis_155 | Tytthaspis_sedecimpunctata_579 | 0.130 |
| Anisosticta_bitriangularis_155 | Tytthaspis_sedecimpunctata_581 | 0.130 |
| Anisosticta_bitriangularis_155 | Tytthaspis_sedecimpunctata_582 | 0.130 |
| Anisosticta_bitriangularis_155 | Tytthaspis_sedecimpunctata_590 | 0.130 |
| Anisosticta_bitriangularis_153 | Tytthaspis_sedecimpunctata_592 | 0.130 |
| Anisosticta_bitriangularis_157 | Tytthaspis_sedecimpunctata_592 | 0.130 |
| Anisosticta_bitriangularis_155 | Tytthaspis_sedecimpunctata_593 | 0.130 |
| Anisosticta_bitriangularis_155 | Tytthaspis_sedecimpunctata_594 | 0.130 |
| Anisosticta_bitriangularis_155 | Tytthaspis_sedecimpunctata_595 | 0.130 |
| Anisosticta_bitriangularis_153 | Tytthaspis_sedecimpunctata_580 | 0.130 |
| Anisosticta_bitriangularis_157 | Tytthaspis_sedecimpunctata_580 | 0.130 |
| Anisosticta_bitriangularis_148 | Tytthaspis_sedecimpunctata_577 | 0.130 |
| Anisosticta_bitriangularis_148 | Tytthaspis_sedecimpunctata_578 | 0.130 |
| Anisosticta_bitriangularis_148 | Tytthaspis_sedecimpunctata_579 | 0.130 |
| Anisosticta_bitriangularis_148 | Tytthaspis_sedecimpunctata_581 | 0.130 |
| Anisosticta_bitriangularis_148 | Tytthaspis_sedecimpunctata_582 | 0.130 |
| Anisosticta_bitriangularis_148 | Tytthaspis_sedecimpunctata_590 | 0.130 |
| Anisosticta_bitriangularis_148 | Tytthaspis_sedecimpunctata_593 | 0.130 |
| Anisosticta_bitriangularis_148 | Tytthaspis_sedecimpunctata_594 | 0.130 |
| Anisosticta_bitriangularis_148 | Tytthaspis_sedecimpunctata_595 | 0.130 |
| Scymnus_loewii_770 | Scymnus_suturalis_833 | 0.131 |
| Scymnus_loewii_771 | Scymnus_suturalis_833 | 0.131 |
| Scymnus_camptodromus_705 | Scymnus_suturalis_843 | 0.131 |
| Scymnus_loewii_770 | Scymnus_suturalis_843 | 0.131 |
| Scymnus_loewii_771 | Scymnus_suturalis_843 | 0.131 |
| Scymnus_camptodromus_705 | Scymnus_suturalis_845 | 0.131 |
| Scymnus_loewii_770 | Scymnus_suturalis_845 | 0.131 |
| Scymnus_loewii_771 | Scymnus_suturalis_845 | 0.131 |
| Scymnus_loewii_770 | Scymnus_suturalis_834 | 0.131 |
| Scymnus_loewii_771 | Scymnus_suturalis_834 | 0.131 |
| Scymnus_camptodromus_712 | Scymnus_suturalis_838 | 0.131 |
| Scymnus_camptodromus_718 | Scymnus_suturalis_838 | 0.131 |
| Scymnus_camptodromus_712 | Scymnus_suturalis_840 | 0.131 |
| Scymnus_camptodromus_718 | Scymnus_suturalis_840 | 0.131 |
| Scymnus_camptodromus_712 | Scymnus_suturalis_845 | 0.131 |
| Scymnus_camptodromus_712 | Scymnus_suturalis_834 | 0.131 |

| | | |
|---|---|---|
| Scymnus_camptodromus_718 | Scymnus_suturalis_834 | 0.131 |
| Anisosticta_bitriangularis_155 | Tytthaspis_sedecimpunctata_584 | 0.132 |
| Anisosticta_bitriangularis_153 | Tytthaspis_sedecimpunctata_577 | 0.132 |
| Anisosticta_bitriangularis_157 | Tytthaspis_sedecimpunctata_577 | 0.132 |
| Anisosticta_bitriangularis_153 | Tytthaspis_sedecimpunctata_578 | 0.132 |
| Anisosticta_bitriangularis_157 | Tytthaspis_sedecimpunctata_578 | 0.132 |
| Anisosticta_bitriangularis_153 | Tytthaspis_sedecimpunctata_579 | 0.132 |
| Anisosticta_bitriangularis_157 | Tytthaspis_sedecimpunctata_579 | 0.132 |
| Anisosticta_bitriangularis_153 | Tytthaspis_sedecimpunctata_581 | 0.132 |
| Anisosticta_bitriangularis_157 | Tytthaspis_sedecimpunctata_581 | 0.132 |
| Anisosticta_bitriangularis_153 | Tytthaspis_sedecimpunctata_582 | 0.132 |
| Anisosticta_bitriangularis_157 | Tytthaspis_sedecimpunctata_582 | 0.132 |
| Anisosticta_bitriangularis_155 | Tytthaspis_sedecimpunctata_583 | 0.132 |
| Anisosticta_bitriangularis_155 | Tytthaspis_sedecimpunctata_585 | 0.132 |
| Anisosticta_bitriangularis_153 | Tytthaspis_sedecimpunctata_590 | 0.132 |
| Anisosticta_bitriangularis_157 | Tytthaspis_sedecimpunctata_590 | 0.132 |
| Anisosticta_bitriangularis_158 | Tytthaspis_sedecimpunctata_592 | 0.132 |
| Anisosticta_bitriangularis_153 | Tytthaspis_sedecimpunctata_593 | 0.132 |
| Anisosticta_bitriangularis_157 | Tytthaspis_sedecimpunctata_593 | 0.132 |
| Anisosticta_bitriangularis_153 | Tytthaspis_sedecimpunctata_594 | 0.132 |
| Anisosticta_bitriangularis_157 | Tytthaspis_sedecimpunctata_594 | 0.132 |
| Anisosticta_bitriangularis_153 | Tytthaspis_sedecimpunctata_595 | 0.132 |
| Anisosticta_bitriangularis_157 | Tytthaspis_sedecimpunctata_595 | 0.132 |
| Anisosticta_bitriangularis_158 | Tytthaspis_sedecimpunctata_580 | 0.132 |
| Anisosticta_bitriangularis_148 | Tytthaspis_sedecimpunctata_584 | 0.132 |
| Anisosticta_bitriangularis_148 | Tytthaspis_sedecimpunctata_583 | 0.132 |
| Anisosticta_bitriangularis_148 | Tytthaspis_sedecimpunctata_585 | 0.132 |
| Anisosticta_bitriangularis_149 | Tytthaspis_sedecimpunctata_592 | 0.132 |
| Anisosticta_bitriangularis_149 | Tytthaspis_sedecimpunctata_580 | 0.132 |
| Anisosticta_bitriangularis_150 | Tytthaspis_sedecimpunctata_586 | 0.133 |
| Anisosticta_bitriangularis_150 | Tytthaspis_sedecimpunctata_588 | 0.133 |
| Anisosticta_bitriangularis_150 | Tytthaspis_sedecimpunctata_591 | 0.133 |
| Didion_punctatum_646 | Scymnus_suturalis_845 | 0.133 |
| Didion_punctatum_646 | Scymnus_suturalis_837 | 0.133 |
| Didion_punctatum_644 | Scymnus_suturalis_845 | 0.133 |
| Didion_punctatum_644 | Scymnus_suturalis_837 | 0.133 |
| Scymnus_loewii_774 | Scymnus_suturalis_833 | 0.133 |
| Scymnus_loewii_774 | Scymnus_suturalis_843 | 0.133 |
| Scymnus_loewii_774 | Scymnus_suturalis_845 | 0.133 |
| Scymnus_camptodromus_705 | Scymnus_suturalis_833 | 0.133 |
| Scymnus_loewii_774 | Scymnus_suturalis_834 | 0.133 |
| Scymnus_camptodromus_705 | Scymnus_suturalis_841 | 0.133 |
| Scymnus_loewii_770 | Scymnus_suturalis_841 | 0.133 |
| Scymnus_loewii_771 | Scymnus_suturalis_841 | 0.133 |
| Scymnus_camptodromus_705 | Scymnus_suturalis_842 | 0.133 |
| Scymnus_loewii_770 | Scymnus_suturalis_842 | 0.133 |
| Scymnus_loewii_771 | Scymnus_suturalis_842 | 0.133 |
| Scymnus_camptodromus_713 | Scymnus_suturalis_843 | 0.133 |
| Scymnus_camptodromus_716 | Scymnus_suturalis_843 | 0.133 |
| Scymnus_camptodromus_705 | Scymnus_suturalis_844 | 0.133 |
| Scymnus_loewii_770 | Scymnus_suturalis_844 | 0.133 |
| Scymnus_loewii_771 | Scymnus_suturalis_844 | 0.133 |
| Scymnus_camptodromus_713 | Scymnus_suturalis_845 | 0.133 |
| Scymnus_camptodromus_716 | Scymnus_suturalis_845 | 0.133 |
| Scymnus_camptodromus_705 | Scymnus_suturalis_846 | 0.133 |
| Scymnus_loewii_770 | Scymnus_suturalis_846 | 0.133 |

Supplementary Table S4 (continued)

| | | |
|---|---|---|
| Scymnus_loewii_771 | Scymnus_suturalis_846 | 0.133 |
| Scymnus_camptodromus_705 | Scymnus_suturalis_847 | 0.133 |
| Scymnus_loewii_770 | Scymnus_suturalis_847 | 0.133 |
| Scymnus_loewii_771 | Scymnus_suturalis_847 | 0.133 |
| Scymnus_camptodromus_705 | Scymnus_suturalis_848 | 0.133 |
| Scymnus_loewii_770 | Scymnus_suturalis_848 | 0.133 |
| Scymnus_loewii_771 | Scymnus_suturalis_848 | 0.133 |
| Scymnus_camptodromus_705 | Scymnus_suturalis_832 | 0.133 |
| Scymnus_loewii_770 | Scymnus_suturalis_832 | 0.133 |
| Scymnus_loewii_771 | Scymnus_suturalis_832 | 0.133 |
| Scymnus_camptodromus_705 | Scymnus_suturalis_835 | 0.133 |
| Scymnus_loewii_770 | Scymnus_suturalis_835 | 0.133 |
| Scymnus_loewii_771 | Scymnus_suturalis_835 | 0.133 |
| Scymnus_camptodromus_705 | Scymnus_suturalis_836 | 0.133 |
| Scymnus_loewii_770 | Scymnus_suturalis_836 | 0.133 |
| Scymnus_loewii_771 | Scymnus_suturalis_836 | 0.133 |
| Scymnus_camptodromus_705 | Scymnus_suturalis_837 | 0.133 |
| Scymnus_loewii_770 | Scymnus_suturalis_837 | 0.133 |
| Scymnus_loewii_771 | Scymnus_suturalis_837 | 0.133 |
| Scymnus_camptodromus_705 | Scymnus_suturalis_839 | 0.133 |
| Scymnus_loewii_770 | Scymnus_suturalis_839 | 0.133 |
| Scymnus_loewii_771 | Scymnus_suturalis_839 | 0.133 |
| Scymnus_auritus_699 | Scymnus_suturalis_843 | 0.133 |
| Scymnus_auritus_699 | Scymnus_suturalis_845 | 0.133 |
| Anisosticta_bitriangularis_153 | Tytthaspis_sedecimpunctata_584 | 0.133 |
| Anisosticta_bitriangularis_157 | Tytthaspis_sedecimpunctata_584 | 0.133 |
| Scymnus_auritus_699 | Scymnus_suturalis_834 | 0.133 |
| Anisosticta_bitriangularis_158 | Tytthaspis_sedecimpunctata_577 | 0.133 |
| Anisosticta_bitriangularis_158 | Tytthaspis_sedecimpunctata_578 | 0.133 |
| Anisosticta_bitriangularis_158 | Tytthaspis_sedecimpunctata_579 | 0.133 |
| Anisosticta_bitriangularis_158 | Tytthaspis_sedecimpunctata_581 | 0.133 |
| Anisosticta_bitriangularis_158 | Tytthaspis_sedecimpunctata_582 | 0.133 |
| Anisosticta_bitriangularis_153 | Tytthaspis_sedecimpunctata_583 | 0.133 |
| Anisosticta_bitriangularis_157 | Tytthaspis_sedecimpunctata_583 | 0.133 |
| Anisosticta_bitriangularis_153 | Tytthaspis_sedecimpunctata_585 | 0.133 |
| Anisosticta_bitriangularis_157 | Tytthaspis_sedecimpunctata_585 | 0.133 |
| Anisosticta_bitriangularis_158 | Tytthaspis_sedecimpunctata_590 | 0.133 |
| Anisosticta_bitriangularis_151 | Tytthaspis_sedecimpunctata_592 | 0.133 |
| Anisosticta_bitriangularis_154 | Tytthaspis_sedecimpunctata_592 | 0.133 |
| Anisosticta_bitriangularis_158 | Tytthaspis_sedecimpunctata_593 | 0.133 |
| Anisosticta_bitriangularis_158 | Tytthaspis_sedecimpunctata_594 | 0.133 |
| Anisosticta_bitriangularis_158 | Tytthaspis_sedecimpunctata_595 | 0.133 |
| Scymnus_abietis_682 | Scymnus_suturalis_833 | 0.133 |
| Scymnus_abietis_683 | Scymnus_suturalis_833 | 0.133 |
| Anisosticta_bitriangularis_151 | Tytthaspis_sedecimpunctata_580 | 0.134 |
| Anisosticta_bitriangularis_154 | Tytthaspis_sedecimpunctata_580 | 0.134 |
| Scymnus_abietis_689 | Scymnus_suturalis_833 | 0.134 |
| Anisosticta_bitriangularis_149 | Tytthaspis_sedecimpunctata_577 | 0.134 |
| Anisosticta_bitriangularis_149 | Tytthaspis_sedecimpunctata_578 | 0.134 |
| Anisosticta_bitriangularis_149 | Tytthaspis_sedecimpunctata_579 | 0.134 |
| Anisosticta_bitriangularis_149 | Tytthaspis_sedecimpunctata_581 | 0.134 |
| Anisosticta_bitriangularis_149 | Tytthaspis_sedecimpunctata_582 | 0.134 |
| Anisosticta_bitriangularis_149 | Tytthaspis_sedecimpunctata_590 | 0.134 |
| Anisosticta_bitriangularis_149 | Tytthaspis_sedecimpunctata_593 | 0.134 |
| Anisosticta_bitriangularis_149 | Tytthaspis_sedecimpunctata_594 | 0.134 |
| Anisosticta_bitriangularis_149 | Tytthaspis_sedecimpunctata_595 | 0.134 |

| | | |
|---|---|---|
| Scymnus_loewii_770 | Scymnus_sinuanodulus_829 | 0.134 |
| Scymnus_loewii_771 | Scymnus_sinuanodulus_829 | 0.134 |
| Anisosticta_bitriangularis_147 | Tytthaspis_sedecimpunctata_592 | 0.134 |
| Anisosticta_bitriangularis_147 | Tytthaspis_sedecimpunctata_580 | 0.134 |
| Chilocorus_nigritus_29 | Tytthaspis_sedecimpunctata_580 | 0.135 |
| Didion_punctatum_645 | Scymnus_suturalis_845 | 0.135 |
| Didion_punctatum_645 | Scymnus_suturalis_837 | 0.135 |
| Didion_punctatum_646 | Scymnus_suturalis_841 | 0.135 |
| Didion_punctatum_646 | Scymnus_suturalis_842 | 0.135 |
| Didion_punctatum_646 | Scymnus_suturalis_844 | 0.135 |
| Didion_punctatum_646 | Scymnus_suturalis_846 | 0.135 |
| Didion_punctatum_646 | Scymnus_suturalis_847 | 0.135 |
| Didion_punctatum_646 | Scymnus_suturalis_848 | 0.135 |
| Didion_punctatum_646 | Scymnus_suturalis_832 | 0.135 |
| Didion_punctatum_646 | Scymnus_suturalis_835 | 0.135 |
| Didion_punctatum_646 | Scymnus_suturalis_836 | 0.135 |
| Didion_punctatum_646 | Scymnus_suturalis_839 | 0.135 |
| Didion_punctatum_644 | Scymnus_suturalis_841 | 0.135 |
| Didion_punctatum_644 | Scymnus_suturalis_842 | 0.135 |
| Didion_punctatum_644 | Scymnus_suturalis_844 | 0.135 |
| Didion_punctatum_644 | Scymnus_suturalis_846 | 0.135 |
| Didion_punctatum_644 | Scymnus_suturalis_847 | 0.135 |
| Didion_punctatum_644 | Scymnus_suturalis_848 | 0.135 |
| Didion_punctatum_644 | Scymnus_suturalis_832 | 0.135 |
| Didion_punctatum_646 | Scymnus_suturalis_833 | 0.135 |
| Didion_punctatum_644 | Scymnus_suturalis_835 | 0.135 |
| Didion_punctatum_644 | Scymnus_suturalis_836 | 0.135 |
| Didion_punctatum_644 | Scymnus_suturalis_839 | 0.135 |
| Scymnus_apetzi_692 | Scymnus_rubromaculatus_822 | 0.135 |
| Scymnus_apetzi_692 | Scymnus_rubromaculatus_823 | 0.135 |
| Scymnus_apetzi_692 | Scymnus_rubromaculatus_824 | 0.135 |
| Scymnus_apetzi_692 | Scymnus_rubromaculatus_825 | 0.135 |
| Scymnus_apetzi_692 | Scymnus_rubromaculatus_826 | 0.135 |
| Scymnus_apetzi_692 | Scymnus_rubromaculatus_827 | 0.135 |
| Scymnus_loewii_774 | Scymnus_suturalis_841 | 0.135 |
| Scymnus_loewii_774 | Scymnus_suturalis_842 | 0.135 |
| Scymnus_loewii_774 | Scymnus_suturalis_844 | 0.135 |
| Scymnus_loewii_774 | Scymnus_suturalis_846 | 0.135 |
| Scymnus_loewii_774 | Scymnus_suturalis_847 | 0.135 |
| Scymnus_loewii_774 | Scymnus_suturalis_848 | 0.135 |
| Scymnus_loewii_774 | Scymnus_suturalis_832 | 0.135 |
| Scymnus_camptodromus_713 | Scymnus_suturalis_833 | 0.135 |
| Scymnus_camptodromus_716 | Scymnus_suturalis_833 | 0.135 |
| Scymnus_loewii_773 | Scymnus_suturalis_833 | 0.135 |
| Scymnus_loewii_774 | Scymnus_suturalis_835 | 0.135 |
| Scymnus_loewii_774 | Scymnus_suturalis_836 | 0.135 |
| Scymnus_loewii_774 | Scymnus_suturalis_837 | 0.135 |
| Scymnus_camptodromus_705 | Scymnus_suturalis_838 | 0.135 |
| Scymnus_loewii_770 | Scymnus_suturalis_838 | 0.135 |
| Scymnus_loewii_771 | Scymnus_suturalis_838 | 0.135 |
| Scymnus_loewii_774 | Scymnus_suturalis_839 | 0.135 |
| Scymnus_camptodromus_705 | Scymnus_suturalis_840 | 0.135 |
| Scymnus_loewii_770 | Scymnus_suturalis_840 | 0.135 |
| Scymnus_loewii_771 | Scymnus_suturalis_840 | 0.135 |
| Scymnus_camptodromus_713 | Scymnus_suturalis_841 | 0.135 |
| Scymnus_camptodromus_716 | Scymnus_suturalis_841 | 0.135 |

| | | |
|---|---|---|
| Scymnus_camptodromus_713 | Scymnus_suturalis_842 | 0.135 |
| Scymnus_camptodromus_716 | Scymnus_suturalis_842 | 0.135 |
| Scymnus_loewii_773 | Scymnus_suturalis_843 | 0.135 |
| Scymnus_camptodromus_713 | Scymnus_suturalis_844 | 0.135 |
| Scymnus_camptodromus_716 | Scymnus_suturalis_844 | 0.135 |
| Scymnus_loewii_773 | Scymnus_suturalis_845 | 0.135 |
| Scymnus_camptodromus_713 | Scymnus_suturalis_846 | 0.135 |
| Scymnus_camptodromus_716 | Scymnus_suturalis_846 | 0.135 |
| Scymnus_camptodromus_713 | Scymnus_suturalis_847 | 0.135 |
| Scymnus_camptodromus_716 | Scymnus_suturalis_847 | 0.135 |
| Scymnus_camptodromus_713 | Scymnus_suturalis_848 | 0.135 |
| Scymnus_camptodromus_716 | Scymnus_suturalis_848 | 0.135 |
| Scymnus_camptodromus_713 | Scymnus_suturalis_832 | 0.135 |
| Scymnus_camptodromus_716 | Scymnus_suturalis_832 | 0.135 |
| Scymnus_camptodromus_705 | Scymnus_suturalis_834 | 0.135 |
| Scymnus_loewii_773 | Scymnus_suturalis_834 | 0.135 |
| Scymnus_camptodromus_713 | Scymnus_suturalis_835 | 0.135 |
| Scymnus_camptodromus_716 | Scymnus_suturalis_835 | 0.135 |
| Scymnus_camptodromus_713 | Scymnus_suturalis_836 | 0.135 |
| Scymnus_camptodromus_716 | Scymnus_suturalis_836 | 0.135 |
| Scymnus_camptodromus_713 | Scymnus_suturalis_837 | 0.135 |
| Scymnus_camptodromus_716 | Scymnus_suturalis_837 | 0.135 |
| Scymnus_camptodromus_713 | Scymnus_suturalis_839 | 0.135 |
| Scymnus_camptodromus_716 | Scymnus_suturalis_839 | 0.135 |
| Scymnus_mimulus_778 | Scymnus_rubromaculatus_822 | 0.135 |
| Scymnus_mimulus_778 | Scymnus_rubromaculatus_823 | 0.135 |
| Scymnus_mimulus_778 | Scymnus_rubromaculatus_824 | 0.135 |
| Scymnus_mimulus_778 | Scymnus_rubromaculatus_825 | 0.135 |
| Scymnus_mimulus_778 | Scymnus_rubromaculatus_826 | 0.135 |
| Scymnus_mimulus_778 | Scymnus_rubromaculatus_827 | 0.135 |
| Scymnus_auritus_699 | Scymnus_suturalis_833 | 0.135 |
| Scymnus_auritus_699 | Scymnus_suturalis_841 | 0.135 |
| Scymnus_auritus_699 | Scymnus_suturalis_842 | 0.135 |
| Scymnus_auritus_699 | Scymnus_suturalis_844 | 0.135 |
| Scymnus_auritus_699 | Scymnus_suturalis_846 | 0.135 |
| Scymnus_auritus_699 | Scymnus_suturalis_847 | 0.135 |
| Scymnus_auritus_699 | Scymnus_suturalis_848 | 0.135 |
| Anisosticta_bitriangularis_158 | Tytthaspis_sedecimpunctata_584 | 0.135 |
| Scymnus_auritus_699 | Scymnus_suturalis_832 | 0.135 |
| Scymnus_auritus_699 | Scymnus_suturalis_835 | 0.135 |
| Scymnus_auritus_699 | Scymnus_suturalis_836 | 0.135 |
| Scymnus_auritus_699 | Scymnus_suturalis_837 | 0.135 |
| Scymnus_auritus_699 | Scymnus_suturalis_839 | 0.135 |
| Scymnus_apetzi_694 | Scymnus_rubromaculatus_822 | 0.135 |
| Scymnus_apetzi_694 | Scymnus_rubromaculatus_823 | 0.135 |
| Scymnus_apetzi_694 | Scymnus_rubromaculatus_824 | 0.135 |
| Scymnus_apetzi_694 | Scymnus_rubromaculatus_825 | 0.135 |
| Scymnus_apetzi_694 | Scymnus_rubromaculatus_826 | 0.135 |
| Scymnus_apetzi_694 | Scymnus_rubromaculatus_827 | 0.135 |
| Anisosticta_bitriangularis_151 | Tytthaspis_sedecimpunctata_577 | 0.135 |
| Anisosticta_bitriangularis_154 | Tytthaspis_sedecimpunctata_577 | 0.135 |
| Anisosticta_bitriangularis_151 | Tytthaspis_sedecimpunctata_578 | 0.135 |
| Anisosticta_bitriangularis_154 | Tytthaspis_sedecimpunctata_578 | 0.135 |
| Anisosticta_bitriangularis_151 | Tytthaspis_sedecimpunctata_579 | 0.135 |
| Anisosticta_bitriangularis_154 | Tytthaspis_sedecimpunctata_579 | 0.135 |
| Anisosticta_bitriangularis_151 | Tytthaspis_sedecimpunctata_581 | 0.135 |

| | | |
|---|---|---|
| Anisosticta_bitriangularis_154 | Tytthaspis_sedecimpunctata_581 | 0.135 |
| Anisosticta_bitriangularis_151 | Tytthaspis_sedecimpunctata_582 | 0.135 |
| Anisosticta_bitriangularis_154 | Tytthaspis_sedecimpunctata_582 | 0.135 |
| Anisosticta_bitriangularis_158 | Tytthaspis_sedecimpunctata_583 | 0.135 |
| Anisosticta_bitriangularis_158 | Tytthaspis_sedecimpunctata_585 | 0.135 |
| Anisosticta_bitriangularis_151 | Tytthaspis_sedecimpunctata_590 | 0.135 |
| Anisosticta_bitriangularis_154 | Tytthaspis_sedecimpunctata_590 | 0.135 |
| Anisosticta_bitriangularis_156 | Tytthaspis_sedecimpunctata_592 | 0.135 |
| Anisosticta_bitriangularis_151 | Tytthaspis_sedecimpunctata_593 | 0.135 |
| Anisosticta_bitriangularis_154 | Tytthaspis_sedecimpunctata_593 | 0.135 |
| Anisosticta_bitriangularis_151 | Tytthaspis_sedecimpunctata_594 | 0.135 |
| Anisosticta_bitriangularis_154 | Tytthaspis_sedecimpunctata_594 | 0.135 |
| Anisosticta_bitriangularis_151 | Tytthaspis_sedecimpunctata_595 | 0.135 |
| Anisosticta_bitriangularis_154 | Tytthaspis_sedecimpunctata_595 | 0.135 |
| Scymnus_abietis_684 | Scymnus_suturalis_833 | 0.135 |
| Scymnus_abietis_685 | Scymnus_suturalis_833 | 0.135 |
| Scymnus_abietis_686 | Scymnus_suturalis_833 | 0.135 |
| Scymnus_abietis_687 | Scymnus_suturalis_833 | 0.135 |
| Scymnus_abietis_688 | Scymnus_suturalis_833 | 0.135 |
| Scymnus_loewii_770 | Scymnus_sinuanodulus_831 | 0.135 |
| Scymnus_loewii_771 | Scymnus_sinuanodulus_831 | 0.135 |
| Anisosticta_bitriangularis_156 | Tytthaspis_sedecimpunctata_580 | 0.135 |
| Anisosticta_bitriangularis_149 | Tytthaspis_sedecimpunctata_584 | 0.136 |
| Anisosticta_bitriangularis_149 | Tytthaspis_sedecimpunctata_583 | 0.136 |
| Anisosticta_bitriangularis_149 | Tytthaspis_sedecimpunctata_585 | 0.136 |
| Scymnus_loewii_774 | Scymnus_sinuanodulus_829 | 0.136 |
| Anisosticta_bitriangularis_147 | Tytthaspis_sedecimpunctata_577 | 0.136 |
| Anisosticta_bitriangularis_147 | Tytthaspis_sedecimpunctata_578 | 0.136 |
| Anisosticta_bitriangularis_147 | Tytthaspis_sedecimpunctata_579 | 0.136 |
| Anisosticta_bitriangularis_147 | Tytthaspis_sedecimpunctata_581 | 0.136 |
| Anisosticta_bitriangularis_147 | Tytthaspis_sedecimpunctata_582 | 0.136 |
| Anisosticta_bitriangularis_147 | Tytthaspis_sedecimpunctata_585 | 0.136 |
| Anisosticta_bitriangularis_147 | Tytthaspis_sedecimpunctata_590 | 0.136 |
| Anisosticta_bitriangularis_147 | Tytthaspis_sedecimpunctata_593 | 0.136 |
| Anisosticta_bitriangularis_147 | Tytthaspis_sedecimpunctata_594 | 0.136 |
| Anisosticta_bitriangularis_147 | Tytthaspis_sedecimpunctata_595 | 0.136 |
| Scymnus_nebulosus_782 | Scymnus_suturalis_843 | 0.136 |
| Scymnus_nebulosus_782 | Scymnus_suturalis_845 | 0.136 |
| Scymnus_nebulosus_782 | Scymnus_suturalis_834 | 0.136 |
| Calvia_quatuordecimguttata_211 | Tytthaspis_sedecimpunctata_583 | 0.136 |
| Calvia_quatuordecimguttata_212 | Tytthaspis_sedecimpunctata_583 | 0.136 |
| Calvia_quatuordecimguttata_213 | Tytthaspis_sedecimpunctata_583 | 0.136 |
| Calvia_quatuordecimguttata_214 | Tytthaspis_sedecimpunctata_583 | 0.136 |
| Calvia_quatuordecimguttata_215 | Tytthaspis_sedecimpunctata_583 | 0.136 |
| Anisosticta_bitriangularis_150 | Tytthaspis_sedecimpunctata_589 | 0.136 |
| Calvia_quatuordecimguttata_211 | Tytthaspis_sedecimpunctata_580 | 0.137 |
| Calvia_quatuordecimguttata_212 | Tytthaspis_sedecimpunctata_580 | 0.137 |
| Calvia_quatuordecimguttata_213 | Tytthaspis_sedecimpunctata_580 | 0.137 |
| Calvia_quatuordecimguttata_214 | Tytthaspis_sedecimpunctata_580 | 0.137 |
| Calvia_quatuordecimguttata_215 | Tytthaspis_sedecimpunctata_580 | 0.137 |
| Chilocorus_nigritus_29 | Tytthaspis_sedecimpunctata_577 | 0.137 |
| Chilocorus_nigritus_29 | Tytthaspis_sedecimpunctata_578 | 0.137 |
| Chilocorus_nigritus_29 | Tytthaspis_sedecimpunctata_579 | 0.137 |
| Chilocorus_nigritus_29 | Tytthaspis_sedecimpunctata_581 | 0.137 |
| Chilocorus_nigritus_29 | Tytthaspis_sedecimpunctata_582 | 0.137 |
| Chilocorus_nigritus_29 | Tytthaspis_sedecimpunctata_585 | 0.137 |

| | | |
|---|---|---|
| Chilocorus_nigritus_30 | Tytthaspis_sedecimpunctata_585 | 0.137 |
| Chilocorus_nigritus_29 | Tytthaspis_sedecimpunctata_590 | 0.137 |
| Chilocorus_nigritus_29 | Tytthaspis_sedecimpunctata_592 | 0.137 |
| Chilocorus_nigritus_29 | Tytthaspis_sedecimpunctata_594 | 0.137 |
| Chilocorus_nigritus_29 | Tytthaspis_sedecimpunctata_595 | 0.137 |
| Chilocorus_nigritus_30 | Tytthaspis_sedecimpunctata_580 | 0.137 |
| Chilocorus_nigritus_29 | Tytthaspis_sedecimpunctata_584 | 0.137 |
| Anisosticta_bitriangularis_150 | Tytthaspis_sedecimpunctata_587 | 0.137 |
| Didion_punctatum_645 | Scymnus_suturalis_841 | 0.137 |
| Didion_punctatum_645 | Scymnus_suturalis_842 | 0.137 |
| Didion_punctatum_645 | Scymnus_suturalis_844 | 0.137 |
| Didion_punctatum_645 | Scymnus_suturalis_846 | 0.137 |
| Didion_punctatum_645 | Scymnus_suturalis_847 | 0.137 |
| Didion_punctatum_645 | Scymnus_suturalis_848 | 0.137 |
| Didion_punctatum_645 | Scymnus_suturalis_832 | 0.137 |
| Didion_punctatum_645 | Scymnus_suturalis_835 | 0.137 |
| Didion_punctatum_645 | Scymnus_suturalis_836 | 0.137 |
| Didion_punctatum_646 | Scymnus_suturalis_838 | 0.137 |
| Didion_punctatum_645 | Scymnus_suturalis_839 | 0.137 |
| Didion_punctatum_646 | Scymnus_suturalis_840 | 0.137 |
| Didion_punctatum_646 | Scymnus_suturalis_843 | 0.137 |
| Didion_punctatum_644 | Scymnus_suturalis_833 | 0.137 |
| Didion_punctatum_645 | Scymnus_suturalis_833 | 0.137 |
| Didion_punctatum_646 | Scymnus_suturalis_834 | 0.137 |
| Didion_punctatum_644 | Scymnus_suturalis_838 | 0.137 |
| Didion_punctatum_644 | Scymnus_suturalis_840 | 0.137 |
| Didion_punctatum_644 | Scymnus_suturalis_843 | 0.137 |
| Didion_punctatum_644 | Scymnus_suturalis_834 | 0.137 |
| Scymnus_loewii_774 | Scymnus_suturalis_838 | 0.137 |
| Scymnus_loewii_774 | Scymnus_suturalis_840 | 0.137 |
| Scymnus_camptodromus_713 | Scymnus_suturalis_838 | 0.137 |
| Scymnus_camptodromus_716 | Scymnus_suturalis_838 | 0.137 |
| Scymnus_camptodromus_713 | Scymnus_suturalis_840 | 0.137 |
| Scymnus_camptodromus_716 | Scymnus_suturalis_840 | 0.137 |
| Scymnus_loewii_773 | Scymnus_suturalis_841 | 0.137 |
| Scymnus_loewii_773 | Scymnus_suturalis_842 | 0.137 |
| Scymnus_auritus_698 | Scymnus_suturalis_843 | 0.137 |
| Scymnus_auritus_700 | Scymnus_suturalis_843 | 0.137 |
| Scymnus_loewii_773 | Scymnus_suturalis_844 | 0.137 |
| Scymnus_auritus_698 | Scymnus_suturalis_845 | 0.137 |
| Scymnus_auritus_700 | Scymnus_suturalis_845 | 0.137 |
| Scymnus_loewii_773 | Scymnus_suturalis_846 | 0.137 |
| Scymnus_loewii_773 | Scymnus_suturalis_847 | 0.137 |
| Scymnus_loewii_773 | Scymnus_suturalis_848 | 0.137 |
| Scymnus_mimulus_777 | Scymnus_rubromaculatus_822 | 0.137 |
| Scymnus_mimulus_777 | Scymnus_rubromaculatus_823 | 0.137 |
| Scymnus_mimulus_777 | Scymnus_rubromaculatus_824 | 0.137 |
| Scymnus_mimulus_777 | Scymnus_rubromaculatus_825 | 0.137 |
| Scymnus_mimulus_777 | Scymnus_rubromaculatus_826 | 0.137 |
| Scymnus_mimulus_777 | Scymnus_rubromaculatus_827 | 0.137 |
| Scymnus_loewii_773 | Scymnus_suturalis_832 | 0.137 |
| Scymnus_camptodromus_713 | Scymnus_suturalis_834 | 0.137 |
| Scymnus_camptodromus_716 | Scymnus_suturalis_834 | 0.137 |
| Scymnus_loewii_773 | Scymnus_suturalis_835 | 0.137 |
| Scymnus_loewii_773 | Scymnus_suturalis_836 | 0.137 |
| Scymnus_loewii_773 | Scymnus_suturalis_837 | 0.137 |

Supplementary Table S4 (continued)

| | | |
|---|---|---|
| Scymnus_loewii_773 | Scymnus_suturalis_839 | 0.137 |
| Scymnus_loewii_770 | Scymnus_sinuanodulus_830 | 0.137 |
| Scymnus_loewii_771 | Scymnus_sinuanodulus_830 | 0.137 |
| Scymnus_auritus_699 | Scymnus_suturalis_838 | 0.137 |
| Scymnus_auritus_699 | Scymnus_suturalis_840 | 0.137 |
| Scymnus_ningshanensis_788 | Scymnus_suturalis_843 | 0.137 |
| Scymnus_ningshanensis_791 | Scymnus_suturalis_843 | 0.137 |
| Scymnus_ningshanensis_792 | Scymnus_suturalis_843 | 0.137 |
| Scymnus_ningshanensis_793 | Scymnus_suturalis_843 | 0.137 |
| Scymnus_ningshanensis_794 | Scymnus_suturalis_843 | 0.137 |
| Scymnus_ningshanensis_795 | Scymnus_suturalis_843 | 0.137 |
| Scymnus_ningshanensis_788 | Scymnus_suturalis_845 | 0.137 |
| Scymnus_ningshanensis_791 | Scymnus_suturalis_845 | 0.137 |
| Scymnus_ningshanensis_792 | Scymnus_suturalis_845 | 0.137 |
| Scymnus_ningshanensis_793 | Scymnus_suturalis_845 | 0.137 |
| Scymnus_ningshanensis_794 | Scymnus_suturalis_845 | 0.137 |
| Scymnus_ningshanensis_795 | Scymnus_suturalis_845 | 0.137 |
| Anisosticta_bitriangularis_151 | Tytthaspis_sedecimpunctata_584 | 0.137 |
| Anisosticta_bitriangularis_154 | Tytthaspis_sedecimpunctata_584 | 0.137 |
| Scymnus_loewii_774 | Scymnus_sinuanodulus_831 | 0.137 |
| Anisosticta_bitriangularis_156 | Tytthaspis_sedecimpunctata_577 | 0.137 |
| Anisosticta_bitriangularis_156 | Tytthaspis_sedecimpunctata_578 | 0.137 |
| Anisosticta_bitriangularis_156 | Tytthaspis_sedecimpunctata_579 | 0.137 |
| Anisosticta_bitriangularis_156 | Tytthaspis_sedecimpunctata_581 | 0.137 |
| Anisosticta_bitriangularis_156 | Tytthaspis_sedecimpunctata_582 | 0.137 |
| Anisosticta_bitriangularis_151 | Tytthaspis_sedecimpunctata_583 | 0.137 |
| Anisosticta_bitriangularis_154 | Tytthaspis_sedecimpunctata_583 | 0.137 |
| Anisosticta_bitriangularis_151 | Tytthaspis_sedecimpunctata_585 | 0.137 |
| Anisosticta_bitriangularis_154 | Tytthaspis_sedecimpunctata_585 | 0.137 |
| Anisosticta_bitriangularis_156 | Tytthaspis_sedecimpunctata_590 | 0.137 |
| Anisosticta_bitriangularis_152 | Tytthaspis_sedecimpunctata_592 | 0.137 |
| Anisosticta_bitriangularis_156 | Tytthaspis_sedecimpunctata_593 | 0.137 |
| Anisosticta_bitriangularis_156 | Tytthaspis_sedecimpunctata_594 | 0.137 |
| Anisosticta_bitriangularis_156 | Tytthaspis_sedecimpunctata_595 | 0.137 |
| Scymnus_abietis_682 | Scymnus_suturalis_845 | 0.137 |
| Scymnus_abietis_683 | Scymnus_suturalis_845 | 0.137 |
| Anisosticta_bitriangularis_152 | Tytthaspis_sedecimpunctata_580 | 0.137 |
| Scymnus_abietis_682 | Scymnus_suturalis_834 | 0.137 |
| Scymnus_abietis_683 | Scymnus_suturalis_834 | 0.137 |
| Scymnus_abietis_682 | Scymnus_suturalis_837 | 0.137 |
| Scymnus_abietis_683 | Scymnus_suturalis_837 | 0.137 |
| Scymnus_apetzi_695 | Scymnus_rubromaculatus_822 | 0.137 |
| Scymnus_apetzi_695 | Scymnus_rubromaculatus_823 | 0.137 |
| Scymnus_apetzi_695 | Scymnus_rubromaculatus_824 | 0.137 |
| Scymnus_apetzi_695 | Scymnus_rubromaculatus_825 | 0.137 |
| Scymnus_apetzi_695 | Scymnus_rubromaculatus_826 | 0.137 |
| Scymnus_apetzi_695 | Scymnus_rubromaculatus_827 | 0.137 |
| Scymnus_abietis_689 | Scymnus_suturalis_845 | 0.137 |
| Scymnus_abietis_689 | Scymnus_suturalis_834 | 0.137 |
| Scymnus_abietis_689 | Scymnus_suturalis_837 | 0.137 |
| Scymnus_apetzi_693 | Scymnus_rubromaculatus_822 | 0.138 |
| Scymnus_apetzi_693 | Scymnus_rubromaculatus_823 | 0.138 |
| Scymnus_apetzi_693 | Scymnus_rubromaculatus_824 | 0.138 |
| Scymnus_apetzi_693 | Scymnus_rubromaculatus_825 | 0.138 |
| Scymnus_apetzi_693 | Scymnus_rubromaculatus_826 | 0.138 |
| Scymnus_apetzi_693 | Scymnus_rubromaculatus_827 | 0.138 |

Supplementary Table S4 (continued)

| | | |
|---|---|---|
| Anisosticta_bitriangularis_147 | Tytthaspis_sedecimpunctata_584 | 0.138 |
| Brumoides_suturalis_1 | Tytthaspis_sedecimpunctata_580 | 0.138 |
| Brumoides_suturalis_2 | Tytthaspis_sedecimpunctata_583 | 0.138 |
| Chilocorus_nigritus_27 | Tytthaspis_sedecimpunctata_585 | 0.138 |
| Brumoides_suturalis_1 | Tytthaspis_sedecimpunctata_583 | 0.138 |
| Brumoides_suturalis_1 | Tytthaspis_sedecimpunctata_585 | 0.138 |
| Brumoides_suturalis_1 | Tytthaspis_sedecimpunctata_592 | 0.138 |
| Anisosticta_bitriangularis_147 | Tytthaspis_sedecimpunctata_583 | 0.138 |
| Chilocorus_nigritus_27 | Tytthaspis_sedecimpunctata_580 | 0.138 |
| Scymnus_nebulosus_782 | Scymnus_suturalis_841 | 0.138 |
| Scymnus_nebulosus_782 | Scymnus_suturalis_842 | 0.138 |
| Scymnus_nebulosus_782 | Scymnus_suturalis_844 | 0.138 |
| Scymnus_nebulosus_782 | Scymnus_suturalis_846 | 0.138 |
| Scymnus_nebulosus_782 | Scymnus_suturalis_847 | 0.138 |
| Scymnus_nebulosus_782 | Scymnus_suturalis_848 | 0.138 |
| Scymnus_nebulosus_782 | Scymnus_suturalis_832 | 0.138 |
| Scymnus_nebulosus_782 | Scymnus_suturalis_835 | 0.138 |
| Scymnus_nebulosus_782 | Scymnus_suturalis_836 | 0.138 |
| Scymnus_nebulosus_782 | Scymnus_suturalis_837 | 0.138 |
| Scymnus_nebulosus_782 | Scymnus_suturalis_839 | 0.138 |
| Calvia_quatuordecimguttata_211 | Tytthaspis_sedecimpunctata_577 | 0.138 |
| Calvia_quatuordecimguttata_212 | Tytthaspis_sedecimpunctata_577 | 0.138 |
| Calvia_quatuordecimguttata_213 | Tytthaspis_sedecimpunctata_577 | 0.138 |
| Calvia_quatuordecimguttata_214 | Tytthaspis_sedecimpunctata_577 | 0.138 |
| Calvia_quatuordecimguttata_215 | Tytthaspis_sedecimpunctata_577 | 0.138 |
| Calvia_quatuordecimguttata_211 | Tytthaspis_sedecimpunctata_578 | 0.138 |
| Calvia_quatuordecimguttata_212 | Tytthaspis_sedecimpunctata_578 | 0.138 |
| Calvia_quatuordecimguttata_213 | Tytthaspis_sedecimpunctata_578 | 0.138 |
| Calvia_quatuordecimguttata_214 | Tytthaspis_sedecimpunctata_578 | 0.138 |
| Calvia_quatuordecimguttata_215 | Tytthaspis_sedecimpunctata_578 | 0.138 |
| Calvia_quatuordecimguttata_211 | Tytthaspis_sedecimpunctata_579 | 0.138 |
| Calvia_quatuordecimguttata_212 | Tytthaspis_sedecimpunctata_579 | 0.138 |
| Calvia_quatuordecimguttata_213 | Tytthaspis_sedecimpunctata_579 | 0.138 |
| Calvia_quatuordecimguttata_214 | Tytthaspis_sedecimpunctata_579 | 0.138 |
| Calvia_quatuordecimguttata_215 | Tytthaspis_sedecimpunctata_579 | 0.138 |
| Calvia_quatuordecimguttata_211 | Tytthaspis_sedecimpunctata_581 | 0.138 |
| Calvia_quatuordecimguttata_212 | Tytthaspis_sedecimpunctata_581 | 0.138 |
| Calvia_quatuordecimguttata_213 | Tytthaspis_sedecimpunctata_581 | 0.138 |
| Calvia_quatuordecimguttata_214 | Tytthaspis_sedecimpunctata_581 | 0.138 |
| Calvia_quatuordecimguttata_215 | Tytthaspis_sedecimpunctata_581 | 0.138 |
| Calvia_quatuordecimguttata_211 | Tytthaspis_sedecimpunctata_582 | 0.138 |
| Calvia_quatuordecimguttata_212 | Tytthaspis_sedecimpunctata_582 | 0.138 |
| Calvia_quatuordecimguttata_213 | Tytthaspis_sedecimpunctata_582 | 0.138 |
| Calvia_quatuordecimguttata_214 | Tytthaspis_sedecimpunctata_582 | 0.138 |
| Calvia_quatuordecimguttata_215 | Tytthaspis_sedecimpunctata_582 | 0.138 |
| Calvia_quatuordecimguttata_201 | Tytthaspis_sedecimpunctata_583 | 0.138 |
| Calvia_quatuordecimguttata_202 | Tytthaspis_sedecimpunctata_583 | 0.138 |
| Calvia_quatuordecimguttata_210 | Tytthaspis_sedecimpunctata_583 | 0.138 |
| Calvia_quatuordecimguttata_211 | Tytthaspis_sedecimpunctata_590 | 0.138 |
| Calvia_quatuordecimguttata_212 | Tytthaspis_sedecimpunctata_590 | 0.138 |
| Calvia_quatuordecimguttata_213 | Tytthaspis_sedecimpunctata_590 | 0.138 |
| Calvia_quatuordecimguttata_214 | Tytthaspis_sedecimpunctata_590 | 0.138 |
| Calvia_quatuordecimguttata_215 | Tytthaspis_sedecimpunctata_590 | 0.138 |
| Calvia_quatuordecimguttata_211 | Tytthaspis_sedecimpunctata_592 | 0.138 |
| Calvia_quatuordecimguttata_212 | Tytthaspis_sedecimpunctata_592 | 0.138 |
| Calvia_quatuordecimguttata_213 | Tytthaspis_sedecimpunctata_592 | 0.138 |

| | | |
|---|---|---|
| Calvia_quatuordecimguttata_214 | Tytthaspis_sedecimpunctata_592 | 0.138 |
| Calvia_quatuordecimguttata_215 | Tytthaspis_sedecimpunctata_592 | 0.138 |
| Calvia_quatuordecimguttata_211 | Tytthaspis_sedecimpunctata_594 | 0.138 |
| Calvia_quatuordecimguttata_212 | Tytthaspis_sedecimpunctata_594 | 0.138 |
| Calvia_quatuordecimguttata_213 | Tytthaspis_sedecimpunctata_594 | 0.138 |
| Calvia_quatuordecimguttata_214 | Tytthaspis_sedecimpunctata_594 | 0.138 |
| Calvia_quatuordecimguttata_215 | Tytthaspis_sedecimpunctata_594 | 0.138 |
| Calvia_quatuordecimguttata_211 | Tytthaspis_sedecimpunctata_595 | 0.138 |
| Calvia_quatuordecimguttata_212 | Tytthaspis_sedecimpunctata_595 | 0.138 |
| Calvia_quatuordecimguttata_213 | Tytthaspis_sedecimpunctata_595 | 0.138 |
| Calvia_quatuordecimguttata_214 | Tytthaspis_sedecimpunctata_595 | 0.138 |
| Calvia_quatuordecimguttata_215 | Tytthaspis_sedecimpunctata_595 | 0.138 |
| Chilocorus_nigritus_30 | Tytthaspis_sedecimpunctata_577 | 0.138 |
| Chilocorus_nigritus_30 | Tytthaspis_sedecimpunctata_578 | 0.138 |
| Chilocorus_nigritus_30 | Tytthaspis_sedecimpunctata_579 | 0.138 |
| Chilocorus_nigritus_30 | Tytthaspis_sedecimpunctata_581 | 0.138 |
| Chilocorus_nigritus_30 | Tytthaspis_sedecimpunctata_582 | 0.138 |
| Chilocorus_nigritus_29 | Tytthaspis_sedecimpunctata_583 | 0.138 |
| Chilocorus_nigritus_30 | Tytthaspis_sedecimpunctata_590 | 0.138 |
| Chilocorus_nigritus_30 | Tytthaspis_sedecimpunctata_592 | 0.138 |
| Chilocorus_nigritus_30 | Tytthaspis_sedecimpunctata_594 | 0.138 |
| Chilocorus_nigritus_30 | Tytthaspis_sedecimpunctata_595 | 0.138 |
| Chilocorus_nigritus_30 | Tytthaspis_sedecimpunctata_584 | 0.138 |
| Chilocorus_nigritus_28 | Tytthaspis_sedecimpunctata_585 | 0.138 |
| Calvia_quatuordecimguttata_201 | Tytthaspis_sedecimpunctata_580 | 0.138 |
| Calvia_quatuordecimguttata_202 | Tytthaspis_sedecimpunctata_580 | 0.138 |
| Calvia_quatuordecimguttata_210 | Tytthaspis_sedecimpunctata_580 | 0.138 |
| Chilocorus_nigritus_28 | Tytthaspis_sedecimpunctata_580 | 0.138 |
| Didion_punctatum_645 | Scymnus_suturalis_838 | 0.138 |
| Didion_punctatum_645 | Scymnus_suturalis_840 | 0.138 |
| Didion_punctatum_645 | Scymnus_suturalis_843 | 0.138 |
| Didion_punctatum_645 | Scymnus_suturalis_834 | 0.138 |
| Scymnus_auritus_698 | Scymnus_suturalis_833 | 0.139 |
| Scymnus_auritus_700 | Scymnus_suturalis_833 | 0.139 |
| Scymnus_loewii_772 | Scymnus_suturalis_833 | 0.139 |
| Scymnus_auritus_700 | Scymnus_suturalis_838 | 0.139 |
| Scymnus_loewii_773 | Scymnus_suturalis_838 | 0.139 |
| Scymnus_loewii_773 | Scymnus_suturalis_840 | 0.139 |
| Scymnus_auritus_698 | Scymnus_suturalis_841 | 0.139 |
| Scymnus_auritus_700 | Scymnus_suturalis_841 | 0.139 |
| Scymnus_auritus_698 | Scymnus_suturalis_842 | 0.139 |
| Scymnus_auritus_700 | Scymnus_suturalis_842 | 0.139 |
| Scymnus_loewii_772 | Scymnus_suturalis_843 | 0.139 |
| Scymnus_auritus_698 | Scymnus_suturalis_844 | 0.139 |
| Scymnus_auritus_700 | Scymnus_suturalis_844 | 0.139 |
| Scymnus_loewii_772 | Scymnus_suturalis_845 | 0.139 |
| Scymnus_auritus_698 | Scymnus_suturalis_846 | 0.139 |
| Scymnus_auritus_700 | Scymnus_suturalis_846 | 0.139 |
| Scymnus_auritus_698 | Scymnus_suturalis_847 | 0.139 |
| Scymnus_auritus_700 | Scymnus_suturalis_847 | 0.139 |
| Scymnus_auritus_698 | Scymnus_suturalis_848 | 0.139 |
| Scymnus_auritus_700 | Scymnus_suturalis_848 | 0.139 |
| Scymnus_mimulus_776 | Scymnus_rubromaculatus_822 | 0.139 |
| Scymnus_mimulus_776 | Scymnus_rubromaculatus_823 | 0.139 |
| Scymnus_mimulus_776 | Scymnus_rubromaculatus_824 | 0.139 |
| Scymnus_mimulus_776 | Scymnus_rubromaculatus_825 | 0.139 |

| | | |
|---|---|---|
| Scymnus_mimulus_776 | Scymnus_rubromaculatus_826 | 0.139 |
| Scymnus_mimulus_776 | Scymnus_rubromaculatus_827 | 0.139 |
| Scymnus_loewii_774 | Scymnus_sinuanodulus_830 | 0.139 |
| Scymnus_auritus_698 | Scymnus_suturalis_832 | 0.139 |
| Scymnus_auritus_700 | Scymnus_suturalis_832 | 0.139 |
| Scymnus_loewii_772 | Scymnus_suturalis_834 | 0.139 |
| Scymnus_auritus_698 | Scymnus_suturalis_835 | 0.139 |
| Scymnus_auritus_700 | Scymnus_suturalis_835 | 0.139 |
| Scymnus_auritus_698 | Scymnus_suturalis_836 | 0.139 |
| Scymnus_auritus_700 | Scymnus_suturalis_836 | 0.139 |
| Scymnus_auritus_698 | Scymnus_suturalis_837 | 0.139 |
| Scymnus_auritus_700 | Scymnus_suturalis_837 | 0.139 |
| Scymnus_auritus_698 | Scymnus_suturalis_839 | 0.139 |
| Scymnus_auritus_700 | Scymnus_suturalis_839 | 0.139 |
| Scymnus_ningshanensis_788 | Scymnus_suturalis_833 | 0.139 |
| Scymnus_ningshanensis_791 | Scymnus_suturalis_833 | 0.139 |
| Scymnus_ningshanensis_792 | Scymnus_suturalis_833 | 0.139 |
| Scymnus_ningshanensis_793 | Scymnus_suturalis_833 | 0.139 |
| Scymnus_ningshanensis_794 | Scymnus_suturalis_833 | 0.139 |
| Scymnus_ningshanensis_795 | Scymnus_suturalis_833 | 0.139 |
| Scymnus_ningshanensis_788 | Scymnus_suturalis_841 | 0.139 |
| Scymnus_ningshanensis_791 | Scymnus_suturalis_841 | 0.139 |
| Scymnus_ningshanensis_792 | Scymnus_suturalis_841 | 0.139 |
| Scymnus_ningshanensis_793 | Scymnus_suturalis_841 | 0.139 |
| Scymnus_ningshanensis_794 | Scymnus_suturalis_841 | 0.139 |
| Scymnus_ningshanensis_795 | Scymnus_suturalis_841 | 0.139 |
| Scymnus_ningshanensis_788 | Scymnus_suturalis_842 | 0.139 |
| Scymnus_ningshanensis_791 | Scymnus_suturalis_842 | 0.139 |
| Scymnus_ningshanensis_792 | Scymnus_suturalis_842 | 0.139 |
| Scymnus_ningshanensis_793 | Scymnus_suturalis_842 | 0.139 |
| Scymnus_ningshanensis_794 | Scymnus_suturalis_842 | 0.139 |
| Scymnus_ningshanensis_795 | Scymnus_suturalis_842 | 0.139 |
| Scymnus_ningshanensis_788 | Scymnus_suturalis_844 | 0.139 |
| Scymnus_ningshanensis_791 | Scymnus_suturalis_844 | 0.139 |
| Scymnus_ningshanensis_792 | Scymnus_suturalis_844 | 0.139 |
| Scymnus_ningshanensis_793 | Scymnus_suturalis_844 | 0.139 |
| Scymnus_ningshanensis_794 | Scymnus_suturalis_844 | 0.139 |
| Scymnus_ningshanensis_795 | Scymnus_suturalis_844 | 0.139 |
| Scymnus_ningshanensis_788 | Scymnus_suturalis_846 | 0.139 |
| Scymnus_ningshanensis_791 | Scymnus_suturalis_846 | 0.139 |
| Scymnus_ningshanensis_792 | Scymnus_suturalis_846 | 0.139 |
| Scymnus_ningshanensis_793 | Scymnus_suturalis_846 | 0.139 |
| Scymnus_ningshanensis_794 | Scymnus_suturalis_846 | 0.139 |
| Scymnus_ningshanensis_795 | Scymnus_suturalis_846 | 0.139 |
| Scymnus_ningshanensis_788 | Scymnus_suturalis_847 | 0.139 |
| Scymnus_ningshanensis_791 | Scymnus_suturalis_847 | 0.139 |
| Scymnus_ningshanensis_792 | Scymnus_suturalis_847 | 0.139 |
| Scymnus_ningshanensis_793 | Scymnus_suturalis_847 | 0.139 |
| Scymnus_ningshanensis_794 | Scymnus_suturalis_847 | 0.139 |
| Scymnus_ningshanensis_795 | Scymnus_suturalis_847 | 0.139 |
| Scymnus_ningshanensis_788 | Scymnus_suturalis_848 | 0.139 |
| Scymnus_ningshanensis_791 | Scymnus_suturalis_848 | 0.139 |
| Scymnus_ningshanensis_792 | Scymnus_suturalis_848 | 0.139 |
| Scymnus_ningshanensis_793 | Scymnus_suturalis_848 | 0.139 |
| Scymnus_ningshanensis_794 | Scymnus_suturalis_848 | 0.139 |
| Scymnus_ningshanensis_795 | Scymnus_suturalis_848 | 0.139 |

Supplementary Table S4 (continued)

| | | |
|---|---|---|
| Anisosticta_bitriangularis_156 | Tytthaspis_sedecimpunctata_584 | 0.139 |
| Anisosticta_bitriangularis_155 | Tytthaspis_sedecimpunctata_586 | 0.139 |
| Anisosticta_bitriangularis_155 | Tytthaspis_sedecimpunctata_588 | 0.139 |
| Anisosticta_bitriangularis_155 | Tytthaspis_sedecimpunctata_591 | 0.139 |
| Scymnus_ningshanensis_788 | Scymnus_suturalis_832 | 0.139 |
| Scymnus_ningshanensis_791 | Scymnus_suturalis_832 | 0.139 |
| Scymnus_ningshanensis_792 | Scymnus_suturalis_832 | 0.139 |
| Scymnus_ningshanensis_793 | Scymnus_suturalis_832 | 0.139 |
| Scymnus_ningshanensis_794 | Scymnus_suturalis_832 | 0.139 |
| Scymnus_ningshanensis_795 | Scymnus_suturalis_832 | 0.139 |
| Scymnus_ningshanensis_788 | Scymnus_suturalis_835 | 0.139 |
| Scymnus_ningshanensis_791 | Scymnus_suturalis_835 | 0.139 |
| Scymnus_ningshanensis_792 | Scymnus_suturalis_835 | 0.139 |
| Scymnus_ningshanensis_793 | Scymnus_suturalis_835 | 0.139 |
| Scymnus_ningshanensis_794 | Scymnus_suturalis_835 | 0.139 |
| Scymnus_ningshanensis_795 | Scymnus_suturalis_835 | 0.139 |
| Scymnus_ningshanensis_788 | Scymnus_suturalis_836 | 0.139 |
| Scymnus_ningshanensis_791 | Scymnus_suturalis_836 | 0.139 |
| Scymnus_ningshanensis_792 | Scymnus_suturalis_836 | 0.139 |
| Scymnus_ningshanensis_793 | Scymnus_suturalis_836 | 0.139 |
| Scymnus_ningshanensis_794 | Scymnus_suturalis_836 | 0.139 |
| Scymnus_ningshanensis_795 | Scymnus_suturalis_836 | 0.139 |
| Scymnus_ningshanensis_788 | Scymnus_suturalis_837 | 0.139 |
| Scymnus_ningshanensis_791 | Scymnus_suturalis_837 | 0.139 |
| Scymnus_ningshanensis_792 | Scymnus_suturalis_837 | 0.139 |
| Scymnus_ningshanensis_793 | Scymnus_suturalis_837 | 0.139 |
| Scymnus_ningshanensis_794 | Scymnus_suturalis_837 | 0.139 |
| Scymnus_ningshanensis_795 | Scymnus_suturalis_837 | 0.139 |
| Scymnus_ningshanensis_788 | Scymnus_suturalis_839 | 0.139 |
| Scymnus_ningshanensis_791 | Scymnus_suturalis_839 | 0.139 |
| Scymnus_ningshanensis_792 | Scymnus_suturalis_839 | 0.139 |
| Scymnus_ningshanensis_793 | Scymnus_suturalis_839 | 0.139 |
| Scymnus_ningshanensis_794 | Scymnus_suturalis_839 | 0.139 |
| Scymnus_ningshanensis_795 | Scymnus_suturalis_839 | 0.139 |
| Anisosticta_bitriangularis_152 | Tytthaspis_sedecimpunctata_577 | 0.139 |
| Anisosticta_bitriangularis_152 | Tytthaspis_sedecimpunctata_578 | 0.139 |
| Anisosticta_bitriangularis_152 | Tytthaspis_sedecimpunctata_579 | 0.139 |
| Anisosticta_bitriangularis_152 | Tytthaspis_sedecimpunctata_581 | 0.139 |
| Anisosticta_bitriangularis_152 | Tytthaspis_sedecimpunctata_582 | 0.139 |
| Anisosticta_bitriangularis_156 | Tytthaspis_sedecimpunctata_583 | 0.139 |
| Anisosticta_bitriangularis_156 | Tytthaspis_sedecimpunctata_585 | 0.139 |
| Anisosticta_bitriangularis_152 | Tytthaspis_sedecimpunctata_590 | 0.139 |
| Anisosticta_bitriangularis_152 | Tytthaspis_sedecimpunctata_593 | 0.139 |
| Anisosticta_bitriangularis_152 | Tytthaspis_sedecimpunctata_594 | 0.139 |
| Anisosticta_bitriangularis_152 | Tytthaspis_sedecimpunctata_595 | 0.139 |
| Scymnus_abietis_682 | Scymnus_suturalis_841 | 0.139 |
| Scymnus_abietis_683 | Scymnus_suturalis_841 | 0.139 |
| Scymnus_abietis_682 | Scymnus_suturalis_842 | 0.139 |
| Scymnus_abietis_683 | Scymnus_suturalis_842 | 0.139 |
| Scymnus_abietis_682 | Scymnus_suturalis_844 | 0.139 |
| Scymnus_abietis_683 | Scymnus_suturalis_844 | 0.139 |
| Scymnus_abietis_684 | Scymnus_suturalis_845 | 0.139 |
| Scymnus_abietis_685 | Scymnus_suturalis_845 | 0.139 |
| Scymnus_abietis_686 | Scymnus_suturalis_845 | 0.139 |
| Scymnus_abietis_687 | Scymnus_suturalis_845 | 0.139 |
| Scymnus_abietis_688 | Scymnus_suturalis_845 | 0.139 |

| | | |
|---|---|---|
| Scymnus_abietis_682 | Scymnus_suturalis_846 | 0.139 |
| Scymnus_abietis_683 | Scymnus_suturalis_846 | 0.139 |
| Scymnus_abietis_682 | Scymnus_suturalis_847 | 0.139 |
| Scymnus_abietis_683 | Scymnus_suturalis_847 | 0.139 |
| Scymnus_abietis_682 | Scymnus_suturalis_848 | 0.139 |
| Scymnus_abietis_683 | Scymnus_suturalis_848 | 0.139 |
| Scymnus_abietis_682 | Scymnus_suturalis_832 | 0.139 |
| Scymnus_abietis_683 | Scymnus_suturalis_832 | 0.139 |
| Scymnus_abietis_684 | Scymnus_suturalis_834 | 0.139 |
| Scymnus_abietis_685 | Scymnus_suturalis_834 | 0.139 |
| Scymnus_abietis_686 | Scymnus_suturalis_834 | 0.139 |
| Scymnus_abietis_687 | Scymnus_suturalis_834 | 0.139 |
| Scymnus_abietis_688 | Scymnus_suturalis_834 | 0.139 |
| Scymnus_abietis_682 | Scymnus_suturalis_835 | 0.139 |
| Scymnus_abietis_683 | Scymnus_suturalis_835 | 0.139 |
| Scymnus_abietis_682 | Scymnus_suturalis_836 | 0.139 |
| Scymnus_abietis_683 | Scymnus_suturalis_836 | 0.139 |
| Scymnus_abietis_684 | Scymnus_suturalis_837 | 0.139 |
| Scymnus_abietis_685 | Scymnus_suturalis_837 | 0.139 |
| Scymnus_abietis_686 | Scymnus_suturalis_837 | 0.139 |
| Scymnus_abietis_687 | Scymnus_suturalis_837 | 0.139 |
| Scymnus_abietis_688 | Scymnus_suturalis_837 | 0.139 |
| Scymnus_abietis_682 | Scymnus_suturalis_839 | 0.139 |
| Scymnus_abietis_683 | Scymnus_suturalis_839 | 0.139 |
| Anisosticta_bitriangularis_148 | Tytthaspis_sedecimpunctata_586 | 0.139 |
| Anisosticta_bitriangularis_148 | Tytthaspis_sedecimpunctata_588 | 0.139 |
| Anisosticta_bitriangularis_148 | Tytthaspis_sedecimpunctata_591 | 0.139 |
| Scymnus_abietis_689 | Scymnus_suturalis_841 | 0.139 |
| Scymnus_abietis_689 | Scymnus_suturalis_842 | 0.139 |
| Scymnus_abietis_689 | Scymnus_suturalis_844 | 0.139 |
| Scymnus_abietis_689 | Scymnus_suturalis_846 | 0.139 |
| Scymnus_abietis_689 | Scymnus_suturalis_847 | 0.139 |
| Scymnus_abietis_689 | Scymnus_suturalis_848 | 0.139 |
| Scymnus_abietis_689 | Scymnus_suturalis_832 | 0.139 |
| Scymnus_abietis_689 | Scymnus_suturalis_835 | 0.139 |
| Scymnus_abietis_689 | Scymnus_suturalis_836 | 0.139 |
| Scymnus_abietis_689 | Scymnus_suturalis_839 | 0.139 |
| Scymnus_sinuanodulus_829 | Scymnus_suturalis_833 | 0.139 |
| Chilocorus_nigritus_26 | Tytthaspis_sedecimpunctata_585 | 0.140 |
| Chilocorus_nigritus_26 | Tytthaspis_sedecimpunctata_580 | 0.140 |
| Chilocorus_nigritus_27 | Tytthaspis_sedecimpunctata_588 | 0.140 |
| Brumoides_suturalis_1 | Tytthaspis_sedecimpunctata_577 | 0.140 |
| Brumoides_suturalis_1 | Tytthaspis_sedecimpunctata_578 | 0.140 |
| Brumoides_suturalis_1 | Tytthaspis_sedecimpunctata_579 | 0.140 |
| Brumoides_suturalis_1 | Tytthaspis_sedecimpunctata_581 | 0.140 |
| Brumoides_suturalis_1 | Tytthaspis_sedecimpunctata_582 | 0.140 |
| Brumoides_suturalis_1 | Tytthaspis_sedecimpunctata_590 | 0.140 |
| Brumoides_suturalis_1 | Tytthaspis_sedecimpunctata_593 | 0.140 |
| Brumoides_suturalis_1 | Tytthaspis_sedecimpunctata_594 | 0.140 |
| Brumoides_suturalis_1 | Tytthaspis_sedecimpunctata_595 | 0.140 |
| Chilocorus_nigritus_27 | Tytthaspis_sedecimpunctata_577 | 0.140 |
| Chilocorus_nigritus_27 | Tytthaspis_sedecimpunctata_578 | 0.140 |
| Chilocorus_nigritus_27 | Tytthaspis_sedecimpunctata_579 | 0.140 |
| Chilocorus_nigritus_27 | Tytthaspis_sedecimpunctata_581 | 0.140 |
| Chilocorus_nigritus_27 | Tytthaspis_sedecimpunctata_582 | 0.140 |
| Chilocorus_nigritus_27 | Tytthaspis_sedecimpunctata_590 | 0.140 |

| | | |
|---|---|---|
| Chilocorus_nigritus_27 | Tytthaspis_sedecimpunctata_592 | 0.140 |
| Chilocorus_nigritus_27 | Tytthaspis_sedecimpunctata_594 | 0.140 |
| Chilocorus_nigritus_27 | Tytthaspis_sedecimpunctata_595 | 0.140 |
| Chilocorus_nigritus_27 | Tytthaspis_sedecimpunctata_584 | 0.140 |
| Scymnus_nebulosus_782 | Scymnus_suturalis_833 | 0.140 |
| Scymnus_nebulosus_782 | Scymnus_suturalis_838 | 0.140 |
| Scymnus_nebulosus_782 | Scymnus_suturalis_840 | 0.140 |
| Scymnus_nebulosus_783 | Scymnus_suturalis_843 | 0.140 |
| Scymnus_nebulosus_783 | Scymnus_suturalis_834 | 0.140 |
| Calvia_quatuordecimguttata_201 | Tytthaspis_sedecimpunctata_577 | 0.140 |
| Calvia_quatuordecimguttata_202 | Tytthaspis_sedecimpunctata_577 | 0.140 |
| Calvia_quatuordecimguttata_210 | Tytthaspis_sedecimpunctata_577 | 0.140 |
| Calvia_quatuordecimguttata_201 | Tytthaspis_sedecimpunctata_578 | 0.140 |
| Calvia_quatuordecimguttata_202 | Tytthaspis_sedecimpunctata_578 | 0.140 |
| Calvia_quatuordecimguttata_210 | Tytthaspis_sedecimpunctata_578 | 0.140 |
| Calvia_quatuordecimguttata_201 | Tytthaspis_sedecimpunctata_579 | 0.140 |
| Calvia_quatuordecimguttata_202 | Tytthaspis_sedecimpunctata_579 | 0.140 |
| Calvia_quatuordecimguttata_210 | Tytthaspis_sedecimpunctata_579 | 0.140 |
| Calvia_quatuordecimguttata_201 | Tytthaspis_sedecimpunctata_581 | 0.140 |
| Calvia_quatuordecimguttata_202 | Tytthaspis_sedecimpunctata_581 | 0.140 |
| Calvia_quatuordecimguttata_210 | Tytthaspis_sedecimpunctata_581 | 0.140 |
| Calvia_quatuordecimguttata_201 | Tytthaspis_sedecimpunctata_582 | 0.140 |
| Calvia_quatuordecimguttata_202 | Tytthaspis_sedecimpunctata_582 | 0.140 |
| Calvia_quatuordecimguttata_210 | Tytthaspis_sedecimpunctata_582 | 0.140 |
| Calvia_quatuordecimguttata_211 | Tytthaspis_sedecimpunctata_585 | 0.140 |
| Calvia_quatuordecimguttata_212 | Tytthaspis_sedecimpunctata_585 | 0.140 |
| Calvia_quatuordecimguttata_213 | Tytthaspis_sedecimpunctata_585 | 0.140 |
| Calvia_quatuordecimguttata_214 | Tytthaspis_sedecimpunctata_585 | 0.140 |
| Calvia_quatuordecimguttata_215 | Tytthaspis_sedecimpunctata_585 | 0.140 |
| Calvia_quatuordecimguttata_201 | Tytthaspis_sedecimpunctata_590 | 0.140 |
| Calvia_quatuordecimguttata_202 | Tytthaspis_sedecimpunctata_590 | 0.140 |
| Calvia_quatuordecimguttata_210 | Tytthaspis_sedecimpunctata_590 | 0.140 |
| Calvia_quatuordecimguttata_201 | Tytthaspis_sedecimpunctata_592 | 0.140 |
| Calvia_quatuordecimguttata_202 | Tytthaspis_sedecimpunctata_592 | 0.140 |
| Calvia_quatuordecimguttata_210 | Tytthaspis_sedecimpunctata_592 | 0.140 |
| Calvia_quatuordecimguttata_201 | Tytthaspis_sedecimpunctata_594 | 0.140 |
| Calvia_quatuordecimguttata_202 | Tytthaspis_sedecimpunctata_594 | 0.140 |
| Calvia_quatuordecimguttata_210 | Tytthaspis_sedecimpunctata_594 | 0.140 |
| Calvia_quatuordecimguttata_201 | Tytthaspis_sedecimpunctata_595 | 0.140 |
| Calvia_quatuordecimguttata_202 | Tytthaspis_sedecimpunctata_595 | 0.140 |
| Calvia_quatuordecimguttata_210 | Tytthaspis_sedecimpunctata_595 | 0.140 |
| Chilocorus_nigritus_30 | Tytthaspis_sedecimpunctata_583 | 0.140 |
| Chilocorus_nigritus_28 | Tytthaspis_sedecimpunctata_588 | 0.140 |
| Chilocorus_nigritus_29 | Tytthaspis_sedecimpunctata_593 | 0.140 |
| Chilocorus_nigritus_28 | Tytthaspis_sedecimpunctata_577 | 0.140 |
| Chilocorus_nigritus_28 | Tytthaspis_sedecimpunctata_578 | 0.140 |
| Chilocorus_nigritus_28 | Tytthaspis_sedecimpunctata_579 | 0.140 |
| Chilocorus_nigritus_28 | Tytthaspis_sedecimpunctata_581 | 0.140 |
| Chilocorus_nigritus_28 | Tytthaspis_sedecimpunctata_582 | 0.140 |
| Chilocorus_nigritus_28 | Tytthaspis_sedecimpunctata_590 | 0.140 |
| Chilocorus_nigritus_29 | Tytthaspis_sedecimpunctata_591 | 0.140 |
| Chilocorus_nigritus_28 | Tytthaspis_sedecimpunctata_592 | 0.140 |
| Chilocorus_nigritus_28 | Tytthaspis_sedecimpunctata_594 | 0.140 |
| Chilocorus_nigritus_28 | Tytthaspis_sedecimpunctata_595 | 0.140 |
| Chilocorus_nigritus_28 | Tytthaspis_sedecimpunctata_584 | 0.140 |
| Scymnus_caurinus_720 | Scymnus_suturalis_843 | 0.140 |

| | | |
|---|---|---|
| Scymnus_caurinus_721 | Scymnus_suturalis_843 | 0.140 |
| Scymnus_caurinus_722 | Scymnus_suturalis_843 | 0.140 |
| Scymnus_caurinus_720 | Scymnus_suturalis_845 | 0.140 |
| Scymnus_caurinus_721 | Scymnus_suturalis_845 | 0.140 |
| Scymnus_caurinus_722 | Scymnus_suturalis_845 | 0.140 |
| Calvia_quatuordecimguttata_211 | Tytthaspis_sedecimpunctata_584 | 0.140 |
| Calvia_quatuordecimguttata_212 | Tytthaspis_sedecimpunctata_584 | 0.140 |
| Calvia_quatuordecimguttata_213 | Tytthaspis_sedecimpunctata_584 | 0.140 |
| Calvia_quatuordecimguttata_214 | Tytthaspis_sedecimpunctata_584 | 0.140 |
| Calvia_quatuordecimguttata_215 | Tytthaspis_sedecimpunctata_584 | 0.140 |
| Scymnus_sinuanodulus_830 | Scymnus_suturalis_833 | 0.141 |
| Scymnus_auritus_698 | Scymnus_suturalis_838 | 0.141 |
| Scymnus_auritus_698 | Scymnus_suturalis_840 | 0.141 |
| Scymnus_auritus_700 | Scymnus_suturalis_840 | 0.141 |
| Scymnus_loewii_772 | Scymnus_suturalis_841 | 0.141 |
| Scymnus_loewii_772 | Scymnus_suturalis_842 | 0.141 |
| Scymnus_auritus_702 | Scymnus_suturalis_843 | 0.141 |
| Scymnus_loewii_772 | Scymnus_suturalis_844 | 0.141 |
| Scymnus_auritus_702 | Scymnus_suturalis_845 | 0.141 |
| Scymnus_loewii_772 | Scymnus_suturalis_846 | 0.141 |
| Scymnus_loewii_772 | Scymnus_suturalis_847 | 0.141 |
| Scymnus_loewii_772 | Scymnus_suturalis_848 | 0.141 |
| Scymnus_mimulus_775 | Scymnus_rubromaculatus_822 | 0.141 |
| Scymnus_mimulus_775 | Scymnus_rubromaculatus_823 | 0.141 |
| Scymnus_mimulus_775 | Scymnus_rubromaculatus_824 | 0.141 |
| Scymnus_mimulus_775 | Scymnus_rubromaculatus_825 | 0.141 |
| Scymnus_mimulus_775 | Scymnus_rubromaculatus_826 | 0.141 |
| Scymnus_mimulus_775 | Scymnus_rubromaculatus_827 | 0.141 |
| Scymnus_loewii_770 | Scymnus_sinuanodulus_828 | 0.141 |
| Scymnus_loewii_771 | Scymnus_sinuanodulus_828 | 0.141 |
| Scymnus_loewii_772 | Scymnus_suturalis_832 | 0.141 |
| Scymnus_auritus_698 | Scymnus_suturalis_834 | 0.141 |
| Scymnus_auritus_700 | Scymnus_suturalis_834 | 0.141 |
| Scymnus_loewii_772 | Scymnus_suturalis_835 | 0.141 |
| Scymnus_loewii_772 | Scymnus_suturalis_836 | 0.141 |
| Scymnus_loewii_772 | Scymnus_suturalis_837 | 0.141 |
| Scymnus_loewii_772 | Scymnus_suturalis_839 | 0.141 |
| Scymnus_sinuanodulus_831 | Scymnus_suturalis_833 | 0.141 |
| Scymnus_ningshanensis_788 | Scymnus_suturalis_838 | 0.141 |
| Scymnus_ningshanensis_791 | Scymnus_suturalis_838 | 0.141 |
| Scymnus_ningshanensis_792 | Scymnus_suturalis_838 | 0.141 |
| Scymnus_ningshanensis_793 | Scymnus_suturalis_838 | 0.141 |
| Scymnus_ningshanensis_794 | Scymnus_suturalis_838 | 0.141 |
| Scymnus_ningshanensis_795 | Scymnus_suturalis_838 | 0.141 |
| Scymnus_ningshanensis_788 | Scymnus_suturalis_840 | 0.141 |
| Scymnus_ningshanensis_791 | Scymnus_suturalis_840 | 0.141 |
| Scymnus_ningshanensis_792 | Scymnus_suturalis_840 | 0.141 |
| Scymnus_ningshanensis_793 | Scymnus_suturalis_840 | 0.141 |
| Scymnus_ningshanensis_794 | Scymnus_suturalis_840 | 0.141 |
| Scymnus_ningshanensis_795 | Scymnus_suturalis_840 | 0.141 |
| Scymnus_ningshanensis_789 | Scymnus_suturalis_843 | 0.141 |
| Scymnus_ningshanensis_790 | Scymnus_suturalis_843 | 0.141 |
| Scymnus_ningshanensis_789 | Scymnus_suturalis_845 | 0.141 |
| Scymnus_ningshanensis_790 | Scymnus_suturalis_845 | 0.141 |
| Anisosticta_bitriangularis_152 | Tytthaspis_sedecimpunctata_584 | 0.141 |
| Anisosticta_bitriangularis_153 | Tytthaspis_sedecimpunctata_586 | 0.141 |

Supplementary Table S4 (continued)

| | | |
|---|---|---|
| Anisosticta_bitriangularis_157 | Tytthaspis_sedecimpunctata_586 | 0.141 |
| Anisosticta_bitriangularis_153 | Tytthaspis_sedecimpunctata_588 | 0.141 |
| Anisosticta_bitriangularis_157 | Tytthaspis_sedecimpunctata_588 | 0.141 |
| Anisosticta_bitriangularis_153 | Tytthaspis_sedecimpunctata_591 | 0.141 |
| Anisosticta_bitriangularis_157 | Tytthaspis_sedecimpunctata_591 | 0.141 |
| Scymnus_ningshanensis_788 | Scymnus_suturalis_834 | 0.141 |
| Scymnus_ningshanensis_791 | Scymnus_suturalis_834 | 0.141 |
| Scymnus_ningshanensis_792 | Scymnus_suturalis_834 | 0.141 |
| Scymnus_ningshanensis_793 | Scymnus_suturalis_834 | 0.141 |
| Scymnus_ningshanensis_794 | Scymnus_suturalis_834 | 0.141 |
| Scymnus_ningshanensis_795 | Scymnus_suturalis_834 | 0.141 |
| Scymnus_caurinus_720 | Scymnus_sinuanodulus_831 | 0.141 |
| Scymnus_caurinus_721 | Scymnus_sinuanodulus_831 | 0.141 |
| Scymnus_caurinus_722 | Scymnus_sinuanodulus_831 | 0.141 |
| Anisosticta_bitriangularis_152 | Tytthaspis_sedecimpunctata_583 | 0.141 |
| Anisosticta_bitriangularis_152 | Tytthaspis_sedecimpunctata_585 | 0.141 |
| Scymnus_abietis_682 | Scymnus_suturalis_838 | 0.141 |
| Scymnus_abietis_683 | Scymnus_suturalis_838 | 0.141 |
| Scymnus_abietis_682 | Scymnus_suturalis_840 | 0.141 |
| Scymnus_abietis_683 | Scymnus_suturalis_840 | 0.141 |
| Scymnus_abietis_684 | Scymnus_suturalis_841 | 0.141 |
| Scymnus_abietis_685 | Scymnus_suturalis_841 | 0.141 |
| Scymnus_abietis_686 | Scymnus_suturalis_841 | 0.141 |
| Scymnus_abietis_687 | Scymnus_suturalis_841 | 0.141 |
| Scymnus_abietis_688 | Scymnus_suturalis_841 | 0.141 |
| Scymnus_abietis_684 | Scymnus_suturalis_842 | 0.141 |
| Scymnus_abietis_685 | Scymnus_suturalis_842 | 0.141 |
| Scymnus_abietis_686 | Scymnus_suturalis_842 | 0.141 |
| Scymnus_abietis_687 | Scymnus_suturalis_842 | 0.141 |
| Scymnus_abietis_688 | Scymnus_suturalis_842 | 0.141 |
| Scymnus_abietis_682 | Scymnus_suturalis_843 | 0.141 |
| Scymnus_abietis_683 | Scymnus_suturalis_843 | 0.141 |
| Scymnus_abietis_684 | Scymnus_suturalis_844 | 0.141 |
| Scymnus_abietis_685 | Scymnus_suturalis_844 | 0.141 |
| Scymnus_abietis_686 | Scymnus_suturalis_844 | 0.141 |
| Scymnus_abietis_687 | Scymnus_suturalis_844 | 0.141 |
| Scymnus_abietis_688 | Scymnus_suturalis_844 | 0.141 |
| Scymnus_abietis_684 | Scymnus_suturalis_846 | 0.141 |
| Scymnus_abietis_685 | Scymnus_suturalis_846 | 0.141 |
| Scymnus_abietis_686 | Scymnus_suturalis_846 | 0.141 |
| Scymnus_abietis_687 | Scymnus_suturalis_846 | 0.141 |
| Scymnus_abietis_688 | Scymnus_suturalis_846 | 0.141 |
| Scymnus_abietis_684 | Scymnus_suturalis_847 | 0.141 |
| Scymnus_abietis_685 | Scymnus_suturalis_847 | 0.141 |
| Scymnus_abietis_686 | Scymnus_suturalis_847 | 0.141 |
| Scymnus_abietis_687 | Scymnus_suturalis_847 | 0.141 |
| Scymnus_abietis_688 | Scymnus_suturalis_847 | 0.141 |
| Scymnus_abietis_684 | Scymnus_suturalis_848 | 0.141 |
| Scymnus_abietis_685 | Scymnus_suturalis_848 | 0.141 |
| Scymnus_abietis_686 | Scymnus_suturalis_848 | 0.141 |
| Scymnus_abietis_687 | Scymnus_suturalis_848 | 0.141 |
| Scymnus_abietis_688 | Scymnus_suturalis_848 | 0.141 |
| Scymnus_abietis_684 | Scymnus_suturalis_832 | 0.141 |
| Scymnus_abietis_685 | Scymnus_suturalis_832 | 0.141 |
| Scymnus_abietis_686 | Scymnus_suturalis_832 | 0.141 |
| Scymnus_abietis_687 | Scymnus_suturalis_832 | 0.141 |

| | | |
|---|---|---|
| Scymnus_abietis_688 | Scymnus_suturalis_832 | 0.141 |
| Scymnus_abietis_684 | Scymnus_suturalis_835 | 0.141 |
| Scymnus_abietis_685 | Scymnus_suturalis_835 | 0.141 |
| Scymnus_abietis_686 | Scymnus_suturalis_835 | 0.141 |
| Scymnus_abietis_687 | Scymnus_suturalis_835 | 0.141 |
| Scymnus_abietis_688 | Scymnus_suturalis_835 | 0.141 |
| Scymnus_abietis_684 | Scymnus_suturalis_836 | 0.141 |
| Scymnus_abietis_685 | Scymnus_suturalis_836 | 0.141 |
| Scymnus_abietis_686 | Scymnus_suturalis_836 | 0.141 |
| Scymnus_abietis_687 | Scymnus_suturalis_836 | 0.141 |
| Scymnus_abietis_688 | Scymnus_suturalis_836 | 0.141 |
| Scymnus_abietis_684 | Scymnus_suturalis_839 | 0.141 |
| Scymnus_abietis_685 | Scymnus_suturalis_839 | 0.141 |
| Scymnus_abietis_686 | Scymnus_suturalis_839 | 0.141 |
| Scymnus_abietis_687 | Scymnus_suturalis_839 | 0.141 |
| Scymnus_abietis_688 | Scymnus_suturalis_839 | 0.141 |
| Scymnus_abietis_689 | Scymnus_suturalis_838 | 0.141 |
| Scymnus_abietis_689 | Scymnus_suturalis_840 | 0.141 |
| Scymnus_abietis_689 | Scymnus_suturalis_843 | 0.141 |
| Scymnus_caurinus_720 | Scymnus_sinuanodulus_829 | 0.141 |
| Scymnus_caurinus_721 | Scymnus_sinuanodulus_829 | 0.141 |
| Scymnus_caurinus_722 | Scymnus_sinuanodulus_829 | 0.141 |
| Scymnus_loewii_772 | Scymnus_sinuanodulus_829 | 0.142 |
| Scymnus_loewii_773 | Scymnus_sinuanodulus_829 | 0.142 |
| Brumoides_suturalis_1 | Tytthaspis_sedecimpunctata_584 | 0.142 |
| Brumoides_suturalis_2 | Tytthaspis_sedecimpunctata_592 | 0.142 |
| Chilocorus_nigritus_26 | Tytthaspis_sedecimpunctata_588 | 0.142 |
| Chilocorus_nigritus_26 | Tytthaspis_sedecimpunctata_577 | 0.142 |
| Chilocorus_nigritus_26 | Tytthaspis_sedecimpunctata_578 | 0.142 |
| Chilocorus_nigritus_26 | Tytthaspis_sedecimpunctata_579 | 0.142 |
| Chilocorus_nigritus_26 | Tytthaspis_sedecimpunctata_581 | 0.142 |
| Chilocorus_nigritus_26 | Tytthaspis_sedecimpunctata_582 | 0.142 |
| Chilocorus_nigritus_26 | Tytthaspis_sedecimpunctata_590 | 0.142 |
| Chilocorus_nigritus_26 | Tytthaspis_sedecimpunctata_592 | 0.142 |
| Chilocorus_nigritus_26 | Tytthaspis_sedecimpunctata_594 | 0.142 |
| Chilocorus_nigritus_26 | Tytthaspis_sedecimpunctata_595 | 0.142 |
| Scymnus_interruptus_762 | Scymnus_suturalis_833 | 0.142 |
| Scymnus_interruptus_762 | Scymnus_suturalis_843 | 0.142 |
| Scymnus_interruptus_762 | Scymnus_suturalis_845 | 0.142 |
| Chilocorus_nigritus_26 | Tytthaspis_sedecimpunctata_584 | 0.142 |
| Chilocorus_nigritus_27 | Tytthaspis_sedecimpunctata_586 | 0.142 |
| Chilocorus_nigritus_27 | Tytthaspis_sedecimpunctata_587 | 0.142 |
| Scymnus_interruptus_762 | Scymnus_suturalis_837 | 0.142 |
| Chilocorus_nigritus_27 | Tytthaspis_sedecimpunctata_583 | 0.142 |
| Exochomus_nigromaculatus_48 | Tytthaspis_sedecimpunctata_593 | 0.142 |
| Scymnus_nebulosus_783 | Scymnus_suturalis_841 | 0.142 |
| Scymnus_nebulosus_783 | Scymnus_suturalis_842 | 0.142 |
| Scymnus_nebulosus_783 | Scymnus_suturalis_844 | 0.142 |
| Scymnus_nebulosus_783 | Scymnus_suturalis_846 | 0.142 |
| Scymnus_nebulosus_783 | Scymnus_suturalis_847 | 0.142 |
| Scymnus_nebulosus_783 | Scymnus_suturalis_848 | 0.142 |
| Scymnus_apetzi_696 | Scymnus_rubromaculatus_822 | 0.142 |
| Scymnus_apetzi_696 | Scymnus_rubromaculatus_823 | 0.142 |
| Scymnus_apetzi_696 | Scymnus_rubromaculatus_824 | 0.142 |
| Scymnus_apetzi_696 | Scymnus_rubromaculatus_825 | 0.142 |
| Scymnus_apetzi_696 | Scymnus_rubromaculatus_826 | 0.142 |

| | | |
|---|---|---|
| Scymnus_apetzi_696 | Scymnus_rubromaculatus_827 | 0.142 |
| Scymnus_nebulosus_783 | Scymnus_suturalis_832 | 0.142 |
| Scymnus_nebulosus_783 | Scymnus_suturalis_835 | 0.142 |
| Scymnus_nebulosus_783 | Scymnus_suturalis_836 | 0.142 |
| Scymnus_nebulosus_783 | Scymnus_suturalis_837 | 0.142 |
| Scymnus_nebulosus_783 | Scymnus_suturalis_839 | 0.142 |
| Chilocorus_nigritus_29 | Tytthaspis_sedecimpunctata_588 | 0.142 |
| Chilocorus_nigritus_30 | Tytthaspis_sedecimpunctata_588 | 0.142 |
| Chilocorus_nigritus_28 | Tytthaspis_sedecimpunctata_586 | 0.142 |
| Chilocorus_nigritus_28 | Tytthaspis_sedecimpunctata_587 | 0.142 |
| Chilocorus_nigritus_30 | Tytthaspis_sedecimpunctata_593 | 0.142 |
| Calvia_quatuordecimguttata_201 | Tytthaspis_sedecimpunctata_585 | 0.142 |
| Calvia_quatuordecimguttata_202 | Tytthaspis_sedecimpunctata_585 | 0.142 |
| Calvia_quatuordecimguttata_210 | Tytthaspis_sedecimpunctata_585 | 0.142 |
| Calvia_quatuordecimguttata_211 | Tytthaspis_sedecimpunctata_593 | 0.142 |
| Calvia_quatuordecimguttata_212 | Tytthaspis_sedecimpunctata_593 | 0.142 |
| Calvia_quatuordecimguttata_213 | Tytthaspis_sedecimpunctata_593 | 0.142 |
| Calvia_quatuordecimguttata_214 | Tytthaspis_sedecimpunctata_593 | 0.142 |
| Calvia_quatuordecimguttata_215 | Tytthaspis_sedecimpunctata_593 | 0.142 |
| Chilocorus_nigritus_28 | Tytthaspis_sedecimpunctata_583 | 0.142 |
| Chilocorus_nigritus_30 | Tytthaspis_sedecimpunctata_591 | 0.142 |
| Scymnus_caurinus_720 | Scymnus_suturalis_841 | 0.142 |
| Scymnus_caurinus_721 | Scymnus_suturalis_841 | 0.142 |
| Scymnus_caurinus_722 | Scymnus_suturalis_841 | 0.142 |
| Scymnus_caurinus_720 | Scymnus_suturalis_842 | 0.142 |
| Scymnus_caurinus_721 | Scymnus_suturalis_842 | 0.142 |
| Scymnus_caurinus_722 | Scymnus_suturalis_842 | 0.142 |
| Scymnus_caurinus_720 | Scymnus_suturalis_844 | 0.142 |
| Scymnus_caurinus_721 | Scymnus_suturalis_844 | 0.142 |
| Scymnus_caurinus_722 | Scymnus_suturalis_844 | 0.142 |
| Scymnus_caurinus_720 | Scymnus_suturalis_846 | 0.142 |
| Scymnus_caurinus_721 | Scymnus_suturalis_846 | 0.142 |
| Scymnus_caurinus_722 | Scymnus_suturalis_846 | 0.142 |
| Scymnus_caurinus_720 | Scymnus_suturalis_847 | 0.142 |
| Scymnus_caurinus_721 | Scymnus_suturalis_847 | 0.142 |
| Scymnus_caurinus_722 | Scymnus_suturalis_847 | 0.142 |
| Scymnus_caurinus_720 | Scymnus_suturalis_848 | 0.142 |
| Scymnus_caurinus_721 | Scymnus_suturalis_848 | 0.142 |
| Scymnus_caurinus_722 | Scymnus_suturalis_848 | 0.142 |
| Calvia_quatuordecimguttata_201 | Tytthaspis_sedecimpunctata_584 | 0.142 |
| Calvia_quatuordecimguttata_202 | Tytthaspis_sedecimpunctata_584 | 0.142 |
| Calvia_quatuordecimguttata_210 | Tytthaspis_sedecimpunctata_584 | 0.142 |
| Scymnus_apetzi_691 | Scymnus_rubromaculatus_822 | 0.142 |
| Scymnus_apetzi_691 | Scymnus_rubromaculatus_823 | 0.142 |
| Scymnus_apetzi_691 | Scymnus_rubromaculatus_824 | 0.142 |
| Scymnus_apetzi_691 | Scymnus_rubromaculatus_825 | 0.142 |
| Scymnus_apetzi_691 | Scymnus_rubromaculatus_826 | 0.142 |
| Scymnus_apetzi_691 | Scymnus_rubromaculatus_827 | 0.142 |
| Scymnus_caurinus_720 | Scymnus_suturalis_832 | 0.142 |
| Scymnus_caurinus_721 | Scymnus_suturalis_832 | 0.142 |
| Scymnus_caurinus_722 | Scymnus_suturalis_832 | 0.142 |
| Scymnus_caurinus_720 | Scymnus_suturalis_835 | 0.142 |
| Scymnus_caurinus_721 | Scymnus_suturalis_835 | 0.142 |
| Scymnus_caurinus_722 | Scymnus_suturalis_835 | 0.142 |
| Scymnus_caurinus_720 | Scymnus_suturalis_836 | 0.142 |
| Scymnus_caurinus_721 | Scymnus_suturalis_836 | 0.142 |

| | | |
|---|---|---|
| Scymnus_caurinus_722 | Scymnus_suturalis_836 | 0.142 |
| Scymnus_caurinus_720 | Scymnus_suturalis_837 | 0.142 |
| Scymnus_caurinus_721 | Scymnus_suturalis_837 | 0.142 |
| Scymnus_caurinus_722 | Scymnus_suturalis_837 | 0.142 |
| Scymnus_caurinus_720 | Scymnus_suturalis_839 | 0.142 |
| Scymnus_caurinus_721 | Scymnus_suturalis_839 | 0.142 |
| Scymnus_caurinus_722 | Scymnus_suturalis_839 | 0.142 |
| Scymnus_pallipediformis_811 | Scymnus_rubromaculatus_822 | 0.142 |
| Scymnus_pallipediformis_811 | Scymnus_rubromaculatus_823 | 0.142 |
| Scymnus_pallipediformis_811 | Scymnus_rubromaculatus_824 | 0.142 |
| Scymnus_pallipediformis_811 | Scymnus_rubromaculatus_825 | 0.142 |
| Scymnus_pallipediformis_811 | Scymnus_rubromaculatus_826 | 0.142 |
| Scymnus_pallipediformis_811 | Scymnus_rubromaculatus_827 | 0.142 |
| Scymnus_loewii_774 | Scymnus_sinuanodulus_828 | 0.143 |
| Scymnus_auritus_702 | Scymnus_suturalis_833 | 0.143 |
| Scymnus_loewii_772 | Scymnus_suturalis_838 | 0.143 |
| Scymnus_loewii_772 | Scymnus_suturalis_840 | 0.143 |
| Scymnus_auritus_702 | Scymnus_suturalis_841 | 0.143 |
| Scymnus_auritus_702 | Scymnus_suturalis_842 | 0.143 |
| Scymnus_auritus_702 | Scymnus_suturalis_844 | 0.143 |
| Scymnus_auritus_702 | Scymnus_suturalis_846 | 0.143 |
| Scymnus_auritus_702 | Scymnus_suturalis_847 | 0.143 |
| Scymnus_auritus_702 | Scymnus_suturalis_848 | 0.143 |
| Anisosticta_novemdecimpunctata_160 | Tytthaspis_sedecimpunctata_585 | 0.143 |
| Anisosticta_novemdecimpunctata_161 | Tytthaspis_sedecimpunctata_585 | 0.143 |
| Anisosticta_novemdecimpunctata_160 | Tytthaspis_sedecimpunctata_592 | 0.143 |
| Anisosticta_novemdecimpunctata_161 | Tytthaspis_sedecimpunctata_592 | 0.143 |
| Scymnus_auritus_702 | Scymnus_suturalis_832 | 0.143 |
| Scymnus_auritus_702 | Scymnus_suturalis_835 | 0.143 |
| Scymnus_auritus_702 | Scymnus_suturalis_836 | 0.143 |
| Scymnus_auritus_702 | Scymnus_suturalis_837 | 0.143 |
| Scymnus_auritus_702 | Scymnus_suturalis_839 | 0.143 |
| Anisosticta_bitriangularis_155 | Tytthaspis_sedecimpunctata_589 | 0.143 |
| Scymnus_ningshanensis_789 | Scymnus_suturalis_833 | 0.143 |
| Scymnus_ningshanensis_790 | Scymnus_suturalis_833 | 0.143 |
| Scymnus_ningshanensis_789 | Scymnus_suturalis_841 | 0.143 |
| Scymnus_ningshanensis_790 | Scymnus_suturalis_841 | 0.143 |
| Scymnus_ningshanensis_789 | Scymnus_suturalis_842 | 0.143 |
| Scymnus_ningshanensis_790 | Scymnus_suturalis_842 | 0.143 |
| Scymnus_ningshanensis_789 | Scymnus_suturalis_844 | 0.143 |
| Scymnus_ningshanensis_790 | Scymnus_suturalis_844 | 0.143 |
| Scymnus_ningshanensis_789 | Scymnus_suturalis_846 | 0.143 |
| Scymnus_ningshanensis_790 | Scymnus_suturalis_846 | 0.143 |
| Scymnus_ningshanensis_789 | Scymnus_suturalis_847 | 0.143 |
| Scymnus_ningshanensis_790 | Scymnus_suturalis_847 | 0.143 |
| Scymnus_ningshanensis_789 | Scymnus_suturalis_848 | 0.143 |
| Scymnus_ningshanensis_790 | Scymnus_suturalis_848 | 0.143 |
| Anisosticta_bitriangularis_158 | Tytthaspis_sedecimpunctata_586 | 0.143 |
| Anisosticta_bitriangularis_155 | Tytthaspis_sedecimpunctata_587 | 0.143 |
| Anisosticta_bitriangularis_158 | Tytthaspis_sedecimpunctata_588 | 0.143 |
| Anisosticta_bitriangularis_158 | Tytthaspis_sedecimpunctata_591 | 0.143 |
| Scymnus_ningshanensis_789 | Scymnus_suturalis_832 | 0.143 |
| Scymnus_ningshanensis_790 | Scymnus_suturalis_832 | 0.143 |
| Scymnus_ningshanensis_789 | Scymnus_suturalis_835 | 0.143 |
| Scymnus_ningshanensis_790 | Scymnus_suturalis_835 | 0.143 |
| Scymnus_ningshanensis_789 | Scymnus_suturalis_836 | 0.143 |

| | | |
|---|---|---|
| Scymnus_ningshanensis_790 | Scymnus_suturalis_836 | 0.143 |
| Scymnus_ningshanensis_789 | Scymnus_suturalis_837 | 0.143 |
| Scymnus_ningshanensis_790 | Scymnus_suturalis_837 | 0.143 |
| Scymnus_ningshanensis_789 | Scymnus_suturalis_839 | 0.143 |
| Scymnus_ningshanensis_790 | Scymnus_suturalis_839 | 0.143 |
| Scymnus_nigrinus_787 | Scymnus_rubromaculatus_822 | 0.143 |
| Scymnus_nigrinus_787 | Scymnus_rubromaculatus_823 | 0.143 |
| Scymnus_nigrinus_787 | Scymnus_rubromaculatus_824 | 0.143 |
| Scymnus_nigrinus_787 | Scymnus_rubromaculatus_825 | 0.143 |
| Scymnus_nigrinus_787 | Scymnus_rubromaculatus_826 | 0.143 |
| Scymnus_nigrinus_787 | Scymnus_rubromaculatus_827 | 0.143 |
| Scymnus_abietis_684 | Scymnus_suturalis_838 | 0.143 |
| Scymnus_abietis_685 | Scymnus_suturalis_838 | 0.143 |
| Scymnus_abietis_686 | Scymnus_suturalis_838 | 0.143 |
| Scymnus_abietis_687 | Scymnus_suturalis_838 | 0.143 |
| Scymnus_abietis_688 | Scymnus_suturalis_838 | 0.143 |
| Scymnus_abietis_684 | Scymnus_suturalis_840 | 0.143 |
| Scymnus_abietis_685 | Scymnus_suturalis_840 | 0.143 |
| Scymnus_abietis_686 | Scymnus_suturalis_840 | 0.143 |
| Scymnus_abietis_687 | Scymnus_suturalis_840 | 0.143 |
| Scymnus_abietis_688 | Scymnus_suturalis_840 | 0.143 |
| Anisosticta_bitriangularis_148 | Tytthaspis_sedecimpunctata_589 | 0.143 |
| Scymnus_loewii_772 | Scymnus_sinuanodulus_831 | 0.143 |
| Scymnus_loewii_773 | Scymnus_sinuanodulus_831 | 0.143 |
| Scymnus_abietis_684 | Scymnus_suturalis_843 | 0.143 |
| Scymnus_abietis_685 | Scymnus_suturalis_843 | 0.143 |
| Scymnus_abietis_686 | Scymnus_suturalis_843 | 0.143 |
| Scymnus_abietis_687 | Scymnus_suturalis_843 | 0.143 |
| Scymnus_abietis_688 | Scymnus_suturalis_843 | 0.143 |
| Anisosticta_bitriangularis_148 | Tytthaspis_sedecimpunctata_587 | 0.143 |
| Anisosticta_bitriangularis_149 | Tytthaspis_sedecimpunctata_586 | 0.143 |
| Anisosticta_bitriangularis_149 | Tytthaspis_sedecimpunctata_588 | 0.143 |
| Anisosticta_bitriangularis_149 | Tytthaspis_sedecimpunctata_591 | 0.143 |
| Scymnus_abietis_682 | Scymnus_sinuanodulus_831 | 0.143 |
| Scymnus_abietis_683 | Scymnus_sinuanodulus_831 | 0.143 |
| Scymnus_abietis_685 | Scymnus_sinuanodulus_831 | 0.143 |
| Scymnus_nebulosus_781 | Scymnus_sinuanodulus_829 | 0.143 |
| Scymnus_sinuanodulus_829 | Scymnus_suturalis_843 | 0.143 |
| Scymnus_sinuanodulus_829 | Scymnus_suturalis_845 | 0.143 |
| Scymnus_sinuanodulus_829 | Scymnus_suturalis_834 | 0.143 |
| Scymnus_sinuanodulus_829 | Scymnus_suturalis_838 | 0.143 |
| Brumoides_suturalis_2 | Tytthaspis_sedecimpunctata_580 | 0.144 |
| Brumoides_suturalis_2 | Tytthaspis_sedecimpunctata_577 | 0.144 |
| Brumoides_suturalis_2 | Tytthaspis_sedecimpunctata_578 | 0.144 |
| Brumoides_suturalis_2 | Tytthaspis_sedecimpunctata_579 | 0.144 |
| Brumoides_suturalis_2 | Tytthaspis_sedecimpunctata_581 | 0.144 |
| Brumoides_suturalis_2 | Tytthaspis_sedecimpunctata_582 | 0.144 |
| Brumoides_suturalis_2 | Tytthaspis_sedecimpunctata_590 | 0.144 |
| Brumoides_suturalis_2 | Tytthaspis_sedecimpunctata_594 | 0.144 |
| Brumoides_suturalis_2 | Tytthaspis_sedecimpunctata_595 | 0.144 |
| Chilocorus_nigritus_26 | Tytthaspis_sedecimpunctata_586 | 0.144 |
| Chilocorus_nigritus_26 | Tytthaspis_sedecimpunctata_587 | 0.144 |
| Brumoides_suturalis_1 | Tytthaspis_sedecimpunctata_588 | 0.144 |
| Chilocorus_nigritus_26 | Tytthaspis_sedecimpunctata_583 | 0.144 |
| Chilocorus_nigritus_25 | Tytthaspis_sedecimpunctata_585 | 0.144 |
| Chilocorus_nigritus_27 | Tytthaspis_sedecimpunctata_589 | 0.144 |

| | | |
|---|---|---|
| Scymnus_interruptus_762 | Scymnus_suturalis_841 | 0.144 |
| Scymnus_interruptus_762 | Scymnus_suturalis_842 | 0.144 |
| Scymnus_interruptus_762 | Scymnus_suturalis_844 | 0.144 |
| Scymnus_interruptus_762 | Scymnus_suturalis_846 | 0.144 |
| Scymnus_interruptus_762 | Scymnus_suturalis_847 | 0.144 |
| Scymnus_interruptus_762 | Scymnus_suturalis_848 | 0.144 |
| Chilocorus_nigritus_25 | Tytthaspis_sedecimpunctata_580 | 0.144 |
| Scymnus_interruptus_762 | Scymnus_suturalis_832 | 0.144 |
| Scymnus_interruptus_762 | Scymnus_suturalis_835 | 0.144 |
| Scymnus_interruptus_762 | Scymnus_suturalis_836 | 0.144 |
| Scymnus_interruptus_762 | Scymnus_suturalis_839 | 0.144 |
| Scymnus_abietis_682 | Scymnus_sinuanodulus_829 | 0.144 |
| Scymnus_abietis_683 | Scymnus_sinuanodulus_829 | 0.144 |
| Scymnus_abietis_685 | Scymnus_sinuanodulus_829 | 0.144 |
| Chilocorus_nigritus_27 | Tytthaspis_sedecimpunctata_593 | 0.144 |
| Hippodamia_variegata_471 | Tytthaspis_sedecimpunctata_585 | 0.144 |
| Exochomus_nigromaculatus_48 | Tytthaspis_sedecimpunctata_588 | 0.144 |
| Chilocorus_nigritus_27 | Tytthaspis_sedecimpunctata_591 | 0.144 |
| Hippodamia_variegata_471 | Tytthaspis_sedecimpunctata_592 | 0.144 |
| Exochomus_nigromaculatus_49 | Tytthaspis_sedecimpunctata_593 | 0.144 |
| Hippodamia_variegata_471 | Tytthaspis_sedecimpunctata_580 | 0.144 |
| Exochomus_nigromaculatus_48 | Tytthaspis_sedecimpunctata_585 | 0.144 |
| Hippodamia_variegata_468 | Tytthaspis_sedecimpunctata_585 | 0.144 |
| Exochomus_nigromaculatus_48 | Tytthaspis_sedecimpunctata_592 | 0.144 |
| Hippodamia_variegata_468 | Tytthaspis_sedecimpunctata_592 | 0.144 |
| Hippodamia_variegata_468 | Tytthaspis_sedecimpunctata_580 | 0.144 |
| Anisosticta_bitriangularis_147 | Tytthaspis_sedecimpunctata_586 | 0.144 |
| Anisosticta_bitriangularis_147 | Tytthaspis_sedecimpunctata_588 | 0.144 |
| Scymnus_nebulosus_783 | Scymnus_suturalis_833 | 0.144 |
| Scymnus_nebulosus_783 | Scymnus_suturalis_838 | 0.144 |
| Scymnus_nebulosus_783 | Scymnus_suturalis_840 | 0.144 |
| Scymnus_nebulosus_783 | Scymnus_suturalis_845 | 0.144 |
| Chilocorus_nigritus_29 | Tytthaspis_sedecimpunctata_586 | 0.144 |
| Chilocorus_nigritus_30 | Tytthaspis_sedecimpunctata_586 | 0.144 |
| Chilocorus_nigritus_28 | Tytthaspis_sedecimpunctata_589 | 0.144 |
| Chilocorus_nigritus_28 | Tytthaspis_sedecimpunctata_593 | 0.144 |
| Calvia_quatuordecimguttata_201 | Tytthaspis_sedecimpunctata_593 | 0.144 |
| Calvia_quatuordecimguttata_202 | Tytthaspis_sedecimpunctata_593 | 0.144 |
| Calvia_quatuordecimguttata_210 | Tytthaspis_sedecimpunctata_593 | 0.144 |
| Scymnus_caurinus_720 | Scymnus_suturalis_833 | 0.144 |
| Scymnus_caurinus_721 | Scymnus_suturalis_833 | 0.144 |
| Scymnus_caurinus_722 | Scymnus_suturalis_833 | 0.144 |
| Scymnus_caurinus_720 | Scymnus_suturalis_838 | 0.144 |
| Scymnus_caurinus_721 | Scymnus_suturalis_838 | 0.144 |
| Scymnus_caurinus_722 | Scymnus_suturalis_838 | 0.144 |
| Scymnus_caurinus_720 | Scymnus_suturalis_840 | 0.144 |
| Scymnus_caurinus_721 | Scymnus_suturalis_840 | 0.144 |
| Scymnus_caurinus_722 | Scymnus_suturalis_840 | 0.144 |
| Chilocorus_nigritus_28 | Tytthaspis_sedecimpunctata_591 | 0.144 |
| Scymnus_apetzi_690 | Scymnus_rubromaculatus_822 | 0.144 |
| Scymnus_apetzi_697 | Scymnus_rubromaculatus_822 | 0.144 |
| Scymnus_apetzi_690 | Scymnus_rubromaculatus_823 | 0.144 |
| Scymnus_apetzi_697 | Scymnus_rubromaculatus_823 | 0.144 |
| Scymnus_apetzi_690 | Scymnus_rubromaculatus_824 | 0.144 |
| Scymnus_apetzi_697 | Scymnus_rubromaculatus_824 | 0.144 |
| Scymnus_apetzi_690 | Scymnus_rubromaculatus_825 | 0.144 |

| | | |
|---|---|---|
| Scymnus_apetzi_697 | Scymnus_rubromaculatus_825 | 0.144 |
| Scymnus_apetzi_690 | Scymnus_rubromaculatus_826 | 0.144 |
| Scymnus_apetzi_697 | Scymnus_rubromaculatus_826 | 0.144 |
| Scymnus_apetzi_690 | Scymnus_rubromaculatus_827 | 0.144 |
| Scymnus_apetzi_697 | Scymnus_rubromaculatus_827 | 0.144 |
| Scymnus_caurinus_720 | Scymnus_suturalis_834 | 0.144 |
| Scymnus_caurinus_721 | Scymnus_suturalis_834 | 0.144 |
| Scymnus_caurinus_722 | Scymnus_suturalis_834 | 0.144 |
| Scymnus_nebulosus_781 | Scymnus_sinuanodulus_830 | 0.144 |
| Scymnus_auritus_703 | Scymnus_suturalis_843 | 0.144 |
| Scymnus_camptodromus_704 | Scymnus_suturalis_843 | 0.144 |
| Scymnus_sinuanodulus_830 | Scymnus_suturalis_843 | 0.144 |
| Scymnus_auritus_703 | Scymnus_suturalis_845 | 0.144 |
| Scymnus_sinuanodulus_830 | Scymnus_suturalis_845 | 0.144 |
| Scymnus_caurinus_720 | Scymnus_sinuanodulus_828 | 0.144 |
| Scymnus_caurinus_721 | Scymnus_sinuanodulus_828 | 0.144 |
| Scymnus_caurinus_722 | Scymnus_sinuanodulus_828 | 0.144 |
| Scymnus_camptodromus_704 | Scymnus_suturalis_834 | 0.144 |
| Scymnus_sinuanodulus_830 | Scymnus_suturalis_834 | 0.144 |
| Scymnus_auritus_702 | Scymnus_suturalis_838 | 0.144 |
| Scymnus_auritus_702 | Scymnus_suturalis_840 | 0.144 |
| Anisosticta_novemdecimpunctata_160 | Tytthaspis_sedecimpunctata_577 | 0.145 |
| Anisosticta_novemdecimpunctata_161 | Tytthaspis_sedecimpunctata_577 | 0.145 |
| Anisosticta_novemdecimpunctata_160 | Tytthaspis_sedecimpunctata_578 | 0.145 |
| Anisosticta_novemdecimpunctata_161 | Tytthaspis_sedecimpunctata_578 | 0.145 |
| Anisosticta_novemdecimpunctata_160 | Tytthaspis_sedecimpunctata_579 | 0.145 |
| Anisosticta_novemdecimpunctata_161 | Tytthaspis_sedecimpunctata_579 | 0.145 |
| Anisosticta_novemdecimpunctata_160 | Tytthaspis_sedecimpunctata_581 | 0.145 |
| Anisosticta_novemdecimpunctata_161 | Tytthaspis_sedecimpunctata_581 | 0.145 |
| Anisosticta_novemdecimpunctata_160 | Tytthaspis_sedecimpunctata_582 | 0.145 |
| Anisosticta_novemdecimpunctata_161 | Tytthaspis_sedecimpunctata_582 | 0.145 |
| Anisosticta_novemdecimpunctata_160 | Tytthaspis_sedecimpunctata_590 | 0.145 |
| Anisosticta_novemdecimpunctata_161 | Tytthaspis_sedecimpunctata_590 | 0.145 |
| Anisosticta_novemdecimpunctata_160 | Tytthaspis_sedecimpunctata_593 | 0.145 |
| Anisosticta_novemdecimpunctata_161 | Tytthaspis_sedecimpunctata_593 | 0.145 |
| Anisosticta_novemdecimpunctata_160 | Tytthaspis_sedecimpunctata_594 | 0.145 |
| Anisosticta_novemdecimpunctata_161 | Tytthaspis_sedecimpunctata_594 | 0.145 |
| Anisosticta_novemdecimpunctata_160 | Tytthaspis_sedecimpunctata_595 | 0.145 |
| Anisosticta_novemdecimpunctata_161 | Tytthaspis_sedecimpunctata_595 | 0.145 |
| Scymnus_auritus_702 | Scymnus_suturalis_834 | 0.145 |
| Scymnus_sinuanodulus_830 | Scymnus_suturalis_838 | 0.145 |
| Scymnus_nebulosus_781 | Scymnus_sinuanodulus_831 | 0.145 |
| Anisosticta_novemdecimpunctata_160 | Tytthaspis_sedecimpunctata_580 | 0.145 |
| Anisosticta_novemdecimpunctata_161 | Tytthaspis_sedecimpunctata_580 | 0.145 |
| Anisosticta_bitriangularis_153 | Tytthaspis_sedecimpunctata_589 | 0.145 |
| Anisosticta_bitriangularis_157 | Tytthaspis_sedecimpunctata_589 | 0.145 |
| Scymnus_loewii_772 | Scymnus_sinuanodulus_830 | 0.145 |
| Scymnus_loewii_773 | Scymnus_sinuanodulus_830 | 0.145 |
| Scymnus_ningshanensis_789 | Scymnus_suturalis_838 | 0.145 |
| Scymnus_ningshanensis_790 | Scymnus_suturalis_838 | 0.145 |
| Scymnus_ningshanensis_789 | Scymnus_suturalis_840 | 0.145 |
| Scymnus_ningshanensis_790 | Scymnus_suturalis_840 | 0.145 |
| Scymnus_sinuanodulus_831 | Scymnus_suturalis_843 | 0.145 |
| Scymnus_sinuanodulus_831 | Scymnus_suturalis_845 | 0.145 |
| Scymnus_sinuanodulus_831 | Scymnus_suturalis_834 | 0.145 |
| Anisosticta_bitriangularis_151 | Tytthaspis_sedecimpunctata_586 | 0.145 |

| | | |
|---|---|---|
| Anisosticta_bitriangularis_154 | Tytthaspis_sedecimpunctata_586 | 0.145 |
| Anisosticta_bitriangularis_153 | Tytthaspis_sedecimpunctata_587 | 0.145 |
| Anisosticta_bitriangularis_157 | Tytthaspis_sedecimpunctata_587 | 0.145 |
| Anisosticta_bitriangularis_151 | Tytthaspis_sedecimpunctata_588 | 0.145 |
| Anisosticta_bitriangularis_154 | Tytthaspis_sedecimpunctata_588 | 0.145 |
| Anisosticta_bitriangularis_151 | Tytthaspis_sedecimpunctata_591 | 0.145 |
| Anisosticta_bitriangularis_154 | Tytthaspis_sedecimpunctata_591 | 0.145 |
| Scymnus_abietis_682 | Scymnus_sinuanodulus_828 | 0.145 |
| Scymnus_abietis_685 | Scymnus_sinuanodulus_828 | 0.145 |
| Scymnus_ningshanensis_789 | Scymnus_suturalis_834 | 0.145 |
| Scymnus_ningshanensis_790 | Scymnus_suturalis_834 | 0.145 |
| Scymnus_abietis_684 | Scymnus_sinuanodulus_831 | 0.145 |
| Scymnus_abietis_686 | Scymnus_sinuanodulus_831 | 0.145 |
| Scymnus_abietis_688 | Scymnus_sinuanodulus_831 | 0.145 |
| Scymnus_sinuanodulus_829 | Scymnus_suturalis_841 | 0.145 |
| Scymnus_sinuanodulus_829 | Scymnus_suturalis_842 | 0.145 |
| Scymnus_sinuanodulus_829 | Scymnus_suturalis_844 | 0.145 |
| Scymnus_sinuanodulus_829 | Scymnus_suturalis_846 | 0.145 |
| Scymnus_sinuanodulus_829 | Scymnus_suturalis_847 | 0.145 |
| Scymnus_sinuanodulus_829 | Scymnus_suturalis_848 | 0.145 |
| Scymnus_sinuanodulus_829 | Scymnus_suturalis_832 | 0.145 |
| Scymnus_sinuanodulus_829 | Scymnus_suturalis_835 | 0.145 |
| Scymnus_sinuanodulus_829 | Scymnus_suturalis_836 | 0.145 |
| Scymnus_sinuanodulus_829 | Scymnus_suturalis_837 | 0.145 |
| Scymnus_sinuanodulus_829 | Scymnus_suturalis_839 | 0.145 |
| Brumoides_suturalis_2 | Tytthaspis_sedecimpunctata_585 | 0.145 |
| Brumoides_suturalis_2 | Tytthaspis_sedecimpunctata_584 | 0.145 |
| Chilocorus_nigritus_26 | Tytthaspis_sedecimpunctata_589 | 0.145 |
| Brumoides_suturalis_1 | Tytthaspis_sedecimpunctata_586 | 0.145 |
| Mulsantina_hudsonica_479 | Tytthaspis_sedecimpunctata_583 | 0.145 |
| Chilocorus_nigritus_25 | Tytthaspis_sedecimpunctata_588 | 0.145 |
| Scymnus_interruptus_759 | Scymnus_suturalis_833 | 0.145 |
| Scymnus_interruptus_761 | Scymnus_suturalis_833 | 0.145 |
| Scymnus_interruptus_763 | Scymnus_suturalis_833 | 0.145 |
| Scymnus_interruptus_764 | Scymnus_suturalis_833 | 0.145 |
| Scymnus_interruptus_766 | Scymnus_suturalis_833 | 0.145 |
| Scymnus_interruptus_769 | Scymnus_suturalis_833 | 0.145 |
| Scymnus_interruptus_759 | Scymnus_suturalis_843 | 0.145 |
| Scymnus_interruptus_761 | Scymnus_suturalis_843 | 0.145 |
| Scymnus_interruptus_763 | Scymnus_suturalis_843 | 0.145 |
| Scymnus_interruptus_764 | Scymnus_suturalis_843 | 0.145 |
| Scymnus_interruptus_766 | Scymnus_suturalis_843 | 0.145 |
| Scymnus_interruptus_769 | Scymnus_suturalis_843 | 0.145 |
| Scymnus_interruptus_759 | Scymnus_suturalis_845 | 0.145 |
| Scymnus_interruptus_761 | Scymnus_suturalis_845 | 0.145 |
| Scymnus_interruptus_763 | Scymnus_suturalis_845 | 0.145 |
| Scymnus_interruptus_764 | Scymnus_suturalis_845 | 0.145 |
| Scymnus_interruptus_766 | Scymnus_suturalis_845 | 0.145 |
| Scymnus_interruptus_769 | Scymnus_suturalis_845 | 0.145 |
| Chilocorus_nigritus_25 | Tytthaspis_sedecimpunctata_577 | 0.145 |
| Chilocorus_nigritus_25 | Tytthaspis_sedecimpunctata_578 | 0.145 |
| Chilocorus_nigritus_25 | Tytthaspis_sedecimpunctata_579 | 0.145 |
| Mulsantina_hudsonica_479 | Tytthaspis_sedecimpunctata_580 | 0.145 |
| Chilocorus_nigritus_25 | Tytthaspis_sedecimpunctata_581 | 0.145 |
| Chilocorus_nigritus_25 | Tytthaspis_sedecimpunctata_582 | 0.145 |
| Chilocorus_nigritus_25 | Tytthaspis_sedecimpunctata_590 | 0.145 |

Supplementary Table S4 (continued)

| | | |
|---|---|---|
| Chilocorus_nigritus_25 | Tytthaspis_sedecimpunctata_592 | 0.145 |
| Chilocorus_nigritus_26 | Tytthaspis_sedecimpunctata_593 | 0.145 |
| Chilocorus_nigritus_25 | Tytthaspis_sedecimpunctata_594 | 0.145 |
| Chilocorus_nigritus_25 | Tytthaspis_sedecimpunctata_595 | 0.145 |
| Scymnus_interruptus_759 | Scymnus_suturalis_837 | 0.145 |
| Scymnus_interruptus_761 | Scymnus_suturalis_837 | 0.145 |
| Scymnus_interruptus_763 | Scymnus_suturalis_837 | 0.145 |
| Scymnus_interruptus_764 | Scymnus_suturalis_837 | 0.145 |
| Scymnus_interruptus_766 | Scymnus_suturalis_837 | 0.145 |
| Scymnus_interruptus_769 | Scymnus_suturalis_837 | 0.145 |
| Scymnus_interruptus_762 | Scymnus_suturalis_838 | 0.145 |
| Scymnus_interruptus_762 | Scymnus_suturalis_840 | 0.145 |
| Chilocorus_nigritus_25 | Tytthaspis_sedecimpunctata_584 | 0.146 |
| Chilocorus_nigritus_26 | Tytthaspis_sedecimpunctata_591 | 0.146 |
| Scymnus_interruptus_762 | Scymnus_suturalis_834 | 0.146 |
| Coelophora_inaequalis_310 | Tytthaspis_sedecimpunctata_585 | 0.146 |
| Coelophora_inaequalis_312 | Tytthaspis_sedecimpunctata_585 | 0.146 |
| Coelophora_inaequalis_314 | Tytthaspis_sedecimpunctata_585 | 0.146 |
| Coelophora_inaequalis_323 | Tytthaspis_sedecimpunctata_585 | 0.146 |
| Coelophora_inaequalis_325 | Tytthaspis_sedecimpunctata_585 | 0.146 |
| Exochomus_nigromaculatus_49 | Tytthaspis_sedecimpunctata_588 | 0.146 |
| Hippodamia_variegata_471 | Tytthaspis_sedecimpunctata_577 | 0.146 |
| Hippodamia_variegata_471 | Tytthaspis_sedecimpunctata_578 | 0.146 |
| Hippodamia_variegata_471 | Tytthaspis_sedecimpunctata_579 | 0.146 |
| Hippodamia_variegata_471 | Tytthaspis_sedecimpunctata_581 | 0.146 |
| Hippodamia_variegata_471 | Tytthaspis_sedecimpunctata_582 | 0.146 |
| Exochomus_nigromaculatus_49 | Tytthaspis_sedecimpunctata_585 | 0.146 |
| Hippodamia_variegata_467 | Tytthaspis_sedecimpunctata_585 | 0.146 |
| Hippodamia_variegata_469 | Tytthaspis_sedecimpunctata_585 | 0.146 |
| Hippodamia_variegata_470 | Tytthaspis_sedecimpunctata_585 | 0.146 |
| Hippodamia_variegata_472 | Tytthaspis_sedecimpunctata_585 | 0.146 |
| Hippodamia_variegata_473 | Tytthaspis_sedecimpunctata_585 | 0.146 |
| Exochomus_nigromaculatus_48 | Tytthaspis_sedecimpunctata_586 | 0.146 |
| Hippodamia_variegata_471 | Tytthaspis_sedecimpunctata_590 | 0.146 |
| Exochomus_nigromaculatus_49 | Tytthaspis_sedecimpunctata_592 | 0.146 |
| Hippodamia_variegata_467 | Tytthaspis_sedecimpunctata_592 | 0.146 |
| Hippodamia_variegata_469 | Tytthaspis_sedecimpunctata_592 | 0.146 |
| Hippodamia_variegata_470 | Tytthaspis_sedecimpunctata_592 | 0.146 |
| Hippodamia_variegata_472 | Tytthaspis_sedecimpunctata_592 | 0.146 |
| Hippodamia_variegata_473 | Tytthaspis_sedecimpunctata_592 | 0.146 |
| Hippodamia_variegata_471 | Tytthaspis_sedecimpunctata_594 | 0.146 |
| Hippodamia_variegata_471 | Tytthaspis_sedecimpunctata_595 | 0.146 |
| Scymnus_abietis_684 | Scymnus_sinuanodulus_829 | 0.146 |
| Scymnus_abietis_686 | Scymnus_sinuanodulus_829 | 0.146 |
| Scymnus_abietis_687 | Scymnus_sinuanodulus_829 | 0.146 |
| Scymnus_abietis_688 | Scymnus_sinuanodulus_829 | 0.146 |
| Exochomus_nigromaculatus_48 | Tytthaspis_sedecimpunctata_577 | 0.146 |
| Hippodamia_variegata_468 | Tytthaspis_sedecimpunctata_577 | 0.146 |
| Exochomus_nigromaculatus_48 | Tytthaspis_sedecimpunctata_578 | 0.146 |
| Hippodamia_variegata_468 | Tytthaspis_sedecimpunctata_578 | 0.146 |
| Exochomus_nigromaculatus_48 | Tytthaspis_sedecimpunctata_579 | 0.146 |
| Hippodamia_variegata_468 | Tytthaspis_sedecimpunctata_579 | 0.146 |
| Hippodamia_variegata_467 | Tytthaspis_sedecimpunctata_580 | 0.146 |
| Hippodamia_variegata_469 | Tytthaspis_sedecimpunctata_580 | 0.146 |
| Hippodamia_variegata_470 | Tytthaspis_sedecimpunctata_580 | 0.146 |
| Hippodamia_variegata_472 | Tytthaspis_sedecimpunctata_580 | 0.146 |

| | | |
|---|---|---|
| Hippodamia_variegata_473 | Tytthaspis_sedecimpunctata_580 | 0.146 |
| Exochomus_nigromaculatus_48 | Tytthaspis_sedecimpunctata_581 | 0.146 |
| Hippodamia_variegata_468 | Tytthaspis_sedecimpunctata_581 | 0.146 |
| Exochomus_nigromaculatus_48 | Tytthaspis_sedecimpunctata_582 | 0.146 |
| Hippodamia_variegata_468 | Tytthaspis_sedecimpunctata_582 | 0.146 |
| Exochomus_nigromaculatus_48 | Tytthaspis_sedecimpunctata_589 | 0.146 |
| Exochomus_nigromaculatus_48 | Tytthaspis_sedecimpunctata_590 | 0.146 |
| Hippodamia_variegata_468 | Tytthaspis_sedecimpunctata_590 | 0.146 |
| Exochomus_nigromaculatus_48 | Tytthaspis_sedecimpunctata_594 | 0.146 |
| Hippodamia_variegata_468 | Tytthaspis_sedecimpunctata_594 | 0.146 |
| Exochomus_nigromaculatus_48 | Tytthaspis_sedecimpunctata_595 | 0.146 |
| Hippodamia_variegata_468 | Tytthaspis_sedecimpunctata_595 | 0.146 |
| Exochomus_nigromaculatus_48 | Tytthaspis_sedecimpunctata_580 | 0.146 |
| Myrrha_octodecimguttata_488 | Tytthaspis_sedecimpunctata_588 | 0.146 |
| Myrrha_octodecimguttata_491 | Tytthaspis_sedecimpunctata_588 | 0.146 |
| Myrrha_octodecimguttata_492 | Tytthaspis_sedecimpunctata_588 | 0.146 |
| Calvia_quatuordecimguttata_211 | Tytthaspis_sedecimpunctata_589 | 0.146 |
| Calvia_quatuordecimguttata_212 | Tytthaspis_sedecimpunctata_589 | 0.146 |
| Calvia_quatuordecimguttata_213 | Tytthaspis_sedecimpunctata_589 | 0.146 |
| Calvia_quatuordecimguttata_214 | Tytthaspis_sedecimpunctata_589 | 0.146 |
| Calvia_quatuordecimguttata_215 | Tytthaspis_sedecimpunctata_589 | 0.146 |
| Chilocorus_nigritus_29 | Tytthaspis_sedecimpunctata_589 | 0.146 |
| Chilocorus_nigritus_30 | Tytthaspis_sedecimpunctata_589 | 0.146 |
| Anisosticta_bitriangularis_147 | Tytthaspis_sedecimpunctata_591 | 0.146 |
| Scymnus_nebulosus_781 | Scymnus_suturalis_843 | 0.146 |
| Calvia_quatuordecimguttata_211 | Tytthaspis_sedecimpunctata_588 | 0.146 |
| Calvia_quatuordecimguttata_212 | Tytthaspis_sedecimpunctata_588 | 0.146 |
| Calvia_quatuordecimguttata_213 | Tytthaspis_sedecimpunctata_588 | 0.146 |
| Calvia_quatuordecimguttata_214 | Tytthaspis_sedecimpunctata_588 | 0.146 |
| Calvia_quatuordecimguttata_215 | Tytthaspis_sedecimpunctata_588 | 0.146 |
| Calvia_quatuordecimguttata_211 | Tytthaspis_sedecimpunctata_591 | 0.146 |
| Calvia_quatuordecimguttata_212 | Tytthaspis_sedecimpunctata_591 | 0.146 |
| Calvia_quatuordecimguttata_213 | Tytthaspis_sedecimpunctata_591 | 0.146 |
| Calvia_quatuordecimguttata_214 | Tytthaspis_sedecimpunctata_591 | 0.146 |
| Calvia_quatuordecimguttata_215 | Tytthaspis_sedecimpunctata_591 | 0.146 |
| Scymnus_nebulosus_781 | Scymnus_suturalis_834 | 0.146 |
| Scymnus_ferrugatus_723 | Scymnus_suturalis_843 | 0.146 |
| Scymnus_ferrugatus_725 | Scymnus_suturalis_843 | 0.146 |
| Scymnus_ferrugatus_726 | Scymnus_suturalis_843 | 0.146 |
| Scymnus_ferrugatus_727 | Scymnus_suturalis_843 | 0.146 |
| Scymnus_ferrugatus_728 | Scymnus_suturalis_843 | 0.146 |
| Scymnus_ferrugatus_730 | Scymnus_suturalis_843 | 0.146 |
| Scymnus_ferrugatus_731 | Scymnus_suturalis_843 | 0.146 |
| Scymnus_ferrugatus_732 | Scymnus_suturalis_843 | 0.146 |
| Scymnus_ferrugatus_733 | Scymnus_suturalis_843 | 0.146 |
| Scymnus_ferrugatus_723 | Scymnus_suturalis_845 | 0.146 |
| Scymnus_ferrugatus_725 | Scymnus_suturalis_845 | 0.146 |
| Scymnus_ferrugatus_726 | Scymnus_suturalis_845 | 0.146 |
| Scymnus_ferrugatus_727 | Scymnus_suturalis_845 | 0.146 |
| Scymnus_ferrugatus_728 | Scymnus_suturalis_845 | 0.146 |
| Scymnus_ferrugatus_730 | Scymnus_suturalis_845 | 0.146 |
| Scymnus_ferrugatus_731 | Scymnus_suturalis_845 | 0.146 |
| Scymnus_ferrugatus_732 | Scymnus_suturalis_845 | 0.146 |
| Scymnus_ferrugatus_733 | Scymnus_suturalis_845 | 0.146 |
| Scymnus_sinuanodulus_828 | Scymnus_suturalis_833 | 0.146 |
| Scymnus_ferrugatus_723 | Scymnus_suturalis_834 | 0.146 |

| | | |
|---|---|---|
| Scymnus_ferrugatus_725 | Scymnus_suturalis_834 | 0.146 |
| Scymnus_ferrugatus_726 | Scymnus_suturalis_834 | 0.146 |
| Scymnus_ferrugatus_727 | Scymnus_suturalis_834 | 0.146 |
| Scymnus_ferrugatus_728 | Scymnus_suturalis_834 | 0.146 |
| Scymnus_ferrugatus_730 | Scymnus_suturalis_834 | 0.146 |
| Scymnus_ferrugatus_731 | Scymnus_suturalis_834 | 0.146 |
| Scymnus_ferrugatus_732 | Scymnus_suturalis_834 | 0.146 |
| Scymnus_ferrugatus_733 | Scymnus_suturalis_834 | 0.146 |
| Scymnus_auritus_703 | Scymnus_suturalis_833 | 0.146 |
| Scymnus_camptodromus_704 | Scymnus_suturalis_833 | 0.146 |
| Scymnus_ferrugatus_723 | Scymnus_suturalis_840 | 0.146 |
| Scymnus_ferrugatus_725 | Scymnus_suturalis_840 | 0.146 |
| Scymnus_ferrugatus_726 | Scymnus_suturalis_840 | 0.146 |
| Scymnus_ferrugatus_727 | Scymnus_suturalis_840 | 0.146 |
| Scymnus_ferrugatus_728 | Scymnus_suturalis_840 | 0.146 |
| Scymnus_ferrugatus_730 | Scymnus_suturalis_840 | 0.146 |
| Scymnus_ferrugatus_731 | Scymnus_suturalis_840 | 0.146 |
| Scymnus_ferrugatus_732 | Scymnus_suturalis_840 | 0.146 |
| Scymnus_ferrugatus_733 | Scymnus_suturalis_840 | 0.146 |
| Coelophora_inaequalis_319 | Tytthaspis_sedecimpunctata_588 | 0.146 |
| Coelophora_inaequalis_319 | Tytthaspis_sedecimpunctata_585 | 0.146 |
| Scymnus_auritus_703 | Scymnus_suturalis_841 | 0.146 |
| Scymnus_camptodromus_704 | Scymnus_suturalis_841 | 0.146 |
| Scymnus_sinuanodulus_830 | Scymnus_suturalis_841 | 0.146 |
| Scymnus_auritus_703 | Scymnus_suturalis_842 | 0.146 |
| Scymnus_camptodromus_704 | Scymnus_suturalis_842 | 0.146 |
| Scymnus_sinuanodulus_830 | Scymnus_suturalis_842 | 0.146 |
| Scymnus_camptodromus_714 | Scymnus_suturalis_843 | 0.146 |
| Scymnus_auritus_703 | Scymnus_suturalis_844 | 0.146 |
| Scymnus_camptodromus_704 | Scymnus_suturalis_844 | 0.146 |
| Scymnus_sinuanodulus_830 | Scymnus_suturalis_844 | 0.146 |
| Scymnus_auritus_703 | Scymnus_suturalis_846 | 0.146 |
| Scymnus_camptodromus_704 | Scymnus_suturalis_846 | 0.146 |
| Scymnus_sinuanodulus_830 | Scymnus_suturalis_846 | 0.146 |
| Scymnus_auritus_703 | Scymnus_suturalis_847 | 0.146 |
| Scymnus_camptodromus_704 | Scymnus_suturalis_847 | 0.146 |
| Scymnus_sinuanodulus_830 | Scymnus_suturalis_847 | 0.146 |
| Scymnus_auritus_703 | Scymnus_suturalis_848 | 0.146 |
| Scymnus_camptodromus_704 | Scymnus_suturalis_848 | 0.146 |
| Scymnus_sinuanodulus_830 | Scymnus_suturalis_848 | 0.146 |
| Anisosticta_novemdecimpunctata_160 | Tytthaspis_sedecimpunctata_584 | 0.146 |
| Anisosticta_novemdecimpunctata_161 | Tytthaspis_sedecimpunctata_584 | 0.146 |
| Anisosticta_novemdecimpunctata_160 | Tytthaspis_sedecimpunctata_586 | 0.146 |
| Anisosticta_novemdecimpunctata_161 | Tytthaspis_sedecimpunctata_586 | 0.146 |
| Anisosticta_novemdecimpunctata_165 | Tytthaspis_sedecimpunctata_586 | 0.146 |
| Anisosticta_novemdecimpunctata_173 | Tytthaspis_sedecimpunctata_586 | 0.146 |
| Anisosticta_novemdecimpunctata_160 | Tytthaspis_sedecimpunctata_588 | 0.146 |
| Anisosticta_novemdecimpunctata_161 | Tytthaspis_sedecimpunctata_588 | 0.146 |
| Anisosticta_novemdecimpunctata_165 | Tytthaspis_sedecimpunctata_588 | 0.146 |
| Anisosticta_novemdecimpunctata_173 | Tytthaspis_sedecimpunctata_588 | 0.146 |
| Scymnus_nigrinus_785 | Scymnus_rubromaculatus_822 | 0.146 |
| Scymnus_nigrinus_786 | Scymnus_rubromaculatus_822 | 0.146 |
| Scymnus_nigrinus_785 | Scymnus_rubromaculatus_823 | 0.146 |
| Scymnus_nigrinus_786 | Scymnus_rubromaculatus_823 | 0.146 |
| Scymnus_nigrinus_785 | Scymnus_rubromaculatus_824 | 0.146 |
| Scymnus_nigrinus_786 | Scymnus_rubromaculatus_824 | 0.146 |

| | | |
|---|---|---|
| Scymnus_nigrinus_785 | Scymnus_rubromaculatus_825 | 0.146 |
| Scymnus_nigrinus_786 | Scymnus_rubromaculatus_825 | 0.146 |
| Scymnus_nigrinus_785 | Scymnus_rubromaculatus_826 | 0.146 |
| Scymnus_nigrinus_786 | Scymnus_rubromaculatus_826 | 0.146 |
| Scymnus_nigrinus_785 | Scymnus_rubromaculatus_827 | 0.146 |
| Scymnus_nigrinus_786 | Scymnus_rubromaculatus_827 | 0.146 |
| Scymnus_auritus_703 | Scymnus_suturalis_832 | 0.146 |
| Scymnus_camptodromus_704 | Scymnus_suturalis_832 | 0.146 |
| Scymnus_sinuanodulus_830 | Scymnus_suturalis_832 | 0.146 |
| Scymnus_camptodromus_714 | Scymnus_suturalis_834 | 0.146 |
| Scymnus_auritus_703 | Scymnus_suturalis_835 | 0.146 |
| Scymnus_camptodromus_704 | Scymnus_suturalis_835 | 0.146 |
| Scymnus_sinuanodulus_830 | Scymnus_suturalis_835 | 0.146 |
| Scymnus_auritus_703 | Scymnus_suturalis_836 | 0.146 |
| Scymnus_camptodromus_704 | Scymnus_suturalis_836 | 0.146 |
| Scymnus_sinuanodulus_830 | Scymnus_suturalis_836 | 0.146 |
| Scymnus_auritus_703 | Scymnus_suturalis_837 | 0.146 |
| Scymnus_camptodromus_704 | Scymnus_suturalis_837 | 0.146 |
| Scymnus_sinuanodulus_830 | Scymnus_suturalis_837 | 0.146 |
| Scymnus_auritus_703 | Scymnus_suturalis_839 | 0.146 |
| Scymnus_camptodromus_704 | Scymnus_suturalis_839 | 0.146 |
| Scymnus_sinuanodulus_830 | Scymnus_suturalis_839 | 0.146 |
| Anisosticta_novemdecimpunctata_160 | Tytthaspis_sedecimpunctata_583 | 0.146 |
| Anisosticta_novemdecimpunctata_161 | Tytthaspis_sedecimpunctata_583 | 0.146 |
| Anisosticta_novemdecimpunctata_165 | Tytthaspis_sedecimpunctata_585 | 0.146 |
| Anisosticta_novemdecimpunctata_173 | Tytthaspis_sedecimpunctata_585 | 0.146 |
| Anisosticta_novemdecimpunctata_165 | Tytthaspis_sedecimpunctata_592 | 0.146 |
| Anisosticta_novemdecimpunctata_173 | Tytthaspis_sedecimpunctata_592 | 0.146 |
| Anisosticta_bitriangularis_158 | Tytthaspis_sedecimpunctata_589 | 0.147 |
| Scymnus_sinuanodulus_831 | Scymnus_suturalis_841 | 0.147 |
| Scymnus_sinuanodulus_831 | Scymnus_suturalis_842 | 0.147 |
| Scymnus_sinuanodulus_831 | Scymnus_suturalis_844 | 0.147 |
| Scymnus_sinuanodulus_831 | Scymnus_suturalis_846 | 0.147 |
| Scymnus_sinuanodulus_831 | Scymnus_suturalis_847 | 0.147 |
| Scymnus_sinuanodulus_831 | Scymnus_suturalis_848 | 0.147 |
| Scymnus_sinuanodulus_831 | Scymnus_suturalis_832 | 0.147 |
| Scymnus_sinuanodulus_831 | Scymnus_suturalis_835 | 0.147 |
| Scymnus_sinuanodulus_831 | Scymnus_suturalis_836 | 0.147 |
| Scymnus_sinuanodulus_831 | Scymnus_suturalis_837 | 0.147 |
| Scymnus_sinuanodulus_831 | Scymnus_suturalis_839 | 0.147 |
| Anisosticta_bitriangularis_156 | Tytthaspis_sedecimpunctata_586 | 0.147 |
| Anisosticta_bitriangularis_158 | Tytthaspis_sedecimpunctata_587 | 0.147 |
| Anisosticta_bitriangularis_156 | Tytthaspis_sedecimpunctata_588 | 0.147 |
| Anisosticta_bitriangularis_156 | Tytthaspis_sedecimpunctata_591 | 0.147 |
| Scymnus_abietis_684 | Scymnus_sinuanodulus_828 | 0.147 |
| Scymnus_abietis_686 | Scymnus_sinuanodulus_828 | 0.147 |
| Scymnus_abietis_688 | Scymnus_sinuanodulus_828 | 0.147 |
| Anisosticta_bitriangularis_149 | Tytthaspis_sedecimpunctata_589 | 0.147 |
| Anisosticta_bitriangularis_149 | Tytthaspis_sedecimpunctata_587 | 0.147 |
| Scymnus_abietis_687 | Scymnus_sinuanodulus_831 | 0.147 |
| Scymnus_sinuanodulus_829 | Scymnus_suturalis_840 | 0.147 |
| Brumoides_suturalis_2 | Tytthaspis_sedecimpunctata_591 | 0.147 |
| Brumoides_suturalis_2 | Tytthaspis_sedecimpunctata_593 | 0.147 |
| Brumoides_suturalis_1 | Tytthaspis_sedecimpunctata_587 | 0.147 |
| Brumoides_suturalis_1 | Tytthaspis_sedecimpunctata_591 | 0.147 |
| Harmonia_conformis_365 | Tytthaspis_sedecimpunctata_580 | 0.147 |

| | | |
|---|---|---|
| Harmonia_conformis_366 | Tytthaspis_sedecimpunctata_580 | 0.147 |
| Harmonia_conformis_367 | Tytthaspis_sedecimpunctata_580 | 0.147 |
| Harmonia_conformis_368 | Tytthaspis_sedecimpunctata_580 | 0.147 |
| Harmonia_conformis_369 | Tytthaspis_sedecimpunctata_580 | 0.147 |
| Harmonia_conformis_370 | Tytthaspis_sedecimpunctata_580 | 0.147 |
| Harmonia_conformis_371 | Tytthaspis_sedecimpunctata_580 | 0.147 |
| Harmonia_conformis_372 | Tytthaspis_sedecimpunctata_580 | 0.147 |
| Harmonia_conformis_373 | Tytthaspis_sedecimpunctata_580 | 0.147 |
| Harmonia_conformis_375 | Tytthaspis_sedecimpunctata_580 | 0.147 |
| Brumoides_suturalis_1 | Tytthaspis_sedecimpunctata_589 | 0.147 |
| Mulsantina_hudsonica_479 | Tytthaspis_sedecimpunctata_577 | 0.147 |
| Mulsantina_hudsonica_479 | Tytthaspis_sedecimpunctata_578 | 0.147 |
| Mulsantina_hudsonica_479 | Tytthaspis_sedecimpunctata_579 | 0.147 |
| Mulsantina_hudsonica_479 | Tytthaspis_sedecimpunctata_581 | 0.147 |
| Mulsantina_hudsonica_479 | Tytthaspis_sedecimpunctata_582 | 0.147 |
| Mulsantina_hudsonica_481 | Tytthaspis_sedecimpunctata_583 | 0.147 |
| Mulsantina_hudsonica_482 | Tytthaspis_sedecimpunctata_583 | 0.147 |
| Chilocorus_nigritus_25 | Tytthaspis_sedecimpunctata_586 | 0.147 |
| Chilocorus_nigritus_25 | Tytthaspis_sedecimpunctata_587 | 0.147 |
| Mulsantina_hudsonica_479 | Tytthaspis_sedecimpunctata_589 | 0.147 |
| Mulsantina_hudsonica_479 | Tytthaspis_sedecimpunctata_590 | 0.147 |
| Mulsantina_hudsonica_479 | Tytthaspis_sedecimpunctata_592 | 0.147 |
| Mulsantina_hudsonica_479 | Tytthaspis_sedecimpunctata_593 | 0.147 |
| Mulsantina_hudsonica_479 | Tytthaspis_sedecimpunctata_594 | 0.147 |
| Mulsantina_hudsonica_479 | Tytthaspis_sedecimpunctata_595 | 0.147 |
| Scymnus_interruptus_759 | Scymnus_suturalis_841 | 0.147 |
| Scymnus_interruptus_761 | Scymnus_suturalis_841 | 0.147 |
| Scymnus_interruptus_763 | Scymnus_suturalis_841 | 0.147 |
| Scymnus_interruptus_764 | Scymnus_suturalis_841 | 0.147 |
| Scymnus_interruptus_766 | Scymnus_suturalis_841 | 0.147 |
| Scymnus_interruptus_769 | Scymnus_suturalis_841 | 0.147 |
| Scymnus_interruptus_759 | Scymnus_suturalis_842 | 0.147 |
| Scymnus_interruptus_761 | Scymnus_suturalis_842 | 0.147 |
| Scymnus_interruptus_763 | Scymnus_suturalis_842 | 0.147 |
| Scymnus_interruptus_764 | Scymnus_suturalis_842 | 0.147 |
| Scymnus_interruptus_766 | Scymnus_suturalis_842 | 0.147 |
| Scymnus_interruptus_769 | Scymnus_suturalis_842 | 0.147 |
| Scymnus_interruptus_759 | Scymnus_suturalis_844 | 0.147 |
| Scymnus_interruptus_761 | Scymnus_suturalis_844 | 0.147 |
| Scymnus_interruptus_763 | Scymnus_suturalis_844 | 0.147 |
| Scymnus_interruptus_764 | Scymnus_suturalis_844 | 0.147 |
| Scymnus_interruptus_766 | Scymnus_suturalis_844 | 0.147 |
| Scymnus_interruptus_769 | Scymnus_suturalis_844 | 0.147 |
| Scymnus_interruptus_759 | Scymnus_suturalis_846 | 0.147 |
| Scymnus_interruptus_761 | Scymnus_suturalis_846 | 0.147 |
| Scymnus_interruptus_763 | Scymnus_suturalis_846 | 0.147 |
| Scymnus_interruptus_764 | Scymnus_suturalis_846 | 0.147 |
| Scymnus_interruptus_766 | Scymnus_suturalis_846 | 0.147 |
| Scymnus_interruptus_769 | Scymnus_suturalis_846 | 0.147 |
| Scymnus_interruptus_759 | Scymnus_suturalis_847 | 0.147 |
| Scymnus_interruptus_761 | Scymnus_suturalis_847 | 0.147 |
| Scymnus_interruptus_763 | Scymnus_suturalis_847 | 0.147 |
| Scymnus_interruptus_764 | Scymnus_suturalis_847 | 0.147 |
| Scymnus_interruptus_766 | Scymnus_suturalis_847 | 0.147 |
| Scymnus_interruptus_769 | Scymnus_suturalis_847 | 0.147 |
| Scymnus_interruptus_759 | Scymnus_suturalis_848 | 0.147 |

| | | |
|---|---|---|
| Scymnus_interruptus_761 | Scymnus_suturalis_848 | 0.147 |
| Scymnus_interruptus_763 | Scymnus_suturalis_848 | 0.147 |
| Scymnus_interruptus_764 | Scymnus_suturalis_848 | 0.147 |
| Scymnus_interruptus_766 | Scymnus_suturalis_848 | 0.147 |
| Scymnus_interruptus_769 | Scymnus_suturalis_848 | 0.147 |
| Mulsantina_hudsonica_481 | Tytthaspis_sedecimpunctata_580 | 0.147 |
| Mulsantina_hudsonica_482 | Tytthaspis_sedecimpunctata_580 | 0.147 |
| Chilocorus_nigritus_25 | Tytthaspis_sedecimpunctata_583 | 0.147 |
| Scymnus_interruptus_759 | Scymnus_suturalis_832 | 0.147 |
| Scymnus_interruptus_761 | Scymnus_suturalis_832 | 0.147 |
| Scymnus_interruptus_763 | Scymnus_suturalis_832 | 0.147 |
| Scymnus_interruptus_764 | Scymnus_suturalis_832 | 0.147 |
| Scymnus_interruptus_766 | Scymnus_suturalis_832 | 0.147 |
| Scymnus_interruptus_769 | Scymnus_suturalis_832 | 0.147 |
| Scymnus_interruptus_759 | Scymnus_suturalis_835 | 0.147 |
| Scymnus_interruptus_761 | Scymnus_suturalis_835 | 0.147 |
| Scymnus_interruptus_763 | Scymnus_suturalis_835 | 0.147 |
| Scymnus_interruptus_764 | Scymnus_suturalis_835 | 0.147 |
| Scymnus_interruptus_766 | Scymnus_suturalis_835 | 0.147 |
| Scymnus_interruptus_769 | Scymnus_suturalis_835 | 0.147 |
| Scymnus_interruptus_759 | Scymnus_suturalis_836 | 0.147 |
| Scymnus_interruptus_761 | Scymnus_suturalis_836 | 0.147 |
| Scymnus_interruptus_763 | Scymnus_suturalis_836 | 0.147 |
| Scymnus_interruptus_764 | Scymnus_suturalis_836 | 0.147 |
| Scymnus_interruptus_766 | Scymnus_suturalis_836 | 0.147 |
| Scymnus_interruptus_769 | Scymnus_suturalis_836 | 0.147 |
| Scymnus_interruptus_759 | Scymnus_suturalis_839 | 0.147 |
| Scymnus_interruptus_761 | Scymnus_suturalis_839 | 0.147 |
| Scymnus_interruptus_763 | Scymnus_suturalis_839 | 0.147 |
| Scymnus_interruptus_764 | Scymnus_suturalis_839 | 0.147 |
| Scymnus_interruptus_766 | Scymnus_suturalis_839 | 0.147 |
| Scymnus_interruptus_769 | Scymnus_suturalis_839 | 0.147 |
| Coelophora_inaequalis_310 | Tytthaspis_sedecimpunctata_588 | 0.147 |
| Coelophora_inaequalis_312 | Tytthaspis_sedecimpunctata_588 | 0.147 |
| Coelophora_inaequalis_314 | Tytthaspis_sedecimpunctata_588 | 0.147 |
| Coelophora_inaequalis_323 | Tytthaspis_sedecimpunctata_588 | 0.147 |
| Coelophora_inaequalis_325 | Tytthaspis_sedecimpunctata_588 | 0.147 |
| Hippodamia_variegata_471 | Tytthaspis_sedecimpunctata_589 | 0.147 |
| Coelophora_inaequalis_310 | Tytthaspis_sedecimpunctata_583 | 0.147 |
| Coelophora_inaequalis_312 | Tytthaspis_sedecimpunctata_583 | 0.147 |
| Coelophora_inaequalis_314 | Tytthaspis_sedecimpunctata_583 | 0.147 |
| Coelophora_inaequalis_323 | Tytthaspis_sedecimpunctata_583 | 0.147 |
| Coelophora_inaequalis_325 | Tytthaspis_sedecimpunctata_583 | 0.147 |
| Coelophora_inaequalis_311 | Tytthaspis_sedecimpunctata_585 | 0.147 |
| Coelophora_inaequalis_313 | Tytthaspis_sedecimpunctata_585 | 0.147 |
| Coelophora_inaequalis_316 | Tytthaspis_sedecimpunctata_585 | 0.147 |
| Coelophora_inaequalis_324 | Tytthaspis_sedecimpunctata_585 | 0.147 |
| Coelophora_inaequalis_326 | Tytthaspis_sedecimpunctata_585 | 0.147 |
| Exochomus_nigromaculatus_49 | Tytthaspis_sedecimpunctata_586 | 0.147 |
| Hippodamia_variegata_471 | Tytthaspis_sedecimpunctata_588 | 0.147 |
| Hippodamia_variegata_468 | Tytthaspis_sedecimpunctata_589 | 0.147 |
| Coelophora_inaequalis_310 | Tytthaspis_sedecimpunctata_592 | 0.147 |
| Coelophora_inaequalis_312 | Tytthaspis_sedecimpunctata_592 | 0.147 |
| Coelophora_inaequalis_314 | Tytthaspis_sedecimpunctata_592 | 0.147 |
| Coelophora_inaequalis_323 | Tytthaspis_sedecimpunctata_592 | 0.147 |
| Coelophora_inaequalis_325 | Tytthaspis_sedecimpunctata_592 | 0.147 |

| | | |
|---|---|---|
| Exochomus_nigromaculatus_49 | Tytthaspis_sedecimpunctata_577 | 0.147 |
| Hippodamia_variegata_467 | Tytthaspis_sedecimpunctata_577 | 0.147 |
| Hippodamia_variegata_469 | Tytthaspis_sedecimpunctata_577 | 0.147 |
| Hippodamia_variegata_470 | Tytthaspis_sedecimpunctata_577 | 0.147 |
| Hippodamia_variegata_472 | Tytthaspis_sedecimpunctata_577 | 0.147 |
| Hippodamia_variegata_473 | Tytthaspis_sedecimpunctata_577 | 0.147 |
| Exochomus_nigromaculatus_49 | Tytthaspis_sedecimpunctata_578 | 0.147 |
| Hippodamia_variegata_467 | Tytthaspis_sedecimpunctata_578 | 0.147 |
| Hippodamia_variegata_469 | Tytthaspis_sedecimpunctata_578 | 0.147 |
| Hippodamia_variegata_470 | Tytthaspis_sedecimpunctata_578 | 0.147 |
| Hippodamia_variegata_472 | Tytthaspis_sedecimpunctata_578 | 0.147 |
| Hippodamia_variegata_473 | Tytthaspis_sedecimpunctata_578 | 0.147 |
| Exochomus_nigromaculatus_49 | Tytthaspis_sedecimpunctata_579 | 0.147 |
| Hippodamia_variegata_467 | Tytthaspis_sedecimpunctata_579 | 0.147 |
| Hippodamia_variegata_469 | Tytthaspis_sedecimpunctata_579 | 0.147 |
| Hippodamia_variegata_470 | Tytthaspis_sedecimpunctata_579 | 0.147 |
| Hippodamia_variegata_472 | Tytthaspis_sedecimpunctata_579 | 0.147 |
| Hippodamia_variegata_473 | Tytthaspis_sedecimpunctata_579 | 0.147 |
| Coelophora_inaequalis_310 | Tytthaspis_sedecimpunctata_580 | 0.147 |
| Coelophora_inaequalis_312 | Tytthaspis_sedecimpunctata_580 | 0.147 |
| Coelophora_inaequalis_323 | Tytthaspis_sedecimpunctata_580 | 0.147 |
| Coelophora_inaequalis_325 | Tytthaspis_sedecimpunctata_580 | 0.147 |
| Exochomus_nigromaculatus_49 | Tytthaspis_sedecimpunctata_581 | 0.147 |
| Hippodamia_variegata_467 | Tytthaspis_sedecimpunctata_581 | 0.147 |
| Hippodamia_variegata_469 | Tytthaspis_sedecimpunctata_581 | 0.147 |
| Hippodamia_variegata_470 | Tytthaspis_sedecimpunctata_581 | 0.147 |
| Hippodamia_variegata_472 | Tytthaspis_sedecimpunctata_581 | 0.147 |
| Hippodamia_variegata_473 | Tytthaspis_sedecimpunctata_581 | 0.147 |
| Exochomus_nigromaculatus_49 | Tytthaspis_sedecimpunctata_582 | 0.147 |
| Hippodamia_variegata_467 | Tytthaspis_sedecimpunctata_582 | 0.147 |
| Hippodamia_variegata_469 | Tytthaspis_sedecimpunctata_582 | 0.147 |
| Hippodamia_variegata_470 | Tytthaspis_sedecimpunctata_582 | 0.147 |
| Hippodamia_variegata_472 | Tytthaspis_sedecimpunctata_582 | 0.147 |
| Hippodamia_variegata_473 | Tytthaspis_sedecimpunctata_582 | 0.147 |
| Hippodamia_variegata_471 | Tytthaspis_sedecimpunctata_583 | 0.147 |
| Coelophora_inaequalis_315 | Tytthaspis_sedecimpunctata_585 | 0.147 |
| Exochomus_nigromaculatus_48 | Tytthaspis_sedecimpunctata_587 | 0.147 |
| Hippodamia_variegata_468 | Tytthaspis_sedecimpunctata_588 | 0.147 |
| Exochomus_nigromaculatus_49 | Tytthaspis_sedecimpunctata_589 | 0.147 |
| Exochomus_nigromaculatus_49 | Tytthaspis_sedecimpunctata_590 | 0.147 |
| Hippodamia_variegata_467 | Tytthaspis_sedecimpunctata_590 | 0.147 |
| Hippodamia_variegata_469 | Tytthaspis_sedecimpunctata_590 | 0.147 |
| Hippodamia_variegata_470 | Tytthaspis_sedecimpunctata_590 | 0.147 |
| Hippodamia_variegata_472 | Tytthaspis_sedecimpunctata_590 | 0.147 |
| Hippodamia_variegata_473 | Tytthaspis_sedecimpunctata_590 | 0.147 |
| Exochomus_nigromaculatus_49 | Tytthaspis_sedecimpunctata_594 | 0.147 |
| Hippodamia_variegata_467 | Tytthaspis_sedecimpunctata_594 | 0.147 |
| Hippodamia_variegata_469 | Tytthaspis_sedecimpunctata_594 | 0.147 |
| Hippodamia_variegata_470 | Tytthaspis_sedecimpunctata_594 | 0.147 |
| Hippodamia_variegata_472 | Tytthaspis_sedecimpunctata_594 | 0.147 |
| Hippodamia_variegata_473 | Tytthaspis_sedecimpunctata_594 | 0.147 |
| Exochomus_nigromaculatus_49 | Tytthaspis_sedecimpunctata_595 | 0.147 |
| Hippodamia_variegata_467 | Tytthaspis_sedecimpunctata_595 | 0.147 |
| Hippodamia_variegata_469 | Tytthaspis_sedecimpunctata_595 | 0.147 |
| Hippodamia_variegata_470 | Tytthaspis_sedecimpunctata_595 | 0.147 |
| Hippodamia_variegata_472 | Tytthaspis_sedecimpunctata_595 | 0.147 |

| | | |
|---|---|---|
| Hippodamia_variegata_473 | Tytthaspis_sedecimpunctata_595 | 0.147 |
| Exochomus_nigromaculatus_49 | Tytthaspis_sedecimpunctata_580 | 0.147 |
| Exochomus_nigromaculatus_48 | Tytthaspis_sedecimpunctata_583 | 0.147 |
| Hippodamia_variegata_468 | Tytthaspis_sedecimpunctata_583 | 0.147 |
| Myrrha_octodecimguttata_499 | Tytthaspis_sedecimpunctata_583 | 0.147 |
| Myrrha_octodecimguttata_488 | Tytthaspis_sedecimpunctata_589 | 0.147 |
| Myrrha_octodecimguttata_491 | Tytthaspis_sedecimpunctata_589 | 0.147 |
| Myrrha_octodecimguttata_492 | Tytthaspis_sedecimpunctata_589 | 0.147 |
| Myrrha_octodecimguttata_499 | Tytthaspis_sedecimpunctata_589 | 0.147 |
| Hippodamia_variegata_471 | Tytthaspis_sedecimpunctata_591 | 0.147 |
| Myrrha_octodecimguttata_499 | Tytthaspis_sedecimpunctata_592 | 0.147 |
| Exochomus_nigromaculatus_48 | Tytthaspis_sedecimpunctata_584 | 0.148 |
| Myrrha_octodecimguttata_488 | Tytthaspis_sedecimpunctata_586 | 0.148 |
| Myrrha_octodecimguttata_491 | Tytthaspis_sedecimpunctata_586 | 0.148 |
| Myrrha_octodecimguttata_492 | Tytthaspis_sedecimpunctata_586 | 0.148 |
| Myrrha_octodecimguttata_489 | Tytthaspis_sedecimpunctata_588 | 0.148 |
| Myrrha_octodecimguttata_499 | Tytthaspis_sedecimpunctata_588 | 0.148 |
| Myrrha_octodecimguttata_500 | Tytthaspis_sedecimpunctata_588 | 0.148 |
| Hippodamia_variegata_468 | Tytthaspis_sedecimpunctata_591 | 0.148 |
| Anisosticta_bitriangularis_147 | Tytthaspis_sedecimpunctata_589 | 0.148 |
| Myrrha_octodecimguttata_493 | Tytthaspis_sedecimpunctata_583 | 0.148 |
| Myrrha_octodecimguttata_495 | Tytthaspis_sedecimpunctata_583 | 0.148 |
| Myrrha_octodecimguttata_498 | Tytthaspis_sedecimpunctata_583 | 0.148 |
| Calvia_quatuordecimguttata_201 | Tytthaspis_sedecimpunctata_589 | 0.148 |
| Calvia_quatuordecimguttata_202 | Tytthaspis_sedecimpunctata_589 | 0.148 |
| Calvia_quatuordecimguttata_210 | Tytthaspis_sedecimpunctata_589 | 0.148 |
| Myrrha_octodecimguttata_493 | Tytthaspis_sedecimpunctata_589 | 0.148 |
| Myrrha_octodecimguttata_495 | Tytthaspis_sedecimpunctata_589 | 0.148 |
| Myrrha_octodecimguttata_498 | Tytthaspis_sedecimpunctata_589 | 0.148 |
| Myrrha_octodecimguttata_493 | Tytthaspis_sedecimpunctata_592 | 0.148 |
| Myrrha_octodecimguttata_495 | Tytthaspis_sedecimpunctata_592 | 0.148 |
| Myrrha_octodecimguttata_498 | Tytthaspis_sedecimpunctata_592 | 0.148 |
| Chilocorus_nigritus_29 | Tytthaspis_sedecimpunctata_587 | 0.148 |
| Chilocorus_nigritus_30 | Tytthaspis_sedecimpunctata_587 | 0.148 |
| Scymnus_nebulosus_781 | Scymnus_suturalis_841 | 0.148 |
| Scymnus_nebulosus_781 | Scymnus_suturalis_842 | 0.148 |
| Scymnus_nebulosus_781 | Scymnus_suturalis_844 | 0.148 |
| Scymnus_nebulosus_781 | Scymnus_suturalis_846 | 0.148 |
| Scymnus_nebulosus_781 | Scymnus_suturalis_847 | 0.148 |
| Scymnus_nebulosus_781 | Scymnus_suturalis_848 | 0.148 |
| Calvia_quatuordecimguttata_209 | Tytthaspis_sedecimpunctata_585 | 0.148 |
| Calvia_quatuordecimguttata_211 | Tytthaspis_sedecimpunctata_586 | 0.148 |
| Calvia_quatuordecimguttata_212 | Tytthaspis_sedecimpunctata_586 | 0.148 |
| Calvia_quatuordecimguttata_213 | Tytthaspis_sedecimpunctata_586 | 0.148 |
| Calvia_quatuordecimguttata_214 | Tytthaspis_sedecimpunctata_586 | 0.148 |
| Calvia_quatuordecimguttata_215 | Tytthaspis_sedecimpunctata_586 | 0.148 |
| Calvia_quatuordecimguttata_201 | Tytthaspis_sedecimpunctata_588 | 0.148 |
| Calvia_quatuordecimguttata_202 | Tytthaspis_sedecimpunctata_588 | 0.148 |
| Calvia_quatuordecimguttata_210 | Tytthaspis_sedecimpunctata_588 | 0.148 |
| Myrrha_octodecimguttata_493 | Tytthaspis_sedecimpunctata_588 | 0.148 |
| Myrrha_octodecimguttata_495 | Tytthaspis_sedecimpunctata_588 | 0.148 |
| Myrrha_octodecimguttata_498 | Tytthaspis_sedecimpunctata_588 | 0.148 |
| Calvia_quatuordecimguttata_201 | Tytthaspis_sedecimpunctata_591 | 0.148 |
| Calvia_quatuordecimguttata_202 | Tytthaspis_sedecimpunctata_591 | 0.148 |
| Calvia_quatuordecimguttata_210 | Tytthaspis_sedecimpunctata_591 | 0.148 |
| Scymnus_nebulosus_781 | Scymnus_suturalis_832 | 0.148 |

| | | |
|---|---|---|
| Scymnus_nebulosus_781 | Scymnus_suturalis_835 | 0.148 |
| Scymnus_nebulosus_781 | Scymnus_suturalis_836 | 0.148 |
| Scymnus_nebulosus_781 | Scymnus_suturalis_837 | 0.148 |
| Scymnus_nebulosus_781 | Scymnus_suturalis_839 | 0.148 |
| Anisosticta_bitriangularis_147 | Tytthaspis_sedecimpunctata_587 | 0.148 |
| Coelophora_inaequalis_318 | Tytthaspis_sedecimpunctata_585 | 0.148 |
| Calvia_quatuordecimguttata_209 | Tytthaspis_sedecimpunctata_580 | 0.148 |
| Calvia_quatuordecimguttata_206 | Tytthaspis_sedecimpunctata_584 | 0.148 |
| Calvia_quatuordecimguttata_209 | Tytthaspis_sedecimpunctata_584 | 0.148 |
| Calvia_quatuordecimguttata_216 | Tytthaspis_sedecimpunctata_584 | 0.148 |
| Coleomegilla_maculata_328 | Tytthaspis_sedecimpunctata_580 | 0.148 |
| Coleomegilla_maculata_332 | Tytthaspis_sedecimpunctata_580 | 0.148 |
| Scymnus_caurinus_720 | Scymnus_rubromaculatus_822 | 0.148 |
| Scymnus_caurinus_721 | Scymnus_rubromaculatus_822 | 0.148 |
| Scymnus_caurinus_722 | Scymnus_rubromaculatus_822 | 0.148 |
| Scymnus_caurinus_720 | Scymnus_rubromaculatus_823 | 0.148 |
| Scymnus_caurinus_721 | Scymnus_rubromaculatus_823 | 0.148 |
| Scymnus_caurinus_722 | Scymnus_rubromaculatus_823 | 0.148 |
| Scymnus_caurinus_720 | Scymnus_rubromaculatus_824 | 0.148 |
| Scymnus_caurinus_721 | Scymnus_rubromaculatus_824 | 0.148 |
| Scymnus_caurinus_722 | Scymnus_rubromaculatus_824 | 0.148 |
| Scymnus_caurinus_720 | Scymnus_rubromaculatus_825 | 0.148 |
| Scymnus_caurinus_721 | Scymnus_rubromaculatus_825 | 0.148 |
| Scymnus_caurinus_722 | Scymnus_rubromaculatus_825 | 0.148 |
| Scymnus_caurinus_720 | Scymnus_rubromaculatus_826 | 0.148 |
| Scymnus_caurinus_721 | Scymnus_rubromaculatus_826 | 0.148 |
| Scymnus_caurinus_722 | Scymnus_rubromaculatus_826 | 0.148 |
| Scymnus_caurinus_720 | Scymnus_rubromaculatus_827 | 0.148 |
| Scymnus_caurinus_721 | Scymnus_rubromaculatus_827 | 0.148 |
| Scymnus_caurinus_722 | Scymnus_rubromaculatus_827 | 0.148 |
| Scymnus_ferrugatus_724 | Scymnus_suturalis_843 | 0.148 |
| Scymnus_ferrugatus_729 | Scymnus_suturalis_843 | 0.148 |
| Scymnus_ferrugatus_724 | Scymnus_suturalis_845 | 0.148 |
| Scymnus_ferrugatus_729 | Scymnus_suturalis_845 | 0.148 |
| Scymnus_nebulosus_782 | Scymnus_rubromaculatus_822 | 0.148 |
| Scymnus_nebulosus_782 | Scymnus_rubromaculatus_823 | 0.148 |
| Scymnus_nebulosus_782 | Scymnus_rubromaculatus_824 | 0.148 |
| Scymnus_nebulosus_782 | Scymnus_rubromaculatus_825 | 0.148 |
| Scymnus_nebulosus_782 | Scymnus_rubromaculatus_826 | 0.148 |
| Scymnus_nebulosus_782 | Scymnus_rubromaculatus_827 | 0.148 |
| Scymnus_ferrugatus_724 | Scymnus_suturalis_834 | 0.148 |
| Scymnus_ferrugatus_729 | Scymnus_suturalis_834 | 0.148 |
| Scymnus_ferrugatus_723 | Scymnus_suturalis_841 | 0.148 |
| Scymnus_ferrugatus_725 | Scymnus_suturalis_841 | 0.148 |
| Scymnus_ferrugatus_726 | Scymnus_suturalis_841 | 0.148 |
| Scymnus_ferrugatus_727 | Scymnus_suturalis_841 | 0.148 |
| Scymnus_ferrugatus_728 | Scymnus_suturalis_841 | 0.148 |
| Scymnus_ferrugatus_730 | Scymnus_suturalis_841 | 0.148 |
| Scymnus_ferrugatus_731 | Scymnus_suturalis_841 | 0.148 |
| Scymnus_ferrugatus_732 | Scymnus_suturalis_841 | 0.148 |
| Scymnus_ferrugatus_733 | Scymnus_suturalis_841 | 0.148 |
| Scymnus_ferrugatus_723 | Scymnus_suturalis_842 | 0.148 |
| Scymnus_ferrugatus_725 | Scymnus_suturalis_842 | 0.148 |
| Scymnus_ferrugatus_726 | Scymnus_suturalis_842 | 0.148 |
| Scymnus_ferrugatus_727 | Scymnus_suturalis_842 | 0.148 |
| Scymnus_ferrugatus_728 | Scymnus_suturalis_842 | 0.148 |

| | | |
|---|---|---|
| Scymnus_ferrugatus_730 | Scymnus_suturalis_842 | 0.148 |
| Scymnus_ferrugatus_731 | Scymnus_suturalis_842 | 0.148 |
| Scymnus_ferrugatus_732 | Scymnus_suturalis_842 | 0.148 |
| Scymnus_ferrugatus_733 | Scymnus_suturalis_842 | 0.148 |
| Scymnus_ferrugatus_723 | Scymnus_suturalis_844 | 0.148 |
| Scymnus_ferrugatus_725 | Scymnus_suturalis_844 | 0.148 |
| Scymnus_ferrugatus_726 | Scymnus_suturalis_844 | 0.148 |
| Scymnus_ferrugatus_727 | Scymnus_suturalis_844 | 0.148 |
| Scymnus_ferrugatus_728 | Scymnus_suturalis_844 | 0.148 |
| Scymnus_ferrugatus_730 | Scymnus_suturalis_844 | 0.148 |
| Scymnus_ferrugatus_731 | Scymnus_suturalis_844 | 0.148 |
| Scymnus_ferrugatus_732 | Scymnus_suturalis_844 | 0.148 |
| Scymnus_ferrugatus_733 | Scymnus_suturalis_844 | 0.148 |
| Scymnus_ferrugatus_723 | Scymnus_suturalis_846 | 0.148 |
| Scymnus_ferrugatus_725 | Scymnus_suturalis_846 | 0.148 |
| Scymnus_ferrugatus_726 | Scymnus_suturalis_846 | 0.148 |
| Scymnus_ferrugatus_727 | Scymnus_suturalis_846 | 0.148 |
| Scymnus_ferrugatus_728 | Scymnus_suturalis_846 | 0.148 |
| Scymnus_ferrugatus_730 | Scymnus_suturalis_846 | 0.148 |
| Scymnus_ferrugatus_731 | Scymnus_suturalis_846 | 0.148 |
| Scymnus_ferrugatus_732 | Scymnus_suturalis_846 | 0.148 |
| Scymnus_ferrugatus_733 | Scymnus_suturalis_846 | 0.148 |
| Scymnus_ferrugatus_723 | Scymnus_suturalis_847 | 0.148 |
| Scymnus_ferrugatus_725 | Scymnus_suturalis_847 | 0.148 |
| Scymnus_ferrugatus_726 | Scymnus_suturalis_847 | 0.148 |
| Scymnus_ferrugatus_727 | Scymnus_suturalis_847 | 0.148 |
| Scymnus_ferrugatus_728 | Scymnus_suturalis_847 | 0.148 |
| Scymnus_ferrugatus_730 | Scymnus_suturalis_847 | 0.148 |
| Scymnus_ferrugatus_731 | Scymnus_suturalis_847 | 0.148 |
| Scymnus_ferrugatus_732 | Scymnus_suturalis_847 | 0.148 |
| Scymnus_ferrugatus_733 | Scymnus_suturalis_847 | 0.148 |
| Scymnus_ferrugatus_723 | Scymnus_suturalis_848 | 0.148 |
| Scymnus_ferrugatus_725 | Scymnus_suturalis_848 | 0.148 |
| Scymnus_ferrugatus_726 | Scymnus_suturalis_848 | 0.148 |
| Scymnus_ferrugatus_727 | Scymnus_suturalis_848 | 0.148 |
| Scymnus_ferrugatus_728 | Scymnus_suturalis_848 | 0.148 |
| Scymnus_ferrugatus_730 | Scymnus_suturalis_848 | 0.148 |
| Scymnus_ferrugatus_731 | Scymnus_suturalis_848 | 0.148 |
| Scymnus_ferrugatus_732 | Scymnus_suturalis_848 | 0.148 |
| Scymnus_ferrugatus_733 | Scymnus_suturalis_848 | 0.148 |
| Scymnus_ferrugatus_723 | Scymnus_suturalis_832 | 0.148 |
| Scymnus_ferrugatus_725 | Scymnus_suturalis_832 | 0.148 |
| Scymnus_ferrugatus_726 | Scymnus_suturalis_832 | 0.148 |
| Scymnus_ferrugatus_727 | Scymnus_suturalis_832 | 0.148 |
| Scymnus_ferrugatus_728 | Scymnus_suturalis_832 | 0.148 |
| Scymnus_ferrugatus_730 | Scymnus_suturalis_832 | 0.148 |
| Scymnus_ferrugatus_731 | Scymnus_suturalis_832 | 0.148 |
| Scymnus_ferrugatus_732 | Scymnus_suturalis_832 | 0.148 |
| Scymnus_ferrugatus_733 | Scymnus_suturalis_832 | 0.148 |
| Scymnus_ferrugatus_723 | Scymnus_suturalis_835 | 0.148 |
| Scymnus_ferrugatus_725 | Scymnus_suturalis_835 | 0.148 |
| Scymnus_ferrugatus_726 | Scymnus_suturalis_835 | 0.148 |
| Scymnus_ferrugatus_727 | Scymnus_suturalis_835 | 0.148 |
| Scymnus_ferrugatus_728 | Scymnus_suturalis_835 | 0.148 |
| Scymnus_ferrugatus_730 | Scymnus_suturalis_835 | 0.148 |
| Scymnus_ferrugatus_731 | Scymnus_suturalis_835 | 0.148 |

| | | |
|---|---|---|
| Scymnus_ferrugatus_732 | Scymnus_suturalis_835 | 0.148 |
| Scymnus_ferrugatus_733 | Scymnus_suturalis_835 | 0.148 |
| Scymnus_ferrugatus_723 | Scymnus_suturalis_836 | 0.148 |
| Scymnus_ferrugatus_725 | Scymnus_suturalis_836 | 0.148 |
| Scymnus_ferrugatus_726 | Scymnus_suturalis_836 | 0.148 |
| Scymnus_ferrugatus_727 | Scymnus_suturalis_836 | 0.148 |
| Scymnus_ferrugatus_728 | Scymnus_suturalis_836 | 0.148 |
| Scymnus_ferrugatus_730 | Scymnus_suturalis_836 | 0.148 |
| Scymnus_ferrugatus_731 | Scymnus_suturalis_836 | 0.148 |
| Scymnus_ferrugatus_732 | Scymnus_suturalis_836 | 0.148 |
| Scymnus_ferrugatus_733 | Scymnus_suturalis_836 | 0.148 |
| Scymnus_ferrugatus_723 | Scymnus_suturalis_837 | 0.148 |
| Scymnus_ferrugatus_725 | Scymnus_suturalis_837 | 0.148 |
| Scymnus_ferrugatus_726 | Scymnus_suturalis_837 | 0.148 |
| Scymnus_ferrugatus_727 | Scymnus_suturalis_837 | 0.148 |
| Scymnus_ferrugatus_728 | Scymnus_suturalis_837 | 0.148 |
| Scymnus_ferrugatus_730 | Scymnus_suturalis_837 | 0.148 |
| Scymnus_ferrugatus_731 | Scymnus_suturalis_837 | 0.148 |
| Scymnus_ferrugatus_732 | Scymnus_suturalis_837 | 0.148 |
| Scymnus_ferrugatus_733 | Scymnus_suturalis_837 | 0.148 |
| Scymnus_ferrugatus_723 | Scymnus_suturalis_839 | 0.148 |
| Scymnus_ferrugatus_725 | Scymnus_suturalis_839 | 0.148 |
| Scymnus_ferrugatus_726 | Scymnus_suturalis_839 | 0.148 |
| Scymnus_ferrugatus_727 | Scymnus_suturalis_839 | 0.148 |
| Scymnus_ferrugatus_728 | Scymnus_suturalis_839 | 0.148 |
| Scymnus_ferrugatus_730 | Scymnus_suturalis_839 | 0.148 |
| Scymnus_ferrugatus_731 | Scymnus_suturalis_839 | 0.148 |
| Scymnus_ferrugatus_732 | Scymnus_suturalis_839 | 0.148 |
| Scymnus_ferrugatus_733 | Scymnus_suturalis_839 | 0.148 |
| Scymnus_ferrugatus_724 | Scymnus_suturalis_840 | 0.148 |
| Scymnus_ferrugatus_729 | Scymnus_suturalis_840 | 0.148 |
| Coelophora_inaequalis_319 | Tytthaspis_sedecimpunctata_586 | 0.148 |
| Scymnus_caurinus_720 | Scymnus_sinuanodulus_830 | 0.148 |
| Scymnus_caurinus_721 | Scymnus_sinuanodulus_830 | 0.148 |
| Scymnus_caurinus_722 | Scymnus_sinuanodulus_830 | 0.148 |
| Scymnus_camptodromus_714 | Scymnus_suturalis_833 | 0.148 |
| Scymnus_auritus_703 | Scymnus_suturalis_838 | 0.148 |
| Scymnus_camptodromus_704 | Scymnus_suturalis_838 | 0.148 |
| Scymnus_auritus_703 | Scymnus_suturalis_840 | 0.148 |
| Scymnus_camptodromus_704 | Scymnus_suturalis_840 | 0.148 |
| Scymnus_sinuanodulus_830 | Scymnus_suturalis_840 | 0.148 |
| Coelophora_inaequalis_319 | Tytthaspis_sedecimpunctata_583 | 0.148 |
| Coelophora_inaequalis_319 | Tytthaspis_sedecimpunctata_592 | 0.148 |
| Scymnus_camptodromus_714 | Scymnus_suturalis_841 | 0.148 |
| Scymnus_camptodromus_714 | Scymnus_suturalis_842 | 0.148 |
| Scymnus_auritus_701 | Scymnus_suturalis_843 | 0.148 |
| Scymnus_camptodromus_714 | Scymnus_suturalis_844 | 0.148 |
| Scymnus_auritus_701 | Scymnus_suturalis_845 | 0.148 |
| Scymnus_camptodromus_704 | Scymnus_suturalis_845 | 0.148 |
| Scymnus_camptodromus_714 | Scymnus_suturalis_846 | 0.148 |
| Scymnus_camptodromus_714 | Scymnus_suturalis_847 | 0.148 |
| Scymnus_camptodromus_714 | Scymnus_suturalis_848 | 0.148 |
| Anisosticta_novemdecimpunctata_169 | Tytthaspis_sedecimpunctata_586 | 0.148 |
| Anisosticta_novemdecimpunctata_172 | Tytthaspis_sedecimpunctata_586 | 0.148 |
| Anisosticta_novemdecimpunctata_169 | Tytthaspis_sedecimpunctata_588 | 0.148 |
| Anisosticta_novemdecimpunctata_172 | Tytthaspis_sedecimpunctata_588 | 0.148 |

| | | |
|---|---|---|
| Scymnus_nigrinus_784 | Scymnus_rubromaculatus_822 | 0.148 |
| Scymnus_nigrinus_784 | Scymnus_rubromaculatus_823 | 0.148 |
| Scymnus_nigrinus_784 | Scymnus_rubromaculatus_824 | 0.148 |
| Scymnus_nigrinus_784 | Scymnus_rubromaculatus_825 | 0.148 |
| Scymnus_nigrinus_784 | Scymnus_rubromaculatus_826 | 0.148 |
| Scymnus_nigrinus_784 | Scymnus_rubromaculatus_827 | 0.148 |
| Scymnus_camptodromus_714 | Scymnus_suturalis_832 | 0.148 |
| Scymnus_auritus_703 | Scymnus_suturalis_834 | 0.148 |
| Scymnus_camptodromus_714 | Scymnus_suturalis_835 | 0.148 |
| Scymnus_camptodromus_714 | Scymnus_suturalis_836 | 0.148 |
| Scymnus_camptodromus_714 | Scymnus_suturalis_837 | 0.148 |
| Scymnus_camptodromus_714 | Scymnus_suturalis_839 | 0.148 |
| Anisosticta_novemdecimpunctata_165 | Tytthaspis_sedecimpunctata_577 | 0.148 |
| Anisosticta_novemdecimpunctata_173 | Tytthaspis_sedecimpunctata_577 | 0.148 |
| Anisosticta_novemdecimpunctata_165 | Tytthaspis_sedecimpunctata_578 | 0.148 |
| Anisosticta_novemdecimpunctata_173 | Tytthaspis_sedecimpunctata_578 | 0.148 |
| Anisosticta_novemdecimpunctata_165 | Tytthaspis_sedecimpunctata_579 | 0.148 |
| Anisosticta_novemdecimpunctata_173 | Tytthaspis_sedecimpunctata_579 | 0.148 |
| Anisosticta_novemdecimpunctata_165 | Tytthaspis_sedecimpunctata_581 | 0.148 |
| Anisosticta_novemdecimpunctata_173 | Tytthaspis_sedecimpunctata_581 | 0.148 |
| Anisosticta_novemdecimpunctata_165 | Tytthaspis_sedecimpunctata_582 | 0.148 |
| Anisosticta_novemdecimpunctata_173 | Tytthaspis_sedecimpunctata_582 | 0.148 |
| Anisosticta_novemdecimpunctata_169 | Tytthaspis_sedecimpunctata_585 | 0.148 |
| Anisosticta_novemdecimpunctata_172 | Tytthaspis_sedecimpunctata_585 | 0.148 |
| Anisosticta_novemdecimpunctata_165 | Tytthaspis_sedecimpunctata_590 | 0.148 |
| Anisosticta_novemdecimpunctata_173 | Tytthaspis_sedecimpunctata_590 | 0.148 |
| Anisosticta_novemdecimpunctata_169 | Tytthaspis_sedecimpunctata_592 | 0.148 |
| Anisosticta_novemdecimpunctata_172 | Tytthaspis_sedecimpunctata_592 | 0.148 |
| Anisosticta_novemdecimpunctata_165 | Tytthaspis_sedecimpunctata_593 | 0.148 |
| Anisosticta_novemdecimpunctata_173 | Tytthaspis_sedecimpunctata_593 | 0.148 |
| Anisosticta_novemdecimpunctata_165 | Tytthaspis_sedecimpunctata_594 | 0.148 |
| Anisosticta_novemdecimpunctata_173 | Tytthaspis_sedecimpunctata_594 | 0.148 |
| Anisosticta_novemdecimpunctata_165 | Tytthaspis_sedecimpunctata_595 | 0.148 |
| Anisosticta_novemdecimpunctata_173 | Tytthaspis_sedecimpunctata_595 | 0.148 |
| Scymnus_loewii_772 | Scymnus_sinuanodulus_828 | 0.148 |
| Scymnus_loewii_773 | Scymnus_sinuanodulus_828 | 0.148 |
| Scymnus_sinuanodulus_831 | Scymnus_suturalis_838 | 0.148 |
| Scymnus_sinuanodulus_831 | Scymnus_suturalis_840 | 0.148 |
| Anisosticta_novemdecimpunctata_165 | Tytthaspis_sedecimpunctata_580 | 0.148 |
| Anisosticta_novemdecimpunctata_173 | Tytthaspis_sedecimpunctata_580 | 0.148 |
| Anisosticta_bitriangularis_151 | Tytthaspis_sedecimpunctata_589 | 0.148 |
| Anisosticta_bitriangularis_154 | Tytthaspis_sedecimpunctata_589 | 0.148 |
| Scymnus_abietis_687 | Scymnus_sinuanodulus_830 | 0.148 |
| Anisosticta_bitriangularis_152 | Tytthaspis_sedecimpunctata_586 | 0.149 |
| Anisosticta_bitriangularis_151 | Tytthaspis_sedecimpunctata_587 | 0.149 |
| Anisosticta_bitriangularis_154 | Tytthaspis_sedecimpunctata_587 | 0.149 |
| Anisosticta_bitriangularis_152 | Tytthaspis_sedecimpunctata_588 | 0.149 |
| Anisosticta_bitriangularis_152 | Tytthaspis_sedecimpunctata_591 | 0.149 |
| Scymnus_abietis_683 | Scymnus_sinuanodulus_828 | 0.149 |
| Scymnus_abietis_687 | Scymnus_sinuanodulus_828 | 0.149 |
| Halyzia_sedecimguttata_351 | Vibidia_duodecimguttata_596 | 0.149 |
| Halyzia_sedecimguttata_353 | Vibidia_duodecimguttata_596 | 0.149 |
| Halyzia_sedecimguttata_351 | Vibidia_duodecimguttata_598 | 0.149 |
| Halyzia_sedecimguttata_353 | Vibidia_duodecimguttata_598 | 0.149 |
| Scymnus_nebulosus_782 | Scymnus_sinuanodulus_829 | 0.149 |
| Harmonia_conformis_365 | Tytthaspis_sedecimpunctata_577 | 0.149 |

| | | |
|---|---|---|
| Harmonia_conformis_366 | Tytthaspis_sedecimpunctata_577 | 0.149 |
| Harmonia_conformis_367 | Tytthaspis_sedecimpunctata_577 | 0.149 |
| Harmonia_conformis_368 | Tytthaspis_sedecimpunctata_577 | 0.149 |
| Harmonia_conformis_369 | Tytthaspis_sedecimpunctata_577 | 0.149 |
| Harmonia_conformis_370 | Tytthaspis_sedecimpunctata_577 | 0.149 |
| Harmonia_conformis_371 | Tytthaspis_sedecimpunctata_577 | 0.149 |
| Harmonia_conformis_372 | Tytthaspis_sedecimpunctata_577 | 0.149 |
| Harmonia_conformis_373 | Tytthaspis_sedecimpunctata_577 | 0.149 |
| Harmonia_conformis_375 | Tytthaspis_sedecimpunctata_577 | 0.149 |
| Harmonia_conformis_365 | Tytthaspis_sedecimpunctata_578 | 0.149 |
| Harmonia_conformis_366 | Tytthaspis_sedecimpunctata_578 | 0.149 |
| Harmonia_conformis_367 | Tytthaspis_sedecimpunctata_578 | 0.149 |
| Harmonia_conformis_368 | Tytthaspis_sedecimpunctata_578 | 0.149 |
| Harmonia_conformis_369 | Tytthaspis_sedecimpunctata_578 | 0.149 |
| Harmonia_conformis_370 | Tytthaspis_sedecimpunctata_578 | 0.149 |
| Harmonia_conformis_371 | Tytthaspis_sedecimpunctata_578 | 0.149 |
| Harmonia_conformis_372 | Tytthaspis_sedecimpunctata_578 | 0.149 |
| Harmonia_conformis_373 | Tytthaspis_sedecimpunctata_578 | 0.149 |
| Harmonia_conformis_375 | Tytthaspis_sedecimpunctata_578 | 0.149 |
| Harmonia_conformis_365 | Tytthaspis_sedecimpunctata_579 | 0.149 |
| Harmonia_conformis_366 | Tytthaspis_sedecimpunctata_579 | 0.149 |
| Harmonia_conformis_367 | Tytthaspis_sedecimpunctata_579 | 0.149 |
| Harmonia_conformis_368 | Tytthaspis_sedecimpunctata_579 | 0.149 |
| Harmonia_conformis_369 | Tytthaspis_sedecimpunctata_579 | 0.149 |
| Harmonia_conformis_370 | Tytthaspis_sedecimpunctata_579 | 0.149 |
| Harmonia_conformis_371 | Tytthaspis_sedecimpunctata_579 | 0.149 |
| Harmonia_conformis_372 | Tytthaspis_sedecimpunctata_579 | 0.149 |
| Harmonia_conformis_373 | Tytthaspis_sedecimpunctata_579 | 0.149 |
| Harmonia_conformis_375 | Tytthaspis_sedecimpunctata_579 | 0.149 |
| Harmonia_conformis_365 | Tytthaspis_sedecimpunctata_581 | 0.149 |
| Harmonia_conformis_366 | Tytthaspis_sedecimpunctata_581 | 0.149 |
| Harmonia_conformis_367 | Tytthaspis_sedecimpunctata_581 | 0.149 |
| Harmonia_conformis_368 | Tytthaspis_sedecimpunctata_581 | 0.149 |
| Harmonia_conformis_369 | Tytthaspis_sedecimpunctata_581 | 0.149 |
| Harmonia_conformis_370 | Tytthaspis_sedecimpunctata_581 | 0.149 |
| Harmonia_conformis_371 | Tytthaspis_sedecimpunctata_581 | 0.149 |
| Harmonia_conformis_372 | Tytthaspis_sedecimpunctata_581 | 0.149 |
| Harmonia_conformis_373 | Tytthaspis_sedecimpunctata_581 | 0.149 |
| Harmonia_conformis_375 | Tytthaspis_sedecimpunctata_581 | 0.149 |
| Harmonia_conformis_365 | Tytthaspis_sedecimpunctata_582 | 0.149 |
| Harmonia_conformis_366 | Tytthaspis_sedecimpunctata_582 | 0.149 |
| Harmonia_conformis_367 | Tytthaspis_sedecimpunctata_582 | 0.149 |
| Harmonia_conformis_368 | Tytthaspis_sedecimpunctata_582 | 0.149 |
| Harmonia_conformis_369 | Tytthaspis_sedecimpunctata_582 | 0.149 |
| Harmonia_conformis_370 | Tytthaspis_sedecimpunctata_582 | 0.149 |
| Harmonia_conformis_371 | Tytthaspis_sedecimpunctata_582 | 0.149 |
| Harmonia_conformis_372 | Tytthaspis_sedecimpunctata_582 | 0.149 |
| Harmonia_conformis_373 | Tytthaspis_sedecimpunctata_582 | 0.149 |
| Harmonia_conformis_375 | Tytthaspis_sedecimpunctata_582 | 0.149 |
| Harmonia_conformis_365 | Tytthaspis_sedecimpunctata_590 | 0.149 |
| Harmonia_conformis_366 | Tytthaspis_sedecimpunctata_590 | 0.149 |
| Harmonia_conformis_367 | Tytthaspis_sedecimpunctata_590 | 0.149 |
| Harmonia_conformis_368 | Tytthaspis_sedecimpunctata_590 | 0.149 |
| Harmonia_conformis_369 | Tytthaspis_sedecimpunctata_590 | 0.149 |
| Harmonia_conformis_370 | Tytthaspis_sedecimpunctata_590 | 0.149 |
| Harmonia_conformis_371 | Tytthaspis_sedecimpunctata_590 | 0.149 |

Supplementary Table S4 (continued)

| | | |
|---|---|---|
| Harmonia_conformis_372 | Tytthaspis_sedecimpunctata_590 | 0.149 |
| Harmonia_conformis_373 | Tytthaspis_sedecimpunctata_590 | 0.149 |
| Harmonia_conformis_375 | Tytthaspis_sedecimpunctata_590 | 0.149 |
| Harmonia_conformis_365 | Tytthaspis_sedecimpunctata_592 | 0.149 |
| Harmonia_conformis_366 | Tytthaspis_sedecimpunctata_592 | 0.149 |
| Harmonia_conformis_367 | Tytthaspis_sedecimpunctata_592 | 0.149 |
| Harmonia_conformis_368 | Tytthaspis_sedecimpunctata_592 | 0.149 |
| Harmonia_conformis_369 | Tytthaspis_sedecimpunctata_592 | 0.149 |
| Harmonia_conformis_370 | Tytthaspis_sedecimpunctata_592 | 0.149 |
| Harmonia_conformis_371 | Tytthaspis_sedecimpunctata_592 | 0.149 |
| Harmonia_conformis_372 | Tytthaspis_sedecimpunctata_592 | 0.149 |
| Harmonia_conformis_373 | Tytthaspis_sedecimpunctata_592 | 0.149 |
| Harmonia_conformis_375 | Tytthaspis_sedecimpunctata_592 | 0.149 |
| Harmonia_conformis_365 | Tytthaspis_sedecimpunctata_594 | 0.149 |
| Harmonia_conformis_366 | Tytthaspis_sedecimpunctata_594 | 0.149 |
| Harmonia_conformis_367 | Tytthaspis_sedecimpunctata_594 | 0.149 |
| Harmonia_conformis_368 | Tytthaspis_sedecimpunctata_594 | 0.149 |
| Harmonia_conformis_369 | Tytthaspis_sedecimpunctata_594 | 0.149 |
| Harmonia_conformis_370 | Tytthaspis_sedecimpunctata_594 | 0.149 |
| Harmonia_conformis_371 | Tytthaspis_sedecimpunctata_594 | 0.149 |
| Harmonia_conformis_372 | Tytthaspis_sedecimpunctata_594 | 0.149 |
| Harmonia_conformis_373 | Tytthaspis_sedecimpunctata_594 | 0.149 |
| Harmonia_conformis_375 | Tytthaspis_sedecimpunctata_594 | 0.149 |
| Harmonia_conformis_365 | Tytthaspis_sedecimpunctata_595 | 0.149 |
| Harmonia_conformis_366 | Tytthaspis_sedecimpunctata_595 | 0.149 |
| Harmonia_conformis_367 | Tytthaspis_sedecimpunctata_595 | 0.149 |
| Harmonia_conformis_368 | Tytthaspis_sedecimpunctata_595 | 0.149 |
| Harmonia_conformis_369 | Tytthaspis_sedecimpunctata_595 | 0.149 |
| Harmonia_conformis_370 | Tytthaspis_sedecimpunctata_595 | 0.149 |
| Harmonia_conformis_371 | Tytthaspis_sedecimpunctata_595 | 0.149 |
| Harmonia_conformis_372 | Tytthaspis_sedecimpunctata_595 | 0.149 |
| Harmonia_conformis_373 | Tytthaspis_sedecimpunctata_595 | 0.149 |
| Harmonia_conformis_375 | Tytthaspis_sedecimpunctata_595 | 0.149 |
| Harmonia_conformis_374 | Tytthaspis_sedecimpunctata_580 | 0.149 |
| Harmonia_conformis_365 | Tytthaspis_sedecimpunctata_584 | 0.149 |
| Harmonia_conformis_366 | Tytthaspis_sedecimpunctata_584 | 0.149 |
| Harmonia_conformis_367 | Tytthaspis_sedecimpunctata_584 | 0.149 |
| Harmonia_conformis_368 | Tytthaspis_sedecimpunctata_584 | 0.149 |
| Harmonia_conformis_369 | Tytthaspis_sedecimpunctata_584 | 0.149 |
| Harmonia_conformis_370 | Tytthaspis_sedecimpunctata_584 | 0.149 |
| Harmonia_conformis_371 | Tytthaspis_sedecimpunctata_584 | 0.149 |
| Harmonia_conformis_372 | Tytthaspis_sedecimpunctata_584 | 0.149 |
| Harmonia_conformis_373 | Tytthaspis_sedecimpunctata_584 | 0.149 |
| Harmonia_conformis_375 | Tytthaspis_sedecimpunctata_584 | 0.149 |
| Mulsantina_hudsonica_479 | Tytthaspis_sedecimpunctata_586 | 0.149 |
| Mulsantina_hudsonica_479 | Tytthaspis_sedecimpunctata_587 | 0.149 |
| Mulsantina_hudsonica_479 | Tytthaspis_sedecimpunctata_588 | 0.149 |
| Chilocorus_nigritus_25 | Tytthaspis_sedecimpunctata_589 | 0.149 |
| Mulsantina_hudsonica_481 | Tytthaspis_sedecimpunctata_577 | 0.149 |
| Mulsantina_hudsonica_482 | Tytthaspis_sedecimpunctata_577 | 0.149 |
| Mulsantina_hudsonica_481 | Tytthaspis_sedecimpunctata_578 | 0.149 |
| Mulsantina_hudsonica_482 | Tytthaspis_sedecimpunctata_578 | 0.149 |
| Mulsantina_hudsonica_481 | Tytthaspis_sedecimpunctata_579 | 0.149 |
| Mulsantina_hudsonica_482 | Tytthaspis_sedecimpunctata_579 | 0.149 |
| Mulsantina_hudsonica_481 | Tytthaspis_sedecimpunctata_581 | 0.149 |
| Mulsantina_hudsonica_482 | Tytthaspis_sedecimpunctata_581 | 0.149 |

| | | |
|---|---|---|
| Mulsantina_hudsonica_481 | Tytthaspis_sedecimpunctata_582 | 0.149 |
| Mulsantina_hudsonica_482 | Tytthaspis_sedecimpunctata_582 | 0.149 |
| Mulsantina_hudsonica_483 | Tytthaspis_sedecimpunctata_583 | 0.149 |
| Mulsantina_hudsonica_479 | Tytthaspis_sedecimpunctata_585 | 0.149 |
| Mulsantina_hudsonica_481 | Tytthaspis_sedecimpunctata_589 | 0.149 |
| Mulsantina_hudsonica_482 | Tytthaspis_sedecimpunctata_589 | 0.149 |
| Mulsantina_hudsonica_481 | Tytthaspis_sedecimpunctata_590 | 0.149 |
| Mulsantina_hudsonica_482 | Tytthaspis_sedecimpunctata_590 | 0.149 |
| Mulsantina_hudsonica_481 | Tytthaspis_sedecimpunctata_592 | 0.149 |
| Mulsantina_hudsonica_482 | Tytthaspis_sedecimpunctata_592 | 0.149 |
| Mulsantina_hudsonica_481 | Tytthaspis_sedecimpunctata_593 | 0.149 |
| Mulsantina_hudsonica_482 | Tytthaspis_sedecimpunctata_593 | 0.149 |
| Mulsantina_hudsonica_481 | Tytthaspis_sedecimpunctata_594 | 0.149 |
| Mulsantina_hudsonica_482 | Tytthaspis_sedecimpunctata_594 | 0.149 |
| Mulsantina_hudsonica_481 | Tytthaspis_sedecimpunctata_595 | 0.149 |
| Mulsantina_hudsonica_482 | Tytthaspis_sedecimpunctata_595 | 0.149 |
| Scymnus_interruptus_759 | Scymnus_suturalis_838 | 0.149 |
| Scymnus_interruptus_761 | Scymnus_suturalis_838 | 0.149 |
| Scymnus_interruptus_763 | Scymnus_suturalis_838 | 0.149 |
| Scymnus_interruptus_764 | Scymnus_suturalis_838 | 0.149 |
| Scymnus_interruptus_766 | Scymnus_suturalis_838 | 0.149 |
| Scymnus_interruptus_769 | Scymnus_suturalis_838 | 0.149 |
| Scymnus_interruptus_759 | Scymnus_suturalis_840 | 0.149 |
| Scymnus_interruptus_761 | Scymnus_suturalis_840 | 0.149 |
| Scymnus_interruptus_763 | Scymnus_suturalis_840 | 0.149 |
| Scymnus_interruptus_764 | Scymnus_suturalis_840 | 0.149 |
| Scymnus_interruptus_766 | Scymnus_suturalis_840 | 0.149 |
| Scymnus_interruptus_769 | Scymnus_suturalis_840 | 0.149 |
| Mulsantina_hudsonica_483 | Tytthaspis_sedecimpunctata_580 | 0.149 |
| Chilocorus_nigritus_25 | Tytthaspis_sedecimpunctata_593 | 0.149 |
| Scymnus_interruptus_759 | Scymnus_suturalis_834 | 0.149 |
| Scymnus_interruptus_761 | Scymnus_suturalis_834 | 0.149 |
| Scymnus_interruptus_763 | Scymnus_suturalis_834 | 0.149 |
| Scymnus_interruptus_764 | Scymnus_suturalis_834 | 0.149 |
| Scymnus_interruptus_766 | Scymnus_suturalis_834 | 0.149 |
| Scymnus_interruptus_769 | Scymnus_suturalis_834 | 0.149 |
| Coelophora_inaequalis_310 | Tytthaspis_sedecimpunctata_586 | 0.149 |
| Coelophora_inaequalis_312 | Tytthaspis_sedecimpunctata_586 | 0.149 |
| Coelophora_inaequalis_314 | Tytthaspis_sedecimpunctata_586 | 0.149 |
| Coelophora_inaequalis_323 | Tytthaspis_sedecimpunctata_586 | 0.149 |
| Coelophora_inaequalis_325 | Tytthaspis_sedecimpunctata_586 | 0.149 |
| Coelophora_inaequalis_311 | Tytthaspis_sedecimpunctata_588 | 0.149 |
| Coelophora_inaequalis_313 | Tytthaspis_sedecimpunctata_588 | 0.149 |
| Coelophora_inaequalis_316 | Tytthaspis_sedecimpunctata_588 | 0.149 |
| Coelophora_inaequalis_324 | Tytthaspis_sedecimpunctata_588 | 0.149 |
| Coelophora_inaequalis_326 | Tytthaspis_sedecimpunctata_588 | 0.149 |
| Hippodamia_variegata_467 | Tytthaspis_sedecimpunctata_589 | 0.149 |
| Hippodamia_variegata_469 | Tytthaspis_sedecimpunctata_589 | 0.149 |
| Hippodamia_variegata_470 | Tytthaspis_sedecimpunctata_589 | 0.149 |
| Hippodamia_variegata_472 | Tytthaspis_sedecimpunctata_589 | 0.149 |
| Hippodamia_variegata_473 | Tytthaspis_sedecimpunctata_589 | 0.149 |
| Chilocorus_nigritus_25 | Tytthaspis_sedecimpunctata_591 | 0.149 |
| Coelophora_inaequalis_310 | Tytthaspis_sedecimpunctata_577 | 0.149 |
| Coelophora_inaequalis_312 | Tytthaspis_sedecimpunctata_577 | 0.149 |
| Coelophora_inaequalis_314 | Tytthaspis_sedecimpunctata_577 | 0.149 |
| Coelophora_inaequalis_323 | Tytthaspis_sedecimpunctata_577 | 0.149 |

| | | |
|---|---|---|
| Coelophora_inaequalis_325 | Tytthaspis_sedecimpunctata_577 | 0.149 |
| Coelophora_inaequalis_310 | Tytthaspis_sedecimpunctata_578 | 0.149 |
| Coelophora_inaequalis_312 | Tytthaspis_sedecimpunctata_578 | 0.149 |
| Coelophora_inaequalis_314 | Tytthaspis_sedecimpunctata_578 | 0.149 |
| Coelophora_inaequalis_323 | Tytthaspis_sedecimpunctata_578 | 0.149 |
| Coelophora_inaequalis_325 | Tytthaspis_sedecimpunctata_578 | 0.149 |
| Coelophora_inaequalis_310 | Tytthaspis_sedecimpunctata_579 | 0.149 |
| Coelophora_inaequalis_312 | Tytthaspis_sedecimpunctata_579 | 0.149 |
| Coelophora_inaequalis_314 | Tytthaspis_sedecimpunctata_579 | 0.149 |
| Coelophora_inaequalis_323 | Tytthaspis_sedecimpunctata_579 | 0.149 |
| Coelophora_inaequalis_325 | Tytthaspis_sedecimpunctata_579 | 0.149 |
| Coelophora_inaequalis_310 | Tytthaspis_sedecimpunctata_581 | 0.149 |
| Coelophora_inaequalis_312 | Tytthaspis_sedecimpunctata_581 | 0.149 |
| Coelophora_inaequalis_314 | Tytthaspis_sedecimpunctata_581 | 0.149 |
| Coelophora_inaequalis_323 | Tytthaspis_sedecimpunctata_581 | 0.149 |
| Coelophora_inaequalis_325 | Tytthaspis_sedecimpunctata_581 | 0.149 |
| Coelophora_inaequalis_310 | Tytthaspis_sedecimpunctata_582 | 0.149 |
| Coelophora_inaequalis_312 | Tytthaspis_sedecimpunctata_582 | 0.149 |
| Coelophora_inaequalis_314 | Tytthaspis_sedecimpunctata_582 | 0.149 |
| Coelophora_inaequalis_323 | Tytthaspis_sedecimpunctata_582 | 0.149 |
| Coelophora_inaequalis_325 | Tytthaspis_sedecimpunctata_582 | 0.149 |
| Coelophora_inaequalis_311 | Tytthaspis_sedecimpunctata_583 | 0.149 |
| Coelophora_inaequalis_313 | Tytthaspis_sedecimpunctata_583 | 0.149 |
| Coelophora_inaequalis_316 | Tytthaspis_sedecimpunctata_583 | 0.149 |
| Coelophora_inaequalis_324 | Tytthaspis_sedecimpunctata_583 | 0.149 |
| Coelophora_inaequalis_326 | Tytthaspis_sedecimpunctata_583 | 0.149 |
| Hippodamia_variegata_471 | Tytthaspis_sedecimpunctata_584 | 0.149 |
| Coelophora_inaequalis_317 | Tytthaspis_sedecimpunctata_585 | 0.149 |
| Hippodamia_variegata_471 | Tytthaspis_sedecimpunctata_586 | 0.149 |
| Exochomus_nigromaculatus_49 | Tytthaspis_sedecimpunctata_587 | 0.149 |
| Hippodamia_variegata_471 | Tytthaspis_sedecimpunctata_587 | 0.149 |
| Coelophora_inaequalis_315 | Tytthaspis_sedecimpunctata_588 | 0.149 |
| Hippodamia_variegata_467 | Tytthaspis_sedecimpunctata_588 | 0.149 |
| Hippodamia_variegata_469 | Tytthaspis_sedecimpunctata_588 | 0.149 |
| Hippodamia_variegata_470 | Tytthaspis_sedecimpunctata_588 | 0.149 |
| Hippodamia_variegata_472 | Tytthaspis_sedecimpunctata_588 | 0.149 |
| Hippodamia_variegata_473 | Tytthaspis_sedecimpunctata_588 | 0.149 |
| Coelophora_inaequalis_310 | Tytthaspis_sedecimpunctata_590 | 0.149 |
| Coelophora_inaequalis_312 | Tytthaspis_sedecimpunctata_590 | 0.149 |
| Coelophora_inaequalis_314 | Tytthaspis_sedecimpunctata_590 | 0.149 |
| Coelophora_inaequalis_323 | Tytthaspis_sedecimpunctata_590 | 0.149 |
| Coelophora_inaequalis_325 | Tytthaspis_sedecimpunctata_590 | 0.149 |
| Coelophora_inaequalis_311 | Tytthaspis_sedecimpunctata_592 | 0.149 |
| Coelophora_inaequalis_313 | Tytthaspis_sedecimpunctata_592 | 0.149 |
| Coelophora_inaequalis_316 | Tytthaspis_sedecimpunctata_592 | 0.149 |
| Coelophora_inaequalis_324 | Tytthaspis_sedecimpunctata_592 | 0.149 |
| Coelophora_inaequalis_326 | Tytthaspis_sedecimpunctata_592 | 0.149 |
| Coelophora_inaequalis_310 | Tytthaspis_sedecimpunctata_593 | 0.149 |
| Coelophora_inaequalis_312 | Tytthaspis_sedecimpunctata_593 | 0.149 |
| Coelophora_inaequalis_314 | Tytthaspis_sedecimpunctata_593 | 0.149 |
| Coelophora_inaequalis_323 | Tytthaspis_sedecimpunctata_593 | 0.149 |
| Coelophora_inaequalis_325 | Tytthaspis_sedecimpunctata_593 | 0.149 |
| Coelophora_inaequalis_310 | Tytthaspis_sedecimpunctata_594 | 0.149 |
| Coelophora_inaequalis_312 | Tytthaspis_sedecimpunctata_594 | 0.149 |
| Coelophora_inaequalis_314 | Tytthaspis_sedecimpunctata_594 | 0.149 |
| Coelophora_inaequalis_323 | Tytthaspis_sedecimpunctata_594 | 0.149 |

| | | |
|---|---|---|
| Coelophora_inaequalis_325 | Tytthaspis_sedecimpunctata_594 | 0.149 |
| Coelophora_inaequalis_310 | Tytthaspis_sedecimpunctata_595 | 0.149 |
| Coelophora_inaequalis_312 | Tytthaspis_sedecimpunctata_595 | 0.149 |
| Coelophora_inaequalis_314 | Tytthaspis_sedecimpunctata_595 | 0.149 |
| Coelophora_inaequalis_323 | Tytthaspis_sedecimpunctata_595 | 0.149 |
| Coelophora_inaequalis_325 | Tytthaspis_sedecimpunctata_595 | 0.149 |
| Scymnus_abietis_689 | Scymnus_sinuanodulus_831 | 0.149 |
| Coelophora_inaequalis_314 | Tytthaspis_sedecimpunctata_580 | 0.149 |
| Coelophora_inaequalis_315 | Tytthaspis_sedecimpunctata_583 | 0.149 |
| Exochomus_nigromaculatus_49 | Tytthaspis_sedecimpunctata_583 | 0.149 |
| Hippodamia_variegata_467 | Tytthaspis_sedecimpunctata_583 | 0.149 |
| Hippodamia_variegata_469 | Tytthaspis_sedecimpunctata_583 | 0.149 |
| Hippodamia_variegata_470 | Tytthaspis_sedecimpunctata_583 | 0.149 |
| Hippodamia_variegata_472 | Tytthaspis_sedecimpunctata_583 | 0.149 |
| Hippodamia_variegata_473 | Tytthaspis_sedecimpunctata_583 | 0.149 |
| Coelophora_inaequalis_310 | Tytthaspis_sedecimpunctata_584 | 0.149 |
| Coelophora_inaequalis_312 | Tytthaspis_sedecimpunctata_584 | 0.149 |
| Coelophora_inaequalis_314 | Tytthaspis_sedecimpunctata_584 | 0.149 |
| Coelophora_inaequalis_323 | Tytthaspis_sedecimpunctata_584 | 0.149 |
| Coelophora_inaequalis_325 | Tytthaspis_sedecimpunctata_584 | 0.149 |
| Hippodamia_variegata_468 | Tytthaspis_sedecimpunctata_584 | 0.149 |
| Hippodamia_variegata_468 | Tytthaspis_sedecimpunctata_586 | 0.149 |
| Hippodamia_variegata_468 | Tytthaspis_sedecimpunctata_587 | 0.149 |
| Coelophora_inaequalis_315 | Tytthaspis_sedecimpunctata_592 | 0.149 |
| Hippodamia_variegata_471 | Tytthaspis_sedecimpunctata_593 | 0.149 |
| Myrrha_octodecimguttata_499 | Tytthaspis_sedecimpunctata_577 | 0.149 |
| Myrrha_octodecimguttata_499 | Tytthaspis_sedecimpunctata_578 | 0.149 |
| Myrrha_octodecimguttata_499 | Tytthaspis_sedecimpunctata_579 | 0.149 |
| Coelophora_inaequalis_315 | Tytthaspis_sedecimpunctata_580 | 0.149 |
| Myrrha_octodecimguttata_499 | Tytthaspis_sedecimpunctata_581 | 0.149 |
| Myrrha_octodecimguttata_499 | Tytthaspis_sedecimpunctata_582 | 0.149 |
| Exochomus_nigromaculatus_49 | Tytthaspis_sedecimpunctata_584 | 0.149 |
| Myrrha_octodecimguttata_489 | Tytthaspis_sedecimpunctata_589 | 0.149 |
| Myrrha_octodecimguttata_500 | Tytthaspis_sedecimpunctata_589 | 0.149 |
| Myrrha_octodecimguttata_499 | Tytthaspis_sedecimpunctata_590 | 0.149 |
| Hippodamia_variegata_467 | Tytthaspis_sedecimpunctata_591 | 0.149 |
| Hippodamia_variegata_469 | Tytthaspis_sedecimpunctata_591 | 0.149 |
| Hippodamia_variegata_470 | Tytthaspis_sedecimpunctata_591 | 0.149 |
| Hippodamia_variegata_472 | Tytthaspis_sedecimpunctata_591 | 0.149 |
| Hippodamia_variegata_473 | Tytthaspis_sedecimpunctata_591 | 0.149 |
| Hippodamia_variegata_468 | Tytthaspis_sedecimpunctata_593 | 0.149 |
| Myrrha_octodecimguttata_499 | Tytthaspis_sedecimpunctata_594 | 0.149 |
| Myrrha_octodecimguttata_499 | Tytthaspis_sedecimpunctata_595 | 0.149 |
| Myrrha_octodecimguttata_499 | Tytthaspis_sedecimpunctata_580 | 0.149 |
| Myrrha_octodecimguttata_485 | Tytthaspis_sedecimpunctata_583 | 0.149 |
| Myrrha_octodecimguttata_486 | Tytthaspis_sedecimpunctata_583 | 0.149 |
| Myrrha_octodecimguttata_487 | Tytthaspis_sedecimpunctata_583 | 0.149 |
| Myrrha_octodecimguttata_490 | Tytthaspis_sedecimpunctata_583 | 0.149 |
| Myrrha_octodecimguttata_494 | Tytthaspis_sedecimpunctata_583 | 0.149 |
| Myrrha_octodecimguttata_496 | Tytthaspis_sedecimpunctata_583 | 0.149 |
| Myrrha_octodecimguttata_497 | Tytthaspis_sedecimpunctata_583 | 0.149 |
| Myrrha_octodecimguttata_501 | Tytthaspis_sedecimpunctata_583 | 0.149 |
| Myrrha_octodecimguttata_502 | Tytthaspis_sedecimpunctata_583 | 0.149 |
| Myrrha_octodecimguttata_489 | Tytthaspis_sedecimpunctata_586 | 0.149 |
| Myrrha_octodecimguttata_499 | Tytthaspis_sedecimpunctata_586 | 0.149 |
| Myrrha_octodecimguttata_500 | Tytthaspis_sedecimpunctata_586 | 0.149 |

Supplementary Table S4 (continued)

| | | |
|---|---|---|
| Myrrha_octodecimguttata_488 | Tytthaspis_sedecimpunctata_587 | 0.149 |
| Myrrha_octodecimguttata_491 | Tytthaspis_sedecimpunctata_587 | 0.149 |
| Myrrha_octodecimguttata_492 | Tytthaspis_sedecimpunctata_587 | 0.149 |
| Myrrha_octodecimguttata_485 | Tytthaspis_sedecimpunctata_589 | 0.149 |
| Myrrha_octodecimguttata_486 | Tytthaspis_sedecimpunctata_589 | 0.149 |
| Myrrha_octodecimguttata_487 | Tytthaspis_sedecimpunctata_589 | 0.149 |
| Myrrha_octodecimguttata_490 | Tytthaspis_sedecimpunctata_589 | 0.149 |
| Myrrha_octodecimguttata_494 | Tytthaspis_sedecimpunctata_589 | 0.149 |
| Myrrha_octodecimguttata_496 | Tytthaspis_sedecimpunctata_589 | 0.149 |
| Myrrha_octodecimguttata_497 | Tytthaspis_sedecimpunctata_589 | 0.149 |
| Myrrha_octodecimguttata_501 | Tytthaspis_sedecimpunctata_589 | 0.149 |
| Myrrha_octodecimguttata_502 | Tytthaspis_sedecimpunctata_589 | 0.149 |
| Myrrha_octodecimguttata_485 | Tytthaspis_sedecimpunctata_592 | 0.149 |
| Myrrha_octodecimguttata_486 | Tytthaspis_sedecimpunctata_592 | 0.149 |
| Myrrha_octodecimguttata_487 | Tytthaspis_sedecimpunctata_592 | 0.149 |
| Myrrha_octodecimguttata_490 | Tytthaspis_sedecimpunctata_592 | 0.149 |
| Myrrha_octodecimguttata_494 | Tytthaspis_sedecimpunctata_592 | 0.149 |
| Myrrha_octodecimguttata_496 | Tytthaspis_sedecimpunctata_592 | 0.149 |
| Myrrha_octodecimguttata_497 | Tytthaspis_sedecimpunctata_592 | 0.149 |
| Myrrha_octodecimguttata_501 | Tytthaspis_sedecimpunctata_592 | 0.149 |
| Myrrha_octodecimguttata_502 | Tytthaspis_sedecimpunctata_592 | 0.149 |
| Myrrha_octodecimguttata_493 | Tytthaspis_sedecimpunctata_577 | 0.149 |
| Myrrha_octodecimguttata_495 | Tytthaspis_sedecimpunctata_577 | 0.149 |
| Myrrha_octodecimguttata_498 | Tytthaspis_sedecimpunctata_577 | 0.149 |
| Myrrha_octodecimguttata_493 | Tytthaspis_sedecimpunctata_578 | 0.149 |
| Myrrha_octodecimguttata_495 | Tytthaspis_sedecimpunctata_578 | 0.149 |
| Myrrha_octodecimguttata_498 | Tytthaspis_sedecimpunctata_578 | 0.149 |
| Myrrha_octodecimguttata_493 | Tytthaspis_sedecimpunctata_579 | 0.149 |
| Myrrha_octodecimguttata_495 | Tytthaspis_sedecimpunctata_579 | 0.149 |
| Myrrha_octodecimguttata_498 | Tytthaspis_sedecimpunctata_579 | 0.149 |
| Myrrha_octodecimguttata_493 | Tytthaspis_sedecimpunctata_581 | 0.149 |
| Myrrha_octodecimguttata_495 | Tytthaspis_sedecimpunctata_581 | 0.149 |
| Myrrha_octodecimguttata_498 | Tytthaspis_sedecimpunctata_581 | 0.149 |
| Myrrha_octodecimguttata_493 | Tytthaspis_sedecimpunctata_582 | 0.149 |
| Myrrha_octodecimguttata_495 | Tytthaspis_sedecimpunctata_582 | 0.149 |
| Myrrha_octodecimguttata_498 | Tytthaspis_sedecimpunctata_582 | 0.149 |
| Myrrha_octodecimguttata_485 | Tytthaspis_sedecimpunctata_588 | 0.149 |
| Myrrha_octodecimguttata_486 | Tytthaspis_sedecimpunctata_588 | 0.149 |
| Myrrha_octodecimguttata_487 | Tytthaspis_sedecimpunctata_588 | 0.149 |
| Myrrha_octodecimguttata_490 | Tytthaspis_sedecimpunctata_588 | 0.149 |
| Myrrha_octodecimguttata_494 | Tytthaspis_sedecimpunctata_588 | 0.149 |
| Myrrha_octodecimguttata_496 | Tytthaspis_sedecimpunctata_588 | 0.149 |
| Myrrha_octodecimguttata_497 | Tytthaspis_sedecimpunctata_588 | 0.149 |
| Myrrha_octodecimguttata_501 | Tytthaspis_sedecimpunctata_588 | 0.149 |
| Myrrha_octodecimguttata_502 | Tytthaspis_sedecimpunctata_588 | 0.149 |
| Myrrha_octodecimguttata_493 | Tytthaspis_sedecimpunctata_590 | 0.149 |
| Myrrha_octodecimguttata_495 | Tytthaspis_sedecimpunctata_590 | 0.149 |
| Myrrha_octodecimguttata_498 | Tytthaspis_sedecimpunctata_590 | 0.149 |
| Myrrha_octodecimguttata_493 | Tytthaspis_sedecimpunctata_594 | 0.149 |
| Myrrha_octodecimguttata_495 | Tytthaspis_sedecimpunctata_594 | 0.149 |
| Myrrha_octodecimguttata_498 | Tytthaspis_sedecimpunctata_594 | 0.149 |
| Myrrha_octodecimguttata_493 | Tytthaspis_sedecimpunctata_595 | 0.149 |
| Myrrha_octodecimguttata_495 | Tytthaspis_sedecimpunctata_595 | 0.149 |
| Myrrha_octodecimguttata_498 | Tytthaspis_sedecimpunctata_595 | 0.149 |
| Scymnus_nebulosus_781 | Scymnus_suturalis_833 | 0.149 |
| Scymnus_nebulosus_781 | Scymnus_suturalis_838 | 0.149 |

Supplementary Table S4 (continued)

| | | |
|---|---|---|
| Scymnus_nebulosus_781 | Scymnus_suturalis_840 | 0.149 |
| Coelophora_inaequalis_318 | Tytthaspis_sedecimpunctata_588 | 0.150 |
| Scymnus_nebulosus_781 | Scymnus_suturalis_845 | 0.150 |
| Calvia_quatuordecimguttata_206 | Tytthaspis_sedecimpunctata_577 | 0.150 |
| Calvia_quatuordecimguttata_209 | Tytthaspis_sedecimpunctata_577 | 0.150 |
| Calvia_quatuordecimguttata_216 | Tytthaspis_sedecimpunctata_577 | 0.150 |
| Calvia_quatuordecimguttata_206 | Tytthaspis_sedecimpunctata_578 | 0.150 |
| Calvia_quatuordecimguttata_209 | Tytthaspis_sedecimpunctata_578 | 0.150 |
| Calvia_quatuordecimguttata_216 | Tytthaspis_sedecimpunctata_578 | 0.150 |
| Calvia_quatuordecimguttata_206 | Tytthaspis_sedecimpunctata_579 | 0.150 |
| Calvia_quatuordecimguttata_209 | Tytthaspis_sedecimpunctata_579 | 0.150 |
| Calvia_quatuordecimguttata_216 | Tytthaspis_sedecimpunctata_579 | 0.150 |
| Myrrha_octodecimguttata_493 | Tytthaspis_sedecimpunctata_580 | 0.150 |
| Myrrha_octodecimguttata_495 | Tytthaspis_sedecimpunctata_580 | 0.150 |
| Myrrha_octodecimguttata_498 | Tytthaspis_sedecimpunctata_580 | 0.150 |
| Calvia_quatuordecimguttata_206 | Tytthaspis_sedecimpunctata_581 | 0.150 |
| Calvia_quatuordecimguttata_209 | Tytthaspis_sedecimpunctata_581 | 0.150 |
| Calvia_quatuordecimguttata_216 | Tytthaspis_sedecimpunctata_581 | 0.150 |
| Calvia_quatuordecimguttata_206 | Tytthaspis_sedecimpunctata_582 | 0.150 |
| Calvia_quatuordecimguttata_209 | Tytthaspis_sedecimpunctata_582 | 0.150 |
| Calvia_quatuordecimguttata_216 | Tytthaspis_sedecimpunctata_582 | 0.150 |
| Calvia_quatuordecimguttata_201 | Tytthaspis_sedecimpunctata_586 | 0.150 |
| Calvia_quatuordecimguttata_202 | Tytthaspis_sedecimpunctata_586 | 0.150 |
| Calvia_quatuordecimguttata_210 | Tytthaspis_sedecimpunctata_586 | 0.150 |
| Myrrha_octodecimguttata_493 | Tytthaspis_sedecimpunctata_586 | 0.150 |
| Myrrha_octodecimguttata_495 | Tytthaspis_sedecimpunctata_586 | 0.150 |
| Myrrha_octodecimguttata_498 | Tytthaspis_sedecimpunctata_586 | 0.150 |
| Calvia_quatuordecimguttata_206 | Tytthaspis_sedecimpunctata_590 | 0.150 |
| Calvia_quatuordecimguttata_209 | Tytthaspis_sedecimpunctata_590 | 0.150 |
| Calvia_quatuordecimguttata_216 | Tytthaspis_sedecimpunctata_590 | 0.150 |
| Calvia_quatuordecimguttata_206 | Tytthaspis_sedecimpunctata_592 | 0.150 |
| Calvia_quatuordecimguttata_209 | Tytthaspis_sedecimpunctata_592 | 0.150 |
| Calvia_quatuordecimguttata_216 | Tytthaspis_sedecimpunctata_592 | 0.150 |
| Calvia_quatuordecimguttata_206 | Tytthaspis_sedecimpunctata_594 | 0.150 |
| Calvia_quatuordecimguttata_209 | Tytthaspis_sedecimpunctata_594 | 0.150 |
| Calvia_quatuordecimguttata_216 | Tytthaspis_sedecimpunctata_594 | 0.150 |
| Calvia_quatuordecimguttata_206 | Tytthaspis_sedecimpunctata_595 | 0.150 |
| Calvia_quatuordecimguttata_209 | Tytthaspis_sedecimpunctata_595 | 0.150 |
| Calvia_quatuordecimguttata_216 | Tytthaspis_sedecimpunctata_595 | 0.150 |
| Coelophora_inaequalis_318 | Tytthaspis_sedecimpunctata_583 | 0.150 |
| Coelophora_inaequalis_318 | Tytthaspis_sedecimpunctata_592 | 0.150 |
| Coelophora_inaequalis_318 | Tytthaspis_sedecimpunctata_580 | 0.150 |
| Coleomegilla_maculata_328 | Tytthaspis_sedecimpunctata_577 | 0.150 |
| Coleomegilla_maculata_332 | Tytthaspis_sedecimpunctata_577 | 0.150 |
| Coleomegilla_maculata_328 | Tytthaspis_sedecimpunctata_578 | 0.150 |
| Coleomegilla_maculata_332 | Tytthaspis_sedecimpunctata_578 | 0.150 |
| Coleomegilla_maculata_328 | Tytthaspis_sedecimpunctata_579 | 0.150 |
| Coleomegilla_maculata_332 | Tytthaspis_sedecimpunctata_579 | 0.150 |
| Calvia_quatuordecimguttata_206 | Tytthaspis_sedecimpunctata_580 | 0.150 |
| Calvia_quatuordecimguttata_216 | Tytthaspis_sedecimpunctata_580 | 0.150 |
| Calvia_quatuordecimguttata_218 | Tytthaspis_sedecimpunctata_580 | 0.150 |
| Coleomegilla_maculata_328 | Tytthaspis_sedecimpunctata_581 | 0.150 |
| Coleomegilla_maculata_332 | Tytthaspis_sedecimpunctata_581 | 0.150 |
| Coleomegilla_maculata_328 | Tytthaspis_sedecimpunctata_582 | 0.150 |
| Coleomegilla_maculata_332 | Tytthaspis_sedecimpunctata_582 | 0.150 |
| Calvia_quatuordecimguttata_207 | Tytthaspis_sedecimpunctata_584 | 0.150 |

Supplementary Table S4 (continued)

| | | |
|---|---|---|
| Calvia_quatuordecimguttata_218 | Tytthaspis_sedecimpunctata_584 | 0.150 |
| Coleomegilla_maculata_328 | Tytthaspis_sedecimpunctata_590 | 0.150 |
| Coleomegilla_maculata_332 | Tytthaspis_sedecimpunctata_590 | 0.150 |
| Coleomegilla_maculata_328 | Tytthaspis_sedecimpunctata_592 | 0.150 |
| Coleomegilla_maculata_332 | Tytthaspis_sedecimpunctata_592 | 0.150 |
| Coleomegilla_maculata_328 | Tytthaspis_sedecimpunctata_594 | 0.150 |
| Coleomegilla_maculata_332 | Tytthaspis_sedecimpunctata_594 | 0.150 |
| Coleomegilla_maculata_328 | Tytthaspis_sedecimpunctata_595 | 0.150 |
| Coleomegilla_maculata_332 | Tytthaspis_sedecimpunctata_595 | 0.150 |
| Calvia_quatuordecimguttata_204 | Tytthaspis_sedecimpunctata_580 | 0.150 |
| Calvia_quatuordecimguttata_208 | Tytthaspis_sedecimpunctata_580 | 0.150 |
| Calvia_quatuordecimguttata_217 | Tytthaspis_sedecimpunctata_580 | 0.150 |
| Coleomegilla_maculata_329 | Tytthaspis_sedecimpunctata_580 | 0.150 |
| Coleomegilla_maculata_330 | Tytthaspis_sedecimpunctata_580 | 0.150 |
| Coleomegilla_maculata_331 | Tytthaspis_sedecimpunctata_580 | 0.150 |
| Coleomegilla_maculata_333 | Tytthaspis_sedecimpunctata_580 | 0.150 |
| Coleomegilla_maculata_334 | Tytthaspis_sedecimpunctata_580 | 0.150 |
| Coleomegilla_maculata_335 | Tytthaspis_sedecimpunctata_580 | 0.150 |
| Coleomegilla_maculata_336 | Tytthaspis_sedecimpunctata_580 | 0.150 |
| Coleomegilla_maculata_337 | Tytthaspis_sedecimpunctata_580 | 0.150 |
| Coleomegilla_maculata_338 | Tytthaspis_sedecimpunctata_580 | 0.150 |
| Coleomegilla_maculata_339 | Tytthaspis_sedecimpunctata_580 | 0.150 |
| Calvia_quatuordecimguttata_204 | Tytthaspis_sedecimpunctata_584 | 0.150 |
| Calvia_quatuordecimguttata_208 | Tytthaspis_sedecimpunctata_584 | 0.150 |
| Calvia_quatuordecimguttata_217 | Tytthaspis_sedecimpunctata_584 | 0.150 |
| Scymnus_abietis_689 | Scymnus_sinuanodulus_829 | 0.150 |
| Scymnus_ferrugatus_724 | Scymnus_suturalis_841 | 0.150 |
| Scymnus_ferrugatus_729 | Scymnus_suturalis_841 | 0.150 |
| Scymnus_ferrugatus_724 | Scymnus_suturalis_842 | 0.150 |
| Scymnus_ferrugatus_729 | Scymnus_suturalis_842 | 0.150 |
| Scymnus_ferrugatus_724 | Scymnus_suturalis_844 | 0.150 |
| Scymnus_ferrugatus_729 | Scymnus_suturalis_844 | 0.150 |
| Scymnus_ferrugatus_724 | Scymnus_suturalis_846 | 0.150 |
| Scymnus_ferrugatus_729 | Scymnus_suturalis_846 | 0.150 |
| Scymnus_ferrugatus_724 | Scymnus_suturalis_847 | 0.150 |
| Scymnus_ferrugatus_729 | Scymnus_suturalis_847 | 0.150 |
| Scymnus_ferrugatus_724 | Scymnus_suturalis_848 | 0.150 |
| Scymnus_ferrugatus_729 | Scymnus_suturalis_848 | 0.150 |
| Scymnus_ferrugatus_724 | Scymnus_suturalis_832 | 0.150 |
| Scymnus_ferrugatus_729 | Scymnus_suturalis_832 | 0.150 |
| Scymnus_ferrugatus_724 | Scymnus_suturalis_835 | 0.150 |
| Scymnus_ferrugatus_729 | Scymnus_suturalis_835 | 0.150 |
| Scymnus_ferrugatus_724 | Scymnus_suturalis_836 | 0.150 |
| Scymnus_ferrugatus_729 | Scymnus_suturalis_836 | 0.150 |
| Scymnus_ferrugatus_724 | Scymnus_suturalis_837 | 0.150 |
| Scymnus_ferrugatus_729 | Scymnus_suturalis_837 | 0.150 |
| Scymnus_ferrugatus_723 | Scymnus_suturalis_838 | 0.150 |
| Scymnus_ferrugatus_725 | Scymnus_suturalis_838 | 0.150 |
| Scymnus_ferrugatus_726 | Scymnus_suturalis_838 | 0.150 |
| Scymnus_ferrugatus_727 | Scymnus_suturalis_838 | 0.150 |
| Scymnus_ferrugatus_728 | Scymnus_suturalis_838 | 0.150 |
| Scymnus_ferrugatus_730 | Scymnus_suturalis_838 | 0.150 |
| Scymnus_ferrugatus_731 | Scymnus_suturalis_838 | 0.150 |
| Scymnus_ferrugatus_732 | Scymnus_suturalis_838 | 0.150 |
| Scymnus_ferrugatus_733 | Scymnus_suturalis_838 | 0.150 |
| Scymnus_ferrugatus_724 | Scymnus_suturalis_839 | 0.150 |

| | | |
|---|---|---|
| Scymnus_ferrugatus_729 | Scymnus_suturalis_839 | 0.150 |
| Scymnus_nebulosus_781 | Scymnus_sinuanodulus_828 | 0.150 |
| Coelophora_inaequalis_319 | Tytthaspis_sedecimpunctata_587 | 0.150 |
| Coelophora_inaequalis_319 | Tytthaspis_sedecimpunctata_589 | 0.150 |
| Coelophora_inaequalis_319 | Tytthaspis_sedecimpunctata_577 | 0.150 |
| Coelophora_inaequalis_319 | Tytthaspis_sedecimpunctata_578 | 0.150 |
| Coelophora_inaequalis_319 | Tytthaspis_sedecimpunctata_579 | 0.150 |
| Coelophora_inaequalis_319 | Tytthaspis_sedecimpunctata_581 | 0.150 |
| Coelophora_inaequalis_319 | Tytthaspis_sedecimpunctata_582 | 0.150 |
| Coelophora_inaequalis_319 | Tytthaspis_sedecimpunctata_590 | 0.150 |
| Coelophora_inaequalis_319 | Tytthaspis_sedecimpunctata_593 | 0.150 |
| Coelophora_inaequalis_319 | Tytthaspis_sedecimpunctata_594 | 0.150 |
| Coelophora_inaequalis_319 | Tytthaspis_sedecimpunctata_595 | 0.150 |
| Scymnus_sinuanodulus_828 | Scymnus_suturalis_843 | 0.150 |
| Scymnus_sinuanodulus_828 | Scymnus_suturalis_845 | 0.150 |
| Anisosticta_novemdecimpunctata_160 | Tytthaspis_sedecimpunctata_589 | 0.150 |
| Anisosticta_novemdecimpunctata_161 | Tytthaspis_sedecimpunctata_589 | 0.150 |
| Anisosticta_novemdecimpunctata_165 | Tytthaspis_sedecimpunctata_589 | 0.150 |
| Anisosticta_novemdecimpunctata_173 | Tytthaspis_sedecimpunctata_589 | 0.150 |
| Scymnus_auritus_701 | Scymnus_suturalis_833 | 0.150 |
| Scymnus_sinuanodulus_828 | Scymnus_suturalis_834 | 0.150 |
| Scymnus_camptodromus_714 | Scymnus_suturalis_838 | 0.150 |
| Scymnus_camptodromus_714 | Scymnus_suturalis_840 | 0.150 |
| Coelophora_inaequalis_319 | Tytthaspis_sedecimpunctata_580 | 0.150 |
| Coelophora_inaequalis_319 | Tytthaspis_sedecimpunctata_584 | 0.150 |
| Scymnus_auritus_701 | Scymnus_suturalis_841 | 0.150 |
| Scymnus_auritus_701 | Scymnus_suturalis_842 | 0.150 |
| Scymnus_auritus_701 | Scymnus_suturalis_844 | 0.150 |
| Scymnus_camptodromus_714 | Scymnus_suturalis_845 | 0.150 |
| Scymnus_auritus_701 | Scymnus_suturalis_846 | 0.150 |
| Scymnus_auritus_701 | Scymnus_suturalis_847 | 0.150 |
| Scymnus_auritus_701 | Scymnus_suturalis_848 | 0.150 |
| Anisosticta_novemdecimpunctata_165 | Tytthaspis_sedecimpunctata_584 | 0.150 |
| Anisosticta_novemdecimpunctata_173 | Tytthaspis_sedecimpunctata_584 | 0.150 |
| Anisosticta_novemdecimpunctata_159 | Tytthaspis_sedecimpunctata_586 | 0.150 |
| Anisosticta_novemdecimpunctata_163 | Tytthaspis_sedecimpunctata_586 | 0.150 |
| Anisosticta_novemdecimpunctata_171 | Tytthaspis_sedecimpunctata_586 | 0.150 |
| Anisosticta_novemdecimpunctata_160 | Tytthaspis_sedecimpunctata_587 | 0.150 |
| Anisosticta_novemdecimpunctata_161 | Tytthaspis_sedecimpunctata_587 | 0.150 |
| Anisosticta_novemdecimpunctata_165 | Tytthaspis_sedecimpunctata_587 | 0.150 |
| Anisosticta_novemdecimpunctata_173 | Tytthaspis_sedecimpunctata_587 | 0.150 |
| Anisosticta_novemdecimpunctata_159 | Tytthaspis_sedecimpunctata_588 | 0.150 |
| Anisosticta_novemdecimpunctata_163 | Tytthaspis_sedecimpunctata_588 | 0.150 |
| Anisosticta_novemdecimpunctata_171 | Tytthaspis_sedecimpunctata_588 | 0.150 |
| Scymnus_nebulosus_782 | Scymnus_sinuanodulus_830 | 0.150 |
| Scymnus_auritus_701 | Scymnus_suturalis_832 | 0.150 |
| Scymnus_auritus_701 | Scymnus_suturalis_835 | 0.150 |
| Scymnus_auritus_701 | Scymnus_suturalis_836 | 0.150 |
| Scymnus_auritus_701 | Scymnus_suturalis_837 | 0.150 |
| Scymnus_sinuanodulus_828 | Scymnus_suturalis_838 | 0.150 |
| Scymnus_auritus_701 | Scymnus_suturalis_839 | 0.150 |
| Anisosticta_novemdecimpunctata_169 | Tytthaspis_sedecimpunctata_577 | 0.150 |
| Anisosticta_novemdecimpunctata_172 | Tytthaspis_sedecimpunctata_577 | 0.150 |
| Anisosticta_novemdecimpunctata_169 | Tytthaspis_sedecimpunctata_578 | 0.150 |
| Anisosticta_novemdecimpunctata_172 | Tytthaspis_sedecimpunctata_578 | 0.150 |
| Anisosticta_novemdecimpunctata_169 | Tytthaspis_sedecimpunctata_579 | 0.150 |

| | | |
|---|---|---|
| Anisosticta_novemdecimpunctata_172 | Tytthaspis_sedecimpunctata_579 | 0.150 |
| Anisosticta_novemdecimpunctata_169 | Tytthaspis_sedecimpunctata_581 | 0.150 |
| Anisosticta_novemdecimpunctata_172 | Tytthaspis_sedecimpunctata_581 | 0.150 |
| Anisosticta_novemdecimpunctata_169 | Tytthaspis_sedecimpunctata_582 | 0.150 |
| Anisosticta_novemdecimpunctata_172 | Tytthaspis_sedecimpunctata_582 | 0.150 |
| Anisosticta_novemdecimpunctata_165 | Tytthaspis_sedecimpunctata_583 | 0.150 |
| Anisosticta_novemdecimpunctata_173 | Tytthaspis_sedecimpunctata_583 | 0.150 |
| Anisosticta_novemdecimpunctata_159 | Tytthaspis_sedecimpunctata_585 | 0.150 |
| Anisosticta_novemdecimpunctata_163 | Tytthaspis_sedecimpunctata_585 | 0.150 |
| Anisosticta_novemdecimpunctata_171 | Tytthaspis_sedecimpunctata_585 | 0.150 |
| Anisosticta_novemdecimpunctata_169 | Tytthaspis_sedecimpunctata_590 | 0.150 |
| Anisosticta_novemdecimpunctata_172 | Tytthaspis_sedecimpunctata_590 | 0.150 |
| Anisosticta_novemdecimpunctata_159 | Tytthaspis_sedecimpunctata_592 | 0.150 |
| Anisosticta_novemdecimpunctata_163 | Tytthaspis_sedecimpunctata_592 | 0.150 |
| Anisosticta_novemdecimpunctata_171 | Tytthaspis_sedecimpunctata_592 | 0.150 |
| Anisosticta_novemdecimpunctata_169 | Tytthaspis_sedecimpunctata_593 | 0.150 |
| Anisosticta_novemdecimpunctata_172 | Tytthaspis_sedecimpunctata_593 | 0.150 |
| Anisosticta_novemdecimpunctata_169 | Tytthaspis_sedecimpunctata_594 | 0.150 |
| Anisosticta_novemdecimpunctata_172 | Tytthaspis_sedecimpunctata_594 | 0.150 |
| Anisosticta_novemdecimpunctata_169 | Tytthaspis_sedecimpunctata_595 | 0.150 |
| Anisosticta_novemdecimpunctata_172 | Tytthaspis_sedecimpunctata_595 | 0.150 |
| Anisosticta_novemdecimpunctata_169 | Tytthaspis_sedecimpunctata_580 | 0.150 |
| Anisosticta_novemdecimpunctata_172 | Tytthaspis_sedecimpunctata_580 | 0.150 |
| Anisosticta_bitriangularis_156 | Tytthaspis_sedecimpunctata_589 | 0.150 |
| Scymnus_nebulosus_782 | Scymnus_sinuanodulus_831 | 0.150 |
| Anisosticta_bitriangularis_156 | Tytthaspis_sedecimpunctata_587 | 0.151 |
| Halyzia_sedecimguttata_345 | Vibidia_duodecimguttata_596 | 0.151 |
| Halyzia_sedecimguttata_349 | Vibidia_duodecimguttata_596 | 0.151 |
| Halyzia_sedecimguttata_350 | Vibidia_duodecimguttata_596 | 0.151 |
| Halyzia_sedecimguttata_345 | Vibidia_duodecimguttata_598 | 0.151 |
| Halyzia_sedecimguttata_349 | Vibidia_duodecimguttata_598 | 0.151 |
| Halyzia_sedecimguttata_350 | Vibidia_duodecimguttata_598 | 0.151 |
| Scymnus_abietis_689 | Scymnus_sinuanodulus_828 | 0.151 |
| Brumoides_suturalis_2 | Tytthaspis_sedecimpunctata_588 | 0.151 |
| Harmonia_conformis_374 | Tytthaspis_sedecimpunctata_577 | 0.151 |
| Harmonia_conformis_374 | Tytthaspis_sedecimpunctata_578 | 0.151 |
| Harmonia_conformis_374 | Tytthaspis_sedecimpunctata_579 | 0.151 |
| Harmonia_conformis_374 | Tytthaspis_sedecimpunctata_581 | 0.151 |
| Harmonia_conformis_374 | Tytthaspis_sedecimpunctata_582 | 0.151 |
| Harmonia_conformis_365 | Tytthaspis_sedecimpunctata_583 | 0.151 |
| Harmonia_conformis_366 | Tytthaspis_sedecimpunctata_583 | 0.151 |
| Harmonia_conformis_367 | Tytthaspis_sedecimpunctata_583 | 0.151 |
| Harmonia_conformis_368 | Tytthaspis_sedecimpunctata_583 | 0.151 |
| Harmonia_conformis_369 | Tytthaspis_sedecimpunctata_583 | 0.151 |
| Harmonia_conformis_370 | Tytthaspis_sedecimpunctata_583 | 0.151 |
| Harmonia_conformis_371 | Tytthaspis_sedecimpunctata_583 | 0.151 |
| Harmonia_conformis_372 | Tytthaspis_sedecimpunctata_583 | 0.151 |
| Harmonia_conformis_373 | Tytthaspis_sedecimpunctata_583 | 0.151 |
| Harmonia_conformis_375 | Tytthaspis_sedecimpunctata_583 | 0.151 |
| Harmonia_conformis_374 | Tytthaspis_sedecimpunctata_590 | 0.151 |
| Harmonia_conformis_374 | Tytthaspis_sedecimpunctata_592 | 0.151 |
| Harmonia_conformis_374 | Tytthaspis_sedecimpunctata_594 | 0.151 |
| Harmonia_conformis_374 | Tytthaspis_sedecimpunctata_595 | 0.151 |
| Harmonia_conformis_374 | Tytthaspis_sedecimpunctata_584 | 0.151 |
| Mulsantina_hudsonica_481 | Tytthaspis_sedecimpunctata_586 | 0.151 |
| Mulsantina_hudsonica_482 | Tytthaspis_sedecimpunctata_586 | 0.151 |

| | | |
|---|---|---|
| Mulsantina_hudsonica_481 | Tytthaspis_sedecimpunctata_587 | 0.151 |
| Mulsantina_hudsonica_482 | Tytthaspis_sedecimpunctata_587 | 0.151 |
| Mulsantina_hudsonica_481 | Tytthaspis_sedecimpunctata_588 | 0.151 |
| Mulsantina_hudsonica_482 | Tytthaspis_sedecimpunctata_588 | 0.151 |
| Mulsantina_hudsonica_483 | Tytthaspis_sedecimpunctata_577 | 0.151 |
| Mulsantina_hudsonica_483 | Tytthaspis_sedecimpunctata_578 | 0.151 |
| Mulsantina_hudsonica_483 | Tytthaspis_sedecimpunctata_579 | 0.151 |
| Mulsantina_hudsonica_483 | Tytthaspis_sedecimpunctata_581 | 0.151 |
| Mulsantina_hudsonica_483 | Tytthaspis_sedecimpunctata_582 | 0.151 |
| Mulsantina_hudsonica_480 | Tytthaspis_sedecimpunctata_583 | 0.151 |
| Mulsantina_hudsonica_481 | Tytthaspis_sedecimpunctata_585 | 0.151 |
| Mulsantina_hudsonica_482 | Tytthaspis_sedecimpunctata_585 | 0.151 |
| Mulsantina_hudsonica_483 | Tytthaspis_sedecimpunctata_589 | 0.151 |
| Mulsantina_hudsonica_483 | Tytthaspis_sedecimpunctata_590 | 0.151 |
| Mulsantina_hudsonica_483 | Tytthaspis_sedecimpunctata_592 | 0.151 |
| Mulsantina_hudsonica_483 | Tytthaspis_sedecimpunctata_593 | 0.151 |
| Mulsantina_hudsonica_483 | Tytthaspis_sedecimpunctata_594 | 0.151 |
| Mulsantina_hudsonica_483 | Tytthaspis_sedecimpunctata_595 | 0.151 |
| Scymnus_interruptus_760 | Scymnus_suturalis_833 | 0.151 |
| Scymnus_interruptus_765 | Scymnus_suturalis_833 | 0.151 |
| Scymnus_interruptus_767 | Scymnus_suturalis_833 | 0.151 |
| Scymnus_interruptus_768 | Scymnus_suturalis_833 | 0.151 |
| Scymnus_interruptus_760 | Scymnus_suturalis_843 | 0.151 |
| Scymnus_interruptus_765 | Scymnus_suturalis_843 | 0.151 |
| Scymnus_interruptus_767 | Scymnus_suturalis_843 | 0.151 |
| Scymnus_interruptus_768 | Scymnus_suturalis_843 | 0.151 |
| Scymnus_interruptus_760 | Scymnus_suturalis_845 | 0.151 |
| Scymnus_interruptus_765 | Scymnus_suturalis_845 | 0.151 |
| Scymnus_interruptus_767 | Scymnus_suturalis_845 | 0.151 |
| Scymnus_interruptus_768 | Scymnus_suturalis_845 | 0.151 |
| Mulsantina_hudsonica_480 | Tytthaspis_sedecimpunctata_580 | 0.151 |
| Coelophora_inaequalis_310 | Tytthaspis_sedecimpunctata_589 | 0.151 |
| Coelophora_inaequalis_312 | Tytthaspis_sedecimpunctata_589 | 0.151 |
| Coelophora_inaequalis_314 | Tytthaspis_sedecimpunctata_589 | 0.151 |
| Coelophora_inaequalis_323 | Tytthaspis_sedecimpunctata_589 | 0.151 |
| Coelophora_inaequalis_325 | Tytthaspis_sedecimpunctata_589 | 0.151 |
| Scymnus_interruptus_760 | Scymnus_suturalis_837 | 0.151 |
| Scymnus_interruptus_765 | Scymnus_suturalis_837 | 0.151 |
| Scymnus_interruptus_767 | Scymnus_suturalis_837 | 0.151 |
| Scymnus_interruptus_768 | Scymnus_suturalis_837 | 0.151 |
| Scymnus_camptodromus_715 | Scymnus_rubromaculatus_822 | 0.151 |
| Scymnus_camptodromus_715 | Scymnus_rubromaculatus_823 | 0.151 |
| Scymnus_camptodromus_715 | Scymnus_rubromaculatus_824 | 0.151 |
| Scymnus_camptodromus_715 | Scymnus_rubromaculatus_825 | 0.151 |
| Scymnus_camptodromus_715 | Scymnus_rubromaculatus_826 | 0.151 |
| Scymnus_camptodromus_715 | Scymnus_rubromaculatus_827 | 0.151 |
| Coelophora_inaequalis_311 | Tytthaspis_sedecimpunctata_586 | 0.151 |
| Coelophora_inaequalis_313 | Tytthaspis_sedecimpunctata_586 | 0.151 |
| Coelophora_inaequalis_316 | Tytthaspis_sedecimpunctata_586 | 0.151 |
| Coelophora_inaequalis_324 | Tytthaspis_sedecimpunctata_586 | 0.151 |
| Coelophora_inaequalis_326 | Tytthaspis_sedecimpunctata_586 | 0.151 |
| Coelophora_inaequalis_310 | Tytthaspis_sedecimpunctata_587 | 0.151 |
| Coelophora_inaequalis_312 | Tytthaspis_sedecimpunctata_587 | 0.151 |
| Coelophora_inaequalis_314 | Tytthaspis_sedecimpunctata_587 | 0.151 |
| Coelophora_inaequalis_323 | Tytthaspis_sedecimpunctata_587 | 0.151 |
| Coelophora_inaequalis_325 | Tytthaspis_sedecimpunctata_587 | 0.151 |

| | | |
|---|---|---|
| Coelophora_inaequalis_317 | Tytthaspis_sedecimpunctata_588 | 0.151 |
| Coelophora_inaequalis_311 | Tytthaspis_sedecimpunctata_577 | 0.151 |
| Coelophora_inaequalis_313 | Tytthaspis_sedecimpunctata_577 | 0.151 |
| Coelophora_inaequalis_316 | Tytthaspis_sedecimpunctata_577 | 0.151 |
| Coelophora_inaequalis_324 | Tytthaspis_sedecimpunctata_577 | 0.151 |
| Coelophora_inaequalis_326 | Tytthaspis_sedecimpunctata_577 | 0.151 |
| Coelophora_inaequalis_311 | Tytthaspis_sedecimpunctata_578 | 0.151 |
| Coelophora_inaequalis_313 | Tytthaspis_sedecimpunctata_578 | 0.151 |
| Coelophora_inaequalis_316 | Tytthaspis_sedecimpunctata_578 | 0.151 |
| Coelophora_inaequalis_324 | Tytthaspis_sedecimpunctata_578 | 0.151 |
| Coelophora_inaequalis_326 | Tytthaspis_sedecimpunctata_578 | 0.151 |
| Coelophora_inaequalis_311 | Tytthaspis_sedecimpunctata_579 | 0.151 |
| Coelophora_inaequalis_313 | Tytthaspis_sedecimpunctata_579 | 0.151 |
| Coelophora_inaequalis_316 | Tytthaspis_sedecimpunctata_579 | 0.151 |
| Coelophora_inaequalis_324 | Tytthaspis_sedecimpunctata_579 | 0.151 |
| Coelophora_inaequalis_326 | Tytthaspis_sedecimpunctata_579 | 0.151 |
| Coelophora_inaequalis_311 | Tytthaspis_sedecimpunctata_581 | 0.151 |
| Coelophora_inaequalis_313 | Tytthaspis_sedecimpunctata_581 | 0.151 |
| Coelophora_inaequalis_316 | Tytthaspis_sedecimpunctata_581 | 0.151 |
| Coelophora_inaequalis_324 | Tytthaspis_sedecimpunctata_581 | 0.151 |
| Coelophora_inaequalis_326 | Tytthaspis_sedecimpunctata_581 | 0.151 |
| Coelophora_inaequalis_311 | Tytthaspis_sedecimpunctata_582 | 0.151 |
| Coelophora_inaequalis_313 | Tytthaspis_sedecimpunctata_582 | 0.151 |
| Coelophora_inaequalis_316 | Tytthaspis_sedecimpunctata_582 | 0.151 |
| Coelophora_inaequalis_324 | Tytthaspis_sedecimpunctata_582 | 0.151 |
| Coelophora_inaequalis_326 | Tytthaspis_sedecimpunctata_582 | 0.151 |
| Coelophora_inaequalis_317 | Tytthaspis_sedecimpunctata_583 | 0.151 |
| Hippodamia_variegata_467 | Tytthaspis_sedecimpunctata_584 | 0.151 |
| Hippodamia_variegata_469 | Tytthaspis_sedecimpunctata_584 | 0.151 |
| Hippodamia_variegata_470 | Tytthaspis_sedecimpunctata_584 | 0.151 |
| Hippodamia_variegata_472 | Tytthaspis_sedecimpunctata_584 | 0.151 |
| Hippodamia_variegata_473 | Tytthaspis_sedecimpunctata_584 | 0.151 |
| Coelophora_inaequalis_315 | Tytthaspis_sedecimpunctata_586 | 0.151 |
| Hippodamia_variegata_467 | Tytthaspis_sedecimpunctata_586 | 0.151 |
| Hippodamia_variegata_469 | Tytthaspis_sedecimpunctata_586 | 0.151 |
| Hippodamia_variegata_470 | Tytthaspis_sedecimpunctata_586 | 0.151 |
| Hippodamia_variegata_472 | Tytthaspis_sedecimpunctata_586 | 0.151 |
| Hippodamia_variegata_473 | Tytthaspis_sedecimpunctata_586 | 0.151 |
| Hippodamia_variegata_467 | Tytthaspis_sedecimpunctata_587 | 0.151 |
| Hippodamia_variegata_469 | Tytthaspis_sedecimpunctata_587 | 0.151 |
| Hippodamia_variegata_470 | Tytthaspis_sedecimpunctata_587 | 0.151 |
| Hippodamia_variegata_472 | Tytthaspis_sedecimpunctata_587 | 0.151 |
| Hippodamia_variegata_473 | Tytthaspis_sedecimpunctata_587 | 0.151 |
| Coelophora_inaequalis_311 | Tytthaspis_sedecimpunctata_590 | 0.151 |
| Coelophora_inaequalis_313 | Tytthaspis_sedecimpunctata_590 | 0.151 |
| Coelophora_inaequalis_316 | Tytthaspis_sedecimpunctata_590 | 0.151 |
| Coelophora_inaequalis_324 | Tytthaspis_sedecimpunctata_590 | 0.151 |
| Coelophora_inaequalis_326 | Tytthaspis_sedecimpunctata_590 | 0.151 |
| Coelophora_inaequalis_317 | Tytthaspis_sedecimpunctata_592 | 0.151 |
| Coelophora_inaequalis_311 | Tytthaspis_sedecimpunctata_593 | 0.151 |
| Coelophora_inaequalis_313 | Tytthaspis_sedecimpunctata_593 | 0.151 |
| Coelophora_inaequalis_316 | Tytthaspis_sedecimpunctata_593 | 0.151 |
| Coelophora_inaequalis_324 | Tytthaspis_sedecimpunctata_593 | 0.151 |
| Coelophora_inaequalis_326 | Tytthaspis_sedecimpunctata_593 | 0.151 |
| Coelophora_inaequalis_311 | Tytthaspis_sedecimpunctata_594 | 0.151 |
| Coelophora_inaequalis_313 | Tytthaspis_sedecimpunctata_594 | 0.151 |

| | | |
|---|---|---|
| Coelophora_inaequalis_316 | Tytthaspis_sedecimpunctata_594 | 0.151 |
| Coelophora_inaequalis_324 | Tytthaspis_sedecimpunctata_594 | 0.151 |
| Coelophora_inaequalis_326 | Tytthaspis_sedecimpunctata_594 | 0.151 |
| Coelophora_inaequalis_311 | Tytthaspis_sedecimpunctata_595 | 0.151 |
| Coelophora_inaequalis_313 | Tytthaspis_sedecimpunctata_595 | 0.151 |
| Coelophora_inaequalis_316 | Tytthaspis_sedecimpunctata_595 | 0.151 |
| Coelophora_inaequalis_324 | Tytthaspis_sedecimpunctata_595 | 0.151 |
| Coelophora_inaequalis_326 | Tytthaspis_sedecimpunctata_595 | 0.151 |
| Coelophora_inaequalis_315 | Tytthaspis_sedecimpunctata_577 | 0.151 |
| Coelophora_inaequalis_315 | Tytthaspis_sedecimpunctata_578 | 0.151 |
| Coelophora_inaequalis_315 | Tytthaspis_sedecimpunctata_579 | 0.151 |
| Coelophora_inaequalis_311 | Tytthaspis_sedecimpunctata_580 | 0.151 |
| Coelophora_inaequalis_313 | Tytthaspis_sedecimpunctata_580 | 0.151 |
| Coelophora_inaequalis_316 | Tytthaspis_sedecimpunctata_580 | 0.151 |
| Coelophora_inaequalis_317 | Tytthaspis_sedecimpunctata_580 | 0.151 |
| Coelophora_inaequalis_324 | Tytthaspis_sedecimpunctata_580 | 0.151 |
| Coelophora_inaequalis_326 | Tytthaspis_sedecimpunctata_580 | 0.151 |
| Coelophora_inaequalis_315 | Tytthaspis_sedecimpunctata_581 | 0.151 |
| Coelophora_inaequalis_315 | Tytthaspis_sedecimpunctata_582 | 0.151 |
| Coelophora_inaequalis_311 | Tytthaspis_sedecimpunctata_584 | 0.151 |
| Coelophora_inaequalis_313 | Tytthaspis_sedecimpunctata_584 | 0.151 |
| Coelophora_inaequalis_316 | Tytthaspis_sedecimpunctata_584 | 0.151 |
| Coelophora_inaequalis_324 | Tytthaspis_sedecimpunctata_584 | 0.151 |
| Coelophora_inaequalis_326 | Tytthaspis_sedecimpunctata_584 | 0.151 |
| Coelophora_inaequalis_315 | Tytthaspis_sedecimpunctata_590 | 0.151 |
| Coelophora_inaequalis_315 | Tytthaspis_sedecimpunctata_593 | 0.151 |
| Hippodamia_variegata_467 | Tytthaspis_sedecimpunctata_593 | 0.151 |
| Hippodamia_variegata_469 | Tytthaspis_sedecimpunctata_593 | 0.151 |
| Hippodamia_variegata_470 | Tytthaspis_sedecimpunctata_593 | 0.151 |
| Hippodamia_variegata_472 | Tytthaspis_sedecimpunctata_593 | 0.151 |
| Hippodamia_variegata_473 | Tytthaspis_sedecimpunctata_593 | 0.151 |
| Coelophora_inaequalis_315 | Tytthaspis_sedecimpunctata_594 | 0.151 |
| Coelophora_inaequalis_315 | Tytthaspis_sedecimpunctata_595 | 0.151 |
| Coelophora_inaequalis_315 | Tytthaspis_sedecimpunctata_584 | 0.151 |
| Myrrha_octodecimguttata_499 | Tytthaspis_sedecimpunctata_585 | 0.151 |
| Myrrha_octodecimguttata_499 | Tytthaspis_sedecimpunctata_593 | 0.151 |
| Myrrha_octodecimguttata_485 | Tytthaspis_sedecimpunctata_577 | 0.151 |
| Myrrha_octodecimguttata_486 | Tytthaspis_sedecimpunctata_577 | 0.151 |
| Myrrha_octodecimguttata_487 | Tytthaspis_sedecimpunctata_577 | 0.151 |
| Myrrha_octodecimguttata_490 | Tytthaspis_sedecimpunctata_577 | 0.151 |
| Myrrha_octodecimguttata_494 | Tytthaspis_sedecimpunctata_577 | 0.151 |
| Myrrha_octodecimguttata_496 | Tytthaspis_sedecimpunctata_577 | 0.151 |
| Myrrha_octodecimguttata_497 | Tytthaspis_sedecimpunctata_577 | 0.151 |
| Myrrha_octodecimguttata_501 | Tytthaspis_sedecimpunctata_577 | 0.151 |
| Myrrha_octodecimguttata_502 | Tytthaspis_sedecimpunctata_577 | 0.151 |
| Myrrha_octodecimguttata_485 | Tytthaspis_sedecimpunctata_578 | 0.151 |
| Myrrha_octodecimguttata_486 | Tytthaspis_sedecimpunctata_578 | 0.151 |
| Myrrha_octodecimguttata_487 | Tytthaspis_sedecimpunctata_578 | 0.151 |
| Myrrha_octodecimguttata_490 | Tytthaspis_sedecimpunctata_578 | 0.151 |
| Myrrha_octodecimguttata_494 | Tytthaspis_sedecimpunctata_578 | 0.151 |
| Myrrha_octodecimguttata_496 | Tytthaspis_sedecimpunctata_578 | 0.151 |
| Myrrha_octodecimguttata_497 | Tytthaspis_sedecimpunctata_578 | 0.151 |
| Myrrha_octodecimguttata_501 | Tytthaspis_sedecimpunctata_578 | 0.151 |
| Myrrha_octodecimguttata_502 | Tytthaspis_sedecimpunctata_578 | 0.151 |
| Myrrha_octodecimguttata_485 | Tytthaspis_sedecimpunctata_579 | 0.151 |
| Myrrha_octodecimguttata_486 | Tytthaspis_sedecimpunctata_579 | 0.151 |

| | | |
|---|---|---|
| Myrrha_octodecimguttata_487 | Tytthaspis_sedecimpunctata_579 | 0.151 |
| Myrrha_octodecimguttata_490 | Tytthaspis_sedecimpunctata_579 | 0.151 |
| Myrrha_octodecimguttata_494 | Tytthaspis_sedecimpunctata_579 | 0.151 |
| Myrrha_octodecimguttata_496 | Tytthaspis_sedecimpunctata_579 | 0.151 |
| Myrrha_octodecimguttata_497 | Tytthaspis_sedecimpunctata_579 | 0.151 |
| Myrrha_octodecimguttata_501 | Tytthaspis_sedecimpunctata_579 | 0.151 |
| Myrrha_octodecimguttata_502 | Tytthaspis_sedecimpunctata_579 | 0.151 |
| Myrrha_octodecimguttata_485 | Tytthaspis_sedecimpunctata_581 | 0.151 |
| Myrrha_octodecimguttata_486 | Tytthaspis_sedecimpunctata_581 | 0.151 |
| Myrrha_octodecimguttata_487 | Tytthaspis_sedecimpunctata_581 | 0.151 |
| Myrrha_octodecimguttata_490 | Tytthaspis_sedecimpunctata_581 | 0.151 |
| Myrrha_octodecimguttata_494 | Tytthaspis_sedecimpunctata_581 | 0.151 |
| Myrrha_octodecimguttata_496 | Tytthaspis_sedecimpunctata_581 | 0.151 |
| Myrrha_octodecimguttata_497 | Tytthaspis_sedecimpunctata_581 | 0.151 |
| Myrrha_octodecimguttata_501 | Tytthaspis_sedecimpunctata_581 | 0.151 |
| Myrrha_octodecimguttata_502 | Tytthaspis_sedecimpunctata_581 | 0.151 |
| Myrrha_octodecimguttata_485 | Tytthaspis_sedecimpunctata_582 | 0.151 |
| Myrrha_octodecimguttata_486 | Tytthaspis_sedecimpunctata_582 | 0.151 |
| Myrrha_octodecimguttata_487 | Tytthaspis_sedecimpunctata_582 | 0.151 |
| Myrrha_octodecimguttata_490 | Tytthaspis_sedecimpunctata_582 | 0.151 |
| Myrrha_octodecimguttata_494 | Tytthaspis_sedecimpunctata_582 | 0.151 |
| Myrrha_octodecimguttata_496 | Tytthaspis_sedecimpunctata_582 | 0.151 |
| Myrrha_octodecimguttata_497 | Tytthaspis_sedecimpunctata_582 | 0.151 |
| Myrrha_octodecimguttata_501 | Tytthaspis_sedecimpunctata_582 | 0.151 |
| Myrrha_octodecimguttata_502 | Tytthaspis_sedecimpunctata_582 | 0.151 |
| Myrrha_octodecimguttata_489 | Tytthaspis_sedecimpunctata_587 | 0.151 |
| Myrrha_octodecimguttata_499 | Tytthaspis_sedecimpunctata_587 | 0.151 |
| Myrrha_octodecimguttata_500 | Tytthaspis_sedecimpunctata_587 | 0.151 |
| Myrrha_octodecimguttata_485 | Tytthaspis_sedecimpunctata_590 | 0.151 |
| Myrrha_octodecimguttata_486 | Tytthaspis_sedecimpunctata_590 | 0.151 |
| Myrrha_octodecimguttata_487 | Tytthaspis_sedecimpunctata_590 | 0.151 |
| Myrrha_octodecimguttata_490 | Tytthaspis_sedecimpunctata_590 | 0.151 |
| Myrrha_octodecimguttata_494 | Tytthaspis_sedecimpunctata_590 | 0.151 |
| Myrrha_octodecimguttata_496 | Tytthaspis_sedecimpunctata_590 | 0.151 |
| Myrrha_octodecimguttata_497 | Tytthaspis_sedecimpunctata_590 | 0.151 |
| Myrrha_octodecimguttata_501 | Tytthaspis_sedecimpunctata_590 | 0.151 |
| Myrrha_octodecimguttata_502 | Tytthaspis_sedecimpunctata_590 | 0.151 |
| Myrrha_octodecimguttata_485 | Tytthaspis_sedecimpunctata_594 | 0.151 |
| Myrrha_octodecimguttata_486 | Tytthaspis_sedecimpunctata_594 | 0.151 |
| Myrrha_octodecimguttata_487 | Tytthaspis_sedecimpunctata_594 | 0.151 |
| Myrrha_octodecimguttata_490 | Tytthaspis_sedecimpunctata_594 | 0.151 |
| Myrrha_octodecimguttata_494 | Tytthaspis_sedecimpunctata_594 | 0.151 |
| Myrrha_octodecimguttata_496 | Tytthaspis_sedecimpunctata_594 | 0.151 |
| Myrrha_octodecimguttata_497 | Tytthaspis_sedecimpunctata_594 | 0.151 |
| Myrrha_octodecimguttata_501 | Tytthaspis_sedecimpunctata_594 | 0.151 |
| Myrrha_octodecimguttata_502 | Tytthaspis_sedecimpunctata_594 | 0.151 |
| Myrrha_octodecimguttata_485 | Tytthaspis_sedecimpunctata_595 | 0.151 |
| Myrrha_octodecimguttata_486 | Tytthaspis_sedecimpunctata_595 | 0.151 |
| Myrrha_octodecimguttata_487 | Tytthaspis_sedecimpunctata_595 | 0.151 |
| Myrrha_octodecimguttata_490 | Tytthaspis_sedecimpunctata_595 | 0.151 |
| Myrrha_octodecimguttata_494 | Tytthaspis_sedecimpunctata_595 | 0.151 |
| Myrrha_octodecimguttata_496 | Tytthaspis_sedecimpunctata_595 | 0.151 |
| Myrrha_octodecimguttata_497 | Tytthaspis_sedecimpunctata_595 | 0.151 |
| Myrrha_octodecimguttata_501 | Tytthaspis_sedecimpunctata_595 | 0.151 |
| Myrrha_octodecimguttata_502 | Tytthaspis_sedecimpunctata_595 | 0.151 |
| Scymnus_rubromaculatus_813 | Scymnus_suturalis_843 | 0.151 |

Supplementary Table S4 (continued)

| | | |
|---|---|---|
| Scymnus_rubromaculatus_815 | Scymnus_suturalis_843 | 0.151 |
| Scymnus_rubromaculatus_816 | Scymnus_suturalis_843 | 0.151 |
| Scymnus_rubromaculatus_817 | Scymnus_suturalis_843 | 0.151 |
| Scymnus_rubromaculatus_818 | Scymnus_suturalis_843 | 0.151 |
| Scymnus_rubromaculatus_819 | Scymnus_suturalis_843 | 0.151 |
| Scymnus_rubromaculatus_820 | Scymnus_suturalis_843 | 0.151 |
| Scymnus_rubromaculatus_821 | Scymnus_suturalis_843 | 0.151 |
| Scymnus_rubromaculatus_822 | Scymnus_suturalis_843 | 0.151 |
| Scymnus_rubromaculatus_823 | Scymnus_suturalis_843 | 0.151 |
| Scymnus_rubromaculatus_824 | Scymnus_suturalis_843 | 0.151 |
| Scymnus_rubromaculatus_825 | Scymnus_suturalis_843 | 0.151 |
| Scymnus_rubromaculatus_826 | Scymnus_suturalis_843 | 0.151 |
| Scymnus_rubromaculatus_827 | Scymnus_suturalis_843 | 0.151 |
| Scymnus_rubromaculatus_813 | Scymnus_suturalis_845 | 0.151 |
| Scymnus_rubromaculatus_815 | Scymnus_suturalis_845 | 0.151 |
| Scymnus_rubromaculatus_816 | Scymnus_suturalis_845 | 0.151 |
| Scymnus_rubromaculatus_817 | Scymnus_suturalis_845 | 0.151 |
| Scymnus_rubromaculatus_818 | Scymnus_suturalis_845 | 0.151 |
| Scymnus_rubromaculatus_819 | Scymnus_suturalis_845 | 0.151 |
| Scymnus_rubromaculatus_820 | Scymnus_suturalis_845 | 0.151 |
| Scymnus_rubromaculatus_821 | Scymnus_suturalis_845 | 0.151 |
| Scymnus_rubromaculatus_822 | Scymnus_suturalis_845 | 0.151 |
| Scymnus_rubromaculatus_823 | Scymnus_suturalis_845 | 0.151 |
| Scymnus_rubromaculatus_824 | Scymnus_suturalis_845 | 0.151 |
| Scymnus_rubromaculatus_825 | Scymnus_suturalis_845 | 0.151 |
| Scymnus_rubromaculatus_826 | Scymnus_suturalis_845 | 0.151 |
| Scymnus_rubromaculatus_827 | Scymnus_suturalis_845 | 0.151 |
| Myrrha_octodecimguttata_485 | Tytthaspis_sedecimpunctata_580 | 0.151 |
| Myrrha_octodecimguttata_486 | Tytthaspis_sedecimpunctata_580 | 0.151 |
| Myrrha_octodecimguttata_487 | Tytthaspis_sedecimpunctata_580 | 0.151 |
| Myrrha_octodecimguttata_490 | Tytthaspis_sedecimpunctata_580 | 0.151 |
| Myrrha_octodecimguttata_494 | Tytthaspis_sedecimpunctata_580 | 0.151 |
| Myrrha_octodecimguttata_496 | Tytthaspis_sedecimpunctata_580 | 0.151 |
| Myrrha_octodecimguttata_497 | Tytthaspis_sedecimpunctata_580 | 0.151 |
| Myrrha_octodecimguttata_501 | Tytthaspis_sedecimpunctata_580 | 0.151 |
| Myrrha_octodecimguttata_502 | Tytthaspis_sedecimpunctata_580 | 0.151 |
| Myrrha_octodecimguttata_493 | Tytthaspis_sedecimpunctata_585 | 0.151 |
| Myrrha_octodecimguttata_495 | Tytthaspis_sedecimpunctata_585 | 0.151 |
| Myrrha_octodecimguttata_498 | Tytthaspis_sedecimpunctata_585 | 0.151 |
| Myrrha_octodecimguttata_485 | Tytthaspis_sedecimpunctata_586 | 0.151 |
| Myrrha_octodecimguttata_486 | Tytthaspis_sedecimpunctata_586 | 0.151 |
| Myrrha_octodecimguttata_487 | Tytthaspis_sedecimpunctata_586 | 0.151 |
| Myrrha_octodecimguttata_490 | Tytthaspis_sedecimpunctata_586 | 0.151 |
| Myrrha_octodecimguttata_494 | Tytthaspis_sedecimpunctata_586 | 0.151 |
| Myrrha_octodecimguttata_496 | Tytthaspis_sedecimpunctata_586 | 0.151 |
| Myrrha_octodecimguttata_497 | Tytthaspis_sedecimpunctata_586 | 0.151 |
| Myrrha_octodecimguttata_501 | Tytthaspis_sedecimpunctata_586 | 0.151 |
| Myrrha_octodecimguttata_502 | Tytthaspis_sedecimpunctata_586 | 0.151 |
| Myrrha_octodecimguttata_493 | Tytthaspis_sedecimpunctata_593 | 0.151 |
| Myrrha_octodecimguttata_495 | Tytthaspis_sedecimpunctata_593 | 0.151 |
| Myrrha_octodecimguttata_498 | Tytthaspis_sedecimpunctata_593 | 0.151 |
| Scymnus_rubromaculatus_813 | Scymnus_suturalis_837 | 0.151 |
| Scymnus_rubromaculatus_815 | Scymnus_suturalis_837 | 0.151 |
| Scymnus_rubromaculatus_816 | Scymnus_suturalis_837 | 0.151 |
| Scymnus_rubromaculatus_817 | Scymnus_suturalis_837 | 0.151 |
| Scymnus_rubromaculatus_818 | Scymnus_suturalis_837 | 0.151 |

| | | |
|---|---|---|
| Scymnus_rubromaculatus_819 | Scymnus_suturalis_837 | 0.151 |
| Scymnus_rubromaculatus_820 | Scymnus_suturalis_837 | 0.151 |
| Scymnus_rubromaculatus_821 | Scymnus_suturalis_837 | 0.151 |
| Scymnus_rubromaculatus_822 | Scymnus_suturalis_837 | 0.151 |
| Scymnus_rubromaculatus_823 | Scymnus_suturalis_837 | 0.151 |
| Scymnus_rubromaculatus_824 | Scymnus_suturalis_837 | 0.151 |
| Scymnus_rubromaculatus_825 | Scymnus_suturalis_837 | 0.151 |
| Scymnus_rubromaculatus_826 | Scymnus_suturalis_837 | 0.151 |
| Scymnus_rubromaculatus_827 | Scymnus_suturalis_837 | 0.151 |
| Coelophora_inaequalis_318 | Tytthaspis_sedecimpunctata_586 | 0.151 |
| Coelophora_inaequalis_318 | Tytthaspis_sedecimpunctata_577 | 0.151 |
| Coelophora_inaequalis_318 | Tytthaspis_sedecimpunctata_578 | 0.151 |
| Coelophora_inaequalis_318 | Tytthaspis_sedecimpunctata_579 | 0.151 |
| Coelophora_inaequalis_318 | Tytthaspis_sedecimpunctata_581 | 0.151 |
| Coelophora_inaequalis_318 | Tytthaspis_sedecimpunctata_582 | 0.151 |
| Coelophora_inaequalis_318 | Tytthaspis_sedecimpunctata_590 | 0.151 |
| Coelophora_inaequalis_318 | Tytthaspis_sedecimpunctata_593 | 0.151 |
| Coelophora_inaequalis_318 | Tytthaspis_sedecimpunctata_594 | 0.151 |
| Coelophora_inaequalis_318 | Tytthaspis_sedecimpunctata_595 | 0.151 |
| Calvia_quatuordecimguttata_207 | Tytthaspis_sedecimpunctata_577 | 0.151 |
| Calvia_quatuordecimguttata_218 | Tytthaspis_sedecimpunctata_577 | 0.151 |
| Calvia_quatuordecimguttata_207 | Tytthaspis_sedecimpunctata_578 | 0.151 |
| Calvia_quatuordecimguttata_218 | Tytthaspis_sedecimpunctata_578 | 0.151 |
| Calvia_quatuordecimguttata_207 | Tytthaspis_sedecimpunctata_579 | 0.151 |
| Calvia_quatuordecimguttata_218 | Tytthaspis_sedecimpunctata_579 | 0.151 |
| Calvia_quatuordecimguttata_207 | Tytthaspis_sedecimpunctata_581 | 0.151 |
| Calvia_quatuordecimguttata_218 | Tytthaspis_sedecimpunctata_581 | 0.151 |
| Calvia_quatuordecimguttata_207 | Tytthaspis_sedecimpunctata_582 | 0.151 |
| Calvia_quatuordecimguttata_218 | Tytthaspis_sedecimpunctata_582 | 0.151 |
| Calvia_quatuordecimguttata_206 | Tytthaspis_sedecimpunctata_583 | 0.151 |
| Calvia_quatuordecimguttata_209 | Tytthaspis_sedecimpunctata_583 | 0.151 |
| Calvia_quatuordecimguttata_216 | Tytthaspis_sedecimpunctata_583 | 0.151 |
| Calvia_quatuordecimguttata_206 | Tytthaspis_sedecimpunctata_585 | 0.151 |
| Calvia_quatuordecimguttata_216 | Tytthaspis_sedecimpunctata_585 | 0.151 |
| Calvia_quatuordecimguttata_211 | Tytthaspis_sedecimpunctata_587 | 0.151 |
| Calvia_quatuordecimguttata_212 | Tytthaspis_sedecimpunctata_587 | 0.151 |
| Calvia_quatuordecimguttata_213 | Tytthaspis_sedecimpunctata_587 | 0.151 |
| Calvia_quatuordecimguttata_214 | Tytthaspis_sedecimpunctata_587 | 0.151 |
| Calvia_quatuordecimguttata_215 | Tytthaspis_sedecimpunctata_587 | 0.151 |
| Myrrha_octodecimguttata_493 | Tytthaspis_sedecimpunctata_587 | 0.151 |
| Myrrha_octodecimguttata_495 | Tytthaspis_sedecimpunctata_587 | 0.151 |
| Myrrha_octodecimguttata_498 | Tytthaspis_sedecimpunctata_587 | 0.151 |
| Calvia_quatuordecimguttata_207 | Tytthaspis_sedecimpunctata_590 | 0.151 |
| Calvia_quatuordecimguttata_218 | Tytthaspis_sedecimpunctata_590 | 0.151 |
| Calvia_quatuordecimguttata_207 | Tytthaspis_sedecimpunctata_592 | 0.151 |
| Calvia_quatuordecimguttata_218 | Tytthaspis_sedecimpunctata_592 | 0.151 |
| Calvia_quatuordecimguttata_218 | Tytthaspis_sedecimpunctata_593 | 0.151 |
| Calvia_quatuordecimguttata_207 | Tytthaspis_sedecimpunctata_594 | 0.151 |
| Calvia_quatuordecimguttata_218 | Tytthaspis_sedecimpunctata_594 | 0.151 |
| Calvia_quatuordecimguttata_207 | Tytthaspis_sedecimpunctata_595 | 0.151 |
| Calvia_quatuordecimguttata_218 | Tytthaspis_sedecimpunctata_595 | 0.151 |
| Coelophora_inaequalis_318 | Tytthaspis_sedecimpunctata_584 | 0.151 |
| Calvia_quatuordecimguttata_204 | Tytthaspis_sedecimpunctata_577 | 0.152 |
| Calvia_quatuordecimguttata_208 | Tytthaspis_sedecimpunctata_577 | 0.152 |
| Calvia_quatuordecimguttata_217 | Tytthaspis_sedecimpunctata_577 | 0.152 |
| Coleomegilla_maculata_329 | Tytthaspis_sedecimpunctata_577 | 0.152 |

| | | |
|---|---|---|
| Coleomegilla_maculata_330 | Tytthaspis_sedecimpunctata_577 | 0.152 |
| Coleomegilla_maculata_331 | Tytthaspis_sedecimpunctata_577 | 0.152 |
| Coleomegilla_maculata_333 | Tytthaspis_sedecimpunctata_577 | 0.152 |
| Coleomegilla_maculata_334 | Tytthaspis_sedecimpunctata_577 | 0.152 |
| Coleomegilla_maculata_335 | Tytthaspis_sedecimpunctata_577 | 0.152 |
| Coleomegilla_maculata_336 | Tytthaspis_sedecimpunctata_577 | 0.152 |
| Coleomegilla_maculata_337 | Tytthaspis_sedecimpunctata_577 | 0.152 |
| Coleomegilla_maculata_338 | Tytthaspis_sedecimpunctata_577 | 0.152 |
| Coleomegilla_maculata_339 | Tytthaspis_sedecimpunctata_577 | 0.152 |
| Calvia_quatuordecimguttata_204 | Tytthaspis_sedecimpunctata_578 | 0.152 |
| Calvia_quatuordecimguttata_208 | Tytthaspis_sedecimpunctata_578 | 0.152 |
| Calvia_quatuordecimguttata_217 | Tytthaspis_sedecimpunctata_578 | 0.152 |
| Coleomegilla_maculata_329 | Tytthaspis_sedecimpunctata_578 | 0.152 |
| Coleomegilla_maculata_330 | Tytthaspis_sedecimpunctata_578 | 0.152 |
| Coleomegilla_maculata_331 | Tytthaspis_sedecimpunctata_578 | 0.152 |
| Coleomegilla_maculata_333 | Tytthaspis_sedecimpunctata_578 | 0.152 |
| Coleomegilla_maculata_334 | Tytthaspis_sedecimpunctata_578 | 0.152 |
| Coleomegilla_maculata_335 | Tytthaspis_sedecimpunctata_578 | 0.152 |
| Coleomegilla_maculata_336 | Tytthaspis_sedecimpunctata_578 | 0.152 |
| Coleomegilla_maculata_337 | Tytthaspis_sedecimpunctata_578 | 0.152 |
| Coleomegilla_maculata_338 | Tytthaspis_sedecimpunctata_578 | 0.152 |
| Coleomegilla_maculata_339 | Tytthaspis_sedecimpunctata_578 | 0.152 |
| Calvia_quatuordecimguttata_204 | Tytthaspis_sedecimpunctata_579 | 0.152 |
| Calvia_quatuordecimguttata_208 | Tytthaspis_sedecimpunctata_579 | 0.152 |
| Calvia_quatuordecimguttata_217 | Tytthaspis_sedecimpunctata_579 | 0.152 |
| Coleomegilla_maculata_329 | Tytthaspis_sedecimpunctata_579 | 0.152 |
| Coleomegilla_maculata_330 | Tytthaspis_sedecimpunctata_579 | 0.152 |
| Coleomegilla_maculata_331 | Tytthaspis_sedecimpunctata_579 | 0.152 |
| Coleomegilla_maculata_333 | Tytthaspis_sedecimpunctata_579 | 0.152 |
| Coleomegilla_maculata_334 | Tytthaspis_sedecimpunctata_579 | 0.152 |
| Coleomegilla_maculata_335 | Tytthaspis_sedecimpunctata_579 | 0.152 |
| Coleomegilla_maculata_336 | Tytthaspis_sedecimpunctata_579 | 0.152 |
| Coleomegilla_maculata_337 | Tytthaspis_sedecimpunctata_579 | 0.152 |
| Coleomegilla_maculata_338 | Tytthaspis_sedecimpunctata_579 | 0.152 |
| Coleomegilla_maculata_339 | Tytthaspis_sedecimpunctata_579 | 0.152 |
| Calvia_quatuordecimguttata_207 | Tytthaspis_sedecimpunctata_580 | 0.152 |
| Calvia_quatuordecimguttata_204 | Tytthaspis_sedecimpunctata_581 | 0.152 |
| Calvia_quatuordecimguttata_208 | Tytthaspis_sedecimpunctata_581 | 0.152 |
| Calvia_quatuordecimguttata_217 | Tytthaspis_sedecimpunctata_581 | 0.152 |
| Coleomegilla_maculata_329 | Tytthaspis_sedecimpunctata_581 | 0.152 |
| Coleomegilla_maculata_330 | Tytthaspis_sedecimpunctata_581 | 0.152 |
| Coleomegilla_maculata_331 | Tytthaspis_sedecimpunctata_581 | 0.152 |
| Coleomegilla_maculata_333 | Tytthaspis_sedecimpunctata_581 | 0.152 |
| Coleomegilla_maculata_334 | Tytthaspis_sedecimpunctata_581 | 0.152 |
| Coleomegilla_maculata_335 | Tytthaspis_sedecimpunctata_581 | 0.152 |
| Coleomegilla_maculata_336 | Tytthaspis_sedecimpunctata_581 | 0.152 |
| Coleomegilla_maculata_337 | Tytthaspis_sedecimpunctata_581 | 0.152 |
| Coleomegilla_maculata_338 | Tytthaspis_sedecimpunctata_581 | 0.152 |
| Coleomegilla_maculata_339 | Tytthaspis_sedecimpunctata_581 | 0.152 |
| Calvia_quatuordecimguttata_204 | Tytthaspis_sedecimpunctata_582 | 0.152 |
| Calvia_quatuordecimguttata_208 | Tytthaspis_sedecimpunctata_582 | 0.152 |
| Calvia_quatuordecimguttata_217 | Tytthaspis_sedecimpunctata_582 | 0.152 |
| Coleomegilla_maculata_329 | Tytthaspis_sedecimpunctata_582 | 0.152 |
| Coleomegilla_maculata_330 | Tytthaspis_sedecimpunctata_582 | 0.152 |
| Coleomegilla_maculata_331 | Tytthaspis_sedecimpunctata_582 | 0.152 |
| Coleomegilla_maculata_333 | Tytthaspis_sedecimpunctata_582 | 0.152 |

| | | |
|---|---|---|
| Coleomegilla_maculata_334 | Tytthaspis_sedecimpunctata_582 | 0.152 |
| Coleomegilla_maculata_335 | Tytthaspis_sedecimpunctata_582 | 0.152 |
| Coleomegilla_maculata_336 | Tytthaspis_sedecimpunctata_582 | 0.152 |
| Coleomegilla_maculata_337 | Tytthaspis_sedecimpunctata_582 | 0.152 |
| Coleomegilla_maculata_338 | Tytthaspis_sedecimpunctata_582 | 0.152 |
| Coleomegilla_maculata_339 | Tytthaspis_sedecimpunctata_582 | 0.152 |
| Coleomegilla_maculata_328 | Tytthaspis_sedecimpunctata_583 | 0.152 |
| Coleomegilla_maculata_332 | Tytthaspis_sedecimpunctata_583 | 0.152 |
| Calvia_quatuordecimguttata_204 | Tytthaspis_sedecimpunctata_590 | 0.152 |
| Calvia_quatuordecimguttata_208 | Tytthaspis_sedecimpunctata_590 | 0.152 |
| Calvia_quatuordecimguttata_217 | Tytthaspis_sedecimpunctata_590 | 0.152 |
| Coleomegilla_maculata_329 | Tytthaspis_sedecimpunctata_590 | 0.152 |
| Coleomegilla_maculata_330 | Tytthaspis_sedecimpunctata_590 | 0.152 |
| Coleomegilla_maculata_331 | Tytthaspis_sedecimpunctata_590 | 0.152 |
| Coleomegilla_maculata_333 | Tytthaspis_sedecimpunctata_590 | 0.152 |
| Coleomegilla_maculata_334 | Tytthaspis_sedecimpunctata_590 | 0.152 |
| Coleomegilla_maculata_335 | Tytthaspis_sedecimpunctata_590 | 0.152 |
| Coleomegilla_maculata_336 | Tytthaspis_sedecimpunctata_590 | 0.152 |
| Coleomegilla_maculata_337 | Tytthaspis_sedecimpunctata_590 | 0.152 |
| Coleomegilla_maculata_338 | Tytthaspis_sedecimpunctata_590 | 0.152 |
| Coleomegilla_maculata_339 | Tytthaspis_sedecimpunctata_590 | 0.152 |
| Calvia_quatuordecimguttata_204 | Tytthaspis_sedecimpunctata_592 | 0.152 |
| Calvia_quatuordecimguttata_208 | Tytthaspis_sedecimpunctata_592 | 0.152 |
| Calvia_quatuordecimguttata_217 | Tytthaspis_sedecimpunctata_592 | 0.152 |
| Coleomegilla_maculata_329 | Tytthaspis_sedecimpunctata_592 | 0.152 |
| Coleomegilla_maculata_330 | Tytthaspis_sedecimpunctata_592 | 0.152 |
| Coleomegilla_maculata_331 | Tytthaspis_sedecimpunctata_592 | 0.152 |
| Coleomegilla_maculata_333 | Tytthaspis_sedecimpunctata_592 | 0.152 |
| Coleomegilla_maculata_334 | Tytthaspis_sedecimpunctata_592 | 0.152 |
| Coleomegilla_maculata_335 | Tytthaspis_sedecimpunctata_592 | 0.152 |
| Coleomegilla_maculata_336 | Tytthaspis_sedecimpunctata_592 | 0.152 |
| Coleomegilla_maculata_337 | Tytthaspis_sedecimpunctata_592 | 0.152 |
| Coleomegilla_maculata_338 | Tytthaspis_sedecimpunctata_592 | 0.152 |
| Coleomegilla_maculata_339 | Tytthaspis_sedecimpunctata_592 | 0.152 |
| Calvia_quatuordecimguttata_204 | Tytthaspis_sedecimpunctata_593 | 0.152 |
| Calvia_quatuordecimguttata_208 | Tytthaspis_sedecimpunctata_593 | 0.152 |
| Calvia_quatuordecimguttata_217 | Tytthaspis_sedecimpunctata_593 | 0.152 |
| Calvia_quatuordecimguttata_204 | Tytthaspis_sedecimpunctata_594 | 0.152 |
| Calvia_quatuordecimguttata_208 | Tytthaspis_sedecimpunctata_594 | 0.152 |
| Calvia_quatuordecimguttata_217 | Tytthaspis_sedecimpunctata_594 | 0.152 |
| Coleomegilla_maculata_329 | Tytthaspis_sedecimpunctata_594 | 0.152 |
| Coleomegilla_maculata_330 | Tytthaspis_sedecimpunctata_594 | 0.152 |
| Coleomegilla_maculata_331 | Tytthaspis_sedecimpunctata_594 | 0.152 |
| Coleomegilla_maculata_333 | Tytthaspis_sedecimpunctata_594 | 0.152 |
| Coleomegilla_maculata_334 | Tytthaspis_sedecimpunctata_594 | 0.152 |
| Coleomegilla_maculata_335 | Tytthaspis_sedecimpunctata_594 | 0.152 |
| Coleomegilla_maculata_336 | Tytthaspis_sedecimpunctata_594 | 0.152 |
| Coleomegilla_maculata_337 | Tytthaspis_sedecimpunctata_594 | 0.152 |
| Coleomegilla_maculata_338 | Tytthaspis_sedecimpunctata_594 | 0.152 |
| Coleomegilla_maculata_339 | Tytthaspis_sedecimpunctata_594 | 0.152 |
| Calvia_quatuordecimguttata_204 | Tytthaspis_sedecimpunctata_595 | 0.152 |
| Calvia_quatuordecimguttata_208 | Tytthaspis_sedecimpunctata_595 | 0.152 |
| Calvia_quatuordecimguttata_217 | Tytthaspis_sedecimpunctata_595 | 0.152 |
| Coleomegilla_maculata_329 | Tytthaspis_sedecimpunctata_595 | 0.152 |
| Coleomegilla_maculata_330 | Tytthaspis_sedecimpunctata_595 | 0.152 |
| Coleomegilla_maculata_331 | Tytthaspis_sedecimpunctata_595 | 0.152 |

| | | |
|---|---|---|
| Coleomegilla_maculata_333 | Tytthaspis_sedecimpunctata_595 | 0.152 |
| Coleomegilla_maculata_334 | Tytthaspis_sedecimpunctata_595 | 0.152 |
| Coleomegilla_maculata_335 | Tytthaspis_sedecimpunctata_595 | 0.152 |
| Coleomegilla_maculata_336 | Tytthaspis_sedecimpunctata_595 | 0.152 |
| Coleomegilla_maculata_337 | Tytthaspis_sedecimpunctata_595 | 0.152 |
| Coleomegilla_maculata_338 | Tytthaspis_sedecimpunctata_595 | 0.152 |
| Coleomegilla_maculata_339 | Tytthaspis_sedecimpunctata_595 | 0.152 |
| Coleomegilla_maculata_327 | Tytthaspis_sedecimpunctata_580 | 0.152 |
| Calvia_quatuordecimguttata_205 | Tytthaspis_sedecimpunctata_580 | 0.152 |
| Calvia_quatuordecimguttata_205 | Tytthaspis_sedecimpunctata_584 | 0.152 |
| Scymnus_ferrugatus_724 | Scymnus_suturalis_838 | 0.152 |
| Scymnus_ferrugatus_729 | Scymnus_suturalis_838 | 0.152 |
| Macronaemia_episcopalis_478 | Tytthaspis_sedecimpunctata_583 | 0.152 |
| Macronaemia_episcopalis_478 | Tytthaspis_sedecimpunctata_592 | 0.152 |
| Scymnus_ferrugatus_723 | Scymnus_suturalis_833 | 0.152 |
| Scymnus_ferrugatus_725 | Scymnus_suturalis_833 | 0.152 |
| Scymnus_ferrugatus_726 | Scymnus_suturalis_833 | 0.152 |
| Scymnus_ferrugatus_727 | Scymnus_suturalis_833 | 0.152 |
| Scymnus_ferrugatus_728 | Scymnus_suturalis_833 | 0.152 |
| Scymnus_ferrugatus_730 | Scymnus_suturalis_833 | 0.152 |
| Scymnus_ferrugatus_731 | Scymnus_suturalis_833 | 0.152 |
| Scymnus_ferrugatus_732 | Scymnus_suturalis_833 | 0.152 |
| Scymnus_ferrugatus_733 | Scymnus_suturalis_833 | 0.152 |
| Scymnus_nigrinus_787 | Scymnus_suturalis_843 | 0.152 |
| Scymnus_nigrinus_787 | Scymnus_suturalis_845 | 0.152 |
| Scymnus_nigrinus_787 | Scymnus_suturalis_837 | 0.152 |
| Scymnus_sinuanodulus_828 | Scymnus_suturalis_841 | 0.152 |
| Scymnus_sinuanodulus_828 | Scymnus_suturalis_842 | 0.152 |
| Scymnus_sinuanodulus_828 | Scymnus_suturalis_844 | 0.152 |
| Scymnus_sinuanodulus_828 | Scymnus_suturalis_846 | 0.152 |
| Scymnus_sinuanodulus_828 | Scymnus_suturalis_847 | 0.152 |
| Scymnus_sinuanodulus_828 | Scymnus_suturalis_848 | 0.152 |
| Anisosticta_novemdecimpunctata_169 | Tytthaspis_sedecimpunctata_589 | 0.152 |
| Anisosticta_novemdecimpunctata_172 | Tytthaspis_sedecimpunctata_589 | 0.152 |
| Scymnus_sinuanodulus_828 | Scymnus_suturalis_832 | 0.152 |
| Scymnus_sinuanodulus_828 | Scymnus_suturalis_835 | 0.152 |
| Scymnus_sinuanodulus_828 | Scymnus_suturalis_836 | 0.152 |
| Scymnus_sinuanodulus_828 | Scymnus_suturalis_837 | 0.152 |
| Scymnus_auritus_701 | Scymnus_suturalis_838 | 0.152 |
| Scymnus_sinuanodulus_828 | Scymnus_suturalis_839 | 0.152 |
| Scymnus_auritus_701 | Scymnus_suturalis_840 | 0.152 |
| Anisosticta_novemdecimpunctata_169 | Tytthaspis_sedecimpunctata_584 | 0.152 |
| Anisosticta_novemdecimpunctata_172 | Tytthaspis_sedecimpunctata_584 | 0.152 |
| Anisosticta_novemdecimpunctata_162 | Tytthaspis_sedecimpunctata_586 | 0.152 |
| Anisosticta_novemdecimpunctata_164 | Tytthaspis_sedecimpunctata_586 | 0.152 |
| Anisosticta_novemdecimpunctata_166 | Tytthaspis_sedecimpunctata_586 | 0.152 |
| Anisosticta_novemdecimpunctata_167 | Tytthaspis_sedecimpunctata_586 | 0.152 |
| Anisosticta_novemdecimpunctata_168 | Tytthaspis_sedecimpunctata_586 | 0.152 |
| Anisosticta_novemdecimpunctata_169 | Tytthaspis_sedecimpunctata_587 | 0.152 |
| Anisosticta_novemdecimpunctata_172 | Tytthaspis_sedecimpunctata_587 | 0.152 |
| Anisosticta_novemdecimpunctata_162 | Tytthaspis_sedecimpunctata_588 | 0.152 |
| Anisosticta_novemdecimpunctata_164 | Tytthaspis_sedecimpunctata_588 | 0.152 |
| Anisosticta_novemdecimpunctata_166 | Tytthaspis_sedecimpunctata_588 | 0.152 |
| Anisosticta_novemdecimpunctata_167 | Tytthaspis_sedecimpunctata_588 | 0.152 |
| Anisosticta_novemdecimpunctata_168 | Tytthaspis_sedecimpunctata_588 | 0.152 |
| Scymnus_auritus_701 | Scymnus_suturalis_834 | 0.152 |

| | | |
|---|---|---|
| Scymnus_camptodromus_717 | Scymnus_rubromaculatus_822 | 0.152 |
| Scymnus_camptodromus_717 | Scymnus_rubromaculatus_823 | 0.152 |
| Scymnus_camptodromus_717 | Scymnus_rubromaculatus_824 | 0.152 |
| Scymnus_camptodromus_717 | Scymnus_rubromaculatus_825 | 0.152 |
| Scymnus_camptodromus_717 | Scymnus_rubromaculatus_826 | 0.152 |
| Scymnus_camptodromus_717 | Scymnus_rubromaculatus_827 | 0.152 |
| Anisosticta_novemdecimpunctata_159 | Tytthaspis_sedecimpunctata_577 | 0.152 |
| Anisosticta_novemdecimpunctata_163 | Tytthaspis_sedecimpunctata_577 | 0.152 |
| Anisosticta_novemdecimpunctata_171 | Tytthaspis_sedecimpunctata_577 | 0.152 |
| Anisosticta_novemdecimpunctata_159 | Tytthaspis_sedecimpunctata_578 | 0.152 |
| Anisosticta_novemdecimpunctata_163 | Tytthaspis_sedecimpunctata_578 | 0.152 |
| Anisosticta_novemdecimpunctata_171 | Tytthaspis_sedecimpunctata_578 | 0.152 |
| Anisosticta_novemdecimpunctata_159 | Tytthaspis_sedecimpunctata_579 | 0.152 |
| Anisosticta_novemdecimpunctata_163 | Tytthaspis_sedecimpunctata_579 | 0.152 |
| Anisosticta_novemdecimpunctata_171 | Tytthaspis_sedecimpunctata_579 | 0.152 |
| Anisosticta_novemdecimpunctata_159 | Tytthaspis_sedecimpunctata_581 | 0.152 |
| Anisosticta_novemdecimpunctata_163 | Tytthaspis_sedecimpunctata_581 | 0.152 |
| Anisosticta_novemdecimpunctata_171 | Tytthaspis_sedecimpunctata_581 | 0.152 |
| Anisosticta_novemdecimpunctata_159 | Tytthaspis_sedecimpunctata_582 | 0.152 |
| Anisosticta_novemdecimpunctata_163 | Tytthaspis_sedecimpunctata_582 | 0.152 |
| Anisosticta_novemdecimpunctata_171 | Tytthaspis_sedecimpunctata_582 | 0.152 |
| Anisosticta_novemdecimpunctata_169 | Tytthaspis_sedecimpunctata_583 | 0.152 |
| Anisosticta_novemdecimpunctata_172 | Tytthaspis_sedecimpunctata_583 | 0.152 |
| Anisosticta_novemdecimpunctata_162 | Tytthaspis_sedecimpunctata_585 | 0.152 |
| Anisosticta_novemdecimpunctata_164 | Tytthaspis_sedecimpunctata_585 | 0.152 |
| Anisosticta_novemdecimpunctata_166 | Tytthaspis_sedecimpunctata_585 | 0.152 |
| Anisosticta_novemdecimpunctata_167 | Tytthaspis_sedecimpunctata_585 | 0.152 |
| Anisosticta_novemdecimpunctata_168 | Tytthaspis_sedecimpunctata_585 | 0.152 |
| Anisosticta_novemdecimpunctata_159 | Tytthaspis_sedecimpunctata_590 | 0.152 |
| Anisosticta_novemdecimpunctata_163 | Tytthaspis_sedecimpunctata_590 | 0.152 |
| Anisosticta_novemdecimpunctata_171 | Tytthaspis_sedecimpunctata_590 | 0.152 |
| Anisosticta_novemdecimpunctata_162 | Tytthaspis_sedecimpunctata_592 | 0.152 |
| Anisosticta_novemdecimpunctata_164 | Tytthaspis_sedecimpunctata_592 | 0.152 |
| Anisosticta_novemdecimpunctata_166 | Tytthaspis_sedecimpunctata_592 | 0.152 |
| Anisosticta_novemdecimpunctata_167 | Tytthaspis_sedecimpunctata_592 | 0.152 |
| Anisosticta_novemdecimpunctata_168 | Tytthaspis_sedecimpunctata_592 | 0.152 |
| Anisosticta_novemdecimpunctata_159 | Tytthaspis_sedecimpunctata_593 | 0.152 |
| Anisosticta_novemdecimpunctata_163 | Tytthaspis_sedecimpunctata_593 | 0.152 |
| Anisosticta_novemdecimpunctata_171 | Tytthaspis_sedecimpunctata_593 | 0.152 |
| Anisosticta_novemdecimpunctata_159 | Tytthaspis_sedecimpunctata_594 | 0.152 |
| Anisosticta_novemdecimpunctata_163 | Tytthaspis_sedecimpunctata_594 | 0.152 |
| Anisosticta_novemdecimpunctata_171 | Tytthaspis_sedecimpunctata_594 | 0.152 |
| Anisosticta_novemdecimpunctata_159 | Tytthaspis_sedecimpunctata_595 | 0.152 |
| Anisosticta_novemdecimpunctata_163 | Tytthaspis_sedecimpunctata_595 | 0.152 |
| Anisosticta_novemdecimpunctata_171 | Tytthaspis_sedecimpunctata_595 | 0.152 |
| Anisosticta_novemdecimpunctata_159 | Tytthaspis_sedecimpunctata_580 | 0.152 |
| Anisosticta_novemdecimpunctata_162 | Tytthaspis_sedecimpunctata_580 | 0.152 |
| Anisosticta_novemdecimpunctata_163 | Tytthaspis_sedecimpunctata_580 | 0.152 |
| Anisosticta_novemdecimpunctata_171 | Tytthaspis_sedecimpunctata_580 | 0.152 |
| Anisosticta_bitriangularis_152 | Tytthaspis_sedecimpunctata_589 | 0.152 |
| Scymnus_abietis_682 | Scymnus_sinuanodulus_830 | 0.152 |
| Scymnus_abietis_683 | Scymnus_sinuanodulus_830 | 0.152 |
| Scymnus_abietis_685 | Scymnus_sinuanodulus_830 | 0.152 |
| Anisosticta_bitriangularis_152 | Tytthaspis_sedecimpunctata_587 | 0.152 |
| Halyzia_sedecimguttata_351 | Vibidia_duodecimguttata_597 | 0.153 |
| Halyzia_sedecimguttata_353 | Vibidia_duodecimguttata_597 | 0.153 |

| | | |
|---|---|---|
| Halyzia_sedecimguttata_351 | Vibidia_duodecimguttata_600 | 0.153 |
| Halyzia_sedecimguttata_353 | Vibidia_duodecimguttata_600 | 0.153 |
| Halyzia_sedecimguttata_352 | Vibidia_duodecimguttata_596 | 0.153 |
| Halyzia_sedecimguttata_352 | Vibidia_duodecimguttata_598 | 0.153 |
| Halyzia_sedecimguttata_351 | Vibidia_duodecimguttata_603 | 0.153 |
| Halyzia_sedecimguttata_353 | Vibidia_duodecimguttata_603 | 0.153 |
| Scymnus_nebulosus_783 | Scymnus_sinuanodulus_829 | 0.153 |
| Brumoides_suturalis_2 | Tytthaspis_sedecimpunctata_586 | 0.153 |
| Harmonia_conformis_374 | Tytthaspis_sedecimpunctata_583 | 0.153 |
| Harmonia_conformis_365 | Tytthaspis_sedecimpunctata_585 | 0.153 |
| Harmonia_conformis_366 | Tytthaspis_sedecimpunctata_585 | 0.153 |
| Harmonia_conformis_367 | Tytthaspis_sedecimpunctata_585 | 0.153 |
| Harmonia_conformis_368 | Tytthaspis_sedecimpunctata_585 | 0.153 |
| Harmonia_conformis_369 | Tytthaspis_sedecimpunctata_585 | 0.153 |
| Harmonia_conformis_370 | Tytthaspis_sedecimpunctata_585 | 0.153 |
| Harmonia_conformis_371 | Tytthaspis_sedecimpunctata_585 | 0.153 |
| Harmonia_conformis_372 | Tytthaspis_sedecimpunctata_585 | 0.153 |
| Harmonia_conformis_373 | Tytthaspis_sedecimpunctata_585 | 0.153 |
| Harmonia_conformis_375 | Tytthaspis_sedecimpunctata_585 | 0.153 |
| Harmonia_conformis_365 | Tytthaspis_sedecimpunctata_593 | 0.153 |
| Harmonia_conformis_366 | Tytthaspis_sedecimpunctata_593 | 0.153 |
| Harmonia_conformis_367 | Tytthaspis_sedecimpunctata_593 | 0.153 |
| Harmonia_conformis_368 | Tytthaspis_sedecimpunctata_593 | 0.153 |
| Harmonia_conformis_369 | Tytthaspis_sedecimpunctata_593 | 0.153 |
| Harmonia_conformis_370 | Tytthaspis_sedecimpunctata_593 | 0.153 |
| Harmonia_conformis_371 | Tytthaspis_sedecimpunctata_593 | 0.153 |
| Harmonia_conformis_372 | Tytthaspis_sedecimpunctata_593 | 0.153 |
| Harmonia_conformis_373 | Tytthaspis_sedecimpunctata_593 | 0.153 |
| Harmonia_conformis_375 | Tytthaspis_sedecimpunctata_593 | 0.153 |
| Harmonia_conformis_365 | Tytthaspis_sedecimpunctata_586 | 0.153 |
| Harmonia_conformis_366 | Tytthaspis_sedecimpunctata_586 | 0.153 |
| Harmonia_conformis_367 | Tytthaspis_sedecimpunctata_586 | 0.153 |
| Harmonia_conformis_368 | Tytthaspis_sedecimpunctata_586 | 0.153 |
| Harmonia_conformis_369 | Tytthaspis_sedecimpunctata_586 | 0.153 |
| Harmonia_conformis_370 | Tytthaspis_sedecimpunctata_586 | 0.153 |
| Harmonia_conformis_371 | Tytthaspis_sedecimpunctata_586 | 0.153 |
| Harmonia_conformis_372 | Tytthaspis_sedecimpunctata_586 | 0.153 |
| Harmonia_conformis_373 | Tytthaspis_sedecimpunctata_586 | 0.153 |
| Harmonia_conformis_375 | Tytthaspis_sedecimpunctata_586 | 0.153 |
| Harmonia_conformis_365 | Tytthaspis_sedecimpunctata_588 | 0.153 |
| Harmonia_conformis_366 | Tytthaspis_sedecimpunctata_588 | 0.153 |
| Harmonia_conformis_367 | Tytthaspis_sedecimpunctata_588 | 0.153 |
| Harmonia_conformis_368 | Tytthaspis_sedecimpunctata_588 | 0.153 |
| Harmonia_conformis_369 | Tytthaspis_sedecimpunctata_588 | 0.153 |
| Harmonia_conformis_370 | Tytthaspis_sedecimpunctata_588 | 0.153 |
| Harmonia_conformis_371 | Tytthaspis_sedecimpunctata_588 | 0.153 |
| Harmonia_conformis_372 | Tytthaspis_sedecimpunctata_588 | 0.153 |
| Harmonia_conformis_373 | Tytthaspis_sedecimpunctata_588 | 0.153 |
| Harmonia_conformis_375 | Tytthaspis_sedecimpunctata_588 | 0.153 |
| Mulsantina_hudsonica_479 | Tytthaspis_sedecimpunctata_584 | 0.153 |
| Mulsantina_hudsonica_483 | Tytthaspis_sedecimpunctata_586 | 0.153 |
| Mulsantina_hudsonica_483 | Tytthaspis_sedecimpunctata_587 | 0.153 |
| Mulsantina_hudsonica_483 | Tytthaspis_sedecimpunctata_588 | 0.153 |
| Mulsantina_hudsonica_480 | Tytthaspis_sedecimpunctata_577 | 0.153 |
| Mulsantina_hudsonica_480 | Tytthaspis_sedecimpunctata_578 | 0.153 |
| Mulsantina_hudsonica_480 | Tytthaspis_sedecimpunctata_579 | 0.153 |

| | | |
|---|---|---|
| Mulsantina_hudsonica_480 | Tytthaspis_sedecimpunctata_581 | 0.153 |
| Mulsantina_hudsonica_480 | Tytthaspis_sedecimpunctata_582 | 0.153 |
| Mulsantina_hudsonica_483 | Tytthaspis_sedecimpunctata_585 | 0.153 |
| Mulsantina_hudsonica_480 | Tytthaspis_sedecimpunctata_589 | 0.153 |
| Mulsantina_hudsonica_480 | Tytthaspis_sedecimpunctata_590 | 0.153 |
| Mulsantina_hudsonica_480 | Tytthaspis_sedecimpunctata_592 | 0.153 |
| Mulsantina_hudsonica_480 | Tytthaspis_sedecimpunctata_593 | 0.153 |
| Mulsantina_hudsonica_480 | Tytthaspis_sedecimpunctata_594 | 0.153 |
| Mulsantina_hudsonica_480 | Tytthaspis_sedecimpunctata_595 | 0.153 |
| Scymnus_interruptus_760 | Scymnus_suturalis_841 | 0.153 |
| Scymnus_interruptus_765 | Scymnus_suturalis_841 | 0.153 |
| Scymnus_interruptus_767 | Scymnus_suturalis_841 | 0.153 |
| Scymnus_interruptus_768 | Scymnus_suturalis_841 | 0.153 |
| Scymnus_interruptus_760 | Scymnus_suturalis_842 | 0.153 |
| Scymnus_interruptus_765 | Scymnus_suturalis_842 | 0.153 |
| Scymnus_interruptus_767 | Scymnus_suturalis_842 | 0.153 |
| Scymnus_interruptus_768 | Scymnus_suturalis_842 | 0.153 |
| Scymnus_interruptus_760 | Scymnus_suturalis_844 | 0.153 |
| Scymnus_interruptus_765 | Scymnus_suturalis_844 | 0.153 |
| Scymnus_interruptus_767 | Scymnus_suturalis_844 | 0.153 |
| Scymnus_interruptus_768 | Scymnus_suturalis_844 | 0.153 |
| Scymnus_interruptus_760 | Scymnus_suturalis_846 | 0.153 |
| Scymnus_interruptus_765 | Scymnus_suturalis_846 | 0.153 |
| Scymnus_interruptus_767 | Scymnus_suturalis_846 | 0.153 |
| Scymnus_interruptus_768 | Scymnus_suturalis_846 | 0.153 |
| Scymnus_interruptus_760 | Scymnus_suturalis_847 | 0.153 |
| Scymnus_interruptus_765 | Scymnus_suturalis_847 | 0.153 |
| Scymnus_interruptus_767 | Scymnus_suturalis_847 | 0.153 |
| Scymnus_interruptus_768 | Scymnus_suturalis_847 | 0.153 |
| Scymnus_interruptus_760 | Scymnus_suturalis_848 | 0.153 |
| Scymnus_interruptus_765 | Scymnus_suturalis_848 | 0.153 |
| Scymnus_interruptus_767 | Scymnus_suturalis_848 | 0.153 |
| Scymnus_interruptus_768 | Scymnus_suturalis_848 | 0.153 |
| Coelophora_inaequalis_311 | Tytthaspis_sedecimpunctata_589 | 0.153 |
| Coelophora_inaequalis_313 | Tytthaspis_sedecimpunctata_589 | 0.153 |
| Coelophora_inaequalis_316 | Tytthaspis_sedecimpunctata_589 | 0.153 |
| Coelophora_inaequalis_324 | Tytthaspis_sedecimpunctata_589 | 0.153 |
| Coelophora_inaequalis_326 | Tytthaspis_sedecimpunctata_589 | 0.153 |
| Scymnus_interruptus_760 | Scymnus_suturalis_832 | 0.153 |
| Scymnus_interruptus_765 | Scymnus_suturalis_832 | 0.153 |
| Scymnus_interruptus_767 | Scymnus_suturalis_832 | 0.153 |
| Scymnus_interruptus_768 | Scymnus_suturalis_832 | 0.153 |
| Scymnus_interruptus_760 | Scymnus_suturalis_835 | 0.153 |
| Scymnus_interruptus_765 | Scymnus_suturalis_835 | 0.153 |
| Scymnus_interruptus_767 | Scymnus_suturalis_835 | 0.153 |
| Scymnus_interruptus_768 | Scymnus_suturalis_835 | 0.153 |
| Scymnus_interruptus_760 | Scymnus_suturalis_836 | 0.153 |
| Scymnus_interruptus_765 | Scymnus_suturalis_836 | 0.153 |
| Scymnus_interruptus_767 | Scymnus_suturalis_836 | 0.153 |
| Scymnus_interruptus_768 | Scymnus_suturalis_836 | 0.153 |
| Scymnus_interruptus_760 | Scymnus_suturalis_839 | 0.153 |
| Scymnus_interruptus_765 | Scymnus_suturalis_839 | 0.153 |
| Scymnus_interruptus_767 | Scymnus_suturalis_839 | 0.153 |
| Scymnus_interruptus_768 | Scymnus_suturalis_839 | 0.153 |
| Coelophora_inaequalis_317 | Tytthaspis_sedecimpunctata_586 | 0.153 |
| Coelophora_inaequalis_311 | Tytthaspis_sedecimpunctata_587 | 0.153 |

| | | |
|---|---|---|
| Coelophora_inaequalis_313 | Tytthaspis_sedecimpunctata_587 | 0.153 |
| Coelophora_inaequalis_316 | Tytthaspis_sedecimpunctata_587 | 0.153 |
| Coelophora_inaequalis_324 | Tytthaspis_sedecimpunctata_587 | 0.153 |
| Coelophora_inaequalis_326 | Tytthaspis_sedecimpunctata_587 | 0.153 |
| Coelophora_inaequalis_315 | Tytthaspis_sedecimpunctata_589 | 0.153 |
| Coelophora_inaequalis_317 | Tytthaspis_sedecimpunctata_577 | 0.153 |
| Coelophora_inaequalis_317 | Tytthaspis_sedecimpunctata_578 | 0.153 |
| Coelophora_inaequalis_317 | Tytthaspis_sedecimpunctata_579 | 0.153 |
| Coelophora_inaequalis_317 | Tytthaspis_sedecimpunctata_581 | 0.153 |
| Coelophora_inaequalis_317 | Tytthaspis_sedecimpunctata_582 | 0.153 |
| Coelophora_inaequalis_315 | Tytthaspis_sedecimpunctata_587 | 0.153 |
| Coelophora_inaequalis_317 | Tytthaspis_sedecimpunctata_590 | 0.153 |
| Coelophora_inaequalis_317 | Tytthaspis_sedecimpunctata_593 | 0.153 |
| Coelophora_inaequalis_317 | Tytthaspis_sedecimpunctata_594 | 0.153 |
| Coelophora_inaequalis_317 | Tytthaspis_sedecimpunctata_595 | 0.153 |
| Coelophora_inaequalis_317 | Tytthaspis_sedecimpunctata_584 | 0.153 |
| Myrrha_octodecimguttata_499 | Tytthaspis_sedecimpunctata_584 | 0.153 |
| Exochomus_nigromaculatus_48 | Tytthaspis_sedecimpunctata_591 | 0.153 |
| Myrrha_octodecimguttata_488 | Tytthaspis_sedecimpunctata_583 | 0.153 |
| Myrrha_octodecimguttata_491 | Tytthaspis_sedecimpunctata_583 | 0.153 |
| Myrrha_octodecimguttata_492 | Tytthaspis_sedecimpunctata_583 | 0.153 |
| Myrrha_octodecimguttata_488 | Tytthaspis_sedecimpunctata_585 | 0.153 |
| Myrrha_octodecimguttata_491 | Tytthaspis_sedecimpunctata_585 | 0.153 |
| Myrrha_octodecimguttata_492 | Tytthaspis_sedecimpunctata_585 | 0.153 |
| Myrrha_octodecimguttata_488 | Tytthaspis_sedecimpunctata_592 | 0.153 |
| Myrrha_octodecimguttata_491 | Tytthaspis_sedecimpunctata_592 | 0.153 |
| Myrrha_octodecimguttata_492 | Tytthaspis_sedecimpunctata_592 | 0.153 |
| Myrrha_octodecimguttata_493 | Tytthaspis_sedecimpunctata_584 | 0.153 |
| Myrrha_octodecimguttata_495 | Tytthaspis_sedecimpunctata_584 | 0.153 |
| Myrrha_octodecimguttata_498 | Tytthaspis_sedecimpunctata_584 | 0.153 |
| Myrrha_octodecimguttata_485 | Tytthaspis_sedecimpunctata_585 | 0.153 |
| Myrrha_octodecimguttata_486 | Tytthaspis_sedecimpunctata_585 | 0.153 |
| Myrrha_octodecimguttata_487 | Tytthaspis_sedecimpunctata_585 | 0.153 |
| Myrrha_octodecimguttata_490 | Tytthaspis_sedecimpunctata_585 | 0.153 |
| Myrrha_octodecimguttata_494 | Tytthaspis_sedecimpunctata_585 | 0.153 |
| Myrrha_octodecimguttata_496 | Tytthaspis_sedecimpunctata_585 | 0.153 |
| Myrrha_octodecimguttata_497 | Tytthaspis_sedecimpunctata_585 | 0.153 |
| Myrrha_octodecimguttata_501 | Tytthaspis_sedecimpunctata_585 | 0.153 |
| Myrrha_octodecimguttata_502 | Tytthaspis_sedecimpunctata_585 | 0.153 |
| Myrrha_octodecimguttata_485 | Tytthaspis_sedecimpunctata_593 | 0.153 |
| Myrrha_octodecimguttata_486 | Tytthaspis_sedecimpunctata_593 | 0.153 |
| Myrrha_octodecimguttata_487 | Tytthaspis_sedecimpunctata_593 | 0.153 |
| Myrrha_octodecimguttata_490 | Tytthaspis_sedecimpunctata_593 | 0.153 |
| Myrrha_octodecimguttata_494 | Tytthaspis_sedecimpunctata_593 | 0.153 |
| Myrrha_octodecimguttata_496 | Tytthaspis_sedecimpunctata_593 | 0.153 |
| Myrrha_octodecimguttata_497 | Tytthaspis_sedecimpunctata_593 | 0.153 |
| Myrrha_octodecimguttata_501 | Tytthaspis_sedecimpunctata_593 | 0.153 |
| Myrrha_octodecimguttata_502 | Tytthaspis_sedecimpunctata_593 | 0.153 |
| Coelophora_inaequalis_318 | Tytthaspis_sedecimpunctata_589 | 0.153 |
| Coelophora_inaequalis_318 | Tytthaspis_sedecimpunctata_587 | 0.153 |
| Scymnus_rubromaculatus_813 | Scymnus_suturalis_841 | 0.153 |
| Scymnus_rubromaculatus_815 | Scymnus_suturalis_841 | 0.153 |
| Scymnus_rubromaculatus_816 | Scymnus_suturalis_841 | 0.153 |
| Scymnus_rubromaculatus_817 | Scymnus_suturalis_841 | 0.153 |
| Scymnus_rubromaculatus_818 | Scymnus_suturalis_841 | 0.153 |
| Scymnus_rubromaculatus_819 | Scymnus_suturalis_841 | 0.153 |

| | | |
|---|---|---|
| Scymnus_rubromaculatus_820 | Scymnus_suturalis_841 | 0.153 |
| Scymnus_rubromaculatus_821 | Scymnus_suturalis_841 | 0.153 |
| Scymnus_rubromaculatus_822 | Scymnus_suturalis_841 | 0.153 |
| Scymnus_rubromaculatus_823 | Scymnus_suturalis_841 | 0.153 |
| Scymnus_rubromaculatus_824 | Scymnus_suturalis_841 | 0.153 |
| Scymnus_rubromaculatus_825 | Scymnus_suturalis_841 | 0.153 |
| Scymnus_rubromaculatus_826 | Scymnus_suturalis_841 | 0.153 |
| Scymnus_rubromaculatus_827 | Scymnus_suturalis_841 | 0.153 |
| Scymnus_rubromaculatus_813 | Scymnus_suturalis_842 | 0.153 |
| Scymnus_rubromaculatus_815 | Scymnus_suturalis_842 | 0.153 |
| Scymnus_rubromaculatus_816 | Scymnus_suturalis_842 | 0.153 |
| Scymnus_rubromaculatus_817 | Scymnus_suturalis_842 | 0.153 |
| Scymnus_rubromaculatus_818 | Scymnus_suturalis_842 | 0.153 |
| Scymnus_rubromaculatus_819 | Scymnus_suturalis_842 | 0.153 |
| Scymnus_rubromaculatus_820 | Scymnus_suturalis_842 | 0.153 |
| Scymnus_rubromaculatus_821 | Scymnus_suturalis_842 | 0.153 |
| Scymnus_rubromaculatus_822 | Scymnus_suturalis_842 | 0.153 |
| Scymnus_rubromaculatus_823 | Scymnus_suturalis_842 | 0.153 |
| Scymnus_rubromaculatus_824 | Scymnus_suturalis_842 | 0.153 |
| Scymnus_rubromaculatus_825 | Scymnus_suturalis_842 | 0.153 |
| Scymnus_rubromaculatus_826 | Scymnus_suturalis_842 | 0.153 |
| Scymnus_rubromaculatus_827 | Scymnus_suturalis_842 | 0.153 |
| Scymnus_rubromaculatus_814 | Scymnus_suturalis_843 | 0.153 |
| Scymnus_rubromaculatus_813 | Scymnus_suturalis_844 | 0.153 |
| Scymnus_rubromaculatus_815 | Scymnus_suturalis_844 | 0.153 |
| Scymnus_rubromaculatus_816 | Scymnus_suturalis_844 | 0.153 |
| Scymnus_rubromaculatus_817 | Scymnus_suturalis_844 | 0.153 |
| Scymnus_rubromaculatus_818 | Scymnus_suturalis_844 | 0.153 |
| Scymnus_rubromaculatus_819 | Scymnus_suturalis_844 | 0.153 |
| Scymnus_rubromaculatus_820 | Scymnus_suturalis_844 | 0.153 |
| Scymnus_rubromaculatus_821 | Scymnus_suturalis_844 | 0.153 |
| Scymnus_rubromaculatus_822 | Scymnus_suturalis_844 | 0.153 |
| Scymnus_rubromaculatus_823 | Scymnus_suturalis_844 | 0.153 |
| Scymnus_rubromaculatus_824 | Scymnus_suturalis_844 | 0.153 |
| Scymnus_rubromaculatus_825 | Scymnus_suturalis_844 | 0.153 |
| Scymnus_rubromaculatus_826 | Scymnus_suturalis_844 | 0.153 |
| Scymnus_rubromaculatus_827 | Scymnus_suturalis_844 | 0.153 |
| Scymnus_rubromaculatus_814 | Scymnus_suturalis_845 | 0.153 |
| Scymnus_rubromaculatus_813 | Scymnus_suturalis_846 | 0.153 |
| Scymnus_rubromaculatus_815 | Scymnus_suturalis_846 | 0.153 |
| Scymnus_rubromaculatus_816 | Scymnus_suturalis_846 | 0.153 |
| Scymnus_rubromaculatus_817 | Scymnus_suturalis_846 | 0.153 |
| Scymnus_rubromaculatus_818 | Scymnus_suturalis_846 | 0.153 |
| Scymnus_rubromaculatus_819 | Scymnus_suturalis_846 | 0.153 |
| Scymnus_rubromaculatus_820 | Scymnus_suturalis_846 | 0.153 |
| Scymnus_rubromaculatus_821 | Scymnus_suturalis_846 | 0.153 |
| Scymnus_rubromaculatus_822 | Scymnus_suturalis_846 | 0.153 |
| Scymnus_rubromaculatus_823 | Scymnus_suturalis_846 | 0.153 |
| Scymnus_rubromaculatus_824 | Scymnus_suturalis_846 | 0.153 |
| Scymnus_rubromaculatus_825 | Scymnus_suturalis_846 | 0.153 |
| Scymnus_rubromaculatus_826 | Scymnus_suturalis_846 | 0.153 |
| Scymnus_rubromaculatus_827 | Scymnus_suturalis_846 | 0.153 |
| Scymnus_rubromaculatus_813 | Scymnus_suturalis_847 | 0.153 |
| Scymnus_rubromaculatus_815 | Scymnus_suturalis_847 | 0.153 |
| Scymnus_rubromaculatus_816 | Scymnus_suturalis_847 | 0.153 |
| Scymnus_rubromaculatus_817 | Scymnus_suturalis_847 | 0.153 |

| | | |
|---|---|---|
| Scymnus_rubromaculatus_818 | Scymnus_suturalis_847 | 0.153 |
| Scymnus_rubromaculatus_819 | Scymnus_suturalis_847 | 0.153 |
| Scymnus_rubromaculatus_820 | Scymnus_suturalis_847 | 0.153 |
| Scymnus_rubromaculatus_821 | Scymnus_suturalis_847 | 0.153 |
| Scymnus_rubromaculatus_822 | Scymnus_suturalis_847 | 0.153 |
| Scymnus_rubromaculatus_823 | Scymnus_suturalis_847 | 0.153 |
| Scymnus_rubromaculatus_824 | Scymnus_suturalis_847 | 0.153 |
| Scymnus_rubromaculatus_825 | Scymnus_suturalis_847 | 0.153 |
| Scymnus_rubromaculatus_826 | Scymnus_suturalis_847 | 0.153 |
| Scymnus_rubromaculatus_827 | Scymnus_suturalis_847 | 0.153 |
| Scymnus_rubromaculatus_813 | Scymnus_suturalis_848 | 0.153 |
| Scymnus_rubromaculatus_815 | Scymnus_suturalis_848 | 0.153 |
| Scymnus_rubromaculatus_816 | Scymnus_suturalis_848 | 0.153 |
| Scymnus_rubromaculatus_817 | Scymnus_suturalis_848 | 0.153 |
| Scymnus_rubromaculatus_818 | Scymnus_suturalis_848 | 0.153 |
| Scymnus_rubromaculatus_819 | Scymnus_suturalis_848 | 0.153 |
| Scymnus_rubromaculatus_820 | Scymnus_suturalis_848 | 0.153 |
| Scymnus_rubromaculatus_821 | Scymnus_suturalis_848 | 0.153 |
| Scymnus_rubromaculatus_822 | Scymnus_suturalis_848 | 0.153 |
| Scymnus_rubromaculatus_823 | Scymnus_suturalis_848 | 0.153 |
| Scymnus_rubromaculatus_824 | Scymnus_suturalis_848 | 0.153 |
| Scymnus_rubromaculatus_825 | Scymnus_suturalis_848 | 0.153 |
| Scymnus_rubromaculatus_826 | Scymnus_suturalis_848 | 0.153 |
| Scymnus_rubromaculatus_827 | Scymnus_suturalis_848 | 0.153 |
| Myrrha_octodecimguttata_485 | Tytthaspis_sedecimpunctata_587 | 0.153 |
| Myrrha_octodecimguttata_486 | Tytthaspis_sedecimpunctata_587 | 0.153 |
| Myrrha_octodecimguttata_487 | Tytthaspis_sedecimpunctata_587 | 0.153 |
| Myrrha_octodecimguttata_490 | Tytthaspis_sedecimpunctata_587 | 0.153 |
| Myrrha_octodecimguttata_494 | Tytthaspis_sedecimpunctata_587 | 0.153 |
| Myrrha_octodecimguttata_496 | Tytthaspis_sedecimpunctata_587 | 0.153 |
| Myrrha_octodecimguttata_497 | Tytthaspis_sedecimpunctata_587 | 0.153 |
| Myrrha_octodecimguttata_501 | Tytthaspis_sedecimpunctata_587 | 0.153 |
| Myrrha_octodecimguttata_502 | Tytthaspis_sedecimpunctata_587 | 0.153 |
| Calvia_quatuordecimguttata_209 | Tytthaspis_sedecimpunctata_588 | 0.153 |
| Scymnus_rubromaculatus_813 | Scymnus_suturalis_832 | 0.153 |
| Scymnus_rubromaculatus_815 | Scymnus_suturalis_832 | 0.153 |
| Scymnus_rubromaculatus_816 | Scymnus_suturalis_832 | 0.153 |
| Scymnus_rubromaculatus_817 | Scymnus_suturalis_832 | 0.153 |
| Scymnus_rubromaculatus_818 | Scymnus_suturalis_832 | 0.153 |
| Scymnus_rubromaculatus_819 | Scymnus_suturalis_832 | 0.153 |
| Scymnus_rubromaculatus_820 | Scymnus_suturalis_832 | 0.153 |
| Scymnus_rubromaculatus_821 | Scymnus_suturalis_832 | 0.153 |
| Scymnus_rubromaculatus_822 | Scymnus_suturalis_832 | 0.153 |
| Scymnus_rubromaculatus_823 | Scymnus_suturalis_832 | 0.153 |
| Scymnus_rubromaculatus_824 | Scymnus_suturalis_832 | 0.153 |
| Scymnus_rubromaculatus_825 | Scymnus_suturalis_832 | 0.153 |
| Scymnus_rubromaculatus_826 | Scymnus_suturalis_832 | 0.153 |
| Scymnus_rubromaculatus_827 | Scymnus_suturalis_832 | 0.153 |
| Scymnus_rubromaculatus_813 | Scymnus_suturalis_835 | 0.153 |
| Scymnus_rubromaculatus_815 | Scymnus_suturalis_835 | 0.153 |
| Scymnus_rubromaculatus_816 | Scymnus_suturalis_835 | 0.153 |
| Scymnus_rubromaculatus_817 | Scymnus_suturalis_835 | 0.153 |
| Scymnus_rubromaculatus_818 | Scymnus_suturalis_835 | 0.153 |
| Scymnus_rubromaculatus_819 | Scymnus_suturalis_835 | 0.153 |
| Scymnus_rubromaculatus_820 | Scymnus_suturalis_835 | 0.153 |
| Scymnus_rubromaculatus_821 | Scymnus_suturalis_835 | 0.153 |

Supplementary Table S4 (continued)

| | | |
|---|---|---|
| Scymnus_rubromaculatus_822 | Scymnus_suturalis_835 | 0.153 |
| Scymnus_rubromaculatus_823 | Scymnus_suturalis_835 | 0.153 |
| Scymnus_rubromaculatus_824 | Scymnus_suturalis_835 | 0.153 |
| Scymnus_rubromaculatus_825 | Scymnus_suturalis_835 | 0.153 |
| Scymnus_rubromaculatus_826 | Scymnus_suturalis_835 | 0.153 |
| Scymnus_rubromaculatus_827 | Scymnus_suturalis_835 | 0.153 |
| Scymnus_rubromaculatus_813 | Scymnus_suturalis_836 | 0.153 |
| Scymnus_rubromaculatus_815 | Scymnus_suturalis_836 | 0.153 |
| Scymnus_rubromaculatus_816 | Scymnus_suturalis_836 | 0.153 |
| Scymnus_rubromaculatus_817 | Scymnus_suturalis_836 | 0.153 |
| Scymnus_rubromaculatus_818 | Scymnus_suturalis_836 | 0.153 |
| Scymnus_rubromaculatus_819 | Scymnus_suturalis_836 | 0.153 |
| Scymnus_rubromaculatus_820 | Scymnus_suturalis_836 | 0.153 |
| Scymnus_rubromaculatus_821 | Scymnus_suturalis_836 | 0.153 |
| Scymnus_rubromaculatus_822 | Scymnus_suturalis_836 | 0.153 |
| Scymnus_rubromaculatus_823 | Scymnus_suturalis_836 | 0.153 |
| Scymnus_rubromaculatus_824 | Scymnus_suturalis_836 | 0.153 |
| Scymnus_rubromaculatus_825 | Scymnus_suturalis_836 | 0.153 |
| Scymnus_rubromaculatus_826 | Scymnus_suturalis_836 | 0.153 |
| Scymnus_rubromaculatus_827 | Scymnus_suturalis_836 | 0.153 |
| Scymnus_rubromaculatus_814 | Scymnus_suturalis_837 | 0.153 |
| Scymnus_rubromaculatus_813 | Scymnus_suturalis_839 | 0.153 |
| Scymnus_rubromaculatus_815 | Scymnus_suturalis_839 | 0.153 |
| Scymnus_rubromaculatus_816 | Scymnus_suturalis_839 | 0.153 |
| Scymnus_rubromaculatus_817 | Scymnus_suturalis_839 | 0.153 |
| Scymnus_rubromaculatus_818 | Scymnus_suturalis_839 | 0.153 |
| Scymnus_rubromaculatus_819 | Scymnus_suturalis_839 | 0.153 |
| Scymnus_rubromaculatus_820 | Scymnus_suturalis_839 | 0.153 |
| Scymnus_rubromaculatus_821 | Scymnus_suturalis_839 | 0.153 |
| Scymnus_rubromaculatus_822 | Scymnus_suturalis_839 | 0.153 |
| Scymnus_rubromaculatus_823 | Scymnus_suturalis_839 | 0.153 |
| Scymnus_rubromaculatus_824 | Scymnus_suturalis_839 | 0.153 |
| Scymnus_rubromaculatus_825 | Scymnus_suturalis_839 | 0.153 |
| Scymnus_rubromaculatus_826 | Scymnus_suturalis_839 | 0.153 |
| Scymnus_rubromaculatus_827 | Scymnus_suturalis_839 | 0.153 |
| Calvia_quatuordecimguttata_207 | Tytthaspis_sedecimpunctata_583 | 0.153 |
| Calvia_quatuordecimguttata_218 | Tytthaspis_sedecimpunctata_583 | 0.153 |
| Coleomegilla_maculata_328 | Tytthaspis_sedecimpunctata_584 | 0.153 |
| Coleomegilla_maculata_332 | Tytthaspis_sedecimpunctata_584 | 0.153 |
| Calvia_quatuordecimguttata_207 | Tytthaspis_sedecimpunctata_585 | 0.153 |
| Calvia_quatuordecimguttata_218 | Tytthaspis_sedecimpunctata_585 | 0.153 |
| Coleomegilla_maculata_328 | Tytthaspis_sedecimpunctata_586 | 0.153 |
| Coleomegilla_maculata_332 | Tytthaspis_sedecimpunctata_586 | 0.153 |
| Calvia_quatuordecimguttata_201 | Tytthaspis_sedecimpunctata_587 | 0.153 |
| Calvia_quatuordecimguttata_202 | Tytthaspis_sedecimpunctata_587 | 0.153 |
| Calvia_quatuordecimguttata_210 | Tytthaspis_sedecimpunctata_587 | 0.153 |
| Coleomegilla_maculata_328 | Tytthaspis_sedecimpunctata_588 | 0.153 |
| Coleomegilla_maculata_332 | Tytthaspis_sedecimpunctata_588 | 0.153 |
| Calvia_quatuordecimguttata_206 | Tytthaspis_sedecimpunctata_593 | 0.153 |
| Calvia_quatuordecimguttata_209 | Tytthaspis_sedecimpunctata_593 | 0.153 |
| Calvia_quatuordecimguttata_216 | Tytthaspis_sedecimpunctata_593 | 0.153 |
| Scymnus_camptodromus_718 | Scymnus_rubromaculatus_822 | 0.153 |
| Scymnus_camptodromus_718 | Scymnus_rubromaculatus_823 | 0.153 |
| Scymnus_camptodromus_718 | Scymnus_rubromaculatus_824 | 0.153 |
| Scymnus_camptodromus_718 | Scymnus_rubromaculatus_825 | 0.153 |
| Scymnus_camptodromus_718 | Scymnus_rubromaculatus_826 | 0.153 |

| | | |
|---|---|---|
| Scymnus_camptodromus_718 | Scymnus_rubromaculatus_827 | 0.153 |
| Coleomegilla_maculata_327 | Tytthaspis_sedecimpunctata_577 | 0.153 |
| Coleomegilla_maculata_327 | Tytthaspis_sedecimpunctata_578 | 0.153 |
| Coleomegilla_maculata_327 | Tytthaspis_sedecimpunctata_579 | 0.153 |
| Coleomegilla_maculata_327 | Tytthaspis_sedecimpunctata_581 | 0.153 |
| Coleomegilla_maculata_327 | Tytthaspis_sedecimpunctata_582 | 0.153 |
| Calvia_quatuordecimguttata_204 | Tytthaspis_sedecimpunctata_583 | 0.153 |
| Calvia_quatuordecimguttata_208 | Tytthaspis_sedecimpunctata_583 | 0.153 |
| Calvia_quatuordecimguttata_217 | Tytthaspis_sedecimpunctata_583 | 0.153 |
| Coleomegilla_maculata_329 | Tytthaspis_sedecimpunctata_583 | 0.153 |
| Coleomegilla_maculata_330 | Tytthaspis_sedecimpunctata_583 | 0.153 |
| Coleomegilla_maculata_331 | Tytthaspis_sedecimpunctata_583 | 0.153 |
| Coleomegilla_maculata_333 | Tytthaspis_sedecimpunctata_583 | 0.153 |
| Coleomegilla_maculata_334 | Tytthaspis_sedecimpunctata_583 | 0.153 |
| Coleomegilla_maculata_335 | Tytthaspis_sedecimpunctata_583 | 0.153 |
| Coleomegilla_maculata_336 | Tytthaspis_sedecimpunctata_583 | 0.153 |
| Coleomegilla_maculata_337 | Tytthaspis_sedecimpunctata_583 | 0.153 |
| Coleomegilla_maculata_338 | Tytthaspis_sedecimpunctata_583 | 0.153 |
| Coleomegilla_maculata_339 | Tytthaspis_sedecimpunctata_583 | 0.153 |
| Calvia_quatuordecimguttata_204 | Tytthaspis_sedecimpunctata_585 | 0.153 |
| Calvia_quatuordecimguttata_208 | Tytthaspis_sedecimpunctata_585 | 0.153 |
| Calvia_quatuordecimguttata_217 | Tytthaspis_sedecimpunctata_585 | 0.153 |
| Coleomegilla_maculata_328 | Tytthaspis_sedecimpunctata_585 | 0.153 |
| Coleomegilla_maculata_332 | Tytthaspis_sedecimpunctata_585 | 0.153 |
| Coleomegilla_maculata_327 | Tytthaspis_sedecimpunctata_590 | 0.153 |
| Coleomegilla_maculata_327 | Tytthaspis_sedecimpunctata_592 | 0.153 |
| Coleomegilla_maculata_327 | Tytthaspis_sedecimpunctata_593 | 0.153 |
| Coleomegilla_maculata_328 | Tytthaspis_sedecimpunctata_593 | 0.153 |
| Coleomegilla_maculata_332 | Tytthaspis_sedecimpunctata_593 | 0.153 |
| Coleomegilla_maculata_327 | Tytthaspis_sedecimpunctata_594 | 0.153 |
| Coleomegilla_maculata_327 | Tytthaspis_sedecimpunctata_595 | 0.153 |
| Scymnus_nigrinus_784 | Scymnus_suturalis_843 | 0.153 |
| Scymnus_nigrinus_784 | Scymnus_suturalis_845 | 0.153 |
| Calvia_quatuordecimguttata_205 | Tytthaspis_sedecimpunctata_577 | 0.153 |
| Calvia_quatuordecimguttata_205 | Tytthaspis_sedecimpunctata_578 | 0.153 |
| Calvia_quatuordecimguttata_205 | Tytthaspis_sedecimpunctata_579 | 0.153 |
| Calvia_quatuordecimguttata_203 | Tytthaspis_sedecimpunctata_580 | 0.153 |
| Calvia_quatuordecimguttata_205 | Tytthaspis_sedecimpunctata_581 | 0.153 |
| Calvia_quatuordecimguttata_205 | Tytthaspis_sedecimpunctata_582 | 0.153 |
| Calvia_quatuordecimguttata_203 | Tytthaspis_sedecimpunctata_584 | 0.153 |
| Calvia_quatuordecimguttata_205 | Tytthaspis_sedecimpunctata_590 | 0.153 |
| Calvia_quatuordecimguttata_205 | Tytthaspis_sedecimpunctata_592 | 0.153 |
| Calvia_quatuordecimguttata_205 | Tytthaspis_sedecimpunctata_593 | 0.153 |
| Calvia_quatuordecimguttata_205 | Tytthaspis_sedecimpunctata_594 | 0.153 |
| Calvia_quatuordecimguttata_205 | Tytthaspis_sedecimpunctata_595 | 0.153 |
| Scymnus_nigrinus_784 | Scymnus_suturalis_837 | 0.153 |
| Scymnus_ferrugatus_724 | Scymnus_suturalis_833 | 0.154 |
| Scymnus_ferrugatus_729 | Scymnus_suturalis_833 | 0.154 |
| Macronaemia_episcopalis_478 | Tytthaspis_sedecimpunctata_577 | 0.154 |
| Macronaemia_episcopalis_478 | Tytthaspis_sedecimpunctata_578 | 0.154 |
| Macronaemia_episcopalis_478 | Tytthaspis_sedecimpunctata_579 | 0.154 |
| Macronaemia_episcopalis_478 | Tytthaspis_sedecimpunctata_580 | 0.154 |
| Macronaemia_episcopalis_478 | Tytthaspis_sedecimpunctata_581 | 0.154 |
| Macronaemia_episcopalis_478 | Tytthaspis_sedecimpunctata_582 | 0.154 |
| Macronaemia_episcopalis_478 | Tytthaspis_sedecimpunctata_585 | 0.154 |
| Macronaemia_episcopalis_478 | Tytthaspis_sedecimpunctata_590 | 0.154 |

| | | |
|---|---|---|
| Macronaemia_episcopalis_478 | Tytthaspis_sedecimpunctata_594 | 0.154 |
| Macronaemia_episcopalis_478 | Tytthaspis_sedecimpunctata_595 | 0.154 |
| Scymnus_nigrinus_787 | Scymnus_suturalis_838 | 0.154 |
| Scymnus_nigrinus_787 | Scymnus_suturalis_841 | 0.154 |
| Scymnus_nigrinus_787 | Scymnus_suturalis_842 | 0.154 |
| Scymnus_nigrinus_787 | Scymnus_suturalis_844 | 0.154 |
| Scymnus_nigrinus_787 | Scymnus_suturalis_846 | 0.154 |
| Scymnus_nigrinus_787 | Scymnus_suturalis_847 | 0.154 |
| Scymnus_nigrinus_787 | Scymnus_suturalis_848 | 0.154 |
| Scymnus_nigrinus_787 | Scymnus_suturalis_832 | 0.154 |
| Scymnus_nigrinus_787 | Scymnus_suturalis_835 | 0.154 |
| Scymnus_nigrinus_787 | Scymnus_suturalis_836 | 0.154 |
| Scymnus_nigrinus_787 | Scymnus_suturalis_839 | 0.154 |
| Scymnus_nebulosus_781 | Scymnus_rubromaculatus_822 | 0.154 |
| Scymnus_nebulosus_781 | Scymnus_rubromaculatus_823 | 0.154 |
| Scymnus_nebulosus_781 | Scymnus_rubromaculatus_824 | 0.154 |
| Scymnus_nebulosus_781 | Scymnus_rubromaculatus_825 | 0.154 |
| Scymnus_nebulosus_781 | Scymnus_rubromaculatus_826 | 0.154 |
| Scymnus_nebulosus_781 | Scymnus_rubromaculatus_827 | 0.154 |
| Scymnus_sinuanodulus_828 | Scymnus_suturalis_840 | 0.154 |
| Anisosticta_novemdecimpunctata_159 | Tytthaspis_sedecimpunctata_589 | 0.154 |
| Anisosticta_novemdecimpunctata_163 | Tytthaspis_sedecimpunctata_589 | 0.154 |
| Anisosticta_novemdecimpunctata_171 | Tytthaspis_sedecimpunctata_589 | 0.154 |
| Scymnus_pallipediformis_812 | Scymnus_rubromaculatus_822 | 0.154 |
| Scymnus_pallipediformis_812 | Scymnus_rubromaculatus_823 | 0.154 |
| Scymnus_pallipediformis_812 | Scymnus_rubromaculatus_824 | 0.154 |
| Scymnus_pallipediformis_812 | Scymnus_rubromaculatus_825 | 0.154 |
| Scymnus_pallipediformis_812 | Scymnus_rubromaculatus_826 | 0.154 |
| Scymnus_pallipediformis_812 | Scymnus_rubromaculatus_827 | 0.154 |
| Scymnus_nebulosus_783 | Scymnus_sinuanodulus_830 | 0.154 |
| Scymnus_interruptus_760 | Scymnus_sinuanodulus_829 | 0.154 |
| Scymnus_interruptus_765 | Scymnus_sinuanodulus_829 | 0.154 |
| Scymnus_interruptus_768 | Scymnus_sinuanodulus_829 | 0.154 |
| Scymnus_camptodromus_711 | Scymnus_rubromaculatus_822 | 0.154 |
| Scymnus_camptodromus_711 | Scymnus_rubromaculatus_823 | 0.154 |
| Scymnus_camptodromus_711 | Scymnus_rubromaculatus_824 | 0.154 |
| Scymnus_camptodromus_711 | Scymnus_rubromaculatus_825 | 0.154 |
| Scymnus_camptodromus_711 | Scymnus_rubromaculatus_826 | 0.154 |
| Scymnus_camptodromus_711 | Scymnus_rubromaculatus_827 | 0.154 |
| Anisosticta_novemdecimpunctata_159 | Tytthaspis_sedecimpunctata_584 | 0.154 |
| Anisosticta_novemdecimpunctata_163 | Tytthaspis_sedecimpunctata_584 | 0.154 |
| Anisosticta_novemdecimpunctata_171 | Tytthaspis_sedecimpunctata_584 | 0.154 |
| Anisosticta_novemdecimpunctata_159 | Tytthaspis_sedecimpunctata_587 | 0.154 |
| Anisosticta_novemdecimpunctata_163 | Tytthaspis_sedecimpunctata_587 | 0.154 |
| Anisosticta_novemdecimpunctata_171 | Tytthaspis_sedecimpunctata_587 | 0.154 |
| Anisosticta_novemdecimpunctata_160 | Tytthaspis_sedecimpunctata_591 | 0.154 |
| Anisosticta_novemdecimpunctata_161 | Tytthaspis_sedecimpunctata_591 | 0.154 |
| Anisosticta_novemdecimpunctata_162 | Tytthaspis_sedecimpunctata_577 | 0.154 |
| Anisosticta_novemdecimpunctata_164 | Tytthaspis_sedecimpunctata_577 | 0.154 |
| Anisosticta_novemdecimpunctata_166 | Tytthaspis_sedecimpunctata_577 | 0.154 |
| Anisosticta_novemdecimpunctata_167 | Tytthaspis_sedecimpunctata_577 | 0.154 |
| Anisosticta_novemdecimpunctata_168 | Tytthaspis_sedecimpunctata_577 | 0.154 |
| Anisosticta_novemdecimpunctata_162 | Tytthaspis_sedecimpunctata_578 | 0.154 |
| Anisosticta_novemdecimpunctata_164 | Tytthaspis_sedecimpunctata_578 | 0.154 |
| Anisosticta_novemdecimpunctata_166 | Tytthaspis_sedecimpunctata_578 | 0.154 |
| Anisosticta_novemdecimpunctata_167 | Tytthaspis_sedecimpunctata_578 | 0.154 |

| | | |
|---|---|---|
| Anisosticta_novemdecimpunctata_168 | Tytthaspis_sedecimpunctata_578 | 0.154 |
| Anisosticta_novemdecimpunctata_162 | Tytthaspis_sedecimpunctata_579 | 0.154 |
| Anisosticta_novemdecimpunctata_164 | Tytthaspis_sedecimpunctata_579 | 0.154 |
| Anisosticta_novemdecimpunctata_166 | Tytthaspis_sedecimpunctata_579 | 0.154 |
| Anisosticta_novemdecimpunctata_167 | Tytthaspis_sedecimpunctata_579 | 0.154 |
| Anisosticta_novemdecimpunctata_168 | Tytthaspis_sedecimpunctata_579 | 0.154 |
| Anisosticta_novemdecimpunctata_162 | Tytthaspis_sedecimpunctata_581 | 0.154 |
| Anisosticta_novemdecimpunctata_164 | Tytthaspis_sedecimpunctata_581 | 0.154 |
| Anisosticta_novemdecimpunctata_166 | Tytthaspis_sedecimpunctata_581 | 0.154 |
| Anisosticta_novemdecimpunctata_167 | Tytthaspis_sedecimpunctata_581 | 0.154 |
| Anisosticta_novemdecimpunctata_168 | Tytthaspis_sedecimpunctata_581 | 0.154 |
| Anisosticta_novemdecimpunctata_162 | Tytthaspis_sedecimpunctata_582 | 0.154 |
| Anisosticta_novemdecimpunctata_164 | Tytthaspis_sedecimpunctata_582 | 0.154 |
| Anisosticta_novemdecimpunctata_166 | Tytthaspis_sedecimpunctata_582 | 0.154 |
| Anisosticta_novemdecimpunctata_167 | Tytthaspis_sedecimpunctata_582 | 0.154 |
| Anisosticta_novemdecimpunctata_168 | Tytthaspis_sedecimpunctata_582 | 0.154 |
| Anisosticta_novemdecimpunctata_159 | Tytthaspis_sedecimpunctata_583 | 0.154 |
| Anisosticta_novemdecimpunctata_163 | Tytthaspis_sedecimpunctata_583 | 0.154 |
| Anisosticta_novemdecimpunctata_171 | Tytthaspis_sedecimpunctata_583 | 0.154 |
| Anisosticta_novemdecimpunctata_162 | Tytthaspis_sedecimpunctata_590 | 0.154 |
| Anisosticta_novemdecimpunctata_164 | Tytthaspis_sedecimpunctata_590 | 0.154 |
| Anisosticta_novemdecimpunctata_166 | Tytthaspis_sedecimpunctata_590 | 0.154 |
| Anisosticta_novemdecimpunctata_167 | Tytthaspis_sedecimpunctata_590 | 0.154 |
| Anisosticta_novemdecimpunctata_168 | Tytthaspis_sedecimpunctata_590 | 0.154 |
| Anisosticta_novemdecimpunctata_162 | Tytthaspis_sedecimpunctata_593 | 0.154 |
| Anisosticta_novemdecimpunctata_164 | Tytthaspis_sedecimpunctata_593 | 0.154 |
| Anisosticta_novemdecimpunctata_166 | Tytthaspis_sedecimpunctata_593 | 0.154 |
| Anisosticta_novemdecimpunctata_167 | Tytthaspis_sedecimpunctata_593 | 0.154 |
| Anisosticta_novemdecimpunctata_168 | Tytthaspis_sedecimpunctata_593 | 0.154 |
| Anisosticta_novemdecimpunctata_162 | Tytthaspis_sedecimpunctata_594 | 0.154 |
| Anisosticta_novemdecimpunctata_164 | Tytthaspis_sedecimpunctata_594 | 0.154 |
| Anisosticta_novemdecimpunctata_166 | Tytthaspis_sedecimpunctata_594 | 0.154 |
| Anisosticta_novemdecimpunctata_167 | Tytthaspis_sedecimpunctata_594 | 0.154 |
| Anisosticta_novemdecimpunctata_168 | Tytthaspis_sedecimpunctata_594 | 0.154 |
| Anisosticta_novemdecimpunctata_162 | Tytthaspis_sedecimpunctata_595 | 0.154 |
| Anisosticta_novemdecimpunctata_164 | Tytthaspis_sedecimpunctata_595 | 0.154 |
| Anisosticta_novemdecimpunctata_166 | Tytthaspis_sedecimpunctata_595 | 0.154 |
| Anisosticta_novemdecimpunctata_167 | Tytthaspis_sedecimpunctata_595 | 0.154 |
| Anisosticta_novemdecimpunctata_168 | Tytthaspis_sedecimpunctata_595 | 0.154 |
| Scymnus_nebulosus_783 | Scymnus_sinuanodulus_831 | 0.154 |
| Anisosticta_novemdecimpunctata_164 | Tytthaspis_sedecimpunctata_580 | 0.154 |
| Anisosticta_novemdecimpunctata_166 | Tytthaspis_sedecimpunctata_580 | 0.154 |
| Anisosticta_novemdecimpunctata_167 | Tytthaspis_sedecimpunctata_580 | 0.154 |
| Anisosticta_novemdecimpunctata_168 | Tytthaspis_sedecimpunctata_580 | 0.154 |
| Halyzia_sedecimguttata_346 | Vibidia_duodecimguttata_596 | 0.154 |
| Halyzia_sedecimguttata_347 | Vibidia_duodecimguttata_596 | 0.154 |
| Halyzia_sedecimguttata_348 | Vibidia_duodecimguttata_596 | 0.154 |
| Halyzia_sedecimguttata_346 | Vibidia_duodecimguttata_598 | 0.154 |
| Halyzia_sedecimguttata_347 | Vibidia_duodecimguttata_598 | 0.154 |
| Halyzia_sedecimguttata_348 | Vibidia_duodecimguttata_598 | 0.154 |
| Halyzia_sedecimguttata_346 | Vibidia_duodecimguttata_603 | 0.154 |
| Halyzia_sedecimguttata_347 | Vibidia_duodecimguttata_603 | 0.154 |
| Halyzia_sedecimguttata_348 | Vibidia_duodecimguttata_603 | 0.154 |
| Scymnus_abietis_684 | Scymnus_sinuanodulus_830 | 0.154 |
| Scymnus_abietis_686 | Scymnus_sinuanodulus_830 | 0.154 |
| Scymnus_abietis_688 | Scymnus_sinuanodulus_830 | 0.154 |

| | | |
|---|---|---|
| Halyzia_sedecimguttata_345 | Vibidia_duodecimguttata_597 | 0.155 |
| Halyzia_sedecimguttata_349 | Vibidia_duodecimguttata_597 | 0.155 |
| Halyzia_sedecimguttata_350 | Vibidia_duodecimguttata_597 | 0.155 |
| Halyzia_sedecimguttata_345 | Vibidia_duodecimguttata_600 | 0.155 |
| Halyzia_sedecimguttata_349 | Vibidia_duodecimguttata_600 | 0.155 |
| Halyzia_sedecimguttata_350 | Vibidia_duodecimguttata_600 | 0.155 |
| Halyzia_sedecimguttata_351 | Vibidia_duodecimguttata_599 | 0.155 |
| Halyzia_sedecimguttata_353 | Vibidia_duodecimguttata_599 | 0.155 |
| Halyzia_sedecimguttata_351 | Vibidia_duodecimguttata_602 | 0.155 |
| Halyzia_sedecimguttata_353 | Vibidia_duodecimguttata_602 | 0.155 |
| Halyzia_sedecimguttata_345 | Vibidia_duodecimguttata_603 | 0.155 |
| Halyzia_sedecimguttata_349 | Vibidia_duodecimguttata_603 | 0.155 |
| Halyzia_sedecimguttata_350 | Vibidia_duodecimguttata_603 | 0.155 |
| Scymnus_abietis_689 | Scymnus_sinuanodulus_830 | 0.155 |
| Brumoides_suturalis_2 | Tytthaspis_sedecimpunctata_589 | 0.155 |
| Brumoides_suturalis_2 | Tytthaspis_sedecimpunctata_587 | 0.155 |
| Harmonia_conformis_374 | Tytthaspis_sedecimpunctata_585 | 0.155 |
| Harmonia_conformis_374 | Tytthaspis_sedecimpunctata_593 | 0.155 |
| Harmonia_conformis_374 | Tytthaspis_sedecimpunctata_586 | 0.155 |
| Harmonia_conformis_374 | Tytthaspis_sedecimpunctata_588 | 0.155 |
| Mulsantina_hudsonica_481 | Tytthaspis_sedecimpunctata_584 | 0.155 |
| Mulsantina_hudsonica_482 | Tytthaspis_sedecimpunctata_584 | 0.155 |
| Mulsantina_hudsonica_480 | Tytthaspis_sedecimpunctata_586 | 0.155 |
| Mulsantina_hudsonica_480 | Tytthaspis_sedecimpunctata_587 | 0.155 |
| Mulsantina_hudsonica_480 | Tytthaspis_sedecimpunctata_588 | 0.155 |
| Mulsantina_hudsonica_479 | Tytthaspis_sedecimpunctata_591 | 0.155 |
| Mulsantina_hudsonica_480 | Tytthaspis_sedecimpunctata_585 | 0.155 |
| Scymnus_interruptus_760 | Scymnus_suturalis_838 | 0.155 |
| Scymnus_interruptus_765 | Scymnus_suturalis_838 | 0.155 |
| Scymnus_interruptus_767 | Scymnus_suturalis_838 | 0.155 |
| Scymnus_interruptus_768 | Scymnus_suturalis_838 | 0.155 |
| Scymnus_interruptus_760 | Scymnus_suturalis_840 | 0.155 |
| Scymnus_interruptus_765 | Scymnus_suturalis_840 | 0.155 |
| Scymnus_interruptus_767 | Scymnus_suturalis_840 | 0.155 |
| Scymnus_interruptus_768 | Scymnus_suturalis_840 | 0.155 |
| Coelophora_inaequalis_317 | Tytthaspis_sedecimpunctata_589 | 0.155 |
| Scymnus_interruptus_760 | Scymnus_suturalis_834 | 0.155 |
| Scymnus_interruptus_765 | Scymnus_suturalis_834 | 0.155 |
| Scymnus_interruptus_767 | Scymnus_suturalis_834 | 0.155 |
| Scymnus_interruptus_768 | Scymnus_suturalis_834 | 0.155 |
| Coelophora_inaequalis_317 | Tytthaspis_sedecimpunctata_587 | 0.155 |
| Scymnus_haemorrhoidalis_747 | Tytthaspis_sedecimpunctata_577 | 0.155 |
| Scymnus_haemorrhoidalis_748 | Tytthaspis_sedecimpunctata_577 | 0.155 |
| Scymnus_haemorrhoidalis_747 | Tytthaspis_sedecimpunctata_578 | 0.155 |
| Scymnus_haemorrhoidalis_748 | Tytthaspis_sedecimpunctata_578 | 0.155 |
| Scymnus_haemorrhoidalis_747 | Tytthaspis_sedecimpunctata_579 | 0.155 |
| Scymnus_haemorrhoidalis_748 | Tytthaspis_sedecimpunctata_579 | 0.155 |
| Scymnus_haemorrhoidalis_747 | Tytthaspis_sedecimpunctata_581 | 0.155 |
| Scymnus_haemorrhoidalis_748 | Tytthaspis_sedecimpunctata_581 | 0.155 |
| Scymnus_haemorrhoidalis_747 | Tytthaspis_sedecimpunctata_582 | 0.155 |
| Scymnus_haemorrhoidalis_748 | Tytthaspis_sedecimpunctata_582 | 0.155 |
| Scymnus_haemorrhoidalis_747 | Tytthaspis_sedecimpunctata_590 | 0.155 |
| Scymnus_haemorrhoidalis_748 | Tytthaspis_sedecimpunctata_590 | 0.155 |
| Exochomus_nigromaculatus_49 | Tytthaspis_sedecimpunctata_591 | 0.155 |
| Scymnus_haemorrhoidalis_747 | Tytthaspis_sedecimpunctata_592 | 0.155 |
| Scymnus_haemorrhoidalis_748 | Tytthaspis_sedecimpunctata_592 | 0.155 |

| | | |
|---|---|---|
| Scymnus_haemorrhoidalis_747 | Tytthaspis_sedecimpunctata_594 | 0.155 |
| Scymnus_haemorrhoidalis_748 | Tytthaspis_sedecimpunctata_594 | 0.155 |
| Scymnus_haemorrhoidalis_747 | Tytthaspis_sedecimpunctata_595 | 0.155 |
| Scymnus_haemorrhoidalis_748 | Tytthaspis_sedecimpunctata_595 | 0.155 |
| Scymnus_haemorrhoidalis_747 | Tytthaspis_sedecimpunctata_580 | 0.155 |
| Scymnus_haemorrhoidalis_748 | Tytthaspis_sedecimpunctata_580 | 0.155 |
| Myrrha_octodecimguttata_488 | Tytthaspis_sedecimpunctata_577 | 0.155 |
| Myrrha_octodecimguttata_491 | Tytthaspis_sedecimpunctata_577 | 0.155 |
| Myrrha_octodecimguttata_492 | Tytthaspis_sedecimpunctata_577 | 0.155 |
| Myrrha_octodecimguttata_488 | Tytthaspis_sedecimpunctata_578 | 0.155 |
| Myrrha_octodecimguttata_491 | Tytthaspis_sedecimpunctata_578 | 0.155 |
| Myrrha_octodecimguttata_492 | Tytthaspis_sedecimpunctata_578 | 0.155 |
| Myrrha_octodecimguttata_488 | Tytthaspis_sedecimpunctata_579 | 0.155 |
| Myrrha_octodecimguttata_491 | Tytthaspis_sedecimpunctata_579 | 0.155 |
| Myrrha_octodecimguttata_492 | Tytthaspis_sedecimpunctata_579 | 0.155 |
| Myrrha_octodecimguttata_488 | Tytthaspis_sedecimpunctata_581 | 0.155 |
| Myrrha_octodecimguttata_491 | Tytthaspis_sedecimpunctata_581 | 0.155 |
| Myrrha_octodecimguttata_492 | Tytthaspis_sedecimpunctata_581 | 0.155 |
| Myrrha_octodecimguttata_488 | Tytthaspis_sedecimpunctata_582 | 0.155 |
| Myrrha_octodecimguttata_491 | Tytthaspis_sedecimpunctata_582 | 0.155 |
| Myrrha_octodecimguttata_492 | Tytthaspis_sedecimpunctata_582 | 0.155 |
| Myrrha_octodecimguttata_489 | Tytthaspis_sedecimpunctata_583 | 0.155 |
| Myrrha_octodecimguttata_500 | Tytthaspis_sedecimpunctata_583 | 0.155 |
| Myrrha_octodecimguttata_485 | Tytthaspis_sedecimpunctata_584 | 0.155 |
| Myrrha_octodecimguttata_486 | Tytthaspis_sedecimpunctata_584 | 0.155 |
| Myrrha_octodecimguttata_487 | Tytthaspis_sedecimpunctata_584 | 0.155 |
| Myrrha_octodecimguttata_490 | Tytthaspis_sedecimpunctata_584 | 0.155 |
| Myrrha_octodecimguttata_494 | Tytthaspis_sedecimpunctata_584 | 0.155 |
| Myrrha_octodecimguttata_496 | Tytthaspis_sedecimpunctata_584 | 0.155 |
| Myrrha_octodecimguttata_497 | Tytthaspis_sedecimpunctata_584 | 0.155 |
| Myrrha_octodecimguttata_501 | Tytthaspis_sedecimpunctata_584 | 0.155 |
| Myrrha_octodecimguttata_502 | Tytthaspis_sedecimpunctata_584 | 0.155 |
| Myrrha_octodecimguttata_489 | Tytthaspis_sedecimpunctata_585 | 0.155 |
| Myrrha_octodecimguttata_500 | Tytthaspis_sedecimpunctata_585 | 0.155 |
| Myrrha_octodecimguttata_488 | Tytthaspis_sedecimpunctata_590 | 0.155 |
| Myrrha_octodecimguttata_491 | Tytthaspis_sedecimpunctata_590 | 0.155 |
| Myrrha_octodecimguttata_492 | Tytthaspis_sedecimpunctata_590 | 0.155 |
| Myrrha_octodecimguttata_489 | Tytthaspis_sedecimpunctata_592 | 0.155 |
| Myrrha_octodecimguttata_500 | Tytthaspis_sedecimpunctata_592 | 0.155 |
| Myrrha_octodecimguttata_488 | Tytthaspis_sedecimpunctata_594 | 0.155 |
| Myrrha_octodecimguttata_491 | Tytthaspis_sedecimpunctata_594 | 0.155 |
| Myrrha_octodecimguttata_492 | Tytthaspis_sedecimpunctata_594 | 0.155 |
| Myrrha_octodecimguttata_488 | Tytthaspis_sedecimpunctata_595 | 0.155 |
| Myrrha_octodecimguttata_491 | Tytthaspis_sedecimpunctata_595 | 0.155 |
| Myrrha_octodecimguttata_492 | Tytthaspis_sedecimpunctata_595 | 0.155 |
| Myrrha_octodecimguttata_488 | Tytthaspis_sedecimpunctata_580 | 0.155 |
| Myrrha_octodecimguttata_491 | Tytthaspis_sedecimpunctata_580 | 0.155 |
| Myrrha_octodecimguttata_492 | Tytthaspis_sedecimpunctata_580 | 0.155 |
| Oenopia_conglobata_522 | Tytthaspis_sedecimpunctata_593 | 0.155 |
| Scymnus_rubromaculatus_813 | Scymnus_suturalis_833 | 0.155 |
| Scymnus_rubromaculatus_815 | Scymnus_suturalis_833 | 0.155 |
| Scymnus_rubromaculatus_816 | Scymnus_suturalis_833 | 0.155 |
| Scymnus_rubromaculatus_817 | Scymnus_suturalis_833 | 0.155 |
| Scymnus_rubromaculatus_818 | Scymnus_suturalis_833 | 0.155 |
| Scymnus_rubromaculatus_819 | Scymnus_suturalis_833 | 0.155 |
| Scymnus_rubromaculatus_820 | Scymnus_suturalis_833 | 0.155 |

| | | |
|---|---|---|
| Scymnus_rubromaculatus_821 | Scymnus_suturalis_833 | 0.155 |
| Scymnus_rubromaculatus_822 | Scymnus_suturalis_833 | 0.155 |
| Scymnus_rubromaculatus_823 | Scymnus_suturalis_833 | 0.155 |
| Scymnus_rubromaculatus_824 | Scymnus_suturalis_833 | 0.155 |
| Scymnus_rubromaculatus_825 | Scymnus_suturalis_833 | 0.155 |
| Scymnus_rubromaculatus_826 | Scymnus_suturalis_833 | 0.155 |
| Scymnus_rubromaculatus_827 | Scymnus_suturalis_833 | 0.155 |
| Scymnus_rubromaculatus_813 | Scymnus_suturalis_838 | 0.155 |
| Scymnus_rubromaculatus_815 | Scymnus_suturalis_838 | 0.155 |
| Scymnus_rubromaculatus_816 | Scymnus_suturalis_838 | 0.155 |
| Scymnus_rubromaculatus_817 | Scymnus_suturalis_838 | 0.155 |
| Scymnus_rubromaculatus_818 | Scymnus_suturalis_838 | 0.155 |
| Scymnus_rubromaculatus_819 | Scymnus_suturalis_838 | 0.155 |
| Scymnus_rubromaculatus_820 | Scymnus_suturalis_838 | 0.155 |
| Scymnus_rubromaculatus_821 | Scymnus_suturalis_838 | 0.155 |
| Scymnus_rubromaculatus_822 | Scymnus_suturalis_838 | 0.155 |
| Scymnus_rubromaculatus_823 | Scymnus_suturalis_838 | 0.155 |
| Scymnus_rubromaculatus_824 | Scymnus_suturalis_838 | 0.155 |
| Scymnus_rubromaculatus_825 | Scymnus_suturalis_838 | 0.155 |
| Scymnus_rubromaculatus_826 | Scymnus_suturalis_838 | 0.155 |
| Scymnus_rubromaculatus_827 | Scymnus_suturalis_838 | 0.155 |
| Scymnus_rubromaculatus_813 | Scymnus_suturalis_840 | 0.155 |
| Scymnus_rubromaculatus_815 | Scymnus_suturalis_840 | 0.155 |
| Scymnus_rubromaculatus_816 | Scymnus_suturalis_840 | 0.155 |
| Scymnus_rubromaculatus_817 | Scymnus_suturalis_840 | 0.155 |
| Scymnus_rubromaculatus_818 | Scymnus_suturalis_840 | 0.155 |
| Scymnus_rubromaculatus_819 | Scymnus_suturalis_840 | 0.155 |
| Scymnus_rubromaculatus_820 | Scymnus_suturalis_840 | 0.155 |
| Scymnus_rubromaculatus_821 | Scymnus_suturalis_840 | 0.155 |
| Scymnus_rubromaculatus_822 | Scymnus_suturalis_840 | 0.155 |
| Scymnus_rubromaculatus_823 | Scymnus_suturalis_840 | 0.155 |
| Scymnus_rubromaculatus_824 | Scymnus_suturalis_840 | 0.155 |
| Scymnus_rubromaculatus_825 | Scymnus_suturalis_840 | 0.155 |
| Scymnus_rubromaculatus_826 | Scymnus_suturalis_840 | 0.155 |
| Scymnus_rubromaculatus_827 | Scymnus_suturalis_840 | 0.155 |
| Scymnus_rubromaculatus_814 | Scymnus_suturalis_841 | 0.155 |
| Scymnus_rubromaculatus_814 | Scymnus_suturalis_842 | 0.155 |
| Scymnus_rubromaculatus_814 | Scymnus_suturalis_844 | 0.155 |
| Scymnus_rubromaculatus_814 | Scymnus_suturalis_846 | 0.155 |
| Scymnus_rubromaculatus_814 | Scymnus_suturalis_847 | 0.155 |
| Scymnus_rubromaculatus_814 | Scymnus_suturalis_848 | 0.155 |
| Calvia_quatuordecimguttata_209 | Tytthaspis_sedecimpunctata_586 | 0.155 |
| Calvia_decemguttata_195 | Tytthaspis_sedecimpunctata_588 | 0.155 |
| Calvia_decemguttata_199 | Tytthaspis_sedecimpunctata_588 | 0.155 |
| Calvia_decemguttata_200 | Tytthaspis_sedecimpunctata_588 | 0.155 |
| Calvia_quatuordecimguttata_218 | Tytthaspis_sedecimpunctata_588 | 0.155 |
| Scymnus_rubromaculatus_814 | Scymnus_suturalis_832 | 0.155 |
| Scymnus_rubromaculatus_813 | Scymnus_suturalis_834 | 0.155 |
| Scymnus_rubromaculatus_815 | Scymnus_suturalis_834 | 0.155 |
| Scymnus_rubromaculatus_816 | Scymnus_suturalis_834 | 0.155 |
| Scymnus_rubromaculatus_817 | Scymnus_suturalis_834 | 0.155 |
| Scymnus_rubromaculatus_818 | Scymnus_suturalis_834 | 0.155 |
| Scymnus_rubromaculatus_819 | Scymnus_suturalis_834 | 0.155 |
| Scymnus_rubromaculatus_820 | Scymnus_suturalis_834 | 0.155 |
| Scymnus_rubromaculatus_821 | Scymnus_suturalis_834 | 0.155 |
| Scymnus_rubromaculatus_822 | Scymnus_suturalis_834 | 0.155 |

| | | |
|---|---|---|
| Scymnus_rubromaculatus_823 | Scymnus_suturalis_834 | 0.155 |
| Scymnus_rubromaculatus_824 | Scymnus_suturalis_834 | 0.155 |
| Scymnus_rubromaculatus_825 | Scymnus_suturalis_834 | 0.155 |
| Scymnus_rubromaculatus_826 | Scymnus_suturalis_834 | 0.155 |
| Scymnus_rubromaculatus_827 | Scymnus_suturalis_834 | 0.155 |
| Scymnus_rubromaculatus_814 | Scymnus_suturalis_835 | 0.155 |
| Scymnus_rubromaculatus_814 | Scymnus_suturalis_836 | 0.155 |
| Scymnus_rubromaculatus_814 | Scymnus_suturalis_839 | 0.155 |
| Coleomegilla_maculata_329 | Tytthaspis_sedecimpunctata_584 | 0.155 |
| Coleomegilla_maculata_330 | Tytthaspis_sedecimpunctata_584 | 0.155 |
| Coleomegilla_maculata_331 | Tytthaspis_sedecimpunctata_584 | 0.155 |
| Coleomegilla_maculata_333 | Tytthaspis_sedecimpunctata_584 | 0.155 |
| Coleomegilla_maculata_334 | Tytthaspis_sedecimpunctata_584 | 0.155 |
| Coleomegilla_maculata_335 | Tytthaspis_sedecimpunctata_584 | 0.155 |
| Coleomegilla_maculata_336 | Tytthaspis_sedecimpunctata_584 | 0.155 |
| Coleomegilla_maculata_337 | Tytthaspis_sedecimpunctata_584 | 0.155 |
| Coleomegilla_maculata_338 | Tytthaspis_sedecimpunctata_584 | 0.155 |
| Coleomegilla_maculata_339 | Tytthaspis_sedecimpunctata_584 | 0.155 |
| Coleomegilla_maculata_329 | Tytthaspis_sedecimpunctata_586 | 0.155 |
| Coleomegilla_maculata_330 | Tytthaspis_sedecimpunctata_586 | 0.155 |
| Coleomegilla_maculata_331 | Tytthaspis_sedecimpunctata_586 | 0.155 |
| Coleomegilla_maculata_333 | Tytthaspis_sedecimpunctata_586 | 0.155 |
| Coleomegilla_maculata_334 | Tytthaspis_sedecimpunctata_586 | 0.155 |
| Coleomegilla_maculata_335 | Tytthaspis_sedecimpunctata_586 | 0.155 |
| Coleomegilla_maculata_336 | Tytthaspis_sedecimpunctata_586 | 0.155 |
| Coleomegilla_maculata_337 | Tytthaspis_sedecimpunctata_586 | 0.155 |
| Coleomegilla_maculata_338 | Tytthaspis_sedecimpunctata_586 | 0.155 |
| Coleomegilla_maculata_339 | Tytthaspis_sedecimpunctata_586 | 0.155 |
| Calvia_quatuordecimguttata_204 | Tytthaspis_sedecimpunctata_588 | 0.155 |
| Calvia_quatuordecimguttata_208 | Tytthaspis_sedecimpunctata_588 | 0.155 |
| Calvia_quatuordecimguttata_217 | Tytthaspis_sedecimpunctata_588 | 0.155 |
| Coleomegilla_maculata_329 | Tytthaspis_sedecimpunctata_588 | 0.155 |
| Coleomegilla_maculata_330 | Tytthaspis_sedecimpunctata_588 | 0.155 |
| Coleomegilla_maculata_331 | Tytthaspis_sedecimpunctata_588 | 0.155 |
| Coleomegilla_maculata_333 | Tytthaspis_sedecimpunctata_588 | 0.155 |
| Coleomegilla_maculata_334 | Tytthaspis_sedecimpunctata_588 | 0.155 |
| Coleomegilla_maculata_335 | Tytthaspis_sedecimpunctata_588 | 0.155 |
| Coleomegilla_maculata_336 | Tytthaspis_sedecimpunctata_588 | 0.155 |
| Coleomegilla_maculata_337 | Tytthaspis_sedecimpunctata_588 | 0.155 |
| Coleomegilla_maculata_338 | Tytthaspis_sedecimpunctata_588 | 0.155 |
| Coleomegilla_maculata_339 | Tytthaspis_sedecimpunctata_588 | 0.155 |
| Calvia_quatuordecimguttata_207 | Tytthaspis_sedecimpunctata_593 | 0.155 |
| Scymnus_haemorrhoidalis_753 | Scymnus_suturalis_843 | 0.155 |
| Scymnus_haemorrhoidalis_753 | Scymnus_suturalis_845 | 0.155 |
| Calvia_quatuordecimguttata_203 | Tytthaspis_sedecimpunctata_577 | 0.155 |
| Calvia_quatuordecimguttata_203 | Tytthaspis_sedecimpunctata_578 | 0.155 |
| Calvia_quatuordecimguttata_203 | Tytthaspis_sedecimpunctata_579 | 0.155 |
| Calvia_quatuordecimguttata_203 | Tytthaspis_sedecimpunctata_581 | 0.155 |
| Calvia_quatuordecimguttata_203 | Tytthaspis_sedecimpunctata_582 | 0.155 |
| Coccinula_quatuordecimpustulata_305 | Tytthaspis_sedecimpunctata_583 | 0.155 |
| Coleomegilla_maculata_327 | Tytthaspis_sedecimpunctata_583 | 0.155 |
| Coleomegilla_maculata_329 | Tytthaspis_sedecimpunctata_585 | 0.155 |
| Coleomegilla_maculata_330 | Tytthaspis_sedecimpunctata_585 | 0.155 |
| Coleomegilla_maculata_331 | Tytthaspis_sedecimpunctata_585 | 0.155 |
| Coleomegilla_maculata_333 | Tytthaspis_sedecimpunctata_585 | 0.155 |
| Coleomegilla_maculata_334 | Tytthaspis_sedecimpunctata_585 | 0.155 |

| | | |
|---|---|---|
| Coleomegilla_maculata_335 | Tytthaspis_sedecimpunctata_585 | 0.155 |
| Coleomegilla_maculata_336 | Tytthaspis_sedecimpunctata_585 | 0.155 |
| Coleomegilla_maculata_337 | Tytthaspis_sedecimpunctata_585 | 0.155 |
| Coleomegilla_maculata_338 | Tytthaspis_sedecimpunctata_585 | 0.155 |
| Coleomegilla_maculata_339 | Tytthaspis_sedecimpunctata_585 | 0.155 |
| Coleomegilla_maculata_328 | Tytthaspis_sedecimpunctata_589 | 0.155 |
| Coleomegilla_maculata_332 | Tytthaspis_sedecimpunctata_589 | 0.155 |
| Calvia_quatuordecimguttata_203 | Tytthaspis_sedecimpunctata_590 | 0.155 |
| Calvia_quatuordecimguttata_203 | Tytthaspis_sedecimpunctata_592 | 0.155 |
| Calvia_quatuordecimguttata_203 | Tytthaspis_sedecimpunctata_593 | 0.155 |
| Coleomegilla_maculata_329 | Tytthaspis_sedecimpunctata_593 | 0.155 |
| Coleomegilla_maculata_330 | Tytthaspis_sedecimpunctata_593 | 0.155 |
| Coleomegilla_maculata_331 | Tytthaspis_sedecimpunctata_593 | 0.155 |
| Coleomegilla_maculata_333 | Tytthaspis_sedecimpunctata_593 | 0.155 |
| Coleomegilla_maculata_334 | Tytthaspis_sedecimpunctata_593 | 0.155 |
| Coleomegilla_maculata_335 | Tytthaspis_sedecimpunctata_593 | 0.155 |
| Coleomegilla_maculata_336 | Tytthaspis_sedecimpunctata_593 | 0.155 |
| Coleomegilla_maculata_337 | Tytthaspis_sedecimpunctata_593 | 0.155 |
| Coleomegilla_maculata_338 | Tytthaspis_sedecimpunctata_593 | 0.155 |
| Coleomegilla_maculata_339 | Tytthaspis_sedecimpunctata_593 | 0.155 |
| Calvia_quatuordecimguttata_203 | Tytthaspis_sedecimpunctata_594 | 0.155 |
| Calvia_quatuordecimguttata_203 | Tytthaspis_sedecimpunctata_595 | 0.155 |
| Scymnus_nigrinus_784 | Scymnus_suturalis_838 | 0.155 |
| Scymnus_interruptus_760 | Scymnus_sinuanodulus_831 | 0.155 |
| Scymnus_interruptus_765 | Scymnus_sinuanodulus_831 | 0.155 |
| Scymnus_interruptus_768 | Scymnus_sinuanodulus_831 | 0.155 |
| Psyllobora_vigintiduopunctata_558 | Tytthaspis_sedecimpunctata_588 | 0.155 |
| Psyllobora_vigintiduopunctata_568 | Tytthaspis_sedecimpunctata_588 | 0.155 |
| Scymnus_nigrinus_784 | Scymnus_suturalis_841 | 0.155 |
| Scymnus_nigrinus_784 | Scymnus_suturalis_842 | 0.155 |
| Scymnus_nigrinus_785 | Scymnus_suturalis_843 | 0.155 |
| Scymnus_nigrinus_786 | Scymnus_suturalis_843 | 0.155 |
| Scymnus_nigrinus_784 | Scymnus_suturalis_844 | 0.155 |
| Scymnus_nigrinus_785 | Scymnus_suturalis_845 | 0.155 |
| Scymnus_nigrinus_786 | Scymnus_suturalis_845 | 0.155 |
| Scymnus_nigrinus_784 | Scymnus_suturalis_846 | 0.155 |
| Scymnus_nigrinus_784 | Scymnus_suturalis_847 | 0.155 |
| Scymnus_nigrinus_784 | Scymnus_suturalis_848 | 0.155 |
| Coccinula_quatuordecimpustulata_305 | Tytthaspis_sedecimpunctata_580 | 0.155 |
| Calvia_quatuordecimguttata_205 | Tytthaspis_sedecimpunctata_583 | 0.155 |
| Calvia_quatuordecimguttata_205 | Tytthaspis_sedecimpunctata_585 | 0.155 |
| Coleomegilla_maculata_328 | Tytthaspis_sedecimpunctata_591 | 0.155 |
| Coleomegilla_maculata_332 | Tytthaspis_sedecimpunctata_591 | 0.155 |
| Scymnus_nigrinus_784 | Scymnus_suturalis_832 | 0.155 |
| Scymnus_nigrinus_784 | Scymnus_suturalis_835 | 0.155 |
| Scymnus_nigrinus_784 | Scymnus_suturalis_836 | 0.155 |
| Scymnus_nigrinus_785 | Scymnus_suturalis_837 | 0.155 |
| Scymnus_nigrinus_786 | Scymnus_suturalis_837 | 0.155 |
| Scymnus_nigrinus_784 | Scymnus_suturalis_839 | 0.155 |
| Scymnus_loewii_770 | Scymnus_rubromaculatus_822 | 0.156 |
| Scymnus_loewii_770 | Scymnus_rubromaculatus_823 | 0.156 |
| Scymnus_loewii_770 | Scymnus_rubromaculatus_824 | 0.156 |
| Scymnus_loewii_770 | Scymnus_rubromaculatus_825 | 0.156 |
| Scymnus_loewii_770 | Scymnus_rubromaculatus_826 | 0.156 |
| Scymnus_loewii_770 | Scymnus_rubromaculatus_827 | 0.156 |
| Macronaemia_episcopalis_478 | Tytthaspis_sedecimpunctata_586 | 0.156 |

Supplementary Table S4 (continued)

| | | |
|---|---|---|
| Macronaemia_episcopalis_478 | Tytthaspis_sedecimpunctata_588 | 0.156 |
| Hippodamia_parenthesis_436 | Tytthaspis_sedecimpunctata_580 | 0.156 |
| Scymnus_nigrinus_787 | Scymnus_suturalis_833 | 0.156 |
| Scymnus_nigrinus_787 | Scymnus_suturalis_840 | 0.156 |
| Scymnus_nigrinus_787 | Scymnus_suturalis_834 | 0.156 |
| Scymnus_nebulosus_783 | Scymnus_rubromaculatus_822 | 0.156 |
| Scymnus_nebulosus_783 | Scymnus_rubromaculatus_823 | 0.156 |
| Scymnus_nebulosus_783 | Scymnus_rubromaculatus_824 | 0.156 |
| Scymnus_nebulosus_783 | Scymnus_rubromaculatus_825 | 0.156 |
| Scymnus_nebulosus_783 | Scymnus_rubromaculatus_826 | 0.156 |
| Scymnus_nebulosus_783 | Scymnus_rubromaculatus_827 | 0.156 |
| Anisosticta_novemdecimpunctata_162 | Tytthaspis_sedecimpunctata_589 | 0.156 |
| Anisosticta_novemdecimpunctata_164 | Tytthaspis_sedecimpunctata_589 | 0.156 |
| Anisosticta_novemdecimpunctata_166 | Tytthaspis_sedecimpunctata_589 | 0.156 |
| Anisosticta_novemdecimpunctata_167 | Tytthaspis_sedecimpunctata_589 | 0.156 |
| Anisosticta_novemdecimpunctata_168 | Tytthaspis_sedecimpunctata_589 | 0.156 |
| Scymnus_nebulosus_782 | Scymnus_sinuanodulus_828 | 0.156 |
| Anisosticta_novemdecimpunctata_162 | Tytthaspis_sedecimpunctata_584 | 0.156 |
| Anisosticta_novemdecimpunctata_164 | Tytthaspis_sedecimpunctata_584 | 0.156 |
| Anisosticta_novemdecimpunctata_166 | Tytthaspis_sedecimpunctata_584 | 0.156 |
| Anisosticta_novemdecimpunctata_167 | Tytthaspis_sedecimpunctata_584 | 0.156 |
| Anisosticta_novemdecimpunctata_168 | Tytthaspis_sedecimpunctata_584 | 0.156 |
| Anisosticta_novemdecimpunctata_162 | Tytthaspis_sedecimpunctata_587 | 0.156 |
| Anisosticta_novemdecimpunctata_164 | Tytthaspis_sedecimpunctata_587 | 0.156 |
| Anisosticta_novemdecimpunctata_166 | Tytthaspis_sedecimpunctata_587 | 0.156 |
| Anisosticta_novemdecimpunctata_167 | Tytthaspis_sedecimpunctata_587 | 0.156 |
| Anisosticta_novemdecimpunctata_168 | Tytthaspis_sedecimpunctata_587 | 0.156 |
| Anisosticta_novemdecimpunctata_162 | Tytthaspis_sedecimpunctata_583 | 0.156 |
| Anisosticta_novemdecimpunctata_164 | Tytthaspis_sedecimpunctata_583 | 0.156 |
| Anisosticta_novemdecimpunctata_166 | Tytthaspis_sedecimpunctata_583 | 0.156 |
| Anisosticta_novemdecimpunctata_167 | Tytthaspis_sedecimpunctata_583 | 0.156 |
| Anisosticta_novemdecimpunctata_168 | Tytthaspis_sedecimpunctata_583 | 0.156 |
| Anisosticta_novemdecimpunctata_170 | Tytthaspis_sedecimpunctata_586 | 0.156 |
| Anisosticta_novemdecimpunctata_170 | Tytthaspis_sedecimpunctata_588 | 0.156 |
| Scymnus_camptodromus_706 | Scymnus_rubromaculatus_822 | 0.156 |
| Scymnus_camptodromus_706 | Scymnus_rubromaculatus_823 | 0.156 |
| Scymnus_camptodromus_706 | Scymnus_rubromaculatus_824 | 0.156 |
| Scymnus_camptodromus_706 | Scymnus_rubromaculatus_825 | 0.156 |
| Scymnus_camptodromus_706 | Scymnus_rubromaculatus_826 | 0.156 |
| Scymnus_camptodromus_706 | Scymnus_rubromaculatus_827 | 0.156 |
| Anisosticta_novemdecimpunctata_170 | Tytthaspis_sedecimpunctata_585 | 0.156 |
| Anisosticta_novemdecimpunctata_170 | Tytthaspis_sedecimpunctata_592 | 0.156 |
| Halyzia_sedecimguttata_346 | Vibidia_duodecimguttata_599 | 0.156 |
| Halyzia_sedecimguttata_347 | Vibidia_duodecimguttata_599 | 0.156 |
| Halyzia_sedecimguttata_348 | Vibidia_duodecimguttata_599 | 0.156 |
| Halyzia_sedecimguttata_346 | Vibidia_duodecimguttata_602 | 0.156 |
| Halyzia_sedecimguttata_347 | Vibidia_duodecimguttata_602 | 0.156 |
| Halyzia_sedecimguttata_348 | Vibidia_duodecimguttata_602 | 0.156 |
| Halyzia_sedecimguttata_352 | Vibidia_duodecimguttata_597 | 0.156 |
| Halyzia_sedecimguttata_352 | Vibidia_duodecimguttata_600 | 0.156 |
| Harmonia_conformis_365 | Tytthaspis_sedecimpunctata_591 | 0.157 |
| Harmonia_conformis_366 | Tytthaspis_sedecimpunctata_591 | 0.157 |
| Harmonia_conformis_367 | Tytthaspis_sedecimpunctata_591 | 0.157 |
| Harmonia_conformis_368 | Tytthaspis_sedecimpunctata_591 | 0.157 |
| Harmonia_conformis_369 | Tytthaspis_sedecimpunctata_591 | 0.157 |
| Harmonia_conformis_370 | Tytthaspis_sedecimpunctata_591 | 0.157 |

| | | |
|---|---|---|
| Harmonia_conformis_371 | Tytthaspis_sedecimpunctata_591 | 0.157 |
| Harmonia_conformis_372 | Tytthaspis_sedecimpunctata_591 | 0.157 |
| Harmonia_conformis_373 | Tytthaspis_sedecimpunctata_591 | 0.157 |
| Harmonia_conformis_375 | Tytthaspis_sedecimpunctata_591 | 0.157 |
| Halyzia_sedecimguttata_345 | Vibidia_duodecimguttata_599 | 0.157 |
| Halyzia_sedecimguttata_349 | Vibidia_duodecimguttata_599 | 0.157 |
| Halyzia_sedecimguttata_350 | Vibidia_duodecimguttata_599 | 0.157 |
| Halyzia_sedecimguttata_351 | Vibidia_duodecimguttata_601 | 0.157 |
| Halyzia_sedecimguttata_353 | Vibidia_duodecimguttata_601 | 0.157 |
| Halyzia_sedecimguttata_345 | Vibidia_duodecimguttata_602 | 0.157 |
| Halyzia_sedecimguttata_349 | Vibidia_duodecimguttata_602 | 0.157 |
| Halyzia_sedecimguttata_350 | Vibidia_duodecimguttata_602 | 0.157 |
| Halyzia_sedecimguttata_352 | Vibidia_duodecimguttata_603 | 0.157 |
| Harmonia_conformis_365 | Tytthaspis_sedecimpunctata_589 | 0.157 |
| Harmonia_conformis_366 | Tytthaspis_sedecimpunctata_589 | 0.157 |
| Harmonia_conformis_367 | Tytthaspis_sedecimpunctata_589 | 0.157 |
| Harmonia_conformis_368 | Tytthaspis_sedecimpunctata_589 | 0.157 |
| Harmonia_conformis_369 | Tytthaspis_sedecimpunctata_589 | 0.157 |
| Harmonia_conformis_370 | Tytthaspis_sedecimpunctata_589 | 0.157 |
| Harmonia_conformis_371 | Tytthaspis_sedecimpunctata_589 | 0.157 |
| Harmonia_conformis_372 | Tytthaspis_sedecimpunctata_589 | 0.157 |
| Harmonia_conformis_373 | Tytthaspis_sedecimpunctata_589 | 0.157 |
| Harmonia_conformis_375 | Tytthaspis_sedecimpunctata_589 | 0.157 |
| Harmonia_conformis_365 | Tytthaspis_sedecimpunctata_587 | 0.157 |
| Harmonia_conformis_366 | Tytthaspis_sedecimpunctata_587 | 0.157 |
| Harmonia_conformis_367 | Tytthaspis_sedecimpunctata_587 | 0.157 |
| Harmonia_conformis_368 | Tytthaspis_sedecimpunctata_587 | 0.157 |
| Harmonia_conformis_369 | Tytthaspis_sedecimpunctata_587 | 0.157 |
| Harmonia_conformis_370 | Tytthaspis_sedecimpunctata_587 | 0.157 |
| Harmonia_conformis_371 | Tytthaspis_sedecimpunctata_587 | 0.157 |
| Harmonia_conformis_372 | Tytthaspis_sedecimpunctata_587 | 0.157 |
| Harmonia_conformis_373 | Tytthaspis_sedecimpunctata_587 | 0.157 |
| Harmonia_conformis_375 | Tytthaspis_sedecimpunctata_587 | 0.157 |
| Mulsantina_hudsonica_483 | Tytthaspis_sedecimpunctata_584 | 0.157 |
| Anatis_mali_128 | Tytthaspis_sedecimpunctata_585 | 0.157 |
| Anatis_mali_129 | Tytthaspis_sedecimpunctata_585 | 0.157 |
| Anatis_mali_130 | Tytthaspis_sedecimpunctata_585 | 0.157 |
| Anatis_mali_131 | Tytthaspis_sedecimpunctata_585 | 0.157 |
| Mulsantina_hudsonica_481 | Tytthaspis_sedecimpunctata_591 | 0.157 |
| Mulsantina_hudsonica_482 | Tytthaspis_sedecimpunctata_591 | 0.157 |
| Anatis_ocellata_132 | Tytthaspis_sedecimpunctata_593 | 0.157 |
| Anatis_ocellata_134 | Tytthaspis_sedecimpunctata_593 | 0.157 |
| Anatis_ocellata_135 | Tytthaspis_sedecimpunctata_593 | 0.157 |
| Anatis_ocellata_138 | Tytthaspis_sedecimpunctata_593 | 0.157 |
| Anatis_ocellata_139 | Tytthaspis_sedecimpunctata_593 | 0.157 |
| Anatis_ocellata_140 | Tytthaspis_sedecimpunctata_593 | 0.157 |
| Anatis_ocellata_141 | Tytthaspis_sedecimpunctata_593 | 0.157 |
| Anatis_ocellata_142 | Tytthaspis_sedecimpunctata_593 | 0.157 |
| Anatis_ocellata_144 | Tytthaspis_sedecimpunctata_593 | 0.157 |
| Anatis_ocellata_145 | Tytthaspis_sedecimpunctata_593 | 0.157 |
| Anatis_ocellata_146 | Tytthaspis_sedecimpunctata_593 | 0.157 |
| Hyperaspis_oregona_658 | Subcoccinella_vigintiquatuorpunctata_630 | 0.157 |
| Hyperaspis_oregona_659 | Subcoccinella_vigintiquatuorpunctata_630 | 0.157 |
| Coelophora_inaequalis_310 | Tytthaspis_sedecimpunctata_591 | 0.157 |
| Coelophora_inaequalis_312 | Tytthaspis_sedecimpunctata_591 | 0.157 |
| Coelophora_inaequalis_314 | Tytthaspis_sedecimpunctata_591 | 0.157 |

| | | |
|---|---|---|
| Coelophora_inaequalis_323 | Tytthaspis_sedecimpunctata_591 | 0.157 |
| Coelophora_inaequalis_325 | Tytthaspis_sedecimpunctata_591 | 0.157 |
| Scymnus_haemorrhoidalis_756 | Tytthaspis_sedecimpunctata_577 | 0.157 |
| Scymnus_haemorrhoidalis_756 | Tytthaspis_sedecimpunctata_578 | 0.157 |
| Scymnus_haemorrhoidalis_756 | Tytthaspis_sedecimpunctata_579 | 0.157 |
| Scymnus_haemorrhoidalis_756 | Tytthaspis_sedecimpunctata_581 | 0.157 |
| Scymnus_haemorrhoidalis_756 | Tytthaspis_sedecimpunctata_582 | 0.157 |
| Scymnus_haemorrhoidalis_747 | Tytthaspis_sedecimpunctata_583 | 0.157 |
| Scymnus_haemorrhoidalis_748 | Tytthaspis_sedecimpunctata_583 | 0.157 |
| Scymnus_haemorrhoidalis_756 | Tytthaspis_sedecimpunctata_590 | 0.157 |
| Scymnus_haemorrhoidalis_756 | Tytthaspis_sedecimpunctata_592 | 0.157 |
| Scymnus_haemorrhoidalis_747 | Tytthaspis_sedecimpunctata_593 | 0.157 |
| Scymnus_haemorrhoidalis_748 | Tytthaspis_sedecimpunctata_593 | 0.157 |
| Scymnus_haemorrhoidalis_756 | Tytthaspis_sedecimpunctata_594 | 0.157 |
| Scymnus_haemorrhoidalis_756 | Tytthaspis_sedecimpunctata_595 | 0.157 |
| Scymnus_haemorrhoidalis_756 | Tytthaspis_sedecimpunctata_580 | 0.157 |
| Myrrha_octodecimguttata_499 | Tytthaspis_sedecimpunctata_591 | 0.157 |
| Scymnus_interruptus_760 | Scymnus_sinuanodulus_830 | 0.157 |
| Scymnus_interruptus_765 | Scymnus_sinuanodulus_830 | 0.157 |
| Scymnus_interruptus_768 | Scymnus_sinuanodulus_830 | 0.157 |
| Myrrha_octodecimguttata_489 | Tytthaspis_sedecimpunctata_577 | 0.157 |
| Myrrha_octodecimguttata_500 | Tytthaspis_sedecimpunctata_577 | 0.157 |
| Myrrha_octodecimguttata_489 | Tytthaspis_sedecimpunctata_578 | 0.157 |
| Myrrha_octodecimguttata_500 | Tytthaspis_sedecimpunctata_578 | 0.157 |
| Myrrha_octodecimguttata_489 | Tytthaspis_sedecimpunctata_579 | 0.157 |
| Myrrha_octodecimguttata_500 | Tytthaspis_sedecimpunctata_579 | 0.157 |
| Myrrha_octodecimguttata_489 | Tytthaspis_sedecimpunctata_581 | 0.157 |
| Myrrha_octodecimguttata_500 | Tytthaspis_sedecimpunctata_581 | 0.157 |
| Myrrha_octodecimguttata_489 | Tytthaspis_sedecimpunctata_582 | 0.157 |
| Myrrha_octodecimguttata_500 | Tytthaspis_sedecimpunctata_582 | 0.157 |
| Myrrha_octodecimguttata_489 | Tytthaspis_sedecimpunctata_590 | 0.157 |
| Myrrha_octodecimguttata_500 | Tytthaspis_sedecimpunctata_590 | 0.157 |
| Myrrha_octodecimguttata_488 | Tytthaspis_sedecimpunctata_593 | 0.157 |
| Myrrha_octodecimguttata_491 | Tytthaspis_sedecimpunctata_593 | 0.157 |
| Myrrha_octodecimguttata_492 | Tytthaspis_sedecimpunctata_593 | 0.157 |
| Myrrha_octodecimguttata_489 | Tytthaspis_sedecimpunctata_594 | 0.157 |
| Myrrha_octodecimguttata_500 | Tytthaspis_sedecimpunctata_594 | 0.157 |
| Myrrha_octodecimguttata_489 | Tytthaspis_sedecimpunctata_595 | 0.157 |
| Myrrha_octodecimguttata_500 | Tytthaspis_sedecimpunctata_595 | 0.157 |
| Myrrha_octodecimguttata_489 | Tytthaspis_sedecimpunctata_580 | 0.157 |
| Myrrha_octodecimguttata_500 | Tytthaspis_sedecimpunctata_580 | 0.157 |
| Oenopia_conglobata_522 | Tytthaspis_sedecimpunctata_583 | 0.157 |
| Calvia_decemguttata_195 | Tytthaspis_sedecimpunctata_589 | 0.157 |
| Calvia_decemguttata_199 | Tytthaspis_sedecimpunctata_589 | 0.157 |
| Calvia_decemguttata_200 | Tytthaspis_sedecimpunctata_589 | 0.157 |
| Calvia_quatuordecimguttata_206 | Tytthaspis_sedecimpunctata_589 | 0.157 |
| Calvia_quatuordecimguttata_209 | Tytthaspis_sedecimpunctata_589 | 0.157 |
| Calvia_quatuordecimguttata_216 | Tytthaspis_sedecimpunctata_589 | 0.157 |
| Myrrha_octodecimguttata_493 | Tytthaspis_sedecimpunctata_591 | 0.157 |
| Myrrha_octodecimguttata_495 | Tytthaspis_sedecimpunctata_591 | 0.157 |
| Myrrha_octodecimguttata_498 | Tytthaspis_sedecimpunctata_591 | 0.157 |
| Oenopia_conglobata_522 | Tytthaspis_sedecimpunctata_592 | 0.157 |
| Scymnus_rubromaculatus_814 | Scymnus_suturalis_833 | 0.157 |
| Scymnus_rubromaculatus_814 | Scymnus_suturalis_838 | 0.157 |
| Scymnus_rubromaculatus_814 | Scymnus_suturalis_840 | 0.157 |
| Scymnus_haemorrhoidalis_747 | Scymnus_suturalis_843 | 0.157 |

| | | |
|---|---|---|
| Scymnus_haemorrhoidalis_748 | Scymnus_suturalis_843 | 0.157 |
| Scymnus_haemorrhoidalis_747 | Scymnus_suturalis_845 | 0.157 |
| Scymnus_haemorrhoidalis_748 | Scymnus_suturalis_845 | 0.157 |
| Calvia_decemguttata_195 | Tytthaspis_sedecimpunctata_586 | 0.157 |
| Calvia_decemguttata_199 | Tytthaspis_sedecimpunctata_586 | 0.157 |
| Calvia_decemguttata_200 | Tytthaspis_sedecimpunctata_586 | 0.157 |
| Calvia_quatuordecimguttata_218 | Tytthaspis_sedecimpunctata_586 | 0.157 |
| Calvia_quatuordecimguttata_206 | Tytthaspis_sedecimpunctata_588 | 0.157 |
| Calvia_quatuordecimguttata_216 | Tytthaspis_sedecimpunctata_588 | 0.157 |
| Macronaemia_episcopalis_477 | Tytthaspis_sedecimpunctata_589 | 0.157 |
| Calvia_quatuordecimguttata_206 | Tytthaspis_sedecimpunctata_591 | 0.157 |
| Calvia_quatuordecimguttata_209 | Tytthaspis_sedecimpunctata_591 | 0.157 |
| Calvia_quatuordecimguttata_216 | Tytthaspis_sedecimpunctata_591 | 0.157 |
| Oenopia_conglobata_522 | Tytthaspis_sedecimpunctata_591 | 0.157 |
| Scymnus_camptodromus_705 | Scymnus_rubromaculatus_822 | 0.157 |
| Scymnus_camptodromus_705 | Scymnus_rubromaculatus_823 | 0.157 |
| Scymnus_camptodromus_705 | Scymnus_rubromaculatus_824 | 0.157 |
| Scymnus_camptodromus_705 | Scymnus_rubromaculatus_825 | 0.157 |
| Scymnus_camptodromus_705 | Scymnus_rubromaculatus_826 | 0.157 |
| Scymnus_camptodromus_705 | Scymnus_rubromaculatus_827 | 0.157 |
| Scymnus_rubromaculatus_814 | Scymnus_suturalis_834 | 0.157 |
| Coleomegilla_maculata_327 | Tytthaspis_sedecimpunctata_584 | 0.157 |
| Calvia_quatuordecimguttata_204 | Tytthaspis_sedecimpunctata_586 | 0.157 |
| Calvia_quatuordecimguttata_208 | Tytthaspis_sedecimpunctata_586 | 0.157 |
| Calvia_quatuordecimguttata_217 | Tytthaspis_sedecimpunctata_586 | 0.157 |
| Coleomegilla_maculata_327 | Tytthaspis_sedecimpunctata_586 | 0.157 |
| Macronaemia_episcopalis_477 | Tytthaspis_sedecimpunctata_586 | 0.157 |
| Coleomegilla_maculata_328 | Tytthaspis_sedecimpunctata_587 | 0.157 |
| Coleomegilla_maculata_332 | Tytthaspis_sedecimpunctata_587 | 0.157 |
| Coleomegilla_maculata_327 | Tytthaspis_sedecimpunctata_588 | 0.157 |
| Macronaemia_episcopalis_477 | Tytthaspis_sedecimpunctata_588 | 0.157 |
| Psyllobora_vigintiduopunctata_558 | Tytthaspis_sedecimpunctata_589 | 0.157 |
| Psyllobora_vigintiduopunctata_568 | Tytthaspis_sedecimpunctata_589 | 0.157 |
| Scymnus_haemorrhoidalis_753 | Scymnus_suturalis_841 | 0.157 |
| Scymnus_haemorrhoidalis_753 | Scymnus_suturalis_842 | 0.157 |
| Scymnus_haemorrhoidalis_752 | Scymnus_suturalis_843 | 0.157 |
| Scymnus_haemorrhoidalis_753 | Scymnus_suturalis_844 | 0.157 |
| Scymnus_haemorrhoidalis_752 | Scymnus_suturalis_845 | 0.157 |
| Scymnus_haemorrhoidalis_753 | Scymnus_suturalis_846 | 0.157 |
| Scymnus_haemorrhoidalis_753 | Scymnus_suturalis_847 | 0.157 |
| Scymnus_haemorrhoidalis_753 | Scymnus_suturalis_848 | 0.157 |
| Coccinula_quatuordecimpustulata_305 | Tytthaspis_sedecimpunctata_577 | 0.157 |
| Coccinula_quatuordecimpustulata_305 | Tytthaspis_sedecimpunctata_578 | 0.157 |
| Coccinula_quatuordecimpustulata_305 | Tytthaspis_sedecimpunctata_579 | 0.157 |
| Hippodamia_parenthesis_432 | Tytthaspis_sedecimpunctata_580 | 0.157 |
| Hippodamia_parenthesis_434 | Tytthaspis_sedecimpunctata_580 | 0.157 |
| Hippodamia_parenthesis_435 | Tytthaspis_sedecimpunctata_580 | 0.157 |
| Hippodamia_parenthesis_437 | Tytthaspis_sedecimpunctata_580 | 0.157 |
| Hippodamia_parenthesis_438 | Tytthaspis_sedecimpunctata_580 | 0.157 |
| Hippodamia_parenthesis_441 | Tytthaspis_sedecimpunctata_580 | 0.157 |
| Hippodamia_parenthesis_442 | Tytthaspis_sedecimpunctata_580 | 0.157 |
| Coccinula_quatuordecimpustulata_305 | Tytthaspis_sedecimpunctata_581 | 0.157 |
| Coccinula_quatuordecimpustulata_305 | Tytthaspis_sedecimpunctata_582 | 0.157 |
| Calvia_quatuordecimguttata_203 | Tytthaspis_sedecimpunctata_583 | 0.157 |
| Coccinula_quatuordecimpustulata_292 | Tytthaspis_sedecimpunctata_583 | 0.157 |
| Coccinula_quatuordecimpustulata_293 | Tytthaspis_sedecimpunctata_583 | 0.157 |

Supplementary Table S4 (continued)

| | | |
|---|---|---|
| Coccinula_quatuordecimpustulata_307 | Tytthaspis_sedecimpunctata_583 | 0.157 |
| Macronaemia_episcopalis_477 | Tytthaspis_sedecimpunctata_583 | 0.157 |
| Calvia_quatuordecimguttata_203 | Tytthaspis_sedecimpunctata_585 | 0.157 |
| Coccinula_quatuordecimpustulata_305 | Tytthaspis_sedecimpunctata_585 | 0.157 |
| Coleomegilla_maculata_327 | Tytthaspis_sedecimpunctata_585 | 0.157 |
| Coleomegilla_maculata_329 | Tytthaspis_sedecimpunctata_589 | 0.157 |
| Coleomegilla_maculata_330 | Tytthaspis_sedecimpunctata_589 | 0.157 |
| Coleomegilla_maculata_331 | Tytthaspis_sedecimpunctata_589 | 0.157 |
| Coleomegilla_maculata_333 | Tytthaspis_sedecimpunctata_589 | 0.157 |
| Coleomegilla_maculata_334 | Tytthaspis_sedecimpunctata_589 | 0.157 |
| Coleomegilla_maculata_335 | Tytthaspis_sedecimpunctata_589 | 0.157 |
| Coleomegilla_maculata_336 | Tytthaspis_sedecimpunctata_589 | 0.157 |
| Coleomegilla_maculata_337 | Tytthaspis_sedecimpunctata_589 | 0.157 |
| Coleomegilla_maculata_338 | Tytthaspis_sedecimpunctata_589 | 0.157 |
| Coleomegilla_maculata_339 | Tytthaspis_sedecimpunctata_589 | 0.157 |
| Coccinula_quatuordecimpustulata_305 | Tytthaspis_sedecimpunctata_590 | 0.157 |
| Coccinula_quatuordecimpustulata_305 | Tytthaspis_sedecimpunctata_592 | 0.157 |
| Macronaemia_episcopalis_477 | Tytthaspis_sedecimpunctata_592 | 0.157 |
| Coccinula_quatuordecimpustulata_305 | Tytthaspis_sedecimpunctata_594 | 0.157 |
| Coccinula_quatuordecimpustulata_305 | Tytthaspis_sedecimpunctata_595 | 0.157 |
| Scymnus_haemorrhoidalis_753 | Scymnus_suturalis_832 | 0.157 |
| Scymnus_nigrinus_784 | Scymnus_suturalis_833 | 0.157 |
| Scymnus_haemorrhoidalis_753 | Scymnus_suturalis_835 | 0.157 |
| Scymnus_haemorrhoidalis_753 | Scymnus_suturalis_836 | 0.157 |
| Scymnus_haemorrhoidalis_753 | Scymnus_suturalis_837 | 0.157 |
| Scymnus_nigrinus_785 | Scymnus_suturalis_838 | 0.157 |
| Scymnus_nigrinus_786 | Scymnus_suturalis_838 | 0.157 |
| Scymnus_haemorrhoidalis_753 | Scymnus_suturalis_839 | 0.157 |
| Scymnus_nigrinus_784 | Scymnus_suturalis_840 | 0.157 |
| Psyllobora_vigintiduopunctata_558 | Tytthaspis_sedecimpunctata_586 | 0.157 |
| Psyllobora_vigintiduopunctata_568 | Tytthaspis_sedecimpunctata_586 | 0.157 |
| Scymnus_nigrinus_785 | Scymnus_suturalis_841 | 0.157 |
| Scymnus_nigrinus_786 | Scymnus_suturalis_841 | 0.157 |
| Scymnus_nigrinus_785 | Scymnus_suturalis_842 | 0.157 |
| Scymnus_nigrinus_786 | Scymnus_suturalis_842 | 0.157 |
| Scymnus_nigrinus_785 | Scymnus_suturalis_844 | 0.157 |
| Scymnus_nigrinus_786 | Scymnus_suturalis_844 | 0.157 |
| Scymnus_nigrinus_785 | Scymnus_suturalis_846 | 0.157 |
| Scymnus_nigrinus_786 | Scymnus_suturalis_846 | 0.157 |
| Scymnus_nigrinus_785 | Scymnus_suturalis_847 | 0.157 |
| Scymnus_nigrinus_786 | Scymnus_suturalis_847 | 0.157 |
| Scymnus_nigrinus_785 | Scymnus_suturalis_848 | 0.157 |
| Scymnus_nigrinus_786 | Scymnus_suturalis_848 | 0.157 |
| Coccinula_quatuordecimpustulata_292 | Tytthaspis_sedecimpunctata_580 | 0.157 |
| Coccinula_quatuordecimpustulata_293 | Tytthaspis_sedecimpunctata_580 | 0.157 |
| Coccinula_quatuordecimpustulata_307 | Tytthaspis_sedecimpunctata_580 | 0.157 |
| Coccinula_quatuordecimpustulata_305 | Tytthaspis_sedecimpunctata_584 | 0.157 |
| Coleomegilla_maculata_329 | Tytthaspis_sedecimpunctata_591 | 0.157 |
| Coleomegilla_maculata_330 | Tytthaspis_sedecimpunctata_591 | 0.157 |
| Coleomegilla_maculata_331 | Tytthaspis_sedecimpunctata_591 | 0.157 |
| Coleomegilla_maculata_333 | Tytthaspis_sedecimpunctata_591 | 0.157 |
| Coleomegilla_maculata_334 | Tytthaspis_sedecimpunctata_591 | 0.157 |
| Coleomegilla_maculata_335 | Tytthaspis_sedecimpunctata_591 | 0.157 |
| Coleomegilla_maculata_336 | Tytthaspis_sedecimpunctata_591 | 0.157 |
| Coleomegilla_maculata_337 | Tytthaspis_sedecimpunctata_591 | 0.157 |
| Coleomegilla_maculata_338 | Tytthaspis_sedecimpunctata_591 | 0.157 |

| | | |
|---|---|---|
| Coleomegilla_maculata_339 | Tytthaspis_sedecimpunctata_591 | 0.157 |
| Scymnus_loewii_774 | Scymnus_rubromaculatus_822 | 0.157 |
| Scymnus_loewii_774 | Scymnus_rubromaculatus_823 | 0.157 |
| Scymnus_loewii_774 | Scymnus_rubromaculatus_824 | 0.157 |
| Scymnus_loewii_774 | Scymnus_rubromaculatus_825 | 0.157 |
| Scymnus_loewii_774 | Scymnus_rubromaculatus_826 | 0.157 |
| Scymnus_loewii_774 | Scymnus_rubromaculatus_827 | 0.157 |
| Scymnus_nigrinus_785 | Scymnus_suturalis_832 | 0.157 |
| Scymnus_nigrinus_786 | Scymnus_suturalis_832 | 0.157 |
| Scymnus_nigrinus_784 | Scymnus_suturalis_834 | 0.157 |
| Scymnus_nigrinus_785 | Scymnus_suturalis_835 | 0.157 |
| Scymnus_nigrinus_786 | Scymnus_suturalis_835 | 0.157 |
| Scymnus_nigrinus_785 | Scymnus_suturalis_836 | 0.157 |
| Scymnus_nigrinus_786 | Scymnus_suturalis_836 | 0.157 |
| Scymnus_nigrinus_785 | Scymnus_suturalis_839 | 0.157 |
| Scymnus_nigrinus_786 | Scymnus_suturalis_839 | 0.157 |
| Scymnus_haemorrhoidalis_753 | Scymnus_suturalis_840 | 0.157 |
| Scymnus_loewii_771 | Scymnus_rubromaculatus_822 | 0.157 |
| Scymnus_mitior_779 | Scymnus_rubromaculatus_822 | 0.157 |
| Scymnus_loewii_771 | Scymnus_rubromaculatus_823 | 0.157 |
| Scymnus_mitior_779 | Scymnus_rubromaculatus_823 | 0.157 |
| Scymnus_loewii_771 | Scymnus_rubromaculatus_824 | 0.157 |
| Scymnus_mitior_779 | Scymnus_rubromaculatus_824 | 0.157 |
| Scymnus_loewii_771 | Scymnus_rubromaculatus_825 | 0.157 |
| Scymnus_mitior_779 | Scymnus_rubromaculatus_825 | 0.157 |
| Scymnus_loewii_771 | Scymnus_rubromaculatus_826 | 0.157 |
| Scymnus_mitior_779 | Scymnus_rubromaculatus_826 | 0.157 |
| Scymnus_loewii_771 | Scymnus_rubromaculatus_827 | 0.157 |
| Scymnus_mitior_779 | Scymnus_rubromaculatus_827 | 0.157 |
| Hippodamia_parenthesis_436 | Tytthaspis_sedecimpunctata_577 | 0.157 |
| Hippodamia_parenthesis_436 | Tytthaspis_sedecimpunctata_578 | 0.157 |
| Hippodamia_parenthesis_436 | Tytthaspis_sedecimpunctata_579 | 0.157 |
| Hippodamia_parenthesis_436 | Tytthaspis_sedecimpunctata_581 | 0.157 |
| Hippodamia_parenthesis_436 | Tytthaspis_sedecimpunctata_582 | 0.157 |
| Hippodamia_parenthesis_436 | Tytthaspis_sedecimpunctata_590 | 0.157 |
| Hippodamia_parenthesis_436 | Tytthaspis_sedecimpunctata_592 | 0.157 |
| Hippodamia_parenthesis_436 | Tytthaspis_sedecimpunctata_594 | 0.157 |
| Hippodamia_parenthesis_436 | Tytthaspis_sedecimpunctata_595 | 0.157 |
| Macronaemia_episcopalis_478 | Tytthaspis_sedecimpunctata_593 | 0.158 |
| Scymnus_frontalis_742 | Scymnus_rubromaculatus_822 | 0.158 |
| Scymnus_frontalis_742 | Scymnus_rubromaculatus_823 | 0.158 |
| Scymnus_frontalis_742 | Scymnus_rubromaculatus_824 | 0.158 |
| Scymnus_frontalis_742 | Scymnus_rubromaculatus_825 | 0.158 |
| Scymnus_frontalis_742 | Scymnus_rubromaculatus_826 | 0.158 |
| Scymnus_frontalis_742 | Scymnus_rubromaculatus_827 | 0.158 |
| Coelophora_inaequalis_319 | Tytthaspis_sedecimpunctata_591 | 0.158 |
| Scymnus_apetzi_695 | Scymnus_suturalis_843 | 0.158 |
| Scymnus_apetzi_695 | Scymnus_suturalis_845 | 0.158 |
| Scymnus_apetzi_695 | Scymnus_suturalis_837 | 0.158 |
| Scymnus_interruptus_767 | Scymnus_sinuanodulus_829 | 0.158 |
| Scymnus_frontalis_735 | Scymnus_rubromaculatus_822 | 0.158 |
| Scymnus_frontalis_735 | Scymnus_rubromaculatus_823 | 0.158 |
| Scymnus_frontalis_735 | Scymnus_rubromaculatus_824 | 0.158 |
| Scymnus_frontalis_735 | Scymnus_rubromaculatus_825 | 0.158 |
| Scymnus_frontalis_735 | Scymnus_rubromaculatus_826 | 0.158 |
| Scymnus_frontalis_735 | Scymnus_rubromaculatus_827 | 0.158 |

| | | |
|---|---|---|
| Scymnus_interruptus_762 | Scymnus_sinuanodulus_829 | 0.158 |
| Anisosticta_novemdecimpunctata_165 | Tytthaspis_sedecimpunctata_591 | 0.158 |
| Anisosticta_novemdecimpunctata_173 | Tytthaspis_sedecimpunctata_591 | 0.158 |
| Brumus_quadripustulatus_5 | Tytthaspis_sedecimpunctata_583 | 0.158 |
| Brumus_quadripustulatus_5 | Tytthaspis_sedecimpunctata_580 | 0.158 |
| Halyzia_sedecimguttata_346 | Vibidia_duodecimguttata_597 | 0.158 |
| Halyzia_sedecimguttata_347 | Vibidia_duodecimguttata_597 | 0.158 |
| Halyzia_sedecimguttata_348 | Vibidia_duodecimguttata_597 | 0.158 |
| Halyzia_sedecimguttata_346 | Vibidia_duodecimguttata_600 | 0.158 |
| Halyzia_sedecimguttata_347 | Vibidia_duodecimguttata_600 | 0.158 |
| Halyzia_sedecimguttata_348 | Vibidia_duodecimguttata_600 | 0.158 |
| Scymnus_nigrinus_787 | Scymnus_sinuanodulus_829 | 0.158 |
| Anisosticta_novemdecimpunctata_170 | Tytthaspis_sedecimpunctata_577 | 0.158 |
| Anisosticta_novemdecimpunctata_170 | Tytthaspis_sedecimpunctata_578 | 0.158 |
| Anisosticta_novemdecimpunctata_170 | Tytthaspis_sedecimpunctata_579 | 0.158 |
| Anisosticta_novemdecimpunctata_170 | Tytthaspis_sedecimpunctata_581 | 0.158 |
| Anisosticta_novemdecimpunctata_170 | Tytthaspis_sedecimpunctata_582 | 0.158 |
| Anisosticta_novemdecimpunctata_170 | Tytthaspis_sedecimpunctata_590 | 0.158 |
| Anisosticta_novemdecimpunctata_170 | Tytthaspis_sedecimpunctata_593 | 0.158 |
| Anisosticta_novemdecimpunctata_170 | Tytthaspis_sedecimpunctata_594 | 0.158 |
| Anisosticta_novemdecimpunctata_170 | Tytthaspis_sedecimpunctata_595 | 0.158 |
| Halyzia_sedecimguttata_346 | Vibidia_duodecimguttata_601 | 0.158 |
| Halyzia_sedecimguttata_347 | Vibidia_duodecimguttata_601 | 0.158 |
| Halyzia_sedecimguttata_348 | Vibidia_duodecimguttata_601 | 0.158 |
| Anisosticta_novemdecimpunctata_170 | Tytthaspis_sedecimpunctata_580 | 0.158 |
| Chilocorus_bipustulatus_18 | Tytthaspis_sedecimpunctata_583 | 0.159 |
| Chilocorus_bipustulatus_20 | Tytthaspis_sedecimpunctata_583 | 0.159 |
| Chilocorus_bipustulatus_18 | Tytthaspis_sedecimpunctata_592 | 0.159 |
| Chilocorus_bipustulatus_20 | Tytthaspis_sedecimpunctata_592 | 0.159 |
| Chilocorus_bipustulatus_18 | Tytthaspis_sedecimpunctata_580 | 0.159 |
| Chilocorus_bipustulatus_20 | Tytthaspis_sedecimpunctata_580 | 0.159 |
| Harmonia_conformis_374 | Tytthaspis_sedecimpunctata_591 | 0.159 |
| Harmonia_conformis_374 | Tytthaspis_sedecimpunctata_589 | 0.159 |
| Coccinella_transversoguttata_267 | Tytthaspis_sedecimpunctata_593 | 0.159 |
| Coccinella_transversoguttata_268 | Tytthaspis_sedecimpunctata_593 | 0.159 |
| Coccinella_transversoguttata_269 | Tytthaspis_sedecimpunctata_593 | 0.159 |
| Coccinella_transversoguttata_270 | Tytthaspis_sedecimpunctata_593 | 0.159 |
| Coccinella_transversoguttata_271 | Tytthaspis_sedecimpunctata_593 | 0.159 |
| Harmonia_conformis_374 | Tytthaspis_sedecimpunctata_587 | 0.159 |
| Mulsantina_hudsonica_480 | Tytthaspis_sedecimpunctata_584 | 0.159 |
| Mulsantina_hudsonica_483 | Tytthaspis_sedecimpunctata_591 | 0.159 |
| Halyzia_sedecimguttata_352 | Vibidia_duodecimguttata_599 | 0.159 |
| Halyzia_sedecimguttata_345 | Vibidia_duodecimguttata_601 | 0.159 |
| Halyzia_sedecimguttata_349 | Vibidia_duodecimguttata_601 | 0.159 |
| Halyzia_sedecimguttata_350 | Vibidia_duodecimguttata_601 | 0.159 |
| Halyzia_sedecimguttata_352 | Vibidia_duodecimguttata_602 | 0.159 |
| Anatis_ocellata_132 | Tytthaspis_sedecimpunctata_583 | 0.159 |
| Anatis_ocellata_134 | Tytthaspis_sedecimpunctata_583 | 0.159 |
| Anatis_ocellata_135 | Tytthaspis_sedecimpunctata_583 | 0.159 |
| Anatis_ocellata_138 | Tytthaspis_sedecimpunctata_583 | 0.159 |
| Anatis_ocellata_139 | Tytthaspis_sedecimpunctata_583 | 0.159 |
| Anatis_ocellata_140 | Tytthaspis_sedecimpunctata_583 | 0.159 |
| Anatis_ocellata_141 | Tytthaspis_sedecimpunctata_583 | 0.159 |
| Anatis_ocellata_142 | Tytthaspis_sedecimpunctata_583 | 0.159 |
| Anatis_ocellata_144 | Tytthaspis_sedecimpunctata_583 | 0.159 |
| Anatis_ocellata_145 | Tytthaspis_sedecimpunctata_583 | 0.159 |

| | | |
|---|---|---|
| Anatis_ocellata_146 | Tytthaspis_sedecimpunctata_583 | 0.159 |
| Anatis_ocellata_132 | Tytthaspis_sedecimpunctata_585 | 0.159 |
| Anatis_ocellata_134 | Tytthaspis_sedecimpunctata_585 | 0.159 |
| Anatis_ocellata_135 | Tytthaspis_sedecimpunctata_585 | 0.159 |
| Anatis_ocellata_138 | Tytthaspis_sedecimpunctata_585 | 0.159 |
| Anatis_ocellata_139 | Tytthaspis_sedecimpunctata_585 | 0.159 |
| Anatis_ocellata_140 | Tytthaspis_sedecimpunctata_585 | 0.159 |
| Anatis_ocellata_141 | Tytthaspis_sedecimpunctata_585 | 0.159 |
| Anatis_ocellata_142 | Tytthaspis_sedecimpunctata_585 | 0.159 |
| Anatis_ocellata_144 | Tytthaspis_sedecimpunctata_585 | 0.159 |
| Anatis_ocellata_145 | Tytthaspis_sedecimpunctata_585 | 0.159 |
| Anatis_ocellata_146 | Tytthaspis_sedecimpunctata_585 | 0.159 |
| Anatis_ocellata_132 | Tytthaspis_sedecimpunctata_592 | 0.159 |
| Anatis_ocellata_134 | Tytthaspis_sedecimpunctata_592 | 0.159 |
| Anatis_ocellata_135 | Tytthaspis_sedecimpunctata_592 | 0.159 |
| Anatis_ocellata_138 | Tytthaspis_sedecimpunctata_592 | 0.159 |
| Anatis_ocellata_139 | Tytthaspis_sedecimpunctata_592 | 0.159 |
| Anatis_ocellata_140 | Tytthaspis_sedecimpunctata_592 | 0.159 |
| Anatis_ocellata_141 | Tytthaspis_sedecimpunctata_592 | 0.159 |
| Anatis_ocellata_142 | Tytthaspis_sedecimpunctata_592 | 0.159 |
| Anatis_ocellata_144 | Tytthaspis_sedecimpunctata_592 | 0.159 |
| Anatis_ocellata_145 | Tytthaspis_sedecimpunctata_592 | 0.159 |
| Anatis_ocellata_146 | Tytthaspis_sedecimpunctata_592 | 0.159 |
| Scymnus_mimulus_776 | Scymnus_suturalis_843 | 0.159 |
| Scymnus_mimulus_776 | Scymnus_suturalis_845 | 0.159 |
| Anatis_ocellata_132 | Tytthaspis_sedecimpunctata_580 | 0.159 |
| Anatis_ocellata_134 | Tytthaspis_sedecimpunctata_580 | 0.159 |
| Anatis_ocellata_135 | Tytthaspis_sedecimpunctata_580 | 0.159 |
| Anatis_ocellata_138 | Tytthaspis_sedecimpunctata_580 | 0.159 |
| Anatis_ocellata_139 | Tytthaspis_sedecimpunctata_580 | 0.159 |
| Anatis_ocellata_140 | Tytthaspis_sedecimpunctata_580 | 0.159 |
| Anatis_ocellata_141 | Tytthaspis_sedecimpunctata_580 | 0.159 |
| Anatis_ocellata_142 | Tytthaspis_sedecimpunctata_580 | 0.159 |
| Anatis_ocellata_144 | Tytthaspis_sedecimpunctata_580 | 0.159 |
| Anatis_ocellata_145 | Tytthaspis_sedecimpunctata_580 | 0.159 |
| Anatis_ocellata_146 | Tytthaspis_sedecimpunctata_580 | 0.159 |
| Coelophora_inaequalis_320 | Tytthaspis_sedecimpunctata_586 | 0.159 |
| Coelophora_inaequalis_321 | Tytthaspis_sedecimpunctata_586 | 0.159 |
| Coelophora_inaequalis_322 | Tytthaspis_sedecimpunctata_586 | 0.159 |
| Psyllobora_vigintiduopunctata_557 | Tytthaspis_sedecimpunctata_588 | 0.159 |
| Psyllobora_vigintiduopunctata_561 | Tytthaspis_sedecimpunctata_588 | 0.159 |
| Psyllobora_vigintiduopunctata_562 | Tytthaspis_sedecimpunctata_588 | 0.159 |
| Psyllobora_vigintiduopunctata_563 | Tytthaspis_sedecimpunctata_588 | 0.159 |
| Psyllobora_vigintiduopunctata_565 | Tytthaspis_sedecimpunctata_588 | 0.159 |
| Psyllobora_vigintiduopunctata_567 | Tytthaspis_sedecimpunctata_588 | 0.159 |
| Scymnus_mimulus_776 | Scymnus_suturalis_837 | 0.159 |
| Hyperaspis_oregona_658 | Subcoccinella_vigintiquatuorpunctata_620 | 0.159 |
| Hyperaspis_oregona_659 | Subcoccinella_vigintiquatuorpunctata_620 | 0.159 |
| Hyperaspis_oregona_658 | Subcoccinella_vigintiquatuorpunctata_621 | 0.159 |
| Hyperaspis_oregona_659 | Subcoccinella_vigintiquatuorpunctata_621 | 0.159 |
| Hyperaspis_oregona_658 | Subcoccinella_vigintiquatuorpunctata_627 | 0.159 |
| Hyperaspis_oregona_659 | Subcoccinella_vigintiquatuorpunctata_627 | 0.159 |
| Hyperaspis_oregona_658 | Subcoccinella_vigintiquatuorpunctata_628 | 0.159 |
| Hyperaspis_oregona_659 | Subcoccinella_vigintiquatuorpunctata_628 | 0.159 |
| Hyperaspis_oregona_658 | Subcoccinella_vigintiquatuorpunctata_629 | 0.159 |
| Hyperaspis_oregona_659 | Subcoccinella_vigintiquatuorpunctata_629 | 0.159 |

| | | |
|---|---|---|
| Hyperaspis_oregona_658 | Subcoccinella_vigintiquatuorpunctata_631 | 0.159 |
| Hyperaspis_oregona_659 | Subcoccinella_vigintiquatuorpunctata_631 | 0.159 |
| Scymnus_haemorrhoidalis_747 | Tytthaspis_sedecimpunctata_584 | 0.159 |
| Scymnus_haemorrhoidalis_748 | Tytthaspis_sedecimpunctata_584 | 0.159 |
| Psyllobora_vigintiduopunctata_556 | Tytthaspis_sedecimpunctata_588 | 0.159 |
| Coelophora_inaequalis_311 | Tytthaspis_sedecimpunctata_591 | 0.159 |
| Coelophora_inaequalis_313 | Tytthaspis_sedecimpunctata_591 | 0.159 |
| Coelophora_inaequalis_316 | Tytthaspis_sedecimpunctata_591 | 0.159 |
| Coelophora_inaequalis_324 | Tytthaspis_sedecimpunctata_591 | 0.159 |
| Coelophora_inaequalis_326 | Tytthaspis_sedecimpunctata_591 | 0.159 |
| Scymnus_haemorrhoidalis_747 | Tytthaspis_sedecimpunctata_591 | 0.159 |
| Scymnus_haemorrhoidalis_748 | Tytthaspis_sedecimpunctata_591 | 0.159 |
| Scymnus_haemorrhoidalis_753 | Tytthaspis_sedecimpunctata_577 | 0.159 |
| Scymnus_haemorrhoidalis_753 | Tytthaspis_sedecimpunctata_578 | 0.159 |
| Scymnus_haemorrhoidalis_753 | Tytthaspis_sedecimpunctata_579 | 0.159 |
| Scymnus_haemorrhoidalis_753 | Tytthaspis_sedecimpunctata_581 | 0.159 |
| Scymnus_haemorrhoidalis_753 | Tytthaspis_sedecimpunctata_582 | 0.159 |
| Scymnus_haemorrhoidalis_756 | Tytthaspis_sedecimpunctata_583 | 0.159 |
| Scymnus_haemorrhoidalis_747 | Tytthaspis_sedecimpunctata_585 | 0.159 |
| Scymnus_haemorrhoidalis_748 | Tytthaspis_sedecimpunctata_585 | 0.159 |
| Scymnus_haemorrhoidalis_753 | Tytthaspis_sedecimpunctata_590 | 0.159 |
| Coelophora_inaequalis_315 | Tytthaspis_sedecimpunctata_591 | 0.159 |
| Scymnus_haemorrhoidalis_753 | Tytthaspis_sedecimpunctata_592 | 0.159 |
| Scymnus_haemorrhoidalis_756 | Tytthaspis_sedecimpunctata_593 | 0.159 |
| Scymnus_haemorrhoidalis_753 | Tytthaspis_sedecimpunctata_594 | 0.159 |
| Scymnus_haemorrhoidalis_753 | Tytthaspis_sedecimpunctata_595 | 0.159 |
| Scymnus_haemorrhoidalis_753 | Tytthaspis_sedecimpunctata_580 | 0.159 |
| Myrrha_octodecimguttata_488 | Tytthaspis_sedecimpunctata_584 | 0.159 |
| Myrrha_octodecimguttata_491 | Tytthaspis_sedecimpunctata_584 | 0.159 |
| Myrrha_octodecimguttata_492 | Tytthaspis_sedecimpunctata_584 | 0.159 |
| Hippodamia_tredecimpunctata_457 | Tytthaspis_sedecimpunctata_592 | 0.159 |
| Hippodamia_tredecimpunctata_458 | Tytthaspis_sedecimpunctata_592 | 0.159 |
| Ceratomegilla_ulkei_222 | Tytthaspis_sedecimpunctata_583 | 0.159 |
| Ceratomegilla_ulkei_223 | Tytthaspis_sedecimpunctata_583 | 0.159 |
| Myrrha_octodecimguttata_485 | Tytthaspis_sedecimpunctata_591 | 0.159 |
| Myrrha_octodecimguttata_486 | Tytthaspis_sedecimpunctata_591 | 0.159 |
| Myrrha_octodecimguttata_487 | Tytthaspis_sedecimpunctata_591 | 0.159 |
| Myrrha_octodecimguttata_490 | Tytthaspis_sedecimpunctata_591 | 0.159 |
| Myrrha_octodecimguttata_494 | Tytthaspis_sedecimpunctata_591 | 0.159 |
| Myrrha_octodecimguttata_496 | Tytthaspis_sedecimpunctata_591 | 0.159 |
| Myrrha_octodecimguttata_497 | Tytthaspis_sedecimpunctata_591 | 0.159 |
| Myrrha_octodecimguttata_501 | Tytthaspis_sedecimpunctata_591 | 0.159 |
| Myrrha_octodecimguttata_502 | Tytthaspis_sedecimpunctata_591 | 0.159 |
| Myrrha_octodecimguttata_489 | Tytthaspis_sedecimpunctata_593 | 0.159 |
| Myrrha_octodecimguttata_500 | Tytthaspis_sedecimpunctata_593 | 0.159 |
| Scymnus_apetzi_692 | Scymnus_suturalis_843 | 0.159 |
| Scymnus_apetzi_692 | Scymnus_suturalis_845 | 0.159 |
| Anatis_ocellata_136 | Tytthaspis_sedecimpunctata_593 | 0.159 |
| Scymnus_apetzi_692 | Scymnus_suturalis_837 | 0.159 |
| Psyllobora_vigintiduopunctata_569 | Tytthaspis_sedecimpunctata_588 | 0.159 |
| Oenopia_conglobata_522 | Tytthaspis_sedecimpunctata_577 | 0.159 |
| Oenopia_conglobata_522 | Tytthaspis_sedecimpunctata_578 | 0.159 |
| Oenopia_conglobata_522 | Tytthaspis_sedecimpunctata_579 | 0.159 |
| Oenopia_conglobata_522 | Tytthaspis_sedecimpunctata_581 | 0.159 |
| Oenopia_conglobata_522 | Tytthaspis_sedecimpunctata_582 | 0.159 |
| Calvia_quatuordecimguttata_207 | Tytthaspis_sedecimpunctata_589 | 0.159 |

| | | |
|---|---|---|
| Oenopia_conglobata_522 | Tytthaspis_sedecimpunctata_590 | 0.159 |
| Oenopia_conglobata_522 | Tytthaspis_sedecimpunctata_594 | 0.159 |
| Oenopia_conglobata_522 | Tytthaspis_sedecimpunctata_595 | 0.159 |
| Coelophora_inaequalis_318 | Tytthaspis_sedecimpunctata_591 | 0.159 |
| Scymnus_haemorrhoidalis_747 | Scymnus_suturalis_841 | 0.159 |
| Scymnus_haemorrhoidalis_748 | Scymnus_suturalis_841 | 0.159 |
| Scymnus_haemorrhoidalis_747 | Scymnus_suturalis_842 | 0.159 |
| Scymnus_haemorrhoidalis_748 | Scymnus_suturalis_842 | 0.159 |
| Scymnus_haemorrhoidalis_756 | Scymnus_suturalis_843 | 0.159 |
| Scymnus_haemorrhoidalis_747 | Scymnus_suturalis_844 | 0.159 |
| Scymnus_haemorrhoidalis_748 | Scymnus_suturalis_844 | 0.159 |
| Scymnus_haemorrhoidalis_756 | Scymnus_suturalis_845 | 0.159 |
| Scymnus_haemorrhoidalis_747 | Scymnus_suturalis_846 | 0.159 |
| Scymnus_haemorrhoidalis_748 | Scymnus_suturalis_846 | 0.159 |
| Scymnus_haemorrhoidalis_747 | Scymnus_suturalis_847 | 0.159 |
| Scymnus_haemorrhoidalis_748 | Scymnus_suturalis_847 | 0.159 |
| Scymnus_haemorrhoidalis_747 | Scymnus_suturalis_848 | 0.159 |
| Scymnus_haemorrhoidalis_748 | Scymnus_suturalis_848 | 0.159 |
| Calvia_quatuordecimguttata_206 | Tytthaspis_sedecimpunctata_586 | 0.159 |
| Calvia_quatuordecimguttata_216 | Tytthaspis_sedecimpunctata_586 | 0.159 |
| Calvia_quatuordecimguttata_209 | Tytthaspis_sedecimpunctata_587 | 0.159 |
| Calvia_quatuordecimguttata_207 | Tytthaspis_sedecimpunctata_588 | 0.159 |
| Calvia_quatuordecimguttata_207 | Tytthaspis_sedecimpunctata_591 | 0.159 |
| Calvia_quatuordecimguttata_218 | Tytthaspis_sedecimpunctata_591 | 0.159 |
| Scymnus_camptodromus_713 | Scymnus_rubromaculatus_822 | 0.159 |
| Scymnus_camptodromus_716 | Scymnus_rubromaculatus_822 | 0.159 |
| Scymnus_camptodromus_713 | Scymnus_rubromaculatus_823 | 0.159 |
| Scymnus_camptodromus_716 | Scymnus_rubromaculatus_823 | 0.159 |
| Scymnus_camptodromus_713 | Scymnus_rubromaculatus_824 | 0.159 |
| Scymnus_camptodromus_716 | Scymnus_rubromaculatus_824 | 0.159 |
| Scymnus_camptodromus_713 | Scymnus_rubromaculatus_825 | 0.159 |
| Scymnus_camptodromus_716 | Scymnus_rubromaculatus_825 | 0.159 |
| Scymnus_camptodromus_713 | Scymnus_rubromaculatus_826 | 0.159 |
| Scymnus_camptodromus_716 | Scymnus_rubromaculatus_826 | 0.159 |
| Scymnus_camptodromus_713 | Scymnus_rubromaculatus_827 | 0.159 |
| Scymnus_camptodromus_716 | Scymnus_rubromaculatus_827 | 0.159 |
| Scymnus_haemorrhoidalis_747 | Scymnus_suturalis_832 | 0.159 |
| Scymnus_haemorrhoidalis_748 | Scymnus_suturalis_832 | 0.159 |
| Scymnus_haemorrhoidalis_747 | Scymnus_suturalis_835 | 0.159 |
| Scymnus_haemorrhoidalis_748 | Scymnus_suturalis_835 | 0.159 |
| Scymnus_haemorrhoidalis_747 | Scymnus_suturalis_836 | 0.159 |
| Scymnus_haemorrhoidalis_748 | Scymnus_suturalis_836 | 0.159 |
| Scymnus_haemorrhoidalis_747 | Scymnus_suturalis_837 | 0.159 |
| Scymnus_haemorrhoidalis_748 | Scymnus_suturalis_837 | 0.159 |
| Scymnus_haemorrhoidalis_747 | Scymnus_suturalis_839 | 0.159 |
| Scymnus_haemorrhoidalis_748 | Scymnus_suturalis_839 | 0.159 |
| Hippodamia_parenthesis_432 | Tytthaspis_sedecimpunctata_577 | 0.159 |
| Hippodamia_parenthesis_434 | Tytthaspis_sedecimpunctata_577 | 0.159 |
| Hippodamia_parenthesis_435 | Tytthaspis_sedecimpunctata_577 | 0.159 |
| Hippodamia_parenthesis_437 | Tytthaspis_sedecimpunctata_577 | 0.159 |
| Hippodamia_parenthesis_438 | Tytthaspis_sedecimpunctata_577 | 0.159 |
| Hippodamia_parenthesis_441 | Tytthaspis_sedecimpunctata_577 | 0.159 |
| Hippodamia_parenthesis_442 | Tytthaspis_sedecimpunctata_577 | 0.159 |
| Hippodamia_parenthesis_432 | Tytthaspis_sedecimpunctata_578 | 0.159 |
| Hippodamia_parenthesis_434 | Tytthaspis_sedecimpunctata_578 | 0.159 |
| Hippodamia_parenthesis_435 | Tytthaspis_sedecimpunctata_578 | 0.159 |

| | | |
|---|---|---|
| Hippodamia_parenthesis_437 | Tytthaspis_sedecimpunctata_578 | 0.159 |
| Hippodamia_parenthesis_438 | Tytthaspis_sedecimpunctata_578 | 0.159 |
| Hippodamia_parenthesis_441 | Tytthaspis_sedecimpunctata_578 | 0.159 |
| Hippodamia_parenthesis_442 | Tytthaspis_sedecimpunctata_578 | 0.159 |
| Hippodamia_parenthesis_432 | Tytthaspis_sedecimpunctata_579 | 0.159 |
| Hippodamia_parenthesis_434 | Tytthaspis_sedecimpunctata_579 | 0.159 |
| Hippodamia_parenthesis_435 | Tytthaspis_sedecimpunctata_579 | 0.159 |
| Hippodamia_parenthesis_437 | Tytthaspis_sedecimpunctata_579 | 0.159 |
| Hippodamia_parenthesis_438 | Tytthaspis_sedecimpunctata_579 | 0.159 |
| Hippodamia_parenthesis_441 | Tytthaspis_sedecimpunctata_579 | 0.159 |
| Hippodamia_parenthesis_442 | Tytthaspis_sedecimpunctata_579 | 0.159 |
| Hippodamia_parenthesis_439 | Tytthaspis_sedecimpunctata_580 | 0.159 |
| Hippodamia_parenthesis_432 | Tytthaspis_sedecimpunctata_581 | 0.159 |
| Hippodamia_parenthesis_434 | Tytthaspis_sedecimpunctata_581 | 0.159 |
| Hippodamia_parenthesis_435 | Tytthaspis_sedecimpunctata_581 | 0.159 |
| Hippodamia_parenthesis_437 | Tytthaspis_sedecimpunctata_581 | 0.159 |
| Hippodamia_parenthesis_438 | Tytthaspis_sedecimpunctata_581 | 0.159 |
| Hippodamia_parenthesis_441 | Tytthaspis_sedecimpunctata_581 | 0.159 |
| Hippodamia_parenthesis_442 | Tytthaspis_sedecimpunctata_581 | 0.159 |
| Hippodamia_parenthesis_432 | Tytthaspis_sedecimpunctata_582 | 0.159 |
| Hippodamia_parenthesis_434 | Tytthaspis_sedecimpunctata_582 | 0.159 |
| Hippodamia_parenthesis_435 | Tytthaspis_sedecimpunctata_582 | 0.159 |
| Hippodamia_parenthesis_437 | Tytthaspis_sedecimpunctata_582 | 0.159 |
| Hippodamia_parenthesis_438 | Tytthaspis_sedecimpunctata_582 | 0.159 |
| Hippodamia_parenthesis_441 | Tytthaspis_sedecimpunctata_582 | 0.159 |
| Hippodamia_parenthesis_442 | Tytthaspis_sedecimpunctata_582 | 0.159 |
| Coccinula_quatuordecimpustulata_305 | Tytthaspis_sedecimpunctata_586 | 0.159 |
| Coleomegilla_maculata_329 | Tytthaspis_sedecimpunctata_587 | 0.159 |
| Coleomegilla_maculata_330 | Tytthaspis_sedecimpunctata_587 | 0.159 |
| Coleomegilla_maculata_331 | Tytthaspis_sedecimpunctata_587 | 0.159 |
| Coleomegilla_maculata_333 | Tytthaspis_sedecimpunctata_587 | 0.159 |
| Coleomegilla_maculata_334 | Tytthaspis_sedecimpunctata_587 | 0.159 |
| Coleomegilla_maculata_335 | Tytthaspis_sedecimpunctata_587 | 0.159 |
| Coleomegilla_maculata_336 | Tytthaspis_sedecimpunctata_587 | 0.159 |
| Coleomegilla_maculata_337 | Tytthaspis_sedecimpunctata_587 | 0.159 |
| Coleomegilla_maculata_338 | Tytthaspis_sedecimpunctata_587 | 0.159 |
| Coleomegilla_maculata_339 | Tytthaspis_sedecimpunctata_587 | 0.159 |
| Hippodamia_parenthesis_432 | Tytthaspis_sedecimpunctata_590 | 0.159 |
| Hippodamia_parenthesis_434 | Tytthaspis_sedecimpunctata_590 | 0.159 |
| Hippodamia_parenthesis_435 | Tytthaspis_sedecimpunctata_590 | 0.159 |
| Hippodamia_parenthesis_437 | Tytthaspis_sedecimpunctata_590 | 0.159 |
| Hippodamia_parenthesis_438 | Tytthaspis_sedecimpunctata_590 | 0.159 |
| Hippodamia_parenthesis_441 | Tytthaspis_sedecimpunctata_590 | 0.159 |
| Hippodamia_parenthesis_442 | Tytthaspis_sedecimpunctata_590 | 0.159 |
| Calvia_quatuordecimguttata_204 | Tytthaspis_sedecimpunctata_591 | 0.159 |
| Calvia_quatuordecimguttata_208 | Tytthaspis_sedecimpunctata_591 | 0.159 |
| Calvia_quatuordecimguttata_217 | Tytthaspis_sedecimpunctata_591 | 0.159 |
| Hippodamia_parenthesis_432 | Tytthaspis_sedecimpunctata_592 | 0.159 |
| Hippodamia_parenthesis_434 | Tytthaspis_sedecimpunctata_592 | 0.159 |
| Hippodamia_parenthesis_435 | Tytthaspis_sedecimpunctata_592 | 0.159 |
| Hippodamia_parenthesis_437 | Tytthaspis_sedecimpunctata_592 | 0.159 |
| Hippodamia_parenthesis_438 | Tytthaspis_sedecimpunctata_592 | 0.159 |
| Hippodamia_parenthesis_441 | Tytthaspis_sedecimpunctata_592 | 0.159 |
| Hippodamia_parenthesis_442 | Tytthaspis_sedecimpunctata_592 | 0.159 |
| Hippodamia_parenthesis_432 | Tytthaspis_sedecimpunctata_594 | 0.159 |
| Hippodamia_parenthesis_434 | Tytthaspis_sedecimpunctata_594 | 0.159 |

| | | |
|---|---|---|
| Hippodamia_parenthesis_435 | Tytthaspis_sedecimpunctata_594 | 0.159 |
| Hippodamia_parenthesis_437 | Tytthaspis_sedecimpunctata_594 | 0.159 |
| Hippodamia_parenthesis_438 | Tytthaspis_sedecimpunctata_594 | 0.159 |
| Hippodamia_parenthesis_441 | Tytthaspis_sedecimpunctata_594 | 0.159 |
| Hippodamia_parenthesis_442 | Tytthaspis_sedecimpunctata_594 | 0.159 |
| Hippodamia_parenthesis_432 | Tytthaspis_sedecimpunctata_595 | 0.159 |
| Hippodamia_parenthesis_434 | Tytthaspis_sedecimpunctata_595 | 0.159 |
| Hippodamia_parenthesis_435 | Tytthaspis_sedecimpunctata_595 | 0.159 |
| Hippodamia_parenthesis_437 | Tytthaspis_sedecimpunctata_595 | 0.159 |
| Hippodamia_parenthesis_438 | Tytthaspis_sedecimpunctata_595 | 0.159 |
| Hippodamia_parenthesis_441 | Tytthaspis_sedecimpunctata_595 | 0.159 |
| Hippodamia_parenthesis_442 | Tytthaspis_sedecimpunctata_595 | 0.159 |
| Scymnus_camptodromus_712 | Scymnus_rubromaculatus_822 | 0.159 |
| Scymnus_camptodromus_712 | Scymnus_rubromaculatus_823 | 0.159 |
| Scymnus_camptodromus_712 | Scymnus_rubromaculatus_824 | 0.159 |
| Scymnus_camptodromus_712 | Scymnus_rubromaculatus_825 | 0.159 |
| Scymnus_camptodromus_712 | Scymnus_rubromaculatus_826 | 0.159 |
| Scymnus_camptodromus_712 | Scymnus_rubromaculatus_827 | 0.159 |
| Scymnus_haemorrhoidalis_753 | Scymnus_suturalis_833 | 0.159 |
| Scymnus_haemorrhoidalis_753 | Scymnus_suturalis_838 | 0.159 |
| Scymnus_haemorrhoidalis_747 | Scymnus_suturalis_840 | 0.159 |
| Scymnus_haemorrhoidalis_748 | Scymnus_suturalis_840 | 0.159 |
| Scymnus_haemorrhoidalis_752 | Scymnus_suturalis_841 | 0.159 |
| Scymnus_haemorrhoidalis_752 | Scymnus_suturalis_842 | 0.159 |
| Scymnus_haemorrhoidalis_752 | Scymnus_suturalis_844 | 0.159 |
| Scymnus_haemorrhoidalis_752 | Scymnus_suturalis_846 | 0.159 |
| Scymnus_haemorrhoidalis_752 | Scymnus_suturalis_847 | 0.159 |
| Scymnus_haemorrhoidalis_752 | Scymnus_suturalis_848 | 0.159 |
| Coccinula_quatuordecimpustulata_292 | Tytthaspis_sedecimpunctata_577 | 0.159 |
| Coccinula_quatuordecimpustulata_293 | Tytthaspis_sedecimpunctata_577 | 0.159 |
| Coccinula_quatuordecimpustulata_307 | Tytthaspis_sedecimpunctata_577 | 0.159 |
| Macronaemia_episcopalis_477 | Tytthaspis_sedecimpunctata_577 | 0.159 |
| Coccinula_quatuordecimpustulata_292 | Tytthaspis_sedecimpunctata_578 | 0.159 |
| Coccinula_quatuordecimpustulata_293 | Tytthaspis_sedecimpunctata_578 | 0.159 |
| Coccinula_quatuordecimpustulata_307 | Tytthaspis_sedecimpunctata_578 | 0.159 |
| Macronaemia_episcopalis_477 | Tytthaspis_sedecimpunctata_578 | 0.159 |
| Coccinula_quatuordecimpustulata_292 | Tytthaspis_sedecimpunctata_579 | 0.159 |
| Coccinula_quatuordecimpustulata_293 | Tytthaspis_sedecimpunctata_579 | 0.159 |
| Coccinula_quatuordecimpustulata_307 | Tytthaspis_sedecimpunctata_579 | 0.159 |
| Macronaemia_episcopalis_477 | Tytthaspis_sedecimpunctata_579 | 0.159 |
| Hippodamia_parenthesis_433 | Tytthaspis_sedecimpunctata_580 | 0.159 |
| Hippodamia_parenthesis_440 | Tytthaspis_sedecimpunctata_580 | 0.159 |
| Coccinula_quatuordecimpustulata_292 | Tytthaspis_sedecimpunctata_581 | 0.159 |
| Coccinula_quatuordecimpustulata_293 | Tytthaspis_sedecimpunctata_581 | 0.159 |
| Coccinula_quatuordecimpustulata_307 | Tytthaspis_sedecimpunctata_581 | 0.159 |
| Macronaemia_episcopalis_477 | Tytthaspis_sedecimpunctata_581 | 0.159 |
| Coccinula_quatuordecimpustulata_292 | Tytthaspis_sedecimpunctata_582 | 0.159 |
| Coccinula_quatuordecimpustulata_293 | Tytthaspis_sedecimpunctata_582 | 0.159 |
| Coccinula_quatuordecimpustulata_307 | Tytthaspis_sedecimpunctata_582 | 0.159 |
| Macronaemia_episcopalis_477 | Tytthaspis_sedecimpunctata_582 | 0.159 |
| Coccinula_quatuordecimpustulata_294 | Tytthaspis_sedecimpunctata_583 | 0.159 |
| Coccinula_quatuordecimpustulata_295 | Tytthaspis_sedecimpunctata_583 | 0.159 |
| Coccinula_quatuordecimpustulata_308 | Tytthaspis_sedecimpunctata_583 | 0.159 |
| Coccinula_quatuordecimpustulata_292 | Tytthaspis_sedecimpunctata_585 | 0.159 |
| Coccinula_quatuordecimpustulata_293 | Tytthaspis_sedecimpunctata_585 | 0.159 |
| Coccinula_quatuordecimpustulata_307 | Tytthaspis_sedecimpunctata_585 | 0.159 |

| | | |
|---|---|---|
| Macronaemia_episcopalis_477 | Tytthaspis_sedecimpunctata_585 | 0.159 |
| Coccinula_quatuordecimpustulata_296 | Tytthaspis_sedecimpunctata_586 | 0.159 |
| Coccinula_quatuordecimpustulata_292 | Tytthaspis_sedecimpunctata_590 | 0.159 |
| Coccinula_quatuordecimpustulata_293 | Tytthaspis_sedecimpunctata_590 | 0.159 |
| Coccinula_quatuordecimpustulata_307 | Tytthaspis_sedecimpunctata_590 | 0.159 |
| Macronaemia_episcopalis_477 | Tytthaspis_sedecimpunctata_590 | 0.159 |
| Coccinula_quatuordecimpustulata_292 | Tytthaspis_sedecimpunctata_592 | 0.159 |
| Coccinula_quatuordecimpustulata_293 | Tytthaspis_sedecimpunctata_592 | 0.159 |
| Coccinula_quatuordecimpustulata_307 | Tytthaspis_sedecimpunctata_592 | 0.159 |
| Coccinula_quatuordecimpustulata_292 | Tytthaspis_sedecimpunctata_593 | 0.159 |
| Coccinula_quatuordecimpustulata_293 | Tytthaspis_sedecimpunctata_593 | 0.159 |
| Coccinula_quatuordecimpustulata_307 | Tytthaspis_sedecimpunctata_593 | 0.159 |
| Coccinula_quatuordecimpustulata_292 | Tytthaspis_sedecimpunctata_594 | 0.159 |
| Coccinula_quatuordecimpustulata_293 | Tytthaspis_sedecimpunctata_594 | 0.159 |
| Coccinula_quatuordecimpustulata_307 | Tytthaspis_sedecimpunctata_594 | 0.159 |
| Macronaemia_episcopalis_477 | Tytthaspis_sedecimpunctata_594 | 0.159 |
| Coccinula_quatuordecimpustulata_292 | Tytthaspis_sedecimpunctata_595 | 0.159 |
| Coccinula_quatuordecimpustulata_293 | Tytthaspis_sedecimpunctata_595 | 0.159 |
| Coccinula_quatuordecimpustulata_307 | Tytthaspis_sedecimpunctata_595 | 0.159 |
| Macronaemia_episcopalis_477 | Tytthaspis_sedecimpunctata_595 | 0.159 |
| Scymnus_haemorrhoidalis_752 | Scymnus_suturalis_832 | 0.159 |
| Scymnus_nigrinus_785 | Scymnus_suturalis_833 | 0.159 |
| Scymnus_nigrinus_786 | Scymnus_suturalis_833 | 0.159 |
| Scymnus_haemorrhoidalis_753 | Scymnus_suturalis_834 | 0.159 |
| Scymnus_haemorrhoidalis_752 | Scymnus_suturalis_835 | 0.159 |
| Scymnus_haemorrhoidalis_752 | Scymnus_suturalis_836 | 0.159 |
| Scymnus_haemorrhoidalis_752 | Scymnus_suturalis_837 | 0.159 |
| Scymnus_haemorrhoidalis_752 | Scymnus_suturalis_839 | 0.159 |
| Scymnus_nigrinus_785 | Scymnus_suturalis_840 | 0.159 |
| Scymnus_nigrinus_786 | Scymnus_suturalis_840 | 0.159 |
| Psyllobora_vigintiduopunctata_558 | Tytthaspis_sedecimpunctata_587 | 0.159 |
| Psyllobora_vigintiduopunctata_568 | Tytthaspis_sedecimpunctata_587 | 0.159 |
| Oenopia_conglobata_524 | Tytthaspis_sedecimpunctata_593 | 0.159 |
| Scymnus_interruptus_767 | Scymnus_sinuanodulus_831 | 0.159 |
| Coccinula_quatuordecimpustulata_294 | Tytthaspis_sedecimpunctata_580 | 0.159 |
| Coccinula_quatuordecimpustulata_295 | Tytthaspis_sedecimpunctata_580 | 0.159 |
| Coccinula_quatuordecimpustulata_308 | Tytthaspis_sedecimpunctata_580 | 0.159 |
| Coccinula_quatuordecimpustulata_296 | Tytthaspis_sedecimpunctata_583 | 0.159 |
| Coccinula_quatuordecimpustulata_292 | Tytthaspis_sedecimpunctata_584 | 0.159 |
| Coccinula_quatuordecimpustulata_293 | Tytthaspis_sedecimpunctata_584 | 0.159 |
| Coccinula_quatuordecimpustulata_307 | Tytthaspis_sedecimpunctata_584 | 0.159 |
| Coleomegilla_maculata_327 | Tytthaspis_sedecimpunctata_591 | 0.159 |
| Scymnus_nigrinus_785 | Scymnus_suturalis_834 | 0.159 |
| Scymnus_nigrinus_786 | Scymnus_suturalis_834 | 0.159 |
| Scymnus_haemorrhoidalis_752 | Scymnus_suturalis_840 | 0.159 |
| Hippodamia_parenthesis_436 | Tytthaspis_sedecimpunctata_588 | 0.159 |
| Macronaemia_episcopalis_478 | Tytthaspis_sedecimpunctata_589 | 0.159 |
| Scymnus_interruptus_762 | Scymnus_sinuanodulus_831 | 0.159 |
| Coccinula_quatuordecimpustulata_296 | Tytthaspis_sedecimpunctata_580 | 0.159 |
| Scymnus_mitior_780 | Scymnus_rubromaculatus_822 | 0.159 |
| Scymnus_mitior_780 | Scymnus_rubromaculatus_823 | 0.159 |
| Scymnus_mitior_780 | Scymnus_rubromaculatus_824 | 0.159 |
| Scymnus_mitior_780 | Scymnus_rubromaculatus_825 | 0.159 |
| Scymnus_mitior_780 | Scymnus_rubromaculatus_826 | 0.159 |
| Scymnus_mitior_780 | Scymnus_rubromaculatus_827 | 0.159 |
| Hippodamia_parenthesis_436 | Tytthaspis_sedecimpunctata_583 | 0.159 |

| | | |
|---|---|---|
| Macronaemia_episcopalis_478 | Tytthaspis_sedecimpunctata_584 | 0.159 |
| Hippodamia_parenthesis_436 | Tytthaspis_sedecimpunctata_585 | 0.159 |
| Macronaemia_episcopalis_478 | Tytthaspis_sedecimpunctata_587 | 0.159 |
| Hippodamia_parenthesis_436 | Tytthaspis_sedecimpunctata_593 | 0.159 |
| Scymnus_nigrinus_787 | Scymnus_sinuanodulus_831 | 0.160 |
| Scymnus_frontalis_736 | Scymnus_rubromaculatus_822 | 0.160 |
| Scymnus_frontalis_737 | Scymnus_rubromaculatus_822 | 0.160 |
| Scymnus_frontalis_738 | Scymnus_rubromaculatus_822 | 0.160 |
| Scymnus_frontalis_739 | Scymnus_rubromaculatus_822 | 0.160 |
| Scymnus_frontalis_740 | Scymnus_rubromaculatus_822 | 0.160 |
| Scymnus_frontalis_741 | Scymnus_rubromaculatus_822 | 0.160 |
| Scymnus_frontalis_743 | Scymnus_rubromaculatus_822 | 0.160 |
| Scymnus_frontalis_744 | Scymnus_rubromaculatus_822 | 0.160 |
| Scymnus_frontalis_736 | Scymnus_rubromaculatus_823 | 0.160 |
| Scymnus_frontalis_737 | Scymnus_rubromaculatus_823 | 0.160 |
| Scymnus_frontalis_738 | Scymnus_rubromaculatus_823 | 0.160 |
| Scymnus_frontalis_739 | Scymnus_rubromaculatus_823 | 0.160 |
| Scymnus_frontalis_740 | Scymnus_rubromaculatus_823 | 0.160 |
| Scymnus_frontalis_741 | Scymnus_rubromaculatus_823 | 0.160 |
| Scymnus_frontalis_743 | Scymnus_rubromaculatus_823 | 0.160 |
| Scymnus_frontalis_744 | Scymnus_rubromaculatus_823 | 0.160 |
| Scymnus_frontalis_736 | Scymnus_rubromaculatus_824 | 0.160 |
| Scymnus_frontalis_737 | Scymnus_rubromaculatus_824 | 0.160 |
| Scymnus_frontalis_738 | Scymnus_rubromaculatus_824 | 0.160 |
| Scymnus_frontalis_739 | Scymnus_rubromaculatus_824 | 0.160 |
| Scymnus_frontalis_740 | Scymnus_rubromaculatus_824 | 0.160 |
| Scymnus_frontalis_741 | Scymnus_rubromaculatus_824 | 0.160 |
| Scymnus_frontalis_743 | Scymnus_rubromaculatus_824 | 0.160 |
| Scymnus_frontalis_744 | Scymnus_rubromaculatus_824 | 0.160 |
| Scymnus_frontalis_736 | Scymnus_rubromaculatus_825 | 0.160 |
| Scymnus_frontalis_737 | Scymnus_rubromaculatus_825 | 0.160 |
| Scymnus_frontalis_738 | Scymnus_rubromaculatus_825 | 0.160 |
| Scymnus_frontalis_739 | Scymnus_rubromaculatus_825 | 0.160 |
| Scymnus_frontalis_740 | Scymnus_rubromaculatus_825 | 0.160 |
| Scymnus_frontalis_741 | Scymnus_rubromaculatus_825 | 0.160 |
| Scymnus_frontalis_743 | Scymnus_rubromaculatus_825 | 0.160 |
| Scymnus_frontalis_744 | Scymnus_rubromaculatus_825 | 0.160 |
| Scymnus_frontalis_736 | Scymnus_rubromaculatus_826 | 0.160 |
| Scymnus_frontalis_737 | Scymnus_rubromaculatus_826 | 0.160 |
| Scymnus_frontalis_738 | Scymnus_rubromaculatus_826 | 0.160 |
| Scymnus_frontalis_739 | Scymnus_rubromaculatus_826 | 0.160 |
| Scymnus_frontalis_740 | Scymnus_rubromaculatus_826 | 0.160 |
| Scymnus_frontalis_741 | Scymnus_rubromaculatus_826 | 0.160 |
| Scymnus_frontalis_743 | Scymnus_rubromaculatus_826 | 0.160 |
| Scymnus_frontalis_744 | Scymnus_rubromaculatus_826 | 0.160 |
| Scymnus_frontalis_736 | Scymnus_rubromaculatus_827 | 0.160 |
| Scymnus_frontalis_737 | Scymnus_rubromaculatus_827 | 0.160 |
| Scymnus_frontalis_738 | Scymnus_rubromaculatus_827 | 0.160 |
| Scymnus_frontalis_739 | Scymnus_rubromaculatus_827 | 0.160 |
| Scymnus_frontalis_740 | Scymnus_rubromaculatus_827 | 0.160 |
| Scymnus_frontalis_741 | Scymnus_rubromaculatus_827 | 0.160 |
| Scymnus_frontalis_743 | Scymnus_rubromaculatus_827 | 0.160 |
| Scymnus_frontalis_744 | Scymnus_rubromaculatus_827 | 0.160 |
| Scymnus_nebulosus_783 | Scymnus_sinuanodulus_828 | 0.160 |
| Scymnus_nigrinus_784 | Scymnus_sinuanodulus_829 | 0.160 |
| Scymnus_apetzi_695 | Scymnus_suturalis_841 | 0.160 |

| | | |
|---|---|---|
| Scymnus_apetzi_695 | Scymnus_suturalis_842 | 0.160 |
| Scymnus_apetzi_695 | Scymnus_suturalis_844 | 0.160 |
| Scymnus_apetzi_695 | Scymnus_suturalis_846 | 0.160 |
| Scymnus_apetzi_695 | Scymnus_suturalis_847 | 0.160 |
| Scymnus_apetzi_695 | Scymnus_suturalis_848 | 0.160 |
| Scymnus_apetzi_695 | Scymnus_suturalis_832 | 0.160 |
| Scymnus_apetzi_695 | Scymnus_suturalis_835 | 0.160 |
| Scymnus_apetzi_695 | Scymnus_suturalis_836 | 0.160 |
| Scymnus_apetzi_695 | Scymnus_suturalis_839 | 0.160 |
| Brumus_quadripustulatus_5 | Tytthaspis_sedecimpunctata_577 | 0.160 |
| Brumus_quadripustulatus_5 | Tytthaspis_sedecimpunctata_578 | 0.160 |
| Brumus_quadripustulatus_5 | Tytthaspis_sedecimpunctata_579 | 0.160 |
| Brumus_quadripustulatus_5 | Tytthaspis_sedecimpunctata_581 | 0.160 |
| Brumus_quadripustulatus_5 | Tytthaspis_sedecimpunctata_582 | 0.160 |
| Brumus_quadripustulatus_5 | Tytthaspis_sedecimpunctata_590 | 0.160 |
| Brumus_quadripustulatus_5 | Tytthaspis_sedecimpunctata_592 | 0.160 |
| Brumus_quadripustulatus_5 | Tytthaspis_sedecimpunctata_594 | 0.160 |
| Brumus_quadripustulatus_5 | Tytthaspis_sedecimpunctata_595 | 0.160 |
| Anisosticta_novemdecimpunctata_170 | Tytthaspis_sedecimpunctata_589 | 0.160 |
| Anisosticta_novemdecimpunctata_169 | Tytthaspis_sedecimpunctata_591 | 0.160 |
| Anisosticta_novemdecimpunctata_172 | Tytthaspis_sedecimpunctata_591 | 0.160 |
| Anisosticta_novemdecimpunctata_170 | Tytthaspis_sedecimpunctata_584 | 0.160 |
| Anisosticta_novemdecimpunctata_170 | Tytthaspis_sedecimpunctata_587 | 0.160 |
| Scymnus_frontalis_746 | Scymnus_rubromaculatus_822 | 0.160 |
| Scymnus_frontalis_746 | Scymnus_rubromaculatus_823 | 0.160 |
| Scymnus_frontalis_746 | Scymnus_rubromaculatus_824 | 0.160 |
| Scymnus_frontalis_746 | Scymnus_rubromaculatus_825 | 0.160 |
| Scymnus_frontalis_746 | Scymnus_rubromaculatus_826 | 0.160 |
| Scymnus_frontalis_746 | Scymnus_rubromaculatus_827 | 0.160 |
| Anisosticta_novemdecimpunctata_170 | Tytthaspis_sedecimpunctata_583 | 0.160 |
| Hyperaspis_proba_661 | Tytthaspis_sedecimpunctata_588 | 0.160 |
| Chilocorus_bipustulatus_23 | Tytthaspis_sedecimpunctata_583 | 0.160 |
| Chilocorus_bipustulatus_18 | Tytthaspis_sedecimpunctata_586 | 0.160 |
| Chilocorus_bipustulatus_20 | Tytthaspis_sedecimpunctata_586 | 0.160 |
| Chilocorus_bipustulatus_18 | Tytthaspis_sedecimpunctata_588 | 0.160 |
| Chilocorus_bipustulatus_20 | Tytthaspis_sedecimpunctata_588 | 0.160 |
| Chilocorus_bipustulatus_23 | Tytthaspis_sedecimpunctata_592 | 0.160 |
| Chilocorus_bipustulatus_18 | Tytthaspis_sedecimpunctata_577 | 0.160 |
| Chilocorus_bipustulatus_20 | Tytthaspis_sedecimpunctata_577 | 0.160 |
| Chilocorus_bipustulatus_18 | Tytthaspis_sedecimpunctata_578 | 0.160 |
| Chilocorus_bipustulatus_20 | Tytthaspis_sedecimpunctata_578 | 0.160 |
| Chilocorus_bipustulatus_18 | Tytthaspis_sedecimpunctata_579 | 0.160 |
| Chilocorus_bipustulatus_20 | Tytthaspis_sedecimpunctata_579 | 0.160 |
| Chilocorus_bipustulatus_23 | Tytthaspis_sedecimpunctata_580 | 0.160 |
| Chilocorus_bipustulatus_18 | Tytthaspis_sedecimpunctata_581 | 0.160 |
| Chilocorus_bipustulatus_20 | Tytthaspis_sedecimpunctata_581 | 0.160 |
| Chilocorus_bipustulatus_18 | Tytthaspis_sedecimpunctata_582 | 0.160 |
| Chilocorus_bipustulatus_20 | Tytthaspis_sedecimpunctata_582 | 0.160 |
| Chilocorus_bipustulatus_24 | Tytthaspis_sedecimpunctata_583 | 0.160 |
| Chilocorus_bipustulatus_18 | Tytthaspis_sedecimpunctata_590 | 0.160 |
| Chilocorus_bipustulatus_20 | Tytthaspis_sedecimpunctata_590 | 0.160 |
| Chilocorus_bipustulatus_24 | Tytthaspis_sedecimpunctata_592 | 0.160 |
| Chilocorus_bipustulatus_18 | Tytthaspis_sedecimpunctata_593 | 0.160 |
| Chilocorus_bipustulatus_20 | Tytthaspis_sedecimpunctata_593 | 0.160 |
| Coccinella_magnifica_229 | Tytthaspis_sedecimpunctata_593 | 0.160 |
| Coccinella_magnifica_230 | Tytthaspis_sedecimpunctata_593 | 0.160 |

| | | |
|---|---|---|
| Coccinella_magnifica_231 | Tytthaspis_sedecimpunctata_593 | 0.160 |
| Chilocorus_bipustulatus_18 | Tytthaspis_sedecimpunctata_594 | 0.160 |
| Chilocorus_bipustulatus_20 | Tytthaspis_sedecimpunctata_594 | 0.160 |
| Chilocorus_bipustulatus_18 | Tytthaspis_sedecimpunctata_595 | 0.160 |
| Chilocorus_bipustulatus_20 | Tytthaspis_sedecimpunctata_595 | 0.160 |
| Chilocorus_bipustulatus_24 | Tytthaspis_sedecimpunctata_580 | 0.160 |
| Chilocorus_stigma_45 | Tytthaspis_sedecimpunctata_583 | 0.160 |
| Chilocorus_stigma_45 | Tytthaspis_sedecimpunctata_580 | 0.160 |
| Coccinella_transversoguttata_267 | Tytthaspis_sedecimpunctata_580 | 0.160 |
| Coccinella_transversoguttata_268 | Tytthaspis_sedecimpunctata_580 | 0.160 |
| Coccinella_transversoguttata_269 | Tytthaspis_sedecimpunctata_580 | 0.160 |
| Coccinella_transversoguttata_270 | Tytthaspis_sedecimpunctata_580 | 0.160 |
| Coccinella_transversoguttata_271 | Tytthaspis_sedecimpunctata_580 | 0.160 |
| Anatis_ocellata_132 | Tytthaspis_sedecimpunctata_589 | 0.160 |
| Anatis_ocellata_134 | Tytthaspis_sedecimpunctata_589 | 0.160 |
| Anatis_ocellata_135 | Tytthaspis_sedecimpunctata_589 | 0.160 |
| Anatis_ocellata_138 | Tytthaspis_sedecimpunctata_589 | 0.160 |
| Anatis_ocellata_139 | Tytthaspis_sedecimpunctata_589 | 0.160 |
| Anatis_ocellata_140 | Tytthaspis_sedecimpunctata_589 | 0.160 |
| Anatis_ocellata_141 | Tytthaspis_sedecimpunctata_589 | 0.160 |
| Anatis_ocellata_142 | Tytthaspis_sedecimpunctata_589 | 0.160 |
| Anatis_ocellata_144 | Tytthaspis_sedecimpunctata_589 | 0.160 |
| Anatis_ocellata_145 | Tytthaspis_sedecimpunctata_589 | 0.160 |
| Anatis_ocellata_146 | Tytthaspis_sedecimpunctata_589 | 0.160 |
| Anatis_mali_128 | Tytthaspis_sedecimpunctata_583 | 0.161 |
| Anatis_mali_129 | Tytthaspis_sedecimpunctata_583 | 0.161 |
| Anatis_mali_130 | Tytthaspis_sedecimpunctata_583 | 0.161 |
| Anatis_mali_131 | Tytthaspis_sedecimpunctata_583 | 0.161 |
| Anatis_ocellata_132 | Tytthaspis_sedecimpunctata_588 | 0.161 |
| Anatis_ocellata_134 | Tytthaspis_sedecimpunctata_588 | 0.161 |
| Anatis_ocellata_135 | Tytthaspis_sedecimpunctata_588 | 0.161 |
| Anatis_ocellata_138 | Tytthaspis_sedecimpunctata_588 | 0.161 |
| Anatis_ocellata_139 | Tytthaspis_sedecimpunctata_588 | 0.161 |
| Anatis_ocellata_140 | Tytthaspis_sedecimpunctata_588 | 0.161 |
| Anatis_ocellata_141 | Tytthaspis_sedecimpunctata_588 | 0.161 |
| Anatis_ocellata_142 | Tytthaspis_sedecimpunctata_588 | 0.161 |
| Anatis_ocellata_144 | Tytthaspis_sedecimpunctata_588 | 0.161 |
| Anatis_ocellata_145 | Tytthaspis_sedecimpunctata_588 | 0.161 |
| Anatis_ocellata_146 | Tytthaspis_sedecimpunctata_588 | 0.161 |
| Mulsantina_hudsonica_480 | Tytthaspis_sedecimpunctata_591 | 0.161 |
| Anatis_mali_128 | Tytthaspis_sedecimpunctata_592 | 0.161 |
| Anatis_mali_129 | Tytthaspis_sedecimpunctata_592 | 0.161 |
| Anatis_mali_130 | Tytthaspis_sedecimpunctata_592 | 0.161 |
| Anatis_mali_131 | Tytthaspis_sedecimpunctata_592 | 0.161 |
| Scymnus_pallipediformis_811 | Scymnus_suturalis_843 | 0.161 |
| Scymnus_pallipediformis_811 | Scymnus_suturalis_845 | 0.161 |
| Anatis_ocellata_132 | Tytthaspis_sedecimpunctata_577 | 0.161 |
| Anatis_ocellata_134 | Tytthaspis_sedecimpunctata_577 | 0.161 |
| Anatis_ocellata_135 | Tytthaspis_sedecimpunctata_577 | 0.161 |
| Anatis_ocellata_138 | Tytthaspis_sedecimpunctata_577 | 0.161 |
| Anatis_ocellata_139 | Tytthaspis_sedecimpunctata_577 | 0.161 |
| Anatis_ocellata_140 | Tytthaspis_sedecimpunctata_577 | 0.161 |
| Anatis_ocellata_141 | Tytthaspis_sedecimpunctata_577 | 0.161 |
| Anatis_ocellata_142 | Tytthaspis_sedecimpunctata_577 | 0.161 |
| Anatis_ocellata_144 | Tytthaspis_sedecimpunctata_577 | 0.161 |
| Anatis_ocellata_145 | Tytthaspis_sedecimpunctata_577 | 0.161 |

| | | |
|---|---|---|
| Anatis_ocellata_146 | Tytthaspis_sedecimpunctata_577 | 0.161 |
| Anatis_ocellata_132 | Tytthaspis_sedecimpunctata_578 | 0.161 |
| Anatis_ocellata_134 | Tytthaspis_sedecimpunctata_578 | 0.161 |
| Anatis_ocellata_135 | Tytthaspis_sedecimpunctata_578 | 0.161 |
| Anatis_ocellata_138 | Tytthaspis_sedecimpunctata_578 | 0.161 |
| Anatis_ocellata_139 | Tytthaspis_sedecimpunctata_578 | 0.161 |
| Anatis_ocellata_140 | Tytthaspis_sedecimpunctata_578 | 0.161 |
| Anatis_ocellata_141 | Tytthaspis_sedecimpunctata_578 | 0.161 |
| Anatis_ocellata_142 | Tytthaspis_sedecimpunctata_578 | 0.161 |
| Anatis_ocellata_144 | Tytthaspis_sedecimpunctata_578 | 0.161 |
| Anatis_ocellata_145 | Tytthaspis_sedecimpunctata_578 | 0.161 |
| Anatis_ocellata_146 | Tytthaspis_sedecimpunctata_578 | 0.161 |
| Anatis_ocellata_132 | Tytthaspis_sedecimpunctata_579 | 0.161 |
| Anatis_ocellata_134 | Tytthaspis_sedecimpunctata_579 | 0.161 |
| Anatis_ocellata_135 | Tytthaspis_sedecimpunctata_579 | 0.161 |
| Anatis_ocellata_138 | Tytthaspis_sedecimpunctata_579 | 0.161 |
| Anatis_ocellata_139 | Tytthaspis_sedecimpunctata_579 | 0.161 |
| Anatis_ocellata_140 | Tytthaspis_sedecimpunctata_579 | 0.161 |
| Anatis_ocellata_141 | Tytthaspis_sedecimpunctata_579 | 0.161 |
| Anatis_ocellata_142 | Tytthaspis_sedecimpunctata_579 | 0.161 |
| Anatis_ocellata_144 | Tytthaspis_sedecimpunctata_579 | 0.161 |
| Anatis_ocellata_145 | Tytthaspis_sedecimpunctata_579 | 0.161 |
| Anatis_ocellata_146 | Tytthaspis_sedecimpunctata_579 | 0.161 |
| Anatis_mali_128 | Tytthaspis_sedecimpunctata_580 | 0.161 |
| Anatis_mali_129 | Tytthaspis_sedecimpunctata_580 | 0.161 |
| Anatis_mali_130 | Tytthaspis_sedecimpunctata_580 | 0.161 |
| Anatis_mali_131 | Tytthaspis_sedecimpunctata_580 | 0.161 |
| Anatis_ocellata_132 | Tytthaspis_sedecimpunctata_581 | 0.161 |
| Anatis_ocellata_134 | Tytthaspis_sedecimpunctata_581 | 0.161 |
| Anatis_ocellata_135 | Tytthaspis_sedecimpunctata_581 | 0.161 |
| Anatis_ocellata_138 | Tytthaspis_sedecimpunctata_581 | 0.161 |
| Anatis_ocellata_139 | Tytthaspis_sedecimpunctata_581 | 0.161 |
| Anatis_ocellata_140 | Tytthaspis_sedecimpunctata_581 | 0.161 |
| Anatis_ocellata_141 | Tytthaspis_sedecimpunctata_581 | 0.161 |
| Anatis_ocellata_142 | Tytthaspis_sedecimpunctata_581 | 0.161 |
| Anatis_ocellata_144 | Tytthaspis_sedecimpunctata_581 | 0.161 |
| Anatis_ocellata_145 | Tytthaspis_sedecimpunctata_581 | 0.161 |
| Anatis_ocellata_146 | Tytthaspis_sedecimpunctata_581 | 0.161 |
| Anatis_ocellata_132 | Tytthaspis_sedecimpunctata_582 | 0.161 |
| Anatis_ocellata_134 | Tytthaspis_sedecimpunctata_582 | 0.161 |
| Anatis_ocellata_135 | Tytthaspis_sedecimpunctata_582 | 0.161 |
| Anatis_ocellata_138 | Tytthaspis_sedecimpunctata_582 | 0.161 |
| Anatis_ocellata_139 | Tytthaspis_sedecimpunctata_582 | 0.161 |
| Anatis_ocellata_140 | Tytthaspis_sedecimpunctata_582 | 0.161 |
| Anatis_ocellata_141 | Tytthaspis_sedecimpunctata_582 | 0.161 |
| Anatis_ocellata_142 | Tytthaspis_sedecimpunctata_582 | 0.161 |
| Anatis_ocellata_144 | Tytthaspis_sedecimpunctata_582 | 0.161 |
| Anatis_ocellata_145 | Tytthaspis_sedecimpunctata_582 | 0.161 |
| Anatis_ocellata_146 | Tytthaspis_sedecimpunctata_582 | 0.161 |
| Hippodamia_alpina_383 | Tytthaspis_sedecimpunctata_586 | 0.161 |
| Hippodamia_alpina_383 | Tytthaspis_sedecimpunctata_588 | 0.161 |
| Psyllobora_vigintiduopunctata_557 | Tytthaspis_sedecimpunctata_589 | 0.161 |
| Psyllobora_vigintiduopunctata_561 | Tytthaspis_sedecimpunctata_589 | 0.161 |
| Psyllobora_vigintiduopunctata_562 | Tytthaspis_sedecimpunctata_589 | 0.161 |
| Psyllobora_vigintiduopunctata_563 | Tytthaspis_sedecimpunctata_589 | 0.161 |
| Psyllobora_vigintiduopunctata_565 | Tytthaspis_sedecimpunctata_589 | 0.161 |

| | | |
|---|---|---|
| Psyllobora_vigintiduopunctata_567 | Tytthaspis_sedecimpunctata_589 | 0.161 |
| Anatis_ocellata_132 | Tytthaspis_sedecimpunctata_590 | 0.161 |
| Anatis_ocellata_134 | Tytthaspis_sedecimpunctata_590 | 0.161 |
| Anatis_ocellata_135 | Tytthaspis_sedecimpunctata_590 | 0.161 |
| Anatis_ocellata_138 | Tytthaspis_sedecimpunctata_590 | 0.161 |
| Anatis_ocellata_139 | Tytthaspis_sedecimpunctata_590 | 0.161 |
| Anatis_ocellata_140 | Tytthaspis_sedecimpunctata_590 | 0.161 |
| Anatis_ocellata_141 | Tytthaspis_sedecimpunctata_590 | 0.161 |
| Anatis_ocellata_142 | Tytthaspis_sedecimpunctata_590 | 0.161 |
| Anatis_ocellata_144 | Tytthaspis_sedecimpunctata_590 | 0.161 |
| Anatis_ocellata_145 | Tytthaspis_sedecimpunctata_590 | 0.161 |
| Anatis_ocellata_146 | Tytthaspis_sedecimpunctata_590 | 0.161 |
| Anatis_ocellata_132 | Tytthaspis_sedecimpunctata_594 | 0.161 |
| Anatis_ocellata_134 | Tytthaspis_sedecimpunctata_594 | 0.161 |
| Anatis_ocellata_135 | Tytthaspis_sedecimpunctata_594 | 0.161 |
| Anatis_ocellata_138 | Tytthaspis_sedecimpunctata_594 | 0.161 |
| Anatis_ocellata_139 | Tytthaspis_sedecimpunctata_594 | 0.161 |
| Anatis_ocellata_140 | Tytthaspis_sedecimpunctata_594 | 0.161 |
| Anatis_ocellata_141 | Tytthaspis_sedecimpunctata_594 | 0.161 |
| Anatis_ocellata_142 | Tytthaspis_sedecimpunctata_594 | 0.161 |
| Anatis_ocellata_144 | Tytthaspis_sedecimpunctata_594 | 0.161 |
| Anatis_ocellata_145 | Tytthaspis_sedecimpunctata_594 | 0.161 |
| Anatis_ocellata_146 | Tytthaspis_sedecimpunctata_594 | 0.161 |
| Anatis_ocellata_132 | Tytthaspis_sedecimpunctata_595 | 0.161 |
| Anatis_ocellata_134 | Tytthaspis_sedecimpunctata_595 | 0.161 |
| Anatis_ocellata_135 | Tytthaspis_sedecimpunctata_595 | 0.161 |
| Anatis_ocellata_138 | Tytthaspis_sedecimpunctata_595 | 0.161 |
| Anatis_ocellata_139 | Tytthaspis_sedecimpunctata_595 | 0.161 |
| Anatis_ocellata_140 | Tytthaspis_sedecimpunctata_595 | 0.161 |
| Anatis_ocellata_141 | Tytthaspis_sedecimpunctata_595 | 0.161 |
| Anatis_ocellata_142 | Tytthaspis_sedecimpunctata_595 | 0.161 |
| Anatis_ocellata_144 | Tytthaspis_sedecimpunctata_595 | 0.161 |
| Anatis_ocellata_145 | Tytthaspis_sedecimpunctata_595 | 0.161 |
| Anatis_ocellata_146 | Tytthaspis_sedecimpunctata_595 | 0.161 |
| Scymnus_mimulus_776 | Scymnus_suturalis_833 | 0.161 |
| Scymnus_pallipediformis_811 | Scymnus_suturalis_837 | 0.161 |
| Halyzia_sedecimguttata_352 | Vibidia_duodecimguttata_601 | 0.161 |
| Scymnus_mimulus_776 | Scymnus_suturalis_841 | 0.161 |
| Scymnus_mimulus_776 | Scymnus_suturalis_842 | 0.161 |
| Scymnus_mimulus_775 | Scymnus_suturalis_843 | 0.161 |
| Scymnus_mimulus_777 | Scymnus_suturalis_843 | 0.161 |
| Scymnus_mimulus_776 | Scymnus_suturalis_844 | 0.161 |
| Scymnus_mimulus_775 | Scymnus_suturalis_845 | 0.161 |
| Scymnus_mimulus_777 | Scymnus_suturalis_845 | 0.161 |
| Scymnus_mimulus_776 | Scymnus_suturalis_846 | 0.161 |
| Scymnus_mimulus_776 | Scymnus_suturalis_847 | 0.161 |
| Scymnus_mimulus_776 | Scymnus_suturalis_848 | 0.161 |
| Hyperaspis_oregona_658 | Subcoccinella_vigintiquatuorpunctata_623 | 0.161 |
| Hyperaspis_oregona_659 | Subcoccinella_vigintiquatuorpunctata_623 | 0.161 |
| Psyllobora_vigintiduopunctata_557 | Tytthaspis_sedecimpunctata_586 | 0.161 |
| Psyllobora_vigintiduopunctata_561 | Tytthaspis_sedecimpunctata_586 | 0.161 |
| Psyllobora_vigintiduopunctata_562 | Tytthaspis_sedecimpunctata_586 | 0.161 |
| Psyllobora_vigintiduopunctata_563 | Tytthaspis_sedecimpunctata_586 | 0.161 |
| Psyllobora_vigintiduopunctata_565 | Tytthaspis_sedecimpunctata_586 | 0.161 |
| Psyllobora_vigintiduopunctata_567 | Tytthaspis_sedecimpunctata_586 | 0.161 |
| Coelophora_inaequalis_320 | Tytthaspis_sedecimpunctata_588 | 0.161 |

| | | |
|---|---|---|
| Coelophora_inaequalis_321 | Tytthaspis_sedecimpunctata_588 | 0.161 |
| Coelophora_inaequalis_322 | Tytthaspis_sedecimpunctata_588 | 0.161 |
| Psyllobora_vigintiduopunctata_564 | Tytthaspis_sedecimpunctata_588 | 0.161 |
| Psyllobora_vigintiduopunctata_570 | Tytthaspis_sedecimpunctata_588 | 0.161 |
| Psyllobora_vigintiduopunctata_572 | Tytthaspis_sedecimpunctata_588 | 0.161 |
| Psyllobora_vigintiduopunctata_556 | Tytthaspis_sedecimpunctata_589 | 0.161 |
| Scymnus_mimulus_776 | Scymnus_suturalis_832 | 0.161 |
| Scymnus_mimulus_776 | Scymnus_suturalis_835 | 0.161 |
| Scymnus_mimulus_776 | Scymnus_suturalis_836 | 0.161 |
| Scymnus_mimulus_775 | Scymnus_suturalis_837 | 0.161 |
| Scymnus_mimulus_777 | Scymnus_suturalis_837 | 0.161 |
| Scymnus_mimulus_776 | Scymnus_suturalis_839 | 0.161 |
| Hyperaspis_proba_660 | Tytthaspis_sedecimpunctata_588 | 0.161 |
| Hyperaspis_oregona_658 | Subcoccinella_vigintiquatuorpunctata_624 | 0.161 |
| Hyperaspis_oregona_659 | Subcoccinella_vigintiquatuorpunctata_624 | 0.161 |
| Hyperaspis_oregona_658 | Subcoccinella_vigintiquatuorpunctata_626 | 0.161 |
| Hyperaspis_oregona_659 | Subcoccinella_vigintiquatuorpunctata_626 | 0.161 |
| Scymnus_haemorrhoidalis_756 | Tytthaspis_sedecimpunctata_584 | 0.161 |
| Psyllobora_vigintiduopunctata_556 | Tytthaspis_sedecimpunctata_586 | 0.161 |
| Scymnus_haemorrhoidalis_747 | Tytthaspis_sedecimpunctata_588 | 0.161 |
| Scymnus_haemorrhoidalis_748 | Tytthaspis_sedecimpunctata_588 | 0.161 |
| Coelophora_inaequalis_317 | Tytthaspis_sedecimpunctata_591 | 0.161 |
| Scymnus_haemorrhoidalis_756 | Tytthaspis_sedecimpunctata_591 | 0.161 |
| Hyperaspis_proba_661 | Scymnus_suturalis_843 | 0.161 |
| Hyperaspis_proba_661 | Scymnus_suturalis_845 | 0.161 |
| Scymnus_haemorrhoidalis_752 | Tytthaspis_sedecimpunctata_577 | 0.161 |
| Scymnus_haemorrhoidalis_752 | Tytthaspis_sedecimpunctata_578 | 0.161 |
| Scymnus_haemorrhoidalis_752 | Tytthaspis_sedecimpunctata_579 | 0.161 |
| Scymnus_haemorrhoidalis_752 | Tytthaspis_sedecimpunctata_581 | 0.161 |
| Scymnus_haemorrhoidalis_752 | Tytthaspis_sedecimpunctata_582 | 0.161 |
| Scymnus_haemorrhoidalis_753 | Tytthaspis_sedecimpunctata_583 | 0.161 |
| Scymnus_haemorrhoidalis_756 | Tytthaspis_sedecimpunctata_585 | 0.161 |
| Scymnus_haemorrhoidalis_752 | Tytthaspis_sedecimpunctata_590 | 0.161 |
| Scymnus_haemorrhoidalis_752 | Tytthaspis_sedecimpunctata_592 | 0.161 |
| Scymnus_haemorrhoidalis_753 | Tytthaspis_sedecimpunctata_593 | 0.161 |
| Scymnus_haemorrhoidalis_752 | Tytthaspis_sedecimpunctata_594 | 0.161 |
| Scymnus_haemorrhoidalis_752 | Tytthaspis_sedecimpunctata_595 | 0.161 |
| Hippodamia_tredecimpunctata_457 | Tytthaspis_sedecimpunctata_577 | 0.161 |
| Hippodamia_tredecimpunctata_458 | Tytthaspis_sedecimpunctata_577 | 0.161 |
| Hippodamia_tredecimpunctata_457 | Tytthaspis_sedecimpunctata_578 | 0.161 |
| Hippodamia_tredecimpunctata_458 | Tytthaspis_sedecimpunctata_578 | 0.161 |
| Hippodamia_tredecimpunctata_457 | Tytthaspis_sedecimpunctata_579 | 0.161 |
| Hippodamia_tredecimpunctata_458 | Tytthaspis_sedecimpunctata_579 | 0.161 |
| Scymnus_haemorrhoidalis_752 | Tytthaspis_sedecimpunctata_580 | 0.161 |
| Hippodamia_tredecimpunctata_457 | Tytthaspis_sedecimpunctata_581 | 0.161 |
| Hippodamia_tredecimpunctata_458 | Tytthaspis_sedecimpunctata_581 | 0.161 |
| Hippodamia_tredecimpunctata_457 | Tytthaspis_sedecimpunctata_582 | 0.161 |
| Hippodamia_tredecimpunctata_458 | Tytthaspis_sedecimpunctata_582 | 0.161 |
| Myrrha_octodecimguttata_489 | Tytthaspis_sedecimpunctata_584 | 0.161 |
| Myrrha_octodecimguttata_500 | Tytthaspis_sedecimpunctata_584 | 0.161 |
| Oenopia_conglobata_522 | Tytthaspis_sedecimpunctata_589 | 0.161 |
| Hippodamia_tredecimpunctata_457 | Tytthaspis_sedecimpunctata_590 | 0.161 |
| Hippodamia_tredecimpunctata_458 | Tytthaspis_sedecimpunctata_590 | 0.161 |
| Hippodamia_tredecimpunctata_459 | Tytthaspis_sedecimpunctata_592 | 0.161 |
| Hippodamia_tredecimpunctata_457 | Tytthaspis_sedecimpunctata_593 | 0.161 |
| Hippodamia_tredecimpunctata_458 | Tytthaspis_sedecimpunctata_593 | 0.161 |

| | | |
|---|---|---|
| Hippodamia_tredecimpunctata_457 | Tytthaspis_sedecimpunctata_594 | 0.161 |
| Hippodamia_tredecimpunctata_458 | Tytthaspis_sedecimpunctata_594 | 0.161 |
| Hippodamia_tredecimpunctata_457 | Tytthaspis_sedecimpunctata_595 | 0.161 |
| Hippodamia_tredecimpunctata_458 | Tytthaspis_sedecimpunctata_595 | 0.161 |
| Scymnus_interruptus_767 | Scymnus_sinuanodulus_830 | 0.161 |
| Scymnus_apetzi_692 | Scymnus_suturalis_841 | 0.161 |
| Scymnus_apetzi_692 | Scymnus_suturalis_842 | 0.161 |
| Scymnus_apetzi_692 | Scymnus_suturalis_844 | 0.161 |
| Scymnus_apetzi_692 | Scymnus_suturalis_846 | 0.161 |
| Scymnus_apetzi_692 | Scymnus_suturalis_847 | 0.161 |
| Scymnus_apetzi_692 | Scymnus_suturalis_848 | 0.161 |
| Anatis_ocellata_136 | Tytthaspis_sedecimpunctata_583 | 0.161 |
| Anatis_ocellata_136 | Tytthaspis_sedecimpunctata_585 | 0.161 |
| Psyllobora_vigintiduopunctata_569 | Tytthaspis_sedecimpunctata_589 | 0.161 |
| Anatis_ocellata_136 | Tytthaspis_sedecimpunctata_592 | 0.161 |
| Scymnus_apetzi_692 | Scymnus_suturalis_832 | 0.161 |
| Scymnus_apetzi_692 | Scymnus_suturalis_835 | 0.161 |
| Scymnus_apetzi_692 | Scymnus_suturalis_836 | 0.161 |
| Scymnus_apetzi_692 | Scymnus_suturalis_839 | 0.161 |
| Ceratomegilla_ulkei_222 | Tytthaspis_sedecimpunctata_577 | 0.161 |
| Ceratomegilla_ulkei_223 | Tytthaspis_sedecimpunctata_577 | 0.161 |
| Ceratomegilla_ulkei_222 | Tytthaspis_sedecimpunctata_578 | 0.161 |
| Ceratomegilla_ulkei_223 | Tytthaspis_sedecimpunctata_578 | 0.161 |
| Ceratomegilla_ulkei_222 | Tytthaspis_sedecimpunctata_579 | 0.161 |
| Ceratomegilla_ulkei_223 | Tytthaspis_sedecimpunctata_579 | 0.161 |
| Oenopia_conglobata_522 | Tytthaspis_sedecimpunctata_580 | 0.161 |
| Ceratomegilla_ulkei_222 | Tytthaspis_sedecimpunctata_581 | 0.161 |
| Ceratomegilla_ulkei_223 | Tytthaspis_sedecimpunctata_581 | 0.161 |
| Ceratomegilla_ulkei_222 | Tytthaspis_sedecimpunctata_582 | 0.161 |
| Ceratomegilla_ulkei_223 | Tytthaspis_sedecimpunctata_582 | 0.161 |
| Halyzia_sedecimguttata_345 | Tytthaspis_sedecimpunctata_583 | 0.161 |
| Halyzia_sedecimguttata_350 | Tytthaspis_sedecimpunctata_583 | 0.161 |
| Halyzia_sedecimguttata_351 | Tytthaspis_sedecimpunctata_583 | 0.161 |
| Oenopia_conglobata_522 | Tytthaspis_sedecimpunctata_588 | 0.161 |
| Ceratomegilla_ulkei_222 | Tytthaspis_sedecimpunctata_590 | 0.161 |
| Ceratomegilla_ulkei_223 | Tytthaspis_sedecimpunctata_590 | 0.161 |
| Ceratomegilla_ulkei_222 | Tytthaspis_sedecimpunctata_592 | 0.161 |
| Ceratomegilla_ulkei_223 | Tytthaspis_sedecimpunctata_592 | 0.161 |
| Ceratomegilla_ulkei_222 | Tytthaspis_sedecimpunctata_594 | 0.161 |
| Ceratomegilla_ulkei_223 | Tytthaspis_sedecimpunctata_594 | 0.161 |
| Ceratomegilla_ulkei_222 | Tytthaspis_sedecimpunctata_595 | 0.161 |
| Ceratomegilla_ulkei_223 | Tytthaspis_sedecimpunctata_595 | 0.161 |
| Scymnus_interruptus_760 | Scymnus_sinuanodulus_828 | 0.161 |
| Scymnus_interruptus_765 | Scymnus_sinuanodulus_828 | 0.161 |
| Scymnus_interruptus_768 | Scymnus_sinuanodulus_828 | 0.161 |
| Scymnus_interruptus_762 | Scymnus_sinuanodulus_830 | 0.161 |
| Anatis_ocellata_136 | Tytthaspis_sedecimpunctata_580 | 0.161 |
| Psyllobora_vigintiduopunctata_569 | Tytthaspis_sedecimpunctata_586 | 0.161 |
| Psyllobora_vigintiduopunctata_559 | Tytthaspis_sedecimpunctata_588 | 0.161 |
| Ceratomegilla_ulkei_222 | Tytthaspis_sedecimpunctata_580 | 0.161 |
| Ceratomegilla_ulkei_223 | Tytthaspis_sedecimpunctata_580 | 0.161 |
| Halyzia_sedecimguttata_345 | Tytthaspis_sedecimpunctata_580 | 0.161 |
| Halyzia_sedecimguttata_350 | Tytthaspis_sedecimpunctata_580 | 0.161 |
| Halyzia_sedecimguttata_351 | Tytthaspis_sedecimpunctata_580 | 0.161 |
| Halyzia_sedecimguttata_346 | Tytthaspis_sedecimpunctata_583 | 0.161 |
| Halyzia_sedecimguttata_347 | Tytthaspis_sedecimpunctata_583 | 0.161 |

| | | |
|---|---|---|
| Halyzia_sedecimguttata_348 | Tytthaspis_sedecimpunctata_583 | 0.161 |
| Oenopia_conglobata_522 | Tytthaspis_sedecimpunctata_585 | 0.161 |
| Calvia_quatuordecimguttata_218 | Tytthaspis_sedecimpunctata_589 | 0.161 |
| Oenopia_conglobata_523 | Tytthaspis_sedecimpunctata_593 | 0.161 |
| Scymnus_haemorrhoidalis_747 | Scymnus_suturalis_833 | 0.161 |
| Scymnus_haemorrhoidalis_748 | Scymnus_suturalis_833 | 0.161 |
| Scymnus_haemorrhoidalis_747 | Scymnus_suturalis_838 | 0.161 |
| Scymnus_haemorrhoidalis_748 | Scymnus_suturalis_838 | 0.161 |
| Hyperaspis_oregona_658 | Subcoccinella_vigintiquatuorpunctata_619 | 0.161 |
| Psyllobora_vigintiduopunctata_566 | Tytthaspis_sedecimpunctata_583 | 0.161 |
| Psyllobora_vigintiduopunctata_566 | Tytthaspis_sedecimpunctata_592 | 0.161 |
| Brumus_quadripustulatus_10 | Tytthaspis_sedecimpunctata_583 | 0.161 |
| Psyllobora_vigintiduopunctata_566 | Tytthaspis_sedecimpunctata_580 | 0.161 |
| Scymnus_haemorrhoidalis_756 | Scymnus_suturalis_841 | 0.161 |
| Scymnus_haemorrhoidalis_756 | Scymnus_suturalis_842 | 0.161 |
| Scymnus_haemorrhoidalis_755 | Scymnus_suturalis_843 | 0.161 |
| Scymnus_haemorrhoidalis_756 | Scymnus_suturalis_844 | 0.161 |
| Scymnus_haemorrhoidalis_755 | Scymnus_suturalis_845 | 0.161 |
| Scymnus_haemorrhoidalis_756 | Scymnus_suturalis_846 | 0.161 |
| Scymnus_haemorrhoidalis_756 | Scymnus_suturalis_847 | 0.161 |
| Scymnus_haemorrhoidalis_756 | Scymnus_suturalis_848 | 0.161 |
| Hippodamia_parenthesis_439 | Tytthaspis_sedecimpunctata_577 | 0.161 |
| Hippodamia_parenthesis_439 | Tytthaspis_sedecimpunctata_578 | 0.161 |
| Hippodamia_parenthesis_439 | Tytthaspis_sedecimpunctata_579 | 0.161 |
| Hippodamia_parenthesis_439 | Tytthaspis_sedecimpunctata_581 | 0.161 |
| Hippodamia_parenthesis_439 | Tytthaspis_sedecimpunctata_582 | 0.161 |
| Calvia_quatuordecimguttata_207 | Tytthaspis_sedecimpunctata_586 | 0.161 |
| Calvia_decemguttata_195 | Tytthaspis_sedecimpunctata_587 | 0.161 |
| Calvia_decemguttata_199 | Tytthaspis_sedecimpunctata_587 | 0.161 |
| Calvia_decemguttata_200 | Tytthaspis_sedecimpunctata_587 | 0.161 |
| Calvia_quatuordecimguttata_207 | Tytthaspis_sedecimpunctata_587 | 0.161 |
| Calvia_quatuordecimguttata_218 | Tytthaspis_sedecimpunctata_587 | 0.161 |
| Hippodamia_parenthesis_432 | Tytthaspis_sedecimpunctata_588 | 0.161 |
| Hippodamia_parenthesis_434 | Tytthaspis_sedecimpunctata_588 | 0.161 |
| Hippodamia_parenthesis_435 | Tytthaspis_sedecimpunctata_588 | 0.161 |
| Hippodamia_parenthesis_437 | Tytthaspis_sedecimpunctata_588 | 0.161 |
| Hippodamia_parenthesis_438 | Tytthaspis_sedecimpunctata_588 | 0.161 |
| Hippodamia_parenthesis_441 | Tytthaspis_sedecimpunctata_588 | 0.161 |
| Hippodamia_parenthesis_442 | Tytthaspis_sedecimpunctata_588 | 0.161 |
| Calvia_quatuordecimguttata_204 | Tytthaspis_sedecimpunctata_589 | 0.161 |
| Calvia_quatuordecimguttata_208 | Tytthaspis_sedecimpunctata_589 | 0.161 |
| Calvia_quatuordecimguttata_217 | Tytthaspis_sedecimpunctata_589 | 0.161 |
| Hippodamia_parenthesis_439 | Tytthaspis_sedecimpunctata_590 | 0.161 |
| Hippodamia_parenthesis_439 | Tytthaspis_sedecimpunctata_592 | 0.161 |
| Hippodamia_parenthesis_439 | Tytthaspis_sedecimpunctata_594 | 0.161 |
| Hippodamia_parenthesis_439 | Tytthaspis_sedecimpunctata_595 | 0.161 |
| Scymnus_haemorrhoidalis_756 | Scymnus_suturalis_832 | 0.161 |
| Scymnus_haemorrhoidalis_747 | Scymnus_suturalis_834 | 0.161 |
| Scymnus_haemorrhoidalis_748 | Scymnus_suturalis_834 | 0.161 |
| Scymnus_haemorrhoidalis_756 | Scymnus_suturalis_835 | 0.161 |
| Scymnus_haemorrhoidalis_756 | Scymnus_suturalis_836 | 0.161 |
| Scymnus_haemorrhoidalis_756 | Scymnus_suturalis_837 | 0.161 |
| Scymnus_haemorrhoidalis_756 | Scymnus_suturalis_839 | 0.161 |
| Brumus_quadripustulatus_10 | Tytthaspis_sedecimpunctata_580 | 0.161 |
| Scymnus_haemorrhoidalis_750 | Scymnus_suturalis_843 | 0.161 |
| Scymnus_haemorrhoidalis_750 | Scymnus_suturalis_845 | 0.161 |

| | | |
|---|---|---|
| Hippodamia_parenthesis_433 | Tytthaspis_sedecimpunctata_577 | 0.161 |
| Hippodamia_parenthesis_440 | Tytthaspis_sedecimpunctata_577 | 0.161 |
| Hippodamia_parenthesis_433 | Tytthaspis_sedecimpunctata_578 | 0.161 |
| Hippodamia_parenthesis_440 | Tytthaspis_sedecimpunctata_578 | 0.161 |
| Hippodamia_parenthesis_433 | Tytthaspis_sedecimpunctata_579 | 0.161 |
| Hippodamia_parenthesis_440 | Tytthaspis_sedecimpunctata_579 | 0.161 |
| Macronaemia_episcopalis_477 | Tytthaspis_sedecimpunctata_580 | 0.161 |
| Hippodamia_parenthesis_433 | Tytthaspis_sedecimpunctata_581 | 0.161 |
| Hippodamia_parenthesis_440 | Tytthaspis_sedecimpunctata_581 | 0.161 |
| Hippodamia_parenthesis_433 | Tytthaspis_sedecimpunctata_582 | 0.161 |
| Hippodamia_parenthesis_440 | Tytthaspis_sedecimpunctata_582 | 0.161 |
| Coccinula_quatuordecimpustulata_306 | Tytthaspis_sedecimpunctata_583 | 0.161 |
| Hippodamia_parenthesis_432 | Tytthaspis_sedecimpunctata_583 | 0.161 |
| Hippodamia_parenthesis_434 | Tytthaspis_sedecimpunctata_583 | 0.161 |
| Hippodamia_parenthesis_435 | Tytthaspis_sedecimpunctata_583 | 0.161 |
| Hippodamia_parenthesis_437 | Tytthaspis_sedecimpunctata_583 | 0.161 |
| Hippodamia_parenthesis_438 | Tytthaspis_sedecimpunctata_583 | 0.161 |
| Hippodamia_parenthesis_441 | Tytthaspis_sedecimpunctata_583 | 0.161 |
| Hippodamia_parenthesis_442 | Tytthaspis_sedecimpunctata_583 | 0.161 |
| Hippodamia_parenthesis_432 | Tytthaspis_sedecimpunctata_585 | 0.161 |
| Hippodamia_parenthesis_434 | Tytthaspis_sedecimpunctata_585 | 0.161 |
| Hippodamia_parenthesis_435 | Tytthaspis_sedecimpunctata_585 | 0.161 |
| Hippodamia_parenthesis_437 | Tytthaspis_sedecimpunctata_585 | 0.161 |
| Hippodamia_parenthesis_438 | Tytthaspis_sedecimpunctata_585 | 0.161 |
| Hippodamia_parenthesis_441 | Tytthaspis_sedecimpunctata_585 | 0.161 |
| Hippodamia_parenthesis_442 | Tytthaspis_sedecimpunctata_585 | 0.161 |
| Coccinula_quatuordecimpustulata_292 | Tytthaspis_sedecimpunctata_586 | 0.161 |
| Coccinula_quatuordecimpustulata_293 | Tytthaspis_sedecimpunctata_586 | 0.161 |
| Coccinula_quatuordecimpustulata_307 | Tytthaspis_sedecimpunctata_586 | 0.161 |
| Calvia_quatuordecimguttata_204 | Tytthaspis_sedecimpunctata_587 | 0.161 |
| Calvia_quatuordecimguttata_208 | Tytthaspis_sedecimpunctata_587 | 0.161 |
| Calvia_quatuordecimguttata_217 | Tytthaspis_sedecimpunctata_587 | 0.161 |
| Coleomegilla_maculata_327 | Tytthaspis_sedecimpunctata_587 | 0.161 |
| Macronaemia_episcopalis_477 | Tytthaspis_sedecimpunctata_587 | 0.161 |
| Coccinula_quatuordecimpustulata_305 | Tytthaspis_sedecimpunctata_588 | 0.161 |
| Hippodamia_parenthesis_433 | Tytthaspis_sedecimpunctata_590 | 0.161 |
| Hippodamia_parenthesis_440 | Tytthaspis_sedecimpunctata_590 | 0.161 |
| Hippodamia_parenthesis_433 | Tytthaspis_sedecimpunctata_592 | 0.161 |
| Hippodamia_parenthesis_440 | Tytthaspis_sedecimpunctata_592 | 0.161 |
| Hippodamia_parenthesis_432 | Tytthaspis_sedecimpunctata_593 | 0.161 |
| Hippodamia_parenthesis_434 | Tytthaspis_sedecimpunctata_593 | 0.161 |
| Hippodamia_parenthesis_435 | Tytthaspis_sedecimpunctata_593 | 0.161 |
| Hippodamia_parenthesis_437 | Tytthaspis_sedecimpunctata_593 | 0.161 |
| Hippodamia_parenthesis_438 | Tytthaspis_sedecimpunctata_593 | 0.161 |
| Hippodamia_parenthesis_441 | Tytthaspis_sedecimpunctata_593 | 0.161 |
| Hippodamia_parenthesis_442 | Tytthaspis_sedecimpunctata_593 | 0.161 |
| Hippodamia_parenthesis_433 | Tytthaspis_sedecimpunctata_594 | 0.161 |
| Hippodamia_parenthesis_440 | Tytthaspis_sedecimpunctata_594 | 0.161 |
| Hippodamia_parenthesis_433 | Tytthaspis_sedecimpunctata_595 | 0.161 |
| Hippodamia_parenthesis_440 | Tytthaspis_sedecimpunctata_595 | 0.161 |
| Scymnus_haemorrhoidalis_752 | Scymnus_suturalis_833 | 0.161 |
| Scymnus_haemorrhoidalis_752 | Scymnus_suturalis_838 | 0.161 |
| Scymnus_haemorrhoidalis_756 | Scymnus_suturalis_840 | 0.161 |
| Scymnus_nigrinus_784 | Scymnus_sinuanodulus_831 | 0.161 |
| Coccinula_quatuordecimpustulata_294 | Tytthaspis_sedecimpunctata_577 | 0.161 |
| Coccinula_quatuordecimpustulata_295 | Tytthaspis_sedecimpunctata_577 | 0.161 |

| | | |
|---|---|---|
| Coccinula_quatuordecimpustulata_308 | Tytthaspis_sedecimpunctata_577 | 0.161 |
| Coccinula_quatuordecimpustulata_294 | Tytthaspis_sedecimpunctata_578 | 0.161 |
| Coccinula_quatuordecimpustulata_295 | Tytthaspis_sedecimpunctata_578 | 0.161 |
| Coccinula_quatuordecimpustulata_308 | Tytthaspis_sedecimpunctata_578 | 0.161 |
| Coccinula_quatuordecimpustulata_294 | Tytthaspis_sedecimpunctata_579 | 0.161 |
| Coccinula_quatuordecimpustulata_295 | Tytthaspis_sedecimpunctata_579 | 0.161 |
| Coccinula_quatuordecimpustulata_308 | Tytthaspis_sedecimpunctata_579 | 0.161 |
| Coccinula_quatuordecimpustulata_306 | Tytthaspis_sedecimpunctata_580 | 0.161 |
| Coccinula_quatuordecimpustulata_294 | Tytthaspis_sedecimpunctata_581 | 0.161 |
| Coccinula_quatuordecimpustulata_295 | Tytthaspis_sedecimpunctata_581 | 0.161 |
| Coccinula_quatuordecimpustulata_308 | Tytthaspis_sedecimpunctata_581 | 0.161 |
| Coccinula_quatuordecimpustulata_294 | Tytthaspis_sedecimpunctata_582 | 0.161 |
| Coccinula_quatuordecimpustulata_295 | Tytthaspis_sedecimpunctata_582 | 0.161 |
| Coccinula_quatuordecimpustulata_308 | Tytthaspis_sedecimpunctata_582 | 0.161 |
| Coccinula_quatuordecimpustulata_298 | Tytthaspis_sedecimpunctata_583 | 0.161 |
| Coccinula_quatuordecimpustulata_299 | Tytthaspis_sedecimpunctata_583 | 0.161 |
| Coccinula_quatuordecimpustulata_301 | Tytthaspis_sedecimpunctata_583 | 0.161 |
| Coccinula_quatuordecimpustulata_302 | Tytthaspis_sedecimpunctata_583 | 0.161 |
| Coccinula_quatuordecimpustulata_303 | Tytthaspis_sedecimpunctata_583 | 0.161 |
| Coccinula_quatuordecimpustulata_309 | Tytthaspis_sedecimpunctata_583 | 0.161 |
| Coccinula_quatuordecimpustulata_294 | Tytthaspis_sedecimpunctata_585 | 0.161 |
| Coccinula_quatuordecimpustulata_295 | Tytthaspis_sedecimpunctata_585 | 0.161 |
| Coccinula_quatuordecimpustulata_308 | Tytthaspis_sedecimpunctata_585 | 0.161 |
| Calvia_quatuordecimguttata_205 | Tytthaspis_sedecimpunctata_588 | 0.161 |
| Coccinula_quatuordecimpustulata_296 | Tytthaspis_sedecimpunctata_588 | 0.161 |
| Coleomegilla_maculata_327 | Tytthaspis_sedecimpunctata_589 | 0.161 |
| Coccinula_quatuordecimpustulata_294 | Tytthaspis_sedecimpunctata_590 | 0.161 |
| Coccinula_quatuordecimpustulata_295 | Tytthaspis_sedecimpunctata_590 | 0.161 |
| Coccinula_quatuordecimpustulata_308 | Tytthaspis_sedecimpunctata_590 | 0.161 |
| Calvia_quatuordecimguttata_205 | Tytthaspis_sedecimpunctata_591 | 0.161 |
| Coccinula_quatuordecimpustulata_294 | Tytthaspis_sedecimpunctata_592 | 0.161 |
| Coccinula_quatuordecimpustulata_295 | Tytthaspis_sedecimpunctata_592 | 0.161 |
| Coccinula_quatuordecimpustulata_308 | Tytthaspis_sedecimpunctata_592 | 0.161 |
| Coccinula_quatuordecimpustulata_294 | Tytthaspis_sedecimpunctata_593 | 0.161 |
| Coccinula_quatuordecimpustulata_295 | Tytthaspis_sedecimpunctata_593 | 0.161 |
| Coccinula_quatuordecimpustulata_305 | Tytthaspis_sedecimpunctata_593 | 0.161 |
| Coccinula_quatuordecimpustulata_308 | Tytthaspis_sedecimpunctata_593 | 0.161 |
| Coccinula_quatuordecimpustulata_294 | Tytthaspis_sedecimpunctata_594 | 0.161 |
| Coccinula_quatuordecimpustulata_295 | Tytthaspis_sedecimpunctata_594 | 0.161 |
| Coccinula_quatuordecimpustulata_308 | Tytthaspis_sedecimpunctata_594 | 0.161 |
| Coccinula_quatuordecimpustulata_294 | Tytthaspis_sedecimpunctata_595 | 0.161 |
| Coccinula_quatuordecimpustulata_295 | Tytthaspis_sedecimpunctata_595 | 0.161 |
| Coccinula_quatuordecimpustulata_308 | Tytthaspis_sedecimpunctata_595 | 0.161 |
| Scymnus_camptodromus_707 | Scymnus_rubromaculatus_822 | 0.161 |
| Scymnus_camptodromus_708 | Scymnus_rubromaculatus_822 | 0.161 |
| Scymnus_camptodromus_709 | Scymnus_rubromaculatus_822 | 0.161 |
| Scymnus_camptodromus_710 | Scymnus_rubromaculatus_822 | 0.161 |
| Scymnus_camptodromus_719 | Scymnus_rubromaculatus_822 | 0.161 |
| Scymnus_camptodromus_707 | Scymnus_rubromaculatus_823 | 0.161 |
| Scymnus_camptodromus_708 | Scymnus_rubromaculatus_823 | 0.161 |
| Scymnus_camptodromus_709 | Scymnus_rubromaculatus_823 | 0.161 |
| Scymnus_camptodromus_710 | Scymnus_rubromaculatus_823 | 0.161 |
| Scymnus_camptodromus_719 | Scymnus_rubromaculatus_823 | 0.161 |
| Scymnus_camptodromus_707 | Scymnus_rubromaculatus_824 | 0.161 |
| Scymnus_camptodromus_708 | Scymnus_rubromaculatus_824 | 0.161 |
| Scymnus_camptodromus_709 | Scymnus_rubromaculatus_824 | 0.161 |

| | | |
|---|---|---|
| Scymnus_camptodromus_710 | Scymnus_rubromaculatus_824 | 0.161 |
| Scymnus_camptodromus_719 | Scymnus_rubromaculatus_824 | 0.161 |
| Scymnus_camptodromus_707 | Scymnus_rubromaculatus_825 | 0.161 |
| Scymnus_camptodromus_708 | Scymnus_rubromaculatus_825 | 0.161 |
| Scymnus_camptodromus_709 | Scymnus_rubromaculatus_825 | 0.161 |
| Scymnus_camptodromus_710 | Scymnus_rubromaculatus_825 | 0.161 |
| Scymnus_camptodromus_719 | Scymnus_rubromaculatus_825 | 0.161 |
| Scymnus_camptodromus_707 | Scymnus_rubromaculatus_826 | 0.161 |
| Scymnus_camptodromus_708 | Scymnus_rubromaculatus_826 | 0.161 |
| Scymnus_camptodromus_709 | Scymnus_rubromaculatus_826 | 0.161 |
| Scymnus_camptodromus_710 | Scymnus_rubromaculatus_826 | 0.161 |
| Scymnus_camptodromus_719 | Scymnus_rubromaculatus_826 | 0.161 |
| Scymnus_camptodromus_707 | Scymnus_rubromaculatus_827 | 0.161 |
| Scymnus_camptodromus_708 | Scymnus_rubromaculatus_827 | 0.161 |
| Scymnus_camptodromus_709 | Scymnus_rubromaculatus_827 | 0.161 |
| Scymnus_camptodromus_710 | Scymnus_rubromaculatus_827 | 0.161 |
| Scymnus_camptodromus_719 | Scymnus_rubromaculatus_827 | 0.161 |
| Scymnus_haemorrhoidalis_752 | Scymnus_suturalis_834 | 0.161 |
| Oenopia_conglobata_524 | Tytthaspis_sedecimpunctata_583 | 0.161 |
| Hippodamia_parenthesis_436 | Tytthaspis_sedecimpunctata_589 | 0.161 |
| Oenopia_conglobata_524 | Tytthaspis_sedecimpunctata_592 | 0.161 |
| Psyllobora_vigintiduopunctata_558 | Tytthaspis_sedecimpunctata_583 | 0.161 |
| Psyllobora_vigintiduopunctata_568 | Tytthaspis_sedecimpunctata_583 | 0.161 |
| Psyllobora_vigintiduopunctata_558 | Tytthaspis_sedecimpunctata_585 | 0.161 |
| Psyllobora_vigintiduopunctata_568 | Tytthaspis_sedecimpunctata_585 | 0.161 |
| Psyllobora_vigintiduopunctata_558 | Tytthaspis_sedecimpunctata_592 | 0.161 |
| Psyllobora_vigintiduopunctata_568 | Tytthaspis_sedecimpunctata_592 | 0.161 |
| Coccinula_quatuordecimpustulata_296 | Tytthaspis_sedecimpunctata_577 | 0.161 |
| Coccinula_quatuordecimpustulata_296 | Tytthaspis_sedecimpunctata_578 | 0.161 |
| Coccinula_quatuordecimpustulata_296 | Tytthaspis_sedecimpunctata_579 | 0.161 |
| Coccinula_quatuordecimpustulata_298 | Tytthaspis_sedecimpunctata_580 | 0.161 |
| Coccinula_quatuordecimpustulata_299 | Tytthaspis_sedecimpunctata_580 | 0.161 |
| Coccinula_quatuordecimpustulata_301 | Tytthaspis_sedecimpunctata_580 | 0.161 |
| Coccinula_quatuordecimpustulata_302 | Tytthaspis_sedecimpunctata_580 | 0.161 |
| Coccinula_quatuordecimpustulata_303 | Tytthaspis_sedecimpunctata_580 | 0.161 |
| Coccinula_quatuordecimpustulata_309 | Tytthaspis_sedecimpunctata_580 | 0.161 |
| Coccinula_quatuordecimpustulata_296 | Tytthaspis_sedecimpunctata_581 | 0.161 |
| Coccinula_quatuordecimpustulata_296 | Tytthaspis_sedecimpunctata_582 | 0.161 |
| Coccinula_quatuordecimpustulata_294 | Tytthaspis_sedecimpunctata_584 | 0.161 |
| Coccinula_quatuordecimpustulata_295 | Tytthaspis_sedecimpunctata_584 | 0.161 |
| Coccinula_quatuordecimpustulata_308 | Tytthaspis_sedecimpunctata_584 | 0.161 |
| Coccinula_quatuordecimpustulata_296 | Tytthaspis_sedecimpunctata_585 | 0.161 |
| Coccinula_quatuordecimpustulata_296 | Tytthaspis_sedecimpunctata_590 | 0.161 |
| Coccinula_quatuordecimpustulata_296 | Tytthaspis_sedecimpunctata_592 | 0.161 |
| Coccinula_quatuordecimpustulata_296 | Tytthaspis_sedecimpunctata_593 | 0.161 |
| Coccinula_quatuordecimpustulata_296 | Tytthaspis_sedecimpunctata_594 | 0.161 |
| Coccinula_quatuordecimpustulata_296 | Tytthaspis_sedecimpunctata_595 | 0.161 |
| Psyllobora_vigintiduopunctata_558 | Tytthaspis_sedecimpunctata_580 | 0.161 |
| Psyllobora_vigintiduopunctata_568 | Tytthaspis_sedecimpunctata_580 | 0.161 |
| Hippodamia_parenthesis_436 | Tytthaspis_sedecimpunctata_586 | 0.161 |
| Oenopia_conglobata_524 | Tytthaspis_sedecimpunctata_591 | 0.161 |
| Macronaemia_episcopalis_478 | Tytthaspis_sedecimpunctata_591 | 0.161 |
| Coccinula_quatuordecimpustulata_296 | Tytthaspis_sedecimpunctata_584 | 0.161 |
| Scymnus_frontalis_745 | Scymnus_rubromaculatus_822 | 0.162 |
| Scymnus_frontalis_745 | Scymnus_rubromaculatus_823 | 0.162 |
| Scymnus_frontalis_745 | Scymnus_rubromaculatus_824 | 0.162 |

| | | |
|---|---|---|
| Scymnus_frontalis_745 | Scymnus_rubromaculatus_825 | 0.162 |
| Scymnus_frontalis_745 | Scymnus_rubromaculatus_826 | 0.162 |
| Scymnus_frontalis_745 | Scymnus_rubromaculatus_827 | 0.162 |
| Scymnus_apetzi_695 | Scymnus_suturalis_838 | 0.162 |
| Scymnus_apetzi_695 | Scymnus_suturalis_840 | 0.162 |
| Scymnus_nigrinus_785 | Scymnus_sinuanodulus_829 | 0.162 |
| Scymnus_nigrinus_786 | Scymnus_sinuanodulus_829 | 0.162 |
| Myrrha_octodecimguttata_485 | Vibidia_duodecimguttata_603 | 0.162 |
| Myrrha_octodecimguttata_486 | Vibidia_duodecimguttata_603 | 0.162 |
| Myrrha_octodecimguttata_487 | Vibidia_duodecimguttata_603 | 0.162 |
| Myrrha_octodecimguttata_490 | Vibidia_duodecimguttata_603 | 0.162 |
| Myrrha_octodecimguttata_494 | Vibidia_duodecimguttata_603 | 0.162 |
| Myrrha_octodecimguttata_496 | Vibidia_duodecimguttata_603 | 0.162 |
| Myrrha_octodecimguttata_497 | Vibidia_duodecimguttata_603 | 0.162 |
| Myrrha_octodecimguttata_501 | Vibidia_duodecimguttata_603 | 0.162 |
| Myrrha_octodecimguttata_502 | Vibidia_duodecimguttata_603 | 0.162 |
| Scymnus_apetzi_695 | Scymnus_suturalis_834 | 0.162 |
| Brumus_quadripustulatus_5 | Tytthaspis_sedecimpunctata_584 | 0.162 |
| Myrrha_octodecimguttata_495 | Vibidia_duodecimguttata_603 | 0.162 |
| Anisosticta_novemdecimpunctata_159 | Tytthaspis_sedecimpunctata_591 | 0.162 |
| Anisosticta_novemdecimpunctata_163 | Tytthaspis_sedecimpunctata_591 | 0.162 |
| Anisosticta_novemdecimpunctata_171 | Tytthaspis_sedecimpunctata_591 | 0.162 |
| Anatis_labiculata_126 | Vibidia_duodecimguttata_596 | 0.162 |
| Anatis_labiculata_126 | Vibidia_duodecimguttata_598 | 0.162 |
| Anatis_labiculata_126 | Vibidia_duodecimguttata_599 | 0.162 |
| Anatis_labiculata_126 | Vibidia_duodecimguttata_601 | 0.162 |
| Anatis_labiculata_126 | Vibidia_duodecimguttata_602 | 0.162 |
| Anatis_labiculata_126 | Vibidia_duodecimguttata_603 | 0.162 |
| Psyllobora_vigintimaculata_574 | Vibidia_duodecimguttata_603 | 0.162 |
| Hyperaspis_proba_661 | Tytthaspis_sedecimpunctata_586 | 0.162 |
| Chilocorus_bipustulatus_23 | Tytthaspis_sedecimpunctata_586 | 0.162 |
| Chilocorus_bipustulatus_23 | Tytthaspis_sedecimpunctata_588 | 0.162 |
| Chilocorus_bipustulatus_18 | Tytthaspis_sedecimpunctata_589 | 0.162 |
| Chilocorus_bipustulatus_20 | Tytthaspis_sedecimpunctata_589 | 0.162 |
| Chilocorus_bipustulatus_23 | Tytthaspis_sedecimpunctata_577 | 0.162 |
| Chilocorus_bipustulatus_23 | Tytthaspis_sedecimpunctata_578 | 0.162 |
| Chilocorus_bipustulatus_23 | Tytthaspis_sedecimpunctata_579 | 0.162 |
| Chilocorus_bipustulatus_23 | Tytthaspis_sedecimpunctata_581 | 0.162 |
| Chilocorus_bipustulatus_23 | Tytthaspis_sedecimpunctata_582 | 0.162 |
| Brumus_quadripustulatus_11 | Tytthaspis_sedecimpunctata_583 | 0.162 |
| Brumus_quadripustulatus_13 | Tytthaspis_sedecimpunctata_583 | 0.162 |
| Brumus_quadripustulatus_14 | Tytthaspis_sedecimpunctata_583 | 0.162 |
| Brumus_quadripustulatus_17 | Tytthaspis_sedecimpunctata_583 | 0.162 |
| Brumus_quadripustulatus_3 | Tytthaspis_sedecimpunctata_583 | 0.162 |
| Brumus_quadripustulatus_4 | Tytthaspis_sedecimpunctata_583 | 0.162 |
| Brumus_quadripustulatus_7 | Tytthaspis_sedecimpunctata_583 | 0.162 |
| Chilocorus_bipustulatus_24 | Tytthaspis_sedecimpunctata_586 | 0.162 |
| Chilocorus_bipustulatus_24 | Tytthaspis_sedecimpunctata_588 | 0.162 |
| Chilocorus_bipustulatus_23 | Tytthaspis_sedecimpunctata_590 | 0.162 |
| Chilocorus_bipustulatus_23 | Tytthaspis_sedecimpunctata_593 | 0.162 |
| Chilocorus_bipustulatus_23 | Tytthaspis_sedecimpunctata_594 | 0.162 |
| Chilocorus_bipustulatus_23 | Tytthaspis_sedecimpunctata_595 | 0.162 |
| Chilocorus_bipustulatus_24 | Tytthaspis_sedecimpunctata_577 | 0.162 |
| Chilocorus_bipustulatus_24 | Tytthaspis_sedecimpunctata_578 | 0.162 |
| Chilocorus_bipustulatus_24 | Tytthaspis_sedecimpunctata_579 | 0.162 |
| Brumus_quadripustulatus_11 | Tytthaspis_sedecimpunctata_580 | 0.162 |

| | | |
|---|---|---|
| Brumus_quadripustulatus_13 | Tytthaspis_sedecimpunctata_580 | 0.162 |
| Brumus_quadripustulatus_14 | Tytthaspis_sedecimpunctata_580 | 0.162 |
| Brumus_quadripustulatus_17 | Tytthaspis_sedecimpunctata_580 | 0.162 |
| Brumus_quadripustulatus_4 | Tytthaspis_sedecimpunctata_580 | 0.162 |
| Brumus_quadripustulatus_7 | Tytthaspis_sedecimpunctata_580 | 0.162 |
| Chilocorus_bipustulatus_24 | Tytthaspis_sedecimpunctata_581 | 0.162 |
| Chilocorus_bipustulatus_24 | Tytthaspis_sedecimpunctata_582 | 0.162 |
| Chilocorus_bipustulatus_19 | Tytthaspis_sedecimpunctata_583 | 0.162 |
| Chilocorus_bipustulatus_22 | Tytthaspis_sedecimpunctata_583 | 0.162 |
| Chilocorus_bipustulatus_18 | Tytthaspis_sedecimpunctata_585 | 0.162 |
| Chilocorus_bipustulatus_20 | Tytthaspis_sedecimpunctata_585 | 0.162 |
| Chilocorus_bipustulatus_24 | Tytthaspis_sedecimpunctata_590 | 0.162 |
| Chilocorus_bipustulatus_19 | Tytthaspis_sedecimpunctata_592 | 0.162 |
| Chilocorus_bipustulatus_22 | Tytthaspis_sedecimpunctata_592 | 0.162 |
| Chilocorus_bipustulatus_24 | Tytthaspis_sedecimpunctata_593 | 0.162 |
| Chilocorus_bipustulatus_24 | Tytthaspis_sedecimpunctata_594 | 0.162 |
| Chilocorus_bipustulatus_24 | Tytthaspis_sedecimpunctata_595 | 0.162 |
| Chilocorus_stigma_45 | Tytthaspis_sedecimpunctata_577 | 0.162 |
| Chilocorus_stigma_45 | Tytthaspis_sedecimpunctata_578 | 0.162 |
| Chilocorus_stigma_45 | Tytthaspis_sedecimpunctata_579 | 0.162 |
| Chilocorus_bipustulatus_19 | Tytthaspis_sedecimpunctata_580 | 0.162 |
| Chilocorus_bipustulatus_22 | Tytthaspis_sedecimpunctata_580 | 0.162 |
| Chilocorus_stigma_45 | Tytthaspis_sedecimpunctata_581 | 0.162 |
| Chilocorus_stigma_45 | Tytthaspis_sedecimpunctata_582 | 0.162 |
| Chilocorus_bipustulatus_18 | Tytthaspis_sedecimpunctata_584 | 0.162 |
| Chilocorus_bipustulatus_20 | Tytthaspis_sedecimpunctata_584 | 0.162 |
| Chilocorus_stigma_45 | Tytthaspis_sedecimpunctata_590 | 0.162 |
| Chilocorus_stigma_45 | Tytthaspis_sedecimpunctata_592 | 0.162 |
| Chilocorus_stigma_45 | Tytthaspis_sedecimpunctata_593 | 0.162 |
| Chilocorus_stigma_45 | Tytthaspis_sedecimpunctata_594 | 0.162 |
| Chilocorus_stigma_45 | Tytthaspis_sedecimpunctata_595 | 0.162 |
| Coccinella_transversoguttata_267 | Tytthaspis_sedecimpunctata_577 | 0.162 |
| Coccinella_transversoguttata_268 | Tytthaspis_sedecimpunctata_577 | 0.162 |
| Coccinella_transversoguttata_269 | Tytthaspis_sedecimpunctata_577 | 0.162 |
| Coccinella_transversoguttata_270 | Tytthaspis_sedecimpunctata_577 | 0.162 |
| Coccinella_transversoguttata_271 | Tytthaspis_sedecimpunctata_577 | 0.162 |
| Coccinella_transversoguttata_267 | Tytthaspis_sedecimpunctata_578 | 0.162 |
| Coccinella_transversoguttata_268 | Tytthaspis_sedecimpunctata_578 | 0.162 |
| Coccinella_transversoguttata_269 | Tytthaspis_sedecimpunctata_578 | 0.162 |
| Coccinella_transversoguttata_270 | Tytthaspis_sedecimpunctata_578 | 0.162 |
| Coccinella_transversoguttata_271 | Tytthaspis_sedecimpunctata_578 | 0.162 |
| Coccinella_transversoguttata_267 | Tytthaspis_sedecimpunctata_579 | 0.162 |
| Coccinella_transversoguttata_268 | Tytthaspis_sedecimpunctata_579 | 0.162 |
| Coccinella_transversoguttata_269 | Tytthaspis_sedecimpunctata_579 | 0.162 |
| Coccinella_transversoguttata_270 | Tytthaspis_sedecimpunctata_579 | 0.162 |
| Coccinella_transversoguttata_271 | Tytthaspis_sedecimpunctata_579 | 0.162 |
| Coccinella_transversoguttata_267 | Tytthaspis_sedecimpunctata_581 | 0.162 |
| Coccinella_transversoguttata_268 | Tytthaspis_sedecimpunctata_581 | 0.162 |
| Coccinella_transversoguttata_269 | Tytthaspis_sedecimpunctata_581 | 0.162 |
| Coccinella_transversoguttata_270 | Tytthaspis_sedecimpunctata_581 | 0.162 |
| Coccinella_transversoguttata_271 | Tytthaspis_sedecimpunctata_581 | 0.162 |
| Coccinella_transversoguttata_267 | Tytthaspis_sedecimpunctata_582 | 0.162 |
| Coccinella_transversoguttata_268 | Tytthaspis_sedecimpunctata_582 | 0.162 |
| Coccinella_transversoguttata_269 | Tytthaspis_sedecimpunctata_582 | 0.162 |
| Coccinella_transversoguttata_270 | Tytthaspis_sedecimpunctata_582 | 0.162 |
| Coccinella_transversoguttata_271 | Tytthaspis_sedecimpunctata_582 | 0.162 |

| | | |
|---|---|---|
| Coccinella_transversoguttata_267 | Tytthaspis_sedecimpunctata_590 | 0.162 |
| Coccinella_transversoguttata_268 | Tytthaspis_sedecimpunctata_590 | 0.162 |
| Coccinella_transversoguttata_269 | Tytthaspis_sedecimpunctata_590 | 0.162 |
| Coccinella_transversoguttata_270 | Tytthaspis_sedecimpunctata_590 | 0.162 |
| Coccinella_transversoguttata_271 | Tytthaspis_sedecimpunctata_590 | 0.162 |
| Coccinella_transversoguttata_267 | Tytthaspis_sedecimpunctata_592 | 0.162 |
| Coccinella_transversoguttata_268 | Tytthaspis_sedecimpunctata_592 | 0.162 |
| Coccinella_transversoguttata_269 | Tytthaspis_sedecimpunctata_592 | 0.162 |
| Coccinella_transversoguttata_270 | Tytthaspis_sedecimpunctata_592 | 0.162 |
| Coccinella_transversoguttata_271 | Tytthaspis_sedecimpunctata_592 | 0.162 |
| Coccinella_transversoguttata_267 | Tytthaspis_sedecimpunctata_594 | 0.162 |
| Coccinella_transversoguttata_268 | Tytthaspis_sedecimpunctata_594 | 0.162 |
| Coccinella_transversoguttata_269 | Tytthaspis_sedecimpunctata_594 | 0.162 |
| Coccinella_transversoguttata_270 | Tytthaspis_sedecimpunctata_594 | 0.162 |
| Coccinella_transversoguttata_271 | Tytthaspis_sedecimpunctata_594 | 0.162 |
| Coccinella_transversoguttata_267 | Tytthaspis_sedecimpunctata_595 | 0.162 |
| Coccinella_transversoguttata_268 | Tytthaspis_sedecimpunctata_595 | 0.162 |
| Coccinella_transversoguttata_269 | Tytthaspis_sedecimpunctata_595 | 0.162 |
| Coccinella_transversoguttata_270 | Tytthaspis_sedecimpunctata_595 | 0.162 |
| Coccinella_transversoguttata_271 | Tytthaspis_sedecimpunctata_595 | 0.162 |
| Harmonia_axyridis_355 | Tytthaspis_sedecimpunctata_583 | 0.162 |
| Harmonia_axyridis_355 | Tytthaspis_sedecimpunctata_592 | 0.162 |
| Anatis_mali_128 | Tytthaspis_sedecimpunctata_577 | 0.162 |
| Anatis_mali_129 | Tytthaspis_sedecimpunctata_577 | 0.162 |
| Anatis_mali_130 | Tytthaspis_sedecimpunctata_577 | 0.162 |
| Anatis_mali_131 | Tytthaspis_sedecimpunctata_577 | 0.162 |
| Anatis_mali_128 | Tytthaspis_sedecimpunctata_578 | 0.162 |
| Anatis_mali_129 | Tytthaspis_sedecimpunctata_578 | 0.162 |
| Anatis_mali_130 | Tytthaspis_sedecimpunctata_578 | 0.162 |
| Anatis_mali_131 | Tytthaspis_sedecimpunctata_578 | 0.162 |
| Anatis_mali_128 | Tytthaspis_sedecimpunctata_579 | 0.162 |
| Anatis_mali_129 | Tytthaspis_sedecimpunctata_579 | 0.162 |
| Anatis_mali_130 | Tytthaspis_sedecimpunctata_579 | 0.162 |
| Anatis_mali_131 | Tytthaspis_sedecimpunctata_579 | 0.162 |
| Anatis_mali_128 | Tytthaspis_sedecimpunctata_581 | 0.162 |
| Anatis_mali_129 | Tytthaspis_sedecimpunctata_581 | 0.162 |
| Anatis_mali_130 | Tytthaspis_sedecimpunctata_581 | 0.162 |
| Anatis_mali_131 | Tytthaspis_sedecimpunctata_581 | 0.162 |
| Anatis_mali_128 | Tytthaspis_sedecimpunctata_582 | 0.162 |
| Anatis_mali_129 | Tytthaspis_sedecimpunctata_582 | 0.162 |
| Anatis_mali_130 | Tytthaspis_sedecimpunctata_582 | 0.162 |
| Anatis_mali_131 | Tytthaspis_sedecimpunctata_582 | 0.162 |
| Anatis_ocellata_132 | Tytthaspis_sedecimpunctata_584 | 0.162 |
| Anatis_ocellata_134 | Tytthaspis_sedecimpunctata_584 | 0.162 |
| Anatis_ocellata_135 | Tytthaspis_sedecimpunctata_584 | 0.162 |
| Anatis_ocellata_138 | Tytthaspis_sedecimpunctata_584 | 0.162 |
| Anatis_ocellata_139 | Tytthaspis_sedecimpunctata_584 | 0.162 |
| Anatis_ocellata_140 | Tytthaspis_sedecimpunctata_584 | 0.162 |
| Anatis_ocellata_141 | Tytthaspis_sedecimpunctata_584 | 0.162 |
| Anatis_ocellata_142 | Tytthaspis_sedecimpunctata_584 | 0.162 |
| Anatis_ocellata_144 | Tytthaspis_sedecimpunctata_584 | 0.162 |
| Anatis_ocellata_145 | Tytthaspis_sedecimpunctata_584 | 0.162 |
| Anatis_ocellata_146 | Tytthaspis_sedecimpunctata_584 | 0.162 |
| Anatis_ocellata_132 | Tytthaspis_sedecimpunctata_586 | 0.162 |
| Anatis_ocellata_134 | Tytthaspis_sedecimpunctata_586 | 0.162 |
| Anatis_ocellata_135 | Tytthaspis_sedecimpunctata_586 | 0.162 |

| | | |
|---|---|---|
| Anatis_ocellata_138 | Tytthaspis_sedecimpunctata_586 | 0.162 |
| Anatis_ocellata_139 | Tytthaspis_sedecimpunctata_586 | 0.162 |
| Anatis_ocellata_140 | Tytthaspis_sedecimpunctata_586 | 0.162 |
| Anatis_ocellata_141 | Tytthaspis_sedecimpunctata_586 | 0.162 |
| Anatis_ocellata_142 | Tytthaspis_sedecimpunctata_586 | 0.162 |
| Anatis_ocellata_144 | Tytthaspis_sedecimpunctata_586 | 0.162 |
| Anatis_ocellata_145 | Tytthaspis_sedecimpunctata_586 | 0.162 |
| Anatis_ocellata_146 | Tytthaspis_sedecimpunctata_586 | 0.162 |
| Anatis_mali_128 | Tytthaspis_sedecimpunctata_590 | 0.162 |
| Anatis_mali_129 | Tytthaspis_sedecimpunctata_590 | 0.162 |
| Anatis_mali_130 | Tytthaspis_sedecimpunctata_590 | 0.162 |
| Anatis_mali_131 | Tytthaspis_sedecimpunctata_590 | 0.162 |
| Anatis_ocellata_132 | Tytthaspis_sedecimpunctata_591 | 0.162 |
| Anatis_ocellata_134 | Tytthaspis_sedecimpunctata_591 | 0.162 |
| Anatis_ocellata_135 | Tytthaspis_sedecimpunctata_591 | 0.162 |
| Anatis_ocellata_138 | Tytthaspis_sedecimpunctata_591 | 0.162 |
| Anatis_ocellata_139 | Tytthaspis_sedecimpunctata_591 | 0.162 |
| Anatis_ocellata_140 | Tytthaspis_sedecimpunctata_591 | 0.162 |
| Anatis_ocellata_141 | Tytthaspis_sedecimpunctata_591 | 0.162 |
| Anatis_ocellata_142 | Tytthaspis_sedecimpunctata_591 | 0.162 |
| Anatis_ocellata_144 | Tytthaspis_sedecimpunctata_591 | 0.162 |
| Anatis_ocellata_145 | Tytthaspis_sedecimpunctata_591 | 0.162 |
| Anatis_ocellata_146 | Tytthaspis_sedecimpunctata_591 | 0.162 |
| Anatis_mali_128 | Tytthaspis_sedecimpunctata_593 | 0.162 |
| Anatis_mali_129 | Tytthaspis_sedecimpunctata_593 | 0.162 |
| Anatis_mali_130 | Tytthaspis_sedecimpunctata_593 | 0.162 |
| Anatis_mali_131 | Tytthaspis_sedecimpunctata_593 | 0.162 |
| Anatis_mali_128 | Tytthaspis_sedecimpunctata_594 | 0.162 |
| Anatis_mali_129 | Tytthaspis_sedecimpunctata_594 | 0.162 |
| Anatis_mali_130 | Tytthaspis_sedecimpunctata_594 | 0.162 |
| Anatis_mali_131 | Tytthaspis_sedecimpunctata_594 | 0.162 |
| Anatis_mali_128 | Tytthaspis_sedecimpunctata_595 | 0.162 |
| Anatis_mali_129 | Tytthaspis_sedecimpunctata_595 | 0.162 |
| Anatis_mali_130 | Tytthaspis_sedecimpunctata_595 | 0.162 |
| Anatis_mali_131 | Tytthaspis_sedecimpunctata_595 | 0.162 |
| Scymnus_pallipediformis_811 | Scymnus_suturalis_841 | 0.162 |
| Scymnus_pallipediformis_811 | Scymnus_suturalis_842 | 0.162 |
| Scymnus_pallipediformis_811 | Scymnus_suturalis_844 | 0.162 |
| Scymnus_pallipediformis_811 | Scymnus_suturalis_846 | 0.162 |
| Scymnus_pallipediformis_811 | Scymnus_suturalis_847 | 0.162 |
| Scymnus_pallipediformis_811 | Scymnus_suturalis_848 | 0.162 |
| Hippodamia_alpina_384 | Tytthaspis_sedecimpunctata_583 | 0.162 |
| Coelophora_inaequalis_320 | Tytthaspis_sedecimpunctata_589 | 0.162 |
| Coelophora_inaequalis_321 | Tytthaspis_sedecimpunctata_589 | 0.162 |
| Coelophora_inaequalis_322 | Tytthaspis_sedecimpunctata_589 | 0.162 |
| Psyllobora_vigintiduopunctata_564 | Tytthaspis_sedecimpunctata_589 | 0.162 |
| Psyllobora_vigintiduopunctata_570 | Tytthaspis_sedecimpunctata_589 | 0.162 |
| Psyllobora_vigintiduopunctata_572 | Tytthaspis_sedecimpunctata_589 | 0.162 |
| Scymnus_pallipediformis_811 | Scymnus_suturalis_832 | 0.162 |
| Scymnus_mimulus_775 | Scymnus_suturalis_833 | 0.162 |
| Scymnus_mimulus_777 | Scymnus_suturalis_833 | 0.162 |
| Scymnus_pallipediformis_811 | Scymnus_suturalis_835 | 0.162 |
| Scymnus_pallipediformis_811 | Scymnus_suturalis_836 | 0.162 |
| Scymnus_mimulus_776 | Scymnus_suturalis_838 | 0.162 |
| Scymnus_pallipediformis_811 | Scymnus_suturalis_839 | 0.162 |
| Scymnus_mimulus_776 | Scymnus_suturalis_840 | 0.162 |

| | | |
|---|---|---|
| Scymnus_mimulus_775 | Scymnus_suturalis_841 | 0.163 |
| Scymnus_mimulus_777 | Scymnus_suturalis_841 | 0.163 |
| Scymnus_mimulus_775 | Scymnus_suturalis_842 | 0.163 |
| Scymnus_mimulus_777 | Scymnus_suturalis_842 | 0.163 |
| Scymnus_mimulus_775 | Scymnus_suturalis_844 | 0.163 |
| Scymnus_mimulus_777 | Scymnus_suturalis_844 | 0.163 |
| Scymnus_mimulus_775 | Scymnus_suturalis_846 | 0.163 |
| Scymnus_mimulus_777 | Scymnus_suturalis_846 | 0.163 |
| Scymnus_mimulus_775 | Scymnus_suturalis_847 | 0.163 |
| Scymnus_mimulus_777 | Scymnus_suturalis_847 | 0.163 |
| Scymnus_mimulus_775 | Scymnus_suturalis_848 | 0.163 |
| Scymnus_mimulus_777 | Scymnus_suturalis_848 | 0.163 |
| Psyllobora_vigintiduopunctata_564 | Tytthaspis_sedecimpunctata_586 | 0.163 |
| Psyllobora_vigintiduopunctata_570 | Tytthaspis_sedecimpunctata_586 | 0.163 |
| Psyllobora_vigintiduopunctata_572 | Tytthaspis_sedecimpunctata_586 | 0.163 |
| Psyllobora_vigintiduopunctata_557 | Tytthaspis_sedecimpunctata_587 | 0.163 |
| Psyllobora_vigintiduopunctata_561 | Tytthaspis_sedecimpunctata_587 | 0.163 |
| Psyllobora_vigintiduopunctata_562 | Tytthaspis_sedecimpunctata_587 | 0.163 |
| Psyllobora_vigintiduopunctata_563 | Tytthaspis_sedecimpunctata_587 | 0.163 |
| Psyllobora_vigintiduopunctata_565 | Tytthaspis_sedecimpunctata_587 | 0.163 |
| Psyllobora_vigintiduopunctata_567 | Tytthaspis_sedecimpunctata_587 | 0.163 |
| Psyllobora_vigintiduopunctata_560 | Tytthaspis_sedecimpunctata_588 | 0.163 |
| Scymnus_mimulus_775 | Scymnus_suturalis_832 | 0.163 |
| Scymnus_mimulus_777 | Scymnus_suturalis_832 | 0.163 |
| Scymnus_mimulus_776 | Scymnus_suturalis_834 | 0.163 |
| Scymnus_mimulus_775 | Scymnus_suturalis_835 | 0.163 |
| Scymnus_mimulus_777 | Scymnus_suturalis_835 | 0.163 |
| Scymnus_mimulus_775 | Scymnus_suturalis_836 | 0.163 |
| Scymnus_mimulus_777 | Scymnus_suturalis_836 | 0.163 |
| Scymnus_mimulus_775 | Scymnus_suturalis_839 | 0.163 |
| Scymnus_mimulus_777 | Scymnus_suturalis_839 | 0.163 |
| Hyperaspis_proba_660 | Tytthaspis_sedecimpunctata_586 | 0.163 |
| Chilocorus_bipustulatus_21 | Tytthaspis_sedecimpunctata_583 | 0.163 |
| Chilocorus_bipustulatus_21 | Tytthaspis_sedecimpunctata_592 | 0.163 |
| Hyperaspis_oregona_658 | Subcoccinella_vigintiquatuorpunctata_622 | 0.163 |
| Hyperaspis_oregona_659 | Subcoccinella_vigintiquatuorpunctata_622 | 0.163 |
| Scymnus_haemorrhoidalis_749 | Tytthaspis_sedecimpunctata_577 | 0.163 |
| Scymnus_haemorrhoidalis_749 | Tytthaspis_sedecimpunctata_578 | 0.163 |
| Scymnus_haemorrhoidalis_749 | Tytthaspis_sedecimpunctata_579 | 0.163 |
| Scymnus_haemorrhoidalis_749 | Tytthaspis_sedecimpunctata_581 | 0.163 |
| Scymnus_haemorrhoidalis_749 | Tytthaspis_sedecimpunctata_582 | 0.163 |
| Psyllobora_vigintiduopunctata_564 | Tytthaspis_sedecimpunctata_583 | 0.163 |
| Scymnus_haemorrhoidalis_753 | Tytthaspis_sedecimpunctata_584 | 0.163 |
| Coelophora_inaequalis_320 | Tytthaspis_sedecimpunctata_585 | 0.163 |
| Coelophora_inaequalis_321 | Tytthaspis_sedecimpunctata_585 | 0.163 |
| Coelophora_inaequalis_322 | Tytthaspis_sedecimpunctata_585 | 0.163 |
| Psyllobora_vigintiduopunctata_564 | Tytthaspis_sedecimpunctata_585 | 0.163 |
| Scymnus_haemorrhoidalis_747 | Tytthaspis_sedecimpunctata_586 | 0.163 |
| Scymnus_haemorrhoidalis_748 | Tytthaspis_sedecimpunctata_586 | 0.163 |
| Psyllobora_vigintiduopunctata_556 | Tytthaspis_sedecimpunctata_587 | 0.163 |
| Scymnus_haemorrhoidalis_753 | Tytthaspis_sedecimpunctata_588 | 0.163 |
| Scymnus_haemorrhoidalis_756 | Tytthaspis_sedecimpunctata_588 | 0.163 |
| Scymnus_haemorrhoidalis_749 | Tytthaspis_sedecimpunctata_590 | 0.163 |
| Scymnus_haemorrhoidalis_753 | Tytthaspis_sedecimpunctata_591 | 0.163 |
| Psyllobora_vigintiduopunctata_564 | Tytthaspis_sedecimpunctata_592 | 0.163 |
| Scymnus_haemorrhoidalis_749 | Tytthaspis_sedecimpunctata_592 | 0.163 |

| | | |
|---|---|---|
| Coelophora_inaequalis_320 | Tytthaspis_sedecimpunctata_593 | 0.163 |
| Coelophora_inaequalis_321 | Tytthaspis_sedecimpunctata_593 | 0.163 |
| Coelophora_inaequalis_322 | Tytthaspis_sedecimpunctata_593 | 0.163 |
| Scymnus_haemorrhoidalis_749 | Tytthaspis_sedecimpunctata_594 | 0.163 |
| Scymnus_haemorrhoidalis_749 | Tytthaspis_sedecimpunctata_595 | 0.163 |
| Chilocorus_bipustulatus_21 | Tytthaspis_sedecimpunctata_580 | 0.163 |
| Hyperaspis_proba_661 | Scymnus_suturalis_841 | 0.163 |
| Hyperaspis_proba_661 | Scymnus_suturalis_842 | 0.163 |
| Rhyzobius_chrysomeloides_85 | Scymnus_suturalis_843 | 0.163 |
| Rhyzobius_chrysomeloides_86 | Scymnus_suturalis_843 | 0.163 |
| Rhyzobius_chrysomeloides_87 | Scymnus_suturalis_843 | 0.163 |
| Rhyzobius_chrysomeloides_88 | Scymnus_suturalis_843 | 0.163 |
| Rhyzobius_chrysomeloides_89 | Scymnus_suturalis_843 | 0.163 |
| Rhyzobius_chrysomeloides_90 | Scymnus_suturalis_843 | 0.163 |
| Rhyzobius_chrysomeloides_91 | Scymnus_suturalis_843 | 0.163 |
| Rhyzobius_chrysomeloides_92 | Scymnus_suturalis_843 | 0.163 |
| Rhyzobius_chrysomeloides_93 | Scymnus_suturalis_843 | 0.163 |
| Rhyzobius_chrysomeloides_94 | Scymnus_suturalis_843 | 0.163 |
| Rhyzobius_chrysomeloides_95 | Scymnus_suturalis_843 | 0.163 |
| Hyperaspis_proba_661 | Scymnus_suturalis_844 | 0.163 |
| Rhyzobius_chrysomeloides_85 | Scymnus_suturalis_845 | 0.163 |
| Rhyzobius_chrysomeloides_86 | Scymnus_suturalis_845 | 0.163 |
| Rhyzobius_chrysomeloides_87 | Scymnus_suturalis_845 | 0.163 |
| Rhyzobius_chrysomeloides_88 | Scymnus_suturalis_845 | 0.163 |
| Rhyzobius_chrysomeloides_89 | Scymnus_suturalis_845 | 0.163 |
| Rhyzobius_chrysomeloides_90 | Scymnus_suturalis_845 | 0.163 |
| Rhyzobius_chrysomeloides_91 | Scymnus_suturalis_845 | 0.163 |
| Rhyzobius_chrysomeloides_92 | Scymnus_suturalis_845 | 0.163 |
| Rhyzobius_chrysomeloides_93 | Scymnus_suturalis_845 | 0.163 |
| Rhyzobius_chrysomeloides_94 | Scymnus_suturalis_845 | 0.163 |
| Rhyzobius_chrysomeloides_95 | Scymnus_suturalis_845 | 0.163 |
| Hyperaspis_proba_661 | Scymnus_suturalis_846 | 0.163 |
| Hyperaspis_proba_661 | Scymnus_suturalis_847 | 0.163 |
| Hyperaspis_proba_661 | Scymnus_suturalis_848 | 0.163 |
| Hippodamia_tredecimpunctata_457 | Tytthaspis_sedecimpunctata_580 | 0.163 |
| Hippodamia_tredecimpunctata_458 | Tytthaspis_sedecimpunctata_580 | 0.163 |
| Psyllobora_vigintiduopunctata_564 | Tytthaspis_sedecimpunctata_580 | 0.163 |
| Scymnus_haemorrhoidalis_749 | Tytthaspis_sedecimpunctata_580 | 0.163 |
| Scymnus_haemorrhoidalis_750 | Tytthaspis_sedecimpunctata_580 | 0.163 |
| Scymnus_haemorrhoidalis_752 | Tytthaspis_sedecimpunctata_583 | 0.163 |
| Scymnus_haemorrhoidalis_753 | Tytthaspis_sedecimpunctata_585 | 0.163 |
| Scymnus_haemorrhoidalis_752 | Tytthaspis_sedecimpunctata_593 | 0.163 |
| Hyperaspis_proba_661 | Scymnus_suturalis_832 | 0.163 |
| Hyperaspis_proba_661 | Scymnus_suturalis_835 | 0.163 |
| Hyperaspis_proba_661 | Scymnus_suturalis_836 | 0.163 |
| Hyperaspis_proba_661 | Scymnus_suturalis_837 | 0.163 |
| Hyperaspis_proba_661 | Scymnus_suturalis_839 | 0.163 |
| Anatis_ocellata_136 | Tytthaspis_sedecimpunctata_589 | 0.163 |
| Hippodamia_tredecimpunctata_459 | Tytthaspis_sedecimpunctata_577 | 0.163 |
| Hippodamia_tredecimpunctata_459 | Tytthaspis_sedecimpunctata_578 | 0.163 |
| Hippodamia_tredecimpunctata_459 | Tytthaspis_sedecimpunctata_579 | 0.163 |
| Hippodamia_notata_421 | Tytthaspis_sedecimpunctata_580 | 0.163 |
| Hippodamia_tredecimpunctata_459 | Tytthaspis_sedecimpunctata_581 | 0.163 |
| Hippodamia_tredecimpunctata_459 | Tytthaspis_sedecimpunctata_582 | 0.163 |
| Ceratomegilla_ulkei_220 | Tytthaspis_sedecimpunctata_583 | 0.163 |
| Hippodamia_tredecimpunctata_457 | Tytthaspis_sedecimpunctata_583 | 0.163 |

| | | |
|---|---|---|
| Hippodamia_tredecimpunctata_458 | Tytthaspis_sedecimpunctata_583 | 0.163 |
| Hippodamia_tredecimpunctata_457 | Tytthaspis_sedecimpunctata_585 | 0.163 |
| Hippodamia_tredecimpunctata_458 | Tytthaspis_sedecimpunctata_585 | 0.163 |
| Halyzia_sedecimguttata_345 | Tytthaspis_sedecimpunctata_588 | 0.163 |
| Halyzia_sedecimguttata_350 | Tytthaspis_sedecimpunctata_588 | 0.163 |
| Halyzia_sedecimguttata_351 | Tytthaspis_sedecimpunctata_588 | 0.163 |
| Hippodamia_tredecimpunctata_459 | Tytthaspis_sedecimpunctata_590 | 0.163 |
| Myrrha_octodecimguttata_488 | Tytthaspis_sedecimpunctata_591 | 0.163 |
| Myrrha_octodecimguttata_491 | Tytthaspis_sedecimpunctata_591 | 0.163 |
| Myrrha_octodecimguttata_492 | Tytthaspis_sedecimpunctata_591 | 0.163 |
| Hippodamia_tredecimpunctata_459 | Tytthaspis_sedecimpunctata_594 | 0.163 |
| Hippodamia_tredecimpunctata_459 | Tytthaspis_sedecimpunctata_595 | 0.163 |
| Rhyzobius_chrysomeloides_85 | Scymnus_suturalis_833 | 0.163 |
| Rhyzobius_chrysomeloides_86 | Scymnus_suturalis_833 | 0.163 |
| Rhyzobius_chrysomeloides_87 | Scymnus_suturalis_833 | 0.163 |
| Rhyzobius_chrysomeloides_88 | Scymnus_suturalis_833 | 0.163 |
| Rhyzobius_chrysomeloides_89 | Scymnus_suturalis_833 | 0.163 |
| Rhyzobius_chrysomeloides_90 | Scymnus_suturalis_833 | 0.163 |
| Rhyzobius_chrysomeloides_91 | Scymnus_suturalis_833 | 0.163 |
| Rhyzobius_chrysomeloides_92 | Scymnus_suturalis_833 | 0.163 |
| Rhyzobius_chrysomeloides_93 | Scymnus_suturalis_833 | 0.163 |
| Rhyzobius_chrysomeloides_94 | Scymnus_suturalis_833 | 0.163 |
| Rhyzobius_chrysomeloides_95 | Scymnus_suturalis_833 | 0.163 |
| Anatis_ocellata_136 | Tytthaspis_sedecimpunctata_588 | 0.163 |
| Scymnus_apetzi_692 | Scymnus_suturalis_838 | 0.163 |
| Scymnus_apetzi_692 | Scymnus_suturalis_840 | 0.163 |
| Anatis_ocellata_136 | Tytthaspis_sedecimpunctata_577 | 0.163 |
| Anatis_ocellata_136 | Tytthaspis_sedecimpunctata_578 | 0.163 |
| Anatis_ocellata_136 | Tytthaspis_sedecimpunctata_579 | 0.163 |
| Anatis_ocellata_136 | Tytthaspis_sedecimpunctata_581 | 0.163 |
| Anatis_ocellata_136 | Tytthaspis_sedecimpunctata_582 | 0.163 |
| Psyllobora_vigintiduopunctata_559 | Tytthaspis_sedecimpunctata_589 | 0.163 |
| Anatis_ocellata_136 | Tytthaspis_sedecimpunctata_590 | 0.163 |
| Anatis_ocellata_136 | Tytthaspis_sedecimpunctata_594 | 0.163 |
| Anatis_ocellata_136 | Tytthaspis_sedecimpunctata_595 | 0.163 |
| Scymnus_apetzi_692 | Scymnus_suturalis_834 | 0.163 |
| Halyzia_sedecimguttata_345 | Tytthaspis_sedecimpunctata_577 | 0.163 |
| Halyzia_sedecimguttata_350 | Tytthaspis_sedecimpunctata_577 | 0.163 |
| Halyzia_sedecimguttata_351 | Tytthaspis_sedecimpunctata_577 | 0.163 |
| Halyzia_sedecimguttata_345 | Tytthaspis_sedecimpunctata_578 | 0.163 |
| Halyzia_sedecimguttata_350 | Tytthaspis_sedecimpunctata_578 | 0.163 |
| Halyzia_sedecimguttata_351 | Tytthaspis_sedecimpunctata_578 | 0.163 |
| Halyzia_sedecimguttata_345 | Tytthaspis_sedecimpunctata_579 | 0.163 |
| Halyzia_sedecimguttata_350 | Tytthaspis_sedecimpunctata_579 | 0.163 |
| Halyzia_sedecimguttata_351 | Tytthaspis_sedecimpunctata_579 | 0.163 |
| Ceratomegilla_ulkei_220 | Tytthaspis_sedecimpunctata_580 | 0.163 |
| Halyzia_sedecimguttata_345 | Tytthaspis_sedecimpunctata_581 | 0.163 |
| Halyzia_sedecimguttata_350 | Tytthaspis_sedecimpunctata_581 | 0.163 |
| Halyzia_sedecimguttata_351 | Tytthaspis_sedecimpunctata_581 | 0.163 |
| Halyzia_sedecimguttata_345 | Tytthaspis_sedecimpunctata_582 | 0.163 |
| Halyzia_sedecimguttata_350 | Tytthaspis_sedecimpunctata_582 | 0.163 |
| Halyzia_sedecimguttata_351 | Tytthaspis_sedecimpunctata_582 | 0.163 |
| Halyzia_sedecimguttata_349 | Tytthaspis_sedecimpunctata_583 | 0.163 |
| Halyzia_sedecimguttata_352 | Tytthaspis_sedecimpunctata_583 | 0.163 |
| Halyzia_sedecimguttata_353 | Tytthaspis_sedecimpunctata_583 | 0.163 |
| Hippodamia_tredecimpunctata_457 | Tytthaspis_sedecimpunctata_584 | 0.163 |

| | | |
|---|---|---|
| Hippodamia_tredecimpunctata_458 | Tytthaspis_sedecimpunctata_584 | 0.163 |
| Halyzia_sedecimguttata_345 | Tytthaspis_sedecimpunctata_585 | 0.163 |
| Halyzia_sedecimguttata_350 | Tytthaspis_sedecimpunctata_585 | 0.163 |
| Halyzia_sedecimguttata_351 | Tytthaspis_sedecimpunctata_585 | 0.163 |
| Oenopia_conglobata_522 | Tytthaspis_sedecimpunctata_586 | 0.163 |
| Halyzia_sedecimguttata_346 | Tytthaspis_sedecimpunctata_588 | 0.163 |
| Halyzia_sedecimguttata_347 | Tytthaspis_sedecimpunctata_588 | 0.163 |
| Halyzia_sedecimguttata_348 | Tytthaspis_sedecimpunctata_588 | 0.163 |
| Halyzia_sedecimguttata_345 | Tytthaspis_sedecimpunctata_590 | 0.163 |
| Halyzia_sedecimguttata_350 | Tytthaspis_sedecimpunctata_590 | 0.163 |
| Halyzia_sedecimguttata_351 | Tytthaspis_sedecimpunctata_590 | 0.163 |
| Halyzia_sedecimguttata_345 | Tytthaspis_sedecimpunctata_592 | 0.163 |
| Halyzia_sedecimguttata_350 | Tytthaspis_sedecimpunctata_592 | 0.163 |
| Halyzia_sedecimguttata_351 | Tytthaspis_sedecimpunctata_592 | 0.163 |
| Hippodamia_convergens_396 | Tytthaspis_sedecimpunctata_592 | 0.163 |
| Hippodamia_convergens_400 | Tytthaspis_sedecimpunctata_592 | 0.163 |
| Hippodamia_convergens_403 | Tytthaspis_sedecimpunctata_592 | 0.163 |
| Hippodamia_moesta_410 | Tytthaspis_sedecimpunctata_592 | 0.163 |
| Halyzia_sedecimguttata_345 | Tytthaspis_sedecimpunctata_593 | 0.163 |
| Halyzia_sedecimguttata_350 | Tytthaspis_sedecimpunctata_593 | 0.163 |
| Halyzia_sedecimguttata_351 | Tytthaspis_sedecimpunctata_593 | 0.163 |
| Halyzia_sedecimguttata_345 | Tytthaspis_sedecimpunctata_594 | 0.163 |
| Halyzia_sedecimguttata_350 | Tytthaspis_sedecimpunctata_594 | 0.163 |
| Halyzia_sedecimguttata_351 | Tytthaspis_sedecimpunctata_594 | 0.163 |
| Halyzia_sedecimguttata_345 | Tytthaspis_sedecimpunctata_595 | 0.163 |
| Halyzia_sedecimguttata_350 | Tytthaspis_sedecimpunctata_595 | 0.163 |
| Halyzia_sedecimguttata_351 | Tytthaspis_sedecimpunctata_595 | 0.163 |
| Psyllobora_vigintiduopunctata_559 | Tytthaspis_sedecimpunctata_586 | 0.163 |
| Psyllobora_vigintiduopunctata_569 | Tytthaspis_sedecimpunctata_587 | 0.163 |
| Halyzia_sedecimguttata_346 | Tytthaspis_sedecimpunctata_577 | 0.163 |
| Halyzia_sedecimguttata_347 | Tytthaspis_sedecimpunctata_577 | 0.163 |
| Halyzia_sedecimguttata_348 | Tytthaspis_sedecimpunctata_577 | 0.163 |
| Halyzia_sedecimguttata_346 | Tytthaspis_sedecimpunctata_578 | 0.163 |
| Halyzia_sedecimguttata_347 | Tytthaspis_sedecimpunctata_578 | 0.163 |
| Halyzia_sedecimguttata_348 | Tytthaspis_sedecimpunctata_578 | 0.163 |
| Halyzia_sedecimguttata_346 | Tytthaspis_sedecimpunctata_579 | 0.163 |
| Halyzia_sedecimguttata_347 | Tytthaspis_sedecimpunctata_579 | 0.163 |
| Halyzia_sedecimguttata_348 | Tytthaspis_sedecimpunctata_579 | 0.163 |
| Halyzia_sedecimguttata_349 | Tytthaspis_sedecimpunctata_580 | 0.163 |
| Halyzia_sedecimguttata_352 | Tytthaspis_sedecimpunctata_580 | 0.163 |
| Halyzia_sedecimguttata_353 | Tytthaspis_sedecimpunctata_580 | 0.163 |
| Hippodamia_convergens_396 | Tytthaspis_sedecimpunctata_580 | 0.163 |
| Hippodamia_convergens_400 | Tytthaspis_sedecimpunctata_580 | 0.163 |
| Hippodamia_convergens_403 | Tytthaspis_sedecimpunctata_580 | 0.163 |
| Hippodamia_moesta_410 | Tytthaspis_sedecimpunctata_580 | 0.163 |
| Halyzia_sedecimguttata_346 | Tytthaspis_sedecimpunctata_581 | 0.163 |
| Halyzia_sedecimguttata_347 | Tytthaspis_sedecimpunctata_581 | 0.163 |
| Halyzia_sedecimguttata_348 | Tytthaspis_sedecimpunctata_581 | 0.163 |
| Halyzia_sedecimguttata_346 | Tytthaspis_sedecimpunctata_582 | 0.163 |
| Halyzia_sedecimguttata_347 | Tytthaspis_sedecimpunctata_582 | 0.163 |
| Halyzia_sedecimguttata_348 | Tytthaspis_sedecimpunctata_582 | 0.163 |
| Coccinula_quatuordecimpustulata_297 | Tytthaspis_sedecimpunctata_583 | 0.163 |
| Oenopia_conglobata_523 | Tytthaspis_sedecimpunctata_583 | 0.163 |
| Ceratomegilla_ulkei_222 | Tytthaspis_sedecimpunctata_584 | 0.163 |
| Ceratomegilla_ulkei_223 | Tytthaspis_sedecimpunctata_584 | 0.163 |
| Hippodamia_parenthesis_439 | Tytthaspis_sedecimpunctata_588 | 0.163 |

Supplementary Table S4 (continued)

| | | |
|---|---|---|
| Hippodamia_parenthesis_432 | Tytthaspis_sedecimpunctata_589 | 0.163 |
| Hippodamia_parenthesis_434 | Tytthaspis_sedecimpunctata_589 | 0.163 |
| Hippodamia_parenthesis_435 | Tytthaspis_sedecimpunctata_589 | 0.163 |
| Hippodamia_parenthesis_437 | Tytthaspis_sedecimpunctata_589 | 0.163 |
| Hippodamia_parenthesis_438 | Tytthaspis_sedecimpunctata_589 | 0.163 |
| Hippodamia_parenthesis_441 | Tytthaspis_sedecimpunctata_589 | 0.163 |
| Hippodamia_parenthesis_442 | Tytthaspis_sedecimpunctata_589 | 0.163 |
| Halyzia_sedecimguttata_346 | Tytthaspis_sedecimpunctata_590 | 0.163 |
| Halyzia_sedecimguttata_347 | Tytthaspis_sedecimpunctata_590 | 0.163 |
| Halyzia_sedecimguttata_348 | Tytthaspis_sedecimpunctata_590 | 0.163 |
| Halyzia_sedecimguttata_346 | Tytthaspis_sedecimpunctata_592 | 0.163 |
| Halyzia_sedecimguttata_347 | Tytthaspis_sedecimpunctata_592 | 0.163 |
| Halyzia_sedecimguttata_348 | Tytthaspis_sedecimpunctata_592 | 0.163 |
| Oenopia_conglobata_523 | Tytthaspis_sedecimpunctata_592 | 0.163 |
| Halyzia_sedecimguttata_346 | Tytthaspis_sedecimpunctata_593 | 0.163 |
| Halyzia_sedecimguttata_347 | Tytthaspis_sedecimpunctata_593 | 0.163 |
| Halyzia_sedecimguttata_348 | Tytthaspis_sedecimpunctata_593 | 0.163 |
| Halyzia_sedecimguttata_346 | Tytthaspis_sedecimpunctata_594 | 0.163 |
| Halyzia_sedecimguttata_347 | Tytthaspis_sedecimpunctata_594 | 0.163 |
| Halyzia_sedecimguttata_348 | Tytthaspis_sedecimpunctata_594 | 0.163 |
| Halyzia_sedecimguttata_346 | Tytthaspis_sedecimpunctata_595 | 0.163 |
| Halyzia_sedecimguttata_347 | Tytthaspis_sedecimpunctata_595 | 0.163 |
| Halyzia_sedecimguttata_348 | Tytthaspis_sedecimpunctata_595 | 0.163 |
| Scymnus_haemorrhoidalis_756 | Scymnus_suturalis_833 | 0.163 |
| Scymnus_haemorrhoidalis_756 | Scymnus_suturalis_838 | 0.163 |
| Psyllobora_vigintiduopunctata_566 | Tytthaspis_sedecimpunctata_577 | 0.163 |
| Psyllobora_vigintiduopunctata_566 | Tytthaspis_sedecimpunctata_578 | 0.163 |
| Psyllobora_vigintiduopunctata_566 | Tytthaspis_sedecimpunctata_579 | 0.163 |
| Psyllobora_vigintiduopunctata_566 | Tytthaspis_sedecimpunctata_581 | 0.163 |
| Psyllobora_vigintiduopunctata_566 | Tytthaspis_sedecimpunctata_582 | 0.163 |
| Psyllobora_vigintiduopunctata_559 | Tytthaspis_sedecimpunctata_583 | 0.163 |
| Psyllobora_vigintiduopunctata_559 | Tytthaspis_sedecimpunctata_585 | 0.163 |
| Psyllobora_vigintiduopunctata_566 | Tytthaspis_sedecimpunctata_590 | 0.163 |
| Psyllobora_vigintiduopunctata_559 | Tytthaspis_sedecimpunctata_592 | 0.163 |
| Psyllobora_vigintiduopunctata_566 | Tytthaspis_sedecimpunctata_594 | 0.163 |
| Psyllobora_vigintiduopunctata_566 | Tytthaspis_sedecimpunctata_595 | 0.163 |
| Brumus_quadripustulatus_10 | Tytthaspis_sedecimpunctata_577 | 0.163 |
| Brumus_quadripustulatus_10 | Tytthaspis_sedecimpunctata_578 | 0.163 |
| Brumus_quadripustulatus_10 | Tytthaspis_sedecimpunctata_579 | 0.163 |
| Brumus_quadripustulatus_10 | Tytthaspis_sedecimpunctata_581 | 0.163 |
| Brumus_quadripustulatus_10 | Tytthaspis_sedecimpunctata_582 | 0.163 |
| Brumus_quadripustulatus_10 | Tytthaspis_sedecimpunctata_590 | 0.163 |
| Brumus_quadripustulatus_10 | Tytthaspis_sedecimpunctata_592 | 0.163 |
| Brumus_quadripustulatus_10 | Tytthaspis_sedecimpunctata_594 | 0.163 |
| Brumus_quadripustulatus_10 | Tytthaspis_sedecimpunctata_595 | 0.163 |
| Hyperaspis_proba_660 | Scymnus_suturalis_843 | 0.163 |
| Hyperaspis_proba_660 | Scymnus_suturalis_845 | 0.163 |
| Psyllobora_vigintiduopunctata_559 | Tytthaspis_sedecimpunctata_580 | 0.163 |
| Scymnus_haemorrhoidalis_755 | Scymnus_suturalis_841 | 0.163 |
| Scymnus_haemorrhoidalis_755 | Scymnus_suturalis_842 | 0.163 |
| Scymnus_haemorrhoidalis_755 | Scymnus_suturalis_844 | 0.163 |
| Scymnus_haemorrhoidalis_755 | Scymnus_suturalis_846 | 0.163 |
| Scymnus_haemorrhoidalis_755 | Scymnus_suturalis_847 | 0.163 |
| Scymnus_haemorrhoidalis_755 | Scymnus_suturalis_848 | 0.163 |
| Coccinula_quatuordecimpustulata_297 | Tytthaspis_sedecimpunctata_580 | 0.163 |
| Halyzia_sedecimguttata_346 | Tytthaspis_sedecimpunctata_580 | 0.163 |

| | | |
|---|---|---|
| Halyzia_sedecimguttata_347 | Tytthaspis_sedecimpunctata_580 | 0.163 |
| Halyzia_sedecimguttata_348 | Tytthaspis_sedecimpunctata_580 | 0.163 |
| Hippodamia_parenthesis_439 | Tytthaspis_sedecimpunctata_583 | 0.163 |
| Hippodamia_parenthesis_439 | Tytthaspis_sedecimpunctata_585 | 0.163 |
| Hippodamia_parenthesis_432 | Tytthaspis_sedecimpunctata_586 | 0.163 |
| Hippodamia_parenthesis_434 | Tytthaspis_sedecimpunctata_586 | 0.163 |
| Hippodamia_parenthesis_435 | Tytthaspis_sedecimpunctata_586 | 0.163 |
| Hippodamia_parenthesis_437 | Tytthaspis_sedecimpunctata_586 | 0.163 |
| Hippodamia_parenthesis_438 | Tytthaspis_sedecimpunctata_586 | 0.163 |
| Hippodamia_parenthesis_441 | Tytthaspis_sedecimpunctata_586 | 0.163 |
| Hippodamia_parenthesis_442 | Tytthaspis_sedecimpunctata_586 | 0.163 |
| Calvia_quatuordecimguttata_206 | Tytthaspis_sedecimpunctata_587 | 0.163 |
| Calvia_quatuordecimguttata_216 | Tytthaspis_sedecimpunctata_587 | 0.163 |
| Hippodamia_parenthesis_433 | Tytthaspis_sedecimpunctata_588 | 0.163 |
| Hippodamia_parenthesis_440 | Tytthaspis_sedecimpunctata_588 | 0.163 |
| Coccinula_quatuordecimpustulata_305 | Tytthaspis_sedecimpunctata_589 | 0.163 |
| Oenopia_conglobata_523 | Tytthaspis_sedecimpunctata_591 | 0.163 |
| Hippodamia_parenthesis_439 | Tytthaspis_sedecimpunctata_593 | 0.163 |
| Scymnus_haemorrhoidalis_755 | Scymnus_suturalis_832 | 0.163 |
| Scymnus_haemorrhoidalis_756 | Scymnus_suturalis_834 | 0.163 |
| Scymnus_haemorrhoidalis_755 | Scymnus_suturalis_835 | 0.163 |
| Scymnus_haemorrhoidalis_755 | Scymnus_suturalis_836 | 0.163 |
| Scymnus_haemorrhoidalis_755 | Scymnus_suturalis_837 | 0.163 |
| Scymnus_haemorrhoidalis_755 | Scymnus_suturalis_839 | 0.163 |
| Scymnus_nigrinus_787 | Scymnus_sinuanodulus_830 | 0.163 |
| Scymnus_haemorrhoidalis_750 | Scymnus_suturalis_841 | 0.163 |
| Scymnus_haemorrhoidalis_750 | Scymnus_suturalis_842 | 0.163 |
| Scymnus_haemorrhoidalis_749 | Scymnus_suturalis_843 | 0.163 |
| Scymnus_haemorrhoidalis_751 | Scymnus_suturalis_843 | 0.163 |
| Scymnus_haemorrhoidalis_754 | Scymnus_suturalis_843 | 0.163 |
| Scymnus_haemorrhoidalis_757 | Scymnus_suturalis_843 | 0.163 |
| Scymnus_haemorrhoidalis_758 | Scymnus_suturalis_843 | 0.163 |
| Scymnus_haemorrhoidalis_750 | Scymnus_suturalis_844 | 0.163 |
| Scymnus_haemorrhoidalis_749 | Scymnus_suturalis_845 | 0.163 |
| Scymnus_haemorrhoidalis_751 | Scymnus_suturalis_845 | 0.163 |
| Scymnus_haemorrhoidalis_754 | Scymnus_suturalis_845 | 0.163 |
| Scymnus_haemorrhoidalis_757 | Scymnus_suturalis_845 | 0.163 |
| Scymnus_haemorrhoidalis_758 | Scymnus_suturalis_845 | 0.163 |
| Scymnus_haemorrhoidalis_750 | Scymnus_suturalis_846 | 0.163 |
| Scymnus_haemorrhoidalis_750 | Scymnus_suturalis_847 | 0.163 |
| Scymnus_haemorrhoidalis_750 | Scymnus_suturalis_848 | 0.163 |
| Coccinula_quatuordecimpustulata_306 | Tytthaspis_sedecimpunctata_577 | 0.163 |
| Coccinula_quatuordecimpustulata_306 | Tytthaspis_sedecimpunctata_578 | 0.163 |
| Coccinula_quatuordecimpustulata_306 | Tytthaspis_sedecimpunctata_579 | 0.163 |
| Coccinula_quatuordecimpustulata_306 | Tytthaspis_sedecimpunctata_581 | 0.163 |
| Coccinula_quatuordecimpustulata_306 | Tytthaspis_sedecimpunctata_582 | 0.163 |
| Coccinula_quatuordecimpustulata_304 | Tytthaspis_sedecimpunctata_583 | 0.163 |
| Hippodamia_parenthesis_433 | Tytthaspis_sedecimpunctata_583 | 0.163 |
| Hippodamia_parenthesis_440 | Tytthaspis_sedecimpunctata_583 | 0.163 |
| Coccinula_quatuordecimpustulata_306 | Tytthaspis_sedecimpunctata_585 | 0.163 |
| Hippodamia_parenthesis_433 | Tytthaspis_sedecimpunctata_585 | 0.163 |
| Hippodamia_parenthesis_440 | Tytthaspis_sedecimpunctata_585 | 0.163 |
| Coccinula_quatuordecimpustulata_294 | Tytthaspis_sedecimpunctata_586 | 0.163 |
| Coccinula_quatuordecimpustulata_295 | Tytthaspis_sedecimpunctata_586 | 0.163 |
| Coccinula_quatuordecimpustulata_308 | Tytthaspis_sedecimpunctata_586 | 0.163 |
| Calvia_quatuordecimguttata_203 | Tytthaspis_sedecimpunctata_588 | 0.163 |

| | | |
|---|---|---|
| Coccinula_quatuordecimpustulata_292 | Tytthaspis_sedecimpunctata_588 | 0.163 |
| Coccinula_quatuordecimpustulata_293 | Tytthaspis_sedecimpunctata_588 | 0.163 |
| Coccinula_quatuordecimpustulata_307 | Tytthaspis_sedecimpunctata_588 | 0.163 |
| Coccinula_quatuordecimpustulata_296 | Tytthaspis_sedecimpunctata_589 | 0.163 |
| Coccinula_quatuordecimpustulata_306 | Tytthaspis_sedecimpunctata_590 | 0.163 |
| Calvia_quatuordecimguttata_203 | Tytthaspis_sedecimpunctata_591 | 0.163 |
| Coccinula_quatuordecimpustulata_305 | Tytthaspis_sedecimpunctata_591 | 0.163 |
| Coccinula_quatuordecimpustulata_306 | Tytthaspis_sedecimpunctata_592 | 0.163 |
| Coccinula_quatuordecimpustulata_306 | Tytthaspis_sedecimpunctata_593 | 0.163 |
| Hippodamia_parenthesis_433 | Tytthaspis_sedecimpunctata_593 | 0.163 |
| Hippodamia_parenthesis_440 | Tytthaspis_sedecimpunctata_593 | 0.163 |
| Coccinula_quatuordecimpustulata_306 | Tytthaspis_sedecimpunctata_594 | 0.163 |
| Coccinula_quatuordecimpustulata_306 | Tytthaspis_sedecimpunctata_595 | 0.163 |
| Scymnus_haemorrhoidalis_750 | Scymnus_suturalis_832 | 0.163 |
| Scymnus_haemorrhoidalis_750 | Scymnus_suturalis_835 | 0.163 |
| Scymnus_haemorrhoidalis_750 | Scymnus_suturalis_836 | 0.163 |
| Scymnus_haemorrhoidalis_750 | Scymnus_suturalis_837 | 0.163 |
| Scymnus_haemorrhoidalis_750 | Scymnus_suturalis_839 | 0.163 |
| Scymnus_haemorrhoidalis_755 | Scymnus_suturalis_840 | 0.163 |
| Hippodamia_arctica_390 | Tytthaspis_sedecimpunctata_580 | 0.163 |
| Scymnus_nigrinus_787 | Scymnus_sinuanodulus_828 | 0.163 |
| Scymnus_nigrinus_785 | Scymnus_sinuanodulus_831 | 0.163 |
| Scymnus_nigrinus_786 | Scymnus_sinuanodulus_831 | 0.163 |
| Coccinula_quatuordecimpustulata_298 | Tytthaspis_sedecimpunctata_577 | 0.163 |
| Coccinula_quatuordecimpustulata_299 | Tytthaspis_sedecimpunctata_577 | 0.163 |
| Coccinula_quatuordecimpustulata_301 | Tytthaspis_sedecimpunctata_577 | 0.163 |
| Coccinula_quatuordecimpustulata_302 | Tytthaspis_sedecimpunctata_577 | 0.163 |
| Coccinula_quatuordecimpustulata_303 | Tytthaspis_sedecimpunctata_577 | 0.163 |
| Coccinula_quatuordecimpustulata_309 | Tytthaspis_sedecimpunctata_577 | 0.163 |
| Coccinula_quatuordecimpustulata_298 | Tytthaspis_sedecimpunctata_578 | 0.163 |
| Coccinula_quatuordecimpustulata_299 | Tytthaspis_sedecimpunctata_578 | 0.163 |
| Coccinula_quatuordecimpustulata_301 | Tytthaspis_sedecimpunctata_578 | 0.163 |
| Coccinula_quatuordecimpustulata_302 | Tytthaspis_sedecimpunctata_578 | 0.163 |
| Coccinula_quatuordecimpustulata_303 | Tytthaspis_sedecimpunctata_578 | 0.163 |
| Coccinula_quatuordecimpustulata_309 | Tytthaspis_sedecimpunctata_578 | 0.163 |
| Coccinula_quatuordecimpustulata_298 | Tytthaspis_sedecimpunctata_579 | 0.163 |
| Coccinula_quatuordecimpustulata_299 | Tytthaspis_sedecimpunctata_579 | 0.163 |
| Coccinula_quatuordecimpustulata_301 | Tytthaspis_sedecimpunctata_579 | 0.163 |
| Coccinula_quatuordecimpustulata_302 | Tytthaspis_sedecimpunctata_579 | 0.163 |
| Coccinula_quatuordecimpustulata_303 | Tytthaspis_sedecimpunctata_579 | 0.163 |
| Coccinula_quatuordecimpustulata_309 | Tytthaspis_sedecimpunctata_579 | 0.163 |
| Coccinula_quatuordecimpustulata_304 | Tytthaspis_sedecimpunctata_580 | 0.163 |
| Coccinula_quatuordecimpustulata_298 | Tytthaspis_sedecimpunctata_581 | 0.163 |
| Coccinula_quatuordecimpustulata_299 | Tytthaspis_sedecimpunctata_581 | 0.163 |
| Coccinula_quatuordecimpustulata_301 | Tytthaspis_sedecimpunctata_581 | 0.163 |
| Coccinula_quatuordecimpustulata_302 | Tytthaspis_sedecimpunctata_581 | 0.163 |
| Coccinula_quatuordecimpustulata_303 | Tytthaspis_sedecimpunctata_581 | 0.163 |
| Coccinula_quatuordecimpustulata_309 | Tytthaspis_sedecimpunctata_581 | 0.163 |
| Coccinula_quatuordecimpustulata_298 | Tytthaspis_sedecimpunctata_582 | 0.163 |
| Coccinula_quatuordecimpustulata_299 | Tytthaspis_sedecimpunctata_582 | 0.163 |
| Coccinula_quatuordecimpustulata_301 | Tytthaspis_sedecimpunctata_582 | 0.163 |
| Coccinula_quatuordecimpustulata_302 | Tytthaspis_sedecimpunctata_582 | 0.163 |
| Coccinula_quatuordecimpustulata_303 | Tytthaspis_sedecimpunctata_582 | 0.163 |
| Coccinula_quatuordecimpustulata_309 | Tytthaspis_sedecimpunctata_582 | 0.163 |
| Coccinula_quatuordecimpustulata_300 | Tytthaspis_sedecimpunctata_583 | 0.163 |
| Coccinula_quatuordecimpustulata_306 | Tytthaspis_sedecimpunctata_584 | 0.163 |

| | | |
|---|---|---|
| Coccinula_quatuordecimpustulata_298 | Tytthaspis_sedecimpunctata_585 | 0.163 |
| Coccinula_quatuordecimpustulata_299 | Tytthaspis_sedecimpunctata_585 | 0.163 |
| Coccinula_quatuordecimpustulata_301 | Tytthaspis_sedecimpunctata_585 | 0.163 |
| Coccinula_quatuordecimpustulata_302 | Tytthaspis_sedecimpunctata_585 | 0.163 |
| Coccinula_quatuordecimpustulata_303 | Tytthaspis_sedecimpunctata_585 | 0.163 |
| Coccinula_quatuordecimpustulata_309 | Tytthaspis_sedecimpunctata_585 | 0.163 |
| Calvia_quatuordecimguttata_205 | Tytthaspis_sedecimpunctata_586 | 0.163 |
| Coccinula_quatuordecimpustulata_298 | Tytthaspis_sedecimpunctata_590 | 0.163 |
| Coccinula_quatuordecimpustulata_299 | Tytthaspis_sedecimpunctata_590 | 0.163 |
| Coccinula_quatuordecimpustulata_301 | Tytthaspis_sedecimpunctata_590 | 0.163 |
| Coccinula_quatuordecimpustulata_302 | Tytthaspis_sedecimpunctata_590 | 0.163 |
| Coccinula_quatuordecimpustulata_303 | Tytthaspis_sedecimpunctata_590 | 0.163 |
| Coccinula_quatuordecimpustulata_309 | Tytthaspis_sedecimpunctata_590 | 0.163 |
| Coccinula_quatuordecimpustulata_298 | Tytthaspis_sedecimpunctata_592 | 0.163 |
| Coccinula_quatuordecimpustulata_299 | Tytthaspis_sedecimpunctata_592 | 0.163 |
| Coccinula_quatuordecimpustulata_301 | Tytthaspis_sedecimpunctata_592 | 0.163 |
| Coccinula_quatuordecimpustulata_302 | Tytthaspis_sedecimpunctata_592 | 0.163 |
| Coccinula_quatuordecimpustulata_303 | Tytthaspis_sedecimpunctata_592 | 0.163 |
| Coccinula_quatuordecimpustulata_309 | Tytthaspis_sedecimpunctata_592 | 0.163 |
| Coccinula_quatuordecimpustulata_298 | Tytthaspis_sedecimpunctata_593 | 0.163 |
| Coccinula_quatuordecimpustulata_299 | Tytthaspis_sedecimpunctata_593 | 0.163 |
| Coccinula_quatuordecimpustulata_301 | Tytthaspis_sedecimpunctata_593 | 0.163 |
| Coccinula_quatuordecimpustulata_302 | Tytthaspis_sedecimpunctata_593 | 0.163 |
| Coccinula_quatuordecimpustulata_303 | Tytthaspis_sedecimpunctata_593 | 0.163 |
| Coccinula_quatuordecimpustulata_309 | Tytthaspis_sedecimpunctata_593 | 0.163 |
| Macronaemia_episcopalis_477 | Tytthaspis_sedecimpunctata_593 | 0.163 |
| Coccinula_quatuordecimpustulata_298 | Tytthaspis_sedecimpunctata_594 | 0.163 |
| Coccinula_quatuordecimpustulata_299 | Tytthaspis_sedecimpunctata_594 | 0.163 |
| Coccinula_quatuordecimpustulata_301 | Tytthaspis_sedecimpunctata_594 | 0.163 |
| Coccinula_quatuordecimpustulata_302 | Tytthaspis_sedecimpunctata_594 | 0.163 |
| Coccinula_quatuordecimpustulata_303 | Tytthaspis_sedecimpunctata_594 | 0.163 |
| Coccinula_quatuordecimpustulata_309 | Tytthaspis_sedecimpunctata_594 | 0.163 |
| Coccinula_quatuordecimpustulata_298 | Tytthaspis_sedecimpunctata_595 | 0.163 |
| Coccinula_quatuordecimpustulata_299 | Tytthaspis_sedecimpunctata_595 | 0.163 |
| Coccinula_quatuordecimpustulata_301 | Tytthaspis_sedecimpunctata_595 | 0.163 |
| Coccinula_quatuordecimpustulata_302 | Tytthaspis_sedecimpunctata_595 | 0.163 |
| Coccinula_quatuordecimpustulata_303 | Tytthaspis_sedecimpunctata_595 | 0.163 |
| Coccinula_quatuordecimpustulata_309 | Tytthaspis_sedecimpunctata_595 | 0.163 |
| Scymnus_haemorrhoidalis_750 | Scymnus_suturalis_840 | 0.163 |
| Oenopia_conglobata_524 | Tytthaspis_sedecimpunctata_577 | 0.163 |
| Oenopia_conglobata_524 | Tytthaspis_sedecimpunctata_578 | 0.163 |
| Oenopia_conglobata_524 | Tytthaspis_sedecimpunctata_579 | 0.163 |
| Oenopia_conglobata_524 | Tytthaspis_sedecimpunctata_581 | 0.163 |
| Oenopia_conglobata_524 | Tytthaspis_sedecimpunctata_582 | 0.163 |
| Oenopia_conglobata_524 | Tytthaspis_sedecimpunctata_590 | 0.163 |
| Oenopia_conglobata_524 | Tytthaspis_sedecimpunctata_594 | 0.163 |
| Oenopia_conglobata_524 | Tytthaspis_sedecimpunctata_595 | 0.163 |
| Psyllobora_vigintiduopunctata_558 | Tytthaspis_sedecimpunctata_577 | 0.163 |
| Psyllobora_vigintiduopunctata_568 | Tytthaspis_sedecimpunctata_577 | 0.163 |
| Psyllobora_vigintiduopunctata_558 | Tytthaspis_sedecimpunctata_578 | 0.163 |
| Psyllobora_vigintiduopunctata_568 | Tytthaspis_sedecimpunctata_578 | 0.163 |
| Psyllobora_vigintiduopunctata_558 | Tytthaspis_sedecimpunctata_579 | 0.163 |
| Psyllobora_vigintiduopunctata_568 | Tytthaspis_sedecimpunctata_579 | 0.163 |
| Psyllobora_vigintiduopunctata_558 | Tytthaspis_sedecimpunctata_581 | 0.163 |
| Psyllobora_vigintiduopunctata_568 | Tytthaspis_sedecimpunctata_581 | 0.163 |
| Psyllobora_vigintiduopunctata_558 | Tytthaspis_sedecimpunctata_582 | 0.163 |

| | | |
|---|---|---|
| Psyllobora_vigintiduopunctata_568 | Tytthaspis_sedecimpunctata_582 | 0.163 |
| Psyllobora_vigintiduopunctata_558 | Tytthaspis_sedecimpunctata_590 | 0.163 |
| Psyllobora_vigintiduopunctata_568 | Tytthaspis_sedecimpunctata_590 | 0.163 |
| Psyllobora_vigintiduopunctata_558 | Tytthaspis_sedecimpunctata_594 | 0.163 |
| Psyllobora_vigintiduopunctata_568 | Tytthaspis_sedecimpunctata_594 | 0.163 |
| Psyllobora_vigintiduopunctata_558 | Tytthaspis_sedecimpunctata_595 | 0.163 |
| Psyllobora_vigintiduopunctata_568 | Tytthaspis_sedecimpunctata_595 | 0.163 |
| Coccinula_quatuordecimpustulata_300 | Tytthaspis_sedecimpunctata_580 | 0.163 |
| Coccinula_quatuordecimpustulata_298 | Tytthaspis_sedecimpunctata_584 | 0.163 |
| Coccinula_quatuordecimpustulata_299 | Tytthaspis_sedecimpunctata_584 | 0.163 |
| Coccinula_quatuordecimpustulata_301 | Tytthaspis_sedecimpunctata_584 | 0.163 |
| Coccinula_quatuordecimpustulata_302 | Tytthaspis_sedecimpunctata_584 | 0.163 |
| Coccinula_quatuordecimpustulata_303 | Tytthaspis_sedecimpunctata_584 | 0.163 |
| Coccinula_quatuordecimpustulata_309 | Tytthaspis_sedecimpunctata_584 | 0.163 |
| Hippodamia_parenthesis_436 | Tytthaspis_sedecimpunctata_587 | 0.163 |
| Hippodamia_parenthesis_436 | Tytthaspis_sedecimpunctata_584 | 0.163 |
| Hippodamia_parenthesis_436 | Tytthaspis_sedecimpunctata_591 | 0.163 |
| Myrrha_octodecimguttata_499 | Vibidia_duodecimguttata_603 | 0.164 |
| Scymnus_frontalis_734 | Scymnus_rubromaculatus_822 | 0.164 |
| Scymnus_frontalis_734 | Scymnus_rubromaculatus_823 | 0.164 |
| Scymnus_frontalis_734 | Scymnus_rubromaculatus_824 | 0.164 |
| Scymnus_frontalis_734 | Scymnus_rubromaculatus_825 | 0.164 |
| Scymnus_frontalis_734 | Scymnus_rubromaculatus_826 | 0.164 |
| Scymnus_frontalis_734 | Scymnus_rubromaculatus_827 | 0.164 |
| Scymnus_apetzi_692 | Scymnus_sinuanodulus_830 | 0.164 |
| Scymnus_auritus_698 | Scymnus_sinuanodulus_831 | 0.164 |
| Myrrha_octodecimguttata_485 | Vibidia_duodecimguttata_599 | 0.164 |
| Myrrha_octodecimguttata_486 | Vibidia_duodecimguttata_599 | 0.164 |
| Myrrha_octodecimguttata_487 | Vibidia_duodecimguttata_599 | 0.164 |
| Myrrha_octodecimguttata_490 | Vibidia_duodecimguttata_599 | 0.164 |
| Myrrha_octodecimguttata_494 | Vibidia_duodecimguttata_599 | 0.164 |
| Myrrha_octodecimguttata_496 | Vibidia_duodecimguttata_599 | 0.164 |
| Myrrha_octodecimguttata_497 | Vibidia_duodecimguttata_599 | 0.164 |
| Myrrha_octodecimguttata_501 | Vibidia_duodecimguttata_599 | 0.164 |
| Myrrha_octodecimguttata_502 | Vibidia_duodecimguttata_599 | 0.164 |
| Myrrha_octodecimguttata_485 | Vibidia_duodecimguttata_602 | 0.164 |
| Myrrha_octodecimguttata_486 | Vibidia_duodecimguttata_602 | 0.164 |
| Myrrha_octodecimguttata_487 | Vibidia_duodecimguttata_602 | 0.164 |
| Myrrha_octodecimguttata_490 | Vibidia_duodecimguttata_602 | 0.164 |
| Myrrha_octodecimguttata_494 | Vibidia_duodecimguttata_602 | 0.164 |
| Myrrha_octodecimguttata_496 | Vibidia_duodecimguttata_602 | 0.164 |
| Myrrha_octodecimguttata_497 | Vibidia_duodecimguttata_602 | 0.164 |
| Myrrha_octodecimguttata_501 | Vibidia_duodecimguttata_602 | 0.164 |
| Myrrha_octodecimguttata_502 | Vibidia_duodecimguttata_602 | 0.164 |
| Scymnus_apetzi_693 | Scymnus_suturalis_843 | 0.164 |
| Scymnus_apetzi_693 | Scymnus_suturalis_845 | 0.164 |
| Scymnus_apetzi_693 | Scymnus_suturalis_837 | 0.164 |
| Scymnus_interruptus_759 | Scymnus_sinuanodulus_829 | 0.164 |
| Scymnus_interruptus_761 | Scymnus_sinuanodulus_829 | 0.164 |
| Scymnus_interruptus_763 | Scymnus_sinuanodulus_829 | 0.164 |
| Scymnus_interruptus_764 | Scymnus_sinuanodulus_829 | 0.164 |
| Scymnus_interruptus_766 | Scymnus_sinuanodulus_829 | 0.164 |
| Scymnus_interruptus_769 | Scymnus_sinuanodulus_829 | 0.164 |
| Brumus_quadripustulatus_5 | Tytthaspis_sedecimpunctata_585 | 0.164 |
| Brumus_quadripustulatus_5 | Tytthaspis_sedecimpunctata_593 | 0.164 |
| Calvia_quatuordecimguttata_202 | Vibidia_duodecimguttata_596 | 0.164 |

| | | |
|---|---|---|
| Calvia_quatuordecimguttata_210 | Vibidia_duodecimguttata_596 | 0.164 |
| Myrrha_octodecimguttata_485 | Vibidia_duodecimguttata_597 | 0.164 |
| Myrrha_octodecimguttata_486 | Vibidia_duodecimguttata_597 | 0.164 |
| Myrrha_octodecimguttata_487 | Vibidia_duodecimguttata_597 | 0.164 |
| Myrrha_octodecimguttata_490 | Vibidia_duodecimguttata_597 | 0.164 |
| Myrrha_octodecimguttata_494 | Vibidia_duodecimguttata_597 | 0.164 |
| Myrrha_octodecimguttata_496 | Vibidia_duodecimguttata_597 | 0.164 |
| Myrrha_octodecimguttata_497 | Vibidia_duodecimguttata_597 | 0.164 |
| Myrrha_octodecimguttata_501 | Vibidia_duodecimguttata_597 | 0.164 |
| Myrrha_octodecimguttata_502 | Vibidia_duodecimguttata_597 | 0.164 |
| Myrrha_octodecimguttata_495 | Vibidia_duodecimguttata_599 | 0.164 |
| Myrrha_octodecimguttata_485 | Vibidia_duodecimguttata_600 | 0.164 |
| Myrrha_octodecimguttata_486 | Vibidia_duodecimguttata_600 | 0.164 |
| Myrrha_octodecimguttata_487 | Vibidia_duodecimguttata_600 | 0.164 |
| Myrrha_octodecimguttata_490 | Vibidia_duodecimguttata_600 | 0.164 |
| Myrrha_octodecimguttata_494 | Vibidia_duodecimguttata_600 | 0.164 |
| Myrrha_octodecimguttata_496 | Vibidia_duodecimguttata_600 | 0.164 |
| Myrrha_octodecimguttata_497 | Vibidia_duodecimguttata_600 | 0.164 |
| Myrrha_octodecimguttata_501 | Vibidia_duodecimguttata_600 | 0.164 |
| Myrrha_octodecimguttata_502 | Vibidia_duodecimguttata_600 | 0.164 |
| Myrrha_octodecimguttata_495 | Vibidia_duodecimguttata_602 | 0.164 |
| Myrrha_octodecimguttata_493 | Vibidia_duodecimguttata_603 | 0.164 |
| Myrrha_octodecimguttata_498 | Vibidia_duodecimguttata_603 | 0.164 |
| Scymnus_apetzi_696 | Scymnus_sinuanodulus_831 | 0.164 |
| Scymnus_apetzi_692 | Scymnus_sinuanodulus_831 | 0.164 |
| Anisosticta_novemdecimpunctata_162 | Tytthaspis_sedecimpunctata_591 | 0.164 |
| Anisosticta_novemdecimpunctata_164 | Tytthaspis_sedecimpunctata_591 | 0.164 |
| Anisosticta_novemdecimpunctata_166 | Tytthaspis_sedecimpunctata_591 | 0.164 |
| Anisosticta_novemdecimpunctata_167 | Tytthaspis_sedecimpunctata_591 | 0.164 |
| Anisosticta_novemdecimpunctata_168 | Tytthaspis_sedecimpunctata_591 | 0.164 |
| Anatis_labiculata_123 | Vibidia_duodecimguttata_596 | 0.164 |
| Anatis_labiculata_124 | Vibidia_duodecimguttata_596 | 0.164 |
| Anatis_labiculata_125 | Vibidia_duodecimguttata_596 | 0.164 |
| Anatis_labiculata_127 | Vibidia_duodecimguttata_596 | 0.164 |
| Myrrha_octodecimguttata_495 | Vibidia_duodecimguttata_597 | 0.164 |
| Anatis_labiculata_123 | Vibidia_duodecimguttata_598 | 0.164 |
| Anatis_labiculata_124 | Vibidia_duodecimguttata_598 | 0.164 |
| Anatis_labiculata_125 | Vibidia_duodecimguttata_598 | 0.164 |
| Anatis_labiculata_127 | Vibidia_duodecimguttata_598 | 0.164 |
| Anatis_labiculata_123 | Vibidia_duodecimguttata_599 | 0.164 |
| Anatis_labiculata_124 | Vibidia_duodecimguttata_599 | 0.164 |
| Anatis_labiculata_125 | Vibidia_duodecimguttata_599 | 0.164 |
| Anatis_labiculata_127 | Vibidia_duodecimguttata_599 | 0.164 |
| Myrrha_octodecimguttata_495 | Vibidia_duodecimguttata_600 | 0.164 |
| Anatis_labiculata_123 | Vibidia_duodecimguttata_601 | 0.164 |
| Anatis_labiculata_124 | Vibidia_duodecimguttata_601 | 0.164 |
| Anatis_labiculata_125 | Vibidia_duodecimguttata_601 | 0.164 |
| Anatis_labiculata_127 | Vibidia_duodecimguttata_601 | 0.164 |
| Anatis_labiculata_123 | Vibidia_duodecimguttata_602 | 0.164 |
| Anatis_labiculata_124 | Vibidia_duodecimguttata_602 | 0.164 |
| Anatis_labiculata_125 | Vibidia_duodecimguttata_602 | 0.164 |
| Anatis_labiculata_127 | Vibidia_duodecimguttata_602 | 0.164 |
| Anatis_labiculata_123 | Vibidia_duodecimguttata_603 | 0.164 |
| Anatis_labiculata_124 | Vibidia_duodecimguttata_603 | 0.164 |
| Anatis_labiculata_125 | Vibidia_duodecimguttata_603 | 0.164 |
| Anatis_labiculata_127 | Vibidia_duodecimguttata_603 | 0.164 |

| | | |
|---|---|---|
| Brumus_quadripustulatus_5 | Scymnus_suturalis_833 | 0.164 |
| Psyllobora_vigintimaculata_574 | Vibidia_duodecimguttata_598 | 0.164 |
| Psyllobora_vigintimaculata_574 | Vibidia_duodecimguttata_599 | 0.164 |
| Psyllobora_vigintimaculata_574 | Vibidia_duodecimguttata_601 | 0.164 |
| Psyllobora_vigintimaculata_574 | Vibidia_duodecimguttata_602 | 0.164 |
| Brumus_quadripustulatus_5 | Scymnus_suturalis_843 | 0.164 |
| Brumus_quadripustulatus_5 | Scymnus_suturalis_845 | 0.164 |
| Scymnus_auritus_698 | Scymnus_sinuanodulus_829 | 0.164 |
| Scymnus_auritus_700 | Scymnus_sinuanodulus_829 | 0.164 |
| Chilocorus_stigma_43 | Tytthaspis_sedecimpunctata_583 | 0.164 |
| Chilocorus_bipustulatus_23 | Tytthaspis_sedecimpunctata_589 | 0.164 |
| Chilocorus_stigma_43 | Tytthaspis_sedecimpunctata_580 | 0.164 |
| Chilocorus_bipustulatus_24 | Tytthaspis_sedecimpunctata_589 | 0.164 |
| Brumus_quadripustulatus_11 | Tytthaspis_sedecimpunctata_577 | 0.164 |
| Brumus_quadripustulatus_13 | Tytthaspis_sedecimpunctata_577 | 0.164 |
| Brumus_quadripustulatus_14 | Tytthaspis_sedecimpunctata_577 | 0.164 |
| Brumus_quadripustulatus_17 | Tytthaspis_sedecimpunctata_577 | 0.164 |
| Brumus_quadripustulatus_3 | Tytthaspis_sedecimpunctata_577 | 0.164 |
| Brumus_quadripustulatus_4 | Tytthaspis_sedecimpunctata_577 | 0.164 |
| Brumus_quadripustulatus_7 | Tytthaspis_sedecimpunctata_577 | 0.164 |
| Brumus_quadripustulatus_11 | Tytthaspis_sedecimpunctata_578 | 0.164 |
| Brumus_quadripustulatus_13 | Tytthaspis_sedecimpunctata_578 | 0.164 |
| Brumus_quadripustulatus_14 | Tytthaspis_sedecimpunctata_578 | 0.164 |
| Brumus_quadripustulatus_17 | Tytthaspis_sedecimpunctata_578 | 0.164 |
| Brumus_quadripustulatus_3 | Tytthaspis_sedecimpunctata_578 | 0.164 |
| Brumus_quadripustulatus_4 | Tytthaspis_sedecimpunctata_578 | 0.164 |
| Brumus_quadripustulatus_7 | Tytthaspis_sedecimpunctata_578 | 0.164 |
| Brumus_quadripustulatus_11 | Tytthaspis_sedecimpunctata_579 | 0.164 |
| Brumus_quadripustulatus_13 | Tytthaspis_sedecimpunctata_579 | 0.164 |
| Brumus_quadripustulatus_14 | Tytthaspis_sedecimpunctata_579 | 0.164 |
| Brumus_quadripustulatus_17 | Tytthaspis_sedecimpunctata_579 | 0.164 |
| Brumus_quadripustulatus_3 | Tytthaspis_sedecimpunctata_579 | 0.164 |
| Brumus_quadripustulatus_4 | Tytthaspis_sedecimpunctata_579 | 0.164 |
| Brumus_quadripustulatus_7 | Tytthaspis_sedecimpunctata_579 | 0.164 |
| Brumus_quadripustulatus_11 | Tytthaspis_sedecimpunctata_581 | 0.164 |
| Brumus_quadripustulatus_13 | Tytthaspis_sedecimpunctata_581 | 0.164 |
| Brumus_quadripustulatus_14 | Tytthaspis_sedecimpunctata_581 | 0.164 |
| Brumus_quadripustulatus_17 | Tytthaspis_sedecimpunctata_581 | 0.164 |
| Brumus_quadripustulatus_3 | Tytthaspis_sedecimpunctata_581 | 0.164 |
| Brumus_quadripustulatus_4 | Tytthaspis_sedecimpunctata_581 | 0.164 |
| Brumus_quadripustulatus_7 | Tytthaspis_sedecimpunctata_581 | 0.164 |
| Brumus_quadripustulatus_11 | Tytthaspis_sedecimpunctata_582 | 0.164 |
| Brumus_quadripustulatus_13 | Tytthaspis_sedecimpunctata_582 | 0.164 |
| Brumus_quadripustulatus_14 | Tytthaspis_sedecimpunctata_582 | 0.164 |
| Brumus_quadripustulatus_17 | Tytthaspis_sedecimpunctata_582 | 0.164 |
| Brumus_quadripustulatus_3 | Tytthaspis_sedecimpunctata_582 | 0.164 |
| Brumus_quadripustulatus_4 | Tytthaspis_sedecimpunctata_582 | 0.164 |
| Brumus_quadripustulatus_7 | Tytthaspis_sedecimpunctata_582 | 0.164 |
| Chilocorus_bipustulatus_23 | Tytthaspis_sedecimpunctata_585 | 0.164 |
| Chilocorus_bipustulatus_19 | Tytthaspis_sedecimpunctata_586 | 0.164 |
| Chilocorus_bipustulatus_22 | Tytthaspis_sedecimpunctata_586 | 0.164 |
| Chilocorus_bipustulatus_18 | Tytthaspis_sedecimpunctata_587 | 0.164 |
| Chilocorus_bipustulatus_20 | Tytthaspis_sedecimpunctata_587 | 0.164 |
| Chilocorus_bipustulatus_19 | Tytthaspis_sedecimpunctata_588 | 0.164 |
| Chilocorus_bipustulatus_22 | Tytthaspis_sedecimpunctata_588 | 0.164 |
| Brumus_quadripustulatus_11 | Tytthaspis_sedecimpunctata_590 | 0.164 |

| | | |
|---|---|---|
| Brumus_quadripustulatus_13 | Tytthaspis_sedecimpunctata_590 | 0.164 |
| Brumus_quadripustulatus_14 | Tytthaspis_sedecimpunctata_590 | 0.164 |
| Brumus_quadripustulatus_17 | Tytthaspis_sedecimpunctata_590 | 0.164 |
| Brumus_quadripustulatus_3 | Tytthaspis_sedecimpunctata_590 | 0.164 |
| Brumus_quadripustulatus_4 | Tytthaspis_sedecimpunctata_590 | 0.164 |
| Brumus_quadripustulatus_7 | Tytthaspis_sedecimpunctata_590 | 0.164 |
| Brumus_quadripustulatus_11 | Tytthaspis_sedecimpunctata_592 | 0.164 |
| Brumus_quadripustulatus_13 | Tytthaspis_sedecimpunctata_592 | 0.164 |
| Brumus_quadripustulatus_14 | Tytthaspis_sedecimpunctata_592 | 0.164 |
| Brumus_quadripustulatus_17 | Tytthaspis_sedecimpunctata_592 | 0.164 |
| Brumus_quadripustulatus_3 | Tytthaspis_sedecimpunctata_592 | 0.164 |
| Brumus_quadripustulatus_4 | Tytthaspis_sedecimpunctata_592 | 0.164 |
| Brumus_quadripustulatus_7 | Tytthaspis_sedecimpunctata_592 | 0.164 |
| Brumus_quadripustulatus_11 | Tytthaspis_sedecimpunctata_594 | 0.164 |
| Brumus_quadripustulatus_13 | Tytthaspis_sedecimpunctata_594 | 0.164 |
| Brumus_quadripustulatus_14 | Tytthaspis_sedecimpunctata_594 | 0.164 |
| Brumus_quadripustulatus_17 | Tytthaspis_sedecimpunctata_594 | 0.164 |
| Brumus_quadripustulatus_3 | Tytthaspis_sedecimpunctata_594 | 0.164 |
| Brumus_quadripustulatus_4 | Tytthaspis_sedecimpunctata_594 | 0.164 |
| Brumus_quadripustulatus_7 | Tytthaspis_sedecimpunctata_594 | 0.164 |
| Brumus_quadripustulatus_11 | Tytthaspis_sedecimpunctata_595 | 0.164 |
| Brumus_quadripustulatus_13 | Tytthaspis_sedecimpunctata_595 | 0.164 |
| Brumus_quadripustulatus_14 | Tytthaspis_sedecimpunctata_595 | 0.164 |
| Brumus_quadripustulatus_17 | Tytthaspis_sedecimpunctata_595 | 0.164 |
| Brumus_quadripustulatus_3 | Tytthaspis_sedecimpunctata_595 | 0.164 |
| Brumus_quadripustulatus_4 | Tytthaspis_sedecimpunctata_595 | 0.164 |
| Brumus_quadripustulatus_7 | Tytthaspis_sedecimpunctata_595 | 0.164 |
| Chilocorus_bipustulatus_19 | Tytthaspis_sedecimpunctata_577 | 0.164 |
| Chilocorus_bipustulatus_22 | Tytthaspis_sedecimpunctata_577 | 0.164 |
| Coccinella_magnifica_228 | Tytthaspis_sedecimpunctata_577 | 0.164 |
| Coccinella_magnifica_229 | Tytthaspis_sedecimpunctata_577 | 0.164 |
| Coccinella_magnifica_230 | Tytthaspis_sedecimpunctata_577 | 0.164 |
| Coccinella_magnifica_231 | Tytthaspis_sedecimpunctata_577 | 0.164 |
| Chilocorus_bipustulatus_19 | Tytthaspis_sedecimpunctata_578 | 0.164 |
| Chilocorus_bipustulatus_22 | Tytthaspis_sedecimpunctata_578 | 0.164 |
| Coccinella_magnifica_228 | Tytthaspis_sedecimpunctata_578 | 0.164 |
| Coccinella_magnifica_229 | Tytthaspis_sedecimpunctata_578 | 0.164 |
| Coccinella_magnifica_230 | Tytthaspis_sedecimpunctata_578 | 0.164 |
| Coccinella_magnifica_231 | Tytthaspis_sedecimpunctata_578 | 0.164 |
| Chilocorus_bipustulatus_19 | Tytthaspis_sedecimpunctata_579 | 0.164 |
| Chilocorus_bipustulatus_22 | Tytthaspis_sedecimpunctata_579 | 0.164 |
| Coccinella_magnifica_228 | Tytthaspis_sedecimpunctata_579 | 0.164 |
| Coccinella_magnifica_229 | Tytthaspis_sedecimpunctata_579 | 0.164 |
| Coccinella_magnifica_230 | Tytthaspis_sedecimpunctata_579 | 0.164 |
| Coccinella_magnifica_231 | Tytthaspis_sedecimpunctata_579 | 0.164 |
| Brumus_quadripustulatus_3 | Tytthaspis_sedecimpunctata_580 | 0.164 |
| Chilocorus_bipustulatus_19 | Tytthaspis_sedecimpunctata_581 | 0.164 |
| Chilocorus_bipustulatus_22 | Tytthaspis_sedecimpunctata_581 | 0.164 |
| Coccinella_magnifica_228 | Tytthaspis_sedecimpunctata_581 | 0.164 |
| Coccinella_magnifica_229 | Tytthaspis_sedecimpunctata_581 | 0.164 |
| Coccinella_magnifica_230 | Tytthaspis_sedecimpunctata_581 | 0.164 |
| Coccinella_magnifica_231 | Tytthaspis_sedecimpunctata_581 | 0.164 |
| Chilocorus_bipustulatus_19 | Tytthaspis_sedecimpunctata_582 | 0.164 |
| Chilocorus_bipustulatus_22 | Tytthaspis_sedecimpunctata_582 | 0.164 |
| Coccinella_magnifica_228 | Tytthaspis_sedecimpunctata_582 | 0.164 |
| Coccinella_magnifica_229 | Tytthaspis_sedecimpunctata_582 | 0.164 |

| | | |
|---|---|---|
| Coccinella_magnifica_230 | Tytthaspis_sedecimpunctata_582 | 0.164 |
| Coccinella_magnifica_231 | Tytthaspis_sedecimpunctata_582 | 0.164 |
| Chilocorus_bipustulatus_23 | Tytthaspis_sedecimpunctata_584 | 0.164 |
| Chilocorus_bipustulatus_24 | Tytthaspis_sedecimpunctata_585 | 0.164 |
| Coccinella_magnifica_228 | Tytthaspis_sedecimpunctata_589 | 0.164 |
| Chilocorus_bipustulatus_19 | Tytthaspis_sedecimpunctata_590 | 0.164 |
| Chilocorus_bipustulatus_22 | Tytthaspis_sedecimpunctata_590 | 0.164 |
| Coccinella_magnifica_228 | Tytthaspis_sedecimpunctata_590 | 0.164 |
| Coccinella_magnifica_229 | Tytthaspis_sedecimpunctata_590 | 0.164 |
| Coccinella_magnifica_230 | Tytthaspis_sedecimpunctata_590 | 0.164 |
| Coccinella_magnifica_231 | Tytthaspis_sedecimpunctata_590 | 0.164 |
| Coccinella_magnifica_228 | Tytthaspis_sedecimpunctata_592 | 0.164 |
| Coccinella_magnifica_229 | Tytthaspis_sedecimpunctata_592 | 0.164 |
| Coccinella_magnifica_230 | Tytthaspis_sedecimpunctata_592 | 0.164 |
| Coccinella_magnifica_231 | Tytthaspis_sedecimpunctata_592 | 0.164 |
| Chilocorus_bipustulatus_19 | Tytthaspis_sedecimpunctata_593 | 0.164 |
| Chilocorus_bipustulatus_22 | Tytthaspis_sedecimpunctata_593 | 0.164 |
| Coccinella_magnifica_228 | Tytthaspis_sedecimpunctata_593 | 0.164 |
| Chilocorus_bipustulatus_19 | Tytthaspis_sedecimpunctata_594 | 0.164 |
| Chilocorus_bipustulatus_22 | Tytthaspis_sedecimpunctata_594 | 0.164 |
| Coccinella_magnifica_228 | Tytthaspis_sedecimpunctata_594 | 0.164 |
| Coccinella_magnifica_229 | Tytthaspis_sedecimpunctata_594 | 0.164 |
| Coccinella_magnifica_230 | Tytthaspis_sedecimpunctata_594 | 0.164 |
| Coccinella_magnifica_231 | Tytthaspis_sedecimpunctata_594 | 0.164 |
| Chilocorus_bipustulatus_19 | Tytthaspis_sedecimpunctata_595 | 0.164 |
| Chilocorus_bipustulatus_22 | Tytthaspis_sedecimpunctata_595 | 0.164 |
| Coccinella_magnifica_228 | Tytthaspis_sedecimpunctata_595 | 0.164 |
| Coccinella_magnifica_229 | Tytthaspis_sedecimpunctata_595 | 0.164 |
| Coccinella_magnifica_230 | Tytthaspis_sedecimpunctata_595 | 0.164 |
| Coccinella_magnifica_231 | Tytthaspis_sedecimpunctata_595 | 0.164 |
| Coccinella_magnifica_228 | Tytthaspis_sedecimpunctata_580 | 0.164 |
| Coccinella_magnifica_229 | Tytthaspis_sedecimpunctata_580 | 0.164 |
| Coccinella_magnifica_230 | Tytthaspis_sedecimpunctata_580 | 0.164 |
| Coccinella_magnifica_231 | Tytthaspis_sedecimpunctata_580 | 0.164 |
| Chilocorus_bipustulatus_24 | Tytthaspis_sedecimpunctata_584 | 0.164 |
| Coccinella_transversoguttata_267 | Tytthaspis_sedecimpunctata_584 | 0.164 |
| Coccinella_transversoguttata_268 | Tytthaspis_sedecimpunctata_584 | 0.164 |
| Coccinella_transversoguttata_269 | Tytthaspis_sedecimpunctata_584 | 0.164 |
| Coccinella_transversoguttata_270 | Tytthaspis_sedecimpunctata_584 | 0.164 |
| Coccinella_transversoguttata_271 | Tytthaspis_sedecimpunctata_584 | 0.164 |
| Adalia_bipunctata_100 | Tytthaspis_sedecimpunctata_588 | 0.164 |
| Coccinella_transversoguttata_265 | Tytthaspis_sedecimpunctata_593 | 0.164 |
| Coccinella_transversoguttata_266 | Tytthaspis_sedecimpunctata_593 | 0.164 |
| Scymnus_apetzi_696 | Scymnus_sinuanodulus_829 | 0.164 |
| Coccinella_transversoguttata_267 | Tytthaspis_sedecimpunctata_583 | 0.164 |
| Coccinella_transversoguttata_268 | Tytthaspis_sedecimpunctata_583 | 0.164 |
| Coccinella_transversoguttata_269 | Tytthaspis_sedecimpunctata_583 | 0.164 |
| Coccinella_transversoguttata_270 | Tytthaspis_sedecimpunctata_583 | 0.164 |
| Coccinella_transversoguttata_271 | Tytthaspis_sedecimpunctata_583 | 0.164 |
| Harmonia_axyridis_354 | Tytthaspis_sedecimpunctata_583 | 0.164 |
| Harmonia_axyridis_356 | Tytthaspis_sedecimpunctata_583 | 0.164 |
| Harmonia_axyridis_357 | Tytthaspis_sedecimpunctata_583 | 0.164 |
| Harmonia_axyridis_358 | Tytthaspis_sedecimpunctata_583 | 0.164 |
| Harmonia_axyridis_359 | Tytthaspis_sedecimpunctata_583 | 0.164 |
| Harmonia_axyridis_360 | Tytthaspis_sedecimpunctata_583 | 0.164 |
| Harmonia_axyridis_361 | Tytthaspis_sedecimpunctata_583 | 0.164 |

| | | |
|---|---|---|
| Harmonia_axyridis_363 | Tytthaspis_sedecimpunctata_583 | 0.164 |
| Harmonia_axyridis_364 | Tytthaspis_sedecimpunctata_583 | 0.164 |
| Chilocorus_stigma_45 | Tytthaspis_sedecimpunctata_584 | 0.164 |
| Adalia_bipunctata_100 | Tytthaspis_sedecimpunctata_585 | 0.164 |
| Harmonia_axyridis_354 | Tytthaspis_sedecimpunctata_592 | 0.164 |
| Harmonia_axyridis_356 | Tytthaspis_sedecimpunctata_592 | 0.164 |
| Harmonia_axyridis_357 | Tytthaspis_sedecimpunctata_592 | 0.164 |
| Harmonia_axyridis_358 | Tytthaspis_sedecimpunctata_592 | 0.164 |
| Harmonia_axyridis_359 | Tytthaspis_sedecimpunctata_592 | 0.164 |
| Harmonia_axyridis_360 | Tytthaspis_sedecimpunctata_592 | 0.164 |
| Harmonia_axyridis_361 | Tytthaspis_sedecimpunctata_592 | 0.164 |
| Harmonia_axyridis_363 | Tytthaspis_sedecimpunctata_592 | 0.164 |
| Harmonia_axyridis_364 | Tytthaspis_sedecimpunctata_592 | 0.164 |
| Harmonia_axyridis_355 | Tytthaspis_sedecimpunctata_577 | 0.164 |
| Harmonia_axyridis_355 | Tytthaspis_sedecimpunctata_578 | 0.164 |
| Harmonia_axyridis_355 | Tytthaspis_sedecimpunctata_579 | 0.164 |
| Harmonia_axyridis_355 | Tytthaspis_sedecimpunctata_581 | 0.164 |
| Harmonia_axyridis_355 | Tytthaspis_sedecimpunctata_582 | 0.164 |
| Harmonia_axyridis_355 | Tytthaspis_sedecimpunctata_590 | 0.164 |
| Coccinella_transversoguttata_267 | Tytthaspis_sedecimpunctata_591 | 0.164 |
| Coccinella_transversoguttata_268 | Tytthaspis_sedecimpunctata_591 | 0.164 |
| Coccinella_transversoguttata_269 | Tytthaspis_sedecimpunctata_591 | 0.164 |
| Coccinella_transversoguttata_270 | Tytthaspis_sedecimpunctata_591 | 0.164 |
| Coccinella_transversoguttata_271 | Tytthaspis_sedecimpunctata_591 | 0.164 |
| Harmonia_axyridis_355 | Tytthaspis_sedecimpunctata_593 | 0.164 |
| Harmonia_axyridis_355 | Tytthaspis_sedecimpunctata_594 | 0.164 |
| Harmonia_axyridis_355 | Tytthaspis_sedecimpunctata_595 | 0.164 |
| Scymnus_apetzi_692 | Scymnus_sinuanodulus_829 | 0.164 |
| Scymnus_apetzi_694 | Scymnus_sinuanodulus_831 | 0.164 |
| Hyperaspis_reppensis_667 | Scymnus_suturalis_843 | 0.164 |
| Hyperaspis_reppensis_667 | Scymnus_suturalis_845 | 0.164 |
| Harmonia_axyridis_355 | Tytthaspis_sedecimpunctata_580 | 0.164 |
| Anatis_ocellata_133 | Tytthaspis_sedecimpunctata_583 | 0.164 |
| Anatis_ocellata_137 | Tytthaspis_sedecimpunctata_583 | 0.164 |
| Hippodamia_alpina_383 | Tytthaspis_sedecimpunctata_583 | 0.164 |
| Anatis_ocellata_133 | Tytthaspis_sedecimpunctata_585 | 0.164 |
| Anatis_ocellata_137 | Tytthaspis_sedecimpunctata_585 | 0.164 |
| Anatis_ocellata_133 | Tytthaspis_sedecimpunctata_592 | 0.164 |
| Anatis_ocellata_137 | Tytthaspis_sedecimpunctata_592 | 0.164 |
| Hyperaspis_reppensis_667 | Scymnus_suturalis_837 | 0.164 |
| Scymnus_pallipediformis_811 | Scymnus_suturalis_838 | 0.164 |
| Scymnus_pallipediformis_811 | Scymnus_suturalis_840 | 0.164 |
| Scymnus_apetzi_691 | Scymnus_suturalis_843 | 0.164 |
| Scymnus_apetzi_696 | Scymnus_suturalis_843 | 0.164 |
| Scymnus_apetzi_691 | Scymnus_suturalis_845 | 0.164 |
| Scymnus_apetzi_696 | Scymnus_suturalis_845 | 0.164 |
| Anatis_ocellata_133 | Tytthaspis_sedecimpunctata_580 | 0.164 |
| Anatis_ocellata_137 | Tytthaspis_sedecimpunctata_580 | 0.164 |
| Scymnus_auritus_699 | Tytthaspis_sedecimpunctata_586 | 0.164 |
| Hippodamia_alpina_383 | Tytthaspis_sedecimpunctata_587 | 0.164 |
| Psyllobora_vigintimaculata_575 | Tytthaspis_sedecimpunctata_588 | 0.164 |
| Psyllobora_vigintimaculata_576 | Tytthaspis_sedecimpunctata_588 | 0.164 |
| Scymnus_auritus_699 | Tytthaspis_sedecimpunctata_588 | 0.164 |
| Psyllobora_vigintiduopunctata_560 | Tytthaspis_sedecimpunctata_589 | 0.164 |
| Scymnus_pallipediformis_811 | Scymnus_suturalis_834 | 0.164 |
| Scymnus_apetzi_691 | Scymnus_suturalis_837 | 0.164 |

| | | |
|---|---|---|
| Scymnus_apetzi_696 | Scymnus_suturalis_837 | 0.164 |
| Scymnus_mimulus_775 | Scymnus_suturalis_838 | 0.164 |
| Scymnus_mimulus_777 | Scymnus_suturalis_838 | 0.164 |
| Scymnus_mimulus_775 | Scymnus_suturalis_840 | 0.164 |
| Scymnus_mimulus_777 | Scymnus_suturalis_840 | 0.164 |
| Hippodamia_alpina_384 | Tytthaspis_sedecimpunctata_586 | 0.164 |
| Psyllobora_vigintiduopunctata_560 | Tytthaspis_sedecimpunctata_586 | 0.164 |
| Coelophora_inaequalis_320 | Tytthaspis_sedecimpunctata_587 | 0.164 |
| Coelophora_inaequalis_321 | Tytthaspis_sedecimpunctata_587 | 0.164 |
| Coelophora_inaequalis_322 | Tytthaspis_sedecimpunctata_587 | 0.164 |
| Psyllobora_vigintiduopunctata_564 | Tytthaspis_sedecimpunctata_587 | 0.164 |
| Psyllobora_vigintiduopunctata_570 | Tytthaspis_sedecimpunctata_587 | 0.164 |
| Psyllobora_vigintiduopunctata_572 | Tytthaspis_sedecimpunctata_587 | 0.164 |
| Hippodamia_alpina_384 | Tytthaspis_sedecimpunctata_588 | 0.164 |
| Scymnus_haemorrhoidalis_747 | Tytthaspis_sedecimpunctata_589 | 0.164 |
| Scymnus_haemorrhoidalis_748 | Tytthaspis_sedecimpunctata_589 | 0.164 |
| Scymnus_mimulus_775 | Scymnus_suturalis_834 | 0.164 |
| Scymnus_mimulus_777 | Scymnus_suturalis_834 | 0.164 |
| Chilocorus_bipustulatus_21 | Tytthaspis_sedecimpunctata_586 | 0.164 |
| Chilocorus_bipustulatus_21 | Tytthaspis_sedecimpunctata_588 | 0.164 |
| Chilocorus_bipustulatus_21 | Tytthaspis_sedecimpunctata_577 | 0.164 |
| Chilocorus_bipustulatus_21 | Tytthaspis_sedecimpunctata_578 | 0.164 |
| Chilocorus_bipustulatus_21 | Tytthaspis_sedecimpunctata_579 | 0.164 |
| Chilocorus_bipustulatus_21 | Tytthaspis_sedecimpunctata_581 | 0.164 |
| Chilocorus_bipustulatus_21 | Tytthaspis_sedecimpunctata_582 | 0.164 |
| Chilocorus_bipustulatus_21 | Tytthaspis_sedecimpunctata_590 | 0.164 |
| Chilocorus_bipustulatus_21 | Tytthaspis_sedecimpunctata_593 | 0.164 |
| Chilocorus_bipustulatus_21 | Tytthaspis_sedecimpunctata_594 | 0.164 |
| Chilocorus_bipustulatus_21 | Tytthaspis_sedecimpunctata_595 | 0.164 |
| Scymnus_mimulus_778 | Scymnus_suturalis_843 | 0.164 |
| Scymnus_mimulus_778 | Scymnus_suturalis_845 | 0.164 |
| Psyllobora_vigintiduopunctata_564 | Tytthaspis_sedecimpunctata_577 | 0.164 |
| Scymnus_haemorrhoidalis_750 | Tytthaspis_sedecimpunctata_577 | 0.164 |
| Scymnus_haemorrhoidalis_751 | Tytthaspis_sedecimpunctata_577 | 0.164 |
| Scymnus_haemorrhoidalis_754 | Tytthaspis_sedecimpunctata_577 | 0.164 |
| Scymnus_haemorrhoidalis_757 | Tytthaspis_sedecimpunctata_577 | 0.164 |
| Scymnus_haemorrhoidalis_758 | Tytthaspis_sedecimpunctata_577 | 0.164 |
| Psyllobora_vigintiduopunctata_564 | Tytthaspis_sedecimpunctata_578 | 0.164 |
| Scymnus_haemorrhoidalis_750 | Tytthaspis_sedecimpunctata_578 | 0.164 |
| Scymnus_haemorrhoidalis_751 | Tytthaspis_sedecimpunctata_578 | 0.164 |
| Scymnus_haemorrhoidalis_754 | Tytthaspis_sedecimpunctata_578 | 0.164 |
| Scymnus_haemorrhoidalis_757 | Tytthaspis_sedecimpunctata_578 | 0.164 |
| Scymnus_haemorrhoidalis_758 | Tytthaspis_sedecimpunctata_578 | 0.164 |
| Psyllobora_vigintiduopunctata_564 | Tytthaspis_sedecimpunctata_579 | 0.164 |
| Scymnus_haemorrhoidalis_750 | Tytthaspis_sedecimpunctata_579 | 0.164 |
| Scymnus_haemorrhoidalis_751 | Tytthaspis_sedecimpunctata_579 | 0.164 |
| Scymnus_haemorrhoidalis_754 | Tytthaspis_sedecimpunctata_579 | 0.164 |
| Scymnus_haemorrhoidalis_757 | Tytthaspis_sedecimpunctata_579 | 0.164 |
| Scymnus_haemorrhoidalis_758 | Tytthaspis_sedecimpunctata_579 | 0.164 |
| Psyllobora_vigintiduopunctata_564 | Tytthaspis_sedecimpunctata_581 | 0.164 |
| Scymnus_haemorrhoidalis_750 | Tytthaspis_sedecimpunctata_581 | 0.164 |
| Scymnus_haemorrhoidalis_751 | Tytthaspis_sedecimpunctata_581 | 0.164 |
| Scymnus_haemorrhoidalis_754 | Tytthaspis_sedecimpunctata_581 | 0.164 |
| Scymnus_haemorrhoidalis_757 | Tytthaspis_sedecimpunctata_581 | 0.164 |
| Scymnus_haemorrhoidalis_758 | Tytthaspis_sedecimpunctata_581 | 0.164 |
| Psyllobora_vigintiduopunctata_564 | Tytthaspis_sedecimpunctata_582 | 0.164 |

| | | |
|---|---|---|
| Scymnus_haemorrhoidalis_750 | Tytthaspis_sedecimpunctata_582 | 0.164 |
| Scymnus_haemorrhoidalis_751 | Tytthaspis_sedecimpunctata_582 | 0.164 |
| Scymnus_haemorrhoidalis_754 | Tytthaspis_sedecimpunctata_582 | 0.164 |
| Scymnus_haemorrhoidalis_757 | Tytthaspis_sedecimpunctata_582 | 0.164 |
| Scymnus_haemorrhoidalis_758 | Tytthaspis_sedecimpunctata_582 | 0.164 |
| Coelophora_inaequalis_320 | Tytthaspis_sedecimpunctata_583 | 0.164 |
| Coelophora_inaequalis_321 | Tytthaspis_sedecimpunctata_583 | 0.164 |
| Coelophora_inaequalis_322 | Tytthaspis_sedecimpunctata_583 | 0.164 |
| Psyllobora_vigintiduopunctata_557 | Tytthaspis_sedecimpunctata_583 | 0.164 |
| Psyllobora_vigintiduopunctata_561 | Tytthaspis_sedecimpunctata_583 | 0.164 |
| Psyllobora_vigintiduopunctata_562 | Tytthaspis_sedecimpunctata_583 | 0.164 |
| Psyllobora_vigintiduopunctata_563 | Tytthaspis_sedecimpunctata_583 | 0.164 |
| Psyllobora_vigintiduopunctata_565 | Tytthaspis_sedecimpunctata_583 | 0.164 |
| Psyllobora_vigintiduopunctata_567 | Tytthaspis_sedecimpunctata_583 | 0.164 |
| Scymnus_haemorrhoidalis_749 | Tytthaspis_sedecimpunctata_583 | 0.164 |
| Scymnus_haemorrhoidalis_752 | Tytthaspis_sedecimpunctata_584 | 0.164 |
| Psyllobora_vigintiduopunctata_557 | Tytthaspis_sedecimpunctata_585 | 0.164 |
| Psyllobora_vigintiduopunctata_561 | Tytthaspis_sedecimpunctata_585 | 0.164 |
| Psyllobora_vigintiduopunctata_562 | Tytthaspis_sedecimpunctata_585 | 0.164 |
| Psyllobora_vigintiduopunctata_563 | Tytthaspis_sedecimpunctata_585 | 0.164 |
| Psyllobora_vigintiduopunctata_565 | Tytthaspis_sedecimpunctata_585 | 0.164 |
| Psyllobora_vigintiduopunctata_567 | Tytthaspis_sedecimpunctata_585 | 0.164 |
| Scymnus_haemorrhoidalis_753 | Tytthaspis_sedecimpunctata_586 | 0.164 |
| Scymnus_haemorrhoidalis_756 | Tytthaspis_sedecimpunctata_586 | 0.164 |
| Scymnus_haemorrhoidalis_747 | Tytthaspis_sedecimpunctata_587 | 0.164 |
| Scymnus_haemorrhoidalis_748 | Tytthaspis_sedecimpunctata_587 | 0.164 |
| Hippodamia_glacialis_408 | Tytthaspis_sedecimpunctata_588 | 0.164 |
| Hippodamia_glacialis_409 | Tytthaspis_sedecimpunctata_588 | 0.164 |
| Scymnus_haemorrhoidalis_752 | Tytthaspis_sedecimpunctata_588 | 0.164 |
| Psyllobora_vigintiduopunctata_564 | Tytthaspis_sedecimpunctata_590 | 0.164 |
| Scymnus_haemorrhoidalis_750 | Tytthaspis_sedecimpunctata_590 | 0.164 |
| Scymnus_haemorrhoidalis_751 | Tytthaspis_sedecimpunctata_590 | 0.164 |
| Scymnus_haemorrhoidalis_754 | Tytthaspis_sedecimpunctata_590 | 0.164 |
| Scymnus_haemorrhoidalis_757 | Tytthaspis_sedecimpunctata_590 | 0.164 |
| Scymnus_haemorrhoidalis_758 | Tytthaspis_sedecimpunctata_590 | 0.164 |
| Scymnus_haemorrhoidalis_752 | Tytthaspis_sedecimpunctata_591 | 0.164 |
| Coelophora_inaequalis_320 | Tytthaspis_sedecimpunctata_592 | 0.164 |
| Coelophora_inaequalis_321 | Tytthaspis_sedecimpunctata_592 | 0.164 |
| Coelophora_inaequalis_322 | Tytthaspis_sedecimpunctata_592 | 0.164 |
| Psyllobora_vigintiduopunctata_557 | Tytthaspis_sedecimpunctata_592 | 0.164 |
| Psyllobora_vigintiduopunctata_561 | Tytthaspis_sedecimpunctata_592 | 0.164 |
| Psyllobora_vigintiduopunctata_562 | Tytthaspis_sedecimpunctata_592 | 0.164 |
| Psyllobora_vigintiduopunctata_563 | Tytthaspis_sedecimpunctata_592 | 0.164 |
| Psyllobora_vigintiduopunctata_565 | Tytthaspis_sedecimpunctata_592 | 0.164 |
| Psyllobora_vigintiduopunctata_567 | Tytthaspis_sedecimpunctata_592 | 0.164 |
| Scymnus_haemorrhoidalis_750 | Tytthaspis_sedecimpunctata_592 | 0.164 |
| Scymnus_haemorrhoidalis_751 | Tytthaspis_sedecimpunctata_592 | 0.164 |
| Scymnus_haemorrhoidalis_754 | Tytthaspis_sedecimpunctata_592 | 0.164 |
| Scymnus_haemorrhoidalis_757 | Tytthaspis_sedecimpunctata_592 | 0.164 |
| Scymnus_haemorrhoidalis_758 | Tytthaspis_sedecimpunctata_592 | 0.164 |
| Psyllobora_vigintiduopunctata_564 | Tytthaspis_sedecimpunctata_593 | 0.164 |
| Scymnus_haemorrhoidalis_749 | Tytthaspis_sedecimpunctata_593 | 0.164 |
| Psyllobora_vigintiduopunctata_564 | Tytthaspis_sedecimpunctata_594 | 0.164 |
| Scymnus_haemorrhoidalis_750 | Tytthaspis_sedecimpunctata_594 | 0.164 |
| Scymnus_haemorrhoidalis_751 | Tytthaspis_sedecimpunctata_594 | 0.164 |
| Scymnus_haemorrhoidalis_754 | Tytthaspis_sedecimpunctata_594 | 0.164 |

| | | |
|---|---|---|
| Scymnus_haemorrhoidalis_757 | Tytthaspis_sedecimpunctata_594 | 0.164 |
| Scymnus_haemorrhoidalis_758 | Tytthaspis_sedecimpunctata_594 | 0.164 |
| Psyllobora_vigintiduopunctata_564 | Tytthaspis_sedecimpunctata_595 | 0.164 |
| Scymnus_haemorrhoidalis_750 | Tytthaspis_sedecimpunctata_595 | 0.164 |
| Scymnus_haemorrhoidalis_751 | Tytthaspis_sedecimpunctata_595 | 0.164 |
| Scymnus_haemorrhoidalis_754 | Tytthaspis_sedecimpunctata_595 | 0.164 |
| Scymnus_haemorrhoidalis_757 | Tytthaspis_sedecimpunctata_595 | 0.164 |
| Scymnus_haemorrhoidalis_758 | Tytthaspis_sedecimpunctata_595 | 0.164 |
| Scymnus_mimulus_778 | Scymnus_suturalis_837 | 0.164 |
| Hyperaspis_proba_661 | Scymnus_suturalis_838 | 0.164 |
| Hyperaspis_proba_661 | Scymnus_suturalis_840 | 0.164 |
| Scymnus_apetzi_695 | Scymnus_sinuanodulus_830 | 0.165 |
| Rhyzobius_chrysomeloides_85 | Scymnus_suturalis_841 | 0.165 |
| Rhyzobius_chrysomeloides_86 | Scymnus_suturalis_841 | 0.165 |
| Rhyzobius_chrysomeloides_87 | Scymnus_suturalis_841 | 0.165 |
| Rhyzobius_chrysomeloides_88 | Scymnus_suturalis_841 | 0.165 |
| Rhyzobius_chrysomeloides_89 | Scymnus_suturalis_841 | 0.165 |
| Rhyzobius_chrysomeloides_90 | Scymnus_suturalis_841 | 0.165 |
| Rhyzobius_chrysomeloides_91 | Scymnus_suturalis_841 | 0.165 |
| Rhyzobius_chrysomeloides_92 | Scymnus_suturalis_841 | 0.165 |
| Rhyzobius_chrysomeloides_93 | Scymnus_suturalis_841 | 0.165 |
| Rhyzobius_chrysomeloides_94 | Scymnus_suturalis_841 | 0.165 |
| Rhyzobius_chrysomeloides_95 | Scymnus_suturalis_841 | 0.165 |
| Rhyzobius_chrysomeloides_85 | Scymnus_suturalis_842 | 0.165 |
| Rhyzobius_chrysomeloides_86 | Scymnus_suturalis_842 | 0.165 |
| Rhyzobius_chrysomeloides_87 | Scymnus_suturalis_842 | 0.165 |
| Rhyzobius_chrysomeloides_88 | Scymnus_suturalis_842 | 0.165 |
| Rhyzobius_chrysomeloides_89 | Scymnus_suturalis_842 | 0.165 |
| Rhyzobius_chrysomeloides_90 | Scymnus_suturalis_842 | 0.165 |
| Rhyzobius_chrysomeloides_91 | Scymnus_suturalis_842 | 0.165 |
| Rhyzobius_chrysomeloides_92 | Scymnus_suturalis_842 | 0.165 |
| Rhyzobius_chrysomeloides_93 | Scymnus_suturalis_842 | 0.165 |
| Rhyzobius_chrysomeloides_94 | Scymnus_suturalis_842 | 0.165 |
| Rhyzobius_chrysomeloides_95 | Scymnus_suturalis_842 | 0.165 |
| Rhyzobius_chrysomeloides_85 | Scymnus_suturalis_844 | 0.165 |
| Rhyzobius_chrysomeloides_86 | Scymnus_suturalis_844 | 0.165 |
| Rhyzobius_chrysomeloides_87 | Scymnus_suturalis_844 | 0.165 |
| Rhyzobius_chrysomeloides_88 | Scymnus_suturalis_844 | 0.165 |
| Rhyzobius_chrysomeloides_89 | Scymnus_suturalis_844 | 0.165 |
| Rhyzobius_chrysomeloides_90 | Scymnus_suturalis_844 | 0.165 |
| Rhyzobius_chrysomeloides_91 | Scymnus_suturalis_844 | 0.165 |
| Rhyzobius_chrysomeloides_92 | Scymnus_suturalis_844 | 0.165 |
| Rhyzobius_chrysomeloides_93 | Scymnus_suturalis_844 | 0.165 |
| Rhyzobius_chrysomeloides_94 | Scymnus_suturalis_844 | 0.165 |
| Rhyzobius_chrysomeloides_95 | Scymnus_suturalis_844 | 0.165 |
| Rhyzobius_chrysomeloides_85 | Scymnus_suturalis_846 | 0.165 |
| Rhyzobius_chrysomeloides_86 | Scymnus_suturalis_846 | 0.165 |
| Rhyzobius_chrysomeloides_87 | Scymnus_suturalis_846 | 0.165 |
| Rhyzobius_chrysomeloides_88 | Scymnus_suturalis_846 | 0.165 |
| Rhyzobius_chrysomeloides_89 | Scymnus_suturalis_846 | 0.165 |
| Rhyzobius_chrysomeloides_90 | Scymnus_suturalis_846 | 0.165 |
| Rhyzobius_chrysomeloides_91 | Scymnus_suturalis_846 | 0.165 |
| Rhyzobius_chrysomeloides_92 | Scymnus_suturalis_846 | 0.165 |
| Rhyzobius_chrysomeloides_93 | Scymnus_suturalis_846 | 0.165 |
| Rhyzobius_chrysomeloides_94 | Scymnus_suturalis_846 | 0.165 |
| Rhyzobius_chrysomeloides_95 | Scymnus_suturalis_846 | 0.165 |

Supplementary Table S4 (continued)

| | | |
|---|---|---|
| Rhyzobius_chrysomeloides_85 | Scymnus_suturalis_847 | 0.165 |
| Rhyzobius_chrysomeloides_86 | Scymnus_suturalis_847 | 0.165 |
| Rhyzobius_chrysomeloides_87 | Scymnus_suturalis_847 | 0.165 |
| Rhyzobius_chrysomeloides_88 | Scymnus_suturalis_847 | 0.165 |
| Rhyzobius_chrysomeloides_89 | Scymnus_suturalis_847 | 0.165 |
| Rhyzobius_chrysomeloides_90 | Scymnus_suturalis_847 | 0.165 |
| Rhyzobius_chrysomeloides_91 | Scymnus_suturalis_847 | 0.165 |
| Rhyzobius_chrysomeloides_92 | Scymnus_suturalis_847 | 0.165 |
| Rhyzobius_chrysomeloides_93 | Scymnus_suturalis_847 | 0.165 |
| Rhyzobius_chrysomeloides_94 | Scymnus_suturalis_847 | 0.165 |
| Rhyzobius_chrysomeloides_95 | Scymnus_suturalis_847 | 0.165 |
| Rhyzobius_chrysomeloides_85 | Scymnus_suturalis_848 | 0.165 |
| Rhyzobius_chrysomeloides_86 | Scymnus_suturalis_848 | 0.165 |
| Rhyzobius_chrysomeloides_87 | Scymnus_suturalis_848 | 0.165 |
| Rhyzobius_chrysomeloides_88 | Scymnus_suturalis_848 | 0.165 |
| Rhyzobius_chrysomeloides_89 | Scymnus_suturalis_848 | 0.165 |
| Rhyzobius_chrysomeloides_90 | Scymnus_suturalis_848 | 0.165 |
| Rhyzobius_chrysomeloides_91 | Scymnus_suturalis_848 | 0.165 |
| Rhyzobius_chrysomeloides_92 | Scymnus_suturalis_848 | 0.165 |
| Rhyzobius_chrysomeloides_93 | Scymnus_suturalis_848 | 0.165 |
| Rhyzobius_chrysomeloides_94 | Scymnus_suturalis_848 | 0.165 |
| Rhyzobius_chrysomeloides_95 | Scymnus_suturalis_848 | 0.165 |
| Hippodamia_notata_421 | Tytthaspis_sedecimpunctata_577 | 0.165 |
| Scymnus_haemorrhoidalis_755 | Tytthaspis_sedecimpunctata_577 | 0.165 |
| Hippodamia_notata_421 | Tytthaspis_sedecimpunctata_578 | 0.165 |
| Scymnus_haemorrhoidalis_755 | Tytthaspis_sedecimpunctata_578 | 0.165 |
| Hippodamia_notata_421 | Tytthaspis_sedecimpunctata_579 | 0.165 |
| Scymnus_haemorrhoidalis_755 | Tytthaspis_sedecimpunctata_579 | 0.165 |
| Coelophora_inaequalis_320 | Tytthaspis_sedecimpunctata_580 | 0.165 |
| Coelophora_inaequalis_321 | Tytthaspis_sedecimpunctata_580 | 0.165 |
| Coelophora_inaequalis_322 | Tytthaspis_sedecimpunctata_580 | 0.165 |
| Hippodamia_tredecimpunctata_459 | Tytthaspis_sedecimpunctata_580 | 0.165 |
| Psyllobora_vigintiduopunctata_557 | Tytthaspis_sedecimpunctata_580 | 0.165 |
| Psyllobora_vigintiduopunctata_561 | Tytthaspis_sedecimpunctata_580 | 0.165 |
| Psyllobora_vigintiduopunctata_562 | Tytthaspis_sedecimpunctata_580 | 0.165 |
| Psyllobora_vigintiduopunctata_563 | Tytthaspis_sedecimpunctata_580 | 0.165 |
| Psyllobora_vigintiduopunctata_565 | Tytthaspis_sedecimpunctata_580 | 0.165 |
| Psyllobora_vigintiduopunctata_567 | Tytthaspis_sedecimpunctata_580 | 0.165 |
| Scymnus_haemorrhoidalis_751 | Tytthaspis_sedecimpunctata_580 | 0.165 |
| Scymnus_haemorrhoidalis_754 | Tytthaspis_sedecimpunctata_580 | 0.165 |
| Scymnus_haemorrhoidalis_757 | Tytthaspis_sedecimpunctata_580 | 0.165 |
| Scymnus_haemorrhoidalis_758 | Tytthaspis_sedecimpunctata_580 | 0.165 |
| Hippodamia_notata_421 | Tytthaspis_sedecimpunctata_581 | 0.165 |
| Scymnus_haemorrhoidalis_755 | Tytthaspis_sedecimpunctata_581 | 0.165 |
| Hippodamia_notata_421 | Tytthaspis_sedecimpunctata_582 | 0.165 |
| Scymnus_haemorrhoidalis_755 | Tytthaspis_sedecimpunctata_582 | 0.165 |
| Psyllobora_vigintiduopunctata_556 | Tytthaspis_sedecimpunctata_583 | 0.165 |
| Hippodamia_notata_421 | Tytthaspis_sedecimpunctata_585 | 0.165 |
| Psyllobora_vigintiduopunctata_556 | Tytthaspis_sedecimpunctata_585 | 0.165 |
| Scymnus_haemorrhoidalis_752 | Tytthaspis_sedecimpunctata_585 | 0.165 |
| Hippodamia_tredecimpunctata_457 | Tytthaspis_sedecimpunctata_587 | 0.165 |
| Hippodamia_tredecimpunctata_458 | Tytthaspis_sedecimpunctata_587 | 0.165 |
| Halyzia_sedecimguttata_353 | Tytthaspis_sedecimpunctata_589 | 0.165 |
| Hippodamia_notata_421 | Tytthaspis_sedecimpunctata_590 | 0.165 |
| Scymnus_haemorrhoidalis_755 | Tytthaspis_sedecimpunctata_590 | 0.165 |
| Hippodamia_notata_421 | Tytthaspis_sedecimpunctata_592 | 0.165 |

Supplementary Table S4 (continued)

| | | |
|---|---|---|
| Psyllobora_vigintiduopunctata_556 | Tytthaspis_sedecimpunctata_592 | 0.165 |
| Scymnus_haemorrhoidalis_755 | Tytthaspis_sedecimpunctata_592 | 0.165 |
| Hippodamia_notata_421 | Tytthaspis_sedecimpunctata_594 | 0.165 |
| Scymnus_haemorrhoidalis_755 | Tytthaspis_sedecimpunctata_594 | 0.165 |
| Hippodamia_notata_421 | Tytthaspis_sedecimpunctata_595 | 0.165 |
| Scymnus_haemorrhoidalis_755 | Tytthaspis_sedecimpunctata_595 | 0.165 |
| Scymnus_nigrinus_784 | Scymnus_sinuanodulus_830 | 0.165 |
| Rhyzobius_chrysomeloides_85 | Scymnus_suturalis_832 | 0.165 |
| Rhyzobius_chrysomeloides_86 | Scymnus_suturalis_832 | 0.165 |
| Rhyzobius_chrysomeloides_87 | Scymnus_suturalis_832 | 0.165 |
| Rhyzobius_chrysomeloides_88 | Scymnus_suturalis_832 | 0.165 |
| Rhyzobius_chrysomeloides_89 | Scymnus_suturalis_832 | 0.165 |
| Rhyzobius_chrysomeloides_90 | Scymnus_suturalis_832 | 0.165 |
| Rhyzobius_chrysomeloides_91 | Scymnus_suturalis_832 | 0.165 |
| Rhyzobius_chrysomeloides_92 | Scymnus_suturalis_832 | 0.165 |
| Rhyzobius_chrysomeloides_93 | Scymnus_suturalis_832 | 0.165 |
| Rhyzobius_chrysomeloides_94 | Scymnus_suturalis_832 | 0.165 |
| Rhyzobius_chrysomeloides_95 | Scymnus_suturalis_832 | 0.165 |
| Hyperaspis_proba_661 | Scymnus_suturalis_834 | 0.165 |
| Rhyzobius_chrysomeloides_85 | Scymnus_suturalis_835 | 0.165 |
| Rhyzobius_chrysomeloides_86 | Scymnus_suturalis_835 | 0.165 |
| Rhyzobius_chrysomeloides_87 | Scymnus_suturalis_835 | 0.165 |
| Rhyzobius_chrysomeloides_88 | Scymnus_suturalis_835 | 0.165 |
| Rhyzobius_chrysomeloides_89 | Scymnus_suturalis_835 | 0.165 |
| Rhyzobius_chrysomeloides_90 | Scymnus_suturalis_835 | 0.165 |
| Rhyzobius_chrysomeloides_91 | Scymnus_suturalis_835 | 0.165 |
| Rhyzobius_chrysomeloides_92 | Scymnus_suturalis_835 | 0.165 |
| Rhyzobius_chrysomeloides_93 | Scymnus_suturalis_835 | 0.165 |
| Rhyzobius_chrysomeloides_94 | Scymnus_suturalis_835 | 0.165 |
| Rhyzobius_chrysomeloides_95 | Scymnus_suturalis_835 | 0.165 |
| Rhyzobius_chrysomeloides_85 | Scymnus_suturalis_836 | 0.165 |
| Rhyzobius_chrysomeloides_86 | Scymnus_suturalis_836 | 0.165 |
| Rhyzobius_chrysomeloides_87 | Scymnus_suturalis_836 | 0.165 |
| Rhyzobius_chrysomeloides_88 | Scymnus_suturalis_836 | 0.165 |
| Rhyzobius_chrysomeloides_89 | Scymnus_suturalis_836 | 0.165 |
| Rhyzobius_chrysomeloides_90 | Scymnus_suturalis_836 | 0.165 |
| Rhyzobius_chrysomeloides_91 | Scymnus_suturalis_836 | 0.165 |
| Rhyzobius_chrysomeloides_92 | Scymnus_suturalis_836 | 0.165 |
| Rhyzobius_chrysomeloides_93 | Scymnus_suturalis_836 | 0.165 |
| Rhyzobius_chrysomeloides_94 | Scymnus_suturalis_836 | 0.165 |
| Rhyzobius_chrysomeloides_95 | Scymnus_suturalis_836 | 0.165 |
| Rhyzobius_chrysomeloides_85 | Scymnus_suturalis_839 | 0.165 |
| Rhyzobius_chrysomeloides_86 | Scymnus_suturalis_839 | 0.165 |
| Rhyzobius_chrysomeloides_87 | Scymnus_suturalis_839 | 0.165 |
| Rhyzobius_chrysomeloides_88 | Scymnus_suturalis_839 | 0.165 |
| Rhyzobius_chrysomeloides_89 | Scymnus_suturalis_839 | 0.165 |
| Rhyzobius_chrysomeloides_90 | Scymnus_suturalis_839 | 0.165 |
| Rhyzobius_chrysomeloides_91 | Scymnus_suturalis_839 | 0.165 |
| Rhyzobius_chrysomeloides_92 | Scymnus_suturalis_839 | 0.165 |
| Rhyzobius_chrysomeloides_93 | Scymnus_suturalis_839 | 0.165 |
| Rhyzobius_chrysomeloides_94 | Scymnus_suturalis_839 | 0.165 |
| Rhyzobius_chrysomeloides_95 | Scymnus_suturalis_839 | 0.165 |
| Anatis_ocellata_136 | Tytthaspis_sedecimpunctata_584 | 0.165 |
| Anatis_ocellata_136 | Tytthaspis_sedecimpunctata_586 | 0.165 |
| Anatis_ocellata_136 | Tytthaspis_sedecimpunctata_591 | 0.165 |
| Ceratomegilla_ulkei_220 | Tytthaspis_sedecimpunctata_577 | 0.165 |

| | | |
|---|---|---|
| Ceratomegilla_ulkei_220 | Tytthaspis_sedecimpunctata_578 | 0.165 |
| Ceratomegilla_ulkei_220 | Tytthaspis_sedecimpunctata_579 | 0.165 |
| Hippodamia_arctica_389 | Tytthaspis_sedecimpunctata_580 | 0.165 |
| Hippodamia_notata_419 | Tytthaspis_sedecimpunctata_580 | 0.165 |
| Hippodamia_notata_429 | Tytthaspis_sedecimpunctata_580 | 0.165 |
| Hippodamia_notata_431 | Tytthaspis_sedecimpunctata_580 | 0.165 |
| Scymnus_haemorrhoidalis_755 | Tytthaspis_sedecimpunctata_580 | 0.165 |
| Ceratomegilla_ulkei_220 | Tytthaspis_sedecimpunctata_581 | 0.165 |
| Ceratomegilla_ulkei_220 | Tytthaspis_sedecimpunctata_582 | 0.165 |
| Ceratomegilla_ulkei_221 | Tytthaspis_sedecimpunctata_583 | 0.165 |
| Hippodamia_tredecimpunctata_459 | Tytthaspis_sedecimpunctata_583 | 0.165 |
| Hippodamia_tredecimpunctata_459 | Tytthaspis_sedecimpunctata_585 | 0.165 |
| Halyzia_sedecimguttata_345 | Tytthaspis_sedecimpunctata_586 | 0.165 |
| Halyzia_sedecimguttata_350 | Tytthaspis_sedecimpunctata_586 | 0.165 |
| Halyzia_sedecimguttata_351 | Tytthaspis_sedecimpunctata_586 | 0.165 |
| Halyzia_sedecimguttata_345 | Tytthaspis_sedecimpunctata_587 | 0.165 |
| Halyzia_sedecimguttata_350 | Tytthaspis_sedecimpunctata_587 | 0.165 |
| Halyzia_sedecimguttata_351 | Tytthaspis_sedecimpunctata_587 | 0.165 |
| Halyzia_sedecimguttata_349 | Tytthaspis_sedecimpunctata_588 | 0.165 |
| Halyzia_sedecimguttata_352 | Tytthaspis_sedecimpunctata_588 | 0.165 |
| Halyzia_sedecimguttata_353 | Tytthaspis_sedecimpunctata_588 | 0.165 |
| Ceratomegilla_ulkei_220 | Tytthaspis_sedecimpunctata_590 | 0.165 |
| Halyzia_sedecimguttata_345 | Tytthaspis_sedecimpunctata_591 | 0.165 |
| Halyzia_sedecimguttata_350 | Tytthaspis_sedecimpunctata_591 | 0.165 |
| Halyzia_sedecimguttata_351 | Tytthaspis_sedecimpunctata_591 | 0.165 |
| Myrrha_octodecimguttata_489 | Tytthaspis_sedecimpunctata_591 | 0.165 |
| Myrrha_octodecimguttata_500 | Tytthaspis_sedecimpunctata_591 | 0.165 |
| Ceratomegilla_ulkei_220 | Tytthaspis_sedecimpunctata_592 | 0.165 |
| Ceratomegilla_ulkei_220 | Tytthaspis_sedecimpunctata_594 | 0.165 |
| Ceratomegilla_ulkei_220 | Tytthaspis_sedecimpunctata_595 | 0.165 |
| Scymnus_nigrinus_784 | Scymnus_sinuanodulus_828 | 0.165 |
| Scymnus_interruptus_759 | Scymnus_sinuanodulus_830 | 0.165 |
| Scymnus_interruptus_761 | Scymnus_sinuanodulus_830 | 0.165 |
| Scymnus_interruptus_763 | Scymnus_sinuanodulus_830 | 0.165 |
| Scymnus_interruptus_764 | Scymnus_sinuanodulus_830 | 0.165 |
| Scymnus_interruptus_766 | Scymnus_sinuanodulus_830 | 0.165 |
| Scymnus_interruptus_769 | Scymnus_sinuanodulus_830 | 0.165 |
| Halyzia_sedecimguttata_349 | Tytthaspis_sedecimpunctata_577 | 0.165 |
| Halyzia_sedecimguttata_352 | Tytthaspis_sedecimpunctata_577 | 0.165 |
| Halyzia_sedecimguttata_353 | Tytthaspis_sedecimpunctata_577 | 0.165 |
| Hippodamia_convergens_396 | Tytthaspis_sedecimpunctata_577 | 0.165 |
| Hippodamia_convergens_400 | Tytthaspis_sedecimpunctata_577 | 0.165 |
| Hippodamia_convergens_403 | Tytthaspis_sedecimpunctata_577 | 0.165 |
| Hippodamia_moesta_410 | Tytthaspis_sedecimpunctata_577 | 0.165 |
| Halyzia_sedecimguttata_349 | Tytthaspis_sedecimpunctata_578 | 0.165 |
| Halyzia_sedecimguttata_352 | Tytthaspis_sedecimpunctata_578 | 0.165 |
| Halyzia_sedecimguttata_353 | Tytthaspis_sedecimpunctata_578 | 0.165 |
| Hippodamia_convergens_396 | Tytthaspis_sedecimpunctata_578 | 0.165 |
| Hippodamia_convergens_400 | Tytthaspis_sedecimpunctata_578 | 0.165 |
| Hippodamia_convergens_403 | Tytthaspis_sedecimpunctata_578 | 0.165 |
| Hippodamia_moesta_410 | Tytthaspis_sedecimpunctata_578 | 0.165 |
| Halyzia_sedecimguttata_349 | Tytthaspis_sedecimpunctata_579 | 0.165 |
| Halyzia_sedecimguttata_352 | Tytthaspis_sedecimpunctata_579 | 0.165 |
| Halyzia_sedecimguttata_353 | Tytthaspis_sedecimpunctata_579 | 0.165 |
| Hippodamia_convergens_396 | Tytthaspis_sedecimpunctata_579 | 0.165 |
| Hippodamia_convergens_400 | Tytthaspis_sedecimpunctata_579 | 0.165 |

| | | |
|---|---|---|
| Hippodamia_convergens_403 | Tytthaspis_sedecimpunctata_579 | 0.165 |
| Hippodamia_moesta_410 | Tytthaspis_sedecimpunctata_579 | 0.165 |
| Ceratomegilla_ulkei_221 | Tytthaspis_sedecimpunctata_580 | 0.165 |
| Halyzia_sedecimguttata_349 | Tytthaspis_sedecimpunctata_581 | 0.165 |
| Halyzia_sedecimguttata_352 | Tytthaspis_sedecimpunctata_581 | 0.165 |
| Halyzia_sedecimguttata_353 | Tytthaspis_sedecimpunctata_581 | 0.165 |
| Hippodamia_convergens_396 | Tytthaspis_sedecimpunctata_581 | 0.165 |
| Hippodamia_convergens_400 | Tytthaspis_sedecimpunctata_581 | 0.165 |
| Hippodamia_convergens_403 | Tytthaspis_sedecimpunctata_581 | 0.165 |
| Hippodamia_moesta_410 | Tytthaspis_sedecimpunctata_581 | 0.165 |
| Halyzia_sedecimguttata_349 | Tytthaspis_sedecimpunctata_582 | 0.165 |
| Halyzia_sedecimguttata_352 | Tytthaspis_sedecimpunctata_582 | 0.165 |
| Halyzia_sedecimguttata_353 | Tytthaspis_sedecimpunctata_582 | 0.165 |
| Hippodamia_convergens_396 | Tytthaspis_sedecimpunctata_582 | 0.165 |
| Hippodamia_convergens_400 | Tytthaspis_sedecimpunctata_582 | 0.165 |
| Hippodamia_convergens_403 | Tytthaspis_sedecimpunctata_582 | 0.165 |
| Hippodamia_moesta_410 | Tytthaspis_sedecimpunctata_582 | 0.165 |
| Hippodamia_convergens_393 | Tytthaspis_sedecimpunctata_583 | 0.165 |
| Hippodamia_convergens_394 | Tytthaspis_sedecimpunctata_583 | 0.165 |
| Hippodamia_convergens_397 | Tytthaspis_sedecimpunctata_583 | 0.165 |
| Hippodamia_convergens_398 | Tytthaspis_sedecimpunctata_583 | 0.165 |
| Hippodamia_convergens_401 | Tytthaspis_sedecimpunctata_583 | 0.165 |
| Hippodamia_convergens_402 | Tytthaspis_sedecimpunctata_583 | 0.165 |
| Hippodamia_tredecimpunctata_459 | Tytthaspis_sedecimpunctata_584 | 0.165 |
| Ceratomegilla_ulkei_222 | Tytthaspis_sedecimpunctata_585 | 0.165 |
| Ceratomegilla_ulkei_223 | Tytthaspis_sedecimpunctata_585 | 0.165 |
| Halyzia_sedecimguttata_349 | Tytthaspis_sedecimpunctata_585 | 0.165 |
| Halyzia_sedecimguttata_352 | Tytthaspis_sedecimpunctata_585 | 0.165 |
| Halyzia_sedecimguttata_353 | Tytthaspis_sedecimpunctata_585 | 0.165 |
| Halyzia_sedecimguttata_346 | Tytthaspis_sedecimpunctata_586 | 0.165 |
| Halyzia_sedecimguttata_347 | Tytthaspis_sedecimpunctata_586 | 0.165 |
| Halyzia_sedecimguttata_348 | Tytthaspis_sedecimpunctata_586 | 0.165 |
| Halyzia_sedecimguttata_346 | Tytthaspis_sedecimpunctata_587 | 0.165 |
| Halyzia_sedecimguttata_347 | Tytthaspis_sedecimpunctata_587 | 0.165 |
| Halyzia_sedecimguttata_348 | Tytthaspis_sedecimpunctata_587 | 0.165 |
| Oenopia_conglobata_522 | Tytthaspis_sedecimpunctata_587 | 0.165 |
| Hippodamia_tredecimpunctata_447 | Tytthaspis_sedecimpunctata_588 | 0.165 |
| Hippodamia_tredecimpunctata_451 | Tytthaspis_sedecimpunctata_588 | 0.165 |
| Hippodamia_tredecimpunctata_454 | Tytthaspis_sedecimpunctata_588 | 0.165 |
| Hippodamia_tredecimpunctata_455 | Tytthaspis_sedecimpunctata_588 | 0.165 |
| Hippodamia_tredecimpunctata_456 | Tytthaspis_sedecimpunctata_588 | 0.165 |
| Hippodamia_parenthesis_439 | Tytthaspis_sedecimpunctata_589 | 0.165 |
| Halyzia_sedecimguttata_349 | Tytthaspis_sedecimpunctata_590 | 0.165 |
| Halyzia_sedecimguttata_352 | Tytthaspis_sedecimpunctata_590 | 0.165 |
| Halyzia_sedecimguttata_353 | Tytthaspis_sedecimpunctata_590 | 0.165 |
| Hippodamia_convergens_396 | Tytthaspis_sedecimpunctata_590 | 0.165 |
| Hippodamia_convergens_400 | Tytthaspis_sedecimpunctata_590 | 0.165 |
| Hippodamia_convergens_403 | Tytthaspis_sedecimpunctata_590 | 0.165 |
| Hippodamia_moesta_410 | Tytthaspis_sedecimpunctata_590 | 0.165 |
| Halyzia_sedecimguttata_346 | Tytthaspis_sedecimpunctata_591 | 0.165 |
| Halyzia_sedecimguttata_347 | Tytthaspis_sedecimpunctata_591 | 0.165 |
| Halyzia_sedecimguttata_348 | Tytthaspis_sedecimpunctata_591 | 0.165 |
| Hippodamia_tredecimpunctata_457 | Tytthaspis_sedecimpunctata_591 | 0.165 |
| Hippodamia_tredecimpunctata_458 | Tytthaspis_sedecimpunctata_591 | 0.165 |
| Halyzia_sedecimguttata_349 | Tytthaspis_sedecimpunctata_592 | 0.165 |
| Halyzia_sedecimguttata_352 | Tytthaspis_sedecimpunctata_592 | 0.165 |

| | | |
|---|---|---|
| Halyzia_sedecimguttata_353 | Tytthaspis_sedecimpunctata_592 | 0.165 |
| Hippodamia_caseyi_391 | Tytthaspis_sedecimpunctata_592 | 0.165 |
| Hippodamia_convergens_393 | Tytthaspis_sedecimpunctata_592 | 0.165 |
| Hippodamia_convergens_394 | Tytthaspis_sedecimpunctata_592 | 0.165 |
| Hippodamia_convergens_397 | Tytthaspis_sedecimpunctata_592 | 0.165 |
| Hippodamia_convergens_398 | Tytthaspis_sedecimpunctata_592 | 0.165 |
| Hippodamia_convergens_399 | Tytthaspis_sedecimpunctata_592 | 0.165 |
| Hippodamia_convergens_401 | Tytthaspis_sedecimpunctata_592 | 0.165 |
| Hippodamia_convergens_402 | Tytthaspis_sedecimpunctata_592 | 0.165 |
| Hippodamia_convergens_404 | Tytthaspis_sedecimpunctata_592 | 0.165 |
| Hippodamia_convergens_405 | Tytthaspis_sedecimpunctata_592 | 0.165 |
| Hippodamia_convergens_406 | Tytthaspis_sedecimpunctata_592 | 0.165 |
| Hippodamia_moesta_411 | Tytthaspis_sedecimpunctata_592 | 0.165 |
| Hippodamia_quinquesignata_443 | Tytthaspis_sedecimpunctata_592 | 0.165 |
| Hippodamia_quinquesignata_444 | Tytthaspis_sedecimpunctata_592 | 0.165 |
| Hippodamia_quinquesignata_445 | Tytthaspis_sedecimpunctata_592 | 0.165 |
| Ceratomegilla_ulkei_222 | Tytthaspis_sedecimpunctata_593 | 0.165 |
| Ceratomegilla_ulkei_223 | Tytthaspis_sedecimpunctata_593 | 0.165 |
| Halyzia_sedecimguttata_349 | Tytthaspis_sedecimpunctata_593 | 0.165 |
| Halyzia_sedecimguttata_352 | Tytthaspis_sedecimpunctata_593 | 0.165 |
| Halyzia_sedecimguttata_353 | Tytthaspis_sedecimpunctata_593 | 0.165 |
| Hippodamia_convergens_396 | Tytthaspis_sedecimpunctata_593 | 0.165 |
| Hippodamia_convergens_400 | Tytthaspis_sedecimpunctata_593 | 0.165 |
| Hippodamia_convergens_403 | Tytthaspis_sedecimpunctata_593 | 0.165 |
| Halyzia_sedecimguttata_349 | Tytthaspis_sedecimpunctata_594 | 0.165 |
| Halyzia_sedecimguttata_352 | Tytthaspis_sedecimpunctata_594 | 0.165 |
| Halyzia_sedecimguttata_353 | Tytthaspis_sedecimpunctata_594 | 0.165 |
| Hippodamia_convergens_396 | Tytthaspis_sedecimpunctata_594 | 0.165 |
| Hippodamia_convergens_400 | Tytthaspis_sedecimpunctata_594 | 0.165 |
| Hippodamia_convergens_403 | Tytthaspis_sedecimpunctata_594 | 0.165 |
| Hippodamia_moesta_410 | Tytthaspis_sedecimpunctata_594 | 0.165 |
| Halyzia_sedecimguttata_349 | Tytthaspis_sedecimpunctata_595 | 0.165 |
| Halyzia_sedecimguttata_352 | Tytthaspis_sedecimpunctata_595 | 0.165 |
| Halyzia_sedecimguttata_353 | Tytthaspis_sedecimpunctata_595 | 0.165 |
| Hippodamia_convergens_396 | Tytthaspis_sedecimpunctata_595 | 0.165 |
| Hippodamia_convergens_400 | Tytthaspis_sedecimpunctata_595 | 0.165 |
| Hippodamia_convergens_403 | Tytthaspis_sedecimpunctata_595 | 0.165 |
| Hippodamia_moesta_410 | Tytthaspis_sedecimpunctata_595 | 0.165 |
| Scymnus_interruptus_767 | Scymnus_sinuanodulus_828 | 0.165 |
| Psyllobora_vigintiduopunctata_559 | Tytthaspis_sedecimpunctata_587 | 0.165 |
| Psyllobora_vigintiduopunctata_566 | Tytthaspis_sedecimpunctata_588 | 0.165 |
| Psyllobora_vigintiduopunctata_566 | Tytthaspis_sedecimpunctata_591 | 0.165 |
| Brumus_quadripustulatus_10 | Tytthaspis_sedecimpunctata_584 | 0.165 |
| Hyperaspis_oregona_659 | Subcoccinella_vigintiquatuorpunctata_619 | 0.165 |
| Psyllobora_vigintiduopunctata_559 | Tytthaspis_sedecimpunctata_577 | 0.165 |
| Psyllobora_vigintiduopunctata_559 | Tytthaspis_sedecimpunctata_578 | 0.165 |
| Psyllobora_vigintiduopunctata_559 | Tytthaspis_sedecimpunctata_579 | 0.165 |
| Psyllobora_vigintiduopunctata_559 | Tytthaspis_sedecimpunctata_581 | 0.165 |
| Psyllobora_vigintiduopunctata_559 | Tytthaspis_sedecimpunctata_582 | 0.165 |
| Psyllobora_vigintiduopunctata_569 | Tytthaspis_sedecimpunctata_583 | 0.165 |
| Psyllobora_vigintiduopunctata_566 | Tytthaspis_sedecimpunctata_585 | 0.165 |
| Psyllobora_vigintiduopunctata_569 | Tytthaspis_sedecimpunctata_585 | 0.165 |
| Psyllobora_vigintiduopunctata_559 | Tytthaspis_sedecimpunctata_590 | 0.165 |
| Psyllobora_vigintiduopunctata_569 | Tytthaspis_sedecimpunctata_592 | 0.165 |
| Psyllobora_vigintiduopunctata_559 | Tytthaspis_sedecimpunctata_593 | 0.165 |
| Psyllobora_vigintiduopunctata_559 | Tytthaspis_sedecimpunctata_594 | 0.165 |

Supplementary Table S4 (continued)

| | | |
|---|---|---|
| Psyllobora_vigintiduopunctata_559 | Tytthaspis_sedecimpunctata_595 | 0.165 |
| Scymnus_apetzi_694 | Scymnus_sinuanodulus_829 | 0.165 |
| Coccinula_quatuordecimpustulata_297 | Tytthaspis_sedecimpunctata_577 | 0.165 |
| Oenopia_conglobata_523 | Tytthaspis_sedecimpunctata_577 | 0.165 |
| Coccinula_quatuordecimpustulata_297 | Tytthaspis_sedecimpunctata_578 | 0.165 |
| Oenopia_conglobata_523 | Tytthaspis_sedecimpunctata_578 | 0.165 |
| Coccinula_quatuordecimpustulata_297 | Tytthaspis_sedecimpunctata_579 | 0.165 |
| Oenopia_conglobata_523 | Tytthaspis_sedecimpunctata_579 | 0.165 |
| Hippodamia_caseyi_391 | Tytthaspis_sedecimpunctata_580 | 0.165 |
| Hippodamia_convergens_393 | Tytthaspis_sedecimpunctata_580 | 0.165 |
| Hippodamia_convergens_394 | Tytthaspis_sedecimpunctata_580 | 0.165 |
| Hippodamia_convergens_397 | Tytthaspis_sedecimpunctata_580 | 0.165 |
| Hippodamia_convergens_398 | Tytthaspis_sedecimpunctata_580 | 0.165 |
| Hippodamia_convergens_399 | Tytthaspis_sedecimpunctata_580 | 0.165 |
| Hippodamia_convergens_401 | Tytthaspis_sedecimpunctata_580 | 0.165 |
| Hippodamia_convergens_402 | Tytthaspis_sedecimpunctata_580 | 0.165 |
| Hippodamia_convergens_404 | Tytthaspis_sedecimpunctata_580 | 0.165 |
| Hippodamia_convergens_405 | Tytthaspis_sedecimpunctata_580 | 0.165 |
| Hippodamia_convergens_406 | Tytthaspis_sedecimpunctata_580 | 0.165 |
| Hippodamia_moesta_411 | Tytthaspis_sedecimpunctata_580 | 0.165 |
| Hippodamia_quinquesignata_443 | Tytthaspis_sedecimpunctata_580 | 0.165 |
| Hippodamia_quinquesignata_444 | Tytthaspis_sedecimpunctata_580 | 0.165 |
| Hippodamia_quinquesignata_445 | Tytthaspis_sedecimpunctata_580 | 0.165 |
| Coccinula_quatuordecimpustulata_297 | Tytthaspis_sedecimpunctata_581 | 0.165 |
| Oenopia_conglobata_523 | Tytthaspis_sedecimpunctata_581 | 0.165 |
| Coccinula_quatuordecimpustulata_297 | Tytthaspis_sedecimpunctata_582 | 0.165 |
| Oenopia_conglobata_523 | Tytthaspis_sedecimpunctata_582 | 0.165 |
| Calvia_decemguttata_195 | Tytthaspis_sedecimpunctata_583 | 0.165 |
| Calvia_decemguttata_199 | Tytthaspis_sedecimpunctata_583 | 0.165 |
| Calvia_decemguttata_200 | Tytthaspis_sedecimpunctata_583 | 0.165 |
| Hippodamia_tredecimpunctata_447 | Tytthaspis_sedecimpunctata_583 | 0.165 |
| Hippodamia_tredecimpunctata_451 | Tytthaspis_sedecimpunctata_583 | 0.165 |
| Hippodamia_tredecimpunctata_454 | Tytthaspis_sedecimpunctata_583 | 0.165 |
| Hippodamia_tredecimpunctata_455 | Tytthaspis_sedecimpunctata_583 | 0.165 |
| Hippodamia_tredecimpunctata_456 | Tytthaspis_sedecimpunctata_583 | 0.165 |
| Halyzia_sedecimguttata_345 | Tytthaspis_sedecimpunctata_584 | 0.165 |
| Halyzia_sedecimguttata_350 | Tytthaspis_sedecimpunctata_584 | 0.165 |
| Halyzia_sedecimguttata_351 | Tytthaspis_sedecimpunctata_584 | 0.165 |
| Hippodamia_tredecimpunctata_447 | Tytthaspis_sedecimpunctata_585 | 0.165 |
| Hippodamia_tredecimpunctata_451 | Tytthaspis_sedecimpunctata_585 | 0.165 |
| Hippodamia_tredecimpunctata_454 | Tytthaspis_sedecimpunctata_585 | 0.165 |
| Hippodamia_tredecimpunctata_455 | Tytthaspis_sedecimpunctata_585 | 0.165 |
| Hippodamia_tredecimpunctata_456 | Tytthaspis_sedecimpunctata_585 | 0.165 |
| Hippodamia_parenthesis_439 | Tytthaspis_sedecimpunctata_586 | 0.165 |
| Hippodamia_parenthesis_433 | Tytthaspis_sedecimpunctata_589 | 0.165 |
| Hippodamia_parenthesis_440 | Tytthaspis_sedecimpunctata_589 | 0.165 |
| Coccinula_quatuordecimpustulata_297 | Tytthaspis_sedecimpunctata_590 | 0.165 |
| Oenopia_conglobata_523 | Tytthaspis_sedecimpunctata_590 | 0.165 |
| Coccinula_quatuordecimpustulata_297 | Tytthaspis_sedecimpunctata_592 | 0.165 |
| Hippodamia_tredecimpunctata_447 | Tytthaspis_sedecimpunctata_592 | 0.165 |
| Hippodamia_tredecimpunctata_451 | Tytthaspis_sedecimpunctata_592 | 0.165 |
| Hippodamia_tredecimpunctata_454 | Tytthaspis_sedecimpunctata_592 | 0.165 |
| Hippodamia_tredecimpunctata_455 | Tytthaspis_sedecimpunctata_592 | 0.165 |
| Hippodamia_tredecimpunctata_456 | Tytthaspis_sedecimpunctata_592 | 0.165 |
| Coccinula_quatuordecimpustulata_297 | Tytthaspis_sedecimpunctata_593 | 0.165 |
| Coccinula_quatuordecimpustulata_297 | Tytthaspis_sedecimpunctata_594 | 0.165 |

Supplementary Table S4 (continued)

| | | |
|---|---|---|
| Oenopia_conglobata_523 | Tytthaspis_sedecimpunctata_594 | 0.165 |
| Coccinula_quatuordecimpustulata_297 | Tytthaspis_sedecimpunctata_595 | 0.165 |
| Oenopia_conglobata_523 | Tytthaspis_sedecimpunctata_595 | 0.165 |
| Scymnus_interruptus_762 | Scymnus_sinuanodulus_828 | 0.165 |
| Scymnus_haemorrhoidalis_755 | Scymnus_suturalis_833 | 0.165 |
| Scymnus_haemorrhoidalis_755 | Scymnus_suturalis_838 | 0.165 |
| Scymnus_apetzi_695 | Scymnus_sinuanodulus_831 | 0.165 |
| Hyperaspis_proba_660 | Scymnus_suturalis_841 | 0.165 |
| Hyperaspis_proba_660 | Scymnus_suturalis_842 | 0.165 |
| Hyperaspis_proba_660 | Scymnus_suturalis_844 | 0.165 |
| Hyperaspis_proba_660 | Scymnus_suturalis_846 | 0.165 |
| Hyperaspis_proba_660 | Scymnus_suturalis_847 | 0.165 |
| Hyperaspis_proba_660 | Scymnus_suturalis_848 | 0.165 |
| Psyllobora_vigintiduopunctata_569 | Tytthaspis_sedecimpunctata_580 | 0.165 |
| Psyllobora_vigintiduopunctata_566 | Tytthaspis_sedecimpunctata_584 | 0.165 |
| Hyperaspis_proba_660 | Scymnus_suturalis_832 | 0.165 |
| Hyperaspis_proba_660 | Scymnus_suturalis_835 | 0.165 |
| Hyperaspis_proba_660 | Scymnus_suturalis_836 | 0.165 |
| Hyperaspis_proba_660 | Scymnus_suturalis_837 | 0.165 |
| Hyperaspis_proba_660 | Scymnus_suturalis_839 | 0.165 |
| Calvia_decemguttata_195 | Tytthaspis_sedecimpunctata_580 | 0.165 |
| Hippodamia_tredecimpunctata_447 | Tytthaspis_sedecimpunctata_580 | 0.165 |
| Hippodamia_tredecimpunctata_451 | Tytthaspis_sedecimpunctata_580 | 0.165 |
| Hippodamia_tredecimpunctata_454 | Tytthaspis_sedecimpunctata_580 | 0.165 |
| Hippodamia_tredecimpunctata_455 | Tytthaspis_sedecimpunctata_580 | 0.165 |
| Hippodamia_tredecimpunctata_456 | Tytthaspis_sedecimpunctata_580 | 0.165 |
| Coccinula_quatuordecimpustulata_297 | Tytthaspis_sedecimpunctata_584 | 0.165 |
| Halyzia_sedecimguttata_346 | Tytthaspis_sedecimpunctata_584 | 0.165 |
| Halyzia_sedecimguttata_347 | Tytthaspis_sedecimpunctata_584 | 0.165 |
| Halyzia_sedecimguttata_348 | Tytthaspis_sedecimpunctata_584 | 0.165 |
| Oenopia_conglobata_522 | Tytthaspis_sedecimpunctata_584 | 0.165 |
| Coccinula_quatuordecimpustulata_306 | Tytthaspis_sedecimpunctata_586 | 0.165 |
| Hippodamia_parenthesis_433 | Tytthaspis_sedecimpunctata_586 | 0.165 |
| Hippodamia_parenthesis_440 | Tytthaspis_sedecimpunctata_586 | 0.165 |
| Hippodamia_parenthesis_432 | Tytthaspis_sedecimpunctata_587 | 0.165 |
| Hippodamia_parenthesis_434 | Tytthaspis_sedecimpunctata_587 | 0.165 |
| Hippodamia_parenthesis_435 | Tytthaspis_sedecimpunctata_587 | 0.165 |
| Hippodamia_parenthesis_437 | Tytthaspis_sedecimpunctata_587 | 0.165 |
| Hippodamia_parenthesis_438 | Tytthaspis_sedecimpunctata_587 | 0.165 |
| Hippodamia_parenthesis_441 | Tytthaspis_sedecimpunctata_587 | 0.165 |
| Hippodamia_parenthesis_442 | Tytthaspis_sedecimpunctata_587 | 0.165 |
| Coccinula_quatuordecimpustulata_292 | Tytthaspis_sedecimpunctata_589 | 0.165 |
| Coccinula_quatuordecimpustulata_293 | Tytthaspis_sedecimpunctata_589 | 0.165 |
| Coccinula_quatuordecimpustulata_307 | Tytthaspis_sedecimpunctata_589 | 0.165 |
| Scymnus_haemorrhoidalis_750 | Scymnus_suturalis_833 | 0.165 |
| Scymnus_haemorrhoidalis_755 | Scymnus_suturalis_834 | 0.165 |
| Scymnus_haemorrhoidalis_750 | Scymnus_suturalis_838 | 0.165 |
| Hippodamia_arctica_390 | Tytthaspis_sedecimpunctata_577 | 0.165 |
| Hippodamia_arctica_390 | Tytthaspis_sedecimpunctata_578 | 0.165 |
| Hippodamia_arctica_390 | Tytthaspis_sedecimpunctata_579 | 0.165 |
| Hippodamia_arctica_390 | Tytthaspis_sedecimpunctata_581 | 0.165 |
| Hippodamia_arctica_390 | Tytthaspis_sedecimpunctata_582 | 0.165 |
| Oenopia_conglobata_524 | Tytthaspis_sedecimpunctata_589 | 0.165 |
| Hippodamia_arctica_390 | Tytthaspis_sedecimpunctata_590 | 0.165 |
| Hippodamia_arctica_390 | Tytthaspis_sedecimpunctata_592 | 0.165 |
| Hippodamia_arctica_390 | Tytthaspis_sedecimpunctata_593 | 0.165 |

| | | |
|---|---|---|
| Hippodamia_arctica_390 | Tytthaspis_sedecimpunctata_594 | 0.165 |
| Hippodamia_arctica_390 | Tytthaspis_sedecimpunctata_595 | 0.165 |
| Scymnus_haemorrhoidalis_749 | Scymnus_suturalis_841 | 0.165 |
| Scymnus_haemorrhoidalis_751 | Scymnus_suturalis_841 | 0.165 |
| Scymnus_haemorrhoidalis_754 | Scymnus_suturalis_841 | 0.165 |
| Scymnus_haemorrhoidalis_757 | Scymnus_suturalis_841 | 0.165 |
| Scymnus_haemorrhoidalis_758 | Scymnus_suturalis_841 | 0.165 |
| Scymnus_haemorrhoidalis_749 | Scymnus_suturalis_842 | 0.165 |
| Scymnus_haemorrhoidalis_751 | Scymnus_suturalis_842 | 0.165 |
| Scymnus_haemorrhoidalis_754 | Scymnus_suturalis_842 | 0.165 |
| Scymnus_haemorrhoidalis_757 | Scymnus_suturalis_842 | 0.165 |
| Scymnus_haemorrhoidalis_758 | Scymnus_suturalis_842 | 0.165 |
| Scymnus_haemorrhoidalis_749 | Scymnus_suturalis_844 | 0.165 |
| Scymnus_haemorrhoidalis_751 | Scymnus_suturalis_844 | 0.165 |
| Scymnus_haemorrhoidalis_754 | Scymnus_suturalis_844 | 0.165 |
| Scymnus_haemorrhoidalis_757 | Scymnus_suturalis_844 | 0.165 |
| Scymnus_haemorrhoidalis_758 | Scymnus_suturalis_844 | 0.165 |
| Scymnus_haemorrhoidalis_749 | Scymnus_suturalis_846 | 0.165 |
| Scymnus_haemorrhoidalis_751 | Scymnus_suturalis_846 | 0.165 |
| Scymnus_haemorrhoidalis_754 | Scymnus_suturalis_846 | 0.165 |
| Scymnus_haemorrhoidalis_757 | Scymnus_suturalis_846 | 0.165 |
| Scymnus_haemorrhoidalis_758 | Scymnus_suturalis_846 | 0.165 |
| Scymnus_haemorrhoidalis_749 | Scymnus_suturalis_847 | 0.165 |
| Scymnus_haemorrhoidalis_751 | Scymnus_suturalis_847 | 0.165 |
| Scymnus_haemorrhoidalis_754 | Scymnus_suturalis_847 | 0.165 |
| Scymnus_haemorrhoidalis_757 | Scymnus_suturalis_847 | 0.165 |
| Scymnus_haemorrhoidalis_758 | Scymnus_suturalis_847 | 0.165 |
| Scymnus_haemorrhoidalis_749 | Scymnus_suturalis_848 | 0.165 |
| Scymnus_haemorrhoidalis_751 | Scymnus_suturalis_848 | 0.165 |
| Scymnus_haemorrhoidalis_754 | Scymnus_suturalis_848 | 0.165 |
| Scymnus_haemorrhoidalis_757 | Scymnus_suturalis_848 | 0.165 |
| Scymnus_haemorrhoidalis_758 | Scymnus_suturalis_848 | 0.165 |
| Coccinula_quatuordecimpustulata_304 | Tytthaspis_sedecimpunctata_577 | 0.165 |
| Coccinula_quatuordecimpustulata_304 | Tytthaspis_sedecimpunctata_578 | 0.165 |
| Coccinula_quatuordecimpustulata_304 | Tytthaspis_sedecimpunctata_579 | 0.165 |
| Coccinula_quatuordecimpustulata_304 | Tytthaspis_sedecimpunctata_581 | 0.165 |
| Coccinula_quatuordecimpustulata_304 | Tytthaspis_sedecimpunctata_582 | 0.165 |
| Macronaemia_episcopalis_477 | Tytthaspis_sedecimpunctata_584 | 0.165 |
| Coccinula_quatuordecimpustulata_304 | Tytthaspis_sedecimpunctata_585 | 0.165 |
| Calvia_quatuordecimguttata_203 | Tytthaspis_sedecimpunctata_586 | 0.165 |
| Coccinula_quatuordecimpustulata_298 | Tytthaspis_sedecimpunctata_586 | 0.165 |
| Coccinula_quatuordecimpustulata_299 | Tytthaspis_sedecimpunctata_586 | 0.165 |
| Coccinula_quatuordecimpustulata_301 | Tytthaspis_sedecimpunctata_586 | 0.165 |
| Coccinula_quatuordecimpustulata_302 | Tytthaspis_sedecimpunctata_586 | 0.165 |
| Coccinula_quatuordecimpustulata_303 | Tytthaspis_sedecimpunctata_586 | 0.165 |
| Coccinula_quatuordecimpustulata_309 | Tytthaspis_sedecimpunctata_586 | 0.165 |
| Coccinula_quatuordecimpustulata_294 | Tytthaspis_sedecimpunctata_588 | 0.165 |
| Coccinula_quatuordecimpustulata_295 | Tytthaspis_sedecimpunctata_588 | 0.165 |
| Coccinula_quatuordecimpustulata_308 | Tytthaspis_sedecimpunctata_588 | 0.165 |
| Calvia_quatuordecimguttata_205 | Tytthaspis_sedecimpunctata_589 | 0.165 |
| Coccinula_quatuordecimpustulata_304 | Tytthaspis_sedecimpunctata_590 | 0.165 |
| Coccinula_quatuordecimpustulata_292 | Tytthaspis_sedecimpunctata_591 | 0.165 |
| Coccinula_quatuordecimpustulata_293 | Tytthaspis_sedecimpunctata_591 | 0.165 |
| Coccinula_quatuordecimpustulata_304 | Tytthaspis_sedecimpunctata_592 | 0.165 |
| Coccinula_quatuordecimpustulata_304 | Tytthaspis_sedecimpunctata_593 | 0.165 |
| Coccinula_quatuordecimpustulata_304 | Tytthaspis_sedecimpunctata_594 | 0.165 |

| | | |
|---|---|---|
| Coccinula_quatuordecimpustulata_304 | Tytthaspis_sedecimpunctata_595 | 0.165 |
| Scymnus_auritus_698 | Scymnus_sinuanodulus_830 | 0.165 |
| Scymnus_auritus_700 | Scymnus_sinuanodulus_830 | 0.165 |
| Scymnus_haemorrhoidalis_749 | Scymnus_suturalis_832 | 0.165 |
| Scymnus_haemorrhoidalis_751 | Scymnus_suturalis_832 | 0.165 |
| Scymnus_haemorrhoidalis_754 | Scymnus_suturalis_832 | 0.165 |
| Scymnus_haemorrhoidalis_757 | Scymnus_suturalis_832 | 0.165 |
| Scymnus_haemorrhoidalis_758 | Scymnus_suturalis_832 | 0.165 |
| Scymnus_haemorrhoidalis_750 | Scymnus_suturalis_834 | 0.165 |
| Scymnus_haemorrhoidalis_749 | Scymnus_suturalis_835 | 0.165 |
| Scymnus_haemorrhoidalis_751 | Scymnus_suturalis_835 | 0.165 |
| Scymnus_haemorrhoidalis_754 | Scymnus_suturalis_835 | 0.165 |
| Scymnus_haemorrhoidalis_757 | Scymnus_suturalis_835 | 0.165 |
| Scymnus_haemorrhoidalis_758 | Scymnus_suturalis_835 | 0.165 |
| Scymnus_haemorrhoidalis_749 | Scymnus_suturalis_836 | 0.165 |
| Scymnus_haemorrhoidalis_751 | Scymnus_suturalis_836 | 0.165 |
| Scymnus_haemorrhoidalis_754 | Scymnus_suturalis_836 | 0.165 |
| Scymnus_haemorrhoidalis_757 | Scymnus_suturalis_836 | 0.165 |
| Scymnus_haemorrhoidalis_758 | Scymnus_suturalis_836 | 0.165 |
| Scymnus_haemorrhoidalis_749 | Scymnus_suturalis_837 | 0.165 |
| Scymnus_haemorrhoidalis_751 | Scymnus_suturalis_837 | 0.165 |
| Scymnus_haemorrhoidalis_754 | Scymnus_suturalis_837 | 0.165 |
| Scymnus_haemorrhoidalis_757 | Scymnus_suturalis_837 | 0.165 |
| Scymnus_haemorrhoidalis_758 | Scymnus_suturalis_837 | 0.165 |
| Scymnus_haemorrhoidalis_749 | Scymnus_suturalis_839 | 0.165 |
| Scymnus_haemorrhoidalis_751 | Scymnus_suturalis_839 | 0.165 |
| Scymnus_haemorrhoidalis_754 | Scymnus_suturalis_839 | 0.165 |
| Scymnus_haemorrhoidalis_757 | Scymnus_suturalis_839 | 0.165 |
| Scymnus_haemorrhoidalis_758 | Scymnus_suturalis_839 | 0.165 |
| Oenopia_conglobata_524 | Tytthaspis_sedecimpunctata_580 | 0.165 |
| Oenopia_conglobata_524 | Tytthaspis_sedecimpunctata_588 | 0.165 |
| Coccinula_quatuordecimpustulata_300 | Tytthaspis_sedecimpunctata_577 | 0.165 |
| Coccinula_quatuordecimpustulata_300 | Tytthaspis_sedecimpunctata_578 | 0.165 |
| Coccinula_quatuordecimpustulata_300 | Tytthaspis_sedecimpunctata_579 | 0.165 |
| Coccinula_quatuordecimpustulata_300 | Tytthaspis_sedecimpunctata_581 | 0.165 |
| Coccinula_quatuordecimpustulata_300 | Tytthaspis_sedecimpunctata_582 | 0.165 |
| Coccinula_quatuordecimpustulata_304 | Tytthaspis_sedecimpunctata_584 | 0.165 |
| Hippodamia_parenthesis_432 | Tytthaspis_sedecimpunctata_584 | 0.165 |
| Hippodamia_parenthesis_434 | Tytthaspis_sedecimpunctata_584 | 0.165 |
| Hippodamia_parenthesis_435 | Tytthaspis_sedecimpunctata_584 | 0.165 |
| Hippodamia_parenthesis_437 | Tytthaspis_sedecimpunctata_584 | 0.165 |
| Hippodamia_parenthesis_438 | Tytthaspis_sedecimpunctata_584 | 0.165 |
| Hippodamia_parenthesis_441 | Tytthaspis_sedecimpunctata_584 | 0.165 |
| Hippodamia_parenthesis_442 | Tytthaspis_sedecimpunctata_584 | 0.165 |
| Coccinula_quatuordecimpustulata_300 | Tytthaspis_sedecimpunctata_585 | 0.165 |
| Coccinula_quatuordecimpustulata_300 | Tytthaspis_sedecimpunctata_590 | 0.165 |
| Hippodamia_parenthesis_432 | Tytthaspis_sedecimpunctata_591 | 0.165 |
| Hippodamia_parenthesis_434 | Tytthaspis_sedecimpunctata_591 | 0.165 |
| Hippodamia_parenthesis_435 | Tytthaspis_sedecimpunctata_591 | 0.165 |
| Hippodamia_parenthesis_437 | Tytthaspis_sedecimpunctata_591 | 0.165 |
| Hippodamia_parenthesis_438 | Tytthaspis_sedecimpunctata_591 | 0.165 |
| Hippodamia_parenthesis_441 | Tytthaspis_sedecimpunctata_591 | 0.165 |
| Hippodamia_parenthesis_442 | Tytthaspis_sedecimpunctata_591 | 0.165 |
| Coccinula_quatuordecimpustulata_300 | Tytthaspis_sedecimpunctata_592 | 0.165 |
| Coccinula_quatuordecimpustulata_300 | Tytthaspis_sedecimpunctata_593 | 0.165 |
| Coccinula_quatuordecimpustulata_300 | Tytthaspis_sedecimpunctata_594 | 0.165 |

| | | |
|---|---|---|
| Coccinula_quatuordecimpustulata_300 | Tytthaspis_sedecimpunctata_595 | 0.165 |
| Scymnus_haemorrhoidalis_749 | Scymnus_suturalis_840 | 0.165 |
| Scymnus_haemorrhoidalis_751 | Scymnus_suturalis_840 | 0.165 |
| Scymnus_haemorrhoidalis_754 | Scymnus_suturalis_840 | 0.165 |
| Scymnus_haemorrhoidalis_757 | Scymnus_suturalis_840 | 0.165 |
| Scymnus_haemorrhoidalis_758 | Scymnus_suturalis_840 | 0.165 |
| Psyllobora_vigintiduopunctata_558 | Tytthaspis_sedecimpunctata_593 | 0.165 |
| Oenopia_conglobata_524 | Tytthaspis_sedecimpunctata_585 | 0.165 |
| Scymnus_interruptus_759 | Scymnus_sinuanodulus_831 | 0.165 |
| Scymnus_interruptus_761 | Scymnus_sinuanodulus_831 | 0.165 |
| Scymnus_interruptus_763 | Scymnus_sinuanodulus_831 | 0.165 |
| Scymnus_interruptus_764 | Scymnus_sinuanodulus_831 | 0.165 |
| Scymnus_interruptus_766 | Scymnus_sinuanodulus_831 | 0.165 |
| Scymnus_interruptus_769 | Scymnus_sinuanodulus_831 | 0.165 |
| Psyllobora_vigintiduopunctata_558 | Tytthaspis_sedecimpunctata_584 | 0.165 |
| Psyllobora_vigintiduopunctata_568 | Tytthaspis_sedecimpunctata_584 | 0.165 |
| Coccinula_quatuordecimpustulata_300 | Tytthaspis_sedecimpunctata_584 | 0.165 |
| Scymnus_loewii_772 | Scymnus_rubromaculatus_822 | 0.165 |
| Scymnus_loewii_773 | Scymnus_rubromaculatus_822 | 0.165 |
| Scymnus_loewii_772 | Scymnus_rubromaculatus_823 | 0.165 |
| Scymnus_loewii_773 | Scymnus_rubromaculatus_823 | 0.165 |
| Scymnus_loewii_772 | Scymnus_rubromaculatus_824 | 0.165 |
| Scymnus_loewii_773 | Scymnus_rubromaculatus_824 | 0.165 |
| Scymnus_loewii_772 | Scymnus_rubromaculatus_825 | 0.165 |
| Scymnus_loewii_773 | Scymnus_rubromaculatus_825 | 0.165 |
| Scymnus_loewii_772 | Scymnus_rubromaculatus_826 | 0.165 |
| Scymnus_loewii_773 | Scymnus_rubromaculatus_826 | 0.165 |
| Scymnus_loewii_772 | Scymnus_rubromaculatus_827 | 0.165 |
| Scymnus_loewii_773 | Scymnus_rubromaculatus_827 | 0.165 |
| Scymnus_apetzi_696 | Scymnus_sinuanodulus_830 | 0.165 |
| Scymnus_apetzi_695 | Scymnus_sinuanodulus_829 | 0.165 |
| Scymnus_nubilus_803 | Scymnus_rubromaculatus_822 | 0.165 |
| Scymnus_nubilus_804 | Scymnus_rubromaculatus_822 | 0.165 |
| Scymnus_nubilus_809 | Scymnus_rubromaculatus_822 | 0.165 |
| Scymnus_nubilus_803 | Scymnus_rubromaculatus_823 | 0.165 |
| Scymnus_nubilus_804 | Scymnus_rubromaculatus_823 | 0.165 |
| Scymnus_nubilus_809 | Scymnus_rubromaculatus_823 | 0.165 |
| Scymnus_nubilus_803 | Scymnus_rubromaculatus_824 | 0.165 |
| Scymnus_nubilus_804 | Scymnus_rubromaculatus_824 | 0.165 |
| Scymnus_nubilus_809 | Scymnus_rubromaculatus_824 | 0.165 |
| Scymnus_nubilus_803 | Scymnus_rubromaculatus_825 | 0.165 |
| Scymnus_nubilus_804 | Scymnus_rubromaculatus_825 | 0.165 |
| Scymnus_nubilus_809 | Scymnus_rubromaculatus_825 | 0.165 |
| Scymnus_nubilus_803 | Scymnus_rubromaculatus_826 | 0.165 |
| Scymnus_nubilus_804 | Scymnus_rubromaculatus_826 | 0.165 |
| Scymnus_nubilus_809 | Scymnus_rubromaculatus_826 | 0.165 |
| Scymnus_nubilus_803 | Scymnus_rubromaculatus_827 | 0.165 |
| Scymnus_nubilus_804 | Scymnus_rubromaculatus_827 | 0.165 |
| Scymnus_nubilus_809 | Scymnus_rubromaculatus_827 | 0.165 |
| Myrrha_octodecimguttata_499 | Vibidia_duodecimguttata_599 | 0.165 |
| Myrrha_octodecimguttata_499 | Vibidia_duodecimguttata_602 | 0.165 |
| Scymnus_apetzi_695 | Scymnus_suturalis_833 | 0.166 |
| Scymnus_auritus_700 | Scymnus_sinuanodulus_831 | 0.166 |
| Scymnus_apetzi_693 | Scymnus_suturalis_841 | 0.166 |
| Scymnus_apetzi_693 | Scymnus_suturalis_842 | 0.166 |
| Scymnus_apetzi_693 | Scymnus_suturalis_844 | 0.166 |

| | | |
|---|---|---|
| Scymnus_apetzi_693 | Scymnus_suturalis_846 | 0.166 |
| Scymnus_apetzi_693 | Scymnus_suturalis_847 | 0.166 |
| Scymnus_apetzi_693 | Scymnus_suturalis_848 | 0.166 |
| Scymnus_apetzi_693 | Scymnus_suturalis_832 | 0.166 |
| Scymnus_apetzi_693 | Scymnus_suturalis_835 | 0.166 |
| Scymnus_apetzi_693 | Scymnus_suturalis_836 | 0.166 |
| Scymnus_apetzi_693 | Scymnus_suturalis_839 | 0.166 |
| Myrrha_octodecimguttata_485 | Vibidia_duodecimguttata_596 | 0.166 |
| Myrrha_octodecimguttata_486 | Vibidia_duodecimguttata_596 | 0.166 |
| Myrrha_octodecimguttata_487 | Vibidia_duodecimguttata_596 | 0.166 |
| Myrrha_octodecimguttata_490 | Vibidia_duodecimguttata_596 | 0.166 |
| Myrrha_octodecimguttata_494 | Vibidia_duodecimguttata_596 | 0.166 |
| Myrrha_octodecimguttata_496 | Vibidia_duodecimguttata_596 | 0.166 |
| Myrrha_octodecimguttata_497 | Vibidia_duodecimguttata_596 | 0.166 |
| Myrrha_octodecimguttata_501 | Vibidia_duodecimguttata_596 | 0.166 |
| Myrrha_octodecimguttata_502 | Vibidia_duodecimguttata_596 | 0.166 |
| Calvia_quatuordecimguttata_202 | Vibidia_duodecimguttata_597 | 0.166 |
| Calvia_quatuordecimguttata_210 | Vibidia_duodecimguttata_597 | 0.166 |
| Myrrha_octodecimguttata_488 | Vibidia_duodecimguttata_597 | 0.166 |
| Myrrha_octodecimguttata_491 | Vibidia_duodecimguttata_597 | 0.166 |
| Myrrha_octodecimguttata_492 | Vibidia_duodecimguttata_597 | 0.166 |
| Myrrha_octodecimguttata_499 | Vibidia_duodecimguttata_597 | 0.166 |
| Calvia_quatuordecimguttata_202 | Vibidia_duodecimguttata_600 | 0.166 |
| Calvia_quatuordecimguttata_210 | Vibidia_duodecimguttata_600 | 0.166 |
| Myrrha_octodecimguttata_488 | Vibidia_duodecimguttata_600 | 0.166 |
| Myrrha_octodecimguttata_491 | Vibidia_duodecimguttata_600 | 0.166 |
| Myrrha_octodecimguttata_492 | Vibidia_duodecimguttata_600 | 0.166 |
| Myrrha_octodecimguttata_499 | Vibidia_duodecimguttata_600 | 0.166 |
| Myrrha_octodecimguttata_485 | Vibidia_duodecimguttata_601 | 0.166 |
| Myrrha_octodecimguttata_486 | Vibidia_duodecimguttata_601 | 0.166 |
| Myrrha_octodecimguttata_487 | Vibidia_duodecimguttata_601 | 0.166 |
| Myrrha_octodecimguttata_490 | Vibidia_duodecimguttata_601 | 0.166 |
| Myrrha_octodecimguttata_494 | Vibidia_duodecimguttata_601 | 0.166 |
| Myrrha_octodecimguttata_496 | Vibidia_duodecimguttata_601 | 0.166 |
| Myrrha_octodecimguttata_497 | Vibidia_duodecimguttata_601 | 0.166 |
| Myrrha_octodecimguttata_501 | Vibidia_duodecimguttata_601 | 0.166 |
| Myrrha_octodecimguttata_502 | Vibidia_duodecimguttata_601 | 0.166 |
| Calvia_quatuordecimguttata_211 | Vibidia_duodecimguttata_596 | 0.166 |
| Calvia_quatuordecimguttata_212 | Vibidia_duodecimguttata_596 | 0.166 |
| Calvia_quatuordecimguttata_213 | Vibidia_duodecimguttata_596 | 0.166 |
| Calvia_quatuordecimguttata_214 | Vibidia_duodecimguttata_596 | 0.166 |
| Calvia_quatuordecimguttata_215 | Vibidia_duodecimguttata_596 | 0.166 |
| Myrrha_octodecimguttata_495 | Vibidia_duodecimguttata_596 | 0.166 |
| Myrrha_octodecimguttata_493 | Vibidia_duodecimguttata_599 | 0.166 |
| Myrrha_octodecimguttata_498 | Vibidia_duodecimguttata_599 | 0.166 |
| Myrrha_octodecimguttata_495 | Vibidia_duodecimguttata_601 | 0.166 |
| Myrrha_octodecimguttata_493 | Vibidia_duodecimguttata_602 | 0.166 |
| Myrrha_octodecimguttata_498 | Vibidia_duodecimguttata_602 | 0.166 |
| Calvia_quatuordecimguttata_202 | Vibidia_duodecimguttata_603 | 0.166 |
| Calvia_quatuordecimguttata_210 | Vibidia_duodecimguttata_603 | 0.166 |
| Scymnus_auritus_699 | Scymnus_sinuanodulus_831 | 0.166 |
| Myrrha_octodecimguttata_493 | Vibidia_duodecimguttata_597 | 0.166 |
| Myrrha_octodecimguttata_498 | Vibidia_duodecimguttata_597 | 0.166 |
| Psyllobora_vigintimaculata_574 | Vibidia_duodecimguttata_597 | 0.166 |
| Myrrha_octodecimguttata_493 | Vibidia_duodecimguttata_600 | 0.166 |
| Myrrha_octodecimguttata_498 | Vibidia_duodecimguttata_600 | 0.166 |

| | | |
|---|---|---|
| Psyllobora_vigintimaculata_574 | Vibidia_duodecimguttata_600 | 0.166 |
| Brumus_quadripustulatus_5 | Scymnus_suturalis_841 | 0.166 |
| Brumus_quadripustulatus_5 | Scymnus_suturalis_842 | 0.166 |
| Brumus_quadripustulatus_5 | Scymnus_suturalis_844 | 0.166 |
| Brumus_quadripustulatus_5 | Scymnus_suturalis_846 | 0.166 |
| Brumus_quadripustulatus_5 | Scymnus_suturalis_847 | 0.166 |
| Brumus_quadripustulatus_5 | Scymnus_suturalis_848 | 0.166 |
| Brumus_quadripustulatus_5 | Scymnus_suturalis_832 | 0.166 |
| Brumus_quadripustulatus_5 | Scymnus_suturalis_835 | 0.166 |
| Brumus_quadripustulatus_5 | Scymnus_suturalis_836 | 0.166 |
| Brumus_quadripustulatus_5 | Scymnus_suturalis_839 | 0.166 |
| Hyperaspis_proba_661 | Tytthaspis_sedecimpunctata_589 | 0.166 |
| Hyperaspis_proba_661 | Tytthaspis_sedecimpunctata_593 | 0.166 |
| Chilocorus_stigma_43 | Tytthaspis_sedecimpunctata_577 | 0.166 |
| Chilocorus_stigma_43 | Tytthaspis_sedecimpunctata_578 | 0.166 |
| Chilocorus_stigma_43 | Tytthaspis_sedecimpunctata_579 | 0.166 |
| Hyperaspis_proba_661 | Tytthaspis_sedecimpunctata_580 | 0.166 |
| Chilocorus_stigma_43 | Tytthaspis_sedecimpunctata_581 | 0.166 |
| Chilocorus_stigma_43 | Tytthaspis_sedecimpunctata_582 | 0.166 |
| Chilocorus_stigma_44 | Tytthaspis_sedecimpunctata_583 | 0.166 |
| Chilocorus_stigma_46 | Tytthaspis_sedecimpunctata_583 | 0.166 |
| Chilocorus_stigma_47 | Tytthaspis_sedecimpunctata_583 | 0.166 |
| Chilocorus_stigma_43 | Tytthaspis_sedecimpunctata_585 | 0.166 |
| Hyperaspis_proba_661 | Tytthaspis_sedecimpunctata_587 | 0.166 |
| Chilocorus_stigma_43 | Tytthaspis_sedecimpunctata_590 | 0.166 |
| Chilocorus_stigma_43 | Tytthaspis_sedecimpunctata_592 | 0.166 |
| Chilocorus_stigma_43 | Tytthaspis_sedecimpunctata_594 | 0.166 |
| Chilocorus_stigma_43 | Tytthaspis_sedecimpunctata_595 | 0.166 |
| Chilocorus_stigma_44 | Tytthaspis_sedecimpunctata_580 | 0.166 |
| Chilocorus_stigma_46 | Tytthaspis_sedecimpunctata_580 | 0.166 |
| Chilocorus_stigma_47 | Tytthaspis_sedecimpunctata_580 | 0.166 |
| Brumus_quadripustulatus_15 | Tytthaspis_sedecimpunctata_583 | 0.166 |
| Brumus_quadripustulatus_16 | Tytthaspis_sedecimpunctata_583 | 0.166 |
| Brumus_quadripustulatus_6 | Tytthaspis_sedecimpunctata_583 | 0.166 |
| Brumus_quadripustulatus_8 | Tytthaspis_sedecimpunctata_583 | 0.166 |
| Brumus_quadripustulatus_11 | Tytthaspis_sedecimpunctata_584 | 0.166 |
| Brumus_quadripustulatus_13 | Tytthaspis_sedecimpunctata_584 | 0.166 |
| Brumus_quadripustulatus_14 | Tytthaspis_sedecimpunctata_584 | 0.166 |
| Brumus_quadripustulatus_17 | Tytthaspis_sedecimpunctata_584 | 0.166 |
| Brumus_quadripustulatus_3 | Tytthaspis_sedecimpunctata_584 | 0.166 |
| Brumus_quadripustulatus_4 | Tytthaspis_sedecimpunctata_584 | 0.166 |
| Brumus_quadripustulatus_7 | Tytthaspis_sedecimpunctata_584 | 0.166 |
| Chilocorus_bipustulatus_23 | Tytthaspis_sedecimpunctata_587 | 0.166 |
| Chilocorus_bipustulatus_19 | Tytthaspis_sedecimpunctata_589 | 0.166 |
| Chilocorus_bipustulatus_22 | Tytthaspis_sedecimpunctata_589 | 0.166 |
| Brumus_quadripustulatus_15 | Tytthaspis_sedecimpunctata_580 | 0.166 |
| Brumus_quadripustulatus_16 | Tytthaspis_sedecimpunctata_580 | 0.166 |
| Brumus_quadripustulatus_6 | Tytthaspis_sedecimpunctata_580 | 0.166 |
| Brumus_quadripustulatus_8 | Tytthaspis_sedecimpunctata_580 | 0.166 |
| Coccinella_magnifica_228 | Tytthaspis_sedecimpunctata_584 | 0.166 |
| Coccinella_magnifica_229 | Tytthaspis_sedecimpunctata_584 | 0.166 |
| Coccinella_magnifica_230 | Tytthaspis_sedecimpunctata_584 | 0.166 |
| Coccinella_magnifica_231 | Tytthaspis_sedecimpunctata_584 | 0.166 |
| Coccinella_magnifica_228 | Tytthaspis_sedecimpunctata_586 | 0.166 |
| Coccinella_magnifica_229 | Tytthaspis_sedecimpunctata_586 | 0.166 |
| Coccinella_magnifica_230 | Tytthaspis_sedecimpunctata_586 | 0.166 |

| | | |
|---|---|---|
| Coccinella_magnifica_231 | Tytthaspis_sedecimpunctata_586 | 0.166 |
| Chilocorus_bipustulatus_24 | Tytthaspis_sedecimpunctata_587 | 0.166 |
| Coccinella_magnifica_228 | Tytthaspis_sedecimpunctata_588 | 0.166 |
| Coccinella_magnifica_229 | Tytthaspis_sedecimpunctata_588 | 0.166 |
| Coccinella_magnifica_230 | Tytthaspis_sedecimpunctata_588 | 0.166 |
| Coccinella_magnifica_231 | Tytthaspis_sedecimpunctata_588 | 0.166 |
| Chilocorus_bipustulatus_18 | Tytthaspis_sedecimpunctata_591 | 0.166 |
| Chilocorus_bipustulatus_20 | Tytthaspis_sedecimpunctata_591 | 0.166 |
| Chilocorus_nigritus_27 | Scymnus_suturalis_843 | 0.166 |
| Chilocorus_nigritus_27 | Scymnus_suturalis_845 | 0.166 |
| Coccinella_magnifica_228 | Tytthaspis_sedecimpunctata_583 | 0.166 |
| Coccinella_magnifica_229 | Tytthaspis_sedecimpunctata_583 | 0.166 |
| Coccinella_magnifica_230 | Tytthaspis_sedecimpunctata_583 | 0.166 |
| Coccinella_magnifica_231 | Tytthaspis_sedecimpunctata_583 | 0.166 |
| Propylaea_quatuordecimpunctata_542 | Tytthaspis_sedecimpunctata_583 | 0.166 |
| Propylaea_quatuordecimpunctata_546 | Tytthaspis_sedecimpunctata_583 | 0.166 |
| Chilocorus_bipustulatus_19 | Tytthaspis_sedecimpunctata_585 | 0.166 |
| Chilocorus_bipustulatus_22 | Tytthaspis_sedecimpunctata_585 | 0.166 |
| Propylaea_quatuordecimpunctata_539 | Tytthaspis_sedecimpunctata_585 | 0.166 |
| Propylaea_quatuordecimpunctata_542 | Tytthaspis_sedecimpunctata_585 | 0.166 |
| Propylaea_quatuordecimpunctata_546 | Tytthaspis_sedecimpunctata_585 | 0.166 |
| Adalia_bipunctata_99 | Tytthaspis_sedecimpunctata_588 | 0.166 |
| Propylaea_quatuordecimpunctata_542 | Tytthaspis_sedecimpunctata_592 | 0.166 |
| Propylaea_quatuordecimpunctata_546 | Tytthaspis_sedecimpunctata_592 | 0.166 |
| Chilocorus_bipustulatus_19 | Tytthaspis_sedecimpunctata_584 | 0.166 |
| Chilocorus_bipustulatus_22 | Tytthaspis_sedecimpunctata_584 | 0.166 |
| Adalia_bipunctata_99 | Tytthaspis_sedecimpunctata_585 | 0.166 |
| Chilocorus_stigma_45 | Tytthaspis_sedecimpunctata_585 | 0.166 |
| Adalia_bipunctata_100 | Tytthaspis_sedecimpunctata_586 | 0.166 |
| Adalia_bipunctata_102 | Tytthaspis_sedecimpunctata_588 | 0.166 |
| Adalia_bipunctata_103 | Tytthaspis_sedecimpunctata_588 | 0.166 |
| Adalia_bipunctata_106 | Tytthaspis_sedecimpunctata_588 | 0.166 |
| Adalia_bipunctata_107 | Tytthaspis_sedecimpunctata_588 | 0.166 |
| Adalia_bipunctata_108 | Tytthaspis_sedecimpunctata_588 | 0.166 |
| Adalia_bipunctata_109 | Tytthaspis_sedecimpunctata_588 | 0.166 |
| Coccinella_magnifica_228 | Tytthaspis_sedecimpunctata_591 | 0.166 |
| Coccinella_magnifica_229 | Tytthaspis_sedecimpunctata_591 | 0.166 |
| Coccinella_magnifica_230 | Tytthaspis_sedecimpunctata_591 | 0.166 |
| Coccinella_magnifica_231 | Tytthaspis_sedecimpunctata_591 | 0.166 |
| Harmonia_axyridis_354 | Tytthaspis_sedecimpunctata_577 | 0.166 |
| Harmonia_axyridis_356 | Tytthaspis_sedecimpunctata_577 | 0.166 |
| Harmonia_axyridis_357 | Tytthaspis_sedecimpunctata_577 | 0.166 |
| Harmonia_axyridis_358 | Tytthaspis_sedecimpunctata_577 | 0.166 |
| Harmonia_axyridis_359 | Tytthaspis_sedecimpunctata_577 | 0.166 |
| Harmonia_axyridis_360 | Tytthaspis_sedecimpunctata_577 | 0.166 |
| Harmonia_axyridis_361 | Tytthaspis_sedecimpunctata_577 | 0.166 |
| Harmonia_axyridis_363 | Tytthaspis_sedecimpunctata_577 | 0.166 |
| Harmonia_axyridis_364 | Tytthaspis_sedecimpunctata_577 | 0.166 |
| Harmonia_axyridis_354 | Tytthaspis_sedecimpunctata_578 | 0.166 |
| Harmonia_axyridis_356 | Tytthaspis_sedecimpunctata_578 | 0.166 |
| Harmonia_axyridis_357 | Tytthaspis_sedecimpunctata_578 | 0.166 |
| Harmonia_axyridis_358 | Tytthaspis_sedecimpunctata_578 | 0.166 |
| Harmonia_axyridis_359 | Tytthaspis_sedecimpunctata_578 | 0.166 |
| Harmonia_axyridis_360 | Tytthaspis_sedecimpunctata_578 | 0.166 |
| Harmonia_axyridis_361 | Tytthaspis_sedecimpunctata_578 | 0.166 |
| Harmonia_axyridis_363 | Tytthaspis_sedecimpunctata_578 | 0.166 |

| | | |
|---|---|---|
| Harmonia_axyridis_364 | Tytthaspis_sedecimpunctata_578 | 0.166 |
| Harmonia_axyridis_354 | Tytthaspis_sedecimpunctata_579 | 0.166 |
| Harmonia_axyridis_356 | Tytthaspis_sedecimpunctata_579 | 0.166 |
| Harmonia_axyridis_357 | Tytthaspis_sedecimpunctata_579 | 0.166 |
| Harmonia_axyridis_358 | Tytthaspis_sedecimpunctata_579 | 0.166 |
| Harmonia_axyridis_359 | Tytthaspis_sedecimpunctata_579 | 0.166 |
| Harmonia_axyridis_360 | Tytthaspis_sedecimpunctata_579 | 0.166 |
| Harmonia_axyridis_361 | Tytthaspis_sedecimpunctata_579 | 0.166 |
| Harmonia_axyridis_363 | Tytthaspis_sedecimpunctata_579 | 0.166 |
| Harmonia_axyridis_364 | Tytthaspis_sedecimpunctata_579 | 0.166 |
| Coccinella_transversoguttata_265 | Tytthaspis_sedecimpunctata_580 | 0.166 |
| Coccinella_transversoguttata_266 | Tytthaspis_sedecimpunctata_580 | 0.166 |
| Coccinella_trifasciata_280 | Tytthaspis_sedecimpunctata_580 | 0.166 |
| Harmonia_axyridis_354 | Tytthaspis_sedecimpunctata_581 | 0.166 |
| Harmonia_axyridis_356 | Tytthaspis_sedecimpunctata_581 | 0.166 |
| Harmonia_axyridis_357 | Tytthaspis_sedecimpunctata_581 | 0.166 |
| Harmonia_axyridis_358 | Tytthaspis_sedecimpunctata_581 | 0.166 |
| Harmonia_axyridis_359 | Tytthaspis_sedecimpunctata_581 | 0.166 |
| Harmonia_axyridis_360 | Tytthaspis_sedecimpunctata_581 | 0.166 |
| Harmonia_axyridis_361 | Tytthaspis_sedecimpunctata_581 | 0.166 |
| Harmonia_axyridis_363 | Tytthaspis_sedecimpunctata_581 | 0.166 |
| Harmonia_axyridis_364 | Tytthaspis_sedecimpunctata_581 | 0.166 |
| Harmonia_axyridis_354 | Tytthaspis_sedecimpunctata_582 | 0.166 |
| Harmonia_axyridis_356 | Tytthaspis_sedecimpunctata_582 | 0.166 |
| Harmonia_axyridis_357 | Tytthaspis_sedecimpunctata_582 | 0.166 |
| Harmonia_axyridis_358 | Tytthaspis_sedecimpunctata_582 | 0.166 |
| Harmonia_axyridis_359 | Tytthaspis_sedecimpunctata_582 | 0.166 |
| Harmonia_axyridis_360 | Tytthaspis_sedecimpunctata_582 | 0.166 |
| Harmonia_axyridis_361 | Tytthaspis_sedecimpunctata_582 | 0.166 |
| Harmonia_axyridis_363 | Tytthaspis_sedecimpunctata_582 | 0.166 |
| Harmonia_axyridis_364 | Tytthaspis_sedecimpunctata_582 | 0.166 |
| Adalia_bipunctata_100 | Tytthaspis_sedecimpunctata_583 | 0.166 |
| Harmonia_axyridis_362 | Tytthaspis_sedecimpunctata_583 | 0.166 |
| Adalia_bipunctata_102 | Tytthaspis_sedecimpunctata_585 | 0.166 |
| Adalia_bipunctata_103 | Tytthaspis_sedecimpunctata_585 | 0.166 |
| Adalia_bipunctata_106 | Tytthaspis_sedecimpunctata_585 | 0.166 |
| Adalia_bipunctata_107 | Tytthaspis_sedecimpunctata_585 | 0.166 |
| Adalia_bipunctata_108 | Tytthaspis_sedecimpunctata_585 | 0.166 |
| Adalia_bipunctata_109 | Tytthaspis_sedecimpunctata_585 | 0.166 |
| Coccinella_transversoguttata_267 | Tytthaspis_sedecimpunctata_585 | 0.166 |
| Coccinella_transversoguttata_268 | Tytthaspis_sedecimpunctata_585 | 0.166 |
| Coccinella_transversoguttata_269 | Tytthaspis_sedecimpunctata_585 | 0.166 |
| Coccinella_transversoguttata_270 | Tytthaspis_sedecimpunctata_585 | 0.166 |
| Coccinella_transversoguttata_271 | Tytthaspis_sedecimpunctata_585 | 0.166 |
| Scymnus_auritus_703 | Tytthaspis_sedecimpunctata_586 | 0.166 |
| Scymnus_auritus_703 | Tytthaspis_sedecimpunctata_588 | 0.166 |
| Anatis_mali_128 | Tytthaspis_sedecimpunctata_589 | 0.166 |
| Anatis_mali_129 | Tytthaspis_sedecimpunctata_589 | 0.166 |
| Anatis_mali_130 | Tytthaspis_sedecimpunctata_589 | 0.166 |
| Anatis_mali_131 | Tytthaspis_sedecimpunctata_589 | 0.166 |
| Anatis_ocellata_133 | Tytthaspis_sedecimpunctata_589 | 0.166 |
| Anatis_ocellata_137 | Tytthaspis_sedecimpunctata_589 | 0.166 |
| Harmonia_axyridis_354 | Tytthaspis_sedecimpunctata_590 | 0.166 |
| Harmonia_axyridis_356 | Tytthaspis_sedecimpunctata_590 | 0.166 |
| Harmonia_axyridis_357 | Tytthaspis_sedecimpunctata_590 | 0.166 |
| Harmonia_axyridis_358 | Tytthaspis_sedecimpunctata_590 | 0.166 |

| | | |
|---|---|---|
| Harmonia_axyridis_359 | Tytthaspis_sedecimpunctata_590 | 0.166 |
| Harmonia_axyridis_360 | Tytthaspis_sedecimpunctata_590 | 0.166 |
| Harmonia_axyridis_361 | Tytthaspis_sedecimpunctata_590 | 0.166 |
| Harmonia_axyridis_363 | Tytthaspis_sedecimpunctata_590 | 0.166 |
| Harmonia_axyridis_364 | Tytthaspis_sedecimpunctata_590 | 0.166 |
| Adalia_bipunctata_100 | Tytthaspis_sedecimpunctata_592 | 0.166 |
| Harmonia_axyridis_362 | Tytthaspis_sedecimpunctata_592 | 0.166 |
| Harmonia_axyridis_354 | Tytthaspis_sedecimpunctata_593 | 0.166 |
| Harmonia_axyridis_356 | Tytthaspis_sedecimpunctata_593 | 0.166 |
| Harmonia_axyridis_357 | Tytthaspis_sedecimpunctata_593 | 0.166 |
| Harmonia_axyridis_358 | Tytthaspis_sedecimpunctata_593 | 0.166 |
| Harmonia_axyridis_359 | Tytthaspis_sedecimpunctata_593 | 0.166 |
| Harmonia_axyridis_360 | Tytthaspis_sedecimpunctata_593 | 0.166 |
| Harmonia_axyridis_361 | Tytthaspis_sedecimpunctata_593 | 0.166 |
| Harmonia_axyridis_363 | Tytthaspis_sedecimpunctata_593 | 0.166 |
| Harmonia_axyridis_364 | Tytthaspis_sedecimpunctata_593 | 0.166 |
| Harmonia_axyridis_354 | Tytthaspis_sedecimpunctata_594 | 0.166 |
| Harmonia_axyridis_356 | Tytthaspis_sedecimpunctata_594 | 0.166 |
| Harmonia_axyridis_357 | Tytthaspis_sedecimpunctata_594 | 0.166 |
| Harmonia_axyridis_358 | Tytthaspis_sedecimpunctata_594 | 0.166 |
| Harmonia_axyridis_359 | Tytthaspis_sedecimpunctata_594 | 0.166 |
| Harmonia_axyridis_360 | Tytthaspis_sedecimpunctata_594 | 0.166 |
| Harmonia_axyridis_361 | Tytthaspis_sedecimpunctata_594 | 0.166 |
| Harmonia_axyridis_363 | Tytthaspis_sedecimpunctata_594 | 0.166 |
| Harmonia_axyridis_364 | Tytthaspis_sedecimpunctata_594 | 0.166 |
| Harmonia_axyridis_354 | Tytthaspis_sedecimpunctata_595 | 0.166 |
| Harmonia_axyridis_356 | Tytthaspis_sedecimpunctata_595 | 0.166 |
| Harmonia_axyridis_357 | Tytthaspis_sedecimpunctata_595 | 0.166 |
| Harmonia_axyridis_358 | Tytthaspis_sedecimpunctata_595 | 0.166 |
| Harmonia_axyridis_359 | Tytthaspis_sedecimpunctata_595 | 0.166 |
| Harmonia_axyridis_360 | Tytthaspis_sedecimpunctata_595 | 0.166 |
| Harmonia_axyridis_361 | Tytthaspis_sedecimpunctata_595 | 0.166 |
| Harmonia_axyridis_363 | Tytthaspis_sedecimpunctata_595 | 0.166 |
| Harmonia_axyridis_364 | Tytthaspis_sedecimpunctata_595 | 0.166 |
| Adalia_bipunctata_100 | Tytthaspis_sedecimpunctata_580 | 0.166 |
| Harmonia_axyridis_354 | Tytthaspis_sedecimpunctata_580 | 0.166 |
| Harmonia_axyridis_356 | Tytthaspis_sedecimpunctata_580 | 0.166 |
| Harmonia_axyridis_357 | Tytthaspis_sedecimpunctata_580 | 0.166 |
| Harmonia_axyridis_358 | Tytthaspis_sedecimpunctata_580 | 0.166 |
| Harmonia_axyridis_359 | Tytthaspis_sedecimpunctata_580 | 0.166 |
| Harmonia_axyridis_360 | Tytthaspis_sedecimpunctata_580 | 0.166 |
| Harmonia_axyridis_361 | Tytthaspis_sedecimpunctata_580 | 0.166 |
| Harmonia_axyridis_363 | Tytthaspis_sedecimpunctata_580 | 0.166 |
| Harmonia_axyridis_364 | Tytthaspis_sedecimpunctata_580 | 0.166 |
| Anatis_mali_128 | Tytthaspis_sedecimpunctata_584 | 0.166 |
| Anatis_mali_129 | Tytthaspis_sedecimpunctata_584 | 0.166 |
| Anatis_mali_130 | Tytthaspis_sedecimpunctata_584 | 0.166 |
| Anatis_mali_131 | Tytthaspis_sedecimpunctata_584 | 0.166 |
| Harmonia_axyridis_355 | Tytthaspis_sedecimpunctata_585 | 0.166 |
| Anatis_mali_128 | Tytthaspis_sedecimpunctata_588 | 0.166 |
| Anatis_mali_129 | Tytthaspis_sedecimpunctata_588 | 0.166 |
| Anatis_mali_130 | Tytthaspis_sedecimpunctata_588 | 0.166 |
| Anatis_mali_131 | Tytthaspis_sedecimpunctata_588 | 0.166 |
| Anatis_ocellata_133 | Tytthaspis_sedecimpunctata_588 | 0.166 |
| Anatis_ocellata_137 | Tytthaspis_sedecimpunctata_588 | 0.166 |
| Psyllobora_renifer_555 | Tytthaspis_sedecimpunctata_592 | 0.166 |

| | | |
|---|---|---|
| Hyperaspis_reppensis_667 | Scymnus_suturalis_841 | 0.166 |
| Hyperaspis_reppensis_667 | Scymnus_suturalis_842 | 0.166 |
| Hyperaspis_reppensis_667 | Scymnus_suturalis_844 | 0.166 |
| Hyperaspis_reppensis_667 | Scymnus_suturalis_846 | 0.166 |
| Hyperaspis_reppensis_667 | Scymnus_suturalis_847 | 0.166 |
| Hyperaspis_reppensis_667 | Scymnus_suturalis_848 | 0.166 |
| Anatis_ocellata_133 | Tytthaspis_sedecimpunctata_577 | 0.166 |
| Anatis_ocellata_137 | Tytthaspis_sedecimpunctata_577 | 0.166 |
| Anatis_ocellata_133 | Tytthaspis_sedecimpunctata_578 | 0.166 |
| Anatis_ocellata_137 | Tytthaspis_sedecimpunctata_578 | 0.166 |
| Anatis_ocellata_133 | Tytthaspis_sedecimpunctata_579 | 0.166 |
| Anatis_ocellata_137 | Tytthaspis_sedecimpunctata_579 | 0.166 |
| Coccinella_trifasciata_273 | Tytthaspis_sedecimpunctata_580 | 0.166 |
| Psyllobora_renifer_555 | Tytthaspis_sedecimpunctata_580 | 0.166 |
| Anatis_ocellata_133 | Tytthaspis_sedecimpunctata_581 | 0.166 |
| Anatis_ocellata_137 | Tytthaspis_sedecimpunctata_581 | 0.166 |
| Anatis_ocellata_133 | Tytthaspis_sedecimpunctata_582 | 0.166 |
| Anatis_ocellata_137 | Tytthaspis_sedecimpunctata_582 | 0.166 |
| Hippodamia_alpina_388 | Tytthaspis_sedecimpunctata_583 | 0.166 |
| Harmonia_axyridis_355 | Tytthaspis_sedecimpunctata_584 | 0.166 |
| Anatis_ocellata_132 | Tytthaspis_sedecimpunctata_587 | 0.166 |
| Anatis_ocellata_134 | Tytthaspis_sedecimpunctata_587 | 0.166 |
| Anatis_ocellata_135 | Tytthaspis_sedecimpunctata_587 | 0.166 |
| Anatis_ocellata_138 | Tytthaspis_sedecimpunctata_587 | 0.166 |
| Anatis_ocellata_139 | Tytthaspis_sedecimpunctata_587 | 0.166 |
| Anatis_ocellata_140 | Tytthaspis_sedecimpunctata_587 | 0.166 |
| Anatis_ocellata_141 | Tytthaspis_sedecimpunctata_587 | 0.166 |
| Anatis_ocellata_142 | Tytthaspis_sedecimpunctata_587 | 0.166 |
| Anatis_ocellata_144 | Tytthaspis_sedecimpunctata_587 | 0.166 |
| Anatis_ocellata_145 | Tytthaspis_sedecimpunctata_587 | 0.166 |
| Anatis_ocellata_146 | Tytthaspis_sedecimpunctata_587 | 0.166 |
| Psyllobora_vigintimaculata_573 | Tytthaspis_sedecimpunctata_588 | 0.166 |
| Hippodamia_alpina_383 | Tytthaspis_sedecimpunctata_589 | 0.166 |
| Psyllobora_vigintimaculata_575 | Tytthaspis_sedecimpunctata_589 | 0.166 |
| Psyllobora_vigintimaculata_576 | Tytthaspis_sedecimpunctata_589 | 0.166 |
| Anatis_ocellata_133 | Tytthaspis_sedecimpunctata_590 | 0.166 |
| Anatis_ocellata_137 | Tytthaspis_sedecimpunctata_590 | 0.166 |
| Coccinella_trifasciata_273 | Tytthaspis_sedecimpunctata_591 | 0.166 |
| Anatis_ocellata_133 | Tytthaspis_sedecimpunctata_593 | 0.166 |
| Anatis_ocellata_137 | Tytthaspis_sedecimpunctata_593 | 0.166 |
| Anatis_ocellata_143 | Tytthaspis_sedecimpunctata_593 | 0.166 |
| Anatis_ocellata_133 | Tytthaspis_sedecimpunctata_594 | 0.166 |
| Anatis_ocellata_137 | Tytthaspis_sedecimpunctata_594 | 0.166 |
| Anatis_ocellata_133 | Tytthaspis_sedecimpunctata_595 | 0.166 |
| Anatis_ocellata_137 | Tytthaspis_sedecimpunctata_595 | 0.166 |
| Hyperaspis_reppensis_667 | Scymnus_suturalis_832 | 0.166 |
| Scymnus_pallipediformis_811 | Scymnus_suturalis_833 | 0.166 |
| Hyperaspis_reppensis_667 | Scymnus_suturalis_835 | 0.166 |
| Hyperaspis_reppensis_667 | Scymnus_suturalis_836 | 0.166 |
| Hyperaspis_reppensis_667 | Scymnus_suturalis_839 | 0.166 |
| Scymnus_apetzi_691 | Scymnus_suturalis_841 | 0.166 |
| Scymnus_apetzi_696 | Scymnus_suturalis_841 | 0.166 |
| Scymnus_apetzi_691 | Scymnus_suturalis_842 | 0.166 |
| Scymnus_apetzi_696 | Scymnus_suturalis_842 | 0.166 |
| Scymnus_apetzi_690 | Scymnus_suturalis_843 | 0.166 |
| Scymnus_apetzi_697 | Scymnus_suturalis_843 | 0.166 |

| | | |
|---|---|---|
| Scymnus_apetzi_691 | Scymnus_suturalis_844 | 0.166 |
| Scymnus_apetzi_696 | Scymnus_suturalis_844 | 0.166 |
| Scymnus_apetzi_690 | Scymnus_suturalis_845 | 0.166 |
| Scymnus_apetzi_697 | Scymnus_suturalis_845 | 0.166 |
| Scymnus_apetzi_691 | Scymnus_suturalis_846 | 0.166 |
| Scymnus_apetzi_696 | Scymnus_suturalis_846 | 0.166 |
| Scymnus_apetzi_691 | Scymnus_suturalis_847 | 0.166 |
| Scymnus_apetzi_696 | Scymnus_suturalis_847 | 0.166 |
| Scymnus_apetzi_691 | Scymnus_suturalis_848 | 0.166 |
| Scymnus_apetzi_696 | Scymnus_suturalis_848 | 0.166 |
| Hippodamia_alpina_388 | Tytthaspis_sedecimpunctata_586 | 0.166 |
| Psyllobora_vigintimaculata_575 | Tytthaspis_sedecimpunctata_586 | 0.166 |
| Psyllobora_vigintimaculata_576 | Tytthaspis_sedecimpunctata_586 | 0.166 |
| Hippodamia_alpina_388 | Tytthaspis_sedecimpunctata_588 | 0.166 |
| Psyllobora_vigintimaculata_574 | Tytthaspis_sedecimpunctata_588 | 0.166 |
| Mulsantina_picta_484 | Tytthaspis_sedecimpunctata_589 | 0.166 |
| Scymnus_apetzi_691 | Scymnus_suturalis_832 | 0.166 |
| Scymnus_apetzi_696 | Scymnus_suturalis_832 | 0.166 |
| Scymnus_apetzi_691 | Scymnus_suturalis_835 | 0.166 |
| Scymnus_apetzi_696 | Scymnus_suturalis_835 | 0.166 |
| Scymnus_apetzi_691 | Scymnus_suturalis_836 | 0.166 |
| Scymnus_apetzi_696 | Scymnus_suturalis_836 | 0.166 |
| Scymnus_apetzi_690 | Scymnus_suturalis_837 | 0.166 |
| Scymnus_apetzi_697 | Scymnus_suturalis_837 | 0.166 |
| Scymnus_apetzi_691 | Scymnus_suturalis_839 | 0.166 |
| Scymnus_apetzi_696 | Scymnus_suturalis_839 | 0.166 |
| Hyperaspis_proba_660 | Tytthaspis_sedecimpunctata_589 | 0.166 |
| Hyperaspis_proba_660 | Tytthaspis_sedecimpunctata_593 | 0.166 |
| Scymnus_auritus_699 | Scymnus_sinuanodulus_829 | 0.166 |
| Hyperaspis_proba_660 | Tytthaspis_sedecimpunctata_580 | 0.166 |
| Hyperaspis_proba_660 | Tytthaspis_sedecimpunctata_587 | 0.166 |
| Chilocorus_bipustulatus_21 | Tytthaspis_sedecimpunctata_589 | 0.166 |
| Hippodamia_alpina_384 | Tytthaspis_sedecimpunctata_580 | 0.166 |
| Scymnus_haemorrhoidalis_749 | Tytthaspis_sedecimpunctata_584 | 0.166 |
| Psyllobora_vigintiduopunctata_560 | Tytthaspis_sedecimpunctata_587 | 0.166 |
| Scymnus_haemorrhoidalis_753 | Tytthaspis_sedecimpunctata_589 | 0.166 |
| Scymnus_haemorrhoidalis_756 | Tytthaspis_sedecimpunctata_589 | 0.166 |
| Scymnus_haemorrhoidalis_749 | Tytthaspis_sedecimpunctata_591 | 0.166 |
| Scymnus_mimulus_778 | Scymnus_suturalis_833 | 0.166 |
| Chilocorus_nigritus_28 | Scymnus_suturalis_843 | 0.166 |
| Chilocorus_nigritus_28 | Scymnus_suturalis_845 | 0.166 |
| Chilocorus_bipustulatus_21 | Tytthaspis_sedecimpunctata_585 | 0.166 |
| Chilocorus_bipustulatus_21 | Tytthaspis_sedecimpunctata_584 | 0.166 |
| Scymnus_mimulus_778 | Scymnus_suturalis_841 | 0.166 |
| Scymnus_mimulus_778 | Scymnus_suturalis_842 | 0.166 |
| Scymnus_mimulus_778 | Scymnus_suturalis_844 | 0.166 |
| Scymnus_mimulus_778 | Scymnus_suturalis_846 | 0.166 |
| Scymnus_mimulus_778 | Scymnus_suturalis_847 | 0.166 |
| Scymnus_mimulus_778 | Scymnus_suturalis_848 | 0.166 |
| Hyperaspis_oregona_658 | Subcoccinella_vigintiquatuorpunctata_625 | 0.166 |
| Hyperaspis_oregona_659 | Subcoccinella_vigintiquatuorpunctata_625 | 0.166 |
| Coelophora_inaequalis_320 | Tytthaspis_sedecimpunctata_577 | 0.166 |
| Coelophora_inaequalis_321 | Tytthaspis_sedecimpunctata_577 | 0.166 |
| Coelophora_inaequalis_322 | Tytthaspis_sedecimpunctata_577 | 0.166 |
| Psyllobora_vigintiduopunctata_557 | Tytthaspis_sedecimpunctata_577 | 0.166 |
| Psyllobora_vigintiduopunctata_561 | Tytthaspis_sedecimpunctata_577 | 0.166 |

| | | |
|---|---|---|
| Psyllobora_vigintiduopunctata_562 | Tytthaspis_sedecimpunctata_577 | 0.166 |
| Psyllobora_vigintiduopunctata_563 | Tytthaspis_sedecimpunctata_577 | 0.166 |
| Psyllobora_vigintiduopunctata_565 | Tytthaspis_sedecimpunctata_577 | 0.166 |
| Psyllobora_vigintiduopunctata_567 | Tytthaspis_sedecimpunctata_577 | 0.166 |
| Coelophora_inaequalis_320 | Tytthaspis_sedecimpunctata_578 | 0.166 |
| Coelophora_inaequalis_321 | Tytthaspis_sedecimpunctata_578 | 0.166 |
| Coelophora_inaequalis_322 | Tytthaspis_sedecimpunctata_578 | 0.166 |
| Psyllobora_vigintiduopunctata_557 | Tytthaspis_sedecimpunctata_578 | 0.166 |
| Psyllobora_vigintiduopunctata_561 | Tytthaspis_sedecimpunctata_578 | 0.166 |
| Psyllobora_vigintiduopunctata_562 | Tytthaspis_sedecimpunctata_578 | 0.166 |
| Psyllobora_vigintiduopunctata_563 | Tytthaspis_sedecimpunctata_578 | 0.166 |
| Psyllobora_vigintiduopunctata_565 | Tytthaspis_sedecimpunctata_578 | 0.166 |
| Psyllobora_vigintiduopunctata_567 | Tytthaspis_sedecimpunctata_578 | 0.166 |
| Coelophora_inaequalis_320 | Tytthaspis_sedecimpunctata_579 | 0.166 |
| Coelophora_inaequalis_321 | Tytthaspis_sedecimpunctata_579 | 0.166 |
| Coelophora_inaequalis_322 | Tytthaspis_sedecimpunctata_579 | 0.166 |
| Psyllobora_vigintiduopunctata_557 | Tytthaspis_sedecimpunctata_579 | 0.166 |
| Psyllobora_vigintiduopunctata_561 | Tytthaspis_sedecimpunctata_579 | 0.166 |
| Psyllobora_vigintiduopunctata_562 | Tytthaspis_sedecimpunctata_579 | 0.166 |
| Psyllobora_vigintiduopunctata_563 | Tytthaspis_sedecimpunctata_579 | 0.166 |
| Psyllobora_vigintiduopunctata_565 | Tytthaspis_sedecimpunctata_579 | 0.166 |
| Psyllobora_vigintiduopunctata_567 | Tytthaspis_sedecimpunctata_579 | 0.166 |
| Coelophora_inaequalis_320 | Tytthaspis_sedecimpunctata_581 | 0.166 |
| Coelophora_inaequalis_321 | Tytthaspis_sedecimpunctata_581 | 0.166 |
| Coelophora_inaequalis_322 | Tytthaspis_sedecimpunctata_581 | 0.166 |
| Psyllobora_vigintiduopunctata_557 | Tytthaspis_sedecimpunctata_581 | 0.166 |
| Psyllobora_vigintiduopunctata_561 | Tytthaspis_sedecimpunctata_581 | 0.166 |
| Psyllobora_vigintiduopunctata_562 | Tytthaspis_sedecimpunctata_581 | 0.166 |
| Psyllobora_vigintiduopunctata_563 | Tytthaspis_sedecimpunctata_581 | 0.166 |
| Psyllobora_vigintiduopunctata_565 | Tytthaspis_sedecimpunctata_581 | 0.166 |
| Psyllobora_vigintiduopunctata_567 | Tytthaspis_sedecimpunctata_581 | 0.166 |
| Coelophora_inaequalis_320 | Tytthaspis_sedecimpunctata_582 | 0.166 |
| Coelophora_inaequalis_321 | Tytthaspis_sedecimpunctata_582 | 0.166 |
| Coelophora_inaequalis_322 | Tytthaspis_sedecimpunctata_582 | 0.166 |
| Psyllobora_vigintiduopunctata_557 | Tytthaspis_sedecimpunctata_582 | 0.166 |
| Psyllobora_vigintiduopunctata_561 | Tytthaspis_sedecimpunctata_582 | 0.166 |
| Psyllobora_vigintiduopunctata_562 | Tytthaspis_sedecimpunctata_582 | 0.166 |
| Psyllobora_vigintiduopunctata_563 | Tytthaspis_sedecimpunctata_582 | 0.166 |
| Psyllobora_vigintiduopunctata_565 | Tytthaspis_sedecimpunctata_582 | 0.166 |
| Psyllobora_vigintiduopunctata_567 | Tytthaspis_sedecimpunctata_582 | 0.166 |
| Psyllobora_vigintiduopunctata_570 | Tytthaspis_sedecimpunctata_583 | 0.166 |
| Psyllobora_vigintiduopunctata_572 | Tytthaspis_sedecimpunctata_583 | 0.166 |
| Scymnus_haemorrhoidalis_750 | Tytthaspis_sedecimpunctata_583 | 0.166 |
| Scymnus_haemorrhoidalis_751 | Tytthaspis_sedecimpunctata_583 | 0.166 |
| Scymnus_haemorrhoidalis_754 | Tytthaspis_sedecimpunctata_583 | 0.166 |
| Scymnus_haemorrhoidalis_757 | Tytthaspis_sedecimpunctata_583 | 0.166 |
| Scymnus_haemorrhoidalis_758 | Tytthaspis_sedecimpunctata_583 | 0.166 |
| Psyllobora_vigintiduopunctata_570 | Tytthaspis_sedecimpunctata_585 | 0.166 |
| Psyllobora_vigintiduopunctata_572 | Tytthaspis_sedecimpunctata_585 | 0.166 |
| Scymnus_haemorrhoidalis_749 | Tytthaspis_sedecimpunctata_585 | 0.166 |
| Hippodamia_glacialis_408 | Tytthaspis_sedecimpunctata_586 | 0.166 |
| Hippodamia_glacialis_409 | Tytthaspis_sedecimpunctata_586 | 0.166 |
| Scymnus_haemorrhoidalis_752 | Tytthaspis_sedecimpunctata_586 | 0.166 |
| Scymnus_haemorrhoidalis_753 | Tytthaspis_sedecimpunctata_587 | 0.166 |
| Scymnus_haemorrhoidalis_756 | Tytthaspis_sedecimpunctata_587 | 0.166 |
| Hippodamia_glacialis_407 | Tytthaspis_sedecimpunctata_588 | 0.166 |

| | | |
|---|---|---|
| Coelophora_inaequalis_320 | Tytthaspis_sedecimpunctata_590 | 0.166 |
| Coelophora_inaequalis_321 | Tytthaspis_sedecimpunctata_590 | 0.166 |
| Coelophora_inaequalis_322 | Tytthaspis_sedecimpunctata_590 | 0.166 |
| Psyllobora_vigintiduopunctata_557 | Tytthaspis_sedecimpunctata_590 | 0.166 |
| Psyllobora_vigintiduopunctata_561 | Tytthaspis_sedecimpunctata_590 | 0.166 |
| Psyllobora_vigintiduopunctata_562 | Tytthaspis_sedecimpunctata_590 | 0.166 |
| Psyllobora_vigintiduopunctata_563 | Tytthaspis_sedecimpunctata_590 | 0.166 |
| Psyllobora_vigintiduopunctata_565 | Tytthaspis_sedecimpunctata_590 | 0.166 |
| Psyllobora_vigintiduopunctata_567 | Tytthaspis_sedecimpunctata_590 | 0.166 |
| Psyllobora_vigintiduopunctata_570 | Tytthaspis_sedecimpunctata_592 | 0.166 |
| Psyllobora_vigintiduopunctata_572 | Tytthaspis_sedecimpunctata_592 | 0.166 |
| Psyllobora_vigintiduopunctata_557 | Tytthaspis_sedecimpunctata_593 | 0.166 |
| Psyllobora_vigintiduopunctata_563 | Tytthaspis_sedecimpunctata_593 | 0.166 |
| Scymnus_haemorrhoidalis_750 | Tytthaspis_sedecimpunctata_593 | 0.166 |
| Scymnus_haemorrhoidalis_751 | Tytthaspis_sedecimpunctata_593 | 0.166 |
| Scymnus_haemorrhoidalis_754 | Tytthaspis_sedecimpunctata_593 | 0.166 |
| Scymnus_haemorrhoidalis_757 | Tytthaspis_sedecimpunctata_593 | 0.166 |
| Scymnus_haemorrhoidalis_758 | Tytthaspis_sedecimpunctata_593 | 0.166 |
| Coelophora_inaequalis_320 | Tytthaspis_sedecimpunctata_594 | 0.166 |
| Coelophora_inaequalis_321 | Tytthaspis_sedecimpunctata_594 | 0.166 |
| Coelophora_inaequalis_322 | Tytthaspis_sedecimpunctata_594 | 0.166 |
| Psyllobora_vigintiduopunctata_557 | Tytthaspis_sedecimpunctata_594 | 0.166 |
| Psyllobora_vigintiduopunctata_561 | Tytthaspis_sedecimpunctata_594 | 0.166 |
| Psyllobora_vigintiduopunctata_562 | Tytthaspis_sedecimpunctata_594 | 0.166 |
| Psyllobora_vigintiduopunctata_563 | Tytthaspis_sedecimpunctata_594 | 0.166 |
| Psyllobora_vigintiduopunctata_565 | Tytthaspis_sedecimpunctata_594 | 0.166 |
| Psyllobora_vigintiduopunctata_567 | Tytthaspis_sedecimpunctata_594 | 0.166 |
| Coelophora_inaequalis_320 | Tytthaspis_sedecimpunctata_595 | 0.166 |
| Coelophora_inaequalis_321 | Tytthaspis_sedecimpunctata_595 | 0.166 |
| Coelophora_inaequalis_322 | Tytthaspis_sedecimpunctata_595 | 0.166 |
| Psyllobora_vigintiduopunctata_557 | Tytthaspis_sedecimpunctata_595 | 0.166 |
| Psyllobora_vigintiduopunctata_561 | Tytthaspis_sedecimpunctata_595 | 0.166 |
| Psyllobora_vigintiduopunctata_562 | Tytthaspis_sedecimpunctata_595 | 0.166 |
| Psyllobora_vigintiduopunctata_563 | Tytthaspis_sedecimpunctata_595 | 0.166 |
| Psyllobora_vigintiduopunctata_565 | Tytthaspis_sedecimpunctata_595 | 0.166 |
| Psyllobora_vigintiduopunctata_567 | Tytthaspis_sedecimpunctata_595 | 0.166 |
| Scymnus_mimulus_778 | Scymnus_suturalis_832 | 0.166 |
| Scymnus_mimulus_778 | Scymnus_suturalis_835 | 0.166 |
| Scymnus_mimulus_778 | Scymnus_suturalis_836 | 0.166 |
| Rhyzobius_chrysomeloides_85 | Scymnus_suturalis_838 | 0.166 |
| Rhyzobius_chrysomeloides_86 | Scymnus_suturalis_838 | 0.166 |
| Rhyzobius_chrysomeloides_87 | Scymnus_suturalis_838 | 0.166 |
| Rhyzobius_chrysomeloides_88 | Scymnus_suturalis_838 | 0.166 |
| Rhyzobius_chrysomeloides_89 | Scymnus_suturalis_838 | 0.166 |
| Rhyzobius_chrysomeloides_90 | Scymnus_suturalis_838 | 0.166 |
| Rhyzobius_chrysomeloides_91 | Scymnus_suturalis_838 | 0.166 |
| Rhyzobius_chrysomeloides_92 | Scymnus_suturalis_838 | 0.166 |
| Rhyzobius_chrysomeloides_93 | Scymnus_suturalis_838 | 0.166 |
| Rhyzobius_chrysomeloides_94 | Scymnus_suturalis_838 | 0.166 |
| Rhyzobius_chrysomeloides_95 | Scymnus_suturalis_838 | 0.166 |
| Scymnus_mimulus_778 | Scymnus_suturalis_839 | 0.166 |
| Rhyzobius_chrysomeloides_85 | Scymnus_suturalis_840 | 0.166 |
| Rhyzobius_chrysomeloides_86 | Scymnus_suturalis_840 | 0.166 |
| Rhyzobius_chrysomeloides_87 | Scymnus_suturalis_840 | 0.166 |
| Rhyzobius_chrysomeloides_88 | Scymnus_suturalis_840 | 0.166 |
| Rhyzobius_chrysomeloides_89 | Scymnus_suturalis_840 | 0.166 |

| | | |
|---|---|---|
| Rhyzobius_chrysomeloides_90 | Scymnus_suturalis_840 | 0.166 |
| Rhyzobius_chrysomeloides_91 | Scymnus_suturalis_840 | 0.166 |
| Rhyzobius_chrysomeloides_92 | Scymnus_suturalis_840 | 0.166 |
| Rhyzobius_chrysomeloides_93 | Scymnus_suturalis_840 | 0.166 |
| Rhyzobius_chrysomeloides_94 | Scymnus_suturalis_840 | 0.166 |
| Rhyzobius_chrysomeloides_95 | Scymnus_suturalis_840 | 0.166 |
| Hippodamia_arctica_389 | Tytthaspis_sedecimpunctata_577 | 0.167 |
| Hippodamia_notata_419 | Tytthaspis_sedecimpunctata_577 | 0.167 |
| Hippodamia_notata_429 | Tytthaspis_sedecimpunctata_577 | 0.167 |
| Hippodamia_notata_431 | Tytthaspis_sedecimpunctata_577 | 0.167 |
| Psyllobora_vigintiduopunctata_556 | Tytthaspis_sedecimpunctata_577 | 0.167 |
| Hippodamia_arctica_389 | Tytthaspis_sedecimpunctata_578 | 0.167 |
| Hippodamia_notata_419 | Tytthaspis_sedecimpunctata_578 | 0.167 |
| Hippodamia_notata_429 | Tytthaspis_sedecimpunctata_578 | 0.167 |
| Hippodamia_notata_431 | Tytthaspis_sedecimpunctata_578 | 0.167 |
| Psyllobora_vigintiduopunctata_556 | Tytthaspis_sedecimpunctata_578 | 0.167 |
| Hippodamia_arctica_389 | Tytthaspis_sedecimpunctata_579 | 0.167 |
| Hippodamia_notata_419 | Tytthaspis_sedecimpunctata_579 | 0.167 |
| Hippodamia_notata_429 | Tytthaspis_sedecimpunctata_579 | 0.167 |
| Hippodamia_notata_431 | Tytthaspis_sedecimpunctata_579 | 0.167 |
| Psyllobora_vigintiduopunctata_556 | Tytthaspis_sedecimpunctata_579 | 0.167 |
| Psyllobora_vigintiduopunctata_570 | Tytthaspis_sedecimpunctata_580 | 0.167 |
| Psyllobora_vigintiduopunctata_572 | Tytthaspis_sedecimpunctata_580 | 0.167 |
| Hippodamia_arctica_389 | Tytthaspis_sedecimpunctata_581 | 0.167 |
| Hippodamia_notata_419 | Tytthaspis_sedecimpunctata_581 | 0.167 |
| Hippodamia_notata_429 | Tytthaspis_sedecimpunctata_581 | 0.167 |
| Hippodamia_notata_431 | Tytthaspis_sedecimpunctata_581 | 0.167 |
| Psyllobora_vigintiduopunctata_556 | Tytthaspis_sedecimpunctata_581 | 0.167 |
| Hippodamia_arctica_389 | Tytthaspis_sedecimpunctata_582 | 0.167 |
| Hippodamia_notata_419 | Tytthaspis_sedecimpunctata_582 | 0.167 |
| Hippodamia_notata_429 | Tytthaspis_sedecimpunctata_582 | 0.167 |
| Hippodamia_notata_431 | Tytthaspis_sedecimpunctata_582 | 0.167 |
| Psyllobora_vigintiduopunctata_556 | Tytthaspis_sedecimpunctata_582 | 0.167 |
| Hippodamia_notata_421 | Tytthaspis_sedecimpunctata_583 | 0.167 |
| Scymnus_haemorrhoidalis_755 | Tytthaspis_sedecimpunctata_583 | 0.167 |
| Psyllobora_vigintiduopunctata_564 | Tytthaspis_sedecimpunctata_584 | 0.167 |
| Hippodamia_notata_419 | Tytthaspis_sedecimpunctata_585 | 0.167 |
| Hippodamia_notata_429 | Tytthaspis_sedecimpunctata_585 | 0.167 |
| Hippodamia_notata_431 | Tytthaspis_sedecimpunctata_585 | 0.167 |
| Hippodamia_tredecimpunctata_459 | Tytthaspis_sedecimpunctata_587 | 0.167 |
| Hippodamia_tredecimpunctata_457 | Tytthaspis_sedecimpunctata_588 | 0.167 |
| Hippodamia_tredecimpunctata_458 | Tytthaspis_sedecimpunctata_588 | 0.167 |
| Halyzia_sedecimguttata_345 | Tytthaspis_sedecimpunctata_589 | 0.167 |
| Halyzia_sedecimguttata_350 | Tytthaspis_sedecimpunctata_589 | 0.167 |
| Halyzia_sedecimguttata_351 | Tytthaspis_sedecimpunctata_589 | 0.167 |
| Hippodamia_arctica_389 | Tytthaspis_sedecimpunctata_590 | 0.167 |
| Hippodamia_notata_419 | Tytthaspis_sedecimpunctata_590 | 0.167 |
| Hippodamia_notata_429 | Tytthaspis_sedecimpunctata_590 | 0.167 |
| Hippodamia_notata_431 | Tytthaspis_sedecimpunctata_590 | 0.167 |
| Psyllobora_vigintiduopunctata_556 | Tytthaspis_sedecimpunctata_590 | 0.167 |
| Hippodamia_arctica_389 | Tytthaspis_sedecimpunctata_592 | 0.167 |
| Hippodamia_notata_419 | Tytthaspis_sedecimpunctata_592 | 0.167 |
| Hippodamia_notata_429 | Tytthaspis_sedecimpunctata_592 | 0.167 |
| Hippodamia_notata_431 | Tytthaspis_sedecimpunctata_592 | 0.167 |
| Hippodamia_arctica_389 | Tytthaspis_sedecimpunctata_593 | 0.167 |
| Psyllobora_vigintiduopunctata_556 | Tytthaspis_sedecimpunctata_593 | 0.167 |

| | | |
|---|---|---|
| Scymnus_haemorrhoidalis_755 | Tytthaspis_sedecimpunctata_593 | 0.167 |
| Hippodamia_arctica_389 | Tytthaspis_sedecimpunctata_594 | 0.167 |
| Hippodamia_notata_419 | Tytthaspis_sedecimpunctata_594 | 0.167 |
| Hippodamia_notata_429 | Tytthaspis_sedecimpunctata_594 | 0.167 |
| Hippodamia_notata_431 | Tytthaspis_sedecimpunctata_594 | 0.167 |
| Psyllobora_vigintiduopunctata_556 | Tytthaspis_sedecimpunctata_594 | 0.167 |
| Hippodamia_arctica_389 | Tytthaspis_sedecimpunctata_595 | 0.167 |
| Hippodamia_notata_419 | Tytthaspis_sedecimpunctata_595 | 0.167 |
| Hippodamia_notata_429 | Tytthaspis_sedecimpunctata_595 | 0.167 |
| Hippodamia_notata_431 | Tytthaspis_sedecimpunctata_595 | 0.167 |
| Psyllobora_vigintiduopunctata_556 | Tytthaspis_sedecimpunctata_595 | 0.167 |
| Scymnus_nigrinus_785 | Scymnus_sinuanodulus_830 | 0.167 |
| Scymnus_nigrinus_786 | Scymnus_sinuanodulus_830 | 0.167 |
| Rhyzobius_chrysomeloides_85 | Scymnus_suturalis_834 | 0.167 |
| Rhyzobius_chrysomeloides_86 | Scymnus_suturalis_834 | 0.167 |
| Rhyzobius_chrysomeloides_87 | Scymnus_suturalis_834 | 0.167 |
| Rhyzobius_chrysomeloides_88 | Scymnus_suturalis_834 | 0.167 |
| Rhyzobius_chrysomeloides_89 | Scymnus_suturalis_834 | 0.167 |
| Rhyzobius_chrysomeloides_90 | Scymnus_suturalis_834 | 0.167 |
| Rhyzobius_chrysomeloides_91 | Scymnus_suturalis_834 | 0.167 |
| Rhyzobius_chrysomeloides_92 | Scymnus_suturalis_834 | 0.167 |
| Rhyzobius_chrysomeloides_93 | Scymnus_suturalis_834 | 0.167 |
| Rhyzobius_chrysomeloides_94 | Scymnus_suturalis_834 | 0.167 |
| Rhyzobius_chrysomeloides_95 | Scymnus_suturalis_834 | 0.167 |
| Rhyzobius_chrysomeloides_85 | Scymnus_suturalis_837 | 0.167 |
| Rhyzobius_chrysomeloides_86 | Scymnus_suturalis_837 | 0.167 |
| Rhyzobius_chrysomeloides_87 | Scymnus_suturalis_837 | 0.167 |
| Rhyzobius_chrysomeloides_88 | Scymnus_suturalis_837 | 0.167 |
| Rhyzobius_chrysomeloides_89 | Scymnus_suturalis_837 | 0.167 |
| Rhyzobius_chrysomeloides_90 | Scymnus_suturalis_837 | 0.167 |
| Rhyzobius_chrysomeloides_91 | Scymnus_suturalis_837 | 0.167 |
| Rhyzobius_chrysomeloides_92 | Scymnus_suturalis_837 | 0.167 |
| Rhyzobius_chrysomeloides_93 | Scymnus_suturalis_837 | 0.167 |
| Rhyzobius_chrysomeloides_94 | Scymnus_suturalis_837 | 0.167 |
| Rhyzobius_chrysomeloides_95 | Scymnus_suturalis_837 | 0.167 |
| Scymnus_apetzi_692 | Scymnus_suturalis_833 | 0.167 |
| Ceratomegilla_ulkei_221 | Tytthaspis_sedecimpunctata_577 | 0.167 |
| Ceratomegilla_ulkei_221 | Tytthaspis_sedecimpunctata_578 | 0.167 |
| Ceratomegilla_ulkei_221 | Tytthaspis_sedecimpunctata_579 | 0.167 |
| Hippodamia_glacialis_408 | Tytthaspis_sedecimpunctata_580 | 0.167 |
| Hippodamia_glacialis_409 | Tytthaspis_sedecimpunctata_580 | 0.167 |
| Psyllobora_vigintiduopunctata_556 | Tytthaspis_sedecimpunctata_580 | 0.167 |
| Ceratomegilla_ulkei_221 | Tytthaspis_sedecimpunctata_581 | 0.167 |
| Ceratomegilla_ulkei_221 | Tytthaspis_sedecimpunctata_582 | 0.167 |
| Hippodamia_notata_421 | Tytthaspis_sedecimpunctata_584 | 0.167 |
| Halyzia_sedecimguttata_349 | Tytthaspis_sedecimpunctata_586 | 0.167 |
| Halyzia_sedecimguttata_352 | Tytthaspis_sedecimpunctata_586 | 0.167 |
| Halyzia_sedecimguttata_353 | Tytthaspis_sedecimpunctata_586 | 0.167 |
| Halyzia_sedecimguttata_349 | Tytthaspis_sedecimpunctata_587 | 0.167 |
| Halyzia_sedecimguttata_352 | Tytthaspis_sedecimpunctata_587 | 0.167 |
| Halyzia_sedecimguttata_353 | Tytthaspis_sedecimpunctata_587 | 0.167 |
| Ceratomegilla_ulkei_222 | Tytthaspis_sedecimpunctata_588 | 0.167 |
| Ceratomegilla_ulkei_223 | Tytthaspis_sedecimpunctata_588 | 0.167 |
| Oenopia_conglobata_523 | Tytthaspis_sedecimpunctata_589 | 0.167 |
| Ceratomegilla_ulkei_221 | Tytthaspis_sedecimpunctata_590 | 0.167 |
| Halyzia_sedecimguttata_349 | Tytthaspis_sedecimpunctata_591 | 0.167 |

| | | |
|---|---|---|
| Halyzia_sedecimguttata_352 | Tytthaspis_sedecimpunctata_591 | 0.167 |
| Halyzia_sedecimguttata_353 | Tytthaspis_sedecimpunctata_591 | 0.167 |
| Ceratomegilla_ulkei_221 | Tytthaspis_sedecimpunctata_592 | 0.167 |
| Hippodamia_tredecimpunctata_459 | Tytthaspis_sedecimpunctata_593 | 0.167 |
| Ceratomegilla_ulkei_221 | Tytthaspis_sedecimpunctata_594 | 0.167 |
| Ceratomegilla_ulkei_221 | Tytthaspis_sedecimpunctata_595 | 0.167 |
| Scymnus_nigrinus_785 | Scymnus_sinuanodulus_828 | 0.167 |
| Scymnus_nigrinus_786 | Scymnus_sinuanodulus_828 | 0.167 |
| Hippodamia_caseyi_391 | Tytthaspis_sedecimpunctata_577 | 0.167 |
| Hippodamia_convergens_393 | Tytthaspis_sedecimpunctata_577 | 0.167 |
| Hippodamia_convergens_394 | Tytthaspis_sedecimpunctata_577 | 0.167 |
| Hippodamia_convergens_397 | Tytthaspis_sedecimpunctata_577 | 0.167 |
| Hippodamia_convergens_398 | Tytthaspis_sedecimpunctata_577 | 0.167 |
| Hippodamia_convergens_399 | Tytthaspis_sedecimpunctata_577 | 0.167 |
| Hippodamia_convergens_401 | Tytthaspis_sedecimpunctata_577 | 0.167 |
| Hippodamia_convergens_402 | Tytthaspis_sedecimpunctata_577 | 0.167 |
| Hippodamia_convergens_404 | Tytthaspis_sedecimpunctata_577 | 0.167 |
| Hippodamia_convergens_405 | Tytthaspis_sedecimpunctata_577 | 0.167 |
| Hippodamia_convergens_406 | Tytthaspis_sedecimpunctata_577 | 0.167 |
| Hippodamia_moesta_411 | Tytthaspis_sedecimpunctata_577 | 0.167 |
| Hippodamia_quinquesignata_443 | Tytthaspis_sedecimpunctata_577 | 0.167 |
| Hippodamia_quinquesignata_444 | Tytthaspis_sedecimpunctata_577 | 0.167 |
| Hippodamia_quinquesignata_445 | Tytthaspis_sedecimpunctata_577 | 0.167 |
| Hippodamia_caseyi_391 | Tytthaspis_sedecimpunctata_578 | 0.167 |
| Hippodamia_convergens_393 | Tytthaspis_sedecimpunctata_578 | 0.167 |
| Hippodamia_convergens_394 | Tytthaspis_sedecimpunctata_578 | 0.167 |
| Hippodamia_convergens_397 | Tytthaspis_sedecimpunctata_578 | 0.167 |
| Hippodamia_convergens_398 | Tytthaspis_sedecimpunctata_578 | 0.167 |
| Hippodamia_convergens_399 | Tytthaspis_sedecimpunctata_578 | 0.167 |
| Hippodamia_convergens_401 | Tytthaspis_sedecimpunctata_578 | 0.167 |
| Hippodamia_convergens_402 | Tytthaspis_sedecimpunctata_578 | 0.167 |
| Hippodamia_convergens_404 | Tytthaspis_sedecimpunctata_578 | 0.167 |
| Hippodamia_convergens_405 | Tytthaspis_sedecimpunctata_578 | 0.167 |
| Hippodamia_convergens_406 | Tytthaspis_sedecimpunctata_578 | 0.167 |
| Hippodamia_moesta_411 | Tytthaspis_sedecimpunctata_578 | 0.167 |
| Hippodamia_quinquesignata_443 | Tytthaspis_sedecimpunctata_578 | 0.167 |
| Hippodamia_quinquesignata_444 | Tytthaspis_sedecimpunctata_578 | 0.167 |
| Hippodamia_quinquesignata_445 | Tytthaspis_sedecimpunctata_578 | 0.167 |
| Hippodamia_caseyi_391 | Tytthaspis_sedecimpunctata_579 | 0.167 |
| Hippodamia_convergens_393 | Tytthaspis_sedecimpunctata_579 | 0.167 |
| Hippodamia_convergens_394 | Tytthaspis_sedecimpunctata_579 | 0.167 |
| Hippodamia_convergens_397 | Tytthaspis_sedecimpunctata_579 | 0.167 |
| Hippodamia_convergens_398 | Tytthaspis_sedecimpunctata_579 | 0.167 |
| Hippodamia_convergens_399 | Tytthaspis_sedecimpunctata_579 | 0.167 |
| Hippodamia_convergens_401 | Tytthaspis_sedecimpunctata_579 | 0.167 |
| Hippodamia_convergens_402 | Tytthaspis_sedecimpunctata_579 | 0.167 |
| Hippodamia_convergens_404 | Tytthaspis_sedecimpunctata_579 | 0.167 |
| Hippodamia_convergens_405 | Tytthaspis_sedecimpunctata_579 | 0.167 |
| Hippodamia_convergens_406 | Tytthaspis_sedecimpunctata_579 | 0.167 |
| Hippodamia_moesta_411 | Tytthaspis_sedecimpunctata_579 | 0.167 |
| Hippodamia_quinquesignata_443 | Tytthaspis_sedecimpunctata_579 | 0.167 |
| Hippodamia_quinquesignata_444 | Tytthaspis_sedecimpunctata_579 | 0.167 |
| Hippodamia_quinquesignata_445 | Tytthaspis_sedecimpunctata_579 | 0.167 |
| Oenopia_conglobata_523 | Tytthaspis_sedecimpunctata_580 | 0.167 |
| Hippodamia_caseyi_391 | Tytthaspis_sedecimpunctata_581 | 0.167 |
| Hippodamia_convergens_393 | Tytthaspis_sedecimpunctata_581 | 0.167 |

Supplementary Table S4 (continued)

| | | |
|---|---|---|
| Hippodamia_convergens_394 | Tytthaspis_sedecimpunctata_581 | 0.167 |
| Hippodamia_convergens_397 | Tytthaspis_sedecimpunctata_581 | 0.167 |
| Hippodamia_convergens_398 | Tytthaspis_sedecimpunctata_581 | 0.167 |
| Hippodamia_convergens_399 | Tytthaspis_sedecimpunctata_581 | 0.167 |
| Hippodamia_convergens_401 | Tytthaspis_sedecimpunctata_581 | 0.167 |
| Hippodamia_convergens_402 | Tytthaspis_sedecimpunctata_581 | 0.167 |
| Hippodamia_convergens_404 | Tytthaspis_sedecimpunctata_581 | 0.167 |
| Hippodamia_convergens_405 | Tytthaspis_sedecimpunctata_581 | 0.167 |
| Hippodamia_convergens_406 | Tytthaspis_sedecimpunctata_581 | 0.167 |
| Hippodamia_moesta_411 | Tytthaspis_sedecimpunctata_581 | 0.167 |
| Hippodamia_quinquesignata_443 | Tytthaspis_sedecimpunctata_581 | 0.167 |
| Hippodamia_quinquesignata_444 | Tytthaspis_sedecimpunctata_581 | 0.167 |
| Hippodamia_quinquesignata_445 | Tytthaspis_sedecimpunctata_581 | 0.167 |
| Hippodamia_caseyi_391 | Tytthaspis_sedecimpunctata_582 | 0.167 |
| Hippodamia_convergens_393 | Tytthaspis_sedecimpunctata_582 | 0.167 |
| Hippodamia_convergens_394 | Tytthaspis_sedecimpunctata_582 | 0.167 |
| Hippodamia_convergens_397 | Tytthaspis_sedecimpunctata_582 | 0.167 |
| Hippodamia_convergens_398 | Tytthaspis_sedecimpunctata_582 | 0.167 |
| Hippodamia_convergens_399 | Tytthaspis_sedecimpunctata_582 | 0.167 |
| Hippodamia_convergens_401 | Tytthaspis_sedecimpunctata_582 | 0.167 |
| Hippodamia_convergens_402 | Tytthaspis_sedecimpunctata_582 | 0.167 |
| Hippodamia_convergens_404 | Tytthaspis_sedecimpunctata_582 | 0.167 |
| Hippodamia_convergens_405 | Tytthaspis_sedecimpunctata_582 | 0.167 |
| Hippodamia_convergens_406 | Tytthaspis_sedecimpunctata_582 | 0.167 |
| Hippodamia_moesta_411 | Tytthaspis_sedecimpunctata_582 | 0.167 |
| Hippodamia_quinquesignata_443 | Tytthaspis_sedecimpunctata_582 | 0.167 |
| Hippodamia_quinquesignata_444 | Tytthaspis_sedecimpunctata_582 | 0.167 |
| Hippodamia_quinquesignata_445 | Tytthaspis_sedecimpunctata_582 | 0.167 |
| Hippodamia_convergens_396 | Tytthaspis_sedecimpunctata_583 | 0.167 |
| Hippodamia_convergens_400 | Tytthaspis_sedecimpunctata_583 | 0.167 |
| Hippodamia_convergens_403 | Tytthaspis_sedecimpunctata_583 | 0.167 |
| Hippodamia_moesta_410 | Tytthaspis_sedecimpunctata_583 | 0.167 |
| Calvia_decemguttata_195 | Tytthaspis_sedecimpunctata_584 | 0.167 |
| Calvia_decemguttata_199 | Tytthaspis_sedecimpunctata_584 | 0.167 |
| Calvia_decemguttata_200 | Tytthaspis_sedecimpunctata_584 | 0.167 |
| Ceratomegilla_ulkei_220 | Tytthaspis_sedecimpunctata_584 | 0.167 |
| Hippodamia_convergens_396 | Tytthaspis_sedecimpunctata_585 | 0.167 |
| Hippodamia_convergens_400 | Tytthaspis_sedecimpunctata_585 | 0.167 |
| Hippodamia_convergens_403 | Tytthaspis_sedecimpunctata_585 | 0.167 |
| Hippodamia_moesta_410 | Tytthaspis_sedecimpunctata_585 | 0.167 |
| Hippodamia_tredecimpunctata_447 | Tytthaspis_sedecimpunctata_586 | 0.167 |
| Hippodamia_tredecimpunctata_451 | Tytthaspis_sedecimpunctata_586 | 0.167 |
| Hippodamia_tredecimpunctata_454 | Tytthaspis_sedecimpunctata_586 | 0.167 |
| Hippodamia_tredecimpunctata_455 | Tytthaspis_sedecimpunctata_586 | 0.167 |
| Hippodamia_tredecimpunctata_456 | Tytthaspis_sedecimpunctata_586 | 0.167 |
| Hippodamia_tredecimpunctata_447 | Tytthaspis_sedecimpunctata_587 | 0.167 |
| Hippodamia_tredecimpunctata_451 | Tytthaspis_sedecimpunctata_587 | 0.167 |
| Hippodamia_tredecimpunctata_454 | Tytthaspis_sedecimpunctata_587 | 0.167 |
| Hippodamia_tredecimpunctata_455 | Tytthaspis_sedecimpunctata_587 | 0.167 |
| Hippodamia_tredecimpunctata_456 | Tytthaspis_sedecimpunctata_587 | 0.167 |
| Hippodamia_tredecimpunctata_446 | Tytthaspis_sedecimpunctata_588 | 0.167 |
| Hippodamia_tredecimpunctata_448 | Tytthaspis_sedecimpunctata_588 | 0.167 |
| Hippodamia_tredecimpunctata_449 | Tytthaspis_sedecimpunctata_588 | 0.167 |
| Hippodamia_tredecimpunctata_450 | Tytthaspis_sedecimpunctata_588 | 0.167 |
| Hippodamia_tredecimpunctata_452 | Tytthaspis_sedecimpunctata_588 | 0.167 |
| Hippodamia_tredecimpunctata_453 | Tytthaspis_sedecimpunctata_588 | 0.167 |

| | | |
|---|---|---|
| Oenopia_conglobata_523 | Tytthaspis_sedecimpunctata_588 | 0.167 |
| Ceratomegilla_ulkei_222 | Tytthaspis_sedecimpunctata_589 | 0.167 |
| Ceratomegilla_ulkei_223 | Tytthaspis_sedecimpunctata_589 | 0.167 |
| Hippodamia_caseyi_391 | Tytthaspis_sedecimpunctata_590 | 0.167 |
| Hippodamia_convergens_393 | Tytthaspis_sedecimpunctata_590 | 0.167 |
| Hippodamia_convergens_394 | Tytthaspis_sedecimpunctata_590 | 0.167 |
| Hippodamia_convergens_397 | Tytthaspis_sedecimpunctata_590 | 0.167 |
| Hippodamia_convergens_398 | Tytthaspis_sedecimpunctata_590 | 0.167 |
| Hippodamia_convergens_399 | Tytthaspis_sedecimpunctata_590 | 0.167 |
| Hippodamia_convergens_401 | Tytthaspis_sedecimpunctata_590 | 0.167 |
| Hippodamia_convergens_402 | Tytthaspis_sedecimpunctata_590 | 0.167 |
| Hippodamia_convergens_404 | Tytthaspis_sedecimpunctata_590 | 0.167 |
| Hippodamia_convergens_405 | Tytthaspis_sedecimpunctata_590 | 0.167 |
| Hippodamia_convergens_406 | Tytthaspis_sedecimpunctata_590 | 0.167 |
| Hippodamia_moesta_411 | Tytthaspis_sedecimpunctata_590 | 0.167 |
| Hippodamia_quinquesignata_443 | Tytthaspis_sedecimpunctata_590 | 0.167 |
| Hippodamia_quinquesignata_444 | Tytthaspis_sedecimpunctata_590 | 0.167 |
| Hippodamia_quinquesignata_445 | Tytthaspis_sedecimpunctata_590 | 0.167 |
| Ceratomegilla_ulkei_220 | Tytthaspis_sedecimpunctata_591 | 0.167 |
| Hippodamia_tredecimpunctata_459 | Tytthaspis_sedecimpunctata_591 | 0.167 |
| Hippodamia_caseyi_392 | Tytthaspis_sedecimpunctata_592 | 0.167 |
| Hippodamia_convergens_395 | Tytthaspis_sedecimpunctata_592 | 0.167 |
| Hippodamia_caseyi_391 | Tytthaspis_sedecimpunctata_593 | 0.167 |
| Hippodamia_convergens_393 | Tytthaspis_sedecimpunctata_593 | 0.167 |
| Hippodamia_convergens_394 | Tytthaspis_sedecimpunctata_593 | 0.167 |
| Hippodamia_convergens_397 | Tytthaspis_sedecimpunctata_593 | 0.167 |
| Hippodamia_convergens_398 | Tytthaspis_sedecimpunctata_593 | 0.167 |
| Hippodamia_convergens_399 | Tytthaspis_sedecimpunctata_593 | 0.167 |
| Hippodamia_convergens_401 | Tytthaspis_sedecimpunctata_593 | 0.167 |
| Hippodamia_convergens_402 | Tytthaspis_sedecimpunctata_593 | 0.167 |
| Hippodamia_convergens_404 | Tytthaspis_sedecimpunctata_593 | 0.167 |
| Hippodamia_convergens_405 | Tytthaspis_sedecimpunctata_593 | 0.167 |
| Hippodamia_convergens_406 | Tytthaspis_sedecimpunctata_593 | 0.167 |
| Hippodamia_moesta_411 | Tytthaspis_sedecimpunctata_593 | 0.167 |
| Hippodamia_quinquesignata_443 | Tytthaspis_sedecimpunctata_593 | 0.167 |
| Hippodamia_quinquesignata_444 | Tytthaspis_sedecimpunctata_593 | 0.167 |
| Hippodamia_quinquesignata_445 | Tytthaspis_sedecimpunctata_593 | 0.167 |
| Hippodamia_caseyi_391 | Tytthaspis_sedecimpunctata_594 | 0.167 |
| Hippodamia_convergens_393 | Tytthaspis_sedecimpunctata_594 | 0.167 |
| Hippodamia_convergens_394 | Tytthaspis_sedecimpunctata_594 | 0.167 |
| Hippodamia_convergens_397 | Tytthaspis_sedecimpunctata_594 | 0.167 |
| Hippodamia_convergens_398 | Tytthaspis_sedecimpunctata_594 | 0.167 |
| Hippodamia_convergens_399 | Tytthaspis_sedecimpunctata_594 | 0.167 |
| Hippodamia_convergens_401 | Tytthaspis_sedecimpunctata_594 | 0.167 |
| Hippodamia_convergens_402 | Tytthaspis_sedecimpunctata_594 | 0.167 |
| Hippodamia_convergens_404 | Tytthaspis_sedecimpunctata_594 | 0.167 |
| Hippodamia_convergens_405 | Tytthaspis_sedecimpunctata_594 | 0.167 |
| Hippodamia_convergens_406 | Tytthaspis_sedecimpunctata_594 | 0.167 |
| Hippodamia_moesta_411 | Tytthaspis_sedecimpunctata_594 | 0.167 |
| Hippodamia_quinquesignata_443 | Tytthaspis_sedecimpunctata_594 | 0.167 |
| Hippodamia_quinquesignata_444 | Tytthaspis_sedecimpunctata_594 | 0.167 |
| Hippodamia_quinquesignata_445 | Tytthaspis_sedecimpunctata_594 | 0.167 |
| Hippodamia_caseyi_391 | Tytthaspis_sedecimpunctata_595 | 0.167 |
| Hippodamia_convergens_393 | Tytthaspis_sedecimpunctata_595 | 0.167 |
| Hippodamia_convergens_394 | Tytthaspis_sedecimpunctata_595 | 0.167 |
| Hippodamia_convergens_397 | Tytthaspis_sedecimpunctata_595 | 0.167 |

| | | |
|---|---|---|
| Hippodamia_convergens_398 | Tytthaspis_sedecimpunctata_595 | 0.167 |
| Hippodamia_convergens_399 | Tytthaspis_sedecimpunctata_595 | 0.167 |
| Hippodamia_convergens_401 | Tytthaspis_sedecimpunctata_595 | 0.167 |
| Hippodamia_convergens_402 | Tytthaspis_sedecimpunctata_595 | 0.167 |
| Hippodamia_convergens_404 | Tytthaspis_sedecimpunctata_595 | 0.167 |
| Hippodamia_convergens_405 | Tytthaspis_sedecimpunctata_595 | 0.167 |
| Hippodamia_convergens_406 | Tytthaspis_sedecimpunctata_595 | 0.167 |
| Hippodamia_moesta_411 | Tytthaspis_sedecimpunctata_595 | 0.167 |
| Hippodamia_quinquesignata_443 | Tytthaspis_sedecimpunctata_595 | 0.167 |
| Hippodamia_quinquesignata_444 | Tytthaspis_sedecimpunctata_595 | 0.167 |
| Hippodamia_quinquesignata_445 | Tytthaspis_sedecimpunctata_595 | 0.167 |
| Scymnus_ningshanensis_788 | Scymnus_rubromaculatus_822 | 0.167 |
| Scymnus_ningshanensis_791 | Scymnus_rubromaculatus_822 | 0.167 |
| Scymnus_ningshanensis_792 | Scymnus_rubromaculatus_822 | 0.167 |
| Scymnus_ningshanensis_793 | Scymnus_rubromaculatus_822 | 0.167 |
| Scymnus_ningshanensis_794 | Scymnus_rubromaculatus_822 | 0.167 |
| Scymnus_ningshanensis_795 | Scymnus_rubromaculatus_822 | 0.167 |
| Scymnus_ningshanensis_788 | Scymnus_rubromaculatus_823 | 0.167 |
| Scymnus_ningshanensis_791 | Scymnus_rubromaculatus_823 | 0.167 |
| Scymnus_ningshanensis_792 | Scymnus_rubromaculatus_823 | 0.167 |
| Scymnus_ningshanensis_793 | Scymnus_rubromaculatus_823 | 0.167 |
| Scymnus_ningshanensis_794 | Scymnus_rubromaculatus_823 | 0.167 |
| Scymnus_ningshanensis_795 | Scymnus_rubromaculatus_823 | 0.167 |
| Scymnus_ningshanensis_788 | Scymnus_rubromaculatus_824 | 0.167 |
| Scymnus_ningshanensis_791 | Scymnus_rubromaculatus_824 | 0.167 |
| Scymnus_ningshanensis_792 | Scymnus_rubromaculatus_824 | 0.167 |
| Scymnus_ningshanensis_793 | Scymnus_rubromaculatus_824 | 0.167 |
| Scymnus_ningshanensis_794 | Scymnus_rubromaculatus_824 | 0.167 |
| Scymnus_ningshanensis_795 | Scymnus_rubromaculatus_824 | 0.167 |
| Scymnus_ningshanensis_788 | Scymnus_rubromaculatus_825 | 0.167 |
| Scymnus_ningshanensis_791 | Scymnus_rubromaculatus_825 | 0.167 |
| Scymnus_ningshanensis_792 | Scymnus_rubromaculatus_825 | 0.167 |
| Scymnus_ningshanensis_793 | Scymnus_rubromaculatus_825 | 0.167 |
| Scymnus_ningshanensis_794 | Scymnus_rubromaculatus_825 | 0.167 |
| Scymnus_ningshanensis_795 | Scymnus_rubromaculatus_825 | 0.167 |
| Scymnus_ningshanensis_788 | Scymnus_rubromaculatus_826 | 0.167 |
| Scymnus_ningshanensis_791 | Scymnus_rubromaculatus_826 | 0.167 |
| Scymnus_ningshanensis_792 | Scymnus_rubromaculatus_826 | 0.167 |
| Scymnus_ningshanensis_793 | Scymnus_rubromaculatus_826 | 0.167 |
| Scymnus_ningshanensis_794 | Scymnus_rubromaculatus_826 | 0.167 |
| Scymnus_ningshanensis_795 | Scymnus_rubromaculatus_826 | 0.167 |
| Scymnus_ningshanensis_788 | Scymnus_rubromaculatus_827 | 0.167 |
| Scymnus_ningshanensis_791 | Scymnus_rubromaculatus_827 | 0.167 |
| Scymnus_ningshanensis_792 | Scymnus_rubromaculatus_827 | 0.167 |
| Scymnus_ningshanensis_793 | Scymnus_rubromaculatus_827 | 0.167 |
| Scymnus_ningshanensis_794 | Scymnus_rubromaculatus_827 | 0.167 |
| Scymnus_ningshanensis_795 | Scymnus_rubromaculatus_827 | 0.167 |
| Psyllobora_vigintiduopunctata_566 | Tytthaspis_sedecimpunctata_586 | 0.167 |
| Brumus_quadripustulatus_10 | Tytthaspis_sedecimpunctata_585 | 0.167 |
| Brumus_quadripustulatus_10 | Tytthaspis_sedecimpunctata_593 | 0.167 |
| Psyllobora_vigintiduopunctata_569 | Tytthaspis_sedecimpunctata_577 | 0.167 |
| Psyllobora_vigintiduopunctata_569 | Tytthaspis_sedecimpunctata_578 | 0.167 |
| Psyllobora_vigintiduopunctata_569 | Tytthaspis_sedecimpunctata_579 | 0.167 |
| Psyllobora_vigintiduopunctata_569 | Tytthaspis_sedecimpunctata_581 | 0.167 |
| Psyllobora_vigintiduopunctata_569 | Tytthaspis_sedecimpunctata_582 | 0.167 |
| Psyllobora_vigintiduopunctata_569 | Tytthaspis_sedecimpunctata_590 | 0.167 |

| | | |
|---|---|---|
| Psyllobora_vigintiduopunctata_566 | Tytthaspis_sedecimpunctata_593 | 0.167 |
| Psyllobora_vigintiduopunctata_569 | Tytthaspis_sedecimpunctata_593 | 0.167 |
| Psyllobora_vigintiduopunctata_569 | Tytthaspis_sedecimpunctata_594 | 0.167 |
| Psyllobora_vigintiduopunctata_569 | Tytthaspis_sedecimpunctata_595 | 0.167 |
| Hyperaspis_proba_660 | Scymnus_suturalis_838 | 0.167 |
| Hyperaspis_proba_660 | Scymnus_suturalis_840 | 0.167 |
| Calvia_decemguttata_195 | Tytthaspis_sedecimpunctata_577 | 0.167 |
| Calvia_decemguttata_199 | Tytthaspis_sedecimpunctata_577 | 0.167 |
| Calvia_decemguttata_200 | Tytthaspis_sedecimpunctata_577 | 0.167 |
| Hippodamia_tredecimpunctata_447 | Tytthaspis_sedecimpunctata_577 | 0.167 |
| Hippodamia_tredecimpunctata_451 | Tytthaspis_sedecimpunctata_577 | 0.167 |
| Hippodamia_tredecimpunctata_454 | Tytthaspis_sedecimpunctata_577 | 0.167 |
| Hippodamia_tredecimpunctata_455 | Tytthaspis_sedecimpunctata_577 | 0.167 |
| Hippodamia_tredecimpunctata_456 | Tytthaspis_sedecimpunctata_577 | 0.167 |
| Calvia_decemguttata_195 | Tytthaspis_sedecimpunctata_578 | 0.167 |
| Calvia_decemguttata_199 | Tytthaspis_sedecimpunctata_578 | 0.167 |
| Calvia_decemguttata_200 | Tytthaspis_sedecimpunctata_578 | 0.167 |
| Hippodamia_tredecimpunctata_447 | Tytthaspis_sedecimpunctata_578 | 0.167 |
| Hippodamia_tredecimpunctata_451 | Tytthaspis_sedecimpunctata_578 | 0.167 |
| Hippodamia_tredecimpunctata_454 | Tytthaspis_sedecimpunctata_578 | 0.167 |
| Hippodamia_tredecimpunctata_455 | Tytthaspis_sedecimpunctata_578 | 0.167 |
| Hippodamia_tredecimpunctata_456 | Tytthaspis_sedecimpunctata_578 | 0.167 |
| Calvia_decemguttata_195 | Tytthaspis_sedecimpunctata_579 | 0.167 |
| Calvia_decemguttata_199 | Tytthaspis_sedecimpunctata_579 | 0.167 |
| Calvia_decemguttata_200 | Tytthaspis_sedecimpunctata_579 | 0.167 |
| Hippodamia_tredecimpunctata_447 | Tytthaspis_sedecimpunctata_579 | 0.167 |
| Hippodamia_tredecimpunctata_451 | Tytthaspis_sedecimpunctata_579 | 0.167 |
| Hippodamia_tredecimpunctata_454 | Tytthaspis_sedecimpunctata_579 | 0.167 |
| Hippodamia_tredecimpunctata_455 | Tytthaspis_sedecimpunctata_579 | 0.167 |
| Hippodamia_tredecimpunctata_456 | Tytthaspis_sedecimpunctata_579 | 0.167 |
| Hippodamia_caseyi_392 | Tytthaspis_sedecimpunctata_580 | 0.167 |
| Hippodamia_convergens_395 | Tytthaspis_sedecimpunctata_580 | 0.167 |
| Calvia_decemguttata_195 | Tytthaspis_sedecimpunctata_581 | 0.167 |
| Calvia_decemguttata_199 | Tytthaspis_sedecimpunctata_581 | 0.167 |
| Calvia_decemguttata_200 | Tytthaspis_sedecimpunctata_581 | 0.167 |
| Hippodamia_tredecimpunctata_447 | Tytthaspis_sedecimpunctata_581 | 0.167 |
| Hippodamia_tredecimpunctata_451 | Tytthaspis_sedecimpunctata_581 | 0.167 |
| Hippodamia_tredecimpunctata_454 | Tytthaspis_sedecimpunctata_581 | 0.167 |
| Hippodamia_tredecimpunctata_455 | Tytthaspis_sedecimpunctata_581 | 0.167 |
| Hippodamia_tredecimpunctata_456 | Tytthaspis_sedecimpunctata_581 | 0.167 |
| Calvia_decemguttata_195 | Tytthaspis_sedecimpunctata_582 | 0.167 |
| Calvia_decemguttata_199 | Tytthaspis_sedecimpunctata_582 | 0.167 |
| Calvia_decemguttata_200 | Tytthaspis_sedecimpunctata_582 | 0.167 |
| Hippodamia_tredecimpunctata_447 | Tytthaspis_sedecimpunctata_582 | 0.167 |
| Hippodamia_tredecimpunctata_451 | Tytthaspis_sedecimpunctata_582 | 0.167 |
| Hippodamia_tredecimpunctata_454 | Tytthaspis_sedecimpunctata_582 | 0.167 |
| Hippodamia_tredecimpunctata_455 | Tytthaspis_sedecimpunctata_582 | 0.167 |
| Hippodamia_tredecimpunctata_456 | Tytthaspis_sedecimpunctata_582 | 0.167 |
| Hippodamia_tredecimpunctata_446 | Tytthaspis_sedecimpunctata_583 | 0.167 |
| Hippodamia_tredecimpunctata_448 | Tytthaspis_sedecimpunctata_583 | 0.167 |
| Hippodamia_tredecimpunctata_449 | Tytthaspis_sedecimpunctata_583 | 0.167 |
| Hippodamia_tredecimpunctata_450 | Tytthaspis_sedecimpunctata_583 | 0.167 |
| Hippodamia_tredecimpunctata_452 | Tytthaspis_sedecimpunctata_583 | 0.167 |
| Hippodamia_tredecimpunctata_453 | Tytthaspis_sedecimpunctata_583 | 0.167 |
| Halyzia_sedecimguttata_349 | Tytthaspis_sedecimpunctata_584 | 0.167 |
| Halyzia_sedecimguttata_352 | Tytthaspis_sedecimpunctata_584 | 0.167 |

Supplementary Table S4 (continued)

| | | |
|---|---|---|
| Halyzia_sedecimguttata_353 | Tytthaspis_sedecimpunctata_584 | 0.167 |
| Hippodamia_convergens_396 | Tytthaspis_sedecimpunctata_584 | 0.167 |
| Hippodamia_convergens_400 | Tytthaspis_sedecimpunctata_584 | 0.167 |
| Hippodamia_convergens_403 | Tytthaspis_sedecimpunctata_584 | 0.167 |
| Hippodamia_moesta_410 | Tytthaspis_sedecimpunctata_584 | 0.167 |
| Calvia_decemguttata_195 | Tytthaspis_sedecimpunctata_585 | 0.167 |
| Calvia_decemguttata_199 | Tytthaspis_sedecimpunctata_585 | 0.167 |
| Calvia_decemguttata_200 | Tytthaspis_sedecimpunctata_585 | 0.167 |
| Coccinula_quatuordecimpustulata_297 | Tytthaspis_sedecimpunctata_585 | 0.167 |
| Halyzia_sedecimguttata_346 | Tytthaspis_sedecimpunctata_585 | 0.167 |
| Halyzia_sedecimguttata_347 | Tytthaspis_sedecimpunctata_585 | 0.167 |
| Halyzia_sedecimguttata_348 | Tytthaspis_sedecimpunctata_585 | 0.167 |
| Hippodamia_tredecimpunctata_446 | Tytthaspis_sedecimpunctata_585 | 0.167 |
| Hippodamia_tredecimpunctata_448 | Tytthaspis_sedecimpunctata_585 | 0.167 |
| Hippodamia_tredecimpunctata_449 | Tytthaspis_sedecimpunctata_585 | 0.167 |
| Hippodamia_tredecimpunctata_450 | Tytthaspis_sedecimpunctata_585 | 0.167 |
| Hippodamia_tredecimpunctata_452 | Tytthaspis_sedecimpunctata_585 | 0.167 |
| Hippodamia_tredecimpunctata_453 | Tytthaspis_sedecimpunctata_585 | 0.167 |
| Oenopia_conglobata_523 | Tytthaspis_sedecimpunctata_585 | 0.167 |
| Hippodamia_parenthesis_439 | Tytthaspis_sedecimpunctata_587 | 0.167 |
| Calvia_decemguttata_195 | Tytthaspis_sedecimpunctata_590 | 0.167 |
| Calvia_decemguttata_199 | Tytthaspis_sedecimpunctata_590 | 0.167 |
| Calvia_decemguttata_200 | Tytthaspis_sedecimpunctata_590 | 0.167 |
| Hippodamia_tredecimpunctata_447 | Tytthaspis_sedecimpunctata_590 | 0.167 |
| Hippodamia_tredecimpunctata_451 | Tytthaspis_sedecimpunctata_590 | 0.167 |
| Hippodamia_tredecimpunctata_454 | Tytthaspis_sedecimpunctata_590 | 0.167 |
| Hippodamia_tredecimpunctata_455 | Tytthaspis_sedecimpunctata_590 | 0.167 |
| Hippodamia_tredecimpunctata_456 | Tytthaspis_sedecimpunctata_590 | 0.167 |
| Ceratomegilla_ulkei_222 | Tytthaspis_sedecimpunctata_591 | 0.167 |
| Ceratomegilla_ulkei_223 | Tytthaspis_sedecimpunctata_591 | 0.167 |
| Calvia_decemguttata_195 | Tytthaspis_sedecimpunctata_592 | 0.167 |
| Calvia_decemguttata_199 | Tytthaspis_sedecimpunctata_592 | 0.167 |
| Calvia_decemguttata_200 | Tytthaspis_sedecimpunctata_592 | 0.167 |
| Hippodamia_tredecimpunctata_446 | Tytthaspis_sedecimpunctata_592 | 0.167 |
| Hippodamia_tredecimpunctata_448 | Tytthaspis_sedecimpunctata_592 | 0.167 |
| Hippodamia_tredecimpunctata_449 | Tytthaspis_sedecimpunctata_592 | 0.167 |
| Hippodamia_tredecimpunctata_450 | Tytthaspis_sedecimpunctata_592 | 0.167 |
| Hippodamia_tredecimpunctata_452 | Tytthaspis_sedecimpunctata_592 | 0.167 |
| Hippodamia_tredecimpunctata_453 | Tytthaspis_sedecimpunctata_592 | 0.167 |
| Calvia_decemguttata_195 | Tytthaspis_sedecimpunctata_594 | 0.167 |
| Calvia_decemguttata_199 | Tytthaspis_sedecimpunctata_594 | 0.167 |
| Calvia_decemguttata_200 | Tytthaspis_sedecimpunctata_594 | 0.167 |
| Hippodamia_tredecimpunctata_447 | Tytthaspis_sedecimpunctata_594 | 0.167 |
| Hippodamia_tredecimpunctata_451 | Tytthaspis_sedecimpunctata_594 | 0.167 |
| Hippodamia_tredecimpunctata_454 | Tytthaspis_sedecimpunctata_594 | 0.167 |
| Hippodamia_tredecimpunctata_455 | Tytthaspis_sedecimpunctata_594 | 0.167 |
| Hippodamia_tredecimpunctata_456 | Tytthaspis_sedecimpunctata_594 | 0.167 |
| Calvia_decemguttata_195 | Tytthaspis_sedecimpunctata_595 | 0.167 |
| Calvia_decemguttata_199 | Tytthaspis_sedecimpunctata_595 | 0.167 |
| Calvia_decemguttata_200 | Tytthaspis_sedecimpunctata_595 | 0.167 |
| Hippodamia_tredecimpunctata_447 | Tytthaspis_sedecimpunctata_595 | 0.167 |
| Hippodamia_tredecimpunctata_451 | Tytthaspis_sedecimpunctata_595 | 0.167 |
| Hippodamia_tredecimpunctata_454 | Tytthaspis_sedecimpunctata_595 | 0.167 |
| Hippodamia_tredecimpunctata_455 | Tytthaspis_sedecimpunctata_595 | 0.167 |
| Hippodamia_tredecimpunctata_456 | Tytthaspis_sedecimpunctata_595 | 0.167 |
| Psyllobora_vigintiduopunctata_559 | Tytthaspis_sedecimpunctata_584 | 0.167 |

| | | |
|---|---|---|
| Hyperaspis_proba_660 | Scymnus_suturalis_834 | 0.167 |
| Scymnus_apetzi_693 | Scymnus_sinuanodulus_831 | 0.167 |
| Calvia_decemguttata_199 | Tytthaspis_sedecimpunctata_580 | 0.167 |
| Calvia_decemguttata_200 | Tytthaspis_sedecimpunctata_580 | 0.167 |
| Hippodamia_tredecimpunctata_446 | Tytthaspis_sedecimpunctata_580 | 0.167 |
| Hippodamia_tredecimpunctata_448 | Tytthaspis_sedecimpunctata_580 | 0.167 |
| Hippodamia_tredecimpunctata_449 | Tytthaspis_sedecimpunctata_580 | 0.167 |
| Hippodamia_tredecimpunctata_450 | Tytthaspis_sedecimpunctata_580 | 0.167 |
| Hippodamia_tredecimpunctata_452 | Tytthaspis_sedecimpunctata_580 | 0.167 |
| Hippodamia_tredecimpunctata_453 | Tytthaspis_sedecimpunctata_580 | 0.167 |
| Coccinula_quatuordecimpustulata_304 | Tytthaspis_sedecimpunctata_586 | 0.167 |
| Hippodamia_parenthesis_433 | Tytthaspis_sedecimpunctata_587 | 0.167 |
| Hippodamia_parenthesis_440 | Tytthaspis_sedecimpunctata_587 | 0.167 |
| Coccinula_quatuordecimpustulata_306 | Tytthaspis_sedecimpunctata_588 | 0.167 |
| Calvia_quatuordecimguttata_203 | Tytthaspis_sedecimpunctata_589 | 0.167 |
| Coccinula_quatuordecimpustulata_294 | Tytthaspis_sedecimpunctata_589 | 0.167 |
| Coccinula_quatuordecimpustulata_295 | Tytthaspis_sedecimpunctata_589 | 0.167 |
| Coccinula_quatuordecimpustulata_308 | Tytthaspis_sedecimpunctata_589 | 0.167 |
| Scymnus_haemorrhoidalis_749 | Scymnus_suturalis_833 | 0.167 |
| Scymnus_haemorrhoidalis_751 | Scymnus_suturalis_833 | 0.167 |
| Scymnus_haemorrhoidalis_754 | Scymnus_suturalis_833 | 0.167 |
| Scymnus_haemorrhoidalis_757 | Scymnus_suturalis_833 | 0.167 |
| Scymnus_haemorrhoidalis_758 | Scymnus_suturalis_833 | 0.167 |
| Scymnus_haemorrhoidalis_749 | Scymnus_suturalis_838 | 0.167 |
| Scymnus_haemorrhoidalis_751 | Scymnus_suturalis_838 | 0.167 |
| Scymnus_haemorrhoidalis_754 | Scymnus_suturalis_838 | 0.167 |
| Scymnus_haemorrhoidalis_757 | Scymnus_suturalis_838 | 0.167 |
| Scymnus_haemorrhoidalis_758 | Scymnus_suturalis_838 | 0.167 |
| Hippodamia_arctica_390 | Tytthaspis_sedecimpunctata_583 | 0.167 |
| Hippodamia_arctica_390 | Tytthaspis_sedecimpunctata_585 | 0.167 |
| Hippodamia_parenthesis_439 | Tytthaspis_sedecimpunctata_584 | 0.167 |
| Coccinula_quatuordecimpustulata_300 | Tytthaspis_sedecimpunctata_586 | 0.167 |
| Coccinula_quatuordecimpustulata_305 | Tytthaspis_sedecimpunctata_587 | 0.167 |
| Coccinula_quatuordecimpustulata_298 | Tytthaspis_sedecimpunctata_588 | 0.167 |
| Coccinula_quatuordecimpustulata_299 | Tytthaspis_sedecimpunctata_588 | 0.167 |
| Coccinula_quatuordecimpustulata_301 | Tytthaspis_sedecimpunctata_588 | 0.167 |
| Coccinula_quatuordecimpustulata_302 | Tytthaspis_sedecimpunctata_588 | 0.167 |
| Coccinula_quatuordecimpustulata_303 | Tytthaspis_sedecimpunctata_588 | 0.167 |
| Coccinula_quatuordecimpustulata_309 | Tytthaspis_sedecimpunctata_588 | 0.167 |
| Coccinula_quatuordecimpustulata_295 | Tytthaspis_sedecimpunctata_591 | 0.167 |
| Coccinula_quatuordecimpustulata_307 | Tytthaspis_sedecimpunctata_591 | 0.167 |
| Hippodamia_parenthesis_439 | Tytthaspis_sedecimpunctata_591 | 0.167 |
| Macronaemia_episcopalis_477 | Tytthaspis_sedecimpunctata_591 | 0.167 |
| Scymnus_haemorrhoidalis_749 | Scymnus_suturalis_834 | 0.167 |
| Scymnus_haemorrhoidalis_751 | Scymnus_suturalis_834 | 0.167 |
| Scymnus_haemorrhoidalis_754 | Scymnus_suturalis_834 | 0.167 |
| Scymnus_haemorrhoidalis_757 | Scymnus_suturalis_834 | 0.167 |
| Scymnus_haemorrhoidalis_758 | Scymnus_suturalis_834 | 0.167 |
| Hippodamia_arctica_390 | Tytthaspis_sedecimpunctata_584 | 0.167 |
| Oenopia_conglobata_524 | Tytthaspis_sedecimpunctata_586 | 0.167 |
| Hyperaspis_proba_660 | Scymnus_rubromaculatus_822 | 0.167 |
| Hyperaspis_proba_660 | Scymnus_rubromaculatus_823 | 0.167 |
| Hyperaspis_proba_660 | Scymnus_rubromaculatus_824 | 0.167 |
| Hyperaspis_proba_660 | Scymnus_rubromaculatus_825 | 0.167 |
| Hyperaspis_proba_660 | Scymnus_rubromaculatus_826 | 0.167 |
| Hyperaspis_proba_660 | Scymnus_rubromaculatus_827 | 0.167 |

| | | |
|---|---|---|
| Psyllobora_vigintiduopunctata_568 | Tytthaspis_sedecimpunctata_593 | 0.167 |
| Hippodamia_parenthesis_433 | Tytthaspis_sedecimpunctata_584 | 0.167 |
| Hippodamia_parenthesis_440 | Tytthaspis_sedecimpunctata_584 | 0.167 |
| Calvia_quatuordecimguttata_205 | Tytthaspis_sedecimpunctata_587 | 0.167 |
| Coccinula_quatuordecimpustulata_296 | Tytthaspis_sedecimpunctata_587 | 0.167 |
| Hippodamia_parenthesis_433 | Tytthaspis_sedecimpunctata_591 | 0.167 |
| Hippodamia_parenthesis_440 | Tytthaspis_sedecimpunctata_591 | 0.167 |
| Scymnus_apetzi_694 | Scymnus_suturalis_843 | 0.167 |
| Scymnus_apetzi_694 | Scymnus_suturalis_837 | 0.167 |
| Scymnus_auritus_699 | Scymnus_sinuanodulus_830 | 0.167 |
| Scymnus_mitior_779 | Scymnus_sinuanodulus_831 | 0.167 |
| Scymnus_nubilus_799 | Scymnus_rubromaculatus_822 | 0.167 |
| Scymnus_nubilus_799 | Scymnus_rubromaculatus_823 | 0.167 |
| Scymnus_nubilus_799 | Scymnus_rubromaculatus_824 | 0.167 |
| Scymnus_nubilus_799 | Scymnus_rubromaculatus_825 | 0.167 |
| Scymnus_nubilus_799 | Scymnus_rubromaculatus_826 | 0.167 |
| Scymnus_nubilus_799 | Scymnus_rubromaculatus_827 | 0.167 |
| Scymnus_apetzi_693 | Scymnus_sinuanodulus_829 | 0.167 |
| Scymnus_nubilus_798 | Scymnus_rubromaculatus_822 | 0.167 |
| Scymnus_nubilus_800 | Scymnus_rubromaculatus_822 | 0.167 |
| Scymnus_nubilus_801 | Scymnus_rubromaculatus_822 | 0.167 |
| Scymnus_nubilus_805 | Scymnus_rubromaculatus_822 | 0.167 |
| Scymnus_nubilus_806 | Scymnus_rubromaculatus_822 | 0.167 |
| Scymnus_nubilus_807 | Scymnus_rubromaculatus_822 | 0.167 |
| Scymnus_nubilus_808 | Scymnus_rubromaculatus_822 | 0.167 |
| Scymnus_nubilus_810 | Scymnus_rubromaculatus_822 | 0.167 |
| Scymnus_nubilus_798 | Scymnus_rubromaculatus_823 | 0.167 |
| Scymnus_nubilus_800 | Scymnus_rubromaculatus_823 | 0.167 |
| Scymnus_nubilus_801 | Scymnus_rubromaculatus_823 | 0.167 |
| Scymnus_nubilus_805 | Scymnus_rubromaculatus_823 | 0.167 |
| Scymnus_nubilus_806 | Scymnus_rubromaculatus_823 | 0.167 |
| Scymnus_nubilus_807 | Scymnus_rubromaculatus_823 | 0.167 |
| Scymnus_nubilus_808 | Scymnus_rubromaculatus_823 | 0.167 |
| Scymnus_nubilus_810 | Scymnus_rubromaculatus_823 | 0.167 |
| Scymnus_nubilus_798 | Scymnus_rubromaculatus_824 | 0.167 |
| Scymnus_nubilus_800 | Scymnus_rubromaculatus_824 | 0.167 |
| Scymnus_nubilus_801 | Scymnus_rubromaculatus_824 | 0.167 |
| Scymnus_nubilus_805 | Scymnus_rubromaculatus_824 | 0.167 |
| Scymnus_nubilus_806 | Scymnus_rubromaculatus_824 | 0.167 |
| Scymnus_nubilus_807 | Scymnus_rubromaculatus_824 | 0.167 |
| Scymnus_nubilus_808 | Scymnus_rubromaculatus_824 | 0.167 |
| Scymnus_nubilus_810 | Scymnus_rubromaculatus_824 | 0.167 |
| Scymnus_nubilus_798 | Scymnus_rubromaculatus_825 | 0.167 |
| Scymnus_nubilus_800 | Scymnus_rubromaculatus_825 | 0.167 |
| Scymnus_nubilus_801 | Scymnus_rubromaculatus_825 | 0.167 |
| Scymnus_nubilus_805 | Scymnus_rubromaculatus_825 | 0.167 |
| Scymnus_nubilus_806 | Scymnus_rubromaculatus_825 | 0.167 |
| Scymnus_nubilus_807 | Scymnus_rubromaculatus_825 | 0.167 |
| Scymnus_nubilus_808 | Scymnus_rubromaculatus_825 | 0.167 |
| Scymnus_nubilus_810 | Scymnus_rubromaculatus_825 | 0.167 |
| Scymnus_nubilus_798 | Scymnus_rubromaculatus_826 | 0.167 |
| Scymnus_nubilus_800 | Scymnus_rubromaculatus_826 | 0.167 |
| Scymnus_nubilus_801 | Scymnus_rubromaculatus_826 | 0.167 |
| Scymnus_nubilus_805 | Scymnus_rubromaculatus_826 | 0.167 |
| Scymnus_nubilus_806 | Scymnus_rubromaculatus_826 | 0.167 |
| Scymnus_nubilus_807 | Scymnus_rubromaculatus_826 | 0.167 |

Supplementary Table S4 (continued)

| | | |
|---|---|---|
| Scymnus_nubilus_808 | Scymnus_rubromaculatus_826 | 0.167 |
| Scymnus_nubilus_810 | Scymnus_rubromaculatus_826 | 0.167 |
| Scymnus_nubilus_798 | Scymnus_rubromaculatus_827 | 0.167 |
| Scymnus_nubilus_800 | Scymnus_rubromaculatus_827 | 0.167 |
| Scymnus_nubilus_801 | Scymnus_rubromaculatus_827 | 0.167 |
| Scymnus_nubilus_805 | Scymnus_rubromaculatus_827 | 0.167 |
| Scymnus_nubilus_806 | Scymnus_rubromaculatus_827 | 0.167 |
| Scymnus_nubilus_807 | Scymnus_rubromaculatus_827 | 0.167 |
| Scymnus_nubilus_808 | Scymnus_rubromaculatus_827 | 0.167 |
| Scymnus_nubilus_810 | Scymnus_rubromaculatus_827 | 0.167 |
| Scymnus_apetzi_693 | Scymnus_suturalis_838 | 0.167 |
| Scymnus_apetzi_693 | Scymnus_suturalis_840 | 0.167 |
| Myrrha_octodecimguttata_488 | Vibidia_duodecimguttata_596 | 0.167 |
| Myrrha_octodecimguttata_491 | Vibidia_duodecimguttata_596 | 0.167 |
| Myrrha_octodecimguttata_492 | Vibidia_duodecimguttata_596 | 0.167 |
| Myrrha_octodecimguttata_499 | Vibidia_duodecimguttata_596 | 0.167 |
| Myrrha_octodecimguttata_499 | Vibidia_duodecimguttata_601 | 0.167 |
| Myrrha_octodecimguttata_488 | Vibidia_duodecimguttata_603 | 0.167 |
| Myrrha_octodecimguttata_491 | Vibidia_duodecimguttata_603 | 0.167 |
| Myrrha_octodecimguttata_492 | Vibidia_duodecimguttata_603 | 0.167 |
| Scymnus_apetzi_693 | Scymnus_suturalis_834 | 0.168 |
| Scymnus_apetzi_691 | Scymnus_sinuanodulus_831 | 0.168 |
| Scymnus_auritus_702 | Scymnus_sinuanodulus_831 | 0.168 |
| Platynaspis_luteorubra_50 | Scymnus_suturalis_843 | 0.168 |
| Platynaspis_luteorubra_50 | Scymnus_suturalis_845 | 0.168 |
| Platynaspis_luteorubra_50 | Scymnus_suturalis_837 | 0.168 |
| Calvia_quatuordecimguttata_206 | Vibidia_duodecimguttata_596 | 0.168 |
| Calvia_quatuordecimguttata_207 | Vibidia_duodecimguttata_596 | 0.168 |
| Calvia_quatuordecimguttata_216 | Vibidia_duodecimguttata_596 | 0.168 |
| Calvia_quatuordecimguttata_211 | Vibidia_duodecimguttata_597 | 0.168 |
| Calvia_quatuordecimguttata_212 | Vibidia_duodecimguttata_597 | 0.168 |
| Calvia_quatuordecimguttata_213 | Vibidia_duodecimguttata_597 | 0.168 |
| Calvia_quatuordecimguttata_214 | Vibidia_duodecimguttata_597 | 0.168 |
| Calvia_quatuordecimguttata_215 | Vibidia_duodecimguttata_597 | 0.168 |
| Myrrha_octodecimguttata_489 | Vibidia_duodecimguttata_597 | 0.168 |
| Myrrha_octodecimguttata_500 | Vibidia_duodecimguttata_597 | 0.168 |
| Calvia_quatuordecimguttata_211 | Vibidia_duodecimguttata_600 | 0.168 |
| Calvia_quatuordecimguttata_212 | Vibidia_duodecimguttata_600 | 0.168 |
| Calvia_quatuordecimguttata_213 | Vibidia_duodecimguttata_600 | 0.168 |
| Calvia_quatuordecimguttata_214 | Vibidia_duodecimguttata_600 | 0.168 |
| Calvia_quatuordecimguttata_215 | Vibidia_duodecimguttata_600 | 0.168 |
| Myrrha_octodecimguttata_489 | Vibidia_duodecimguttata_600 | 0.168 |
| Myrrha_octodecimguttata_500 | Vibidia_duodecimguttata_600 | 0.168 |
| Calvia_quatuordecimguttata_201 | Vibidia_duodecimguttata_596 | 0.168 |
| Myrrha_octodecimguttata_493 | Vibidia_duodecimguttata_596 | 0.168 |
| Myrrha_octodecimguttata_498 | Vibidia_duodecimguttata_596 | 0.168 |
| Anatis_labiculata_126 | Vibidia_duodecimguttata_597 | 0.168 |
| Calvia_quatuordecimguttata_202 | Vibidia_duodecimguttata_598 | 0.168 |
| Calvia_quatuordecimguttata_210 | Vibidia_duodecimguttata_598 | 0.168 |
| Calvia_quatuordecimguttata_202 | Vibidia_duodecimguttata_599 | 0.168 |
| Calvia_quatuordecimguttata_210 | Vibidia_duodecimguttata_599 | 0.168 |
| Anatis_labiculata_126 | Vibidia_duodecimguttata_600 | 0.168 |
| Calvia_quatuordecimguttata_202 | Vibidia_duodecimguttata_601 | 0.168 |
| Calvia_quatuordecimguttata_210 | Vibidia_duodecimguttata_601 | 0.168 |
| Myrrha_octodecimguttata_493 | Vibidia_duodecimguttata_601 | 0.168 |
| Myrrha_octodecimguttata_498 | Vibidia_duodecimguttata_601 | 0.168 |

| | | |
|---|---|---|
| Calvia_quatuordecimguttata_202 | Vibidia_duodecimguttata_602 | 0.168 |
| Calvia_quatuordecimguttata_210 | Vibidia_duodecimguttata_602 | 0.168 |
| Calvia_quatuordecimguttata_211 | Vibidia_duodecimguttata_603 | 0.168 |
| Calvia_quatuordecimguttata_212 | Vibidia_duodecimguttata_603 | 0.168 |
| Calvia_quatuordecimguttata_213 | Vibidia_duodecimguttata_603 | 0.168 |
| Calvia_quatuordecimguttata_214 | Vibidia_duodecimguttata_603 | 0.168 |
| Calvia_quatuordecimguttata_215 | Vibidia_duodecimguttata_603 | 0.168 |
| Scymnus_abietis_682 | Scymnus_rubromaculatus_822 | 0.168 |
| Scymnus_abietis_685 | Scymnus_rubromaculatus_822 | 0.168 |
| Scymnus_abietis_682 | Scymnus_rubromaculatus_823 | 0.168 |
| Scymnus_abietis_685 | Scymnus_rubromaculatus_823 | 0.168 |
| Scymnus_abietis_682 | Scymnus_rubromaculatus_824 | 0.168 |
| Scymnus_abietis_685 | Scymnus_rubromaculatus_824 | 0.168 |
| Scymnus_abietis_682 | Scymnus_rubromaculatus_825 | 0.168 |
| Scymnus_abietis_685 | Scymnus_rubromaculatus_825 | 0.168 |
| Scymnus_abietis_682 | Scymnus_rubromaculatus_826 | 0.168 |
| Scymnus_abietis_685 | Scymnus_rubromaculatus_826 | 0.168 |
| Scymnus_abietis_682 | Scymnus_rubromaculatus_827 | 0.168 |
| Scymnus_abietis_685 | Scymnus_rubromaculatus_827 | 0.168 |
| Scymnus_mitior_779 | Scymnus_sinuanodulus_829 | 0.168 |
| Scymnus_apetzi_694 | Scymnus_sinuanodulus_830 | 0.168 |
| Brumus_quadripustulatus_5 | Scymnus_suturalis_838 | 0.168 |
| Brumus_quadripustulatus_5 | Scymnus_suturalis_840 | 0.168 |
| Brumus_quadripustulatus_5 | Scymnus_suturalis_834 | 0.168 |
| Brumus_quadripustulatus_5 | Scymnus_suturalis_837 | 0.168 |
| Hyperaspis_proba_661 | Tytthaspis_sedecimpunctata_577 | 0.168 |
| Hyperaspis_proba_661 | Tytthaspis_sedecimpunctata_578 | 0.168 |
| Hyperaspis_proba_661 | Tytthaspis_sedecimpunctata_579 | 0.168 |
| Hyperaspis_proba_661 | Tytthaspis_sedecimpunctata_581 | 0.168 |
| Hyperaspis_proba_661 | Tytthaspis_sedecimpunctata_582 | 0.168 |
| Hyperaspis_proba_661 | Tytthaspis_sedecimpunctata_585 | 0.168 |
| Hyperaspis_proba_661 | Tytthaspis_sedecimpunctata_590 | 0.168 |
| Hyperaspis_proba_661 | Tytthaspis_sedecimpunctata_592 | 0.168 |
| Hyperaspis_proba_661 | Tytthaspis_sedecimpunctata_594 | 0.168 |
| Hyperaspis_proba_661 | Tytthaspis_sedecimpunctata_595 | 0.168 |
| Chilocorus_stigma_44 | Tytthaspis_sedecimpunctata_577 | 0.168 |
| Chilocorus_stigma_46 | Tytthaspis_sedecimpunctata_577 | 0.168 |
| Chilocorus_stigma_47 | Tytthaspis_sedecimpunctata_577 | 0.168 |
| Chilocorus_stigma_44 | Tytthaspis_sedecimpunctata_578 | 0.168 |
| Chilocorus_stigma_46 | Tytthaspis_sedecimpunctata_578 | 0.168 |
| Chilocorus_stigma_47 | Tytthaspis_sedecimpunctata_578 | 0.168 |
| Chilocorus_stigma_44 | Tytthaspis_sedecimpunctata_579 | 0.168 |
| Chilocorus_stigma_46 | Tytthaspis_sedecimpunctata_579 | 0.168 |
| Chilocorus_stigma_47 | Tytthaspis_sedecimpunctata_579 | 0.168 |
| Chilocorus_stigma_44 | Tytthaspis_sedecimpunctata_581 | 0.168 |
| Chilocorus_stigma_46 | Tytthaspis_sedecimpunctata_581 | 0.168 |
| Chilocorus_stigma_47 | Tytthaspis_sedecimpunctata_581 | 0.168 |
| Chilocorus_stigma_44 | Tytthaspis_sedecimpunctata_582 | 0.168 |
| Chilocorus_stigma_46 | Tytthaspis_sedecimpunctata_582 | 0.168 |
| Chilocorus_stigma_47 | Tytthaspis_sedecimpunctata_582 | 0.168 |
| Chilocorus_stigma_44 | Tytthaspis_sedecimpunctata_585 | 0.168 |
| Chilocorus_stigma_46 | Tytthaspis_sedecimpunctata_585 | 0.168 |
| Chilocorus_stigma_47 | Tytthaspis_sedecimpunctata_585 | 0.168 |
| Chilocorus_stigma_44 | Tytthaspis_sedecimpunctata_590 | 0.168 |
| Chilocorus_stigma_46 | Tytthaspis_sedecimpunctata_590 | 0.168 |
| Chilocorus_stigma_47 | Tytthaspis_sedecimpunctata_590 | 0.168 |

| | | |
|---|---|---|
| Chilocorus_stigma_44 | Tytthaspis_sedecimpunctata_592 | 0.168 |
| Chilocorus_stigma_46 | Tytthaspis_sedecimpunctata_592 | 0.168 |
| Chilocorus_stigma_47 | Tytthaspis_sedecimpunctata_592 | 0.168 |
| Chilocorus_stigma_46 | Tytthaspis_sedecimpunctata_593 | 0.168 |
| Chilocorus_stigma_44 | Tytthaspis_sedecimpunctata_594 | 0.168 |
| Chilocorus_stigma_46 | Tytthaspis_sedecimpunctata_594 | 0.168 |
| Chilocorus_stigma_47 | Tytthaspis_sedecimpunctata_594 | 0.168 |
| Chilocorus_stigma_44 | Tytthaspis_sedecimpunctata_595 | 0.168 |
| Chilocorus_stigma_46 | Tytthaspis_sedecimpunctata_595 | 0.168 |
| Chilocorus_stigma_47 | Tytthaspis_sedecimpunctata_595 | 0.168 |
| Brumus_quadripustulatus_15 | Tytthaspis_sedecimpunctata_577 | 0.168 |
| Brumus_quadripustulatus_16 | Tytthaspis_sedecimpunctata_577 | 0.168 |
| Brumus_quadripustulatus_6 | Tytthaspis_sedecimpunctata_577 | 0.168 |
| Brumus_quadripustulatus_8 | Tytthaspis_sedecimpunctata_577 | 0.168 |
| Brumus_quadripustulatus_15 | Tytthaspis_sedecimpunctata_578 | 0.168 |
| Brumus_quadripustulatus_16 | Tytthaspis_sedecimpunctata_578 | 0.168 |
| Brumus_quadripustulatus_6 | Tytthaspis_sedecimpunctata_578 | 0.168 |
| Brumus_quadripustulatus_8 | Tytthaspis_sedecimpunctata_578 | 0.168 |
| Brumus_quadripustulatus_15 | Tytthaspis_sedecimpunctata_579 | 0.168 |
| Brumus_quadripustulatus_16 | Tytthaspis_sedecimpunctata_579 | 0.168 |
| Brumus_quadripustulatus_6 | Tytthaspis_sedecimpunctata_579 | 0.168 |
| Brumus_quadripustulatus_8 | Tytthaspis_sedecimpunctata_579 | 0.168 |
| Brumus_quadripustulatus_15 | Tytthaspis_sedecimpunctata_581 | 0.168 |
| Brumus_quadripustulatus_16 | Tytthaspis_sedecimpunctata_581 | 0.168 |
| Brumus_quadripustulatus_6 | Tytthaspis_sedecimpunctata_581 | 0.168 |
| Brumus_quadripustulatus_8 | Tytthaspis_sedecimpunctata_581 | 0.168 |
| Brumus_quadripustulatus_15 | Tytthaspis_sedecimpunctata_582 | 0.168 |
| Brumus_quadripustulatus_16 | Tytthaspis_sedecimpunctata_582 | 0.168 |
| Brumus_quadripustulatus_6 | Tytthaspis_sedecimpunctata_582 | 0.168 |
| Brumus_quadripustulatus_8 | Tytthaspis_sedecimpunctata_582 | 0.168 |
| Brumus_quadripustulatus_12 | Tytthaspis_sedecimpunctata_583 | 0.168 |
| Chilocorus_stigma_43 | Tytthaspis_sedecimpunctata_584 | 0.168 |
| Brumus_quadripustulatus_15 | Tytthaspis_sedecimpunctata_590 | 0.168 |
| Brumus_quadripustulatus_16 | Tytthaspis_sedecimpunctata_590 | 0.168 |
| Brumus_quadripustulatus_6 | Tytthaspis_sedecimpunctata_590 | 0.168 |
| Brumus_quadripustulatus_8 | Tytthaspis_sedecimpunctata_590 | 0.168 |
| Chilocorus_bipustulatus_23 | Tytthaspis_sedecimpunctata_591 | 0.168 |
| Brumus_quadripustulatus_15 | Tytthaspis_sedecimpunctata_592 | 0.168 |
| Brumus_quadripustulatus_16 | Tytthaspis_sedecimpunctata_592 | 0.168 |
| Brumus_quadripustulatus_6 | Tytthaspis_sedecimpunctata_592 | 0.168 |
| Brumus_quadripustulatus_8 | Tytthaspis_sedecimpunctata_592 | 0.168 |
| Brumus_quadripustulatus_15 | Tytthaspis_sedecimpunctata_594 | 0.168 |
| Brumus_quadripustulatus_16 | Tytthaspis_sedecimpunctata_594 | 0.168 |
| Brumus_quadripustulatus_6 | Tytthaspis_sedecimpunctata_594 | 0.168 |
| Brumus_quadripustulatus_8 | Tytthaspis_sedecimpunctata_594 | 0.168 |
| Brumus_quadripustulatus_15 | Tytthaspis_sedecimpunctata_595 | 0.168 |
| Brumus_quadripustulatus_16 | Tytthaspis_sedecimpunctata_595 | 0.168 |
| Brumus_quadripustulatus_6 | Tytthaspis_sedecimpunctata_595 | 0.168 |
| Brumus_quadripustulatus_8 | Tytthaspis_sedecimpunctata_595 | 0.168 |
| Scymnus_apetzi_691 | Scymnus_sinuanodulus_829 | 0.168 |
| Anisosticta_novemdecimpunctata_170 | Tytthaspis_sedecimpunctata_591 | 0.168 |
| Psyllobora_vigintimaculata_574 | Vibidia_duodecimguttata_596 | 0.168 |
| Psyllobora_vigintimaculata_575 | Vibidia_duodecimguttata_599 | 0.168 |
| Psyllobora_vigintimaculata_576 | Vibidia_duodecimguttata_599 | 0.168 |
| Psyllobora_vigintimaculata_575 | Vibidia_duodecimguttata_601 | 0.168 |
| Psyllobora_vigintimaculata_576 | Vibidia_duodecimguttata_601 | 0.168 |

| | | |
|---|---|---|
| Psyllobora_vigintimaculata_575 | Vibidia_duodecimguttata_602 | 0.168 |
| Psyllobora_vigintimaculata_576 | Vibidia_duodecimguttata_602 | 0.168 |
| Hyperaspis_reppensis_662 | Scymnus_suturalis_843 | 0.168 |
| Hyperaspis_reppensis_663 | Scymnus_suturalis_843 | 0.168 |
| Hyperaspis_reppensis_662 | Scymnus_suturalis_845 | 0.168 |
| Hyperaspis_reppensis_663 | Scymnus_suturalis_845 | 0.168 |
| Brumus_quadripustulatus_12 | Tytthaspis_sedecimpunctata_580 | 0.168 |
| Propylaea_quatuordecimpunctata_542 | Tytthaspis_sedecimpunctata_584 | 0.168 |
| Propylaea_quatuordecimpunctata_546 | Tytthaspis_sedecimpunctata_584 | 0.168 |
| Brumus_quadripustulatus_11 | Tytthaspis_sedecimpunctata_585 | 0.168 |
| Brumus_quadripustulatus_13 | Tytthaspis_sedecimpunctata_585 | 0.168 |
| Brumus_quadripustulatus_14 | Tytthaspis_sedecimpunctata_585 | 0.168 |
| Brumus_quadripustulatus_17 | Tytthaspis_sedecimpunctata_585 | 0.168 |
| Brumus_quadripustulatus_3 | Tytthaspis_sedecimpunctata_585 | 0.168 |
| Brumus_quadripustulatus_4 | Tytthaspis_sedecimpunctata_585 | 0.168 |
| Brumus_quadripustulatus_7 | Tytthaspis_sedecimpunctata_585 | 0.168 |
| Chilocorus_bipustulatus_19 | Tytthaspis_sedecimpunctata_587 | 0.168 |
| Chilocorus_bipustulatus_22 | Tytthaspis_sedecimpunctata_587 | 0.168 |
| Chilocorus_bipustulatus_24 | Tytthaspis_sedecimpunctata_591 | 0.168 |
| Brumus_quadripustulatus_11 | Tytthaspis_sedecimpunctata_593 | 0.168 |
| Brumus_quadripustulatus_13 | Tytthaspis_sedecimpunctata_593 | 0.168 |
| Brumus_quadripustulatus_14 | Tytthaspis_sedecimpunctata_593 | 0.168 |
| Brumus_quadripustulatus_17 | Tytthaspis_sedecimpunctata_593 | 0.168 |
| Brumus_quadripustulatus_3 | Tytthaspis_sedecimpunctata_593 | 0.168 |
| Brumus_quadripustulatus_4 | Tytthaspis_sedecimpunctata_593 | 0.168 |
| Brumus_quadripustulatus_7 | Tytthaspis_sedecimpunctata_593 | 0.168 |
| Hyperaspis_reppensis_662 | Scymnus_suturalis_837 | 0.168 |
| Hyperaspis_reppensis_663 | Scymnus_suturalis_837 | 0.168 |
| Brumus_quadripustulatus_5 | Scymnus_rubromaculatus_822 | 0.168 |
| Brumus_quadripustulatus_5 | Scymnus_rubromaculatus_823 | 0.168 |
| Brumus_quadripustulatus_5 | Scymnus_rubromaculatus_824 | 0.168 |
| Brumus_quadripustulatus_5 | Scymnus_rubromaculatus_825 | 0.168 |
| Brumus_quadripustulatus_5 | Scymnus_rubromaculatus_826 | 0.168 |
| Brumus_quadripustulatus_5 | Scymnus_rubromaculatus_827 | 0.168 |
| Chilocorus_nigritus_27 | Scymnus_suturalis_841 | 0.168 |
| Chilocorus_nigritus_27 | Scymnus_suturalis_842 | 0.168 |
| Chilocorus_nigritus_27 | Scymnus_suturalis_844 | 0.168 |
| Chilocorus_nigritus_27 | Scymnus_suturalis_846 | 0.168 |
| Chilocorus_nigritus_27 | Scymnus_suturalis_847 | 0.168 |
| Chilocorus_nigritus_27 | Scymnus_suturalis_848 | 0.168 |
| Propylaea_quatuordecimpunctata_542 | Tytthaspis_sedecimpunctata_577 | 0.168 |
| Propylaea_quatuordecimpunctata_546 | Tytthaspis_sedecimpunctata_577 | 0.168 |
| Propylaea_quatuordecimpunctata_542 | Tytthaspis_sedecimpunctata_578 | 0.168 |
| Propylaea_quatuordecimpunctata_546 | Tytthaspis_sedecimpunctata_578 | 0.168 |
| Propylaea_quatuordecimpunctata_542 | Tytthaspis_sedecimpunctata_579 | 0.168 |
| Propylaea_quatuordecimpunctata_546 | Tytthaspis_sedecimpunctata_579 | 0.168 |
| Propylaea_quatuordecimpunctata_542 | Tytthaspis_sedecimpunctata_581 | 0.168 |
| Propylaea_quatuordecimpunctata_546 | Tytthaspis_sedecimpunctata_581 | 0.168 |
| Propylaea_quatuordecimpunctata_542 | Tytthaspis_sedecimpunctata_582 | 0.168 |
| Propylaea_quatuordecimpunctata_546 | Tytthaspis_sedecimpunctata_582 | 0.168 |
| Propylaea_quatuordecimpunctata_532 | Tytthaspis_sedecimpunctata_583 | 0.168 |
| Propylaea_quatuordecimpunctata_533 | Tytthaspis_sedecimpunctata_583 | 0.168 |
| Propylaea_quatuordecimpunctata_534 | Tytthaspis_sedecimpunctata_583 | 0.168 |
| Propylaea_quatuordecimpunctata_535 | Tytthaspis_sedecimpunctata_583 | 0.168 |
| Propylaea_quatuordecimpunctata_536 | Tytthaspis_sedecimpunctata_583 | 0.168 |
| Propylaea_quatuordecimpunctata_537 | Tytthaspis_sedecimpunctata_583 | 0.168 |

| | | |
|---|---|---|
| Propylaea_quatuordecimpunctata_538 | Tytthaspis_sedecimpunctata_583 | 0.168 |
| Propylaea_quatuordecimpunctata_541 | Tytthaspis_sedecimpunctata_583 | 0.168 |
| Propylaea_quatuordecimpunctata_544 | Tytthaspis_sedecimpunctata_583 | 0.168 |
| Propylaea_quatuordecimpunctata_549 | Tytthaspis_sedecimpunctata_583 | 0.168 |
| Coccinella_magnifica_228 | Tytthaspis_sedecimpunctata_585 | 0.168 |
| Coccinella_magnifica_229 | Tytthaspis_sedecimpunctata_585 | 0.168 |
| Coccinella_magnifica_230 | Tytthaspis_sedecimpunctata_585 | 0.168 |
| Coccinella_magnifica_231 | Tytthaspis_sedecimpunctata_585 | 0.168 |
| Propylaea_quatuordecimpunctata_532 | Tytthaspis_sedecimpunctata_585 | 0.168 |
| Propylaea_quatuordecimpunctata_533 | Tytthaspis_sedecimpunctata_585 | 0.168 |
| Propylaea_quatuordecimpunctata_534 | Tytthaspis_sedecimpunctata_585 | 0.168 |
| Propylaea_quatuordecimpunctata_535 | Tytthaspis_sedecimpunctata_585 | 0.168 |
| Propylaea_quatuordecimpunctata_536 | Tytthaspis_sedecimpunctata_585 | 0.168 |
| Propylaea_quatuordecimpunctata_537 | Tytthaspis_sedecimpunctata_585 | 0.168 |
| Propylaea_quatuordecimpunctata_538 | Tytthaspis_sedecimpunctata_585 | 0.168 |
| Propylaea_quatuordecimpunctata_541 | Tytthaspis_sedecimpunctata_585 | 0.168 |
| Propylaea_quatuordecimpunctata_544 | Tytthaspis_sedecimpunctata_585 | 0.168 |
| Propylaea_quatuordecimpunctata_549 | Tytthaspis_sedecimpunctata_585 | 0.168 |
| Adalia_bipunctata_99 | Tytthaspis_sedecimpunctata_586 | 0.168 |
| Adalia_bipunctata_105 | Tytthaspis_sedecimpunctata_588 | 0.168 |
| Coccinella_magnifica_229 | Tytthaspis_sedecimpunctata_589 | 0.168 |
| Coccinella_magnifica_230 | Tytthaspis_sedecimpunctata_589 | 0.168 |
| Coccinella_magnifica_231 | Tytthaspis_sedecimpunctata_589 | 0.168 |
| Propylaea_quatuordecimpunctata_542 | Tytthaspis_sedecimpunctata_590 | 0.168 |
| Propylaea_quatuordecimpunctata_546 | Tytthaspis_sedecimpunctata_590 | 0.168 |
| Chilocorus_stigma_45 | Tytthaspis_sedecimpunctata_591 | 0.168 |
| Propylaea_quatuordecimpunctata_532 | Tytthaspis_sedecimpunctata_592 | 0.168 |
| Propylaea_quatuordecimpunctata_533 | Tytthaspis_sedecimpunctata_592 | 0.168 |
| Propylaea_quatuordecimpunctata_534 | Tytthaspis_sedecimpunctata_592 | 0.168 |
| Propylaea_quatuordecimpunctata_535 | Tytthaspis_sedecimpunctata_592 | 0.168 |
| Propylaea_quatuordecimpunctata_536 | Tytthaspis_sedecimpunctata_592 | 0.168 |
| Propylaea_quatuordecimpunctata_537 | Tytthaspis_sedecimpunctata_592 | 0.168 |
| Propylaea_quatuordecimpunctata_538 | Tytthaspis_sedecimpunctata_592 | 0.168 |
| Propylaea_quatuordecimpunctata_541 | Tytthaspis_sedecimpunctata_592 | 0.168 |
| Propylaea_quatuordecimpunctata_544 | Tytthaspis_sedecimpunctata_592 | 0.168 |
| Propylaea_quatuordecimpunctata_549 | Tytthaspis_sedecimpunctata_592 | 0.168 |
| Propylaea_quatuordecimpunctata_542 | Tytthaspis_sedecimpunctata_593 | 0.168 |
| Propylaea_quatuordecimpunctata_546 | Tytthaspis_sedecimpunctata_593 | 0.168 |
| Propylaea_quatuordecimpunctata_542 | Tytthaspis_sedecimpunctata_594 | 0.168 |
| Propylaea_quatuordecimpunctata_546 | Tytthaspis_sedecimpunctata_594 | 0.168 |
| Propylaea_quatuordecimpunctata_542 | Tytthaspis_sedecimpunctata_595 | 0.168 |
| Propylaea_quatuordecimpunctata_546 | Tytthaspis_sedecimpunctata_595 | 0.168 |
| Chilocorus_nigritus_27 | Scymnus_suturalis_832 | 0.168 |
| Chilocorus_nigritus_27 | Scymnus_suturalis_835 | 0.168 |
| Chilocorus_nigritus_27 | Scymnus_suturalis_836 | 0.168 |
| Chilocorus_nigritus_27 | Scymnus_suturalis_839 | 0.168 |
| Hyperaspis_oregona_658 | Scymnus_suturalis_843 | 0.168 |
| Hyperaspis_oregona_658 | Scymnus_suturalis_845 | 0.168 |
| Coccinella_transversoguttata_265 | Tytthaspis_sedecimpunctata_577 | 0.168 |
| Coccinella_transversoguttata_266 | Tytthaspis_sedecimpunctata_577 | 0.168 |
| Coccinella_trifasciata_280 | Tytthaspis_sedecimpunctata_577 | 0.168 |
| Coccinella_transversoguttata_265 | Tytthaspis_sedecimpunctata_578 | 0.168 |
| Coccinella_transversoguttata_266 | Tytthaspis_sedecimpunctata_578 | 0.168 |
| Coccinella_trifasciata_280 | Tytthaspis_sedecimpunctata_578 | 0.168 |
| Coccinella_transversoguttata_265 | Tytthaspis_sedecimpunctata_579 | 0.168 |
| Coccinella_transversoguttata_266 | Tytthaspis_sedecimpunctata_579 | 0.168 |

| | | |
|---|---|---|
| Coccinella_trifasciata_280 | Tytthaspis_sedecimpunctata_579 | 0.168 |
| Propylaea_quatuordecimpunctata_542 | Tytthaspis_sedecimpunctata_580 | 0.168 |
| Propylaea_quatuordecimpunctata_546 | Tytthaspis_sedecimpunctata_580 | 0.168 |
| Coccinella_transversoguttata_265 | Tytthaspis_sedecimpunctata_581 | 0.168 |
| Coccinella_transversoguttata_266 | Tytthaspis_sedecimpunctata_581 | 0.168 |
| Coccinella_trifasciata_280 | Tytthaspis_sedecimpunctata_581 | 0.168 |
| Coccinella_transversoguttata_265 | Tytthaspis_sedecimpunctata_582 | 0.168 |
| Coccinella_transversoguttata_266 | Tytthaspis_sedecimpunctata_582 | 0.168 |
| Coccinella_trifasciata_280 | Tytthaspis_sedecimpunctata_582 | 0.168 |
| Adalia_bipunctata_99 | Tytthaspis_sedecimpunctata_583 | 0.168 |
| Adalia_bipunctata_105 | Tytthaspis_sedecimpunctata_585 | 0.168 |
| Adalia_bipunctata_102 | Tytthaspis_sedecimpunctata_586 | 0.168 |
| Adalia_bipunctata_103 | Tytthaspis_sedecimpunctata_586 | 0.168 |
| Adalia_bipunctata_106 | Tytthaspis_sedecimpunctata_586 | 0.168 |
| Adalia_bipunctata_107 | Tytthaspis_sedecimpunctata_586 | 0.168 |
| Adalia_bipunctata_108 | Tytthaspis_sedecimpunctata_586 | 0.168 |
| Adalia_bipunctata_109 | Tytthaspis_sedecimpunctata_586 | 0.168 |
| Coccinella_transversoguttata_267 | Tytthaspis_sedecimpunctata_586 | 0.168 |
| Coccinella_transversoguttata_268 | Tytthaspis_sedecimpunctata_586 | 0.168 |
| Coccinella_transversoguttata_269 | Tytthaspis_sedecimpunctata_586 | 0.168 |
| Coccinella_transversoguttata_270 | Tytthaspis_sedecimpunctata_586 | 0.168 |
| Coccinella_transversoguttata_271 | Tytthaspis_sedecimpunctata_586 | 0.168 |
| Adalia_bipunctata_101 | Tytthaspis_sedecimpunctata_588 | 0.168 |
| Adalia_bipunctata_104 | Tytthaspis_sedecimpunctata_588 | 0.168 |
| Coccinella_transversoguttata_267 | Tytthaspis_sedecimpunctata_588 | 0.168 |
| Coccinella_transversoguttata_268 | Tytthaspis_sedecimpunctata_588 | 0.168 |
| Coccinella_transversoguttata_269 | Tytthaspis_sedecimpunctata_588 | 0.168 |
| Coccinella_transversoguttata_270 | Tytthaspis_sedecimpunctata_588 | 0.168 |
| Coccinella_transversoguttata_271 | Tytthaspis_sedecimpunctata_588 | 0.168 |
| Coccinella_transversoguttata_265 | Tytthaspis_sedecimpunctata_590 | 0.168 |
| Coccinella_transversoguttata_266 | Tytthaspis_sedecimpunctata_590 | 0.168 |
| Coccinella_trifasciata_280 | Tytthaspis_sedecimpunctata_590 | 0.168 |
| Adalia_bipunctata_99 | Tytthaspis_sedecimpunctata_592 | 0.168 |
| Coccinella_transversoguttata_265 | Tytthaspis_sedecimpunctata_592 | 0.168 |
| Coccinella_transversoguttata_266 | Tytthaspis_sedecimpunctata_592 | 0.168 |
| Coccinella_trifasciata_280 | Tytthaspis_sedecimpunctata_592 | 0.168 |
| Coccinella_trifasciata_280 | Tytthaspis_sedecimpunctata_593 | 0.168 |
| Coccinella_transversoguttata_265 | Tytthaspis_sedecimpunctata_594 | 0.168 |
| Coccinella_transversoguttata_266 | Tytthaspis_sedecimpunctata_594 | 0.168 |
| Coccinella_trifasciata_280 | Tytthaspis_sedecimpunctata_594 | 0.168 |
| Coccinella_transversoguttata_265 | Tytthaspis_sedecimpunctata_595 | 0.168 |
| Coccinella_transversoguttata_266 | Tytthaspis_sedecimpunctata_595 | 0.168 |
| Coccinella_trifasciata_280 | Tytthaspis_sedecimpunctata_595 | 0.168 |
| Hyperaspis_oregona_658 | Scymnus_suturalis_837 | 0.168 |
| Scymnus_auritus_702 | Scymnus_sinuanodulus_829 | 0.168 |
| Adalia_bipunctata_100 | Tytthaspis_sedecimpunctata_577 | 0.168 |
| Harmonia_axyridis_362 | Tytthaspis_sedecimpunctata_577 | 0.168 |
| Adalia_bipunctata_100 | Tytthaspis_sedecimpunctata_578 | 0.168 |
| Harmonia_axyridis_362 | Tytthaspis_sedecimpunctata_578 | 0.168 |
| Adalia_bipunctata_100 | Tytthaspis_sedecimpunctata_579 | 0.168 |
| Harmonia_axyridis_362 | Tytthaspis_sedecimpunctata_579 | 0.168 |
| Coccinella_trifasciata_272 | Tytthaspis_sedecimpunctata_580 | 0.168 |
| Coccinella_trifasciata_276 | Tytthaspis_sedecimpunctata_580 | 0.168 |
| Adalia_bipunctata_100 | Tytthaspis_sedecimpunctata_581 | 0.168 |
| Harmonia_axyridis_362 | Tytthaspis_sedecimpunctata_581 | 0.168 |
| Adalia_bipunctata_100 | Tytthaspis_sedecimpunctata_582 | 0.168 |

| | | |
|---|---|---|
| Harmonia_axyridis_362 | Tytthaspis_sedecimpunctata_582 | 0.168 |
| Adalia_bipunctata_102 | Tytthaspis_sedecimpunctata_583 | 0.168 |
| Adalia_bipunctata_103 | Tytthaspis_sedecimpunctata_583 | 0.168 |
| Adalia_bipunctata_106 | Tytthaspis_sedecimpunctata_583 | 0.168 |
| Adalia_bipunctata_107 | Tytthaspis_sedecimpunctata_583 | 0.168 |
| Adalia_bipunctata_108 | Tytthaspis_sedecimpunctata_583 | 0.168 |
| Adalia_bipunctata_109 | Tytthaspis_sedecimpunctata_583 | 0.168 |
| Adalia_bipunctata_101 | Tytthaspis_sedecimpunctata_585 | 0.168 |
| Adalia_bipunctata_104 | Tytthaspis_sedecimpunctata_585 | 0.168 |
| Harmonia_axyridis_354 | Tytthaspis_sedecimpunctata_585 | 0.168 |
| Harmonia_axyridis_356 | Tytthaspis_sedecimpunctata_585 | 0.168 |
| Harmonia_axyridis_357 | Tytthaspis_sedecimpunctata_585 | 0.168 |
| Harmonia_axyridis_358 | Tytthaspis_sedecimpunctata_585 | 0.168 |
| Harmonia_axyridis_359 | Tytthaspis_sedecimpunctata_585 | 0.168 |
| Harmonia_axyridis_360 | Tytthaspis_sedecimpunctata_585 | 0.168 |
| Harmonia_axyridis_361 | Tytthaspis_sedecimpunctata_585 | 0.168 |
| Harmonia_axyridis_363 | Tytthaspis_sedecimpunctata_585 | 0.168 |
| Harmonia_axyridis_364 | Tytthaspis_sedecimpunctata_585 | 0.168 |
| Adalia_bipunctata_100 | Tytthaspis_sedecimpunctata_590 | 0.168 |
| Harmonia_axyridis_362 | Tytthaspis_sedecimpunctata_590 | 0.168 |
| Adalia_bipunctata_102 | Tytthaspis_sedecimpunctata_592 | 0.168 |
| Adalia_bipunctata_103 | Tytthaspis_sedecimpunctata_592 | 0.168 |
| Adalia_bipunctata_106 | Tytthaspis_sedecimpunctata_592 | 0.168 |
| Adalia_bipunctata_107 | Tytthaspis_sedecimpunctata_592 | 0.168 |
| Adalia_bipunctata_108 | Tytthaspis_sedecimpunctata_592 | 0.168 |
| Adalia_bipunctata_109 | Tytthaspis_sedecimpunctata_592 | 0.168 |
| Psyllobora_renifer_551 | Tytthaspis_sedecimpunctata_592 | 0.168 |
| Psyllobora_renifer_552 | Tytthaspis_sedecimpunctata_592 | 0.168 |
| Psyllobora_renifer_553 | Tytthaspis_sedecimpunctata_592 | 0.168 |
| Psyllobora_renifer_554 | Tytthaspis_sedecimpunctata_592 | 0.168 |
| Harmonia_axyridis_362 | Tytthaspis_sedecimpunctata_593 | 0.168 |
| Adalia_bipunctata_100 | Tytthaspis_sedecimpunctata_594 | 0.168 |
| Harmonia_axyridis_362 | Tytthaspis_sedecimpunctata_594 | 0.168 |
| Adalia_bipunctata_100 | Tytthaspis_sedecimpunctata_595 | 0.168 |
| Harmonia_axyridis_362 | Tytthaspis_sedecimpunctata_595 | 0.168 |
| Coccinella_trifasciata_273 | Tytthaspis_sedecimpunctata_577 | 0.168 |
| Psyllobora_renifer_555 | Tytthaspis_sedecimpunctata_577 | 0.168 |
| Coccinella_trifasciata_273 | Tytthaspis_sedecimpunctata_578 | 0.168 |
| Psyllobora_renifer_555 | Tytthaspis_sedecimpunctata_578 | 0.168 |
| Coccinella_trifasciata_273 | Tytthaspis_sedecimpunctata_579 | 0.168 |
| Psyllobora_renifer_555 | Tytthaspis_sedecimpunctata_579 | 0.168 |
| Adalia_bipunctata_102 | Tytthaspis_sedecimpunctata_580 | 0.168 |
| Adalia_bipunctata_103 | Tytthaspis_sedecimpunctata_580 | 0.168 |
| Adalia_bipunctata_106 | Tytthaspis_sedecimpunctata_580 | 0.168 |
| Adalia_bipunctata_107 | Tytthaspis_sedecimpunctata_580 | 0.168 |
| Adalia_bipunctata_108 | Tytthaspis_sedecimpunctata_580 | 0.168 |
| Adalia_bipunctata_109 | Tytthaspis_sedecimpunctata_580 | 0.168 |
| Harmonia_axyridis_362 | Tytthaspis_sedecimpunctata_580 | 0.168 |
| Psyllobora_renifer_551 | Tytthaspis_sedecimpunctata_580 | 0.168 |
| Psyllobora_renifer_552 | Tytthaspis_sedecimpunctata_580 | 0.168 |
| Psyllobora_renifer_553 | Tytthaspis_sedecimpunctata_580 | 0.168 |
| Psyllobora_renifer_554 | Tytthaspis_sedecimpunctata_580 | 0.168 |
| Coccinella_trifasciata_273 | Tytthaspis_sedecimpunctata_581 | 0.168 |
| Psyllobora_renifer_555 | Tytthaspis_sedecimpunctata_581 | 0.168 |
| Coccinella_trifasciata_273 | Tytthaspis_sedecimpunctata_582 | 0.168 |
| Psyllobora_renifer_555 | Tytthaspis_sedecimpunctata_582 | 0.168 |

| | | |
|---|---|---|
| Hippodamia_alpina_385 | Tytthaspis_sedecimpunctata_583 | 0.168 |
| Hippodamia_alpina_386 | Tytthaspis_sedecimpunctata_583 | 0.168 |
| Hippodamia_alpina_387 | Tytthaspis_sedecimpunctata_583 | 0.168 |
| Anatis_ocellata_133 | Tytthaspis_sedecimpunctata_584 | 0.168 |
| Anatis_ocellata_137 | Tytthaspis_sedecimpunctata_584 | 0.168 |
| Harmonia_axyridis_354 | Tytthaspis_sedecimpunctata_584 | 0.168 |
| Harmonia_axyridis_356 | Tytthaspis_sedecimpunctata_584 | 0.168 |
| Harmonia_axyridis_357 | Tytthaspis_sedecimpunctata_584 | 0.168 |
| Harmonia_axyridis_358 | Tytthaspis_sedecimpunctata_584 | 0.168 |
| Harmonia_axyridis_359 | Tytthaspis_sedecimpunctata_584 | 0.168 |
| Harmonia_axyridis_360 | Tytthaspis_sedecimpunctata_584 | 0.168 |
| Harmonia_axyridis_361 | Tytthaspis_sedecimpunctata_584 | 0.168 |
| Harmonia_axyridis_363 | Tytthaspis_sedecimpunctata_584 | 0.168 |
| Harmonia_axyridis_364 | Tytthaspis_sedecimpunctata_584 | 0.168 |
| Psyllobora_renifer_555 | Tytthaspis_sedecimpunctata_585 | 0.168 |
| Anatis_mali_128 | Tytthaspis_sedecimpunctata_586 | 0.168 |
| Anatis_mali_129 | Tytthaspis_sedecimpunctata_586 | 0.168 |
| Anatis_mali_130 | Tytthaspis_sedecimpunctata_586 | 0.168 |
| Anatis_mali_131 | Tytthaspis_sedecimpunctata_586 | 0.168 |
| Anatis_ocellata_133 | Tytthaspis_sedecimpunctata_586 | 0.168 |
| Anatis_ocellata_137 | Tytthaspis_sedecimpunctata_586 | 0.168 |
| Coccinella_quinquepunctata_232 | Tytthaspis_sedecimpunctata_586 | 0.168 |
| Scymnus_auritus_698 | Tytthaspis_sedecimpunctata_586 | 0.168 |
| Scymnus_auritus_698 | Tytthaspis_sedecimpunctata_588 | 0.168 |
| Psyllobora_vigintimaculata_573 | Tytthaspis_sedecimpunctata_589 | 0.168 |
| Coccinella_trifasciata_273 | Tytthaspis_sedecimpunctata_590 | 0.168 |
| Psyllobora_renifer_555 | Tytthaspis_sedecimpunctata_590 | 0.168 |
| Anatis_mali_128 | Tytthaspis_sedecimpunctata_591 | 0.168 |
| Anatis_mali_129 | Tytthaspis_sedecimpunctata_591 | 0.168 |
| Anatis_mali_130 | Tytthaspis_sedecimpunctata_591 | 0.168 |
| Anatis_mali_131 | Tytthaspis_sedecimpunctata_591 | 0.168 |
| Anatis_ocellata_133 | Tytthaspis_sedecimpunctata_591 | 0.168 |
| Anatis_ocellata_137 | Tytthaspis_sedecimpunctata_591 | 0.168 |
| Coccinella_trifasciata_273 | Tytthaspis_sedecimpunctata_592 | 0.168 |
| Coccinella_trifasciata_273 | Tytthaspis_sedecimpunctata_593 | 0.168 |
| Coccinella_trifasciata_273 | Tytthaspis_sedecimpunctata_594 | 0.168 |
| Psyllobora_renifer_555 | Tytthaspis_sedecimpunctata_594 | 0.168 |
| Coccinella_trifasciata_273 | Tytthaspis_sedecimpunctata_595 | 0.168 |
| Psyllobora_renifer_555 | Tytthaspis_sedecimpunctata_595 | 0.168 |
| Hyperaspis_reppensis_667 | Scymnus_suturalis_833 | 0.168 |
| Hyperaspis_reppensis_667 | Scymnus_suturalis_838 | 0.168 |
| Hyperaspis_reppensis_667 | Scymnus_suturalis_840 | 0.168 |
| Hyperaspis_reppensis_664 | Scymnus_suturalis_843 | 0.168 |
| Hyperaspis_reppensis_664 | Scymnus_suturalis_845 | 0.168 |
| Anatis_ocellata_143 | Tytthaspis_sedecimpunctata_583 | 0.168 |
| Anatis_ocellata_143 | Tytthaspis_sedecimpunctata_585 | 0.168 |
| Hippodamia_alpina_385 | Tytthaspis_sedecimpunctata_586 | 0.168 |
| Hippodamia_alpina_386 | Tytthaspis_sedecimpunctata_586 | 0.168 |
| Hippodamia_alpina_387 | Tytthaspis_sedecimpunctata_586 | 0.168 |
| Mulsantina_picta_484 | Tytthaspis_sedecimpunctata_586 | 0.168 |
| Psyllobora_vigintimaculata_573 | Tytthaspis_sedecimpunctata_586 | 0.168 |
| Mulsantina_picta_484 | Tytthaspis_sedecimpunctata_587 | 0.168 |
| Hippodamia_alpina_385 | Tytthaspis_sedecimpunctata_588 | 0.168 |
| Hippodamia_alpina_386 | Tytthaspis_sedecimpunctata_588 | 0.168 |
| Hippodamia_alpina_387 | Tytthaspis_sedecimpunctata_588 | 0.168 |
| Mulsantina_picta_484 | Tytthaspis_sedecimpunctata_588 | 0.168 |

| | | |
|---|---|---|
| Psyllobora_vigintimaculata_574 | Tytthaspis_sedecimpunctata_589 | 0.168 |
| Scymnus_auritus_699 | Tytthaspis_sedecimpunctata_589 | 0.168 |
| Anatis_ocellata_143 | Tytthaspis_sedecimpunctata_592 | 0.168 |
| Hyperaspis_reppensis_667 | Scymnus_suturalis_834 | 0.168 |
| Hyperaspis_reppensis_664 | Scymnus_suturalis_837 | 0.168 |
| Scymnus_apetzi_691 | Scymnus_suturalis_838 | 0.168 |
| Scymnus_apetzi_696 | Scymnus_suturalis_838 | 0.168 |
| Scymnus_apetzi_691 | Scymnus_suturalis_840 | 0.168 |
| Scymnus_apetzi_696 | Scymnus_suturalis_840 | 0.168 |
| Hyperaspis_proba_660 | Tytthaspis_sedecimpunctata_577 | 0.168 |
| Hyperaspis_proba_660 | Tytthaspis_sedecimpunctata_578 | 0.168 |
| Hyperaspis_proba_660 | Tytthaspis_sedecimpunctata_579 | 0.168 |
| Hyperaspis_proba_660 | Tytthaspis_sedecimpunctata_581 | 0.168 |
| Hyperaspis_proba_660 | Tytthaspis_sedecimpunctata_582 | 0.168 |
| Hyperaspis_proba_660 | Tytthaspis_sedecimpunctata_585 | 0.168 |
| Hyperaspis_proba_660 | Tytthaspis_sedecimpunctata_590 | 0.168 |
| Hyperaspis_proba_660 | Tytthaspis_sedecimpunctata_592 | 0.168 |
| Hyperaspis_proba_660 | Tytthaspis_sedecimpunctata_594 | 0.168 |
| Hyperaspis_proba_660 | Tytthaspis_sedecimpunctata_595 | 0.168 |
| Scymnus_apetzi_690 | Scymnus_suturalis_841 | 0.168 |
| Scymnus_apetzi_697 | Scymnus_suturalis_841 | 0.168 |
| Scymnus_apetzi_690 | Scymnus_suturalis_842 | 0.168 |
| Scymnus_apetzi_697 | Scymnus_suturalis_842 | 0.168 |
| Scymnus_apetzi_690 | Scymnus_suturalis_844 | 0.168 |
| Scymnus_apetzi_697 | Scymnus_suturalis_844 | 0.168 |
| Scymnus_apetzi_690 | Scymnus_suturalis_846 | 0.168 |
| Scymnus_apetzi_697 | Scymnus_suturalis_846 | 0.168 |
| Scymnus_apetzi_690 | Scymnus_suturalis_847 | 0.168 |
| Scymnus_apetzi_697 | Scymnus_suturalis_847 | 0.168 |
| Scymnus_apetzi_690 | Scymnus_suturalis_848 | 0.168 |
| Scymnus_apetzi_697 | Scymnus_suturalis_848 | 0.168 |
| Hippodamia_alpina_384 | Tytthaspis_sedecimpunctata_577 | 0.168 |
| Hippodamia_alpina_384 | Tytthaspis_sedecimpunctata_578 | 0.168 |
| Hippodamia_alpina_384 | Tytthaspis_sedecimpunctata_579 | 0.168 |
| Anatis_ocellata_143 | Tytthaspis_sedecimpunctata_580 | 0.168 |
| Hippodamia_alpina_383 | Tytthaspis_sedecimpunctata_580 | 0.168 |
| Hippodamia_alpina_384 | Tytthaspis_sedecimpunctata_581 | 0.168 |
| Hippodamia_alpina_384 | Tytthaspis_sedecimpunctata_582 | 0.168 |
| Mulsantina_picta_484 | Tytthaspis_sedecimpunctata_583 | 0.168 |
| Psyllobora_vigintiduopunctata_571 | Tytthaspis_sedecimpunctata_583 | 0.168 |
| Hippodamia_alpina_384 | Tytthaspis_sedecimpunctata_585 | 0.168 |
| Psyllobora_vigintimaculata_574 | Tytthaspis_sedecimpunctata_586 | 0.168 |
| Psyllobora_vigintimaculata_575 | Tytthaspis_sedecimpunctata_587 | 0.168 |
| Psyllobora_vigintimaculata_576 | Tytthaspis_sedecimpunctata_587 | 0.168 |
| Hippodamia_alpina_384 | Tytthaspis_sedecimpunctata_589 | 0.168 |
| Hippodamia_alpina_384 | Tytthaspis_sedecimpunctata_590 | 0.168 |
| Hippodamia_alpina_384 | Tytthaspis_sedecimpunctata_592 | 0.168 |
| Psyllobora_vigintiduopunctata_571 | Tytthaspis_sedecimpunctata_592 | 0.168 |
| Hippodamia_alpina_384 | Tytthaspis_sedecimpunctata_593 | 0.168 |
| Hippodamia_alpina_384 | Tytthaspis_sedecimpunctata_594 | 0.168 |
| Hippodamia_alpina_384 | Tytthaspis_sedecimpunctata_595 | 0.168 |
| Scymnus_apetzi_690 | Scymnus_suturalis_832 | 0.168 |
| Scymnus_apetzi_697 | Scymnus_suturalis_832 | 0.168 |
| Scymnus_apetzi_691 | Scymnus_suturalis_834 | 0.168 |
| Scymnus_apetzi_696 | Scymnus_suturalis_834 | 0.168 |
| Scymnus_apetzi_690 | Scymnus_suturalis_835 | 0.168 |

| | | |
|---|---|---|
| Scymnus_apetzi_697 | Scymnus_suturalis_835 | 0.168 |
| Scymnus_apetzi_690 | Scymnus_suturalis_836 | 0.168 |
| Scymnus_apetzi_697 | Scymnus_suturalis_836 | 0.168 |
| Scymnus_apetzi_690 | Scymnus_suturalis_839 | 0.168 |
| Scymnus_apetzi_697 | Scymnus_suturalis_839 | 0.168 |
| Chilocorus_bipustulatus_21 | Tytthaspis_sedecimpunctata_587 | 0.168 |
| Chilocorus_nigritus_28 | Scymnus_suturalis_841 | 0.168 |
| Chilocorus_nigritus_28 | Scymnus_suturalis_842 | 0.168 |
| Chilocorus_nigritus_28 | Scymnus_suturalis_844 | 0.168 |
| Chilocorus_nigritus_28 | Scymnus_suturalis_846 | 0.168 |
| Chilocorus_nigritus_28 | Scymnus_suturalis_847 | 0.168 |
| Chilocorus_nigritus_28 | Scymnus_suturalis_848 | 0.168 |
| Chilocorus_nigritus_28 | Scymnus_suturalis_832 | 0.168 |
| Chilocorus_nigritus_28 | Scymnus_suturalis_835 | 0.168 |
| Chilocorus_nigritus_28 | Scymnus_suturalis_836 | 0.168 |
| Chilocorus_nigritus_28 | Scymnus_suturalis_839 | 0.168 |
| Mulsantina_picta_484 | Tytthaspis_sedecimpunctata_580 | 0.168 |
| Scymnus_haemorrhoidalis_750 | Tytthaspis_sedecimpunctata_584 | 0.168 |
| Scymnus_haemorrhoidalis_751 | Tytthaspis_sedecimpunctata_584 | 0.168 |
| Scymnus_haemorrhoidalis_754 | Tytthaspis_sedecimpunctata_584 | 0.168 |
| Scymnus_haemorrhoidalis_757 | Tytthaspis_sedecimpunctata_584 | 0.168 |
| Scymnus_haemorrhoidalis_758 | Tytthaspis_sedecimpunctata_584 | 0.168 |
| Hippodamia_alpina_384 | Tytthaspis_sedecimpunctata_587 | 0.168 |
| Scymnus_haemorrhoidalis_749 | Tytthaspis_sedecimpunctata_588 | 0.168 |
| Scymnus_haemorrhoidalis_752 | Tytthaspis_sedecimpunctata_589 | 0.168 |
| Scymnus_haemorrhoidalis_750 | Tytthaspis_sedecimpunctata_591 | 0.168 |
| Scymnus_haemorrhoidalis_751 | Tytthaspis_sedecimpunctata_591 | 0.168 |
| Scymnus_haemorrhoidalis_754 | Tytthaspis_sedecimpunctata_591 | 0.168 |
| Scymnus_haemorrhoidalis_757 | Tytthaspis_sedecimpunctata_591 | 0.168 |
| Scymnus_haemorrhoidalis_758 | Tytthaspis_sedecimpunctata_591 | 0.168 |
| Scymnus_mimulus_778 | Scymnus_suturalis_838 | 0.168 |
| Scymnus_mimulus_778 | Scymnus_suturalis_840 | 0.168 |
| Psyllobora_vigintiduopunctata_570 | Tytthaspis_sedecimpunctata_577 | 0.168 |
| Psyllobora_vigintiduopunctata_572 | Tytthaspis_sedecimpunctata_577 | 0.168 |
| Psyllobora_vigintiduopunctata_570 | Tytthaspis_sedecimpunctata_578 | 0.168 |
| Psyllobora_vigintiduopunctata_572 | Tytthaspis_sedecimpunctata_578 | 0.168 |
| Psyllobora_vigintiduopunctata_570 | Tytthaspis_sedecimpunctata_579 | 0.168 |
| Psyllobora_vigintiduopunctata_572 | Tytthaspis_sedecimpunctata_579 | 0.168 |
| Psyllobora_vigintiduopunctata_570 | Tytthaspis_sedecimpunctata_581 | 0.168 |
| Psyllobora_vigintiduopunctata_572 | Tytthaspis_sedecimpunctata_581 | 0.168 |
| Psyllobora_vigintiduopunctata_570 | Tytthaspis_sedecimpunctata_582 | 0.168 |
| Psyllobora_vigintiduopunctata_572 | Tytthaspis_sedecimpunctata_582 | 0.168 |
| Psyllobora_vigintiduopunctata_560 | Tytthaspis_sedecimpunctata_583 | 0.168 |
| Scymnus_haemorrhoidalis_755 | Tytthaspis_sedecimpunctata_584 | 0.168 |
| Psyllobora_vigintiduopunctata_560 | Tytthaspis_sedecimpunctata_585 | 0.168 |
| Scymnus_haemorrhoidalis_750 | Tytthaspis_sedecimpunctata_585 | 0.168 |
| Scymnus_haemorrhoidalis_751 | Tytthaspis_sedecimpunctata_585 | 0.168 |
| Scymnus_haemorrhoidalis_754 | Tytthaspis_sedecimpunctata_585 | 0.168 |
| Scymnus_haemorrhoidalis_757 | Tytthaspis_sedecimpunctata_585 | 0.168 |
| Scymnus_haemorrhoidalis_758 | Tytthaspis_sedecimpunctata_585 | 0.168 |
| Hippodamia_glacialis_407 | Tytthaspis_sedecimpunctata_586 | 0.168 |
| Hippodamia_glacialis_408 | Tytthaspis_sedecimpunctata_587 | 0.168 |
| Hippodamia_glacialis_409 | Tytthaspis_sedecimpunctata_587 | 0.168 |
| Scymnus_haemorrhoidalis_752 | Tytthaspis_sedecimpunctata_587 | 0.168 |
| Psyllobora_vigintiduopunctata_570 | Tytthaspis_sedecimpunctata_590 | 0.168 |
| Psyllobora_vigintiduopunctata_572 | Tytthaspis_sedecimpunctata_590 | 0.168 |

| | | |
|---|---|---|
| Scymnus_haemorrhoidalis_755 | Tytthaspis_sedecimpunctata_591 | 0.168 |
| Psyllobora_vigintiduopunctata_560 | Tytthaspis_sedecimpunctata_592 | 0.168 |
| Psyllobora_vigintiduopunctata_570 | Tytthaspis_sedecimpunctata_594 | 0.168 |
| Psyllobora_vigintiduopunctata_572 | Tytthaspis_sedecimpunctata_594 | 0.168 |
| Psyllobora_vigintiduopunctata_570 | Tytthaspis_sedecimpunctata_595 | 0.168 |
| Psyllobora_vigintiduopunctata_572 | Tytthaspis_sedecimpunctata_595 | 0.168 |
| Hyperaspis_oregona_658 | Scymnus_rubromaculatus_822 | 0.168 |
| Hyperaspis_oregona_658 | Scymnus_rubromaculatus_823 | 0.168 |
| Hyperaspis_oregona_658 | Scymnus_rubromaculatus_824 | 0.168 |
| Hyperaspis_oregona_658 | Scymnus_rubromaculatus_825 | 0.168 |
| Hyperaspis_oregona_658 | Scymnus_rubromaculatus_826 | 0.168 |
| Hyperaspis_oregona_658 | Scymnus_rubromaculatus_827 | 0.168 |
| Hyperaspis_proba_661 | Scymnus_suturalis_833 | 0.168 |
| Scymnus_mimulus_778 | Scymnus_suturalis_834 | 0.168 |
| Hyperaspis_reppensis_664 | Subcoccinella_vigintiquatuorpunctata_630 | 0.168 |
| Hyperaspis_reppensis_666 | Subcoccinella_vigintiquatuorpunctata_630 | 0.168 |
| Hyperaspis_reppensis_667 | Subcoccinella_vigintiquatuorpunctata_630 | 0.168 |
| Hippodamia_glacialis_408 | Tytthaspis_sedecimpunctata_577 | 0.168 |
| Hippodamia_glacialis_409 | Tytthaspis_sedecimpunctata_577 | 0.168 |
| Hippodamia_glacialis_408 | Tytthaspis_sedecimpunctata_578 | 0.168 |
| Hippodamia_glacialis_409 | Tytthaspis_sedecimpunctata_578 | 0.168 |
| Hippodamia_glacialis_408 | Tytthaspis_sedecimpunctata_579 | 0.168 |
| Hippodamia_glacialis_409 | Tytthaspis_sedecimpunctata_579 | 0.168 |
| Hippodamia_notata_415 | Tytthaspis_sedecimpunctata_580 | 0.168 |
| Psyllobora_vigintiduopunctata_560 | Tytthaspis_sedecimpunctata_580 | 0.168 |
| Hippodamia_glacialis_408 | Tytthaspis_sedecimpunctata_581 | 0.168 |
| Hippodamia_glacialis_409 | Tytthaspis_sedecimpunctata_581 | 0.168 |
| Hippodamia_glacialis_408 | Tytthaspis_sedecimpunctata_582 | 0.168 |
| Hippodamia_glacialis_409 | Tytthaspis_sedecimpunctata_582 | 0.168 |
| Hippodamia_arctica_389 | Tytthaspis_sedecimpunctata_583 | 0.168 |
| Hippodamia_notata_419 | Tytthaspis_sedecimpunctata_583 | 0.168 |
| Hippodamia_notata_429 | Tytthaspis_sedecimpunctata_583 | 0.168 |
| Hippodamia_notata_431 | Tytthaspis_sedecimpunctata_583 | 0.168 |
| Coelophora_inaequalis_320 | Tytthaspis_sedecimpunctata_584 | 0.168 |
| Coelophora_inaequalis_321 | Tytthaspis_sedecimpunctata_584 | 0.168 |
| Coelophora_inaequalis_322 | Tytthaspis_sedecimpunctata_584 | 0.168 |
| Psyllobora_vigintiduopunctata_557 | Tytthaspis_sedecimpunctata_584 | 0.168 |
| Psyllobora_vigintiduopunctata_561 | Tytthaspis_sedecimpunctata_584 | 0.168 |
| Psyllobora_vigintiduopunctata_562 | Tytthaspis_sedecimpunctata_584 | 0.168 |
| Psyllobora_vigintiduopunctata_563 | Tytthaspis_sedecimpunctata_584 | 0.168 |
| Psyllobora_vigintiduopunctata_565 | Tytthaspis_sedecimpunctata_584 | 0.168 |
| Psyllobora_vigintiduopunctata_567 | Tytthaspis_sedecimpunctata_584 | 0.168 |
| Hippodamia_arctica_389 | Tytthaspis_sedecimpunctata_585 | 0.168 |
| Scymnus_haemorrhoidalis_755 | Tytthaspis_sedecimpunctata_585 | 0.168 |
| Hippodamia_tredecimpunctata_457 | Tytthaspis_sedecimpunctata_586 | 0.168 |
| Hippodamia_tredecimpunctata_458 | Tytthaspis_sedecimpunctata_586 | 0.168 |
| Hippodamia_tredecimpunctata_459 | Tytthaspis_sedecimpunctata_588 | 0.168 |
| Halyzia_sedecimguttata_349 | Tytthaspis_sedecimpunctata_589 | 0.168 |
| Halyzia_sedecimguttata_352 | Tytthaspis_sedecimpunctata_589 | 0.168 |
| Hippodamia_glacialis_408 | Tytthaspis_sedecimpunctata_590 | 0.168 |
| Hippodamia_glacialis_409 | Tytthaspis_sedecimpunctata_590 | 0.168 |
| Hippodamia_glacialis_408 | Tytthaspis_sedecimpunctata_593 | 0.168 |
| Hippodamia_glacialis_409 | Tytthaspis_sedecimpunctata_593 | 0.168 |
| Hippodamia_notata_421 | Tytthaspis_sedecimpunctata_593 | 0.168 |
| Hippodamia_glacialis_408 | Tytthaspis_sedecimpunctata_594 | 0.168 |
| Hippodamia_glacialis_409 | Tytthaspis_sedecimpunctata_594 | 0.168 |

| | | |
|---|---|---|
| Hippodamia_glacialis_408 | Tytthaspis_sedecimpunctata_595 | 0.168 |
| Hippodamia_glacialis_409 | Tytthaspis_sedecimpunctata_595 | 0.168 |
| Anatis_ocellata_136 | Tytthaspis_sedecimpunctata_587 | 0.168 |
| Scymnus_apetzi_693 | Scymnus_sinuanodulus_830 | 0.168 |
| Hippodamia_glacialis_407 | Tytthaspis_sedecimpunctata_580 | 0.169 |
| Hippodamia_notata_413 | Tytthaspis_sedecimpunctata_580 | 0.169 |
| Hippodamia_notata_418 | Tytthaspis_sedecimpunctata_580 | 0.169 |
| Ceratomegilla_ulkei_219 | Tytthaspis_sedecimpunctata_583 | 0.169 |
| Hippodamia_arctica_389 | Tytthaspis_sedecimpunctata_584 | 0.169 |
| Hippodamia_notata_419 | Tytthaspis_sedecimpunctata_584 | 0.169 |
| Hippodamia_notata_429 | Tytthaspis_sedecimpunctata_584 | 0.169 |
| Hippodamia_notata_431 | Tytthaspis_sedecimpunctata_584 | 0.169 |
| Psyllobora_vigintiduopunctata_556 | Tytthaspis_sedecimpunctata_584 | 0.169 |
| Ceratomegilla_ulkei_220 | Tytthaspis_sedecimpunctata_585 | 0.169 |
| Ceratomegilla_ulkei_222 | Tytthaspis_sedecimpunctata_586 | 0.169 |
| Ceratomegilla_ulkei_223 | Tytthaspis_sedecimpunctata_586 | 0.169 |
| Ceratomegilla_ulkei_222 | Tytthaspis_sedecimpunctata_587 | 0.169 |
| Ceratomegilla_ulkei_223 | Tytthaspis_sedecimpunctata_587 | 0.169 |
| Hippodamia_convergens_396 | Tytthaspis_sedecimpunctata_588 | 0.169 |
| Hippodamia_convergens_400 | Tytthaspis_sedecimpunctata_588 | 0.169 |
| Hippodamia_convergens_403 | Tytthaspis_sedecimpunctata_588 | 0.169 |
| Hippodamia_moesta_410 | Tytthaspis_sedecimpunctata_588 | 0.169 |
| Halyzia_sedecimguttata_346 | Tytthaspis_sedecimpunctata_589 | 0.169 |
| Halyzia_sedecimguttata_347 | Tytthaspis_sedecimpunctata_589 | 0.169 |
| Halyzia_sedecimguttata_348 | Tytthaspis_sedecimpunctata_589 | 0.169 |
| Hippodamia_notata_421 | Tytthaspis_sedecimpunctata_591 | 0.169 |
| Ceratomegilla_ulkei_220 | Tytthaspis_sedecimpunctata_593 | 0.169 |
| Psyllobora_vigintiduopunctata_566 | Tytthaspis_sedecimpunctata_589 | 0.169 |
| Psyllobora_vigintiduopunctata_566 | Tytthaspis_sedecimpunctata_587 | 0.169 |
| Hippodamia_caseyi_392 | Tytthaspis_sedecimpunctata_577 | 0.169 |
| Hippodamia_convergens_395 | Tytthaspis_sedecimpunctata_577 | 0.169 |
| Hippodamia_caseyi_392 | Tytthaspis_sedecimpunctata_578 | 0.169 |
| Hippodamia_convergens_395 | Tytthaspis_sedecimpunctata_578 | 0.169 |
| Hippodamia_caseyi_392 | Tytthaspis_sedecimpunctata_579 | 0.169 |
| Hippodamia_convergens_395 | Tytthaspis_sedecimpunctata_579 | 0.169 |
| Ceratomegilla_ulkei_219 | Tytthaspis_sedecimpunctata_580 | 0.169 |
| Hippodamia_caseyi_392 | Tytthaspis_sedecimpunctata_581 | 0.169 |
| Hippodamia_convergens_395 | Tytthaspis_sedecimpunctata_581 | 0.169 |
| Hippodamia_caseyi_392 | Tytthaspis_sedecimpunctata_582 | 0.169 |
| Hippodamia_convergens_395 | Tytthaspis_sedecimpunctata_582 | 0.169 |
| Hippodamia_caseyi_391 | Tytthaspis_sedecimpunctata_583 | 0.169 |
| Hippodamia_convergens_399 | Tytthaspis_sedecimpunctata_583 | 0.169 |
| Hippodamia_convergens_404 | Tytthaspis_sedecimpunctata_583 | 0.169 |
| Hippodamia_convergens_405 | Tytthaspis_sedecimpunctata_583 | 0.169 |
| Hippodamia_convergens_406 | Tytthaspis_sedecimpunctata_583 | 0.169 |
| Hippodamia_moesta_411 | Tytthaspis_sedecimpunctata_583 | 0.169 |
| Hippodamia_quinquesignata_443 | Tytthaspis_sedecimpunctata_583 | 0.169 |
| Hippodamia_quinquesignata_444 | Tytthaspis_sedecimpunctata_583 | 0.169 |
| Hippodamia_quinquesignata_445 | Tytthaspis_sedecimpunctata_583 | 0.169 |
| Ceratomegilla_ulkei_221 | Tytthaspis_sedecimpunctata_584 | 0.169 |
| Hippodamia_caseyi_391 | Tytthaspis_sedecimpunctata_585 | 0.169 |
| Hippodamia_convergens_393 | Tytthaspis_sedecimpunctata_585 | 0.169 |
| Hippodamia_convergens_394 | Tytthaspis_sedecimpunctata_585 | 0.169 |
| Hippodamia_convergens_397 | Tytthaspis_sedecimpunctata_585 | 0.169 |
| Hippodamia_convergens_398 | Tytthaspis_sedecimpunctata_585 | 0.169 |
| Hippodamia_convergens_399 | Tytthaspis_sedecimpunctata_585 | 0.169 |

| | | |
|---|---|---|
| Hippodamia_convergens_401 | Tytthaspis_sedecimpunctata_585 | 0.169 |
| Hippodamia_convergens_402 | Tytthaspis_sedecimpunctata_585 | 0.169 |
| Hippodamia_convergens_404 | Tytthaspis_sedecimpunctata_585 | 0.169 |
| Hippodamia_convergens_405 | Tytthaspis_sedecimpunctata_585 | 0.169 |
| Hippodamia_convergens_406 | Tytthaspis_sedecimpunctata_585 | 0.169 |
| Hippodamia_moesta_411 | Tytthaspis_sedecimpunctata_585 | 0.169 |
| Hippodamia_quinquesignata_443 | Tytthaspis_sedecimpunctata_585 | 0.169 |
| Hippodamia_quinquesignata_444 | Tytthaspis_sedecimpunctata_585 | 0.169 |
| Hippodamia_quinquesignata_445 | Tytthaspis_sedecimpunctata_585 | 0.169 |
| Coccinula_quatuordecimpustulata_297 | Tytthaspis_sedecimpunctata_586 | 0.169 |
| Hippodamia_tredecimpunctata_446 | Tytthaspis_sedecimpunctata_586 | 0.169 |
| Hippodamia_tredecimpunctata_448 | Tytthaspis_sedecimpunctata_586 | 0.169 |
| Hippodamia_tredecimpunctata_449 | Tytthaspis_sedecimpunctata_586 | 0.169 |
| Hippodamia_tredecimpunctata_450 | Tytthaspis_sedecimpunctata_586 | 0.169 |
| Hippodamia_tredecimpunctata_452 | Tytthaspis_sedecimpunctata_586 | 0.169 |
| Hippodamia_tredecimpunctata_453 | Tytthaspis_sedecimpunctata_586 | 0.169 |
| Oenopia_conglobata_523 | Tytthaspis_sedecimpunctata_586 | 0.169 |
| Hippodamia_tredecimpunctata_446 | Tytthaspis_sedecimpunctata_587 | 0.169 |
| Hippodamia_tredecimpunctata_448 | Tytthaspis_sedecimpunctata_587 | 0.169 |
| Hippodamia_tredecimpunctata_449 | Tytthaspis_sedecimpunctata_587 | 0.169 |
| Hippodamia_tredecimpunctata_450 | Tytthaspis_sedecimpunctata_587 | 0.169 |
| Hippodamia_tredecimpunctata_452 | Tytthaspis_sedecimpunctata_587 | 0.169 |
| Hippodamia_tredecimpunctata_453 | Tytthaspis_sedecimpunctata_587 | 0.169 |
| Hippodamia_caseyi_392 | Tytthaspis_sedecimpunctata_590 | 0.169 |
| Hippodamia_convergens_395 | Tytthaspis_sedecimpunctata_590 | 0.169 |
| Calvia_decemguttata_195 | Tytthaspis_sedecimpunctata_591 | 0.169 |
| Calvia_decemguttata_199 | Tytthaspis_sedecimpunctata_591 | 0.169 |
| Calvia_decemguttata_200 | Tytthaspis_sedecimpunctata_591 | 0.169 |
| Ceratomegilla_ulkei_221 | Tytthaspis_sedecimpunctata_591 | 0.169 |
| Hippodamia_caseyi_392 | Tytthaspis_sedecimpunctata_593 | 0.169 |
| Hippodamia_convergens_395 | Tytthaspis_sedecimpunctata_593 | 0.169 |
| Hippodamia_moesta_410 | Tytthaspis_sedecimpunctata_593 | 0.169 |
| Hippodamia_caseyi_392 | Tytthaspis_sedecimpunctata_594 | 0.169 |
| Hippodamia_convergens_395 | Tytthaspis_sedecimpunctata_594 | 0.169 |
| Hippodamia_caseyi_392 | Tytthaspis_sedecimpunctata_595 | 0.169 |
| Hippodamia_convergens_395 | Tytthaspis_sedecimpunctata_595 | 0.169 |
| Hyperaspis_proba_661 | Scymnus_rubromaculatus_822 | 0.169 |
| Hyperaspis_proba_661 | Scymnus_rubromaculatus_823 | 0.169 |
| Hyperaspis_proba_661 | Scymnus_rubromaculatus_824 | 0.169 |
| Hyperaspis_proba_661 | Scymnus_rubromaculatus_825 | 0.169 |
| Hyperaspis_proba_661 | Scymnus_rubromaculatus_826 | 0.169 |
| Hyperaspis_proba_661 | Scymnus_rubromaculatus_827 | 0.169 |
| Hippodamia_tredecimpunctata_446 | Tytthaspis_sedecimpunctata_577 | 0.169 |
| Hippodamia_tredecimpunctata_448 | Tytthaspis_sedecimpunctata_577 | 0.169 |
| Hippodamia_tredecimpunctata_449 | Tytthaspis_sedecimpunctata_577 | 0.169 |
| Hippodamia_tredecimpunctata_450 | Tytthaspis_sedecimpunctata_577 | 0.169 |
| Hippodamia_tredecimpunctata_452 | Tytthaspis_sedecimpunctata_577 | 0.169 |
| Hippodamia_tredecimpunctata_453 | Tytthaspis_sedecimpunctata_577 | 0.169 |
| Hippodamia_tredecimpunctata_446 | Tytthaspis_sedecimpunctata_578 | 0.169 |
| Hippodamia_tredecimpunctata_448 | Tytthaspis_sedecimpunctata_578 | 0.169 |
| Hippodamia_tredecimpunctata_449 | Tytthaspis_sedecimpunctata_578 | 0.169 |
| Hippodamia_tredecimpunctata_450 | Tytthaspis_sedecimpunctata_578 | 0.169 |
| Hippodamia_tredecimpunctata_452 | Tytthaspis_sedecimpunctata_578 | 0.169 |
| Hippodamia_tredecimpunctata_453 | Tytthaspis_sedecimpunctata_578 | 0.169 |
| Hippodamia_tredecimpunctata_446 | Tytthaspis_sedecimpunctata_579 | 0.169 |
| Hippodamia_tredecimpunctata_448 | Tytthaspis_sedecimpunctata_579 | 0.169 |

| | | |
|---|---|---|
| Hippodamia_tredecimpunctata_449 | Tytthaspis_sedecimpunctata_579 | 0.169 |
| Hippodamia_tredecimpunctata_450 | Tytthaspis_sedecimpunctata_579 | 0.169 |
| Hippodamia_tredecimpunctata_452 | Tytthaspis_sedecimpunctata_579 | 0.169 |
| Hippodamia_tredecimpunctata_453 | Tytthaspis_sedecimpunctata_579 | 0.169 |
| Hippodamia_tredecimpunctata_446 | Tytthaspis_sedecimpunctata_581 | 0.169 |
| Hippodamia_tredecimpunctata_448 | Tytthaspis_sedecimpunctata_581 | 0.169 |
| Hippodamia_tredecimpunctata_449 | Tytthaspis_sedecimpunctata_581 | 0.169 |
| Hippodamia_tredecimpunctata_450 | Tytthaspis_sedecimpunctata_581 | 0.169 |
| Hippodamia_tredecimpunctata_452 | Tytthaspis_sedecimpunctata_581 | 0.169 |
| Hippodamia_tredecimpunctata_453 | Tytthaspis_sedecimpunctata_581 | 0.169 |
| Hippodamia_tredecimpunctata_446 | Tytthaspis_sedecimpunctata_582 | 0.169 |
| Hippodamia_tredecimpunctata_448 | Tytthaspis_sedecimpunctata_582 | 0.169 |
| Hippodamia_tredecimpunctata_449 | Tytthaspis_sedecimpunctata_582 | 0.169 |
| Hippodamia_tredecimpunctata_450 | Tytthaspis_sedecimpunctata_582 | 0.169 |
| Hippodamia_tredecimpunctata_452 | Tytthaspis_sedecimpunctata_582 | 0.169 |
| Hippodamia_tredecimpunctata_453 | Tytthaspis_sedecimpunctata_582 | 0.169 |
| Hippodamia_caseyi_391 | Tytthaspis_sedecimpunctata_584 | 0.169 |
| Hippodamia_convergens_393 | Tytthaspis_sedecimpunctata_584 | 0.169 |
| Hippodamia_convergens_394 | Tytthaspis_sedecimpunctata_584 | 0.169 |
| Hippodamia_convergens_397 | Tytthaspis_sedecimpunctata_584 | 0.169 |
| Hippodamia_convergens_398 | Tytthaspis_sedecimpunctata_584 | 0.169 |
| Hippodamia_convergens_399 | Tytthaspis_sedecimpunctata_584 | 0.169 |
| Hippodamia_convergens_401 | Tytthaspis_sedecimpunctata_584 | 0.169 |
| Hippodamia_convergens_402 | Tytthaspis_sedecimpunctata_584 | 0.169 |
| Hippodamia_convergens_404 | Tytthaspis_sedecimpunctata_584 | 0.169 |
| Hippodamia_convergens_405 | Tytthaspis_sedecimpunctata_584 | 0.169 |
| Hippodamia_convergens_406 | Tytthaspis_sedecimpunctata_584 | 0.169 |
| Hippodamia_moesta_411 | Tytthaspis_sedecimpunctata_584 | 0.169 |
| Hippodamia_quinquesignata_443 | Tytthaspis_sedecimpunctata_584 | 0.169 |
| Hippodamia_quinquesignata_444 | Tytthaspis_sedecimpunctata_584 | 0.169 |
| Hippodamia_quinquesignata_445 | Tytthaspis_sedecimpunctata_584 | 0.169 |
| Coccinula_quatuordecimpustulata_306 | Tytthaspis_sedecimpunctata_589 | 0.169 |
| Hippodamia_tredecimpunctata_446 | Tytthaspis_sedecimpunctata_590 | 0.169 |
| Hippodamia_tredecimpunctata_448 | Tytthaspis_sedecimpunctata_590 | 0.169 |
| Hippodamia_tredecimpunctata_449 | Tytthaspis_sedecimpunctata_590 | 0.169 |
| Hippodamia_tredecimpunctata_450 | Tytthaspis_sedecimpunctata_590 | 0.169 |
| Hippodamia_tredecimpunctata_452 | Tytthaspis_sedecimpunctata_590 | 0.169 |
| Hippodamia_tredecimpunctata_453 | Tytthaspis_sedecimpunctata_590 | 0.169 |
| Hippodamia_convergens_396 | Tytthaspis_sedecimpunctata_591 | 0.169 |
| Hippodamia_convergens_400 | Tytthaspis_sedecimpunctata_591 | 0.169 |
| Hippodamia_convergens_403 | Tytthaspis_sedecimpunctata_591 | 0.169 |
| Hippodamia_moesta_410 | Tytthaspis_sedecimpunctata_591 | 0.169 |
| Hippodamia_tredecimpunctata_446 | Tytthaspis_sedecimpunctata_594 | 0.169 |
| Hippodamia_tredecimpunctata_448 | Tytthaspis_sedecimpunctata_594 | 0.169 |
| Hippodamia_tredecimpunctata_449 | Tytthaspis_sedecimpunctata_594 | 0.169 |
| Hippodamia_tredecimpunctata_450 | Tytthaspis_sedecimpunctata_594 | 0.169 |
| Hippodamia_tredecimpunctata_452 | Tytthaspis_sedecimpunctata_594 | 0.169 |
| Hippodamia_tredecimpunctata_453 | Tytthaspis_sedecimpunctata_594 | 0.169 |
| Hippodamia_tredecimpunctata_446 | Tytthaspis_sedecimpunctata_595 | 0.169 |
| Hippodamia_tredecimpunctata_448 | Tytthaspis_sedecimpunctata_595 | 0.169 |
| Hippodamia_tredecimpunctata_449 | Tytthaspis_sedecimpunctata_595 | 0.169 |
| Hippodamia_tredecimpunctata_450 | Tytthaspis_sedecimpunctata_595 | 0.169 |
| Hippodamia_tredecimpunctata_452 | Tytthaspis_sedecimpunctata_595 | 0.169 |
| Hippodamia_tredecimpunctata_453 | Tytthaspis_sedecimpunctata_595 | 0.169 |
| Psyllobora_vigintiduopunctata_569 | Tytthaspis_sedecimpunctata_584 | 0.169 |
| Hippodamia_tredecimpunctata_447 | Tytthaspis_sedecimpunctata_584 | 0.169 |

Supplementary Table S4 (continued)

| | | |
|---|---|---|
| Hippodamia_tredecimpunctata_451 | Tytthaspis_sedecimpunctata_584 | 0.169 |
| Hippodamia_tredecimpunctata_454 | Tytthaspis_sedecimpunctata_584 | 0.169 |
| Hippodamia_tredecimpunctata_455 | Tytthaspis_sedecimpunctata_584 | 0.169 |
| Hippodamia_tredecimpunctata_456 | Tytthaspis_sedecimpunctata_584 | 0.169 |
| Coccinula_quatuordecimpustulata_304 | Tytthaspis_sedecimpunctata_588 | 0.169 |
| Coccinula_quatuordecimpustulata_298 | Tytthaspis_sedecimpunctata_589 | 0.169 |
| Coccinula_quatuordecimpustulata_299 | Tytthaspis_sedecimpunctata_589 | 0.169 |
| Coccinula_quatuordecimpustulata_301 | Tytthaspis_sedecimpunctata_589 | 0.169 |
| Coccinula_quatuordecimpustulata_302 | Tytthaspis_sedecimpunctata_589 | 0.169 |
| Coccinula_quatuordecimpustulata_303 | Tytthaspis_sedecimpunctata_589 | 0.169 |
| Coccinula_quatuordecimpustulata_309 | Tytthaspis_sedecimpunctata_589 | 0.169 |
| Calvia_quatuordecimguttata_203 | Tytthaspis_sedecimpunctata_587 | 0.169 |
| Coccinula_quatuordecimpustulata_292 | Tytthaspis_sedecimpunctata_587 | 0.169 |
| Coccinula_quatuordecimpustulata_293 | Tytthaspis_sedecimpunctata_587 | 0.169 |
| Coccinula_quatuordecimpustulata_307 | Tytthaspis_sedecimpunctata_587 | 0.169 |
| Coccinula_quatuordecimpustulata_300 | Tytthaspis_sedecimpunctata_588 | 0.169 |
| Coccinula_quatuordecimpustulata_294 | Tytthaspis_sedecimpunctata_591 | 0.169 |
| Coccinula_quatuordecimpustulata_298 | Tytthaspis_sedecimpunctata_591 | 0.169 |
| Coccinula_quatuordecimpustulata_308 | Tytthaspis_sedecimpunctata_591 | 0.169 |
| Scymnus_auritus_702 | Scymnus_sinuanodulus_830 | 0.169 |
| Psyllobora_vigintiduopunctata_558 | Tytthaspis_sedecimpunctata_591 | 0.169 |
| Psyllobora_vigintiduopunctata_568 | Tytthaspis_sedecimpunctata_591 | 0.169 |
| Oenopia_conglobata_524 | Tytthaspis_sedecimpunctata_587 | 0.169 |
| Hyperaspis_proba_661 | Scymnus_sinuanodulus_831 | 0.169 |
| Scymnus_mimulus_775 | Scymnus_sinuanodulus_831 | 0.169 |
| Scymnus_mimulus_776 | Scymnus_sinuanodulus_831 | 0.169 |
| Coccinula_quatuordecimpustulata_296 | Tytthaspis_sedecimpunctata_591 | 0.169 |
| Scymnus_auritus_698 | Scymnus_sinuanodulus_828 | 0.169 |
| Scymnus_auritus_700 | Scymnus_sinuanodulus_828 | 0.169 |
| Scymnus_apetzi_694 | Scymnus_suturalis_841 | 0.169 |
| Scymnus_apetzi_694 | Scymnus_suturalis_842 | 0.169 |
| Scymnus_apetzi_694 | Scymnus_suturalis_844 | 0.169 |
| Scymnus_apetzi_694 | Scymnus_suturalis_846 | 0.169 |
| Scymnus_apetzi_694 | Scymnus_suturalis_847 | 0.169 |
| Scymnus_apetzi_694 | Scymnus_suturalis_848 | 0.169 |
| Scymnus_apetzi_694 | Scymnus_suturalis_832 | 0.169 |
| Scymnus_apetzi_694 | Scymnus_suturalis_835 | 0.169 |
| Scymnus_apetzi_694 | Scymnus_suturalis_836 | 0.169 |
| Scymnus_apetzi_694 | Scymnus_suturalis_839 | 0.169 |
| Oenopia_conglobata_524 | Tytthaspis_sedecimpunctata_584 | 0.169 |
| Scymnus_mitior_780 | Scymnus_sinuanodulus_831 | 0.169 |
| Scymnus_nubilus_796 | Scymnus_rubromaculatus_822 | 0.169 |
| Scymnus_nubilus_802 | Scymnus_rubromaculatus_822 | 0.169 |
| Scymnus_nubilus_796 | Scymnus_rubromaculatus_823 | 0.169 |
| Scymnus_nubilus_802 | Scymnus_rubromaculatus_823 | 0.169 |
| Scymnus_nubilus_796 | Scymnus_rubromaculatus_824 | 0.169 |
| Scymnus_nubilus_802 | Scymnus_rubromaculatus_824 | 0.169 |
| Scymnus_nubilus_796 | Scymnus_rubromaculatus_825 | 0.169 |
| Scymnus_nubilus_802 | Scymnus_rubromaculatus_825 | 0.169 |
| Scymnus_nubilus_796 | Scymnus_rubromaculatus_826 | 0.169 |
| Scymnus_nubilus_802 | Scymnus_rubromaculatus_826 | 0.169 |
| Scymnus_nubilus_796 | Scymnus_rubromaculatus_827 | 0.169 |
| Scymnus_nubilus_802 | Scymnus_rubromaculatus_827 | 0.169 |
| Scymnus_apetzi_696 | Scymnus_sinuanodulus_828 | 0.169 |
| Scymnus_apetzi_692 | Scymnus_sinuanodulus_828 | 0.169 |
| Brumus_quadripustulatus_5 | Tytthaspis_sedecimpunctata_589 | 0.169 |

| | | |
|---|---|---|
| Myrrha_octodecimguttata_489 | Vibidia_duodecimguttata_596 | 0.169 |
| Myrrha_octodecimguttata_500 | Vibidia_duodecimguttata_596 | 0.169 |
| Myrrha_octodecimguttata_488 | Vibidia_duodecimguttata_599 | 0.169 |
| Myrrha_octodecimguttata_491 | Vibidia_duodecimguttata_599 | 0.169 |
| Myrrha_octodecimguttata_492 | Vibidia_duodecimguttata_599 | 0.169 |
| Myrrha_octodecimguttata_488 | Vibidia_duodecimguttata_602 | 0.169 |
| Myrrha_octodecimguttata_491 | Vibidia_duodecimguttata_602 | 0.169 |
| Myrrha_octodecimguttata_492 | Vibidia_duodecimguttata_602 | 0.169 |
| Myrrha_octodecimguttata_489 | Vibidia_duodecimguttata_603 | 0.169 |
| Myrrha_octodecimguttata_500 | Vibidia_duodecimguttata_603 | 0.169 |
| Brumus_quadripustulatus_5 | Tytthaspis_sedecimpunctata_588 | 0.169 |
| Brumus_quadripustulatus_5 | Tytthaspis_sedecimpunctata_591 | 0.169 |
| Platynaspis_luteorubra_50 | Scymnus_suturalis_833 | 0.169 |
| Hyperaspis_proba_661 | Scymnus_sinuanodulus_829 | 0.169 |
| Scymnus_mimulus_775 | Scymnus_sinuanodulus_829 | 0.169 |
| Scymnus_mimulus_776 | Scymnus_sinuanodulus_829 | 0.169 |
| Scymnus_apetzi_690 | Scymnus_sinuanodulus_831 | 0.169 |
| Scymnus_apetzi_697 | Scymnus_sinuanodulus_831 | 0.169 |
| Platynaspis_luteorubra_50 | Scymnus_suturalis_841 | 0.170 |
| Platynaspis_luteorubra_50 | Scymnus_suturalis_842 | 0.170 |
| Platynaspis_luteorubra_50 | Scymnus_suturalis_844 | 0.170 |
| Platynaspis_luteorubra_50 | Scymnus_suturalis_846 | 0.170 |
| Platynaspis_luteorubra_50 | Scymnus_suturalis_847 | 0.170 |
| Platynaspis_luteorubra_50 | Scymnus_suturalis_848 | 0.170 |
| Platynaspis_luteorubra_50 | Scymnus_suturalis_832 | 0.170 |
| Platynaspis_luteorubra_50 | Scymnus_suturalis_835 | 0.170 |
| Platynaspis_luteorubra_50 | Scymnus_suturalis_836 | 0.170 |
| Platynaspis_luteorubra_50 | Scymnus_suturalis_839 | 0.170 |
| Calvia_quatuordecimguttata_209 | Vibidia_duodecimguttata_596 | 0.170 |
| Calvia_quatuordecimguttata_218 | Vibidia_duodecimguttata_596 | 0.170 |
| Calvia_quatuordecimguttata_201 | Vibidia_duodecimguttata_597 | 0.170 |
| Myrrha_octodecimguttata_485 | Vibidia_duodecimguttata_598 | 0.170 |
| Myrrha_octodecimguttata_486 | Vibidia_duodecimguttata_598 | 0.170 |
| Myrrha_octodecimguttata_487 | Vibidia_duodecimguttata_598 | 0.170 |
| Myrrha_octodecimguttata_490 | Vibidia_duodecimguttata_598 | 0.170 |
| Myrrha_octodecimguttata_494 | Vibidia_duodecimguttata_598 | 0.170 |
| Myrrha_octodecimguttata_496 | Vibidia_duodecimguttata_598 | 0.170 |
| Myrrha_octodecimguttata_497 | Vibidia_duodecimguttata_598 | 0.170 |
| Myrrha_octodecimguttata_501 | Vibidia_duodecimguttata_598 | 0.170 |
| Myrrha_octodecimguttata_502 | Vibidia_duodecimguttata_598 | 0.170 |
| Calvia_quatuordecimguttata_201 | Vibidia_duodecimguttata_600 | 0.170 |
| Scymnus_camptodromus_715 | Tytthaspis_sedecimpunctata_589 | 0.170 |
| Scymnus_camptodromus_715 | Tytthaspis_sedecimpunctata_586 | 0.170 |
| Scymnus_camptodromus_715 | Tytthaspis_sedecimpunctata_588 | 0.170 |
| Calvia_quatuordecimguttata_204 | Vibidia_duodecimguttata_596 | 0.170 |
| Calvia_quatuordecimguttata_208 | Vibidia_duodecimguttata_596 | 0.170 |
| Calvia_quatuordecimguttata_217 | Vibidia_duodecimguttata_596 | 0.170 |
| Anatis_labiculata_123 | Vibidia_duodecimguttata_597 | 0.170 |
| Anatis_labiculata_124 | Vibidia_duodecimguttata_597 | 0.170 |
| Anatis_labiculata_125 | Vibidia_duodecimguttata_597 | 0.170 |
| Anatis_labiculata_127 | Vibidia_duodecimguttata_597 | 0.170 |
| Calvia_quatuordecimguttata_211 | Vibidia_duodecimguttata_598 | 0.170 |
| Calvia_quatuordecimguttata_212 | Vibidia_duodecimguttata_598 | 0.170 |
| Calvia_quatuordecimguttata_213 | Vibidia_duodecimguttata_598 | 0.170 |
| Calvia_quatuordecimguttata_214 | Vibidia_duodecimguttata_598 | 0.170 |
| Calvia_quatuordecimguttata_215 | Vibidia_duodecimguttata_598 | 0.170 |

Supplementary Table S4 (continued)

| | | |
|---|---|---|
| Myrrha_octodecimguttata_495 | Vibidia_duodecimguttata_598 | 0.170 |
| Calvia_quatuordecimguttata_211 | Vibidia_duodecimguttata_599 | 0.170 |
| Calvia_quatuordecimguttata_212 | Vibidia_duodecimguttata_599 | 0.170 |
| Calvia_quatuordecimguttata_213 | Vibidia_duodecimguttata_599 | 0.170 |
| Calvia_quatuordecimguttata_214 | Vibidia_duodecimguttata_599 | 0.170 |
| Calvia_quatuordecimguttata_215 | Vibidia_duodecimguttata_599 | 0.170 |
| Anatis_labiculata_123 | Vibidia_duodecimguttata_600 | 0.170 |
| Anatis_labiculata_124 | Vibidia_duodecimguttata_600 | 0.170 |
| Anatis_labiculata_125 | Vibidia_duodecimguttata_600 | 0.170 |
| Anatis_labiculata_127 | Vibidia_duodecimguttata_600 | 0.170 |
| Calvia_quatuordecimguttata_211 | Vibidia_duodecimguttata_601 | 0.170 |
| Calvia_quatuordecimguttata_212 | Vibidia_duodecimguttata_601 | 0.170 |
| Calvia_quatuordecimguttata_213 | Vibidia_duodecimguttata_601 | 0.170 |
| Calvia_quatuordecimguttata_214 | Vibidia_duodecimguttata_601 | 0.170 |
| Calvia_quatuordecimguttata_215 | Vibidia_duodecimguttata_601 | 0.170 |
| Calvia_quatuordecimguttata_211 | Vibidia_duodecimguttata_602 | 0.170 |
| Calvia_quatuordecimguttata_212 | Vibidia_duodecimguttata_602 | 0.170 |
| Calvia_quatuordecimguttata_213 | Vibidia_duodecimguttata_602 | 0.170 |
| Calvia_quatuordecimguttata_214 | Vibidia_duodecimguttata_602 | 0.170 |
| Calvia_quatuordecimguttata_215 | Vibidia_duodecimguttata_602 | 0.170 |
| Calvia_quatuordecimguttata_201 | Vibidia_duodecimguttata_603 | 0.170 |
| Scymnus_abietis_683 | Scymnus_rubromaculatus_822 | 0.170 |
| Scymnus_abietis_684 | Scymnus_rubromaculatus_822 | 0.170 |
| Scymnus_abietis_686 | Scymnus_rubromaculatus_822 | 0.170 |
| Scymnus_abietis_688 | Scymnus_rubromaculatus_822 | 0.170 |
| Scymnus_abietis_683 | Scymnus_rubromaculatus_823 | 0.170 |
| Scymnus_abietis_684 | Scymnus_rubromaculatus_823 | 0.170 |
| Scymnus_abietis_686 | Scymnus_rubromaculatus_823 | 0.170 |
| Scymnus_abietis_688 | Scymnus_rubromaculatus_823 | 0.170 |
| Scymnus_abietis_683 | Scymnus_rubromaculatus_824 | 0.170 |
| Scymnus_abietis_684 | Scymnus_rubromaculatus_824 | 0.170 |
| Scymnus_abietis_686 | Scymnus_rubromaculatus_824 | 0.170 |
| Scymnus_abietis_688 | Scymnus_rubromaculatus_824 | 0.170 |
| Scymnus_abietis_683 | Scymnus_rubromaculatus_825 | 0.170 |
| Scymnus_abietis_684 | Scymnus_rubromaculatus_825 | 0.170 |
| Scymnus_abietis_686 | Scymnus_rubromaculatus_825 | 0.170 |
| Scymnus_abietis_688 | Scymnus_rubromaculatus_825 | 0.170 |
| Scymnus_abietis_683 | Scymnus_rubromaculatus_826 | 0.170 |
| Scymnus_abietis_684 | Scymnus_rubromaculatus_826 | 0.170 |
| Scymnus_abietis_686 | Scymnus_rubromaculatus_826 | 0.170 |
| Scymnus_abietis_688 | Scymnus_rubromaculatus_826 | 0.170 |
| Scymnus_abietis_683 | Scymnus_rubromaculatus_827 | 0.170 |
| Scymnus_abietis_684 | Scymnus_rubromaculatus_827 | 0.170 |
| Scymnus_abietis_686 | Scymnus_rubromaculatus_827 | 0.170 |
| Scymnus_abietis_688 | Scymnus_rubromaculatus_827 | 0.170 |
| Scymnus_mitior_780 | Scymnus_sinuanodulus_829 | 0.170 |
| Hyperaspis_proba_661 | Tytthaspis_sedecimpunctata_583 | 0.170 |
| Chilocorus_stigma_43 | Tytthaspis_sedecimpunctata_591 | 0.170 |
| Scymnus_apetzi_694 | Scymnus_sinuanodulus_828 | 0.170 |
| Brumus_quadripustulatus_15 | Tytthaspis_sedecimpunctata_584 | 0.170 |
| Brumus_quadripustulatus_16 | Tytthaspis_sedecimpunctata_584 | 0.170 |
| Brumus_quadripustulatus_6 | Tytthaspis_sedecimpunctata_584 | 0.170 |
| Brumus_quadripustulatus_8 | Tytthaspis_sedecimpunctata_584 | 0.170 |
| Chilocorus_stigma_43 | Tytthaspis_sedecimpunctata_593 | 0.170 |
| Chilocorus_nigritus_26 | Scymnus_suturalis_843 | 0.170 |
| Chilocorus_nigritus_26 | Scymnus_suturalis_845 | 0.170 |

| | | |
|---|---|---|
| Brumus_quadripustulatus_12 | Tytthaspis_sedecimpunctata_577 | 0.170 |
| Brumus_quadripustulatus_12 | Tytthaspis_sedecimpunctata_578 | 0.170 |
| Brumus_quadripustulatus_12 | Tytthaspis_sedecimpunctata_579 | 0.170 |
| Brumus_quadripustulatus_12 | Tytthaspis_sedecimpunctata_581 | 0.170 |
| Brumus_quadripustulatus_12 | Tytthaspis_sedecimpunctata_582 | 0.170 |
| Brumus_quadripustulatus_9 | Tytthaspis_sedecimpunctata_583 | 0.170 |
| Chilocorus_stigma_44 | Tytthaspis_sedecimpunctata_584 | 0.170 |
| Chilocorus_stigma_46 | Tytthaspis_sedecimpunctata_584 | 0.170 |
| Chilocorus_stigma_47 | Tytthaspis_sedecimpunctata_584 | 0.170 |
| Brumus_quadripustulatus_12 | Tytthaspis_sedecimpunctata_590 | 0.170 |
| Brumus_quadripustulatus_12 | Tytthaspis_sedecimpunctata_592 | 0.170 |
| Brumus_quadripustulatus_12 | Tytthaspis_sedecimpunctata_594 | 0.170 |
| Brumus_quadripustulatus_12 | Tytthaspis_sedecimpunctata_595 | 0.170 |
| Hyperaspis_reppensis_662 | Scymnus_suturalis_841 | 0.170 |
| Hyperaspis_reppensis_663 | Scymnus_suturalis_841 | 0.170 |
| Hyperaspis_reppensis_662 | Scymnus_suturalis_842 | 0.170 |
| Hyperaspis_reppensis_663 | Scymnus_suturalis_842 | 0.170 |
| Hyperaspis_reppensis_662 | Scymnus_suturalis_844 | 0.170 |
| Hyperaspis_reppensis_663 | Scymnus_suturalis_844 | 0.170 |
| Hyperaspis_reppensis_662 | Scymnus_suturalis_846 | 0.170 |
| Hyperaspis_reppensis_663 | Scymnus_suturalis_846 | 0.170 |
| Hyperaspis_reppensis_662 | Scymnus_suturalis_847 | 0.170 |
| Hyperaspis_reppensis_663 | Scymnus_suturalis_847 | 0.170 |
| Hyperaspis_reppensis_662 | Scymnus_suturalis_848 | 0.170 |
| Hyperaspis_reppensis_663 | Scymnus_suturalis_848 | 0.170 |
| Brumus_quadripustulatus_9 | Tytthaspis_sedecimpunctata_580 | 0.170 |
| Propylaea_quatuordecimpunctata_532 | Tytthaspis_sedecimpunctata_584 | 0.170 |
| Propylaea_quatuordecimpunctata_533 | Tytthaspis_sedecimpunctata_584 | 0.170 |
| Propylaea_quatuordecimpunctata_534 | Tytthaspis_sedecimpunctata_584 | 0.170 |
| Propylaea_quatuordecimpunctata_535 | Tytthaspis_sedecimpunctata_584 | 0.170 |
| Propylaea_quatuordecimpunctata_536 | Tytthaspis_sedecimpunctata_584 | 0.170 |
| Propylaea_quatuordecimpunctata_537 | Tytthaspis_sedecimpunctata_584 | 0.170 |
| Propylaea_quatuordecimpunctata_538 | Tytthaspis_sedecimpunctata_584 | 0.170 |
| Propylaea_quatuordecimpunctata_541 | Tytthaspis_sedecimpunctata_584 | 0.170 |
| Propylaea_quatuordecimpunctata_544 | Tytthaspis_sedecimpunctata_584 | 0.170 |
| Propylaea_quatuordecimpunctata_549 | Tytthaspis_sedecimpunctata_584 | 0.170 |
| Coccinella_magnifica_228 | Tytthaspis_sedecimpunctata_587 | 0.170 |
| Coccinella_magnifica_229 | Tytthaspis_sedecimpunctata_587 | 0.170 |
| Coccinella_magnifica_230 | Tytthaspis_sedecimpunctata_587 | 0.170 |
| Coccinella_magnifica_231 | Tytthaspis_sedecimpunctata_587 | 0.170 |
| Adalia_bipunctata_99 | Tytthaspis_sedecimpunctata_589 | 0.170 |
| Chilocorus_bipustulatus_19 | Tytthaspis_sedecimpunctata_591 | 0.170 |
| Chilocorus_bipustulatus_22 | Tytthaspis_sedecimpunctata_591 | 0.170 |
| Hyperaspis_reppensis_662 | Scymnus_suturalis_832 | 0.170 |
| Hyperaspis_reppensis_663 | Scymnus_suturalis_832 | 0.170 |
| Hyperaspis_reppensis_662 | Scymnus_suturalis_835 | 0.170 |
| Hyperaspis_reppensis_663 | Scymnus_suturalis_835 | 0.170 |
| Hyperaspis_reppensis_662 | Scymnus_suturalis_836 | 0.170 |
| Hyperaspis_reppensis_663 | Scymnus_suturalis_836 | 0.170 |
| Chilocorus_nigritus_27 | Scymnus_suturalis_838 | 0.170 |
| Hyperaspis_reppensis_662 | Scymnus_suturalis_839 | 0.170 |
| Hyperaspis_reppensis_663 | Scymnus_suturalis_839 | 0.170 |
| Chilocorus_nigritus_27 | Scymnus_suturalis_840 | 0.170 |
| Propylaea_quatuordecimpunctata_532 | Tytthaspis_sedecimpunctata_577 | 0.170 |
| Propylaea_quatuordecimpunctata_533 | Tytthaspis_sedecimpunctata_577 | 0.170 |
| Propylaea_quatuordecimpunctata_534 | Tytthaspis_sedecimpunctata_577 | 0.170 |

| | | |
|---|---|---|
| Propylaea_quatuordecimpunctata_535 | Tytthaspis_sedecimpunctata_577 | 0.170 |
| Propylaea_quatuordecimpunctata_536 | Tytthaspis_sedecimpunctata_577 | 0.170 |
| Propylaea_quatuordecimpunctata_537 | Tytthaspis_sedecimpunctata_577 | 0.170 |
| Propylaea_quatuordecimpunctata_538 | Tytthaspis_sedecimpunctata_577 | 0.170 |
| Propylaea_quatuordecimpunctata_541 | Tytthaspis_sedecimpunctata_577 | 0.170 |
| Propylaea_quatuordecimpunctata_544 | Tytthaspis_sedecimpunctata_577 | 0.170 |
| Propylaea_quatuordecimpunctata_549 | Tytthaspis_sedecimpunctata_577 | 0.170 |
| Propylaea_quatuordecimpunctata_532 | Tytthaspis_sedecimpunctata_578 | 0.170 |
| Propylaea_quatuordecimpunctata_533 | Tytthaspis_sedecimpunctata_578 | 0.170 |
| Propylaea_quatuordecimpunctata_534 | Tytthaspis_sedecimpunctata_578 | 0.170 |
| Propylaea_quatuordecimpunctata_535 | Tytthaspis_sedecimpunctata_578 | 0.170 |
| Propylaea_quatuordecimpunctata_536 | Tytthaspis_sedecimpunctata_578 | 0.170 |
| Propylaea_quatuordecimpunctata_537 | Tytthaspis_sedecimpunctata_578 | 0.170 |
| Propylaea_quatuordecimpunctata_538 | Tytthaspis_sedecimpunctata_578 | 0.170 |
| Propylaea_quatuordecimpunctata_541 | Tytthaspis_sedecimpunctata_578 | 0.170 |
| Propylaea_quatuordecimpunctata_544 | Tytthaspis_sedecimpunctata_578 | 0.170 |
| Propylaea_quatuordecimpunctata_549 | Tytthaspis_sedecimpunctata_578 | 0.170 |
| Propylaea_quatuordecimpunctata_532 | Tytthaspis_sedecimpunctata_579 | 0.170 |
| Propylaea_quatuordecimpunctata_533 | Tytthaspis_sedecimpunctata_579 | 0.170 |
| Propylaea_quatuordecimpunctata_534 | Tytthaspis_sedecimpunctata_579 | 0.170 |
| Propylaea_quatuordecimpunctata_535 | Tytthaspis_sedecimpunctata_579 | 0.170 |
| Propylaea_quatuordecimpunctata_536 | Tytthaspis_sedecimpunctata_579 | 0.170 |
| Propylaea_quatuordecimpunctata_537 | Tytthaspis_sedecimpunctata_579 | 0.170 |
| Propylaea_quatuordecimpunctata_538 | Tytthaspis_sedecimpunctata_579 | 0.170 |
| Propylaea_quatuordecimpunctata_541 | Tytthaspis_sedecimpunctata_579 | 0.170 |
| Propylaea_quatuordecimpunctata_544 | Tytthaspis_sedecimpunctata_579 | 0.170 |
| Propylaea_quatuordecimpunctata_549 | Tytthaspis_sedecimpunctata_579 | 0.170 |
| Propylaea_quatuordecimpunctata_532 | Tytthaspis_sedecimpunctata_581 | 0.170 |
| Propylaea_quatuordecimpunctata_533 | Tytthaspis_sedecimpunctata_581 | 0.170 |
| Propylaea_quatuordecimpunctata_534 | Tytthaspis_sedecimpunctata_581 | 0.170 |
| Propylaea_quatuordecimpunctata_535 | Tytthaspis_sedecimpunctata_581 | 0.170 |
| Propylaea_quatuordecimpunctata_536 | Tytthaspis_sedecimpunctata_581 | 0.170 |
| Propylaea_quatuordecimpunctata_537 | Tytthaspis_sedecimpunctata_581 | 0.170 |
| Propylaea_quatuordecimpunctata_538 | Tytthaspis_sedecimpunctata_581 | 0.170 |
| Propylaea_quatuordecimpunctata_541 | Tytthaspis_sedecimpunctata_581 | 0.170 |
| Propylaea_quatuordecimpunctata_544 | Tytthaspis_sedecimpunctata_581 | 0.170 |
| Propylaea_quatuordecimpunctata_549 | Tytthaspis_sedecimpunctata_581 | 0.170 |
| Propylaea_quatuordecimpunctata_532 | Tytthaspis_sedecimpunctata_582 | 0.170 |
| Propylaea_quatuordecimpunctata_533 | Tytthaspis_sedecimpunctata_582 | 0.170 |
| Propylaea_quatuordecimpunctata_534 | Tytthaspis_sedecimpunctata_582 | 0.170 |
| Propylaea_quatuordecimpunctata_535 | Tytthaspis_sedecimpunctata_582 | 0.170 |
| Propylaea_quatuordecimpunctata_536 | Tytthaspis_sedecimpunctata_582 | 0.170 |
| Propylaea_quatuordecimpunctata_537 | Tytthaspis_sedecimpunctata_582 | 0.170 |
| Propylaea_quatuordecimpunctata_538 | Tytthaspis_sedecimpunctata_582 | 0.170 |
| Propylaea_quatuordecimpunctata_541 | Tytthaspis_sedecimpunctata_582 | 0.170 |
| Propylaea_quatuordecimpunctata_544 | Tytthaspis_sedecimpunctata_582 | 0.170 |
| Propylaea_quatuordecimpunctata_549 | Tytthaspis_sedecimpunctata_582 | 0.170 |
| Propylaea_quatuordecimpunctata_539 | Tytthaspis_sedecimpunctata_583 | 0.170 |
| Propylaea_quatuordecimpunctata_540 | Tytthaspis_sedecimpunctata_583 | 0.170 |
| Propylaea_quatuordecimpunctata_543 | Tytthaspis_sedecimpunctata_583 | 0.170 |
| Propylaea_quatuordecimpunctata_548 | Tytthaspis_sedecimpunctata_583 | 0.170 |
| Coccinella_transversoguttata_265 | Tytthaspis_sedecimpunctata_584 | 0.170 |
| Coccinella_transversoguttata_266 | Tytthaspis_sedecimpunctata_584 | 0.170 |
| Propylaea_quatuordecimpunctata_540 | Tytthaspis_sedecimpunctata_585 | 0.170 |
| Propylaea_quatuordecimpunctata_543 | Tytthaspis_sedecimpunctata_585 | 0.170 |
| Propylaea_quatuordecimpunctata_548 | Tytthaspis_sedecimpunctata_585 | 0.170 |

| | | |
|---|---|---|
| Adalia_bipunctata_105 | Tytthaspis_sedecimpunctata_586 | 0.170 |
| Scymnus_camptodromus_704 | Tytthaspis_sedecimpunctata_586 | 0.170 |
| Scymnus_camptodromus_704 | Tytthaspis_sedecimpunctata_588 | 0.170 |
| Adalia_bipunctata_100 | Tytthaspis_sedecimpunctata_589 | 0.170 |
| Propylaea_quatuordecimpunctata_542 | Tytthaspis_sedecimpunctata_589 | 0.170 |
| Propylaea_quatuordecimpunctata_546 | Tytthaspis_sedecimpunctata_589 | 0.170 |
| Propylaea_quatuordecimpunctata_532 | Tytthaspis_sedecimpunctata_590 | 0.170 |
| Propylaea_quatuordecimpunctata_533 | Tytthaspis_sedecimpunctata_590 | 0.170 |
| Propylaea_quatuordecimpunctata_534 | Tytthaspis_sedecimpunctata_590 | 0.170 |
| Propylaea_quatuordecimpunctata_535 | Tytthaspis_sedecimpunctata_590 | 0.170 |
| Propylaea_quatuordecimpunctata_536 | Tytthaspis_sedecimpunctata_590 | 0.170 |
| Propylaea_quatuordecimpunctata_537 | Tytthaspis_sedecimpunctata_590 | 0.170 |
| Propylaea_quatuordecimpunctata_538 | Tytthaspis_sedecimpunctata_590 | 0.170 |
| Propylaea_quatuordecimpunctata_541 | Tytthaspis_sedecimpunctata_590 | 0.170 |
| Propylaea_quatuordecimpunctata_544 | Tytthaspis_sedecimpunctata_590 | 0.170 |
| Propylaea_quatuordecimpunctata_549 | Tytthaspis_sedecimpunctata_590 | 0.170 |
| Propylaea_quatuordecimpunctata_539 | Tytthaspis_sedecimpunctata_592 | 0.170 |
| Propylaea_quatuordecimpunctata_540 | Tytthaspis_sedecimpunctata_592 | 0.170 |
| Propylaea_quatuordecimpunctata_543 | Tytthaspis_sedecimpunctata_592 | 0.170 |
| Propylaea_quatuordecimpunctata_548 | Tytthaspis_sedecimpunctata_592 | 0.170 |
| Propylaea_quatuordecimpunctata_532 | Tytthaspis_sedecimpunctata_593 | 0.170 |
| Propylaea_quatuordecimpunctata_533 | Tytthaspis_sedecimpunctata_593 | 0.170 |
| Propylaea_quatuordecimpunctata_534 | Tytthaspis_sedecimpunctata_593 | 0.170 |
| Propylaea_quatuordecimpunctata_535 | Tytthaspis_sedecimpunctata_593 | 0.170 |
| Propylaea_quatuordecimpunctata_536 | Tytthaspis_sedecimpunctata_593 | 0.170 |
| Propylaea_quatuordecimpunctata_537 | Tytthaspis_sedecimpunctata_593 | 0.170 |
| Propylaea_quatuordecimpunctata_538 | Tytthaspis_sedecimpunctata_593 | 0.170 |
| Propylaea_quatuordecimpunctata_541 | Tytthaspis_sedecimpunctata_593 | 0.170 |
| Propylaea_quatuordecimpunctata_544 | Tytthaspis_sedecimpunctata_593 | 0.170 |
| Propylaea_quatuordecimpunctata_549 | Tytthaspis_sedecimpunctata_593 | 0.170 |
| Propylaea_quatuordecimpunctata_532 | Tytthaspis_sedecimpunctata_594 | 0.170 |
| Propylaea_quatuordecimpunctata_533 | Tytthaspis_sedecimpunctata_594 | 0.170 |
| Propylaea_quatuordecimpunctata_534 | Tytthaspis_sedecimpunctata_594 | 0.170 |
| Propylaea_quatuordecimpunctata_535 | Tytthaspis_sedecimpunctata_594 | 0.170 |
| Propylaea_quatuordecimpunctata_536 | Tytthaspis_sedecimpunctata_594 | 0.170 |
| Propylaea_quatuordecimpunctata_537 | Tytthaspis_sedecimpunctata_594 | 0.170 |
| Propylaea_quatuordecimpunctata_538 | Tytthaspis_sedecimpunctata_594 | 0.170 |
| Propylaea_quatuordecimpunctata_541 | Tytthaspis_sedecimpunctata_594 | 0.170 |
| Propylaea_quatuordecimpunctata_544 | Tytthaspis_sedecimpunctata_594 | 0.170 |
| Propylaea_quatuordecimpunctata_549 | Tytthaspis_sedecimpunctata_594 | 0.170 |
| Propylaea_quatuordecimpunctata_532 | Tytthaspis_sedecimpunctata_595 | 0.170 |
| Propylaea_quatuordecimpunctata_533 | Tytthaspis_sedecimpunctata_595 | 0.170 |
| Propylaea_quatuordecimpunctata_534 | Tytthaspis_sedecimpunctata_595 | 0.170 |
| Propylaea_quatuordecimpunctata_535 | Tytthaspis_sedecimpunctata_595 | 0.170 |
| Propylaea_quatuordecimpunctata_536 | Tytthaspis_sedecimpunctata_595 | 0.170 |
| Propylaea_quatuordecimpunctata_537 | Tytthaspis_sedecimpunctata_595 | 0.170 |
| Propylaea_quatuordecimpunctata_538 | Tytthaspis_sedecimpunctata_595 | 0.170 |
| Propylaea_quatuordecimpunctata_541 | Tytthaspis_sedecimpunctata_595 | 0.170 |
| Propylaea_quatuordecimpunctata_544 | Tytthaspis_sedecimpunctata_595 | 0.170 |
| Propylaea_quatuordecimpunctata_549 | Tytthaspis_sedecimpunctata_595 | 0.170 |
| Chilocorus_nigritus_27 | Scymnus_suturalis_834 | 0.170 |
| Chilocorus_nigritus_27 | Scymnus_suturalis_837 | 0.170 |
| Scymnus_apetzi_690 | Scymnus_sinuanodulus_829 | 0.170 |
| Scymnus_apetzi_697 | Scymnus_sinuanodulus_829 | 0.170 |
| Psyllobora_vigintimaculata_575 | Vibidia_duodecimguttata_603 | 0.170 |
| Psyllobora_vigintimaculata_576 | Vibidia_duodecimguttata_603 | 0.170 |

| | | |
|---|---|---|
| Hyperaspis_oregona_658 | Scymnus_suturalis_841 | 0.170 |
| Hyperaspis_oregona_658 | Scymnus_suturalis_842 | 0.170 |
| Hyperaspis_oregona_658 | Scymnus_suturalis_844 | 0.170 |
| Hyperaspis_oregona_658 | Scymnus_suturalis_846 | 0.170 |
| Hyperaspis_oregona_658 | Scymnus_suturalis_847 | 0.170 |
| Hyperaspis_oregona_658 | Scymnus_suturalis_848 | 0.170 |
| Adalia_bipunctata_99 | Tytthaspis_sedecimpunctata_577 | 0.170 |
| Coccinella_trifasciata_272 | Tytthaspis_sedecimpunctata_577 | 0.170 |
| Coccinella_trifasciata_276 | Tytthaspis_sedecimpunctata_577 | 0.170 |
| Adalia_bipunctata_99 | Tytthaspis_sedecimpunctata_578 | 0.170 |
| Coccinella_trifasciata_272 | Tytthaspis_sedecimpunctata_578 | 0.170 |
| Coccinella_trifasciata_276 | Tytthaspis_sedecimpunctata_578 | 0.170 |
| Adalia_bipunctata_99 | Tytthaspis_sedecimpunctata_579 | 0.170 |
| Coccinella_trifasciata_272 | Tytthaspis_sedecimpunctata_579 | 0.170 |
| Coccinella_trifasciata_276 | Tytthaspis_sedecimpunctata_579 | 0.170 |
| Propylaea_quatuordecimpunctata_532 | Tytthaspis_sedecimpunctata_580 | 0.170 |
| Propylaea_quatuordecimpunctata_533 | Tytthaspis_sedecimpunctata_580 | 0.170 |
| Propylaea_quatuordecimpunctata_534 | Tytthaspis_sedecimpunctata_580 | 0.170 |
| Propylaea_quatuordecimpunctata_535 | Tytthaspis_sedecimpunctata_580 | 0.170 |
| Propylaea_quatuordecimpunctata_536 | Tytthaspis_sedecimpunctata_580 | 0.170 |
| Propylaea_quatuordecimpunctata_537 | Tytthaspis_sedecimpunctata_580 | 0.170 |
| Propylaea_quatuordecimpunctata_538 | Tytthaspis_sedecimpunctata_580 | 0.170 |
| Propylaea_quatuordecimpunctata_541 | Tytthaspis_sedecimpunctata_580 | 0.170 |
| Propylaea_quatuordecimpunctata_544 | Tytthaspis_sedecimpunctata_580 | 0.170 |
| Propylaea_quatuordecimpunctata_549 | Tytthaspis_sedecimpunctata_580 | 0.170 |
| Adalia_bipunctata_99 | Tytthaspis_sedecimpunctata_581 | 0.170 |
| Coccinella_trifasciata_272 | Tytthaspis_sedecimpunctata_581 | 0.170 |
| Coccinella_trifasciata_276 | Tytthaspis_sedecimpunctata_581 | 0.170 |
| Adalia_bipunctata_99 | Tytthaspis_sedecimpunctata_582 | 0.170 |
| Coccinella_trifasciata_272 | Tytthaspis_sedecimpunctata_582 | 0.170 |
| Coccinella_trifasciata_276 | Tytthaspis_sedecimpunctata_582 | 0.170 |
| Adalia_bipunctata_105 | Tytthaspis_sedecimpunctata_583 | 0.170 |
| Coccinella_transversoguttata_265 | Tytthaspis_sedecimpunctata_583 | 0.170 |
| Coccinella_transversoguttata_266 | Tytthaspis_sedecimpunctata_583 | 0.170 |
| Coccinella_trifasciata_280 | Tytthaspis_sedecimpunctata_583 | 0.170 |
| Adalia_bipunctata_101 | Tytthaspis_sedecimpunctata_586 | 0.170 |
| Adalia_bipunctata_104 | Tytthaspis_sedecimpunctata_586 | 0.170 |
| Adalia_bipunctata_100 | Tytthaspis_sedecimpunctata_587 | 0.170 |
| Harmonia_axyridis_355 | Tytthaspis_sedecimpunctata_589 | 0.170 |
| Adalia_bipunctata_99 | Tytthaspis_sedecimpunctata_590 | 0.170 |
| Coccinella_trifasciata_272 | Tytthaspis_sedecimpunctata_590 | 0.170 |
| Coccinella_trifasciata_276 | Tytthaspis_sedecimpunctata_590 | 0.170 |
| Adalia_bipunctata_105 | Tytthaspis_sedecimpunctata_592 | 0.170 |
| Aphidecta_obliterata_178 | Tytthaspis_sedecimpunctata_592 | 0.170 |
| Coccinella_trifasciata_272 | Tytthaspis_sedecimpunctata_592 | 0.170 |
| Coccinella_trifasciata_276 | Tytthaspis_sedecimpunctata_592 | 0.170 |
| Adalia_bipunctata_99 | Tytthaspis_sedecimpunctata_593 | 0.170 |
| Coccinella_trifasciata_272 | Tytthaspis_sedecimpunctata_593 | 0.170 |
| Coccinella_trifasciata_276 | Tytthaspis_sedecimpunctata_593 | 0.170 |
| Adalia_bipunctata_99 | Tytthaspis_sedecimpunctata_594 | 0.170 |
| Coccinella_trifasciata_272 | Tytthaspis_sedecimpunctata_594 | 0.170 |
| Coccinella_trifasciata_276 | Tytthaspis_sedecimpunctata_594 | 0.170 |
| Adalia_bipunctata_99 | Tytthaspis_sedecimpunctata_595 | 0.170 |
| Coccinella_trifasciata_272 | Tytthaspis_sedecimpunctata_595 | 0.170 |
| Coccinella_trifasciata_276 | Tytthaspis_sedecimpunctata_595 | 0.170 |
| Hyperaspis_oregona_658 | Scymnus_suturalis_832 | 0.170 |

| | | |
|---|---|---|
| Hyperaspis_oregona_658 | Scymnus_suturalis_835 | 0.170 |
| Hyperaspis_oregona_658 | Scymnus_suturalis_836 | 0.170 |
| Hyperaspis_oregona_658 | Scymnus_suturalis_839 | 0.170 |
| Hyperaspis_campestris_651 | Scymnus_suturalis_843 | 0.170 |
| Hyperaspis_campestris_652 | Scymnus_suturalis_843 | 0.170 |
| Hyperaspis_campestris_653 | Scymnus_suturalis_843 | 0.170 |
| Hyperaspis_campestris_651 | Scymnus_suturalis_845 | 0.170 |
| Hyperaspis_campestris_652 | Scymnus_suturalis_845 | 0.170 |
| Hyperaspis_campestris_653 | Scymnus_suturalis_845 | 0.170 |
| Adalia_bipunctata_102 | Tytthaspis_sedecimpunctata_577 | 0.170 |
| Adalia_bipunctata_103 | Tytthaspis_sedecimpunctata_577 | 0.170 |
| Adalia_bipunctata_106 | Tytthaspis_sedecimpunctata_577 | 0.170 |
| Adalia_bipunctata_107 | Tytthaspis_sedecimpunctata_577 | 0.170 |
| Adalia_bipunctata_108 | Tytthaspis_sedecimpunctata_577 | 0.170 |
| Adalia_bipunctata_109 | Tytthaspis_sedecimpunctata_577 | 0.170 |
| Psyllobora_renifer_551 | Tytthaspis_sedecimpunctata_577 | 0.170 |
| Psyllobora_renifer_552 | Tytthaspis_sedecimpunctata_577 | 0.170 |
| Psyllobora_renifer_553 | Tytthaspis_sedecimpunctata_577 | 0.170 |
| Psyllobora_renifer_554 | Tytthaspis_sedecimpunctata_577 | 0.170 |
| Adalia_bipunctata_102 | Tytthaspis_sedecimpunctata_578 | 0.170 |
| Adalia_bipunctata_103 | Tytthaspis_sedecimpunctata_578 | 0.170 |
| Adalia_bipunctata_106 | Tytthaspis_sedecimpunctata_578 | 0.170 |
| Adalia_bipunctata_107 | Tytthaspis_sedecimpunctata_578 | 0.170 |
| Adalia_bipunctata_108 | Tytthaspis_sedecimpunctata_578 | 0.170 |
| Adalia_bipunctata_109 | Tytthaspis_sedecimpunctata_578 | 0.170 |
| Psyllobora_renifer_551 | Tytthaspis_sedecimpunctata_578 | 0.170 |
| Psyllobora_renifer_552 | Tytthaspis_sedecimpunctata_578 | 0.170 |
| Psyllobora_renifer_553 | Tytthaspis_sedecimpunctata_578 | 0.170 |
| Psyllobora_renifer_554 | Tytthaspis_sedecimpunctata_578 | 0.170 |
| Adalia_bipunctata_102 | Tytthaspis_sedecimpunctata_579 | 0.170 |
| Adalia_bipunctata_103 | Tytthaspis_sedecimpunctata_579 | 0.170 |
| Adalia_bipunctata_106 | Tytthaspis_sedecimpunctata_579 | 0.170 |
| Adalia_bipunctata_107 | Tytthaspis_sedecimpunctata_579 | 0.170 |
| Adalia_bipunctata_108 | Tytthaspis_sedecimpunctata_579 | 0.170 |
| Adalia_bipunctata_109 | Tytthaspis_sedecimpunctata_579 | 0.170 |
| Psyllobora_renifer_551 | Tytthaspis_sedecimpunctata_579 | 0.170 |
| Psyllobora_renifer_552 | Tytthaspis_sedecimpunctata_579 | 0.170 |
| Psyllobora_renifer_553 | Tytthaspis_sedecimpunctata_579 | 0.170 |
| Psyllobora_renifer_554 | Tytthaspis_sedecimpunctata_579 | 0.170 |
| Adalia_bipunctata_99 | Tytthaspis_sedecimpunctata_580 | 0.170 |
| Aphidecta_obliterata_178 | Tytthaspis_sedecimpunctata_580 | 0.170 |
| Coccinella_hieroglyphica_225 | Tytthaspis_sedecimpunctata_580 | 0.170 |
| Coccinella_trifasciata_275 | Tytthaspis_sedecimpunctata_580 | 0.170 |
| Coccinella_trifasciata_277 | Tytthaspis_sedecimpunctata_580 | 0.170 |
| Coccinella_trifasciata_278 | Tytthaspis_sedecimpunctata_580 | 0.170 |
| Coccinella_trifasciata_279 | Tytthaspis_sedecimpunctata_580 | 0.170 |
| Coccinella_trifasciata_281 | Tytthaspis_sedecimpunctata_580 | 0.170 |
| Adalia_bipunctata_102 | Tytthaspis_sedecimpunctata_581 | 0.170 |
| Adalia_bipunctata_103 | Tytthaspis_sedecimpunctata_581 | 0.170 |
| Adalia_bipunctata_106 | Tytthaspis_sedecimpunctata_581 | 0.170 |
| Adalia_bipunctata_107 | Tytthaspis_sedecimpunctata_581 | 0.170 |
| Adalia_bipunctata_108 | Tytthaspis_sedecimpunctata_581 | 0.170 |
| Adalia_bipunctata_109 | Tytthaspis_sedecimpunctata_581 | 0.170 |
| Psyllobora_renifer_551 | Tytthaspis_sedecimpunctata_581 | 0.170 |
| Psyllobora_renifer_552 | Tytthaspis_sedecimpunctata_581 | 0.170 |
| Psyllobora_renifer_553 | Tytthaspis_sedecimpunctata_581 | 0.170 |

| | | |
|---|---|---|
| Psyllobora_renifer_554 | Tytthaspis_sedecimpunctata_581 | 0.170 |
| Adalia_bipunctata_102 | Tytthaspis_sedecimpunctata_582 | 0.170 |
| Adalia_bipunctata_103 | Tytthaspis_sedecimpunctata_582 | 0.170 |
| Adalia_bipunctata_106 | Tytthaspis_sedecimpunctata_582 | 0.170 |
| Adalia_bipunctata_107 | Tytthaspis_sedecimpunctata_582 | 0.170 |
| Adalia_bipunctata_108 | Tytthaspis_sedecimpunctata_582 | 0.170 |
| Adalia_bipunctata_109 | Tytthaspis_sedecimpunctata_582 | 0.170 |
| Psyllobora_renifer_551 | Tytthaspis_sedecimpunctata_582 | 0.170 |
| Psyllobora_renifer_552 | Tytthaspis_sedecimpunctata_582 | 0.170 |
| Psyllobora_renifer_553 | Tytthaspis_sedecimpunctata_582 | 0.170 |
| Psyllobora_renifer_554 | Tytthaspis_sedecimpunctata_582 | 0.170 |
| Adalia_bipunctata_101 | Tytthaspis_sedecimpunctata_583 | 0.170 |
| Adalia_bipunctata_104 | Tytthaspis_sedecimpunctata_583 | 0.170 |
| Coccinella_trifasciata_273 | Tytthaspis_sedecimpunctata_584 | 0.170 |
| Harmonia_axyridis_362 | Tytthaspis_sedecimpunctata_585 | 0.170 |
| Psyllobora_renifer_551 | Tytthaspis_sedecimpunctata_585 | 0.170 |
| Psyllobora_renifer_552 | Tytthaspis_sedecimpunctata_585 | 0.170 |
| Psyllobora_renifer_553 | Tytthaspis_sedecimpunctata_585 | 0.170 |
| Psyllobora_renifer_554 | Tytthaspis_sedecimpunctata_585 | 0.170 |
| Scymnus_auritus_703 | Tytthaspis_sedecimpunctata_587 | 0.170 |
| Harmonia_axyridis_355 | Tytthaspis_sedecimpunctata_588 | 0.170 |
| Anatis_ocellata_143 | Tytthaspis_sedecimpunctata_589 | 0.170 |
| Coccinella_transversoguttata_267 | Tytthaspis_sedecimpunctata_589 | 0.170 |
| Coccinella_transversoguttata_268 | Tytthaspis_sedecimpunctata_589 | 0.170 |
| Coccinella_transversoguttata_269 | Tytthaspis_sedecimpunctata_589 | 0.170 |
| Coccinella_transversoguttata_270 | Tytthaspis_sedecimpunctata_589 | 0.170 |
| Coccinella_transversoguttata_271 | Tytthaspis_sedecimpunctata_589 | 0.170 |
| Adalia_bipunctata_102 | Tytthaspis_sedecimpunctata_590 | 0.170 |
| Adalia_bipunctata_103 | Tytthaspis_sedecimpunctata_590 | 0.170 |
| Adalia_bipunctata_106 | Tytthaspis_sedecimpunctata_590 | 0.170 |
| Adalia_bipunctata_107 | Tytthaspis_sedecimpunctata_590 | 0.170 |
| Adalia_bipunctata_108 | Tytthaspis_sedecimpunctata_590 | 0.170 |
| Adalia_bipunctata_109 | Tytthaspis_sedecimpunctata_590 | 0.170 |
| Psyllobora_renifer_551 | Tytthaspis_sedecimpunctata_590 | 0.170 |
| Psyllobora_renifer_552 | Tytthaspis_sedecimpunctata_590 | 0.170 |
| Psyllobora_renifer_553 | Tytthaspis_sedecimpunctata_590 | 0.170 |
| Psyllobora_renifer_554 | Tytthaspis_sedecimpunctata_590 | 0.170 |
| Coccinella_transversoguttata_265 | Tytthaspis_sedecimpunctata_591 | 0.170 |
| Coccinella_transversoguttata_266 | Tytthaspis_sedecimpunctata_591 | 0.170 |
| Coccinella_trifasciata_275 | Tytthaspis_sedecimpunctata_591 | 0.170 |
| Coccinella_trifasciata_278 | Tytthaspis_sedecimpunctata_591 | 0.170 |
| Coccinella_trifasciata_279 | Tytthaspis_sedecimpunctata_591 | 0.170 |
| Coccinella_trifasciata_280 | Tytthaspis_sedecimpunctata_591 | 0.170 |
| Coccinella_trifasciata_281 | Tytthaspis_sedecimpunctata_591 | 0.170 |
| Psyllobora_renifer_555 | Tytthaspis_sedecimpunctata_591 | 0.170 |
| Adalia_bipunctata_101 | Tytthaspis_sedecimpunctata_592 | 0.170 |
| Adalia_bipunctata_104 | Tytthaspis_sedecimpunctata_592 | 0.170 |
| Adalia_bipunctata_102 | Tytthaspis_sedecimpunctata_594 | 0.170 |
| Adalia_bipunctata_103 | Tytthaspis_sedecimpunctata_594 | 0.170 |
| Adalia_bipunctata_106 | Tytthaspis_sedecimpunctata_594 | 0.170 |
| Adalia_bipunctata_107 | Tytthaspis_sedecimpunctata_594 | 0.170 |
| Adalia_bipunctata_108 | Tytthaspis_sedecimpunctata_594 | 0.170 |
| Adalia_bipunctata_109 | Tytthaspis_sedecimpunctata_594 | 0.170 |
| Psyllobora_renifer_551 | Tytthaspis_sedecimpunctata_594 | 0.170 |
| Psyllobora_renifer_552 | Tytthaspis_sedecimpunctata_594 | 0.170 |
| Psyllobora_renifer_553 | Tytthaspis_sedecimpunctata_594 | 0.170 |

| | | |
|---|---|---|
| Psyllobora_renifer_554 | Tytthaspis_sedecimpunctata_594 | 0.170 |
| Adalia_bipunctata_102 | Tytthaspis_sedecimpunctata_595 | 0.170 |
| Adalia_bipunctata_103 | Tytthaspis_sedecimpunctata_595 | 0.170 |
| Adalia_bipunctata_106 | Tytthaspis_sedecimpunctata_595 | 0.170 |
| Adalia_bipunctata_107 | Tytthaspis_sedecimpunctata_595 | 0.170 |
| Adalia_bipunctata_108 | Tytthaspis_sedecimpunctata_595 | 0.170 |
| Adalia_bipunctata_109 | Tytthaspis_sedecimpunctata_595 | 0.170 |
| Psyllobora_renifer_551 | Tytthaspis_sedecimpunctata_595 | 0.170 |
| Psyllobora_renifer_552 | Tytthaspis_sedecimpunctata_595 | 0.170 |
| Psyllobora_renifer_553 | Tytthaspis_sedecimpunctata_595 | 0.170 |
| Psyllobora_renifer_554 | Tytthaspis_sedecimpunctata_595 | 0.170 |
| Adalia_bipunctata_101 | Tytthaspis_sedecimpunctata_580 | 0.170 |
| Adalia_bipunctata_104 | Tytthaspis_sedecimpunctata_580 | 0.170 |
| Coccinella_trifasciata_274 | Tytthaspis_sedecimpunctata_580 | 0.170 |
| Coccinella_trifasciata_273 | Tytthaspis_sedecimpunctata_583 | 0.170 |
| Psyllobora_renifer_555 | Tytthaspis_sedecimpunctata_583 | 0.170 |
| Coccinella_quinquepunctata_235 | Tytthaspis_sedecimpunctata_584 | 0.170 |
| Coccinella_quinquepunctata_238 | Tytthaspis_sedecimpunctata_584 | 0.170 |
| Coccinella_quinquepunctata_240 | Tytthaspis_sedecimpunctata_584 | 0.170 |
| Harmonia_axyridis_362 | Tytthaspis_sedecimpunctata_584 | 0.170 |
| Anatis_ocellata_143 | Tytthaspis_sedecimpunctata_588 | 0.170 |
| Coccinella_quinquepunctata_232 | Tytthaspis_sedecimpunctata_588 | 0.170 |
| Hyperaspis_reppensis_664 | Scymnus_suturalis_841 | 0.170 |
| Hyperaspis_reppensis_664 | Scymnus_suturalis_842 | 0.170 |
| Hyperaspis_reppensis_665 | Scymnus_suturalis_843 | 0.170 |
| Hyperaspis_reppensis_666 | Scymnus_suturalis_843 | 0.170 |
| Hyperaspis_reppensis_664 | Scymnus_suturalis_844 | 0.170 |
| Hyperaspis_reppensis_665 | Scymnus_suturalis_845 | 0.170 |
| Hyperaspis_reppensis_666 | Scymnus_suturalis_845 | 0.170 |
| Hyperaspis_reppensis_664 | Scymnus_suturalis_846 | 0.170 |
| Hyperaspis_reppensis_664 | Scymnus_suturalis_847 | 0.170 |
| Hyperaspis_reppensis_664 | Scymnus_suturalis_848 | 0.170 |
| Anatis_ocellata_143 | Tytthaspis_sedecimpunctata_577 | 0.170 |
| Coccinella_quinquepunctata_235 | Tytthaspis_sedecimpunctata_577 | 0.170 |
| Coccinella_quinquepunctata_238 | Tytthaspis_sedecimpunctata_577 | 0.170 |
| Coccinella_quinquepunctata_240 | Tytthaspis_sedecimpunctata_577 | 0.170 |
| Hippodamia_alpina_383 | Tytthaspis_sedecimpunctata_577 | 0.170 |
| Anatis_ocellata_143 | Tytthaspis_sedecimpunctata_578 | 0.170 |
| Coccinella_quinquepunctata_235 | Tytthaspis_sedecimpunctata_578 | 0.170 |
| Coccinella_quinquepunctata_238 | Tytthaspis_sedecimpunctata_578 | 0.170 |
| Coccinella_quinquepunctata_240 | Tytthaspis_sedecimpunctata_578 | 0.170 |
| Hippodamia_alpina_383 | Tytthaspis_sedecimpunctata_578 | 0.170 |
| Anatis_ocellata_143 | Tytthaspis_sedecimpunctata_579 | 0.170 |
| Coccinella_quinquepunctata_235 | Tytthaspis_sedecimpunctata_579 | 0.170 |
| Coccinella_quinquepunctata_238 | Tytthaspis_sedecimpunctata_579 | 0.170 |
| Coccinella_quinquepunctata_240 | Tytthaspis_sedecimpunctata_579 | 0.170 |
| Hippodamia_alpina_383 | Tytthaspis_sedecimpunctata_579 | 0.170 |
| Anatis_ocellata_143 | Tytthaspis_sedecimpunctata_581 | 0.170 |
| Coccinella_quinquepunctata_235 | Tytthaspis_sedecimpunctata_581 | 0.170 |
| Coccinella_quinquepunctata_238 | Tytthaspis_sedecimpunctata_581 | 0.170 |
| Coccinella_quinquepunctata_240 | Tytthaspis_sedecimpunctata_581 | 0.170 |
| Hippodamia_alpina_383 | Tytthaspis_sedecimpunctata_581 | 0.170 |
| Anatis_ocellata_143 | Tytthaspis_sedecimpunctata_582 | 0.170 |
| Coccinella_quinquepunctata_235 | Tytthaspis_sedecimpunctata_582 | 0.170 |
| Coccinella_quinquepunctata_238 | Tytthaspis_sedecimpunctata_582 | 0.170 |
| Coccinella_quinquepunctata_240 | Tytthaspis_sedecimpunctata_582 | 0.170 |

| | | |
|---|---|---|
| Hippodamia_alpina_383 | Tytthaspis_sedecimpunctata_582 | 0.170 |
| Hippodamia_alpina_384 | Tytthaspis_sedecimpunctata_584 | 0.170 |
| Psyllobora_renifer_555 | Tytthaspis_sedecimpunctata_584 | 0.170 |
| Coccinella_quinquepunctata_235 | Tytthaspis_sedecimpunctata_585 | 0.170 |
| Coccinella_quinquepunctata_238 | Tytthaspis_sedecimpunctata_585 | 0.170 |
| Coccinella_quinquepunctata_240 | Tytthaspis_sedecimpunctata_585 | 0.170 |
| Hippodamia_alpina_383 | Tytthaspis_sedecimpunctata_585 | 0.170 |
| Psyllobora_vigintimaculata_575 | Tytthaspis_sedecimpunctata_585 | 0.170 |
| Psyllobora_vigintimaculata_576 | Tytthaspis_sedecimpunctata_585 | 0.170 |
| Psyllobora_vigintimaculata_573 | Tytthaspis_sedecimpunctata_587 | 0.170 |
| Psyllobora_renifer_555 | Tytthaspis_sedecimpunctata_588 | 0.170 |
| Psyllobora_vigintiduopunctata_571 | Tytthaspis_sedecimpunctata_588 | 0.170 |
| Hippodamia_alpina_388 | Tytthaspis_sedecimpunctata_589 | 0.170 |
| Illeis_galbula_474 | Tytthaspis_sedecimpunctata_589 | 0.170 |
| Illeis_galbula_475 | Tytthaspis_sedecimpunctata_589 | 0.170 |
| Illeis_galbula_476 | Tytthaspis_sedecimpunctata_589 | 0.170 |
| Anatis_ocellata_143 | Tytthaspis_sedecimpunctata_590 | 0.170 |
| Coccinella_quinquepunctata_235 | Tytthaspis_sedecimpunctata_590 | 0.170 |
| Coccinella_quinquepunctata_238 | Tytthaspis_sedecimpunctata_590 | 0.170 |
| Coccinella_quinquepunctata_240 | Tytthaspis_sedecimpunctata_590 | 0.170 |
| Hippodamia_alpina_383 | Tytthaspis_sedecimpunctata_590 | 0.170 |
| Coccinella_quinquepunctata_235 | Tytthaspis_sedecimpunctata_592 | 0.170 |
| Coccinella_quinquepunctata_238 | Tytthaspis_sedecimpunctata_592 | 0.170 |
| Coccinella_quinquepunctata_240 | Tytthaspis_sedecimpunctata_592 | 0.170 |
| Hippodamia_alpina_383 | Tytthaspis_sedecimpunctata_592 | 0.170 |
| Hippodamia_alpina_383 | Tytthaspis_sedecimpunctata_593 | 0.170 |
| Anatis_ocellata_143 | Tytthaspis_sedecimpunctata_594 | 0.170 |
| Coccinella_quinquepunctata_235 | Tytthaspis_sedecimpunctata_594 | 0.170 |
| Coccinella_quinquepunctata_238 | Tytthaspis_sedecimpunctata_594 | 0.170 |
| Coccinella_quinquepunctata_240 | Tytthaspis_sedecimpunctata_594 | 0.170 |
| Hippodamia_alpina_383 | Tytthaspis_sedecimpunctata_594 | 0.170 |
| Anatis_ocellata_143 | Tytthaspis_sedecimpunctata_595 | 0.170 |
| Coccinella_quinquepunctata_235 | Tytthaspis_sedecimpunctata_595 | 0.170 |
| Coccinella_quinquepunctata_238 | Tytthaspis_sedecimpunctata_595 | 0.170 |
| Coccinella_quinquepunctata_240 | Tytthaspis_sedecimpunctata_595 | 0.170 |
| Hippodamia_alpina_383 | Tytthaspis_sedecimpunctata_595 | 0.170 |
| Didion_punctatum_646 | Scymnus_rubromaculatus_822 | 0.170 |
| Didion_punctatum_646 | Scymnus_rubromaculatus_823 | 0.170 |
| Didion_punctatum_646 | Scymnus_rubromaculatus_824 | 0.170 |
| Didion_punctatum_646 | Scymnus_rubromaculatus_825 | 0.170 |
| Didion_punctatum_646 | Scymnus_rubromaculatus_826 | 0.170 |
| Didion_punctatum_646 | Scymnus_rubromaculatus_827 | 0.170 |
| Hyperaspis_reppensis_664 | Scymnus_suturalis_832 | 0.170 |
| Hyperaspis_reppensis_664 | Scymnus_suturalis_835 | 0.170 |
| Hyperaspis_reppensis_664 | Scymnus_suturalis_836 | 0.170 |
| Hyperaspis_reppensis_665 | Scymnus_suturalis_837 | 0.170 |
| Hyperaspis_reppensis_666 | Scymnus_suturalis_837 | 0.170 |
| Scymnus_apetzi_690 | Scymnus_suturalis_838 | 0.170 |
| Scymnus_apetzi_697 | Scymnus_suturalis_838 | 0.170 |
| Hyperaspis_reppensis_664 | Scymnus_suturalis_839 | 0.170 |
| Scymnus_apetzi_690 | Scymnus_suturalis_840 | 0.170 |
| Scymnus_apetzi_697 | Scymnus_suturalis_840 | 0.170 |
| Hyperaspis_proba_660 | Tytthaspis_sedecimpunctata_583 | 0.170 |
| Mulsantina_picta_484 | Tytthaspis_sedecimpunctata_577 | 0.170 |
| Psyllobora_vigintiduopunctata_571 | Tytthaspis_sedecimpunctata_577 | 0.170 |
| Mulsantina_picta_484 | Tytthaspis_sedecimpunctata_578 | 0.170 |

Supplementary Table S4 (continued)

| | | |
|---|---|---|
| Psyllobora_vigintiduopunctata_571 | Tytthaspis_sedecimpunctata_578 | 0.170 |
| Mulsantina_picta_484 | Tytthaspis_sedecimpunctata_579 | 0.170 |
| Psyllobora_vigintiduopunctata_571 | Tytthaspis_sedecimpunctata_579 | 0.170 |
| Coccinella_quinquepunctata_235 | Tytthaspis_sedecimpunctata_580 | 0.170 |
| Coccinella_quinquepunctata_238 | Tytthaspis_sedecimpunctata_580 | 0.170 |
| Coccinella_quinquepunctata_240 | Tytthaspis_sedecimpunctata_580 | 0.170 |
| Hippodamia_alpina_388 | Tytthaspis_sedecimpunctata_580 | 0.170 |
| Illeis_galbula_474 | Tytthaspis_sedecimpunctata_580 | 0.170 |
| Illeis_galbula_475 | Tytthaspis_sedecimpunctata_580 | 0.170 |
| Illeis_galbula_476 | Tytthaspis_sedecimpunctata_580 | 0.170 |
| Mulsantina_picta_484 | Tytthaspis_sedecimpunctata_581 | 0.170 |
| Psyllobora_vigintiduopunctata_571 | Tytthaspis_sedecimpunctata_581 | 0.170 |
| Mulsantina_picta_484 | Tytthaspis_sedecimpunctata_582 | 0.170 |
| Psyllobora_vigintiduopunctata_571 | Tytthaspis_sedecimpunctata_582 | 0.170 |
| Hippodamia_alpina_388 | Tytthaspis_sedecimpunctata_587 | 0.170 |
| Psyllobora_vigintimaculata_574 | Tytthaspis_sedecimpunctata_587 | 0.170 |
| Scymnus_auritus_699 | Tytthaspis_sedecimpunctata_587 | 0.170 |
| Illeis_galbula_474 | Tytthaspis_sedecimpunctata_588 | 0.170 |
| Illeis_galbula_475 | Tytthaspis_sedecimpunctata_588 | 0.170 |
| Illeis_galbula_476 | Tytthaspis_sedecimpunctata_588 | 0.170 |
| Mulsantina_picta_484 | Tytthaspis_sedecimpunctata_590 | 0.170 |
| Psyllobora_vigintiduopunctata_571 | Tytthaspis_sedecimpunctata_590 | 0.170 |
| Mulsantina_picta_484 | Tytthaspis_sedecimpunctata_592 | 0.170 |
| Mulsantina_picta_484 | Tytthaspis_sedecimpunctata_593 | 0.170 |
| Psyllobora_vigintiduopunctata_571 | Tytthaspis_sedecimpunctata_593 | 0.170 |
| Mulsantina_picta_484 | Tytthaspis_sedecimpunctata_594 | 0.170 |
| Psyllobora_vigintiduopunctata_571 | Tytthaspis_sedecimpunctata_594 | 0.170 |
| Mulsantina_picta_484 | Tytthaspis_sedecimpunctata_595 | 0.170 |
| Psyllobora_vigintiduopunctata_571 | Tytthaspis_sedecimpunctata_595 | 0.170 |
| Didion_punctatum_644 | Scymnus_rubromaculatus_822 | 0.170 |
| Didion_punctatum_644 | Scymnus_rubromaculatus_823 | 0.170 |
| Didion_punctatum_644 | Scymnus_rubromaculatus_824 | 0.170 |
| Didion_punctatum_644 | Scymnus_rubromaculatus_825 | 0.170 |
| Didion_punctatum_644 | Scymnus_rubromaculatus_826 | 0.170 |
| Didion_punctatum_644 | Scymnus_rubromaculatus_827 | 0.170 |
| Brumus_quadripustulatus_15 | Scymnus_suturalis_833 | 0.170 |
| Brumus_quadripustulatus_16 | Scymnus_suturalis_833 | 0.170 |
| Brumus_quadripustulatus_6 | Scymnus_suturalis_833 | 0.170 |
| Brumus_quadripustulatus_8 | Scymnus_suturalis_833 | 0.170 |
| Scymnus_apetzi_690 | Scymnus_suturalis_834 | 0.170 |
| Scymnus_apetzi_697 | Scymnus_suturalis_834 | 0.170 |
| Chilocorus_bipustulatus_21 | Tytthaspis_sedecimpunctata_591 | 0.170 |
| Chilocorus_nigritus_28 | Scymnus_suturalis_838 | 0.170 |
| Chilocorus_nigritus_28 | Scymnus_suturalis_840 | 0.170 |
| Chilocorus_nigritus_28 | Scymnus_suturalis_834 | 0.170 |
| Chilocorus_nigritus_28 | Scymnus_suturalis_837 | 0.170 |
| Brumus_quadripustulatus_15 | Scymnus_suturalis_843 | 0.170 |
| Brumus_quadripustulatus_16 | Scymnus_suturalis_843 | 0.170 |
| Brumus_quadripustulatus_6 | Scymnus_suturalis_843 | 0.170 |
| Brumus_quadripustulatus_8 | Scymnus_suturalis_843 | 0.170 |
| Brumus_quadripustulatus_15 | Scymnus_suturalis_845 | 0.170 |
| Brumus_quadripustulatus_16 | Scymnus_suturalis_845 | 0.170 |
| Brumus_quadripustulatus_6 | Scymnus_suturalis_845 | 0.170 |
| Brumus_quadripustulatus_8 | Scymnus_suturalis_845 | 0.170 |
| Psyllobora_vigintiduopunctata_571 | Tytthaspis_sedecimpunctata_580 | 0.170 |
| Scymnus_auritus_699 | Tytthaspis_sedecimpunctata_583 | 0.170 |

| | | |
|---|---|---|
| Scymnus_auritus_699 | Tytthaspis_sedecimpunctata_585 | 0.170 |
| Scymnus_haemorrhoidalis_749 | Tytthaspis_sedecimpunctata_586 | 0.170 |
| Scymnus_haemorrhoidalis_750 | Tytthaspis_sedecimpunctata_588 | 0.170 |
| Scymnus_haemorrhoidalis_751 | Tytthaspis_sedecimpunctata_588 | 0.170 |
| Scymnus_haemorrhoidalis_754 | Tytthaspis_sedecimpunctata_588 | 0.170 |
| Scymnus_haemorrhoidalis_757 | Tytthaspis_sedecimpunctata_588 | 0.170 |
| Scymnus_haemorrhoidalis_758 | Tytthaspis_sedecimpunctata_588 | 0.170 |
| Hippodamia_glacialis_408 | Tytthaspis_sedecimpunctata_589 | 0.170 |
| Hippodamia_glacialis_409 | Tytthaspis_sedecimpunctata_589 | 0.170 |
| Psyllobora_vigintiduopunctata_564 | Tytthaspis_sedecimpunctata_591 | 0.170 |
| Scymnus_auritus_699 | Tytthaspis_sedecimpunctata_592 | 0.170 |
| Nephus_quadrimaculatus_669 | Scymnus_suturalis_843 | 0.170 |
| Nephus_quadrimaculatus_670 | Scymnus_suturalis_843 | 0.170 |
| Nephus_quadrimaculatus_671 | Scymnus_suturalis_843 | 0.170 |
| Nephus_quadrimaculatus_672 | Scymnus_suturalis_843 | 0.170 |
| Nephus_quadrimaculatus_673 | Scymnus_suturalis_843 | 0.170 |
| Nephus_quadrimaculatus_669 | Scymnus_suturalis_845 | 0.170 |
| Nephus_quadrimaculatus_670 | Scymnus_suturalis_845 | 0.170 |
| Nephus_quadrimaculatus_671 | Scymnus_suturalis_845 | 0.170 |
| Nephus_quadrimaculatus_672 | Scymnus_suturalis_845 | 0.170 |
| Nephus_quadrimaculatus_673 | Scymnus_suturalis_845 | 0.170 |
| Hippodamia_notata_415 | Tytthaspis_sedecimpunctata_577 | 0.170 |
| Psyllobora_vigintiduopunctata_560 | Tytthaspis_sedecimpunctata_577 | 0.170 |
| Hippodamia_notata_415 | Tytthaspis_sedecimpunctata_578 | 0.170 |
| Psyllobora_vigintiduopunctata_560 | Tytthaspis_sedecimpunctata_578 | 0.170 |
| Hippodamia_notata_415 | Tytthaspis_sedecimpunctata_579 | 0.170 |
| Psyllobora_vigintiduopunctata_560 | Tytthaspis_sedecimpunctata_579 | 0.170 |
| Hippodamia_notata_422 | Tytthaspis_sedecimpunctata_580 | 0.170 |
| Hippodamia_notata_423 | Tytthaspis_sedecimpunctata_580 | 0.170 |
| Hippodamia_notata_424 | Tytthaspis_sedecimpunctata_580 | 0.170 |
| Hippodamia_notata_426 | Tytthaspis_sedecimpunctata_580 | 0.170 |
| Hippodamia_notata_427 | Tytthaspis_sedecimpunctata_580 | 0.170 |
| Scymnus_auritus_699 | Tytthaspis_sedecimpunctata_580 | 0.170 |
| Hippodamia_notata_415 | Tytthaspis_sedecimpunctata_581 | 0.170 |
| Psyllobora_vigintiduopunctata_560 | Tytthaspis_sedecimpunctata_581 | 0.170 |
| Hippodamia_notata_415 | Tytthaspis_sedecimpunctata_582 | 0.170 |
| Psyllobora_vigintiduopunctata_560 | Tytthaspis_sedecimpunctata_582 | 0.170 |
| Hippodamia_glacialis_407 | Tytthaspis_sedecimpunctata_587 | 0.170 |
| Scymnus_haemorrhoidalis_755 | Tytthaspis_sedecimpunctata_588 | 0.170 |
| Hippodamia_notata_415 | Tytthaspis_sedecimpunctata_590 | 0.170 |
| Psyllobora_vigintiduopunctata_560 | Tytthaspis_sedecimpunctata_590 | 0.170 |
| Hippodamia_notata_415 | Tytthaspis_sedecimpunctata_592 | 0.170 |
| Hippodamia_notata_415 | Tytthaspis_sedecimpunctata_593 | 0.170 |
| Psyllobora_vigintiduopunctata_560 | Tytthaspis_sedecimpunctata_593 | 0.170 |
| Psyllobora_vigintiduopunctata_561 | Tytthaspis_sedecimpunctata_593 | 0.170 |
| Psyllobora_vigintiduopunctata_562 | Tytthaspis_sedecimpunctata_593 | 0.170 |
| Psyllobora_vigintiduopunctata_565 | Tytthaspis_sedecimpunctata_593 | 0.170 |
| Psyllobora_vigintiduopunctata_567 | Tytthaspis_sedecimpunctata_593 | 0.170 |
| Hippodamia_notata_415 | Tytthaspis_sedecimpunctata_594 | 0.170 |
| Psyllobora_vigintiduopunctata_560 | Tytthaspis_sedecimpunctata_594 | 0.170 |
| Hippodamia_notata_415 | Tytthaspis_sedecimpunctata_595 | 0.170 |
| Psyllobora_vigintiduopunctata_560 | Tytthaspis_sedecimpunctata_595 | 0.170 |
| Nephus_quadrimaculatus_669 | Scymnus_suturalis_834 | 0.170 |
| Nephus_quadrimaculatus_670 | Scymnus_suturalis_834 | 0.170 |
| Nephus_quadrimaculatus_671 | Scymnus_suturalis_834 | 0.170 |
| Nephus_quadrimaculatus_672 | Scymnus_suturalis_834 | 0.170 |

| | | |
|---|---|---|
| Nephus_quadrimaculatus_673 | Scymnus_suturalis_834 | 0.170 |
| Scymnus_apetzi_695 | Scymnus_sinuanodulus_828 | 0.170 |
| Hyperaspis_reppensis_664 | Subcoccinella_vigintiquatuorpunctata_622 | 0.170 |
| Hyperaspis_reppensis_666 | Subcoccinella_vigintiquatuorpunctata_622 | 0.170 |
| Hyperaspis_reppensis_664 | Subcoccinella_vigintiquatuorpunctata_627 | 0.170 |
| Hyperaspis_reppensis_666 | Subcoccinella_vigintiquatuorpunctata_627 | 0.170 |
| Hyperaspis_reppensis_667 | Subcoccinella_vigintiquatuorpunctata_627 | 0.170 |
| Hyperaspis_reppensis_665 | Subcoccinella_vigintiquatuorpunctata_630 | 0.170 |
| Hippodamia_glacialis_407 | Tytthaspis_sedecimpunctata_577 | 0.170 |
| Hippodamia_notata_413 | Tytthaspis_sedecimpunctata_577 | 0.170 |
| Hippodamia_notata_418 | Tytthaspis_sedecimpunctata_577 | 0.170 |
| Hippodamia_glacialis_407 | Tytthaspis_sedecimpunctata_578 | 0.170 |
| Hippodamia_notata_413 | Tytthaspis_sedecimpunctata_578 | 0.170 |
| Hippodamia_notata_418 | Tytthaspis_sedecimpunctata_578 | 0.170 |
| Hippodamia_glacialis_407 | Tytthaspis_sedecimpunctata_579 | 0.170 |
| Hippodamia_notata_413 | Tytthaspis_sedecimpunctata_579 | 0.170 |
| Hippodamia_notata_418 | Tytthaspis_sedecimpunctata_579 | 0.170 |
| Hippodamia_notata_412 | Tytthaspis_sedecimpunctata_580 | 0.170 |
| Hippodamia_notata_414 | Tytthaspis_sedecimpunctata_580 | 0.170 |
| Hippodamia_notata_416 | Tytthaspis_sedecimpunctata_580 | 0.170 |
| Hippodamia_notata_417 | Tytthaspis_sedecimpunctata_580 | 0.170 |
| Hippodamia_notata_420 | Tytthaspis_sedecimpunctata_580 | 0.170 |
| Hippodamia_notata_425 | Tytthaspis_sedecimpunctata_580 | 0.170 |
| Hippodamia_notata_428 | Tytthaspis_sedecimpunctata_580 | 0.170 |
| Hippodamia_notata_430 | Tytthaspis_sedecimpunctata_580 | 0.170 |
| Hippodamia_glacialis_407 | Tytthaspis_sedecimpunctata_581 | 0.170 |
| Hippodamia_notata_413 | Tytthaspis_sedecimpunctata_581 | 0.170 |
| Hippodamia_notata_418 | Tytthaspis_sedecimpunctata_581 | 0.170 |
| Hippodamia_glacialis_407 | Tytthaspis_sedecimpunctata_582 | 0.170 |
| Hippodamia_notata_413 | Tytthaspis_sedecimpunctata_582 | 0.170 |
| Hippodamia_notata_418 | Tytthaspis_sedecimpunctata_582 | 0.170 |
| Hippodamia_glacialis_408 | Tytthaspis_sedecimpunctata_583 | 0.170 |
| Hippodamia_glacialis_409 | Tytthaspis_sedecimpunctata_583 | 0.170 |
| Psyllobora_vigintiduopunctata_570 | Tytthaspis_sedecimpunctata_584 | 0.170 |
| Psyllobora_vigintiduopunctata_572 | Tytthaspis_sedecimpunctata_584 | 0.170 |
| Hippodamia_glacialis_408 | Tytthaspis_sedecimpunctata_585 | 0.170 |
| Hippodamia_glacialis_409 | Tytthaspis_sedecimpunctata_585 | 0.170 |
| Hippodamia_notata_413 | Tytthaspis_sedecimpunctata_585 | 0.170 |
| Hippodamia_notata_418 | Tytthaspis_sedecimpunctata_585 | 0.170 |
| Hippodamia_tredecimpunctata_459 | Tytthaspis_sedecimpunctata_586 | 0.170 |
| Ceratomegilla_ulkei_220 | Tytthaspis_sedecimpunctata_588 | 0.170 |
| Hippodamia_glacialis_407 | Tytthaspis_sedecimpunctata_590 | 0.170 |
| Hippodamia_notata_413 | Tytthaspis_sedecimpunctata_590 | 0.170 |
| Hippodamia_notata_418 | Tytthaspis_sedecimpunctata_590 | 0.170 |
| Hippodamia_glacialis_408 | Tytthaspis_sedecimpunctata_592 | 0.170 |
| Hippodamia_glacialis_409 | Tytthaspis_sedecimpunctata_592 | 0.170 |
| Hippodamia_notata_413 | Tytthaspis_sedecimpunctata_592 | 0.170 |
| Hippodamia_notata_418 | Tytthaspis_sedecimpunctata_592 | 0.170 |
| Hippodamia_glacialis_407 | Tytthaspis_sedecimpunctata_593 | 0.170 |
| Hippodamia_notata_419 | Tytthaspis_sedecimpunctata_593 | 0.170 |
| Hippodamia_notata_429 | Tytthaspis_sedecimpunctata_593 | 0.170 |
| Hippodamia_notata_431 | Tytthaspis_sedecimpunctata_593 | 0.170 |
| Hippodamia_glacialis_407 | Tytthaspis_sedecimpunctata_594 | 0.170 |
| Hippodamia_notata_413 | Tytthaspis_sedecimpunctata_594 | 0.170 |
| Hippodamia_notata_418 | Tytthaspis_sedecimpunctata_594 | 0.170 |
| Hippodamia_glacialis_407 | Tytthaspis_sedecimpunctata_595 | 0.170 |

| | | |
|---|---|---|
| Hippodamia_notata_413 | Tytthaspis_sedecimpunctata_595 | 0.170 |
| Hippodamia_notata_418 | Tytthaspis_sedecimpunctata_595 | 0.170 |
| Nephus_quadrimaculatus_669 | Scymnus_suturalis_840 | 0.170 |
| Nephus_quadrimaculatus_670 | Scymnus_suturalis_840 | 0.170 |
| Nephus_quadrimaculatus_671 | Scymnus_suturalis_840 | 0.170 |
| Nephus_quadrimaculatus_672 | Scymnus_suturalis_840 | 0.170 |
| Nephus_quadrimaculatus_673 | Scymnus_suturalis_840 | 0.170 |
| Ceratomegilla_ulkei_219 | Tytthaspis_sedecimpunctata_577 | 0.170 |
| Ceratomegilla_ulkei_219 | Tytthaspis_sedecimpunctata_578 | 0.170 |
| Ceratomegilla_ulkei_219 | Tytthaspis_sedecimpunctata_579 | 0.170 |
| Ceratomegilla_ulkei_219 | Tytthaspis_sedecimpunctata_581 | 0.170 |
| Ceratomegilla_ulkei_219 | Tytthaspis_sedecimpunctata_582 | 0.170 |
| Ceratomegilla_ulkei_221 | Tytthaspis_sedecimpunctata_585 | 0.170 |
| Hippodamia_convergens_396 | Tytthaspis_sedecimpunctata_586 | 0.170 |
| Hippodamia_convergens_400 | Tytthaspis_sedecimpunctata_586 | 0.170 |
| Hippodamia_convergens_403 | Tytthaspis_sedecimpunctata_586 | 0.170 |
| Hippodamia_moesta_410 | Tytthaspis_sedecimpunctata_586 | 0.170 |
| Hippodamia_caseyi_391 | Tytthaspis_sedecimpunctata_588 | 0.170 |
| Hippodamia_convergens_393 | Tytthaspis_sedecimpunctata_588 | 0.170 |
| Hippodamia_convergens_394 | Tytthaspis_sedecimpunctata_588 | 0.170 |
| Hippodamia_convergens_397 | Tytthaspis_sedecimpunctata_588 | 0.170 |
| Hippodamia_convergens_398 | Tytthaspis_sedecimpunctata_588 | 0.170 |
| Hippodamia_convergens_399 | Tytthaspis_sedecimpunctata_588 | 0.170 |
| Hippodamia_convergens_401 | Tytthaspis_sedecimpunctata_588 | 0.170 |
| Hippodamia_convergens_402 | Tytthaspis_sedecimpunctata_588 | 0.170 |
| Hippodamia_convergens_404 | Tytthaspis_sedecimpunctata_588 | 0.170 |
| Hippodamia_convergens_405 | Tytthaspis_sedecimpunctata_588 | 0.170 |
| Hippodamia_convergens_406 | Tytthaspis_sedecimpunctata_588 | 0.170 |
| Hippodamia_moesta_411 | Tytthaspis_sedecimpunctata_588 | 0.170 |
| Hippodamia_quinquesignata_443 | Tytthaspis_sedecimpunctata_588 | 0.170 |
| Hippodamia_quinquesignata_444 | Tytthaspis_sedecimpunctata_588 | 0.170 |
| Hippodamia_quinquesignata_445 | Tytthaspis_sedecimpunctata_588 | 0.170 |
| Ceratomegilla_ulkei_220 | Tytthaspis_sedecimpunctata_589 | 0.170 |
| Hippodamia_tredecimpunctata_447 | Tytthaspis_sedecimpunctata_589 | 0.170 |
| Hippodamia_tredecimpunctata_451 | Tytthaspis_sedecimpunctata_589 | 0.170 |
| Hippodamia_tredecimpunctata_454 | Tytthaspis_sedecimpunctata_589 | 0.170 |
| Hippodamia_tredecimpunctata_455 | Tytthaspis_sedecimpunctata_589 | 0.170 |
| Hippodamia_tredecimpunctata_456 | Tytthaspis_sedecimpunctata_589 | 0.170 |
| Ceratomegilla_ulkei_219 | Tytthaspis_sedecimpunctata_590 | 0.170 |
| Hippodamia_notata_419 | Tytthaspis_sedecimpunctata_591 | 0.170 |
| Hippodamia_notata_429 | Tytthaspis_sedecimpunctata_591 | 0.170 |
| Hippodamia_notata_431 | Tytthaspis_sedecimpunctata_591 | 0.170 |
| Ceratomegilla_ulkei_219 | Tytthaspis_sedecimpunctata_592 | 0.170 |
| Ceratomegilla_ulkei_221 | Tytthaspis_sedecimpunctata_593 | 0.170 |
| Ceratomegilla_ulkei_219 | Tytthaspis_sedecimpunctata_594 | 0.170 |
| Ceratomegilla_ulkei_219 | Tytthaspis_sedecimpunctata_595 | 0.170 |
| Psyllobora_vigintiduopunctata_559 | Tytthaspis_sedecimpunctata_591 | 0.171 |
| Diomus_terminatus_647 | Scymnus_suturalis_843 | 0.171 |
| Diomus_terminatus_648 | Scymnus_suturalis_843 | 0.171 |
| Diomus_terminatus_647 | Scymnus_suturalis_845 | 0.171 |
| Diomus_terminatus_648 | Scymnus_suturalis_845 | 0.171 |
| Hippodamia_caseyi_392 | Tytthaspis_sedecimpunctata_583 | 0.171 |
| Hippodamia_convergens_395 | Tytthaspis_sedecimpunctata_583 | 0.171 |
| Hippodamia_caseyi_392 | Tytthaspis_sedecimpunctata_585 | 0.171 |
| Hippodamia_convergens_395 | Tytthaspis_sedecimpunctata_585 | 0.171 |
| Oenopia_conglobata_523 | Tytthaspis_sedecimpunctata_587 | 0.171 |

| | | |
|---|---|---|
| Coccinula_quatuordecimpustulata_297 | Tytthaspis_sedecimpunctata_588 | 0.171 |
| Scymnus_ningshanensis_789 | Scymnus_rubromaculatus_822 | 0.171 |
| Scymnus_ningshanensis_790 | Scymnus_rubromaculatus_822 | 0.171 |
| Scymnus_ningshanensis_789 | Scymnus_rubromaculatus_823 | 0.171 |
| Scymnus_ningshanensis_790 | Scymnus_rubromaculatus_823 | 0.171 |
| Scymnus_ningshanensis_789 | Scymnus_rubromaculatus_824 | 0.171 |
| Scymnus_ningshanensis_790 | Scymnus_rubromaculatus_824 | 0.171 |
| Scymnus_ningshanensis_789 | Scymnus_rubromaculatus_825 | 0.171 |
| Scymnus_ningshanensis_790 | Scymnus_rubromaculatus_825 | 0.171 |
| Scymnus_ningshanensis_789 | Scymnus_rubromaculatus_826 | 0.171 |
| Scymnus_ningshanensis_790 | Scymnus_rubromaculatus_826 | 0.171 |
| Scymnus_ningshanensis_789 | Scymnus_rubromaculatus_827 | 0.171 |
| Scymnus_ningshanensis_790 | Scymnus_rubromaculatus_827 | 0.171 |
| Scymnus_interruptus_759 | Scymnus_sinuanodulus_828 | 0.171 |
| Scymnus_interruptus_761 | Scymnus_sinuanodulus_828 | 0.171 |
| Scymnus_interruptus_763 | Scymnus_sinuanodulus_828 | 0.171 |
| Scymnus_interruptus_764 | Scymnus_sinuanodulus_828 | 0.171 |
| Scymnus_interruptus_766 | Scymnus_sinuanodulus_828 | 0.171 |
| Scymnus_interruptus_769 | Scymnus_sinuanodulus_828 | 0.171 |
| Diomus_terminatus_647 | Scymnus_suturalis_834 | 0.171 |
| Diomus_terminatus_648 | Scymnus_suturalis_834 | 0.171 |
| Scymnus_camptodromus_717 | Tytthaspis_sedecimpunctata_589 | 0.171 |
| Hyperaspis_proba_660 | Scymnus_suturalis_833 | 0.171 |
| Hippodamia_caseyi_392 | Tytthaspis_sedecimpunctata_584 | 0.171 |
| Hippodamia_convergens_395 | Tytthaspis_sedecimpunctata_584 | 0.171 |
| Hippodamia_caseyi_391 | Tytthaspis_sedecimpunctata_591 | 0.171 |
| Hippodamia_convergens_393 | Tytthaspis_sedecimpunctata_591 | 0.171 |
| Hippodamia_convergens_394 | Tytthaspis_sedecimpunctata_591 | 0.171 |
| Hippodamia_convergens_397 | Tytthaspis_sedecimpunctata_591 | 0.171 |
| Hippodamia_convergens_398 | Tytthaspis_sedecimpunctata_591 | 0.171 |
| Hippodamia_convergens_399 | Tytthaspis_sedecimpunctata_591 | 0.171 |
| Hippodamia_convergens_401 | Tytthaspis_sedecimpunctata_591 | 0.171 |
| Hippodamia_convergens_402 | Tytthaspis_sedecimpunctata_591 | 0.171 |
| Hippodamia_convergens_404 | Tytthaspis_sedecimpunctata_591 | 0.171 |
| Hippodamia_convergens_405 | Tytthaspis_sedecimpunctata_591 | 0.171 |
| Hippodamia_convergens_406 | Tytthaspis_sedecimpunctata_591 | 0.171 |
| Hippodamia_moesta_411 | Tytthaspis_sedecimpunctata_591 | 0.171 |
| Hippodamia_quinquesignata_443 | Tytthaspis_sedecimpunctata_591 | 0.171 |
| Hippodamia_quinquesignata_444 | Tytthaspis_sedecimpunctata_591 | 0.171 |
| Hippodamia_quinquesignata_445 | Tytthaspis_sedecimpunctata_591 | 0.171 |
| Calvia_decemguttata_195 | Tytthaspis_sedecimpunctata_593 | 0.171 |
| Calvia_decemguttata_199 | Tytthaspis_sedecimpunctata_593 | 0.171 |
| Calvia_decemguttata_200 | Tytthaspis_sedecimpunctata_593 | 0.171 |
| Hippodamia_tredecimpunctata_447 | Tytthaspis_sedecimpunctata_593 | 0.171 |
| Hippodamia_tredecimpunctata_451 | Tytthaspis_sedecimpunctata_593 | 0.171 |
| Hippodamia_tredecimpunctata_454 | Tytthaspis_sedecimpunctata_593 | 0.171 |
| Hippodamia_tredecimpunctata_455 | Tytthaspis_sedecimpunctata_593 | 0.171 |
| Hippodamia_tredecimpunctata_456 | Tytthaspis_sedecimpunctata_593 | 0.171 |
| Scymnus_camptodromus_717 | Tytthaspis_sedecimpunctata_586 | 0.171 |
| Scymnus_camptodromus_717 | Tytthaspis_sedecimpunctata_588 | 0.171 |
| Scymnus_camptodromus_711 | Tytthaspis_sedecimpunctata_589 | 0.171 |
| Brumus_quadripustulatus_10 | Scymnus_suturalis_833 | 0.171 |
| Hyperaspis_reppensis_664 | Subcoccinella_vigintiquatuorpunctata_619 | 0.171 |
| Hyperaspis_reppensis_666 | Subcoccinella_vigintiquatuorpunctata_619 | 0.171 |
| Hyperaspis_reppensis_667 | Subcoccinella_vigintiquatuorpunctata_619 | 0.171 |
| Brumus_quadripustulatus_10 | Scymnus_suturalis_843 | 0.171 |

| | | |
|---|---|---|
| Brumus_quadripustulatus_10 | Scymnus_suturalis_845 | 0.171 |
| Scymnus_camptodromus_711 | Tytthaspis_sedecimpunctata_586 | 0.171 |
| Scymnus_camptodromus_711 | Tytthaspis_sedecimpunctata_588 | 0.171 |
| Hippodamia_tredecimpunctata_446 | Tytthaspis_sedecimpunctata_584 | 0.171 |
| Hippodamia_tredecimpunctata_448 | Tytthaspis_sedecimpunctata_584 | 0.171 |
| Hippodamia_tredecimpunctata_449 | Tytthaspis_sedecimpunctata_584 | 0.171 |
| Hippodamia_tredecimpunctata_450 | Tytthaspis_sedecimpunctata_584 | 0.171 |
| Hippodamia_tredecimpunctata_452 | Tytthaspis_sedecimpunctata_584 | 0.171 |
| Hippodamia_tredecimpunctata_453 | Tytthaspis_sedecimpunctata_584 | 0.171 |
| Oenopia_conglobata_523 | Tytthaspis_sedecimpunctata_584 | 0.171 |
| Coccinula_quatuordecimpustulata_300 | Tytthaspis_sedecimpunctata_589 | 0.171 |
| Coccinula_quatuordecimpustulata_304 | Tytthaspis_sedecimpunctata_591 | 0.171 |
| Coccinula_quatuordecimpustulata_306 | Tytthaspis_sedecimpunctata_591 | 0.171 |
| Coccinula_quatuordecimpustulata_294 | Tytthaspis_sedecimpunctata_587 | 0.171 |
| Coccinula_quatuordecimpustulata_295 | Tytthaspis_sedecimpunctata_587 | 0.171 |
| Coccinula_quatuordecimpustulata_308 | Tytthaspis_sedecimpunctata_587 | 0.171 |
| Coccinula_quatuordecimpustulata_299 | Tytthaspis_sedecimpunctata_591 | 0.171 |
| Coccinula_quatuordecimpustulata_301 | Tytthaspis_sedecimpunctata_591 | 0.171 |
| Coccinula_quatuordecimpustulata_302 | Tytthaspis_sedecimpunctata_591 | 0.171 |
| Coccinula_quatuordecimpustulata_303 | Tytthaspis_sedecimpunctata_591 | 0.171 |
| Coccinula_quatuordecimpustulata_309 | Tytthaspis_sedecimpunctata_591 | 0.171 |
| Scymnus_pallipediformis_811 | Scymnus_sinuanodulus_831 | 0.171 |
| Scymnus_apetzi_694 | Scymnus_suturalis_833 | 0.171 |
| Scymnus_apetzi_694 | Scymnus_suturalis_838 | 0.171 |
| Scymnus_apetzi_694 | Scymnus_suturalis_840 | 0.171 |
| Scymnus_mimulus_777 | Scymnus_sinuanodulus_831 | 0.171 |
| Scymnus_apetzi_694 | Scymnus_suturalis_845 | 0.171 |
| Scymnus_apetzi_694 | Scymnus_suturalis_834 | 0.171 |
| Anatis_mali_128 | Vibidia_duodecimguttata_596 | 0.171 |
| Anatis_mali_129 | Vibidia_duodecimguttata_596 | 0.171 |
| Anatis_mali_130 | Vibidia_duodecimguttata_596 | 0.171 |
| Anatis_mali_131 | Vibidia_duodecimguttata_596 | 0.171 |
| Anatis_mali_128 | Vibidia_duodecimguttata_603 | 0.171 |
| Anatis_mali_129 | Vibidia_duodecimguttata_603 | 0.171 |
| Anatis_mali_130 | Vibidia_duodecimguttata_603 | 0.171 |
| Anatis_mali_131 | Vibidia_duodecimguttata_603 | 0.171 |
| Scymnus_camptodromus_706 | Tytthaspis_sedecimpunctata_586 | 0.171 |
| Scymnus_camptodromus_706 | Tytthaspis_sedecimpunctata_588 | 0.171 |
| Scymnus_mimulus_778 | Scymnus_sinuanodulus_831 | 0.171 |
| Scymnus_nubilus_797 | Scymnus_rubromaculatus_822 | 0.171 |
| Scymnus_nubilus_797 | Scymnus_rubromaculatus_823 | 0.171 |
| Scymnus_nubilus_797 | Scymnus_rubromaculatus_824 | 0.171 |
| Scymnus_nubilus_797 | Scymnus_rubromaculatus_825 | 0.171 |
| Scymnus_nubilus_797 | Scymnus_rubromaculatus_826 | 0.171 |
| Scymnus_nubilus_797 | Scymnus_rubromaculatus_827 | 0.171 |
| Scymnus_auritus_699 | Scymnus_sinuanodulus_828 | 0.171 |
| Scymnus_apetzi_691 | Scymnus_sinuanodulus_830 | 0.171 |
| Hyperaspis_proba_660 | Scymnus_sinuanodulus_831 | 0.171 |
| Psyllobora_vigintiduopunctata_559 | Vibidia_duodecimguttata_596 | 0.171 |
| Platynaspis_luteorubra_50 | Tytthaspis_sedecimpunctata_583 | 0.171 |
| Scymnus_pallipediformis_811 | Scymnus_sinuanodulus_829 | 0.171 |
| Scymnus_apetzi_693 | Scymnus_suturalis_833 | 0.171 |
| Brumus_quadripustulatus_5 | Tytthaspis_sedecimpunctata_586 | 0.171 |
| Platynaspis_luteorubra_50 | Scymnus_suturalis_838 | 0.171 |
| Platynaspis_luteorubra_50 | Scymnus_suturalis_840 | 0.171 |
| Myrrha_octodecimguttata_488 | Vibidia_duodecimguttata_598 | 0.171 |

| | | |
|---|---|---|
| Myrrha_octodecimguttata_491 | Vibidia_duodecimguttata_598 | 0.171 |
| Myrrha_octodecimguttata_492 | Vibidia_duodecimguttata_598 | 0.171 |
| Myrrha_octodecimguttata_499 | Vibidia_duodecimguttata_598 | 0.171 |
| Myrrha_octodecimguttata_489 | Vibidia_duodecimguttata_599 | 0.171 |
| Myrrha_octodecimguttata_500 | Vibidia_duodecimguttata_599 | 0.171 |
| Myrrha_octodecimguttata_488 | Vibidia_duodecimguttata_601 | 0.171 |
| Myrrha_octodecimguttata_491 | Vibidia_duodecimguttata_601 | 0.171 |
| Myrrha_octodecimguttata_492 | Vibidia_duodecimguttata_601 | 0.171 |
| Myrrha_octodecimguttata_489 | Vibidia_duodecimguttata_602 | 0.171 |
| Myrrha_octodecimguttata_500 | Vibidia_duodecimguttata_602 | 0.171 |
| Platynaspis_luteorubra_50 | Scymnus_suturalis_834 | 0.171 |
| Scymnus_mimulus_777 | Scymnus_sinuanodulus_829 | 0.171 |
| Scymnus_mimulus_778 | Scymnus_sinuanodulus_829 | 0.172 |
| Calvia_quatuordecimguttata_206 | Vibidia_duodecimguttata_598 | 0.172 |
| Calvia_quatuordecimguttata_207 | Vibidia_duodecimguttata_598 | 0.172 |
| Calvia_quatuordecimguttata_216 | Vibidia_duodecimguttata_598 | 0.172 |
| Calvia_quatuordecimguttata_206 | Vibidia_duodecimguttata_599 | 0.172 |
| Calvia_quatuordecimguttata_207 | Vibidia_duodecimguttata_599 | 0.172 |
| Calvia_quatuordecimguttata_216 | Vibidia_duodecimguttata_599 | 0.172 |
| Calvia_quatuordecimguttata_206 | Vibidia_duodecimguttata_601 | 0.172 |
| Calvia_quatuordecimguttata_207 | Vibidia_duodecimguttata_601 | 0.172 |
| Calvia_quatuordecimguttata_216 | Vibidia_duodecimguttata_601 | 0.172 |
| Calvia_quatuordecimguttata_206 | Vibidia_duodecimguttata_602 | 0.172 |
| Calvia_quatuordecimguttata_207 | Vibidia_duodecimguttata_602 | 0.172 |
| Calvia_quatuordecimguttata_216 | Vibidia_duodecimguttata_602 | 0.172 |
| Calvia_quatuordecimguttata_209 | Vibidia_duodecimguttata_603 | 0.172 |
| Calvia_quatuordecimguttata_218 | Vibidia_duodecimguttata_603 | 0.172 |
| Calvia_quatuordecimguttata_201 | Vibidia_duodecimguttata_598 | 0.172 |
| Myrrha_octodecimguttata_493 | Vibidia_duodecimguttata_598 | 0.172 |
| Myrrha_octodecimguttata_498 | Vibidia_duodecimguttata_598 | 0.172 |
| Calvia_quatuordecimguttata_201 | Vibidia_duodecimguttata_599 | 0.172 |
| Calvia_quatuordecimguttata_201 | Vibidia_duodecimguttata_601 | 0.172 |
| Calvia_quatuordecimguttata_201 | Vibidia_duodecimguttata_602 | 0.172 |
| Calvia_quatuordecimguttata_204 | Vibidia_duodecimguttata_603 | 0.172 |
| Calvia_quatuordecimguttata_208 | Vibidia_duodecimguttata_603 | 0.172 |
| Calvia_quatuordecimguttata_217 | Vibidia_duodecimguttata_603 | 0.172 |
| Scymnus_abietis_687 | Scymnus_rubromaculatus_822 | 0.172 |
| Scymnus_abietis_687 | Scymnus_rubromaculatus_823 | 0.172 |
| Scymnus_abietis_687 | Scymnus_rubromaculatus_824 | 0.172 |
| Scymnus_abietis_687 | Scymnus_rubromaculatus_825 | 0.172 |
| Scymnus_abietis_687 | Scymnus_rubromaculatus_826 | 0.172 |
| Scymnus_abietis_687 | Scymnus_rubromaculatus_827 | 0.172 |
| Scymnus_abietis_689 | Scymnus_rubromaculatus_822 | 0.172 |
| Scymnus_abietis_689 | Scymnus_rubromaculatus_823 | 0.172 |
| Scymnus_abietis_689 | Scymnus_rubromaculatus_824 | 0.172 |
| Scymnus_abietis_689 | Scymnus_rubromaculatus_825 | 0.172 |
| Scymnus_abietis_689 | Scymnus_rubromaculatus_826 | 0.172 |
| Scymnus_abietis_689 | Scymnus_rubromaculatus_827 | 0.172 |
| Hyperaspis_proba_660 | Scymnus_sinuanodulus_829 | 0.172 |
| Hyperaspis_proba_661 | Tytthaspis_sedecimpunctata_584 | 0.172 |
| Chilocorus_stigma_43 | Tytthaspis_sedecimpunctata_586 | 0.172 |
| Chilocorus_stigma_43 | Tytthaspis_sedecimpunctata_588 | 0.172 |
| Chilocorus_stigma_44 | Tytthaspis_sedecimpunctata_591 | 0.172 |
| Chilocorus_stigma_46 | Tytthaspis_sedecimpunctata_591 | 0.172 |
| Chilocorus_stigma_47 | Tytthaspis_sedecimpunctata_591 | 0.172 |
| Brumus_quadripustulatus_12 | Tytthaspis_sedecimpunctata_584 | 0.172 |

| | | |
|---|---|---|
| Chilocorus_stigma_44 | Tytthaspis_sedecimpunctata_593 | 0.172 |
| Chilocorus_stigma_47 | Tytthaspis_sedecimpunctata_593 | 0.172 |
| Chilocorus_nigritus_26 | Scymnus_suturalis_841 | 0.172 |
| Chilocorus_nigritus_26 | Scymnus_suturalis_842 | 0.172 |
| Chilocorus_nigritus_25 | Scymnus_suturalis_843 | 0.172 |
| Chilocorus_nigritus_26 | Scymnus_suturalis_844 | 0.172 |
| Chilocorus_nigritus_25 | Scymnus_suturalis_845 | 0.172 |
| Chilocorus_nigritus_26 | Scymnus_suturalis_846 | 0.172 |
| Chilocorus_nigritus_26 | Scymnus_suturalis_847 | 0.172 |
| Chilocorus_nigritus_26 | Scymnus_suturalis_848 | 0.172 |
| Brumus_quadripustulatus_9 | Tytthaspis_sedecimpunctata_577 | 0.172 |
| Brumus_quadripustulatus_9 | Tytthaspis_sedecimpunctata_578 | 0.172 |
| Brumus_quadripustulatus_9 | Tytthaspis_sedecimpunctata_579 | 0.172 |
| Brumus_quadripustulatus_9 | Tytthaspis_sedecimpunctata_581 | 0.172 |
| Brumus_quadripustulatus_9 | Tytthaspis_sedecimpunctata_582 | 0.172 |
| Brumus_quadripustulatus_15 | Tytthaspis_sedecimpunctata_585 | 0.172 |
| Brumus_quadripustulatus_16 | Tytthaspis_sedecimpunctata_585 | 0.172 |
| Brumus_quadripustulatus_6 | Tytthaspis_sedecimpunctata_585 | 0.172 |
| Brumus_quadripustulatus_8 | Tytthaspis_sedecimpunctata_585 | 0.172 |
| Brumus_quadripustulatus_9 | Tytthaspis_sedecimpunctata_590 | 0.172 |
| Brumus_quadripustulatus_9 | Tytthaspis_sedecimpunctata_592 | 0.172 |
| Brumus_quadripustulatus_15 | Tytthaspis_sedecimpunctata_593 | 0.172 |
| Brumus_quadripustulatus_16 | Tytthaspis_sedecimpunctata_593 | 0.172 |
| Brumus_quadripustulatus_6 | Tytthaspis_sedecimpunctata_593 | 0.172 |
| Brumus_quadripustulatus_8 | Tytthaspis_sedecimpunctata_593 | 0.172 |
| Brumus_quadripustulatus_9 | Tytthaspis_sedecimpunctata_594 | 0.172 |
| Brumus_quadripustulatus_9 | Tytthaspis_sedecimpunctata_595 | 0.172 |
| Chilocorus_nigritus_26 | Scymnus_suturalis_832 | 0.172 |
| Chilocorus_nigritus_26 | Scymnus_suturalis_835 | 0.172 |
| Chilocorus_nigritus_26 | Scymnus_suturalis_836 | 0.172 |
| Hyperaspis_reppensis_662 | Scymnus_suturalis_838 | 0.172 |
| Hyperaspis_reppensis_663 | Scymnus_suturalis_838 | 0.172 |
| Chilocorus_nigritus_26 | Scymnus_suturalis_839 | 0.172 |
| Hyperaspis_reppensis_662 | Scymnus_suturalis_840 | 0.172 |
| Hyperaspis_reppensis_663 | Scymnus_suturalis_840 | 0.172 |
| Propylaea_quatuordecimpunctata_539 | Tytthaspis_sedecimpunctata_584 | 0.172 |
| Propylaea_quatuordecimpunctata_540 | Tytthaspis_sedecimpunctata_584 | 0.172 |
| Propylaea_quatuordecimpunctata_543 | Tytthaspis_sedecimpunctata_584 | 0.172 |
| Propylaea_quatuordecimpunctata_548 | Tytthaspis_sedecimpunctata_584 | 0.172 |
| Propylaea_quatuordecimpunctata_539 | Tytthaspis_sedecimpunctata_586 | 0.172 |
| Propylaea_quatuordecimpunctata_542 | Tytthaspis_sedecimpunctata_586 | 0.172 |
| Propylaea_quatuordecimpunctata_546 | Tytthaspis_sedecimpunctata_586 | 0.172 |
| Propylaea_quatuordecimpunctata_539 | Tytthaspis_sedecimpunctata_588 | 0.172 |
| Propylaea_quatuordecimpunctata_542 | Tytthaspis_sedecimpunctata_588 | 0.172 |
| Propylaea_quatuordecimpunctata_546 | Tytthaspis_sedecimpunctata_588 | 0.172 |
| Adalia_bipunctata_105 | Tytthaspis_sedecimpunctata_589 | 0.172 |
| Scymnus_camptodromus_704 | Tytthaspis_sedecimpunctata_589 | 0.172 |
| Hyperaspis_reppensis_662 | Scymnus_suturalis_834 | 0.172 |
| Hyperaspis_reppensis_663 | Scymnus_suturalis_834 | 0.172 |
| Platynaspis_luteorubra_51 | Scymnus_suturalis_843 | 0.172 |
| Platynaspis_luteorubra_53 | Scymnus_suturalis_843 | 0.172 |
| Platynaspis_luteorubra_58 | Scymnus_suturalis_843 | 0.172 |
| Platynaspis_luteorubra_51 | Scymnus_suturalis_845 | 0.172 |
| Platynaspis_luteorubra_53 | Scymnus_suturalis_845 | 0.172 |
| Platynaspis_luteorubra_58 | Scymnus_suturalis_845 | 0.172 |
| Propylaea_quatuordecimpunctata_539 | Tytthaspis_sedecimpunctata_577 | 0.172 |

Supplementary Table S4 (continued)

| | | |
|---|---|---|
| Propylaea_quatuordecimpunctata_540 | Tytthaspis_sedecimpunctata_577 | 0.172 |
| Propylaea_quatuordecimpunctata_543 | Tytthaspis_sedecimpunctata_577 | 0.172 |
| Propylaea_quatuordecimpunctata_548 | Tytthaspis_sedecimpunctata_577 | 0.172 |
| Propylaea_quatuordecimpunctata_539 | Tytthaspis_sedecimpunctata_578 | 0.172 |
| Propylaea_quatuordecimpunctata_540 | Tytthaspis_sedecimpunctata_578 | 0.172 |
| Propylaea_quatuordecimpunctata_543 | Tytthaspis_sedecimpunctata_578 | 0.172 |
| Propylaea_quatuordecimpunctata_548 | Tytthaspis_sedecimpunctata_578 | 0.172 |
| Propylaea_quatuordecimpunctata_539 | Tytthaspis_sedecimpunctata_579 | 0.172 |
| Propylaea_quatuordecimpunctata_540 | Tytthaspis_sedecimpunctata_579 | 0.172 |
| Propylaea_quatuordecimpunctata_543 | Tytthaspis_sedecimpunctata_579 | 0.172 |
| Propylaea_quatuordecimpunctata_548 | Tytthaspis_sedecimpunctata_579 | 0.172 |
| Propylaea_quatuordecimpunctata_539 | Tytthaspis_sedecimpunctata_581 | 0.172 |
| Propylaea_quatuordecimpunctata_540 | Tytthaspis_sedecimpunctata_581 | 0.172 |
| Propylaea_quatuordecimpunctata_543 | Tytthaspis_sedecimpunctata_581 | 0.172 |
| Propylaea_quatuordecimpunctata_548 | Tytthaspis_sedecimpunctata_581 | 0.172 |
| Propylaea_quatuordecimpunctata_539 | Tytthaspis_sedecimpunctata_582 | 0.172 |
| Propylaea_quatuordecimpunctata_540 | Tytthaspis_sedecimpunctata_582 | 0.172 |
| Propylaea_quatuordecimpunctata_543 | Tytthaspis_sedecimpunctata_582 | 0.172 |
| Propylaea_quatuordecimpunctata_548 | Tytthaspis_sedecimpunctata_582 | 0.172 |
| Propylaea_quatuordecimpunctata_545 | Tytthaspis_sedecimpunctata_583 | 0.172 |
| Coccinella_hieroglyphica_225 | Tytthaspis_sedecimpunctata_584 | 0.172 |
| Coccinella_trifasciata_280 | Tytthaspis_sedecimpunctata_584 | 0.172 |
| Propylaea_quatuordecimpunctata_545 | Tytthaspis_sedecimpunctata_585 | 0.172 |
| Chilocorus_stigma_45 | Tytthaspis_sedecimpunctata_586 | 0.172 |
| Scymnus_camptodromus_714 | Tytthaspis_sedecimpunctata_586 | 0.172 |
| Adalia_bipunctata_99 | Tytthaspis_sedecimpunctata_587 | 0.172 |
| Aphidecta_obliterata_178 | Tytthaspis_sedecimpunctata_588 | 0.172 |
| Chilocorus_stigma_45 | Tytthaspis_sedecimpunctata_588 | 0.172 |
| Scymnus_camptodromus_714 | Tytthaspis_sedecimpunctata_588 | 0.172 |
| Adalia_bipunctata_102 | Tytthaspis_sedecimpunctata_589 | 0.172 |
| Adalia_bipunctata_103 | Tytthaspis_sedecimpunctata_589 | 0.172 |
| Adalia_bipunctata_106 | Tytthaspis_sedecimpunctata_589 | 0.172 |
| Adalia_bipunctata_107 | Tytthaspis_sedecimpunctata_589 | 0.172 |
| Adalia_bipunctata_108 | Tytthaspis_sedecimpunctata_589 | 0.172 |
| Adalia_bipunctata_109 | Tytthaspis_sedecimpunctata_589 | 0.172 |
| Harmonia_axyridis_354 | Tytthaspis_sedecimpunctata_589 | 0.172 |
| Harmonia_axyridis_356 | Tytthaspis_sedecimpunctata_589 | 0.172 |
| Harmonia_axyridis_357 | Tytthaspis_sedecimpunctata_589 | 0.172 |
| Harmonia_axyridis_358 | Tytthaspis_sedecimpunctata_589 | 0.172 |
| Harmonia_axyridis_359 | Tytthaspis_sedecimpunctata_589 | 0.172 |
| Harmonia_axyridis_360 | Tytthaspis_sedecimpunctata_589 | 0.172 |
| Harmonia_axyridis_361 | Tytthaspis_sedecimpunctata_589 | 0.172 |
| Harmonia_axyridis_363 | Tytthaspis_sedecimpunctata_589 | 0.172 |
| Harmonia_axyridis_364 | Tytthaspis_sedecimpunctata_589 | 0.172 |
| Propylaea_quatuordecimpunctata_532 | Tytthaspis_sedecimpunctata_589 | 0.172 |
| Propylaea_quatuordecimpunctata_533 | Tytthaspis_sedecimpunctata_589 | 0.172 |
| Propylaea_quatuordecimpunctata_535 | Tytthaspis_sedecimpunctata_589 | 0.172 |
| Propylaea_quatuordecimpunctata_536 | Tytthaspis_sedecimpunctata_589 | 0.172 |
| Propylaea_quatuordecimpunctata_537 | Tytthaspis_sedecimpunctata_589 | 0.172 |
| Propylaea_quatuordecimpunctata_541 | Tytthaspis_sedecimpunctata_589 | 0.172 |
| Propylaea_quatuordecimpunctata_539 | Tytthaspis_sedecimpunctata_590 | 0.172 |
| Propylaea_quatuordecimpunctata_540 | Tytthaspis_sedecimpunctata_590 | 0.172 |
| Propylaea_quatuordecimpunctata_543 | Tytthaspis_sedecimpunctata_590 | 0.172 |
| Propylaea_quatuordecimpunctata_548 | Tytthaspis_sedecimpunctata_590 | 0.172 |
| Aphidecta_obliterata_179 | Tytthaspis_sedecimpunctata_592 | 0.172 |
| Propylaea_quatuordecimpunctata_545 | Tytthaspis_sedecimpunctata_592 | 0.172 |

| | | |
|---|---|---|
| Propylaea_quatuordecimpunctata_539 | Tytthaspis_sedecimpunctata_593 | 0.172 |
| Propylaea_quatuordecimpunctata_540 | Tytthaspis_sedecimpunctata_593 | 0.172 |
| Propylaea_quatuordecimpunctata_543 | Tytthaspis_sedecimpunctata_593 | 0.172 |
| Propylaea_quatuordecimpunctata_548 | Tytthaspis_sedecimpunctata_593 | 0.172 |
| Propylaea_quatuordecimpunctata_539 | Tytthaspis_sedecimpunctata_594 | 0.172 |
| Propylaea_quatuordecimpunctata_540 | Tytthaspis_sedecimpunctata_594 | 0.172 |
| Propylaea_quatuordecimpunctata_543 | Tytthaspis_sedecimpunctata_594 | 0.172 |
| Propylaea_quatuordecimpunctata_548 | Tytthaspis_sedecimpunctata_594 | 0.172 |
| Propylaea_quatuordecimpunctata_539 | Tytthaspis_sedecimpunctata_595 | 0.172 |
| Propylaea_quatuordecimpunctata_540 | Tytthaspis_sedecimpunctata_595 | 0.172 |
| Propylaea_quatuordecimpunctata_543 | Tytthaspis_sedecimpunctata_595 | 0.172 |
| Propylaea_quatuordecimpunctata_548 | Tytthaspis_sedecimpunctata_595 | 0.172 |
| Hyperaspis_reppensis_662 | Scymnus_suturalis_833 | 0.172 |
| Hyperaspis_reppensis_663 | Scymnus_suturalis_833 | 0.172 |
| Platynaspis_luteorubra_51 | Scymnus_suturalis_837 | 0.172 |
| Platynaspis_luteorubra_53 | Scymnus_suturalis_837 | 0.172 |
| Platynaspis_luteorubra_58 | Scymnus_suturalis_837 | 0.172 |
| Hyperaspis_oregona_658 | Scymnus_suturalis_838 | 0.172 |
| Hyperaspis_oregona_658 | Scymnus_suturalis_840 | 0.172 |
| Anatis_ocellata_132 | Scymnus_suturalis_843 | 0.172 |
| Anatis_ocellata_134 | Scymnus_suturalis_843 | 0.172 |
| Anatis_ocellata_135 | Scymnus_suturalis_843 | 0.172 |
| Anatis_ocellata_138 | Scymnus_suturalis_843 | 0.172 |
| Anatis_ocellata_139 | Scymnus_suturalis_843 | 0.172 |
| Anatis_ocellata_140 | Scymnus_suturalis_843 | 0.172 |
| Anatis_ocellata_141 | Scymnus_suturalis_843 | 0.172 |
| Anatis_ocellata_142 | Scymnus_suturalis_843 | 0.172 |
| Anatis_ocellata_144 | Scymnus_suturalis_843 | 0.172 |
| Anatis_ocellata_145 | Scymnus_suturalis_843 | 0.172 |
| Anatis_ocellata_146 | Scymnus_suturalis_843 | 0.172 |
| Hyperaspis_oregona_659 | Scymnus_suturalis_843 | 0.172 |
| Anatis_ocellata_132 | Scymnus_suturalis_845 | 0.172 |
| Anatis_ocellata_134 | Scymnus_suturalis_845 | 0.172 |
| Anatis_ocellata_135 | Scymnus_suturalis_845 | 0.172 |
| Anatis_ocellata_138 | Scymnus_suturalis_845 | 0.172 |
| Anatis_ocellata_139 | Scymnus_suturalis_845 | 0.172 |
| Anatis_ocellata_140 | Scymnus_suturalis_845 | 0.172 |
| Anatis_ocellata_141 | Scymnus_suturalis_845 | 0.172 |
| Anatis_ocellata_142 | Scymnus_suturalis_845 | 0.172 |
| Anatis_ocellata_144 | Scymnus_suturalis_845 | 0.172 |
| Anatis_ocellata_145 | Scymnus_suturalis_845 | 0.172 |
| Anatis_ocellata_146 | Scymnus_suturalis_845 | 0.172 |
| Hyperaspis_oregona_659 | Scymnus_suturalis_845 | 0.172 |
| Adalia_bipunctata_105 | Tytthaspis_sedecimpunctata_577 | 0.172 |
| Aphidecta_obliterata_178 | Tytthaspis_sedecimpunctata_577 | 0.172 |
| Coccinella_hieroglyphica_225 | Tytthaspis_sedecimpunctata_577 | 0.172 |
| Coccinella_trifasciata_275 | Tytthaspis_sedecimpunctata_577 | 0.172 |
| Coccinella_trifasciata_277 | Tytthaspis_sedecimpunctata_577 | 0.172 |
| Coccinella_trifasciata_278 | Tytthaspis_sedecimpunctata_577 | 0.172 |
| Coccinella_trifasciata_279 | Tytthaspis_sedecimpunctata_577 | 0.172 |
| Coccinella_trifasciata_281 | Tytthaspis_sedecimpunctata_577 | 0.172 |
| Adalia_bipunctata_105 | Tytthaspis_sedecimpunctata_578 | 0.172 |
| Aphidecta_obliterata_178 | Tytthaspis_sedecimpunctata_578 | 0.172 |
| Coccinella_hieroglyphica_225 | Tytthaspis_sedecimpunctata_578 | 0.172 |
| Coccinella_trifasciata_275 | Tytthaspis_sedecimpunctata_578 | 0.172 |
| Coccinella_trifasciata_277 | Tytthaspis_sedecimpunctata_578 | 0.172 |

| | | |
|---|---|---|
| Coccinella_trifasciata_278 | Tytthaspis_sedecimpunctata_578 | 0.172 |
| Coccinella_trifasciata_279 | Tytthaspis_sedecimpunctata_578 | 0.172 |
| Coccinella_trifasciata_281 | Tytthaspis_sedecimpunctata_578 | 0.172 |
| Adalia_bipunctata_105 | Tytthaspis_sedecimpunctata_579 | 0.172 |
| Aphidecta_obliterata_178 | Tytthaspis_sedecimpunctata_579 | 0.172 |
| Coccinella_hieroglyphica_225 | Tytthaspis_sedecimpunctata_579 | 0.172 |
| Coccinella_trifasciata_275 | Tytthaspis_sedecimpunctata_579 | 0.172 |
| Coccinella_trifasciata_277 | Tytthaspis_sedecimpunctata_579 | 0.172 |
| Coccinella_trifasciata_278 | Tytthaspis_sedecimpunctata_579 | 0.172 |
| Coccinella_trifasciata_279 | Tytthaspis_sedecimpunctata_579 | 0.172 |
| Coccinella_trifasciata_281 | Tytthaspis_sedecimpunctata_579 | 0.172 |
| Aphidecta_obliterata_179 | Tytthaspis_sedecimpunctata_580 | 0.172 |
| Propylaea_quatuordecimpunctata_539 | Tytthaspis_sedecimpunctata_580 | 0.172 |
| Propylaea_quatuordecimpunctata_540 | Tytthaspis_sedecimpunctata_580 | 0.172 |
| Propylaea_quatuordecimpunctata_543 | Tytthaspis_sedecimpunctata_580 | 0.172 |
| Propylaea_quatuordecimpunctata_548 | Tytthaspis_sedecimpunctata_580 | 0.172 |
| Adalia_bipunctata_105 | Tytthaspis_sedecimpunctata_581 | 0.172 |
| Aphidecta_obliterata_178 | Tytthaspis_sedecimpunctata_581 | 0.172 |
| Coccinella_hieroglyphica_225 | Tytthaspis_sedecimpunctata_581 | 0.172 |
| Coccinella_trifasciata_275 | Tytthaspis_sedecimpunctata_581 | 0.172 |
| Coccinella_trifasciata_277 | Tytthaspis_sedecimpunctata_581 | 0.172 |
| Coccinella_trifasciata_278 | Tytthaspis_sedecimpunctata_581 | 0.172 |
| Coccinella_trifasciata_279 | Tytthaspis_sedecimpunctata_581 | 0.172 |
| Coccinella_trifasciata_281 | Tytthaspis_sedecimpunctata_581 | 0.172 |
| Adalia_bipunctata_105 | Tytthaspis_sedecimpunctata_582 | 0.172 |
| Aphidecta_obliterata_178 | Tytthaspis_sedecimpunctata_582 | 0.172 |
| Coccinella_hieroglyphica_225 | Tytthaspis_sedecimpunctata_582 | 0.172 |
| Coccinella_trifasciata_275 | Tytthaspis_sedecimpunctata_582 | 0.172 |
| Coccinella_trifasciata_277 | Tytthaspis_sedecimpunctata_582 | 0.172 |
| Coccinella_trifasciata_278 | Tytthaspis_sedecimpunctata_582 | 0.172 |
| Coccinella_trifasciata_279 | Tytthaspis_sedecimpunctata_582 | 0.172 |
| Coccinella_trifasciata_281 | Tytthaspis_sedecimpunctata_582 | 0.172 |
| Coccinella_trifasciata_272 | Tytthaspis_sedecimpunctata_583 | 0.172 |
| Coccinella_trifasciata_276 | Tytthaspis_sedecimpunctata_583 | 0.172 |
| Adalia_bipunctata_100 | Tytthaspis_sedecimpunctata_584 | 0.172 |
| Coccinella_hieroglyphica_225 | Tytthaspis_sedecimpunctata_585 | 0.172 |
| Coccinella_transversoguttata_265 | Tytthaspis_sedecimpunctata_585 | 0.172 |
| Coccinella_transversoguttata_266 | Tytthaspis_sedecimpunctata_585 | 0.172 |
| Coccinella_trifasciata_280 | Tytthaspis_sedecimpunctata_585 | 0.172 |
| Adalia_bipunctata_102 | Tytthaspis_sedecimpunctata_587 | 0.172 |
| Adalia_bipunctata_103 | Tytthaspis_sedecimpunctata_587 | 0.172 |
| Adalia_bipunctata_106 | Tytthaspis_sedecimpunctata_587 | 0.172 |
| Adalia_bipunctata_107 | Tytthaspis_sedecimpunctata_587 | 0.172 |
| Adalia_bipunctata_108 | Tytthaspis_sedecimpunctata_587 | 0.172 |
| Adalia_bipunctata_109 | Tytthaspis_sedecimpunctata_587 | 0.172 |
| Coccinella_transversoguttata_267 | Tytthaspis_sedecimpunctata_587 | 0.172 |
| Coccinella_transversoguttata_268 | Tytthaspis_sedecimpunctata_587 | 0.172 |
| Coccinella_transversoguttata_269 | Tytthaspis_sedecimpunctata_587 | 0.172 |
| Coccinella_transversoguttata_270 | Tytthaspis_sedecimpunctata_587 | 0.172 |
| Coccinella_transversoguttata_271 | Tytthaspis_sedecimpunctata_587 | 0.172 |
| Adalia_bipunctata_98 | Tytthaspis_sedecimpunctata_588 | 0.172 |
| Harmonia_axyridis_354 | Tytthaspis_sedecimpunctata_588 | 0.172 |
| Harmonia_axyridis_356 | Tytthaspis_sedecimpunctata_588 | 0.172 |
| Harmonia_axyridis_357 | Tytthaspis_sedecimpunctata_588 | 0.172 |
| Harmonia_axyridis_358 | Tytthaspis_sedecimpunctata_588 | 0.172 |
| Harmonia_axyridis_359 | Tytthaspis_sedecimpunctata_588 | 0.172 |

| | | |
|---|---|---|
| Harmonia_axyridis_360 | Tytthaspis_sedecimpunctata_588 | 0.172 |
| Harmonia_axyridis_361 | Tytthaspis_sedecimpunctata_588 | 0.172 |
| Harmonia_axyridis_363 | Tytthaspis_sedecimpunctata_588 | 0.172 |
| Harmonia_axyridis_364 | Tytthaspis_sedecimpunctata_588 | 0.172 |
| Scymnus_auritus_703 | Tytthaspis_sedecimpunctata_589 | 0.172 |
| Adalia_bipunctata_105 | Tytthaspis_sedecimpunctata_590 | 0.172 |
| Aphidecta_obliterata_178 | Tytthaspis_sedecimpunctata_590 | 0.172 |
| Coccinella_hieroglyphica_225 | Tytthaspis_sedecimpunctata_590 | 0.172 |
| Coccinella_trifasciata_275 | Tytthaspis_sedecimpunctata_590 | 0.172 |
| Coccinella_trifasciata_277 | Tytthaspis_sedecimpunctata_590 | 0.172 |
| Coccinella_trifasciata_278 | Tytthaspis_sedecimpunctata_590 | 0.172 |
| Coccinella_trifasciata_279 | Tytthaspis_sedecimpunctata_590 | 0.172 |
| Coccinella_trifasciata_281 | Tytthaspis_sedecimpunctata_590 | 0.172 |
| Coccinella_trifasciata_274 | Tytthaspis_sedecimpunctata_591 | 0.172 |
| Psyllobora_renifer_551 | Tytthaspis_sedecimpunctata_591 | 0.172 |
| Psyllobora_renifer_552 | Tytthaspis_sedecimpunctata_591 | 0.172 |
| Psyllobora_renifer_553 | Tytthaspis_sedecimpunctata_591 | 0.172 |
| Psyllobora_renifer_554 | Tytthaspis_sedecimpunctata_591 | 0.172 |
| Coccinella_hieroglyphica_225 | Tytthaspis_sedecimpunctata_592 | 0.172 |
| Coccinella_trifasciata_275 | Tytthaspis_sedecimpunctata_592 | 0.172 |
| Coccinella_trifasciata_277 | Tytthaspis_sedecimpunctata_592 | 0.172 |
| Coccinella_trifasciata_278 | Tytthaspis_sedecimpunctata_592 | 0.172 |
| Coccinella_trifasciata_279 | Tytthaspis_sedecimpunctata_592 | 0.172 |
| Coccinella_trifasciata_281 | Tytthaspis_sedecimpunctata_592 | 0.172 |
| Adalia_bipunctata_105 | Tytthaspis_sedecimpunctata_593 | 0.172 |
| Aphidecta_obliterata_178 | Tytthaspis_sedecimpunctata_593 | 0.172 |
| Coccinella_trifasciata_275 | Tytthaspis_sedecimpunctata_593 | 0.172 |
| Coccinella_trifasciata_277 | Tytthaspis_sedecimpunctata_593 | 0.172 |
| Coccinella_trifasciata_278 | Tytthaspis_sedecimpunctata_593 | 0.172 |
| Coccinella_trifasciata_279 | Tytthaspis_sedecimpunctata_593 | 0.172 |
| Coccinella_trifasciata_281 | Tytthaspis_sedecimpunctata_593 | 0.172 |
| Adalia_bipunctata_105 | Tytthaspis_sedecimpunctata_594 | 0.172 |
| Aphidecta_obliterata_178 | Tytthaspis_sedecimpunctata_594 | 0.172 |
| Coccinella_hieroglyphica_225 | Tytthaspis_sedecimpunctata_594 | 0.172 |
| Coccinella_trifasciata_275 | Tytthaspis_sedecimpunctata_594 | 0.172 |
| Coccinella_trifasciata_277 | Tytthaspis_sedecimpunctata_594 | 0.172 |
| Coccinella_trifasciata_278 | Tytthaspis_sedecimpunctata_594 | 0.172 |
| Coccinella_trifasciata_279 | Tytthaspis_sedecimpunctata_594 | 0.172 |
| Coccinella_trifasciata_281 | Tytthaspis_sedecimpunctata_594 | 0.172 |
| Adalia_bipunctata_105 | Tytthaspis_sedecimpunctata_595 | 0.172 |
| Aphidecta_obliterata_178 | Tytthaspis_sedecimpunctata_595 | 0.172 |
| Coccinella_hieroglyphica_225 | Tytthaspis_sedecimpunctata_595 | 0.172 |
| Coccinella_trifasciata_275 | Tytthaspis_sedecimpunctata_595 | 0.172 |
| Coccinella_trifasciata_277 | Tytthaspis_sedecimpunctata_595 | 0.172 |
| Coccinella_trifasciata_278 | Tytthaspis_sedecimpunctata_595 | 0.172 |
| Coccinella_trifasciata_279 | Tytthaspis_sedecimpunctata_595 | 0.172 |
| Coccinella_trifasciata_281 | Tytthaspis_sedecimpunctata_595 | 0.172 |
| Platynaspis_luteorubra_55 | Scymnus_suturalis_833 | 0.172 |
| Hyperaspis_oregona_658 | Scymnus_suturalis_834 | 0.172 |
| Hyperaspis_oregona_659 | Scymnus_suturalis_837 | 0.172 |
| Hyperaspis_campestris_651 | Scymnus_suturalis_841 | 0.172 |
| Hyperaspis_campestris_652 | Scymnus_suturalis_841 | 0.172 |
| Hyperaspis_campestris_653 | Scymnus_suturalis_841 | 0.172 |
| Hyperaspis_campestris_651 | Scymnus_suturalis_842 | 0.172 |
| Hyperaspis_campestris_652 | Scymnus_suturalis_842 | 0.172 |
| Hyperaspis_campestris_653 | Scymnus_suturalis_842 | 0.172 |

Supplementary Table S4 (continued)

| | | |
|---|---|---|
| Scymnus_pallipediformis_812 | Scymnus_suturalis_843 | 0.172 |
| Hyperaspis_campestris_651 | Scymnus_suturalis_844 | 0.172 |
| Hyperaspis_campestris_652 | Scymnus_suturalis_844 | 0.172 |
| Hyperaspis_campestris_653 | Scymnus_suturalis_844 | 0.172 |
| Scymnus_pallipediformis_812 | Scymnus_suturalis_845 | 0.172 |
| Hyperaspis_campestris_651 | Scymnus_suturalis_846 | 0.172 |
| Hyperaspis_campestris_652 | Scymnus_suturalis_846 | 0.172 |
| Hyperaspis_campestris_653 | Scymnus_suturalis_846 | 0.172 |
| Hyperaspis_campestris_651 | Scymnus_suturalis_847 | 0.172 |
| Hyperaspis_campestris_652 | Scymnus_suturalis_847 | 0.172 |
| Hyperaspis_campestris_653 | Scymnus_suturalis_847 | 0.172 |
| Hyperaspis_campestris_651 | Scymnus_suturalis_848 | 0.172 |
| Hyperaspis_campestris_652 | Scymnus_suturalis_848 | 0.172 |
| Hyperaspis_campestris_653 | Scymnus_suturalis_848 | 0.172 |
| Adalia_bipunctata_101 | Tytthaspis_sedecimpunctata_577 | 0.172 |
| Adalia_bipunctata_104 | Tytthaspis_sedecimpunctata_577 | 0.172 |
| Coccinella_trifasciata_274 | Tytthaspis_sedecimpunctata_577 | 0.172 |
| Adalia_bipunctata_101 | Tytthaspis_sedecimpunctata_578 | 0.172 |
| Adalia_bipunctata_104 | Tytthaspis_sedecimpunctata_578 | 0.172 |
| Coccinella_trifasciata_274 | Tytthaspis_sedecimpunctata_578 | 0.172 |
| Adalia_bipunctata_101 | Tytthaspis_sedecimpunctata_579 | 0.172 |
| Adalia_bipunctata_104 | Tytthaspis_sedecimpunctata_579 | 0.172 |
| Coccinella_trifasciata_274 | Tytthaspis_sedecimpunctata_579 | 0.172 |
| Adalia_bipunctata_105 | Tytthaspis_sedecimpunctata_580 | 0.172 |
| Adalia_bipunctata_101 | Tytthaspis_sedecimpunctata_581 | 0.172 |
| Adalia_bipunctata_104 | Tytthaspis_sedecimpunctata_581 | 0.172 |
| Coccinella_trifasciata_274 | Tytthaspis_sedecimpunctata_581 | 0.172 |
| Adalia_bipunctata_101 | Tytthaspis_sedecimpunctata_582 | 0.172 |
| Adalia_bipunctata_104 | Tytthaspis_sedecimpunctata_582 | 0.172 |
| Coccinella_trifasciata_274 | Tytthaspis_sedecimpunctata_582 | 0.172 |
| Psyllobora_renifer_551 | Tytthaspis_sedecimpunctata_583 | 0.172 |
| Psyllobora_renifer_552 | Tytthaspis_sedecimpunctata_583 | 0.172 |
| Psyllobora_renifer_553 | Tytthaspis_sedecimpunctata_583 | 0.172 |
| Psyllobora_renifer_554 | Tytthaspis_sedecimpunctata_583 | 0.172 |
| Harmonia_axyridis_355 | Tytthaspis_sedecimpunctata_586 | 0.172 |
| Harmonia_axyridis_355 | Tytthaspis_sedecimpunctata_587 | 0.172 |
| Coccinella_trifasciata_273 | Tytthaspis_sedecimpunctata_588 | 0.172 |
| Scymnus_auritus_698 | Tytthaspis_sedecimpunctata_589 | 0.172 |
| Adalia_bipunctata_101 | Tytthaspis_sedecimpunctata_590 | 0.172 |
| Adalia_bipunctata_104 | Tytthaspis_sedecimpunctata_590 | 0.172 |
| Coccinella_trifasciata_274 | Tytthaspis_sedecimpunctata_590 | 0.172 |
| Coccinella_trifasciata_272 | Tytthaspis_sedecimpunctata_591 | 0.172 |
| Coccinella_trifasciata_276 | Tytthaspis_sedecimpunctata_591 | 0.172 |
| Harmonia_axyridis_355 | Tytthaspis_sedecimpunctata_591 | 0.172 |
| Coccinella_trifasciata_274 | Tytthaspis_sedecimpunctata_592 | 0.172 |
| Adalia_bipunctata_100 | Tytthaspis_sedecimpunctata_593 | 0.172 |
| Adalia_bipunctata_101 | Tytthaspis_sedecimpunctata_593 | 0.172 |
| Adalia_bipunctata_104 | Tytthaspis_sedecimpunctata_593 | 0.172 |
| Coccinella_trifasciata_274 | Tytthaspis_sedecimpunctata_593 | 0.172 |
| Adalia_bipunctata_101 | Tytthaspis_sedecimpunctata_594 | 0.172 |
| Adalia_bipunctata_104 | Tytthaspis_sedecimpunctata_594 | 0.172 |
| Coccinella_trifasciata_274 | Tytthaspis_sedecimpunctata_594 | 0.172 |
| Adalia_bipunctata_101 | Tytthaspis_sedecimpunctata_595 | 0.172 |
| Adalia_bipunctata_104 | Tytthaspis_sedecimpunctata_595 | 0.172 |
| Coccinella_trifasciata_274 | Tytthaspis_sedecimpunctata_595 | 0.172 |
| Hyperaspis_campestris_651 | Scymnus_suturalis_832 | 0.172 |

| | | |
|---|---|---|
| Hyperaspis_campestris_652 | Scymnus_suturalis_832 | 0.172 |
| Hyperaspis_campestris_653 | Scymnus_suturalis_832 | 0.172 |
| Hyperaspis_campestris_651 | Scymnus_suturalis_835 | 0.172 |
| Hyperaspis_campestris_652 | Scymnus_suturalis_835 | 0.172 |
| Hyperaspis_campestris_653 | Scymnus_suturalis_835 | 0.172 |
| Hyperaspis_campestris_651 | Scymnus_suturalis_836 | 0.172 |
| Hyperaspis_campestris_652 | Scymnus_suturalis_836 | 0.172 |
| Hyperaspis_campestris_653 | Scymnus_suturalis_836 | 0.172 |
| Hyperaspis_campestris_651 | Scymnus_suturalis_837 | 0.172 |
| Hyperaspis_campestris_652 | Scymnus_suturalis_837 | 0.172 |
| Hyperaspis_campestris_653 | Scymnus_suturalis_837 | 0.172 |
| Scymnus_pallipediformis_812 | Scymnus_suturalis_837 | 0.172 |
| Hyperaspis_campestris_651 | Scymnus_suturalis_839 | 0.172 |
| Hyperaspis_campestris_652 | Scymnus_suturalis_839 | 0.172 |
| Hyperaspis_campestris_653 | Scymnus_suturalis_839 | 0.172 |
| Psyllobora_vigintimaculata_575 | Vibidia_duodecimguttata_598 | 0.172 |
| Psyllobora_vigintimaculata_576 | Vibidia_duodecimguttata_598 | 0.172 |
| Anatis_ocellata_143 | Tytthaspis_sedecimpunctata_584 | 0.172 |
| Coccinella_quinquepunctata_232 | Tytthaspis_sedecimpunctata_584 | 0.172 |
| Coccinella_quinquepunctata_236 | Tytthaspis_sedecimpunctata_584 | 0.172 |
| Coccinella_quinquepunctata_239 | Tytthaspis_sedecimpunctata_584 | 0.172 |
| Hippodamia_alpina_383 | Tytthaspis_sedecimpunctata_584 | 0.172 |
| Psyllobora_renifer_551 | Tytthaspis_sedecimpunctata_584 | 0.172 |
| Psyllobora_renifer_552 | Tytthaspis_sedecimpunctata_584 | 0.172 |
| Psyllobora_renifer_553 | Tytthaspis_sedecimpunctata_584 | 0.172 |
| Psyllobora_renifer_554 | Tytthaspis_sedecimpunctata_584 | 0.172 |
| Coccinella_trifasciata_273 | Tytthaspis_sedecimpunctata_585 | 0.172 |
| Psyllobora_vigintimaculata_573 | Tytthaspis_sedecimpunctata_585 | 0.172 |
| Anatis_ocellata_143 | Tytthaspis_sedecimpunctata_586 | 0.172 |
| Scymnus_auritus_700 | Tytthaspis_sedecimpunctata_586 | 0.172 |
| Anatis_mali_128 | Tytthaspis_sedecimpunctata_587 | 0.172 |
| Anatis_mali_129 | Tytthaspis_sedecimpunctata_587 | 0.172 |
| Anatis_mali_130 | Tytthaspis_sedecimpunctata_587 | 0.172 |
| Anatis_mali_131 | Tytthaspis_sedecimpunctata_587 | 0.172 |
| Anatis_ocellata_133 | Tytthaspis_sedecimpunctata_587 | 0.172 |
| Anatis_ocellata_137 | Tytthaspis_sedecimpunctata_587 | 0.172 |
| Coccinella_hieroglyphica_226 | Tytthaspis_sedecimpunctata_588 | 0.172 |
| Coccinella_quinquepunctata_235 | Tytthaspis_sedecimpunctata_588 | 0.172 |
| Coccinella_quinquepunctata_238 | Tytthaspis_sedecimpunctata_588 | 0.172 |
| Coccinella_quinquepunctata_240 | Tytthaspis_sedecimpunctata_588 | 0.172 |
| Psyllobora_renifer_551 | Tytthaspis_sedecimpunctata_588 | 0.172 |
| Psyllobora_renifer_552 | Tytthaspis_sedecimpunctata_588 | 0.172 |
| Psyllobora_renifer_553 | Tytthaspis_sedecimpunctata_588 | 0.172 |
| Psyllobora_renifer_554 | Tytthaspis_sedecimpunctata_588 | 0.172 |
| Scymnus_auritus_700 | Tytthaspis_sedecimpunctata_588 | 0.172 |
| Psyllobora_vigintiduopunctata_571 | Tytthaspis_sedecimpunctata_589 | 0.172 |
| Anatis_ocellata_143 | Tytthaspis_sedecimpunctata_591 | 0.172 |
| Psyllobora_renifer_555 | Tytthaspis_sedecimpunctata_593 | 0.172 |
| Didion_punctatum_645 | Scymnus_rubromaculatus_822 | 0.172 |
| Didion_punctatum_645 | Scymnus_rubromaculatus_823 | 0.172 |
| Didion_punctatum_645 | Scymnus_rubromaculatus_824 | 0.172 |
| Didion_punctatum_645 | Scymnus_rubromaculatus_825 | 0.172 |
| Didion_punctatum_645 | Scymnus_rubromaculatus_826 | 0.172 |
| Didion_punctatum_645 | Scymnus_rubromaculatus_827 | 0.172 |
| Hyperaspis_reppensis_664 | Scymnus_suturalis_833 | 0.172 |
| Hyperaspis_reppensis_664 | Scymnus_suturalis_838 | 0.172 |

Supplementary Table S4 (continued)

| | | |
|---|---|---|
| Hyperaspis_reppensis_664 | Scymnus_suturalis_840 | 0.172 |
| Hyperaspis_proba_660 | Tytthaspis_sedecimpunctata_584 | 0.172 |
| Hyperaspis_reppensis_665 | Scymnus_suturalis_841 | 0.172 |
| Hyperaspis_reppensis_666 | Scymnus_suturalis_841 | 0.172 |
| Hyperaspis_reppensis_665 | Scymnus_suturalis_842 | 0.172 |
| Hyperaspis_reppensis_666 | Scymnus_suturalis_842 | 0.172 |
| Hyperaspis_reppensis_665 | Scymnus_suturalis_844 | 0.172 |
| Hyperaspis_reppensis_666 | Scymnus_suturalis_844 | 0.172 |
| Hyperaspis_reppensis_665 | Scymnus_suturalis_846 | 0.172 |
| Hyperaspis_reppensis_666 | Scymnus_suturalis_846 | 0.172 |
| Hyperaspis_reppensis_665 | Scymnus_suturalis_847 | 0.172 |
| Hyperaspis_reppensis_666 | Scymnus_suturalis_847 | 0.172 |
| Hyperaspis_reppensis_665 | Scymnus_suturalis_848 | 0.172 |
| Hyperaspis_reppensis_666 | Scymnus_suturalis_848 | 0.172 |
| Coccinella_quinquepunctata_232 | Tytthaspis_sedecimpunctata_577 | 0.172 |
| Coccinella_quinquepunctata_236 | Tytthaspis_sedecimpunctata_577 | 0.172 |
| Coccinella_quinquepunctata_239 | Tytthaspis_sedecimpunctata_577 | 0.172 |
| Hippodamia_alpina_388 | Tytthaspis_sedecimpunctata_577 | 0.172 |
| Illeis_galbula_474 | Tytthaspis_sedecimpunctata_577 | 0.172 |
| Illeis_galbula_475 | Tytthaspis_sedecimpunctata_577 | 0.172 |
| Illeis_galbula_476 | Tytthaspis_sedecimpunctata_577 | 0.172 |
| Coccinella_quinquepunctata_232 | Tytthaspis_sedecimpunctata_578 | 0.172 |
| Coccinella_quinquepunctata_236 | Tytthaspis_sedecimpunctata_578 | 0.172 |
| Coccinella_quinquepunctata_239 | Tytthaspis_sedecimpunctata_578 | 0.172 |
| Hippodamia_alpina_388 | Tytthaspis_sedecimpunctata_578 | 0.172 |
| Illeis_galbula_474 | Tytthaspis_sedecimpunctata_578 | 0.172 |
| Illeis_galbula_475 | Tytthaspis_sedecimpunctata_578 | 0.172 |
| Illeis_galbula_476 | Tytthaspis_sedecimpunctata_578 | 0.172 |
| Coccinella_quinquepunctata_232 | Tytthaspis_sedecimpunctata_579 | 0.172 |
| Coccinella_quinquepunctata_236 | Tytthaspis_sedecimpunctata_579 | 0.172 |
| Coccinella_quinquepunctata_239 | Tytthaspis_sedecimpunctata_579 | 0.172 |
| Hippodamia_alpina_388 | Tytthaspis_sedecimpunctata_579 | 0.172 |
| Illeis_galbula_474 | Tytthaspis_sedecimpunctata_579 | 0.172 |
| Illeis_galbula_475 | Tytthaspis_sedecimpunctata_579 | 0.172 |
| Illeis_galbula_476 | Tytthaspis_sedecimpunctata_579 | 0.172 |
| Hippodamia_alpina_385 | Tytthaspis_sedecimpunctata_580 | 0.172 |
| Hippodamia_alpina_386 | Tytthaspis_sedecimpunctata_580 | 0.172 |
| Hippodamia_alpina_387 | Tytthaspis_sedecimpunctata_580 | 0.172 |
| Coccinella_quinquepunctata_232 | Tytthaspis_sedecimpunctata_581 | 0.172 |
| Coccinella_quinquepunctata_236 | Tytthaspis_sedecimpunctata_581 | 0.172 |
| Coccinella_quinquepunctata_239 | Tytthaspis_sedecimpunctata_581 | 0.172 |
| Hippodamia_alpina_388 | Tytthaspis_sedecimpunctata_581 | 0.172 |
| Illeis_galbula_474 | Tytthaspis_sedecimpunctata_581 | 0.172 |
| Illeis_galbula_475 | Tytthaspis_sedecimpunctata_581 | 0.172 |
| Illeis_galbula_476 | Tytthaspis_sedecimpunctata_581 | 0.172 |
| Coccinella_quinquepunctata_232 | Tytthaspis_sedecimpunctata_582 | 0.172 |
| Coccinella_quinquepunctata_236 | Tytthaspis_sedecimpunctata_582 | 0.172 |
| Coccinella_quinquepunctata_239 | Tytthaspis_sedecimpunctata_582 | 0.172 |
| Hippodamia_alpina_388 | Tytthaspis_sedecimpunctata_582 | 0.172 |
| Illeis_galbula_474 | Tytthaspis_sedecimpunctata_582 | 0.172 |
| Illeis_galbula_475 | Tytthaspis_sedecimpunctata_582 | 0.172 |
| Illeis_galbula_476 | Tytthaspis_sedecimpunctata_582 | 0.172 |
| Coccinella_quinquepunctata_235 | Tytthaspis_sedecimpunctata_583 | 0.172 |
| Coccinella_quinquepunctata_238 | Tytthaspis_sedecimpunctata_583 | 0.172 |
| Coccinella_quinquepunctata_240 | Tytthaspis_sedecimpunctata_583 | 0.172 |
| Scymnus_auritus_698 | Tytthaspis_sedecimpunctata_583 | 0.172 |

| | | |
|---|---|---|
| Coccinella_quinquepunctata_232 | Tytthaspis_sedecimpunctata_585 | 0.172 |
| Coccinella_quinquepunctata_236 | Tytthaspis_sedecimpunctata_585 | 0.172 |
| Coccinella_quinquepunctata_239 | Tytthaspis_sedecimpunctata_585 | 0.172 |
| Hippodamia_alpina_388 | Tytthaspis_sedecimpunctata_585 | 0.172 |
| Psyllobora_vigintimaculata_574 | Tytthaspis_sedecimpunctata_585 | 0.172 |
| Scymnus_auritus_698 | Tytthaspis_sedecimpunctata_585 | 0.172 |
| Psyllobora_renifer_555 | Tytthaspis_sedecimpunctata_586 | 0.172 |
| Psyllobora_vigintiduopunctata_571 | Tytthaspis_sedecimpunctata_586 | 0.172 |
| Hippodamia_alpina_385 | Tytthaspis_sedecimpunctata_587 | 0.172 |
| Hippodamia_alpina_386 | Tytthaspis_sedecimpunctata_587 | 0.172 |
| Hippodamia_alpina_387 | Tytthaspis_sedecimpunctata_587 | 0.172 |
| Coccinella_quinquepunctata_232 | Tytthaspis_sedecimpunctata_590 | 0.172 |
| Coccinella_quinquepunctata_236 | Tytthaspis_sedecimpunctata_590 | 0.172 |
| Coccinella_quinquepunctata_239 | Tytthaspis_sedecimpunctata_590 | 0.172 |
| Hippodamia_alpina_388 | Tytthaspis_sedecimpunctata_590 | 0.172 |
| Illeis_galbula_474 | Tytthaspis_sedecimpunctata_590 | 0.172 |
| Illeis_galbula_475 | Tytthaspis_sedecimpunctata_590 | 0.172 |
| Illeis_galbula_476 | Tytthaspis_sedecimpunctata_590 | 0.172 |
| Psyllobora_vigintiduopunctata_571 | Tytthaspis_sedecimpunctata_591 | 0.172 |
| Coccinella_quinquepunctata_232 | Tytthaspis_sedecimpunctata_592 | 0.172 |
| Coccinella_quinquepunctata_236 | Tytthaspis_sedecimpunctata_592 | 0.172 |
| Coccinella_quinquepunctata_239 | Tytthaspis_sedecimpunctata_592 | 0.172 |
| Hippodamia_alpina_388 | Tytthaspis_sedecimpunctata_592 | 0.172 |
| Illeis_galbula_474 | Tytthaspis_sedecimpunctata_592 | 0.172 |
| Illeis_galbula_475 | Tytthaspis_sedecimpunctata_592 | 0.172 |
| Illeis_galbula_476 | Tytthaspis_sedecimpunctata_592 | 0.172 |
| Psyllobora_vigintimaculata_575 | Tytthaspis_sedecimpunctata_592 | 0.172 |
| Psyllobora_vigintimaculata_576 | Tytthaspis_sedecimpunctata_592 | 0.172 |
| Scymnus_auritus_698 | Tytthaspis_sedecimpunctata_592 | 0.172 |
| Hippodamia_alpina_388 | Tytthaspis_sedecimpunctata_593 | 0.172 |
| Coccinella_quinquepunctata_232 | Tytthaspis_sedecimpunctata_594 | 0.172 |
| Coccinella_quinquepunctata_236 | Tytthaspis_sedecimpunctata_594 | 0.172 |
| Coccinella_quinquepunctata_239 | Tytthaspis_sedecimpunctata_594 | 0.172 |
| Hippodamia_alpina_388 | Tytthaspis_sedecimpunctata_594 | 0.172 |
| Illeis_galbula_474 | Tytthaspis_sedecimpunctata_594 | 0.172 |
| Illeis_galbula_475 | Tytthaspis_sedecimpunctata_594 | 0.172 |
| Illeis_galbula_476 | Tytthaspis_sedecimpunctata_594 | 0.172 |
| Coccinella_quinquepunctata_232 | Tytthaspis_sedecimpunctata_595 | 0.172 |
| Coccinella_quinquepunctata_236 | Tytthaspis_sedecimpunctata_595 | 0.172 |
| Coccinella_quinquepunctata_239 | Tytthaspis_sedecimpunctata_595 | 0.172 |
| Hippodamia_alpina_388 | Tytthaspis_sedecimpunctata_595 | 0.172 |
| Illeis_galbula_474 | Tytthaspis_sedecimpunctata_595 | 0.172 |
| Illeis_galbula_475 | Tytthaspis_sedecimpunctata_595 | 0.172 |
| Illeis_galbula_476 | Tytthaspis_sedecimpunctata_595 | 0.172 |
| Hyperaspis_reppensis_665 | Scymnus_suturalis_832 | 0.172 |
| Hyperaspis_reppensis_666 | Scymnus_suturalis_832 | 0.172 |
| Brumus_quadripustulatus_17 | Scymnus_suturalis_833 | 0.172 |
| Scymnus_apetzi_691 | Scymnus_suturalis_833 | 0.172 |
| Scymnus_apetzi_696 | Scymnus_suturalis_833 | 0.172 |
| Hyperaspis_reppensis_664 | Scymnus_suturalis_834 | 0.172 |
| Hyperaspis_reppensis_665 | Scymnus_suturalis_835 | 0.172 |
| Hyperaspis_reppensis_666 | Scymnus_suturalis_835 | 0.172 |
| Hyperaspis_reppensis_665 | Scymnus_suturalis_836 | 0.172 |
| Hyperaspis_reppensis_666 | Scymnus_suturalis_836 | 0.172 |
| Hyperaspis_reppensis_665 | Scymnus_suturalis_839 | 0.172 |
| Hyperaspis_reppensis_666 | Scymnus_suturalis_839 | 0.172 |

| | | |
|---|---|---|
| Brumus_quadripustulatus_17 | Scymnus_suturalis_843 | 0.172 |
| Brumus_quadripustulatus_17 | Scymnus_suturalis_845 | 0.172 |
| Coccinella_quinquepunctata_232 | Tytthaspis_sedecimpunctata_580 | 0.172 |
| Coccinella_quinquepunctata_236 | Tytthaspis_sedecimpunctata_580 | 0.172 |
| Coccinella_quinquepunctata_239 | Tytthaspis_sedecimpunctata_580 | 0.172 |
| Psyllobora_vigintimaculata_575 | Tytthaspis_sedecimpunctata_580 | 0.172 |
| Psyllobora_vigintimaculata_576 | Tytthaspis_sedecimpunctata_580 | 0.172 |
| Scymnus_auritus_698 | Tytthaspis_sedecimpunctata_580 | 0.172 |
| Mulsantina_picta_484 | Tytthaspis_sedecimpunctata_585 | 0.172 |
| Psyllobora_vigintiduopunctata_571 | Tytthaspis_sedecimpunctata_585 | 0.172 |
| Illeis_galbula_474 | Tytthaspis_sedecimpunctata_586 | 0.172 |
| Illeis_galbula_475 | Tytthaspis_sedecimpunctata_586 | 0.172 |
| Illeis_galbula_476 | Tytthaspis_sedecimpunctata_586 | 0.172 |
| Scymnus_haemorrhoidalis_749 | Tytthaspis_sedecimpunctata_589 | 0.172 |
| Coccinella_quinquepunctata_235 | Tytthaspis_sedecimpunctata_591 | 0.172 |
| Coccinella_quinquepunctata_238 | Tytthaspis_sedecimpunctata_591 | 0.172 |
| Coccinella_quinquepunctata_240 | Tytthaspis_sedecimpunctata_591 | 0.172 |
| Hyperaspis_lateralis_657 | Scymnus_rubromaculatus_822 | 0.172 |
| Hyperaspis_lateralis_657 | Scymnus_rubromaculatus_823 | 0.172 |
| Hyperaspis_lateralis_657 | Scymnus_rubromaculatus_824 | 0.172 |
| Hyperaspis_lateralis_657 | Scymnus_rubromaculatus_825 | 0.172 |
| Hyperaspis_lateralis_657 | Scymnus_rubromaculatus_826 | 0.172 |
| Hyperaspis_lateralis_657 | Scymnus_rubromaculatus_827 | 0.172 |
| Brumus_quadripustulatus_12 | Scymnus_suturalis_833 | 0.172 |
| Coccidula_scutellata_81 | Scymnus_sinuanodulus_831 | 0.172 |
| Coccidula_scutellata_82 | Scymnus_sinuanodulus_831 | 0.172 |
| Brumus_quadripustulatus_15 | Scymnus_suturalis_841 | 0.172 |
| Brumus_quadripustulatus_16 | Scymnus_suturalis_841 | 0.172 |
| Brumus_quadripustulatus_6 | Scymnus_suturalis_841 | 0.172 |
| Brumus_quadripustulatus_8 | Scymnus_suturalis_841 | 0.172 |
| Brumus_quadripustulatus_15 | Scymnus_suturalis_842 | 0.172 |
| Brumus_quadripustulatus_16 | Scymnus_suturalis_842 | 0.172 |
| Brumus_quadripustulatus_6 | Scymnus_suturalis_842 | 0.172 |
| Brumus_quadripustulatus_8 | Scymnus_suturalis_842 | 0.172 |
| Brumus_quadripustulatus_12 | Scymnus_suturalis_843 | 0.172 |
| Brumus_quadripustulatus_15 | Scymnus_suturalis_844 | 0.172 |
| Brumus_quadripustulatus_16 | Scymnus_suturalis_844 | 0.172 |
| Brumus_quadripustulatus_6 | Scymnus_suturalis_844 | 0.172 |
| Brumus_quadripustulatus_8 | Scymnus_suturalis_844 | 0.172 |
| Brumus_quadripustulatus_12 | Scymnus_suturalis_845 | 0.172 |
| Brumus_quadripustulatus_15 | Scymnus_suturalis_846 | 0.172 |
| Brumus_quadripustulatus_16 | Scymnus_suturalis_846 | 0.172 |
| Brumus_quadripustulatus_6 | Scymnus_suturalis_846 | 0.172 |
| Brumus_quadripustulatus_8 | Scymnus_suturalis_846 | 0.172 |
| Brumus_quadripustulatus_15 | Scymnus_suturalis_847 | 0.172 |
| Brumus_quadripustulatus_16 | Scymnus_suturalis_847 | 0.172 |
| Brumus_quadripustulatus_6 | Scymnus_suturalis_847 | 0.172 |
| Brumus_quadripustulatus_8 | Scymnus_suturalis_847 | 0.172 |
| Brumus_quadripustulatus_15 | Scymnus_suturalis_848 | 0.172 |
| Brumus_quadripustulatus_16 | Scymnus_suturalis_848 | 0.172 |
| Brumus_quadripustulatus_6 | Scymnus_suturalis_848 | 0.172 |
| Brumus_quadripustulatus_8 | Scymnus_suturalis_848 | 0.172 |
| Hippodamia_notata_422 | Tytthaspis_sedecimpunctata_577 | 0.172 |
| Hippodamia_notata_423 | Tytthaspis_sedecimpunctata_577 | 0.172 |
| Hippodamia_notata_424 | Tytthaspis_sedecimpunctata_577 | 0.172 |
| Hippodamia_notata_426 | Tytthaspis_sedecimpunctata_577 | 0.172 |

Supplementary Table S4 (continued)

| | | |
|---|---|---|
| Hippodamia_notata_427 | Tytthaspis_sedecimpunctata_577 | 0.172 |
| Scymnus_auritus_699 | Tytthaspis_sedecimpunctata_577 | 0.172 |
| Hippodamia_notata_422 | Tytthaspis_sedecimpunctata_578 | 0.172 |
| Hippodamia_notata_423 | Tytthaspis_sedecimpunctata_578 | 0.172 |
| Hippodamia_notata_424 | Tytthaspis_sedecimpunctata_578 | 0.172 |
| Hippodamia_notata_426 | Tytthaspis_sedecimpunctata_578 | 0.172 |
| Hippodamia_notata_427 | Tytthaspis_sedecimpunctata_578 | 0.172 |
| Scymnus_auritus_699 | Tytthaspis_sedecimpunctata_578 | 0.172 |
| Hippodamia_notata_422 | Tytthaspis_sedecimpunctata_579 | 0.172 |
| Hippodamia_notata_423 | Tytthaspis_sedecimpunctata_579 | 0.172 |
| Hippodamia_notata_424 | Tytthaspis_sedecimpunctata_579 | 0.172 |
| Hippodamia_notata_426 | Tytthaspis_sedecimpunctata_579 | 0.172 |
| Hippodamia_notata_427 | Tytthaspis_sedecimpunctata_579 | 0.172 |
| Scymnus_auritus_699 | Tytthaspis_sedecimpunctata_579 | 0.172 |
| Hippodamia_notata_422 | Tytthaspis_sedecimpunctata_581 | 0.172 |
| Hippodamia_notata_423 | Tytthaspis_sedecimpunctata_581 | 0.172 |
| Hippodamia_notata_424 | Tytthaspis_sedecimpunctata_581 | 0.172 |
| Hippodamia_notata_426 | Tytthaspis_sedecimpunctata_581 | 0.172 |
| Hippodamia_notata_427 | Tytthaspis_sedecimpunctata_581 | 0.172 |
| Scymnus_auritus_699 | Tytthaspis_sedecimpunctata_581 | 0.172 |
| Hippodamia_notata_422 | Tytthaspis_sedecimpunctata_582 | 0.172 |
| Hippodamia_notata_423 | Tytthaspis_sedecimpunctata_582 | 0.172 |
| Hippodamia_notata_424 | Tytthaspis_sedecimpunctata_582 | 0.172 |
| Hippodamia_notata_426 | Tytthaspis_sedecimpunctata_582 | 0.172 |
| Hippodamia_notata_427 | Tytthaspis_sedecimpunctata_582 | 0.172 |
| Scymnus_auritus_699 | Tytthaspis_sedecimpunctata_582 | 0.172 |
| Cycloneda_sanguinea_340 | Tytthaspis_sedecimpunctata_585 | 0.172 |
| Cycloneda_sanguinea_341 | Tytthaspis_sedecimpunctata_585 | 0.172 |
| Cycloneda_sanguinea_343 | Tytthaspis_sedecimpunctata_585 | 0.172 |
| Cycloneda_sanguinea_344 | Tytthaspis_sedecimpunctata_585 | 0.172 |
| Hippodamia_notata_422 | Tytthaspis_sedecimpunctata_585 | 0.172 |
| Hippodamia_notata_423 | Tytthaspis_sedecimpunctata_585 | 0.172 |
| Hippodamia_notata_424 | Tytthaspis_sedecimpunctata_585 | 0.172 |
| Hippodamia_notata_426 | Tytthaspis_sedecimpunctata_585 | 0.172 |
| Hippodamia_notata_427 | Tytthaspis_sedecimpunctata_585 | 0.172 |
| Scymnus_haemorrhoidalis_750 | Tytthaspis_sedecimpunctata_586 | 0.172 |
| Scymnus_haemorrhoidalis_751 | Tytthaspis_sedecimpunctata_586 | 0.172 |
| Scymnus_haemorrhoidalis_754 | Tytthaspis_sedecimpunctata_586 | 0.172 |
| Scymnus_haemorrhoidalis_757 | Tytthaspis_sedecimpunctata_586 | 0.172 |
| Scymnus_haemorrhoidalis_758 | Tytthaspis_sedecimpunctata_586 | 0.172 |
| Scymnus_haemorrhoidalis_749 | Tytthaspis_sedecimpunctata_587 | 0.172 |
| Hippodamia_glacialis_407 | Tytthaspis_sedecimpunctata_589 | 0.172 |
| Hippodamia_notata_422 | Tytthaspis_sedecimpunctata_590 | 0.172 |
| Hippodamia_notata_423 | Tytthaspis_sedecimpunctata_590 | 0.172 |
| Hippodamia_notata_424 | Tytthaspis_sedecimpunctata_590 | 0.172 |
| Hippodamia_notata_426 | Tytthaspis_sedecimpunctata_590 | 0.172 |
| Hippodamia_notata_427 | Tytthaspis_sedecimpunctata_590 | 0.172 |
| Scymnus_auritus_699 | Tytthaspis_sedecimpunctata_590 | 0.172 |
| Coelophora_inaequalis_320 | Tytthaspis_sedecimpunctata_591 | 0.172 |
| Coelophora_inaequalis_321 | Tytthaspis_sedecimpunctata_591 | 0.172 |
| Coelophora_inaequalis_322 | Tytthaspis_sedecimpunctata_591 | 0.172 |
| Psyllobora_vigintiduopunctata_557 | Tytthaspis_sedecimpunctata_591 | 0.172 |
| Psyllobora_vigintiduopunctata_561 | Tytthaspis_sedecimpunctata_591 | 0.172 |
| Psyllobora_vigintiduopunctata_562 | Tytthaspis_sedecimpunctata_591 | 0.172 |
| Psyllobora_vigintiduopunctata_563 | Tytthaspis_sedecimpunctata_591 | 0.172 |
| Psyllobora_vigintiduopunctata_565 | Tytthaspis_sedecimpunctata_591 | 0.172 |

| | | |
|---|---|---|
| Psyllobora_vigintiduopunctata_567 | Tytthaspis_sedecimpunctata_591 | 0.172 |
| Hippodamia_notata_422 | Tytthaspis_sedecimpunctata_592 | 0.172 |
| Hippodamia_notata_423 | Tytthaspis_sedecimpunctata_592 | 0.172 |
| Hippodamia_notata_424 | Tytthaspis_sedecimpunctata_592 | 0.172 |
| Hippodamia_notata_426 | Tytthaspis_sedecimpunctata_592 | 0.172 |
| Hippodamia_notata_427 | Tytthaspis_sedecimpunctata_592 | 0.172 |
| Hippodamia_notata_422 | Tytthaspis_sedecimpunctata_593 | 0.172 |
| Hippodamia_notata_423 | Tytthaspis_sedecimpunctata_593 | 0.172 |
| Hippodamia_notata_424 | Tytthaspis_sedecimpunctata_593 | 0.172 |
| Hippodamia_notata_426 | Tytthaspis_sedecimpunctata_593 | 0.172 |
| Hippodamia_notata_427 | Tytthaspis_sedecimpunctata_593 | 0.172 |
| Hippodamia_notata_422 | Tytthaspis_sedecimpunctata_594 | 0.172 |
| Hippodamia_notata_423 | Tytthaspis_sedecimpunctata_594 | 0.172 |
| Hippodamia_notata_424 | Tytthaspis_sedecimpunctata_594 | 0.172 |
| Hippodamia_notata_426 | Tytthaspis_sedecimpunctata_594 | 0.172 |
| Hippodamia_notata_427 | Tytthaspis_sedecimpunctata_594 | 0.172 |
| Scymnus_auritus_699 | Tytthaspis_sedecimpunctata_594 | 0.172 |
| Hippodamia_notata_422 | Tytthaspis_sedecimpunctata_595 | 0.172 |
| Hippodamia_notata_423 | Tytthaspis_sedecimpunctata_595 | 0.172 |
| Hippodamia_notata_424 | Tytthaspis_sedecimpunctata_595 | 0.172 |
| Hippodamia_notata_426 | Tytthaspis_sedecimpunctata_595 | 0.172 |
| Hippodamia_notata_427 | Tytthaspis_sedecimpunctata_595 | 0.172 |
| Scymnus_auritus_699 | Tytthaspis_sedecimpunctata_595 | 0.172 |
| Hyperaspis_lateralis_655 | Scymnus_rubromaculatus_822 | 0.172 |
| Hyperaspis_lateralis_655 | Scymnus_rubromaculatus_823 | 0.172 |
| Hyperaspis_lateralis_655 | Scymnus_rubromaculatus_824 | 0.172 |
| Hyperaspis_lateralis_655 | Scymnus_rubromaculatus_825 | 0.172 |
| Hyperaspis_lateralis_655 | Scymnus_rubromaculatus_826 | 0.172 |
| Hyperaspis_lateralis_655 | Scymnus_rubromaculatus_827 | 0.172 |
| Brumus_quadripustulatus_15 | Scymnus_suturalis_832 | 0.172 |
| Brumus_quadripustulatus_16 | Scymnus_suturalis_832 | 0.172 |
| Brumus_quadripustulatus_6 | Scymnus_suturalis_832 | 0.172 |
| Brumus_quadripustulatus_8 | Scymnus_suturalis_832 | 0.172 |
| Nephus_quadrimaculatus_669 | Scymnus_suturalis_833 | 0.172 |
| Nephus_quadrimaculatus_670 | Scymnus_suturalis_833 | 0.172 |
| Nephus_quadrimaculatus_671 | Scymnus_suturalis_833 | 0.172 |
| Nephus_quadrimaculatus_672 | Scymnus_suturalis_833 | 0.172 |
| Nephus_quadrimaculatus_673 | Scymnus_suturalis_833 | 0.172 |
| Brumus_quadripustulatus_15 | Scymnus_suturalis_835 | 0.172 |
| Brumus_quadripustulatus_16 | Scymnus_suturalis_835 | 0.172 |
| Brumus_quadripustulatus_6 | Scymnus_suturalis_835 | 0.172 |
| Brumus_quadripustulatus_8 | Scymnus_suturalis_835 | 0.172 |
| Brumus_quadripustulatus_15 | Scymnus_suturalis_836 | 0.172 |
| Brumus_quadripustulatus_16 | Scymnus_suturalis_836 | 0.172 |
| Brumus_quadripustulatus_6 | Scymnus_suturalis_836 | 0.172 |
| Brumus_quadripustulatus_8 | Scymnus_suturalis_836 | 0.172 |
| Brumus_quadripustulatus_15 | Scymnus_suturalis_839 | 0.172 |
| Brumus_quadripustulatus_16 | Scymnus_suturalis_839 | 0.172 |
| Brumus_quadripustulatus_6 | Scymnus_suturalis_839 | 0.172 |
| Brumus_quadripustulatus_8 | Scymnus_suturalis_839 | 0.172 |
| Nephus_quadrimaculatus_669 | Scymnus_suturalis_841 | 0.172 |
| Nephus_quadrimaculatus_670 | Scymnus_suturalis_841 | 0.172 |
| Nephus_quadrimaculatus_671 | Scymnus_suturalis_841 | 0.172 |
| Nephus_quadrimaculatus_672 | Scymnus_suturalis_841 | 0.172 |
| Nephus_quadrimaculatus_673 | Scymnus_suturalis_841 | 0.172 |
| Nephus_quadrimaculatus_669 | Scymnus_suturalis_842 | 0.172 |

| | | |
|---|---|---|
| Nephus_quadrimaculatus_670 | Scymnus_suturalis_842 | 0.172 |
| Nephus_quadrimaculatus_671 | Scymnus_suturalis_842 | 0.172 |
| Nephus_quadrimaculatus_672 | Scymnus_suturalis_842 | 0.172 |
| Nephus_quadrimaculatus_673 | Scymnus_suturalis_842 | 0.172 |
| Nephus_quadrimaculatus_668 | Scymnus_suturalis_843 | 0.172 |
| Nephus_quadrimaculatus_669 | Scymnus_suturalis_844 | 0.172 |
| Nephus_quadrimaculatus_670 | Scymnus_suturalis_844 | 0.172 |
| Nephus_quadrimaculatus_671 | Scymnus_suturalis_844 | 0.172 |
| Nephus_quadrimaculatus_672 | Scymnus_suturalis_844 | 0.172 |
| Nephus_quadrimaculatus_673 | Scymnus_suturalis_844 | 0.172 |
| Nephus_quadrimaculatus_668 | Scymnus_suturalis_845 | 0.172 |
| Nephus_quadrimaculatus_669 | Scymnus_suturalis_846 | 0.172 |
| Nephus_quadrimaculatus_670 | Scymnus_suturalis_846 | 0.172 |
| Nephus_quadrimaculatus_671 | Scymnus_suturalis_846 | 0.172 |
| Nephus_quadrimaculatus_672 | Scymnus_suturalis_846 | 0.172 |
| Nephus_quadrimaculatus_673 | Scymnus_suturalis_846 | 0.172 |
| Nephus_quadrimaculatus_669 | Scymnus_suturalis_847 | 0.172 |
| Nephus_quadrimaculatus_670 | Scymnus_suturalis_847 | 0.172 |
| Nephus_quadrimaculatus_671 | Scymnus_suturalis_847 | 0.172 |
| Nephus_quadrimaculatus_672 | Scymnus_suturalis_847 | 0.172 |
| Nephus_quadrimaculatus_673 | Scymnus_suturalis_847 | 0.172 |
| Nephus_quadrimaculatus_669 | Scymnus_suturalis_848 | 0.172 |
| Nephus_quadrimaculatus_670 | Scymnus_suturalis_848 | 0.172 |
| Nephus_quadrimaculatus_671 | Scymnus_suturalis_848 | 0.172 |
| Nephus_quadrimaculatus_672 | Scymnus_suturalis_848 | 0.172 |
| Nephus_quadrimaculatus_673 | Scymnus_suturalis_848 | 0.172 |
| Hyperaspis_reppensis_664 | Subcoccinella_vigintiquatuorpunctata_623 | 0.172 |
| Hyperaspis_reppensis_666 | Subcoccinella_vigintiquatuorpunctata_623 | 0.172 |
| Hyperaspis_reppensis_667 | Subcoccinella_vigintiquatuorpunctata_623 | 0.172 |
| Hippodamia_notata_412 | Tytthaspis_sedecimpunctata_577 | 0.172 |
| Hippodamia_notata_414 | Tytthaspis_sedecimpunctata_577 | 0.172 |
| Hippodamia_notata_416 | Tytthaspis_sedecimpunctata_577 | 0.172 |
| Hippodamia_notata_417 | Tytthaspis_sedecimpunctata_577 | 0.172 |
| Hippodamia_notata_420 | Tytthaspis_sedecimpunctata_577 | 0.172 |
| Hippodamia_notata_425 | Tytthaspis_sedecimpunctata_577 | 0.172 |
| Hippodamia_notata_428 | Tytthaspis_sedecimpunctata_577 | 0.172 |
| Hippodamia_notata_430 | Tytthaspis_sedecimpunctata_577 | 0.172 |
| Hippodamia_notata_412 | Tytthaspis_sedecimpunctata_578 | 0.172 |
| Hippodamia_notata_414 | Tytthaspis_sedecimpunctata_578 | 0.172 |
| Hippodamia_notata_416 | Tytthaspis_sedecimpunctata_578 | 0.172 |
| Hippodamia_notata_417 | Tytthaspis_sedecimpunctata_578 | 0.172 |
| Hippodamia_notata_420 | Tytthaspis_sedecimpunctata_578 | 0.172 |
| Hippodamia_notata_425 | Tytthaspis_sedecimpunctata_578 | 0.172 |
| Hippodamia_notata_428 | Tytthaspis_sedecimpunctata_578 | 0.172 |
| Hippodamia_notata_430 | Tytthaspis_sedecimpunctata_578 | 0.172 |
| Hippodamia_notata_412 | Tytthaspis_sedecimpunctata_579 | 0.172 |
| Hippodamia_notata_414 | Tytthaspis_sedecimpunctata_579 | 0.172 |
| Hippodamia_notata_416 | Tytthaspis_sedecimpunctata_579 | 0.172 |
| Hippodamia_notata_417 | Tytthaspis_sedecimpunctata_579 | 0.172 |
| Hippodamia_notata_420 | Tytthaspis_sedecimpunctata_579 | 0.172 |
| Hippodamia_notata_425 | Tytthaspis_sedecimpunctata_579 | 0.172 |
| Hippodamia_notata_428 | Tytthaspis_sedecimpunctata_579 | 0.172 |
| Hippodamia_notata_430 | Tytthaspis_sedecimpunctata_579 | 0.172 |
| Hippodamia_notata_412 | Tytthaspis_sedecimpunctata_581 | 0.172 |
| Hippodamia_notata_414 | Tytthaspis_sedecimpunctata_581 | 0.172 |
| Hippodamia_notata_416 | Tytthaspis_sedecimpunctata_581 | 0.172 |

| | | |
|---|---|---|
| Hippodamia_notata_417 | Tytthaspis_sedecimpunctata_581 | 0.172 |
| Hippodamia_notata_420 | Tytthaspis_sedecimpunctata_581 | 0.172 |
| Hippodamia_notata_425 | Tytthaspis_sedecimpunctata_581 | 0.172 |
| Hippodamia_notata_428 | Tytthaspis_sedecimpunctata_581 | 0.172 |
| Hippodamia_notata_430 | Tytthaspis_sedecimpunctata_581 | 0.172 |
| Hippodamia_notata_412 | Tytthaspis_sedecimpunctata_582 | 0.172 |
| Hippodamia_notata_414 | Tytthaspis_sedecimpunctata_582 | 0.172 |
| Hippodamia_notata_416 | Tytthaspis_sedecimpunctata_582 | 0.172 |
| Hippodamia_notata_417 | Tytthaspis_sedecimpunctata_582 | 0.172 |
| Hippodamia_notata_420 | Tytthaspis_sedecimpunctata_582 | 0.172 |
| Hippodamia_notata_425 | Tytthaspis_sedecimpunctata_582 | 0.172 |
| Hippodamia_notata_428 | Tytthaspis_sedecimpunctata_582 | 0.172 |
| Hippodamia_notata_430 | Tytthaspis_sedecimpunctata_582 | 0.172 |
| Hippodamia_notata_415 | Tytthaspis_sedecimpunctata_583 | 0.172 |
| Hippodamia_glacialis_408 | Tytthaspis_sedecimpunctata_584 | 0.172 |
| Hippodamia_glacialis_409 | Tytthaspis_sedecimpunctata_584 | 0.172 |
| Hippodamia_notata_412 | Tytthaspis_sedecimpunctata_585 | 0.172 |
| Hippodamia_notata_414 | Tytthaspis_sedecimpunctata_585 | 0.172 |
| Hippodamia_notata_416 | Tytthaspis_sedecimpunctata_585 | 0.172 |
| Hippodamia_notata_417 | Tytthaspis_sedecimpunctata_585 | 0.172 |
| Hippodamia_notata_420 | Tytthaspis_sedecimpunctata_585 | 0.172 |
| Hippodamia_notata_425 | Tytthaspis_sedecimpunctata_585 | 0.172 |
| Hippodamia_notata_428 | Tytthaspis_sedecimpunctata_585 | 0.172 |
| Hippodamia_notata_430 | Tytthaspis_sedecimpunctata_585 | 0.172 |
| Scymnus_haemorrhoidalis_755 | Tytthaspis_sedecimpunctata_586 | 0.172 |
| Cycloneda_sanguinea_340 | Tytthaspis_sedecimpunctata_588 | 0.172 |
| Cycloneda_sanguinea_341 | Tytthaspis_sedecimpunctata_588 | 0.172 |
| Cycloneda_sanguinea_343 | Tytthaspis_sedecimpunctata_588 | 0.172 |
| Cycloneda_sanguinea_344 | Tytthaspis_sedecimpunctata_588 | 0.172 |
| Hippodamia_tredecimpunctata_457 | Tytthaspis_sedecimpunctata_589 | 0.172 |
| Hippodamia_tredecimpunctata_458 | Tytthaspis_sedecimpunctata_589 | 0.172 |
| Hippodamia_notata_412 | Tytthaspis_sedecimpunctata_590 | 0.172 |
| Hippodamia_notata_414 | Tytthaspis_sedecimpunctata_590 | 0.172 |
| Hippodamia_notata_416 | Tytthaspis_sedecimpunctata_590 | 0.172 |
| Hippodamia_notata_417 | Tytthaspis_sedecimpunctata_590 | 0.172 |
| Hippodamia_notata_420 | Tytthaspis_sedecimpunctata_590 | 0.172 |
| Hippodamia_notata_425 | Tytthaspis_sedecimpunctata_590 | 0.172 |
| Hippodamia_notata_428 | Tytthaspis_sedecimpunctata_590 | 0.172 |
| Hippodamia_notata_430 | Tytthaspis_sedecimpunctata_590 | 0.172 |
| Psyllobora_vigintiduopunctata_556 | Tytthaspis_sedecimpunctata_591 | 0.172 |
| Hippodamia_notata_412 | Tytthaspis_sedecimpunctata_592 | 0.172 |
| Hippodamia_notata_414 | Tytthaspis_sedecimpunctata_592 | 0.172 |
| Hippodamia_notata_416 | Tytthaspis_sedecimpunctata_592 | 0.172 |
| Hippodamia_notata_417 | Tytthaspis_sedecimpunctata_592 | 0.172 |
| Hippodamia_notata_420 | Tytthaspis_sedecimpunctata_592 | 0.172 |
| Hippodamia_notata_425 | Tytthaspis_sedecimpunctata_592 | 0.172 |
| Hippodamia_notata_428 | Tytthaspis_sedecimpunctata_592 | 0.172 |
| Hippodamia_notata_430 | Tytthaspis_sedecimpunctata_592 | 0.172 |
| Hippodamia_notata_412 | Tytthaspis_sedecimpunctata_593 | 0.172 |
| Hippodamia_notata_414 | Tytthaspis_sedecimpunctata_593 | 0.172 |
| Hippodamia_notata_416 | Tytthaspis_sedecimpunctata_593 | 0.172 |
| Hippodamia_notata_417 | Tytthaspis_sedecimpunctata_593 | 0.172 |
| Hippodamia_notata_420 | Tytthaspis_sedecimpunctata_593 | 0.172 |
| Hippodamia_notata_425 | Tytthaspis_sedecimpunctata_593 | 0.172 |
| Hippodamia_notata_428 | Tytthaspis_sedecimpunctata_593 | 0.172 |
| Hippodamia_notata_430 | Tytthaspis_sedecimpunctata_593 | 0.172 |

| | | |
|---|---|---|
| Psyllobora_vigintiduopunctata_570 | Tytthaspis_sedecimpunctata_593 | 0.172 |
| Psyllobora_vigintiduopunctata_572 | Tytthaspis_sedecimpunctata_593 | 0.172 |
| Hippodamia_notata_412 | Tytthaspis_sedecimpunctata_594 | 0.172 |
| Hippodamia_notata_414 | Tytthaspis_sedecimpunctata_594 | 0.172 |
| Hippodamia_notata_416 | Tytthaspis_sedecimpunctata_594 | 0.172 |
| Hippodamia_notata_417 | Tytthaspis_sedecimpunctata_594 | 0.172 |
| Hippodamia_notata_420 | Tytthaspis_sedecimpunctata_594 | 0.172 |
| Hippodamia_notata_425 | Tytthaspis_sedecimpunctata_594 | 0.172 |
| Hippodamia_notata_428 | Tytthaspis_sedecimpunctata_594 | 0.172 |
| Hippodamia_notata_430 | Tytthaspis_sedecimpunctata_594 | 0.172 |
| Hippodamia_notata_412 | Tytthaspis_sedecimpunctata_595 | 0.172 |
| Hippodamia_notata_414 | Tytthaspis_sedecimpunctata_595 | 0.172 |
| Hippodamia_notata_416 | Tytthaspis_sedecimpunctata_595 | 0.172 |
| Hippodamia_notata_417 | Tytthaspis_sedecimpunctata_595 | 0.172 |
| Hippodamia_notata_420 | Tytthaspis_sedecimpunctata_595 | 0.172 |
| Hippodamia_notata_425 | Tytthaspis_sedecimpunctata_595 | 0.172 |
| Hippodamia_notata_428 | Tytthaspis_sedecimpunctata_595 | 0.172 |
| Hippodamia_notata_430 | Tytthaspis_sedecimpunctata_595 | 0.172 |
| Hyperaspis_oregona_659 | Scymnus_rubromaculatus_822 | 0.172 |
| Hyperaspis_oregona_659 | Scymnus_rubromaculatus_823 | 0.172 |
| Hyperaspis_oregona_659 | Scymnus_rubromaculatus_824 | 0.172 |
| Hyperaspis_oregona_659 | Scymnus_rubromaculatus_825 | 0.172 |
| Hyperaspis_oregona_659 | Scymnus_rubromaculatus_826 | 0.172 |
| Hyperaspis_oregona_659 | Scymnus_rubromaculatus_827 | 0.172 |
| Nephus_quadrimaculatus_669 | Scymnus_suturalis_832 | 0.172 |
| Nephus_quadrimaculatus_670 | Scymnus_suturalis_832 | 0.172 |
| Nephus_quadrimaculatus_671 | Scymnus_suturalis_832 | 0.172 |
| Nephus_quadrimaculatus_672 | Scymnus_suturalis_832 | 0.172 |
| Nephus_quadrimaculatus_673 | Scymnus_suturalis_832 | 0.172 |
| Nephus_quadrimaculatus_668 | Scymnus_suturalis_834 | 0.172 |
| Nephus_quadrimaculatus_669 | Scymnus_suturalis_835 | 0.172 |
| Nephus_quadrimaculatus_670 | Scymnus_suturalis_835 | 0.172 |
| Nephus_quadrimaculatus_671 | Scymnus_suturalis_835 | 0.172 |
| Nephus_quadrimaculatus_672 | Scymnus_suturalis_835 | 0.172 |
| Nephus_quadrimaculatus_673 | Scymnus_suturalis_835 | 0.172 |
| Nephus_quadrimaculatus_669 | Scymnus_suturalis_836 | 0.172 |
| Nephus_quadrimaculatus_670 | Scymnus_suturalis_836 | 0.172 |
| Nephus_quadrimaculatus_671 | Scymnus_suturalis_836 | 0.172 |
| Nephus_quadrimaculatus_672 | Scymnus_suturalis_836 | 0.172 |
| Nephus_quadrimaculatus_673 | Scymnus_suturalis_836 | 0.172 |
| Nephus_quadrimaculatus_669 | Scymnus_suturalis_837 | 0.172 |
| Nephus_quadrimaculatus_670 | Scymnus_suturalis_837 | 0.172 |
| Nephus_quadrimaculatus_671 | Scymnus_suturalis_837 | 0.172 |
| Nephus_quadrimaculatus_672 | Scymnus_suturalis_837 | 0.172 |
| Nephus_quadrimaculatus_673 | Scymnus_suturalis_837 | 0.172 |
| Nephus_quadrimaculatus_669 | Scymnus_suturalis_839 | 0.172 |
| Nephus_quadrimaculatus_670 | Scymnus_suturalis_839 | 0.172 |
| Nephus_quadrimaculatus_671 | Scymnus_suturalis_839 | 0.172 |
| Nephus_quadrimaculatus_672 | Scymnus_suturalis_839 | 0.172 |
| Nephus_quadrimaculatus_673 | Scymnus_suturalis_839 | 0.172 |
| Didion_punctatum_646 | Scymnus_sinuanodulus_831 | 0.172 |
| Hyperaspis_reppensis_664 | Subcoccinella_vigintiquatuorpunctata_620 | 0.172 |
| Hyperaspis_reppensis_666 | Subcoccinella_vigintiquatuorpunctata_620 | 0.172 |
| Hyperaspis_reppensis_667 | Subcoccinella_vigintiquatuorpunctata_620 | 0.172 |
| Hyperaspis_reppensis_664 | Subcoccinella_vigintiquatuorpunctata_621 | 0.172 |
| Hyperaspis_reppensis_666 | Subcoccinella_vigintiquatuorpunctata_621 | 0.172 |

| | | |
|---|---|---|
| Hyperaspis_reppensis_667 | Subcoccinella_vigintiquatuorpunctata_621 | 0.172 |
| Hyperaspis_reppensis_665 | Subcoccinella_vigintiquatuorpunctata_622 | 0.172 |
| Hyperaspis_reppensis_665 | Subcoccinella_vigintiquatuorpunctata_627 | 0.172 |
| Hyperaspis_reppensis_664 | Subcoccinella_vigintiquatuorpunctata_628 | 0.172 |
| Hyperaspis_reppensis_666 | Subcoccinella_vigintiquatuorpunctata_628 | 0.172 |
| Hyperaspis_reppensis_667 | Subcoccinella_vigintiquatuorpunctata_628 | 0.172 |
| Hyperaspis_reppensis_664 | Subcoccinella_vigintiquatuorpunctata_629 | 0.172 |
| Hyperaspis_reppensis_666 | Subcoccinella_vigintiquatuorpunctata_629 | 0.172 |
| Hyperaspis_reppensis_667 | Subcoccinella_vigintiquatuorpunctata_629 | 0.172 |
| Hyperaspis_reppensis_664 | Subcoccinella_vigintiquatuorpunctata_631 | 0.172 |
| Hyperaspis_reppensis_666 | Subcoccinella_vigintiquatuorpunctata_631 | 0.172 |
| Hyperaspis_reppensis_667 | Subcoccinella_vigintiquatuorpunctata_631 | 0.172 |
| Hippodamia_glacialis_407 | Tytthaspis_sedecimpunctata_583 | 0.172 |
| Hippodamia_notata_413 | Tytthaspis_sedecimpunctata_583 | 0.172 |
| Hippodamia_notata_418 | Tytthaspis_sedecimpunctata_583 | 0.172 |
| Hippodamia_notata_415 | Tytthaspis_sedecimpunctata_584 | 0.172 |
| Psyllobora_vigintiduopunctata_560 | Tytthaspis_sedecimpunctata_584 | 0.172 |
| Hippodamia_glacialis_407 | Tytthaspis_sedecimpunctata_585 | 0.172 |
| Ceratomegilla_ulkei_220 | Tytthaspis_sedecimpunctata_586 | 0.172 |
| Ceratomegilla_ulkei_220 | Tytthaspis_sedecimpunctata_587 | 0.172 |
| Ceratomegilla_ulkei_221 | Tytthaspis_sedecimpunctata_588 | 0.172 |
| Hippodamia_convergens_396 | Tytthaspis_sedecimpunctata_589 | 0.172 |
| Hippodamia_convergens_400 | Tytthaspis_sedecimpunctata_589 | 0.172 |
| Hippodamia_convergens_403 | Tytthaspis_sedecimpunctata_589 | 0.172 |
| Hippodamia_moesta_410 | Tytthaspis_sedecimpunctata_589 | 0.172 |
| Hippodamia_glacialis_407 | Tytthaspis_sedecimpunctata_592 | 0.172 |
| Nephus_quadrimaculatus_668 | Scymnus_suturalis_840 | 0.172 |
| Didion_punctatum_644 | Scymnus_sinuanodulus_831 | 0.172 |
| Scymnus_apetzi_693 | Scymnus_sinuanodulus_828 | 0.172 |
| Brumus_quadripustulatus_10 | Tytthaspis_sedecimpunctata_589 | 0.172 |
| Hippodamia_notata_413 | Tytthaspis_sedecimpunctata_584 | 0.172 |
| Hippodamia_notata_418 | Tytthaspis_sedecimpunctata_584 | 0.172 |
| Hippodamia_caseyi_391 | Tytthaspis_sedecimpunctata_586 | 0.172 |
| Hippodamia_convergens_393 | Tytthaspis_sedecimpunctata_586 | 0.172 |
| Hippodamia_convergens_394 | Tytthaspis_sedecimpunctata_586 | 0.172 |
| Hippodamia_convergens_397 | Tytthaspis_sedecimpunctata_586 | 0.172 |
| Hippodamia_convergens_398 | Tytthaspis_sedecimpunctata_586 | 0.172 |
| Hippodamia_convergens_399 | Tytthaspis_sedecimpunctata_586 | 0.172 |
| Hippodamia_convergens_401 | Tytthaspis_sedecimpunctata_586 | 0.172 |
| Hippodamia_convergens_402 | Tytthaspis_sedecimpunctata_586 | 0.172 |
| Hippodamia_convergens_404 | Tytthaspis_sedecimpunctata_586 | 0.172 |
| Hippodamia_convergens_405 | Tytthaspis_sedecimpunctata_586 | 0.172 |
| Hippodamia_convergens_406 | Tytthaspis_sedecimpunctata_586 | 0.172 |
| Hippodamia_moesta_411 | Tytthaspis_sedecimpunctata_586 | 0.172 |
| Hippodamia_quinquesignata_443 | Tytthaspis_sedecimpunctata_586 | 0.172 |
| Hippodamia_quinquesignata_444 | Tytthaspis_sedecimpunctata_586 | 0.172 |
| Hippodamia_quinquesignata_445 | Tytthaspis_sedecimpunctata_586 | 0.172 |
| Hippodamia_convergens_396 | Tytthaspis_sedecimpunctata_587 | 0.172 |
| Hippodamia_convergens_400 | Tytthaspis_sedecimpunctata_587 | 0.172 |
| Hippodamia_convergens_403 | Tytthaspis_sedecimpunctata_587 | 0.172 |
| Hippodamia_moesta_410 | Tytthaspis_sedecimpunctata_587 | 0.172 |
| Hippodamia_caseyi_392 | Tytthaspis_sedecimpunctata_588 | 0.172 |
| Hippodamia_convergens_395 | Tytthaspis_sedecimpunctata_588 | 0.172 |
| Ceratomegilla_ulkei_221 | Tytthaspis_sedecimpunctata_589 | 0.172 |
| Coccinula_quatuordecimpustulata_297 | Tytthaspis_sedecimpunctata_589 | 0.172 |
| Hippodamia_tredecimpunctata_446 | Tytthaspis_sedecimpunctata_589 | 0.172 |

| | | |
|---|---|---|
| Hippodamia_tredecimpunctata_448 | Tytthaspis_sedecimpunctata_589 | 0.172 |
| Hippodamia_tredecimpunctata_449 | Tytthaspis_sedecimpunctata_589 | 0.172 |
| Hippodamia_tredecimpunctata_450 | Tytthaspis_sedecimpunctata_589 | 0.172 |
| Hippodamia_tredecimpunctata_452 | Tytthaspis_sedecimpunctata_589 | 0.172 |
| Hippodamia_tredecimpunctata_453 | Tytthaspis_sedecimpunctata_589 | 0.172 |
| Brumus_quadripustulatus_10 | Tytthaspis_sedecimpunctata_588 | 0.172 |
| Brumus_quadripustulatus_10 | Tytthaspis_sedecimpunctata_591 | 0.172 |
| Psyllobora_vigintiduopunctata_569 | Tytthaspis_sedecimpunctata_591 | 0.173 |
| Diomus_terminatus_647 | Scymnus_suturalis_841 | 0.173 |
| Diomus_terminatus_648 | Scymnus_suturalis_841 | 0.173 |
| Diomus_terminatus_647 | Scymnus_suturalis_842 | 0.173 |
| Diomus_terminatus_648 | Scymnus_suturalis_842 | 0.173 |
| Diomus_terminatus_647 | Scymnus_suturalis_844 | 0.173 |
| Diomus_terminatus_648 | Scymnus_suturalis_844 | 0.173 |
| Diomus_terminatus_647 | Scymnus_suturalis_846 | 0.173 |
| Diomus_terminatus_648 | Scymnus_suturalis_846 | 0.173 |
| Diomus_terminatus_647 | Scymnus_suturalis_847 | 0.173 |
| Diomus_terminatus_648 | Scymnus_suturalis_847 | 0.173 |
| Diomus_terminatus_647 | Scymnus_suturalis_848 | 0.173 |
| Diomus_terminatus_648 | Scymnus_suturalis_848 | 0.173 |
| Ceratomegilla_ulkei_219 | Tytthaspis_sedecimpunctata_584 | 0.173 |
| Ceratomegilla_ulkei_219 | Tytthaspis_sedecimpunctata_591 | 0.173 |
| Coccinula_quatuordecimpustulata_297 | Tytthaspis_sedecimpunctata_591 | 0.173 |
| Diomus_terminatus_647 | Scymnus_suturalis_832 | 0.173 |
| Diomus_terminatus_648 | Scymnus_suturalis_832 | 0.173 |
| Diomus_terminatus_647 | Scymnus_suturalis_835 | 0.173 |
| Diomus_terminatus_648 | Scymnus_suturalis_835 | 0.173 |
| Diomus_terminatus_647 | Scymnus_suturalis_836 | 0.173 |
| Diomus_terminatus_648 | Scymnus_suturalis_836 | 0.173 |
| Diomus_terminatus_647 | Scymnus_suturalis_837 | 0.173 |
| Diomus_terminatus_648 | Scymnus_suturalis_837 | 0.173 |
| Diomus_terminatus_647 | Scymnus_suturalis_839 | 0.173 |
| Diomus_terminatus_648 | Scymnus_suturalis_839 | 0.173 |
| Hippodamia_caseyi_392 | Tytthaspis_sedecimpunctata_591 | 0.173 |
| Hippodamia_convergens_395 | Tytthaspis_sedecimpunctata_591 | 0.173 |
| Hippodamia_tredecimpunctata_446 | Tytthaspis_sedecimpunctata_593 | 0.173 |
| Hippodamia_tredecimpunctata_448 | Tytthaspis_sedecimpunctata_593 | 0.173 |
| Hippodamia_tredecimpunctata_449 | Tytthaspis_sedecimpunctata_593 | 0.173 |
| Hippodamia_tredecimpunctata_450 | Tytthaspis_sedecimpunctata_593 | 0.173 |
| Hippodamia_tredecimpunctata_452 | Tytthaspis_sedecimpunctata_593 | 0.173 |
| Hippodamia_tredecimpunctata_453 | Tytthaspis_sedecimpunctata_593 | 0.173 |
| Scymnus_mimulus_775 | Scymnus_sinuanodulus_830 | 0.173 |
| Scymnus_mimulus_776 | Scymnus_sinuanodulus_830 | 0.173 |
| Diomus_terminatus_647 | Scymnus_suturalis_833 | 0.173 |
| Diomus_terminatus_648 | Scymnus_suturalis_833 | 0.173 |
| Hyperaspis_reppensis_665 | Subcoccinella_vigintiquatuorpunctata_619 | 0.173 |
| Brumus_quadripustulatus_10 | Scymnus_suturalis_841 | 0.173 |
| Brumus_quadripustulatus_10 | Scymnus_suturalis_842 | 0.173 |
| Brumus_quadripustulatus_10 | Scymnus_suturalis_844 | 0.173 |
| Brumus_quadripustulatus_10 | Scymnus_suturalis_846 | 0.173 |
| Brumus_quadripustulatus_10 | Scymnus_suturalis_847 | 0.173 |
| Brumus_quadripustulatus_10 | Scymnus_suturalis_848 | 0.173 |
| Brumus_quadripustulatus_10 | Scymnus_suturalis_832 | 0.173 |
| Brumus_quadripustulatus_10 | Scymnus_suturalis_835 | 0.173 |
| Brumus_quadripustulatus_10 | Scymnus_suturalis_836 | 0.173 |
| Brumus_quadripustulatus_10 | Scymnus_suturalis_839 | 0.173 |

| | | |
|---|---|---|
| Coccidula_scutellata_81 | Scymnus_sinuanodulus_829 | 0.173 |
| Coccidula_scutellata_82 | Scymnus_sinuanodulus_829 | 0.173 |
| Coccinula_quatuordecimpustulata_306 | Tytthaspis_sedecimpunctata_587 | 0.173 |
| Scymnus_mitior_779 | Scymnus_sinuanodulus_828 | 0.173 |
| Hippodamia_arctica_390 | Tytthaspis_sedecimpunctata_589 | 0.173 |
| Calvia_decemguttata_187 | Tytthaspis_sedecimpunctata_583 | 0.173 |
| Calvia_decemguttata_188 | Tytthaspis_sedecimpunctata_583 | 0.173 |
| Calvia_decemguttata_189 | Tytthaspis_sedecimpunctata_583 | 0.173 |
| Calvia_decemguttata_190 | Tytthaspis_sedecimpunctata_583 | 0.173 |
| Calvia_decemguttata_191 | Tytthaspis_sedecimpunctata_583 | 0.173 |
| Calvia_decemguttata_192 | Tytthaspis_sedecimpunctata_583 | 0.173 |
| Calvia_decemguttata_196 | Tytthaspis_sedecimpunctata_583 | 0.173 |
| Calvia_decemguttata_197 | Tytthaspis_sedecimpunctata_583 | 0.173 |
| Calvia_decemguttata_198 | Tytthaspis_sedecimpunctata_583 | 0.173 |
| Coccinula_quatuordecimpustulata_298 | Tytthaspis_sedecimpunctata_587 | 0.173 |
| Coccinula_quatuordecimpustulata_299 | Tytthaspis_sedecimpunctata_587 | 0.173 |
| Coccinula_quatuordecimpustulata_301 | Tytthaspis_sedecimpunctata_587 | 0.173 |
| Coccinula_quatuordecimpustulata_302 | Tytthaspis_sedecimpunctata_587 | 0.173 |
| Coccinula_quatuordecimpustulata_303 | Tytthaspis_sedecimpunctata_587 | 0.173 |
| Coccinula_quatuordecimpustulata_309 | Tytthaspis_sedecimpunctata_587 | 0.173 |
| Coccinula_quatuordecimpustulata_300 | Tytthaspis_sedecimpunctata_591 | 0.173 |
| Anatis_mali_128 | Vibidia_duodecimguttata_597 | 0.173 |
| Anatis_mali_129 | Vibidia_duodecimguttata_597 | 0.173 |
| Anatis_mali_130 | Vibidia_duodecimguttata_597 | 0.173 |
| Anatis_mali_131 | Vibidia_duodecimguttata_597 | 0.173 |
| Anatis_mali_128 | Vibidia_duodecimguttata_600 | 0.173 |
| Anatis_mali_129 | Vibidia_duodecimguttata_600 | 0.173 |
| Anatis_mali_130 | Vibidia_duodecimguttata_600 | 0.173 |
| Anatis_mali_131 | Vibidia_duodecimguttata_600 | 0.173 |
| Calvia_decemguttata_195 | Vibidia_duodecimguttata_603 | 0.173 |
| Scymnus_mitior_779 | Scymnus_sinuanodulus_830 | 0.173 |
| Didion_punctatum_646 | Scymnus_sinuanodulus_829 | 0.173 |
| Hyperaspis_reppensis_664 | Scymnus_sinuanodulus_831 | 0.173 |
| Hyperaspis_reppensis_667 | Scymnus_sinuanodulus_831 | 0.173 |
| Scymnus_haemorrhoidalis_756 | Scymnus_sinuanodulus_831 | 0.173 |
| Calvia_decemguttata_187 | Tytthaspis_sedecimpunctata_588 | 0.173 |
| Calvia_decemguttata_188 | Tytthaspis_sedecimpunctata_588 | 0.173 |
| Calvia_decemguttata_189 | Tytthaspis_sedecimpunctata_588 | 0.173 |
| Calvia_decemguttata_190 | Tytthaspis_sedecimpunctata_588 | 0.173 |
| Calvia_decemguttata_191 | Tytthaspis_sedecimpunctata_588 | 0.173 |
| Calvia_decemguttata_192 | Tytthaspis_sedecimpunctata_588 | 0.173 |
| Calvia_decemguttata_196 | Tytthaspis_sedecimpunctata_588 | 0.173 |
| Calvia_decemguttata_197 | Tytthaspis_sedecimpunctata_588 | 0.173 |
| Calvia_decemguttata_198 | Tytthaspis_sedecimpunctata_588 | 0.173 |
| Anatis_mali_128 | Vibidia_duodecimguttata_598 | 0.173 |
| Anatis_mali_129 | Vibidia_duodecimguttata_598 | 0.173 |
| Anatis_mali_130 | Vibidia_duodecimguttata_598 | 0.173 |
| Anatis_mali_131 | Vibidia_duodecimguttata_598 | 0.173 |
| Anatis_mali_128 | Vibidia_duodecimguttata_599 | 0.173 |
| Anatis_mali_129 | Vibidia_duodecimguttata_599 | 0.173 |
| Anatis_mali_130 | Vibidia_duodecimguttata_599 | 0.173 |
| Anatis_mali_131 | Vibidia_duodecimguttata_599 | 0.173 |
| Anatis_mali_128 | Vibidia_duodecimguttata_601 | 0.173 |
| Anatis_mali_129 | Vibidia_duodecimguttata_601 | 0.173 |
| Anatis_mali_130 | Vibidia_duodecimguttata_601 | 0.173 |
| Anatis_mali_131 | Vibidia_duodecimguttata_601 | 0.173 |

Supplementary Table S4 (continued)

| | | |
|---|---|---|
| Anatis_mali_128 | Vibidia_duodecimguttata_602 | 0.173 |
| Anatis_mali_129 | Vibidia_duodecimguttata_602 | 0.173 |
| Anatis_mali_130 | Vibidia_duodecimguttata_602 | 0.173 |
| Anatis_mali_131 | Vibidia_duodecimguttata_602 | 0.173 |
| Scymnus_auritus_702 | Scymnus_sinuanodulus_828 | 0.173 |
| Coccinella_undecimpunctata_287 | Tytthaspis_sedecimpunctata_589 | 0.173 |
| Didion_punctatum_644 | Scymnus_sinuanodulus_829 | 0.173 |
| Scymnus_apetzi_691 | Scymnus_sinuanodulus_828 | 0.173 |
| Psyllobora_vigintiduopunctata_559 | Vibidia_duodecimguttata_597 | 0.173 |
| Psyllobora_vigintiduopunctata_559 | Vibidia_duodecimguttata_600 | 0.173 |
| Scymnus_apetzi_690 | Scymnus_sinuanodulus_830 | 0.173 |
| Scymnus_apetzi_697 | Scymnus_sinuanodulus_830 | 0.173 |
| Platynaspis_luteorubra_50 | Tytthaspis_sedecimpunctata_577 | 0.173 |
| Platynaspis_luteorubra_50 | Tytthaspis_sedecimpunctata_578 | 0.173 |
| Platynaspis_luteorubra_50 | Tytthaspis_sedecimpunctata_579 | 0.173 |
| Platynaspis_luteorubra_50 | Tytthaspis_sedecimpunctata_581 | 0.173 |
| Platynaspis_luteorubra_50 | Tytthaspis_sedecimpunctata_582 | 0.173 |
| Platynaspis_luteorubra_50 | Tytthaspis_sedecimpunctata_585 | 0.173 |
| Platynaspis_luteorubra_50 | Tytthaspis_sedecimpunctata_590 | 0.173 |
| Platynaspis_luteorubra_50 | Tytthaspis_sedecimpunctata_592 | 0.173 |
| Platynaspis_luteorubra_50 | Tytthaspis_sedecimpunctata_593 | 0.173 |
| Platynaspis_luteorubra_50 | Tytthaspis_sedecimpunctata_594 | 0.173 |
| Platynaspis_luteorubra_50 | Tytthaspis_sedecimpunctata_595 | 0.173 |
| Platynaspis_luteorubra_50 | Tytthaspis_sedecimpunctata_588 | 0.173 |
| Psyllobora_vigintiduopunctata_569 | Vibidia_duodecimguttata_596 | 0.173 |
| Psyllobora_vigintiduopunctata_559 | Vibidia_duodecimguttata_598 | 0.173 |
| Psyllobora_vigintiduopunctata_559 | Vibidia_duodecimguttata_603 | 0.173 |
| Brumus_quadripustulatus_5 | Tytthaspis_sedecimpunctata_587 | 0.173 |
| Scymnus_auritus_701 | Scymnus_sinuanodulus_831 | 0.173 |
| Hyperaspis_reppensis_664 | Scymnus_sinuanodulus_829 | 0.173 |
| Hyperaspis_reppensis_667 | Scymnus_sinuanodulus_829 | 0.173 |
| Scymnus_haemorrhoidalis_756 | Scymnus_sinuanodulus_829 | 0.173 |
| Calvia_quatuordecimguttata_206 | Vibidia_duodecimguttata_597 | 0.173 |
| Calvia_quatuordecimguttata_207 | Vibidia_duodecimguttata_597 | 0.173 |
| Calvia_quatuordecimguttata_216 | Vibidia_duodecimguttata_597 | 0.173 |
| Myrrha_octodecimguttata_489 | Vibidia_duodecimguttata_598 | 0.173 |
| Myrrha_octodecimguttata_500 | Vibidia_duodecimguttata_598 | 0.173 |
| Calvia_quatuordecimguttata_206 | Vibidia_duodecimguttata_600 | 0.173 |
| Calvia_quatuordecimguttata_207 | Vibidia_duodecimguttata_600 | 0.173 |
| Calvia_quatuordecimguttata_216 | Vibidia_duodecimguttata_600 | 0.173 |
| Myrrha_octodecimguttata_489 | Vibidia_duodecimguttata_601 | 0.173 |
| Myrrha_octodecimguttata_500 | Vibidia_duodecimguttata_601 | 0.173 |
| Calvia_quatuordecimguttata_205 | Vibidia_duodecimguttata_596 | 0.174 |
| Calvia_quatuordecimguttata_209 | Vibidia_duodecimguttata_598 | 0.174 |
| Calvia_quatuordecimguttata_218 | Vibidia_duodecimguttata_598 | 0.174 |
| Calvia_quatuordecimguttata_209 | Vibidia_duodecimguttata_599 | 0.174 |
| Calvia_quatuordecimguttata_218 | Vibidia_duodecimguttata_599 | 0.174 |
| Calvia_quatuordecimguttata_209 | Vibidia_duodecimguttata_601 | 0.174 |
| Calvia_quatuordecimguttata_218 | Vibidia_duodecimguttata_601 | 0.174 |
| Calvia_quatuordecimguttata_209 | Vibidia_duodecimguttata_602 | 0.174 |
| Calvia_quatuordecimguttata_218 | Vibidia_duodecimguttata_602 | 0.174 |
| Calvia_quatuordecimguttata_206 | Vibidia_duodecimguttata_603 | 0.174 |
| Calvia_quatuordecimguttata_207 | Vibidia_duodecimguttata_603 | 0.174 |
| Calvia_quatuordecimguttata_216 | Vibidia_duodecimguttata_603 | 0.174 |
| Scymnus_ferrugatus_731 | Scymnus_rubromaculatus_822 | 0.174 |
| Scymnus_ferrugatus_731 | Scymnus_rubromaculatus_823 | 0.174 |

| | | |
|---|---|---|
| Scymnus_ferrugatus_731 | Scymnus_rubromaculatus_824 | 0.174 |
| Scymnus_ferrugatus_731 | Scymnus_rubromaculatus_825 | 0.174 |
| Scymnus_ferrugatus_731 | Scymnus_rubromaculatus_826 | 0.174 |
| Scymnus_ferrugatus_731 | Scymnus_rubromaculatus_827 | 0.174 |
| Platynaspis_luteorubra_51 | Tytthaspis_sedecimpunctata_583 | 0.174 |
| Platynaspis_luteorubra_53 | Tytthaspis_sedecimpunctata_583 | 0.174 |
| Platynaspis_luteorubra_58 | Tytthaspis_sedecimpunctata_583 | 0.174 |
| Hyperaspis_proba_661 | Tytthaspis_sedecimpunctata_591 | 0.174 |
| Chilocorus_stigma_44 | Tytthaspis_sedecimpunctata_586 | 0.174 |
| Chilocorus_stigma_46 | Tytthaspis_sedecimpunctata_586 | 0.174 |
| Chilocorus_stigma_47 | Tytthaspis_sedecimpunctata_586 | 0.174 |
| Chilocorus_stigma_44 | Tytthaspis_sedecimpunctata_588 | 0.174 |
| Chilocorus_stigma_46 | Tytthaspis_sedecimpunctata_588 | 0.174 |
| Chilocorus_stigma_47 | Tytthaspis_sedecimpunctata_588 | 0.174 |
| Brumus_quadripustulatus_9 | Tytthaspis_sedecimpunctata_584 | 0.174 |
| Brumus_quadripustulatus_11 | Tytthaspis_sedecimpunctata_589 | 0.174 |
| Brumus_quadripustulatus_13 | Tytthaspis_sedecimpunctata_589 | 0.174 |
| Brumus_quadripustulatus_14 | Tytthaspis_sedecimpunctata_589 | 0.174 |
| Brumus_quadripustulatus_17 | Tytthaspis_sedecimpunctata_589 | 0.174 |
| Brumus_quadripustulatus_3 | Tytthaspis_sedecimpunctata_589 | 0.174 |
| Brumus_quadripustulatus_4 | Tytthaspis_sedecimpunctata_589 | 0.174 |
| Brumus_quadripustulatus_7 | Tytthaspis_sedecimpunctata_589 | 0.174 |
| Coccidula_scutellata_81 | Scymnus_suturalis_837 | 0.174 |
| Coccidula_scutellata_82 | Scymnus_suturalis_837 | 0.174 |
| Chilocorus_nigritus_26 | Scymnus_suturalis_838 | 0.174 |
| Chilocorus_nigritus_26 | Scymnus_suturalis_840 | 0.174 |
| Calvia_quatuordecimguttata_204 | Vibidia_duodecimguttata_598 | 0.174 |
| Calvia_quatuordecimguttata_208 | Vibidia_duodecimguttata_598 | 0.174 |
| Calvia_quatuordecimguttata_217 | Vibidia_duodecimguttata_598 | 0.174 |
| Calvia_quatuordecimguttata_204 | Vibidia_duodecimguttata_599 | 0.174 |
| Calvia_quatuordecimguttata_208 | Vibidia_duodecimguttata_599 | 0.174 |
| Calvia_quatuordecimguttata_217 | Vibidia_duodecimguttata_599 | 0.174 |
| Calvia_quatuordecimguttata_204 | Vibidia_duodecimguttata_601 | 0.174 |
| Calvia_quatuordecimguttata_208 | Vibidia_duodecimguttata_601 | 0.174 |
| Calvia_quatuordecimguttata_217 | Vibidia_duodecimguttata_601 | 0.174 |
| Calvia_quatuordecimguttata_204 | Vibidia_duodecimguttata_602 | 0.174 |
| Calvia_quatuordecimguttata_208 | Vibidia_duodecimguttata_602 | 0.174 |
| Calvia_quatuordecimguttata_217 | Vibidia_duodecimguttata_602 | 0.174 |
| Chilocorus_nigritus_25 | Scymnus_suturalis_841 | 0.174 |
| Chilocorus_nigritus_25 | Scymnus_suturalis_842 | 0.174 |
| Chilocorus_nigritus_25 | Scymnus_suturalis_844 | 0.174 |
| Chilocorus_nigritus_25 | Scymnus_suturalis_846 | 0.174 |
| Chilocorus_nigritus_25 | Scymnus_suturalis_847 | 0.174 |
| Chilocorus_nigritus_25 | Scymnus_suturalis_848 | 0.174 |
| Brumus_quadripustulatus_12 | Tytthaspis_sedecimpunctata_585 | 0.174 |
| Brumus_quadripustulatus_11 | Tytthaspis_sedecimpunctata_588 | 0.174 |
| Brumus_quadripustulatus_13 | Tytthaspis_sedecimpunctata_588 | 0.174 |
| Brumus_quadripustulatus_14 | Tytthaspis_sedecimpunctata_588 | 0.174 |
| Brumus_quadripustulatus_17 | Tytthaspis_sedecimpunctata_588 | 0.174 |
| Brumus_quadripustulatus_3 | Tytthaspis_sedecimpunctata_588 | 0.174 |
| Brumus_quadripustulatus_4 | Tytthaspis_sedecimpunctata_588 | 0.174 |
| Brumus_quadripustulatus_7 | Tytthaspis_sedecimpunctata_588 | 0.174 |
| Brumus_quadripustulatus_11 | Tytthaspis_sedecimpunctata_591 | 0.174 |
| Brumus_quadripustulatus_13 | Tytthaspis_sedecimpunctata_591 | 0.174 |
| Brumus_quadripustulatus_14 | Tytthaspis_sedecimpunctata_591 | 0.174 |
| Brumus_quadripustulatus_17 | Tytthaspis_sedecimpunctata_591 | 0.174 |

| | | |
|---|---|---|
| Brumus_quadripustulatus_3 | Tytthaspis_sedecimpunctata_591 | 0.174 |
| Brumus_quadripustulatus_4 | Tytthaspis_sedecimpunctata_591 | 0.174 |
| Brumus_quadripustulatus_7 | Tytthaspis_sedecimpunctata_591 | 0.174 |
| Brumus_quadripustulatus_12 | Tytthaspis_sedecimpunctata_593 | 0.174 |
| Chilocorus_nigritus_25 | Scymnus_suturalis_832 | 0.174 |
| Chilocorus_nigritus_26 | Scymnus_suturalis_834 | 0.174 |
| Chilocorus_nigritus_25 | Scymnus_suturalis_835 | 0.174 |
| Chilocorus_nigritus_25 | Scymnus_suturalis_836 | 0.174 |
| Chilocorus_nigritus_26 | Scymnus_suturalis_837 | 0.174 |
| Chilocorus_nigritus_25 | Scymnus_suturalis_839 | 0.174 |
| Propylaea_quatuordecimpunctata_545 | Tytthaspis_sedecimpunctata_584 | 0.174 |
| Propylaea_quatuordecimpunctata_532 | Tytthaspis_sedecimpunctata_586 | 0.174 |
| Propylaea_quatuordecimpunctata_533 | Tytthaspis_sedecimpunctata_586 | 0.174 |
| Propylaea_quatuordecimpunctata_534 | Tytthaspis_sedecimpunctata_586 | 0.174 |
| Propylaea_quatuordecimpunctata_535 | Tytthaspis_sedecimpunctata_586 | 0.174 |
| Propylaea_quatuordecimpunctata_536 | Tytthaspis_sedecimpunctata_586 | 0.174 |
| Propylaea_quatuordecimpunctata_537 | Tytthaspis_sedecimpunctata_586 | 0.174 |
| Propylaea_quatuordecimpunctata_538 | Tytthaspis_sedecimpunctata_586 | 0.174 |
| Propylaea_quatuordecimpunctata_541 | Tytthaspis_sedecimpunctata_586 | 0.174 |
| Propylaea_quatuordecimpunctata_544 | Tytthaspis_sedecimpunctata_586 | 0.174 |
| Propylaea_quatuordecimpunctata_549 | Tytthaspis_sedecimpunctata_586 | 0.174 |
| Aphidecta_obliterata_179 | Tytthaspis_sedecimpunctata_588 | 0.174 |
| Propylaea_quatuordecimpunctata_532 | Tytthaspis_sedecimpunctata_588 | 0.174 |
| Propylaea_quatuordecimpunctata_533 | Tytthaspis_sedecimpunctata_588 | 0.174 |
| Propylaea_quatuordecimpunctata_534 | Tytthaspis_sedecimpunctata_588 | 0.174 |
| Propylaea_quatuordecimpunctata_535 | Tytthaspis_sedecimpunctata_588 | 0.174 |
| Propylaea_quatuordecimpunctata_536 | Tytthaspis_sedecimpunctata_588 | 0.174 |
| Propylaea_quatuordecimpunctata_537 | Tytthaspis_sedecimpunctata_588 | 0.174 |
| Propylaea_quatuordecimpunctata_538 | Tytthaspis_sedecimpunctata_588 | 0.174 |
| Propylaea_quatuordecimpunctata_541 | Tytthaspis_sedecimpunctata_588 | 0.174 |
| Propylaea_quatuordecimpunctata_544 | Tytthaspis_sedecimpunctata_588 | 0.174 |
| Propylaea_quatuordecimpunctata_549 | Tytthaspis_sedecimpunctata_588 | 0.174 |
| Aphidecta_obliterata_178 | Tytthaspis_sedecimpunctata_589 | 0.174 |
| Scymnus_camptodromus_714 | Tytthaspis_sedecimpunctata_589 | 0.174 |
| Propylaea_quatuordecimpunctata_542 | Tytthaspis_sedecimpunctata_591 | 0.174 |
| Propylaea_quatuordecimpunctata_546 | Tytthaspis_sedecimpunctata_591 | 0.174 |
| Coccidula_scutellata_81 | Scymnus_sinuanodulus_828 | 0.174 |
| Coccidula_scutellata_82 | Scymnus_sinuanodulus_828 | 0.174 |
| Chilocorus_nigritus_27 | Scymnus_suturalis_833 | 0.174 |
| Platynaspis_luteorubra_51 | Scymnus_suturalis_833 | 0.174 |
| Platynaspis_luteorubra_53 | Scymnus_suturalis_833 | 0.174 |
| Platynaspis_luteorubra_58 | Scymnus_suturalis_833 | 0.174 |
| Platynaspis_luteorubra_51 | Scymnus_suturalis_841 | 0.174 |
| Platynaspis_luteorubra_53 | Scymnus_suturalis_841 | 0.174 |
| Platynaspis_luteorubra_58 | Scymnus_suturalis_841 | 0.174 |
| Platynaspis_luteorubra_51 | Scymnus_suturalis_842 | 0.174 |
| Platynaspis_luteorubra_53 | Scymnus_suturalis_842 | 0.174 |
| Platynaspis_luteorubra_58 | Scymnus_suturalis_842 | 0.174 |
| Platynaspis_luteorubra_51 | Scymnus_suturalis_844 | 0.174 |
| Platynaspis_luteorubra_53 | Scymnus_suturalis_844 | 0.174 |
| Platynaspis_luteorubra_58 | Scymnus_suturalis_844 | 0.174 |
| Platynaspis_luteorubra_51 | Scymnus_suturalis_846 | 0.174 |
| Platynaspis_luteorubra_53 | Scymnus_suturalis_846 | 0.174 |
| Platynaspis_luteorubra_58 | Scymnus_suturalis_846 | 0.174 |
| Platynaspis_luteorubra_51 | Scymnus_suturalis_847 | 0.174 |
| Platynaspis_luteorubra_53 | Scymnus_suturalis_847 | 0.174 |

| | | |
|---|---|---|
| Platynaspis_luteorubra_58 | Scymnus_suturalis_847 | 0.174 |
| Platynaspis_luteorubra_51 | Scymnus_suturalis_848 | 0.174 |
| Platynaspis_luteorubra_53 | Scymnus_suturalis_848 | 0.174 |
| Platynaspis_luteorubra_58 | Scymnus_suturalis_848 | 0.174 |
| Aphidecta_obliterata_179 | Tytthaspis_sedecimpunctata_577 | 0.174 |
| Propylaea_quatuordecimpunctata_545 | Tytthaspis_sedecimpunctata_577 | 0.174 |
| Aphidecta_obliterata_179 | Tytthaspis_sedecimpunctata_578 | 0.174 |
| Propylaea_quatuordecimpunctata_545 | Tytthaspis_sedecimpunctata_578 | 0.174 |
| Aphidecta_obliterata_179 | Tytthaspis_sedecimpunctata_579 | 0.174 |
| Propylaea_quatuordecimpunctata_545 | Tytthaspis_sedecimpunctata_579 | 0.174 |
| Aphidecta_obliterata_179 | Tytthaspis_sedecimpunctata_581 | 0.174 |
| Propylaea_quatuordecimpunctata_545 | Tytthaspis_sedecimpunctata_581 | 0.174 |
| Aphidecta_obliterata_179 | Tytthaspis_sedecimpunctata_582 | 0.174 |
| Propylaea_quatuordecimpunctata_545 | Tytthaspis_sedecimpunctata_582 | 0.174 |
| Propylaea_quatuordecimpunctata_547 | Tytthaspis_sedecimpunctata_583 | 0.174 |
| Adalia_bipunctata_99 | Tytthaspis_sedecimpunctata_584 | 0.174 |
| Coccinella_trifasciata_272 | Tytthaspis_sedecimpunctata_584 | 0.174 |
| Coccinella_trifasciata_276 | Tytthaspis_sedecimpunctata_584 | 0.174 |
| Propylaea_quatuordecimpunctata_547 | Tytthaspis_sedecimpunctata_585 | 0.174 |
| Aphidecta_obliterata_178 | Tytthaspis_sedecimpunctata_586 | 0.174 |
| Coccinella_transversoguttata_265 | Tytthaspis_sedecimpunctata_586 | 0.174 |
| Coccinella_transversoguttata_266 | Tytthaspis_sedecimpunctata_586 | 0.174 |
| Adalia_bipunctata_105 | Tytthaspis_sedecimpunctata_587 | 0.174 |
| Coccinella_hieroglyphica_225 | Tytthaspis_sedecimpunctata_588 | 0.174 |
| Coccinella_transversoguttata_265 | Tytthaspis_sedecimpunctata_588 | 0.174 |
| Coccinella_transversoguttata_266 | Tytthaspis_sedecimpunctata_588 | 0.174 |
| Adalia_bipunctata_101 | Tytthaspis_sedecimpunctata_589 | 0.174 |
| Adalia_bipunctata_104 | Tytthaspis_sedecimpunctata_589 | 0.174 |
| Harmonia_axyridis_362 | Tytthaspis_sedecimpunctata_589 | 0.174 |
| Propylaea_quatuordecimpunctata_539 | Tytthaspis_sedecimpunctata_589 | 0.174 |
| Aphidecta_obliterata_179 | Tytthaspis_sedecimpunctata_590 | 0.174 |
| Propylaea_quatuordecimpunctata_545 | Tytthaspis_sedecimpunctata_590 | 0.174 |
| Aphidecta_obliterata_178 | Tytthaspis_sedecimpunctata_591 | 0.174 |
| Aphidecta_obliterata_175 | Tytthaspis_sedecimpunctata_592 | 0.174 |
| Aphidecta_obliterata_177 | Tytthaspis_sedecimpunctata_592 | 0.174 |
| Aphidecta_obliterata_180 | Tytthaspis_sedecimpunctata_592 | 0.174 |
| Aphidecta_obliterata_182 | Tytthaspis_sedecimpunctata_592 | 0.174 |
| Propylaea_quatuordecimpunctata_547 | Tytthaspis_sedecimpunctata_592 | 0.174 |
| Aphidecta_obliterata_179 | Tytthaspis_sedecimpunctata_593 | 0.174 |
| Propylaea_quatuordecimpunctata_545 | Tytthaspis_sedecimpunctata_593 | 0.174 |
| Aphidecta_obliterata_179 | Tytthaspis_sedecimpunctata_594 | 0.174 |
| Propylaea_quatuordecimpunctata_545 | Tytthaspis_sedecimpunctata_594 | 0.174 |
| Aphidecta_obliterata_179 | Tytthaspis_sedecimpunctata_595 | 0.174 |
| Propylaea_quatuordecimpunctata_545 | Tytthaspis_sedecimpunctata_595 | 0.174 |
| Platynaspis_luteorubra_51 | Scymnus_suturalis_832 | 0.174 |
| Platynaspis_luteorubra_53 | Scymnus_suturalis_832 | 0.174 |
| Platynaspis_luteorubra_58 | Scymnus_suturalis_832 | 0.174 |
| Anatis_ocellata_132 | Scymnus_suturalis_833 | 0.174 |
| Anatis_ocellata_134 | Scymnus_suturalis_833 | 0.174 |
| Anatis_ocellata_135 | Scymnus_suturalis_833 | 0.174 |
| Anatis_ocellata_138 | Scymnus_suturalis_833 | 0.174 |
| Anatis_ocellata_139 | Scymnus_suturalis_833 | 0.174 |
| Anatis_ocellata_140 | Scymnus_suturalis_833 | 0.174 |
| Anatis_ocellata_141 | Scymnus_suturalis_833 | 0.174 |
| Anatis_ocellata_142 | Scymnus_suturalis_833 | 0.174 |
| Anatis_ocellata_144 | Scymnus_suturalis_833 | 0.174 |

| | | |
|---|---|---|
| Anatis_ocellata_145 | Scymnus_suturalis_833 | 0.174 |
| Anatis_ocellata_146 | Scymnus_suturalis_833 | 0.174 |
| Hyperaspis_oregona_658 | Scymnus_suturalis_833 | 0.174 |
| Platynaspis_luteorubra_51 | Scymnus_suturalis_835 | 0.174 |
| Platynaspis_luteorubra_53 | Scymnus_suturalis_835 | 0.174 |
| Platynaspis_luteorubra_58 | Scymnus_suturalis_835 | 0.174 |
| Platynaspis_luteorubra_51 | Scymnus_suturalis_836 | 0.174 |
| Platynaspis_luteorubra_53 | Scymnus_suturalis_836 | 0.174 |
| Platynaspis_luteorubra_58 | Scymnus_suturalis_836 | 0.174 |
| Platynaspis_luteorubra_51 | Scymnus_suturalis_839 | 0.174 |
| Platynaspis_luteorubra_53 | Scymnus_suturalis_839 | 0.174 |
| Platynaspis_luteorubra_58 | Scymnus_suturalis_839 | 0.174 |
| Psyllobora_vigintimaculata_575 | Vibidia_duodecimguttata_597 | 0.174 |
| Psyllobora_vigintimaculata_576 | Vibidia_duodecimguttata_597 | 0.174 |
| Psyllobora_vigintimaculata_575 | Vibidia_duodecimguttata_600 | 0.174 |
| Psyllobora_vigintimaculata_576 | Vibidia_duodecimguttata_600 | 0.174 |
| Anatis_ocellata_132 | Scymnus_suturalis_841 | 0.174 |
| Anatis_ocellata_134 | Scymnus_suturalis_841 | 0.174 |
| Anatis_ocellata_135 | Scymnus_suturalis_841 | 0.174 |
| Anatis_ocellata_138 | Scymnus_suturalis_841 | 0.174 |
| Anatis_ocellata_139 | Scymnus_suturalis_841 | 0.174 |
| Anatis_ocellata_140 | Scymnus_suturalis_841 | 0.174 |
| Anatis_ocellata_141 | Scymnus_suturalis_841 | 0.174 |
| Anatis_ocellata_142 | Scymnus_suturalis_841 | 0.174 |
| Anatis_ocellata_144 | Scymnus_suturalis_841 | 0.174 |
| Anatis_ocellata_145 | Scymnus_suturalis_841 | 0.174 |
| Anatis_ocellata_146 | Scymnus_suturalis_841 | 0.174 |
| Hyperaspis_oregona_659 | Scymnus_suturalis_841 | 0.174 |
| Anatis_ocellata_132 | Scymnus_suturalis_842 | 0.174 |
| Anatis_ocellata_134 | Scymnus_suturalis_842 | 0.174 |
| Anatis_ocellata_135 | Scymnus_suturalis_842 | 0.174 |
| Anatis_ocellata_138 | Scymnus_suturalis_842 | 0.174 |
| Anatis_ocellata_139 | Scymnus_suturalis_842 | 0.174 |
| Anatis_ocellata_140 | Scymnus_suturalis_842 | 0.174 |
| Anatis_ocellata_141 | Scymnus_suturalis_842 | 0.174 |
| Anatis_ocellata_142 | Scymnus_suturalis_842 | 0.174 |
| Anatis_ocellata_144 | Scymnus_suturalis_842 | 0.174 |
| Anatis_ocellata_145 | Scymnus_suturalis_842 | 0.174 |
| Anatis_ocellata_146 | Scymnus_suturalis_842 | 0.174 |
| Hyperaspis_oregona_659 | Scymnus_suturalis_842 | 0.174 |
| Anatis_ocellata_132 | Scymnus_suturalis_844 | 0.174 |
| Anatis_ocellata_134 | Scymnus_suturalis_844 | 0.174 |
| Anatis_ocellata_135 | Scymnus_suturalis_844 | 0.174 |
| Anatis_ocellata_138 | Scymnus_suturalis_844 | 0.174 |
| Anatis_ocellata_139 | Scymnus_suturalis_844 | 0.174 |
| Anatis_ocellata_140 | Scymnus_suturalis_844 | 0.174 |
| Anatis_ocellata_141 | Scymnus_suturalis_844 | 0.174 |
| Anatis_ocellata_142 | Scymnus_suturalis_844 | 0.174 |
| Anatis_ocellata_144 | Scymnus_suturalis_844 | 0.174 |
| Anatis_ocellata_145 | Scymnus_suturalis_844 | 0.174 |
| Anatis_ocellata_146 | Scymnus_suturalis_844 | 0.174 |
| Hyperaspis_oregona_659 | Scymnus_suturalis_844 | 0.174 |
| Anatis_ocellata_132 | Scymnus_suturalis_846 | 0.174 |
| Anatis_ocellata_134 | Scymnus_suturalis_846 | 0.174 |
| Anatis_ocellata_135 | Scymnus_suturalis_846 | 0.174 |
| Anatis_ocellata_138 | Scymnus_suturalis_846 | 0.174 |

| | | |
|---|---|---|
| Anatis_ocellata_139 | Scymnus_suturalis_846 | 0.174 |
| Anatis_ocellata_140 | Scymnus_suturalis_846 | 0.174 |
| Anatis_ocellata_141 | Scymnus_suturalis_846 | 0.174 |
| Anatis_ocellata_142 | Scymnus_suturalis_846 | 0.174 |
| Anatis_ocellata_144 | Scymnus_suturalis_846 | 0.174 |
| Anatis_ocellata_145 | Scymnus_suturalis_846 | 0.174 |
| Anatis_ocellata_146 | Scymnus_suturalis_846 | 0.174 |
| Hyperaspis_oregona_659 | Scymnus_suturalis_846 | 0.174 |
| Anatis_ocellata_132 | Scymnus_suturalis_847 | 0.174 |
| Anatis_ocellata_134 | Scymnus_suturalis_847 | 0.174 |
| Anatis_ocellata_135 | Scymnus_suturalis_847 | 0.174 |
| Anatis_ocellata_138 | Scymnus_suturalis_847 | 0.174 |
| Anatis_ocellata_139 | Scymnus_suturalis_847 | 0.174 |
| Anatis_ocellata_140 | Scymnus_suturalis_847 | 0.174 |
| Anatis_ocellata_141 | Scymnus_suturalis_847 | 0.174 |
| Anatis_ocellata_142 | Scymnus_suturalis_847 | 0.174 |
| Anatis_ocellata_144 | Scymnus_suturalis_847 | 0.174 |
| Anatis_ocellata_145 | Scymnus_suturalis_847 | 0.174 |
| Anatis_ocellata_146 | Scymnus_suturalis_847 | 0.174 |
| Hyperaspis_oregona_659 | Scymnus_suturalis_847 | 0.174 |
| Anatis_ocellata_132 | Scymnus_suturalis_848 | 0.174 |
| Anatis_ocellata_134 | Scymnus_suturalis_848 | 0.174 |
| Anatis_ocellata_135 | Scymnus_suturalis_848 | 0.174 |
| Anatis_ocellata_138 | Scymnus_suturalis_848 | 0.174 |
| Anatis_ocellata_139 | Scymnus_suturalis_848 | 0.174 |
| Anatis_ocellata_140 | Scymnus_suturalis_848 | 0.174 |
| Anatis_ocellata_141 | Scymnus_suturalis_848 | 0.174 |
| Anatis_ocellata_142 | Scymnus_suturalis_848 | 0.174 |
| Anatis_ocellata_144 | Scymnus_suturalis_848 | 0.174 |
| Anatis_ocellata_145 | Scymnus_suturalis_848 | 0.174 |
| Anatis_ocellata_146 | Scymnus_suturalis_848 | 0.174 |
| Hyperaspis_oregona_659 | Scymnus_suturalis_848 | 0.174 |
| Aphidecta_obliterata_175 | Tytthaspis_sedecimpunctata_580 | 0.174 |
| Aphidecta_obliterata_177 | Tytthaspis_sedecimpunctata_580 | 0.174 |
| Aphidecta_obliterata_180 | Tytthaspis_sedecimpunctata_580 | 0.174 |
| Aphidecta_obliterata_182 | Tytthaspis_sedecimpunctata_580 | 0.174 |
| Propylaea_quatuordecimpunctata_545 | Tytthaspis_sedecimpunctata_580 | 0.174 |
| Aphidecta_obliterata_178 | Tytthaspis_sedecimpunctata_583 | 0.174 |
| Coccinella_hieroglyphica_225 | Tytthaspis_sedecimpunctata_583 | 0.174 |
| Coccinella_trifasciata_275 | Tytthaspis_sedecimpunctata_583 | 0.174 |
| Coccinella_trifasciata_277 | Tytthaspis_sedecimpunctata_583 | 0.174 |
| Coccinella_trifasciata_278 | Tytthaspis_sedecimpunctata_583 | 0.174 |
| Coccinella_trifasciata_279 | Tytthaspis_sedecimpunctata_583 | 0.174 |
| Coccinella_trifasciata_281 | Tytthaspis_sedecimpunctata_583 | 0.174 |
| Adalia_bipunctata_102 | Tytthaspis_sedecimpunctata_584 | 0.174 |
| Adalia_bipunctata_103 | Tytthaspis_sedecimpunctata_584 | 0.174 |
| Adalia_bipunctata_106 | Tytthaspis_sedecimpunctata_584 | 0.174 |
| Adalia_bipunctata_107 | Tytthaspis_sedecimpunctata_584 | 0.174 |
| Adalia_bipunctata_108 | Tytthaspis_sedecimpunctata_584 | 0.174 |
| Adalia_bipunctata_109 | Tytthaspis_sedecimpunctata_584 | 0.174 |
| Aphidecta_obliterata_178 | Tytthaspis_sedecimpunctata_585 | 0.174 |
| Coccinella_trifasciata_272 | Tytthaspis_sedecimpunctata_585 | 0.174 |
| Coccinella_trifasciata_276 | Tytthaspis_sedecimpunctata_585 | 0.174 |
| Adalia_bipunctata_98 | Tytthaspis_sedecimpunctata_586 | 0.174 |
| Harmonia_axyridis_354 | Tytthaspis_sedecimpunctata_586 | 0.174 |
| Harmonia_axyridis_356 | Tytthaspis_sedecimpunctata_586 | 0.174 |

| | | |
|---|---|---|
| Harmonia_axyridis_357 | Tytthaspis_sedecimpunctata_586 | 0.174 |
| Harmonia_axyridis_358 | Tytthaspis_sedecimpunctata_586 | 0.174 |
| Harmonia_axyridis_359 | Tytthaspis_sedecimpunctata_586 | 0.174 |
| Harmonia_axyridis_360 | Tytthaspis_sedecimpunctata_586 | 0.174 |
| Harmonia_axyridis_361 | Tytthaspis_sedecimpunctata_586 | 0.174 |
| Harmonia_axyridis_363 | Tytthaspis_sedecimpunctata_586 | 0.174 |
| Harmonia_axyridis_364 | Tytthaspis_sedecimpunctata_586 | 0.174 |
| Adalia_bipunctata_101 | Tytthaspis_sedecimpunctata_587 | 0.174 |
| Adalia_bipunctata_104 | Tytthaspis_sedecimpunctata_587 | 0.174 |
| Harmonia_axyridis_354 | Tytthaspis_sedecimpunctata_587 | 0.174 |
| Harmonia_axyridis_356 | Tytthaspis_sedecimpunctata_587 | 0.174 |
| Harmonia_axyridis_357 | Tytthaspis_sedecimpunctata_587 | 0.174 |
| Harmonia_axyridis_358 | Tytthaspis_sedecimpunctata_587 | 0.174 |
| Harmonia_axyridis_359 | Tytthaspis_sedecimpunctata_587 | 0.174 |
| Harmonia_axyridis_360 | Tytthaspis_sedecimpunctata_587 | 0.174 |
| Harmonia_axyridis_361 | Tytthaspis_sedecimpunctata_587 | 0.174 |
| Harmonia_axyridis_363 | Tytthaspis_sedecimpunctata_587 | 0.174 |
| Harmonia_axyridis_364 | Tytthaspis_sedecimpunctata_587 | 0.174 |
| Harmonia_axyridis_362 | Tytthaspis_sedecimpunctata_588 | 0.174 |
| Scymnus_camptodromus_712 | Tytthaspis_sedecimpunctata_589 | 0.174 |
| Harmonia_axyridis_354 | Tytthaspis_sedecimpunctata_591 | 0.174 |
| Harmonia_axyridis_356 | Tytthaspis_sedecimpunctata_591 | 0.174 |
| Harmonia_axyridis_357 | Tytthaspis_sedecimpunctata_591 | 0.174 |
| Harmonia_axyridis_358 | Tytthaspis_sedecimpunctata_591 | 0.174 |
| Harmonia_axyridis_359 | Tytthaspis_sedecimpunctata_591 | 0.174 |
| Harmonia_axyridis_360 | Tytthaspis_sedecimpunctata_591 | 0.174 |
| Harmonia_axyridis_361 | Tytthaspis_sedecimpunctata_591 | 0.174 |
| Harmonia_axyridis_363 | Tytthaspis_sedecimpunctata_591 | 0.174 |
| Harmonia_axyridis_364 | Tytthaspis_sedecimpunctata_591 | 0.174 |
| Anatis_ocellata_132 | Scymnus_suturalis_832 | 0.174 |
| Anatis_ocellata_134 | Scymnus_suturalis_832 | 0.174 |
| Anatis_ocellata_135 | Scymnus_suturalis_832 | 0.174 |
| Anatis_ocellata_138 | Scymnus_suturalis_832 | 0.174 |
| Anatis_ocellata_139 | Scymnus_suturalis_832 | 0.174 |
| Anatis_ocellata_140 | Scymnus_suturalis_832 | 0.174 |
| Anatis_ocellata_141 | Scymnus_suturalis_832 | 0.174 |
| Anatis_ocellata_142 | Scymnus_suturalis_832 | 0.174 |
| Anatis_ocellata_144 | Scymnus_suturalis_832 | 0.174 |
| Anatis_ocellata_145 | Scymnus_suturalis_832 | 0.174 |
| Anatis_ocellata_146 | Scymnus_suturalis_832 | 0.174 |
| Hyperaspis_oregona_659 | Scymnus_suturalis_832 | 0.174 |
| Anatis_ocellata_132 | Scymnus_suturalis_835 | 0.174 |
| Anatis_ocellata_134 | Scymnus_suturalis_835 | 0.174 |
| Anatis_ocellata_135 | Scymnus_suturalis_835 | 0.174 |
| Anatis_ocellata_138 | Scymnus_suturalis_835 | 0.174 |
| Anatis_ocellata_139 | Scymnus_suturalis_835 | 0.174 |
| Anatis_ocellata_140 | Scymnus_suturalis_835 | 0.174 |
| Anatis_ocellata_141 | Scymnus_suturalis_835 | 0.174 |
| Anatis_ocellata_142 | Scymnus_suturalis_835 | 0.174 |
| Anatis_ocellata_144 | Scymnus_suturalis_835 | 0.174 |
| Anatis_ocellata_145 | Scymnus_suturalis_835 | 0.174 |
| Anatis_ocellata_146 | Scymnus_suturalis_835 | 0.174 |
| Hyperaspis_oregona_659 | Scymnus_suturalis_835 | 0.174 |
| Anatis_ocellata_132 | Scymnus_suturalis_836 | 0.174 |
| Anatis_ocellata_134 | Scymnus_suturalis_836 | 0.174 |
| Anatis_ocellata_135 | Scymnus_suturalis_836 | 0.174 |

| | | |
|---|---|---|
| Anatis_ocellata_138 | Scymnus_suturalis_836 | 0.174 |
| Anatis_ocellata_139 | Scymnus_suturalis_836 | 0.174 |
| Anatis_ocellata_140 | Scymnus_suturalis_836 | 0.174 |
| Anatis_ocellata_141 | Scymnus_suturalis_836 | 0.174 |
| Anatis_ocellata_142 | Scymnus_suturalis_836 | 0.174 |
| Anatis_ocellata_144 | Scymnus_suturalis_836 | 0.174 |
| Anatis_ocellata_145 | Scymnus_suturalis_836 | 0.174 |
| Anatis_ocellata_146 | Scymnus_suturalis_836 | 0.174 |
| Hyperaspis_oregona_659 | Scymnus_suturalis_836 | 0.174 |
| Hyperaspis_campestris_651 | Scymnus_suturalis_838 | 0.174 |
| Hyperaspis_campestris_652 | Scymnus_suturalis_838 | 0.174 |
| Hyperaspis_campestris_653 | Scymnus_suturalis_838 | 0.174 |
| Anatis_ocellata_132 | Scymnus_suturalis_839 | 0.174 |
| Anatis_ocellata_134 | Scymnus_suturalis_839 | 0.174 |
| Anatis_ocellata_135 | Scymnus_suturalis_839 | 0.174 |
| Anatis_ocellata_138 | Scymnus_suturalis_839 | 0.174 |
| Anatis_ocellata_139 | Scymnus_suturalis_839 | 0.174 |
| Anatis_ocellata_140 | Scymnus_suturalis_839 | 0.174 |
| Anatis_ocellata_141 | Scymnus_suturalis_839 | 0.174 |
| Anatis_ocellata_142 | Scymnus_suturalis_839 | 0.174 |
| Anatis_ocellata_144 | Scymnus_suturalis_839 | 0.174 |
| Anatis_ocellata_145 | Scymnus_suturalis_839 | 0.174 |
| Anatis_ocellata_146 | Scymnus_suturalis_839 | 0.174 |
| Hyperaspis_oregona_659 | Scymnus_suturalis_839 | 0.174 |
| Hyperaspis_campestris_651 | Scymnus_suturalis_840 | 0.174 |
| Hyperaspis_campestris_652 | Scymnus_suturalis_840 | 0.174 |
| Hyperaspis_campestris_653 | Scymnus_suturalis_840 | 0.174 |
| Scymnus_pallipediformis_812 | Scymnus_suturalis_841 | 0.174 |
| Scymnus_pallipediformis_812 | Scymnus_suturalis_842 | 0.174 |
| Platynaspis_luteorubra_55 | Scymnus_suturalis_843 | 0.174 |
| Platynaspis_luteorubra_61 | Scymnus_suturalis_843 | 0.174 |
| Scymnus_pallipediformis_812 | Scymnus_suturalis_844 | 0.174 |
| Platynaspis_luteorubra_55 | Scymnus_suturalis_845 | 0.174 |
| Platynaspis_luteorubra_61 | Scymnus_suturalis_845 | 0.174 |
| Scymnus_pallipediformis_812 | Scymnus_suturalis_846 | 0.174 |
| Scymnus_pallipediformis_812 | Scymnus_suturalis_847 | 0.174 |
| Scymnus_pallipediformis_812 | Scymnus_suturalis_848 | 0.174 |
| Coccinella_hieroglyphica_227 | Tytthaspis_sedecimpunctata_580 | 0.174 |
| Coccinella_trifasciata_274 | Tytthaspis_sedecimpunctata_583 | 0.174 |
| Coccinella_trifasciata_273 | Tytthaspis_sedecimpunctata_586 | 0.174 |
| Scymnus_camptodromus_712 | Tytthaspis_sedecimpunctata_586 | 0.174 |
| Anatis_labiculata_126 | Tytthaspis_sedecimpunctata_588 | 0.174 |
| Scymnus_camptodromus_712 | Tytthaspis_sedecimpunctata_588 | 0.174 |
| Coccinella_quinquepunctata_236 | Tytthaspis_sedecimpunctata_589 | 0.174 |
| Coccinella_hieroglyphica_225 | Tytthaspis_sedecimpunctata_591 | 0.174 |
| Coccinella_trifasciata_277 | Tytthaspis_sedecimpunctata_591 | 0.174 |
| Adalia_bipunctata_102 | Tytthaspis_sedecimpunctata_593 | 0.174 |
| Adalia_bipunctata_103 | Tytthaspis_sedecimpunctata_593 | 0.174 |
| Adalia_bipunctata_106 | Tytthaspis_sedecimpunctata_593 | 0.174 |
| Adalia_bipunctata_107 | Tytthaspis_sedecimpunctata_593 | 0.174 |
| Adalia_bipunctata_108 | Tytthaspis_sedecimpunctata_593 | 0.174 |
| Adalia_bipunctata_109 | Tytthaspis_sedecimpunctata_593 | 0.174 |
| Psyllobora_renifer_551 | Tytthaspis_sedecimpunctata_593 | 0.174 |
| Psyllobora_renifer_552 | Tytthaspis_sedecimpunctata_593 | 0.174 |
| Psyllobora_renifer_553 | Tytthaspis_sedecimpunctata_593 | 0.174 |
| Psyllobora_renifer_554 | Tytthaspis_sedecimpunctata_593 | 0.174 |

| | | |
|---|---|---|
| Scymnus_pallipediformis_812 | Scymnus_suturalis_832 | 0.174 |
| Hyperaspis_campestris_651 | Scymnus_suturalis_834 | 0.174 |
| Hyperaspis_campestris_652 | Scymnus_suturalis_834 | 0.174 |
| Hyperaspis_campestris_653 | Scymnus_suturalis_834 | 0.174 |
| Scymnus_pallipediformis_812 | Scymnus_suturalis_835 | 0.174 |
| Scymnus_pallipediformis_812 | Scymnus_suturalis_836 | 0.174 |
| Platynaspis_luteorubra_55 | Scymnus_suturalis_837 | 0.174 |
| Platynaspis_luteorubra_61 | Scymnus_suturalis_837 | 0.174 |
| Scymnus_pallipediformis_812 | Scymnus_suturalis_839 | 0.174 |
| Scymnus_auritus_701 | Scymnus_sinuanodulus_829 | 0.174 |
| Hippodamia_alpina_385 | Tytthaspis_sedecimpunctata_577 | 0.174 |
| Hippodamia_alpina_386 | Tytthaspis_sedecimpunctata_577 | 0.174 |
| Hippodamia_alpina_387 | Tytthaspis_sedecimpunctata_577 | 0.174 |
| Hippodamia_alpina_385 | Tytthaspis_sedecimpunctata_578 | 0.174 |
| Hippodamia_alpina_386 | Tytthaspis_sedecimpunctata_578 | 0.174 |
| Hippodamia_alpina_387 | Tytthaspis_sedecimpunctata_578 | 0.174 |
| Hippodamia_alpina_385 | Tytthaspis_sedecimpunctata_579 | 0.174 |
| Hippodamia_alpina_386 | Tytthaspis_sedecimpunctata_579 | 0.174 |
| Hippodamia_alpina_387 | Tytthaspis_sedecimpunctata_579 | 0.174 |
| Hippodamia_alpina_385 | Tytthaspis_sedecimpunctata_581 | 0.174 |
| Hippodamia_alpina_386 | Tytthaspis_sedecimpunctata_581 | 0.174 |
| Hippodamia_alpina_387 | Tytthaspis_sedecimpunctata_581 | 0.174 |
| Hippodamia_alpina_385 | Tytthaspis_sedecimpunctata_582 | 0.174 |
| Hippodamia_alpina_386 | Tytthaspis_sedecimpunctata_582 | 0.174 |
| Hippodamia_alpina_387 | Tytthaspis_sedecimpunctata_582 | 0.174 |
| Scymnus_auritus_703 | Tytthaspis_sedecimpunctata_583 | 0.174 |
| Coccinella_hieroglyphica_226 | Tytthaspis_sedecimpunctata_584 | 0.174 |
| Coccinella_quinquepunctata_237 | Tytthaspis_sedecimpunctata_584 | 0.174 |
| Hippodamia_alpina_388 | Tytthaspis_sedecimpunctata_584 | 0.174 |
| Hippodamia_alpina_385 | Tytthaspis_sedecimpunctata_585 | 0.174 |
| Hippodamia_alpina_386 | Tytthaspis_sedecimpunctata_585 | 0.174 |
| Hippodamia_alpina_387 | Tytthaspis_sedecimpunctata_585 | 0.174 |
| Scymnus_auritus_703 | Tytthaspis_sedecimpunctata_585 | 0.174 |
| Coccinella_hieroglyphica_226 | Tytthaspis_sedecimpunctata_586 | 0.174 |
| Coccinella_quinquepunctata_235 | Tytthaspis_sedecimpunctata_586 | 0.174 |
| Coccinella_quinquepunctata_238 | Tytthaspis_sedecimpunctata_586 | 0.174 |
| Coccinella_quinquepunctata_240 | Tytthaspis_sedecimpunctata_586 | 0.174 |
| Psyllobora_renifer_551 | Tytthaspis_sedecimpunctata_586 | 0.174 |
| Psyllobora_renifer_552 | Tytthaspis_sedecimpunctata_586 | 0.174 |
| Psyllobora_renifer_553 | Tytthaspis_sedecimpunctata_586 | 0.174 |
| Psyllobora_renifer_554 | Tytthaspis_sedecimpunctata_586 | 0.174 |
| Scymnus_auritus_698 | Tytthaspis_sedecimpunctata_587 | 0.174 |
| Coccinella_quinquepunctata_236 | Tytthaspis_sedecimpunctata_588 | 0.174 |
| Coccinella_quinquepunctata_239 | Tytthaspis_sedecimpunctata_588 | 0.174 |
| Coccinella_trifasciata_273 | Tytthaspis_sedecimpunctata_589 | 0.174 |
| Hippodamia_alpina_385 | Tytthaspis_sedecimpunctata_589 | 0.174 |
| Hippodamia_alpina_386 | Tytthaspis_sedecimpunctata_589 | 0.174 |
| Hippodamia_alpina_387 | Tytthaspis_sedecimpunctata_589 | 0.174 |
| Hippodamia_alpina_385 | Tytthaspis_sedecimpunctata_590 | 0.174 |
| Hippodamia_alpina_386 | Tytthaspis_sedecimpunctata_590 | 0.174 |
| Hippodamia_alpina_387 | Tytthaspis_sedecimpunctata_590 | 0.174 |
| Illeis_galbula_474 | Tytthaspis_sedecimpunctata_591 | 0.174 |
| Illeis_galbula_475 | Tytthaspis_sedecimpunctata_591 | 0.174 |
| Illeis_galbula_476 | Tytthaspis_sedecimpunctata_591 | 0.174 |
| Hippodamia_alpina_385 | Tytthaspis_sedecimpunctata_592 | 0.174 |
| Hippodamia_alpina_386 | Tytthaspis_sedecimpunctata_592 | 0.174 |

| | | |
|---|---|---|
| Hippodamia_alpina_387 | Tytthaspis_sedecimpunctata_592 | 0.174 |
| Psyllobora_vigintimaculata_573 | Tytthaspis_sedecimpunctata_592 | 0.174 |
| Scymnus_auritus_703 | Tytthaspis_sedecimpunctata_592 | 0.174 |
| Hippodamia_alpina_385 | Tytthaspis_sedecimpunctata_593 | 0.174 |
| Hippodamia_alpina_386 | Tytthaspis_sedecimpunctata_593 | 0.174 |
| Hippodamia_alpina_387 | Tytthaspis_sedecimpunctata_593 | 0.174 |
| Hippodamia_alpina_385 | Tytthaspis_sedecimpunctata_594 | 0.174 |
| Hippodamia_alpina_386 | Tytthaspis_sedecimpunctata_594 | 0.174 |
| Hippodamia_alpina_387 | Tytthaspis_sedecimpunctata_594 | 0.174 |
| Hippodamia_alpina_385 | Tytthaspis_sedecimpunctata_595 | 0.174 |
| Hippodamia_alpina_386 | Tytthaspis_sedecimpunctata_595 | 0.174 |
| Hippodamia_alpina_387 | Tytthaspis_sedecimpunctata_595 | 0.174 |
| Hyperaspis_reppensis_665 | Scymnus_suturalis_833 | 0.174 |
| Hyperaspis_reppensis_666 | Scymnus_suturalis_833 | 0.174 |
| Hyperaspis_reppensis_665 | Scymnus_suturalis_838 | 0.174 |
| Hyperaspis_reppensis_666 | Scymnus_suturalis_838 | 0.174 |
| Hyperaspis_reppensis_665 | Scymnus_suturalis_840 | 0.174 |
| Hyperaspis_reppensis_666 | Scymnus_suturalis_840 | 0.174 |
| Hyperaspis_proba_660 | Tytthaspis_sedecimpunctata_591 | 0.174 |
| Coccinella_hieroglyphica_226 | Tytthaspis_sedecimpunctata_577 | 0.174 |
| Coccinella_quinquepunctata_237 | Tytthaspis_sedecimpunctata_577 | 0.174 |
| Psyllobora_vigintimaculata_575 | Tytthaspis_sedecimpunctata_577 | 0.174 |
| Psyllobora_vigintimaculata_576 | Tytthaspis_sedecimpunctata_577 | 0.174 |
| Scymnus_auritus_698 | Tytthaspis_sedecimpunctata_577 | 0.174 |
| Coccinella_hieroglyphica_226 | Tytthaspis_sedecimpunctata_578 | 0.174 |
| Coccinella_quinquepunctata_237 | Tytthaspis_sedecimpunctata_578 | 0.174 |
| Psyllobora_vigintimaculata_575 | Tytthaspis_sedecimpunctata_578 | 0.174 |
| Psyllobora_vigintimaculata_576 | Tytthaspis_sedecimpunctata_578 | 0.174 |
| Scymnus_auritus_698 | Tytthaspis_sedecimpunctata_578 | 0.174 |
| Coccinella_hieroglyphica_226 | Tytthaspis_sedecimpunctata_579 | 0.174 |
| Coccinella_quinquepunctata_237 | Tytthaspis_sedecimpunctata_579 | 0.174 |
| Psyllobora_vigintimaculata_575 | Tytthaspis_sedecimpunctata_579 | 0.174 |
| Psyllobora_vigintimaculata_576 | Tytthaspis_sedecimpunctata_579 | 0.174 |
| Scymnus_auritus_698 | Tytthaspis_sedecimpunctata_579 | 0.174 |
| Psyllobora_vigintimaculata_573 | Tytthaspis_sedecimpunctata_580 | 0.174 |
| Scymnus_auritus_703 | Tytthaspis_sedecimpunctata_580 | 0.174 |
| Coccinella_hieroglyphica_226 | Tytthaspis_sedecimpunctata_581 | 0.174 |
| Coccinella_quinquepunctata_237 | Tytthaspis_sedecimpunctata_581 | 0.174 |
| Psyllobora_vigintimaculata_575 | Tytthaspis_sedecimpunctata_581 | 0.174 |
| Psyllobora_vigintimaculata_576 | Tytthaspis_sedecimpunctata_581 | 0.174 |
| Scymnus_auritus_698 | Tytthaspis_sedecimpunctata_581 | 0.174 |
| Coccinella_hieroglyphica_226 | Tytthaspis_sedecimpunctata_582 | 0.174 |
| Coccinella_quinquepunctata_237 | Tytthaspis_sedecimpunctata_582 | 0.174 |
| Psyllobora_vigintimaculata_575 | Tytthaspis_sedecimpunctata_582 | 0.174 |
| Psyllobora_vigintimaculata_576 | Tytthaspis_sedecimpunctata_582 | 0.174 |
| Scymnus_auritus_698 | Tytthaspis_sedecimpunctata_582 | 0.174 |
| Coccinella_quinquepunctata_232 | Tytthaspis_sedecimpunctata_583 | 0.174 |
| Coccinella_quinquepunctata_236 | Tytthaspis_sedecimpunctata_583 | 0.174 |
| Coccinella_quinquepunctata_239 | Tytthaspis_sedecimpunctata_583 | 0.174 |
| Coccinella_hieroglyphica_226 | Tytthaspis_sedecimpunctata_585 | 0.174 |
| Coccinella_quinquepunctata_237 | Tytthaspis_sedecimpunctata_585 | 0.174 |
| Illeis_galbula_474 | Tytthaspis_sedecimpunctata_585 | 0.174 |
| Illeis_galbula_475 | Tytthaspis_sedecimpunctata_585 | 0.174 |
| Illeis_galbula_476 | Tytthaspis_sedecimpunctata_585 | 0.174 |
| Psyllobora_renifer_555 | Tytthaspis_sedecimpunctata_587 | 0.174 |
| Psyllobora_vigintiduopunctata_571 | Tytthaspis_sedecimpunctata_587 | 0.174 |

| | | |
|---|---|---|
| Coccinella_hieroglyphica_226 | Tytthaspis_sedecimpunctata_590 | 0.174 |
| Coccinella_quinquepunctata_237 | Tytthaspis_sedecimpunctata_590 | 0.174 |
| Psyllobora_vigintimaculata_575 | Tytthaspis_sedecimpunctata_590 | 0.174 |
| Psyllobora_vigintimaculata_576 | Tytthaspis_sedecimpunctata_590 | 0.174 |
| Scymnus_auritus_698 | Tytthaspis_sedecimpunctata_590 | 0.174 |
| Hippodamia_alpina_384 | Tytthaspis_sedecimpunctata_591 | 0.174 |
| Coccinella_hieroglyphica_226 | Tytthaspis_sedecimpunctata_592 | 0.174 |
| Coccinella_quinquepunctata_237 | Tytthaspis_sedecimpunctata_592 | 0.174 |
| Psyllobora_vigintimaculata_574 | Tytthaspis_sedecimpunctata_592 | 0.174 |
| Coccinella_quinquepunctata_235 | Tytthaspis_sedecimpunctata_593 | 0.174 |
| Coccinella_quinquepunctata_237 | Tytthaspis_sedecimpunctata_593 | 0.174 |
| Coccinella_quinquepunctata_238 | Tytthaspis_sedecimpunctata_593 | 0.174 |
| Coccinella_quinquepunctata_240 | Tytthaspis_sedecimpunctata_593 | 0.174 |
| Illeis_galbula_474 | Tytthaspis_sedecimpunctata_593 | 0.174 |
| Illeis_galbula_475 | Tytthaspis_sedecimpunctata_593 | 0.174 |
| Illeis_galbula_476 | Tytthaspis_sedecimpunctata_593 | 0.174 |
| Psyllobora_vigintimaculata_575 | Tytthaspis_sedecimpunctata_593 | 0.174 |
| Psyllobora_vigintimaculata_576 | Tytthaspis_sedecimpunctata_593 | 0.174 |
| Coccinella_hieroglyphica_226 | Tytthaspis_sedecimpunctata_594 | 0.174 |
| Coccinella_quinquepunctata_237 | Tytthaspis_sedecimpunctata_594 | 0.174 |
| Psyllobora_vigintimaculata_575 | Tytthaspis_sedecimpunctata_594 | 0.174 |
| Psyllobora_vigintimaculata_576 | Tytthaspis_sedecimpunctata_594 | 0.174 |
| Scymnus_auritus_698 | Tytthaspis_sedecimpunctata_594 | 0.174 |
| Coccinella_hieroglyphica_226 | Tytthaspis_sedecimpunctata_595 | 0.174 |
| Coccinella_quinquepunctata_237 | Tytthaspis_sedecimpunctata_595 | 0.174 |
| Psyllobora_vigintimaculata_575 | Tytthaspis_sedecimpunctata_595 | 0.174 |
| Psyllobora_vigintimaculata_576 | Tytthaspis_sedecimpunctata_595 | 0.174 |
| Scymnus_auritus_698 | Tytthaspis_sedecimpunctata_595 | 0.174 |
| Brumus_quadripustulatus_11 | Scymnus_suturalis_833 | 0.174 |
| Brumus_quadripustulatus_13 | Scymnus_suturalis_833 | 0.174 |
| Brumus_quadripustulatus_14 | Scymnus_suturalis_833 | 0.174 |
| Brumus_quadripustulatus_3 | Scymnus_suturalis_833 | 0.174 |
| Brumus_quadripustulatus_4 | Scymnus_suturalis_833 | 0.174 |
| Brumus_quadripustulatus_7 | Scymnus_suturalis_833 | 0.174 |
| Scymnus_apetzi_690 | Scymnus_suturalis_833 | 0.174 |
| Scymnus_apetzi_697 | Scymnus_suturalis_833 | 0.174 |
| Hyperaspis_reppensis_665 | Scymnus_suturalis_834 | 0.174 |
| Hyperaspis_reppensis_666 | Scymnus_suturalis_834 | 0.174 |
| Chilocorus_nigritus_29 | Scymnus_suturalis_843 | 0.174 |
| Chilocorus_nigritus_29 | Scymnus_suturalis_845 | 0.174 |
| Coccidula_scutellata_77 | Scymnus_sinuanodulus_831 | 0.174 |
| Chilocorus_nigritus_28 | Scymnus_suturalis_833 | 0.174 |
| Brumus_quadripustulatus_17 | Scymnus_suturalis_841 | 0.174 |
| Brumus_quadripustulatus_17 | Scymnus_suturalis_842 | 0.174 |
| Brumus_quadripustulatus_11 | Scymnus_suturalis_843 | 0.174 |
| Brumus_quadripustulatus_13 | Scymnus_suturalis_843 | 0.174 |
| Brumus_quadripustulatus_14 | Scymnus_suturalis_843 | 0.174 |
| Brumus_quadripustulatus_3 | Scymnus_suturalis_843 | 0.174 |
| Brumus_quadripustulatus_4 | Scymnus_suturalis_843 | 0.174 |
| Brumus_quadripustulatus_7 | Scymnus_suturalis_843 | 0.174 |
| Brumus_quadripustulatus_17 | Scymnus_suturalis_844 | 0.174 |
| Brumus_quadripustulatus_11 | Scymnus_suturalis_845 | 0.174 |
| Brumus_quadripustulatus_13 | Scymnus_suturalis_845 | 0.174 |
| Brumus_quadripustulatus_14 | Scymnus_suturalis_845 | 0.174 |
| Brumus_quadripustulatus_3 | Scymnus_suturalis_845 | 0.174 |
| Brumus_quadripustulatus_4 | Scymnus_suturalis_845 | 0.174 |

| | | |
|---|---|---|
| Brumus_quadripustulatus_7 | Scymnus_suturalis_845 | 0.174 |
| Brumus_quadripustulatus_17 | Scymnus_suturalis_846 | 0.174 |
| Brumus_quadripustulatus_17 | Scymnus_suturalis_847 | 0.174 |
| Brumus_quadripustulatus_17 | Scymnus_suturalis_848 | 0.174 |
| Coccinella_hieroglyphica_226 | Tytthaspis_sedecimpunctata_580 | 0.174 |
| Coccinella_quinquepunctata_237 | Tytthaspis_sedecimpunctata_580 | 0.174 |
| Psyllobora_vigintimaculata_574 | Tytthaspis_sedecimpunctata_580 | 0.174 |
| Illeis_galbula_474 | Tytthaspis_sedecimpunctata_587 | 0.174 |
| Illeis_galbula_475 | Tytthaspis_sedecimpunctata_587 | 0.174 |
| Illeis_galbula_476 | Tytthaspis_sedecimpunctata_587 | 0.174 |
| Scymnus_haemorrhoidalis_750 | Tytthaspis_sedecimpunctata_589 | 0.174 |
| Scymnus_haemorrhoidalis_751 | Tytthaspis_sedecimpunctata_589 | 0.174 |
| Scymnus_haemorrhoidalis_754 | Tytthaspis_sedecimpunctata_589 | 0.174 |
| Scymnus_haemorrhoidalis_757 | Tytthaspis_sedecimpunctata_589 | 0.174 |
| Scymnus_haemorrhoidalis_758 | Tytthaspis_sedecimpunctata_589 | 0.174 |
| Coccinella_quinquepunctata_232 | Tytthaspis_sedecimpunctata_591 | 0.174 |
| Coccinella_quinquepunctata_236 | Tytthaspis_sedecimpunctata_591 | 0.174 |
| Coccinella_quinquepunctata_239 | Tytthaspis_sedecimpunctata_591 | 0.174 |
| Hyperaspis_lateralis_654 | Scymnus_rubromaculatus_822 | 0.174 |
| Hyperaspis_lateralis_654 | Scymnus_rubromaculatus_823 | 0.174 |
| Hyperaspis_lateralis_654 | Scymnus_rubromaculatus_824 | 0.174 |
| Hyperaspis_lateralis_654 | Scymnus_rubromaculatus_825 | 0.174 |
| Hyperaspis_lateralis_654 | Scymnus_rubromaculatus_826 | 0.174 |
| Hyperaspis_lateralis_654 | Scymnus_rubromaculatus_827 | 0.174 |
| Brumus_quadripustulatus_17 | Scymnus_suturalis_832 | 0.174 |
| Brumus_quadripustulatus_9 | Scymnus_suturalis_833 | 0.174 |
| Brumus_quadripustulatus_17 | Scymnus_suturalis_835 | 0.174 |
| Brumus_quadripustulatus_17 | Scymnus_suturalis_836 | 0.174 |
| Brumus_quadripustulatus_15 | Scymnus_suturalis_838 | 0.174 |
| Brumus_quadripustulatus_16 | Scymnus_suturalis_838 | 0.174 |
| Brumus_quadripustulatus_6 | Scymnus_suturalis_838 | 0.174 |
| Brumus_quadripustulatus_8 | Scymnus_suturalis_838 | 0.174 |
| Brumus_quadripustulatus_17 | Scymnus_suturalis_839 | 0.174 |
| Brumus_quadripustulatus_15 | Scymnus_suturalis_840 | 0.174 |
| Brumus_quadripustulatus_16 | Scymnus_suturalis_840 | 0.174 |
| Brumus_quadripustulatus_6 | Scymnus_suturalis_840 | 0.174 |
| Brumus_quadripustulatus_8 | Scymnus_suturalis_840 | 0.174 |
| Anatis_ocellata_136 | Scymnus_suturalis_843 | 0.174 |
| Anatis_ocellata_136 | Scymnus_suturalis_845 | 0.174 |
| Brumus_quadripustulatus_12 | Scymnus_suturalis_841 | 0.174 |
| Brumus_quadripustulatus_12 | Scymnus_suturalis_842 | 0.174 |
| Brumus_quadripustulatus_9 | Scymnus_suturalis_843 | 0.174 |
| Brumus_quadripustulatus_12 | Scymnus_suturalis_844 | 0.174 |
| Brumus_quadripustulatus_9 | Scymnus_suturalis_845 | 0.174 |
| Brumus_quadripustulatus_12 | Scymnus_suturalis_846 | 0.174 |
| Brumus_quadripustulatus_12 | Scymnus_suturalis_847 | 0.174 |
| Brumus_quadripustulatus_12 | Scymnus_suturalis_848 | 0.174 |
| Hippodamia_notata_422 | Tytthaspis_sedecimpunctata_583 | 0.174 |
| Hippodamia_notata_423 | Tytthaspis_sedecimpunctata_583 | 0.174 |
| Hippodamia_notata_424 | Tytthaspis_sedecimpunctata_583 | 0.174 |
| Hippodamia_notata_426 | Tytthaspis_sedecimpunctata_583 | 0.174 |
| Hippodamia_notata_427 | Tytthaspis_sedecimpunctata_583 | 0.174 |
| Psyllobora_vigintiduopunctata_571 | Tytthaspis_sedecimpunctata_584 | 0.174 |
| Cycloneda_sanguinea_342 | Tytthaspis_sedecimpunctata_585 | 0.174 |
| Scymnus_haemorrhoidalis_750 | Tytthaspis_sedecimpunctata_587 | 0.174 |
| Scymnus_haemorrhoidalis_751 | Tytthaspis_sedecimpunctata_587 | 0.174 |

| | | |
|---|---|---|
| Scymnus_haemorrhoidalis_754 | Tytthaspis_sedecimpunctata_587 | 0.174 |
| Scymnus_haemorrhoidalis_757 | Tytthaspis_sedecimpunctata_587 | 0.174 |
| Scymnus_haemorrhoidalis_758 | Tytthaspis_sedecimpunctata_587 | 0.174 |
| Scymnus_haemorrhoidalis_755 | Tytthaspis_sedecimpunctata_589 | 0.174 |
| Psyllobora_vigintiduopunctata_570 | Tytthaspis_sedecimpunctata_591 | 0.174 |
| Psyllobora_vigintiduopunctata_572 | Tytthaspis_sedecimpunctata_591 | 0.174 |
| Scymnus_auritus_699 | Scymnus_rubromaculatus_822 | 0.174 |
| Scymnus_auritus_699 | Scymnus_rubromaculatus_823 | 0.174 |
| Scymnus_auritus_699 | Scymnus_rubromaculatus_824 | 0.174 |
| Scymnus_auritus_699 | Scymnus_rubromaculatus_825 | 0.174 |
| Scymnus_auritus_699 | Scymnus_rubromaculatus_826 | 0.174 |
| Scymnus_auritus_699 | Scymnus_rubromaculatus_827 | 0.174 |
| Brumus_quadripustulatus_12 | Scymnus_suturalis_832 | 0.174 |
| Nephus_quadrimaculatus_668 | Scymnus_suturalis_833 | 0.174 |
| Brumus_quadripustulatus_15 | Scymnus_suturalis_834 | 0.174 |
| Brumus_quadripustulatus_16 | Scymnus_suturalis_834 | 0.174 |
| Brumus_quadripustulatus_6 | Scymnus_suturalis_834 | 0.174 |
| Brumus_quadripustulatus_8 | Scymnus_suturalis_834 | 0.174 |
| Brumus_quadripustulatus_12 | Scymnus_suturalis_835 | 0.174 |
| Brumus_quadripustulatus_12 | Scymnus_suturalis_836 | 0.174 |
| Brumus_quadripustulatus_15 | Scymnus_suturalis_837 | 0.174 |
| Brumus_quadripustulatus_16 | Scymnus_suturalis_837 | 0.174 |
| Brumus_quadripustulatus_6 | Scymnus_suturalis_837 | 0.174 |
| Brumus_quadripustulatus_8 | Scymnus_suturalis_837 | 0.174 |
| Nephus_quadrimaculatus_669 | Scymnus_suturalis_838 | 0.174 |
| Nephus_quadrimaculatus_670 | Scymnus_suturalis_838 | 0.174 |
| Nephus_quadrimaculatus_671 | Scymnus_suturalis_838 | 0.174 |
| Nephus_quadrimaculatus_672 | Scymnus_suturalis_838 | 0.174 |
| Nephus_quadrimaculatus_673 | Scymnus_suturalis_838 | 0.174 |
| Brumus_quadripustulatus_12 | Scymnus_suturalis_839 | 0.174 |
| Didion_punctatum_645 | Scymnus_sinuanodulus_831 | 0.174 |
| Nephus_quadrimaculatus_668 | Scymnus_suturalis_841 | 0.174 |
| Nephus_quadrimaculatus_668 | Scymnus_suturalis_842 | 0.174 |
| Nephus_quadrimaculatus_668 | Scymnus_suturalis_844 | 0.174 |
| Nephus_quadrimaculatus_668 | Scymnus_suturalis_846 | 0.174 |
| Nephus_quadrimaculatus_668 | Scymnus_suturalis_847 | 0.174 |
| Nephus_quadrimaculatus_668 | Scymnus_suturalis_848 | 0.174 |
| Hyperaspis_reppensis_665 | Subcoccinella_vigintiquatuorpunctata_623 | 0.174 |
| Hippodamia_notata_412 | Tytthaspis_sedecimpunctata_583 | 0.174 |
| Hippodamia_notata_414 | Tytthaspis_sedecimpunctata_583 | 0.174 |
| Hippodamia_notata_416 | Tytthaspis_sedecimpunctata_583 | 0.174 |
| Hippodamia_notata_417 | Tytthaspis_sedecimpunctata_583 | 0.174 |
| Hippodamia_notata_420 | Tytthaspis_sedecimpunctata_583 | 0.174 |
| Hippodamia_notata_425 | Tytthaspis_sedecimpunctata_583 | 0.174 |
| Hippodamia_notata_428 | Tytthaspis_sedecimpunctata_583 | 0.174 |
| Hippodamia_notata_430 | Tytthaspis_sedecimpunctata_583 | 0.174 |
| Hippodamia_glacialis_407 | Tytthaspis_sedecimpunctata_584 | 0.174 |
| Hippodamia_notata_422 | Tytthaspis_sedecimpunctata_584 | 0.174 |
| Hippodamia_notata_423 | Tytthaspis_sedecimpunctata_584 | 0.174 |
| Hippodamia_notata_424 | Tytthaspis_sedecimpunctata_584 | 0.174 |
| Hippodamia_notata_426 | Tytthaspis_sedecimpunctata_584 | 0.174 |
| Hippodamia_notata_427 | Tytthaspis_sedecimpunctata_584 | 0.174 |
| Scymnus_auritus_699 | Tytthaspis_sedecimpunctata_584 | 0.174 |
| Hippodamia_notata_415 | Tytthaspis_sedecimpunctata_585 | 0.174 |
| Cycloneda_sanguinea_340 | Tytthaspis_sedecimpunctata_586 | 0.174 |
| Cycloneda_sanguinea_341 | Tytthaspis_sedecimpunctata_586 | 0.174 |

| | | |
|---|---|---|
| Cycloneda_sanguinea_343 | Tytthaspis_sedecimpunctata_586 | 0.174 |
| Cycloneda_sanguinea_344 | Tytthaspis_sedecimpunctata_586 | 0.174 |
| Hippodamia_notata_429 | Tytthaspis_sedecimpunctata_586 | 0.174 |
| Scymnus_haemorrhoidalis_755 | Tytthaspis_sedecimpunctata_587 | 0.174 |
| Cycloneda_sanguinea_342 | Tytthaspis_sedecimpunctata_588 | 0.174 |
| Hippodamia_notata_429 | Tytthaspis_sedecimpunctata_588 | 0.174 |
| Hippodamia_tredecimpunctata_459 | Tytthaspis_sedecimpunctata_589 | 0.174 |
| Scymnus_auritus_699 | Tytthaspis_sedecimpunctata_591 | 0.174 |
| Neoharmonia_venusta_520 | Tytthaspis_sedecimpunctata_592 | 0.174 |
| Nephus_quadrimaculatus_668 | Scymnus_suturalis_832 | 0.174 |
| Nephus_quadrimaculatus_668 | Scymnus_suturalis_835 | 0.174 |
| Nephus_quadrimaculatus_668 | Scymnus_suturalis_836 | 0.174 |
| Nephus_quadrimaculatus_668 | Scymnus_suturalis_837 | 0.174 |
| Nephus_quadrimaculatus_668 | Scymnus_suturalis_839 | 0.174 |
| Hyperaspis_reppensis_665 | Subcoccinella_vigintiquatuorpunctata_620 | 0.174 |
| Hyperaspis_reppensis_665 | Subcoccinella_vigintiquatuorpunctata_621 | 0.174 |
| Hyperaspis_reppensis_667 | Subcoccinella_vigintiquatuorpunctata_622 | 0.174 |
| Hyperaspis_reppensis_664 | Subcoccinella_vigintiquatuorpunctata_626 | 0.174 |
| Hyperaspis_reppensis_666 | Subcoccinella_vigintiquatuorpunctata_626 | 0.174 |
| Hyperaspis_reppensis_667 | Subcoccinella_vigintiquatuorpunctata_626 | 0.174 |
| Hyperaspis_reppensis_665 | Subcoccinella_vigintiquatuorpunctata_628 | 0.174 |
| Hyperaspis_reppensis_665 | Subcoccinella_vigintiquatuorpunctata_629 | 0.174 |
| Hyperaspis_reppensis_665 | Subcoccinella_vigintiquatuorpunctata_631 | 0.174 |
| Neoharmonia_venusta_520 | Tytthaspis_sedecimpunctata_580 | 0.174 |
| Hippodamia_notata_412 | Tytthaspis_sedecimpunctata_584 | 0.174 |
| Hippodamia_notata_414 | Tytthaspis_sedecimpunctata_584 | 0.174 |
| Hippodamia_notata_416 | Tytthaspis_sedecimpunctata_584 | 0.174 |
| Hippodamia_notata_417 | Tytthaspis_sedecimpunctata_584 | 0.174 |
| Hippodamia_notata_420 | Tytthaspis_sedecimpunctata_584 | 0.174 |
| Hippodamia_notata_425 | Tytthaspis_sedecimpunctata_584 | 0.174 |
| Hippodamia_notata_428 | Tytthaspis_sedecimpunctata_584 | 0.174 |
| Hippodamia_notata_430 | Tytthaspis_sedecimpunctata_584 | 0.174 |
| Ceratomegilla_ulkei_221 | Tytthaspis_sedecimpunctata_586 | 0.174 |
| Ceratomegilla_ulkei_221 | Tytthaspis_sedecimpunctata_587 | 0.174 |
| Hippodamia_arctica_389 | Tytthaspis_sedecimpunctata_589 | 0.174 |
| Hippodamia_caseyi_391 | Tytthaspis_sedecimpunctata_589 | 0.174 |
| Hippodamia_convergens_393 | Tytthaspis_sedecimpunctata_589 | 0.174 |
| Hippodamia_convergens_394 | Tytthaspis_sedecimpunctata_589 | 0.174 |
| Hippodamia_convergens_397 | Tytthaspis_sedecimpunctata_589 | 0.174 |
| Hippodamia_convergens_398 | Tytthaspis_sedecimpunctata_589 | 0.174 |
| Hippodamia_convergens_399 | Tytthaspis_sedecimpunctata_589 | 0.174 |
| Hippodamia_convergens_401 | Tytthaspis_sedecimpunctata_589 | 0.174 |
| Hippodamia_convergens_402 | Tytthaspis_sedecimpunctata_589 | 0.174 |
| Hippodamia_convergens_404 | Tytthaspis_sedecimpunctata_589 | 0.174 |
| Hippodamia_convergens_405 | Tytthaspis_sedecimpunctata_589 | 0.174 |
| Hippodamia_convergens_406 | Tytthaspis_sedecimpunctata_589 | 0.174 |
| Hippodamia_moesta_411 | Tytthaspis_sedecimpunctata_589 | 0.174 |
| Hippodamia_quinquesignata_443 | Tytthaspis_sedecimpunctata_589 | 0.174 |
| Hippodamia_quinquesignata_444 | Tytthaspis_sedecimpunctata_589 | 0.174 |
| Hippodamia_quinquesignata_445 | Tytthaspis_sedecimpunctata_589 | 0.174 |
| Hippodamia_notata_415 | Tytthaspis_sedecimpunctata_591 | 0.174 |
| Neoharmonia_venusta_521 | Tytthaspis_sedecimpunctata_592 | 0.174 |
| Hippodamia_notata_413 | Tytthaspis_sedecimpunctata_593 | 0.174 |
| Hippodamia_notata_418 | Tytthaspis_sedecimpunctata_593 | 0.174 |
| Scymnus_haemorrhoidalis_747 | Scymnus_rubromaculatus_822 | 0.174 |
| Scymnus_haemorrhoidalis_748 | Scymnus_rubromaculatus_822 | 0.174 |

| | | |
|---|---|---|
| Scymnus_haemorrhoidalis_747 | Scymnus_rubromaculatus_823 | 0.174 |
| Scymnus_haemorrhoidalis_748 | Scymnus_rubromaculatus_823 | 0.174 |
| Scymnus_haemorrhoidalis_747 | Scymnus_rubromaculatus_824 | 0.174 |
| Scymnus_haemorrhoidalis_748 | Scymnus_rubromaculatus_824 | 0.174 |
| Scymnus_haemorrhoidalis_747 | Scymnus_rubromaculatus_825 | 0.174 |
| Scymnus_haemorrhoidalis_748 | Scymnus_rubromaculatus_825 | 0.174 |
| Scymnus_haemorrhoidalis_747 | Scymnus_rubromaculatus_826 | 0.174 |
| Scymnus_haemorrhoidalis_748 | Scymnus_rubromaculatus_826 | 0.174 |
| Scymnus_haemorrhoidalis_747 | Scymnus_rubromaculatus_827 | 0.174 |
| Scymnus_haemorrhoidalis_748 | Scymnus_rubromaculatus_827 | 0.174 |
| Brumus_quadripustulatus_10 | Tytthaspis_sedecimpunctata_586 | 0.174 |
| Neoharmonia_venusta_521 | Tytthaspis_sedecimpunctata_580 | 0.174 |
| Ceratomegilla_ulkei_219 | Tytthaspis_sedecimpunctata_585 | 0.174 |
| Hippodamia_caseyi_392 | Tytthaspis_sedecimpunctata_586 | 0.174 |
| Hippodamia_convergens_395 | Tytthaspis_sedecimpunctata_586 | 0.174 |
| Hippodamia_caseyi_391 | Tytthaspis_sedecimpunctata_587 | 0.174 |
| Hippodamia_convergens_393 | Tytthaspis_sedecimpunctata_587 | 0.174 |
| Hippodamia_convergens_394 | Tytthaspis_sedecimpunctata_587 | 0.174 |
| Hippodamia_convergens_397 | Tytthaspis_sedecimpunctata_587 | 0.174 |
| Hippodamia_convergens_398 | Tytthaspis_sedecimpunctata_587 | 0.174 |
| Hippodamia_convergens_399 | Tytthaspis_sedecimpunctata_587 | 0.174 |
| Hippodamia_convergens_401 | Tytthaspis_sedecimpunctata_587 | 0.174 |
| Hippodamia_convergens_402 | Tytthaspis_sedecimpunctata_587 | 0.174 |
| Hippodamia_convergens_404 | Tytthaspis_sedecimpunctata_587 | 0.174 |
| Hippodamia_convergens_405 | Tytthaspis_sedecimpunctata_587 | 0.174 |
| Hippodamia_convergens_406 | Tytthaspis_sedecimpunctata_587 | 0.174 |
| Hippodamia_moesta_411 | Tytthaspis_sedecimpunctata_587 | 0.174 |
| Hippodamia_quinquesignata_443 | Tytthaspis_sedecimpunctata_587 | 0.174 |
| Hippodamia_quinquesignata_444 | Tytthaspis_sedecimpunctata_587 | 0.174 |
| Hippodamia_quinquesignata_445 | Tytthaspis_sedecimpunctata_587 | 0.174 |
| Hippodamia_glacialis_408 | Tytthaspis_sedecimpunctata_591 | 0.174 |
| Hippodamia_glacialis_409 | Tytthaspis_sedecimpunctata_591 | 0.174 |
| Hippodamia_notata_413 | Tytthaspis_sedecimpunctata_591 | 0.174 |
| Hippodamia_notata_418 | Tytthaspis_sedecimpunctata_591 | 0.174 |
| Ceratomegilla_ulkei_219 | Tytthaspis_sedecimpunctata_593 | 0.174 |
| Myzia_pullata_519 | Vibidia_duodecimguttata_596 | 0.174 |
| Scymnus_camptodromus_704 | Scymnus_rubromaculatus_822 | 0.174 |
| Scymnus_haemorrhoidalis_750 | Scymnus_rubromaculatus_822 | 0.174 |
| Scymnus_camptodromus_704 | Scymnus_rubromaculatus_823 | 0.174 |
| Scymnus_haemorrhoidalis_750 | Scymnus_rubromaculatus_823 | 0.174 |
| Scymnus_camptodromus_704 | Scymnus_rubromaculatus_824 | 0.174 |
| Scymnus_haemorrhoidalis_750 | Scymnus_rubromaculatus_824 | 0.174 |
| Scymnus_camptodromus_704 | Scymnus_rubromaculatus_825 | 0.174 |
| Scymnus_haemorrhoidalis_750 | Scymnus_rubromaculatus_825 | 0.174 |
| Scymnus_camptodromus_704 | Scymnus_rubromaculatus_826 | 0.174 |
| Scymnus_haemorrhoidalis_750 | Scymnus_rubromaculatus_826 | 0.174 |
| Scymnus_camptodromus_704 | Scymnus_rubromaculatus_827 | 0.174 |
| Scymnus_haemorrhoidalis_750 | Scymnus_rubromaculatus_827 | 0.174 |
| Scymnus_pallipediformis_811 | Scymnus_sinuanodulus_828 | 0.174 |
| Hyperaspis_proba_661 | Scymnus_sinuanodulus_830 | 0.174 |
| Diomus_terminatus_647 | Scymnus_suturalis_838 | 0.174 |
| Diomus_terminatus_648 | Scymnus_suturalis_838 | 0.174 |
| Diomus_terminatus_647 | Scymnus_suturalis_840 | 0.174 |
| Diomus_terminatus_648 | Scymnus_suturalis_840 | 0.174 |
| Hyperaspis_proba_661 | Scymnus_sinuanodulus_828 | 0.175 |
| Scymnus_mimulus_775 | Scymnus_sinuanodulus_828 | 0.175 |

| | | |
|---|---|---|
| Scymnus_mimulus_776 | Scymnus_sinuanodulus_828 | 0.175 |
| Brumus_quadripustulatus_10 | Scymnus_suturalis_838 | 0.175 |
| Brumus_quadripustulatus_10 | Scymnus_suturalis_840 | 0.175 |
| Coccinula_quatuordecimpustulata_304 | Tytthaspis_sedecimpunctata_589 | 0.175 |
| Scymnus_mimulus_777 | Scymnus_sinuanodulus_830 | 0.175 |
| Brumus_quadripustulatus_10 | Scymnus_suturalis_834 | 0.175 |
| Brumus_quadripustulatus_10 | Scymnus_suturalis_837 | 0.175 |
| Hippodamia_arctica_390 | Tytthaspis_sedecimpunctata_587 | 0.175 |
| Hippodamia_arctica_390 | Tytthaspis_sedecimpunctata_588 | 0.175 |
| Hippodamia_arctica_390 | Tytthaspis_sedecimpunctata_591 | 0.175 |
| Coccidula_scutellata_77 | Scymnus_sinuanodulus_829 | 0.175 |
| Scymnus_mitior_779 | Scymnus_suturalis_845 | 0.175 |
| Calvia_decemguttata_187 | Tytthaspis_sedecimpunctata_584 | 0.175 |
| Calvia_decemguttata_188 | Tytthaspis_sedecimpunctata_584 | 0.175 |
| Calvia_decemguttata_189 | Tytthaspis_sedecimpunctata_584 | 0.175 |
| Calvia_decemguttata_190 | Tytthaspis_sedecimpunctata_584 | 0.175 |
| Calvia_decemguttata_191 | Tytthaspis_sedecimpunctata_584 | 0.175 |
| Calvia_decemguttata_192 | Tytthaspis_sedecimpunctata_584 | 0.175 |
| Calvia_decemguttata_196 | Tytthaspis_sedecimpunctata_584 | 0.175 |
| Calvia_decemguttata_197 | Tytthaspis_sedecimpunctata_584 | 0.175 |
| Calvia_decemguttata_198 | Tytthaspis_sedecimpunctata_584 | 0.175 |
| Coccinula_quatuordecimpustulata_304 | Tytthaspis_sedecimpunctata_587 | 0.175 |
| Hippodamia_tredecimpunctata_447 | Tytthaspis_sedecimpunctata_591 | 0.175 |
| Hippodamia_tredecimpunctata_448 | Tytthaspis_sedecimpunctata_591 | 0.175 |
| Hippodamia_tredecimpunctata_451 | Tytthaspis_sedecimpunctata_591 | 0.175 |
| Hippodamia_tredecimpunctata_454 | Tytthaspis_sedecimpunctata_591 | 0.175 |
| Hippodamia_tredecimpunctata_455 | Tytthaspis_sedecimpunctata_591 | 0.175 |
| Hippodamia_tredecimpunctata_456 | Tytthaspis_sedecimpunctata_591 | 0.175 |
| Scymnus_mitior_780 | Scymnus_sinuanodulus_828 | 0.175 |
| Scymnus_auritus_701 | Scymnus_sinuanodulus_830 | 0.175 |
| Scymnus_mimulus_778 | Scymnus_sinuanodulus_830 | 0.175 |
| Scymnus_mitior_779 | Scymnus_suturalis_834 | 0.175 |
| Scymnus_rubromaculatus_813 | Scymnus_sinuanodulus_831 | 0.175 |
| Scymnus_rubromaculatus_815 | Scymnus_sinuanodulus_831 | 0.175 |
| Scymnus_rubromaculatus_816 | Scymnus_sinuanodulus_831 | 0.175 |
| Scymnus_rubromaculatus_817 | Scymnus_sinuanodulus_831 | 0.175 |
| Scymnus_rubromaculatus_818 | Scymnus_sinuanodulus_831 | 0.175 |
| Scymnus_rubromaculatus_819 | Scymnus_sinuanodulus_831 | 0.175 |
| Scymnus_rubromaculatus_820 | Scymnus_sinuanodulus_831 | 0.175 |
| Scymnus_rubromaculatus_821 | Scymnus_sinuanodulus_831 | 0.175 |
| Scymnus_rubromaculatus_822 | Scymnus_sinuanodulus_831 | 0.175 |
| Scymnus_rubromaculatus_823 | Scymnus_sinuanodulus_831 | 0.175 |
| Scymnus_rubromaculatus_824 | Scymnus_sinuanodulus_831 | 0.175 |
| Scymnus_rubromaculatus_825 | Scymnus_sinuanodulus_831 | 0.175 |
| Scymnus_rubromaculatus_826 | Scymnus_sinuanodulus_831 | 0.175 |
| Scymnus_rubromaculatus_827 | Scymnus_sinuanodulus_831 | 0.175 |
| Brumus_quadripustulatus_10 | Scymnus_rubromaculatus_822 | 0.175 |
| Brumus_quadripustulatus_10 | Scymnus_rubromaculatus_823 | 0.175 |
| Brumus_quadripustulatus_10 | Scymnus_rubromaculatus_824 | 0.175 |
| Brumus_quadripustulatus_10 | Scymnus_rubromaculatus_825 | 0.175 |
| Brumus_quadripustulatus_10 | Scymnus_rubromaculatus_826 | 0.175 |
| Brumus_quadripustulatus_10 | Scymnus_rubromaculatus_827 | 0.175 |
| Didion_punctatum_645 | Scymnus_sinuanodulus_829 | 0.175 |
| Hyperaspis_oregona_658 | Scymnus_sinuanodulus_831 | 0.175 |
| Calvia_decemguttata_187 | Tytthaspis_sedecimpunctata_577 | 0.175 |
| Calvia_decemguttata_188 | Tytthaspis_sedecimpunctata_577 | 0.175 |

| | | |
|---|---|---|
| Calvia_decemguttata_189 | Tytthaspis_sedecimpunctata_577 | 0.175 |
| Calvia_decemguttata_190 | Tytthaspis_sedecimpunctata_577 | 0.175 |
| Calvia_decemguttata_191 | Tytthaspis_sedecimpunctata_577 | 0.175 |
| Calvia_decemguttata_192 | Tytthaspis_sedecimpunctata_577 | 0.175 |
| Calvia_decemguttata_196 | Tytthaspis_sedecimpunctata_577 | 0.175 |
| Calvia_decemguttata_197 | Tytthaspis_sedecimpunctata_577 | 0.175 |
| Calvia_decemguttata_198 | Tytthaspis_sedecimpunctata_577 | 0.175 |
| Calvia_decemguttata_187 | Tytthaspis_sedecimpunctata_578 | 0.175 |
| Calvia_decemguttata_188 | Tytthaspis_sedecimpunctata_578 | 0.175 |
| Calvia_decemguttata_189 | Tytthaspis_sedecimpunctata_578 | 0.175 |
| Calvia_decemguttata_190 | Tytthaspis_sedecimpunctata_578 | 0.175 |
| Calvia_decemguttata_191 | Tytthaspis_sedecimpunctata_578 | 0.175 |
| Calvia_decemguttata_192 | Tytthaspis_sedecimpunctata_578 | 0.175 |
| Calvia_decemguttata_196 | Tytthaspis_sedecimpunctata_578 | 0.175 |
| Calvia_decemguttata_197 | Tytthaspis_sedecimpunctata_578 | 0.175 |
| Calvia_decemguttata_198 | Tytthaspis_sedecimpunctata_578 | 0.175 |
| Calvia_decemguttata_187 | Tytthaspis_sedecimpunctata_579 | 0.175 |
| Calvia_decemguttata_188 | Tytthaspis_sedecimpunctata_579 | 0.175 |
| Calvia_decemguttata_189 | Tytthaspis_sedecimpunctata_579 | 0.175 |
| Calvia_decemguttata_190 | Tytthaspis_sedecimpunctata_579 | 0.175 |
| Calvia_decemguttata_191 | Tytthaspis_sedecimpunctata_579 | 0.175 |
| Calvia_decemguttata_192 | Tytthaspis_sedecimpunctata_579 | 0.175 |
| Calvia_decemguttata_196 | Tytthaspis_sedecimpunctata_579 | 0.175 |
| Calvia_decemguttata_197 | Tytthaspis_sedecimpunctata_579 | 0.175 |
| Calvia_decemguttata_198 | Tytthaspis_sedecimpunctata_579 | 0.175 |
| Calvia_decemguttata_187 | Tytthaspis_sedecimpunctata_581 | 0.175 |
| Calvia_decemguttata_188 | Tytthaspis_sedecimpunctata_581 | 0.175 |
| Calvia_decemguttata_189 | Tytthaspis_sedecimpunctata_581 | 0.175 |
| Calvia_decemguttata_190 | Tytthaspis_sedecimpunctata_581 | 0.175 |
| Calvia_decemguttata_191 | Tytthaspis_sedecimpunctata_581 | 0.175 |
| Calvia_decemguttata_192 | Tytthaspis_sedecimpunctata_581 | 0.175 |
| Calvia_decemguttata_196 | Tytthaspis_sedecimpunctata_581 | 0.175 |
| Calvia_decemguttata_197 | Tytthaspis_sedecimpunctata_581 | 0.175 |
| Calvia_decemguttata_198 | Tytthaspis_sedecimpunctata_581 | 0.175 |
| Calvia_decemguttata_187 | Tytthaspis_sedecimpunctata_582 | 0.175 |
| Calvia_decemguttata_188 | Tytthaspis_sedecimpunctata_582 | 0.175 |
| Calvia_decemguttata_189 | Tytthaspis_sedecimpunctata_582 | 0.175 |
| Calvia_decemguttata_190 | Tytthaspis_sedecimpunctata_582 | 0.175 |
| Calvia_decemguttata_191 | Tytthaspis_sedecimpunctata_582 | 0.175 |
| Calvia_decemguttata_192 | Tytthaspis_sedecimpunctata_582 | 0.175 |
| Calvia_decemguttata_196 | Tytthaspis_sedecimpunctata_582 | 0.175 |
| Calvia_decemguttata_197 | Tytthaspis_sedecimpunctata_582 | 0.175 |
| Calvia_decemguttata_198 | Tytthaspis_sedecimpunctata_582 | 0.175 |
| Calvia_decemguttata_193 | Tytthaspis_sedecimpunctata_583 | 0.175 |
| Calvia_decemguttata_194 | Tytthaspis_sedecimpunctata_583 | 0.175 |
| Calvia_decemguttata_187 | Tytthaspis_sedecimpunctata_585 | 0.175 |
| Calvia_decemguttata_188 | Tytthaspis_sedecimpunctata_585 | 0.175 |
| Calvia_decemguttata_189 | Tytthaspis_sedecimpunctata_585 | 0.175 |
| Calvia_decemguttata_190 | Tytthaspis_sedecimpunctata_585 | 0.175 |
| Calvia_decemguttata_191 | Tytthaspis_sedecimpunctata_585 | 0.175 |
| Calvia_decemguttata_192 | Tytthaspis_sedecimpunctata_585 | 0.175 |
| Calvia_decemguttata_196 | Tytthaspis_sedecimpunctata_585 | 0.175 |
| Calvia_decemguttata_197 | Tytthaspis_sedecimpunctata_585 | 0.175 |
| Calvia_decemguttata_198 | Tytthaspis_sedecimpunctata_585 | 0.175 |
| Coccinula_quatuordecimpustulata_300 | Tytthaspis_sedecimpunctata_587 | 0.175 |
| Calvia_decemguttata_187 | Tytthaspis_sedecimpunctata_589 | 0.175 |

| | | |
|---|---|---|
| Calvia_decemguttata_188 | Tytthaspis_sedecimpunctata_589 | 0.175 |
| Calvia_decemguttata_189 | Tytthaspis_sedecimpunctata_589 | 0.175 |
| Calvia_decemguttata_190 | Tytthaspis_sedecimpunctata_589 | 0.175 |
| Calvia_decemguttata_191 | Tytthaspis_sedecimpunctata_589 | 0.175 |
| Calvia_decemguttata_192 | Tytthaspis_sedecimpunctata_589 | 0.175 |
| Calvia_decemguttata_196 | Tytthaspis_sedecimpunctata_589 | 0.175 |
| Calvia_decemguttata_197 | Tytthaspis_sedecimpunctata_589 | 0.175 |
| Calvia_decemguttata_198 | Tytthaspis_sedecimpunctata_589 | 0.175 |
| Calvia_decemguttata_187 | Tytthaspis_sedecimpunctata_590 | 0.175 |
| Calvia_decemguttata_188 | Tytthaspis_sedecimpunctata_590 | 0.175 |
| Calvia_decemguttata_189 | Tytthaspis_sedecimpunctata_590 | 0.175 |
| Calvia_decemguttata_190 | Tytthaspis_sedecimpunctata_590 | 0.175 |
| Calvia_decemguttata_191 | Tytthaspis_sedecimpunctata_590 | 0.175 |
| Calvia_decemguttata_192 | Tytthaspis_sedecimpunctata_590 | 0.175 |
| Calvia_decemguttata_196 | Tytthaspis_sedecimpunctata_590 | 0.175 |
| Calvia_decemguttata_197 | Tytthaspis_sedecimpunctata_590 | 0.175 |
| Calvia_decemguttata_198 | Tytthaspis_sedecimpunctata_590 | 0.175 |
| Calvia_decemguttata_187 | Tytthaspis_sedecimpunctata_592 | 0.175 |
| Calvia_decemguttata_188 | Tytthaspis_sedecimpunctata_592 | 0.175 |
| Calvia_decemguttata_189 | Tytthaspis_sedecimpunctata_592 | 0.175 |
| Calvia_decemguttata_190 | Tytthaspis_sedecimpunctata_592 | 0.175 |
| Calvia_decemguttata_191 | Tytthaspis_sedecimpunctata_592 | 0.175 |
| Calvia_decemguttata_192 | Tytthaspis_sedecimpunctata_592 | 0.175 |
| Calvia_decemguttata_196 | Tytthaspis_sedecimpunctata_592 | 0.175 |
| Calvia_decemguttata_197 | Tytthaspis_sedecimpunctata_592 | 0.175 |
| Calvia_decemguttata_198 | Tytthaspis_sedecimpunctata_592 | 0.175 |
| Calvia_decemguttata_187 | Tytthaspis_sedecimpunctata_593 | 0.175 |
| Calvia_decemguttata_188 | Tytthaspis_sedecimpunctata_593 | 0.175 |
| Calvia_decemguttata_189 | Tytthaspis_sedecimpunctata_593 | 0.175 |
| Calvia_decemguttata_190 | Tytthaspis_sedecimpunctata_593 | 0.175 |
| Calvia_decemguttata_191 | Tytthaspis_sedecimpunctata_593 | 0.175 |
| Calvia_decemguttata_192 | Tytthaspis_sedecimpunctata_593 | 0.175 |
| Calvia_decemguttata_196 | Tytthaspis_sedecimpunctata_593 | 0.175 |
| Calvia_decemguttata_197 | Tytthaspis_sedecimpunctata_593 | 0.175 |
| Calvia_decemguttata_198 | Tytthaspis_sedecimpunctata_593 | 0.175 |
| Calvia_decemguttata_187 | Tytthaspis_sedecimpunctata_594 | 0.175 |
| Calvia_decemguttata_188 | Tytthaspis_sedecimpunctata_594 | 0.175 |
| Calvia_decemguttata_189 | Tytthaspis_sedecimpunctata_594 | 0.175 |
| Calvia_decemguttata_190 | Tytthaspis_sedecimpunctata_594 | 0.175 |
| Calvia_decemguttata_191 | Tytthaspis_sedecimpunctata_594 | 0.175 |
| Calvia_decemguttata_192 | Tytthaspis_sedecimpunctata_594 | 0.175 |
| Calvia_decemguttata_196 | Tytthaspis_sedecimpunctata_594 | 0.175 |
| Calvia_decemguttata_197 | Tytthaspis_sedecimpunctata_594 | 0.175 |
| Calvia_decemguttata_198 | Tytthaspis_sedecimpunctata_594 | 0.175 |
| Calvia_decemguttata_187 | Tytthaspis_sedecimpunctata_595 | 0.175 |
| Calvia_decemguttata_188 | Tytthaspis_sedecimpunctata_595 | 0.175 |
| Calvia_decemguttata_189 | Tytthaspis_sedecimpunctata_595 | 0.175 |
| Calvia_decemguttata_190 | Tytthaspis_sedecimpunctata_595 | 0.175 |
| Calvia_decemguttata_191 | Tytthaspis_sedecimpunctata_595 | 0.175 |
| Calvia_decemguttata_192 | Tytthaspis_sedecimpunctata_595 | 0.175 |
| Calvia_decemguttata_196 | Tytthaspis_sedecimpunctata_595 | 0.175 |
| Calvia_decemguttata_197 | Tytthaspis_sedecimpunctata_595 | 0.175 |
| Calvia_decemguttata_198 | Tytthaspis_sedecimpunctata_595 | 0.175 |
| Psyllobora_vigintiduopunctata_557 | Vibidia_duodecimguttata_596 | 0.175 |
| Psyllobora_vigintiduopunctata_563 | Vibidia_duodecimguttata_596 | 0.175 |
| Calvia_decemguttata_195 | Vibidia_duodecimguttata_599 | 0.175 |

| | | |
|---|---|---|
| Calvia_decemguttata_195 | Vibidia_duodecimguttata_601 | 0.175 |
| Calvia_decemguttata_195 | Vibidia_duodecimguttata_602 | 0.175 |
| Calvia_decemguttata_187 | Vibidia_duodecimguttata_603 | 0.175 |
| Calvia_decemguttata_188 | Vibidia_duodecimguttata_603 | 0.175 |
| Calvia_decemguttata_189 | Vibidia_duodecimguttata_603 | 0.175 |
| Calvia_decemguttata_190 | Vibidia_duodecimguttata_603 | 0.175 |
| Calvia_decemguttata_191 | Vibidia_duodecimguttata_603 | 0.175 |
| Calvia_decemguttata_192 | Vibidia_duodecimguttata_603 | 0.175 |
| Calvia_decemguttata_196 | Vibidia_duodecimguttata_603 | 0.175 |
| Calvia_decemguttata_197 | Vibidia_duodecimguttata_603 | 0.175 |
| Calvia_decemguttata_198 | Vibidia_duodecimguttata_603 | 0.175 |
| Calvia_decemguttata_199 | Vibidia_duodecimguttata_603 | 0.175 |
| Calvia_decemguttata_200 | Vibidia_duodecimguttata_603 | 0.175 |
| Scymnus_mitior_780 | Scymnus_sinuanodulus_830 | 0.175 |
| Scymnus_camptodromus_706 | Tytthaspis_sedecimpunctata_583 | 0.175 |
| Coccinella_undecimpunctata_287 | Tytthaspis_sedecimpunctata_588 | 0.175 |
| Scymnus_camptodromus_706 | Tytthaspis_sedecimpunctata_589 | 0.175 |
| Hyperaspis_reppensis_666 | Scymnus_sinuanodulus_831 | 0.175 |
| Scymnus_haemorrhoidalis_747 | Scymnus_sinuanodulus_831 | 0.175 |
| Scymnus_haemorrhoidalis_748 | Scymnus_sinuanodulus_831 | 0.175 |
| Scymnus_haemorrhoidalis_755 | Scymnus_sinuanodulus_831 | 0.175 |
| Scymnus_camptodromus_706 | Tytthaspis_sedecimpunctata_580 | 0.175 |
| Calvia_decemguttata_187 | Tytthaspis_sedecimpunctata_586 | 0.175 |
| Calvia_decemguttata_188 | Tytthaspis_sedecimpunctata_586 | 0.175 |
| Calvia_decemguttata_189 | Tytthaspis_sedecimpunctata_586 | 0.175 |
| Calvia_decemguttata_190 | Tytthaspis_sedecimpunctata_586 | 0.175 |
| Calvia_decemguttata_191 | Tytthaspis_sedecimpunctata_586 | 0.175 |
| Calvia_decemguttata_192 | Tytthaspis_sedecimpunctata_586 | 0.175 |
| Calvia_decemguttata_196 | Tytthaspis_sedecimpunctata_586 | 0.175 |
| Calvia_decemguttata_197 | Tytthaspis_sedecimpunctata_586 | 0.175 |
| Calvia_decemguttata_198 | Tytthaspis_sedecimpunctata_586 | 0.175 |
| Calvia_decemguttata_193 | Tytthaspis_sedecimpunctata_588 | 0.175 |
| Calvia_decemguttata_194 | Tytthaspis_sedecimpunctata_588 | 0.175 |
| Scymnus_apetzi_690 | Scymnus_sinuanodulus_828 | 0.175 |
| Scymnus_apetzi_697 | Scymnus_sinuanodulus_828 | 0.175 |
| Psyllobora_vigintiduopunctata_569 | Vibidia_duodecimguttata_597 | 0.175 |
| Psyllobora_vigintiduopunctata_569 | Vibidia_duodecimguttata_600 | 0.175 |
| Scymnus_haemorrhoidalis_753 | Scymnus_sinuanodulus_831 | 0.175 |
| Platynaspis_luteorubra_50 | Tytthaspis_sedecimpunctata_580 | 0.175 |
| Platynaspis_luteorubra_50 | Tytthaspis_sedecimpunctata_584 | 0.175 |
| Platynaspis_luteorubra_50 | Tytthaspis_sedecimpunctata_586 | 0.175 |
| Psyllobora_vigintiduopunctata_569 | Vibidia_duodecimguttata_598 | 0.175 |
| Psyllobora_vigintiduopunctata_559 | Vibidia_duodecimguttata_599 | 0.175 |
| Psyllobora_vigintiduopunctata_559 | Vibidia_duodecimguttata_601 | 0.175 |
| Psyllobora_vigintiduopunctata_559 | Vibidia_duodecimguttata_602 | 0.175 |
| Psyllobora_vigintiduopunctata_569 | Vibidia_duodecimguttata_603 | 0.175 |
| Hyperaspis_oregona_658 | Scymnus_sinuanodulus_829 | 0.175 |
| Scymnus_rubromaculatus_813 | Scymnus_sinuanodulus_829 | 0.175 |
| Scymnus_rubromaculatus_815 | Scymnus_sinuanodulus_829 | 0.175 |
| Scymnus_rubromaculatus_816 | Scymnus_sinuanodulus_829 | 0.175 |
| Scymnus_rubromaculatus_817 | Scymnus_sinuanodulus_829 | 0.175 |
| Scymnus_rubromaculatus_818 | Scymnus_sinuanodulus_829 | 0.175 |
| Scymnus_rubromaculatus_819 | Scymnus_sinuanodulus_829 | 0.175 |
| Scymnus_rubromaculatus_820 | Scymnus_sinuanodulus_829 | 0.175 |
| Scymnus_rubromaculatus_821 | Scymnus_sinuanodulus_829 | 0.175 |
| Scymnus_rubromaculatus_822 | Scymnus_sinuanodulus_829 | 0.175 |

| | | |
|---|---|---|
| Scymnus_rubromaculatus_823 | Scymnus_sinuanodulus_829 | 0.175 |
| Scymnus_rubromaculatus_824 | Scymnus_sinuanodulus_829 | 0.175 |
| Scymnus_rubromaculatus_825 | Scymnus_sinuanodulus_829 | 0.175 |
| Scymnus_rubromaculatus_826 | Scymnus_sinuanodulus_829 | 0.175 |
| Scymnus_rubromaculatus_827 | Scymnus_sinuanodulus_829 | 0.175 |
| Hyperaspis_reppensis_666 | Scymnus_sinuanodulus_829 | 0.175 |
| Scymnus_haemorrhoidalis_747 | Scymnus_sinuanodulus_829 | 0.175 |
| Scymnus_haemorrhoidalis_748 | Scymnus_sinuanodulus_829 | 0.175 |
| Scymnus_haemorrhoidalis_755 | Scymnus_sinuanodulus_829 | 0.175 |
| Calvia_quatuordecimguttata_203 | Vibidia_duodecimguttata_596 | 0.175 |
| Calvia_quatuordecimguttata_209 | Vibidia_duodecimguttata_597 | 0.175 |
| Calvia_quatuordecimguttata_218 | Vibidia_duodecimguttata_597 | 0.175 |
| Calvia_quatuordecimguttata_209 | Vibidia_duodecimguttata_600 | 0.175 |
| Calvia_quatuordecimguttata_218 | Vibidia_duodecimguttata_600 | 0.175 |
| Scymnus_camptodromus_715 | Tytthaspis_sedecimpunctata_583 | 0.175 |
| Scymnus_camptodromus_715 | Tytthaspis_sedecimpunctata_580 | 0.176 |
| Scymnus_camptodromus_715 | Tytthaspis_sedecimpunctata_587 | 0.176 |
| Platynaspis_luteorubra_51 | Tytthaspis_sedecimpunctata_577 | 0.176 |
| Platynaspis_luteorubra_53 | Tytthaspis_sedecimpunctata_577 | 0.176 |
| Platynaspis_luteorubra_58 | Tytthaspis_sedecimpunctata_577 | 0.176 |
| Platynaspis_luteorubra_51 | Tytthaspis_sedecimpunctata_578 | 0.176 |
| Platynaspis_luteorubra_53 | Tytthaspis_sedecimpunctata_578 | 0.176 |
| Platynaspis_luteorubra_58 | Tytthaspis_sedecimpunctata_578 | 0.176 |
| Platynaspis_luteorubra_51 | Tytthaspis_sedecimpunctata_579 | 0.176 |
| Platynaspis_luteorubra_53 | Tytthaspis_sedecimpunctata_579 | 0.176 |
| Platynaspis_luteorubra_58 | Tytthaspis_sedecimpunctata_579 | 0.176 |
| Platynaspis_luteorubra_51 | Tytthaspis_sedecimpunctata_581 | 0.176 |
| Platynaspis_luteorubra_53 | Tytthaspis_sedecimpunctata_581 | 0.176 |
| Platynaspis_luteorubra_58 | Tytthaspis_sedecimpunctata_581 | 0.176 |
| Platynaspis_luteorubra_51 | Tytthaspis_sedecimpunctata_582 | 0.176 |
| Platynaspis_luteorubra_53 | Tytthaspis_sedecimpunctata_582 | 0.176 |
| Platynaspis_luteorubra_58 | Tytthaspis_sedecimpunctata_582 | 0.176 |
| Platynaspis_luteorubra_51 | Tytthaspis_sedecimpunctata_590 | 0.176 |
| Platynaspis_luteorubra_53 | Tytthaspis_sedecimpunctata_590 | 0.176 |
| Platynaspis_luteorubra_58 | Tytthaspis_sedecimpunctata_590 | 0.176 |
| Platynaspis_luteorubra_51 | Tytthaspis_sedecimpunctata_592 | 0.176 |
| Platynaspis_luteorubra_53 | Tytthaspis_sedecimpunctata_592 | 0.176 |
| Platynaspis_luteorubra_58 | Tytthaspis_sedecimpunctata_592 | 0.176 |
| Platynaspis_luteorubra_51 | Tytthaspis_sedecimpunctata_593 | 0.176 |
| Platynaspis_luteorubra_53 | Tytthaspis_sedecimpunctata_593 | 0.176 |
| Platynaspis_luteorubra_58 | Tytthaspis_sedecimpunctata_593 | 0.176 |
| Platynaspis_luteorubra_51 | Tytthaspis_sedecimpunctata_594 | 0.176 |
| Platynaspis_luteorubra_53 | Tytthaspis_sedecimpunctata_594 | 0.176 |
| Platynaspis_luteorubra_58 | Tytthaspis_sedecimpunctata_594 | 0.176 |
| Platynaspis_luteorubra_51 | Tytthaspis_sedecimpunctata_595 | 0.176 |
| Platynaspis_luteorubra_53 | Tytthaspis_sedecimpunctata_595 | 0.176 |
| Platynaspis_luteorubra_58 | Tytthaspis_sedecimpunctata_595 | 0.176 |
| Calvia_quatuordecimguttata_204 | Vibidia_duodecimguttata_597 | 0.176 |
| Calvia_quatuordecimguttata_208 | Vibidia_duodecimguttata_597 | 0.176 |
| Calvia_quatuordecimguttata_217 | Vibidia_duodecimguttata_597 | 0.176 |
| Calvia_quatuordecimguttata_204 | Vibidia_duodecimguttata_600 | 0.176 |
| Calvia_quatuordecimguttata_208 | Vibidia_duodecimguttata_600 | 0.176 |
| Calvia_quatuordecimguttata_217 | Vibidia_duodecimguttata_600 | 0.176 |
| Calvia_quatuordecimguttata_205 | Vibidia_duodecimguttata_603 | 0.176 |
| Hyperaspis_reppensis_667 | Tytthaspis_sedecimpunctata_586 | 0.176 |
| Hyperaspis_reppensis_667 | Tytthaspis_sedecimpunctata_588 | 0.176 |

| | | |
|---|---|---|
| Chilocorus_stigma_43 | Tytthaspis_sedecimpunctata_589 | 0.176 |
| Chilocorus_stigma_43 | Tytthaspis_sedecimpunctata_587 | 0.176 |
| Coccidula_scutellata_81 | Scymnus_suturalis_833 | 0.176 |
| Coccidula_scutellata_82 | Scymnus_suturalis_833 | 0.176 |
| Coccidula_scutellata_77 | Scymnus_suturalis_837 | 0.176 |
| Scymnus_haemorrhoidalis_753 | Scymnus_sinuanodulus_829 | 0.176 |
| Coccidula_scutellata_81 | Scymnus_suturalis_843 | 0.176 |
| Coccidula_scutellata_82 | Scymnus_suturalis_843 | 0.176 |
| Coccidula_scutellata_81 | Scymnus_suturalis_845 | 0.176 |
| Coccidula_scutellata_82 | Scymnus_suturalis_845 | 0.176 |
| Coccidula_scutellata_80 | Scymnus_suturalis_837 | 0.176 |
| Chilocorus_nigritus_25 | Scymnus_suturalis_838 | 0.176 |
| Chilocorus_nigritus_25 | Scymnus_suturalis_840 | 0.176 |
| Brumus_quadripustulatus_9 | Tytthaspis_sedecimpunctata_585 | 0.176 |
| Brumus_quadripustulatus_11 | Tytthaspis_sedecimpunctata_586 | 0.176 |
| Brumus_quadripustulatus_13 | Tytthaspis_sedecimpunctata_586 | 0.176 |
| Brumus_quadripustulatus_14 | Tytthaspis_sedecimpunctata_586 | 0.176 |
| Brumus_quadripustulatus_17 | Tytthaspis_sedecimpunctata_586 | 0.176 |
| Brumus_quadripustulatus_3 | Tytthaspis_sedecimpunctata_586 | 0.176 |
| Brumus_quadripustulatus_4 | Tytthaspis_sedecimpunctata_586 | 0.176 |
| Brumus_quadripustulatus_7 | Tytthaspis_sedecimpunctata_586 | 0.176 |
| Aphidecta_obliterata_179 | Tytthaspis_sedecimpunctata_589 | 0.176 |
| Brumus_quadripustulatus_9 | Tytthaspis_sedecimpunctata_593 | 0.176 |
| Coccidula_scutellata_77 | Scymnus_sinuanodulus_828 | 0.176 |
| Chilocorus_nigritus_25 | Scymnus_suturalis_834 | 0.176 |
| Chilocorus_nigritus_25 | Scymnus_suturalis_837 | 0.176 |
| Myzia_pullata_517 | Tytthaspis_sedecimpunctata_580 | 0.176 |
| Propylaea_quatuordecimpunctata_547 | Tytthaspis_sedecimpunctata_584 | 0.176 |
| Aphidecta_obliterata_179 | Tytthaspis_sedecimpunctata_586 | 0.176 |
| Myzia_pullata_517 | Tytthaspis_sedecimpunctata_586 | 0.176 |
| Propylaea_quatuordecimpunctata_540 | Tytthaspis_sedecimpunctata_586 | 0.176 |
| Propylaea_quatuordecimpunctata_543 | Tytthaspis_sedecimpunctata_586 | 0.176 |
| Propylaea_quatuordecimpunctata_548 | Tytthaspis_sedecimpunctata_586 | 0.176 |
| Propylaea_quatuordecimpunctata_539 | Tytthaspis_sedecimpunctata_587 | 0.176 |
| Propylaea_quatuordecimpunctata_542 | Tytthaspis_sedecimpunctata_587 | 0.176 |
| Propylaea_quatuordecimpunctata_546 | Tytthaspis_sedecimpunctata_587 | 0.176 |
| Aphidecta_obliterata_175 | Tytthaspis_sedecimpunctata_588 | 0.176 |
| Aphidecta_obliterata_177 | Tytthaspis_sedecimpunctata_588 | 0.176 |
| Aphidecta_obliterata_180 | Tytthaspis_sedecimpunctata_588 | 0.176 |
| Aphidecta_obliterata_182 | Tytthaspis_sedecimpunctata_588 | 0.176 |
| Myzia_pullata_517 | Tytthaspis_sedecimpunctata_588 | 0.176 |
| Propylaea_quatuordecimpunctata_540 | Tytthaspis_sedecimpunctata_588 | 0.176 |
| Propylaea_quatuordecimpunctata_543 | Tytthaspis_sedecimpunctata_588 | 0.176 |
| Propylaea_quatuordecimpunctata_548 | Tytthaspis_sedecimpunctata_588 | 0.176 |
| Chilocorus_stigma_45 | Tytthaspis_sedecimpunctata_589 | 0.176 |
| Propylaea_quatuordecimpunctata_532 | Tytthaspis_sedecimpunctata_591 | 0.176 |
| Propylaea_quatuordecimpunctata_533 | Tytthaspis_sedecimpunctata_591 | 0.176 |
| Propylaea_quatuordecimpunctata_534 | Tytthaspis_sedecimpunctata_591 | 0.176 |
| Propylaea_quatuordecimpunctata_535 | Tytthaspis_sedecimpunctata_591 | 0.176 |
| Propylaea_quatuordecimpunctata_536 | Tytthaspis_sedecimpunctata_591 | 0.176 |
| Propylaea_quatuordecimpunctata_537 | Tytthaspis_sedecimpunctata_591 | 0.176 |
| Propylaea_quatuordecimpunctata_538 | Tytthaspis_sedecimpunctata_591 | 0.176 |
| Propylaea_quatuordecimpunctata_541 | Tytthaspis_sedecimpunctata_591 | 0.176 |
| Propylaea_quatuordecimpunctata_544 | Tytthaspis_sedecimpunctata_591 | 0.176 |
| Propylaea_quatuordecimpunctata_549 | Tytthaspis_sedecimpunctata_591 | 0.176 |
| Platynaspis_luteorubra_51 | Scymnus_suturalis_838 | 0.176 |

| | | |
|---|---|---|
| Platynaspis_luteorubra_53 | Scymnus_suturalis_838 | 0.176 |
| Platynaspis_luteorubra_58 | Scymnus_suturalis_838 | 0.176 |
| Platynaspis_luteorubra_51 | Scymnus_suturalis_840 | 0.176 |
| Platynaspis_luteorubra_53 | Scymnus_suturalis_840 | 0.176 |
| Platynaspis_luteorubra_58 | Scymnus_suturalis_840 | 0.176 |
| Anatis_ocellata_133 | Scymnus_suturalis_843 | 0.176 |
| Anatis_ocellata_137 | Scymnus_suturalis_843 | 0.176 |
| Anatis_ocellata_133 | Scymnus_suturalis_845 | 0.176 |
| Anatis_ocellata_137 | Scymnus_suturalis_845 | 0.176 |
| Aphidecta_obliterata_175 | Tytthaspis_sedecimpunctata_577 | 0.176 |
| Aphidecta_obliterata_177 | Tytthaspis_sedecimpunctata_577 | 0.176 |
| Aphidecta_obliterata_180 | Tytthaspis_sedecimpunctata_577 | 0.176 |
| Aphidecta_obliterata_182 | Tytthaspis_sedecimpunctata_577 | 0.176 |
| Propylaea_quatuordecimpunctata_547 | Tytthaspis_sedecimpunctata_577 | 0.176 |
| Aphidecta_obliterata_175 | Tytthaspis_sedecimpunctata_578 | 0.176 |
| Aphidecta_obliterata_177 | Tytthaspis_sedecimpunctata_578 | 0.176 |
| Aphidecta_obliterata_180 | Tytthaspis_sedecimpunctata_578 | 0.176 |
| Aphidecta_obliterata_182 | Tytthaspis_sedecimpunctata_578 | 0.176 |
| Propylaea_quatuordecimpunctata_547 | Tytthaspis_sedecimpunctata_578 | 0.176 |
| Aphidecta_obliterata_175 | Tytthaspis_sedecimpunctata_579 | 0.176 |
| Aphidecta_obliterata_177 | Tytthaspis_sedecimpunctata_579 | 0.176 |
| Aphidecta_obliterata_180 | Tytthaspis_sedecimpunctata_579 | 0.176 |
| Aphidecta_obliterata_182 | Tytthaspis_sedecimpunctata_579 | 0.176 |
| Propylaea_quatuordecimpunctata_547 | Tytthaspis_sedecimpunctata_579 | 0.176 |
| Aphidecta_obliterata_175 | Tytthaspis_sedecimpunctata_581 | 0.176 |
| Aphidecta_obliterata_177 | Tytthaspis_sedecimpunctata_581 | 0.176 |
| Aphidecta_obliterata_180 | Tytthaspis_sedecimpunctata_581 | 0.176 |
| Aphidecta_obliterata_182 | Tytthaspis_sedecimpunctata_581 | 0.176 |
| Propylaea_quatuordecimpunctata_547 | Tytthaspis_sedecimpunctata_581 | 0.176 |
| Aphidecta_obliterata_175 | Tytthaspis_sedecimpunctata_582 | 0.176 |
| Aphidecta_obliterata_177 | Tytthaspis_sedecimpunctata_582 | 0.176 |
| Aphidecta_obliterata_180 | Tytthaspis_sedecimpunctata_582 | 0.176 |
| Aphidecta_obliterata_182 | Tytthaspis_sedecimpunctata_582 | 0.176 |
| Propylaea_quatuordecimpunctata_547 | Tytthaspis_sedecimpunctata_582 | 0.176 |
| Aphidecta_obliterata_179 | Tytthaspis_sedecimpunctata_583 | 0.176 |
| Adalia_bipunctata_105 | Tytthaspis_sedecimpunctata_584 | 0.176 |
| Coccinella_hieroglyphica_227 | Tytthaspis_sedecimpunctata_584 | 0.176 |
| Coccinella_trifasciata_275 | Tytthaspis_sedecimpunctata_584 | 0.176 |
| Coccinella_trifasciata_277 | Tytthaspis_sedecimpunctata_584 | 0.176 |
| Coccinella_trifasciata_278 | Tytthaspis_sedecimpunctata_584 | 0.176 |
| Coccinella_trifasciata_279 | Tytthaspis_sedecimpunctata_584 | 0.176 |
| Coccinella_trifasciata_281 | Tytthaspis_sedecimpunctata_584 | 0.176 |
| Aphidecta_obliterata_179 | Tytthaspis_sedecimpunctata_585 | 0.176 |
| Coccinella_hieroglyphica_225 | Tytthaspis_sedecimpunctata_586 | 0.176 |
| Aphidecta_obliterata_178 | Tytthaspis_sedecimpunctata_587 | 0.176 |
| Chilocorus_stigma_45 | Tytthaspis_sedecimpunctata_587 | 0.176 |
| Scymnus_camptodromus_704 | Tytthaspis_sedecimpunctata_587 | 0.176 |
| Coccinella_trifasciata_280 | Tytthaspis_sedecimpunctata_588 | 0.176 |
| Propylaea_quatuordecimpunctata_534 | Tytthaspis_sedecimpunctata_589 | 0.176 |
| Propylaea_quatuordecimpunctata_538 | Tytthaspis_sedecimpunctata_589 | 0.176 |
| Propylaea_quatuordecimpunctata_544 | Tytthaspis_sedecimpunctata_589 | 0.176 |
| Propylaea_quatuordecimpunctata_545 | Tytthaspis_sedecimpunctata_589 | 0.176 |
| Propylaea_quatuordecimpunctata_549 | Tytthaspis_sedecimpunctata_589 | 0.176 |
| Aphidecta_obliterata_175 | Tytthaspis_sedecimpunctata_590 | 0.176 |
| Aphidecta_obliterata_177 | Tytthaspis_sedecimpunctata_590 | 0.176 |
| Aphidecta_obliterata_180 | Tytthaspis_sedecimpunctata_590 | 0.176 |

| | | |
|---|---|---|
| Aphidecta_obliterata_182 | Tytthaspis_sedecimpunctata_590 | 0.176 |
| Propylaea_quatuordecimpunctata_547 | Tytthaspis_sedecimpunctata_590 | 0.176 |
| Anisosticta_novemdecimpunctata_174 | Tytthaspis_sedecimpunctata_592 | 0.176 |
| Aphidecta_obliterata_184 | Tytthaspis_sedecimpunctata_592 | 0.176 |
| Aphidecta_obliterata_175 | Tytthaspis_sedecimpunctata_593 | 0.176 |
| Aphidecta_obliterata_177 | Tytthaspis_sedecimpunctata_593 | 0.176 |
| Aphidecta_obliterata_180 | Tytthaspis_sedecimpunctata_593 | 0.176 |
| Aphidecta_obliterata_182 | Tytthaspis_sedecimpunctata_593 | 0.176 |
| Propylaea_quatuordecimpunctata_547 | Tytthaspis_sedecimpunctata_593 | 0.176 |
| Aphidecta_obliterata_175 | Tytthaspis_sedecimpunctata_594 | 0.176 |
| Aphidecta_obliterata_177 | Tytthaspis_sedecimpunctata_594 | 0.176 |
| Aphidecta_obliterata_180 | Tytthaspis_sedecimpunctata_594 | 0.176 |
| Aphidecta_obliterata_182 | Tytthaspis_sedecimpunctata_594 | 0.176 |
| Propylaea_quatuordecimpunctata_547 | Tytthaspis_sedecimpunctata_594 | 0.176 |
| Aphidecta_obliterata_175 | Tytthaspis_sedecimpunctata_595 | 0.176 |
| Aphidecta_obliterata_177 | Tytthaspis_sedecimpunctata_595 | 0.176 |
| Aphidecta_obliterata_180 | Tytthaspis_sedecimpunctata_595 | 0.176 |
| Aphidecta_obliterata_182 | Tytthaspis_sedecimpunctata_595 | 0.176 |
| Propylaea_quatuordecimpunctata_547 | Tytthaspis_sedecimpunctata_595 | 0.176 |
| Platynaspis_luteorubra_51 | Scymnus_suturalis_834 | 0.176 |
| Platynaspis_luteorubra_53 | Scymnus_suturalis_834 | 0.176 |
| Platynaspis_luteorubra_58 | Scymnus_suturalis_834 | 0.176 |
| Anatis_ocellata_132 | Scymnus_suturalis_838 | 0.176 |
| Anatis_ocellata_134 | Scymnus_suturalis_838 | 0.176 |
| Anatis_ocellata_135 | Scymnus_suturalis_838 | 0.176 |
| Anatis_ocellata_138 | Scymnus_suturalis_838 | 0.176 |
| Anatis_ocellata_139 | Scymnus_suturalis_838 | 0.176 |
| Anatis_ocellata_140 | Scymnus_suturalis_838 | 0.176 |
| Anatis_ocellata_141 | Scymnus_suturalis_838 | 0.176 |
| Anatis_ocellata_142 | Scymnus_suturalis_838 | 0.176 |
| Anatis_ocellata_144 | Scymnus_suturalis_838 | 0.176 |
| Anatis_ocellata_145 | Scymnus_suturalis_838 | 0.176 |
| Anatis_ocellata_146 | Scymnus_suturalis_838 | 0.176 |
| Hyperaspis_oregona_659 | Scymnus_suturalis_838 | 0.176 |
| Anatis_ocellata_132 | Scymnus_suturalis_840 | 0.176 |
| Anatis_ocellata_134 | Scymnus_suturalis_840 | 0.176 |
| Anatis_ocellata_135 | Scymnus_suturalis_840 | 0.176 |
| Anatis_ocellata_138 | Scymnus_suturalis_840 | 0.176 |
| Anatis_ocellata_139 | Scymnus_suturalis_840 | 0.176 |
| Anatis_ocellata_140 | Scymnus_suturalis_840 | 0.176 |
| Anatis_ocellata_141 | Scymnus_suturalis_840 | 0.176 |
| Anatis_ocellata_142 | Scymnus_suturalis_840 | 0.176 |
| Anatis_ocellata_144 | Scymnus_suturalis_840 | 0.176 |
| Anatis_ocellata_145 | Scymnus_suturalis_840 | 0.176 |
| Anatis_ocellata_146 | Scymnus_suturalis_840 | 0.176 |
| Hyperaspis_oregona_659 | Scymnus_suturalis_840 | 0.176 |
| Hyperaspis_lateralis_656 | Scymnus_suturalis_843 | 0.176 |
| Platynaspis_luteorubra_52 | Scymnus_suturalis_843 | 0.176 |
| Platynaspis_luteorubra_56 | Scymnus_suturalis_843 | 0.176 |
| Platynaspis_luteorubra_60 | Scymnus_suturalis_843 | 0.176 |
| Hyperaspis_lateralis_656 | Scymnus_suturalis_845 | 0.176 |
| Platynaspis_luteorubra_52 | Scymnus_suturalis_845 | 0.176 |
| Platynaspis_luteorubra_56 | Scymnus_suturalis_845 | 0.176 |
| Platynaspis_luteorubra_60 | Scymnus_suturalis_845 | 0.176 |
| Coccinella_hieroglyphica_227 | Tytthaspis_sedecimpunctata_577 | 0.176 |
| Coccinella_hieroglyphica_227 | Tytthaspis_sedecimpunctata_578 | 0.176 |

| | | |
|---|---|---|
| Coccinella_hieroglyphica_227 | Tytthaspis_sedecimpunctata_579 | 0.176 |
| Anisosticta_novemdecimpunctata_174 | Tytthaspis_sedecimpunctata_580 | 0.176 |
| Aphidecta_obliterata_184 | Tytthaspis_sedecimpunctata_580 | 0.176 |
| Propylaea_quatuordecimpunctata_547 | Tytthaspis_sedecimpunctata_580 | 0.176 |
| Coccinella_hieroglyphica_227 | Tytthaspis_sedecimpunctata_581 | 0.176 |
| Coccinella_hieroglyphica_227 | Tytthaspis_sedecimpunctata_582 | 0.176 |
| Adalia_bipunctata_101 | Tytthaspis_sedecimpunctata_584 | 0.176 |
| Adalia_bipunctata_104 | Tytthaspis_sedecimpunctata_584 | 0.176 |
| Coccinella_trifasciata_274 | Tytthaspis_sedecimpunctata_584 | 0.176 |
| Coccinella_hieroglyphica_227 | Tytthaspis_sedecimpunctata_585 | 0.176 |
| Coccinella_trifasciata_275 | Tytthaspis_sedecimpunctata_585 | 0.176 |
| Coccinella_trifasciata_277 | Tytthaspis_sedecimpunctata_585 | 0.176 |
| Coccinella_trifasciata_278 | Tytthaspis_sedecimpunctata_585 | 0.176 |
| Coccinella_trifasciata_279 | Tytthaspis_sedecimpunctata_585 | 0.176 |
| Coccinella_trifasciata_281 | Tytthaspis_sedecimpunctata_585 | 0.176 |
| Harmonia_axyridis_362 | Tytthaspis_sedecimpunctata_586 | 0.176 |
| Harmonia_axyridis_362 | Tytthaspis_sedecimpunctata_587 | 0.176 |
| Anatis_labiculata_123 | Tytthaspis_sedecimpunctata_589 | 0.176 |
| Anatis_labiculata_124 | Tytthaspis_sedecimpunctata_589 | 0.176 |
| Anatis_labiculata_125 | Tytthaspis_sedecimpunctata_589 | 0.176 |
| Anatis_labiculata_126 | Tytthaspis_sedecimpunctata_589 | 0.176 |
| Anatis_labiculata_127 | Tytthaspis_sedecimpunctata_589 | 0.176 |
| Coccinella_transversoguttata_265 | Tytthaspis_sedecimpunctata_589 | 0.176 |
| Coccinella_transversoguttata_266 | Tytthaspis_sedecimpunctata_589 | 0.176 |
| Coccinella_hieroglyphica_227 | Tytthaspis_sedecimpunctata_590 | 0.176 |
| Adalia_bipunctata_100 | Tytthaspis_sedecimpunctata_591 | 0.176 |
| Harmonia_axyridis_362 | Tytthaspis_sedecimpunctata_591 | 0.176 |
| Coccinella_hieroglyphica_227 | Tytthaspis_sedecimpunctata_592 | 0.176 |
| Coccinella_hieroglyphica_225 | Tytthaspis_sedecimpunctata_593 | 0.176 |
| Coccinella_hieroglyphica_227 | Tytthaspis_sedecimpunctata_594 | 0.176 |
| Coccinella_hieroglyphica_227 | Tytthaspis_sedecimpunctata_595 | 0.176 |
| Platynaspis_luteorubra_61 | Scymnus_suturalis_833 | 0.176 |
| Anatis_ocellata_132 | Scymnus_suturalis_834 | 0.176 |
| Anatis_ocellata_134 | Scymnus_suturalis_834 | 0.176 |
| Anatis_ocellata_135 | Scymnus_suturalis_834 | 0.176 |
| Anatis_ocellata_138 | Scymnus_suturalis_834 | 0.176 |
| Anatis_ocellata_139 | Scymnus_suturalis_834 | 0.176 |
| Anatis_ocellata_140 | Scymnus_suturalis_834 | 0.176 |
| Anatis_ocellata_141 | Scymnus_suturalis_834 | 0.176 |
| Anatis_ocellata_142 | Scymnus_suturalis_834 | 0.176 |
| Anatis_ocellata_144 | Scymnus_suturalis_834 | 0.176 |
| Anatis_ocellata_145 | Scymnus_suturalis_834 | 0.176 |
| Anatis_ocellata_146 | Scymnus_suturalis_834 | 0.176 |
| Hyperaspis_oregona_659 | Scymnus_suturalis_834 | 0.176 |
| Anatis_ocellata_132 | Scymnus_suturalis_837 | 0.176 |
| Anatis_ocellata_134 | Scymnus_suturalis_837 | 0.176 |
| Anatis_ocellata_135 | Scymnus_suturalis_837 | 0.176 |
| Anatis_ocellata_138 | Scymnus_suturalis_837 | 0.176 |
| Anatis_ocellata_139 | Scymnus_suturalis_837 | 0.176 |
| Anatis_ocellata_140 | Scymnus_suturalis_837 | 0.176 |
| Anatis_ocellata_141 | Scymnus_suturalis_837 | 0.176 |
| Anatis_ocellata_142 | Scymnus_suturalis_837 | 0.176 |
| Anatis_ocellata_144 | Scymnus_suturalis_837 | 0.176 |
| Anatis_ocellata_145 | Scymnus_suturalis_837 | 0.176 |
| Anatis_ocellata_146 | Scymnus_suturalis_837 | 0.176 |
| Platynaspis_luteorubra_52 | Scymnus_suturalis_837 | 0.176 |

| | | |
|---|---|---|
| Platynaspis_luteorubra_56 | Scymnus_suturalis_837 | 0.176 |
| Platynaspis_luteorubra_60 | Scymnus_suturalis_837 | 0.176 |
| Scymnus_pallipediformis_812 | Scymnus_suturalis_838 | 0.176 |
| Scymnus_pallipediformis_812 | Scymnus_suturalis_840 | 0.176 |
| Platynaspis_luteorubra_55 | Scymnus_suturalis_841 | 0.176 |
| Platynaspis_luteorubra_61 | Scymnus_suturalis_841 | 0.176 |
| Platynaspis_luteorubra_55 | Scymnus_suturalis_842 | 0.176 |
| Platynaspis_luteorubra_61 | Scymnus_suturalis_842 | 0.176 |
| Platynaspis_luteorubra_54 | Scymnus_suturalis_843 | 0.176 |
| Platynaspis_luteorubra_57 | Scymnus_suturalis_843 | 0.176 |
| Platynaspis_luteorubra_59 | Scymnus_suturalis_843 | 0.176 |
| Platynaspis_luteorubra_55 | Scymnus_suturalis_844 | 0.176 |
| Platynaspis_luteorubra_61 | Scymnus_suturalis_844 | 0.176 |
| Platynaspis_luteorubra_54 | Scymnus_suturalis_845 | 0.176 |
| Platynaspis_luteorubra_57 | Scymnus_suturalis_845 | 0.176 |
| Platynaspis_luteorubra_59 | Scymnus_suturalis_845 | 0.176 |
| Platynaspis_luteorubra_55 | Scymnus_suturalis_846 | 0.176 |
| Platynaspis_luteorubra_61 | Scymnus_suturalis_846 | 0.176 |
| Platynaspis_luteorubra_55 | Scymnus_suturalis_847 | 0.176 |
| Platynaspis_luteorubra_61 | Scymnus_suturalis_847 | 0.176 |
| Platynaspis_luteorubra_55 | Scymnus_suturalis_848 | 0.176 |
| Platynaspis_luteorubra_61 | Scymnus_suturalis_848 | 0.176 |
| Aphidecta_obliterata_178 | Tytthaspis_sedecimpunctata_584 | 0.176 |
| Hippodamia_alpina_385 | Tytthaspis_sedecimpunctata_584 | 0.176 |
| Hippodamia_alpina_386 | Tytthaspis_sedecimpunctata_584 | 0.176 |
| Hippodamia_alpina_387 | Tytthaspis_sedecimpunctata_584 | 0.176 |
| Adalia_bipunctata_98 | Tytthaspis_sedecimpunctata_585 | 0.176 |
| Coccinella_trifasciata_274 | Tytthaspis_sedecimpunctata_585 | 0.176 |
| Anatis_labiculata_126 | Tytthaspis_sedecimpunctata_586 | 0.176 |
| Coccinella_trifasciata_273 | Tytthaspis_sedecimpunctata_587 | 0.176 |
| Anatis_labiculata_123 | Tytthaspis_sedecimpunctata_588 | 0.176 |
| Anatis_labiculata_124 | Tytthaspis_sedecimpunctata_588 | 0.176 |
| Anatis_labiculata_125 | Tytthaspis_sedecimpunctata_588 | 0.176 |
| Anatis_labiculata_127 | Tytthaspis_sedecimpunctata_588 | 0.176 |
| Coccinella_quinquepunctata_232 | Tytthaspis_sedecimpunctata_589 | 0.176 |
| Coccinella_quinquepunctata_235 | Tytthaspis_sedecimpunctata_589 | 0.176 |
| Coccinella_quinquepunctata_237 | Tytthaspis_sedecimpunctata_589 | 0.176 |
| Coccinella_quinquepunctata_238 | Tytthaspis_sedecimpunctata_589 | 0.176 |
| Coccinella_quinquepunctata_240 | Tytthaspis_sedecimpunctata_589 | 0.176 |
| Coccinella_undecimpunctata_282 | Tytthaspis_sedecimpunctata_589 | 0.176 |
| Coccinella_undecimpunctata_283 | Tytthaspis_sedecimpunctata_589 | 0.176 |
| Coccinella_undecimpunctata_284 | Tytthaspis_sedecimpunctata_589 | 0.176 |
| Scymnus_auritus_700 | Tytthaspis_sedecimpunctata_589 | 0.176 |
| Adalia_bipunctata_98 | Tytthaspis_sedecimpunctata_593 | 0.176 |
| Platynaspis_luteorubra_55 | Scymnus_suturalis_832 | 0.176 |
| Platynaspis_luteorubra_61 | Scymnus_suturalis_832 | 0.176 |
| Scymnus_pallipediformis_812 | Scymnus_suturalis_834 | 0.176 |
| Platynaspis_luteorubra_55 | Scymnus_suturalis_835 | 0.176 |
| Platynaspis_luteorubra_61 | Scymnus_suturalis_835 | 0.176 |
| Platynaspis_luteorubra_55 | Scymnus_suturalis_836 | 0.176 |
| Platynaspis_luteorubra_61 | Scymnus_suturalis_836 | 0.176 |
| Platynaspis_luteorubra_54 | Scymnus_suturalis_837 | 0.176 |
| Platynaspis_luteorubra_57 | Scymnus_suturalis_837 | 0.176 |
| Platynaspis_luteorubra_59 | Scymnus_suturalis_837 | 0.176 |
| Platynaspis_luteorubra_55 | Scymnus_suturalis_839 | 0.176 |
| Platynaspis_luteorubra_61 | Scymnus_suturalis_839 | 0.176 |

| | | |
|---|---|---|
| Psyllobora_vigintimaculata_573 | Tytthaspis_sedecimpunctata_577 | 0.176 |
| Scymnus_auritus_703 | Tytthaspis_sedecimpunctata_577 | 0.176 |
| Psyllobora_vigintimaculata_573 | Tytthaspis_sedecimpunctata_578 | 0.176 |
| Scymnus_auritus_703 | Tytthaspis_sedecimpunctata_578 | 0.176 |
| Psyllobora_vigintimaculata_573 | Tytthaspis_sedecimpunctata_579 | 0.176 |
| Scymnus_auritus_703 | Tytthaspis_sedecimpunctata_579 | 0.176 |
| Psyllobora_vigintimaculata_573 | Tytthaspis_sedecimpunctata_581 | 0.176 |
| Scymnus_auritus_703 | Tytthaspis_sedecimpunctata_581 | 0.176 |
| Psyllobora_vigintimaculata_573 | Tytthaspis_sedecimpunctata_582 | 0.176 |
| Scymnus_auritus_703 | Tytthaspis_sedecimpunctata_582 | 0.176 |
| Illeis_galbula_474 | Tytthaspis_sedecimpunctata_584 | 0.176 |
| Illeis_galbula_475 | Tytthaspis_sedecimpunctata_584 | 0.176 |
| Illeis_galbula_476 | Tytthaspis_sedecimpunctata_584 | 0.176 |
| Coccinella_quinquepunctata_236 | Tytthaspis_sedecimpunctata_586 | 0.176 |
| Coccinella_quinquepunctata_239 | Tytthaspis_sedecimpunctata_586 | 0.176 |
| Scymnus_auritus_702 | Tytthaspis_sedecimpunctata_586 | 0.176 |
| Anatis_ocellata_143 | Tytthaspis_sedecimpunctata_587 | 0.176 |
| Coccinella_hieroglyphica_226 | Tytthaspis_sedecimpunctata_587 | 0.176 |
| Coccinella_quinquepunctata_232 | Tytthaspis_sedecimpunctata_587 | 0.176 |
| Coccinella_quinquepunctata_235 | Tytthaspis_sedecimpunctata_587 | 0.176 |
| Coccinella_quinquepunctata_238 | Tytthaspis_sedecimpunctata_587 | 0.176 |
| Coccinella_quinquepunctata_240 | Tytthaspis_sedecimpunctata_587 | 0.176 |
| Psyllobora_renifer_551 | Tytthaspis_sedecimpunctata_587 | 0.176 |
| Psyllobora_renifer_552 | Tytthaspis_sedecimpunctata_587 | 0.176 |
| Psyllobora_renifer_553 | Tytthaspis_sedecimpunctata_587 | 0.176 |
| Psyllobora_renifer_554 | Tytthaspis_sedecimpunctata_587 | 0.176 |
| Coccinella_quinquepunctata_237 | Tytthaspis_sedecimpunctata_588 | 0.176 |
| Scymnus_auritus_702 | Tytthaspis_sedecimpunctata_588 | 0.176 |
| Psyllobora_renifer_555 | Tytthaspis_sedecimpunctata_589 | 0.176 |
| Psyllobora_vigintimaculata_573 | Tytthaspis_sedecimpunctata_590 | 0.176 |
| Scymnus_auritus_703 | Tytthaspis_sedecimpunctata_590 | 0.176 |
| Hippodamia_alpina_383 | Tytthaspis_sedecimpunctata_591 | 0.176 |
| Psyllobora_vigintimaculata_575 | Tytthaspis_sedecimpunctata_591 | 0.176 |
| Psyllobora_vigintimaculata_576 | Tytthaspis_sedecimpunctata_591 | 0.176 |
| Psyllobora_vigintimaculata_573 | Tytthaspis_sedecimpunctata_593 | 0.176 |
| Psyllobora_vigintimaculata_573 | Tytthaspis_sedecimpunctata_594 | 0.176 |
| Scymnus_auritus_703 | Tytthaspis_sedecimpunctata_594 | 0.176 |
| Psyllobora_vigintimaculata_573 | Tytthaspis_sedecimpunctata_595 | 0.176 |
| Scymnus_auritus_703 | Tytthaspis_sedecimpunctata_595 | 0.176 |
| Psyllobora_vigintimaculata_574 | Tytthaspis_sedecimpunctata_577 | 0.176 |
| Psyllobora_vigintimaculata_574 | Tytthaspis_sedecimpunctata_578 | 0.176 |
| Psyllobora_vigintimaculata_574 | Tytthaspis_sedecimpunctata_579 | 0.176 |
| Psyllobora_vigintimaculata_574 | Tytthaspis_sedecimpunctata_581 | 0.176 |
| Psyllobora_vigintimaculata_574 | Tytthaspis_sedecimpunctata_582 | 0.176 |
| Coccinella_hieroglyphica_226 | Tytthaspis_sedecimpunctata_583 | 0.176 |
| Coccinella_quinquepunctata_237 | Tytthaspis_sedecimpunctata_583 | 0.176 |
| Psyllobora_vigintimaculata_575 | Tytthaspis_sedecimpunctata_583 | 0.176 |
| Psyllobora_vigintimaculata_576 | Tytthaspis_sedecimpunctata_583 | 0.176 |
| Scymnus_auritus_700 | Tytthaspis_sedecimpunctata_583 | 0.176 |
| Mulsantina_picta_484 | Tytthaspis_sedecimpunctata_584 | 0.176 |
| Scymnus_auritus_700 | Tytthaspis_sedecimpunctata_585 | 0.176 |
| Psyllobora_vigintimaculata_574 | Tytthaspis_sedecimpunctata_590 | 0.176 |
| Scymnus_auritus_700 | Tytthaspis_sedecimpunctata_592 | 0.176 |
| Coccinella_quinquepunctata_232 | Tytthaspis_sedecimpunctata_593 | 0.176 |
| Coccinella_quinquepunctata_236 | Tytthaspis_sedecimpunctata_593 | 0.176 |
| Coccinella_quinquepunctata_239 | Tytthaspis_sedecimpunctata_593 | 0.176 |

| | | |
|---|---|---|
| Psyllobora_vigintimaculata_574 | Tytthaspis_sedecimpunctata_593 | 0.176 |
| Psyllobora_vigintimaculata_574 | Tytthaspis_sedecimpunctata_594 | 0.176 |
| Psyllobora_vigintimaculata_574 | Tytthaspis_sedecimpunctata_595 | 0.176 |
| Brumus_quadripustulatus_15 | Scymnus_rubromaculatus_822 | 0.176 |
| Brumus_quadripustulatus_16 | Scymnus_rubromaculatus_822 | 0.176 |
| Brumus_quadripustulatus_6 | Scymnus_rubromaculatus_822 | 0.176 |
| Brumus_quadripustulatus_8 | Scymnus_rubromaculatus_822 | 0.176 |
| Scymnus_auritus_698 | Scymnus_rubromaculatus_822 | 0.176 |
| Scymnus_auritus_700 | Scymnus_rubromaculatus_822 | 0.176 |
| Brumus_quadripustulatus_15 | Scymnus_rubromaculatus_823 | 0.176 |
| Brumus_quadripustulatus_16 | Scymnus_rubromaculatus_823 | 0.176 |
| Brumus_quadripustulatus_6 | Scymnus_rubromaculatus_823 | 0.176 |
| Brumus_quadripustulatus_8 | Scymnus_rubromaculatus_823 | 0.176 |
| Scymnus_auritus_698 | Scymnus_rubromaculatus_823 | 0.176 |
| Scymnus_auritus_700 | Scymnus_rubromaculatus_823 | 0.176 |
| Brumus_quadripustulatus_15 | Scymnus_rubromaculatus_824 | 0.176 |
| Brumus_quadripustulatus_16 | Scymnus_rubromaculatus_824 | 0.176 |
| Brumus_quadripustulatus_6 | Scymnus_rubromaculatus_824 | 0.176 |
| Brumus_quadripustulatus_8 | Scymnus_rubromaculatus_824 | 0.176 |
| Scymnus_auritus_698 | Scymnus_rubromaculatus_824 | 0.176 |
| Scymnus_auritus_700 | Scymnus_rubromaculatus_824 | 0.176 |
| Brumus_quadripustulatus_15 | Scymnus_rubromaculatus_825 | 0.176 |
| Brumus_quadripustulatus_16 | Scymnus_rubromaculatus_825 | 0.176 |
| Brumus_quadripustulatus_6 | Scymnus_rubromaculatus_825 | 0.176 |
| Brumus_quadripustulatus_8 | Scymnus_rubromaculatus_825 | 0.176 |
| Scymnus_auritus_698 | Scymnus_rubromaculatus_825 | 0.176 |
| Scymnus_auritus_700 | Scymnus_rubromaculatus_825 | 0.176 |
| Brumus_quadripustulatus_15 | Scymnus_rubromaculatus_826 | 0.176 |
| Brumus_quadripustulatus_16 | Scymnus_rubromaculatus_826 | 0.176 |
| Brumus_quadripustulatus_6 | Scymnus_rubromaculatus_826 | 0.176 |
| Brumus_quadripustulatus_8 | Scymnus_rubromaculatus_826 | 0.176 |
| Scymnus_auritus_698 | Scymnus_rubromaculatus_826 | 0.176 |
| Scymnus_auritus_700 | Scymnus_rubromaculatus_826 | 0.176 |
| Brumus_quadripustulatus_15 | Scymnus_rubromaculatus_827 | 0.176 |
| Brumus_quadripustulatus_16 | Scymnus_rubromaculatus_827 | 0.176 |
| Brumus_quadripustulatus_6 | Scymnus_rubromaculatus_827 | 0.176 |
| Brumus_quadripustulatus_8 | Scymnus_rubromaculatus_827 | 0.176 |
| Scymnus_auritus_698 | Scymnus_rubromaculatus_827 | 0.176 |
| Scymnus_auritus_700 | Scymnus_rubromaculatus_827 | 0.176 |
| Brumus_quadripustulatus_17 | Scymnus_suturalis_838 | 0.176 |
| Brumus_quadripustulatus_17 | Scymnus_suturalis_840 | 0.176 |
| Chilocorus_nigritus_29 | Scymnus_suturalis_841 | 0.176 |
| Chilocorus_nigritus_29 | Scymnus_suturalis_842 | 0.176 |
| Chilocorus_nigritus_30 | Scymnus_suturalis_843 | 0.176 |
| Chilocorus_nigritus_29 | Scymnus_suturalis_844 | 0.176 |
| Chilocorus_nigritus_30 | Scymnus_suturalis_845 | 0.176 |
| Chilocorus_nigritus_29 | Scymnus_suturalis_846 | 0.176 |
| Chilocorus_nigritus_29 | Scymnus_suturalis_847 | 0.176 |
| Chilocorus_nigritus_29 | Scymnus_suturalis_848 | 0.176 |
| Chilocorus_nigritus_29 | Scymnus_suturalis_832 | 0.176 |
| Chilocorus_nigritus_29 | Scymnus_suturalis_835 | 0.176 |
| Chilocorus_nigritus_29 | Scymnus_suturalis_836 | 0.176 |
| Chilocorus_nigritus_29 | Scymnus_suturalis_839 | 0.176 |
| Psyllobora_vigintimaculata_575 | Vibidia_duodecimguttata_596 | 0.176 |
| Psyllobora_vigintimaculata_576 | Vibidia_duodecimguttata_596 | 0.176 |
| Coccidula_scutellata_80 | Scymnus_sinuanodulus_831 | 0.176 |

| | | |
|---|---|---|
| Anatis_ocellata_136 | Scymnus_suturalis_833 | 0.176 |
| Brumus_quadripustulatus_11 | Scymnus_suturalis_841 | 0.176 |
| Brumus_quadripustulatus_13 | Scymnus_suturalis_841 | 0.176 |
| Brumus_quadripustulatus_14 | Scymnus_suturalis_841 | 0.176 |
| Brumus_quadripustulatus_3 | Scymnus_suturalis_841 | 0.176 |
| Brumus_quadripustulatus_4 | Scymnus_suturalis_841 | 0.176 |
| Brumus_quadripustulatus_7 | Scymnus_suturalis_841 | 0.176 |
| Brumus_quadripustulatus_11 | Scymnus_suturalis_842 | 0.176 |
| Brumus_quadripustulatus_13 | Scymnus_suturalis_842 | 0.176 |
| Brumus_quadripustulatus_14 | Scymnus_suturalis_842 | 0.176 |
| Brumus_quadripustulatus_3 | Scymnus_suturalis_842 | 0.176 |
| Brumus_quadripustulatus_4 | Scymnus_suturalis_842 | 0.176 |
| Brumus_quadripustulatus_7 | Scymnus_suturalis_842 | 0.176 |
| Brumus_quadripustulatus_11 | Scymnus_suturalis_844 | 0.176 |
| Brumus_quadripustulatus_13 | Scymnus_suturalis_844 | 0.176 |
| Brumus_quadripustulatus_14 | Scymnus_suturalis_844 | 0.176 |
| Brumus_quadripustulatus_3 | Scymnus_suturalis_844 | 0.176 |
| Brumus_quadripustulatus_4 | Scymnus_suturalis_844 | 0.176 |
| Brumus_quadripustulatus_7 | Scymnus_suturalis_844 | 0.176 |
| Brumus_quadripustulatus_11 | Scymnus_suturalis_846 | 0.176 |
| Brumus_quadripustulatus_13 | Scymnus_suturalis_846 | 0.176 |
| Brumus_quadripustulatus_14 | Scymnus_suturalis_846 | 0.176 |
| Brumus_quadripustulatus_3 | Scymnus_suturalis_846 | 0.176 |
| Brumus_quadripustulatus_4 | Scymnus_suturalis_846 | 0.176 |
| Brumus_quadripustulatus_7 | Scymnus_suturalis_846 | 0.176 |
| Brumus_quadripustulatus_11 | Scymnus_suturalis_847 | 0.176 |
| Brumus_quadripustulatus_13 | Scymnus_suturalis_847 | 0.176 |
| Brumus_quadripustulatus_14 | Scymnus_suturalis_847 | 0.176 |
| Brumus_quadripustulatus_3 | Scymnus_suturalis_847 | 0.176 |
| Brumus_quadripustulatus_4 | Scymnus_suturalis_847 | 0.176 |
| Brumus_quadripustulatus_7 | Scymnus_suturalis_847 | 0.176 |
| Brumus_quadripustulatus_11 | Scymnus_suturalis_848 | 0.176 |
| Brumus_quadripustulatus_13 | Scymnus_suturalis_848 | 0.176 |
| Brumus_quadripustulatus_14 | Scymnus_suturalis_848 | 0.176 |
| Brumus_quadripustulatus_3 | Scymnus_suturalis_848 | 0.176 |
| Brumus_quadripustulatus_4 | Scymnus_suturalis_848 | 0.176 |
| Brumus_quadripustulatus_7 | Scymnus_suturalis_848 | 0.176 |
| Scymnus_auritus_700 | Tytthaspis_sedecimpunctata_580 | 0.176 |
| Cycloneda_sanguinea_340 | Tytthaspis_sedecimpunctata_584 | 0.176 |
| Cycloneda_sanguinea_341 | Tytthaspis_sedecimpunctata_584 | 0.176 |
| Cycloneda_sanguinea_343 | Tytthaspis_sedecimpunctata_584 | 0.176 |
| Cycloneda_sanguinea_344 | Tytthaspis_sedecimpunctata_584 | 0.176 |
| Psyllobora_vigintimaculata_575 | Tytthaspis_sedecimpunctata_584 | 0.176 |
| Psyllobora_vigintimaculata_576 | Tytthaspis_sedecimpunctata_584 | 0.176 |
| Scymnus_auritus_698 | Tytthaspis_sedecimpunctata_584 | 0.176 |
| Coccinella_hieroglyphica_226 | Tytthaspis_sedecimpunctata_591 | 0.176 |
| Coccinella_quinquepunctata_237 | Tytthaspis_sedecimpunctata_591 | 0.176 |
| Scymnus_auritus_698 | Tytthaspis_sedecimpunctata_591 | 0.176 |
| Brumus_quadripustulatus_11 | Scymnus_suturalis_832 | 0.176 |
| Brumus_quadripustulatus_13 | Scymnus_suturalis_832 | 0.176 |
| Brumus_quadripustulatus_14 | Scymnus_suturalis_832 | 0.176 |
| Brumus_quadripustulatus_3 | Scymnus_suturalis_832 | 0.176 |
| Brumus_quadripustulatus_4 | Scymnus_suturalis_832 | 0.176 |
| Brumus_quadripustulatus_7 | Scymnus_suturalis_832 | 0.176 |
| Brumus_quadripustulatus_17 | Scymnus_suturalis_834 | 0.176 |
| Brumus_quadripustulatus_11 | Scymnus_suturalis_835 | 0.176 |

| | | |
|---|---|---|
| Brumus_quadripustulatus_13 | Scymnus_suturalis_835 | 0.176 |
| Brumus_quadripustulatus_14 | Scymnus_suturalis_835 | 0.176 |
| Brumus_quadripustulatus_3 | Scymnus_suturalis_835 | 0.176 |
| Brumus_quadripustulatus_4 | Scymnus_suturalis_835 | 0.176 |
| Brumus_quadripustulatus_7 | Scymnus_suturalis_835 | 0.176 |
| Brumus_quadripustulatus_11 | Scymnus_suturalis_836 | 0.176 |
| Brumus_quadripustulatus_13 | Scymnus_suturalis_836 | 0.176 |
| Brumus_quadripustulatus_14 | Scymnus_suturalis_836 | 0.176 |
| Brumus_quadripustulatus_3 | Scymnus_suturalis_836 | 0.176 |
| Brumus_quadripustulatus_4 | Scymnus_suturalis_836 | 0.176 |
| Brumus_quadripustulatus_7 | Scymnus_suturalis_836 | 0.176 |
| Brumus_quadripustulatus_17 | Scymnus_suturalis_837 | 0.176 |
| Brumus_quadripustulatus_12 | Scymnus_suturalis_838 | 0.176 |
| Brumus_quadripustulatus_11 | Scymnus_suturalis_839 | 0.176 |
| Brumus_quadripustulatus_13 | Scymnus_suturalis_839 | 0.176 |
| Brumus_quadripustulatus_14 | Scymnus_suturalis_839 | 0.176 |
| Brumus_quadripustulatus_3 | Scymnus_suturalis_839 | 0.176 |
| Brumus_quadripustulatus_4 | Scymnus_suturalis_839 | 0.176 |
| Brumus_quadripustulatus_7 | Scymnus_suturalis_839 | 0.176 |
| Brumus_quadripustulatus_12 | Scymnus_suturalis_840 | 0.176 |
| Anatis_ocellata_136 | Scymnus_suturalis_841 | 0.176 |
| Anatis_ocellata_136 | Scymnus_suturalis_842 | 0.176 |
| Anatis_ocellata_136 | Scymnus_suturalis_844 | 0.176 |
| Anatis_ocellata_136 | Scymnus_suturalis_846 | 0.176 |
| Anatis_ocellata_136 | Scymnus_suturalis_847 | 0.176 |
| Anatis_ocellata_136 | Scymnus_suturalis_848 | 0.176 |
| Anatis_ocellata_136 | Scymnus_suturalis_832 | 0.176 |
| Anatis_ocellata_136 | Scymnus_suturalis_835 | 0.176 |
| Anatis_ocellata_136 | Scymnus_suturalis_836 | 0.176 |
| Anatis_ocellata_136 | Scymnus_suturalis_839 | 0.176 |
| Brumus_quadripustulatus_9 | Scymnus_suturalis_841 | 0.176 |
| Brumus_quadripustulatus_9 | Scymnus_suturalis_842 | 0.176 |
| Brumus_quadripustulatus_9 | Scymnus_suturalis_844 | 0.176 |
| Brumus_quadripustulatus_9 | Scymnus_suturalis_846 | 0.176 |
| Brumus_quadripustulatus_9 | Scymnus_suturalis_847 | 0.176 |
| Brumus_quadripustulatus_9 | Scymnus_suturalis_848 | 0.176 |
| Cycloneda_sanguinea_340 | Tytthaspis_sedecimpunctata_577 | 0.176 |
| Cycloneda_sanguinea_341 | Tytthaspis_sedecimpunctata_577 | 0.176 |
| Cycloneda_sanguinea_343 | Tytthaspis_sedecimpunctata_577 | 0.176 |
| Cycloneda_sanguinea_344 | Tytthaspis_sedecimpunctata_577 | 0.176 |
| Cycloneda_sanguinea_340 | Tytthaspis_sedecimpunctata_578 | 0.176 |
| Cycloneda_sanguinea_341 | Tytthaspis_sedecimpunctata_578 | 0.176 |
| Cycloneda_sanguinea_343 | Tytthaspis_sedecimpunctata_578 | 0.176 |
| Cycloneda_sanguinea_344 | Tytthaspis_sedecimpunctata_578 | 0.176 |
| Cycloneda_sanguinea_340 | Tytthaspis_sedecimpunctata_579 | 0.176 |
| Cycloneda_sanguinea_341 | Tytthaspis_sedecimpunctata_579 | 0.176 |
| Cycloneda_sanguinea_343 | Tytthaspis_sedecimpunctata_579 | 0.176 |
| Cycloneda_sanguinea_344 | Tytthaspis_sedecimpunctata_579 | 0.176 |
| Cycloneda_sanguinea_340 | Tytthaspis_sedecimpunctata_581 | 0.176 |
| Cycloneda_sanguinea_341 | Tytthaspis_sedecimpunctata_581 | 0.176 |
| Cycloneda_sanguinea_343 | Tytthaspis_sedecimpunctata_581 | 0.176 |
| Cycloneda_sanguinea_344 | Tytthaspis_sedecimpunctata_581 | 0.176 |
| Cycloneda_sanguinea_340 | Tytthaspis_sedecimpunctata_582 | 0.176 |
| Cycloneda_sanguinea_341 | Tytthaspis_sedecimpunctata_582 | 0.176 |
| Cycloneda_sanguinea_343 | Tytthaspis_sedecimpunctata_582 | 0.176 |
| Cycloneda_sanguinea_344 | Tytthaspis_sedecimpunctata_582 | 0.176 |

Supplementary Table S4 (continued)

| | | |
|---|---|---|
| Hippodamia_notata_412 | Tytthaspis_sedecimpunctata_586 | 0.176 |
| Hippodamia_notata_414 | Tytthaspis_sedecimpunctata_586 | 0.176 |
| Hippodamia_notata_416 | Tytthaspis_sedecimpunctata_586 | 0.176 |
| Hippodamia_notata_417 | Tytthaspis_sedecimpunctata_586 | 0.176 |
| Hippodamia_notata_420 | Tytthaspis_sedecimpunctata_586 | 0.176 |
| Hippodamia_notata_425 | Tytthaspis_sedecimpunctata_586 | 0.176 |
| Hippodamia_notata_428 | Tytthaspis_sedecimpunctata_586 | 0.176 |
| Hippodamia_notata_430 | Tytthaspis_sedecimpunctata_586 | 0.176 |
| Hippodamia_notata_412 | Tytthaspis_sedecimpunctata_588 | 0.176 |
| Hippodamia_notata_414 | Tytthaspis_sedecimpunctata_588 | 0.176 |
| Hippodamia_notata_416 | Tytthaspis_sedecimpunctata_588 | 0.176 |
| Hippodamia_notata_417 | Tytthaspis_sedecimpunctata_588 | 0.176 |
| Hippodamia_notata_420 | Tytthaspis_sedecimpunctata_588 | 0.176 |
| Hippodamia_notata_425 | Tytthaspis_sedecimpunctata_588 | 0.176 |
| Hippodamia_notata_428 | Tytthaspis_sedecimpunctata_588 | 0.176 |
| Hippodamia_notata_430 | Tytthaspis_sedecimpunctata_588 | 0.176 |
| Cycloneda_sanguinea_340 | Tytthaspis_sedecimpunctata_589 | 0.176 |
| Cycloneda_sanguinea_341 | Tytthaspis_sedecimpunctata_589 | 0.176 |
| Cycloneda_sanguinea_343 | Tytthaspis_sedecimpunctata_589 | 0.176 |
| Cycloneda_sanguinea_344 | Tytthaspis_sedecimpunctata_589 | 0.176 |
| Hippodamia_notata_421 | Tytthaspis_sedecimpunctata_589 | 0.176 |
| Hippodamia_notata_429 | Tytthaspis_sedecimpunctata_589 | 0.176 |
| Cycloneda_sanguinea_340 | Tytthaspis_sedecimpunctata_590 | 0.176 |
| Cycloneda_sanguinea_341 | Tytthaspis_sedecimpunctata_590 | 0.176 |
| Cycloneda_sanguinea_343 | Tytthaspis_sedecimpunctata_590 | 0.176 |
| Cycloneda_sanguinea_344 | Tytthaspis_sedecimpunctata_590 | 0.176 |
| Psyllobora_vigintiduopunctata_560 | Tytthaspis_sedecimpunctata_591 | 0.176 |
| Cycloneda_sanguinea_340 | Tytthaspis_sedecimpunctata_592 | 0.176 |
| Cycloneda_sanguinea_341 | Tytthaspis_sedecimpunctata_592 | 0.176 |
| Cycloneda_sanguinea_343 | Tytthaspis_sedecimpunctata_592 | 0.176 |
| Cycloneda_sanguinea_344 | Tytthaspis_sedecimpunctata_592 | 0.176 |
| Scymnus_auritus_699 | Tytthaspis_sedecimpunctata_593 | 0.176 |
| Cycloneda_sanguinea_340 | Tytthaspis_sedecimpunctata_594 | 0.176 |
| Cycloneda_sanguinea_341 | Tytthaspis_sedecimpunctata_594 | 0.176 |
| Cycloneda_sanguinea_343 | Tytthaspis_sedecimpunctata_594 | 0.176 |
| Cycloneda_sanguinea_344 | Tytthaspis_sedecimpunctata_594 | 0.176 |
| Cycloneda_sanguinea_340 | Tytthaspis_sedecimpunctata_595 | 0.176 |
| Cycloneda_sanguinea_341 | Tytthaspis_sedecimpunctata_595 | 0.176 |
| Cycloneda_sanguinea_343 | Tytthaspis_sedecimpunctata_595 | 0.176 |
| Cycloneda_sanguinea_344 | Tytthaspis_sedecimpunctata_595 | 0.176 |
| Hyperaspis_reppensis_664 | Scymnus_rubromaculatus_822 | 0.176 |
| Hyperaspis_reppensis_666 | Scymnus_rubromaculatus_822 | 0.176 |
| Hyperaspis_reppensis_664 | Scymnus_rubromaculatus_823 | 0.176 |
| Hyperaspis_reppensis_666 | Scymnus_rubromaculatus_823 | 0.176 |
| Hyperaspis_reppensis_664 | Scymnus_rubromaculatus_824 | 0.176 |
| Hyperaspis_reppensis_666 | Scymnus_rubromaculatus_824 | 0.176 |
| Hyperaspis_reppensis_664 | Scymnus_rubromaculatus_825 | 0.176 |
| Hyperaspis_reppensis_666 | Scymnus_rubromaculatus_825 | 0.176 |
| Hyperaspis_reppensis_664 | Scymnus_rubromaculatus_826 | 0.176 |
| Hyperaspis_reppensis_666 | Scymnus_rubromaculatus_826 | 0.176 |
| Hyperaspis_reppensis_664 | Scymnus_rubromaculatus_827 | 0.176 |
| Hyperaspis_reppensis_666 | Scymnus_rubromaculatus_827 | 0.176 |
| Brumus_quadripustulatus_9 | Scymnus_suturalis_832 | 0.176 |
| Brumus_quadripustulatus_12 | Scymnus_suturalis_834 | 0.176 |
| Brumus_quadripustulatus_9 | Scymnus_suturalis_835 | 0.176 |
| Brumus_quadripustulatus_9 | Scymnus_suturalis_836 | 0.176 |

| | | |
|---|---|---|
| Brumus_quadripustulatus_12 | Scymnus_suturalis_837 | 0.176 |
| Nephus_quadrimaculatus_668 | Scymnus_suturalis_838 | 0.176 |
| Brumus_quadripustulatus_9 | Scymnus_suturalis_839 | 0.176 |
| Neoharmonia_venusta_520 | Tytthaspis_sedecimpunctata_577 | 0.176 |
| Neoharmonia_venusta_520 | Tytthaspis_sedecimpunctata_578 | 0.176 |
| Neoharmonia_venusta_520 | Tytthaspis_sedecimpunctata_579 | 0.176 |
| Cycloneda_sanguinea_340 | Tytthaspis_sedecimpunctata_580 | 0.176 |
| Cycloneda_sanguinea_341 | Tytthaspis_sedecimpunctata_580 | 0.176 |
| Cycloneda_sanguinea_343 | Tytthaspis_sedecimpunctata_580 | 0.176 |
| Cycloneda_sanguinea_344 | Tytthaspis_sedecimpunctata_580 | 0.176 |
| Neoharmonia_venusta_520 | Tytthaspis_sedecimpunctata_581 | 0.176 |
| Neoharmonia_venusta_520 | Tytthaspis_sedecimpunctata_582 | 0.176 |
| Cycloneda_sanguinea_342 | Tytthaspis_sedecimpunctata_586 | 0.176 |
| Hippodamia_notata_421 | Tytthaspis_sedecimpunctata_586 | 0.176 |
| Hippodamia_arctica_389 | Tytthaspis_sedecimpunctata_587 | 0.176 |
| Hippodamia_notata_429 | Tytthaspis_sedecimpunctata_587 | 0.176 |
| Hippodamia_arctica_389 | Tytthaspis_sedecimpunctata_588 | 0.176 |
| Hippodamia_notata_421 | Tytthaspis_sedecimpunctata_588 | 0.176 |
| Neoharmonia_venusta_520 | Tytthaspis_sedecimpunctata_589 | 0.176 |
| Neoharmonia_venusta_520 | Tytthaspis_sedecimpunctata_590 | 0.176 |
| Hippodamia_arctica_389 | Tytthaspis_sedecimpunctata_591 | 0.176 |
| Hippodamia_notata_422 | Tytthaspis_sedecimpunctata_591 | 0.176 |
| Hippodamia_notata_423 | Tytthaspis_sedecimpunctata_591 | 0.176 |
| Hippodamia_notata_424 | Tytthaspis_sedecimpunctata_591 | 0.176 |
| Hippodamia_notata_426 | Tytthaspis_sedecimpunctata_591 | 0.176 |
| Hippodamia_notata_427 | Tytthaspis_sedecimpunctata_591 | 0.176 |
| Neoharmonia_venusta_520 | Tytthaspis_sedecimpunctata_593 | 0.176 |
| Neoharmonia_venusta_520 | Tytthaspis_sedecimpunctata_594 | 0.176 |
| Neoharmonia_venusta_520 | Tytthaspis_sedecimpunctata_595 | 0.176 |
| Scymnus_ferrugatus_723 | Scymnus_sinuanodulus_829 | 0.176 |
| Scymnus_ferrugatus_725 | Scymnus_sinuanodulus_829 | 0.176 |
| Scymnus_ferrugatus_726 | Scymnus_sinuanodulus_829 | 0.176 |
| Scymnus_ferrugatus_727 | Scymnus_sinuanodulus_829 | 0.176 |
| Scymnus_ferrugatus_728 | Scymnus_sinuanodulus_829 | 0.176 |
| Scymnus_ferrugatus_730 | Scymnus_sinuanodulus_829 | 0.176 |
| Scymnus_ferrugatus_731 | Scymnus_sinuanodulus_829 | 0.176 |
| Scymnus_ferrugatus_732 | Scymnus_sinuanodulus_829 | 0.176 |
| Scymnus_ferrugatus_733 | Scymnus_sinuanodulus_829 | 0.176 |
| Chilocorus_nigritus_25 | Scymnus_sinuanodulus_831 | 0.176 |
| Hyperaspis_reppensis_664 | Subcoccinella_vigintiquatuorpunctata_624 | 0.176 |
| Hyperaspis_reppensis_666 | Subcoccinella_vigintiquatuorpunctata_624 | 0.176 |
| Hyperaspis_reppensis_667 | Subcoccinella_vigintiquatuorpunctata_624 | 0.176 |
| Hyperaspis_reppensis_665 | Subcoccinella_vigintiquatuorpunctata_626 | 0.176 |
| Neoharmonia_venusta_521 | Tytthaspis_sedecimpunctata_577 | 0.176 |
| Neoharmonia_venusta_521 | Tytthaspis_sedecimpunctata_578 | 0.176 |
| Neoharmonia_venusta_521 | Tytthaspis_sedecimpunctata_579 | 0.176 |
| Neoharmonia_venusta_521 | Tytthaspis_sedecimpunctata_581 | 0.176 |
| Neoharmonia_venusta_521 | Tytthaspis_sedecimpunctata_582 | 0.176 |
| Ceratomegilla_ulkei_219 | Tytthaspis_sedecimpunctata_588 | 0.176 |
| Hippodamia_caseyi_392 | Tytthaspis_sedecimpunctata_589 | 0.176 |
| Hippodamia_convergens_395 | Tytthaspis_sedecimpunctata_589 | 0.176 |
| Neoharmonia_venusta_521 | Tytthaspis_sedecimpunctata_589 | 0.176 |
| Neoharmonia_venusta_521 | Tytthaspis_sedecimpunctata_590 | 0.176 |
| Hippodamia_notata_412 | Tytthaspis_sedecimpunctata_591 | 0.176 |
| Hippodamia_notata_414 | Tytthaspis_sedecimpunctata_591 | 0.176 |
| Hippodamia_notata_416 | Tytthaspis_sedecimpunctata_591 | 0.176 |

Supplementary Table S4 (continued)

| | | |
|---|---|---|
| Hippodamia_notata_417 | Tytthaspis_sedecimpunctata_591 | 0.176 |
| Hippodamia_notata_420 | Tytthaspis_sedecimpunctata_591 | 0.176 |
| Hippodamia_notata_425 | Tytthaspis_sedecimpunctata_591 | 0.176 |
| Hippodamia_notata_428 | Tytthaspis_sedecimpunctata_591 | 0.176 |
| Hippodamia_notata_430 | Tytthaspis_sedecimpunctata_591 | 0.176 |
| Neoharmonia_venusta_521 | Tytthaspis_sedecimpunctata_594 | 0.176 |
| Neoharmonia_venusta_521 | Tytthaspis_sedecimpunctata_595 | 0.176 |
| Scymnus_haemorrhoidalis_755 | Scymnus_rubromaculatus_822 | 0.176 |
| Scymnus_haemorrhoidalis_756 | Scymnus_rubromaculatus_822 | 0.176 |
| Scymnus_haemorrhoidalis_755 | Scymnus_rubromaculatus_823 | 0.176 |
| Scymnus_haemorrhoidalis_756 | Scymnus_rubromaculatus_823 | 0.176 |
| Scymnus_haemorrhoidalis_755 | Scymnus_rubromaculatus_824 | 0.176 |
| Scymnus_haemorrhoidalis_756 | Scymnus_rubromaculatus_824 | 0.176 |
| Scymnus_haemorrhoidalis_755 | Scymnus_rubromaculatus_825 | 0.176 |
| Scymnus_haemorrhoidalis_756 | Scymnus_rubromaculatus_825 | 0.176 |
| Scymnus_haemorrhoidalis_755 | Scymnus_rubromaculatus_826 | 0.176 |
| Scymnus_haemorrhoidalis_756 | Scymnus_rubromaculatus_826 | 0.176 |
| Scymnus_haemorrhoidalis_755 | Scymnus_rubromaculatus_827 | 0.176 |
| Scymnus_haemorrhoidalis_756 | Scymnus_rubromaculatus_827 | 0.176 |
| Scymnus_pallipediformis_811 | Scymnus_sinuanodulus_830 | 0.176 |
| Brumus_quadripustulatus_10 | Tytthaspis_sedecimpunctata_587 | 0.176 |
| Hippodamia_caseyi_392 | Tytthaspis_sedecimpunctata_587 | 0.176 |
| Hippodamia_convergens_395 | Tytthaspis_sedecimpunctata_587 | 0.176 |
| Ceratomegilla_ulkei_219 | Tytthaspis_sedecimpunctata_589 | 0.176 |
| Hippodamia_glacialis_407 | Tytthaspis_sedecimpunctata_591 | 0.176 |
| Scymnus_camptodromus_714 | Scymnus_rubromaculatus_822 | 0.176 |
| Scymnus_haemorrhoidalis_751 | Scymnus_rubromaculatus_822 | 0.176 |
| Scymnus_haemorrhoidalis_754 | Scymnus_rubromaculatus_822 | 0.176 |
| Scymnus_haemorrhoidalis_757 | Scymnus_rubromaculatus_822 | 0.176 |
| Scymnus_haemorrhoidalis_758 | Scymnus_rubromaculatus_822 | 0.176 |
| Scymnus_camptodromus_714 | Scymnus_rubromaculatus_823 | 0.176 |
| Scymnus_haemorrhoidalis_751 | Scymnus_rubromaculatus_823 | 0.176 |
| Scymnus_haemorrhoidalis_754 | Scymnus_rubromaculatus_823 | 0.176 |
| Scymnus_haemorrhoidalis_757 | Scymnus_rubromaculatus_823 | 0.176 |
| Scymnus_haemorrhoidalis_758 | Scymnus_rubromaculatus_823 | 0.176 |
| Scymnus_camptodromus_714 | Scymnus_rubromaculatus_824 | 0.176 |
| Scymnus_haemorrhoidalis_751 | Scymnus_rubromaculatus_824 | 0.176 |
| Scymnus_haemorrhoidalis_754 | Scymnus_rubromaculatus_824 | 0.176 |
| Scymnus_haemorrhoidalis_757 | Scymnus_rubromaculatus_824 | 0.176 |
| Scymnus_haemorrhoidalis_758 | Scymnus_rubromaculatus_824 | 0.176 |
| Scymnus_camptodromus_714 | Scymnus_rubromaculatus_825 | 0.176 |
| Scymnus_haemorrhoidalis_751 | Scymnus_rubromaculatus_825 | 0.176 |
| Scymnus_haemorrhoidalis_754 | Scymnus_rubromaculatus_825 | 0.176 |
| Scymnus_haemorrhoidalis_757 | Scymnus_rubromaculatus_825 | 0.176 |
| Scymnus_haemorrhoidalis_758 | Scymnus_rubromaculatus_825 | 0.176 |
| Scymnus_camptodromus_714 | Scymnus_rubromaculatus_826 | 0.176 |
| Scymnus_haemorrhoidalis_751 | Scymnus_rubromaculatus_826 | 0.176 |
| Scymnus_haemorrhoidalis_754 | Scymnus_rubromaculatus_826 | 0.176 |
| Scymnus_haemorrhoidalis_757 | Scymnus_rubromaculatus_826 | 0.176 |
| Scymnus_haemorrhoidalis_758 | Scymnus_rubromaculatus_826 | 0.176 |
| Scymnus_camptodromus_714 | Scymnus_rubromaculatus_827 | 0.176 |
| Scymnus_haemorrhoidalis_751 | Scymnus_rubromaculatus_827 | 0.176 |
| Scymnus_haemorrhoidalis_754 | Scymnus_rubromaculatus_827 | 0.176 |
| Scymnus_haemorrhoidalis_757 | Scymnus_rubromaculatus_827 | 0.176 |
| Scymnus_haemorrhoidalis_758 | Scymnus_rubromaculatus_827 | 0.176 |
| Coccidula_scutellata_84 | Scymnus_sinuanodulus_831 | 0.176 |

| | | |
|---|---|---|
| Scymnus_camptodromus_717 | Tytthaspis_sedecimpunctata_583 | 0.176 |
| Coccinula_quatuordecimpustulata_297 | Tytthaspis_sedecimpunctata_587 | 0.177 |
| Scymnus_haemorrhoidalis_752 | Scymnus_rubromaculatus_822 | 0.177 |
| Scymnus_haemorrhoidalis_753 | Scymnus_rubromaculatus_822 | 0.177 |
| Scymnus_haemorrhoidalis_752 | Scymnus_rubromaculatus_823 | 0.177 |
| Scymnus_haemorrhoidalis_753 | Scymnus_rubromaculatus_823 | 0.177 |
| Scymnus_haemorrhoidalis_752 | Scymnus_rubromaculatus_824 | 0.177 |
| Scymnus_haemorrhoidalis_753 | Scymnus_rubromaculatus_824 | 0.177 |
| Scymnus_haemorrhoidalis_752 | Scymnus_rubromaculatus_825 | 0.177 |
| Scymnus_haemorrhoidalis_753 | Scymnus_rubromaculatus_825 | 0.177 |
| Scymnus_haemorrhoidalis_752 | Scymnus_rubromaculatus_826 | 0.177 |
| Scymnus_haemorrhoidalis_753 | Scymnus_rubromaculatus_826 | 0.177 |
| Scymnus_haemorrhoidalis_752 | Scymnus_rubromaculatus_827 | 0.177 |
| Scymnus_haemorrhoidalis_753 | Scymnus_rubromaculatus_827 | 0.177 |
| Scymnus_mimulus_777 | Scymnus_sinuanodulus_828 | 0.177 |
| Scymnus_pallipediformis_812 | Scymnus_sinuanodulus_831 | 0.177 |
| Scymnus_camptodromus_717 | Tytthaspis_sedecimpunctata_580 | 0.177 |
| Scymnus_camptodromus_711 | Tytthaspis_sedecimpunctata_583 | 0.177 |
| Scymnus_camptodromus_717 | Tytthaspis_sedecimpunctata_587 | 0.177 |
| Scymnus_camptodromus_711 | Tytthaspis_sedecimpunctata_580 | 0.177 |
| Scymnus_camptodromus_711 | Tytthaspis_sedecimpunctata_587 | 0.177 |
| Scymnus_mimulus_778 | Scymnus_sinuanodulus_828 | 0.177 |
| Scymnus_mitior_779 | Scymnus_suturalis_833 | 0.177 |
| Hyperaspis_proba_660 | Subcoccinella_vigintiquatuorpunctata_630 | 0.177 |
| Hippodamia_arctica_390 | Tytthaspis_sedecimpunctata_586 | 0.177 |
| Coccidula_scutellata_80 | Scymnus_sinuanodulus_829 | 0.177 |
| Hyperaspis_proba_660 | Scymnus_sinuanodulus_830 | 0.177 |
| Scymnus_rubromaculatus_814 | Scymnus_sinuanodulus_831 | 0.177 |
| Scymnus_mitior_779 | Scymnus_suturalis_841 | 0.177 |
| Scymnus_mitior_779 | Scymnus_suturalis_842 | 0.177 |
| Scymnus_mitior_779 | Scymnus_suturalis_844 | 0.177 |
| Scymnus_mitior_780 | Scymnus_suturalis_845 | 0.177 |
| Scymnus_mitior_779 | Scymnus_suturalis_846 | 0.177 |
| Scymnus_mitior_779 | Scymnus_suturalis_847 | 0.177 |
| Scymnus_mitior_779 | Scymnus_suturalis_848 | 0.177 |
| Calvia_decemguttata_187 | Tytthaspis_sedecimpunctata_580 | 0.177 |
| Calvia_decemguttata_188 | Tytthaspis_sedecimpunctata_580 | 0.177 |
| Calvia_decemguttata_189 | Tytthaspis_sedecimpunctata_580 | 0.177 |
| Calvia_decemguttata_190 | Tytthaspis_sedecimpunctata_580 | 0.177 |
| Calvia_decemguttata_191 | Tytthaspis_sedecimpunctata_580 | 0.177 |
| Calvia_decemguttata_192 | Tytthaspis_sedecimpunctata_580 | 0.177 |
| Calvia_decemguttata_196 | Tytthaspis_sedecimpunctata_580 | 0.177 |
| Calvia_decemguttata_197 | Tytthaspis_sedecimpunctata_580 | 0.177 |
| Calvia_decemguttata_198 | Tytthaspis_sedecimpunctata_580 | 0.177 |
| Calvia_decemguttata_193 | Tytthaspis_sedecimpunctata_584 | 0.177 |
| Calvia_decemguttata_194 | Tytthaspis_sedecimpunctata_584 | 0.177 |
| Hippodamia_tredecimpunctata_446 | Tytthaspis_sedecimpunctata_591 | 0.177 |
| Hippodamia_tredecimpunctata_449 | Tytthaspis_sedecimpunctata_591 | 0.177 |
| Hippodamia_tredecimpunctata_450 | Tytthaspis_sedecimpunctata_591 | 0.177 |
| Hippodamia_tredecimpunctata_452 | Tytthaspis_sedecimpunctata_591 | 0.177 |
| Hippodamia_tredecimpunctata_453 | Tytthaspis_sedecimpunctata_591 | 0.177 |
| Psyllobora_vigintiduopunctata_557 | Vibidia_duodecimguttata_597 | 0.177 |
| Psyllobora_vigintiduopunctata_563 | Vibidia_duodecimguttata_597 | 0.177 |
| Psyllobora_vigintiduopunctata_557 | Vibidia_duodecimguttata_600 | 0.177 |
| Psyllobora_vigintiduopunctata_563 | Vibidia_duodecimguttata_600 | 0.177 |
| Scymnus_mitior_779 | Scymnus_suturalis_832 | 0.177 |

| | | |
|---|---|---|
| Scymnus_mitior_780 | Scymnus_suturalis_834 | 0.177 |
| Scymnus_mitior_779 | Scymnus_suturalis_835 | 0.177 |
| Scymnus_mitior_779 | Scymnus_suturalis_836 | 0.177 |
| Scymnus_mitior_779 | Scymnus_suturalis_837 | 0.177 |
| Scymnus_mitior_779 | Scymnus_suturalis_839 | 0.177 |
| Chilocorus_nigritus_25 | Scymnus_sinuanodulus_829 | 0.177 |
| Hyperaspis_proba_660 | Scymnus_sinuanodulus_828 | 0.177 |
| Calvia_decemguttata_193 | Tytthaspis_sedecimpunctata_577 | 0.177 |
| Calvia_decemguttata_194 | Tytthaspis_sedecimpunctata_577 | 0.177 |
| Calvia_decemguttata_193 | Tytthaspis_sedecimpunctata_578 | 0.177 |
| Calvia_decemguttata_194 | Tytthaspis_sedecimpunctata_578 | 0.177 |
| Calvia_decemguttata_193 | Tytthaspis_sedecimpunctata_579 | 0.177 |
| Calvia_decemguttata_194 | Tytthaspis_sedecimpunctata_579 | 0.177 |
| Calvia_decemguttata_193 | Tytthaspis_sedecimpunctata_581 | 0.177 |
| Calvia_decemguttata_194 | Tytthaspis_sedecimpunctata_581 | 0.177 |
| Calvia_decemguttata_193 | Tytthaspis_sedecimpunctata_582 | 0.177 |
| Calvia_decemguttata_194 | Tytthaspis_sedecimpunctata_582 | 0.177 |
| Calvia_decemguttata_193 | Tytthaspis_sedecimpunctata_585 | 0.177 |
| Calvia_decemguttata_194 | Tytthaspis_sedecimpunctata_585 | 0.177 |
| Calvia_decemguttata_193 | Tytthaspis_sedecimpunctata_589 | 0.177 |
| Calvia_decemguttata_194 | Tytthaspis_sedecimpunctata_589 | 0.177 |
| Calvia_decemguttata_193 | Tytthaspis_sedecimpunctata_590 | 0.177 |
| Calvia_decemguttata_194 | Tytthaspis_sedecimpunctata_590 | 0.177 |
| Calvia_decemguttata_193 | Tytthaspis_sedecimpunctata_592 | 0.177 |
| Calvia_decemguttata_194 | Tytthaspis_sedecimpunctata_592 | 0.177 |
| Calvia_decemguttata_193 | Tytthaspis_sedecimpunctata_593 | 0.177 |
| Calvia_decemguttata_194 | Tytthaspis_sedecimpunctata_593 | 0.177 |
| Calvia_decemguttata_193 | Tytthaspis_sedecimpunctata_594 | 0.177 |
| Calvia_decemguttata_194 | Tytthaspis_sedecimpunctata_594 | 0.177 |
| Calvia_decemguttata_193 | Tytthaspis_sedecimpunctata_595 | 0.177 |
| Calvia_decemguttata_194 | Tytthaspis_sedecimpunctata_595 | 0.177 |
| Calvia_decemguttata_195 | Vibidia_duodecimguttata_596 | 0.177 |
| Psyllobora_vigintiduopunctata_560 | Vibidia_duodecimguttata_596 | 0.177 |
| Psyllobora_vigintiduopunctata_564 | Vibidia_duodecimguttata_596 | 0.177 |
| Psyllobora_vigintiduopunctata_570 | Vibidia_duodecimguttata_596 | 0.177 |
| Psyllobora_vigintiduopunctata_572 | Vibidia_duodecimguttata_596 | 0.177 |
| Calvia_decemguttata_195 | Vibidia_duodecimguttata_598 | 0.177 |
| Psyllobora_vigintiduopunctata_557 | Vibidia_duodecimguttata_598 | 0.177 |
| Psyllobora_vigintiduopunctata_563 | Vibidia_duodecimguttata_598 | 0.177 |
| Calvia_decemguttata_187 | Vibidia_duodecimguttata_599 | 0.177 |
| Calvia_decemguttata_188 | Vibidia_duodecimguttata_599 | 0.177 |
| Calvia_decemguttata_189 | Vibidia_duodecimguttata_599 | 0.177 |
| Calvia_decemguttata_190 | Vibidia_duodecimguttata_599 | 0.177 |
| Calvia_decemguttata_191 | Vibidia_duodecimguttata_599 | 0.177 |
| Calvia_decemguttata_192 | Vibidia_duodecimguttata_599 | 0.177 |
| Calvia_decemguttata_196 | Vibidia_duodecimguttata_599 | 0.177 |
| Calvia_decemguttata_197 | Vibidia_duodecimguttata_599 | 0.177 |
| Calvia_decemguttata_198 | Vibidia_duodecimguttata_599 | 0.177 |
| Calvia_decemguttata_199 | Vibidia_duodecimguttata_599 | 0.177 |
| Calvia_decemguttata_200 | Vibidia_duodecimguttata_599 | 0.177 |
| Calvia_decemguttata_187 | Vibidia_duodecimguttata_601 | 0.177 |
| Calvia_decemguttata_188 | Vibidia_duodecimguttata_601 | 0.177 |
| Calvia_decemguttata_189 | Vibidia_duodecimguttata_601 | 0.177 |
| Calvia_decemguttata_190 | Vibidia_duodecimguttata_601 | 0.177 |
| Calvia_decemguttata_191 | Vibidia_duodecimguttata_601 | 0.177 |
| Calvia_decemguttata_192 | Vibidia_duodecimguttata_601 | 0.177 |

Supplementary Table S4 (continued)

| | | |
|---|---|---|
| Calvia_decemguttata_196 | Vibidia_duodecimguttata_601 | 0.177 |
| Calvia_decemguttata_197 | Vibidia_duodecimguttata_601 | 0.177 |
| Calvia_decemguttata_198 | Vibidia_duodecimguttata_601 | 0.177 |
| Calvia_decemguttata_199 | Vibidia_duodecimguttata_601 | 0.177 |
| Calvia_decemguttata_200 | Vibidia_duodecimguttata_601 | 0.177 |
| Calvia_decemguttata_187 | Vibidia_duodecimguttata_602 | 0.177 |
| Calvia_decemguttata_188 | Vibidia_duodecimguttata_602 | 0.177 |
| Calvia_decemguttata_189 | Vibidia_duodecimguttata_602 | 0.177 |
| Calvia_decemguttata_190 | Vibidia_duodecimguttata_602 | 0.177 |
| Calvia_decemguttata_191 | Vibidia_duodecimguttata_602 | 0.177 |
| Calvia_decemguttata_192 | Vibidia_duodecimguttata_602 | 0.177 |
| Calvia_decemguttata_196 | Vibidia_duodecimguttata_602 | 0.177 |
| Calvia_decemguttata_197 | Vibidia_duodecimguttata_602 | 0.177 |
| Calvia_decemguttata_198 | Vibidia_duodecimguttata_602 | 0.177 |
| Calvia_decemguttata_199 | Vibidia_duodecimguttata_602 | 0.177 |
| Calvia_decemguttata_200 | Vibidia_duodecimguttata_602 | 0.177 |
| Calvia_decemguttata_193 | Vibidia_duodecimguttata_603 | 0.177 |
| Calvia_decemguttata_194 | Vibidia_duodecimguttata_603 | 0.177 |
| Psyllobora_vigintiduopunctata_557 | Vibidia_duodecimguttata_603 | 0.177 |
| Psyllobora_vigintiduopunctata_563 | Vibidia_duodecimguttata_603 | 0.177 |
| Scymnus_camptodromus_706 | Tytthaspis_sedecimpunctata_577 | 0.177 |
| Scymnus_camptodromus_706 | Tytthaspis_sedecimpunctata_578 | 0.177 |
| Scymnus_camptodromus_706 | Tytthaspis_sedecimpunctata_579 | 0.177 |
| Scymnus_camptodromus_706 | Tytthaspis_sedecimpunctata_581 | 0.177 |
| Scymnus_camptodromus_706 | Tytthaspis_sedecimpunctata_582 | 0.177 |
| Coccinella_undecimpunctata_287 | Tytthaspis_sedecimpunctata_584 | 0.177 |
| Scymnus_camptodromus_706 | Tytthaspis_sedecimpunctata_585 | 0.177 |
| Coccinella_undecimpunctata_287 | Tytthaspis_sedecimpunctata_586 | 0.177 |
| Scymnus_camptodromus_706 | Tytthaspis_sedecimpunctata_590 | 0.177 |
| Scymnus_camptodromus_706 | Tytthaspis_sedecimpunctata_592 | 0.177 |
| Scymnus_camptodromus_706 | Tytthaspis_sedecimpunctata_593 | 0.177 |
| Scymnus_camptodromus_706 | Tytthaspis_sedecimpunctata_594 | 0.177 |
| Scymnus_camptodromus_706 | Tytthaspis_sedecimpunctata_595 | 0.177 |
| Coccinella_undecimpunctata_287 | Tytthaspis_sedecimpunctata_583 | 0.177 |
| Scymnus_camptodromus_706 | Tytthaspis_sedecimpunctata_587 | 0.177 |
| Hyperaspis_reppensis_665 | Scymnus_sinuanodulus_831 | 0.177 |
| Calvia_decemguttata_193 | Tytthaspis_sedecimpunctata_586 | 0.177 |
| Calvia_decemguttata_194 | Tytthaspis_sedecimpunctata_586 | 0.177 |
| Calvia_decemguttata_187 | Vibidia_duodecimguttata_597 | 0.177 |
| Calvia_decemguttata_188 | Vibidia_duodecimguttata_597 | 0.177 |
| Calvia_decemguttata_189 | Vibidia_duodecimguttata_597 | 0.177 |
| Calvia_decemguttata_190 | Vibidia_duodecimguttata_597 | 0.177 |
| Calvia_decemguttata_191 | Vibidia_duodecimguttata_597 | 0.177 |
| Calvia_decemguttata_192 | Vibidia_duodecimguttata_597 | 0.177 |
| Calvia_decemguttata_195 | Vibidia_duodecimguttata_597 | 0.177 |
| Calvia_decemguttata_196 | Vibidia_duodecimguttata_597 | 0.177 |
| Calvia_decemguttata_197 | Vibidia_duodecimguttata_597 | 0.177 |
| Calvia_decemguttata_198 | Vibidia_duodecimguttata_597 | 0.177 |
| Calvia_decemguttata_187 | Vibidia_duodecimguttata_600 | 0.177 |
| Calvia_decemguttata_188 | Vibidia_duodecimguttata_600 | 0.177 |
| Calvia_decemguttata_189 | Vibidia_duodecimguttata_600 | 0.177 |
| Calvia_decemguttata_190 | Vibidia_duodecimguttata_600 | 0.177 |
| Calvia_decemguttata_191 | Vibidia_duodecimguttata_600 | 0.177 |
| Calvia_decemguttata_192 | Vibidia_duodecimguttata_600 | 0.177 |
| Calvia_decemguttata_195 | Vibidia_duodecimguttata_600 | 0.177 |
| Calvia_decemguttata_196 | Vibidia_duodecimguttata_600 | 0.177 |

| | | |
|---|---|---|
| Calvia_decemguttata_197 | Vibidia_duodecimguttata_600 | 0.177 |
| Calvia_decemguttata_198 | Vibidia_duodecimguttata_600 | 0.177 |
| Coccidula_scutellata_84 | Scymnus_sinuanodulus_829 | 0.177 |
| Coccinella_undecimpunctata_287 | Tytthaspis_sedecimpunctata_580 | 0.177 |
| Scymnus_pallipediformis_812 | Scymnus_sinuanodulus_829 | 0.177 |
| Platynaspis_luteorubra_50 | Tytthaspis_sedecimpunctata_589 | 0.177 |
| Platynaspis_luteorubra_50 | Tytthaspis_sedecimpunctata_587 | 0.177 |
| Scymnus_haemorrhoidalis_752 | Scymnus_sinuanodulus_831 | 0.177 |
| Psyllobora_vigintiduopunctata_569 | Vibidia_duodecimguttata_599 | 0.177 |
| Psyllobora_vigintiduopunctata_569 | Vibidia_duodecimguttata_601 | 0.177 |
| Psyllobora_vigintiduopunctata_569 | Vibidia_duodecimguttata_602 | 0.177 |
| Scymnus_rubromaculatus_814 | Scymnus_sinuanodulus_829 | 0.177 |
| Scymnus_auritus_703 | Scymnus_sinuanodulus_831 | 0.177 |
| Hyperaspis_reppensis_665 | Scymnus_sinuanodulus_829 | 0.177 |
| Psyllobora_vigintiduopunctata_558 | Vibidia_duodecimguttata_596 | 0.177 |
| Psyllobora_vigintiduopunctata_568 | Vibidia_duodecimguttata_596 | 0.177 |
| Scymnus_camptodromus_715 | Tytthaspis_sedecimpunctata_577 | 0.177 |
| Scymnus_camptodromus_715 | Tytthaspis_sedecimpunctata_578 | 0.177 |
| Scymnus_camptodromus_715 | Tytthaspis_sedecimpunctata_579 | 0.177 |
| Scymnus_camptodromus_715 | Tytthaspis_sedecimpunctata_581 | 0.177 |
| Scymnus_camptodromus_715 | Tytthaspis_sedecimpunctata_582 | 0.177 |
| Scymnus_camptodromus_715 | Tytthaspis_sedecimpunctata_585 | 0.177 |
| Scymnus_camptodromus_715 | Tytthaspis_sedecimpunctata_590 | 0.177 |
| Scymnus_camptodromus_715 | Tytthaspis_sedecimpunctata_592 | 0.177 |
| Scymnus_camptodromus_715 | Tytthaspis_sedecimpunctata_593 | 0.177 |
| Scymnus_camptodromus_715 | Tytthaspis_sedecimpunctata_594 | 0.177 |
| Scymnus_camptodromus_715 | Tytthaspis_sedecimpunctata_595 | 0.177 |
| Calvia_quatuordecimguttata_203 | Vibidia_duodecimguttata_603 | 0.177 |
| Scymnus_ferrugatus_723 | Scymnus_sinuanodulus_830 | 0.177 |
| Scymnus_ferrugatus_725 | Scymnus_sinuanodulus_830 | 0.177 |
| Scymnus_ferrugatus_726 | Scymnus_sinuanodulus_830 | 0.177 |
| Scymnus_ferrugatus_727 | Scymnus_sinuanodulus_830 | 0.177 |
| Scymnus_ferrugatus_728 | Scymnus_sinuanodulus_830 | 0.177 |
| Scymnus_ferrugatus_730 | Scymnus_sinuanodulus_830 | 0.177 |
| Scymnus_ferrugatus_731 | Scymnus_sinuanodulus_830 | 0.177 |
| Scymnus_ferrugatus_732 | Scymnus_sinuanodulus_830 | 0.177 |
| Scymnus_ferrugatus_733 | Scymnus_sinuanodulus_830 | 0.177 |
| Platynaspis_luteorubra_51 | Tytthaspis_sedecimpunctata_580 | 0.177 |
| Platynaspis_luteorubra_53 | Tytthaspis_sedecimpunctata_580 | 0.177 |
| Platynaspis_luteorubra_58 | Tytthaspis_sedecimpunctata_580 | 0.177 |
| Platynaspis_luteorubra_51 | Tytthaspis_sedecimpunctata_585 | 0.177 |
| Platynaspis_luteorubra_53 | Tytthaspis_sedecimpunctata_585 | 0.177 |
| Platynaspis_luteorubra_58 | Tytthaspis_sedecimpunctata_585 | 0.177 |
| Platynaspis_luteorubra_51 | Tytthaspis_sedecimpunctata_589 | 0.177 |
| Platynaspis_luteorubra_53 | Tytthaspis_sedecimpunctata_589 | 0.177 |
| Platynaspis_luteorubra_58 | Tytthaspis_sedecimpunctata_589 | 0.177 |
| Platynaspis_luteorubra_51 | Tytthaspis_sedecimpunctata_584 | 0.178 |
| Platynaspis_luteorubra_53 | Tytthaspis_sedecimpunctata_584 | 0.178 |
| Platynaspis_luteorubra_58 | Tytthaspis_sedecimpunctata_584 | 0.178 |
| Platynaspis_luteorubra_51 | Tytthaspis_sedecimpunctata_588 | 0.178 |
| Platynaspis_luteorubra_53 | Tytthaspis_sedecimpunctata_588 | 0.178 |
| Platynaspis_luteorubra_58 | Tytthaspis_sedecimpunctata_588 | 0.178 |
| Hyperaspis_reppensis_667 | Tytthaspis_sedecimpunctata_585 | 0.178 |
| Chilocorus_stigma_44 | Tytthaspis_sedecimpunctata_589 | 0.178 |
| Chilocorus_stigma_46 | Tytthaspis_sedecimpunctata_589 | 0.178 |
| Chilocorus_stigma_47 | Tytthaspis_sedecimpunctata_589 | 0.178 |

| | | |
|---|---|---|
| Coccidula_scutellata_77 | Scymnus_suturalis_833 | 0.178 |
| Coccidula_scutellata_77 | Scymnus_suturalis_843 | 0.178 |
| Coccidula_scutellata_77 | Scymnus_suturalis_845 | 0.178 |
| Chilocorus_stigma_44 | Tytthaspis_sedecimpunctata_587 | 0.178 |
| Chilocorus_stigma_46 | Tytthaspis_sedecimpunctata_587 | 0.178 |
| Chilocorus_stigma_47 | Tytthaspis_sedecimpunctata_587 | 0.178 |
| Brumus_quadripustulatus_15 | Tytthaspis_sedecimpunctata_589 | 0.178 |
| Brumus_quadripustulatus_16 | Tytthaspis_sedecimpunctata_589 | 0.178 |
| Brumus_quadripustulatus_6 | Tytthaspis_sedecimpunctata_589 | 0.178 |
| Brumus_quadripustulatus_8 | Tytthaspis_sedecimpunctata_589 | 0.178 |
| Myzia_oblongoguttata_504 | Tytthaspis_sedecimpunctata_593 | 0.178 |
| Myzia_oblongoguttata_505 | Tytthaspis_sedecimpunctata_593 | 0.178 |
| Myzia_oblongoguttata_507 | Tytthaspis_sedecimpunctata_593 | 0.178 |
| Myzia_oblongoguttata_508 | Tytthaspis_sedecimpunctata_593 | 0.178 |
| Myzia_oblongoguttata_509 | Tytthaspis_sedecimpunctata_593 | 0.178 |
| Myzia_oblongoguttata_510 | Tytthaspis_sedecimpunctata_593 | 0.178 |
| Myzia_oblongoguttata_511 | Tytthaspis_sedecimpunctata_593 | 0.178 |
| Myzia_oblongoguttata_512 | Tytthaspis_sedecimpunctata_593 | 0.178 |
| Myzia_oblongoguttata_513 | Tytthaspis_sedecimpunctata_593 | 0.178 |
| Myzia_oblongoguttata_514 | Tytthaspis_sedecimpunctata_593 | 0.178 |
| Myzia_oblongoguttata_516 | Tytthaspis_sedecimpunctata_593 | 0.178 |
| Coccidula_scutellata_80 | Scymnus_suturalis_833 | 0.178 |
| Coccidula_scutellata_81 | Scymnus_suturalis_841 | 0.178 |
| Coccidula_scutellata_82 | Scymnus_suturalis_841 | 0.178 |
| Coccidula_scutellata_81 | Scymnus_suturalis_842 | 0.178 |
| Coccidula_scutellata_82 | Scymnus_suturalis_842 | 0.178 |
| Coccidula_scutellata_80 | Scymnus_suturalis_843 | 0.178 |
| Coccidula_scutellata_81 | Scymnus_suturalis_844 | 0.178 |
| Coccidula_scutellata_82 | Scymnus_suturalis_844 | 0.178 |
| Coccidula_scutellata_80 | Scymnus_suturalis_845 | 0.178 |
| Coccidula_scutellata_81 | Scymnus_suturalis_846 | 0.178 |
| Coccidula_scutellata_82 | Scymnus_suturalis_846 | 0.178 |
| Coccidula_scutellata_81 | Scymnus_suturalis_847 | 0.178 |
| Coccidula_scutellata_82 | Scymnus_suturalis_847 | 0.178 |
| Coccidula_scutellata_81 | Scymnus_suturalis_848 | 0.178 |
| Coccidula_scutellata_82 | Scymnus_suturalis_848 | 0.178 |
| Brumus_quadripustulatus_15 | Tytthaspis_sedecimpunctata_588 | 0.178 |
| Brumus_quadripustulatus_16 | Tytthaspis_sedecimpunctata_588 | 0.178 |
| Brumus_quadripustulatus_6 | Tytthaspis_sedecimpunctata_588 | 0.178 |
| Brumus_quadripustulatus_8 | Tytthaspis_sedecimpunctata_588 | 0.178 |
| Scymnus_loewii_771 | Tytthaspis_sedecimpunctata_588 | 0.178 |
| Brumus_quadripustulatus_15 | Tytthaspis_sedecimpunctata_591 | 0.178 |
| Brumus_quadripustulatus_16 | Tytthaspis_sedecimpunctata_591 | 0.178 |
| Brumus_quadripustulatus_6 | Tytthaspis_sedecimpunctata_591 | 0.178 |
| Brumus_quadripustulatus_8 | Tytthaspis_sedecimpunctata_591 | 0.178 |
| Coccidula_scutellata_81 | Scymnus_suturalis_832 | 0.178 |
| Coccidula_scutellata_82 | Scymnus_suturalis_832 | 0.178 |
| Chilocorus_nigritus_26 | Scymnus_suturalis_833 | 0.178 |
| Coccidula_scutellata_81 | Scymnus_suturalis_835 | 0.178 |
| Coccidula_scutellata_82 | Scymnus_suturalis_835 | 0.178 |
| Coccidula_scutellata_81 | Scymnus_suturalis_836 | 0.178 |
| Coccidula_scutellata_82 | Scymnus_suturalis_836 | 0.178 |
| Coccidula_scutellata_78 | Scymnus_suturalis_837 | 0.178 |
| Coccidula_scutellata_79 | Scymnus_suturalis_837 | 0.178 |
| Coccidula_scutellata_81 | Scymnus_suturalis_839 | 0.178 |
| Coccidula_scutellata_82 | Scymnus_suturalis_839 | 0.178 |

Supplementary Table S4 (continued)

| | | |
|---|---|---|
| Calvia_quatuordecimguttata_205 | Vibidia_duodecimguttata_598 | 0.178 |
| Calvia_quatuordecimguttata_205 | Vibidia_duodecimguttata_599 | 0.178 |
| Calvia_quatuordecimguttata_205 | Vibidia_duodecimguttata_601 | 0.178 |
| Calvia_quatuordecimguttata_205 | Vibidia_duodecimguttata_602 | 0.178 |
| Scymnus_ferrugatus_723 | Scymnus_rubromaculatus_822 | 0.178 |
| Scymnus_ferrugatus_725 | Scymnus_rubromaculatus_822 | 0.178 |
| Scymnus_ferrugatus_726 | Scymnus_rubromaculatus_822 | 0.178 |
| Scymnus_ferrugatus_727 | Scymnus_rubromaculatus_822 | 0.178 |
| Scymnus_ferrugatus_728 | Scymnus_rubromaculatus_822 | 0.178 |
| Scymnus_ferrugatus_730 | Scymnus_rubromaculatus_822 | 0.178 |
| Scymnus_ferrugatus_732 | Scymnus_rubromaculatus_822 | 0.178 |
| Scymnus_ferrugatus_733 | Scymnus_rubromaculatus_822 | 0.178 |
| Scymnus_ferrugatus_723 | Scymnus_rubromaculatus_823 | 0.178 |
| Scymnus_ferrugatus_725 | Scymnus_rubromaculatus_823 | 0.178 |
| Scymnus_ferrugatus_726 | Scymnus_rubromaculatus_823 | 0.178 |
| Scymnus_ferrugatus_727 | Scymnus_rubromaculatus_823 | 0.178 |
| Scymnus_ferrugatus_728 | Scymnus_rubromaculatus_823 | 0.178 |
| Scymnus_ferrugatus_730 | Scymnus_rubromaculatus_823 | 0.178 |
| Scymnus_ferrugatus_732 | Scymnus_rubromaculatus_823 | 0.178 |
| Scymnus_ferrugatus_733 | Scymnus_rubromaculatus_823 | 0.178 |
| Scymnus_ferrugatus_723 | Scymnus_rubromaculatus_824 | 0.178 |
| Scymnus_ferrugatus_725 | Scymnus_rubromaculatus_824 | 0.178 |
| Scymnus_ferrugatus_726 | Scymnus_rubromaculatus_824 | 0.178 |
| Scymnus_ferrugatus_727 | Scymnus_rubromaculatus_824 | 0.178 |
| Scymnus_ferrugatus_728 | Scymnus_rubromaculatus_824 | 0.178 |
| Scymnus_ferrugatus_730 | Scymnus_rubromaculatus_824 | 0.178 |
| Scymnus_ferrugatus_732 | Scymnus_rubromaculatus_824 | 0.178 |
| Scymnus_ferrugatus_733 | Scymnus_rubromaculatus_824 | 0.178 |
| Scymnus_ferrugatus_723 | Scymnus_rubromaculatus_825 | 0.178 |
| Scymnus_ferrugatus_725 | Scymnus_rubromaculatus_825 | 0.178 |
| Scymnus_ferrugatus_726 | Scymnus_rubromaculatus_825 | 0.178 |
| Scymnus_ferrugatus_727 | Scymnus_rubromaculatus_825 | 0.178 |
| Scymnus_ferrugatus_728 | Scymnus_rubromaculatus_825 | 0.178 |
| Scymnus_ferrugatus_730 | Scymnus_rubromaculatus_825 | 0.178 |
| Scymnus_ferrugatus_732 | Scymnus_rubromaculatus_825 | 0.178 |
| Scymnus_ferrugatus_733 | Scymnus_rubromaculatus_825 | 0.178 |
| Scymnus_ferrugatus_723 | Scymnus_rubromaculatus_826 | 0.178 |
| Scymnus_ferrugatus_725 | Scymnus_rubromaculatus_826 | 0.178 |
| Scymnus_ferrugatus_726 | Scymnus_rubromaculatus_826 | 0.178 |
| Scymnus_ferrugatus_727 | Scymnus_rubromaculatus_826 | 0.178 |
| Scymnus_ferrugatus_728 | Scymnus_rubromaculatus_826 | 0.178 |
| Scymnus_ferrugatus_730 | Scymnus_rubromaculatus_826 | 0.178 |
| Scymnus_ferrugatus_732 | Scymnus_rubromaculatus_826 | 0.178 |
| Scymnus_ferrugatus_733 | Scymnus_rubromaculatus_826 | 0.178 |
| Scymnus_ferrugatus_723 | Scymnus_rubromaculatus_827 | 0.178 |
| Scymnus_ferrugatus_725 | Scymnus_rubromaculatus_827 | 0.178 |
| Scymnus_ferrugatus_726 | Scymnus_rubromaculatus_827 | 0.178 |
| Scymnus_ferrugatus_727 | Scymnus_rubromaculatus_827 | 0.178 |
| Scymnus_ferrugatus_728 | Scymnus_rubromaculatus_827 | 0.178 |
| Scymnus_ferrugatus_730 | Scymnus_rubromaculatus_827 | 0.178 |
| Scymnus_ferrugatus_732 | Scymnus_rubromaculatus_827 | 0.178 |
| Scymnus_ferrugatus_733 | Scymnus_rubromaculatus_827 | 0.178 |
| Myzia_pullata_517 | Tytthaspis_sedecimpunctata_577 | 0.178 |
| Myzia_pullata_517 | Tytthaspis_sedecimpunctata_578 | 0.178 |
| Myzia_pullata_517 | Tytthaspis_sedecimpunctata_579 | 0.178 |
| Myzia_pullata_517 | Tytthaspis_sedecimpunctata_581 | 0.178 |

| | | |
|---|---|---|
| Myzia_pullata_517 | Tytthaspis_sedecimpunctata_582 | 0.178 |
| Brumus_quadripustulatus_11 | Tytthaspis_sedecimpunctata_587 | 0.178 |
| Brumus_quadripustulatus_13 | Tytthaspis_sedecimpunctata_587 | 0.178 |
| Brumus_quadripustulatus_14 | Tytthaspis_sedecimpunctata_587 | 0.178 |
| Brumus_quadripustulatus_17 | Tytthaspis_sedecimpunctata_587 | 0.178 |
| Brumus_quadripustulatus_3 | Tytthaspis_sedecimpunctata_587 | 0.178 |
| Brumus_quadripustulatus_4 | Tytthaspis_sedecimpunctata_587 | 0.178 |
| Brumus_quadripustulatus_7 | Tytthaspis_sedecimpunctata_587 | 0.178 |
| Aphidecta_obliterata_175 | Tytthaspis_sedecimpunctata_589 | 0.178 |
| Aphidecta_obliterata_177 | Tytthaspis_sedecimpunctata_589 | 0.178 |
| Aphidecta_obliterata_180 | Tytthaspis_sedecimpunctata_589 | 0.178 |
| Aphidecta_obliterata_182 | Tytthaspis_sedecimpunctata_589 | 0.178 |
| Myzia_pullata_517 | Tytthaspis_sedecimpunctata_590 | 0.178 |
| Myzia_pullata_517 | Tytthaspis_sedecimpunctata_592 | 0.178 |
| Myzia_pullata_517 | Tytthaspis_sedecimpunctata_593 | 0.178 |
| Myzia_pullata_517 | Tytthaspis_sedecimpunctata_594 | 0.178 |
| Myzia_pullata_517 | Tytthaspis_sedecimpunctata_595 | 0.178 |
| Scymnus_haemorrhoidalis_752 | Scymnus_sinuanodulus_829 | 0.178 |
| Myzia_pullata_518 | Tytthaspis_sedecimpunctata_580 | 0.178 |
| Aphidecta_obliterata_175 | Tytthaspis_sedecimpunctata_586 | 0.178 |
| Aphidecta_obliterata_177 | Tytthaspis_sedecimpunctata_586 | 0.178 |
| Aphidecta_obliterata_180 | Tytthaspis_sedecimpunctata_586 | 0.178 |
| Aphidecta_obliterata_182 | Tytthaspis_sedecimpunctata_586 | 0.178 |
| Myzia_pullata_518 | Tytthaspis_sedecimpunctata_586 | 0.178 |
| Propylaea_quatuordecimpunctata_545 | Tytthaspis_sedecimpunctata_586 | 0.178 |
| Aphidecta_obliterata_179 | Tytthaspis_sedecimpunctata_587 | 0.178 |
| Propylaea_quatuordecimpunctata_532 | Tytthaspis_sedecimpunctata_587 | 0.178 |
| Propylaea_quatuordecimpunctata_533 | Tytthaspis_sedecimpunctata_587 | 0.178 |
| Propylaea_quatuordecimpunctata_534 | Tytthaspis_sedecimpunctata_587 | 0.178 |
| Propylaea_quatuordecimpunctata_535 | Tytthaspis_sedecimpunctata_587 | 0.178 |
| Propylaea_quatuordecimpunctata_536 | Tytthaspis_sedecimpunctata_587 | 0.178 |
| Propylaea_quatuordecimpunctata_537 | Tytthaspis_sedecimpunctata_587 | 0.178 |
| Propylaea_quatuordecimpunctata_538 | Tytthaspis_sedecimpunctata_587 | 0.178 |
| Propylaea_quatuordecimpunctata_541 | Tytthaspis_sedecimpunctata_587 | 0.178 |
| Propylaea_quatuordecimpunctata_544 | Tytthaspis_sedecimpunctata_587 | 0.178 |
| Propylaea_quatuordecimpunctata_549 | Tytthaspis_sedecimpunctata_587 | 0.178 |
| Anisosticta_novemdecimpunctata_174 | Tytthaspis_sedecimpunctata_588 | 0.178 |
| Aphidecta_obliterata_184 | Tytthaspis_sedecimpunctata_588 | 0.178 |
| Myzia_pullata_518 | Tytthaspis_sedecimpunctata_588 | 0.178 |
| Propylaea_quatuordecimpunctata_545 | Tytthaspis_sedecimpunctata_588 | 0.178 |
| Coccinella_hieroglyphica_225 | Tytthaspis_sedecimpunctata_589 | 0.178 |
| Aphidecta_obliterata_179 | Tytthaspis_sedecimpunctata_591 | 0.178 |
| Propylaea_quatuordecimpunctata_539 | Tytthaspis_sedecimpunctata_591 | 0.178 |
| Propylaea_quatuordecimpunctata_540 | Tytthaspis_sedecimpunctata_591 | 0.178 |
| Propylaea_quatuordecimpunctata_543 | Tytthaspis_sedecimpunctata_591 | 0.178 |
| Propylaea_quatuordecimpunctata_548 | Tytthaspis_sedecimpunctata_591 | 0.178 |
| Coccidula_scutellata_80 | Scymnus_sinuanodulus_828 | 0.178 |
| Anatis_ocellata_133 | Scymnus_suturalis_833 | 0.178 |
| Anatis_ocellata_137 | Scymnus_suturalis_833 | 0.178 |
| Anatis_ocellata_133 | Scymnus_suturalis_841 | 0.178 |
| Anatis_ocellata_137 | Scymnus_suturalis_841 | 0.178 |
| Anatis_ocellata_133 | Scymnus_suturalis_842 | 0.178 |
| Anatis_ocellata_137 | Scymnus_suturalis_842 | 0.178 |
| Anatis_ocellata_133 | Scymnus_suturalis_844 | 0.178 |
| Anatis_ocellata_137 | Scymnus_suturalis_844 | 0.178 |
| Anatis_ocellata_133 | Scymnus_suturalis_846 | 0.178 |

Supplementary Table S4 (continued)

| | | |
|---|---|---|
| Anatis_ocellata_137 | Scymnus_suturalis_846 | 0.178 |
| Anatis_ocellata_133 | Scymnus_suturalis_847 | 0.178 |
| Anatis_ocellata_137 | Scymnus_suturalis_847 | 0.178 |
| Anatis_ocellata_133 | Scymnus_suturalis_848 | 0.178 |
| Anatis_ocellata_137 | Scymnus_suturalis_848 | 0.178 |
| Anisosticta_novemdecimpunctata_174 | Tytthaspis_sedecimpunctata_577 | 0.178 |
| Aphidecta_obliterata_184 | Tytthaspis_sedecimpunctata_577 | 0.178 |
| Anisosticta_novemdecimpunctata_174 | Tytthaspis_sedecimpunctata_578 | 0.178 |
| Aphidecta_obliterata_184 | Tytthaspis_sedecimpunctata_578 | 0.178 |
| Anisosticta_novemdecimpunctata_174 | Tytthaspis_sedecimpunctata_579 | 0.178 |
| Aphidecta_obliterata_184 | Tytthaspis_sedecimpunctata_579 | 0.178 |
| Anisosticta_novemdecimpunctata_174 | Tytthaspis_sedecimpunctata_581 | 0.178 |
| Aphidecta_obliterata_184 | Tytthaspis_sedecimpunctata_581 | 0.178 |
| Anisosticta_novemdecimpunctata_174 | Tytthaspis_sedecimpunctata_582 | 0.178 |
| Aphidecta_obliterata_184 | Tytthaspis_sedecimpunctata_582 | 0.178 |
| Aphidecta_obliterata_175 | Tytthaspis_sedecimpunctata_583 | 0.178 |
| Aphidecta_obliterata_177 | Tytthaspis_sedecimpunctata_583 | 0.178 |
| Aphidecta_obliterata_180 | Tytthaspis_sedecimpunctata_583 | 0.178 |
| Aphidecta_obliterata_182 | Tytthaspis_sedecimpunctata_583 | 0.178 |
| Aphidecta_obliterata_175 | Tytthaspis_sedecimpunctata_585 | 0.178 |
| Aphidecta_obliterata_177 | Tytthaspis_sedecimpunctata_585 | 0.178 |
| Aphidecta_obliterata_180 | Tytthaspis_sedecimpunctata_585 | 0.178 |
| Aphidecta_obliterata_182 | Tytthaspis_sedecimpunctata_585 | 0.178 |
| Coccinella_trifasciata_280 | Tytthaspis_sedecimpunctata_586 | 0.178 |
| Coccinella_hieroglyphica_225 | Tytthaspis_sedecimpunctata_587 | 0.178 |
| Coccinella_transversoguttata_265 | Tytthaspis_sedecimpunctata_587 | 0.178 |
| Coccinella_transversoguttata_266 | Tytthaspis_sedecimpunctata_587 | 0.178 |
| Scymnus_camptodromus_714 | Tytthaspis_sedecimpunctata_587 | 0.178 |
| Coccinella_hieroglyphica_227 | Tytthaspis_sedecimpunctata_588 | 0.178 |
| Coccinella_trifasciata_272 | Tytthaspis_sedecimpunctata_588 | 0.178 |
| Coccinella_trifasciata_276 | Tytthaspis_sedecimpunctata_588 | 0.178 |
| Adalia_bipunctata_98 | Tytthaspis_sedecimpunctata_589 | 0.178 |
| Propylaea_quatuordecimpunctata_540 | Tytthaspis_sedecimpunctata_589 | 0.178 |
| Propylaea_quatuordecimpunctata_543 | Tytthaspis_sedecimpunctata_589 | 0.178 |
| Propylaea_quatuordecimpunctata_547 | Tytthaspis_sedecimpunctata_589 | 0.178 |
| Propylaea_quatuordecimpunctata_548 | Tytthaspis_sedecimpunctata_589 | 0.178 |
| Anisosticta_novemdecimpunctata_174 | Tytthaspis_sedecimpunctata_590 | 0.178 |
| Aphidecta_obliterata_184 | Tytthaspis_sedecimpunctata_590 | 0.178 |
| Adalia_bipunctata_99 | Tytthaspis_sedecimpunctata_591 | 0.178 |
| Aphidecta_obliterata_181 | Tytthaspis_sedecimpunctata_592 | 0.178 |
| Anisosticta_novemdecimpunctata_174 | Tytthaspis_sedecimpunctata_593 | 0.178 |
| Aphidecta_obliterata_184 | Tytthaspis_sedecimpunctata_593 | 0.178 |
| Anisosticta_novemdecimpunctata_174 | Tytthaspis_sedecimpunctata_594 | 0.178 |
| Aphidecta_obliterata_184 | Tytthaspis_sedecimpunctata_594 | 0.178 |
| Anisosticta_novemdecimpunctata_174 | Tytthaspis_sedecimpunctata_595 | 0.178 |
| Aphidecta_obliterata_184 | Tytthaspis_sedecimpunctata_595 | 0.178 |
| Anatis_ocellata_133 | Scymnus_suturalis_832 | 0.178 |
| Anatis_ocellata_137 | Scymnus_suturalis_832 | 0.178 |
| Hyperaspis_oregona_659 | Scymnus_suturalis_833 | 0.178 |
| Platynaspis_luteorubra_52 | Scymnus_suturalis_833 | 0.178 |
| Platynaspis_luteorubra_56 | Scymnus_suturalis_833 | 0.178 |
| Platynaspis_luteorubra_60 | Scymnus_suturalis_833 | 0.178 |
| Anatis_ocellata_133 | Scymnus_suturalis_835 | 0.178 |
| Anatis_ocellata_137 | Scymnus_suturalis_835 | 0.178 |
| Anatis_ocellata_133 | Scymnus_suturalis_836 | 0.178 |
| Anatis_ocellata_137 | Scymnus_suturalis_836 | 0.178 |

| | | |
|---|---|---|
| Anatis_ocellata_133 | Scymnus_suturalis_839 | 0.178 |
| Anatis_ocellata_137 | Scymnus_suturalis_839 | 0.178 |
| Scymnus_ferrugatus_723 | Scymnus_sinuanodulus_831 | 0.178 |
| Scymnus_ferrugatus_725 | Scymnus_sinuanodulus_831 | 0.178 |
| Scymnus_ferrugatus_726 | Scymnus_sinuanodulus_831 | 0.178 |
| Scymnus_ferrugatus_727 | Scymnus_sinuanodulus_831 | 0.178 |
| Scymnus_ferrugatus_728 | Scymnus_sinuanodulus_831 | 0.178 |
| Scymnus_ferrugatus_730 | Scymnus_sinuanodulus_831 | 0.178 |
| Scymnus_ferrugatus_731 | Scymnus_sinuanodulus_831 | 0.178 |
| Scymnus_ferrugatus_732 | Scymnus_sinuanodulus_831 | 0.178 |
| Scymnus_ferrugatus_733 | Scymnus_sinuanodulus_831 | 0.178 |
| Hyperaspis_lateralis_656 | Scymnus_suturalis_841 | 0.178 |
| Platynaspis_luteorubra_52 | Scymnus_suturalis_841 | 0.178 |
| Platynaspis_luteorubra_56 | Scymnus_suturalis_841 | 0.178 |
| Platynaspis_luteorubra_60 | Scymnus_suturalis_841 | 0.178 |
| Hyperaspis_lateralis_656 | Scymnus_suturalis_842 | 0.178 |
| Platynaspis_luteorubra_52 | Scymnus_suturalis_842 | 0.178 |
| Platynaspis_luteorubra_56 | Scymnus_suturalis_842 | 0.178 |
| Platynaspis_luteorubra_60 | Scymnus_suturalis_842 | 0.178 |
| Hyperaspis_lateralis_654 | Scymnus_suturalis_843 | 0.178 |
| Hyperaspis_lateralis_657 | Scymnus_suturalis_843 | 0.178 |
| Hyperaspis_lateralis_656 | Scymnus_suturalis_844 | 0.178 |
| Platynaspis_luteorubra_52 | Scymnus_suturalis_844 | 0.178 |
| Platynaspis_luteorubra_56 | Scymnus_suturalis_844 | 0.178 |
| Platynaspis_luteorubra_60 | Scymnus_suturalis_844 | 0.178 |
| Hyperaspis_lateralis_654 | Scymnus_suturalis_845 | 0.178 |
| Hyperaspis_lateralis_657 | Scymnus_suturalis_845 | 0.178 |
| Hyperaspis_lateralis_656 | Scymnus_suturalis_846 | 0.178 |
| Platynaspis_luteorubra_52 | Scymnus_suturalis_846 | 0.178 |
| Platynaspis_luteorubra_56 | Scymnus_suturalis_846 | 0.178 |
| Platynaspis_luteorubra_60 | Scymnus_suturalis_846 | 0.178 |
| Hyperaspis_lateralis_656 | Scymnus_suturalis_847 | 0.178 |
| Platynaspis_luteorubra_52 | Scymnus_suturalis_847 | 0.178 |
| Platynaspis_luteorubra_56 | Scymnus_suturalis_847 | 0.178 |
| Platynaspis_luteorubra_60 | Scymnus_suturalis_847 | 0.178 |
| Hyperaspis_lateralis_656 | Scymnus_suturalis_848 | 0.178 |
| Platynaspis_luteorubra_52 | Scymnus_suturalis_848 | 0.178 |
| Platynaspis_luteorubra_56 | Scymnus_suturalis_848 | 0.178 |
| Platynaspis_luteorubra_60 | Scymnus_suturalis_848 | 0.178 |
| Aphidecta_obliterata_181 | Tytthaspis_sedecimpunctata_580 | 0.178 |
| Coccinella_hieroglyphica_227 | Tytthaspis_sedecimpunctata_583 | 0.178 |
| Aphidecta_obliterata_179 | Tytthaspis_sedecimpunctata_584 | 0.178 |
| Anatis_labiculata_123 | Tytthaspis_sedecimpunctata_585 | 0.178 |
| Anatis_labiculata_124 | Tytthaspis_sedecimpunctata_585 | 0.178 |
| Anatis_labiculata_125 | Tytthaspis_sedecimpunctata_585 | 0.178 |
| Anatis_labiculata_127 | Tytthaspis_sedecimpunctata_585 | 0.178 |
| Adalia_bipunctata_98 | Tytthaspis_sedecimpunctata_587 | 0.178 |
| Coccinella_undecimpunctata_282 | Tytthaspis_sedecimpunctata_588 | 0.178 |
| Coccinella_undecimpunctata_283 | Tytthaspis_sedecimpunctata_588 | 0.178 |
| Coccinella_undecimpunctata_284 | Tytthaspis_sedecimpunctata_588 | 0.178 |
| Adalia_bipunctata_102 | Tytthaspis_sedecimpunctata_591 | 0.178 |
| Adalia_bipunctata_103 | Tytthaspis_sedecimpunctata_591 | 0.178 |
| Adalia_bipunctata_106 | Tytthaspis_sedecimpunctata_591 | 0.178 |
| Adalia_bipunctata_107 | Tytthaspis_sedecimpunctata_591 | 0.178 |
| Adalia_bipunctata_108 | Tytthaspis_sedecimpunctata_591 | 0.178 |
| Adalia_bipunctata_109 | Tytthaspis_sedecimpunctata_591 | 0.178 |

| | | |
|---|---|---|
| Didion_punctatum_646 | Scymnus_sinuanodulus_830 | 0.178 |
| Hyperaspis_lateralis_656 | Scymnus_suturalis_832 | 0.178 |
| Platynaspis_luteorubra_52 | Scymnus_suturalis_832 | 0.178 |
| Platynaspis_luteorubra_56 | Scymnus_suturalis_832 | 0.178 |
| Platynaspis_luteorubra_60 | Scymnus_suturalis_832 | 0.178 |
| Hyperaspis_campestris_651 | Scymnus_suturalis_833 | 0.178 |
| Hyperaspis_campestris_652 | Scymnus_suturalis_833 | 0.178 |
| Hyperaspis_campestris_653 | Scymnus_suturalis_833 | 0.178 |
| Platynaspis_luteorubra_54 | Scymnus_suturalis_833 | 0.178 |
| Platynaspis_luteorubra_57 | Scymnus_suturalis_833 | 0.178 |
| Platynaspis_luteorubra_59 | Scymnus_suturalis_833 | 0.178 |
| Hyperaspis_lateralis_656 | Scymnus_suturalis_835 | 0.178 |
| Platynaspis_luteorubra_52 | Scymnus_suturalis_835 | 0.178 |
| Platynaspis_luteorubra_56 | Scymnus_suturalis_835 | 0.178 |
| Platynaspis_luteorubra_60 | Scymnus_suturalis_835 | 0.178 |
| Hyperaspis_lateralis_656 | Scymnus_suturalis_836 | 0.178 |
| Platynaspis_luteorubra_52 | Scymnus_suturalis_836 | 0.178 |
| Platynaspis_luteorubra_56 | Scymnus_suturalis_836 | 0.178 |
| Platynaspis_luteorubra_60 | Scymnus_suturalis_836 | 0.178 |
| Hyperaspis_lateralis_656 | Scymnus_suturalis_837 | 0.178 |
| Platynaspis_luteorubra_55 | Scymnus_suturalis_838 | 0.178 |
| Platynaspis_luteorubra_61 | Scymnus_suturalis_838 | 0.178 |
| Hyperaspis_lateralis_656 | Scymnus_suturalis_839 | 0.178 |
| Platynaspis_luteorubra_52 | Scymnus_suturalis_839 | 0.178 |
| Platynaspis_luteorubra_56 | Scymnus_suturalis_839 | 0.178 |
| Platynaspis_luteorubra_60 | Scymnus_suturalis_839 | 0.178 |
| Platynaspis_luteorubra_55 | Scymnus_suturalis_840 | 0.178 |
| Platynaspis_luteorubra_61 | Scymnus_suturalis_840 | 0.178 |
| Platynaspis_luteorubra_54 | Scymnus_suturalis_841 | 0.178 |
| Platynaspis_luteorubra_57 | Scymnus_suturalis_841 | 0.178 |
| Platynaspis_luteorubra_59 | Scymnus_suturalis_841 | 0.178 |
| Platynaspis_luteorubra_54 | Scymnus_suturalis_842 | 0.178 |
| Platynaspis_luteorubra_57 | Scymnus_suturalis_842 | 0.178 |
| Platynaspis_luteorubra_59 | Scymnus_suturalis_842 | 0.178 |
| Hyperaspis_lateralis_655 | Scymnus_suturalis_843 | 0.178 |
| Platynaspis_luteorubra_54 | Scymnus_suturalis_844 | 0.178 |
| Platynaspis_luteorubra_57 | Scymnus_suturalis_844 | 0.178 |
| Platynaspis_luteorubra_59 | Scymnus_suturalis_844 | 0.178 |
| Hyperaspis_lateralis_655 | Scymnus_suturalis_845 | 0.178 |
| Platynaspis_luteorubra_54 | Scymnus_suturalis_846 | 0.178 |
| Platynaspis_luteorubra_57 | Scymnus_suturalis_846 | 0.178 |
| Platynaspis_luteorubra_59 | Scymnus_suturalis_846 | 0.178 |
| Platynaspis_luteorubra_54 | Scymnus_suturalis_847 | 0.178 |
| Platynaspis_luteorubra_57 | Scymnus_suturalis_847 | 0.178 |
| Platynaspis_luteorubra_59 | Scymnus_suturalis_847 | 0.178 |
| Platynaspis_luteorubra_54 | Scymnus_suturalis_848 | 0.178 |
| Platynaspis_luteorubra_57 | Scymnus_suturalis_848 | 0.178 |
| Platynaspis_luteorubra_59 | Scymnus_suturalis_848 | 0.178 |
| Adalia_bipunctata_98 | Tytthaspis_sedecimpunctata_583 | 0.178 |
| Anatis_labiculata_123 | Tytthaspis_sedecimpunctata_586 | 0.178 |
| Anatis_labiculata_124 | Tytthaspis_sedecimpunctata_586 | 0.178 |
| Anatis_labiculata_125 | Tytthaspis_sedecimpunctata_586 | 0.178 |
| Anatis_labiculata_127 | Tytthaspis_sedecimpunctata_586 | 0.178 |
| Scymnus_auritus_701 | Tytthaspis_sedecimpunctata_586 | 0.178 |
| Anatis_labiculata_126 | Tytthaspis_sedecimpunctata_587 | 0.178 |
| Scymnus_auritus_701 | Tytthaspis_sedecimpunctata_588 | 0.178 |

Supplementary Table S4 (continued)

| | | |
|---|---|---|
| Coccinella_hieroglyphica_226 | Tytthaspis_sedecimpunctata_589 | 0.178 |
| Coccinella_quinquepunctata_239 | Tytthaspis_sedecimpunctata_589 | 0.178 |
| Coccinella_undecimpunctata_285 | Tytthaspis_sedecimpunctata_589 | 0.178 |
| Coccinella_undecimpunctata_286 | Tytthaspis_sedecimpunctata_589 | 0.178 |
| Coccinella_undecimpunctata_288 | Tytthaspis_sedecimpunctata_589 | 0.178 |
| Psyllobora_renifer_551 | Tytthaspis_sedecimpunctata_589 | 0.178 |
| Psyllobora_renifer_552 | Tytthaspis_sedecimpunctata_589 | 0.178 |
| Psyllobora_renifer_553 | Tytthaspis_sedecimpunctata_589 | 0.178 |
| Psyllobora_renifer_554 | Tytthaspis_sedecimpunctata_589 | 0.178 |
| Coccinella_hieroglyphica_227 | Tytthaspis_sedecimpunctata_591 | 0.178 |
| Psyllobora_vigintimaculata_573 | Tytthaspis_sedecimpunctata_591 | 0.178 |
| Adalia_bipunctata_98 | Tytthaspis_sedecimpunctata_592 | 0.178 |
| Didion_punctatum_646 | Scymnus_sinuanodulus_828 | 0.178 |
| Didion_punctatum_644 | Scymnus_sinuanodulus_830 | 0.178 |
| Platynaspis_luteorubra_54 | Scymnus_suturalis_832 | 0.178 |
| Platynaspis_luteorubra_57 | Scymnus_suturalis_832 | 0.178 |
| Platynaspis_luteorubra_59 | Scymnus_suturalis_832 | 0.178 |
| Platynaspis_luteorubra_55 | Scymnus_suturalis_834 | 0.178 |
| Platynaspis_luteorubra_61 | Scymnus_suturalis_834 | 0.178 |
| Platynaspis_luteorubra_54 | Scymnus_suturalis_835 | 0.178 |
| Platynaspis_luteorubra_57 | Scymnus_suturalis_835 | 0.178 |
| Platynaspis_luteorubra_59 | Scymnus_suturalis_835 | 0.178 |
| Platynaspis_luteorubra_54 | Scymnus_suturalis_836 | 0.178 |
| Platynaspis_luteorubra_57 | Scymnus_suturalis_836 | 0.178 |
| Platynaspis_luteorubra_59 | Scymnus_suturalis_836 | 0.178 |
| Platynaspis_luteorubra_54 | Scymnus_suturalis_839 | 0.178 |
| Platynaspis_luteorubra_57 | Scymnus_suturalis_839 | 0.178 |
| Platynaspis_luteorubra_59 | Scymnus_suturalis_839 | 0.178 |
| Adalia_bipunctata_98 | Tytthaspis_sedecimpunctata_580 | 0.178 |
| Olla_vnigrum_525 | Tytthaspis_sedecimpunctata_583 | 0.178 |
| Olla_vnigrum_529 | Tytthaspis_sedecimpunctata_583 | 0.178 |
| Olla_vnigrum_531 | Tytthaspis_sedecimpunctata_583 | 0.178 |
| Psyllobora_vigintimaculata_573 | Tytthaspis_sedecimpunctata_583 | 0.178 |
| Coccinella_quinquepunctata_237 | Tytthaspis_sedecimpunctata_586 | 0.178 |
| Coccinella_quinquepunctata_236 | Tytthaspis_sedecimpunctata_587 | 0.178 |
| Coccinella_quinquepunctata_239 | Tytthaspis_sedecimpunctata_587 | 0.178 |
| Scymnus_auritus_700 | Tytthaspis_sedecimpunctata_587 | 0.178 |
| Hippodamia_alpina_388 | Tytthaspis_sedecimpunctata_591 | 0.178 |
| Brumus_quadripustulatus_17 | Scymnus_rubromaculatus_822 | 0.178 |
| Brumus_quadripustulatus_17 | Scymnus_rubromaculatus_823 | 0.178 |
| Brumus_quadripustulatus_17 | Scymnus_rubromaculatus_824 | 0.178 |
| Brumus_quadripustulatus_17 | Scymnus_rubromaculatus_825 | 0.178 |
| Brumus_quadripustulatus_17 | Scymnus_rubromaculatus_826 | 0.178 |
| Brumus_quadripustulatus_17 | Scymnus_rubromaculatus_827 | 0.178 |
| Didion_punctatum_644 | Scymnus_sinuanodulus_828 | 0.178 |
| Chilocorus_nigritus_25 | Scymnus_sinuanodulus_830 | 0.178 |
| Coccinella_quinquepunctata_234 | Tytthaspis_sedecimpunctata_591 | 0.178 |
| Chilocorus_nigritus_29 | Scymnus_suturalis_838 | 0.178 |
| Chilocorus_nigritus_29 | Scymnus_suturalis_840 | 0.178 |
| Scymnus_auritus_700 | Tytthaspis_sedecimpunctata_577 | 0.178 |
| Scymnus_auritus_700 | Tytthaspis_sedecimpunctata_578 | 0.178 |
| Scymnus_auritus_700 | Tytthaspis_sedecimpunctata_579 | 0.178 |
| Scymnus_auritus_700 | Tytthaspis_sedecimpunctata_581 | 0.178 |
| Scymnus_auritus_700 | Tytthaspis_sedecimpunctata_582 | 0.178 |
| Illeis_galbula_474 | Tytthaspis_sedecimpunctata_583 | 0.178 |
| Illeis_galbula_475 | Tytthaspis_sedecimpunctata_583 | 0.178 |

| | | |
|---|---|---|
| Illeis_galbula_476 | Tytthaspis_sedecimpunctata_583 | 0.178 |
| Psyllobora_vigintimaculata_574 | Tytthaspis_sedecimpunctata_583 | 0.178 |
| Psyllobora_vigintimaculata_573 | Tytthaspis_sedecimpunctata_584 | 0.178 |
| Scymnus_auritus_703 | Tytthaspis_sedecimpunctata_584 | 0.178 |
| Scymnus_auritus_700 | Tytthaspis_sedecimpunctata_590 | 0.178 |
| Mulsantina_picta_484 | Tytthaspis_sedecimpunctata_591 | 0.178 |
| Scymnus_auritus_703 | Tytthaspis_sedecimpunctata_591 | 0.178 |
| Coccinella_hieroglyphica_226 | Tytthaspis_sedecimpunctata_593 | 0.178 |
| Scymnus_auritus_698 | Tytthaspis_sedecimpunctata_593 | 0.178 |
| Scymnus_auritus_700 | Tytthaspis_sedecimpunctata_594 | 0.178 |
| Scymnus_auritus_700 | Tytthaspis_sedecimpunctata_595 | 0.178 |
| Brumus_quadripustulatus_11 | Scymnus_suturalis_838 | 0.178 |
| Brumus_quadripustulatus_13 | Scymnus_suturalis_838 | 0.178 |
| Brumus_quadripustulatus_14 | Scymnus_suturalis_838 | 0.178 |
| Brumus_quadripustulatus_3 | Scymnus_suturalis_838 | 0.178 |
| Brumus_quadripustulatus_4 | Scymnus_suturalis_838 | 0.178 |
| Brumus_quadripustulatus_7 | Scymnus_suturalis_838 | 0.178 |
| Brumus_quadripustulatus_11 | Scymnus_suturalis_840 | 0.178 |
| Brumus_quadripustulatus_13 | Scymnus_suturalis_840 | 0.178 |
| Brumus_quadripustulatus_14 | Scymnus_suturalis_840 | 0.178 |
| Brumus_quadripustulatus_3 | Scymnus_suturalis_840 | 0.178 |
| Brumus_quadripustulatus_4 | Scymnus_suturalis_840 | 0.178 |
| Brumus_quadripustulatus_7 | Scymnus_suturalis_840 | 0.178 |
| Chilocorus_nigritus_30 | Scymnus_suturalis_841 | 0.178 |
| Chilocorus_nigritus_30 | Scymnus_suturalis_842 | 0.178 |
| Chilocorus_nigritus_30 | Scymnus_suturalis_844 | 0.178 |
| Chilocorus_nigritus_30 | Scymnus_suturalis_846 | 0.178 |
| Chilocorus_nigritus_30 | Scymnus_suturalis_847 | 0.178 |
| Chilocorus_nigritus_30 | Scymnus_suturalis_848 | 0.178 |
| Chilocorus_nigritus_30 | Scymnus_suturalis_832 | 0.178 |
| Chilocorus_nigritus_29 | Scymnus_suturalis_834 | 0.178 |
| Chilocorus_nigritus_30 | Scymnus_suturalis_835 | 0.178 |
| Chilocorus_nigritus_30 | Scymnus_suturalis_836 | 0.178 |
| Chilocorus_nigritus_29 | Scymnus_suturalis_837 | 0.178 |
| Chilocorus_nigritus_30 | Scymnus_suturalis_839 | 0.178 |
| Scymnus_auritus_703 | Scymnus_sinuanodulus_829 | 0.178 |
| Coccidula_scutellata_84 | Scymnus_sinuanodulus_828 | 0.178 |
| Coccidula_scutellata_78 | Scymnus_sinuanodulus_831 | 0.178 |
| Coccidula_scutellata_79 | Scymnus_sinuanodulus_831 | 0.178 |
| Anatis_ocellata_136 | Scymnus_suturalis_838 | 0.178 |
| Anatis_ocellata_136 | Scymnus_suturalis_840 | 0.178 |
| Cycloneda_sanguinea_342 | Tytthaspis_sedecimpunctata_584 | 0.178 |
| Psyllobora_vigintimaculata_574 | Tytthaspis_sedecimpunctata_584 | 0.178 |
| Hippodamia_undecimnotata_460 | Tytthaspis_sedecimpunctata_585 | 0.178 |
| Hippodamia_undecimnotata_461 | Tytthaspis_sedecimpunctata_585 | 0.178 |
| Hippodamia_undecimnotata_462 | Tytthaspis_sedecimpunctata_585 | 0.178 |
| Hippodamia_undecimnotata_463 | Tytthaspis_sedecimpunctata_585 | 0.178 |
| Hippodamia_undecimnotata_464 | Tytthaspis_sedecimpunctata_585 | 0.178 |
| Hippodamia_undecimnotata_465 | Tytthaspis_sedecimpunctata_585 | 0.178 |
| Hippodamia_undecimnotata_466 | Tytthaspis_sedecimpunctata_585 | 0.178 |
| Hippodamia_notata_412 | Tytthaspis_sedecimpunctata_589 | 0.178 |
| Hippodamia_notata_414 | Tytthaspis_sedecimpunctata_589 | 0.178 |
| Hippodamia_notata_416 | Tytthaspis_sedecimpunctata_589 | 0.178 |
| Hippodamia_notata_417 | Tytthaspis_sedecimpunctata_589 | 0.178 |
| Hippodamia_notata_420 | Tytthaspis_sedecimpunctata_589 | 0.178 |
| Hippodamia_notata_425 | Tytthaspis_sedecimpunctata_589 | 0.178 |

| | | |
|---|---|---|
| Hippodamia_notata_428 | Tytthaspis_sedecimpunctata_589 | 0.178 |
| Hippodamia_notata_430 | Tytthaspis_sedecimpunctata_589 | 0.178 |
| Hyperaspis_lateralis_656 | Scymnus_rubromaculatus_822 | 0.178 |
| Hyperaspis_lateralis_656 | Scymnus_rubromaculatus_823 | 0.178 |
| Hyperaspis_lateralis_656 | Scymnus_rubromaculatus_824 | 0.178 |
| Hyperaspis_lateralis_656 | Scymnus_rubromaculatus_825 | 0.178 |
| Hyperaspis_lateralis_656 | Scymnus_rubromaculatus_826 | 0.178 |
| Hyperaspis_lateralis_656 | Scymnus_rubromaculatus_827 | 0.178 |
| Brumus_quadripustulatus_11 | Scymnus_suturalis_834 | 0.178 |
| Brumus_quadripustulatus_13 | Scymnus_suturalis_834 | 0.178 |
| Brumus_quadripustulatus_14 | Scymnus_suturalis_834 | 0.178 |
| Brumus_quadripustulatus_3 | Scymnus_suturalis_834 | 0.178 |
| Brumus_quadripustulatus_4 | Scymnus_suturalis_834 | 0.178 |
| Brumus_quadripustulatus_7 | Scymnus_suturalis_834 | 0.178 |
| Brumus_quadripustulatus_11 | Scymnus_suturalis_837 | 0.178 |
| Brumus_quadripustulatus_13 | Scymnus_suturalis_837 | 0.178 |
| Brumus_quadripustulatus_14 | Scymnus_suturalis_837 | 0.178 |
| Brumus_quadripustulatus_3 | Scymnus_suturalis_837 | 0.178 |
| Brumus_quadripustulatus_4 | Scymnus_suturalis_837 | 0.178 |
| Brumus_quadripustulatus_7 | Scymnus_suturalis_837 | 0.178 |
| Brumus_quadripustulatus_9 | Scymnus_suturalis_838 | 0.178 |
| Brumus_quadripustulatus_9 | Scymnus_suturalis_840 | 0.178 |
| Anatis_ocellata_136 | Scymnus_suturalis_834 | 0.178 |
| Anatis_ocellata_136 | Scymnus_suturalis_837 | 0.178 |
| Scymnus_ferrugatus_724 | Scymnus_sinuanodulus_829 | 0.178 |
| Scymnus_ferrugatus_729 | Scymnus_sinuanodulus_829 | 0.178 |
| Brumoides_suturalis_2 | Scymnus_suturalis_843 | 0.178 |
| Brumoides_suturalis_2 | Scymnus_suturalis_845 | 0.178 |
| Cycloneda_sanguinea_342 | Tytthaspis_sedecimpunctata_577 | 0.178 |
| Cycloneda_sanguinea_342 | Tytthaspis_sedecimpunctata_578 | 0.178 |
| Cycloneda_sanguinea_342 | Tytthaspis_sedecimpunctata_579 | 0.178 |
| Cycloneda_sanguinea_342 | Tytthaspis_sedecimpunctata_581 | 0.178 |
| Cycloneda_sanguinea_342 | Tytthaspis_sedecimpunctata_582 | 0.178 |
| Cycloneda_sanguinea_340 | Tytthaspis_sedecimpunctata_583 | 0.178 |
| Cycloneda_sanguinea_341 | Tytthaspis_sedecimpunctata_583 | 0.178 |
| Cycloneda_sanguinea_343 | Tytthaspis_sedecimpunctata_583 | 0.178 |
| Cycloneda_sanguinea_344 | Tytthaspis_sedecimpunctata_583 | 0.178 |
| Hippodamia_notata_415 | Tytthaspis_sedecimpunctata_586 | 0.178 |
| Hippodamia_notata_412 | Tytthaspis_sedecimpunctata_587 | 0.178 |
| Hippodamia_notata_414 | Tytthaspis_sedecimpunctata_587 | 0.178 |
| Hippodamia_notata_416 | Tytthaspis_sedecimpunctata_587 | 0.178 |
| Hippodamia_notata_417 | Tytthaspis_sedecimpunctata_587 | 0.178 |
| Hippodamia_notata_420 | Tytthaspis_sedecimpunctata_587 | 0.178 |
| Hippodamia_notata_425 | Tytthaspis_sedecimpunctata_587 | 0.178 |
| Hippodamia_notata_428 | Tytthaspis_sedecimpunctata_587 | 0.178 |
| Hippodamia_notata_430 | Tytthaspis_sedecimpunctata_587 | 0.178 |
| Hippodamia_notata_415 | Tytthaspis_sedecimpunctata_588 | 0.178 |
| Neoharmonia_venusta_520 | Tytthaspis_sedecimpunctata_588 | 0.178 |
| Cycloneda_sanguinea_342 | Tytthaspis_sedecimpunctata_589 | 0.178 |
| Hippodamia_notata_419 | Tytthaspis_sedecimpunctata_589 | 0.178 |
| Hippodamia_notata_431 | Tytthaspis_sedecimpunctata_589 | 0.178 |
| Cycloneda_sanguinea_342 | Tytthaspis_sedecimpunctata_590 | 0.178 |
| Neoharmonia_venusta_520 | Tytthaspis_sedecimpunctata_591 | 0.178 |
| Cycloneda_sanguinea_342 | Tytthaspis_sedecimpunctata_592 | 0.178 |
| Cycloneda_sanguinea_342 | Tytthaspis_sedecimpunctata_594 | 0.178 |
| Cycloneda_sanguinea_342 | Tytthaspis_sedecimpunctata_595 | 0.178 |

Supplementary Table S4 (continued)

| | | |
|---|---|---|
| Hyperaspis_reppensis_665 | Scymnus_rubromaculatus_822 | 0.178 |
| Hyperaspis_reppensis_665 | Scymnus_rubromaculatus_823 | 0.178 |
| Hyperaspis_reppensis_665 | Scymnus_rubromaculatus_824 | 0.178 |
| Hyperaspis_reppensis_665 | Scymnus_rubromaculatus_825 | 0.178 |
| Hyperaspis_reppensis_665 | Scymnus_rubromaculatus_826 | 0.178 |
| Hyperaspis_reppensis_665 | Scymnus_rubromaculatus_827 | 0.178 |
| Brumoides_suturalis_2 | Scymnus_suturalis_834 | 0.178 |
| Brumus_quadripustulatus_9 | Scymnus_suturalis_834 | 0.178 |
| Brumus_quadripustulatus_9 | Scymnus_suturalis_837 | 0.178 |
| Chilocorus_nigritus_26 | Scymnus_sinuanodulus_831 | 0.178 |
| Cycloneda_sanguinea_342 | Tytthaspis_sedecimpunctata_580 | 0.178 |
| Neoharmonia_venusta_520 | Tytthaspis_sedecimpunctata_583 | 0.178 |
| Neoharmonia_venusta_520 | Tytthaspis_sedecimpunctata_585 | 0.178 |
| Hippodamia_arctica_389 | Tytthaspis_sedecimpunctata_586 | 0.178 |
| Hippodamia_notata_413 | Tytthaspis_sedecimpunctata_586 | 0.178 |
| Hippodamia_notata_418 | Tytthaspis_sedecimpunctata_586 | 0.178 |
| Hippodamia_notata_419 | Tytthaspis_sedecimpunctata_586 | 0.178 |
| Hippodamia_notata_431 | Tytthaspis_sedecimpunctata_586 | 0.178 |
| Cycloneda_sanguinea_340 | Tytthaspis_sedecimpunctata_587 | 0.178 |
| Cycloneda_sanguinea_341 | Tytthaspis_sedecimpunctata_587 | 0.178 |
| Cycloneda_sanguinea_343 | Tytthaspis_sedecimpunctata_587 | 0.178 |
| Cycloneda_sanguinea_344 | Tytthaspis_sedecimpunctata_587 | 0.178 |
| Hippodamia_notata_421 | Tytthaspis_sedecimpunctata_587 | 0.178 |
| Hippodamia_notata_413 | Tytthaspis_sedecimpunctata_588 | 0.178 |
| Hippodamia_notata_418 | Tytthaspis_sedecimpunctata_588 | 0.178 |
| Hippodamia_notata_419 | Tytthaspis_sedecimpunctata_588 | 0.178 |
| Hippodamia_notata_431 | Tytthaspis_sedecimpunctata_588 | 0.178 |
| Neoharmonia_venusta_521 | Tytthaspis_sedecimpunctata_588 | 0.178 |
| Neoharmonia_venusta_521 | Tytthaspis_sedecimpunctata_591 | 0.178 |
| Aphidecta_obliterata_179 | Vibidia_duodecimguttata_597 | 0.178 |
| Aphidecta_obliterata_179 | Vibidia_duodecimguttata_600 | 0.178 |
| Hyperaspis_campestris_651 | Subcoccinella_vigintiquatuorpunctata_622 | 0.178 |
| Hyperaspis_campestris_652 | Subcoccinella_vigintiquatuorpunctata_622 | 0.178 |
| Hyperaspis_campestris_653 | Subcoccinella_vigintiquatuorpunctata_622 | 0.178 |
| Hyperaspis_reppensis_665 | Subcoccinella_vigintiquatuorpunctata_624 | 0.178 |
| Hyperaspis_reppensis_664 | Subcoccinella_vigintiquatuorpunctata_625 | 0.178 |
| Hyperaspis_reppensis_666 | Subcoccinella_vigintiquatuorpunctata_625 | 0.178 |
| Hyperaspis_reppensis_667 | Subcoccinella_vigintiquatuorpunctata_625 | 0.178 |
| Hyperaspis_campestris_651 | Subcoccinella_vigintiquatuorpunctata_627 | 0.178 |
| Hyperaspis_campestris_652 | Subcoccinella_vigintiquatuorpunctata_627 | 0.178 |
| Hyperaspis_campestris_653 | Subcoccinella_vigintiquatuorpunctata_627 | 0.178 |
| Hyperaspis_campestris_651 | Subcoccinella_vigintiquatuorpunctata_630 | 0.178 |
| Hyperaspis_campestris_652 | Subcoccinella_vigintiquatuorpunctata_630 | 0.178 |
| Hyperaspis_campestris_653 | Subcoccinella_vigintiquatuorpunctata_630 | 0.178 |
| Hyperaspis_proba_661 | Subcoccinella_vigintiquatuorpunctata_630 | 0.178 |
| Neoharmonia_venusta_521 | Tytthaspis_sedecimpunctata_583 | 0.178 |
| Neoharmonia_venusta_520 | Tytthaspis_sedecimpunctata_584 | 0.178 |
| Neoharmonia_venusta_521 | Tytthaspis_sedecimpunctata_585 | 0.178 |
| Ceratomegilla_ulkei_219 | Tytthaspis_sedecimpunctata_586 | 0.178 |
| Ceratomegilla_ulkei_219 | Tytthaspis_sedecimpunctata_587 | 0.178 |
| Myzia_pullata_519 | Vibidia_duodecimguttata_597 | 0.178 |
| Aphidecta_obliterata_181 | Vibidia_duodecimguttata_598 | 0.178 |
| Myzia_pullata_519 | Vibidia_duodecimguttata_600 | 0.178 |
| Scymnus_rubromaculatus_813 | Scymnus_sinuanodulus_828 | 0.178 |
| Scymnus_rubromaculatus_815 | Scymnus_sinuanodulus_828 | 0.178 |
| Scymnus_rubromaculatus_816 | Scymnus_sinuanodulus_828 | 0.178 |

| | | |
|---|---|---|
| Scymnus_rubromaculatus_817 | Scymnus_sinuanodulus_828 | 0.178 |
| Scymnus_rubromaculatus_818 | Scymnus_sinuanodulus_828 | 0.178 |
| Scymnus_rubromaculatus_819 | Scymnus_sinuanodulus_828 | 0.178 |
| Scymnus_rubromaculatus_820 | Scymnus_sinuanodulus_828 | 0.178 |
| Scymnus_rubromaculatus_821 | Scymnus_sinuanodulus_828 | 0.178 |
| Scymnus_rubromaculatus_822 | Scymnus_sinuanodulus_828 | 0.178 |
| Scymnus_rubromaculatus_823 | Scymnus_sinuanodulus_828 | 0.178 |
| Scymnus_rubromaculatus_824 | Scymnus_sinuanodulus_828 | 0.178 |
| Scymnus_rubromaculatus_825 | Scymnus_sinuanodulus_828 | 0.178 |
| Scymnus_rubromaculatus_826 | Scymnus_sinuanodulus_828 | 0.178 |
| Scymnus_rubromaculatus_827 | Scymnus_sinuanodulus_828 | 0.178 |
| Hyperaspis_oregona_658 | Scymnus_sinuanodulus_830 | 0.178 |
| Scymnus_frontalis_734 | Scymnus_sinuanodulus_831 | 0.178 |
| Scymnus_frontalis_742 | Scymnus_sinuanodulus_831 | 0.178 |
| Scymnus_frontalis_745 | Scymnus_sinuanodulus_831 | 0.178 |
| Neoharmonia_venusta_521 | Tytthaspis_sedecimpunctata_584 | 0.178 |
| Myzia_pullata_519 | Vibidia_duodecimguttata_598 | 0.178 |
| Scymnus_haemorrhoidalis_749 | Scymnus_rubromaculatus_822 | 0.178 |
| Scymnus_haemorrhoidalis_749 | Scymnus_rubromaculatus_823 | 0.178 |
| Scymnus_haemorrhoidalis_749 | Scymnus_rubromaculatus_824 | 0.178 |
| Scymnus_haemorrhoidalis_749 | Scymnus_rubromaculatus_825 | 0.178 |
| Scymnus_haemorrhoidalis_749 | Scymnus_rubromaculatus_826 | 0.178 |
| Scymnus_haemorrhoidalis_749 | Scymnus_rubromaculatus_827 | 0.178 |
| Hyperaspis_reppensis_664 | Scymnus_sinuanodulus_830 | 0.178 |
| Hyperaspis_reppensis_667 | Scymnus_sinuanodulus_830 | 0.178 |
| Scymnus_haemorrhoidalis_756 | Scymnus_sinuanodulus_830 | 0.178 |
| Scymnus_camptodromus_717 | Tytthaspis_sedecimpunctata_577 | 0.178 |
| Scymnus_camptodromus_717 | Tytthaspis_sedecimpunctata_578 | 0.178 |
| Scymnus_camptodromus_717 | Tytthaspis_sedecimpunctata_579 | 0.178 |
| Scymnus_camptodromus_717 | Tytthaspis_sedecimpunctata_581 | 0.178 |
| Scymnus_camptodromus_717 | Tytthaspis_sedecimpunctata_582 | 0.178 |
| Scymnus_camptodromus_717 | Tytthaspis_sedecimpunctata_585 | 0.178 |
| Scymnus_camptodromus_717 | Tytthaspis_sedecimpunctata_590 | 0.178 |
| Scymnus_camptodromus_717 | Tytthaspis_sedecimpunctata_592 | 0.178 |
| Scymnus_camptodromus_717 | Tytthaspis_sedecimpunctata_593 | 0.178 |
| Scymnus_camptodromus_717 | Tytthaspis_sedecimpunctata_594 | 0.178 |
| Scymnus_camptodromus_717 | Tytthaspis_sedecimpunctata_595 | 0.178 |
| Hyperaspis_reppensis_664 | Scymnus_sinuanodulus_828 | 0.179 |
| Hyperaspis_reppensis_667 | Scymnus_sinuanodulus_828 | 0.179 |
| Scymnus_haemorrhoidalis_756 | Scymnus_sinuanodulus_828 | 0.179 |
| Scymnus_camptodromus_711 | Tytthaspis_sedecimpunctata_577 | 0.179 |
| Scymnus_camptodromus_711 | Tytthaspis_sedecimpunctata_578 | 0.179 |
| Scymnus_camptodromus_711 | Tytthaspis_sedecimpunctata_579 | 0.179 |
| Scymnus_camptodromus_711 | Tytthaspis_sedecimpunctata_581 | 0.179 |
| Scymnus_camptodromus_711 | Tytthaspis_sedecimpunctata_582 | 0.179 |
| Scymnus_camptodromus_711 | Tytthaspis_sedecimpunctata_585 | 0.179 |
| Scymnus_camptodromus_711 | Tytthaspis_sedecimpunctata_590 | 0.179 |
| Scymnus_camptodromus_711 | Tytthaspis_sedecimpunctata_592 | 0.179 |
| Scymnus_camptodromus_711 | Tytthaspis_sedecimpunctata_593 | 0.179 |
| Scymnus_camptodromus_711 | Tytthaspis_sedecimpunctata_594 | 0.179 |
| Scymnus_camptodromus_711 | Tytthaspis_sedecimpunctata_595 | 0.179 |
| Coccidula_scutellata_78 | Scymnus_sinuanodulus_829 | 0.179 |
| Coccidula_scutellata_79 | Scymnus_sinuanodulus_829 | 0.179 |
| Scymnus_mitior_780 | Scymnus_suturalis_833 | 0.179 |
| Scymnus_mitior_779 | Scymnus_suturalis_838 | 0.179 |
| Scymnus_mitior_779 | Scymnus_suturalis_840 | 0.179 |

| | | |
|---|---|---|
| Hyperaspis_proba_660 | Subcoccinella_vigintiquatuorpunctata_624 | 0.179 |
| Hyperaspis_proba_660 | Subcoccinella_vigintiquatuorpunctata_627 | 0.179 |
| Scymnus_frontalis_735 | Scymnus_sinuanodulus_831 | 0.179 |
| Scymnus_mitior_780 | Scymnus_suturalis_841 | 0.179 |
| Scymnus_mitior_780 | Scymnus_suturalis_842 | 0.179 |
| Scymnus_mitior_779 | Scymnus_suturalis_843 | 0.179 |
| Scymnus_mitior_780 | Scymnus_suturalis_844 | 0.179 |
| Scymnus_mitior_780 | Scymnus_suturalis_846 | 0.179 |
| Scymnus_mitior_780 | Scymnus_suturalis_847 | 0.179 |
| Scymnus_mitior_780 | Scymnus_suturalis_848 | 0.179 |
| Calvia_decemguttata_193 | Tytthaspis_sedecimpunctata_580 | 0.179 |
| Calvia_decemguttata_194 | Tytthaspis_sedecimpunctata_580 | 0.179 |
| Psyllobora_vigintiduopunctata_556 | Vibidia_duodecimguttata_596 | 0.179 |
| Psyllobora_vigintiduopunctata_560 | Vibidia_duodecimguttata_597 | 0.179 |
| Psyllobora_vigintiduopunctata_564 | Vibidia_duodecimguttata_597 | 0.179 |
| Psyllobora_vigintiduopunctata_570 | Vibidia_duodecimguttata_597 | 0.179 |
| Psyllobora_vigintiduopunctata_572 | Vibidia_duodecimguttata_597 | 0.179 |
| Psyllobora_vigintiduopunctata_560 | Vibidia_duodecimguttata_600 | 0.179 |
| Psyllobora_vigintiduopunctata_564 | Vibidia_duodecimguttata_600 | 0.179 |
| Psyllobora_vigintiduopunctata_570 | Vibidia_duodecimguttata_600 | 0.179 |
| Psyllobora_vigintiduopunctata_572 | Vibidia_duodecimguttata_600 | 0.179 |
| Scymnus_auritus_703 | Scymnus_sinuanodulus_830 | 0.179 |
| Scymnus_mitior_780 | Scymnus_suturalis_832 | 0.179 |
| Scymnus_mitior_780 | Scymnus_suturalis_835 | 0.179 |
| Scymnus_mitior_780 | Scymnus_suturalis_836 | 0.179 |
| Scymnus_mitior_780 | Scymnus_suturalis_837 | 0.179 |
| Scymnus_mitior_780 | Scymnus_suturalis_839 | 0.179 |
| Chilocorus_nigritus_26 | Scymnus_sinuanodulus_829 | 0.179 |
| Coccinella_undecimpunctata_287 | Tytthaspis_sedecimpunctata_587 | 0.179 |
| Calvia_decemguttata_187 | Vibidia_duodecimguttata_596 | 0.179 |
| Calvia_decemguttata_188 | Vibidia_duodecimguttata_596 | 0.179 |
| Calvia_decemguttata_189 | Vibidia_duodecimguttata_596 | 0.179 |
| Calvia_decemguttata_190 | Vibidia_duodecimguttata_596 | 0.179 |
| Calvia_decemguttata_191 | Vibidia_duodecimguttata_596 | 0.179 |
| Calvia_decemguttata_192 | Vibidia_duodecimguttata_596 | 0.179 |
| Calvia_decemguttata_196 | Vibidia_duodecimguttata_596 | 0.179 |
| Calvia_decemguttata_197 | Vibidia_duodecimguttata_596 | 0.179 |
| Calvia_decemguttata_198 | Vibidia_duodecimguttata_596 | 0.179 |
| Calvia_decemguttata_199 | Vibidia_duodecimguttata_596 | 0.179 |
| Calvia_decemguttata_200 | Vibidia_duodecimguttata_596 | 0.179 |
| Psyllobora_vigintiduopunctata_561 | Vibidia_duodecimguttata_596 | 0.179 |
| Psyllobora_vigintiduopunctata_562 | Vibidia_duodecimguttata_596 | 0.179 |
| Psyllobora_vigintiduopunctata_565 | Vibidia_duodecimguttata_596 | 0.179 |
| Psyllobora_vigintiduopunctata_567 | Vibidia_duodecimguttata_596 | 0.179 |
| Calvia_decemguttata_187 | Vibidia_duodecimguttata_598 | 0.179 |
| Calvia_decemguttata_188 | Vibidia_duodecimguttata_598 | 0.179 |
| Calvia_decemguttata_189 | Vibidia_duodecimguttata_598 | 0.179 |
| Calvia_decemguttata_190 | Vibidia_duodecimguttata_598 | 0.179 |
| Calvia_decemguttata_191 | Vibidia_duodecimguttata_598 | 0.179 |
| Calvia_decemguttata_192 | Vibidia_duodecimguttata_598 | 0.179 |
| Calvia_decemguttata_196 | Vibidia_duodecimguttata_598 | 0.179 |
| Calvia_decemguttata_197 | Vibidia_duodecimguttata_598 | 0.179 |
| Calvia_decemguttata_198 | Vibidia_duodecimguttata_598 | 0.179 |
| Calvia_decemguttata_199 | Vibidia_duodecimguttata_598 | 0.179 |
| Calvia_decemguttata_200 | Vibidia_duodecimguttata_598 | 0.179 |
| Psyllobora_vigintiduopunctata_560 | Vibidia_duodecimguttata_598 | 0.179 |

| | | |
|---|---|---|
| Psyllobora_vigintiduopunctata_564 | Vibidia_duodecimguttata_598 | 0.179 |
| Psyllobora_vigintiduopunctata_570 | Vibidia_duodecimguttata_598 | 0.179 |
| Psyllobora_vigintiduopunctata_572 | Vibidia_duodecimguttata_598 | 0.179 |
| Calvia_decemguttata_193 | Vibidia_duodecimguttata_599 | 0.179 |
| Calvia_decemguttata_194 | Vibidia_duodecimguttata_599 | 0.179 |
| Psyllobora_vigintiduopunctata_557 | Vibidia_duodecimguttata_599 | 0.179 |
| Psyllobora_vigintiduopunctata_563 | Vibidia_duodecimguttata_599 | 0.179 |
| Calvia_decemguttata_193 | Vibidia_duodecimguttata_601 | 0.179 |
| Calvia_decemguttata_194 | Vibidia_duodecimguttata_601 | 0.179 |
| Psyllobora_vigintiduopunctata_557 | Vibidia_duodecimguttata_601 | 0.179 |
| Psyllobora_vigintiduopunctata_563 | Vibidia_duodecimguttata_601 | 0.179 |
| Calvia_decemguttata_193 | Vibidia_duodecimguttata_602 | 0.179 |
| Calvia_decemguttata_194 | Vibidia_duodecimguttata_602 | 0.179 |
| Psyllobora_vigintiduopunctata_557 | Vibidia_duodecimguttata_602 | 0.179 |
| Psyllobora_vigintiduopunctata_563 | Vibidia_duodecimguttata_602 | 0.179 |
| Psyllobora_vigintiduopunctata_560 | Vibidia_duodecimguttata_603 | 0.179 |
| Psyllobora_vigintiduopunctata_564 | Vibidia_duodecimguttata_603 | 0.179 |
| Psyllobora_vigintiduopunctata_570 | Vibidia_duodecimguttata_603 | 0.179 |
| Psyllobora_vigintiduopunctata_572 | Vibidia_duodecimguttata_603 | 0.179 |
| Scymnus_auritus_701 | Scymnus_sinuanodulus_828 | 0.179 |
| Coccinella_undecimpunctata_287 | Tytthaspis_sedecimpunctata_577 | 0.179 |
| Coccinella_undecimpunctata_287 | Tytthaspis_sedecimpunctata_578 | 0.179 |
| Coccinella_undecimpunctata_287 | Tytthaspis_sedecimpunctata_579 | 0.179 |
| Coccinella_undecimpunctata_287 | Tytthaspis_sedecimpunctata_581 | 0.179 |
| Coccinella_undecimpunctata_287 | Tytthaspis_sedecimpunctata_582 | 0.179 |
| Scymnus_camptodromus_706 | Tytthaspis_sedecimpunctata_584 | 0.179 |
| Coccinella_undecimpunctata_287 | Tytthaspis_sedecimpunctata_585 | 0.179 |
| Coccinella_undecimpunctata_287 | Tytthaspis_sedecimpunctata_590 | 0.179 |
| Coccinella_undecimpunctata_287 | Tytthaspis_sedecimpunctata_592 | 0.179 |
| Coccinella_undecimpunctata_287 | Tytthaspis_sedecimpunctata_594 | 0.179 |
| Coccinella_undecimpunctata_287 | Tytthaspis_sedecimpunctata_595 | 0.179 |
| Scymnus_frontalis_734 | Scymnus_sinuanodulus_829 | 0.179 |
| Scymnus_frontalis_742 | Scymnus_sinuanodulus_829 | 0.179 |
| Scymnus_frontalis_745 | Scymnus_sinuanodulus_829 | 0.179 |
| Hyperaspis_proba_660 | Subcoccinella_vigintiquatuorpunctata_619 | 0.179 |
| Calvia_decemguttata_187 | Tytthaspis_sedecimpunctata_587 | 0.179 |
| Calvia_decemguttata_188 | Tytthaspis_sedecimpunctata_587 | 0.179 |
| Calvia_decemguttata_189 | Tytthaspis_sedecimpunctata_587 | 0.179 |
| Calvia_decemguttata_190 | Tytthaspis_sedecimpunctata_587 | 0.179 |
| Calvia_decemguttata_191 | Tytthaspis_sedecimpunctata_587 | 0.179 |
| Calvia_decemguttata_192 | Tytthaspis_sedecimpunctata_587 | 0.179 |
| Calvia_decemguttata_196 | Tytthaspis_sedecimpunctata_587 | 0.179 |
| Calvia_decemguttata_197 | Tytthaspis_sedecimpunctata_587 | 0.179 |
| Calvia_decemguttata_198 | Tytthaspis_sedecimpunctata_587 | 0.179 |
| Anatis_ocellata_133 | Vibidia_duodecimguttata_596 | 0.179 |
| Anatis_ocellata_137 | Vibidia_duodecimguttata_596 | 0.179 |
| Anatis_ocellata_143 | Vibidia_duodecimguttata_596 | 0.179 |
| Myzia_oblongoguttata_506 | Vibidia_duodecimguttata_596 | 0.179 |
| Calvia_decemguttata_193 | Vibidia_duodecimguttata_597 | 0.179 |
| Calvia_decemguttata_194 | Vibidia_duodecimguttata_597 | 0.179 |
| Calvia_decemguttata_199 | Vibidia_duodecimguttata_597 | 0.179 |
| Calvia_decemguttata_200 | Vibidia_duodecimguttata_597 | 0.179 |
| Calvia_decemguttata_193 | Vibidia_duodecimguttata_600 | 0.179 |
| Calvia_decemguttata_194 | Vibidia_duodecimguttata_600 | 0.179 |
| Calvia_decemguttata_199 | Vibidia_duodecimguttata_600 | 0.179 |
| Calvia_decemguttata_200 | Vibidia_duodecimguttata_600 | 0.179 |

| | | |
|---|---|---|
| Coccinella_undecimpunctata_287 | Tytthaspis_sedecimpunctata_591 | 0.179 |
| Scymnus_frontalis_746 | Scymnus_sinuanodulus_831 | 0.179 |
| Platynaspis_luteorubra_50 | Tytthaspis_sedecimpunctata_591 | 0.179 |
| Scymnus_haemorrhoidalis_749 | Scymnus_sinuanodulus_831 | 0.179 |
| Anatis_ocellata_132 | Vibidia_duodecimguttata_596 | 0.179 |
| Anatis_ocellata_134 | Vibidia_duodecimguttata_596 | 0.179 |
| Anatis_ocellata_135 | Vibidia_duodecimguttata_596 | 0.179 |
| Anatis_ocellata_138 | Vibidia_duodecimguttata_596 | 0.179 |
| Anatis_ocellata_139 | Vibidia_duodecimguttata_596 | 0.179 |
| Anatis_ocellata_140 | Vibidia_duodecimguttata_596 | 0.179 |
| Anatis_ocellata_141 | Vibidia_duodecimguttata_596 | 0.179 |
| Anatis_ocellata_142 | Vibidia_duodecimguttata_596 | 0.179 |
| Anatis_ocellata_144 | Vibidia_duodecimguttata_596 | 0.179 |
| Anatis_ocellata_145 | Vibidia_duodecimguttata_596 | 0.179 |
| Anatis_ocellata_146 | Vibidia_duodecimguttata_596 | 0.179 |
| Scymnus_frontalis_735 | Scymnus_sinuanodulus_829 | 0.179 |
| Psyllobora_vigintiduopunctata_558 | Vibidia_duodecimguttata_597 | 0.179 |
| Psyllobora_vigintiduopunctata_568 | Vibidia_duodecimguttata_597 | 0.179 |
| Psyllobora_vigintiduopunctata_558 | Vibidia_duodecimguttata_600 | 0.179 |
| Psyllobora_vigintiduopunctata_568 | Vibidia_duodecimguttata_600 | 0.179 |
| Scymnus_ferrugatus_724 | Scymnus_sinuanodulus_830 | 0.179 |
| Scymnus_ferrugatus_729 | Scymnus_sinuanodulus_830 | 0.179 |
| Anatis_ocellata_136 | Vibidia_duodecimguttata_596 | 0.179 |
| Platynaspis_luteorubra_55 | Tytthaspis_sedecimpunctata_593 | 0.179 |
| Platynaspis_luteorubra_51 | Tytthaspis_sedecimpunctata_586 | 0.179 |
| Platynaspis_luteorubra_53 | Tytthaspis_sedecimpunctata_586 | 0.179 |
| Platynaspis_luteorubra_58 | Tytthaspis_sedecimpunctata_586 | 0.179 |
| Platynaspis_luteorubra_55 | Tytthaspis_sedecimpunctata_588 | 0.179 |
| Psyllobora_vigintiduopunctata_558 | Vibidia_duodecimguttata_598 | 0.179 |
| Psyllobora_vigintiduopunctata_568 | Vibidia_duodecimguttata_598 | 0.179 |
| Psyllobora_vigintiduopunctata_558 | Vibidia_duodecimguttata_603 | 0.179 |
| Psyllobora_vigintiduopunctata_568 | Vibidia_duodecimguttata_603 | 0.179 |
| Hyperaspis_reppensis_667 | Tytthaspis_sedecimpunctata_589 | 0.179 |
| Hyperaspis_reppensis_667 | Tytthaspis_sedecimpunctata_592 | 0.179 |
| Hyperaspis_reppensis_667 | Tytthaspis_sedecimpunctata_580 | 0.179 |
| Hyperaspis_reppensis_664 | Tytthaspis_sedecimpunctata_586 | 0.179 |
| Hyperaspis_reppensis_664 | Tytthaspis_sedecimpunctata_588 | 0.179 |
| Calvia_quatuordecimguttata_205 | Vibidia_duodecimguttata_597 | 0.180 |
| Calvia_quatuordecimguttata_203 | Vibidia_duodecimguttata_598 | 0.180 |
| Calvia_quatuordecimguttata_203 | Vibidia_duodecimguttata_599 | 0.180 |
| Calvia_quatuordecimguttata_205 | Vibidia_duodecimguttata_600 | 0.180 |
| Calvia_quatuordecimguttata_203 | Vibidia_duodecimguttata_601 | 0.180 |
| Calvia_quatuordecimguttata_203 | Vibidia_duodecimguttata_602 | 0.180 |
| Scymnus_ferrugatus_724 | Scymnus_rubromaculatus_822 | 0.180 |
| Scymnus_ferrugatus_729 | Scymnus_rubromaculatus_822 | 0.180 |
| Scymnus_ferrugatus_724 | Scymnus_rubromaculatus_823 | 0.180 |
| Scymnus_ferrugatus_729 | Scymnus_rubromaculatus_823 | 0.180 |
| Scymnus_ferrugatus_724 | Scymnus_rubromaculatus_824 | 0.180 |
| Scymnus_ferrugatus_729 | Scymnus_rubromaculatus_824 | 0.180 |
| Scymnus_ferrugatus_724 | Scymnus_rubromaculatus_825 | 0.180 |
| Scymnus_ferrugatus_729 | Scymnus_rubromaculatus_825 | 0.180 |
| Scymnus_ferrugatus_724 | Scymnus_rubromaculatus_826 | 0.180 |
| Scymnus_ferrugatus_729 | Scymnus_rubromaculatus_826 | 0.180 |
| Scymnus_ferrugatus_724 | Scymnus_rubromaculatus_827 | 0.180 |
| Scymnus_ferrugatus_729 | Scymnus_rubromaculatus_827 | 0.180 |
| Coccidula_scutellata_77 | Scymnus_suturalis_841 | 0.180 |

| | | |
|---|---|---|
| Coccidula_scutellata_77 | Scymnus_suturalis_842 | 0.180 |
| Coccidula_scutellata_77 | Scymnus_suturalis_844 | 0.180 |
| Coccidula_scutellata_77 | Scymnus_suturalis_846 | 0.180 |
| Coccidula_scutellata_77 | Scymnus_suturalis_847 | 0.180 |
| Coccidula_scutellata_77 | Scymnus_suturalis_848 | 0.180 |
| Scymnus_loewii_771 | Tytthaspis_sedecimpunctata_583 | 0.180 |
| Brumus_quadripustulatus_12 | Tytthaspis_sedecimpunctata_589 | 0.180 |
| Scymnus_loewii_773 | Tytthaspis_sedecimpunctata_589 | 0.180 |
| Myzia_oblongoguttata_506 | Tytthaspis_sedecimpunctata_593 | 0.180 |
| Scymnus_ningshanensis_788 | Tytthaspis_sedecimpunctata_593 | 0.180 |
| Scymnus_ningshanensis_791 | Tytthaspis_sedecimpunctata_593 | 0.180 |
| Scymnus_ningshanensis_792 | Tytthaspis_sedecimpunctata_593 | 0.180 |
| Scymnus_ningshanensis_793 | Tytthaspis_sedecimpunctata_593 | 0.180 |
| Scymnus_ningshanensis_794 | Tytthaspis_sedecimpunctata_593 | 0.180 |
| Scymnus_ningshanensis_795 | Tytthaspis_sedecimpunctata_593 | 0.180 |
| Coccidula_scutellata_77 | Scymnus_suturalis_832 | 0.180 |
| Coccidula_scutellata_78 | Scymnus_suturalis_833 | 0.180 |
| Coccidula_scutellata_79 | Scymnus_suturalis_833 | 0.180 |
| Coccidula_scutellata_77 | Scymnus_suturalis_835 | 0.180 |
| Coccidula_scutellata_77 | Scymnus_suturalis_836 | 0.180 |
| Coccidula_scutellata_81 | Scymnus_suturalis_838 | 0.180 |
| Coccidula_scutellata_82 | Scymnus_suturalis_838 | 0.180 |
| Coccidula_scutellata_77 | Scymnus_suturalis_839 | 0.180 |
| Coccidula_scutellata_81 | Scymnus_suturalis_840 | 0.180 |
| Coccidula_scutellata_82 | Scymnus_suturalis_840 | 0.180 |
| Scymnus_camptodromus_715 | Tytthaspis_sedecimpunctata_584 | 0.180 |
| Scymnus_frontalis_746 | Scymnus_sinuanodulus_829 | 0.180 |
| Scymnus_haemorrhoidalis_749 | Scymnus_sinuanodulus_829 | 0.180 |
| Coccidula_scutellata_80 | Scymnus_suturalis_841 | 0.180 |
| Coccidula_scutellata_80 | Scymnus_suturalis_842 | 0.180 |
| Chilocorus_stigma_43 | Scymnus_suturalis_843 | 0.180 |
| Coccidula_scutellata_78 | Scymnus_suturalis_843 | 0.180 |
| Coccidula_scutellata_79 | Scymnus_suturalis_843 | 0.180 |
| Coccidula_scutellata_80 | Scymnus_suturalis_844 | 0.180 |
| Chilocorus_stigma_43 | Scymnus_suturalis_845 | 0.180 |
| Coccidula_scutellata_78 | Scymnus_suturalis_845 | 0.180 |
| Coccidula_scutellata_79 | Scymnus_suturalis_845 | 0.180 |
| Coccidula_scutellata_80 | Scymnus_suturalis_846 | 0.180 |
| Coccidula_scutellata_80 | Scymnus_suturalis_847 | 0.180 |
| Coccidula_scutellata_80 | Scymnus_suturalis_848 | 0.180 |
| Myzia_oblongoguttata_504 | Tytthaspis_sedecimpunctata_580 | 0.180 |
| Myzia_oblongoguttata_505 | Tytthaspis_sedecimpunctata_580 | 0.180 |
| Myzia_oblongoguttata_507 | Tytthaspis_sedecimpunctata_580 | 0.180 |
| Myzia_oblongoguttata_508 | Tytthaspis_sedecimpunctata_580 | 0.180 |
| Myzia_oblongoguttata_509 | Tytthaspis_sedecimpunctata_580 | 0.180 |
| Myzia_oblongoguttata_510 | Tytthaspis_sedecimpunctata_580 | 0.180 |
| Myzia_oblongoguttata_511 | Tytthaspis_sedecimpunctata_580 | 0.180 |
| Myzia_oblongoguttata_512 | Tytthaspis_sedecimpunctata_580 | 0.180 |
| Myzia_oblongoguttata_513 | Tytthaspis_sedecimpunctata_580 | 0.180 |
| Myzia_oblongoguttata_514 | Tytthaspis_sedecimpunctata_580 | 0.180 |
| Myzia_oblongoguttata_516 | Tytthaspis_sedecimpunctata_580 | 0.180 |
| Myzia_pullata_517 | Tytthaspis_sedecimpunctata_584 | 0.180 |
| Brumus_quadripustulatus_15 | Tytthaspis_sedecimpunctata_586 | 0.180 |
| Brumus_quadripustulatus_16 | Tytthaspis_sedecimpunctata_586 | 0.180 |
| Brumus_quadripustulatus_6 | Tytthaspis_sedecimpunctata_586 | 0.180 |
| Brumus_quadripustulatus_8 | Tytthaspis_sedecimpunctata_586 | 0.180 |

| | | |
|---|---|---|
| Myzia_oblongoguttata_504 | Tytthaspis_sedecimpunctata_586 | 0.180 |
| Myzia_oblongoguttata_505 | Tytthaspis_sedecimpunctata_586 | 0.180 |
| Myzia_oblongoguttata_507 | Tytthaspis_sedecimpunctata_586 | 0.180 |
| Myzia_oblongoguttata_508 | Tytthaspis_sedecimpunctata_586 | 0.180 |
| Myzia_oblongoguttata_509 | Tytthaspis_sedecimpunctata_586 | 0.180 |
| Myzia_oblongoguttata_510 | Tytthaspis_sedecimpunctata_586 | 0.180 |
| Myzia_oblongoguttata_511 | Tytthaspis_sedecimpunctata_586 | 0.180 |
| Myzia_oblongoguttata_512 | Tytthaspis_sedecimpunctata_586 | 0.180 |
| Myzia_oblongoguttata_513 | Tytthaspis_sedecimpunctata_586 | 0.180 |
| Myzia_oblongoguttata_514 | Tytthaspis_sedecimpunctata_586 | 0.180 |
| Myzia_oblongoguttata_516 | Tytthaspis_sedecimpunctata_586 | 0.180 |
| Scymnus_loewii_771 | Tytthaspis_sedecimpunctata_586 | 0.180 |
| Brumus_quadripustulatus_12 | Tytthaspis_sedecimpunctata_588 | 0.180 |
| Myzia_oblongoguttata_504 | Tytthaspis_sedecimpunctata_588 | 0.180 |
| Myzia_oblongoguttata_505 | Tytthaspis_sedecimpunctata_588 | 0.180 |
| Myzia_oblongoguttata_507 | Tytthaspis_sedecimpunctata_588 | 0.180 |
| Myzia_oblongoguttata_508 | Tytthaspis_sedecimpunctata_588 | 0.180 |
| Myzia_oblongoguttata_509 | Tytthaspis_sedecimpunctata_588 | 0.180 |
| Myzia_oblongoguttata_510 | Tytthaspis_sedecimpunctata_588 | 0.180 |
| Myzia_oblongoguttata_511 | Tytthaspis_sedecimpunctata_588 | 0.180 |
| Myzia_oblongoguttata_512 | Tytthaspis_sedecimpunctata_588 | 0.180 |
| Myzia_oblongoguttata_513 | Tytthaspis_sedecimpunctata_588 | 0.180 |
| Myzia_oblongoguttata_514 | Tytthaspis_sedecimpunctata_588 | 0.180 |
| Myzia_oblongoguttata_516 | Tytthaspis_sedecimpunctata_588 | 0.180 |
| Scymnus_loewii_770 | Tytthaspis_sedecimpunctata_588 | 0.180 |
| Scymnus_loewii_773 | Tytthaspis_sedecimpunctata_588 | 0.180 |
| Brumus_quadripustulatus_12 | Tytthaspis_sedecimpunctata_591 | 0.180 |
| Coccidula_scutellata_80 | Scymnus_suturalis_832 | 0.180 |
| Chilocorus_nigritus_25 | Scymnus_suturalis_833 | 0.180 |
| Coccidula_scutellata_81 | Scymnus_suturalis_834 | 0.180 |
| Coccidula_scutellata_82 | Scymnus_suturalis_834 | 0.180 |
| Coccidula_scutellata_80 | Scymnus_suturalis_835 | 0.180 |
| Coccidula_scutellata_80 | Scymnus_suturalis_836 | 0.180 |
| Coccidula_scutellata_80 | Scymnus_suturalis_839 | 0.180 |
| Myzia_pullata_517 | Scymnus_suturalis_843 | 0.180 |
| Myzia_pullata_518 | Tytthaspis_sedecimpunctata_577 | 0.180 |
| Myzia_pullata_518 | Tytthaspis_sedecimpunctata_578 | 0.180 |
| Myzia_pullata_518 | Tytthaspis_sedecimpunctata_579 | 0.180 |
| Didion_punctatum_645 | Tytthaspis_sedecimpunctata_580 | 0.180 |
| Myzia_pullata_518 | Tytthaspis_sedecimpunctata_581 | 0.180 |
| Myzia_pullata_518 | Tytthaspis_sedecimpunctata_582 | 0.180 |
| Myzia_pullata_517 | Tytthaspis_sedecimpunctata_583 | 0.180 |
| Anisosticta_novemdecimpunctata_174 | Tytthaspis_sedecimpunctata_589 | 0.180 |
| Aphidecta_obliterata_184 | Tytthaspis_sedecimpunctata_589 | 0.180 |
| Myzia_pullata_517 | Tytthaspis_sedecimpunctata_589 | 0.180 |
| Myzia_pullata_518 | Tytthaspis_sedecimpunctata_590 | 0.180 |
| Myzia_pullata_518 | Tytthaspis_sedecimpunctata_592 | 0.180 |
| Myzia_pullata_518 | Tytthaspis_sedecimpunctata_593 | 0.180 |
| Myzia_pullata_518 | Tytthaspis_sedecimpunctata_594 | 0.180 |
| Myzia_pullata_518 | Tytthaspis_sedecimpunctata_595 | 0.180 |
| Chilocorus_stigma_43 | Scymnus_suturalis_833 | 0.180 |
| Didion_punctatum_646 | Tytthaspis_sedecimpunctata_580 | 0.180 |
| Scymnus_camptodromus_704 | Tytthaspis_sedecimpunctata_583 | 0.180 |
| Anisosticta_novemdecimpunctata_174 | Tytthaspis_sedecimpunctata_586 | 0.180 |
| Aphidecta_obliterata_184 | Tytthaspis_sedecimpunctata_586 | 0.180 |
| Propylaea_quatuordecimpunctata_547 | Tytthaspis_sedecimpunctata_586 | 0.180 |

| | | |
|---|---|---|
| Aphidecta_obliterata_175 | Tytthaspis_sedecimpunctata_587 | 0.180 |
| Aphidecta_obliterata_177 | Tytthaspis_sedecimpunctata_587 | 0.180 |
| Aphidecta_obliterata_180 | Tytthaspis_sedecimpunctata_587 | 0.180 |
| Aphidecta_obliterata_182 | Tytthaspis_sedecimpunctata_587 | 0.180 |
| Propylaea_quatuordecimpunctata_540 | Tytthaspis_sedecimpunctata_587 | 0.180 |
| Propylaea_quatuordecimpunctata_543 | Tytthaspis_sedecimpunctata_587 | 0.180 |
| Propylaea_quatuordecimpunctata_548 | Tytthaspis_sedecimpunctata_587 | 0.180 |
| Aphidecta_obliterata_181 | Tytthaspis_sedecimpunctata_588 | 0.180 |
| Propylaea_quatuordecimpunctata_547 | Tytthaspis_sedecimpunctata_588 | 0.180 |
| Aphidecta_obliterata_175 | Tytthaspis_sedecimpunctata_591 | 0.180 |
| Aphidecta_obliterata_177 | Tytthaspis_sedecimpunctata_591 | 0.180 |
| Aphidecta_obliterata_180 | Tytthaspis_sedecimpunctata_591 | 0.180 |
| Aphidecta_obliterata_182 | Tytthaspis_sedecimpunctata_591 | 0.180 |
| Propylaea_quatuordecimpunctata_545 | Tytthaspis_sedecimpunctata_591 | 0.180 |
| Aphidecta_obliterata_185 | Tytthaspis_sedecimpunctata_592 | 0.180 |
| Aphidecta_obliterata_186 | Tytthaspis_sedecimpunctata_592 | 0.180 |
| Coccidula_scutellata_78 | Scymnus_sinuanodulus_828 | 0.180 |
| Coccidula_scutellata_79 | Scymnus_sinuanodulus_828 | 0.180 |
| Myzia_pullata_517 | Scymnus_suturalis_833 | 0.180 |
| Anatis_ocellata_133 | Scymnus_suturalis_838 | 0.180 |
| Anatis_ocellata_137 | Scymnus_suturalis_838 | 0.180 |
| Anatis_ocellata_133 | Scymnus_suturalis_840 | 0.180 |
| Anatis_ocellata_137 | Scymnus_suturalis_840 | 0.180 |
| Scymnus_ferrugatus_724 | Scymnus_sinuanodulus_831 | 0.180 |
| Scymnus_ferrugatus_729 | Scymnus_sinuanodulus_831 | 0.180 |
| Anatis_ocellata_143 | Scymnus_suturalis_843 | 0.180 |
| Anatis_ocellata_143 | Scymnus_suturalis_845 | 0.180 |
| Aphidecta_obliterata_181 | Tytthaspis_sedecimpunctata_577 | 0.180 |
| Aphidecta_obliterata_181 | Tytthaspis_sedecimpunctata_578 | 0.180 |
| Aphidecta_obliterata_181 | Tytthaspis_sedecimpunctata_579 | 0.180 |
| Aphidecta_obliterata_185 | Tytthaspis_sedecimpunctata_580 | 0.180 |
| Aphidecta_obliterata_186 | Tytthaspis_sedecimpunctata_580 | 0.180 |
| Didion_punctatum_644 | Tytthaspis_sedecimpunctata_580 | 0.180 |
| Scymnus_camptodromus_704 | Tytthaspis_sedecimpunctata_580 | 0.180 |
| Aphidecta_obliterata_181 | Tytthaspis_sedecimpunctata_581 | 0.180 |
| Aphidecta_obliterata_181 | Tytthaspis_sedecimpunctata_582 | 0.180 |
| Anisosticta_novemdecimpunctata_174 | Tytthaspis_sedecimpunctata_583 | 0.180 |
| Aphidecta_obliterata_184 | Tytthaspis_sedecimpunctata_583 | 0.180 |
| Anisosticta_novemdecimpunctata_174 | Tytthaspis_sedecimpunctata_585 | 0.180 |
| Aphidecta_obliterata_184 | Tytthaspis_sedecimpunctata_585 | 0.180 |
| Coccinella_hieroglyphica_227 | Tytthaspis_sedecimpunctata_586 | 0.180 |
| Coccinella_trifasciata_272 | Tytthaspis_sedecimpunctata_586 | 0.180 |
| Coccinella_trifasciata_276 | Tytthaspis_sedecimpunctata_586 | 0.180 |
| Coccinella_trifasciata_280 | Tytthaspis_sedecimpunctata_587 | 0.180 |
| Coccinella_trifasciata_275 | Tytthaspis_sedecimpunctata_588 | 0.180 |
| Coccinella_trifasciata_277 | Tytthaspis_sedecimpunctata_588 | 0.180 |
| Coccinella_trifasciata_278 | Tytthaspis_sedecimpunctata_588 | 0.180 |
| Coccinella_trifasciata_279 | Tytthaspis_sedecimpunctata_588 | 0.180 |
| Coccinella_trifasciata_281 | Tytthaspis_sedecimpunctata_588 | 0.180 |
| Scymnus_caurinus_720 | Tytthaspis_sedecimpunctata_588 | 0.180 |
| Scymnus_caurinus_721 | Tytthaspis_sedecimpunctata_588 | 0.180 |
| Scymnus_caurinus_722 | Tytthaspis_sedecimpunctata_588 | 0.180 |
| Aphidecta_obliterata_181 | Tytthaspis_sedecimpunctata_590 | 0.180 |
| Adalia_bipunctata_105 | Tytthaspis_sedecimpunctata_591 | 0.180 |
| Aphidecta_obliterata_181 | Tytthaspis_sedecimpunctata_594 | 0.180 |
| Aphidecta_obliterata_181 | Tytthaspis_sedecimpunctata_595 | 0.180 |

| | | |
|---|---|---|
| Didion_punctatum_645 | Scymnus_sinuanodulus_830 | 0.180 |
| Anatis_ocellata_133 | Scymnus_suturalis_834 | 0.180 |
| Anatis_ocellata_137 | Scymnus_suturalis_834 | 0.180 |
| Anatis_ocellata_133 | Scymnus_suturalis_837 | 0.180 |
| Anatis_ocellata_137 | Scymnus_suturalis_837 | 0.180 |
| Hyperaspis_lateralis_656 | Scymnus_suturalis_838 | 0.180 |
| Platynaspis_luteorubra_52 | Scymnus_suturalis_838 | 0.180 |
| Platynaspis_luteorubra_56 | Scymnus_suturalis_838 | 0.180 |
| Platynaspis_luteorubra_60 | Scymnus_suturalis_838 | 0.180 |
| Hyperaspis_lateralis_656 | Scymnus_suturalis_840 | 0.180 |
| Platynaspis_luteorubra_52 | Scymnus_suturalis_840 | 0.180 |
| Platynaspis_luteorubra_56 | Scymnus_suturalis_840 | 0.180 |
| Platynaspis_luteorubra_60 | Scymnus_suturalis_840 | 0.180 |
| Hyperaspis_lateralis_654 | Scymnus_suturalis_841 | 0.180 |
| Hyperaspis_lateralis_657 | Scymnus_suturalis_841 | 0.180 |
| Hyperaspis_lateralis_654 | Scymnus_suturalis_842 | 0.180 |
| Hyperaspis_lateralis_657 | Scymnus_suturalis_842 | 0.180 |
| Hyperaspis_lateralis_654 | Scymnus_suturalis_844 | 0.180 |
| Hyperaspis_lateralis_657 | Scymnus_suturalis_844 | 0.180 |
| Hyperaspis_lateralis_654 | Scymnus_suturalis_846 | 0.180 |
| Hyperaspis_lateralis_657 | Scymnus_suturalis_846 | 0.180 |
| Hyperaspis_lateralis_654 | Scymnus_suturalis_847 | 0.180 |
| Hyperaspis_lateralis_657 | Scymnus_suturalis_847 | 0.180 |
| Hyperaspis_lateralis_654 | Scymnus_suturalis_848 | 0.180 |
| Hyperaspis_lateralis_657 | Scymnus_suturalis_848 | 0.180 |
| Anatis_labiculata_123 | Tytthaspis_sedecimpunctata_583 | 0.180 |
| Anatis_labiculata_124 | Tytthaspis_sedecimpunctata_583 | 0.180 |
| Anatis_labiculata_125 | Tytthaspis_sedecimpunctata_583 | 0.180 |
| Anatis_labiculata_127 | Tytthaspis_sedecimpunctata_583 | 0.180 |
| Scymnus_camptodromus_712 | Tytthaspis_sedecimpunctata_583 | 0.180 |
| Aphidecta_obliterata_175 | Tytthaspis_sedecimpunctata_584 | 0.180 |
| Aphidecta_obliterata_177 | Tytthaspis_sedecimpunctata_584 | 0.180 |
| Aphidecta_obliterata_180 | Tytthaspis_sedecimpunctata_584 | 0.180 |
| Aphidecta_obliterata_182 | Tytthaspis_sedecimpunctata_584 | 0.180 |
| Coccinella_undecimpunctata_282 | Tytthaspis_sedecimpunctata_584 | 0.180 |
| Coccinella_undecimpunctata_283 | Tytthaspis_sedecimpunctata_584 | 0.180 |
| Coccinella_undecimpunctata_284 | Tytthaspis_sedecimpunctata_584 | 0.180 |
| Anatis_labiculata_126 | Tytthaspis_sedecimpunctata_585 | 0.180 |
| Coccinella_undecimpunctata_282 | Tytthaspis_sedecimpunctata_586 | 0.180 |
| Coccinella_undecimpunctata_283 | Tytthaspis_sedecimpunctata_586 | 0.180 |
| Coccinella_undecimpunctata_284 | Tytthaspis_sedecimpunctata_586 | 0.180 |
| Coccinella_trifasciata_274 | Tytthaspis_sedecimpunctata_588 | 0.180 |
| Coccinella_undecimpunctata_285 | Tytthaspis_sedecimpunctata_588 | 0.180 |
| Coccinella_undecimpunctata_286 | Tytthaspis_sedecimpunctata_588 | 0.180 |
| Coccinella_undecimpunctata_288 | Tytthaspis_sedecimpunctata_588 | 0.180 |
| Coccinella_trifasciata_272 | Tytthaspis_sedecimpunctata_589 | 0.180 |
| Coccinella_trifasciata_276 | Tytthaspis_sedecimpunctata_589 | 0.180 |
| Coccinella_trifasciata_280 | Tytthaspis_sedecimpunctata_589 | 0.180 |
| Scymnus_auritus_701 | Tytthaspis_sedecimpunctata_589 | 0.180 |
| Adalia_bipunctata_101 | Tytthaspis_sedecimpunctata_591 | 0.180 |
| Adalia_bipunctata_104 | Tytthaspis_sedecimpunctata_591 | 0.180 |
| Coccinella_septempunctata_254 | Tytthaspis_sedecimpunctata_591 | 0.180 |
| Anatis_labiculata_123 | Tytthaspis_sedecimpunctata_592 | 0.180 |
| Anatis_labiculata_124 | Tytthaspis_sedecimpunctata_592 | 0.180 |
| Anatis_labiculata_125 | Tytthaspis_sedecimpunctata_592 | 0.180 |
| Anatis_labiculata_127 | Tytthaspis_sedecimpunctata_592 | 0.180 |

| | | |
|---|---|---|
| Coccinella_hieroglyphica_227 | Tytthaspis_sedecimpunctata_593 | 0.180 |
| Didion_punctatum_645 | Scymnus_sinuanodulus_828 | 0.180 |
| Hyperaspis_lateralis_654 | Scymnus_suturalis_832 | 0.180 |
| Hyperaspis_lateralis_657 | Scymnus_suturalis_832 | 0.180 |
| Scymnus_pallipediformis_812 | Scymnus_suturalis_833 | 0.180 |
| Hyperaspis_lateralis_656 | Scymnus_suturalis_834 | 0.180 |
| Platynaspis_luteorubra_52 | Scymnus_suturalis_834 | 0.180 |
| Platynaspis_luteorubra_56 | Scymnus_suturalis_834 | 0.180 |
| Platynaspis_luteorubra_60 | Scymnus_suturalis_834 | 0.180 |
| Hyperaspis_lateralis_654 | Scymnus_suturalis_835 | 0.180 |
| Hyperaspis_lateralis_657 | Scymnus_suturalis_835 | 0.180 |
| Hyperaspis_lateralis_654 | Scymnus_suturalis_836 | 0.180 |
| Hyperaspis_lateralis_657 | Scymnus_suturalis_836 | 0.180 |
| Hyperaspis_lateralis_654 | Scymnus_suturalis_837 | 0.180 |
| Hyperaspis_lateralis_657 | Scymnus_suturalis_837 | 0.180 |
| Platynaspis_luteorubra_54 | Scymnus_suturalis_838 | 0.180 |
| Platynaspis_luteorubra_57 | Scymnus_suturalis_838 | 0.180 |
| Platynaspis_luteorubra_59 | Scymnus_suturalis_838 | 0.180 |
| Hyperaspis_lateralis_654 | Scymnus_suturalis_839 | 0.180 |
| Hyperaspis_lateralis_657 | Scymnus_suturalis_839 | 0.180 |
| Platynaspis_luteorubra_54 | Scymnus_suturalis_840 | 0.180 |
| Platynaspis_luteorubra_57 | Scymnus_suturalis_840 | 0.180 |
| Platynaspis_luteorubra_59 | Scymnus_suturalis_840 | 0.180 |
| Coccinella_quinquepunctata_233 | Tytthaspis_sedecimpunctata_584 | 0.180 |
| Coccinella_quinquepunctata_234 | Tytthaspis_sedecimpunctata_584 | 0.180 |
| Hyperaspis_lateralis_655 | Scymnus_suturalis_841 | 0.180 |
| Hyperaspis_lateralis_655 | Scymnus_suturalis_842 | 0.180 |
| Hyperaspis_lateralis_655 | Scymnus_suturalis_844 | 0.180 |
| Hyperaspis_lateralis_655 | Scymnus_suturalis_846 | 0.180 |
| Hyperaspis_lateralis_655 | Scymnus_suturalis_847 | 0.180 |
| Hyperaspis_lateralis_655 | Scymnus_suturalis_848 | 0.180 |
| Hyperaspis_lateralis_655 | Stethorus_punctillum_850 | 0.180 |
| Adalia_bipunctata_98 | Tytthaspis_sedecimpunctata_577 | 0.180 |
| Adalia_bipunctata_98 | Tytthaspis_sedecimpunctata_578 | 0.180 |
| Adalia_bipunctata_98 | Tytthaspis_sedecimpunctata_579 | 0.180 |
| Anatis_labiculata_123 | Tytthaspis_sedecimpunctata_580 | 0.180 |
| Anatis_labiculata_124 | Tytthaspis_sedecimpunctata_580 | 0.180 |
| Anatis_labiculata_125 | Tytthaspis_sedecimpunctata_580 | 0.180 |
| Anatis_labiculata_127 | Tytthaspis_sedecimpunctata_580 | 0.180 |
| Scymnus_camptodromus_712 | Tytthaspis_sedecimpunctata_580 | 0.180 |
| Adalia_bipunctata_98 | Tytthaspis_sedecimpunctata_581 | 0.180 |
| Adalia_bipunctata_98 | Tytthaspis_sedecimpunctata_582 | 0.180 |
| Coccinella_undecimpunctata_282 | Tytthaspis_sedecimpunctata_583 | 0.180 |
| Coccinella_undecimpunctata_283 | Tytthaspis_sedecimpunctata_583 | 0.180 |
| Coccinella_undecimpunctata_284 | Tytthaspis_sedecimpunctata_583 | 0.180 |
| Olla_vnigrum_530 | Tytthaspis_sedecimpunctata_583 | 0.180 |
| Anatis_labiculata_123 | Tytthaspis_sedecimpunctata_587 | 0.180 |
| Anatis_labiculata_124 | Tytthaspis_sedecimpunctata_587 | 0.180 |
| Anatis_labiculata_125 | Tytthaspis_sedecimpunctata_587 | 0.180 |
| Anatis_labiculata_127 | Tytthaspis_sedecimpunctata_587 | 0.180 |
| Scymnus_camptodromus_712 | Tytthaspis_sedecimpunctata_587 | 0.180 |
| Scymnus_auritus_702 | Tytthaspis_sedecimpunctata_589 | 0.180 |
| Adalia_bipunctata_98 | Tytthaspis_sedecimpunctata_590 | 0.180 |
| Hippodamia_alpina_385 | Tytthaspis_sedecimpunctata_591 | 0.180 |
| Hippodamia_alpina_386 | Tytthaspis_sedecimpunctata_591 | 0.180 |
| Hippodamia_alpina_387 | Tytthaspis_sedecimpunctata_591 | 0.180 |

| | | |
|---|---|---|
| Psyllobora_renifer_550 | Tytthaspis_sedecimpunctata_593 | 0.180 |
| Adalia_bipunctata_98 | Tytthaspis_sedecimpunctata_594 | 0.180 |
| Adalia_bipunctata_98 | Tytthaspis_sedecimpunctata_595 | 0.180 |
| Chilocorus_nigritus_27 | Scymnus_rubromaculatus_822 | 0.180 |
| Chilocorus_nigritus_27 | Scymnus_rubromaculatus_823 | 0.180 |
| Chilocorus_nigritus_27 | Scymnus_rubromaculatus_824 | 0.180 |
| Chilocorus_nigritus_27 | Scymnus_rubromaculatus_825 | 0.180 |
| Chilocorus_nigritus_27 | Scymnus_rubromaculatus_826 | 0.180 |
| Chilocorus_nigritus_27 | Scymnus_rubromaculatus_827 | 0.180 |
| Hyperaspis_lateralis_655 | Scymnus_suturalis_832 | 0.180 |
| Platynaspis_luteorubra_54 | Scymnus_suturalis_834 | 0.180 |
| Platynaspis_luteorubra_57 | Scymnus_suturalis_834 | 0.180 |
| Platynaspis_luteorubra_59 | Scymnus_suturalis_834 | 0.180 |
| Hyperaspis_lateralis_655 | Scymnus_suturalis_835 | 0.180 |
| Hyperaspis_lateralis_655 | Scymnus_suturalis_836 | 0.180 |
| Hyperaspis_lateralis_655 | Scymnus_suturalis_837 | 0.180 |
| Hyperaspis_lateralis_655 | Scymnus_suturalis_839 | 0.180 |
| Coccinella_quinquepunctata_233 | Tytthaspis_sedecimpunctata_577 | 0.180 |
| Coccinella_quinquepunctata_234 | Tytthaspis_sedecimpunctata_577 | 0.180 |
| Coccinella_quinquepunctata_233 | Tytthaspis_sedecimpunctata_578 | 0.180 |
| Coccinella_quinquepunctata_234 | Tytthaspis_sedecimpunctata_578 | 0.180 |
| Coccinella_quinquepunctata_233 | Tytthaspis_sedecimpunctata_579 | 0.180 |
| Coccinella_quinquepunctata_234 | Tytthaspis_sedecimpunctata_579 | 0.180 |
| Coccinella_quinquepunctata_233 | Tytthaspis_sedecimpunctata_581 | 0.180 |
| Coccinella_quinquepunctata_234 | Tytthaspis_sedecimpunctata_581 | 0.180 |
| Coccinella_quinquepunctata_233 | Tytthaspis_sedecimpunctata_582 | 0.180 |
| Coccinella_quinquepunctata_234 | Tytthaspis_sedecimpunctata_582 | 0.180 |
| Coccinella_quinquepunctata_233 | Tytthaspis_sedecimpunctata_585 | 0.180 |
| Coccinella_quinquepunctata_233 | Tytthaspis_sedecimpunctata_590 | 0.180 |
| Coccinella_quinquepunctata_234 | Tytthaspis_sedecimpunctata_590 | 0.180 |
| Coccinella_quinquepunctata_233 | Tytthaspis_sedecimpunctata_592 | 0.180 |
| Coccinella_quinquepunctata_234 | Tytthaspis_sedecimpunctata_592 | 0.180 |
| Coccinella_quinquepunctata_233 | Tytthaspis_sedecimpunctata_593 | 0.180 |
| Coccinella_quinquepunctata_234 | Tytthaspis_sedecimpunctata_593 | 0.180 |
| Coccinella_quinquepunctata_233 | Tytthaspis_sedecimpunctata_594 | 0.180 |
| Coccinella_quinquepunctata_234 | Tytthaspis_sedecimpunctata_594 | 0.180 |
| Coccinella_quinquepunctata_233 | Tytthaspis_sedecimpunctata_595 | 0.180 |
| Coccinella_quinquepunctata_234 | Tytthaspis_sedecimpunctata_595 | 0.180 |
| Olla_vnigrum_525 | Tytthaspis_sedecimpunctata_577 | 0.180 |
| Olla_vnigrum_529 | Tytthaspis_sedecimpunctata_577 | 0.180 |
| Olla_vnigrum_531 | Tytthaspis_sedecimpunctata_577 | 0.180 |
| Olla_vnigrum_525 | Tytthaspis_sedecimpunctata_578 | 0.180 |
| Olla_vnigrum_529 | Tytthaspis_sedecimpunctata_578 | 0.180 |
| Olla_vnigrum_531 | Tytthaspis_sedecimpunctata_578 | 0.180 |
| Olla_vnigrum_525 | Tytthaspis_sedecimpunctata_579 | 0.180 |
| Olla_vnigrum_529 | Tytthaspis_sedecimpunctata_579 | 0.180 |
| Olla_vnigrum_531 | Tytthaspis_sedecimpunctata_579 | 0.180 |
| Coccinella_undecimpunctata_282 | Tytthaspis_sedecimpunctata_580 | 0.180 |
| Coccinella_undecimpunctata_283 | Tytthaspis_sedecimpunctata_580 | 0.180 |
| Coccinella_undecimpunctata_284 | Tytthaspis_sedecimpunctata_580 | 0.180 |
| Olla_vnigrum_525 | Tytthaspis_sedecimpunctata_581 | 0.180 |
| Olla_vnigrum_529 | Tytthaspis_sedecimpunctata_581 | 0.180 |
| Olla_vnigrum_531 | Tytthaspis_sedecimpunctata_581 | 0.180 |
| Olla_vnigrum_525 | Tytthaspis_sedecimpunctata_582 | 0.180 |
| Olla_vnigrum_529 | Tytthaspis_sedecimpunctata_582 | 0.180 |
| Olla_vnigrum_531 | Tytthaspis_sedecimpunctata_582 | 0.180 |

| | | |
|---|---|---|
| Coccinella_quinquepunctata_237 | Tytthaspis_sedecimpunctata_587 | 0.180 |
| Olla_vnigrum_525 | Tytthaspis_sedecimpunctata_590 | 0.180 |
| Olla_vnigrum_529 | Tytthaspis_sedecimpunctata_590 | 0.180 |
| Olla_vnigrum_531 | Tytthaspis_sedecimpunctata_590 | 0.180 |
| Olla_vnigrum_525 | Tytthaspis_sedecimpunctata_592 | 0.180 |
| Olla_vnigrum_529 | Tytthaspis_sedecimpunctata_592 | 0.180 |
| Olla_vnigrum_531 | Tytthaspis_sedecimpunctata_592 | 0.180 |
| Scymnus_auritus_703 | Tytthaspis_sedecimpunctata_593 | 0.180 |
| Olla_vnigrum_525 | Tytthaspis_sedecimpunctata_594 | 0.180 |
| Olla_vnigrum_529 | Tytthaspis_sedecimpunctata_594 | 0.180 |
| Olla_vnigrum_531 | Tytthaspis_sedecimpunctata_594 | 0.180 |
| Olla_vnigrum_525 | Tytthaspis_sedecimpunctata_595 | 0.180 |
| Olla_vnigrum_529 | Tytthaspis_sedecimpunctata_595 | 0.180 |
| Olla_vnigrum_531 | Tytthaspis_sedecimpunctata_595 | 0.180 |
| Brumus_quadripustulatus_11 | Scymnus_rubromaculatus_822 | 0.180 |
| Brumus_quadripustulatus_13 | Scymnus_rubromaculatus_822 | 0.180 |
| Brumus_quadripustulatus_14 | Scymnus_rubromaculatus_822 | 0.180 |
| Brumus_quadripustulatus_3 | Scymnus_rubromaculatus_822 | 0.180 |
| Brumus_quadripustulatus_4 | Scymnus_rubromaculatus_822 | 0.180 |
| Brumus_quadripustulatus_7 | Scymnus_rubromaculatus_822 | 0.180 |
| Brumus_quadripustulatus_11 | Scymnus_rubromaculatus_823 | 0.180 |
| Brumus_quadripustulatus_13 | Scymnus_rubromaculatus_823 | 0.180 |
| Brumus_quadripustulatus_14 | Scymnus_rubromaculatus_823 | 0.180 |
| Brumus_quadripustulatus_3 | Scymnus_rubromaculatus_823 | 0.180 |
| Brumus_quadripustulatus_4 | Scymnus_rubromaculatus_823 | 0.180 |
| Brumus_quadripustulatus_7 | Scymnus_rubromaculatus_823 | 0.180 |
| Brumus_quadripustulatus_11 | Scymnus_rubromaculatus_824 | 0.180 |
| Brumus_quadripustulatus_13 | Scymnus_rubromaculatus_824 | 0.180 |
| Brumus_quadripustulatus_14 | Scymnus_rubromaculatus_824 | 0.180 |
| Brumus_quadripustulatus_3 | Scymnus_rubromaculatus_824 | 0.180 |
| Brumus_quadripustulatus_4 | Scymnus_rubromaculatus_824 | 0.180 |
| Brumus_quadripustulatus_7 | Scymnus_rubromaculatus_824 | 0.180 |
| Brumus_quadripustulatus_11 | Scymnus_rubromaculatus_825 | 0.180 |
| Brumus_quadripustulatus_13 | Scymnus_rubromaculatus_825 | 0.180 |
| Brumus_quadripustulatus_14 | Scymnus_rubromaculatus_825 | 0.180 |
| Brumus_quadripustulatus_3 | Scymnus_rubromaculatus_825 | 0.180 |
| Brumus_quadripustulatus_4 | Scymnus_rubromaculatus_825 | 0.180 |
| Brumus_quadripustulatus_7 | Scymnus_rubromaculatus_825 | 0.180 |
| Brumus_quadripustulatus_11 | Scymnus_rubromaculatus_826 | 0.180 |
| Brumus_quadripustulatus_13 | Scymnus_rubromaculatus_826 | 0.180 |
| Brumus_quadripustulatus_14 | Scymnus_rubromaculatus_826 | 0.180 |
| Brumus_quadripustulatus_3 | Scymnus_rubromaculatus_826 | 0.180 |
| Brumus_quadripustulatus_4 | Scymnus_rubromaculatus_826 | 0.180 |
| Brumus_quadripustulatus_7 | Scymnus_rubromaculatus_826 | 0.180 |
| Brumus_quadripustulatus_11 | Scymnus_rubromaculatus_827 | 0.180 |
| Brumus_quadripustulatus_13 | Scymnus_rubromaculatus_827 | 0.180 |
| Brumus_quadripustulatus_14 | Scymnus_rubromaculatus_827 | 0.180 |
| Brumus_quadripustulatus_3 | Scymnus_rubromaculatus_827 | 0.180 |
| Brumus_quadripustulatus_4 | Scymnus_rubromaculatus_827 | 0.180 |
| Brumus_quadripustulatus_7 | Scymnus_rubromaculatus_827 | 0.180 |
| Chilocorus_nigritus_26 | Scymnus_sinuanodulus_830 | 0.180 |
| Coccidula_scutellata_84 | Scymnus_suturalis_837 | 0.180 |
| Chilocorus_nigritus_30 | Scymnus_suturalis_838 | 0.180 |
| Chilocorus_nigritus_30 | Scymnus_suturalis_840 | 0.180 |
| Coccinella_quinquepunctata_233 | Tytthaspis_sedecimpunctata_580 | 0.180 |
| Coccinella_quinquepunctata_234 | Tytthaspis_sedecimpunctata_580 | 0.180 |

| | | |
|---|---|---|
| Chilocorus_nigritus_30 | Scymnus_suturalis_834 | 0.180 |
| Chilocorus_nigritus_30 | Scymnus_suturalis_837 | 0.180 |
| Scymnus_nubilus_799 | Subcoccinella_vigintiquatuorpunctata_624 | 0.180 |
| Scymnus_auritus_702 | Tytthaspis_sedecimpunctata_583 | 0.180 |
| Olla_vnigrum_525 | Tytthaspis_sedecimpunctata_584 | 0.180 |
| Olla_vnigrum_529 | Tytthaspis_sedecimpunctata_584 | 0.180 |
| Olla_vnigrum_531 | Tytthaspis_sedecimpunctata_584 | 0.180 |
| Scymnus_auritus_702 | Tytthaspis_sedecimpunctata_585 | 0.180 |
| Hippodamia_notata_422 | Tytthaspis_sedecimpunctata_589 | 0.180 |
| Hippodamia_notata_423 | Tytthaspis_sedecimpunctata_589 | 0.180 |
| Hippodamia_notata_424 | Tytthaspis_sedecimpunctata_589 | 0.180 |
| Hippodamia_notata_426 | Tytthaspis_sedecimpunctata_589 | 0.180 |
| Hippodamia_notata_427 | Tytthaspis_sedecimpunctata_589 | 0.180 |
| Scymnus_auritus_702 | Tytthaspis_sedecimpunctata_592 | 0.180 |
| Brumus_quadripustulatus_9 | Scymnus_rubromaculatus_822 | 0.180 |
| Brumus_quadripustulatus_9 | Scymnus_rubromaculatus_823 | 0.180 |
| Brumus_quadripustulatus_9 | Scymnus_rubromaculatus_824 | 0.180 |
| Brumus_quadripustulatus_9 | Scymnus_rubromaculatus_825 | 0.180 |
| Brumus_quadripustulatus_9 | Scymnus_rubromaculatus_826 | 0.180 |
| Brumus_quadripustulatus_9 | Scymnus_rubromaculatus_827 | 0.180 |
| Brumoides_suturalis_1 | Scymnus_suturalis_843 | 0.180 |
| Brumoides_suturalis_1 | Scymnus_suturalis_845 | 0.180 |
| Rhyzobius_litura_97 | Subcoccinella_vigintiquatuorpunctata_630 | 0.180 |
| Scymnus_auritus_702 | Tytthaspis_sedecimpunctata_580 | 0.180 |
| Scymnus_auritus_700 | Tytthaspis_sedecimpunctata_584 | 0.180 |
| Hippodamia_notata_422 | Tytthaspis_sedecimpunctata_586 | 0.180 |
| Hippodamia_notata_423 | Tytthaspis_sedecimpunctata_586 | 0.180 |
| Hippodamia_notata_424 | Tytthaspis_sedecimpunctata_586 | 0.180 |
| Hippodamia_notata_426 | Tytthaspis_sedecimpunctata_586 | 0.180 |
| Hippodamia_notata_427 | Tytthaspis_sedecimpunctata_586 | 0.180 |
| Hippodamia_notata_422 | Tytthaspis_sedecimpunctata_588 | 0.180 |
| Hippodamia_notata_423 | Tytthaspis_sedecimpunctata_588 | 0.180 |
| Hippodamia_notata_424 | Tytthaspis_sedecimpunctata_588 | 0.180 |
| Hippodamia_notata_426 | Tytthaspis_sedecimpunctata_588 | 0.180 |
| Hippodamia_notata_427 | Tytthaspis_sedecimpunctata_588 | 0.180 |
| Hippodamia_notata_415 | Tytthaspis_sedecimpunctata_589 | 0.180 |
| Scymnus_auritus_700 | Tytthaspis_sedecimpunctata_591 | 0.180 |
| Scymnus_pallipediformis_812 | Scymnus_sinuanodulus_830 | 0.180 |
| Brumoides_suturalis_1 | Scymnus_suturalis_834 | 0.180 |
| Brumoides_suturalis_2 | Scymnus_suturalis_841 | 0.180 |
| Brumoides_suturalis_2 | Scymnus_suturalis_842 | 0.180 |
| Scymnus_nubilus_799 | Scymnus_suturalis_843 | 0.180 |
| Brumoides_suturalis_2 | Scymnus_suturalis_844 | 0.180 |
| Scymnus_nubilus_799 | Scymnus_suturalis_845 | 0.180 |
| Brumoides_suturalis_2 | Scymnus_suturalis_846 | 0.180 |
| Brumoides_suturalis_2 | Scymnus_suturalis_847 | 0.180 |
| Brumoides_suturalis_2 | Scymnus_suturalis_848 | 0.180 |
| Hippodamia_undecimnotata_460 | Tytthaspis_sedecimpunctata_580 | 0.180 |
| Hippodamia_undecimnotata_461 | Tytthaspis_sedecimpunctata_580 | 0.180 |
| Hippodamia_undecimnotata_462 | Tytthaspis_sedecimpunctata_580 | 0.180 |
| Hippodamia_undecimnotata_463 | Tytthaspis_sedecimpunctata_580 | 0.180 |
| Hippodamia_undecimnotata_464 | Tytthaspis_sedecimpunctata_580 | 0.180 |
| Hippodamia_undecimnotata_465 | Tytthaspis_sedecimpunctata_580 | 0.180 |
| Hippodamia_undecimnotata_466 | Tytthaspis_sedecimpunctata_580 | 0.180 |
| Cycloneda_sanguinea_342 | Tytthaspis_sedecimpunctata_583 | 0.180 |
| Neoharmonia_venusta_520 | Tytthaspis_sedecimpunctata_586 | 0.180 |

| | | |
|---|---|---|
| Hippodamia_notata_415 | Tytthaspis_sedecimpunctata_587 | 0.180 |
| Hippodamia_notata_413 | Tytthaspis_sedecimpunctata_589 | 0.180 |
| Hippodamia_notata_418 | Tytthaspis_sedecimpunctata_589 | 0.180 |
| Cycloneda_sanguinea_340 | Tytthaspis_sedecimpunctata_593 | 0.180 |
| Cycloneda_sanguinea_341 | Tytthaspis_sedecimpunctata_593 | 0.180 |
| Cycloneda_sanguinea_343 | Tytthaspis_sedecimpunctata_593 | 0.180 |
| Cycloneda_sanguinea_344 | Tytthaspis_sedecimpunctata_593 | 0.180 |
| Brumoides_suturalis_2 | Scymnus_suturalis_832 | 0.180 |
| Scymnus_nubilus_799 | Scymnus_suturalis_834 | 0.180 |
| Brumoides_suturalis_2 | Scymnus_suturalis_835 | 0.180 |
| Brumoides_suturalis_2 | Scymnus_suturalis_836 | 0.180 |
| Scymnus_nubilus_799 | Scymnus_suturalis_837 | 0.180 |
| Brumoides_suturalis_2 | Scymnus_suturalis_839 | 0.180 |
| Chilocorus_nigritus_28 | Scymnus_rubromaculatus_822 | 0.180 |
| Chilocorus_nigritus_28 | Scymnus_rubromaculatus_823 | 0.180 |
| Chilocorus_nigritus_28 | Scymnus_rubromaculatus_824 | 0.180 |
| Chilocorus_nigritus_28 | Scymnus_rubromaculatus_825 | 0.180 |
| Chilocorus_nigritus_28 | Scymnus_rubromaculatus_826 | 0.180 |
| Chilocorus_nigritus_28 | Scymnus_rubromaculatus_827 | 0.180 |
| Neoharmonia_venusta_521 | Tytthaspis_sedecimpunctata_586 | 0.180 |
| Cycloneda_sanguinea_342 | Tytthaspis_sedecimpunctata_587 | 0.180 |
| Hippodamia_notata_413 | Tytthaspis_sedecimpunctata_587 | 0.180 |
| Hippodamia_notata_418 | Tytthaspis_sedecimpunctata_587 | 0.180 |
| Hippodamia_notata_419 | Tytthaspis_sedecimpunctata_587 | 0.180 |
| Hippodamia_notata_431 | Tytthaspis_sedecimpunctata_587 | 0.180 |
| Cycloneda_sanguinea_340 | Tytthaspis_sedecimpunctata_591 | 0.180 |
| Cycloneda_sanguinea_341 | Tytthaspis_sedecimpunctata_591 | 0.180 |
| Cycloneda_sanguinea_343 | Tytthaspis_sedecimpunctata_591 | 0.180 |
| Cycloneda_sanguinea_344 | Tytthaspis_sedecimpunctata_591 | 0.180 |
| Coccinella_transversoguttata_265 | Vibidia_duodecimguttata_596 | 0.180 |
| Coccinella_transversoguttata_266 | Vibidia_duodecimguttata_596 | 0.180 |
| Aphidecta_obliterata_175 | Vibidia_duodecimguttata_597 | 0.180 |
| Aphidecta_obliterata_177 | Vibidia_duodecimguttata_597 | 0.180 |
| Aphidecta_obliterata_180 | Vibidia_duodecimguttata_597 | 0.180 |
| Aphidecta_obliterata_181 | Vibidia_duodecimguttata_597 | 0.180 |
| Aphidecta_obliterata_182 | Vibidia_duodecimguttata_597 | 0.180 |
| Aphidecta_obliterata_184 | Vibidia_duodecimguttata_597 | 0.180 |
| Aphidecta_obliterata_175 | Vibidia_duodecimguttata_600 | 0.180 |
| Aphidecta_obliterata_177 | Vibidia_duodecimguttata_600 | 0.180 |
| Aphidecta_obliterata_180 | Vibidia_duodecimguttata_600 | 0.180 |
| Aphidecta_obliterata_181 | Vibidia_duodecimguttata_600 | 0.180 |
| Aphidecta_obliterata_182 | Vibidia_duodecimguttata_600 | 0.180 |
| Aphidecta_obliterata_184 | Vibidia_duodecimguttata_600 | 0.180 |
| Scymnus_nubilus_799 | Scymnus_suturalis_840 | 0.180 |
| Hyperaspis_campestris_651 | Subcoccinella_vigintiquatuorpunctata_620 | 0.180 |
| Hyperaspis_campestris_652 | Subcoccinella_vigintiquatuorpunctata_620 | 0.180 |
| Hyperaspis_campestris_653 | Subcoccinella_vigintiquatuorpunctata_620 | 0.180 |
| Hyperaspis_campestris_651 | Subcoccinella_vigintiquatuorpunctata_621 | 0.180 |
| Hyperaspis_campestris_652 | Subcoccinella_vigintiquatuorpunctata_621 | 0.180 |
| Hyperaspis_campestris_653 | Subcoccinella_vigintiquatuorpunctata_621 | 0.180 |
| Hyperaspis_campestris_651 | Subcoccinella_vigintiquatuorpunctata_624 | 0.180 |
| Hyperaspis_campestris_652 | Subcoccinella_vigintiquatuorpunctata_624 | 0.180 |
| Hyperaspis_campestris_653 | Subcoccinella_vigintiquatuorpunctata_624 | 0.180 |
| Hyperaspis_proba_661 | Subcoccinella_vigintiquatuorpunctata_624 | 0.180 |
| Hyperaspis_reppensis_665 | Subcoccinella_vigintiquatuorpunctata_625 | 0.180 |
| Hyperaspis_proba_661 | Subcoccinella_vigintiquatuorpunctata_627 | 0.180 |

| | | |
|---|---|---|
| Hyperaspis_campestris_651 | Subcoccinella_vigintiquatuorpunctata_628 | 0.180 |
| Hyperaspis_campestris_652 | Subcoccinella_vigintiquatuorpunctata_628 | 0.180 |
| Hyperaspis_campestris_653 | Subcoccinella_vigintiquatuorpunctata_628 | 0.180 |
| Hyperaspis_campestris_651 | Subcoccinella_vigintiquatuorpunctata_629 | 0.180 |
| Hyperaspis_campestris_652 | Subcoccinella_vigintiquatuorpunctata_629 | 0.180 |
| Hyperaspis_campestris_653 | Subcoccinella_vigintiquatuorpunctata_629 | 0.180 |
| Hyperaspis_campestris_651 | Subcoccinella_vigintiquatuorpunctata_631 | 0.180 |
| Hyperaspis_campestris_652 | Subcoccinella_vigintiquatuorpunctata_631 | 0.180 |
| Hyperaspis_campestris_653 | Subcoccinella_vigintiquatuorpunctata_631 | 0.180 |
| Neoharmonia_venusta_521 | Tytthaspis_sedecimpunctata_593 | 0.180 |
| Aphidecta_obliterata_179 | Vibidia_duodecimguttata_596 | 0.180 |
| Aphidecta_obliterata_179 | Vibidia_duodecimguttata_598 | 0.180 |
| Aphidecta_obliterata_179 | Vibidia_duodecimguttata_603 | 0.180 |
| Scymnus_rubromaculatus_814 | Scymnus_sinuanodulus_828 | 0.180 |
| Scymnus_frontalis_736 | Scymnus_sinuanodulus_831 | 0.180 |
| Scymnus_frontalis_737 | Scymnus_sinuanodulus_831 | 0.180 |
| Scymnus_frontalis_738 | Scymnus_sinuanodulus_831 | 0.180 |
| Scymnus_frontalis_739 | Scymnus_sinuanodulus_831 | 0.180 |
| Scymnus_frontalis_740 | Scymnus_sinuanodulus_831 | 0.180 |
| Scymnus_frontalis_741 | Scymnus_sinuanodulus_831 | 0.180 |
| Scymnus_frontalis_743 | Scymnus_sinuanodulus_831 | 0.180 |
| Scymnus_frontalis_744 | Scymnus_sinuanodulus_831 | 0.180 |
| Chilocorus_bipustulatus_21 | Scymnus_sinuanodulus_831 | 0.180 |
| Hyperaspis_campestris_651 | Subcoccinella_vigintiquatuorpunctata_623 | 0.180 |
| Hyperaspis_campestris_652 | Subcoccinella_vigintiquatuorpunctata_623 | 0.180 |
| Hyperaspis_campestris_653 | Subcoccinella_vigintiquatuorpunctata_623 | 0.180 |
| Hyperaspis_oregona_658 | Scymnus_sinuanodulus_828 | 0.180 |
| Hyperaspis_reppensis_666 | Scymnus_sinuanodulus_830 | 0.180 |
| Scymnus_haemorrhoidalis_747 | Scymnus_sinuanodulus_830 | 0.180 |
| Scymnus_haemorrhoidalis_748 | Scymnus_sinuanodulus_830 | 0.180 |
| Scymnus_haemorrhoidalis_755 | Scymnus_sinuanodulus_830 | 0.180 |
| Hyperaspis_binotata_649 | Scymnus_sinuanodulus_831 | 0.180 |
| Scymnus_camptodromus_717 | Tytthaspis_sedecimpunctata_584 | 0.181 |
| Myzia_pullata_517 | Vibidia_duodecimguttata_596 | 0.181 |
| Hyperaspis_reppensis_666 | Scymnus_sinuanodulus_828 | 0.181 |
| Scymnus_haemorrhoidalis_747 | Scymnus_sinuanodulus_828 | 0.181 |
| Scymnus_haemorrhoidalis_748 | Scymnus_sinuanodulus_828 | 0.181 |
| Scymnus_haemorrhoidalis_755 | Scymnus_sinuanodulus_828 | 0.181 |
| Scymnus_haemorrhoidalis_749 | Subcoccinella_vigintiquatuorpunctata_619 | 0.181 |
| Scymnus_haemorrhoidalis_750 | Subcoccinella_vigintiquatuorpunctata_619 | 0.181 |
| Scymnus_camptodromus_711 | Tytthaspis_sedecimpunctata_584 | 0.181 |
| Hyperaspis_proba_661 | Subcoccinella_vigintiquatuorpunctata_619 | 0.181 |
| Hyperaspis_proba_660 | Subcoccinella_vigintiquatuorpunctata_620 | 0.181 |
| Hyperaspis_proba_660 | Subcoccinella_vigintiquatuorpunctata_621 | 0.181 |
| Hyperaspis_proba_660 | Subcoccinella_vigintiquatuorpunctata_628 | 0.181 |
| Hyperaspis_proba_660 | Subcoccinella_vigintiquatuorpunctata_629 | 0.181 |
| Hyperaspis_proba_660 | Subcoccinella_vigintiquatuorpunctata_631 | 0.181 |
| Psyllobora_vigintiduopunctata_556 | Vibidia_duodecimguttata_597 | 0.181 |
| Psyllobora_vigintiduopunctata_556 | Vibidia_duodecimguttata_600 | 0.181 |
| Scymnus_haemorrhoidalis_753 | Scymnus_sinuanodulus_830 | 0.181 |
| Scymnus_mitior_780 | Scymnus_suturalis_838 | 0.181 |
| Scymnus_mitior_780 | Scymnus_suturalis_840 | 0.181 |
| Scymnus_mitior_780 | Scymnus_suturalis_843 | 0.181 |
| Calvia_decemguttata_187 | Tytthaspis_sedecimpunctata_591 | 0.181 |
| Calvia_decemguttata_188 | Tytthaspis_sedecimpunctata_591 | 0.181 |
| Calvia_decemguttata_189 | Tytthaspis_sedecimpunctata_591 | 0.181 |

| | | |
|---|---|---|
| Calvia_decemguttata_190 | Tytthaspis_sedecimpunctata_591 | 0.181 |
| Calvia_decemguttata_191 | Tytthaspis_sedecimpunctata_591 | 0.181 |
| Calvia_decemguttata_192 | Tytthaspis_sedecimpunctata_591 | 0.181 |
| Calvia_decemguttata_196 | Tytthaspis_sedecimpunctata_591 | 0.181 |
| Calvia_decemguttata_197 | Tytthaspis_sedecimpunctata_591 | 0.181 |
| Calvia_decemguttata_198 | Tytthaspis_sedecimpunctata_591 | 0.181 |
| Psyllobora_vigintiduopunctata_561 | Vibidia_duodecimguttata_597 | 0.181 |
| Psyllobora_vigintiduopunctata_562 | Vibidia_duodecimguttata_597 | 0.181 |
| Psyllobora_vigintiduopunctata_565 | Vibidia_duodecimguttata_597 | 0.181 |
| Psyllobora_vigintiduopunctata_567 | Vibidia_duodecimguttata_597 | 0.181 |
| Psyllobora_vigintiduopunctata_556 | Vibidia_duodecimguttata_598 | 0.181 |
| Psyllobora_vigintiduopunctata_561 | Vibidia_duodecimguttata_600 | 0.181 |
| Psyllobora_vigintiduopunctata_562 | Vibidia_duodecimguttata_600 | 0.181 |
| Psyllobora_vigintiduopunctata_565 | Vibidia_duodecimguttata_600 | 0.181 |
| Psyllobora_vigintiduopunctata_567 | Vibidia_duodecimguttata_600 | 0.181 |
| Psyllobora_vigintiduopunctata_556 | Vibidia_duodecimguttata_603 | 0.181 |
| Scymnus_haemorrhoidalis_753 | Scymnus_sinuanodulus_828 | 0.181 |
| Scymnus_camptodromus_706 | Tytthaspis_sedecimpunctata_591 | 0.181 |
| Hyperaspis_oregona_659 | Scymnus_sinuanodulus_831 | 0.181 |
| Calvia_decemguttata_193 | Vibidia_duodecimguttata_596 | 0.181 |
| Calvia_decemguttata_194 | Vibidia_duodecimguttata_596 | 0.181 |
| Anatis_ocellata_133 | Vibidia_duodecimguttata_597 | 0.181 |
| Anatis_ocellata_137 | Vibidia_duodecimguttata_597 | 0.181 |
| Anatis_ocellata_143 | Vibidia_duodecimguttata_597 | 0.181 |
| Calvia_decemguttata_193 | Vibidia_duodecimguttata_598 | 0.181 |
| Calvia_decemguttata_194 | Vibidia_duodecimguttata_598 | 0.181 |
| Psyllobora_vigintiduopunctata_561 | Vibidia_duodecimguttata_598 | 0.181 |
| Psyllobora_vigintiduopunctata_562 | Vibidia_duodecimguttata_598 | 0.181 |
| Psyllobora_vigintiduopunctata_565 | Vibidia_duodecimguttata_598 | 0.181 |
| Psyllobora_vigintiduopunctata_567 | Vibidia_duodecimguttata_598 | 0.181 |
| Psyllobora_vigintiduopunctata_560 | Vibidia_duodecimguttata_599 | 0.181 |
| Psyllobora_vigintiduopunctata_564 | Vibidia_duodecimguttata_599 | 0.181 |
| Psyllobora_vigintiduopunctata_570 | Vibidia_duodecimguttata_599 | 0.181 |
| Psyllobora_vigintiduopunctata_572 | Vibidia_duodecimguttata_599 | 0.181 |
| Anatis_ocellata_133 | Vibidia_duodecimguttata_600 | 0.181 |
| Anatis_ocellata_137 | Vibidia_duodecimguttata_600 | 0.181 |
| Anatis_ocellata_143 | Vibidia_duodecimguttata_600 | 0.181 |
| Psyllobora_vigintiduopunctata_560 | Vibidia_duodecimguttata_601 | 0.181 |
| Psyllobora_vigintiduopunctata_564 | Vibidia_duodecimguttata_601 | 0.181 |
| Psyllobora_vigintiduopunctata_570 | Vibidia_duodecimguttata_601 | 0.181 |
| Psyllobora_vigintiduopunctata_572 | Vibidia_duodecimguttata_601 | 0.181 |
| Psyllobora_vigintiduopunctata_560 | Vibidia_duodecimguttata_602 | 0.181 |
| Psyllobora_vigintiduopunctata_564 | Vibidia_duodecimguttata_602 | 0.181 |
| Psyllobora_vigintiduopunctata_570 | Vibidia_duodecimguttata_602 | 0.181 |
| Psyllobora_vigintiduopunctata_572 | Vibidia_duodecimguttata_602 | 0.181 |
| Psyllobora_vigintiduopunctata_561 | Vibidia_duodecimguttata_603 | 0.181 |
| Psyllobora_vigintiduopunctata_562 | Vibidia_duodecimguttata_603 | 0.181 |
| Psyllobora_vigintiduopunctata_565 | Vibidia_duodecimguttata_603 | 0.181 |
| Psyllobora_vigintiduopunctata_567 | Vibidia_duodecimguttata_603 | 0.181 |
| Scymnus_frontalis_736 | Scymnus_sinuanodulus_829 | 0.181 |
| Scymnus_frontalis_737 | Scymnus_sinuanodulus_829 | 0.181 |
| Scymnus_frontalis_738 | Scymnus_sinuanodulus_829 | 0.181 |
| Scymnus_frontalis_739 | Scymnus_sinuanodulus_829 | 0.181 |
| Scymnus_frontalis_740 | Scymnus_sinuanodulus_829 | 0.181 |
| Scymnus_frontalis_741 | Scymnus_sinuanodulus_829 | 0.181 |
| Scymnus_frontalis_743 | Scymnus_sinuanodulus_829 | 0.181 |

| | | |
|---|---|---|
| Scymnus_frontalis_744 | Scymnus_sinuanodulus_829 | 0.181 |
| Chilocorus_bipustulatus_21 | Scymnus_sinuanodulus_829 | 0.181 |
| Calvia_decemguttata_193 | Tytthaspis_sedecimpunctata_587 | 0.181 |
| Calvia_decemguttata_194 | Tytthaspis_sedecimpunctata_587 | 0.181 |
| Myzia_oblongoguttata_503 | Vibidia_duodecimguttata_596 | 0.181 |
| Myzia_oblongoguttata_504 | Vibidia_duodecimguttata_596 | 0.181 |
| Myzia_oblongoguttata_505 | Vibidia_duodecimguttata_596 | 0.181 |
| Myzia_oblongoguttata_507 | Vibidia_duodecimguttata_596 | 0.181 |
| Myzia_oblongoguttata_508 | Vibidia_duodecimguttata_596 | 0.181 |
| Myzia_oblongoguttata_509 | Vibidia_duodecimguttata_596 | 0.181 |
| Myzia_oblongoguttata_510 | Vibidia_duodecimguttata_596 | 0.181 |
| Myzia_oblongoguttata_511 | Vibidia_duodecimguttata_596 | 0.181 |
| Myzia_oblongoguttata_512 | Vibidia_duodecimguttata_596 | 0.181 |
| Myzia_oblongoguttata_513 | Vibidia_duodecimguttata_596 | 0.181 |
| Myzia_oblongoguttata_514 | Vibidia_duodecimguttata_596 | 0.181 |
| Myzia_oblongoguttata_516 | Vibidia_duodecimguttata_596 | 0.181 |
| Anatis_ocellata_132 | Vibidia_duodecimguttata_597 | 0.181 |
| Anatis_ocellata_134 | Vibidia_duodecimguttata_597 | 0.181 |
| Anatis_ocellata_135 | Vibidia_duodecimguttata_597 | 0.181 |
| Anatis_ocellata_138 | Vibidia_duodecimguttata_597 | 0.181 |
| Anatis_ocellata_139 | Vibidia_duodecimguttata_597 | 0.181 |
| Anatis_ocellata_140 | Vibidia_duodecimguttata_597 | 0.181 |
| Anatis_ocellata_141 | Vibidia_duodecimguttata_597 | 0.181 |
| Anatis_ocellata_142 | Vibidia_duodecimguttata_597 | 0.181 |
| Anatis_ocellata_144 | Vibidia_duodecimguttata_597 | 0.181 |
| Anatis_ocellata_145 | Vibidia_duodecimguttata_597 | 0.181 |
| Anatis_ocellata_146 | Vibidia_duodecimguttata_597 | 0.181 |
| Anatis_ocellata_133 | Vibidia_duodecimguttata_598 | 0.181 |
| Anatis_ocellata_137 | Vibidia_duodecimguttata_598 | 0.181 |
| Anatis_ocellata_143 | Vibidia_duodecimguttata_598 | 0.181 |
| Myzia_oblongoguttata_506 | Vibidia_duodecimguttata_598 | 0.181 |
| Anatis_ocellata_132 | Vibidia_duodecimguttata_600 | 0.181 |
| Anatis_ocellata_134 | Vibidia_duodecimguttata_600 | 0.181 |
| Anatis_ocellata_135 | Vibidia_duodecimguttata_600 | 0.181 |
| Anatis_ocellata_138 | Vibidia_duodecimguttata_600 | 0.181 |
| Anatis_ocellata_139 | Vibidia_duodecimguttata_600 | 0.181 |
| Anatis_ocellata_140 | Vibidia_duodecimguttata_600 | 0.181 |
| Anatis_ocellata_141 | Vibidia_duodecimguttata_600 | 0.181 |
| Anatis_ocellata_142 | Vibidia_duodecimguttata_600 | 0.181 |
| Anatis_ocellata_144 | Vibidia_duodecimguttata_600 | 0.181 |
| Anatis_ocellata_145 | Vibidia_duodecimguttata_600 | 0.181 |
| Anatis_ocellata_146 | Vibidia_duodecimguttata_600 | 0.181 |
| Scymnus_haemorrhoidalis_751 | Scymnus_sinuanodulus_831 | 0.181 |
| Scymnus_haemorrhoidalis_754 | Scymnus_sinuanodulus_831 | 0.181 |
| Scymnus_haemorrhoidalis_757 | Scymnus_sinuanodulus_831 | 0.181 |
| Scymnus_haemorrhoidalis_758 | Scymnus_sinuanodulus_831 | 0.181 |
| Hyperaspis_binotata_649 | Scymnus_sinuanodulus_829 | 0.181 |
| Anatis_ocellata_132 | Vibidia_duodecimguttata_598 | 0.181 |
| Anatis_ocellata_134 | Vibidia_duodecimguttata_598 | 0.181 |
| Anatis_ocellata_135 | Vibidia_duodecimguttata_598 | 0.181 |
| Anatis_ocellata_138 | Vibidia_duodecimguttata_598 | 0.181 |
| Anatis_ocellata_139 | Vibidia_duodecimguttata_598 | 0.181 |
| Anatis_ocellata_140 | Vibidia_duodecimguttata_598 | 0.181 |
| Anatis_ocellata_141 | Vibidia_duodecimguttata_598 | 0.181 |
| Anatis_ocellata_142 | Vibidia_duodecimguttata_598 | 0.181 |
| Anatis_ocellata_144 | Vibidia_duodecimguttata_598 | 0.181 |

Supplementary Table S4 (continued)

| | | |
|---|---|---|
| Anatis_ocellata_145 | Vibidia_duodecimguttata_598 | 0.181 |
| Anatis_ocellata_146 | Vibidia_duodecimguttata_598 | 0.181 |
| Anatis_ocellata_136 | Vibidia_duodecimguttata_597 | 0.181 |
| Anatis_ocellata_136 | Vibidia_duodecimguttata_600 | 0.181 |
| Hyperaspis_oregona_659 | Scymnus_sinuanodulus_829 | 0.181 |
| Platynaspis_luteorubra_51 | Tytthaspis_sedecimpunctata_591 | 0.181 |
| Platynaspis_luteorubra_53 | Tytthaspis_sedecimpunctata_591 | 0.181 |
| Platynaspis_luteorubra_58 | Tytthaspis_sedecimpunctata_591 | 0.181 |
| Platynaspis_luteorubra_55 | Tytthaspis_sedecimpunctata_583 | 0.181 |
| Platynaspis_luteorubra_55 | Tytthaspis_sedecimpunctata_585 | 0.181 |
| Hyperaspis_oregona_658 | Tytthaspis_sedecimpunctata_586 | 0.181 |
| Hyperaspis_oregona_658 | Tytthaspis_sedecimpunctata_588 | 0.181 |
| Platynaspis_luteorubra_55 | Tytthaspis_sedecimpunctata_586 | 0.181 |
| Platynaspis_luteorubra_51 | Tytthaspis_sedecimpunctata_587 | 0.181 |
| Platynaspis_luteorubra_53 | Tytthaspis_sedecimpunctata_587 | 0.181 |
| Platynaspis_luteorubra_58 | Tytthaspis_sedecimpunctata_587 | 0.181 |
| Calvia_quatuordecimguttata_203 | Vibidia_duodecimguttata_597 | 0.181 |
| Calvia_quatuordecimguttata_203 | Vibidia_duodecimguttata_600 | 0.181 |
| Hyperaspis_reppensis_667 | Tytthaspis_sedecimpunctata_577 | 0.181 |
| Hyperaspis_reppensis_667 | Tytthaspis_sedecimpunctata_578 | 0.181 |
| Hyperaspis_reppensis_667 | Tytthaspis_sedecimpunctata_579 | 0.181 |
| Hyperaspis_reppensis_667 | Tytthaspis_sedecimpunctata_581 | 0.181 |
| Hyperaspis_reppensis_667 | Tytthaspis_sedecimpunctata_582 | 0.181 |
| Hyperaspis_reppensis_664 | Tytthaspis_sedecimpunctata_585 | 0.181 |
| Hyperaspis_reppensis_667 | Tytthaspis_sedecimpunctata_590 | 0.181 |
| Hyperaspis_reppensis_667 | Tytthaspis_sedecimpunctata_593 | 0.181 |
| Hyperaspis_reppensis_667 | Tytthaspis_sedecimpunctata_594 | 0.181 |
| Hyperaspis_reppensis_667 | Tytthaspis_sedecimpunctata_595 | 0.181 |
| Anatis_ocellata_136 | Vibidia_duodecimguttata_598 | 0.181 |
| Chilocorus_renipustulatus_31 | Tytthaspis_sedecimpunctata_580 | 0.181 |
| Chilocorus_renipustulatus_32 | Tytthaspis_sedecimpunctata_580 | 0.181 |
| Chilocorus_renipustulatus_35 | Tytthaspis_sedecimpunctata_580 | 0.181 |
| Chilocorus_renipustulatus_36 | Tytthaspis_sedecimpunctata_580 | 0.181 |
| Chilocorus_renipustulatus_37 | Tytthaspis_sedecimpunctata_580 | 0.181 |
| Chilocorus_renipustulatus_38 | Tytthaspis_sedecimpunctata_580 | 0.181 |
| Chilocorus_renipustulatus_40 | Tytthaspis_sedecimpunctata_580 | 0.181 |
| Chilocorus_renipustulatus_41 | Tytthaspis_sedecimpunctata_580 | 0.181 |
| Chilocorus_renipustulatus_42 | Tytthaspis_sedecimpunctata_580 | 0.181 |
| Hyperaspis_reppensis_667 | Tytthaspis_sedecimpunctata_584 | 0.181 |
| Hyperaspis_reppensis_666 | Tytthaspis_sedecimpunctata_586 | 0.181 |
| Hyperaspis_reppensis_664 | Tytthaspis_sedecimpunctata_587 | 0.181 |
| Hyperaspis_reppensis_667 | Tytthaspis_sedecimpunctata_587 | 0.181 |
| Hyperaspis_reppensis_666 | Tytthaspis_sedecimpunctata_588 | 0.181 |
| Coccidula_scutellata_77 | Scymnus_suturalis_838 | 0.181 |
| Coccidula_scutellata_77 | Scymnus_suturalis_840 | 0.181 |
| Propylaea_quatuordecimpunctata_534 | Scymnus_suturalis_843 | 0.181 |
| Propylaea_quatuordecimpunctata_538 | Scymnus_suturalis_843 | 0.181 |
| Propylaea_quatuordecimpunctata_540 | Scymnus_suturalis_843 | 0.181 |
| Propylaea_quatuordecimpunctata_543 | Scymnus_suturalis_843 | 0.181 |
| Propylaea_quatuordecimpunctata_544 | Scymnus_suturalis_843 | 0.181 |
| Propylaea_quatuordecimpunctata_548 | Scymnus_suturalis_843 | 0.181 |
| Propylaea_quatuordecimpunctata_549 | Scymnus_suturalis_843 | 0.181 |
| Propylaea_quatuordecimpunctata_534 | Scymnus_suturalis_845 | 0.181 |
| Propylaea_quatuordecimpunctata_538 | Scymnus_suturalis_845 | 0.181 |
| Propylaea_quatuordecimpunctata_540 | Scymnus_suturalis_845 | 0.181 |
| Propylaea_quatuordecimpunctata_543 | Scymnus_suturalis_845 | 0.181 |

| | | |
|---|---|---|
| Propylaea_quatuordecimpunctata_544 | Scymnus_suturalis_845 | 0.181 |
| Propylaea_quatuordecimpunctata_548 | Scymnus_suturalis_845 | 0.181 |
| Propylaea_quatuordecimpunctata_549 | Scymnus_suturalis_845 | 0.181 |
| Myzia_oblongoguttata_504 | Tytthaspis_sedecimpunctata_577 | 0.181 |
| Myzia_oblongoguttata_505 | Tytthaspis_sedecimpunctata_577 | 0.181 |
| Myzia_oblongoguttata_507 | Tytthaspis_sedecimpunctata_577 | 0.181 |
| Myzia_oblongoguttata_508 | Tytthaspis_sedecimpunctata_577 | 0.181 |
| Myzia_oblongoguttata_509 | Tytthaspis_sedecimpunctata_577 | 0.181 |
| Myzia_oblongoguttata_510 | Tytthaspis_sedecimpunctata_577 | 0.181 |
| Myzia_oblongoguttata_511 | Tytthaspis_sedecimpunctata_577 | 0.181 |
| Myzia_oblongoguttata_512 | Tytthaspis_sedecimpunctata_577 | 0.181 |
| Myzia_oblongoguttata_513 | Tytthaspis_sedecimpunctata_577 | 0.181 |
| Myzia_oblongoguttata_514 | Tytthaspis_sedecimpunctata_577 | 0.181 |
| Myzia_oblongoguttata_516 | Tytthaspis_sedecimpunctata_577 | 0.181 |
| Scymnus_loewii_771 | Tytthaspis_sedecimpunctata_577 | 0.181 |
| Myzia_oblongoguttata_504 | Tytthaspis_sedecimpunctata_578 | 0.181 |
| Myzia_oblongoguttata_505 | Tytthaspis_sedecimpunctata_578 | 0.181 |
| Myzia_oblongoguttata_507 | Tytthaspis_sedecimpunctata_578 | 0.181 |
| Myzia_oblongoguttata_508 | Tytthaspis_sedecimpunctata_578 | 0.181 |
| Myzia_oblongoguttata_509 | Tytthaspis_sedecimpunctata_578 | 0.181 |
| Myzia_oblongoguttata_510 | Tytthaspis_sedecimpunctata_578 | 0.181 |
| Myzia_oblongoguttata_511 | Tytthaspis_sedecimpunctata_578 | 0.181 |
| Myzia_oblongoguttata_512 | Tytthaspis_sedecimpunctata_578 | 0.181 |
| Myzia_oblongoguttata_513 | Tytthaspis_sedecimpunctata_578 | 0.181 |
| Myzia_oblongoguttata_514 | Tytthaspis_sedecimpunctata_578 | 0.181 |
| Myzia_oblongoguttata_516 | Tytthaspis_sedecimpunctata_578 | 0.181 |
| Scymnus_loewii_771 | Tytthaspis_sedecimpunctata_578 | 0.181 |
| Myzia_oblongoguttata_504 | Tytthaspis_sedecimpunctata_579 | 0.181 |
| Myzia_oblongoguttata_505 | Tytthaspis_sedecimpunctata_579 | 0.181 |
| Myzia_oblongoguttata_507 | Tytthaspis_sedecimpunctata_579 | 0.181 |
| Myzia_oblongoguttata_508 | Tytthaspis_sedecimpunctata_579 | 0.181 |
| Myzia_oblongoguttata_509 | Tytthaspis_sedecimpunctata_579 | 0.181 |
| Myzia_oblongoguttata_510 | Tytthaspis_sedecimpunctata_579 | 0.181 |
| Myzia_oblongoguttata_511 | Tytthaspis_sedecimpunctata_579 | 0.181 |
| Myzia_oblongoguttata_512 | Tytthaspis_sedecimpunctata_579 | 0.181 |
| Myzia_oblongoguttata_513 | Tytthaspis_sedecimpunctata_579 | 0.181 |
| Myzia_oblongoguttata_514 | Tytthaspis_sedecimpunctata_579 | 0.181 |
| Myzia_oblongoguttata_516 | Tytthaspis_sedecimpunctata_579 | 0.181 |
| Scymnus_loewii_771 | Tytthaspis_sedecimpunctata_579 | 0.181 |
| Myzia_oblongoguttata_504 | Tytthaspis_sedecimpunctata_581 | 0.181 |
| Myzia_oblongoguttata_505 | Tytthaspis_sedecimpunctata_581 | 0.181 |
| Myzia_oblongoguttata_507 | Tytthaspis_sedecimpunctata_581 | 0.181 |
| Myzia_oblongoguttata_508 | Tytthaspis_sedecimpunctata_581 | 0.181 |
| Myzia_oblongoguttata_509 | Tytthaspis_sedecimpunctata_581 | 0.181 |
| Myzia_oblongoguttata_510 | Tytthaspis_sedecimpunctata_581 | 0.181 |
| Myzia_oblongoguttata_511 | Tytthaspis_sedecimpunctata_581 | 0.181 |
| Myzia_oblongoguttata_512 | Tytthaspis_sedecimpunctata_581 | 0.181 |
| Myzia_oblongoguttata_513 | Tytthaspis_sedecimpunctata_581 | 0.181 |
| Myzia_oblongoguttata_514 | Tytthaspis_sedecimpunctata_581 | 0.181 |
| Myzia_oblongoguttata_516 | Tytthaspis_sedecimpunctata_581 | 0.181 |
| Scymnus_loewii_771 | Tytthaspis_sedecimpunctata_581 | 0.181 |
| Myzia_oblongoguttata_504 | Tytthaspis_sedecimpunctata_582 | 0.181 |
| Myzia_oblongoguttata_505 | Tytthaspis_sedecimpunctata_582 | 0.181 |
| Myzia_oblongoguttata_507 | Tytthaspis_sedecimpunctata_582 | 0.181 |
| Myzia_oblongoguttata_508 | Tytthaspis_sedecimpunctata_582 | 0.181 |
| Myzia_oblongoguttata_509 | Tytthaspis_sedecimpunctata_582 | 0.181 |

| | | |
|---|---|---|
| Myzia_oblongoguttata_510 | Tytthaspis_sedecimpunctata_582 | 0.181 |
| Myzia_oblongoguttata_511 | Tytthaspis_sedecimpunctata_582 | 0.181 |
| Myzia_oblongoguttata_512 | Tytthaspis_sedecimpunctata_582 | 0.181 |
| Myzia_oblongoguttata_513 | Tytthaspis_sedecimpunctata_582 | 0.181 |
| Myzia_oblongoguttata_514 | Tytthaspis_sedecimpunctata_582 | 0.181 |
| Myzia_oblongoguttata_516 | Tytthaspis_sedecimpunctata_582 | 0.181 |
| Scymnus_loewii_771 | Tytthaspis_sedecimpunctata_582 | 0.181 |
| Scymnus_loewii_770 | Tytthaspis_sedecimpunctata_583 | 0.181 |
| Scymnus_loewii_773 | Tytthaspis_sedecimpunctata_583 | 0.181 |
| Scymnus_nebulosus_782 | Tytthaspis_sedecimpunctata_583 | 0.181 |
| Myzia_oblongoguttata_504 | Tytthaspis_sedecimpunctata_585 | 0.181 |
| Myzia_oblongoguttata_505 | Tytthaspis_sedecimpunctata_585 | 0.181 |
| Myzia_oblongoguttata_507 | Tytthaspis_sedecimpunctata_585 | 0.181 |
| Myzia_oblongoguttata_508 | Tytthaspis_sedecimpunctata_585 | 0.181 |
| Myzia_oblongoguttata_509 | Tytthaspis_sedecimpunctata_585 | 0.181 |
| Myzia_oblongoguttata_510 | Tytthaspis_sedecimpunctata_585 | 0.181 |
| Myzia_oblongoguttata_511 | Tytthaspis_sedecimpunctata_585 | 0.181 |
| Myzia_oblongoguttata_512 | Tytthaspis_sedecimpunctata_585 | 0.181 |
| Myzia_oblongoguttata_513 | Tytthaspis_sedecimpunctata_585 | 0.181 |
| Myzia_oblongoguttata_514 | Tytthaspis_sedecimpunctata_585 | 0.181 |
| Myzia_oblongoguttata_516 | Tytthaspis_sedecimpunctata_585 | 0.181 |
| Scymnus_loewii_771 | Tytthaspis_sedecimpunctata_585 | 0.181 |
| Brumus_quadripustulatus_9 | Tytthaspis_sedecimpunctata_589 | 0.181 |
| Scymnus_loewii_771 | Tytthaspis_sedecimpunctata_589 | 0.181 |
| Myzia_oblongoguttata_504 | Tytthaspis_sedecimpunctata_590 | 0.181 |
| Myzia_oblongoguttata_505 | Tytthaspis_sedecimpunctata_590 | 0.181 |
| Myzia_oblongoguttata_507 | Tytthaspis_sedecimpunctata_590 | 0.181 |
| Myzia_oblongoguttata_508 | Tytthaspis_sedecimpunctata_590 | 0.181 |
| Myzia_oblongoguttata_509 | Tytthaspis_sedecimpunctata_590 | 0.181 |
| Myzia_oblongoguttata_510 | Tytthaspis_sedecimpunctata_590 | 0.181 |
| Myzia_oblongoguttata_511 | Tytthaspis_sedecimpunctata_590 | 0.181 |
| Myzia_oblongoguttata_512 | Tytthaspis_sedecimpunctata_590 | 0.181 |
| Myzia_oblongoguttata_513 | Tytthaspis_sedecimpunctata_590 | 0.181 |
| Myzia_oblongoguttata_514 | Tytthaspis_sedecimpunctata_590 | 0.181 |
| Myzia_oblongoguttata_516 | Tytthaspis_sedecimpunctata_590 | 0.181 |
| Scymnus_loewii_771 | Tytthaspis_sedecimpunctata_590 | 0.181 |
| Myzia_oblongoguttata_504 | Tytthaspis_sedecimpunctata_592 | 0.181 |
| Myzia_oblongoguttata_505 | Tytthaspis_sedecimpunctata_592 | 0.181 |
| Myzia_oblongoguttata_507 | Tytthaspis_sedecimpunctata_592 | 0.181 |
| Myzia_oblongoguttata_508 | Tytthaspis_sedecimpunctata_592 | 0.181 |
| Myzia_oblongoguttata_509 | Tytthaspis_sedecimpunctata_592 | 0.181 |
| Myzia_oblongoguttata_510 | Tytthaspis_sedecimpunctata_592 | 0.181 |
| Myzia_oblongoguttata_511 | Tytthaspis_sedecimpunctata_592 | 0.181 |
| Myzia_oblongoguttata_512 | Tytthaspis_sedecimpunctata_592 | 0.181 |
| Myzia_oblongoguttata_513 | Tytthaspis_sedecimpunctata_592 | 0.181 |
| Myzia_oblongoguttata_514 | Tytthaspis_sedecimpunctata_592 | 0.181 |
| Myzia_oblongoguttata_516 | Tytthaspis_sedecimpunctata_592 | 0.181 |
| Scymnus_loewii_771 | Tytthaspis_sedecimpunctata_592 | 0.181 |
| Coccinella_transversalis_257 | Tytthaspis_sedecimpunctata_593 | 0.181 |
| Coccinella_transversalis_259 | Tytthaspis_sedecimpunctata_593 | 0.181 |
| Coccinella_transversalis_260 | Tytthaspis_sedecimpunctata_593 | 0.181 |
| Coccinella_transversalis_261 | Tytthaspis_sedecimpunctata_593 | 0.181 |
| Coccinella_transversalis_262 | Tytthaspis_sedecimpunctata_593 | 0.181 |
| Coccinella_transversalis_263 | Tytthaspis_sedecimpunctata_593 | 0.181 |
| Coccinella_transversalis_264 | Tytthaspis_sedecimpunctata_593 | 0.181 |
| Myzia_oblongoguttata_503 | Tytthaspis_sedecimpunctata_593 | 0.181 |

| | | |
|---|---|---|
| Myzia_oblongoguttata_515 | Tytthaspis_sedecimpunctata_593 | 0.181 |
| Scymnus_loewii_771 | Tytthaspis_sedecimpunctata_593 | 0.181 |
| Myzia_oblongoguttata_504 | Tytthaspis_sedecimpunctata_594 | 0.181 |
| Myzia_oblongoguttata_505 | Tytthaspis_sedecimpunctata_594 | 0.181 |
| Myzia_oblongoguttata_507 | Tytthaspis_sedecimpunctata_594 | 0.181 |
| Myzia_oblongoguttata_508 | Tytthaspis_sedecimpunctata_594 | 0.181 |
| Myzia_oblongoguttata_509 | Tytthaspis_sedecimpunctata_594 | 0.181 |
| Myzia_oblongoguttata_510 | Tytthaspis_sedecimpunctata_594 | 0.181 |
| Myzia_oblongoguttata_511 | Tytthaspis_sedecimpunctata_594 | 0.181 |
| Myzia_oblongoguttata_512 | Tytthaspis_sedecimpunctata_594 | 0.181 |
| Myzia_oblongoguttata_513 | Tytthaspis_sedecimpunctata_594 | 0.181 |
| Myzia_oblongoguttata_514 | Tytthaspis_sedecimpunctata_594 | 0.181 |
| Myzia_oblongoguttata_516 | Tytthaspis_sedecimpunctata_594 | 0.181 |
| Scymnus_loewii_771 | Tytthaspis_sedecimpunctata_594 | 0.181 |
| Myzia_oblongoguttata_504 | Tytthaspis_sedecimpunctata_595 | 0.181 |
| Myzia_oblongoguttata_505 | Tytthaspis_sedecimpunctata_595 | 0.181 |
| Myzia_oblongoguttata_507 | Tytthaspis_sedecimpunctata_595 | 0.181 |
| Myzia_oblongoguttata_508 | Tytthaspis_sedecimpunctata_595 | 0.181 |
| Myzia_oblongoguttata_509 | Tytthaspis_sedecimpunctata_595 | 0.181 |
| Myzia_oblongoguttata_510 | Tytthaspis_sedecimpunctata_595 | 0.181 |
| Myzia_oblongoguttata_511 | Tytthaspis_sedecimpunctata_595 | 0.181 |
| Myzia_oblongoguttata_512 | Tytthaspis_sedecimpunctata_595 | 0.181 |
| Myzia_oblongoguttata_513 | Tytthaspis_sedecimpunctata_595 | 0.181 |
| Myzia_oblongoguttata_514 | Tytthaspis_sedecimpunctata_595 | 0.181 |
| Myzia_oblongoguttata_516 | Tytthaspis_sedecimpunctata_595 | 0.181 |
| Scymnus_loewii_771 | Tytthaspis_sedecimpunctata_595 | 0.181 |
| Coccidula_scutellata_77 | Scymnus_suturalis_834 | 0.181 |
| Coccidula_scutellata_80 | Scymnus_suturalis_838 | 0.181 |
| Coccidula_scutellata_80 | Scymnus_suturalis_840 | 0.181 |
| Psyllobora_vigintiduopunctata_558 | Vibidia_duodecimguttata_599 | 0.181 |
| Psyllobora_vigintiduopunctata_568 | Vibidia_duodecimguttata_599 | 0.181 |
| Psyllobora_vigintiduopunctata_558 | Vibidia_duodecimguttata_601 | 0.181 |
| Psyllobora_vigintiduopunctata_568 | Vibidia_duodecimguttata_601 | 0.181 |
| Psyllobora_vigintiduopunctata_558 | Vibidia_duodecimguttata_602 | 0.181 |
| Psyllobora_vigintiduopunctata_568 | Vibidia_duodecimguttata_602 | 0.181 |
| Chilocorus_stigma_43 | Scymnus_suturalis_841 | 0.182 |
| Coccidula_scutellata_78 | Scymnus_suturalis_841 | 0.182 |
| Coccidula_scutellata_79 | Scymnus_suturalis_841 | 0.182 |
| Chilocorus_stigma_43 | Scymnus_suturalis_842 | 0.182 |
| Coccidula_scutellata_78 | Scymnus_suturalis_842 | 0.182 |
| Coccidula_scutellata_79 | Scymnus_suturalis_842 | 0.182 |
| Chilocorus_stigma_43 | Scymnus_suturalis_844 | 0.182 |
| Coccidula_scutellata_78 | Scymnus_suturalis_844 | 0.182 |
| Coccidula_scutellata_79 | Scymnus_suturalis_844 | 0.182 |
| Chilocorus_stigma_43 | Scymnus_suturalis_846 | 0.182 |
| Coccidula_scutellata_78 | Scymnus_suturalis_846 | 0.182 |
| Coccidula_scutellata_79 | Scymnus_suturalis_846 | 0.182 |
| Chilocorus_stigma_43 | Scymnus_suturalis_847 | 0.182 |
| Coccidula_scutellata_78 | Scymnus_suturalis_847 | 0.182 |
| Coccidula_scutellata_79 | Scymnus_suturalis_847 | 0.182 |
| Chilocorus_stigma_43 | Scymnus_suturalis_848 | 0.182 |
| Coccidula_scutellata_78 | Scymnus_suturalis_848 | 0.182 |
| Coccidula_scutellata_79 | Scymnus_suturalis_848 | 0.182 |
| Didion_punctatum_645 | Tytthaspis_sedecimpunctata_577 | 0.182 |
| Didion_punctatum_645 | Tytthaspis_sedecimpunctata_578 | 0.182 |
| Didion_punctatum_645 | Tytthaspis_sedecimpunctata_579 | 0.182 |

| | | |
|---|---|---|
| Myzia_oblongoguttata_506 | Tytthaspis_sedecimpunctata_580 | 0.182 |
| Scymnus_nebulosus_782 | Tytthaspis_sedecimpunctata_580 | 0.182 |
| Scymnus_ningshanensis_788 | Tytthaspis_sedecimpunctata_580 | 0.182 |
| Scymnus_ningshanensis_791 | Tytthaspis_sedecimpunctata_580 | 0.182 |
| Scymnus_ningshanensis_792 | Tytthaspis_sedecimpunctata_580 | 0.182 |
| Scymnus_ningshanensis_793 | Tytthaspis_sedecimpunctata_580 | 0.182 |
| Scymnus_ningshanensis_794 | Tytthaspis_sedecimpunctata_580 | 0.182 |
| Scymnus_ningshanensis_795 | Tytthaspis_sedecimpunctata_580 | 0.182 |
| Didion_punctatum_645 | Tytthaspis_sedecimpunctata_581 | 0.182 |
| Didion_punctatum_645 | Tytthaspis_sedecimpunctata_582 | 0.182 |
| Myzia_pullata_518 | Tytthaspis_sedecimpunctata_584 | 0.182 |
| Brumus_quadripustulatus_12 | Tytthaspis_sedecimpunctata_586 | 0.182 |
| Myzia_oblongoguttata_506 | Tytthaspis_sedecimpunctata_586 | 0.182 |
| Scymnus_loewii_770 | Tytthaspis_sedecimpunctata_586 | 0.182 |
| Scymnus_loewii_773 | Tytthaspis_sedecimpunctata_586 | 0.182 |
| Brumus_quadripustulatus_15 | Tytthaspis_sedecimpunctata_587 | 0.182 |
| Brumus_quadripustulatus_16 | Tytthaspis_sedecimpunctata_587 | 0.182 |
| Brumus_quadripustulatus_6 | Tytthaspis_sedecimpunctata_587 | 0.182 |
| Brumus_quadripustulatus_8 | Tytthaspis_sedecimpunctata_587 | 0.182 |
| Brumus_quadripustulatus_9 | Tytthaspis_sedecimpunctata_588 | 0.182 |
| Myzia_oblongoguttata_506 | Tytthaspis_sedecimpunctata_588 | 0.182 |
| Didion_punctatum_645 | Tytthaspis_sedecimpunctata_590 | 0.182 |
| Brumus_quadripustulatus_9 | Tytthaspis_sedecimpunctata_591 | 0.182 |
| Didion_punctatum_645 | Tytthaspis_sedecimpunctata_593 | 0.182 |
| Didion_punctatum_645 | Tytthaspis_sedecimpunctata_594 | 0.182 |
| Didion_punctatum_645 | Tytthaspis_sedecimpunctata_595 | 0.182 |
| Chilocorus_stigma_43 | Scymnus_suturalis_832 | 0.182 |
| Coccidula_scutellata_78 | Scymnus_suturalis_832 | 0.182 |
| Coccidula_scutellata_79 | Scymnus_suturalis_832 | 0.182 |
| Coccidula_scutellata_80 | Scymnus_suturalis_834 | 0.182 |
| Chilocorus_stigma_43 | Scymnus_suturalis_835 | 0.182 |
| Coccidula_scutellata_78 | Scymnus_suturalis_835 | 0.182 |
| Coccidula_scutellata_79 | Scymnus_suturalis_835 | 0.182 |
| Chilocorus_stigma_43 | Scymnus_suturalis_836 | 0.182 |
| Coccidula_scutellata_78 | Scymnus_suturalis_836 | 0.182 |
| Coccidula_scutellata_79 | Scymnus_suturalis_836 | 0.182 |
| Chilocorus_stigma_43 | Scymnus_suturalis_837 | 0.182 |
| Coccidula_scutellata_83 | Scymnus_suturalis_837 | 0.182 |
| Chilocorus_stigma_43 | Scymnus_suturalis_839 | 0.182 |
| Coccidula_scutellata_78 | Scymnus_suturalis_839 | 0.182 |
| Coccidula_scutellata_79 | Scymnus_suturalis_839 | 0.182 |
| Myzia_pullata_517 | Scymnus_suturalis_841 | 0.182 |
| Myzia_pullata_517 | Scymnus_suturalis_842 | 0.182 |
| Myzia_pullata_518 | Scymnus_suturalis_843 | 0.182 |
| Myzia_pullata_517 | Scymnus_suturalis_844 | 0.182 |
| Myzia_pullata_517 | Scymnus_suturalis_846 | 0.182 |
| Myzia_pullata_517 | Scymnus_suturalis_847 | 0.182 |
| Myzia_pullata_517 | Scymnus_suturalis_848 | 0.182 |
| Didion_punctatum_646 | Tytthaspis_sedecimpunctata_577 | 0.182 |
| Didion_punctatum_646 | Tytthaspis_sedecimpunctata_578 | 0.182 |
| Didion_punctatum_646 | Tytthaspis_sedecimpunctata_579 | 0.182 |
| Didion_punctatum_646 | Tytthaspis_sedecimpunctata_581 | 0.182 |
| Didion_punctatum_646 | Tytthaspis_sedecimpunctata_582 | 0.182 |
| Myzia_pullata_518 | Tytthaspis_sedecimpunctata_583 | 0.182 |
| Myzia_pullata_517 | Tytthaspis_sedecimpunctata_585 | 0.182 |
| Aphidecta_obliterata_185 | Tytthaspis_sedecimpunctata_588 | 0.182 |

| | | |
|---|---|---|
| Aphidecta_obliterata_186 | Tytthaspis_sedecimpunctata_588 | 0.182 |
| Aphidecta_obliterata_181 | Tytthaspis_sedecimpunctata_589 | 0.182 |
| Myzia_pullata_518 | Tytthaspis_sedecimpunctata_589 | 0.182 |
| Didion_punctatum_646 | Tytthaspis_sedecimpunctata_590 | 0.182 |
| Didion_punctatum_646 | Tytthaspis_sedecimpunctata_593 | 0.182 |
| Didion_punctatum_646 | Tytthaspis_sedecimpunctata_594 | 0.182 |
| Didion_punctatum_646 | Tytthaspis_sedecimpunctata_595 | 0.182 |
| Coccidula_scutellata_81 | Scymnus_sinuanodulus_830 | 0.182 |
| Coccidula_scutellata_82 | Scymnus_sinuanodulus_830 | 0.182 |
| Myzia_pullata_517 | Scymnus_suturalis_832 | 0.182 |
| Myzia_pullata_517 | Scymnus_suturalis_835 | 0.182 |
| Myzia_pullata_517 | Scymnus_suturalis_836 | 0.182 |
| Myzia_pullata_517 | Scymnus_suturalis_837 | 0.182 |
| Myzia_pullata_517 | Scymnus_suturalis_839 | 0.182 |
| Scymnus_haemorrhoidalis_751 | Scymnus_sinuanodulus_829 | 0.182 |
| Scymnus_haemorrhoidalis_754 | Scymnus_sinuanodulus_829 | 0.182 |
| Scymnus_haemorrhoidalis_757 | Scymnus_sinuanodulus_829 | 0.182 |
| Scymnus_haemorrhoidalis_758 | Scymnus_sinuanodulus_829 | 0.182 |
| Aphidecta_obliterata_185 | Tytthaspis_sedecimpunctata_577 | 0.182 |
| Aphidecta_obliterata_186 | Tytthaspis_sedecimpunctata_577 | 0.182 |
| Didion_punctatum_644 | Tytthaspis_sedecimpunctata_577 | 0.182 |
| Scymnus_camptodromus_704 | Tytthaspis_sedecimpunctata_577 | 0.182 |
| Aphidecta_obliterata_185 | Tytthaspis_sedecimpunctata_578 | 0.182 |
| Aphidecta_obliterata_186 | Tytthaspis_sedecimpunctata_578 | 0.182 |
| Didion_punctatum_644 | Tytthaspis_sedecimpunctata_578 | 0.182 |
| Scymnus_camptodromus_704 | Tytthaspis_sedecimpunctata_578 | 0.182 |
| Aphidecta_obliterata_185 | Tytthaspis_sedecimpunctata_579 | 0.182 |
| Aphidecta_obliterata_186 | Tytthaspis_sedecimpunctata_579 | 0.182 |
| Didion_punctatum_644 | Tytthaspis_sedecimpunctata_579 | 0.182 |
| Scymnus_camptodromus_704 | Tytthaspis_sedecimpunctata_579 | 0.182 |
| Aphidecta_obliterata_185 | Tytthaspis_sedecimpunctata_581 | 0.182 |
| Aphidecta_obliterata_186 | Tytthaspis_sedecimpunctata_581 | 0.182 |
| Didion_punctatum_644 | Tytthaspis_sedecimpunctata_581 | 0.182 |
| Scymnus_camptodromus_704 | Tytthaspis_sedecimpunctata_581 | 0.182 |
| Aphidecta_obliterata_185 | Tytthaspis_sedecimpunctata_582 | 0.182 |
| Aphidecta_obliterata_186 | Tytthaspis_sedecimpunctata_582 | 0.182 |
| Didion_punctatum_644 | Tytthaspis_sedecimpunctata_582 | 0.182 |
| Scymnus_camptodromus_704 | Tytthaspis_sedecimpunctata_582 | 0.182 |
| Scymnus_camptodromus_714 | Tytthaspis_sedecimpunctata_583 | 0.182 |
| Scymnus_camptodromus_704 | Tytthaspis_sedecimpunctata_585 | 0.182 |
| Aphidecta_obliterata_181 | Tytthaspis_sedecimpunctata_586 | 0.182 |
| Anisosticta_novemdecimpunctata_174 | Tytthaspis_sedecimpunctata_587 | 0.182 |
| Aphidecta_obliterata_184 | Tytthaspis_sedecimpunctata_587 | 0.182 |
| Myzia_pullata_517 | Tytthaspis_sedecimpunctata_587 | 0.182 |
| Propylaea_quatuordecimpunctata_545 | Tytthaspis_sedecimpunctata_587 | 0.182 |
| Coccinella_hieroglyphica_227 | Tytthaspis_sedecimpunctata_589 | 0.182 |
| Aphidecta_obliterata_185 | Tytthaspis_sedecimpunctata_590 | 0.182 |
| Aphidecta_obliterata_186 | Tytthaspis_sedecimpunctata_590 | 0.182 |
| Didion_punctatum_644 | Tytthaspis_sedecimpunctata_590 | 0.182 |
| Scymnus_camptodromus_704 | Tytthaspis_sedecimpunctata_590 | 0.182 |
| Anisosticta_novemdecimpunctata_174 | Tytthaspis_sedecimpunctata_591 | 0.182 |
| Aphidecta_obliterata_184 | Tytthaspis_sedecimpunctata_591 | 0.182 |
| Propylaea_quatuordecimpunctata_547 | Tytthaspis_sedecimpunctata_591 | 0.182 |
| Aphidecta_obliterata_183 | Tytthaspis_sedecimpunctata_592 | 0.182 |
| Scymnus_camptodromus_704 | Tytthaspis_sedecimpunctata_592 | 0.182 |
| Aphidecta_obliterata_185 | Tytthaspis_sedecimpunctata_593 | 0.182 |

| | | |
|---|---|---|
| Aphidecta_obliterata_186 | Tytthaspis_sedecimpunctata_593 | 0.182 |
| Didion_punctatum_644 | Tytthaspis_sedecimpunctata_593 | 0.182 |
| Scymnus_camptodromus_704 | Tytthaspis_sedecimpunctata_593 | 0.182 |
| Aphidecta_obliterata_185 | Tytthaspis_sedecimpunctata_594 | 0.182 |
| Aphidecta_obliterata_186 | Tytthaspis_sedecimpunctata_594 | 0.182 |
| Didion_punctatum_644 | Tytthaspis_sedecimpunctata_594 | 0.182 |
| Scymnus_camptodromus_704 | Tytthaspis_sedecimpunctata_594 | 0.182 |
| Aphidecta_obliterata_185 | Tytthaspis_sedecimpunctata_595 | 0.182 |
| Aphidecta_obliterata_186 | Tytthaspis_sedecimpunctata_595 | 0.182 |
| Didion_punctatum_644 | Tytthaspis_sedecimpunctata_595 | 0.182 |
| Scymnus_camptodromus_704 | Tytthaspis_sedecimpunctata_595 | 0.182 |
| Anatis_ocellata_143 | Scymnus_suturalis_833 | 0.182 |
| Myzia_pullata_518 | Scymnus_suturalis_833 | 0.182 |
| Brumus_quadripustulatus_5 | Scymnus_sinuanodulus_831 | 0.182 |
| Anatis_ocellata_143 | Scymnus_suturalis_841 | 0.182 |
| Anatis_ocellata_143 | Scymnus_suturalis_842 | 0.182 |
| Anatis_ocellata_143 | Scymnus_suturalis_844 | 0.182 |
| Anatis_ocellata_143 | Scymnus_suturalis_846 | 0.182 |
| Anatis_ocellata_143 | Scymnus_suturalis_847 | 0.182 |
| Anatis_ocellata_143 | Scymnus_suturalis_848 | 0.182 |
| Scymnus_nebulosus_782 | Subcoccinella_vigintiquatuorpunctata_630 | 0.182 |
| Coccinella_septempunctata_254 | Tytthaspis_sedecimpunctata_577 | 0.182 |
| Coccinella_septempunctata_254 | Tytthaspis_sedecimpunctata_578 | 0.182 |
| Coccinella_septempunctata_254 | Tytthaspis_sedecimpunctata_579 | 0.182 |
| Aphidecta_obliterata_183 | Tytthaspis_sedecimpunctata_580 | 0.182 |
| Scymnus_camptodromus_714 | Tytthaspis_sedecimpunctata_580 | 0.182 |
| Coccinella_septempunctata_254 | Tytthaspis_sedecimpunctata_581 | 0.182 |
| Coccinella_septempunctata_254 | Tytthaspis_sedecimpunctata_582 | 0.182 |
| Aphidecta_obliterata_181 | Tytthaspis_sedecimpunctata_583 | 0.182 |
| Scymnus_interruptus_760 | Tytthaspis_sedecimpunctata_583 | 0.182 |
| Scymnus_interruptus_765 | Tytthaspis_sedecimpunctata_583 | 0.182 |
| Scymnus_interruptus_768 | Tytthaspis_sedecimpunctata_583 | 0.182 |
| Aphidecta_obliterata_181 | Tytthaspis_sedecimpunctata_585 | 0.182 |
| Coccinella_septempunctata_254 | Tytthaspis_sedecimpunctata_585 | 0.182 |
| Coccinella_trifasciata_275 | Tytthaspis_sedecimpunctata_586 | 0.182 |
| Coccinella_trifasciata_277 | Tytthaspis_sedecimpunctata_586 | 0.182 |
| Coccinella_trifasciata_278 | Tytthaspis_sedecimpunctata_586 | 0.182 |
| Coccinella_trifasciata_279 | Tytthaspis_sedecimpunctata_586 | 0.182 |
| Coccinella_trifasciata_281 | Tytthaspis_sedecimpunctata_586 | 0.182 |
| Scymnus_caurinus_720 | Tytthaspis_sedecimpunctata_586 | 0.182 |
| Scymnus_caurinus_721 | Tytthaspis_sedecimpunctata_586 | 0.182 |
| Scymnus_caurinus_722 | Tytthaspis_sedecimpunctata_586 | 0.182 |
| Coccinella_hieroglyphica_227 | Tytthaspis_sedecimpunctata_587 | 0.182 |
| Coccinella_trifasciata_272 | Tytthaspis_sedecimpunctata_587 | 0.182 |
| Coccinella_trifasciata_276 | Tytthaspis_sedecimpunctata_587 | 0.182 |
| Coccinella_septempunctata_254 | Tytthaspis_sedecimpunctata_590 | 0.182 |
| Anatis_labiculata_123 | Tytthaspis_sedecimpunctata_591 | 0.182 |
| Anatis_labiculata_124 | Tytthaspis_sedecimpunctata_591 | 0.182 |
| Anatis_labiculata_125 | Tytthaspis_sedecimpunctata_591 | 0.182 |
| Anatis_labiculata_127 | Tytthaspis_sedecimpunctata_591 | 0.182 |
| Coccinella_septempunctata_242 | Tytthaspis_sedecimpunctata_591 | 0.182 |
| Coccinella_septempunctata_244 | Tytthaspis_sedecimpunctata_591 | 0.182 |
| Coccinella_septempunctata_245 | Tytthaspis_sedecimpunctata_591 | 0.182 |
| Coccinella_septempunctata_247 | Tytthaspis_sedecimpunctata_591 | 0.182 |
| Coccinella_septempunctata_248 | Tytthaspis_sedecimpunctata_591 | 0.182 |
| Coccinella_septempunctata_249 | Tytthaspis_sedecimpunctata_591 | 0.182 |

| | | |
|---|---|---|
| Coccinella_septempunctata_252 | Tytthaspis_sedecimpunctata_591 | 0.182 |
| Coccinella_septempunctata_255 | Tytthaspis_sedecimpunctata_591 | 0.182 |
| Coccinella_septempunctata_256 | Tytthaspis_sedecimpunctata_591 | 0.182 |
| Aphidecta_obliterata_176 | Tytthaspis_sedecimpunctata_592 | 0.182 |
| Coccinella_septempunctata_254 | Tytthaspis_sedecimpunctata_592 | 0.182 |
| Coccinella_septempunctata_254 | Tytthaspis_sedecimpunctata_593 | 0.182 |
| Coccinella_septempunctata_254 | Tytthaspis_sedecimpunctata_594 | 0.182 |
| Coccinella_septempunctata_254 | Tytthaspis_sedecimpunctata_595 | 0.182 |
| Chilocorus_nigritus_26 | Scymnus_rubromaculatus_822 | 0.182 |
| Chilocorus_nigritus_26 | Scymnus_rubromaculatus_823 | 0.182 |
| Chilocorus_nigritus_26 | Scymnus_rubromaculatus_824 | 0.182 |
| Chilocorus_nigritus_26 | Scymnus_rubromaculatus_825 | 0.182 |
| Chilocorus_nigritus_26 | Scymnus_rubromaculatus_826 | 0.182 |
| Chilocorus_nigritus_26 | Scymnus_rubromaculatus_827 | 0.182 |
| Anatis_ocellata_143 | Scymnus_suturalis_832 | 0.182 |
| Anatis_ocellata_143 | Scymnus_suturalis_835 | 0.182 |
| Anatis_ocellata_143 | Scymnus_suturalis_836 | 0.182 |
| Hyperaspis_lateralis_654 | Scymnus_suturalis_838 | 0.182 |
| Hyperaspis_lateralis_657 | Scymnus_suturalis_838 | 0.182 |
| Anatis_ocellata_143 | Scymnus_suturalis_839 | 0.182 |
| Hyperaspis_lateralis_654 | Scymnus_suturalis_840 | 0.182 |
| Hyperaspis_lateralis_657 | Scymnus_suturalis_840 | 0.182 |
| Hyperaspis_lateralis_656 | Stethorus_punctillum_849 | 0.182 |
| Hyperaspis_lateralis_656 | Stethorus_punctillum_850 | 0.182 |
| Hyperaspis_lateralis_656 | Stethorus_punctillum_854 | 0.182 |
| Hyperaspis_lateralis_656 | Stethorus_punctillum_856 | 0.182 |
| Anatis_labiculata_123 | Tytthaspis_sedecimpunctata_577 | 0.182 |
| Anatis_labiculata_124 | Tytthaspis_sedecimpunctata_577 | 0.182 |
| Anatis_labiculata_125 | Tytthaspis_sedecimpunctata_577 | 0.182 |
| Anatis_labiculata_127 | Tytthaspis_sedecimpunctata_577 | 0.182 |
| Scymnus_camptodromus_712 | Tytthaspis_sedecimpunctata_577 | 0.182 |
| Anatis_labiculata_123 | Tytthaspis_sedecimpunctata_578 | 0.182 |
| Anatis_labiculata_124 | Tytthaspis_sedecimpunctata_578 | 0.182 |
| Anatis_labiculata_125 | Tytthaspis_sedecimpunctata_578 | 0.182 |
| Anatis_labiculata_127 | Tytthaspis_sedecimpunctata_578 | 0.182 |
| Scymnus_camptodromus_712 | Tytthaspis_sedecimpunctata_578 | 0.182 |
| Anatis_labiculata_123 | Tytthaspis_sedecimpunctata_579 | 0.182 |
| Anatis_labiculata_124 | Tytthaspis_sedecimpunctata_579 | 0.182 |
| Anatis_labiculata_125 | Tytthaspis_sedecimpunctata_579 | 0.182 |
| Anatis_labiculata_127 | Tytthaspis_sedecimpunctata_579 | 0.182 |
| Scymnus_camptodromus_712 | Tytthaspis_sedecimpunctata_579 | 0.182 |
| Aphidecta_obliterata_176 | Tytthaspis_sedecimpunctata_580 | 0.182 |
| Coccinella_septempunctata_254 | Tytthaspis_sedecimpunctata_580 | 0.182 |
| Anatis_labiculata_123 | Tytthaspis_sedecimpunctata_581 | 0.182 |
| Anatis_labiculata_124 | Tytthaspis_sedecimpunctata_581 | 0.182 |
| Anatis_labiculata_125 | Tytthaspis_sedecimpunctata_581 | 0.182 |
| Anatis_labiculata_127 | Tytthaspis_sedecimpunctata_581 | 0.182 |
| Scymnus_camptodromus_712 | Tytthaspis_sedecimpunctata_581 | 0.182 |
| Anatis_labiculata_123 | Tytthaspis_sedecimpunctata_582 | 0.182 |
| Anatis_labiculata_124 | Tytthaspis_sedecimpunctata_582 | 0.182 |
| Anatis_labiculata_125 | Tytthaspis_sedecimpunctata_582 | 0.182 |
| Anatis_labiculata_127 | Tytthaspis_sedecimpunctata_582 | 0.182 |
| Scymnus_camptodromus_712 | Tytthaspis_sedecimpunctata_582 | 0.182 |
| Anatis_labiculata_126 | Tytthaspis_sedecimpunctata_583 | 0.182 |
| Anisosticta_novemdecimpunctata_174 | Tytthaspis_sedecimpunctata_584 | 0.182 |
| Aphidecta_obliterata_181 | Tytthaspis_sedecimpunctata_584 | 0.182 |

| | | |
|---|---|---|
| Aphidecta_obliterata_184 | Tytthaspis_sedecimpunctata_584 | 0.182 |
| Coccinella_septempunctata_254 | Tytthaspis_sedecimpunctata_584 | 0.182 |
| Coccinella_undecimpunctata_285 | Tytthaspis_sedecimpunctata_584 | 0.182 |
| Coccinella_undecimpunctata_286 | Tytthaspis_sedecimpunctata_584 | 0.182 |
| Coccinella_undecimpunctata_288 | Tytthaspis_sedecimpunctata_584 | 0.182 |
| Scymnus_camptodromus_712 | Tytthaspis_sedecimpunctata_585 | 0.182 |
| Coccinella_trifasciata_274 | Tytthaspis_sedecimpunctata_586 | 0.182 |
| Coccinella_undecimpunctata_285 | Tytthaspis_sedecimpunctata_586 | 0.182 |
| Coccinella_undecimpunctata_286 | Tytthaspis_sedecimpunctata_586 | 0.182 |
| Coccinella_undecimpunctata_288 | Tytthaspis_sedecimpunctata_586 | 0.182 |
| Coccinella_undecimpunctata_282 | Tytthaspis_sedecimpunctata_587 | 0.182 |
| Coccinella_undecimpunctata_283 | Tytthaspis_sedecimpunctata_587 | 0.182 |
| Coccinella_undecimpunctata_284 | Tytthaspis_sedecimpunctata_587 | 0.182 |
| Anatis_labiculata_123 | Tytthaspis_sedecimpunctata_590 | 0.182 |
| Anatis_labiculata_124 | Tytthaspis_sedecimpunctata_590 | 0.182 |
| Anatis_labiculata_125 | Tytthaspis_sedecimpunctata_590 | 0.182 |
| Anatis_labiculata_127 | Tytthaspis_sedecimpunctata_590 | 0.182 |
| Scymnus_camptodromus_712 | Tytthaspis_sedecimpunctata_590 | 0.182 |
| Coccinella_septempunctata_250 | Tytthaspis_sedecimpunctata_591 | 0.182 |
| Coccinella_septempunctata_251 | Tytthaspis_sedecimpunctata_591 | 0.182 |
| Anatis_labiculata_126 | Tytthaspis_sedecimpunctata_592 | 0.182 |
| Scymnus_camptodromus_712 | Tytthaspis_sedecimpunctata_592 | 0.182 |
| Anatis_labiculata_123 | Tytthaspis_sedecimpunctata_593 | 0.182 |
| Anatis_labiculata_124 | Tytthaspis_sedecimpunctata_593 | 0.182 |
| Anatis_labiculata_125 | Tytthaspis_sedecimpunctata_593 | 0.182 |
| Anatis_labiculata_127 | Tytthaspis_sedecimpunctata_593 | 0.182 |
| Scymnus_camptodromus_712 | Tytthaspis_sedecimpunctata_593 | 0.182 |
| Anatis_labiculata_123 | Tytthaspis_sedecimpunctata_594 | 0.182 |
| Anatis_labiculata_124 | Tytthaspis_sedecimpunctata_594 | 0.182 |
| Anatis_labiculata_125 | Tytthaspis_sedecimpunctata_594 | 0.182 |
| Anatis_labiculata_127 | Tytthaspis_sedecimpunctata_594 | 0.182 |
| Scymnus_camptodromus_712 | Tytthaspis_sedecimpunctata_594 | 0.182 |
| Anatis_labiculata_123 | Tytthaspis_sedecimpunctata_595 | 0.182 |
| Anatis_labiculata_124 | Tytthaspis_sedecimpunctata_595 | 0.182 |
| Anatis_labiculata_125 | Tytthaspis_sedecimpunctata_595 | 0.182 |
| Anatis_labiculata_127 | Tytthaspis_sedecimpunctata_595 | 0.182 |
| Scymnus_camptodromus_712 | Tytthaspis_sedecimpunctata_595 | 0.182 |
| Hyperaspis_lateralis_654 | Scymnus_suturalis_834 | 0.182 |
| Hyperaspis_lateralis_657 | Scymnus_suturalis_834 | 0.182 |
| Hyperaspis_lateralis_655 | Scymnus_suturalis_838 | 0.182 |
| Hyperaspis_lateralis_655 | Scymnus_suturalis_840 | 0.182 |
| Coccinella_quinquepunctata_233 | Tytthaspis_sedecimpunctata_588 | 0.182 |
| Macronaemia_episcopalis_478 | Scymnus_suturalis_833 | 0.182 |
| Hyperaspis_lateralis_655 | Stethorus_punctillum_849 | 0.182 |
| Coccinella_undecimpunctata_282 | Tytthaspis_sedecimpunctata_577 | 0.182 |
| Coccinella_undecimpunctata_283 | Tytthaspis_sedecimpunctata_577 | 0.182 |
| Coccinella_undecimpunctata_284 | Tytthaspis_sedecimpunctata_577 | 0.182 |
| Olla_vnigrum_530 | Tytthaspis_sedecimpunctata_577 | 0.182 |
| Coccinella_undecimpunctata_282 | Tytthaspis_sedecimpunctata_578 | 0.182 |
| Coccinella_undecimpunctata_283 | Tytthaspis_sedecimpunctata_578 | 0.182 |
| Coccinella_undecimpunctata_284 | Tytthaspis_sedecimpunctata_578 | 0.182 |
| Olla_vnigrum_530 | Tytthaspis_sedecimpunctata_578 | 0.182 |
| Coccinella_undecimpunctata_282 | Tytthaspis_sedecimpunctata_579 | 0.182 |
| Coccinella_undecimpunctata_283 | Tytthaspis_sedecimpunctata_579 | 0.182 |
| Coccinella_undecimpunctata_284 | Tytthaspis_sedecimpunctata_579 | 0.182 |
| Olla_vnigrum_530 | Tytthaspis_sedecimpunctata_579 | 0.182 |

| | | |
|---|---|---|
| Anatis_labiculata_126 | Tytthaspis_sedecimpunctata_580 | 0.182 |
| Olla_vnigrum_525 | Tytthaspis_sedecimpunctata_580 | 0.182 |
| Olla_vnigrum_529 | Tytthaspis_sedecimpunctata_580 | 0.182 |
| Olla_vnigrum_531 | Tytthaspis_sedecimpunctata_580 | 0.182 |
| Coccinella_undecimpunctata_282 | Tytthaspis_sedecimpunctata_581 | 0.182 |
| Coccinella_undecimpunctata_283 | Tytthaspis_sedecimpunctata_581 | 0.182 |
| Coccinella_undecimpunctata_284 | Tytthaspis_sedecimpunctata_581 | 0.182 |
| Olla_vnigrum_530 | Tytthaspis_sedecimpunctata_581 | 0.182 |
| Coccinella_undecimpunctata_282 | Tytthaspis_sedecimpunctata_582 | 0.182 |
| Coccinella_undecimpunctata_283 | Tytthaspis_sedecimpunctata_582 | 0.182 |
| Coccinella_undecimpunctata_284 | Tytthaspis_sedecimpunctata_582 | 0.182 |
| Olla_vnigrum_530 | Tytthaspis_sedecimpunctata_582 | 0.182 |
| Coccinella_undecimpunctata_285 | Tytthaspis_sedecimpunctata_583 | 0.182 |
| Coccinella_undecimpunctata_286 | Tytthaspis_sedecimpunctata_583 | 0.182 |
| Coccinella_undecimpunctata_288 | Tytthaspis_sedecimpunctata_583 | 0.182 |
| Psyllobora_renifer_550 | Tytthaspis_sedecimpunctata_583 | 0.182 |
| Anatis_labiculata_123 | Tytthaspis_sedecimpunctata_584 | 0.182 |
| Anatis_labiculata_124 | Tytthaspis_sedecimpunctata_584 | 0.182 |
| Anatis_labiculata_125 | Tytthaspis_sedecimpunctata_584 | 0.182 |
| Anatis_labiculata_127 | Tytthaspis_sedecimpunctata_584 | 0.182 |
| Coccinella_undecimpunctata_282 | Tytthaspis_sedecimpunctata_585 | 0.182 |
| Coccinella_undecimpunctata_283 | Tytthaspis_sedecimpunctata_585 | 0.182 |
| Coccinella_undecimpunctata_284 | Tytthaspis_sedecimpunctata_585 | 0.182 |
| Scymnus_camptodromus_707 | Tytthaspis_sedecimpunctata_589 | 0.182 |
| Scymnus_camptodromus_708 | Tytthaspis_sedecimpunctata_589 | 0.182 |
| Scymnus_camptodromus_709 | Tytthaspis_sedecimpunctata_589 | 0.182 |
| Scymnus_camptodromus_710 | Tytthaspis_sedecimpunctata_589 | 0.182 |
| Coccinella_undecimpunctata_282 | Tytthaspis_sedecimpunctata_590 | 0.182 |
| Coccinella_undecimpunctata_283 | Tytthaspis_sedecimpunctata_590 | 0.182 |
| Coccinella_undecimpunctata_284 | Tytthaspis_sedecimpunctata_590 | 0.182 |
| Olla_vnigrum_530 | Tytthaspis_sedecimpunctata_590 | 0.182 |
| Olla_vnigrum_525 | Tytthaspis_sedecimpunctata_591 | 0.182 |
| Olla_vnigrum_529 | Tytthaspis_sedecimpunctata_591 | 0.182 |
| Olla_vnigrum_531 | Tytthaspis_sedecimpunctata_591 | 0.182 |
| Coccinella_undecimpunctata_282 | Tytthaspis_sedecimpunctata_592 | 0.182 |
| Coccinella_undecimpunctata_283 | Tytthaspis_sedecimpunctata_592 | 0.182 |
| Coccinella_undecimpunctata_284 | Tytthaspis_sedecimpunctata_592 | 0.182 |
| Olla_vnigrum_530 | Tytthaspis_sedecimpunctata_592 | 0.182 |
| Coccinella_undecimpunctata_282 | Tytthaspis_sedecimpunctata_594 | 0.182 |
| Coccinella_undecimpunctata_283 | Tytthaspis_sedecimpunctata_594 | 0.182 |
| Coccinella_undecimpunctata_284 | Tytthaspis_sedecimpunctata_594 | 0.182 |
| Olla_vnigrum_530 | Tytthaspis_sedecimpunctata_594 | 0.182 |
| Coccinella_undecimpunctata_282 | Tytthaspis_sedecimpunctata_595 | 0.182 |
| Coccinella_undecimpunctata_283 | Tytthaspis_sedecimpunctata_595 | 0.182 |
| Coccinella_undecimpunctata_284 | Tytthaspis_sedecimpunctata_595 | 0.182 |
| Olla_vnigrum_530 | Tytthaspis_sedecimpunctata_595 | 0.182 |
| Chilocorus_nigritus_25 | Scymnus_sinuanodulus_828 | 0.182 |
| Hyperaspis_lateralis_655 | Scymnus_suturalis_834 | 0.182 |
| Coccidula_scutellata_84 | Scymnus_suturalis_833 | 0.182 |
| Coccinella_quinquepunctata_233 | Tytthaspis_sedecimpunctata_583 | 0.182 |
| Coccinella_quinquepunctata_234 | Tytthaspis_sedecimpunctata_583 | 0.182 |
| Scymnus_nubilus_800 | Subcoccinella_vigintiquatuorpunctata_624 | 0.182 |
| Scymnus_nubilus_801 | Subcoccinella_vigintiquatuorpunctata_624 | 0.182 |
| Scymnus_nubilus_805 | Subcoccinella_vigintiquatuorpunctata_624 | 0.182 |
| Scymnus_nubilus_806 | Subcoccinella_vigintiquatuorpunctata_624 | 0.182 |
| Scymnus_nubilus_807 | Subcoccinella_vigintiquatuorpunctata_624 | 0.182 |

| | | |
|---|---|---|
| Scymnus_nubilus_810 | Subcoccinella_vigintiquatuorpunctata_624 | 0.182 |
| Coccinella_undecimpunctata_285 | Tytthaspis_sedecimpunctata_580 | 0.182 |
| Coccinella_undecimpunctata_286 | Tytthaspis_sedecimpunctata_580 | 0.182 |
| Coccinella_undecimpunctata_288 | Tytthaspis_sedecimpunctata_580 | 0.182 |
| Psyllobora_renifer_550 | Tytthaspis_sedecimpunctata_580 | 0.182 |
| Olla_vnigrum_526 | Tytthaspis_sedecimpunctata_583 | 0.182 |
| Olla_vnigrum_530 | Tytthaspis_sedecimpunctata_584 | 0.182 |
| Psyllobora_renifer_550 | Tytthaspis_sedecimpunctata_584 | 0.182 |
| Olla_vnigrum_525 | Tytthaspis_sedecimpunctata_585 | 0.182 |
| Olla_vnigrum_529 | Tytthaspis_sedecimpunctata_585 | 0.182 |
| Olla_vnigrum_531 | Tytthaspis_sedecimpunctata_585 | 0.182 |
| Scymnus_camptodromus_707 | Tytthaspis_sedecimpunctata_586 | 0.182 |
| Scymnus_camptodromus_708 | Tytthaspis_sedecimpunctata_586 | 0.182 |
| Scymnus_camptodromus_709 | Tytthaspis_sedecimpunctata_586 | 0.182 |
| Scymnus_camptodromus_710 | Tytthaspis_sedecimpunctata_586 | 0.182 |
| Scymnus_auritus_702 | Tytthaspis_sedecimpunctata_587 | 0.182 |
| Coccinella_undecimpunctata_291 | Tytthaspis_sedecimpunctata_588 | 0.182 |
| Scymnus_camptodromus_707 | Tytthaspis_sedecimpunctata_588 | 0.182 |
| Scymnus_camptodromus_708 | Tytthaspis_sedecimpunctata_588 | 0.182 |
| Scymnus_camptodromus_709 | Tytthaspis_sedecimpunctata_588 | 0.182 |
| Scymnus_camptodromus_710 | Tytthaspis_sedecimpunctata_588 | 0.182 |
| Coccinella_undecimpunctata_282 | Tytthaspis_sedecimpunctata_591 | 0.182 |
| Coccinella_undecimpunctata_283 | Tytthaspis_sedecimpunctata_591 | 0.182 |
| Coccinella_undecimpunctata_284 | Tytthaspis_sedecimpunctata_591 | 0.182 |
| Psyllobora_vigintimaculata_574 | Tytthaspis_sedecimpunctata_591 | 0.182 |
| Olla_vnigrum_525 | Tytthaspis_sedecimpunctata_593 | 0.182 |
| Olla_vnigrum_529 | Tytthaspis_sedecimpunctata_593 | 0.182 |
| Olla_vnigrum_531 | Tytthaspis_sedecimpunctata_593 | 0.182 |
| Nephus_quadrimaculatus_669 | Scymnus_rubromaculatus_822 | 0.182 |
| Nephus_quadrimaculatus_671 | Scymnus_rubromaculatus_822 | 0.182 |
| Nephus_quadrimaculatus_672 | Scymnus_rubromaculatus_822 | 0.182 |
| Scymnus_auritus_703 | Scymnus_rubromaculatus_822 | 0.182 |
| Nephus_quadrimaculatus_669 | Scymnus_rubromaculatus_823 | 0.182 |
| Nephus_quadrimaculatus_671 | Scymnus_rubromaculatus_823 | 0.182 |
| Nephus_quadrimaculatus_672 | Scymnus_rubromaculatus_823 | 0.182 |
| Scymnus_auritus_703 | Scymnus_rubromaculatus_823 | 0.182 |
| Nephus_quadrimaculatus_669 | Scymnus_rubromaculatus_824 | 0.182 |
| Nephus_quadrimaculatus_671 | Scymnus_rubromaculatus_824 | 0.182 |
| Nephus_quadrimaculatus_672 | Scymnus_rubromaculatus_824 | 0.182 |
| Scymnus_auritus_703 | Scymnus_rubromaculatus_824 | 0.182 |
| Nephus_quadrimaculatus_669 | Scymnus_rubromaculatus_825 | 0.182 |
| Nephus_quadrimaculatus_671 | Scymnus_rubromaculatus_825 | 0.182 |
| Nephus_quadrimaculatus_672 | Scymnus_rubromaculatus_825 | 0.182 |
| Scymnus_auritus_703 | Scymnus_rubromaculatus_825 | 0.182 |
| Nephus_quadrimaculatus_669 | Scymnus_rubromaculatus_826 | 0.182 |
| Nephus_quadrimaculatus_671 | Scymnus_rubromaculatus_826 | 0.182 |
| Nephus_quadrimaculatus_672 | Scymnus_rubromaculatus_826 | 0.182 |
| Scymnus_auritus_703 | Scymnus_rubromaculatus_826 | 0.182 |
| Nephus_quadrimaculatus_669 | Scymnus_rubromaculatus_827 | 0.182 |
| Nephus_quadrimaculatus_671 | Scymnus_rubromaculatus_827 | 0.182 |
| Nephus_quadrimaculatus_672 | Scymnus_rubromaculatus_827 | 0.182 |
| Scymnus_auritus_703 | Scymnus_rubromaculatus_827 | 0.182 |
| Coccidula_scutellata_84 | Scymnus_suturalis_843 | 0.182 |
| Coccidula_scutellata_84 | Scymnus_suturalis_845 | 0.182 |
| Chilocorus_nigritus_29 | Scymnus_suturalis_833 | 0.182 |
| Coccinella_quinquepunctata_233 | Tytthaspis_sedecimpunctata_591 | 0.182 |

| | | |
|---|---|---|
| Coccinella_undecimpunctata_287 | Scymnus_sinuanodulus_829 | 0.182 |
| Scymnus_auritus_702 | Tytthaspis_sedecimpunctata_577 | 0.182 |
| Scymnus_auritus_702 | Tytthaspis_sedecimpunctata_578 | 0.182 |
| Scymnus_auritus_702 | Tytthaspis_sedecimpunctata_579 | 0.182 |
| Scymnus_auritus_702 | Tytthaspis_sedecimpunctata_581 | 0.182 |
| Scymnus_auritus_702 | Tytthaspis_sedecimpunctata_582 | 0.182 |
| Scymnus_auritus_702 | Tytthaspis_sedecimpunctata_590 | 0.182 |
| Scymnus_auritus_700 | Tytthaspis_sedecimpunctata_593 | 0.182 |
| Scymnus_auritus_702 | Tytthaspis_sedecimpunctata_594 | 0.182 |
| Scymnus_auritus_702 | Tytthaspis_sedecimpunctata_595 | 0.182 |
| Brumus_quadripustulatus_12 | Scymnus_rubromaculatus_822 | 0.182 |
| Scymnus_auritus_702 | Scymnus_rubromaculatus_822 | 0.182 |
| Brumus_quadripustulatus_12 | Scymnus_rubromaculatus_823 | 0.182 |
| Scymnus_auritus_702 | Scymnus_rubromaculatus_823 | 0.182 |
| Brumus_quadripustulatus_12 | Scymnus_rubromaculatus_824 | 0.182 |
| Scymnus_auritus_702 | Scymnus_rubromaculatus_824 | 0.182 |
| Brumus_quadripustulatus_12 | Scymnus_rubromaculatus_825 | 0.182 |
| Scymnus_auritus_702 | Scymnus_rubromaculatus_825 | 0.182 |
| Brumus_quadripustulatus_12 | Scymnus_rubromaculatus_826 | 0.182 |
| Scymnus_auritus_702 | Scymnus_rubromaculatus_826 | 0.182 |
| Brumus_quadripustulatus_12 | Scymnus_rubromaculatus_827 | 0.182 |
| Scymnus_auritus_702 | Scymnus_rubromaculatus_827 | 0.182 |
| Coccidula_scutellata_83 | Scymnus_sinuanodulus_831 | 0.182 |
| Brumoides_suturalis_1 | Scymnus_suturalis_841 | 0.182 |
| Brumoides_suturalis_1 | Scymnus_suturalis_842 | 0.182 |
| Exochomus_nigromaculatus_49 | Scymnus_suturalis_843 | 0.182 |
| Brumoides_suturalis_1 | Scymnus_suturalis_844 | 0.182 |
| Exochomus_nigromaculatus_49 | Scymnus_suturalis_845 | 0.182 |
| Brumoides_suturalis_1 | Scymnus_suturalis_846 | 0.182 |
| Brumoides_suturalis_1 | Scymnus_suturalis_847 | 0.182 |
| Brumoides_suturalis_1 | Scymnus_suturalis_848 | 0.182 |
| Rhyzobius_litura_97 | Subcoccinella_vigintiquatuorpunctata_627 | 0.182 |
| Rhyzobius_litura_96 | Subcoccinella_vigintiquatuorpunctata_630 | 0.182 |
| Hippodamia_undecimnotata_460 | Tytthaspis_sedecimpunctata_577 | 0.182 |
| Hippodamia_undecimnotata_461 | Tytthaspis_sedecimpunctata_577 | 0.182 |
| Hippodamia_undecimnotata_462 | Tytthaspis_sedecimpunctata_577 | 0.182 |
| Hippodamia_undecimnotata_463 | Tytthaspis_sedecimpunctata_577 | 0.182 |
| Hippodamia_undecimnotata_464 | Tytthaspis_sedecimpunctata_577 | 0.182 |
| Hippodamia_undecimnotata_465 | Tytthaspis_sedecimpunctata_577 | 0.182 |
| Hippodamia_undecimnotata_466 | Tytthaspis_sedecimpunctata_577 | 0.182 |
| Hippodamia_undecimnotata_460 | Tytthaspis_sedecimpunctata_578 | 0.182 |
| Hippodamia_undecimnotata_461 | Tytthaspis_sedecimpunctata_578 | 0.182 |
| Hippodamia_undecimnotata_462 | Tytthaspis_sedecimpunctata_578 | 0.182 |
| Hippodamia_undecimnotata_463 | Tytthaspis_sedecimpunctata_578 | 0.182 |
| Hippodamia_undecimnotata_464 | Tytthaspis_sedecimpunctata_578 | 0.182 |
| Hippodamia_undecimnotata_465 | Tytthaspis_sedecimpunctata_578 | 0.182 |
| Hippodamia_undecimnotata_466 | Tytthaspis_sedecimpunctata_578 | 0.182 |
| Hippodamia_undecimnotata_460 | Tytthaspis_sedecimpunctata_579 | 0.182 |
| Hippodamia_undecimnotata_461 | Tytthaspis_sedecimpunctata_579 | 0.182 |
| Hippodamia_undecimnotata_462 | Tytthaspis_sedecimpunctata_579 | 0.182 |
| Hippodamia_undecimnotata_463 | Tytthaspis_sedecimpunctata_579 | 0.182 |
| Hippodamia_undecimnotata_464 | Tytthaspis_sedecimpunctata_579 | 0.182 |
| Hippodamia_undecimnotata_465 | Tytthaspis_sedecimpunctata_579 | 0.182 |
| Hippodamia_undecimnotata_466 | Tytthaspis_sedecimpunctata_579 | 0.182 |
| Hippodamia_undecimnotata_460 | Tytthaspis_sedecimpunctata_581 | 0.182 |
| Hippodamia_undecimnotata_461 | Tytthaspis_sedecimpunctata_581 | 0.182 |

| | | |
|---|---|---|
| Hippodamia_undecimnotata_462 | Tytthaspis_sedecimpunctata_581 | 0.182 |
| Hippodamia_undecimnotata_463 | Tytthaspis_sedecimpunctata_581 | 0.182 |
| Hippodamia_undecimnotata_464 | Tytthaspis_sedecimpunctata_581 | 0.182 |
| Hippodamia_undecimnotata_465 | Tytthaspis_sedecimpunctata_581 | 0.182 |
| Hippodamia_undecimnotata_466 | Tytthaspis_sedecimpunctata_581 | 0.182 |
| Hippodamia_undecimnotata_460 | Tytthaspis_sedecimpunctata_582 | 0.182 |
| Hippodamia_undecimnotata_461 | Tytthaspis_sedecimpunctata_582 | 0.182 |
| Hippodamia_undecimnotata_462 | Tytthaspis_sedecimpunctata_582 | 0.182 |
| Hippodamia_undecimnotata_463 | Tytthaspis_sedecimpunctata_582 | 0.182 |
| Hippodamia_undecimnotata_464 | Tytthaspis_sedecimpunctata_582 | 0.182 |
| Hippodamia_undecimnotata_465 | Tytthaspis_sedecimpunctata_582 | 0.182 |
| Hippodamia_undecimnotata_466 | Tytthaspis_sedecimpunctata_582 | 0.182 |
| Hippodamia_notata_422 | Tytthaspis_sedecimpunctata_587 | 0.182 |
| Hippodamia_notata_423 | Tytthaspis_sedecimpunctata_587 | 0.182 |
| Hippodamia_notata_424 | Tytthaspis_sedecimpunctata_587 | 0.182 |
| Hippodamia_notata_426 | Tytthaspis_sedecimpunctata_587 | 0.182 |
| Hippodamia_notata_427 | Tytthaspis_sedecimpunctata_587 | 0.182 |
| Hippodamia_undecimnotata_460 | Tytthaspis_sedecimpunctata_590 | 0.182 |
| Hippodamia_undecimnotata_461 | Tytthaspis_sedecimpunctata_590 | 0.182 |
| Hippodamia_undecimnotata_462 | Tytthaspis_sedecimpunctata_590 | 0.182 |
| Hippodamia_undecimnotata_463 | Tytthaspis_sedecimpunctata_590 | 0.182 |
| Hippodamia_undecimnotata_464 | Tytthaspis_sedecimpunctata_590 | 0.182 |
| Hippodamia_undecimnotata_465 | Tytthaspis_sedecimpunctata_590 | 0.182 |
| Hippodamia_undecimnotata_466 | Tytthaspis_sedecimpunctata_590 | 0.182 |
| Hippodamia_undecimnotata_460 | Tytthaspis_sedecimpunctata_592 | 0.182 |
| Hippodamia_undecimnotata_461 | Tytthaspis_sedecimpunctata_592 | 0.182 |
| Hippodamia_undecimnotata_462 | Tytthaspis_sedecimpunctata_592 | 0.182 |
| Hippodamia_undecimnotata_463 | Tytthaspis_sedecimpunctata_592 | 0.182 |
| Hippodamia_undecimnotata_464 | Tytthaspis_sedecimpunctata_592 | 0.182 |
| Hippodamia_undecimnotata_465 | Tytthaspis_sedecimpunctata_592 | 0.182 |
| Hippodamia_undecimnotata_466 | Tytthaspis_sedecimpunctata_592 | 0.182 |
| Hippodamia_undecimnotata_460 | Tytthaspis_sedecimpunctata_594 | 0.182 |
| Hippodamia_undecimnotata_461 | Tytthaspis_sedecimpunctata_594 | 0.182 |
| Hippodamia_undecimnotata_462 | Tytthaspis_sedecimpunctata_594 | 0.182 |
| Hippodamia_undecimnotata_463 | Tytthaspis_sedecimpunctata_594 | 0.182 |
| Hippodamia_undecimnotata_464 | Tytthaspis_sedecimpunctata_594 | 0.182 |
| Hippodamia_undecimnotata_465 | Tytthaspis_sedecimpunctata_594 | 0.182 |
| Hippodamia_undecimnotata_466 | Tytthaspis_sedecimpunctata_594 | 0.182 |
| Hippodamia_undecimnotata_460 | Tytthaspis_sedecimpunctata_595 | 0.182 |
| Hippodamia_undecimnotata_461 | Tytthaspis_sedecimpunctata_595 | 0.182 |
| Hippodamia_undecimnotata_462 | Tytthaspis_sedecimpunctata_595 | 0.182 |
| Hippodamia_undecimnotata_463 | Tytthaspis_sedecimpunctata_595 | 0.182 |
| Hippodamia_undecimnotata_464 | Tytthaspis_sedecimpunctata_595 | 0.182 |
| Hippodamia_undecimnotata_465 | Tytthaspis_sedecimpunctata_595 | 0.182 |
| Hippodamia_undecimnotata_466 | Tytthaspis_sedecimpunctata_595 | 0.182 |
| Scymnus_frontalis_734 | Scymnus_sinuanodulus_830 | 0.182 |
| Scymnus_frontalis_742 | Scymnus_sinuanodulus_830 | 0.182 |
| Scymnus_frontalis_745 | Scymnus_sinuanodulus_830 | 0.182 |
| Brumoides_suturalis_1 | Scymnus_suturalis_832 | 0.182 |
| Brumoides_suturalis_1 | Scymnus_suturalis_835 | 0.182 |
| Brumoides_suturalis_1 | Scymnus_suturalis_836 | 0.182 |
| Brumoides_suturalis_2 | Scymnus_suturalis_838 | 0.182 |
| Brumoides_suturalis_1 | Scymnus_suturalis_839 | 0.182 |
| Brumoides_suturalis_2 | Scymnus_suturalis_840 | 0.182 |
| Scymnus_nigrinus_787 | Tytthaspis_sedecimpunctata_588 | 0.182 |
| Chilocorus_bipustulatus_18 | Scymnus_sinuanodulus_831 | 0.182 |

| | | |
|---|---|---|
| Chilocorus_bipustulatus_19 | Scymnus_sinuanodulus_831 | 0.182 |
| Chilocorus_bipustulatus_20 | Scymnus_sinuanodulus_831 | 0.182 |
| Chilocorus_bipustulatus_22 | Scymnus_sinuanodulus_831 | 0.182 |
| Chilocorus_bipustulatus_24 | Scymnus_sinuanodulus_831 | 0.182 |
| Chilocorus_stigma_43 | Scymnus_sinuanodulus_831 | 0.182 |
| Scymnus_nubilus_799 | Scymnus_suturalis_841 | 0.182 |
| Scymnus_nubilus_799 | Scymnus_suturalis_842 | 0.182 |
| Scymnus_nubilus_799 | Scymnus_suturalis_844 | 0.182 |
| Scymnus_nubilus_799 | Scymnus_suturalis_846 | 0.182 |
| Scymnus_nubilus_799 | Scymnus_suturalis_847 | 0.182 |
| Scymnus_nubilus_799 | Scymnus_suturalis_848 | 0.182 |
| Scymnus_haemorrhoidalis_747 | Subcoccinella_vigintiquatuorpunctata_630 | 0.182 |
| Scymnus_haemorrhoidalis_748 | Subcoccinella_vigintiquatuorpunctata_630 | 0.182 |
| Hippodamia_undecimnotata_460 | Tytthaspis_sedecimpunctata_584 | 0.182 |
| Hippodamia_undecimnotata_461 | Tytthaspis_sedecimpunctata_584 | 0.182 |
| Hippodamia_undecimnotata_462 | Tytthaspis_sedecimpunctata_584 | 0.182 |
| Hippodamia_undecimnotata_463 | Tytthaspis_sedecimpunctata_584 | 0.182 |
| Hippodamia_undecimnotata_464 | Tytthaspis_sedecimpunctata_584 | 0.182 |
| Hippodamia_undecimnotata_465 | Tytthaspis_sedecimpunctata_584 | 0.182 |
| Hippodamia_undecimnotata_466 | Tytthaspis_sedecimpunctata_584 | 0.182 |
| Neoharmonia_venusta_520 | Tytthaspis_sedecimpunctata_587 | 0.182 |
| Cycloneda_sanguinea_342 | Tytthaspis_sedecimpunctata_593 | 0.182 |
| Coccinella_transversoguttata_265 | Vibidia_duodecimguttata_597 | 0.182 |
| Coccinella_transversoguttata_266 | Vibidia_duodecimguttata_597 | 0.182 |
| Coccinella_transversoguttata_265 | Vibidia_duodecimguttata_600 | 0.182 |
| Coccinella_transversoguttata_266 | Vibidia_duodecimguttata_600 | 0.182 |
| Hyperaspis_reppensis_667 | Scymnus_rubromaculatus_822 | 0.182 |
| Hyperaspis_reppensis_667 | Scymnus_rubromaculatus_823 | 0.182 |
| Hyperaspis_reppensis_667 | Scymnus_rubromaculatus_824 | 0.182 |
| Hyperaspis_reppensis_667 | Scymnus_rubromaculatus_825 | 0.182 |
| Hyperaspis_reppensis_667 | Scymnus_rubromaculatus_826 | 0.182 |
| Hyperaspis_reppensis_667 | Scymnus_rubromaculatus_827 | 0.182 |
| Scymnus_pallipediformis_812 | Scymnus_sinuanodulus_828 | 0.182 |
| Scymnus_nubilus_799 | Scymnus_suturalis_832 | 0.182 |
| Scymnus_nubilus_799 | Scymnus_suturalis_835 | 0.182 |
| Scymnus_nubilus_799 | Scymnus_suturalis_836 | 0.182 |
| Brumoides_suturalis_2 | Scymnus_suturalis_837 | 0.182 |
| Scymnus_nubilus_799 | Scymnus_suturalis_839 | 0.182 |
| Coccinella_undecimpunctata_289 | Tytthaspis_sedecimpunctata_588 | 0.182 |
| Coccinella_undecimpunctata_290 | Tytthaspis_sedecimpunctata_588 | 0.182 |
| Chilocorus_nigritus_29 | Scymnus_rubromaculatus_822 | 0.182 |
| Chilocorus_nigritus_29 | Scymnus_rubromaculatus_823 | 0.182 |
| Chilocorus_nigritus_29 | Scymnus_rubromaculatus_824 | 0.182 |
| Chilocorus_nigritus_29 | Scymnus_rubromaculatus_825 | 0.182 |
| Chilocorus_nigritus_29 | Scymnus_rubromaculatus_826 | 0.182 |
| Chilocorus_nigritus_29 | Scymnus_rubromaculatus_827 | 0.182 |
| Chilocorus_nigritus_27 | Scymnus_sinuanodulus_831 | 0.182 |
| Brumus_quadripustulatus_5 | Scymnus_sinuanodulus_829 | 0.182 |
| Scymnus_haemorrhoidalis_749 | Subcoccinella_vigintiquatuorpunctata_620 | 0.182 |
| Scymnus_haemorrhoidalis_750 | Subcoccinella_vigintiquatuorpunctata_620 | 0.182 |
| Scymnus_haemorrhoidalis_749 | Subcoccinella_vigintiquatuorpunctata_621 | 0.182 |
| Scymnus_haemorrhoidalis_750 | Subcoccinella_vigintiquatuorpunctata_621 | 0.182 |
| Scymnus_haemorrhoidalis_749 | Subcoccinella_vigintiquatuorpunctata_628 | 0.182 |
| Scymnus_haemorrhoidalis_750 | Subcoccinella_vigintiquatuorpunctata_628 | 0.182 |
| Scymnus_haemorrhoidalis_749 | Subcoccinella_vigintiquatuorpunctata_629 | 0.182 |
| Scymnus_haemorrhoidalis_750 | Subcoccinella_vigintiquatuorpunctata_629 | 0.182 |

| | | |
|---|---|---|
| Scymnus_haemorrhoidalis_749 | Subcoccinella_vigintiquatuorpunctata_630 | 0.182 |
| Scymnus_haemorrhoidalis_750 | Subcoccinella_vigintiquatuorpunctata_630 | 0.182 |
| Scymnus_haemorrhoidalis_749 | Subcoccinella_vigintiquatuorpunctata_631 | 0.182 |
| Scymnus_haemorrhoidalis_750 | Subcoccinella_vigintiquatuorpunctata_631 | 0.182 |
| Neoharmonia_venusta_521 | Tytthaspis_sedecimpunctata_587 | 0.182 |
| Cycloneda_sanguinea_342 | Tytthaspis_sedecimpunctata_591 | 0.182 |
| Anisosticta_novemdecimpunctata_174 | Vibidia_duodecimguttata_597 | 0.182 |
| Coccinella_transversoguttata_265 | Vibidia_duodecimguttata_598 | 0.182 |
| Coccinella_transversoguttata_266 | Vibidia_duodecimguttata_598 | 0.182 |
| Anisosticta_novemdecimpunctata_174 | Vibidia_duodecimguttata_600 | 0.182 |
| Hyperaspis_proba_661 | Subcoccinella_vigintiquatuorpunctata_620 | 0.182 |
| Hyperaspis_proba_661 | Subcoccinella_vigintiquatuorpunctata_621 | 0.182 |
| Hyperaspis_campestris_651 | Subcoccinella_vigintiquatuorpunctata_626 | 0.182 |
| Hyperaspis_campestris_652 | Subcoccinella_vigintiquatuorpunctata_626 | 0.182 |
| Hyperaspis_campestris_653 | Subcoccinella_vigintiquatuorpunctata_626 | 0.182 |
| Hyperaspis_proba_661 | Subcoccinella_vigintiquatuorpunctata_628 | 0.182 |
| Hyperaspis_proba_661 | Subcoccinella_vigintiquatuorpunctata_629 | 0.182 |
| Hyperaspis_proba_661 | Subcoccinella_vigintiquatuorpunctata_631 | 0.182 |
| Aphidecta_obliterata_175 | Vibidia_duodecimguttata_596 | 0.182 |
| Aphidecta_obliterata_177 | Vibidia_duodecimguttata_596 | 0.182 |
| Aphidecta_obliterata_180 | Vibidia_duodecimguttata_596 | 0.182 |
| Aphidecta_obliterata_181 | Vibidia_duodecimguttata_596 | 0.182 |
| Aphidecta_obliterata_182 | Vibidia_duodecimguttata_596 | 0.182 |
| Aphidecta_obliterata_184 | Vibidia_duodecimguttata_596 | 0.182 |
| Aphidecta_obliterata_175 | Vibidia_duodecimguttata_598 | 0.182 |
| Aphidecta_obliterata_176 | Vibidia_duodecimguttata_598 | 0.182 |
| Aphidecta_obliterata_177 | Vibidia_duodecimguttata_598 | 0.182 |
| Aphidecta_obliterata_180 | Vibidia_duodecimguttata_598 | 0.182 |
| Aphidecta_obliterata_182 | Vibidia_duodecimguttata_598 | 0.182 |
| Aphidecta_obliterata_184 | Vibidia_duodecimguttata_598 | 0.182 |
| Aphidecta_obliterata_179 | Vibidia_duodecimguttata_599 | 0.182 |
| Aphidecta_obliterata_179 | Vibidia_duodecimguttata_601 | 0.182 |
| Aphidecta_obliterata_179 | Vibidia_duodecimguttata_602 | 0.182 |
| Aphidecta_obliterata_175 | Vibidia_duodecimguttata_603 | 0.182 |
| Aphidecta_obliterata_177 | Vibidia_duodecimguttata_603 | 0.182 |
| Aphidecta_obliterata_180 | Vibidia_duodecimguttata_603 | 0.182 |
| Aphidecta_obliterata_181 | Vibidia_duodecimguttata_603 | 0.182 |
| Aphidecta_obliterata_182 | Vibidia_duodecimguttata_603 | 0.182 |
| Aphidecta_obliterata_184 | Vibidia_duodecimguttata_603 | 0.182 |
| Scymnus_frontalis_735 | Scymnus_sinuanodulus_830 | 0.182 |
| Calvia_quatuordecimguttata_202 | Scymnus_sinuanodulus_831 | 0.182 |
| Calvia_quatuordecimguttata_210 | Scymnus_sinuanodulus_831 | 0.182 |
| Myzia_pullata_519 | Vibidia_duodecimguttata_599 | 0.182 |
| Myzia_pullata_519 | Vibidia_duodecimguttata_601 | 0.182 |
| Myzia_pullata_519 | Vibidia_duodecimguttata_602 | 0.182 |
| Hyperaspis_reppensis_665 | Scymnus_sinuanodulus_830 | 0.182 |
| Scymnus_rubromaculatus_813 | Scymnus_sinuanodulus_830 | 0.182 |
| Scymnus_rubromaculatus_815 | Scymnus_sinuanodulus_830 | 0.182 |
| Scymnus_rubromaculatus_816 | Scymnus_sinuanodulus_830 | 0.182 |
| Scymnus_rubromaculatus_817 | Scymnus_sinuanodulus_830 | 0.182 |
| Scymnus_rubromaculatus_818 | Scymnus_sinuanodulus_830 | 0.182 |
| Scymnus_rubromaculatus_819 | Scymnus_sinuanodulus_830 | 0.182 |
| Scymnus_rubromaculatus_820 | Scymnus_sinuanodulus_830 | 0.182 |
| Scymnus_rubromaculatus_821 | Scymnus_sinuanodulus_830 | 0.182 |
| Scymnus_rubromaculatus_822 | Scymnus_sinuanodulus_830 | 0.182 |
| Scymnus_rubromaculatus_823 | Scymnus_sinuanodulus_830 | 0.182 |

| | | |
|---|---|---|
| Scymnus_rubromaculatus_824 | Scymnus_sinuanodulus_830 | 0.182 |
| Scymnus_rubromaculatus_825 | Scymnus_sinuanodulus_830 | 0.182 |
| Scymnus_rubromaculatus_826 | Scymnus_sinuanodulus_830 | 0.182 |
| Scymnus_rubromaculatus_827 | Scymnus_sinuanodulus_830 | 0.182 |
| Chilocorus_nigritus_29 | Scymnus_sinuanodulus_831 | 0.182 |
| Scymnus_haemorrhoidalis_747 | Subcoccinella_vigintiquatuorpunctata_619 | 0.182 |
| Scymnus_haemorrhoidalis_748 | Subcoccinella_vigintiquatuorpunctata_619 | 0.182 |
| Calvia_quatuordecimguttata_206 | Scymnus_sinuanodulus_831 | 0.182 |
| Calvia_quatuordecimguttata_216 | Scymnus_sinuanodulus_831 | 0.182 |
| Chilocorus_nigritus_28 | Scymnus_sinuanodulus_831 | 0.183 |
| Myzia_pullata_518 | Vibidia_duodecimguttata_596 | 0.183 |
| Hyperaspis_reppensis_665 | Scymnus_sinuanodulus_828 | 0.183 |
| Scymnus_haemorrhoidalis_751 | Subcoccinella_vigintiquatuorpunctata_619 | 0.183 |
| Scymnus_haemorrhoidalis_754 | Subcoccinella_vigintiquatuorpunctata_619 | 0.183 |
| Scymnus_haemorrhoidalis_757 | Subcoccinella_vigintiquatuorpunctata_619 | 0.183 |
| Scymnus_haemorrhoidalis_758 | Subcoccinella_vigintiquatuorpunctata_619 | 0.183 |
| Hyperaspis_proba_660 | Subcoccinella_vigintiquatuorpunctata_623 | 0.183 |
| Coccidula_scutellata_83 | Scymnus_sinuanodulus_829 | 0.183 |
| Chilocorus_bipustulatus_18 | Scymnus_sinuanodulus_829 | 0.183 |
| Chilocorus_bipustulatus_19 | Scymnus_sinuanodulus_829 | 0.183 |
| Chilocorus_bipustulatus_20 | Scymnus_sinuanodulus_829 | 0.183 |
| Chilocorus_bipustulatus_22 | Scymnus_sinuanodulus_829 | 0.183 |
| Chilocorus_bipustulatus_24 | Scymnus_sinuanodulus_829 | 0.183 |
| Hyperaspis_campestris_651 | Subcoccinella_vigintiquatuorpunctata_619 | 0.183 |
| Hyperaspis_campestris_652 | Subcoccinella_vigintiquatuorpunctata_619 | 0.183 |
| Hyperaspis_campestris_653 | Subcoccinella_vigintiquatuorpunctata_619 | 0.183 |
| Scymnus_haemorrhoidalis_752 | Subcoccinella_vigintiquatuorpunctata_619 | 0.183 |
| Scymnus_haemorrhoidalis_753 | Subcoccinella_vigintiquatuorpunctata_619 | 0.183 |
| Hyperaspis_proba_660 | Subcoccinella_vigintiquatuorpunctata_622 | 0.183 |
| Hyperaspis_proba_660 | Subcoccinella_vigintiquatuorpunctata_625 | 0.183 |
| Hyperaspis_proba_660 | Subcoccinella_vigintiquatuorpunctata_626 | 0.183 |
| Hyperaspis_binotata_650 | Scymnus_sinuanodulus_831 | 0.183 |
| Scymnus_haemorrhoidalis_752 | Scymnus_sinuanodulus_830 | 0.183 |
| Scymnus_frontalis_746 | Scymnus_sinuanodulus_830 | 0.183 |
| Chilocorus_nigritus_27 | Scymnus_sinuanodulus_829 | 0.183 |
| Chilocorus_stigma_43 | Scymnus_sinuanodulus_829 | 0.183 |
| Calvia_decemguttata_193 | Tytthaspis_sedecimpunctata_591 | 0.183 |
| Calvia_decemguttata_194 | Tytthaspis_sedecimpunctata_591 | 0.183 |
| Psyllobora_vigintiduopunctata_556 | Vibidia_duodecimguttata_599 | 0.183 |
| Psyllobora_vigintiduopunctata_556 | Vibidia_duodecimguttata_601 | 0.183 |
| Psyllobora_vigintiduopunctata_556 | Vibidia_duodecimguttata_602 | 0.183 |
| Scymnus_haemorrhoidalis_752 | Scymnus_sinuanodulus_828 | 0.183 |
| Myzia_oblongoguttata_506 | Vibidia_duodecimguttata_597 | 0.183 |
| Psyllobora_vigintiduopunctata_561 | Vibidia_duodecimguttata_599 | 0.183 |
| Psyllobora_vigintiduopunctata_562 | Vibidia_duodecimguttata_599 | 0.183 |
| Psyllobora_vigintiduopunctata_565 | Vibidia_duodecimguttata_599 | 0.183 |
| Psyllobora_vigintiduopunctata_567 | Vibidia_duodecimguttata_599 | 0.183 |
| Myzia_oblongoguttata_506 | Vibidia_duodecimguttata_600 | 0.183 |
| Psyllobora_vigintiduopunctata_561 | Vibidia_duodecimguttata_601 | 0.183 |
| Psyllobora_vigintiduopunctata_562 | Vibidia_duodecimguttata_601 | 0.183 |
| Psyllobora_vigintiduopunctata_565 | Vibidia_duodecimguttata_601 | 0.183 |
| Psyllobora_vigintiduopunctata_567 | Vibidia_duodecimguttata_601 | 0.183 |
| Psyllobora_vigintiduopunctata_561 | Vibidia_duodecimguttata_602 | 0.183 |
| Psyllobora_vigintiduopunctata_562 | Vibidia_duodecimguttata_602 | 0.183 |
| Psyllobora_vigintiduopunctata_565 | Vibidia_duodecimguttata_602 | 0.183 |
| Psyllobora_vigintiduopunctata_567 | Vibidia_duodecimguttata_602 | 0.183 |

| | | |
|---|---|---|
| Scymnus_auritus_703 | Scymnus_sinuanodulus_828 | 0.183 |
| Coccinella_undecimpunctata_287 | Tytthaspis_sedecimpunctata_593 | 0.183 |
| Calvia_quatuordecimguttata_202 | Scymnus_sinuanodulus_829 | 0.183 |
| Calvia_quatuordecimguttata_210 | Scymnus_sinuanodulus_829 | 0.183 |
| Calvia_quatuordecimguttata_206 | Scymnus_sinuanodulus_829 | 0.183 |
| Calvia_quatuordecimguttata_216 | Scymnus_sinuanodulus_829 | 0.183 |
| Scymnus_apetzi_693 | Subcoccinella_vigintiquatuorpunctata_630 | 0.183 |
| Chilocorus_nigritus_29 | Scymnus_sinuanodulus_829 | 0.183 |
| Coccinella_quinquepunctata_237 | Vibidia_duodecimguttata_596 | 0.183 |
| Myzia_oblongoguttata_515 | Vibidia_duodecimguttata_596 | 0.183 |
| Myzia_oblongoguttata_503 | Vibidia_duodecimguttata_598 | 0.183 |
| Myzia_oblongoguttata_504 | Vibidia_duodecimguttata_598 | 0.183 |
| Myzia_oblongoguttata_505 | Vibidia_duodecimguttata_598 | 0.183 |
| Myzia_oblongoguttata_507 | Vibidia_duodecimguttata_598 | 0.183 |
| Myzia_oblongoguttata_508 | Vibidia_duodecimguttata_598 | 0.183 |
| Myzia_oblongoguttata_509 | Vibidia_duodecimguttata_598 | 0.183 |
| Myzia_oblongoguttata_510 | Vibidia_duodecimguttata_598 | 0.183 |
| Myzia_oblongoguttata_511 | Vibidia_duodecimguttata_598 | 0.183 |
| Myzia_oblongoguttata_512 | Vibidia_duodecimguttata_598 | 0.183 |
| Myzia_oblongoguttata_513 | Vibidia_duodecimguttata_598 | 0.183 |
| Myzia_oblongoguttata_514 | Vibidia_duodecimguttata_598 | 0.183 |
| Myzia_oblongoguttata_516 | Vibidia_duodecimguttata_598 | 0.183 |
| Anatis_ocellata_133 | Vibidia_duodecimguttata_603 | 0.183 |
| Anatis_ocellata_137 | Vibidia_duodecimguttata_603 | 0.183 |
| Anatis_ocellata_143 | Vibidia_duodecimguttata_603 | 0.183 |
| Chilocorus_nigritus_28 | Scymnus_sinuanodulus_829 | 0.183 |
| Scymnus_haemorrhoidalis_750 | Scymnus_sinuanodulus_831 | 0.183 |
| Anatis_ocellata_132 | Vibidia_duodecimguttata_603 | 0.183 |
| Anatis_ocellata_134 | Vibidia_duodecimguttata_603 | 0.183 |
| Anatis_ocellata_135 | Vibidia_duodecimguttata_603 | 0.183 |
| Anatis_ocellata_138 | Vibidia_duodecimguttata_603 | 0.183 |
| Anatis_ocellata_139 | Vibidia_duodecimguttata_603 | 0.183 |
| Anatis_ocellata_140 | Vibidia_duodecimguttata_603 | 0.183 |
| Anatis_ocellata_141 | Vibidia_duodecimguttata_603 | 0.183 |
| Anatis_ocellata_142 | Vibidia_duodecimguttata_603 | 0.183 |
| Anatis_ocellata_144 | Vibidia_duodecimguttata_603 | 0.183 |
| Anatis_ocellata_145 | Vibidia_duodecimguttata_603 | 0.183 |
| Anatis_ocellata_146 | Vibidia_duodecimguttata_603 | 0.183 |
| Psyllobora_vigintiduopunctata_566 | Vibidia_duodecimguttata_603 | 0.183 |
| Hyperaspis_binotata_650 | Scymnus_sinuanodulus_829 | 0.183 |
| Coccinella_undecimpunctata_287 | Scymnus_sinuanodulus_831 | 0.183 |
| Hyperaspis_oregona_658 | Tytthaspis_sedecimpunctata_585 | 0.183 |
| Platynaspis_luteorubra_55 | Tytthaspis_sedecimpunctata_577 | 0.183 |
| Platynaspis_luteorubra_55 | Tytthaspis_sedecimpunctata_578 | 0.183 |
| Platynaspis_luteorubra_55 | Tytthaspis_sedecimpunctata_579 | 0.183 |
| Platynaspis_luteorubra_55 | Tytthaspis_sedecimpunctata_581 | 0.183 |
| Platynaspis_luteorubra_55 | Tytthaspis_sedecimpunctata_582 | 0.183 |
| Platynaspis_luteorubra_55 | Tytthaspis_sedecimpunctata_589 | 0.183 |
| Platynaspis_luteorubra_55 | Tytthaspis_sedecimpunctata_590 | 0.183 |
| Platynaspis_luteorubra_55 | Tytthaspis_sedecimpunctata_592 | 0.183 |
| Platynaspis_luteorubra_61 | Tytthaspis_sedecimpunctata_593 | 0.183 |
| Platynaspis_luteorubra_55 | Tytthaspis_sedecimpunctata_594 | 0.183 |
| Platynaspis_luteorubra_55 | Tytthaspis_sedecimpunctata_595 | 0.183 |
| Platynaspis_luteorubra_55 | Tytthaspis_sedecimpunctata_587 | 0.183 |
| Platynaspis_luteorubra_61 | Tytthaspis_sedecimpunctata_588 | 0.183 |
| Chilocorus_renipustulatus_31 | Tytthaspis_sedecimpunctata_577 | 0.183 |

| | | |
|---|---|---|
| Chilocorus_renipustulatus_32 | Tytthaspis_sedecimpunctata_577 | 0.183 |
| Chilocorus_renipustulatus_35 | Tytthaspis_sedecimpunctata_577 | 0.183 |
| Chilocorus_renipustulatus_36 | Tytthaspis_sedecimpunctata_577 | 0.183 |
| Chilocorus_renipustulatus_37 | Tytthaspis_sedecimpunctata_577 | 0.183 |
| Chilocorus_renipustulatus_38 | Tytthaspis_sedecimpunctata_577 | 0.183 |
| Chilocorus_renipustulatus_40 | Tytthaspis_sedecimpunctata_577 | 0.183 |
| Chilocorus_renipustulatus_41 | Tytthaspis_sedecimpunctata_577 | 0.183 |
| Chilocorus_renipustulatus_42 | Tytthaspis_sedecimpunctata_577 | 0.183 |
| Chilocorus_renipustulatus_31 | Tytthaspis_sedecimpunctata_578 | 0.183 |
| Chilocorus_renipustulatus_32 | Tytthaspis_sedecimpunctata_578 | 0.183 |
| Chilocorus_renipustulatus_35 | Tytthaspis_sedecimpunctata_578 | 0.183 |
| Chilocorus_renipustulatus_36 | Tytthaspis_sedecimpunctata_578 | 0.183 |
| Chilocorus_renipustulatus_37 | Tytthaspis_sedecimpunctata_578 | 0.183 |
| Chilocorus_renipustulatus_38 | Tytthaspis_sedecimpunctata_578 | 0.183 |
| Chilocorus_renipustulatus_40 | Tytthaspis_sedecimpunctata_578 | 0.183 |
| Chilocorus_renipustulatus_41 | Tytthaspis_sedecimpunctata_578 | 0.183 |
| Chilocorus_renipustulatus_42 | Tytthaspis_sedecimpunctata_578 | 0.183 |
| Chilocorus_renipustulatus_31 | Tytthaspis_sedecimpunctata_579 | 0.183 |
| Chilocorus_renipustulatus_32 | Tytthaspis_sedecimpunctata_579 | 0.183 |
| Chilocorus_renipustulatus_35 | Tytthaspis_sedecimpunctata_579 | 0.183 |
| Chilocorus_renipustulatus_36 | Tytthaspis_sedecimpunctata_579 | 0.183 |
| Chilocorus_renipustulatus_37 | Tytthaspis_sedecimpunctata_579 | 0.183 |
| Chilocorus_renipustulatus_38 | Tytthaspis_sedecimpunctata_579 | 0.183 |
| Chilocorus_renipustulatus_40 | Tytthaspis_sedecimpunctata_579 | 0.183 |
| Chilocorus_renipustulatus_41 | Tytthaspis_sedecimpunctata_579 | 0.183 |
| Chilocorus_renipustulatus_42 | Tytthaspis_sedecimpunctata_579 | 0.183 |
| Chilocorus_renipustulatus_31 | Tytthaspis_sedecimpunctata_581 | 0.183 |
| Chilocorus_renipustulatus_32 | Tytthaspis_sedecimpunctata_581 | 0.183 |
| Chilocorus_renipustulatus_35 | Tytthaspis_sedecimpunctata_581 | 0.183 |
| Chilocorus_renipustulatus_36 | Tytthaspis_sedecimpunctata_581 | 0.183 |
| Chilocorus_renipustulatus_37 | Tytthaspis_sedecimpunctata_581 | 0.183 |
| Chilocorus_renipustulatus_38 | Tytthaspis_sedecimpunctata_581 | 0.183 |
| Chilocorus_renipustulatus_40 | Tytthaspis_sedecimpunctata_581 | 0.183 |
| Chilocorus_renipustulatus_41 | Tytthaspis_sedecimpunctata_581 | 0.183 |
| Chilocorus_renipustulatus_42 | Tytthaspis_sedecimpunctata_581 | 0.183 |
| Chilocorus_renipustulatus_31 | Tytthaspis_sedecimpunctata_582 | 0.183 |
| Chilocorus_renipustulatus_32 | Tytthaspis_sedecimpunctata_582 | 0.183 |
| Chilocorus_renipustulatus_35 | Tytthaspis_sedecimpunctata_582 | 0.183 |
| Chilocorus_renipustulatus_36 | Tytthaspis_sedecimpunctata_582 | 0.183 |
| Chilocorus_renipustulatus_37 | Tytthaspis_sedecimpunctata_582 | 0.183 |
| Chilocorus_renipustulatus_38 | Tytthaspis_sedecimpunctata_582 | 0.183 |
| Chilocorus_renipustulatus_40 | Tytthaspis_sedecimpunctata_582 | 0.183 |
| Chilocorus_renipustulatus_41 | Tytthaspis_sedecimpunctata_582 | 0.183 |
| Chilocorus_renipustulatus_42 | Tytthaspis_sedecimpunctata_582 | 0.183 |
| Chilocorus_renipustulatus_39 | Tytthaspis_sedecimpunctata_583 | 0.183 |
| Hyperaspis_reppensis_667 | Tytthaspis_sedecimpunctata_583 | 0.183 |
| Chilocorus_renipustulatus_31 | Tytthaspis_sedecimpunctata_585 | 0.183 |
| Chilocorus_renipustulatus_32 | Tytthaspis_sedecimpunctata_585 | 0.183 |
| Chilocorus_renipustulatus_35 | Tytthaspis_sedecimpunctata_585 | 0.183 |
| Chilocorus_renipustulatus_36 | Tytthaspis_sedecimpunctata_585 | 0.183 |
| Chilocorus_renipustulatus_37 | Tytthaspis_sedecimpunctata_585 | 0.183 |
| Chilocorus_renipustulatus_38 | Tytthaspis_sedecimpunctata_585 | 0.183 |
| Chilocorus_renipustulatus_40 | Tytthaspis_sedecimpunctata_585 | 0.183 |
| Chilocorus_renipustulatus_41 | Tytthaspis_sedecimpunctata_585 | 0.183 |
| Chilocorus_renipustulatus_42 | Tytthaspis_sedecimpunctata_585 | 0.183 |
| Hyperaspis_reppensis_666 | Tytthaspis_sedecimpunctata_585 | 0.183 |

| | | |
|---|---|---|
| Hyperaspis_reppensis_664 | Tytthaspis_sedecimpunctata_589 | 0.183 |
| Chilocorus_renipustulatus_31 | Tytthaspis_sedecimpunctata_590 | 0.183 |
| Chilocorus_renipustulatus_32 | Tytthaspis_sedecimpunctata_590 | 0.183 |
| Chilocorus_renipustulatus_35 | Tytthaspis_sedecimpunctata_590 | 0.183 |
| Chilocorus_renipustulatus_36 | Tytthaspis_sedecimpunctata_590 | 0.183 |
| Chilocorus_renipustulatus_37 | Tytthaspis_sedecimpunctata_590 | 0.183 |
| Chilocorus_renipustulatus_38 | Tytthaspis_sedecimpunctata_590 | 0.183 |
| Chilocorus_renipustulatus_40 | Tytthaspis_sedecimpunctata_590 | 0.183 |
| Chilocorus_renipustulatus_41 | Tytthaspis_sedecimpunctata_590 | 0.183 |
| Chilocorus_renipustulatus_42 | Tytthaspis_sedecimpunctata_590 | 0.183 |
| Chilocorus_renipustulatus_31 | Tytthaspis_sedecimpunctata_592 | 0.183 |
| Chilocorus_renipustulatus_32 | Tytthaspis_sedecimpunctata_592 | 0.183 |
| Chilocorus_renipustulatus_35 | Tytthaspis_sedecimpunctata_592 | 0.183 |
| Chilocorus_renipustulatus_36 | Tytthaspis_sedecimpunctata_592 | 0.183 |
| Chilocorus_renipustulatus_37 | Tytthaspis_sedecimpunctata_592 | 0.183 |
| Chilocorus_renipustulatus_38 | Tytthaspis_sedecimpunctata_592 | 0.183 |
| Chilocorus_renipustulatus_40 | Tytthaspis_sedecimpunctata_592 | 0.183 |
| Chilocorus_renipustulatus_41 | Tytthaspis_sedecimpunctata_592 | 0.183 |
| Chilocorus_renipustulatus_42 | Tytthaspis_sedecimpunctata_592 | 0.183 |
| Hyperaspis_reppensis_664 | Tytthaspis_sedecimpunctata_592 | 0.183 |
| Chilocorus_renipustulatus_31 | Tytthaspis_sedecimpunctata_593 | 0.183 |
| Chilocorus_renipustulatus_32 | Tytthaspis_sedecimpunctata_593 | 0.183 |
| Chilocorus_renipustulatus_35 | Tytthaspis_sedecimpunctata_593 | 0.183 |
| Chilocorus_renipustulatus_36 | Tytthaspis_sedecimpunctata_593 | 0.183 |
| Chilocorus_renipustulatus_37 | Tytthaspis_sedecimpunctata_593 | 0.183 |
| Chilocorus_renipustulatus_38 | Tytthaspis_sedecimpunctata_593 | 0.183 |
| Chilocorus_renipustulatus_40 | Tytthaspis_sedecimpunctata_593 | 0.183 |
| Chilocorus_renipustulatus_41 | Tytthaspis_sedecimpunctata_593 | 0.183 |
| Chilocorus_renipustulatus_42 | Tytthaspis_sedecimpunctata_593 | 0.183 |
| Chilocorus_renipustulatus_31 | Tytthaspis_sedecimpunctata_594 | 0.183 |
| Chilocorus_renipustulatus_32 | Tytthaspis_sedecimpunctata_594 | 0.183 |
| Chilocorus_renipustulatus_35 | Tytthaspis_sedecimpunctata_594 | 0.183 |
| Chilocorus_renipustulatus_36 | Tytthaspis_sedecimpunctata_594 | 0.183 |
| Chilocorus_renipustulatus_37 | Tytthaspis_sedecimpunctata_594 | 0.183 |
| Chilocorus_renipustulatus_38 | Tytthaspis_sedecimpunctata_594 | 0.183 |
| Chilocorus_renipustulatus_40 | Tytthaspis_sedecimpunctata_594 | 0.183 |
| Chilocorus_renipustulatus_41 | Tytthaspis_sedecimpunctata_594 | 0.183 |
| Chilocorus_renipustulatus_42 | Tytthaspis_sedecimpunctata_594 | 0.183 |
| Chilocorus_renipustulatus_31 | Tytthaspis_sedecimpunctata_595 | 0.183 |
| Chilocorus_renipustulatus_32 | Tytthaspis_sedecimpunctata_595 | 0.183 |
| Chilocorus_renipustulatus_35 | Tytthaspis_sedecimpunctata_595 | 0.183 |
| Chilocorus_renipustulatus_36 | Tytthaspis_sedecimpunctata_595 | 0.183 |
| Chilocorus_renipustulatus_37 | Tytthaspis_sedecimpunctata_595 | 0.183 |
| Chilocorus_renipustulatus_38 | Tytthaspis_sedecimpunctata_595 | 0.183 |
| Chilocorus_renipustulatus_40 | Tytthaspis_sedecimpunctata_595 | 0.183 |
| Chilocorus_renipustulatus_41 | Tytthaspis_sedecimpunctata_595 | 0.183 |
| Chilocorus_renipustulatus_42 | Tytthaspis_sedecimpunctata_595 | 0.183 |
| Scymnus_camptodromus_715 | Tytthaspis_sedecimpunctata_591 | 0.183 |
| Chilocorus_renipustulatus_39 | Tytthaspis_sedecimpunctata_580 | 0.183 |
| Hyperaspis_reppensis_664 | Tytthaspis_sedecimpunctata_580 | 0.183 |
| Scymnus_loewii_771 | Tytthaspis_sedecimpunctata_580 | 0.183 |
| Hyperaspis_reppensis_665 | Tytthaspis_sedecimpunctata_586 | 0.183 |
| Hyperaspis_reppensis_666 | Tytthaspis_sedecimpunctata_587 | 0.183 |
| Hyperaspis_reppensis_665 | Tytthaspis_sedecimpunctata_588 | 0.183 |
| Scymnus_ferrugatus_723 | Scymnus_sinuanodulus_828 | 0.183 |
| Scymnus_ferrugatus_725 | Scymnus_sinuanodulus_828 | 0.183 |

| | | |
|---|---|---|
| Scymnus_ferrugatus_726 | Scymnus_sinuanodulus_828 | 0.183 |
| Scymnus_ferrugatus_727 | Scymnus_sinuanodulus_828 | 0.183 |
| Scymnus_ferrugatus_728 | Scymnus_sinuanodulus_828 | 0.183 |
| Scymnus_ferrugatus_730 | Scymnus_sinuanodulus_828 | 0.183 |
| Scymnus_ferrugatus_731 | Scymnus_sinuanodulus_828 | 0.183 |
| Scymnus_ferrugatus_732 | Scymnus_sinuanodulus_828 | 0.183 |
| Scymnus_ferrugatus_733 | Scymnus_sinuanodulus_828 | 0.183 |
| Propylaea_quatuordecimpunctata_534 | Scymnus_suturalis_841 | 0.183 |
| Propylaea_quatuordecimpunctata_538 | Scymnus_suturalis_841 | 0.183 |
| Propylaea_quatuordecimpunctata_540 | Scymnus_suturalis_841 | 0.183 |
| Propylaea_quatuordecimpunctata_543 | Scymnus_suturalis_841 | 0.183 |
| Propylaea_quatuordecimpunctata_544 | Scymnus_suturalis_841 | 0.183 |
| Propylaea_quatuordecimpunctata_548 | Scymnus_suturalis_841 | 0.183 |
| Propylaea_quatuordecimpunctata_549 | Scymnus_suturalis_841 | 0.183 |
| Propylaea_quatuordecimpunctata_534 | Scymnus_suturalis_842 | 0.183 |
| Propylaea_quatuordecimpunctata_538 | Scymnus_suturalis_842 | 0.183 |
| Propylaea_quatuordecimpunctata_540 | Scymnus_suturalis_842 | 0.183 |
| Propylaea_quatuordecimpunctata_543 | Scymnus_suturalis_842 | 0.183 |
| Propylaea_quatuordecimpunctata_544 | Scymnus_suturalis_842 | 0.183 |
| Propylaea_quatuordecimpunctata_548 | Scymnus_suturalis_842 | 0.183 |
| Propylaea_quatuordecimpunctata_549 | Scymnus_suturalis_842 | 0.183 |
| Propylaea_quatuordecimpunctata_539 | Scymnus_suturalis_843 | 0.183 |
| Propylaea_quatuordecimpunctata_545 | Scymnus_suturalis_843 | 0.183 |
| Propylaea_quatuordecimpunctata_547 | Scymnus_suturalis_843 | 0.183 |
| Propylaea_quatuordecimpunctata_534 | Scymnus_suturalis_844 | 0.183 |
| Propylaea_quatuordecimpunctata_538 | Scymnus_suturalis_844 | 0.183 |
| Propylaea_quatuordecimpunctata_540 | Scymnus_suturalis_844 | 0.183 |
| Propylaea_quatuordecimpunctata_543 | Scymnus_suturalis_844 | 0.183 |
| Propylaea_quatuordecimpunctata_544 | Scymnus_suturalis_844 | 0.183 |
| Propylaea_quatuordecimpunctata_548 | Scymnus_suturalis_844 | 0.183 |
| Propylaea_quatuordecimpunctata_549 | Scymnus_suturalis_844 | 0.183 |
| Propylaea_quatuordecimpunctata_539 | Scymnus_suturalis_845 | 0.183 |
| Propylaea_quatuordecimpunctata_545 | Scymnus_suturalis_845 | 0.183 |
| Propylaea_quatuordecimpunctata_547 | Scymnus_suturalis_845 | 0.183 |
| Propylaea_quatuordecimpunctata_534 | Scymnus_suturalis_846 | 0.183 |
| Propylaea_quatuordecimpunctata_538 | Scymnus_suturalis_846 | 0.183 |
| Propylaea_quatuordecimpunctata_540 | Scymnus_suturalis_846 | 0.183 |
| Propylaea_quatuordecimpunctata_543 | Scymnus_suturalis_846 | 0.183 |
| Propylaea_quatuordecimpunctata_544 | Scymnus_suturalis_846 | 0.183 |
| Propylaea_quatuordecimpunctata_548 | Scymnus_suturalis_846 | 0.183 |
| Propylaea_quatuordecimpunctata_549 | Scymnus_suturalis_846 | 0.183 |
| Propylaea_quatuordecimpunctata_534 | Scymnus_suturalis_847 | 0.183 |
| Propylaea_quatuordecimpunctata_538 | Scymnus_suturalis_847 | 0.183 |
| Propylaea_quatuordecimpunctata_540 | Scymnus_suturalis_847 | 0.183 |
| Propylaea_quatuordecimpunctata_543 | Scymnus_suturalis_847 | 0.183 |
| Propylaea_quatuordecimpunctata_544 | Scymnus_suturalis_847 | 0.183 |
| Propylaea_quatuordecimpunctata_548 | Scymnus_suturalis_847 | 0.183 |
| Propylaea_quatuordecimpunctata_549 | Scymnus_suturalis_847 | 0.183 |
| Propylaea_quatuordecimpunctata_534 | Scymnus_suturalis_848 | 0.183 |
| Propylaea_quatuordecimpunctata_538 | Scymnus_suturalis_848 | 0.183 |
| Propylaea_quatuordecimpunctata_540 | Scymnus_suturalis_848 | 0.183 |
| Propylaea_quatuordecimpunctata_543 | Scymnus_suturalis_848 | 0.183 |
| Propylaea_quatuordecimpunctata_544 | Scymnus_suturalis_848 | 0.183 |
| Propylaea_quatuordecimpunctata_548 | Scymnus_suturalis_848 | 0.183 |
| Propylaea_quatuordecimpunctata_549 | Scymnus_suturalis_848 | 0.183 |
| Myzia_oblongoguttata_506 | Tytthaspis_sedecimpunctata_577 | 0.183 |

| | | |
|---|---|---|
| Scymnus_loewii_770 | Tytthaspis_sedecimpunctata_577 | 0.183 |
| Scymnus_loewii_773 | Tytthaspis_sedecimpunctata_577 | 0.183 |
| Scymnus_nebulosus_782 | Tytthaspis_sedecimpunctata_577 | 0.183 |
| Scymnus_ningshanensis_788 | Tytthaspis_sedecimpunctata_577 | 0.183 |
| Scymnus_ningshanensis_791 | Tytthaspis_sedecimpunctata_577 | 0.183 |
| Scymnus_ningshanensis_792 | Tytthaspis_sedecimpunctata_577 | 0.183 |
| Scymnus_ningshanensis_793 | Tytthaspis_sedecimpunctata_577 | 0.183 |
| Scymnus_ningshanensis_794 | Tytthaspis_sedecimpunctata_577 | 0.183 |
| Scymnus_ningshanensis_795 | Tytthaspis_sedecimpunctata_577 | 0.183 |
| Myzia_oblongoguttata_506 | Tytthaspis_sedecimpunctata_578 | 0.183 |
| Scymnus_loewii_770 | Tytthaspis_sedecimpunctata_578 | 0.183 |
| Scymnus_loewii_773 | Tytthaspis_sedecimpunctata_578 | 0.183 |
| Scymnus_nebulosus_782 | Tytthaspis_sedecimpunctata_578 | 0.183 |
| Scymnus_ningshanensis_788 | Tytthaspis_sedecimpunctata_578 | 0.183 |
| Scymnus_ningshanensis_791 | Tytthaspis_sedecimpunctata_578 | 0.183 |
| Scymnus_ningshanensis_792 | Tytthaspis_sedecimpunctata_578 | 0.183 |
| Scymnus_ningshanensis_793 | Tytthaspis_sedecimpunctata_578 | 0.183 |
| Scymnus_ningshanensis_794 | Tytthaspis_sedecimpunctata_578 | 0.183 |
| Scymnus_ningshanensis_795 | Tytthaspis_sedecimpunctata_578 | 0.183 |
| Myzia_oblongoguttata_506 | Tytthaspis_sedecimpunctata_579 | 0.183 |
| Scymnus_loewii_770 | Tytthaspis_sedecimpunctata_579 | 0.183 |
| Scymnus_loewii_773 | Tytthaspis_sedecimpunctata_579 | 0.183 |
| Scymnus_nebulosus_782 | Tytthaspis_sedecimpunctata_579 | 0.183 |
| Scymnus_ningshanensis_788 | Tytthaspis_sedecimpunctata_579 | 0.183 |
| Scymnus_ningshanensis_791 | Tytthaspis_sedecimpunctata_579 | 0.183 |
| Scymnus_ningshanensis_792 | Tytthaspis_sedecimpunctata_579 | 0.183 |
| Scymnus_ningshanensis_793 | Tytthaspis_sedecimpunctata_579 | 0.183 |
| Scymnus_ningshanensis_794 | Tytthaspis_sedecimpunctata_579 | 0.183 |
| Scymnus_ningshanensis_795 | Tytthaspis_sedecimpunctata_579 | 0.183 |
| Myzia_oblongoguttata_506 | Tytthaspis_sedecimpunctata_581 | 0.183 |
| Scymnus_loewii_770 | Tytthaspis_sedecimpunctata_581 | 0.183 |
| Scymnus_loewii_773 | Tytthaspis_sedecimpunctata_581 | 0.183 |
| Scymnus_nebulosus_782 | Tytthaspis_sedecimpunctata_581 | 0.183 |
| Scymnus_ningshanensis_788 | Tytthaspis_sedecimpunctata_581 | 0.183 |
| Scymnus_ningshanensis_791 | Tytthaspis_sedecimpunctata_581 | 0.183 |
| Scymnus_ningshanensis_792 | Tytthaspis_sedecimpunctata_581 | 0.183 |
| Scymnus_ningshanensis_793 | Tytthaspis_sedecimpunctata_581 | 0.183 |
| Scymnus_ningshanensis_794 | Tytthaspis_sedecimpunctata_581 | 0.183 |
| Scymnus_ningshanensis_795 | Tytthaspis_sedecimpunctata_581 | 0.183 |
| Myzia_oblongoguttata_506 | Tytthaspis_sedecimpunctata_582 | 0.183 |
| Scymnus_loewii_770 | Tytthaspis_sedecimpunctata_582 | 0.183 |
| Scymnus_loewii_773 | Tytthaspis_sedecimpunctata_582 | 0.183 |
| Scymnus_nebulosus_782 | Tytthaspis_sedecimpunctata_582 | 0.183 |
| Scymnus_ningshanensis_788 | Tytthaspis_sedecimpunctata_582 | 0.183 |
| Scymnus_ningshanensis_791 | Tytthaspis_sedecimpunctata_582 | 0.183 |
| Scymnus_ningshanensis_792 | Tytthaspis_sedecimpunctata_582 | 0.183 |
| Scymnus_ningshanensis_793 | Tytthaspis_sedecimpunctata_582 | 0.183 |
| Scymnus_ningshanensis_794 | Tytthaspis_sedecimpunctata_582 | 0.183 |
| Scymnus_ningshanensis_795 | Tytthaspis_sedecimpunctata_582 | 0.183 |
| Coccinella_transversalis_257 | Tytthaspis_sedecimpunctata_583 | 0.183 |
| Coccinella_transversalis_259 | Tytthaspis_sedecimpunctata_583 | 0.183 |
| Coccinella_transversalis_260 | Tytthaspis_sedecimpunctata_583 | 0.183 |
| Coccinella_transversalis_261 | Tytthaspis_sedecimpunctata_583 | 0.183 |
| Coccinella_transversalis_262 | Tytthaspis_sedecimpunctata_583 | 0.183 |
| Coccinella_transversalis_263 | Tytthaspis_sedecimpunctata_583 | 0.183 |
| Coccinella_transversalis_264 | Tytthaspis_sedecimpunctata_583 | 0.183 |

Supplementary Table S4 (continued)

| | | |
|---|---|---|
| Myzia_oblongoguttata_504 | Tytthaspis_sedecimpunctata_583 | 0.183 |
| Myzia_oblongoguttata_505 | Tytthaspis_sedecimpunctata_583 | 0.183 |
| Myzia_oblongoguttata_507 | Tytthaspis_sedecimpunctata_583 | 0.183 |
| Myzia_oblongoguttata_508 | Tytthaspis_sedecimpunctata_583 | 0.183 |
| Myzia_oblongoguttata_509 | Tytthaspis_sedecimpunctata_583 | 0.183 |
| Myzia_oblongoguttata_510 | Tytthaspis_sedecimpunctata_583 | 0.183 |
| Myzia_oblongoguttata_511 | Tytthaspis_sedecimpunctata_583 | 0.183 |
| Myzia_oblongoguttata_512 | Tytthaspis_sedecimpunctata_583 | 0.183 |
| Myzia_oblongoguttata_513 | Tytthaspis_sedecimpunctata_583 | 0.183 |
| Myzia_oblongoguttata_514 | Tytthaspis_sedecimpunctata_583 | 0.183 |
| Myzia_oblongoguttata_516 | Tytthaspis_sedecimpunctata_583 | 0.183 |
| Coccinella_transversalis_257 | Tytthaspis_sedecimpunctata_585 | 0.183 |
| Coccinella_transversalis_259 | Tytthaspis_sedecimpunctata_585 | 0.183 |
| Coccinella_transversalis_260 | Tytthaspis_sedecimpunctata_585 | 0.183 |
| Coccinella_transversalis_261 | Tytthaspis_sedecimpunctata_585 | 0.183 |
| Coccinella_transversalis_262 | Tytthaspis_sedecimpunctata_585 | 0.183 |
| Coccinella_transversalis_263 | Tytthaspis_sedecimpunctata_585 | 0.183 |
| Coccinella_transversalis_264 | Tytthaspis_sedecimpunctata_585 | 0.183 |
| Myzia_oblongoguttata_506 | Tytthaspis_sedecimpunctata_585 | 0.183 |
| Scymnus_loewii_770 | Tytthaspis_sedecimpunctata_585 | 0.183 |
| Scymnus_loewii_773 | Tytthaspis_sedecimpunctata_585 | 0.183 |
| Scymnus_nebulosus_782 | Tytthaspis_sedecimpunctata_585 | 0.183 |
| Scymnus_ningshanensis_788 | Tytthaspis_sedecimpunctata_585 | 0.183 |
| Scymnus_ningshanensis_791 | Tytthaspis_sedecimpunctata_585 | 0.183 |
| Scymnus_ningshanensis_792 | Tytthaspis_sedecimpunctata_585 | 0.183 |
| Scymnus_ningshanensis_793 | Tytthaspis_sedecimpunctata_585 | 0.183 |
| Scymnus_ningshanensis_794 | Tytthaspis_sedecimpunctata_585 | 0.183 |
| Scymnus_ningshanensis_795 | Tytthaspis_sedecimpunctata_585 | 0.183 |
| Didion_punctatum_645 | Tytthaspis_sedecimpunctata_588 | 0.183 |
| Scymnus_loewii_774 | Tytthaspis_sedecimpunctata_588 | 0.183 |
| Myzia_oblongoguttata_504 | Tytthaspis_sedecimpunctata_589 | 0.183 |
| Myzia_oblongoguttata_505 | Tytthaspis_sedecimpunctata_589 | 0.183 |
| Myzia_oblongoguttata_507 | Tytthaspis_sedecimpunctata_589 | 0.183 |
| Myzia_oblongoguttata_508 | Tytthaspis_sedecimpunctata_589 | 0.183 |
| Myzia_oblongoguttata_509 | Tytthaspis_sedecimpunctata_589 | 0.183 |
| Myzia_oblongoguttata_510 | Tytthaspis_sedecimpunctata_589 | 0.183 |
| Myzia_oblongoguttata_511 | Tytthaspis_sedecimpunctata_589 | 0.183 |
| Myzia_oblongoguttata_512 | Tytthaspis_sedecimpunctata_589 | 0.183 |
| Myzia_oblongoguttata_513 | Tytthaspis_sedecimpunctata_589 | 0.183 |
| Myzia_oblongoguttata_514 | Tytthaspis_sedecimpunctata_589 | 0.183 |
| Myzia_oblongoguttata_516 | Tytthaspis_sedecimpunctata_589 | 0.183 |
| Scymnus_loewii_770 | Tytthaspis_sedecimpunctata_589 | 0.183 |
| Scymnus_loewii_772 | Tytthaspis_sedecimpunctata_589 | 0.183 |
| Myzia_oblongoguttata_506 | Tytthaspis_sedecimpunctata_590 | 0.183 |
| Scymnus_loewii_770 | Tytthaspis_sedecimpunctata_590 | 0.183 |
| Scymnus_loewii_773 | Tytthaspis_sedecimpunctata_590 | 0.183 |
| Scymnus_nebulosus_782 | Tytthaspis_sedecimpunctata_590 | 0.183 |
| Scymnus_ningshanensis_788 | Tytthaspis_sedecimpunctata_590 | 0.183 |
| Scymnus_ningshanensis_791 | Tytthaspis_sedecimpunctata_590 | 0.183 |
| Scymnus_ningshanensis_792 | Tytthaspis_sedecimpunctata_590 | 0.183 |
| Scymnus_ningshanensis_793 | Tytthaspis_sedecimpunctata_590 | 0.183 |
| Scymnus_ningshanensis_794 | Tytthaspis_sedecimpunctata_590 | 0.183 |
| Scymnus_ningshanensis_795 | Tytthaspis_sedecimpunctata_590 | 0.183 |
| Myzia_oblongoguttata_506 | Tytthaspis_sedecimpunctata_592 | 0.183 |
| Scymnus_loewii_770 | Tytthaspis_sedecimpunctata_592 | 0.183 |
| Scymnus_loewii_773 | Tytthaspis_sedecimpunctata_592 | 0.183 |

| | | |
|---|---|---|
| Scymnus_nebulosus_782 | Tytthaspis_sedecimpunctata_592 | 0.183 |
| Scymnus_ningshanensis_788 | Tytthaspis_sedecimpunctata_592 | 0.183 |
| Scymnus_ningshanensis_791 | Tytthaspis_sedecimpunctata_592 | 0.183 |
| Scymnus_ningshanensis_792 | Tytthaspis_sedecimpunctata_592 | 0.183 |
| Scymnus_ningshanensis_793 | Tytthaspis_sedecimpunctata_592 | 0.183 |
| Scymnus_ningshanensis_794 | Tytthaspis_sedecimpunctata_592 | 0.183 |
| Scymnus_ningshanensis_795 | Tytthaspis_sedecimpunctata_592 | 0.183 |
| Scymnus_loewii_770 | Tytthaspis_sedecimpunctata_593 | 0.183 |
| Scymnus_loewii_773 | Tytthaspis_sedecimpunctata_593 | 0.183 |
| Scymnus_nebulosus_782 | Tytthaspis_sedecimpunctata_593 | 0.183 |
| Scymnus_ningshanensis_789 | Tytthaspis_sedecimpunctata_593 | 0.183 |
| Scymnus_ningshanensis_790 | Tytthaspis_sedecimpunctata_593 | 0.183 |
| Myzia_oblongoguttata_506 | Tytthaspis_sedecimpunctata_594 | 0.183 |
| Scymnus_loewii_770 | Tytthaspis_sedecimpunctata_594 | 0.183 |
| Scymnus_loewii_773 | Tytthaspis_sedecimpunctata_594 | 0.183 |
| Scymnus_nebulosus_782 | Tytthaspis_sedecimpunctata_594 | 0.183 |
| Scymnus_ningshanensis_788 | Tytthaspis_sedecimpunctata_594 | 0.183 |
| Scymnus_ningshanensis_791 | Tytthaspis_sedecimpunctata_594 | 0.183 |
| Scymnus_ningshanensis_792 | Tytthaspis_sedecimpunctata_594 | 0.183 |
| Scymnus_ningshanensis_793 | Tytthaspis_sedecimpunctata_594 | 0.183 |
| Scymnus_ningshanensis_794 | Tytthaspis_sedecimpunctata_594 | 0.183 |
| Scymnus_ningshanensis_795 | Tytthaspis_sedecimpunctata_594 | 0.183 |
| Myzia_oblongoguttata_506 | Tytthaspis_sedecimpunctata_595 | 0.183 |
| Scymnus_loewii_770 | Tytthaspis_sedecimpunctata_595 | 0.183 |
| Scymnus_loewii_773 | Tytthaspis_sedecimpunctata_595 | 0.183 |
| Scymnus_nebulosus_782 | Tytthaspis_sedecimpunctata_595 | 0.183 |
| Scymnus_ningshanensis_788 | Tytthaspis_sedecimpunctata_595 | 0.183 |
| Scymnus_ningshanensis_791 | Tytthaspis_sedecimpunctata_595 | 0.183 |
| Scymnus_ningshanensis_792 | Tytthaspis_sedecimpunctata_595 | 0.183 |
| Scymnus_ningshanensis_793 | Tytthaspis_sedecimpunctata_595 | 0.183 |
| Scymnus_ningshanensis_794 | Tytthaspis_sedecimpunctata_595 | 0.183 |
| Scymnus_ningshanensis_795 | Tytthaspis_sedecimpunctata_595 | 0.183 |
| Propylaea_quatuordecimpunctata_534 | Scymnus_suturalis_832 | 0.183 |
| Propylaea_quatuordecimpunctata_538 | Scymnus_suturalis_832 | 0.183 |
| Propylaea_quatuordecimpunctata_540 | Scymnus_suturalis_832 | 0.183 |
| Propylaea_quatuordecimpunctata_543 | Scymnus_suturalis_832 | 0.183 |
| Propylaea_quatuordecimpunctata_544 | Scymnus_suturalis_832 | 0.183 |
| Propylaea_quatuordecimpunctata_548 | Scymnus_suturalis_832 | 0.183 |
| Propylaea_quatuordecimpunctata_549 | Scymnus_suturalis_832 | 0.183 |
| Coccidula_scutellata_83 | Scymnus_suturalis_833 | 0.183 |
| Propylaea_quatuordecimpunctata_534 | Scymnus_suturalis_835 | 0.183 |
| Propylaea_quatuordecimpunctata_538 | Scymnus_suturalis_835 | 0.183 |
| Propylaea_quatuordecimpunctata_540 | Scymnus_suturalis_835 | 0.183 |
| Propylaea_quatuordecimpunctata_543 | Scymnus_suturalis_835 | 0.183 |
| Propylaea_quatuordecimpunctata_544 | Scymnus_suturalis_835 | 0.183 |
| Propylaea_quatuordecimpunctata_548 | Scymnus_suturalis_835 | 0.183 |
| Propylaea_quatuordecimpunctata_549 | Scymnus_suturalis_835 | 0.183 |
| Propylaea_quatuordecimpunctata_534 | Scymnus_suturalis_836 | 0.183 |
| Propylaea_quatuordecimpunctata_538 | Scymnus_suturalis_836 | 0.183 |
| Propylaea_quatuordecimpunctata_540 | Scymnus_suturalis_836 | 0.183 |
| Propylaea_quatuordecimpunctata_543 | Scymnus_suturalis_836 | 0.183 |
| Propylaea_quatuordecimpunctata_544 | Scymnus_suturalis_836 | 0.183 |
| Propylaea_quatuordecimpunctata_548 | Scymnus_suturalis_836 | 0.183 |
| Propylaea_quatuordecimpunctata_549 | Scymnus_suturalis_836 | 0.183 |
| Chilocorus_stigma_43 | Scymnus_suturalis_838 | 0.183 |
| Coccidula_scutellata_78 | Scymnus_suturalis_838 | 0.183 |

Supplementary Table S4 (continued)

| | | |
|---|---|---|
| Coccidula_scutellata_79 | Scymnus_suturalis_838 | 0.183 |
| Propylaea_quatuordecimpunctata_534 | Scymnus_suturalis_839 | 0.183 |
| Propylaea_quatuordecimpunctata_538 | Scymnus_suturalis_839 | 0.183 |
| Propylaea_quatuordecimpunctata_540 | Scymnus_suturalis_839 | 0.183 |
| Propylaea_quatuordecimpunctata_543 | Scymnus_suturalis_839 | 0.183 |
| Propylaea_quatuordecimpunctata_544 | Scymnus_suturalis_839 | 0.183 |
| Propylaea_quatuordecimpunctata_548 | Scymnus_suturalis_839 | 0.183 |
| Propylaea_quatuordecimpunctata_549 | Scymnus_suturalis_839 | 0.183 |
| Chilocorus_stigma_43 | Scymnus_suturalis_840 | 0.183 |
| Coccidula_scutellata_78 | Scymnus_suturalis_840 | 0.183 |
| Coccidula_scutellata_79 | Scymnus_suturalis_840 | 0.183 |
| Anatis_ocellata_136 | Vibidia_duodecimguttata_603 | 0.183 |
| Chilocorus_stigma_47 | Scymnus_suturalis_843 | 0.183 |
| Coccidula_scutellata_83 | Scymnus_suturalis_843 | 0.183 |
| Chilocorus_stigma_47 | Scymnus_suturalis_845 | 0.183 |
| Coccidula_scutellata_83 | Scymnus_suturalis_845 | 0.183 |
| Coccinella_transversalis_257 | Tytthaspis_sedecimpunctata_580 | 0.183 |
| Coccinella_transversalis_259 | Tytthaspis_sedecimpunctata_580 | 0.183 |
| Coccinella_transversalis_260 | Tytthaspis_sedecimpunctata_580 | 0.183 |
| Coccinella_transversalis_261 | Tytthaspis_sedecimpunctata_580 | 0.183 |
| Coccinella_transversalis_262 | Tytthaspis_sedecimpunctata_580 | 0.183 |
| Coccinella_transversalis_263 | Tytthaspis_sedecimpunctata_580 | 0.183 |
| Coccinella_transversalis_264 | Tytthaspis_sedecimpunctata_580 | 0.183 |
| Myzia_oblongoguttata_503 | Tytthaspis_sedecimpunctata_580 | 0.183 |
| Myzia_oblongoguttata_515 | Tytthaspis_sedecimpunctata_580 | 0.183 |
| Didion_punctatum_645 | Tytthaspis_sedecimpunctata_583 | 0.183 |
| Scymnus_loewii_771 | Tytthaspis_sedecimpunctata_584 | 0.183 |
| Brumus_quadripustulatus_9 | Tytthaspis_sedecimpunctata_586 | 0.183 |
| Myzia_oblongoguttata_503 | Tytthaspis_sedecimpunctata_586 | 0.183 |
| Myzia_oblongoguttata_515 | Tytthaspis_sedecimpunctata_586 | 0.183 |
| Scymnus_nebulosus_782 | Tytthaspis_sedecimpunctata_586 | 0.183 |
| Brumus_quadripustulatus_12 | Tytthaspis_sedecimpunctata_587 | 0.183 |
| Myzia_oblongoguttata_504 | Tytthaspis_sedecimpunctata_587 | 0.183 |
| Myzia_oblongoguttata_505 | Tytthaspis_sedecimpunctata_587 | 0.183 |
| Myzia_oblongoguttata_507 | Tytthaspis_sedecimpunctata_587 | 0.183 |
| Myzia_oblongoguttata_508 | Tytthaspis_sedecimpunctata_587 | 0.183 |
| Myzia_oblongoguttata_509 | Tytthaspis_sedecimpunctata_587 | 0.183 |
| Myzia_oblongoguttata_510 | Tytthaspis_sedecimpunctata_587 | 0.183 |
| Myzia_oblongoguttata_511 | Tytthaspis_sedecimpunctata_587 | 0.183 |
| Myzia_oblongoguttata_512 | Tytthaspis_sedecimpunctata_587 | 0.183 |
| Myzia_oblongoguttata_513 | Tytthaspis_sedecimpunctata_587 | 0.183 |
| Myzia_oblongoguttata_514 | Tytthaspis_sedecimpunctata_587 | 0.183 |
| Myzia_oblongoguttata_516 | Tytthaspis_sedecimpunctata_587 | 0.183 |
| Scymnus_loewii_771 | Tytthaspis_sedecimpunctata_587 | 0.183 |
| Didion_punctatum_646 | Tytthaspis_sedecimpunctata_588 | 0.183 |
| Myzia_oblongoguttata_503 | Tytthaspis_sedecimpunctata_588 | 0.183 |
| Myzia_oblongoguttata_515 | Tytthaspis_sedecimpunctata_588 | 0.183 |
| Scymnus_loewii_772 | Tytthaspis_sedecimpunctata_588 | 0.183 |
| Scymnus_nebulosus_782 | Tytthaspis_sedecimpunctata_588 | 0.183 |
| Aphidecta_obliterata_185 | Tytthaspis_sedecimpunctata_589 | 0.183 |
| Aphidecta_obliterata_186 | Tytthaspis_sedecimpunctata_589 | 0.183 |
| Myzia_pullata_517 | Tytthaspis_sedecimpunctata_591 | 0.183 |
| Didion_punctatum_645 | Tytthaspis_sedecimpunctata_592 | 0.183 |
| Coccidula_scutellata_77 | Scymnus_sinuanodulus_830 | 0.183 |
| Chilocorus_stigma_43 | Scymnus_suturalis_834 | 0.183 |
| Coccidula_scutellata_78 | Scymnus_suturalis_834 | 0.183 |

| | | |
|---|---|---|
| Coccidula_scutellata_79 | Scymnus_suturalis_834 | 0.183 |
| Myzia_pullata_517 | Scymnus_suturalis_838 | 0.183 |
| Myzia_pullata_517 | Scymnus_suturalis_840 | 0.183 |
| Myzia_pullata_518 | Scymnus_suturalis_841 | 0.184 |
| Myzia_pullata_518 | Scymnus_suturalis_842 | 0.184 |
| Myrrha_octodecimguttata_488 | Scymnus_suturalis_843 | 0.184 |
| Myrrha_octodecimguttata_491 | Scymnus_suturalis_843 | 0.184 |
| Myrrha_octodecimguttata_492 | Scymnus_suturalis_843 | 0.184 |
| Myzia_pullata_518 | Scymnus_suturalis_844 | 0.184 |
| Myrrha_octodecimguttata_488 | Scymnus_suturalis_845 | 0.184 |
| Myrrha_octodecimguttata_491 | Scymnus_suturalis_845 | 0.184 |
| Myrrha_octodecimguttata_492 | Scymnus_suturalis_845 | 0.184 |
| Myzia_pullata_517 | Scymnus_suturalis_845 | 0.184 |
| Myzia_pullata_518 | Scymnus_suturalis_846 | 0.184 |
| Myzia_pullata_518 | Scymnus_suturalis_847 | 0.184 |
| Myzia_pullata_518 | Scymnus_suturalis_848 | 0.184 |
| Didion_punctatum_646 | Tytthaspis_sedecimpunctata_583 | 0.184 |
| Myzia_pullata_518 | Tytthaspis_sedecimpunctata_585 | 0.184 |
| Aphidecta_obliterata_185 | Tytthaspis_sedecimpunctata_586 | 0.184 |
| Aphidecta_obliterata_186 | Tytthaspis_sedecimpunctata_586 | 0.184 |
| Aphidecta_obliterata_183 | Tytthaspis_sedecimpunctata_588 | 0.184 |
| Didion_punctatum_644 | Tytthaspis_sedecimpunctata_588 | 0.184 |
| Aphidecta_obliterata_185 | Tytthaspis_sedecimpunctata_591 | 0.184 |
| Aphidecta_obliterata_186 | Tytthaspis_sedecimpunctata_591 | 0.184 |
| Didion_punctatum_646 | Tytthaspis_sedecimpunctata_592 | 0.184 |
| Cheilomenes_sexmaculata_224 | Tytthaspis_sedecimpunctata_593 | 0.184 |
| Myzia_pullata_518 | Scymnus_suturalis_832 | 0.184 |
| Chilocorus_stigma_47 | Scymnus_suturalis_833 | 0.184 |
| Myzia_pullata_517 | Scymnus_suturalis_834 | 0.184 |
| Myzia_pullata_518 | Scymnus_suturalis_835 | 0.184 |
| Myzia_pullata_518 | Scymnus_suturalis_836 | 0.184 |
| Myzia_pullata_518 | Scymnus_suturalis_837 | 0.184 |
| Myzia_pullata_518 | Scymnus_suturalis_839 | 0.184 |
| Illeis_galbula_474 | Vibidia_duodecimguttata_596 | 0.184 |
| Illeis_galbula_475 | Vibidia_duodecimguttata_596 | 0.184 |
| Illeis_galbula_476 | Vibidia_duodecimguttata_596 | 0.184 |
| Anatis_labiculata_123 | Scymnus_suturalis_843 | 0.184 |
| Anatis_labiculata_124 | Scymnus_suturalis_843 | 0.184 |
| Anatis_labiculata_125 | Scymnus_suturalis_843 | 0.184 |
| Anatis_labiculata_127 | Scymnus_suturalis_843 | 0.184 |
| Anatis_labiculata_123 | Scymnus_suturalis_845 | 0.184 |
| Anatis_labiculata_124 | Scymnus_suturalis_845 | 0.184 |
| Anatis_labiculata_125 | Scymnus_suturalis_845 | 0.184 |
| Anatis_labiculata_127 | Scymnus_suturalis_845 | 0.184 |
| Aphidecta_obliterata_183 | Tytthaspis_sedecimpunctata_577 | 0.184 |
| Coccinella_septempunctata_242 | Tytthaspis_sedecimpunctata_577 | 0.184 |
| Coccinella_septempunctata_244 | Tytthaspis_sedecimpunctata_577 | 0.184 |
| Coccinella_septempunctata_245 | Tytthaspis_sedecimpunctata_577 | 0.184 |
| Coccinella_septempunctata_247 | Tytthaspis_sedecimpunctata_577 | 0.184 |
| Coccinella_septempunctata_248 | Tytthaspis_sedecimpunctata_577 | 0.184 |
| Coccinella_septempunctata_249 | Tytthaspis_sedecimpunctata_577 | 0.184 |
| Coccinella_septempunctata_252 | Tytthaspis_sedecimpunctata_577 | 0.184 |
| Coccinella_septempunctata_255 | Tytthaspis_sedecimpunctata_577 | 0.184 |
| Coccinella_septempunctata_256 | Tytthaspis_sedecimpunctata_577 | 0.184 |
| Scymnus_camptodromus_714 | Tytthaspis_sedecimpunctata_577 | 0.184 |
| Aphidecta_obliterata_183 | Tytthaspis_sedecimpunctata_578 | 0.184 |

| | | |
|---|---|---|
| Coccinella_septempunctata_242 | Tytthaspis_sedecimpunctata_578 | 0.184 |
| Coccinella_septempunctata_244 | Tytthaspis_sedecimpunctata_578 | 0.184 |
| Coccinella_septempunctata_245 | Tytthaspis_sedecimpunctata_578 | 0.184 |
| Coccinella_septempunctata_247 | Tytthaspis_sedecimpunctata_578 | 0.184 |
| Coccinella_septempunctata_248 | Tytthaspis_sedecimpunctata_578 | 0.184 |
| Coccinella_septempunctata_249 | Tytthaspis_sedecimpunctata_578 | 0.184 |
| Coccinella_septempunctata_252 | Tytthaspis_sedecimpunctata_578 | 0.184 |
| Coccinella_septempunctata_255 | Tytthaspis_sedecimpunctata_578 | 0.184 |
| Coccinella_septempunctata_256 | Tytthaspis_sedecimpunctata_578 | 0.184 |
| Scymnus_camptodromus_714 | Tytthaspis_sedecimpunctata_578 | 0.184 |
| Aphidecta_obliterata_183 | Tytthaspis_sedecimpunctata_579 | 0.184 |
| Coccinella_septempunctata_242 | Tytthaspis_sedecimpunctata_579 | 0.184 |
| Coccinella_septempunctata_244 | Tytthaspis_sedecimpunctata_579 | 0.184 |
| Coccinella_septempunctata_245 | Tytthaspis_sedecimpunctata_579 | 0.184 |
| Coccinella_septempunctata_247 | Tytthaspis_sedecimpunctata_579 | 0.184 |
| Coccinella_septempunctata_248 | Tytthaspis_sedecimpunctata_579 | 0.184 |
| Coccinella_septempunctata_249 | Tytthaspis_sedecimpunctata_579 | 0.184 |
| Coccinella_septempunctata_252 | Tytthaspis_sedecimpunctata_579 | 0.184 |
| Coccinella_septempunctata_255 | Tytthaspis_sedecimpunctata_579 | 0.184 |
| Coccinella_septempunctata_256 | Tytthaspis_sedecimpunctata_579 | 0.184 |
| Scymnus_camptodromus_714 | Tytthaspis_sedecimpunctata_579 | 0.184 |
| Aphidecta_obliterata_183 | Tytthaspis_sedecimpunctata_581 | 0.184 |
| Coccinella_septempunctata_242 | Tytthaspis_sedecimpunctata_581 | 0.184 |
| Coccinella_septempunctata_244 | Tytthaspis_sedecimpunctata_581 | 0.184 |
| Coccinella_septempunctata_245 | Tytthaspis_sedecimpunctata_581 | 0.184 |
| Coccinella_septempunctata_247 | Tytthaspis_sedecimpunctata_581 | 0.184 |
| Coccinella_septempunctata_248 | Tytthaspis_sedecimpunctata_581 | 0.184 |
| Coccinella_septempunctata_249 | Tytthaspis_sedecimpunctata_581 | 0.184 |
| Coccinella_septempunctata_252 | Tytthaspis_sedecimpunctata_581 | 0.184 |
| Coccinella_septempunctata_255 | Tytthaspis_sedecimpunctata_581 | 0.184 |
| Coccinella_septempunctata_256 | Tytthaspis_sedecimpunctata_581 | 0.184 |
| Scymnus_camptodromus_714 | Tytthaspis_sedecimpunctata_581 | 0.184 |
| Aphidecta_obliterata_183 | Tytthaspis_sedecimpunctata_582 | 0.184 |
| Coccinella_septempunctata_242 | Tytthaspis_sedecimpunctata_582 | 0.184 |
| Coccinella_septempunctata_244 | Tytthaspis_sedecimpunctata_582 | 0.184 |
| Coccinella_septempunctata_245 | Tytthaspis_sedecimpunctata_582 | 0.184 |
| Coccinella_septempunctata_247 | Tytthaspis_sedecimpunctata_582 | 0.184 |
| Coccinella_septempunctata_248 | Tytthaspis_sedecimpunctata_582 | 0.184 |
| Coccinella_septempunctata_249 | Tytthaspis_sedecimpunctata_582 | 0.184 |
| Coccinella_septempunctata_252 | Tytthaspis_sedecimpunctata_582 | 0.184 |
| Coccinella_septempunctata_255 | Tytthaspis_sedecimpunctata_582 | 0.184 |
| Coccinella_septempunctata_256 | Tytthaspis_sedecimpunctata_582 | 0.184 |
| Scymnus_camptodromus_714 | Tytthaspis_sedecimpunctata_582 | 0.184 |
| Aphidecta_obliterata_185 | Tytthaspis_sedecimpunctata_583 | 0.184 |
| Aphidecta_obliterata_186 | Tytthaspis_sedecimpunctata_583 | 0.184 |
| Didion_punctatum_644 | Tytthaspis_sedecimpunctata_583 | 0.184 |
| Aphidecta_obliterata_185 | Tytthaspis_sedecimpunctata_585 | 0.184 |
| Aphidecta_obliterata_186 | Tytthaspis_sedecimpunctata_585 | 0.184 |
| Coccinella_septempunctata_242 | Tytthaspis_sedecimpunctata_585 | 0.184 |
| Coccinella_septempunctata_244 | Tytthaspis_sedecimpunctata_585 | 0.184 |
| Coccinella_septempunctata_245 | Tytthaspis_sedecimpunctata_585 | 0.184 |
| Coccinella_septempunctata_247 | Tytthaspis_sedecimpunctata_585 | 0.184 |
| Coccinella_septempunctata_248 | Tytthaspis_sedecimpunctata_585 | 0.184 |
| Coccinella_septempunctata_249 | Tytthaspis_sedecimpunctata_585 | 0.184 |
| Coccinella_septempunctata_252 | Tytthaspis_sedecimpunctata_585 | 0.184 |
| Coccinella_septempunctata_255 | Tytthaspis_sedecimpunctata_585 | 0.184 |

| | | |
|---|---|---|
| Coccinella_septempunctata_256 | Tytthaspis_sedecimpunctata_585 | 0.184 |
| Scymnus_camptodromus_714 | Tytthaspis_sedecimpunctata_585 | 0.184 |
| Aphidecta_obliterata_181 | Tytthaspis_sedecimpunctata_587 | 0.184 |
| Myzia_pullata_518 | Tytthaspis_sedecimpunctata_587 | 0.184 |
| Propylaea_quatuordecimpunctata_547 | Tytthaspis_sedecimpunctata_587 | 0.184 |
| Aphidecta_obliterata_176 | Tytthaspis_sedecimpunctata_588 | 0.184 |
| Scymnus_caurinus_720 | Tytthaspis_sedecimpunctata_589 | 0.184 |
| Scymnus_caurinus_721 | Tytthaspis_sedecimpunctata_589 | 0.184 |
| Scymnus_caurinus_722 | Tytthaspis_sedecimpunctata_589 | 0.184 |
| Aphidecta_obliterata_183 | Tytthaspis_sedecimpunctata_590 | 0.184 |
| Coccinella_septempunctata_242 | Tytthaspis_sedecimpunctata_590 | 0.184 |
| Coccinella_septempunctata_244 | Tytthaspis_sedecimpunctata_590 | 0.184 |
| Coccinella_septempunctata_245 | Tytthaspis_sedecimpunctata_590 | 0.184 |
| Coccinella_septempunctata_247 | Tytthaspis_sedecimpunctata_590 | 0.184 |
| Coccinella_septempunctata_248 | Tytthaspis_sedecimpunctata_590 | 0.184 |
| Coccinella_septempunctata_249 | Tytthaspis_sedecimpunctata_590 | 0.184 |
| Coccinella_septempunctata_252 | Tytthaspis_sedecimpunctata_590 | 0.184 |
| Coccinella_septempunctata_255 | Tytthaspis_sedecimpunctata_590 | 0.184 |
| Coccinella_septempunctata_256 | Tytthaspis_sedecimpunctata_590 | 0.184 |
| Scymnus_camptodromus_714 | Tytthaspis_sedecimpunctata_590 | 0.184 |
| Aphidecta_obliterata_181 | Tytthaspis_sedecimpunctata_591 | 0.184 |
| Coccinella_septempunctata_242 | Tytthaspis_sedecimpunctata_592 | 0.184 |
| Coccinella_septempunctata_244 | Tytthaspis_sedecimpunctata_592 | 0.184 |
| Coccinella_septempunctata_245 | Tytthaspis_sedecimpunctata_592 | 0.184 |
| Coccinella_septempunctata_247 | Tytthaspis_sedecimpunctata_592 | 0.184 |
| Coccinella_septempunctata_248 | Tytthaspis_sedecimpunctata_592 | 0.184 |
| Coccinella_septempunctata_249 | Tytthaspis_sedecimpunctata_592 | 0.184 |
| Coccinella_septempunctata_252 | Tytthaspis_sedecimpunctata_592 | 0.184 |
| Coccinella_septempunctata_255 | Tytthaspis_sedecimpunctata_592 | 0.184 |
| Coccinella_septempunctata_256 | Tytthaspis_sedecimpunctata_592 | 0.184 |
| Didion_punctatum_644 | Tytthaspis_sedecimpunctata_592 | 0.184 |
| Scymnus_camptodromus_714 | Tytthaspis_sedecimpunctata_592 | 0.184 |
| Aphidecta_obliterata_183 | Tytthaspis_sedecimpunctata_593 | 0.184 |
| Coccinella_septempunctata_242 | Tytthaspis_sedecimpunctata_593 | 0.184 |
| Coccinella_septempunctata_244 | Tytthaspis_sedecimpunctata_593 | 0.184 |
| Coccinella_septempunctata_245 | Tytthaspis_sedecimpunctata_593 | 0.184 |
| Coccinella_septempunctata_247 | Tytthaspis_sedecimpunctata_593 | 0.184 |
| Coccinella_septempunctata_248 | Tytthaspis_sedecimpunctata_593 | 0.184 |
| Coccinella_septempunctata_249 | Tytthaspis_sedecimpunctata_593 | 0.184 |
| Coccinella_septempunctata_252 | Tytthaspis_sedecimpunctata_593 | 0.184 |
| Coccinella_septempunctata_255 | Tytthaspis_sedecimpunctata_593 | 0.184 |
| Coccinella_septempunctata_256 | Tytthaspis_sedecimpunctata_593 | 0.184 |
| Scymnus_camptodromus_714 | Tytthaspis_sedecimpunctata_593 | 0.184 |
| Aphidecta_obliterata_183 | Tytthaspis_sedecimpunctata_594 | 0.184 |
| Coccinella_septempunctata_242 | Tytthaspis_sedecimpunctata_594 | 0.184 |
| Coccinella_septempunctata_244 | Tytthaspis_sedecimpunctata_594 | 0.184 |
| Coccinella_septempunctata_245 | Tytthaspis_sedecimpunctata_594 | 0.184 |
| Coccinella_septempunctata_247 | Tytthaspis_sedecimpunctata_594 | 0.184 |
| Coccinella_septempunctata_248 | Tytthaspis_sedecimpunctata_594 | 0.184 |
| Coccinella_septempunctata_249 | Tytthaspis_sedecimpunctata_594 | 0.184 |
| Coccinella_septempunctata_252 | Tytthaspis_sedecimpunctata_594 | 0.184 |
| Coccinella_septempunctata_255 | Tytthaspis_sedecimpunctata_594 | 0.184 |
| Coccinella_septempunctata_256 | Tytthaspis_sedecimpunctata_594 | 0.184 |
| Scymnus_camptodromus_714 | Tytthaspis_sedecimpunctata_594 | 0.184 |
| Aphidecta_obliterata_183 | Tytthaspis_sedecimpunctata_595 | 0.184 |
| Coccinella_septempunctata_242 | Tytthaspis_sedecimpunctata_595 | 0.184 |

| | | |
|---|---|---|
| Coccinella_septempunctata_244 | Tytthaspis_sedecimpunctata_595 | 0.184 |
| Coccinella_septempunctata_245 | Tytthaspis_sedecimpunctata_595 | 0.184 |
| Coccinella_septempunctata_247 | Tytthaspis_sedecimpunctata_595 | 0.184 |
| Coccinella_septempunctata_248 | Tytthaspis_sedecimpunctata_595 | 0.184 |
| Coccinella_septempunctata_249 | Tytthaspis_sedecimpunctata_595 | 0.184 |
| Coccinella_septempunctata_252 | Tytthaspis_sedecimpunctata_595 | 0.184 |
| Coccinella_septempunctata_255 | Tytthaspis_sedecimpunctata_595 | 0.184 |
| Coccinella_septempunctata_256 | Tytthaspis_sedecimpunctata_595 | 0.184 |
| Scymnus_camptodromus_714 | Tytthaspis_sedecimpunctata_595 | 0.184 |
| Coccidula_scutellata_83 | Scymnus_sinuanodulus_828 | 0.184 |
| Anatis_labiculata_123 | Scymnus_suturalis_834 | 0.184 |
| Anatis_labiculata_124 | Scymnus_suturalis_834 | 0.184 |
| Anatis_labiculata_125 | Scymnus_suturalis_834 | 0.184 |
| Anatis_labiculata_127 | Scymnus_suturalis_834 | 0.184 |
| Anatis_ocellata_143 | Scymnus_suturalis_838 | 0.184 |
| Anatis_ocellata_143 | Scymnus_suturalis_840 | 0.184 |
| Scymnus_haemorrhoidalis_750 | Scymnus_sinuanodulus_829 | 0.184 |
| Scymnus_nebulosus_782 | Subcoccinella_vigintiquatuorpunctata_627 | 0.184 |
| Aphidecta_obliterata_176 | Tytthaspis_sedecimpunctata_577 | 0.184 |
| Coccinella_septempunctata_250 | Tytthaspis_sedecimpunctata_577 | 0.184 |
| Coccinella_septempunctata_251 | Tytthaspis_sedecimpunctata_577 | 0.184 |
| Aphidecta_obliterata_176 | Tytthaspis_sedecimpunctata_578 | 0.184 |
| Coccinella_septempunctata_250 | Tytthaspis_sedecimpunctata_578 | 0.184 |
| Coccinella_septempunctata_251 | Tytthaspis_sedecimpunctata_578 | 0.184 |
| Aphidecta_obliterata_176 | Tytthaspis_sedecimpunctata_579 | 0.184 |
| Coccinella_septempunctata_250 | Tytthaspis_sedecimpunctata_579 | 0.184 |
| Coccinella_septempunctata_251 | Tytthaspis_sedecimpunctata_579 | 0.184 |
| Coccinella_septempunctata_242 | Tytthaspis_sedecimpunctata_580 | 0.184 |
| Coccinella_septempunctata_244 | Tytthaspis_sedecimpunctata_580 | 0.184 |
| Coccinella_septempunctata_245 | Tytthaspis_sedecimpunctata_580 | 0.184 |
| Coccinella_septempunctata_247 | Tytthaspis_sedecimpunctata_580 | 0.184 |
| Coccinella_septempunctata_248 | Tytthaspis_sedecimpunctata_580 | 0.184 |
| Coccinella_septempunctata_249 | Tytthaspis_sedecimpunctata_580 | 0.184 |
| Coccinella_septempunctata_252 | Tytthaspis_sedecimpunctata_580 | 0.184 |
| Coccinella_septempunctata_255 | Tytthaspis_sedecimpunctata_580 | 0.184 |
| Coccinella_septempunctata_256 | Tytthaspis_sedecimpunctata_580 | 0.184 |
| Aphidecta_obliterata_176 | Tytthaspis_sedecimpunctata_581 | 0.184 |
| Coccinella_septempunctata_250 | Tytthaspis_sedecimpunctata_581 | 0.184 |
| Coccinella_septempunctata_251 | Tytthaspis_sedecimpunctata_581 | 0.184 |
| Aphidecta_obliterata_176 | Tytthaspis_sedecimpunctata_582 | 0.184 |
| Coccinella_septempunctata_250 | Tytthaspis_sedecimpunctata_582 | 0.184 |
| Coccinella_septempunctata_251 | Tytthaspis_sedecimpunctata_582 | 0.184 |
| Coccinella_septempunctata_254 | Tytthaspis_sedecimpunctata_583 | 0.184 |
| Coccinella_septempunctata_242 | Tytthaspis_sedecimpunctata_584 | 0.184 |
| Coccinella_septempunctata_244 | Tytthaspis_sedecimpunctata_584 | 0.184 |
| Coccinella_septempunctata_245 | Tytthaspis_sedecimpunctata_584 | 0.184 |
| Coccinella_septempunctata_247 | Tytthaspis_sedecimpunctata_584 | 0.184 |
| Coccinella_septempunctata_248 | Tytthaspis_sedecimpunctata_584 | 0.184 |
| Coccinella_septempunctata_249 | Tytthaspis_sedecimpunctata_584 | 0.184 |
| Coccinella_septempunctata_252 | Tytthaspis_sedecimpunctata_584 | 0.184 |
| Coccinella_septempunctata_255 | Tytthaspis_sedecimpunctata_584 | 0.184 |
| Coccinella_septempunctata_256 | Tytthaspis_sedecimpunctata_584 | 0.184 |
| Scymnus_camptodromus_704 | Tytthaspis_sedecimpunctata_584 | 0.184 |
| Coccinella_septempunctata_250 | Tytthaspis_sedecimpunctata_585 | 0.184 |
| Coccinella_septempunctata_251 | Tytthaspis_sedecimpunctata_585 | 0.184 |
| Coccinella_trifasciata_275 | Tytthaspis_sedecimpunctata_587 | 0.184 |

| | | |
|---|---|---|
| Coccinella_trifasciata_277 | Tytthaspis_sedecimpunctata_587 | 0.184 |
| Coccinella_trifasciata_278 | Tytthaspis_sedecimpunctata_587 | 0.184 |
| Coccinella_trifasciata_279 | Tytthaspis_sedecimpunctata_587 | 0.184 |
| Coccinella_trifasciata_281 | Tytthaspis_sedecimpunctata_587 | 0.184 |
| Aphidecta_obliterata_176 | Tytthaspis_sedecimpunctata_590 | 0.184 |
| Coccinella_septempunctata_250 | Tytthaspis_sedecimpunctata_590 | 0.184 |
| Coccinella_septempunctata_251 | Tytthaspis_sedecimpunctata_590 | 0.184 |
| Anatis_labiculata_126 | Tytthaspis_sedecimpunctata_591 | 0.184 |
| Coccinella_septempunctata_241 | Tytthaspis_sedecimpunctata_591 | 0.184 |
| Coccinella_septempunctata_243 | Tytthaspis_sedecimpunctata_591 | 0.184 |
| Coccinella_septempunctata_253 | Tytthaspis_sedecimpunctata_591 | 0.184 |
| Coccinella_septempunctata_250 | Tytthaspis_sedecimpunctata_592 | 0.184 |
| Coccinella_septempunctata_251 | Tytthaspis_sedecimpunctata_592 | 0.184 |
| Aphidecta_obliterata_176 | Tytthaspis_sedecimpunctata_593 | 0.184 |
| Aphidecta_obliterata_181 | Tytthaspis_sedecimpunctata_593 | 0.184 |
| Coccinella_septempunctata_250 | Tytthaspis_sedecimpunctata_593 | 0.184 |
| Coccinella_septempunctata_251 | Tytthaspis_sedecimpunctata_593 | 0.184 |
| Aphidecta_obliterata_176 | Tytthaspis_sedecimpunctata_594 | 0.184 |
| Coccinella_septempunctata_250 | Tytthaspis_sedecimpunctata_594 | 0.184 |
| Coccinella_septempunctata_251 | Tytthaspis_sedecimpunctata_594 | 0.184 |
| Aphidecta_obliterata_176 | Tytthaspis_sedecimpunctata_595 | 0.184 |
| Coccinella_septempunctata_250 | Tytthaspis_sedecimpunctata_595 | 0.184 |
| Coccinella_septempunctata_251 | Tytthaspis_sedecimpunctata_595 | 0.184 |
| Hyperaspis_lateralis_656 | Scymnus_suturalis_833 | 0.184 |
| Anatis_ocellata_143 | Scymnus_suturalis_834 | 0.184 |
| Anatis_ocellata_143 | Scymnus_suturalis_837 | 0.184 |
| Hyperaspis_binotata_649 | Scymnus_suturalis_843 | 0.184 |
| Hyperaspis_binotata_649 | Scymnus_suturalis_845 | 0.184 |
| Hyperaspis_lateralis_656 | Stethorus_punctillum_852 | 0.184 |
| Hyperaspis_binotata_649 | Subcoccinella_vigintiquatuorpunctata_630 | 0.184 |
| Anatis_labiculata_126 | Tytthaspis_sedecimpunctata_577 | 0.184 |
| Anatis_labiculata_126 | Tytthaspis_sedecimpunctata_578 | 0.184 |
| Anatis_labiculata_126 | Tytthaspis_sedecimpunctata_579 | 0.184 |
| Coccinella_septempunctata_250 | Tytthaspis_sedecimpunctata_580 | 0.184 |
| Coccinella_septempunctata_251 | Tytthaspis_sedecimpunctata_580 | 0.184 |
| Olla_vnigrum_530 | Tytthaspis_sedecimpunctata_580 | 0.184 |
| Anatis_labiculata_126 | Tytthaspis_sedecimpunctata_581 | 0.184 |
| Anatis_labiculata_126 | Tytthaspis_sedecimpunctata_582 | 0.184 |
| Adalia_bipunctata_98 | Tytthaspis_sedecimpunctata_584 | 0.184 |
| Coccinella_septempunctata_250 | Tytthaspis_sedecimpunctata_584 | 0.184 |
| Coccinella_septempunctata_251 | Tytthaspis_sedecimpunctata_584 | 0.184 |
| Coccinella_trifasciata_274 | Tytthaspis_sedecimpunctata_587 | 0.184 |
| Coccinella_undecimpunctata_285 | Tytthaspis_sedecimpunctata_587 | 0.184 |
| Coccinella_undecimpunctata_286 | Tytthaspis_sedecimpunctata_587 | 0.184 |
| Coccinella_undecimpunctata_288 | Tytthaspis_sedecimpunctata_587 | 0.184 |
| Coccinella_trifasciata_275 | Tytthaspis_sedecimpunctata_589 | 0.184 |
| Coccinella_trifasciata_277 | Tytthaspis_sedecimpunctata_589 | 0.184 |
| Coccinella_trifasciata_278 | Tytthaspis_sedecimpunctata_589 | 0.184 |
| Coccinella_trifasciata_279 | Tytthaspis_sedecimpunctata_589 | 0.184 |
| Coccinella_trifasciata_281 | Tytthaspis_sedecimpunctata_589 | 0.184 |
| Scymnus_camptodromus_718 | Tytthaspis_sedecimpunctata_589 | 0.184 |
| Anatis_labiculata_126 | Tytthaspis_sedecimpunctata_590 | 0.184 |
| Olla_vnigrum_530 | Tytthaspis_sedecimpunctata_591 | 0.184 |
| Anatis_labiculata_126 | Tytthaspis_sedecimpunctata_593 | 0.184 |
| Anatis_labiculata_126 | Tytthaspis_sedecimpunctata_594 | 0.184 |
| Anatis_labiculata_126 | Tytthaspis_sedecimpunctata_595 | 0.184 |

Supplementary Table S4 (continued)

| | | |
|---|---|---|
| Hyperaspis_binotata_649 | Scymnus_suturalis_834 | 0.184 |
| Coccinella_quinquepunctata_233 | Tytthaspis_sedecimpunctata_586 | 0.184 |
| Scymnus_frontalis_734 | Scymnus_suturalis_843 | 0.184 |
| Scymnus_frontalis_745 | Scymnus_suturalis_843 | 0.184 |
| Scymnus_frontalis_734 | Scymnus_suturalis_845 | 0.184 |
| Scymnus_frontalis_745 | Scymnus_suturalis_845 | 0.184 |
| Hyperaspis_lateralis_656 | Stethorus_punctillum_859 | 0.184 |
| Coccinella_undecimpunctata_285 | Tytthaspis_sedecimpunctata_577 | 0.184 |
| Coccinella_undecimpunctata_286 | Tytthaspis_sedecimpunctata_577 | 0.184 |
| Coccinella_undecimpunctata_288 | Tytthaspis_sedecimpunctata_577 | 0.184 |
| Psyllobora_renifer_550 | Tytthaspis_sedecimpunctata_577 | 0.184 |
| Coccinella_undecimpunctata_285 | Tytthaspis_sedecimpunctata_578 | 0.184 |
| Coccinella_undecimpunctata_286 | Tytthaspis_sedecimpunctata_578 | 0.184 |
| Coccinella_undecimpunctata_288 | Tytthaspis_sedecimpunctata_578 | 0.184 |
| Psyllobora_renifer_550 | Tytthaspis_sedecimpunctata_578 | 0.184 |
| Coccinella_undecimpunctata_285 | Tytthaspis_sedecimpunctata_579 | 0.184 |
| Coccinella_undecimpunctata_286 | Tytthaspis_sedecimpunctata_579 | 0.184 |
| Coccinella_undecimpunctata_288 | Tytthaspis_sedecimpunctata_579 | 0.184 |
| Psyllobora_renifer_550 | Tytthaspis_sedecimpunctata_579 | 0.184 |
| Coccinella_undecimpunctata_285 | Tytthaspis_sedecimpunctata_581 | 0.184 |
| Coccinella_undecimpunctata_286 | Tytthaspis_sedecimpunctata_581 | 0.184 |
| Coccinella_undecimpunctata_288 | Tytthaspis_sedecimpunctata_581 | 0.184 |
| Psyllobora_renifer_550 | Tytthaspis_sedecimpunctata_581 | 0.184 |
| Coccinella_undecimpunctata_285 | Tytthaspis_sedecimpunctata_582 | 0.184 |
| Coccinella_undecimpunctata_286 | Tytthaspis_sedecimpunctata_582 | 0.184 |
| Coccinella_undecimpunctata_288 | Tytthaspis_sedecimpunctata_582 | 0.184 |
| Psyllobora_renifer_550 | Tytthaspis_sedecimpunctata_582 | 0.184 |
| Scymnus_camptodromus_707 | Tytthaspis_sedecimpunctata_583 | 0.184 |
| Scymnus_camptodromus_708 | Tytthaspis_sedecimpunctata_583 | 0.184 |
| Scymnus_camptodromus_709 | Tytthaspis_sedecimpunctata_583 | 0.184 |
| Scymnus_camptodromus_710 | Tytthaspis_sedecimpunctata_583 | 0.184 |
| Anatis_labiculata_126 | Tytthaspis_sedecimpunctata_584 | 0.184 |
| Scymnus_camptodromus_712 | Tytthaspis_sedecimpunctata_584 | 0.184 |
| Coccinella_undecimpunctata_285 | Tytthaspis_sedecimpunctata_585 | 0.184 |
| Coccinella_undecimpunctata_286 | Tytthaspis_sedecimpunctata_585 | 0.184 |
| Coccinella_undecimpunctata_288 | Tytthaspis_sedecimpunctata_585 | 0.184 |
| Olla_vnigrum_530 | Tytthaspis_sedecimpunctata_585 | 0.184 |
| Scymnus_camptodromus_718 | Tytthaspis_sedecimpunctata_586 | 0.184 |
| Scymnus_auritus_701 | Tytthaspis_sedecimpunctata_587 | 0.184 |
| Scymnus_camptodromus_718 | Tytthaspis_sedecimpunctata_588 | 0.184 |
| Coccinella_trifasciata_274 | Tytthaspis_sedecimpunctata_589 | 0.184 |
| Psyllobora_renifer_550 | Tytthaspis_sedecimpunctata_589 | 0.184 |
| Coccinella_undecimpunctata_285 | Tytthaspis_sedecimpunctata_590 | 0.184 |
| Coccinella_undecimpunctata_286 | Tytthaspis_sedecimpunctata_590 | 0.184 |
| Coccinella_undecimpunctata_288 | Tytthaspis_sedecimpunctata_590 | 0.184 |
| Psyllobora_renifer_550 | Tytthaspis_sedecimpunctata_590 | 0.184 |
| Coccinella_undecimpunctata_285 | Tytthaspis_sedecimpunctata_592 | 0.184 |
| Coccinella_undecimpunctata_286 | Tytthaspis_sedecimpunctata_592 | 0.184 |
| Coccinella_undecimpunctata_288 | Tytthaspis_sedecimpunctata_592 | 0.184 |
| Psyllobora_renifer_550 | Tytthaspis_sedecimpunctata_592 | 0.184 |
| Olla_vnigrum_530 | Tytthaspis_sedecimpunctata_593 | 0.184 |
| Coccinella_undecimpunctata_285 | Tytthaspis_sedecimpunctata_594 | 0.184 |
| Coccinella_undecimpunctata_286 | Tytthaspis_sedecimpunctata_594 | 0.184 |
| Coccinella_undecimpunctata_288 | Tytthaspis_sedecimpunctata_594 | 0.184 |
| Psyllobora_renifer_550 | Tytthaspis_sedecimpunctata_594 | 0.184 |
| Coccinella_undecimpunctata_285 | Tytthaspis_sedecimpunctata_595 | 0.184 |

| | | |
|---|---|---|
| Coccinella_undecimpunctata_286 | Tytthaspis_sedecimpunctata_595 | 0.184 |
| Coccinella_undecimpunctata_288 | Tytthaspis_sedecimpunctata_595 | 0.184 |
| Psyllobora_renifer_550 | Tytthaspis_sedecimpunctata_595 | 0.184 |
| Chilocorus_nigritus_26 | Scymnus_sinuanodulus_828 | 0.184 |
| Scymnus_frontalis_734 | Scymnus_suturalis_837 | 0.184 |
| Scymnus_frontalis_745 | Scymnus_suturalis_837 | 0.184 |
| Coccinella_quinquepunctata_234 | Tytthaspis_sedecimpunctata_585 | 0.184 |
| Coccidula_scutellata_84 | Scymnus_suturalis_841 | 0.184 |
| Coccidula_scutellata_84 | Scymnus_suturalis_842 | 0.184 |
| Coccidula_scutellata_84 | Scymnus_suturalis_844 | 0.184 |
| Coccidula_scutellata_84 | Scymnus_suturalis_846 | 0.184 |
| Coccidula_scutellata_84 | Scymnus_suturalis_847 | 0.184 |
| Coccidula_scutellata_84 | Scymnus_suturalis_848 | 0.184 |
| Coccidula_scutellata_84 | Scymnus_suturalis_832 | 0.184 |
| Chilocorus_nigritus_30 | Scymnus_suturalis_833 | 0.184 |
| Coccidula_scutellata_84 | Scymnus_suturalis_835 | 0.184 |
| Coccidula_scutellata_84 | Scymnus_suturalis_836 | 0.184 |
| Coccidula_scutellata_84 | Scymnus_suturalis_839 | 0.184 |
| Hyperaspis_binotata_650 | Scymnus_suturalis_843 | 0.184 |
| Hyperaspis_binotata_650 | Scymnus_suturalis_845 | 0.184 |
| Hyperaspis_lateralis_655 | Stethorus_punctillum_859 | 0.184 |
| Scymnus_nubilus_803 | Subcoccinella_vigintiquatuorpunctata_624 | 0.184 |
| Scymnus_nubilus_804 | Subcoccinella_vigintiquatuorpunctata_624 | 0.184 |
| Scymnus_nubilus_809 | Subcoccinella_vigintiquatuorpunctata_624 | 0.184 |
| Hyperaspis_binotata_650 | Subcoccinella_vigintiquatuorpunctata_630 | 0.184 |
| Olla_vnigrum_526 | Tytthaspis_sedecimpunctata_577 | 0.184 |
| Olla_vnigrum_526 | Tytthaspis_sedecimpunctata_578 | 0.184 |
| Olla_vnigrum_526 | Tytthaspis_sedecimpunctata_579 | 0.184 |
| Scymnus_camptodromus_707 | Tytthaspis_sedecimpunctata_580 | 0.184 |
| Scymnus_camptodromus_708 | Tytthaspis_sedecimpunctata_580 | 0.184 |
| Scymnus_camptodromus_709 | Tytthaspis_sedecimpunctata_580 | 0.184 |
| Scymnus_camptodromus_710 | Tytthaspis_sedecimpunctata_580 | 0.184 |
| Olla_vnigrum_526 | Tytthaspis_sedecimpunctata_581 | 0.184 |
| Olla_vnigrum_526 | Tytthaspis_sedecimpunctata_582 | 0.184 |
| Olla_vnigrum_527 | Tytthaspis_sedecimpunctata_583 | 0.184 |
| Olla_vnigrum_528 | Tytthaspis_sedecimpunctata_583 | 0.184 |
| Coccinella_undecimpunctata_291 | Tytthaspis_sedecimpunctata_584 | 0.184 |
| Coccinella_undecimpunctata_291 | Tytthaspis_sedecimpunctata_586 | 0.184 |
| Scymnus_camptodromus_707 | Tytthaspis_sedecimpunctata_587 | 0.184 |
| Scymnus_camptodromus_708 | Tytthaspis_sedecimpunctata_587 | 0.184 |
| Scymnus_camptodromus_709 | Tytthaspis_sedecimpunctata_587 | 0.184 |
| Scymnus_camptodromus_710 | Tytthaspis_sedecimpunctata_587 | 0.184 |
| Olla_vnigrum_526 | Tytthaspis_sedecimpunctata_590 | 0.184 |
| Coccinella_undecimpunctata_285 | Tytthaspis_sedecimpunctata_591 | 0.184 |
| Coccinella_undecimpunctata_286 | Tytthaspis_sedecimpunctata_591 | 0.184 |
| Coccinella_undecimpunctata_288 | Tytthaspis_sedecimpunctata_591 | 0.184 |
| Psyllobora_renifer_550 | Tytthaspis_sedecimpunctata_591 | 0.184 |
| Olla_vnigrum_526 | Tytthaspis_sedecimpunctata_592 | 0.184 |
| Olla_vnigrum_526 | Tytthaspis_sedecimpunctata_594 | 0.184 |
| Olla_vnigrum_526 | Tytthaspis_sedecimpunctata_595 | 0.184 |
| Nephus_quadrimaculatus_668 | Scymnus_rubromaculatus_822 | 0.184 |
| Nephus_quadrimaculatus_673 | Scymnus_rubromaculatus_822 | 0.184 |
| Nephus_quadrimaculatus_668 | Scymnus_rubromaculatus_823 | 0.184 |
| Nephus_quadrimaculatus_673 | Scymnus_rubromaculatus_823 | 0.184 |
| Nephus_quadrimaculatus_668 | Scymnus_rubromaculatus_824 | 0.184 |
| Nephus_quadrimaculatus_673 | Scymnus_rubromaculatus_824 | 0.184 |

| | | |
|---|---|---|
| Nephus_quadrimaculatus_668 | Scymnus_rubromaculatus_825 | 0.184 |
| Nephus_quadrimaculatus_673 | Scymnus_rubromaculatus_825 | 0.184 |
| Nephus_quadrimaculatus_668 | Scymnus_rubromaculatus_826 | 0.184 |
| Nephus_quadrimaculatus_673 | Scymnus_rubromaculatus_826 | 0.184 |
| Nephus_quadrimaculatus_668 | Scymnus_rubromaculatus_827 | 0.184 |
| Nephus_quadrimaculatus_673 | Scymnus_rubromaculatus_827 | 0.184 |
| Chilocorus_nigritus_27 | Scymnus_sinuanodulus_830 | 0.184 |
| Hyperaspis_binotata_650 | Scymnus_suturalis_834 | 0.184 |
| Scymnus_apetzi_692 | Subcoccinella_vigintiquatuorpunctata_630 | 0.184 |
| Exochomus_nigromaculatus_48 | Scymnus_suturalis_843 | 0.184 |
| Exochomus_nigromaculatus_48 | Scymnus_suturalis_845 | 0.184 |
| Scymnus_nubilus_799 | Subcoccinella_vigintiquatuorpunctata_622 | 0.184 |
| Scymnus_nubilus_799 | Subcoccinella_vigintiquatuorpunctata_625 | 0.184 |
| Coccidula_scutellata_77 | Subcoccinella_vigintiquatuorpunctata_627 | 0.184 |
| Coccidula_scutellata_77 | Subcoccinella_vigintiquatuorpunctata_630 | 0.184 |
| Scymnus_nubilus_799 | Subcoccinella_vigintiquatuorpunctata_630 | 0.184 |
| Coccinella_undecimpunctata_291 | Tytthaspis_sedecimpunctata_583 | 0.184 |
| Olla_vnigrum_526 | Tytthaspis_sedecimpunctata_584 | 0.184 |
| Coccinella_undecimpunctata_291 | Tytthaspis_sedecimpunctata_589 | 0.184 |
| Scymnus_frontalis_734 | Scymnus_sinuanodulus_828 | 0.184 |
| Scymnus_frontalis_742 | Scymnus_sinuanodulus_828 | 0.184 |
| Scymnus_frontalis_745 | Scymnus_sinuanodulus_828 | 0.184 |
| Brumoides_suturalis_1 | Scymnus_suturalis_838 | 0.184 |
| Brumoides_suturalis_1 | Scymnus_suturalis_840 | 0.184 |
| Chilocorus_bipustulatus_23 | Scymnus_sinuanodulus_831 | 0.184 |
| Exochomus_nigromaculatus_49 | Scymnus_suturalis_841 | 0.184 |
| Exochomus_nigromaculatus_49 | Scymnus_suturalis_842 | 0.184 |
| Henosepilachna_argus_616 | Scymnus_suturalis_843 | 0.184 |
| Exochomus_nigromaculatus_49 | Scymnus_suturalis_844 | 0.184 |
| Henosepilachna_argus_616 | Scymnus_suturalis_845 | 0.184 |
| Exochomus_nigromaculatus_49 | Scymnus_suturalis_846 | 0.184 |
| Exochomus_nigromaculatus_49 | Scymnus_suturalis_847 | 0.184 |
| Exochomus_nigromaculatus_49 | Scymnus_suturalis_848 | 0.184 |
| Rhyzobius_litura_97 | Subcoccinella_vigintiquatuorpunctata_620 | 0.184 |
| Rhyzobius_litura_97 | Subcoccinella_vigintiquatuorpunctata_621 | 0.184 |
| Rhyzobius_litura_97 | Subcoccinella_vigintiquatuorpunctata_624 | 0.184 |
| Rhyzobius_litura_97 | Subcoccinella_vigintiquatuorpunctata_626 | 0.184 |
| Coccidula_scutellata_81 | Subcoccinella_vigintiquatuorpunctata_627 | 0.184 |
| Coccidula_scutellata_82 | Subcoccinella_vigintiquatuorpunctata_627 | 0.184 |
| Rhyzobius_litura_96 | Subcoccinella_vigintiquatuorpunctata_627 | 0.184 |
| Rhyzobius_litura_97 | Subcoccinella_vigintiquatuorpunctata_628 | 0.184 |
| Rhyzobius_litura_97 | Subcoccinella_vigintiquatuorpunctata_629 | 0.184 |
| Coccidula_scutellata_81 | Subcoccinella_vigintiquatuorpunctata_630 | 0.184 |
| Coccidula_scutellata_82 | Subcoccinella_vigintiquatuorpunctata_630 | 0.184 |
| Rhyzobius_litura_97 | Subcoccinella_vigintiquatuorpunctata_631 | 0.184 |
| Coccinella_undecimpunctata_291 | Tytthaspis_sedecimpunctata_580 | 0.184 |
| Scymnus_auritus_702 | Tytthaspis_sedecimpunctata_584 | 0.184 |
| Scymnus_auritus_702 | Tytthaspis_sedecimpunctata_591 | 0.184 |
| Calvia_quatuordecimguttata_202 | Scymnus_sinuanodulus_830 | 0.184 |
| Calvia_quatuordecimguttata_210 | Scymnus_sinuanodulus_830 | 0.184 |
| Scymnus_frontalis_736 | Scymnus_sinuanodulus_830 | 0.184 |
| Scymnus_frontalis_737 | Scymnus_sinuanodulus_830 | 0.184 |
| Scymnus_frontalis_738 | Scymnus_sinuanodulus_830 | 0.184 |
| Scymnus_frontalis_739 | Scymnus_sinuanodulus_830 | 0.184 |
| Scymnus_frontalis_740 | Scymnus_sinuanodulus_830 | 0.184 |
| Scymnus_frontalis_741 | Scymnus_sinuanodulus_830 | 0.184 |

| | | |
|---|---|---|
| Scymnus_frontalis_743 | Scymnus_sinuanodulus_830 | 0.184 |
| Scymnus_frontalis_744 | Scymnus_sinuanodulus_830 | 0.184 |
| Exochomus_nigromaculatus_49 | Scymnus_suturalis_832 | 0.184 |
| Exochomus_nigromaculatus_49 | Scymnus_suturalis_835 | 0.184 |
| Exochomus_nigromaculatus_49 | Scymnus_suturalis_836 | 0.184 |
| Brumoides_suturalis_1 | Scymnus_suturalis_837 | 0.184 |
| Scymnus_nubilus_799 | Scymnus_suturalis_838 | 0.184 |
| Exochomus_nigromaculatus_49 | Scymnus_suturalis_839 | 0.184 |
| Hyperaspis_binotata_649 | Subcoccinella_vigintiquatuorpunctata_619 | 0.184 |
| Scymnus_nigrinus_787 | Tytthaspis_sedecimpunctata_586 | 0.184 |
| Scymnus_frontalis_735 | Scymnus_suturalis_843 | 0.184 |
| Scymnus_frontalis_735 | Scymnus_suturalis_845 | 0.184 |
| Chilocorus_nigritus_29 | Scymnus_sinuanodulus_830 | 0.184 |
| Chilocorus_stigma_44 | Scymnus_sinuanodulus_831 | 0.184 |
| Chilocorus_stigma_45 | Scymnus_sinuanodulus_831 | 0.184 |
| Chilocorus_stigma_46 | Scymnus_sinuanodulus_831 | 0.184 |
| Scymnus_frontalis_735 | Scymnus_suturalis_837 | 0.184 |
| Scymnus_haemorrhoidalis_747 | Subcoccinella_vigintiquatuorpunctata_620 | 0.184 |
| Scymnus_haemorrhoidalis_748 | Subcoccinella_vigintiquatuorpunctata_620 | 0.184 |
| Scymnus_haemorrhoidalis_747 | Subcoccinella_vigintiquatuorpunctata_621 | 0.184 |
| Scymnus_haemorrhoidalis_748 | Subcoccinella_vigintiquatuorpunctata_621 | 0.184 |
| Rhyzobius_litura_97 | Subcoccinella_vigintiquatuorpunctata_623 | 0.184 |
| Scymnus_haemorrhoidalis_749 | Subcoccinella_vigintiquatuorpunctata_623 | 0.184 |
| Scymnus_haemorrhoidalis_750 | Subcoccinella_vigintiquatuorpunctata_623 | 0.184 |
| Scymnus_haemorrhoidalis_747 | Subcoccinella_vigintiquatuorpunctata_627 | 0.184 |
| Scymnus_haemorrhoidalis_748 | Subcoccinella_vigintiquatuorpunctata_627 | 0.184 |
| Scymnus_haemorrhoidalis_747 | Subcoccinella_vigintiquatuorpunctata_628 | 0.184 |
| Scymnus_haemorrhoidalis_748 | Subcoccinella_vigintiquatuorpunctata_628 | 0.184 |
| Scymnus_haemorrhoidalis_747 | Subcoccinella_vigintiquatuorpunctata_629 | 0.184 |
| Scymnus_haemorrhoidalis_748 | Subcoccinella_vigintiquatuorpunctata_629 | 0.184 |
| Scymnus_haemorrhoidalis_755 | Subcoccinella_vigintiquatuorpunctata_630 | 0.184 |
| Scymnus_haemorrhoidalis_756 | Subcoccinella_vigintiquatuorpunctata_630 | 0.184 |
| Scymnus_haemorrhoidalis_747 | Subcoccinella_vigintiquatuorpunctata_631 | 0.184 |
| Scymnus_haemorrhoidalis_748 | Subcoccinella_vigintiquatuorpunctata_631 | 0.184 |
| Calvia_quatuordecimguttata_206 | Scymnus_sinuanodulus_830 | 0.184 |
| Calvia_quatuordecimguttata_216 | Scymnus_sinuanodulus_830 | 0.184 |
| Hyperaspis_binotata_650 | Subcoccinella_vigintiquatuorpunctata_619 | 0.184 |
| Coccinella_undecimpunctata_289 | Tytthaspis_sedecimpunctata_584 | 0.184 |
| Coccinella_undecimpunctata_290 | Tytthaspis_sedecimpunctata_584 | 0.184 |
| Coccinella_undecimpunctata_289 | Tytthaspis_sedecimpunctata_586 | 0.184 |
| Coccinella_undecimpunctata_290 | Tytthaspis_sedecimpunctata_586 | 0.184 |
| Chilocorus_nigritus_30 | Scymnus_rubromaculatus_822 | 0.184 |
| Chilocorus_nigritus_30 | Scymnus_rubromaculatus_823 | 0.184 |
| Chilocorus_nigritus_30 | Scymnus_rubromaculatus_824 | 0.184 |
| Chilocorus_nigritus_30 | Scymnus_rubromaculatus_825 | 0.184 |
| Chilocorus_nigritus_30 | Scymnus_rubromaculatus_826 | 0.184 |
| Chilocorus_nigritus_30 | Scymnus_rubromaculatus_827 | 0.184 |
| Chilocorus_nigritus_28 | Scymnus_sinuanodulus_830 | 0.184 |
| Scymnus_haemorrhoidalis_751 | Subcoccinella_vigintiquatuorpunctata_620 | 0.184 |
| Scymnus_haemorrhoidalis_754 | Subcoccinella_vigintiquatuorpunctata_620 | 0.184 |
| Scymnus_haemorrhoidalis_757 | Subcoccinella_vigintiquatuorpunctata_620 | 0.184 |
| Scymnus_haemorrhoidalis_758 | Subcoccinella_vigintiquatuorpunctata_620 | 0.184 |
| Scymnus_haemorrhoidalis_751 | Subcoccinella_vigintiquatuorpunctata_621 | 0.184 |
| Scymnus_haemorrhoidalis_754 | Subcoccinella_vigintiquatuorpunctata_621 | 0.184 |
| Scymnus_haemorrhoidalis_757 | Subcoccinella_vigintiquatuorpunctata_621 | 0.184 |
| Scymnus_haemorrhoidalis_758 | Subcoccinella_vigintiquatuorpunctata_621 | 0.184 |

Supplementary Table S4 (continued)

| | | |
|---|---|---|
| Hyperaspis_proba_661 | Subcoccinella_vigintiquatuorpunctata_623 | 0.184 |
| Scymnus_haemorrhoidalis_749 | Subcoccinella_vigintiquatuorpunctata_626 | 0.184 |
| Scymnus_haemorrhoidalis_750 | Subcoccinella_vigintiquatuorpunctata_626 | 0.184 |
| Scymnus_haemorrhoidalis_749 | Subcoccinella_vigintiquatuorpunctata_627 | 0.184 |
| Scymnus_haemorrhoidalis_750 | Subcoccinella_vigintiquatuorpunctata_627 | 0.184 |
| Scymnus_haemorrhoidalis_751 | Subcoccinella_vigintiquatuorpunctata_628 | 0.184 |
| Scymnus_haemorrhoidalis_754 | Subcoccinella_vigintiquatuorpunctata_628 | 0.184 |
| Scymnus_haemorrhoidalis_757 | Subcoccinella_vigintiquatuorpunctata_628 | 0.184 |
| Scymnus_haemorrhoidalis_758 | Subcoccinella_vigintiquatuorpunctata_628 | 0.184 |
| Scymnus_haemorrhoidalis_751 | Subcoccinella_vigintiquatuorpunctata_629 | 0.184 |
| Scymnus_haemorrhoidalis_754 | Subcoccinella_vigintiquatuorpunctata_629 | 0.184 |
| Scymnus_haemorrhoidalis_757 | Subcoccinella_vigintiquatuorpunctata_629 | 0.184 |
| Scymnus_haemorrhoidalis_758 | Subcoccinella_vigintiquatuorpunctata_629 | 0.184 |
| Scymnus_haemorrhoidalis_751 | Subcoccinella_vigintiquatuorpunctata_630 | 0.184 |
| Scymnus_haemorrhoidalis_754 | Subcoccinella_vigintiquatuorpunctata_630 | 0.184 |
| Scymnus_haemorrhoidalis_757 | Subcoccinella_vigintiquatuorpunctata_630 | 0.184 |
| Scymnus_haemorrhoidalis_758 | Subcoccinella_vigintiquatuorpunctata_630 | 0.184 |
| Scymnus_haemorrhoidalis_751 | Subcoccinella_vigintiquatuorpunctata_631 | 0.184 |
| Scymnus_haemorrhoidalis_754 | Subcoccinella_vigintiquatuorpunctata_631 | 0.184 |
| Scymnus_haemorrhoidalis_757 | Subcoccinella_vigintiquatuorpunctata_631 | 0.184 |
| Scymnus_haemorrhoidalis_758 | Subcoccinella_vigintiquatuorpunctata_631 | 0.184 |
| Aphidecta_obliterata_176 | Vibidia_duodecimguttata_597 | 0.184 |
| Aphidecta_obliterata_176 | Vibidia_duodecimguttata_600 | 0.184 |
| Coccidula_scutellata_77 | Subcoccinella_vigintiquatuorpunctata_619 | 0.184 |
| Coccinella_undecimpunctata_289 | Tytthaspis_sedecimpunctata_583 | 0.184 |
| Coccinella_undecimpunctata_290 | Tytthaspis_sedecimpunctata_583 | 0.184 |
| Coccinella_undecimpunctata_289 | Tytthaspis_sedecimpunctata_589 | 0.184 |
| Coccinella_undecimpunctata_290 | Tytthaspis_sedecimpunctata_589 | 0.184 |
| Scymnus_frontalis_735 | Scymnus_sinuanodulus_828 | 0.184 |
| Scymnus_nubilus_797 | Scymnus_suturalis_843 | 0.184 |
| Scymnus_nubilus_797 | Scymnus_suturalis_845 | 0.184 |
| Scymnus_haemorrhoidalis_752 | Subcoccinella_vigintiquatuorpunctata_620 | 0.184 |
| Scymnus_haemorrhoidalis_753 | Subcoccinella_vigintiquatuorpunctata_620 | 0.184 |
| Scymnus_haemorrhoidalis_752 | Subcoccinella_vigintiquatuorpunctata_621 | 0.184 |
| Scymnus_haemorrhoidalis_753 | Subcoccinella_vigintiquatuorpunctata_621 | 0.184 |
| Hyperaspis_proba_661 | Subcoccinella_vigintiquatuorpunctata_622 | 0.184 |
| Hyperaspis_proba_661 | Subcoccinella_vigintiquatuorpunctata_625 | 0.184 |
| Hyperaspis_proba_661 | Subcoccinella_vigintiquatuorpunctata_626 | 0.184 |
| Scymnus_haemorrhoidalis_752 | Subcoccinella_vigintiquatuorpunctata_628 | 0.184 |
| Scymnus_haemorrhoidalis_753 | Subcoccinella_vigintiquatuorpunctata_628 | 0.184 |
| Scymnus_haemorrhoidalis_752 | Subcoccinella_vigintiquatuorpunctata_629 | 0.184 |
| Scymnus_haemorrhoidalis_753 | Subcoccinella_vigintiquatuorpunctata_629 | 0.184 |
| Scymnus_haemorrhoidalis_752 | Subcoccinella_vigintiquatuorpunctata_630 | 0.184 |
| Scymnus_haemorrhoidalis_753 | Subcoccinella_vigintiquatuorpunctata_630 | 0.184 |
| Scymnus_haemorrhoidalis_752 | Subcoccinella_vigintiquatuorpunctata_631 | 0.184 |
| Scymnus_haemorrhoidalis_753 | Subcoccinella_vigintiquatuorpunctata_631 | 0.184 |
| Anisosticta_novemdecimpunctata_174 | Vibidia_duodecimguttata_596 | 0.184 |
| Anisosticta_novemdecimpunctata_174 | Vibidia_duodecimguttata_598 | 0.184 |
| Aphidecta_obliterata_175 | Vibidia_duodecimguttata_599 | 0.184 |
| Aphidecta_obliterata_177 | Vibidia_duodecimguttata_599 | 0.184 |
| Aphidecta_obliterata_180 | Vibidia_duodecimguttata_599 | 0.184 |
| Aphidecta_obliterata_181 | Vibidia_duodecimguttata_599 | 0.184 |
| Aphidecta_obliterata_182 | Vibidia_duodecimguttata_599 | 0.184 |
| Aphidecta_obliterata_184 | Vibidia_duodecimguttata_599 | 0.184 |
| Aphidecta_obliterata_175 | Vibidia_duodecimguttata_601 | 0.184 |
| Aphidecta_obliterata_177 | Vibidia_duodecimguttata_601 | 0.184 |

| | | |
|---|---|---|
| Aphidecta_obliterata_180 | Vibidia_duodecimguttata_601 | 0.184 |
| Aphidecta_obliterata_181 | Vibidia_duodecimguttata_601 | 0.184 |
| Aphidecta_obliterata_182 | Vibidia_duodecimguttata_601 | 0.184 |
| Aphidecta_obliterata_184 | Vibidia_duodecimguttata_601 | 0.184 |
| Aphidecta_obliterata_175 | Vibidia_duodecimguttata_602 | 0.184 |
| Aphidecta_obliterata_177 | Vibidia_duodecimguttata_602 | 0.184 |
| Aphidecta_obliterata_180 | Vibidia_duodecimguttata_602 | 0.184 |
| Aphidecta_obliterata_181 | Vibidia_duodecimguttata_602 | 0.184 |
| Aphidecta_obliterata_182 | Vibidia_duodecimguttata_602 | 0.184 |
| Aphidecta_obliterata_184 | Vibidia_duodecimguttata_602 | 0.184 |
| Anisosticta_novemdecimpunctata_174 | Vibidia_duodecimguttata_603 | 0.184 |
| Hyperaspis_oregona_659 | Scymnus_sinuanodulus_830 | 0.184 |
| Scymnus_nubilus_797 | Scymnus_suturalis_834 | 0.184 |
| Scymnus_nubilus_797 | Scymnus_suturalis_837 | 0.184 |
| Coccidula_scutellata_81 | Subcoccinella_vigintiquatuorpunctata_619 | 0.184 |
| Coccidula_scutellata_82 | Subcoccinella_vigintiquatuorpunctata_619 | 0.184 |
| Rhyzobius_litura_97 | Subcoccinella_vigintiquatuorpunctata_619 | 0.184 |
| Coccinella_undecimpunctata_289 | Tytthaspis_sedecimpunctata_580 | 0.184 |
| Coccinella_undecimpunctata_290 | Tytthaspis_sedecimpunctata_580 | 0.184 |
| Calvia_quatuordecimguttata_211 | Scymnus_sinuanodulus_831 | 0.184 |
| Calvia_quatuordecimguttata_212 | Scymnus_sinuanodulus_831 | 0.184 |
| Calvia_quatuordecimguttata_213 | Scymnus_sinuanodulus_831 | 0.184 |
| Calvia_quatuordecimguttata_214 | Scymnus_sinuanodulus_831 | 0.184 |
| Calvia_quatuordecimguttata_215 | Scymnus_sinuanodulus_831 | 0.184 |
| Scymnus_camptodromus_717 | Tytthaspis_sedecimpunctata_591 | 0.184 |
| Scymnus_camptodromus_711 | Tytthaspis_sedecimpunctata_591 | 0.184 |
| Chilocorus_nigritus_30 | Scymnus_sinuanodulus_831 | 0.184 |
| Myzia_pullata_517 | Vibidia_duodecimguttata_597 | 0.184 |
| Myzia_pullata_517 | Vibidia_duodecimguttata_600 | 0.184 |
| Myzia_pullata_519 | Vibidia_duodecimguttata_603 | 0.184 |
| Scymnus_rubromaculatus_814 | Scymnus_sinuanodulus_830 | 0.184 |
| Scymnus_nubilus_797 | Scymnus_suturalis_840 | 0.184 |
| Scymnus_haemorrhoidalis_755 | Subcoccinella_vigintiquatuorpunctata_619 | 0.184 |
| Scymnus_haemorrhoidalis_756 | Subcoccinella_vigintiquatuorpunctata_619 | 0.184 |
| Calvia_quatuordecimguttata_207 | Scymnus_sinuanodulus_831 | 0.184 |
| Chilocorus_bipustulatus_23 | Scymnus_sinuanodulus_829 | 0.185 |
| Myzia_pullata_517 | Vibidia_duodecimguttata_598 | 0.185 |
| Myzia_pullata_517 | Vibidia_duodecimguttata_599 | 0.185 |
| Myzia_pullata_517 | Vibidia_duodecimguttata_601 | 0.185 |
| Myzia_pullata_517 | Vibidia_duodecimguttata_602 | 0.185 |
| Myzia_pullata_517 | Vibidia_duodecimguttata_603 | 0.185 |
| Scymnus_haemorrhoidalis_749 | Scymnus_sinuanodulus_830 | 0.185 |
| Scymnus_frontalis_746 | Scymnus_sinuanodulus_828 | 0.185 |
| Psyllobora_vigintiduopunctata_571 | Vibidia_duodecimguttata_603 | 0.185 |
| Scymnus_haemorrhoidalis_749 | Scymnus_sinuanodulus_828 | 0.185 |
| Chilocorus_stigma_44 | Scymnus_sinuanodulus_829 | 0.185 |
| Chilocorus_stigma_45 | Scymnus_sinuanodulus_829 | 0.185 |
| Chilocorus_stigma_46 | Scymnus_sinuanodulus_829 | 0.185 |
| Coccinella_undecimpunctata_282 | Scymnus_sinuanodulus_829 | 0.185 |
| Coccinella_undecimpunctata_283 | Scymnus_sinuanodulus_829 | 0.185 |
| Coccinella_undecimpunctata_284 | Scymnus_sinuanodulus_829 | 0.185 |
| Scymnus_apetzi_695 | Subcoccinella_vigintiquatuorpunctata_630 | 0.185 |
| Calvia_quatuordecimguttata_211 | Scymnus_sinuanodulus_829 | 0.185 |
| Calvia_quatuordecimguttata_212 | Scymnus_sinuanodulus_829 | 0.185 |
| Calvia_quatuordecimguttata_213 | Scymnus_sinuanodulus_829 | 0.185 |
| Calvia_quatuordecimguttata_214 | Scymnus_sinuanodulus_829 | 0.185 |

| | | |
|---|---|---|
| Calvia_quatuordecimguttata_215 | Scymnus_sinuanodulus_829 | 0.185 |
| Myzia_oblongoguttata_503 | Vibidia_duodecimguttata_597 | 0.185 |
| Myzia_oblongoguttata_504 | Vibidia_duodecimguttata_597 | 0.185 |
| Myzia_oblongoguttata_505 | Vibidia_duodecimguttata_597 | 0.185 |
| Myzia_oblongoguttata_507 | Vibidia_duodecimguttata_597 | 0.185 |
| Myzia_oblongoguttata_508 | Vibidia_duodecimguttata_597 | 0.185 |
| Myzia_oblongoguttata_509 | Vibidia_duodecimguttata_597 | 0.185 |
| Myzia_oblongoguttata_510 | Vibidia_duodecimguttata_597 | 0.185 |
| Myzia_oblongoguttata_511 | Vibidia_duodecimguttata_597 | 0.185 |
| Myzia_oblongoguttata_512 | Vibidia_duodecimguttata_597 | 0.185 |
| Myzia_oblongoguttata_513 | Vibidia_duodecimguttata_597 | 0.185 |
| Myzia_oblongoguttata_514 | Vibidia_duodecimguttata_597 | 0.185 |
| Myzia_oblongoguttata_516 | Vibidia_duodecimguttata_597 | 0.185 |
| Myzia_oblongoguttata_503 | Vibidia_duodecimguttata_600 | 0.185 |
| Myzia_oblongoguttata_504 | Vibidia_duodecimguttata_600 | 0.185 |
| Myzia_oblongoguttata_505 | Vibidia_duodecimguttata_600 | 0.185 |
| Myzia_oblongoguttata_507 | Vibidia_duodecimguttata_600 | 0.185 |
| Myzia_oblongoguttata_508 | Vibidia_duodecimguttata_600 | 0.185 |
| Myzia_oblongoguttata_509 | Vibidia_duodecimguttata_600 | 0.185 |
| Myzia_oblongoguttata_510 | Vibidia_duodecimguttata_600 | 0.185 |
| Myzia_oblongoguttata_511 | Vibidia_duodecimguttata_600 | 0.185 |
| Myzia_oblongoguttata_512 | Vibidia_duodecimguttata_600 | 0.185 |
| Myzia_oblongoguttata_513 | Vibidia_duodecimguttata_600 | 0.185 |
| Myzia_oblongoguttata_514 | Vibidia_duodecimguttata_600 | 0.185 |
| Myzia_oblongoguttata_516 | Vibidia_duodecimguttata_600 | 0.185 |
| Calvia_quatuordecimguttata_207 | Scymnus_sinuanodulus_829 | 0.185 |
| Scymnus_apetzi_693 | Subcoccinella_vigintiquatuorpunctata_627 | 0.185 |
| Chilocorus_nigritus_30 | Scymnus_sinuanodulus_829 | 0.185 |
| Coccinella_hieroglyphica_226 | Vibidia_duodecimguttata_596 | 0.185 |
| Coccinella_quinquepunctata_236 | Vibidia_duodecimguttata_596 | 0.185 |
| Myzia_oblongoguttata_515 | Vibidia_duodecimguttata_598 | 0.185 |
| Psyllobora_renifer_550 | Vibidia_duodecimguttata_598 | 0.185 |
| Anatis_ocellata_133 | Vibidia_duodecimguttata_599 | 0.185 |
| Anatis_ocellata_137 | Vibidia_duodecimguttata_599 | 0.185 |
| Anatis_ocellata_143 | Vibidia_duodecimguttata_599 | 0.185 |
| Anatis_ocellata_133 | Vibidia_duodecimguttata_601 | 0.185 |
| Anatis_ocellata_137 | Vibidia_duodecimguttata_601 | 0.185 |
| Anatis_ocellata_143 | Vibidia_duodecimguttata_601 | 0.185 |
| Anatis_ocellata_133 | Vibidia_duodecimguttata_602 | 0.185 |
| Anatis_ocellata_137 | Vibidia_duodecimguttata_602 | 0.185 |
| Anatis_ocellata_143 | Vibidia_duodecimguttata_602 | 0.185 |
| Anatis_ocellata_132 | Vibidia_duodecimguttata_599 | 0.185 |
| Anatis_ocellata_134 | Vibidia_duodecimguttata_599 | 0.185 |
| Anatis_ocellata_135 | Vibidia_duodecimguttata_599 | 0.185 |
| Anatis_ocellata_138 | Vibidia_duodecimguttata_599 | 0.185 |
| Anatis_ocellata_139 | Vibidia_duodecimguttata_599 | 0.185 |
| Anatis_ocellata_140 | Vibidia_duodecimguttata_599 | 0.185 |
| Anatis_ocellata_141 | Vibidia_duodecimguttata_599 | 0.185 |
| Anatis_ocellata_142 | Vibidia_duodecimguttata_599 | 0.185 |
| Anatis_ocellata_144 | Vibidia_duodecimguttata_599 | 0.185 |
| Anatis_ocellata_145 | Vibidia_duodecimguttata_599 | 0.185 |
| Anatis_ocellata_146 | Vibidia_duodecimguttata_599 | 0.185 |
| Anatis_ocellata_132 | Vibidia_duodecimguttata_601 | 0.185 |
| Anatis_ocellata_134 | Vibidia_duodecimguttata_601 | 0.185 |
| Anatis_ocellata_135 | Vibidia_duodecimguttata_601 | 0.185 |
| Anatis_ocellata_138 | Vibidia_duodecimguttata_601 | 0.185 |

| | | |
|---|---|---|
| Anatis_ocellata_139 | Vibidia_duodecimguttata_601 | 0.185 |
| Anatis_ocellata_140 | Vibidia_duodecimguttata_601 | 0.185 |
| Anatis_ocellata_141 | Vibidia_duodecimguttata_601 | 0.185 |
| Anatis_ocellata_142 | Vibidia_duodecimguttata_601 | 0.185 |
| Anatis_ocellata_144 | Vibidia_duodecimguttata_601 | 0.185 |
| Anatis_ocellata_145 | Vibidia_duodecimguttata_601 | 0.185 |
| Anatis_ocellata_146 | Vibidia_duodecimguttata_601 | 0.185 |
| Anatis_ocellata_132 | Vibidia_duodecimguttata_602 | 0.185 |
| Anatis_ocellata_134 | Vibidia_duodecimguttata_602 | 0.185 |
| Anatis_ocellata_135 | Vibidia_duodecimguttata_602 | 0.185 |
| Anatis_ocellata_138 | Vibidia_duodecimguttata_602 | 0.185 |
| Anatis_ocellata_139 | Vibidia_duodecimguttata_602 | 0.185 |
| Anatis_ocellata_140 | Vibidia_duodecimguttata_602 | 0.185 |
| Anatis_ocellata_141 | Vibidia_duodecimguttata_602 | 0.185 |
| Anatis_ocellata_142 | Vibidia_duodecimguttata_602 | 0.185 |
| Anatis_ocellata_144 | Vibidia_duodecimguttata_602 | 0.185 |
| Anatis_ocellata_145 | Vibidia_duodecimguttata_602 | 0.185 |
| Anatis_ocellata_146 | Vibidia_duodecimguttata_602 | 0.185 |
| Psyllobora_vigintiduopunctata_566 | Vibidia_duodecimguttata_596 | 0.185 |
| Psyllobora_vigintiduopunctata_566 | Vibidia_duodecimguttata_599 | 0.185 |
| Psyllobora_vigintiduopunctata_566 | Vibidia_duodecimguttata_601 | 0.185 |
| Psyllobora_vigintiduopunctata_566 | Vibidia_duodecimguttata_602 | 0.185 |
| Hyperaspis_campestris_651 | Tytthaspis_sedecimpunctata_585 | 0.185 |
| Hyperaspis_campestris_652 | Tytthaspis_sedecimpunctata_585 | 0.185 |
| Hyperaspis_campestris_653 | Tytthaspis_sedecimpunctata_585 | 0.185 |
| Platynaspis_luteorubra_55 | Tytthaspis_sedecimpunctata_580 | 0.185 |
| Hyperaspis_oregona_658 | Tytthaspis_sedecimpunctata_589 | 0.185 |
| Hyperaspis_oregona_658 | Tytthaspis_sedecimpunctata_592 | 0.185 |
| Platynaspis_luteorubra_52 | Tytthaspis_sedecimpunctata_593 | 0.185 |
| Platynaspis_luteorubra_56 | Tytthaspis_sedecimpunctata_593 | 0.185 |
| Platynaspis_luteorubra_60 | Tytthaspis_sedecimpunctata_593 | 0.185 |
| Hyperaspis_oregona_658 | Tytthaspis_sedecimpunctata_580 | 0.185 |
| Platynaspis_luteorubra_61 | Tytthaspis_sedecimpunctata_583 | 0.185 |
| Platynaspis_luteorubra_61 | Tytthaspis_sedecimpunctata_585 | 0.185 |
| Chilocorus_renipustulatus_33 | Tytthaspis_sedecimpunctata_586 | 0.185 |
| Hyperaspis_oregona_659 | Tytthaspis_sedecimpunctata_586 | 0.185 |
| Chilocorus_renipustulatus_33 | Tytthaspis_sedecimpunctata_588 | 0.185 |
| Hyperaspis_oregona_659 | Tytthaspis_sedecimpunctata_588 | 0.185 |
| Platynaspis_luteorubra_52 | Tytthaspis_sedecimpunctata_588 | 0.185 |
| Platynaspis_luteorubra_56 | Tytthaspis_sedecimpunctata_588 | 0.185 |
| Platynaspis_luteorubra_60 | Tytthaspis_sedecimpunctata_588 | 0.185 |
| Platynaspis_luteorubra_54 | Tytthaspis_sedecimpunctata_593 | 0.185 |
| Platynaspis_luteorubra_57 | Tytthaspis_sedecimpunctata_593 | 0.185 |
| Platynaspis_luteorubra_59 | Tytthaspis_sedecimpunctata_593 | 0.185 |
| Platynaspis_luteorubra_55 | Tytthaspis_sedecimpunctata_584 | 0.185 |
| Platynaspis_luteorubra_61 | Tytthaspis_sedecimpunctata_586 | 0.185 |
| Platynaspis_luteorubra_54 | Tytthaspis_sedecimpunctata_588 | 0.185 |
| Platynaspis_luteorubra_57 | Tytthaspis_sedecimpunctata_588 | 0.185 |
| Platynaspis_luteorubra_59 | Tytthaspis_sedecimpunctata_588 | 0.185 |
| Chilocorus_renipustulatus_33 | Tytthaspis_sedecimpunctata_593 | 0.185 |
| Scymnus_ferrugatus_724 | Scymnus_sinuanodulus_828 | 0.185 |
| Scymnus_ferrugatus_729 | Scymnus_sinuanodulus_828 | 0.185 |
| Chilocorus_renipustulatus_39 | Tytthaspis_sedecimpunctata_577 | 0.185 |
| Hyperaspis_reppensis_664 | Tytthaspis_sedecimpunctata_577 | 0.185 |
| Scymnus_mitior_779 | Tytthaspis_sedecimpunctata_577 | 0.185 |
| Chilocorus_renipustulatus_39 | Tytthaspis_sedecimpunctata_578 | 0.185 |

| | | |
|---|---|---|
| Hyperaspis_reppensis_664 | Tytthaspis_sedecimpunctata_578 | 0.185 |
| Scymnus_mitior_779 | Tytthaspis_sedecimpunctata_578 | 0.185 |
| Chilocorus_renipustulatus_39 | Tytthaspis_sedecimpunctata_579 | 0.185 |
| Hyperaspis_reppensis_664 | Tytthaspis_sedecimpunctata_579 | 0.185 |
| Scymnus_mitior_779 | Tytthaspis_sedecimpunctata_579 | 0.185 |
| Chilocorus_renipustulatus_34 | Tytthaspis_sedecimpunctata_580 | 0.185 |
| Chilocorus_renipustulatus_39 | Tytthaspis_sedecimpunctata_581 | 0.185 |
| Hyperaspis_reppensis_664 | Tytthaspis_sedecimpunctata_581 | 0.185 |
| Scymnus_mitior_779 | Tytthaspis_sedecimpunctata_581 | 0.185 |
| Chilocorus_renipustulatus_39 | Tytthaspis_sedecimpunctata_582 | 0.185 |
| Hyperaspis_reppensis_664 | Tytthaspis_sedecimpunctata_582 | 0.185 |
| Scymnus_mitior_779 | Tytthaspis_sedecimpunctata_582 | 0.185 |
| Chilocorus_renipustulatus_31 | Tytthaspis_sedecimpunctata_583 | 0.185 |
| Chilocorus_renipustulatus_32 | Tytthaspis_sedecimpunctata_583 | 0.185 |
| Chilocorus_renipustulatus_35 | Tytthaspis_sedecimpunctata_583 | 0.185 |
| Chilocorus_renipustulatus_36 | Tytthaspis_sedecimpunctata_583 | 0.185 |
| Chilocorus_renipustulatus_37 | Tytthaspis_sedecimpunctata_583 | 0.185 |
| Chilocorus_renipustulatus_38 | Tytthaspis_sedecimpunctata_583 | 0.185 |
| Chilocorus_renipustulatus_40 | Tytthaspis_sedecimpunctata_583 | 0.185 |
| Chilocorus_renipustulatus_41 | Tytthaspis_sedecimpunctata_583 | 0.185 |
| Chilocorus_renipustulatus_42 | Tytthaspis_sedecimpunctata_583 | 0.185 |
| Chilocorus_renipustulatus_39 | Tytthaspis_sedecimpunctata_585 | 0.185 |
| Hyperaspis_reppensis_665 | Tytthaspis_sedecimpunctata_585 | 0.185 |
| Scymnus_mitior_779 | Tytthaspis_sedecimpunctata_585 | 0.185 |
| Hyperaspis_reppensis_666 | Tytthaspis_sedecimpunctata_589 | 0.185 |
| Chilocorus_renipustulatus_39 | Tytthaspis_sedecimpunctata_590 | 0.185 |
| Hyperaspis_reppensis_664 | Tytthaspis_sedecimpunctata_590 | 0.185 |
| Scymnus_mitior_779 | Tytthaspis_sedecimpunctata_590 | 0.185 |
| Chilocorus_renipustulatus_39 | Tytthaspis_sedecimpunctata_592 | 0.185 |
| Hyperaspis_reppensis_666 | Tytthaspis_sedecimpunctata_592 | 0.185 |
| Scymnus_mitior_779 | Tytthaspis_sedecimpunctata_592 | 0.185 |
| Chilocorus_renipustulatus_39 | Tytthaspis_sedecimpunctata_593 | 0.185 |
| Hyperaspis_reppensis_664 | Tytthaspis_sedecimpunctata_593 | 0.185 |
| Scymnus_mitior_779 | Tytthaspis_sedecimpunctata_593 | 0.185 |
| Chilocorus_renipustulatus_39 | Tytthaspis_sedecimpunctata_594 | 0.185 |
| Hyperaspis_reppensis_664 | Tytthaspis_sedecimpunctata_594 | 0.185 |
| Scymnus_mitior_779 | Tytthaspis_sedecimpunctata_594 | 0.185 |
| Chilocorus_renipustulatus_39 | Tytthaspis_sedecimpunctata_595 | 0.185 |
| Hyperaspis_reppensis_664 | Tytthaspis_sedecimpunctata_595 | 0.185 |
| Scymnus_mitior_779 | Tytthaspis_sedecimpunctata_595 | 0.185 |
| Hyperaspis_reppensis_666 | Tytthaspis_sedecimpunctata_580 | 0.185 |
| Scymnus_loewii_770 | Tytthaspis_sedecimpunctata_580 | 0.185 |
| Scymnus_loewii_773 | Tytthaspis_sedecimpunctata_580 | 0.185 |
| Scymnus_mitior_779 | Tytthaspis_sedecimpunctata_580 | 0.185 |
| Scymnus_loewii_774 | Tytthaspis_sedecimpunctata_583 | 0.185 |
| Chilocorus_renipustulatus_31 | Tytthaspis_sedecimpunctata_584 | 0.185 |
| Chilocorus_renipustulatus_32 | Tytthaspis_sedecimpunctata_584 | 0.185 |
| Chilocorus_renipustulatus_35 | Tytthaspis_sedecimpunctata_584 | 0.185 |
| Chilocorus_renipustulatus_36 | Tytthaspis_sedecimpunctata_584 | 0.185 |
| Chilocorus_renipustulatus_37 | Tytthaspis_sedecimpunctata_584 | 0.185 |
| Chilocorus_renipustulatus_38 | Tytthaspis_sedecimpunctata_584 | 0.185 |
| Chilocorus_renipustulatus_40 | Tytthaspis_sedecimpunctata_584 | 0.185 |
| Chilocorus_renipustulatus_41 | Tytthaspis_sedecimpunctata_584 | 0.185 |
| Chilocorus_renipustulatus_42 | Tytthaspis_sedecimpunctata_584 | 0.185 |
| Hyperaspis_reppensis_664 | Tytthaspis_sedecimpunctata_584 | 0.185 |
| Hyperaspis_reppensis_665 | Tytthaspis_sedecimpunctata_587 | 0.185 |

| | | |
|---|---|---|
| Didion_punctatum_645 | Tytthaspis_sedecimpunctata_589 | 0.185 |
| Propylaea_quatuordecimpunctata_534 | Scymnus_suturalis_838 | 0.185 |
| Propylaea_quatuordecimpunctata_538 | Scymnus_suturalis_838 | 0.185 |
| Propylaea_quatuordecimpunctata_540 | Scymnus_suturalis_838 | 0.185 |
| Propylaea_quatuordecimpunctata_543 | Scymnus_suturalis_838 | 0.185 |
| Propylaea_quatuordecimpunctata_544 | Scymnus_suturalis_838 | 0.185 |
| Propylaea_quatuordecimpunctata_548 | Scymnus_suturalis_838 | 0.185 |
| Propylaea_quatuordecimpunctata_549 | Scymnus_suturalis_838 | 0.185 |
| Propylaea_quatuordecimpunctata_534 | Scymnus_suturalis_840 | 0.185 |
| Propylaea_quatuordecimpunctata_538 | Scymnus_suturalis_840 | 0.185 |
| Propylaea_quatuordecimpunctata_540 | Scymnus_suturalis_840 | 0.185 |
| Propylaea_quatuordecimpunctata_543 | Scymnus_suturalis_840 | 0.185 |
| Propylaea_quatuordecimpunctata_544 | Scymnus_suturalis_840 | 0.185 |
| Propylaea_quatuordecimpunctata_548 | Scymnus_suturalis_840 | 0.185 |
| Propylaea_quatuordecimpunctata_549 | Scymnus_suturalis_840 | 0.185 |
| Propylaea_quatuordecimpunctata_539 | Scymnus_suturalis_841 | 0.185 |
| Propylaea_quatuordecimpunctata_545 | Scymnus_suturalis_841 | 0.185 |
| Propylaea_quatuordecimpunctata_547 | Scymnus_suturalis_841 | 0.185 |
| Propylaea_quatuordecimpunctata_539 | Scymnus_suturalis_842 | 0.185 |
| Propylaea_quatuordecimpunctata_545 | Scymnus_suturalis_842 | 0.185 |
| Propylaea_quatuordecimpunctata_547 | Scymnus_suturalis_842 | 0.185 |
| Propylaea_quatuordecimpunctata_532 | Scymnus_suturalis_843 | 0.185 |
| Propylaea_quatuordecimpunctata_533 | Scymnus_suturalis_843 | 0.185 |
| Propylaea_quatuordecimpunctata_535 | Scymnus_suturalis_843 | 0.185 |
| Propylaea_quatuordecimpunctata_536 | Scymnus_suturalis_843 | 0.185 |
| Propylaea_quatuordecimpunctata_537 | Scymnus_suturalis_843 | 0.185 |
| Propylaea_quatuordecimpunctata_542 | Scymnus_suturalis_843 | 0.185 |
| Propylaea_quatuordecimpunctata_546 | Scymnus_suturalis_843 | 0.185 |
| Propylaea_quatuordecimpunctata_539 | Scymnus_suturalis_844 | 0.185 |
| Propylaea_quatuordecimpunctata_545 | Scymnus_suturalis_844 | 0.185 |
| Propylaea_quatuordecimpunctata_547 | Scymnus_suturalis_844 | 0.185 |
| Propylaea_quatuordecimpunctata_532 | Scymnus_suturalis_845 | 0.185 |
| Propylaea_quatuordecimpunctata_533 | Scymnus_suturalis_845 | 0.185 |
| Propylaea_quatuordecimpunctata_535 | Scymnus_suturalis_845 | 0.185 |
| Propylaea_quatuordecimpunctata_536 | Scymnus_suturalis_845 | 0.185 |
| Propylaea_quatuordecimpunctata_537 | Scymnus_suturalis_845 | 0.185 |
| Propylaea_quatuordecimpunctata_542 | Scymnus_suturalis_845 | 0.185 |
| Propylaea_quatuordecimpunctata_546 | Scymnus_suturalis_845 | 0.185 |
| Propylaea_quatuordecimpunctata_539 | Scymnus_suturalis_846 | 0.185 |
| Propylaea_quatuordecimpunctata_545 | Scymnus_suturalis_846 | 0.185 |
| Propylaea_quatuordecimpunctata_547 | Scymnus_suturalis_846 | 0.185 |
| Propylaea_quatuordecimpunctata_539 | Scymnus_suturalis_847 | 0.185 |
| Propylaea_quatuordecimpunctata_545 | Scymnus_suturalis_847 | 0.185 |
| Propylaea_quatuordecimpunctata_547 | Scymnus_suturalis_847 | 0.185 |
| Propylaea_quatuordecimpunctata_539 | Scymnus_suturalis_848 | 0.185 |
| Propylaea_quatuordecimpunctata_545 | Scymnus_suturalis_848 | 0.185 |
| Propylaea_quatuordecimpunctata_547 | Scymnus_suturalis_848 | 0.185 |
| Coccinella_transversalis_257 | Tytthaspis_sedecimpunctata_577 | 0.185 |
| Coccinella_transversalis_259 | Tytthaspis_sedecimpunctata_577 | 0.185 |
| Coccinella_transversalis_260 | Tytthaspis_sedecimpunctata_577 | 0.185 |
| Coccinella_transversalis_261 | Tytthaspis_sedecimpunctata_577 | 0.185 |
| Coccinella_transversalis_262 | Tytthaspis_sedecimpunctata_577 | 0.185 |
| Coccinella_transversalis_263 | Tytthaspis_sedecimpunctata_577 | 0.185 |
| Coccinella_transversalis_264 | Tytthaspis_sedecimpunctata_577 | 0.185 |
| Myzia_oblongoguttata_503 | Tytthaspis_sedecimpunctata_577 | 0.185 |
| Myzia_oblongoguttata_515 | Tytthaspis_sedecimpunctata_577 | 0.185 |

| | | |
|---|---|---|
| Coccinella_transversalis_257 | Tytthaspis_sedecimpunctata_578 | 0.185 |
| Coccinella_transversalis_259 | Tytthaspis_sedecimpunctata_578 | 0.185 |
| Coccinella_transversalis_260 | Tytthaspis_sedecimpunctata_578 | 0.185 |
| Coccinella_transversalis_261 | Tytthaspis_sedecimpunctata_578 | 0.185 |
| Coccinella_transversalis_262 | Tytthaspis_sedecimpunctata_578 | 0.185 |
| Coccinella_transversalis_263 | Tytthaspis_sedecimpunctata_578 | 0.185 |
| Coccinella_transversalis_264 | Tytthaspis_sedecimpunctata_578 | 0.185 |
| Myzia_oblongoguttata_503 | Tytthaspis_sedecimpunctata_578 | 0.185 |
| Myzia_oblongoguttata_515 | Tytthaspis_sedecimpunctata_578 | 0.185 |
| Coccinella_transversalis_257 | Tytthaspis_sedecimpunctata_579 | 0.185 |
| Coccinella_transversalis_259 | Tytthaspis_sedecimpunctata_579 | 0.185 |
| Coccinella_transversalis_260 | Tytthaspis_sedecimpunctata_579 | 0.185 |
| Coccinella_transversalis_261 | Tytthaspis_sedecimpunctata_579 | 0.185 |
| Coccinella_transversalis_262 | Tytthaspis_sedecimpunctata_579 | 0.185 |
| Coccinella_transversalis_263 | Tytthaspis_sedecimpunctata_579 | 0.185 |
| Coccinella_transversalis_264 | Tytthaspis_sedecimpunctata_579 | 0.185 |
| Myzia_oblongoguttata_503 | Tytthaspis_sedecimpunctata_579 | 0.185 |
| Myzia_oblongoguttata_515 | Tytthaspis_sedecimpunctata_579 | 0.185 |
| Coccinella_transversalis_257 | Tytthaspis_sedecimpunctata_581 | 0.185 |
| Coccinella_transversalis_259 | Tytthaspis_sedecimpunctata_581 | 0.185 |
| Coccinella_transversalis_260 | Tytthaspis_sedecimpunctata_581 | 0.185 |
| Coccinella_transversalis_261 | Tytthaspis_sedecimpunctata_581 | 0.185 |
| Coccinella_transversalis_262 | Tytthaspis_sedecimpunctata_581 | 0.185 |
| Coccinella_transversalis_263 | Tytthaspis_sedecimpunctata_581 | 0.185 |
| Coccinella_transversalis_264 | Tytthaspis_sedecimpunctata_581 | 0.185 |
| Myzia_oblongoguttata_503 | Tytthaspis_sedecimpunctata_581 | 0.185 |
| Myzia_oblongoguttata_515 | Tytthaspis_sedecimpunctata_581 | 0.185 |
| Coccinella_transversalis_257 | Tytthaspis_sedecimpunctata_582 | 0.185 |
| Coccinella_transversalis_259 | Tytthaspis_sedecimpunctata_582 | 0.185 |
| Coccinella_transversalis_260 | Tytthaspis_sedecimpunctata_582 | 0.185 |
| Coccinella_transversalis_261 | Tytthaspis_sedecimpunctata_582 | 0.185 |
| Coccinella_transversalis_262 | Tytthaspis_sedecimpunctata_582 | 0.185 |
| Coccinella_transversalis_263 | Tytthaspis_sedecimpunctata_582 | 0.185 |
| Coccinella_transversalis_264 | Tytthaspis_sedecimpunctata_582 | 0.185 |
| Myzia_oblongoguttata_503 | Tytthaspis_sedecimpunctata_582 | 0.185 |
| Myzia_oblongoguttata_515 | Tytthaspis_sedecimpunctata_582 | 0.185 |
| Myzia_oblongoguttata_506 | Tytthaspis_sedecimpunctata_583 | 0.185 |
| Scymnus_loewii_772 | Tytthaspis_sedecimpunctata_583 | 0.185 |
| Scymnus_ningshanensis_788 | Tytthaspis_sedecimpunctata_583 | 0.185 |
| Scymnus_ningshanensis_791 | Tytthaspis_sedecimpunctata_583 | 0.185 |
| Scymnus_ningshanensis_792 | Tytthaspis_sedecimpunctata_583 | 0.185 |
| Scymnus_ningshanensis_793 | Tytthaspis_sedecimpunctata_583 | 0.185 |
| Scymnus_ningshanensis_794 | Tytthaspis_sedecimpunctata_583 | 0.185 |
| Scymnus_ningshanensis_795 | Tytthaspis_sedecimpunctata_583 | 0.185 |
| Didion_punctatum_645 | Tytthaspis_sedecimpunctata_584 | 0.185 |
| Myzia_oblongoguttata_503 | Tytthaspis_sedecimpunctata_585 | 0.185 |
| Myzia_oblongoguttata_515 | Tytthaspis_sedecimpunctata_585 | 0.185 |
| Didion_punctatum_645 | Tytthaspis_sedecimpunctata_586 | 0.185 |
| Scymnus_loewii_774 | Tytthaspis_sedecimpunctata_586 | 0.185 |
| Didion_punctatum_646 | Tytthaspis_sedecimpunctata_589 | 0.185 |
| Myzia_oblongoguttata_506 | Tytthaspis_sedecimpunctata_589 | 0.185 |
| Coccinella_transversalis_257 | Tytthaspis_sedecimpunctata_590 | 0.185 |
| Coccinella_transversalis_259 | Tytthaspis_sedecimpunctata_590 | 0.185 |
| Coccinella_transversalis_260 | Tytthaspis_sedecimpunctata_590 | 0.185 |
| Coccinella_transversalis_261 | Tytthaspis_sedecimpunctata_590 | 0.185 |
| Coccinella_transversalis_262 | Tytthaspis_sedecimpunctata_590 | 0.185 |

| | | |
|---|---|---|
| Coccinella_transversalis_263 | Tytthaspis_sedecimpunctata_590 | 0.185 |
| Coccinella_transversalis_264 | Tytthaspis_sedecimpunctata_590 | 0.185 |
| Myzia_oblongoguttata_503 | Tytthaspis_sedecimpunctata_590 | 0.185 |
| Myzia_oblongoguttata_515 | Tytthaspis_sedecimpunctata_590 | 0.185 |
| Coccinella_transversalis_257 | Tytthaspis_sedecimpunctata_592 | 0.185 |
| Coccinella_transversalis_259 | Tytthaspis_sedecimpunctata_592 | 0.185 |
| Coccinella_transversalis_260 | Tytthaspis_sedecimpunctata_592 | 0.185 |
| Coccinella_transversalis_261 | Tytthaspis_sedecimpunctata_592 | 0.185 |
| Coccinella_transversalis_262 | Tytthaspis_sedecimpunctata_592 | 0.185 |
| Coccinella_transversalis_263 | Tytthaspis_sedecimpunctata_592 | 0.185 |
| Coccinella_transversalis_264 | Tytthaspis_sedecimpunctata_592 | 0.185 |
| Myzia_oblongoguttata_503 | Tytthaspis_sedecimpunctata_592 | 0.185 |
| Myzia_oblongoguttata_515 | Tytthaspis_sedecimpunctata_592 | 0.185 |
| Coccinella_transversalis_257 | Tytthaspis_sedecimpunctata_594 | 0.185 |
| Coccinella_transversalis_259 | Tytthaspis_sedecimpunctata_594 | 0.185 |
| Coccinella_transversalis_260 | Tytthaspis_sedecimpunctata_594 | 0.185 |
| Coccinella_transversalis_261 | Tytthaspis_sedecimpunctata_594 | 0.185 |
| Coccinella_transversalis_262 | Tytthaspis_sedecimpunctata_594 | 0.185 |
| Coccinella_transversalis_263 | Tytthaspis_sedecimpunctata_594 | 0.185 |
| Coccinella_transversalis_264 | Tytthaspis_sedecimpunctata_594 | 0.185 |
| Myzia_oblongoguttata_503 | Tytthaspis_sedecimpunctata_594 | 0.185 |
| Myzia_oblongoguttata_515 | Tytthaspis_sedecimpunctata_594 | 0.185 |
| Coccinella_transversalis_257 | Tytthaspis_sedecimpunctata_595 | 0.185 |
| Coccinella_transversalis_259 | Tytthaspis_sedecimpunctata_595 | 0.185 |
| Coccinella_transversalis_260 | Tytthaspis_sedecimpunctata_595 | 0.185 |
| Coccinella_transversalis_261 | Tytthaspis_sedecimpunctata_595 | 0.185 |
| Coccinella_transversalis_262 | Tytthaspis_sedecimpunctata_595 | 0.185 |
| Coccinella_transversalis_263 | Tytthaspis_sedecimpunctata_595 | 0.185 |
| Coccinella_transversalis_264 | Tytthaspis_sedecimpunctata_595 | 0.185 |
| Myzia_oblongoguttata_503 | Tytthaspis_sedecimpunctata_595 | 0.185 |
| Myzia_oblongoguttata_515 | Tytthaspis_sedecimpunctata_595 | 0.185 |
| Propylaea_quatuordecimpunctata_539 | Scymnus_suturalis_832 | 0.185 |
| Propylaea_quatuordecimpunctata_545 | Scymnus_suturalis_832 | 0.185 |
| Propylaea_quatuordecimpunctata_547 | Scymnus_suturalis_832 | 0.185 |
| Propylaea_quatuordecimpunctata_534 | Scymnus_suturalis_834 | 0.185 |
| Propylaea_quatuordecimpunctata_538 | Scymnus_suturalis_834 | 0.185 |
| Propylaea_quatuordecimpunctata_540 | Scymnus_suturalis_834 | 0.185 |
| Propylaea_quatuordecimpunctata_543 | Scymnus_suturalis_834 | 0.185 |
| Propylaea_quatuordecimpunctata_544 | Scymnus_suturalis_834 | 0.185 |
| Propylaea_quatuordecimpunctata_548 | Scymnus_suturalis_834 | 0.185 |
| Propylaea_quatuordecimpunctata_549 | Scymnus_suturalis_834 | 0.185 |
| Propylaea_quatuordecimpunctata_539 | Scymnus_suturalis_835 | 0.185 |
| Propylaea_quatuordecimpunctata_545 | Scymnus_suturalis_835 | 0.185 |
| Propylaea_quatuordecimpunctata_547 | Scymnus_suturalis_835 | 0.185 |
| Propylaea_quatuordecimpunctata_539 | Scymnus_suturalis_836 | 0.185 |
| Propylaea_quatuordecimpunctata_545 | Scymnus_suturalis_836 | 0.185 |
| Propylaea_quatuordecimpunctata_547 | Scymnus_suturalis_836 | 0.185 |
| Propylaea_quatuordecimpunctata_534 | Scymnus_suturalis_837 | 0.185 |
| Propylaea_quatuordecimpunctata_538 | Scymnus_suturalis_837 | 0.185 |
| Propylaea_quatuordecimpunctata_540 | Scymnus_suturalis_837 | 0.185 |
| Propylaea_quatuordecimpunctata_543 | Scymnus_suturalis_837 | 0.185 |
| Propylaea_quatuordecimpunctata_544 | Scymnus_suturalis_837 | 0.185 |
| Propylaea_quatuordecimpunctata_548 | Scymnus_suturalis_837 | 0.185 |
| Propylaea_quatuordecimpunctata_549 | Scymnus_suturalis_837 | 0.185 |
| Propylaea_quatuordecimpunctata_539 | Scymnus_suturalis_839 | 0.185 |
| Propylaea_quatuordecimpunctata_545 | Scymnus_suturalis_839 | 0.185 |

| | | |
|---|---|---|
| Propylaea_quatuordecimpunctata_547 | Scymnus_suturalis_839 | 0.185 |
| Chilocorus_stigma_47 | Scymnus_suturalis_841 | 0.185 |
| Coccidula_scutellata_83 | Scymnus_suturalis_841 | 0.185 |
| Chilocorus_stigma_47 | Scymnus_suturalis_842 | 0.185 |
| Coccidula_scutellata_83 | Scymnus_suturalis_842 | 0.185 |
| Chilocorus_stigma_44 | Scymnus_suturalis_843 | 0.185 |
| Chilocorus_stigma_46 | Scymnus_suturalis_843 | 0.185 |
| Chilocorus_stigma_47 | Scymnus_suturalis_844 | 0.185 |
| Coccidula_scutellata_83 | Scymnus_suturalis_844 | 0.185 |
| Chilocorus_stigma_44 | Scymnus_suturalis_845 | 0.185 |
| Chilocorus_stigma_46 | Scymnus_suturalis_845 | 0.185 |
| Chilocorus_stigma_47 | Scymnus_suturalis_846 | 0.185 |
| Coccidula_scutellata_83 | Scymnus_suturalis_846 | 0.185 |
| Chilocorus_stigma_47 | Scymnus_suturalis_847 | 0.185 |
| Coccidula_scutellata_83 | Scymnus_suturalis_847 | 0.185 |
| Chilocorus_stigma_47 | Scymnus_suturalis_848 | 0.185 |
| Coccidula_scutellata_83 | Scymnus_suturalis_848 | 0.185 |
| Hyperaspis_oregona_658 | Stethorus_punctillum_850 | 0.185 |
| Didion_punctatum_646 | Tytthaspis_sedecimpunctata_584 | 0.185 |
| Scymnus_loewii_770 | Tytthaspis_sedecimpunctata_584 | 0.185 |
| Scymnus_nebulosus_782 | Tytthaspis_sedecimpunctata_584 | 0.185 |
| Scymnus_ningshanensis_788 | Tytthaspis_sedecimpunctata_584 | 0.185 |
| Scymnus_ningshanensis_791 | Tytthaspis_sedecimpunctata_584 | 0.185 |
| Scymnus_ningshanensis_792 | Tytthaspis_sedecimpunctata_584 | 0.185 |
| Scymnus_ningshanensis_793 | Tytthaspis_sedecimpunctata_584 | 0.185 |
| Scymnus_ningshanensis_794 | Tytthaspis_sedecimpunctata_584 | 0.185 |
| Scymnus_ningshanensis_795 | Tytthaspis_sedecimpunctata_584 | 0.185 |
| Didion_punctatum_646 | Tytthaspis_sedecimpunctata_586 | 0.185 |
| Scymnus_loewii_772 | Tytthaspis_sedecimpunctata_586 | 0.185 |
| Scymnus_ningshanensis_788 | Tytthaspis_sedecimpunctata_586 | 0.185 |
| Scymnus_ningshanensis_791 | Tytthaspis_sedecimpunctata_586 | 0.185 |
| Scymnus_ningshanensis_792 | Tytthaspis_sedecimpunctata_586 | 0.185 |
| Scymnus_ningshanensis_793 | Tytthaspis_sedecimpunctata_586 | 0.185 |
| Scymnus_ningshanensis_794 | Tytthaspis_sedecimpunctata_586 | 0.185 |
| Scymnus_ningshanensis_795 | Tytthaspis_sedecimpunctata_586 | 0.185 |
| Brumus_quadripustulatus_9 | Tytthaspis_sedecimpunctata_587 | 0.185 |
| Myzia_oblongoguttata_506 | Tytthaspis_sedecimpunctata_587 | 0.185 |
| Scymnus_loewii_770 | Tytthaspis_sedecimpunctata_587 | 0.185 |
| Scymnus_loewii_773 | Tytthaspis_sedecimpunctata_587 | 0.185 |
| Scymnus_ningshanensis_788 | Tytthaspis_sedecimpunctata_588 | 0.185 |
| Scymnus_ningshanensis_791 | Tytthaspis_sedecimpunctata_588 | 0.185 |
| Scymnus_ningshanensis_792 | Tytthaspis_sedecimpunctata_588 | 0.185 |
| Scymnus_ningshanensis_793 | Tytthaspis_sedecimpunctata_588 | 0.185 |
| Scymnus_ningshanensis_794 | Tytthaspis_sedecimpunctata_588 | 0.185 |
| Scymnus_ningshanensis_795 | Tytthaspis_sedecimpunctata_588 | 0.185 |
| Aphidecta_obliterata_183 | Tytthaspis_sedecimpunctata_589 | 0.185 |
| Didion_punctatum_644 | Tytthaspis_sedecimpunctata_589 | 0.185 |
| Coccinella_transversalis_257 | Tytthaspis_sedecimpunctata_591 | 0.185 |
| Coccinella_transversalis_259 | Tytthaspis_sedecimpunctata_591 | 0.185 |
| Coccinella_transversalis_260 | Tytthaspis_sedecimpunctata_591 | 0.185 |
| Coccinella_transversalis_261 | Tytthaspis_sedecimpunctata_591 | 0.185 |
| Coccinella_transversalis_262 | Tytthaspis_sedecimpunctata_591 | 0.185 |
| Coccinella_transversalis_263 | Tytthaspis_sedecimpunctata_591 | 0.185 |
| Coccinella_transversalis_264 | Tytthaspis_sedecimpunctata_591 | 0.185 |
| Myzia_oblongoguttata_504 | Tytthaspis_sedecimpunctata_591 | 0.185 |
| Myzia_oblongoguttata_505 | Tytthaspis_sedecimpunctata_591 | 0.185 |

| | | |
|---|---|---|
| Myzia_oblongoguttata_507 | Tytthaspis_sedecimpunctata_591 | 0.185 |
| Myzia_oblongoguttata_508 | Tytthaspis_sedecimpunctata_591 | 0.185 |
| Myzia_oblongoguttata_509 | Tytthaspis_sedecimpunctata_591 | 0.185 |
| Myzia_oblongoguttata_510 | Tytthaspis_sedecimpunctata_591 | 0.185 |
| Myzia_oblongoguttata_511 | Tytthaspis_sedecimpunctata_591 | 0.185 |
| Myzia_oblongoguttata_512 | Tytthaspis_sedecimpunctata_591 | 0.185 |
| Myzia_oblongoguttata_513 | Tytthaspis_sedecimpunctata_591 | 0.185 |
| Myzia_oblongoguttata_514 | Tytthaspis_sedecimpunctata_591 | 0.185 |
| Myzia_oblongoguttata_516 | Tytthaspis_sedecimpunctata_591 | 0.185 |
| Myzia_pullata_518 | Tytthaspis_sedecimpunctata_591 | 0.185 |
| Scymnus_ningshanensis_788 | Tytthaspis_sedecimpunctata_591 | 0.185 |
| Scymnus_ningshanensis_791 | Tytthaspis_sedecimpunctata_591 | 0.185 |
| Scymnus_ningshanensis_792 | Tytthaspis_sedecimpunctata_591 | 0.185 |
| Scymnus_ningshanensis_793 | Tytthaspis_sedecimpunctata_591 | 0.185 |
| Scymnus_ningshanensis_794 | Tytthaspis_sedecimpunctata_591 | 0.185 |
| Scymnus_ningshanensis_795 | Tytthaspis_sedecimpunctata_591 | 0.185 |
| Chilocorus_stigma_47 | Scymnus_suturalis_832 | 0.185 |
| Coccidula_scutellata_83 | Scymnus_suturalis_832 | 0.185 |
| Propylaea_quatuordecimpunctata_534 | Scymnus_suturalis_833 | 0.185 |
| Propylaea_quatuordecimpunctata_538 | Scymnus_suturalis_833 | 0.185 |
| Propylaea_quatuordecimpunctata_540 | Scymnus_suturalis_833 | 0.185 |
| Propylaea_quatuordecimpunctata_543 | Scymnus_suturalis_833 | 0.185 |
| Propylaea_quatuordecimpunctata_544 | Scymnus_suturalis_833 | 0.185 |
| Propylaea_quatuordecimpunctata_548 | Scymnus_suturalis_833 | 0.185 |
| Propylaea_quatuordecimpunctata_549 | Scymnus_suturalis_833 | 0.185 |
| Chilocorus_stigma_47 | Scymnus_suturalis_835 | 0.185 |
| Coccidula_scutellata_83 | Scymnus_suturalis_835 | 0.185 |
| Chilocorus_stigma_47 | Scymnus_suturalis_836 | 0.185 |
| Coccidula_scutellata_83 | Scymnus_suturalis_836 | 0.185 |
| Chilocorus_stigma_47 | Scymnus_suturalis_837 | 0.185 |
| Myrrha_octodecimguttata_488 | Scymnus_suturalis_838 | 0.185 |
| Myrrha_octodecimguttata_491 | Scymnus_suturalis_838 | 0.185 |
| Myrrha_octodecimguttata_492 | Scymnus_suturalis_838 | 0.185 |
| Myzia_pullata_518 | Scymnus_suturalis_838 | 0.185 |
| Chilocorus_stigma_47 | Scymnus_suturalis_839 | 0.185 |
| Coccidula_scutellata_83 | Scymnus_suturalis_839 | 0.185 |
| Myzia_pullata_518 | Scymnus_suturalis_840 | 0.185 |
| Illeis_galbula_474 | Vibidia_duodecimguttata_597 | 0.185 |
| Illeis_galbula_475 | Vibidia_duodecimguttata_597 | 0.185 |
| Illeis_galbula_476 | Vibidia_duodecimguttata_597 | 0.185 |
| Illeis_galbula_474 | Vibidia_duodecimguttata_600 | 0.185 |
| Illeis_galbula_475 | Vibidia_duodecimguttata_600 | 0.185 |
| Illeis_galbula_476 | Vibidia_duodecimguttata_600 | 0.185 |
| Anatis_ocellata_136 | Vibidia_duodecimguttata_599 | 0.186 |
| Anatis_ocellata_136 | Vibidia_duodecimguttata_601 | 0.186 |
| Anatis_ocellata_136 | Vibidia_duodecimguttata_602 | 0.186 |
| Myrrha_octodecimguttata_488 | Scymnus_suturalis_841 | 0.186 |
| Myrrha_octodecimguttata_491 | Scymnus_suturalis_841 | 0.186 |
| Myrrha_octodecimguttata_492 | Scymnus_suturalis_841 | 0.186 |
| Myrrha_octodecimguttata_488 | Scymnus_suturalis_842 | 0.186 |
| Myrrha_octodecimguttata_491 | Scymnus_suturalis_842 | 0.186 |
| Myrrha_octodecimguttata_492 | Scymnus_suturalis_842 | 0.186 |
| Myrrha_octodecimguttata_489 | Scymnus_suturalis_843 | 0.186 |
| Myrrha_octodecimguttata_493 | Scymnus_suturalis_843 | 0.186 |
| Myrrha_octodecimguttata_495 | Scymnus_suturalis_843 | 0.186 |
| Myrrha_octodecimguttata_498 | Scymnus_suturalis_843 | 0.186 |

| | | |
|---|---|---|
| Myrrha_octodecimguttata_500 | Scymnus_suturalis_843 | 0.186 |
| Myrrha_octodecimguttata_488 | Scymnus_suturalis_844 | 0.186 |
| Myrrha_octodecimguttata_491 | Scymnus_suturalis_844 | 0.186 |
| Myrrha_octodecimguttata_492 | Scymnus_suturalis_844 | 0.186 |
| Myrrha_octodecimguttata_489 | Scymnus_suturalis_845 | 0.186 |
| Myrrha_octodecimguttata_493 | Scymnus_suturalis_845 | 0.186 |
| Myrrha_octodecimguttata_495 | Scymnus_suturalis_845 | 0.186 |
| Myrrha_octodecimguttata_498 | Scymnus_suturalis_845 | 0.186 |
| Myrrha_octodecimguttata_500 | Scymnus_suturalis_845 | 0.186 |
| Myzia_pullata_518 | Scymnus_suturalis_845 | 0.186 |
| Myrrha_octodecimguttata_488 | Scymnus_suturalis_846 | 0.186 |
| Myrrha_octodecimguttata_491 | Scymnus_suturalis_846 | 0.186 |
| Myrrha_octodecimguttata_492 | Scymnus_suturalis_846 | 0.186 |
| Myrrha_octodecimguttata_488 | Scymnus_suturalis_847 | 0.186 |
| Myrrha_octodecimguttata_491 | Scymnus_suturalis_847 | 0.186 |
| Myrrha_octodecimguttata_492 | Scymnus_suturalis_847 | 0.186 |
| Myrrha_octodecimguttata_488 | Scymnus_suturalis_848 | 0.186 |
| Myrrha_octodecimguttata_491 | Scymnus_suturalis_848 | 0.186 |
| Myrrha_octodecimguttata_492 | Scymnus_suturalis_848 | 0.186 |
| Hyperaspis_lateralis_655 | Subcoccinella_vigintiquatuorpunctata_627 | 0.186 |
| Hyperaspis_lateralis_655 | Subcoccinella_vigintiquatuorpunctata_630 | 0.186 |
| Cheilomenes_sexmaculata_224 | Tytthaspis_sedecimpunctata_583 | 0.186 |
| Didion_punctatum_644 | Tytthaspis_sedecimpunctata_584 | 0.186 |
| Cheilomenes_sexmaculata_224 | Tytthaspis_sedecimpunctata_585 | 0.186 |
| Aphidecta_obliterata_183 | Tytthaspis_sedecimpunctata_586 | 0.186 |
| Didion_punctatum_644 | Tytthaspis_sedecimpunctata_586 | 0.186 |
| Myzia_pullata_519 | Tytthaspis_sedecimpunctata_586 | 0.186 |
| Aphidecta_obliterata_185 | Tytthaspis_sedecimpunctata_587 | 0.186 |
| Aphidecta_obliterata_186 | Tytthaspis_sedecimpunctata_587 | 0.186 |
| Myzia_pullata_519 | Tytthaspis_sedecimpunctata_588 | 0.186 |
| Aphidecta_obliterata_176 | Tytthaspis_sedecimpunctata_589 | 0.186 |
| Aphidecta_obliterata_183 | Tytthaspis_sedecimpunctata_591 | 0.186 |
| Didion_punctatum_645 | Tytthaspis_sedecimpunctata_591 | 0.186 |
| Coccidula_scutellata_80 | Scymnus_sinuanodulus_830 | 0.186 |
| Myrrha_octodecimguttata_488 | Scymnus_suturalis_832 | 0.186 |
| Myrrha_octodecimguttata_491 | Scymnus_suturalis_832 | 0.186 |
| Myrrha_octodecimguttata_492 | Scymnus_suturalis_832 | 0.186 |
| Chilocorus_stigma_44 | Scymnus_suturalis_833 | 0.186 |
| Chilocorus_stigma_46 | Scymnus_suturalis_833 | 0.186 |
| Myzia_pullata_518 | Scymnus_suturalis_834 | 0.186 |
| Myrrha_octodecimguttata_488 | Scymnus_suturalis_835 | 0.186 |
| Myrrha_octodecimguttata_491 | Scymnus_suturalis_835 | 0.186 |
| Myrrha_octodecimguttata_492 | Scymnus_suturalis_835 | 0.186 |
| Myrrha_octodecimguttata_488 | Scymnus_suturalis_836 | 0.186 |
| Myrrha_octodecimguttata_491 | Scymnus_suturalis_836 | 0.186 |
| Myrrha_octodecimguttata_492 | Scymnus_suturalis_836 | 0.186 |
| Myrrha_octodecimguttata_488 | Scymnus_suturalis_839 | 0.186 |
| Myrrha_octodecimguttata_491 | Scymnus_suturalis_839 | 0.186 |
| Myrrha_octodecimguttata_492 | Scymnus_suturalis_839 | 0.186 |
| Anatis_labiculata_123 | Scymnus_suturalis_840 | 0.186 |
| Anatis_labiculata_124 | Scymnus_suturalis_840 | 0.186 |
| Anatis_labiculata_125 | Scymnus_suturalis_840 | 0.186 |
| Anatis_labiculata_127 | Scymnus_suturalis_840 | 0.186 |
| Anatis_labiculata_123 | Scymnus_suturalis_842 | 0.186 |
| Anatis_labiculata_124 | Scymnus_suturalis_842 | 0.186 |
| Anatis_labiculata_125 | Scymnus_suturalis_842 | 0.186 |

| | | |
|---|---|---|
| Anatis_labiculata_127 | Scymnus_suturalis_842 | 0.186 |
| Anatis_labiculata_126 | Scymnus_suturalis_843 | 0.186 |
| Anatis_labiculata_123 | Scymnus_suturalis_844 | 0.186 |
| Anatis_labiculata_124 | Scymnus_suturalis_844 | 0.186 |
| Anatis_labiculata_125 | Scymnus_suturalis_844 | 0.186 |
| Anatis_labiculata_127 | Scymnus_suturalis_844 | 0.186 |
| Anatis_labiculata_126 | Scymnus_suturalis_845 | 0.186 |
| Anatis_labiculata_123 | Scymnus_suturalis_846 | 0.186 |
| Anatis_labiculata_124 | Scymnus_suturalis_846 | 0.186 |
| Anatis_labiculata_125 | Scymnus_suturalis_846 | 0.186 |
| Anatis_labiculata_127 | Scymnus_suturalis_846 | 0.186 |
| Anatis_labiculata_123 | Scymnus_suturalis_847 | 0.186 |
| Anatis_labiculata_124 | Scymnus_suturalis_847 | 0.186 |
| Anatis_labiculata_125 | Scymnus_suturalis_847 | 0.186 |
| Anatis_labiculata_127 | Scymnus_suturalis_847 | 0.186 |
| Anatis_labiculata_123 | Scymnus_suturalis_848 | 0.186 |
| Anatis_labiculata_124 | Scymnus_suturalis_848 | 0.186 |
| Anatis_labiculata_125 | Scymnus_suturalis_848 | 0.186 |
| Anatis_labiculata_127 | Scymnus_suturalis_848 | 0.186 |
| Coccinella_septempunctata_241 | Tytthaspis_sedecimpunctata_577 | 0.186 |
| Coccinella_septempunctata_243 | Tytthaspis_sedecimpunctata_577 | 0.186 |
| Coccinella_septempunctata_253 | Tytthaspis_sedecimpunctata_577 | 0.186 |
| Coccinella_septempunctata_241 | Tytthaspis_sedecimpunctata_578 | 0.186 |
| Coccinella_septempunctata_243 | Tytthaspis_sedecimpunctata_578 | 0.186 |
| Coccinella_septempunctata_253 | Tytthaspis_sedecimpunctata_578 | 0.186 |
| Coccinella_septempunctata_241 | Tytthaspis_sedecimpunctata_579 | 0.186 |
| Coccinella_septempunctata_243 | Tytthaspis_sedecimpunctata_579 | 0.186 |
| Coccinella_septempunctata_253 | Tytthaspis_sedecimpunctata_579 | 0.186 |
| Cheilomenes_sexmaculata_224 | Tytthaspis_sedecimpunctata_580 | 0.186 |
| Coccinella_septempunctata_241 | Tytthaspis_sedecimpunctata_581 | 0.186 |
| Coccinella_septempunctata_243 | Tytthaspis_sedecimpunctata_581 | 0.186 |
| Coccinella_septempunctata_253 | Tytthaspis_sedecimpunctata_581 | 0.186 |
| Coccinella_septempunctata_241 | Tytthaspis_sedecimpunctata_582 | 0.186 |
| Coccinella_septempunctata_243 | Tytthaspis_sedecimpunctata_582 | 0.186 |
| Coccinella_septempunctata_253 | Tytthaspis_sedecimpunctata_582 | 0.186 |
| Aphidecta_obliterata_183 | Tytthaspis_sedecimpunctata_583 | 0.186 |
| Coccinella_septempunctata_242 | Tytthaspis_sedecimpunctata_583 | 0.186 |
| Coccinella_septempunctata_244 | Tytthaspis_sedecimpunctata_583 | 0.186 |
| Coccinella_septempunctata_245 | Tytthaspis_sedecimpunctata_583 | 0.186 |
| Coccinella_septempunctata_247 | Tytthaspis_sedecimpunctata_583 | 0.186 |
| Coccinella_septempunctata_248 | Tytthaspis_sedecimpunctata_583 | 0.186 |
| Coccinella_septempunctata_249 | Tytthaspis_sedecimpunctata_583 | 0.186 |
| Coccinella_septempunctata_252 | Tytthaspis_sedecimpunctata_583 | 0.186 |
| Coccinella_septempunctata_255 | Tytthaspis_sedecimpunctata_583 | 0.186 |
| Coccinella_septempunctata_256 | Tytthaspis_sedecimpunctata_583 | 0.186 |
| Scymnus_caurinus_720 | Tytthaspis_sedecimpunctata_583 | 0.186 |
| Scymnus_caurinus_721 | Tytthaspis_sedecimpunctata_583 | 0.186 |
| Scymnus_caurinus_722 | Tytthaspis_sedecimpunctata_583 | 0.186 |
| Aphidecta_obliterata_183 | Tytthaspis_sedecimpunctata_585 | 0.186 |
| Coccinella_septempunctata_241 | Tytthaspis_sedecimpunctata_585 | 0.186 |
| Coccinella_septempunctata_243 | Tytthaspis_sedecimpunctata_585 | 0.186 |
| Coccinella_septempunctata_253 | Tytthaspis_sedecimpunctata_585 | 0.186 |
| Scymnus_caurinus_720 | Tytthaspis_sedecimpunctata_585 | 0.186 |
| Scymnus_caurinus_721 | Tytthaspis_sedecimpunctata_585 | 0.186 |
| Scymnus_caurinus_722 | Tytthaspis_sedecimpunctata_585 | 0.186 |
| Aphidecta_obliterata_176 | Tytthaspis_sedecimpunctata_586 | 0.186 |

| | | |
|---|---|---|
| Coccinella_septempunctata_254 | Tytthaspis_sedecimpunctata_586 | 0.186 |
| Coccinella_septempunctata_254 | Tytthaspis_sedecimpunctata_588 | 0.186 |
| Coccinella_septempunctata_241 | Tytthaspis_sedecimpunctata_590 | 0.186 |
| Coccinella_septempunctata_243 | Tytthaspis_sedecimpunctata_590 | 0.186 |
| Coccinella_septempunctata_253 | Tytthaspis_sedecimpunctata_590 | 0.186 |
| Aphidecta_obliterata_176 | Tytthaspis_sedecimpunctata_591 | 0.186 |
| Didion_punctatum_646 | Tytthaspis_sedecimpunctata_591 | 0.186 |
| Coccinella_septempunctata_241 | Tytthaspis_sedecimpunctata_592 | 0.186 |
| Coccinella_septempunctata_243 | Tytthaspis_sedecimpunctata_592 | 0.186 |
| Coccinella_septempunctata_253 | Tytthaspis_sedecimpunctata_592 | 0.186 |
| Scymnus_caurinus_720 | Tytthaspis_sedecimpunctata_592 | 0.186 |
| Scymnus_caurinus_721 | Tytthaspis_sedecimpunctata_592 | 0.186 |
| Scymnus_caurinus_722 | Tytthaspis_sedecimpunctata_592 | 0.186 |
| Coccinella_septempunctata_241 | Tytthaspis_sedecimpunctata_593 | 0.186 |
| Coccinella_septempunctata_243 | Tytthaspis_sedecimpunctata_593 | 0.186 |
| Coccinella_septempunctata_253 | Tytthaspis_sedecimpunctata_593 | 0.186 |
| Coccinella_septempunctata_241 | Tytthaspis_sedecimpunctata_594 | 0.186 |
| Coccinella_septempunctata_243 | Tytthaspis_sedecimpunctata_594 | 0.186 |
| Coccinella_septempunctata_253 | Tytthaspis_sedecimpunctata_594 | 0.186 |
| Coccinella_septempunctata_241 | Tytthaspis_sedecimpunctata_595 | 0.186 |
| Coccinella_septempunctata_243 | Tytthaspis_sedecimpunctata_595 | 0.186 |
| Coccinella_septempunctata_253 | Tytthaspis_sedecimpunctata_595 | 0.186 |
| Anatis_labiculata_123 | Scymnus_suturalis_832 | 0.186 |
| Anatis_labiculata_124 | Scymnus_suturalis_832 | 0.186 |
| Anatis_labiculata_125 | Scymnus_suturalis_832 | 0.186 |
| Anatis_labiculata_127 | Scymnus_suturalis_832 | 0.186 |
| Anatis_labiculata_126 | Scymnus_suturalis_834 | 0.186 |
| Anatis_labiculata_123 | Scymnus_suturalis_835 | 0.186 |
| Anatis_labiculata_124 | Scymnus_suturalis_835 | 0.186 |
| Anatis_labiculata_125 | Scymnus_suturalis_835 | 0.186 |
| Anatis_labiculata_127 | Scymnus_suturalis_835 | 0.186 |
| Anatis_labiculata_123 | Scymnus_suturalis_836 | 0.186 |
| Anatis_labiculata_124 | Scymnus_suturalis_836 | 0.186 |
| Anatis_labiculata_125 | Scymnus_suturalis_836 | 0.186 |
| Anatis_labiculata_127 | Scymnus_suturalis_836 | 0.186 |
| Anatis_labiculata_123 | Scymnus_suturalis_839 | 0.186 |
| Anatis_labiculata_124 | Scymnus_suturalis_839 | 0.186 |
| Anatis_labiculata_125 | Scymnus_suturalis_839 | 0.186 |
| Anatis_labiculata_127 | Scymnus_suturalis_839 | 0.186 |
| Anatis_labiculata_123 | Scymnus_suturalis_841 | 0.186 |
| Anatis_labiculata_124 | Scymnus_suturalis_841 | 0.186 |
| Anatis_labiculata_125 | Scymnus_suturalis_841 | 0.186 |
| Anatis_labiculata_127 | Scymnus_suturalis_841 | 0.186 |
| Anatis_mali_128 | Scymnus_suturalis_843 | 0.186 |
| Anatis_mali_129 | Scymnus_suturalis_843 | 0.186 |
| Anatis_mali_130 | Scymnus_suturalis_843 | 0.186 |
| Anatis_mali_131 | Scymnus_suturalis_843 | 0.186 |
| Anatis_mali_128 | Scymnus_suturalis_845 | 0.186 |
| Anatis_mali_129 | Scymnus_suturalis_845 | 0.186 |
| Anatis_mali_130 | Scymnus_suturalis_845 | 0.186 |
| Anatis_mali_131 | Scymnus_suturalis_845 | 0.186 |
| Scymnus_nebulosus_782 | Subcoccinella_vigintiquatuorpunctata_620 | 0.186 |
| Scymnus_nebulosus_782 | Subcoccinella_vigintiquatuorpunctata_621 | 0.186 |
| Scymnus_nebulosus_782 | Subcoccinella_vigintiquatuorpunctata_624 | 0.186 |
| Scymnus_nebulosus_782 | Subcoccinella_vigintiquatuorpunctata_628 | 0.186 |
| Scymnus_nebulosus_782 | Subcoccinella_vigintiquatuorpunctata_629 | 0.186 |

| | | |
|---|---|---|
| Scymnus_nebulosus_782 | Subcoccinella_vigintiquatuorpunctata_631 | 0.186 |
| Coccinella_septempunctata_241 | Tytthaspis_sedecimpunctata_580 | 0.186 |
| Coccinella_septempunctata_243 | Tytthaspis_sedecimpunctata_580 | 0.186 |
| Coccinella_septempunctata_253 | Tytthaspis_sedecimpunctata_580 | 0.186 |
| Scymnus_caurinus_720 | Tytthaspis_sedecimpunctata_580 | 0.186 |
| Scymnus_caurinus_721 | Tytthaspis_sedecimpunctata_580 | 0.186 |
| Scymnus_caurinus_722 | Tytthaspis_sedecimpunctata_580 | 0.186 |
| Aphidecta_obliterata_176 | Tytthaspis_sedecimpunctata_583 | 0.186 |
| Brachiacantha_rotunda_632 | Tytthaspis_sedecimpunctata_583 | 0.186 |
| Brachiacantha_rotunda_633 | Tytthaspis_sedecimpunctata_583 | 0.186 |
| Brachiacantha_rotunda_635 | Tytthaspis_sedecimpunctata_583 | 0.186 |
| Brachiacantha_rotunda_636 | Tytthaspis_sedecimpunctata_583 | 0.186 |
| Coccinella_septempunctata_250 | Tytthaspis_sedecimpunctata_583 | 0.186 |
| Coccinella_septempunctata_251 | Tytthaspis_sedecimpunctata_583 | 0.186 |
| Scymnus_interruptus_767 | Tytthaspis_sedecimpunctata_583 | 0.186 |
| Aphidecta_obliterata_185 | Tytthaspis_sedecimpunctata_584 | 0.186 |
| Aphidecta_obliterata_186 | Tytthaspis_sedecimpunctata_584 | 0.186 |
| Coccinella_septempunctata_241 | Tytthaspis_sedecimpunctata_584 | 0.186 |
| Coccinella_septempunctata_243 | Tytthaspis_sedecimpunctata_584 | 0.186 |
| Coccinella_septempunctata_253 | Tytthaspis_sedecimpunctata_584 | 0.186 |
| Scymnus_camptodromus_714 | Tytthaspis_sedecimpunctata_584 | 0.186 |
| Scymnus_caurinus_720 | Tytthaspis_sedecimpunctata_584 | 0.186 |
| Scymnus_caurinus_721 | Tytthaspis_sedecimpunctata_584 | 0.186 |
| Scymnus_caurinus_722 | Tytthaspis_sedecimpunctata_584 | 0.186 |
| Aphidecta_obliterata_176 | Tytthaspis_sedecimpunctata_585 | 0.186 |
| Brachiacantha_rotunda_632 | Tytthaspis_sedecimpunctata_585 | 0.186 |
| Brachiacantha_rotunda_633 | Tytthaspis_sedecimpunctata_585 | 0.186 |
| Brachiacantha_rotunda_635 | Tytthaspis_sedecimpunctata_585 | 0.186 |
| Brachiacantha_rotunda_636 | Tytthaspis_sedecimpunctata_585 | 0.186 |
| Scymnus_caurinus_720 | Tytthaspis_sedecimpunctata_587 | 0.186 |
| Scymnus_caurinus_721 | Tytthaspis_sedecimpunctata_587 | 0.186 |
| Scymnus_caurinus_722 | Tytthaspis_sedecimpunctata_587 | 0.186 |
| Scymnus_nigrinus_785 | Tytthaspis_sedecimpunctata_588 | 0.186 |
| Scymnus_nigrinus_786 | Tytthaspis_sedecimpunctata_588 | 0.186 |
| Scymnus_camptodromus_705 | Tytthaspis_sedecimpunctata_589 | 0.186 |
| Scymnus_camptodromus_713 | Tytthaspis_sedecimpunctata_589 | 0.186 |
| Scymnus_camptodromus_716 | Tytthaspis_sedecimpunctata_589 | 0.186 |
| Coccinella_septempunctata_246 | Tytthaspis_sedecimpunctata_591 | 0.186 |
| Didion_punctatum_644 | Tytthaspis_sedecimpunctata_591 | 0.186 |
| Scymnus_interruptus_760 | Tytthaspis_sedecimpunctata_592 | 0.186 |
| Scymnus_interruptus_765 | Tytthaspis_sedecimpunctata_592 | 0.186 |
| Scymnus_interruptus_768 | Tytthaspis_sedecimpunctata_592 | 0.186 |
| Brachiacantha_rotunda_632 | Tytthaspis_sedecimpunctata_593 | 0.186 |
| Brachiacantha_rotunda_633 | Tytthaspis_sedecimpunctata_593 | 0.186 |
| Brachiacantha_rotunda_635 | Tytthaspis_sedecimpunctata_593 | 0.186 |
| Brachiacantha_rotunda_636 | Tytthaspis_sedecimpunctata_593 | 0.186 |
| Chilocorus_nigritus_25 | Scymnus_rubromaculatus_822 | 0.186 |
| Chilocorus_nigritus_25 | Scymnus_rubromaculatus_823 | 0.186 |
| Chilocorus_nigritus_25 | Scymnus_rubromaculatus_824 | 0.186 |
| Chilocorus_nigritus_25 | Scymnus_rubromaculatus_825 | 0.186 |
| Chilocorus_nigritus_25 | Scymnus_rubromaculatus_826 | 0.186 |
| Chilocorus_nigritus_25 | Scymnus_rubromaculatus_827 | 0.186 |
| Hyperaspis_lateralis_654 | Scymnus_suturalis_833 | 0.186 |
| Hyperaspis_lateralis_657 | Scymnus_suturalis_833 | 0.186 |
| Illeis_galbula_474 | Vibidia_duodecimguttata_598 | 0.186 |
| Illeis_galbula_475 | Vibidia_duodecimguttata_598 | 0.186 |

| | | |
|---|---|---|
| Illeis_galbula_476 | Vibidia_duodecimguttata_598 | 0.186 |
| Hyperaspis_binotata_649 | Scymnus_suturalis_841 | 0.186 |
| Hyperaspis_binotata_649 | Scymnus_suturalis_842 | 0.186 |
| Hyperaspis_binotata_649 | Scymnus_suturalis_844 | 0.186 |
| Hyperaspis_binotata_649 | Scymnus_suturalis_846 | 0.186 |
| Hyperaspis_binotata_649 | Scymnus_suturalis_847 | 0.186 |
| Hyperaspis_binotata_649 | Scymnus_suturalis_848 | 0.186 |
| Hyperaspis_lateralis_656 | Stethorus_punctillum_857 | 0.186 |
| Hyperaspis_lateralis_656 | Stethorus_punctillum_860 | 0.186 |
| Hyperaspis_binotata_649 | Subcoccinella_vigintiquatuorpunctata_620 | 0.186 |
| Hyperaspis_binotata_649 | Subcoccinella_vigintiquatuorpunctata_621 | 0.186 |
| Hyperaspis_binotata_649 | Subcoccinella_vigintiquatuorpunctata_627 | 0.186 |
| Hyperaspis_binotata_649 | Subcoccinella_vigintiquatuorpunctata_628 | 0.186 |
| Hyperaspis_binotata_649 | Subcoccinella_vigintiquatuorpunctata_629 | 0.186 |
| Hyperaspis_binotata_649 | Subcoccinella_vigintiquatuorpunctata_631 | 0.186 |
| Scymnus_interruptus_760 | Tytthaspis_sedecimpunctata_580 | 0.186 |
| Scymnus_interruptus_765 | Tytthaspis_sedecimpunctata_580 | 0.186 |
| Scymnus_interruptus_768 | Tytthaspis_sedecimpunctata_580 | 0.186 |
| Scymnus_camptodromus_718 | Tytthaspis_sedecimpunctata_583 | 0.186 |
| Aphidecta_obliterata_176 | Tytthaspis_sedecimpunctata_584 | 0.186 |
| Scymnus_camptodromus_705 | Tytthaspis_sedecimpunctata_586 | 0.186 |
| Scymnus_camptodromus_713 | Tytthaspis_sedecimpunctata_586 | 0.186 |
| Scymnus_camptodromus_716 | Tytthaspis_sedecimpunctata_586 | 0.186 |
| Brachiacantha_rotunda_632 | Tytthaspis_sedecimpunctata_588 | 0.186 |
| Brachiacantha_rotunda_633 | Tytthaspis_sedecimpunctata_588 | 0.186 |
| Brachiacantha_rotunda_635 | Tytthaspis_sedecimpunctata_588 | 0.186 |
| Brachiacantha_rotunda_636 | Tytthaspis_sedecimpunctata_588 | 0.186 |
| Psyllobora_renifer_550 | Tytthaspis_sedecimpunctata_588 | 0.186 |
| Scymnus_camptodromus_705 | Tytthaspis_sedecimpunctata_588 | 0.186 |
| Scymnus_camptodromus_713 | Tytthaspis_sedecimpunctata_588 | 0.186 |
| Scymnus_camptodromus_716 | Tytthaspis_sedecimpunctata_588 | 0.186 |
| Scymnus_interruptus_760 | Tytthaspis_sedecimpunctata_588 | 0.186 |
| Scymnus_interruptus_765 | Tytthaspis_sedecimpunctata_588 | 0.186 |
| Scymnus_interruptus_768 | Tytthaspis_sedecimpunctata_588 | 0.186 |
| Hyperaspis_binotata_649 | Scymnus_suturalis_832 | 0.186 |
| Hyperaspis_lateralis_655 | Scymnus_suturalis_833 | 0.186 |
| Hyperaspis_binotata_649 | Scymnus_suturalis_835 | 0.186 |
| Hyperaspis_binotata_649 | Scymnus_suturalis_836 | 0.186 |
| Hyperaspis_binotata_649 | Scymnus_suturalis_839 | 0.186 |
| Scymnus_frontalis_734 | Scymnus_suturalis_840 | 0.186 |
| Scymnus_frontalis_745 | Scymnus_suturalis_840 | 0.186 |
| Coccinella_quinquepunctata_233 | Tytthaspis_sedecimpunctata_589 | 0.186 |
| Brumus_quadripustulatus_3 | Scymnus_sinuanodulus_831 | 0.186 |
| Scymnus_frontalis_734 | Scymnus_suturalis_841 | 0.186 |
| Scymnus_frontalis_745 | Scymnus_suturalis_841 | 0.186 |
| Scymnus_frontalis_734 | Scymnus_suturalis_842 | 0.186 |
| Scymnus_frontalis_745 | Scymnus_suturalis_842 | 0.186 |
| Scymnus_frontalis_736 | Scymnus_suturalis_843 | 0.186 |
| Scymnus_frontalis_737 | Scymnus_suturalis_843 | 0.186 |
| Scymnus_frontalis_738 | Scymnus_suturalis_843 | 0.186 |
| Scymnus_frontalis_739 | Scymnus_suturalis_843 | 0.186 |
| Scymnus_frontalis_740 | Scymnus_suturalis_843 | 0.186 |
| Scymnus_frontalis_741 | Scymnus_suturalis_843 | 0.186 |
| Scymnus_frontalis_743 | Scymnus_suturalis_843 | 0.186 |
| Scymnus_frontalis_744 | Scymnus_suturalis_843 | 0.186 |
| Scymnus_frontalis_734 | Scymnus_suturalis_844 | 0.186 |

Supplementary Table S4 (continued)

| | | |
|---|---|---|
| Scymnus_frontalis_745 | Scymnus_suturalis_844 | 0.186 |
| Scymnus_frontalis_736 | Scymnus_suturalis_845 | 0.186 |
| Scymnus_frontalis_737 | Scymnus_suturalis_845 | 0.186 |
| Scymnus_frontalis_738 | Scymnus_suturalis_845 | 0.186 |
| Scymnus_frontalis_739 | Scymnus_suturalis_845 | 0.186 |
| Scymnus_frontalis_740 | Scymnus_suturalis_845 | 0.186 |
| Scymnus_frontalis_741 | Scymnus_suturalis_845 | 0.186 |
| Scymnus_frontalis_743 | Scymnus_suturalis_845 | 0.186 |
| Scymnus_frontalis_744 | Scymnus_suturalis_845 | 0.186 |
| Scymnus_frontalis_734 | Scymnus_suturalis_846 | 0.186 |
| Scymnus_frontalis_745 | Scymnus_suturalis_846 | 0.186 |
| Scymnus_frontalis_734 | Scymnus_suturalis_847 | 0.186 |
| Scymnus_frontalis_745 | Scymnus_suturalis_847 | 0.186 |
| Scymnus_frontalis_734 | Scymnus_suturalis_848 | 0.186 |
| Scymnus_frontalis_745 | Scymnus_suturalis_848 | 0.186 |
| Hyperaspis_lateralis_655 | Stethorus_punctillum_854 | 0.186 |
| Hyperaspis_lateralis_655 | Stethorus_punctillum_856 | 0.186 |
| Hyperaspis_lateralis_655 | Stethorus_punctillum_857 | 0.186 |
| Hyperaspis_binotata_649 | Subcoccinella_vigintiquatuorpunctata_623 | 0.186 |
| Scymnus_camptodromus_707 | Tytthaspis_sedecimpunctata_577 | 0.186 |
| Scymnus_camptodromus_708 | Tytthaspis_sedecimpunctata_577 | 0.186 |
| Scymnus_camptodromus_709 | Tytthaspis_sedecimpunctata_577 | 0.186 |
| Scymnus_camptodromus_710 | Tytthaspis_sedecimpunctata_577 | 0.186 |
| Scymnus_camptodromus_707 | Tytthaspis_sedecimpunctata_578 | 0.186 |
| Scymnus_camptodromus_708 | Tytthaspis_sedecimpunctata_578 | 0.186 |
| Scymnus_camptodromus_709 | Tytthaspis_sedecimpunctata_578 | 0.186 |
| Scymnus_camptodromus_710 | Tytthaspis_sedecimpunctata_578 | 0.186 |
| Scymnus_camptodromus_707 | Tytthaspis_sedecimpunctata_579 | 0.186 |
| Scymnus_camptodromus_708 | Tytthaspis_sedecimpunctata_579 | 0.186 |
| Scymnus_camptodromus_709 | Tytthaspis_sedecimpunctata_579 | 0.186 |
| Scymnus_camptodromus_710 | Tytthaspis_sedecimpunctata_579 | 0.186 |
| Olla_vnigrum_526 | Tytthaspis_sedecimpunctata_580 | 0.186 |
| Scymnus_camptodromus_718 | Tytthaspis_sedecimpunctata_580 | 0.186 |
| Scymnus_camptodromus_707 | Tytthaspis_sedecimpunctata_581 | 0.186 |
| Scymnus_camptodromus_708 | Tytthaspis_sedecimpunctata_581 | 0.186 |
| Scymnus_camptodromus_709 | Tytthaspis_sedecimpunctata_581 | 0.186 |
| Scymnus_camptodromus_710 | Tytthaspis_sedecimpunctata_581 | 0.186 |
| Scymnus_camptodromus_707 | Tytthaspis_sedecimpunctata_582 | 0.186 |
| Scymnus_camptodromus_708 | Tytthaspis_sedecimpunctata_582 | 0.186 |
| Scymnus_camptodromus_709 | Tytthaspis_sedecimpunctata_582 | 0.186 |
| Scymnus_camptodromus_710 | Tytthaspis_sedecimpunctata_582 | 0.186 |
| Psyllobora_renifer_550 | Tytthaspis_sedecimpunctata_585 | 0.186 |
| Scymnus_camptodromus_707 | Tytthaspis_sedecimpunctata_585 | 0.186 |
| Scymnus_camptodromus_708 | Tytthaspis_sedecimpunctata_585 | 0.186 |
| Scymnus_camptodromus_709 | Tytthaspis_sedecimpunctata_585 | 0.186 |
| Scymnus_camptodromus_710 | Tytthaspis_sedecimpunctata_585 | 0.186 |
| Scymnus_camptodromus_719 | Tytthaspis_sedecimpunctata_589 | 0.186 |
| Scymnus_camptodromus_707 | Tytthaspis_sedecimpunctata_590 | 0.186 |
| Scymnus_camptodromus_708 | Tytthaspis_sedecimpunctata_590 | 0.186 |
| Scymnus_camptodromus_709 | Tytthaspis_sedecimpunctata_590 | 0.186 |
| Scymnus_camptodromus_710 | Tytthaspis_sedecimpunctata_590 | 0.186 |
| Olla_vnigrum_526 | Tytthaspis_sedecimpunctata_591 | 0.186 |
| Scymnus_camptodromus_707 | Tytthaspis_sedecimpunctata_592 | 0.186 |
| Scymnus_camptodromus_708 | Tytthaspis_sedecimpunctata_592 | 0.186 |
| Scymnus_camptodromus_709 | Tytthaspis_sedecimpunctata_592 | 0.186 |
| Scymnus_camptodromus_710 | Tytthaspis_sedecimpunctata_592 | 0.186 |

| | | |
|---|---|---|
| Coccinella_undecimpunctata_282 | Tytthaspis_sedecimpunctata_593 | 0.186 |
| Coccinella_undecimpunctata_283 | Tytthaspis_sedecimpunctata_593 | 0.186 |
| Coccinella_undecimpunctata_284 | Tytthaspis_sedecimpunctata_593 | 0.186 |
| Scymnus_camptodromus_707 | Tytthaspis_sedecimpunctata_593 | 0.186 |
| Scymnus_camptodromus_708 | Tytthaspis_sedecimpunctata_593 | 0.186 |
| Scymnus_camptodromus_709 | Tytthaspis_sedecimpunctata_593 | 0.186 |
| Scymnus_camptodromus_710 | Tytthaspis_sedecimpunctata_593 | 0.186 |
| Scymnus_camptodromus_707 | Tytthaspis_sedecimpunctata_594 | 0.186 |
| Scymnus_camptodromus_708 | Tytthaspis_sedecimpunctata_594 | 0.186 |
| Scymnus_camptodromus_709 | Tytthaspis_sedecimpunctata_594 | 0.186 |
| Scymnus_camptodromus_710 | Tytthaspis_sedecimpunctata_594 | 0.186 |
| Scymnus_camptodromus_707 | Tytthaspis_sedecimpunctata_595 | 0.186 |
| Scymnus_camptodromus_708 | Tytthaspis_sedecimpunctata_595 | 0.186 |
| Scymnus_camptodromus_709 | Tytthaspis_sedecimpunctata_595 | 0.186 |
| Scymnus_camptodromus_710 | Tytthaspis_sedecimpunctata_595 | 0.186 |
| Scymnus_frontalis_734 | Scymnus_suturalis_832 | 0.186 |
| Scymnus_frontalis_745 | Scymnus_suturalis_832 | 0.186 |
| Scymnus_frontalis_734 | Scymnus_suturalis_835 | 0.186 |
| Scymnus_frontalis_745 | Scymnus_suturalis_835 | 0.186 |
| Scymnus_frontalis_734 | Scymnus_suturalis_836 | 0.186 |
| Scymnus_frontalis_745 | Scymnus_suturalis_836 | 0.186 |
| Scymnus_frontalis_736 | Scymnus_suturalis_837 | 0.186 |
| Scymnus_frontalis_737 | Scymnus_suturalis_837 | 0.186 |
| Scymnus_frontalis_738 | Scymnus_suturalis_837 | 0.186 |
| Scymnus_frontalis_739 | Scymnus_suturalis_837 | 0.186 |
| Scymnus_frontalis_740 | Scymnus_suturalis_837 | 0.186 |
| Scymnus_frontalis_741 | Scymnus_suturalis_837 | 0.186 |
| Scymnus_frontalis_743 | Scymnus_suturalis_837 | 0.186 |
| Scymnus_frontalis_744 | Scymnus_suturalis_837 | 0.186 |
| Scymnus_frontalis_734 | Scymnus_suturalis_839 | 0.186 |
| Scymnus_frontalis_745 | Scymnus_suturalis_839 | 0.186 |
| Macronaemia_episcopalis_478 | Scymnus_suturalis_843 | 0.186 |
| Macronaemia_episcopalis_478 | Scymnus_suturalis_845 | 0.186 |
| Coccidula_scutellata_84 | Scymnus_suturalis_838 | 0.186 |
| Coccidula_scutellata_84 | Scymnus_suturalis_840 | 0.186 |
| Coccinella_quinquepunctata_233 | Tytthaspis_sedecimpunctata_587 | 0.186 |
| Hyperaspis_lateralis_655 | Subcoccinella_vigintiquatuorpunctata_619 | 0.186 |
| Coccidula_scutellata_84 | Scymnus_suturalis_834 | 0.186 |
| Hyperaspis_binotata_650 | Scymnus_suturalis_841 | 0.186 |
| Hyperaspis_binotata_650 | Scymnus_suturalis_842 | 0.186 |
| Hyperaspis_binotata_650 | Scymnus_suturalis_844 | 0.186 |
| Hyperaspis_binotata_650 | Scymnus_suturalis_846 | 0.186 |
| Hyperaspis_binotata_650 | Scymnus_suturalis_847 | 0.186 |
| Hyperaspis_binotata_650 | Scymnus_suturalis_848 | 0.186 |
| Hyperaspis_lateralis_656 | Stethorus_punctillum_853 | 0.186 |
| Hyperaspis_binotata_650 | Subcoccinella_vigintiquatuorpunctata_620 | 0.186 |
| Hyperaspis_binotata_650 | Subcoccinella_vigintiquatuorpunctata_621 | 0.186 |
| Scymnus_nubilus_800 | Subcoccinella_vigintiquatuorpunctata_622 | 0.186 |
| Scymnus_nubilus_801 | Subcoccinella_vigintiquatuorpunctata_622 | 0.186 |
| Scymnus_nubilus_802 | Subcoccinella_vigintiquatuorpunctata_622 | 0.186 |
| Scymnus_nubilus_805 | Subcoccinella_vigintiquatuorpunctata_622 | 0.186 |
| Scymnus_nubilus_806 | Subcoccinella_vigintiquatuorpunctata_622 | 0.186 |
| Scymnus_nubilus_807 | Subcoccinella_vigintiquatuorpunctata_622 | 0.186 |
| Scymnus_nubilus_810 | Subcoccinella_vigintiquatuorpunctata_622 | 0.186 |
| Scymnus_nubilus_798 | Subcoccinella_vigintiquatuorpunctata_624 | 0.186 |
| Scymnus_nubilus_802 | Subcoccinella_vigintiquatuorpunctata_624 | 0.186 |

| | | |
|---|---|---|
| Scymnus_nubilus_808 | Subcoccinella_vigintiquatuorpunctata_624 | 0.186 |
| Scymnus_nubilus_800 | Subcoccinella_vigintiquatuorpunctata_625 | 0.186 |
| Scymnus_nubilus_801 | Subcoccinella_vigintiquatuorpunctata_625 | 0.186 |
| Scymnus_nubilus_802 | Subcoccinella_vigintiquatuorpunctata_625 | 0.186 |
| Scymnus_nubilus_805 | Subcoccinella_vigintiquatuorpunctata_625 | 0.186 |
| Scymnus_nubilus_806 | Subcoccinella_vigintiquatuorpunctata_625 | 0.186 |
| Scymnus_nubilus_807 | Subcoccinella_vigintiquatuorpunctata_625 | 0.186 |
| Scymnus_nubilus_810 | Subcoccinella_vigintiquatuorpunctata_625 | 0.186 |
| Hyperaspis_binotata_650 | Subcoccinella_vigintiquatuorpunctata_627 | 0.186 |
| Hyperaspis_binotata_650 | Subcoccinella_vigintiquatuorpunctata_628 | 0.186 |
| Hyperaspis_binotata_650 | Subcoccinella_vigintiquatuorpunctata_629 | 0.186 |
| Scymnus_nubilus_800 | Subcoccinella_vigintiquatuorpunctata_630 | 0.186 |
| Scymnus_nubilus_801 | Subcoccinella_vigintiquatuorpunctata_630 | 0.186 |
| Scymnus_nubilus_802 | Subcoccinella_vigintiquatuorpunctata_630 | 0.186 |
| Scymnus_nubilus_805 | Subcoccinella_vigintiquatuorpunctata_630 | 0.186 |
| Scymnus_nubilus_806 | Subcoccinella_vigintiquatuorpunctata_630 | 0.186 |
| Scymnus_nubilus_807 | Subcoccinella_vigintiquatuorpunctata_630 | 0.186 |
| Scymnus_nubilus_810 | Subcoccinella_vigintiquatuorpunctata_630 | 0.186 |
| Hyperaspis_binotata_650 | Subcoccinella_vigintiquatuorpunctata_631 | 0.186 |
| Olla_vnigrum_527 | Tytthaspis_sedecimpunctata_577 | 0.186 |
| Olla_vnigrum_528 | Tytthaspis_sedecimpunctata_577 | 0.186 |
| Olla_vnigrum_527 | Tytthaspis_sedecimpunctata_578 | 0.186 |
| Olla_vnigrum_528 | Tytthaspis_sedecimpunctata_578 | 0.186 |
| Olla_vnigrum_527 | Tytthaspis_sedecimpunctata_579 | 0.186 |
| Olla_vnigrum_528 | Tytthaspis_sedecimpunctata_579 | 0.186 |
| Olla_vnigrum_527 | Tytthaspis_sedecimpunctata_581 | 0.186 |
| Olla_vnigrum_528 | Tytthaspis_sedecimpunctata_581 | 0.186 |
| Olla_vnigrum_527 | Tytthaspis_sedecimpunctata_582 | 0.186 |
| Olla_vnigrum_528 | Tytthaspis_sedecimpunctata_582 | 0.186 |
| Scymnus_auritus_701 | Tytthaspis_sedecimpunctata_583 | 0.186 |
| Olla_vnigrum_526 | Tytthaspis_sedecimpunctata_585 | 0.186 |
| Scymnus_auritus_701 | Tytthaspis_sedecimpunctata_585 | 0.186 |
| Scymnus_camptodromus_719 | Tytthaspis_sedecimpunctata_586 | 0.186 |
| Coccinella_undecimpunctata_291 | Tytthaspis_sedecimpunctata_587 | 0.186 |
| Scymnus_camptodromus_719 | Tytthaspis_sedecimpunctata_588 | 0.186 |
| Olla_vnigrum_527 | Tytthaspis_sedecimpunctata_590 | 0.186 |
| Olla_vnigrum_528 | Tytthaspis_sedecimpunctata_590 | 0.186 |
| Olla_vnigrum_527 | Tytthaspis_sedecimpunctata_592 | 0.186 |
| Olla_vnigrum_528 | Tytthaspis_sedecimpunctata_592 | 0.186 |
| Scymnus_auritus_701 | Tytthaspis_sedecimpunctata_592 | 0.186 |
| Olla_vnigrum_526 | Tytthaspis_sedecimpunctata_593 | 0.186 |
| Olla_vnigrum_527 | Tytthaspis_sedecimpunctata_594 | 0.186 |
| Olla_vnigrum_528 | Tytthaspis_sedecimpunctata_594 | 0.186 |
| Olla_vnigrum_527 | Tytthaspis_sedecimpunctata_595 | 0.186 |
| Olla_vnigrum_528 | Tytthaspis_sedecimpunctata_595 | 0.186 |
| Nephus_quadrimaculatus_670 | Scymnus_rubromaculatus_822 | 0.186 |
| Nephus_quadrimaculatus_670 | Scymnus_rubromaculatus_823 | 0.186 |
| Nephus_quadrimaculatus_670 | Scymnus_rubromaculatus_824 | 0.186 |
| Nephus_quadrimaculatus_670 | Scymnus_rubromaculatus_825 | 0.186 |
| Nephus_quadrimaculatus_670 | Scymnus_rubromaculatus_826 | 0.186 |
| Nephus_quadrimaculatus_670 | Scymnus_rubromaculatus_827 | 0.186 |
| Hyperaspis_binotata_650 | Scymnus_suturalis_832 | 0.186 |
| Hyperaspis_binotata_650 | Scymnus_suturalis_835 | 0.186 |
| Hyperaspis_binotata_650 | Scymnus_suturalis_836 | 0.186 |
| Hyperaspis_binotata_650 | Scymnus_suturalis_839 | 0.186 |
| Scymnus_apetzi_692 | Subcoccinella_vigintiquatuorpunctata_627 | 0.186 |

| | | |
|---|---|---|
| Chilocorus_bipustulatus_21 | Scymnus_sinuanodulus_830 | 0.186 |
| Coccidula_scutellata_84 | Scymnus_sinuanodulus_830 | 0.186 |
| Exochomus_nigromaculatus_48 | Scymnus_suturalis_841 | 0.186 |
| Exochomus_nigromaculatus_48 | Scymnus_suturalis_842 | 0.186 |
| Hippodamia_parenthesis_439 | Scymnus_suturalis_843 | 0.186 |
| Exochomus_nigromaculatus_48 | Scymnus_suturalis_844 | 0.186 |
| Exochomus_nigromaculatus_48 | Scymnus_suturalis_846 | 0.186 |
| Exochomus_nigromaculatus_48 | Scymnus_suturalis_847 | 0.186 |
| Exochomus_nigromaculatus_48 | Scymnus_suturalis_848 | 0.186 |
| Hyperaspis_lateralis_655 | Stethorus_punctillum_853 | 0.186 |
| Coccidula_scutellata_77 | Subcoccinella_vigintiquatuorpunctata_620 | 0.186 |
| Coccidula_scutellata_77 | Subcoccinella_vigintiquatuorpunctata_621 | 0.186 |
| Coccidula_scutellata_77 | Subcoccinella_vigintiquatuorpunctata_622 | 0.186 |
| Hyperaspis_binotata_650 | Subcoccinella_vigintiquatuorpunctata_623 | 0.186 |
| Scymnus_nubilus_799 | Subcoccinella_vigintiquatuorpunctata_627 | 0.186 |
| Coccidula_scutellata_77 | Subcoccinella_vigintiquatuorpunctata_628 | 0.186 |
| Coccidula_scutellata_77 | Subcoccinella_vigintiquatuorpunctata_629 | 0.186 |
| Coccidula_scutellata_77 | Subcoccinella_vigintiquatuorpunctata_631 | 0.186 |
| Coccinella_undecimpunctata_291 | Tytthaspis_sedecimpunctata_577 | 0.186 |
| Coccinella_undecimpunctata_291 | Tytthaspis_sedecimpunctata_578 | 0.186 |
| Coccinella_undecimpunctata_291 | Tytthaspis_sedecimpunctata_579 | 0.186 |
| Scymnus_auritus_701 | Tytthaspis_sedecimpunctata_580 | 0.186 |
| Coccinella_undecimpunctata_291 | Tytthaspis_sedecimpunctata_581 | 0.186 |
| Coccinella_undecimpunctata_291 | Tytthaspis_sedecimpunctata_582 | 0.186 |
| Olla_vnigrum_527 | Tytthaspis_sedecimpunctata_584 | 0.186 |
| Olla_vnigrum_528 | Tytthaspis_sedecimpunctata_584 | 0.186 |
| Coccinella_undecimpunctata_291 | Tytthaspis_sedecimpunctata_585 | 0.186 |
| Coccinella_undecimpunctata_291 | Tytthaspis_sedecimpunctata_590 | 0.186 |
| Coccinella_undecimpunctata_291 | Tytthaspis_sedecimpunctata_592 | 0.186 |
| Coccinella_undecimpunctata_291 | Tytthaspis_sedecimpunctata_593 | 0.186 |
| Scymnus_auritus_702 | Tytthaspis_sedecimpunctata_593 | 0.186 |
| Coccinella_undecimpunctata_291 | Tytthaspis_sedecimpunctata_594 | 0.186 |
| Coccinella_undecimpunctata_291 | Tytthaspis_sedecimpunctata_595 | 0.186 |
| Calvia_quatuordecimguttata_202 | Scymnus_sinuanodulus_828 | 0.186 |
| Calvia_quatuordecimguttata_210 | Scymnus_sinuanodulus_828 | 0.186 |
| Scymnus_frontalis_736 | Scymnus_sinuanodulus_828 | 0.186 |
| Scymnus_frontalis_737 | Scymnus_sinuanodulus_828 | 0.186 |
| Scymnus_frontalis_738 | Scymnus_sinuanodulus_828 | 0.186 |
| Scymnus_frontalis_739 | Scymnus_sinuanodulus_828 | 0.186 |
| Scymnus_frontalis_740 | Scymnus_sinuanodulus_828 | 0.186 |
| Scymnus_frontalis_741 | Scymnus_sinuanodulus_828 | 0.186 |
| Scymnus_frontalis_743 | Scymnus_sinuanodulus_828 | 0.186 |
| Scymnus_frontalis_744 | Scymnus_sinuanodulus_828 | 0.186 |
| Hyperaspis_binotata_649 | Scymnus_sinuanodulus_830 | 0.186 |
| Exochomus_nigromaculatus_48 | Scymnus_suturalis_832 | 0.186 |
| Brumoides_suturalis_1 | Scymnus_suturalis_833 | 0.186 |
| Hippodamia_parenthesis_439 | Scymnus_suturalis_834 | 0.186 |
| Exochomus_nigromaculatus_48 | Scymnus_suturalis_835 | 0.186 |
| Exochomus_nigromaculatus_48 | Scymnus_suturalis_836 | 0.186 |
| Exochomus_nigromaculatus_49 | Scymnus_suturalis_838 | 0.186 |
| Exochomus_nigromaculatus_48 | Scymnus_suturalis_839 | 0.186 |
| Exochomus_nigromaculatus_49 | Scymnus_suturalis_840 | 0.186 |
| Hyperaspis_proba_660 | Stethorus_punctillum_853 | 0.186 |
| Scymnus_nebulosus_782 | Subcoccinella_vigintiquatuorpunctata_619 | 0.186 |
| Scymnus_nigrinus_787 | Tytthaspis_sedecimpunctata_583 | 0.186 |
| Scymnus_nigrinus_787 | Tytthaspis_sedecimpunctata_585 | 0.186 |

Supplementary Table S4 (continued)

| | | |
|---|---|---|
| Scymnus_nigrinus_787 | Tytthaspis_sedecimpunctata_589 | 0.186 |
| Scymnus_nigrinus_787 | Tytthaspis_sedecimpunctata_592 | 0.186 |
| Chilocorus_bipustulatus_21 | Scymnus_sinuanodulus_828 | 0.186 |
| Scymnus_nigrinus_787 | Tytthaspis_sedecimpunctata_587 | 0.186 |
| Scymnus_frontalis_735 | Scymnus_suturalis_840 | 0.186 |
| Henosepilachna_argus_616 | Scymnus_suturalis_841 | 0.186 |
| Henosepilachna_argus_616 | Scymnus_suturalis_842 | 0.186 |
| Henosepilachna_argus_615 | Scymnus_suturalis_843 | 0.186 |
| Henosepilachna_argus_617 | Scymnus_suturalis_843 | 0.186 |
| Henosepilachna_argus_618 | Scymnus_suturalis_843 | 0.186 |
| Henosepilachna_argus_616 | Scymnus_suturalis_844 | 0.186 |
| Henosepilachna_argus_615 | Scymnus_suturalis_845 | 0.186 |
| Henosepilachna_argus_617 | Scymnus_suturalis_845 | 0.186 |
| Henosepilachna_argus_618 | Scymnus_suturalis_845 | 0.186 |
| Henosepilachna_argus_616 | Scymnus_suturalis_846 | 0.186 |
| Henosepilachna_argus_616 | Scymnus_suturalis_847 | 0.186 |
| Henosepilachna_argus_616 | Scymnus_suturalis_848 | 0.186 |
| Coccidula_scutellata_81 | Subcoccinella_vigintiquatuorpunctata_620 | 0.186 |
| Coccidula_scutellata_82 | Subcoccinella_vigintiquatuorpunctata_620 | 0.186 |
| Rhyzobius_litura_96 | Subcoccinella_vigintiquatuorpunctata_620 | 0.186 |
| Coccidula_scutellata_81 | Subcoccinella_vigintiquatuorpunctata_621 | 0.186 |
| Coccidula_scutellata_82 | Subcoccinella_vigintiquatuorpunctata_621 | 0.186 |
| Rhyzobius_litura_96 | Subcoccinella_vigintiquatuorpunctata_621 | 0.186 |
| Coccidula_scutellata_81 | Subcoccinella_vigintiquatuorpunctata_622 | 0.186 |
| Coccidula_scutellata_82 | Subcoccinella_vigintiquatuorpunctata_622 | 0.186 |
| Rhyzobius_litura_97 | Subcoccinella_vigintiquatuorpunctata_622 | 0.186 |
| Scymnus_haemorrhoidalis_747 | Subcoccinella_vigintiquatuorpunctata_623 | 0.186 |
| Scymnus_haemorrhoidalis_748 | Subcoccinella_vigintiquatuorpunctata_623 | 0.186 |
| Rhyzobius_litura_96 | Subcoccinella_vigintiquatuorpunctata_624 | 0.186 |
| Rhyzobius_litura_96 | Subcoccinella_vigintiquatuorpunctata_626 | 0.186 |
| Coccidula_scutellata_81 | Subcoccinella_vigintiquatuorpunctata_628 | 0.186 |
| Coccidula_scutellata_82 | Subcoccinella_vigintiquatuorpunctata_628 | 0.186 |
| Rhyzobius_litura_96 | Subcoccinella_vigintiquatuorpunctata_628 | 0.186 |
| Coccidula_scutellata_81 | Subcoccinella_vigintiquatuorpunctata_629 | 0.186 |
| Coccidula_scutellata_82 | Subcoccinella_vigintiquatuorpunctata_629 | 0.186 |
| Rhyzobius_litura_96 | Subcoccinella_vigintiquatuorpunctata_629 | 0.186 |
| Coccidula_scutellata_81 | Subcoccinella_vigintiquatuorpunctata_631 | 0.186 |
| Coccidula_scutellata_82 | Subcoccinella_vigintiquatuorpunctata_631 | 0.186 |
| Rhyzobius_litura_96 | Subcoccinella_vigintiquatuorpunctata_631 | 0.186 |
| Coccinella_undecimpunctata_291 | Tytthaspis_sedecimpunctata_591 | 0.186 |
| Hippodamia_undecimnotata_460 | Tytthaspis_sedecimpunctata_593 | 0.186 |
| Hippodamia_undecimnotata_461 | Tytthaspis_sedecimpunctata_593 | 0.186 |
| Hippodamia_undecimnotata_462 | Tytthaspis_sedecimpunctata_593 | 0.186 |
| Hippodamia_undecimnotata_463 | Tytthaspis_sedecimpunctata_593 | 0.186 |
| Hippodamia_undecimnotata_464 | Tytthaspis_sedecimpunctata_593 | 0.186 |
| Hippodamia_undecimnotata_465 | Tytthaspis_sedecimpunctata_593 | 0.186 |
| Hippodamia_undecimnotata_466 | Tytthaspis_sedecimpunctata_593 | 0.186 |
| Calvia_quatuordecimguttata_206 | Scymnus_sinuanodulus_828 | 0.186 |
| Calvia_quatuordecimguttata_216 | Scymnus_sinuanodulus_828 | 0.186 |
| Hyperaspis_binotata_649 | Scymnus_sinuanodulus_828 | 0.186 |
| Calvia_quatuordecimguttata_211 | Scymnus_sinuanodulus_830 | 0.186 |
| Calvia_quatuordecimguttata_212 | Scymnus_sinuanodulus_830 | 0.186 |
| Calvia_quatuordecimguttata_213 | Scymnus_sinuanodulus_830 | 0.186 |
| Calvia_quatuordecimguttata_214 | Scymnus_sinuanodulus_830 | 0.186 |
| Calvia_quatuordecimguttata_215 | Scymnus_sinuanodulus_830 | 0.186 |
| Henosepilachna_argus_616 | Scymnus_suturalis_832 | 0.186 |

| | | |
|---|---|---|
| Scymnus_nubilus_799 | Scymnus_suturalis_833 | 0.186 |
| Exochomus_nigromaculatus_49 | Scymnus_suturalis_834 | 0.186 |
| Henosepilachna_argus_616 | Scymnus_suturalis_835 | 0.186 |
| Henosepilachna_argus_616 | Scymnus_suturalis_836 | 0.186 |
| Exochomus_nigromaculatus_49 | Scymnus_suturalis_837 | 0.186 |
| Henosepilachna_argus_616 | Scymnus_suturalis_839 | 0.186 |
| Scymnus_frontalis_735 | Scymnus_suturalis_841 | 0.186 |
| Scymnus_frontalis_735 | Scymnus_suturalis_842 | 0.186 |
| Scymnus_frontalis_735 | Scymnus_suturalis_844 | 0.186 |
| Scymnus_frontalis_735 | Scymnus_suturalis_846 | 0.186 |
| Scymnus_frontalis_735 | Scymnus_suturalis_847 | 0.186 |
| Scymnus_frontalis_735 | Scymnus_suturalis_848 | 0.186 |
| Chilocorus_nigritus_30 | Scymnus_sinuanodulus_830 | 0.186 |
| Chilocorus_stigma_47 | Scymnus_sinuanodulus_831 | 0.186 |
| Scymnus_frontalis_735 | Scymnus_suturalis_832 | 0.186 |
| Scymnus_frontalis_735 | Scymnus_suturalis_835 | 0.186 |
| Scymnus_frontalis_735 | Scymnus_suturalis_836 | 0.186 |
| Scymnus_frontalis_735 | Scymnus_suturalis_839 | 0.186 |
| Scymnus_haemorrhoidalis_755 | Subcoccinella_vigintiquatuorpunctata_620 | 0.186 |
| Scymnus_haemorrhoidalis_756 | Subcoccinella_vigintiquatuorpunctata_620 | 0.186 |
| Scymnus_haemorrhoidalis_755 | Subcoccinella_vigintiquatuorpunctata_621 | 0.186 |
| Scymnus_haemorrhoidalis_756 | Subcoccinella_vigintiquatuorpunctata_621 | 0.186 |
| Rhyzobius_litura_96 | Subcoccinella_vigintiquatuorpunctata_623 | 0.186 |
| Scymnus_haemorrhoidalis_751 | Subcoccinella_vigintiquatuorpunctata_623 | 0.186 |
| Scymnus_haemorrhoidalis_754 | Subcoccinella_vigintiquatuorpunctata_623 | 0.186 |
| Scymnus_haemorrhoidalis_757 | Subcoccinella_vigintiquatuorpunctata_623 | 0.186 |
| Scymnus_haemorrhoidalis_758 | Subcoccinella_vigintiquatuorpunctata_623 | 0.186 |
| Scymnus_haemorrhoidalis_747 | Subcoccinella_vigintiquatuorpunctata_624 | 0.186 |
| Scymnus_haemorrhoidalis_748 | Subcoccinella_vigintiquatuorpunctata_624 | 0.186 |
| Scymnus_haemorrhoidalis_747 | Subcoccinella_vigintiquatuorpunctata_626 | 0.186 |
| Scymnus_haemorrhoidalis_748 | Subcoccinella_vigintiquatuorpunctata_626 | 0.186 |
| Scymnus_haemorrhoidalis_755 | Subcoccinella_vigintiquatuorpunctata_627 | 0.186 |
| Scymnus_haemorrhoidalis_756 | Subcoccinella_vigintiquatuorpunctata_627 | 0.186 |
| Scymnus_haemorrhoidalis_755 | Subcoccinella_vigintiquatuorpunctata_628 | 0.186 |
| Scymnus_haemorrhoidalis_756 | Subcoccinella_vigintiquatuorpunctata_628 | 0.186 |
| Scymnus_haemorrhoidalis_755 | Subcoccinella_vigintiquatuorpunctata_629 | 0.186 |
| Scymnus_haemorrhoidalis_756 | Subcoccinella_vigintiquatuorpunctata_629 | 0.186 |
| Scymnus_haemorrhoidalis_755 | Subcoccinella_vigintiquatuorpunctata_631 | 0.186 |
| Scymnus_haemorrhoidalis_756 | Subcoccinella_vigintiquatuorpunctata_631 | 0.186 |
| Coccinella_transversoguttata_267 | Vibidia_duodecimguttata_596 | 0.186 |
| Coccinella_transversoguttata_268 | Vibidia_duodecimguttata_596 | 0.186 |
| Coccinella_transversoguttata_269 | Vibidia_duodecimguttata_596 | 0.186 |
| Coccinella_transversoguttata_270 | Vibidia_duodecimguttata_596 | 0.186 |
| Coccinella_transversoguttata_271 | Vibidia_duodecimguttata_596 | 0.186 |
| Aphidecta_obliterata_185 | Vibidia_duodecimguttata_597 | 0.186 |
| Aphidecta_obliterata_186 | Vibidia_duodecimguttata_597 | 0.186 |
| Aphidecta_obliterata_185 | Vibidia_duodecimguttata_600 | 0.186 |
| Aphidecta_obliterata_186 | Vibidia_duodecimguttata_600 | 0.186 |
| Calvia_quatuordecimguttata_207 | Scymnus_sinuanodulus_830 | 0.186 |
| Psyllobora_vigintimaculata_573 | Vibidia_duodecimguttata_603 | 0.186 |
| Hippodamia_parenthesis_436 | Scymnus_suturalis_843 | 0.186 |
| Coccinella_undecimpunctata_289 | Tytthaspis_sedecimpunctata_587 | 0.186 |
| Coccinella_undecimpunctata_290 | Tytthaspis_sedecimpunctata_587 | 0.186 |
| Coccinella_undecimpunctata_282 | Scymnus_sinuanodulus_831 | 0.186 |
| Coccinella_undecimpunctata_283 | Scymnus_sinuanodulus_831 | 0.186 |
| Coccinella_undecimpunctata_284 | Scymnus_sinuanodulus_831 | 0.186 |

| | | |
|---|---|---|
| Scymnus_nubilus_796 | Scymnus_suturalis_843 | 0.186 |
| Scymnus_nubilus_798 | Scymnus_suturalis_843 | 0.186 |
| Scymnus_nubilus_800 | Scymnus_suturalis_843 | 0.186 |
| Scymnus_nubilus_801 | Scymnus_suturalis_843 | 0.186 |
| Scymnus_nubilus_802 | Scymnus_suturalis_843 | 0.186 |
| Scymnus_nubilus_805 | Scymnus_suturalis_843 | 0.186 |
| Scymnus_nubilus_806 | Scymnus_suturalis_843 | 0.186 |
| Scymnus_nubilus_807 | Scymnus_suturalis_843 | 0.186 |
| Scymnus_nubilus_810 | Scymnus_suturalis_843 | 0.186 |
| Scymnus_nubilus_796 | Scymnus_suturalis_845 | 0.186 |
| Scymnus_nubilus_798 | Scymnus_suturalis_845 | 0.186 |
| Scymnus_nubilus_800 | Scymnus_suturalis_845 | 0.186 |
| Scymnus_nubilus_801 | Scymnus_suturalis_845 | 0.186 |
| Scymnus_nubilus_802 | Scymnus_suturalis_845 | 0.186 |
| Scymnus_nubilus_805 | Scymnus_suturalis_845 | 0.186 |
| Scymnus_nubilus_806 | Scymnus_suturalis_845 | 0.186 |
| Scymnus_nubilus_807 | Scymnus_suturalis_845 | 0.186 |
| Scymnus_nubilus_810 | Scymnus_suturalis_845 | 0.186 |
| Scymnus_haemorrhoidalis_752 | Subcoccinella_vigintiquatuorpunctata_623 | 0.186 |
| Scymnus_haemorrhoidalis_753 | Subcoccinella_vigintiquatuorpunctata_623 | 0.186 |
| Scymnus_haemorrhoidalis_749 | Subcoccinella_vigintiquatuorpunctata_624 | 0.186 |
| Scymnus_haemorrhoidalis_750 | Subcoccinella_vigintiquatuorpunctata_624 | 0.186 |
| Scymnus_haemorrhoidalis_751 | Subcoccinella_vigintiquatuorpunctata_626 | 0.186 |
| Scymnus_haemorrhoidalis_754 | Subcoccinella_vigintiquatuorpunctata_626 | 0.186 |
| Scymnus_haemorrhoidalis_757 | Subcoccinella_vigintiquatuorpunctata_626 | 0.186 |
| Scymnus_haemorrhoidalis_758 | Subcoccinella_vigintiquatuorpunctata_626 | 0.186 |
| Scymnus_haemorrhoidalis_751 | Subcoccinella_vigintiquatuorpunctata_627 | 0.186 |
| Scymnus_haemorrhoidalis_754 | Subcoccinella_vigintiquatuorpunctata_627 | 0.186 |
| Scymnus_haemorrhoidalis_757 | Subcoccinella_vigintiquatuorpunctata_627 | 0.186 |
| Scymnus_haemorrhoidalis_758 | Subcoccinella_vigintiquatuorpunctata_627 | 0.186 |
| Coccinella_transversoguttata_265 | Vibidia_duodecimguttata_599 | 0.186 |
| Coccinella_transversoguttata_266 | Vibidia_duodecimguttata_599 | 0.186 |
| Coccinella_transversoguttata_265 | Vibidia_duodecimguttata_601 | 0.186 |
| Coccinella_transversoguttata_266 | Vibidia_duodecimguttata_601 | 0.186 |
| Coccinella_transversoguttata_265 | Vibidia_duodecimguttata_602 | 0.186 |
| Coccinella_transversoguttata_266 | Vibidia_duodecimguttata_602 | 0.186 |
| Scymnus_nubilus_796 | Scymnus_suturalis_834 | 0.186 |
| Scymnus_nubilus_798 | Scymnus_suturalis_834 | 0.186 |
| Scymnus_nubilus_800 | Scymnus_suturalis_834 | 0.186 |
| Scymnus_nubilus_801 | Scymnus_suturalis_834 | 0.186 |
| Scymnus_nubilus_802 | Scymnus_suturalis_834 | 0.186 |
| Scymnus_nubilus_805 | Scymnus_suturalis_834 | 0.186 |
| Scymnus_nubilus_806 | Scymnus_suturalis_834 | 0.186 |
| Scymnus_nubilus_807 | Scymnus_suturalis_834 | 0.186 |
| Scymnus_nubilus_810 | Scymnus_suturalis_834 | 0.186 |
| Scymnus_nubilus_796 | Scymnus_suturalis_837 | 0.186 |
| Scymnus_nubilus_798 | Scymnus_suturalis_837 | 0.186 |
| Scymnus_nubilus_800 | Scymnus_suturalis_837 | 0.186 |
| Scymnus_nubilus_801 | Scymnus_suturalis_837 | 0.186 |
| Scymnus_nubilus_802 | Scymnus_suturalis_837 | 0.186 |
| Scymnus_nubilus_805 | Scymnus_suturalis_837 | 0.186 |
| Scymnus_nubilus_806 | Scymnus_suturalis_837 | 0.186 |
| Scymnus_nubilus_807 | Scymnus_suturalis_837 | 0.186 |
| Scymnus_nubilus_810 | Scymnus_suturalis_837 | 0.186 |
| Coccinella_undecimpunctata_289 | Tytthaspis_sedecimpunctata_577 | 0.186 |
| Coccinella_undecimpunctata_290 | Tytthaspis_sedecimpunctata_577 | 0.186 |

| | | |
|---|---|---|
| Coccinella_undecimpunctata_289 | Tytthaspis_sedecimpunctata_578 | 0.186 |
| Coccinella_undecimpunctata_290 | Tytthaspis_sedecimpunctata_578 | 0.186 |
| Coccinella_undecimpunctata_289 | Tytthaspis_sedecimpunctata_579 | 0.186 |
| Coccinella_undecimpunctata_290 | Tytthaspis_sedecimpunctata_579 | 0.186 |
| Coccinella_undecimpunctata_289 | Tytthaspis_sedecimpunctata_581 | 0.186 |
| Coccinella_undecimpunctata_290 | Tytthaspis_sedecimpunctata_581 | 0.186 |
| Coccinella_undecimpunctata_289 | Tytthaspis_sedecimpunctata_582 | 0.186 |
| Coccinella_undecimpunctata_290 | Tytthaspis_sedecimpunctata_582 | 0.186 |
| Coccinella_undecimpunctata_289 | Tytthaspis_sedecimpunctata_585 | 0.186 |
| Coccinella_undecimpunctata_290 | Tytthaspis_sedecimpunctata_585 | 0.186 |
| Coccinella_undecimpunctata_289 | Tytthaspis_sedecimpunctata_590 | 0.186 |
| Coccinella_undecimpunctata_290 | Tytthaspis_sedecimpunctata_590 | 0.186 |
| Coccinella_undecimpunctata_289 | Tytthaspis_sedecimpunctata_592 | 0.186 |
| Coccinella_undecimpunctata_290 | Tytthaspis_sedecimpunctata_592 | 0.186 |
| Coccinella_undecimpunctata_289 | Tytthaspis_sedecimpunctata_593 | 0.186 |
| Coccinella_undecimpunctata_290 | Tytthaspis_sedecimpunctata_593 | 0.186 |
| Coccinella_undecimpunctata_289 | Tytthaspis_sedecimpunctata_594 | 0.186 |
| Coccinella_undecimpunctata_290 | Tytthaspis_sedecimpunctata_594 | 0.186 |
| Coccinella_undecimpunctata_289 | Tytthaspis_sedecimpunctata_595 | 0.186 |
| Coccinella_undecimpunctata_290 | Tytthaspis_sedecimpunctata_595 | 0.186 |
| Brumus_quadripustulatus_3 | Scymnus_sinuanodulus_829 | 0.186 |
| Scymnus_nubilus_797 | Scymnus_suturalis_841 | 0.186 |
| Scymnus_nubilus_797 | Scymnus_suturalis_842 | 0.186 |
| Scymnus_nubilus_797 | Scymnus_suturalis_844 | 0.186 |
| Scymnus_nubilus_797 | Scymnus_suturalis_846 | 0.186 |
| Scymnus_nubilus_797 | Scymnus_suturalis_847 | 0.186 |
| Scymnus_nubilus_797 | Scymnus_suturalis_848 | 0.186 |
| Hyperaspis_campestris_651 | Subcoccinella_vigintiquatuorpunctata_625 | 0.186 |
| Hyperaspis_campestris_652 | Subcoccinella_vigintiquatuorpunctata_625 | 0.186 |
| Hyperaspis_campestris_653 | Subcoccinella_vigintiquatuorpunctata_625 | 0.186 |
| Scymnus_haemorrhoidalis_752 | Subcoccinella_vigintiquatuorpunctata_626 | 0.186 |
| Scymnus_haemorrhoidalis_753 | Subcoccinella_vigintiquatuorpunctata_626 | 0.186 |
| Scymnus_haemorrhoidalis_752 | Subcoccinella_vigintiquatuorpunctata_627 | 0.186 |
| Scymnus_haemorrhoidalis_753 | Subcoccinella_vigintiquatuorpunctata_627 | 0.186 |
| Aphidecta_obliterata_176 | Vibidia_duodecimguttata_596 | 0.186 |
| Anisosticta_novemdecimpunctata_174 | Vibidia_duodecimguttata_599 | 0.186 |
| Anisosticta_novemdecimpunctata_174 | Vibidia_duodecimguttata_601 | 0.186 |
| Anisosticta_novemdecimpunctata_174 | Vibidia_duodecimguttata_602 | 0.186 |
| Aphidecta_obliterata_176 | Vibidia_duodecimguttata_603 | 0.186 |
| Scymnus_nubilus_797 | Scymnus_suturalis_832 | 0.186 |
| Scymnus_nubilus_797 | Scymnus_suturalis_835 | 0.186 |
| Scymnus_nubilus_797 | Scymnus_suturalis_836 | 0.186 |
| Scymnus_nubilus_797 | Scymnus_suturalis_839 | 0.186 |
| Scymnus_nubilus_796 | Scymnus_suturalis_840 | 0.186 |
| Scymnus_nubilus_800 | Scymnus_suturalis_840 | 0.186 |
| Scymnus_nubilus_801 | Scymnus_suturalis_840 | 0.186 |
| Scymnus_nubilus_802 | Scymnus_suturalis_840 | 0.186 |
| Scymnus_nubilus_805 | Scymnus_suturalis_840 | 0.186 |
| Scymnus_nubilus_806 | Scymnus_suturalis_840 | 0.186 |
| Scymnus_nubilus_807 | Scymnus_suturalis_840 | 0.186 |
| Scymnus_nubilus_810 | Scymnus_suturalis_840 | 0.186 |
| Coccidula_scutellata_80 | Subcoccinella_vigintiquatuorpunctata_619 | 0.186 |
| Rhyzobius_litura_96 | Subcoccinella_vigintiquatuorpunctata_619 | 0.186 |
| Coccinella_undecimpunctata_289 | Tytthaspis_sedecimpunctata_591 | 0.186 |
| Coccinella_undecimpunctata_290 | Tytthaspis_sedecimpunctata_591 | 0.186 |
| Calvia_quatuordecimguttata_201 | Scymnus_sinuanodulus_831 | 0.186 |

| | | |
|---|---|---|
| Scymnus_frontalis_746 | Scymnus_suturalis_843 | 0.186 |
| Scymnus_frontalis_746 | Scymnus_suturalis_845 | 0.186 |
| Scymnus_frontalis_746 | Scymnus_suturalis_837 | 0.186 |
| Myzia_pullata_518 | Vibidia_duodecimguttata_597 | 0.186 |
| Myzia_pullata_518 | Vibidia_duodecimguttata_600 | 0.186 |
| Hyperaspis_oregona_659 | Scymnus_sinuanodulus_828 | 0.186 |
| Myzia_pullata_518 | Vibidia_duodecimguttata_598 | 0.187 |
| Myzia_pullata_518 | Vibidia_duodecimguttata_599 | 0.187 |
| Myzia_pullata_518 | Vibidia_duodecimguttata_601 | 0.187 |
| Myzia_pullata_518 | Vibidia_duodecimguttata_602 | 0.187 |
| Myzia_pullata_518 | Vibidia_duodecimguttata_603 | 0.187 |
| Scymnus_haemorrhoidalis_751 | Scymnus_sinuanodulus_830 | 0.187 |
| Scymnus_haemorrhoidalis_754 | Scymnus_sinuanodulus_830 | 0.187 |
| Scymnus_haemorrhoidalis_757 | Scymnus_sinuanodulus_830 | 0.187 |
| Scymnus_haemorrhoidalis_758 | Scymnus_sinuanodulus_830 | 0.187 |
| Scymnus_apetzi_694 | Subcoccinella_vigintiquatuorpunctata_630 | 0.187 |
| Coccinella_trifasciata_273 | Vibidia_duodecimguttata_596 | 0.187 |
| Psyllobora_vigintiduopunctata_571 | Vibidia_duodecimguttata_596 | 0.187 |
| Psyllobora_vigintiduopunctata_571 | Vibidia_duodecimguttata_599 | 0.187 |
| Psyllobora_vigintiduopunctata_571 | Vibidia_duodecimguttata_601 | 0.187 |
| Psyllobora_vigintiduopunctata_571 | Vibidia_duodecimguttata_602 | 0.187 |
| Scymnus_haemorrhoidalis_751 | Scymnus_sinuanodulus_828 | 0.187 |
| Scymnus_haemorrhoidalis_754 | Scymnus_sinuanodulus_828 | 0.187 |
| Scymnus_haemorrhoidalis_757 | Scymnus_sinuanodulus_828 | 0.187 |
| Scymnus_haemorrhoidalis_758 | Scymnus_sinuanodulus_828 | 0.187 |
| Chilocorus_stigma_47 | Scymnus_sinuanodulus_829 | 0.187 |
| Coccinella_undecimpunctata_285 | Scymnus_sinuanodulus_829 | 0.187 |
| Coccinella_undecimpunctata_286 | Scymnus_sinuanodulus_829 | 0.187 |
| Coccinella_undecimpunctata_288 | Scymnus_sinuanodulus_829 | 0.187 |
| Coccinella_undecimpunctata_287 | Scymnus_sinuanodulus_830 | 0.187 |
| Scymnus_apetzi_695 | Subcoccinella_vigintiquatuorpunctata_627 | 0.187 |
| Coccinella_undecimpunctata_287 | Scymnus_sinuanodulus_828 | 0.187 |
| Calvia_quatuordecimguttata_201 | Scymnus_sinuanodulus_829 | 0.187 |
| Coccinella_quinquepunctata_237 | Vibidia_duodecimguttata_597 | 0.187 |
| Coelophora_inaequalis_312 | Vibidia_duodecimguttata_597 | 0.187 |
| Coelophora_inaequalis_314 | Vibidia_duodecimguttata_597 | 0.187 |
| Coelophora_inaequalis_317 | Vibidia_duodecimguttata_597 | 0.187 |
| Coelophora_inaequalis_325 | Vibidia_duodecimguttata_597 | 0.187 |
| Myzia_oblongoguttata_515 | Vibidia_duodecimguttata_597 | 0.187 |
| Coccinella_quinquepunctata_237 | Vibidia_duodecimguttata_600 | 0.187 |
| Coelophora_inaequalis_312 | Vibidia_duodecimguttata_600 | 0.187 |
| Coelophora_inaequalis_314 | Vibidia_duodecimguttata_600 | 0.187 |
| Coelophora_inaequalis_317 | Vibidia_duodecimguttata_600 | 0.187 |
| Coelophora_inaequalis_325 | Vibidia_duodecimguttata_600 | 0.187 |
| Myzia_oblongoguttata_515 | Vibidia_duodecimguttata_600 | 0.187 |
| Scymnus_apetzi_693 | Subcoccinella_vigintiquatuorpunctata_620 | 0.187 |
| Scymnus_apetzi_693 | Subcoccinella_vigintiquatuorpunctata_621 | 0.187 |
| Scymnus_apetzi_693 | Subcoccinella_vigintiquatuorpunctata_628 | 0.187 |
| Scymnus_apetzi_693 | Subcoccinella_vigintiquatuorpunctata_629 | 0.187 |
| Scymnus_apetzi_693 | Subcoccinella_vigintiquatuorpunctata_631 | 0.187 |
| Coccinella_quinquepunctata_235 | Vibidia_duodecimguttata_596 | 0.187 |
| Coccinella_quinquepunctata_238 | Vibidia_duodecimguttata_596 | 0.187 |
| Coccinella_quinquepunctata_239 | Vibidia_duodecimguttata_596 | 0.187 |
| Coccinella_quinquepunctata_240 | Vibidia_duodecimguttata_596 | 0.187 |
| Coccinella_undecimpunctata_282 | Vibidia_duodecimguttata_596 | 0.187 |
| Coccinella_undecimpunctata_283 | Vibidia_duodecimguttata_596 | 0.187 |

| | | |
|---|---|---|
| Coccinella_undecimpunctata_284 | Vibidia_duodecimguttata_596 | 0.187 |
| Coelophora_inaequalis_320 | Vibidia_duodecimguttata_597 | 0.187 |
| Coelophora_inaequalis_321 | Vibidia_duodecimguttata_597 | 0.187 |
| Coelophora_inaequalis_322 | Vibidia_duodecimguttata_597 | 0.187 |
| Coccinella_quinquepunctata_237 | Vibidia_duodecimguttata_598 | 0.187 |
| Myzia_oblongoguttata_506 | Vibidia_duodecimguttata_599 | 0.187 |
| Coelophora_inaequalis_320 | Vibidia_duodecimguttata_600 | 0.187 |
| Coelophora_inaequalis_321 | Vibidia_duodecimguttata_600 | 0.187 |
| Coelophora_inaequalis_322 | Vibidia_duodecimguttata_600 | 0.187 |
| Psyllobora_renifer_550 | Vibidia_duodecimguttata_601 | 0.187 |
| Myzia_oblongoguttata_506 | Vibidia_duodecimguttata_602 | 0.187 |
| Coccinella_hieroglyphica_226 | Vibidia_duodecimguttata_603 | 0.187 |
| Coccinella_quinquepunctata_236 | Vibidia_duodecimguttata_603 | 0.187 |
| Coccinella_undecimpunctata_282 | Vibidia_duodecimguttata_603 | 0.187 |
| Coccinella_undecimpunctata_283 | Vibidia_duodecimguttata_603 | 0.187 |
| Coccinella_undecimpunctata_284 | Vibidia_duodecimguttata_603 | 0.187 |
| Myzia_oblongoguttata_506 | Vibidia_duodecimguttata_603 | 0.187 |
| Psyllobora_vigintiduopunctata_566 | Vibidia_duodecimguttata_597 | 0.187 |
| Psyllobora_vigintiduopunctata_566 | Vibidia_duodecimguttata_600 | 0.187 |
| Brumus_quadripustulatus_5 | Scymnus_sinuanodulus_830 | 0.187 |
| Hyperaspis_campestris_651 | Tytthaspis_sedecimpunctata_592 | 0.187 |
| Hyperaspis_campestris_652 | Tytthaspis_sedecimpunctata_592 | 0.187 |
| Hyperaspis_campestris_653 | Tytthaspis_sedecimpunctata_592 | 0.187 |
| Hyperaspis_oregona_658 | Tytthaspis_sedecimpunctata_577 | 0.187 |
| Hyperaspis_oregona_658 | Tytthaspis_sedecimpunctata_578 | 0.187 |
| Hyperaspis_oregona_658 | Tytthaspis_sedecimpunctata_579 | 0.187 |
| Hyperaspis_campestris_651 | Tytthaspis_sedecimpunctata_580 | 0.187 |
| Hyperaspis_campestris_652 | Tytthaspis_sedecimpunctata_580 | 0.187 |
| Hyperaspis_campestris_653 | Tytthaspis_sedecimpunctata_580 | 0.187 |
| Hyperaspis_oregona_658 | Tytthaspis_sedecimpunctata_581 | 0.187 |
| Hyperaspis_oregona_658 | Tytthaspis_sedecimpunctata_582 | 0.187 |
| Platynaspis_luteorubra_52 | Tytthaspis_sedecimpunctata_583 | 0.187 |
| Platynaspis_luteorubra_56 | Tytthaspis_sedecimpunctata_583 | 0.187 |
| Platynaspis_luteorubra_60 | Tytthaspis_sedecimpunctata_583 | 0.187 |
| Hyperaspis_oregona_659 | Tytthaspis_sedecimpunctata_585 | 0.187 |
| Platynaspis_luteorubra_52 | Tytthaspis_sedecimpunctata_585 | 0.187 |
| Platynaspis_luteorubra_56 | Tytthaspis_sedecimpunctata_585 | 0.187 |
| Platynaspis_luteorubra_60 | Tytthaspis_sedecimpunctata_585 | 0.187 |
| Hyperaspis_oregona_658 | Tytthaspis_sedecimpunctata_590 | 0.187 |
| Hyperaspis_oregona_658 | Tytthaspis_sedecimpunctata_593 | 0.187 |
| Hyperaspis_oregona_658 | Tytthaspis_sedecimpunctata_594 | 0.187 |
| Hyperaspis_oregona_658 | Tytthaspis_sedecimpunctata_595 | 0.187 |
| Psyllobora_renifer_550 | Vibidia_duodecimguttata_597 | 0.187 |
| Psyllobora_renifer_550 | Vibidia_duodecimguttata_600 | 0.187 |
| Platynaspis_luteorubra_61 | Tytthaspis_sedecimpunctata_577 | 0.187 |
| Platynaspis_luteorubra_61 | Tytthaspis_sedecimpunctata_578 | 0.187 |
| Platynaspis_luteorubra_61 | Tytthaspis_sedecimpunctata_579 | 0.187 |
| Platynaspis_luteorubra_61 | Tytthaspis_sedecimpunctata_581 | 0.187 |
| Platynaspis_luteorubra_61 | Tytthaspis_sedecimpunctata_582 | 0.187 |
| Platynaspis_luteorubra_54 | Tytthaspis_sedecimpunctata_583 | 0.187 |
| Platynaspis_luteorubra_57 | Tytthaspis_sedecimpunctata_583 | 0.187 |
| Platynaspis_luteorubra_59 | Tytthaspis_sedecimpunctata_583 | 0.187 |
| Platynaspis_luteorubra_54 | Tytthaspis_sedecimpunctata_585 | 0.187 |
| Platynaspis_luteorubra_57 | Tytthaspis_sedecimpunctata_585 | 0.187 |
| Platynaspis_luteorubra_59 | Tytthaspis_sedecimpunctata_585 | 0.187 |
| Chilocorus_renipustulatus_34 | Tytthaspis_sedecimpunctata_586 | 0.187 |

| | | |
|---|---|---|
| Platynaspis_luteorubra_52 | Tytthaspis_sedecimpunctata_586 | 0.187 |
| Platynaspis_luteorubra_56 | Tytthaspis_sedecimpunctata_586 | 0.187 |
| Platynaspis_luteorubra_60 | Tytthaspis_sedecimpunctata_586 | 0.187 |
| Hyperaspis_oregona_658 | Tytthaspis_sedecimpunctata_587 | 0.187 |
| Chilocorus_renipustulatus_34 | Tytthaspis_sedecimpunctata_588 | 0.187 |
| Platynaspis_luteorubra_61 | Tytthaspis_sedecimpunctata_589 | 0.187 |
| Platynaspis_luteorubra_61 | Tytthaspis_sedecimpunctata_590 | 0.187 |
| Platynaspis_luteorubra_61 | Tytthaspis_sedecimpunctata_592 | 0.187 |
| Platynaspis_luteorubra_61 | Tytthaspis_sedecimpunctata_594 | 0.187 |
| Platynaspis_luteorubra_61 | Tytthaspis_sedecimpunctata_595 | 0.187 |
| Brumus_quadripustulatus_5 | Scymnus_sinuanodulus_828 | 0.187 |
| Chilocorus_renipustulatus_34 | Tytthaspis_sedecimpunctata_577 | 0.187 |
| Chilocorus_renipustulatus_34 | Tytthaspis_sedecimpunctata_578 | 0.187 |
| Chilocorus_renipustulatus_34 | Tytthaspis_sedecimpunctata_579 | 0.187 |
| Chilocorus_renipustulatus_34 | Tytthaspis_sedecimpunctata_581 | 0.187 |
| Chilocorus_renipustulatus_34 | Tytthaspis_sedecimpunctata_582 | 0.187 |
| Chilocorus_renipustulatus_34 | Tytthaspis_sedecimpunctata_585 | 0.187 |
| Chilocorus_renipustulatus_31 | Tytthaspis_sedecimpunctata_586 | 0.187 |
| Chilocorus_renipustulatus_32 | Tytthaspis_sedecimpunctata_586 | 0.187 |
| Chilocorus_renipustulatus_35 | Tytthaspis_sedecimpunctata_586 | 0.187 |
| Chilocorus_renipustulatus_36 | Tytthaspis_sedecimpunctata_586 | 0.187 |
| Chilocorus_renipustulatus_37 | Tytthaspis_sedecimpunctata_586 | 0.187 |
| Chilocorus_renipustulatus_38 | Tytthaspis_sedecimpunctata_586 | 0.187 |
| Chilocorus_renipustulatus_40 | Tytthaspis_sedecimpunctata_586 | 0.187 |
| Chilocorus_renipustulatus_41 | Tytthaspis_sedecimpunctata_586 | 0.187 |
| Chilocorus_renipustulatus_42 | Tytthaspis_sedecimpunctata_586 | 0.187 |
| Platynaspis_luteorubra_54 | Tytthaspis_sedecimpunctata_586 | 0.187 |
| Platynaspis_luteorubra_57 | Tytthaspis_sedecimpunctata_586 | 0.187 |
| Platynaspis_luteorubra_59 | Tytthaspis_sedecimpunctata_586 | 0.187 |
| Platynaspis_luteorubra_61 | Tytthaspis_sedecimpunctata_587 | 0.187 |
| Chilocorus_renipustulatus_31 | Tytthaspis_sedecimpunctata_588 | 0.187 |
| Chilocorus_renipustulatus_32 | Tytthaspis_sedecimpunctata_588 | 0.187 |
| Chilocorus_renipustulatus_35 | Tytthaspis_sedecimpunctata_588 | 0.187 |
| Chilocorus_renipustulatus_36 | Tytthaspis_sedecimpunctata_588 | 0.187 |
| Chilocorus_renipustulatus_37 | Tytthaspis_sedecimpunctata_588 | 0.187 |
| Chilocorus_renipustulatus_38 | Tytthaspis_sedecimpunctata_588 | 0.187 |
| Chilocorus_renipustulatus_40 | Tytthaspis_sedecimpunctata_588 | 0.187 |
| Chilocorus_renipustulatus_41 | Tytthaspis_sedecimpunctata_588 | 0.187 |
| Chilocorus_renipustulatus_42 | Tytthaspis_sedecimpunctata_588 | 0.187 |
| Scymnus_mitior_779 | Tytthaspis_sedecimpunctata_588 | 0.187 |
| Chilocorus_renipustulatus_34 | Tytthaspis_sedecimpunctata_590 | 0.187 |
| Hyperaspis_reppensis_667 | Tytthaspis_sedecimpunctata_591 | 0.187 |
| Chilocorus_renipustulatus_34 | Tytthaspis_sedecimpunctata_592 | 0.187 |
| Chilocorus_renipustulatus_34 | Tytthaspis_sedecimpunctata_593 | 0.187 |
| Chilocorus_renipustulatus_34 | Tytthaspis_sedecimpunctata_594 | 0.187 |
| Chilocorus_renipustulatus_34 | Tytthaspis_sedecimpunctata_595 | 0.187 |
| Psyllobora_vigintiduopunctata_566 | Vibidia_duodecimguttata_598 | 0.187 |
| Hyperaspis_reppensis_666 | Tytthaspis_sedecimpunctata_577 | 0.187 |
| Scymnus_mitior_780 | Tytthaspis_sedecimpunctata_577 | 0.187 |
| Hyperaspis_reppensis_666 | Tytthaspis_sedecimpunctata_578 | 0.187 |
| Scymnus_mitior_780 | Tytthaspis_sedecimpunctata_578 | 0.187 |
| Hyperaspis_reppensis_666 | Tytthaspis_sedecimpunctata_579 | 0.187 |
| Scymnus_mitior_780 | Tytthaspis_sedecimpunctata_579 | 0.187 |
| Hyperaspis_reppensis_666 | Tytthaspis_sedecimpunctata_581 | 0.187 |
| Scymnus_mitior_780 | Tytthaspis_sedecimpunctata_581 | 0.187 |
| Hyperaspis_reppensis_666 | Tytthaspis_sedecimpunctata_582 | 0.187 |

| | | |
|---|---|---|
| Scymnus_mitior_780 | Tytthaspis_sedecimpunctata_582 | 0.187 |
| Hyperaspis_reppensis_664 | Tytthaspis_sedecimpunctata_583 | 0.187 |
| Scymnus_mitior_779 | Tytthaspis_sedecimpunctata_583 | 0.187 |
| Scymnus_mitior_780 | Tytthaspis_sedecimpunctata_585 | 0.187 |
| Hyperaspis_reppensis_665 | Tytthaspis_sedecimpunctata_589 | 0.187 |
| Hyperaspis_reppensis_666 | Tytthaspis_sedecimpunctata_590 | 0.187 |
| Scymnus_mitior_780 | Tytthaspis_sedecimpunctata_590 | 0.187 |
| Hyperaspis_reppensis_665 | Tytthaspis_sedecimpunctata_592 | 0.187 |
| Scymnus_mitior_780 | Tytthaspis_sedecimpunctata_592 | 0.187 |
| Hyperaspis_reppensis_666 | Tytthaspis_sedecimpunctata_593 | 0.187 |
| Scymnus_mitior_780 | Tytthaspis_sedecimpunctata_593 | 0.187 |
| Hyperaspis_reppensis_666 | Tytthaspis_sedecimpunctata_594 | 0.187 |
| Scymnus_mitior_780 | Tytthaspis_sedecimpunctata_594 | 0.187 |
| Hyperaspis_reppensis_666 | Tytthaspis_sedecimpunctata_595 | 0.187 |
| Scymnus_mitior_780 | Tytthaspis_sedecimpunctata_595 | 0.187 |
| Platynaspis_luteorubra_50 | Scymnus_rubromaculatus_822 | 0.187 |
| Platynaspis_luteorubra_50 | Scymnus_rubromaculatus_823 | 0.187 |
| Platynaspis_luteorubra_50 | Scymnus_rubromaculatus_824 | 0.187 |
| Platynaspis_luteorubra_50 | Scymnus_rubromaculatus_825 | 0.187 |
| Platynaspis_luteorubra_50 | Scymnus_rubromaculatus_826 | 0.187 |
| Platynaspis_luteorubra_50 | Scymnus_rubromaculatus_827 | 0.187 |
| Scymnus_loewii_774 | Tytthaspis_sedecimpunctata_577 | 0.187 |
| Scymnus_loewii_774 | Tytthaspis_sedecimpunctata_578 | 0.187 |
| Scymnus_loewii_774 | Tytthaspis_sedecimpunctata_579 | 0.187 |
| Hyperaspis_reppensis_665 | Tytthaspis_sedecimpunctata_580 | 0.187 |
| Scymnus_mitior_780 | Tytthaspis_sedecimpunctata_580 | 0.187 |
| Scymnus_loewii_774 | Tytthaspis_sedecimpunctata_581 | 0.187 |
| Scymnus_loewii_774 | Tytthaspis_sedecimpunctata_582 | 0.187 |
| Chilocorus_renipustulatus_39 | Tytthaspis_sedecimpunctata_584 | 0.187 |
| Coccinella_transversalis_257 | Tytthaspis_sedecimpunctata_584 | 0.187 |
| Coccinella_transversalis_259 | Tytthaspis_sedecimpunctata_584 | 0.187 |
| Coccinella_transversalis_260 | Tytthaspis_sedecimpunctata_584 | 0.187 |
| Coccinella_transversalis_261 | Tytthaspis_sedecimpunctata_584 | 0.187 |
| Coccinella_transversalis_262 | Tytthaspis_sedecimpunctata_584 | 0.187 |
| Coccinella_transversalis_263 | Tytthaspis_sedecimpunctata_584 | 0.187 |
| Coccinella_transversalis_264 | Tytthaspis_sedecimpunctata_584 | 0.187 |
| Hyperaspis_reppensis_666 | Tytthaspis_sedecimpunctata_584 | 0.187 |
| Myzia_oblongoguttata_504 | Tytthaspis_sedecimpunctata_584 | 0.187 |
| Myzia_oblongoguttata_505 | Tytthaspis_sedecimpunctata_584 | 0.187 |
| Myzia_oblongoguttata_507 | Tytthaspis_sedecimpunctata_584 | 0.187 |
| Myzia_oblongoguttata_508 | Tytthaspis_sedecimpunctata_584 | 0.187 |
| Myzia_oblongoguttata_509 | Tytthaspis_sedecimpunctata_584 | 0.187 |
| Myzia_oblongoguttata_510 | Tytthaspis_sedecimpunctata_584 | 0.187 |
| Myzia_oblongoguttata_511 | Tytthaspis_sedecimpunctata_584 | 0.187 |
| Myzia_oblongoguttata_512 | Tytthaspis_sedecimpunctata_584 | 0.187 |
| Myzia_oblongoguttata_513 | Tytthaspis_sedecimpunctata_584 | 0.187 |
| Myzia_oblongoguttata_514 | Tytthaspis_sedecimpunctata_584 | 0.187 |
| Myzia_oblongoguttata_516 | Tytthaspis_sedecimpunctata_584 | 0.187 |
| Scymnus_mitior_779 | Tytthaspis_sedecimpunctata_584 | 0.187 |
| Scymnus_loewii_774 | Tytthaspis_sedecimpunctata_585 | 0.187 |
| Scymnus_loewii_774 | Tytthaspis_sedecimpunctata_589 | 0.187 |
| Scymnus_loewii_774 | Tytthaspis_sedecimpunctata_590 | 0.187 |
| Scymnus_loewii_771 | Tytthaspis_sedecimpunctata_591 | 0.187 |
| Scymnus_mitior_779 | Tytthaspis_sedecimpunctata_591 | 0.187 |
| Scymnus_loewii_774 | Tytthaspis_sedecimpunctata_592 | 0.187 |
| Coccinella_transversalis_258 | Tytthaspis_sedecimpunctata_593 | 0.187 |

| | | |
|---|---|---|
| Scymnus_loewii_774 | Tytthaspis_sedecimpunctata_593 | 0.187 |
| Scymnus_loewii_774 | Tytthaspis_sedecimpunctata_594 | 0.187 |
| Scymnus_loewii_774 | Tytthaspis_sedecimpunctata_595 | 0.187 |
| Coccidula_scutellata_80 | Scymnus_rubromaculatus_822 | 0.187 |
| Coccidula_scutellata_81 | Scymnus_rubromaculatus_822 | 0.187 |
| Coccidula_scutellata_82 | Scymnus_rubromaculatus_822 | 0.187 |
| Coccidula_scutellata_80 | Scymnus_rubromaculatus_823 | 0.187 |
| Coccidula_scutellata_81 | Scymnus_rubromaculatus_823 | 0.187 |
| Coccidula_scutellata_82 | Scymnus_rubromaculatus_823 | 0.187 |
| Coccidula_scutellata_80 | Scymnus_rubromaculatus_824 | 0.187 |
| Coccidula_scutellata_81 | Scymnus_rubromaculatus_824 | 0.187 |
| Coccidula_scutellata_82 | Scymnus_rubromaculatus_824 | 0.187 |
| Coccidula_scutellata_80 | Scymnus_rubromaculatus_825 | 0.187 |
| Coccidula_scutellata_81 | Scymnus_rubromaculatus_825 | 0.187 |
| Coccidula_scutellata_82 | Scymnus_rubromaculatus_825 | 0.187 |
| Coccidula_scutellata_80 | Scymnus_rubromaculatus_826 | 0.187 |
| Coccidula_scutellata_81 | Scymnus_rubromaculatus_826 | 0.187 |
| Coccidula_scutellata_82 | Scymnus_rubromaculatus_826 | 0.187 |
| Coccidula_scutellata_80 | Scymnus_rubromaculatus_827 | 0.187 |
| Coccidula_scutellata_81 | Scymnus_rubromaculatus_827 | 0.187 |
| Coccidula_scutellata_82 | Scymnus_rubromaculatus_827 | 0.187 |
| Propylaea_quatuordecimpunctata_539 | Scymnus_suturalis_838 | 0.187 |
| Propylaea_quatuordecimpunctata_545 | Scymnus_suturalis_838 | 0.187 |
| Propylaea_quatuordecimpunctata_547 | Scymnus_suturalis_838 | 0.187 |
| Propylaea_quatuordecimpunctata_539 | Scymnus_suturalis_840 | 0.187 |
| Propylaea_quatuordecimpunctata_545 | Scymnus_suturalis_840 | 0.187 |
| Propylaea_quatuordecimpunctata_547 | Scymnus_suturalis_840 | 0.187 |
| Propylaea_quatuordecimpunctata_532 | Scymnus_suturalis_841 | 0.187 |
| Propylaea_quatuordecimpunctata_533 | Scymnus_suturalis_841 | 0.187 |
| Propylaea_quatuordecimpunctata_535 | Scymnus_suturalis_841 | 0.187 |
| Propylaea_quatuordecimpunctata_536 | Scymnus_suturalis_841 | 0.187 |
| Propylaea_quatuordecimpunctata_537 | Scymnus_suturalis_841 | 0.187 |
| Propylaea_quatuordecimpunctata_542 | Scymnus_suturalis_841 | 0.187 |
| Propylaea_quatuordecimpunctata_546 | Scymnus_suturalis_841 | 0.187 |
| Propylaea_quatuordecimpunctata_532 | Scymnus_suturalis_842 | 0.187 |
| Propylaea_quatuordecimpunctata_533 | Scymnus_suturalis_842 | 0.187 |
| Propylaea_quatuordecimpunctata_535 | Scymnus_suturalis_842 | 0.187 |
| Propylaea_quatuordecimpunctata_536 | Scymnus_suturalis_842 | 0.187 |
| Propylaea_quatuordecimpunctata_537 | Scymnus_suturalis_842 | 0.187 |
| Propylaea_quatuordecimpunctata_542 | Scymnus_suturalis_842 | 0.187 |
| Propylaea_quatuordecimpunctata_546 | Scymnus_suturalis_842 | 0.187 |
| Propylaea_quatuordecimpunctata_541 | Scymnus_suturalis_843 | 0.187 |
| Propylaea_quatuordecimpunctata_532 | Scymnus_suturalis_844 | 0.187 |
| Propylaea_quatuordecimpunctata_533 | Scymnus_suturalis_844 | 0.187 |
| Propylaea_quatuordecimpunctata_535 | Scymnus_suturalis_844 | 0.187 |
| Propylaea_quatuordecimpunctata_536 | Scymnus_suturalis_844 | 0.187 |
| Propylaea_quatuordecimpunctata_537 | Scymnus_suturalis_844 | 0.187 |
| Propylaea_quatuordecimpunctata_542 | Scymnus_suturalis_844 | 0.187 |
| Propylaea_quatuordecimpunctata_546 | Scymnus_suturalis_844 | 0.187 |
| Propylaea_quatuordecimpunctata_541 | Scymnus_suturalis_845 | 0.187 |
| Propylaea_quatuordecimpunctata_532 | Scymnus_suturalis_846 | 0.187 |
| Propylaea_quatuordecimpunctata_533 | Scymnus_suturalis_846 | 0.187 |
| Propylaea_quatuordecimpunctata_535 | Scymnus_suturalis_846 | 0.187 |
| Propylaea_quatuordecimpunctata_536 | Scymnus_suturalis_846 | 0.187 |
| Propylaea_quatuordecimpunctata_537 | Scymnus_suturalis_846 | 0.187 |
| Propylaea_quatuordecimpunctata_542 | Scymnus_suturalis_846 | 0.187 |

| | | |
|---|---|---|
| Propylaea_quatuordecimpunctata_546 | Scymnus_suturalis_846 | 0.187 |
| Propylaea_quatuordecimpunctata_532 | Scymnus_suturalis_847 | 0.187 |
| Propylaea_quatuordecimpunctata_533 | Scymnus_suturalis_847 | 0.187 |
| Propylaea_quatuordecimpunctata_535 | Scymnus_suturalis_847 | 0.187 |
| Propylaea_quatuordecimpunctata_536 | Scymnus_suturalis_847 | 0.187 |
| Propylaea_quatuordecimpunctata_537 | Scymnus_suturalis_847 | 0.187 |
| Propylaea_quatuordecimpunctata_542 | Scymnus_suturalis_847 | 0.187 |
| Propylaea_quatuordecimpunctata_546 | Scymnus_suturalis_847 | 0.187 |
| Propylaea_quatuordecimpunctata_532 | Scymnus_suturalis_848 | 0.187 |
| Propylaea_quatuordecimpunctata_533 | Scymnus_suturalis_848 | 0.187 |
| Propylaea_quatuordecimpunctata_535 | Scymnus_suturalis_848 | 0.187 |
| Propylaea_quatuordecimpunctata_536 | Scymnus_suturalis_848 | 0.187 |
| Propylaea_quatuordecimpunctata_537 | Scymnus_suturalis_848 | 0.187 |
| Propylaea_quatuordecimpunctata_542 | Scymnus_suturalis_848 | 0.187 |
| Propylaea_quatuordecimpunctata_546 | Scymnus_suturalis_848 | 0.187 |
| Scymnus_loewii_772 | Tytthaspis_sedecimpunctata_577 | 0.187 |
| Scymnus_ningshanensis_789 | Tytthaspis_sedecimpunctata_577 | 0.187 |
| Scymnus_ningshanensis_790 | Tytthaspis_sedecimpunctata_577 | 0.187 |
| Scymnus_loewii_772 | Tytthaspis_sedecimpunctata_578 | 0.187 |
| Scymnus_ningshanensis_789 | Tytthaspis_sedecimpunctata_578 | 0.187 |
| Scymnus_ningshanensis_790 | Tytthaspis_sedecimpunctata_578 | 0.187 |
| Scymnus_loewii_772 | Tytthaspis_sedecimpunctata_579 | 0.187 |
| Scymnus_ningshanensis_789 | Tytthaspis_sedecimpunctata_579 | 0.187 |
| Scymnus_ningshanensis_790 | Tytthaspis_sedecimpunctata_579 | 0.187 |
| Scymnus_loewii_772 | Tytthaspis_sedecimpunctata_581 | 0.187 |
| Scymnus_ningshanensis_789 | Tytthaspis_sedecimpunctata_581 | 0.187 |
| Scymnus_ningshanensis_790 | Tytthaspis_sedecimpunctata_581 | 0.187 |
| Scymnus_loewii_772 | Tytthaspis_sedecimpunctata_582 | 0.187 |
| Scymnus_ningshanensis_789 | Tytthaspis_sedecimpunctata_582 | 0.187 |
| Scymnus_ningshanensis_790 | Tytthaspis_sedecimpunctata_582 | 0.187 |
| Myzia_oblongoguttata_503 | Tytthaspis_sedecimpunctata_583 | 0.187 |
| Myzia_oblongoguttata_515 | Tytthaspis_sedecimpunctata_583 | 0.187 |
| Scymnus_loewii_772 | Tytthaspis_sedecimpunctata_585 | 0.187 |
| Scymnus_ningshanensis_789 | Tytthaspis_sedecimpunctata_585 | 0.187 |
| Scymnus_ningshanensis_790 | Tytthaspis_sedecimpunctata_585 | 0.187 |
| Myzia_oblongoguttata_503 | Tytthaspis_sedecimpunctata_589 | 0.187 |
| Myzia_oblongoguttata_515 | Tytthaspis_sedecimpunctata_589 | 0.187 |
| Scymnus_nebulosus_782 | Tytthaspis_sedecimpunctata_589 | 0.187 |
| Scymnus_ningshanensis_788 | Tytthaspis_sedecimpunctata_589 | 0.187 |
| Scymnus_ningshanensis_791 | Tytthaspis_sedecimpunctata_589 | 0.187 |
| Scymnus_ningshanensis_792 | Tytthaspis_sedecimpunctata_589 | 0.187 |
| Scymnus_ningshanensis_793 | Tytthaspis_sedecimpunctata_589 | 0.187 |
| Scymnus_ningshanensis_794 | Tytthaspis_sedecimpunctata_589 | 0.187 |
| Scymnus_ningshanensis_795 | Tytthaspis_sedecimpunctata_589 | 0.187 |
| Scymnus_loewii_772 | Tytthaspis_sedecimpunctata_590 | 0.187 |
| Scymnus_ningshanensis_789 | Tytthaspis_sedecimpunctata_590 | 0.187 |
| Scymnus_ningshanensis_790 | Tytthaspis_sedecimpunctata_590 | 0.187 |
| Scymnus_loewii_772 | Tytthaspis_sedecimpunctata_592 | 0.187 |
| Scymnus_ningshanensis_789 | Tytthaspis_sedecimpunctata_592 | 0.187 |
| Scymnus_ningshanensis_790 | Tytthaspis_sedecimpunctata_592 | 0.187 |
| Scymnus_loewii_772 | Tytthaspis_sedecimpunctata_593 | 0.187 |
| Scymnus_loewii_772 | Tytthaspis_sedecimpunctata_594 | 0.187 |
| Scymnus_ningshanensis_789 | Tytthaspis_sedecimpunctata_594 | 0.187 |
| Scymnus_ningshanensis_790 | Tytthaspis_sedecimpunctata_594 | 0.187 |
| Scymnus_loewii_772 | Tytthaspis_sedecimpunctata_595 | 0.187 |
| Scymnus_ningshanensis_789 | Tytthaspis_sedecimpunctata_595 | 0.187 |

Supplementary Table S4 (continued)

| | | |
|---|---|---|
| Scymnus_ningshanensis_790 | Tytthaspis_sedecimpunctata_595 | 0.187 |
| Propylaea_quatuordecimpunctata_532 | Scymnus_suturalis_832 | 0.187 |
| Propylaea_quatuordecimpunctata_533 | Scymnus_suturalis_832 | 0.187 |
| Propylaea_quatuordecimpunctata_535 | Scymnus_suturalis_832 | 0.187 |
| Propylaea_quatuordecimpunctata_536 | Scymnus_suturalis_832 | 0.187 |
| Propylaea_quatuordecimpunctata_537 | Scymnus_suturalis_832 | 0.187 |
| Propylaea_quatuordecimpunctata_542 | Scymnus_suturalis_832 | 0.187 |
| Propylaea_quatuordecimpunctata_546 | Scymnus_suturalis_832 | 0.187 |
| Propylaea_quatuordecimpunctata_539 | Scymnus_suturalis_834 | 0.187 |
| Propylaea_quatuordecimpunctata_545 | Scymnus_suturalis_834 | 0.187 |
| Propylaea_quatuordecimpunctata_547 | Scymnus_suturalis_834 | 0.187 |
| Propylaea_quatuordecimpunctata_532 | Scymnus_suturalis_835 | 0.187 |
| Propylaea_quatuordecimpunctata_533 | Scymnus_suturalis_835 | 0.187 |
| Propylaea_quatuordecimpunctata_535 | Scymnus_suturalis_835 | 0.187 |
| Propylaea_quatuordecimpunctata_536 | Scymnus_suturalis_835 | 0.187 |
| Propylaea_quatuordecimpunctata_537 | Scymnus_suturalis_835 | 0.187 |
| Propylaea_quatuordecimpunctata_542 | Scymnus_suturalis_835 | 0.187 |
| Propylaea_quatuordecimpunctata_546 | Scymnus_suturalis_835 | 0.187 |
| Propylaea_quatuordecimpunctata_532 | Scymnus_suturalis_836 | 0.187 |
| Propylaea_quatuordecimpunctata_533 | Scymnus_suturalis_836 | 0.187 |
| Propylaea_quatuordecimpunctata_535 | Scymnus_suturalis_836 | 0.187 |
| Propylaea_quatuordecimpunctata_536 | Scymnus_suturalis_836 | 0.187 |
| Propylaea_quatuordecimpunctata_537 | Scymnus_suturalis_836 | 0.187 |
| Propylaea_quatuordecimpunctata_542 | Scymnus_suturalis_836 | 0.187 |
| Propylaea_quatuordecimpunctata_546 | Scymnus_suturalis_836 | 0.187 |
| Propylaea_quatuordecimpunctata_539 | Scymnus_suturalis_837 | 0.187 |
| Propylaea_quatuordecimpunctata_545 | Scymnus_suturalis_837 | 0.187 |
| Propylaea_quatuordecimpunctata_547 | Scymnus_suturalis_837 | 0.187 |
| Chilocorus_stigma_47 | Scymnus_suturalis_838 | 0.187 |
| Coccidula_scutellata_83 | Scymnus_suturalis_838 | 0.187 |
| Propylaea_quatuordecimpunctata_532 | Scymnus_suturalis_839 | 0.187 |
| Propylaea_quatuordecimpunctata_533 | Scymnus_suturalis_839 | 0.187 |
| Propylaea_quatuordecimpunctata_535 | Scymnus_suturalis_839 | 0.187 |
| Propylaea_quatuordecimpunctata_536 | Scymnus_suturalis_839 | 0.187 |
| Propylaea_quatuordecimpunctata_537 | Scymnus_suturalis_839 | 0.187 |
| Propylaea_quatuordecimpunctata_542 | Scymnus_suturalis_839 | 0.187 |
| Propylaea_quatuordecimpunctata_546 | Scymnus_suturalis_839 | 0.187 |
| Chilocorus_stigma_47 | Scymnus_suturalis_840 | 0.187 |
| Coccidula_scutellata_83 | Scymnus_suturalis_840 | 0.187 |
| Chilocorus_stigma_44 | Scymnus_suturalis_841 | 0.187 |
| Chilocorus_stigma_46 | Scymnus_suturalis_841 | 0.187 |
| Chilocorus_stigma_44 | Scymnus_suturalis_842 | 0.187 |
| Chilocorus_stigma_46 | Scymnus_suturalis_842 | 0.187 |
| Myrrha_octodecimguttata_485 | Scymnus_suturalis_843 | 0.187 |
| Myrrha_octodecimguttata_486 | Scymnus_suturalis_843 | 0.187 |
| Myrrha_octodecimguttata_487 | Scymnus_suturalis_843 | 0.187 |
| Myrrha_octodecimguttata_490 | Scymnus_suturalis_843 | 0.187 |
| Myrrha_octodecimguttata_494 | Scymnus_suturalis_843 | 0.187 |
| Myrrha_octodecimguttata_496 | Scymnus_suturalis_843 | 0.187 |
| Myrrha_octodecimguttata_497 | Scymnus_suturalis_843 | 0.187 |
| Myrrha_octodecimguttata_501 | Scymnus_suturalis_843 | 0.187 |
| Myrrha_octodecimguttata_502 | Scymnus_suturalis_843 | 0.187 |
| Chilocorus_stigma_44 | Scymnus_suturalis_844 | 0.187 |
| Chilocorus_stigma_46 | Scymnus_suturalis_844 | 0.187 |
| Myrrha_octodecimguttata_485 | Scymnus_suturalis_845 | 0.187 |
| Myrrha_octodecimguttata_486 | Scymnus_suturalis_845 | 0.187 |

| | | |
|---|---|---|
| Myrrha_octodecimguttata_487 | Scymnus_suturalis_845 | 0.187 |
| Myrrha_octodecimguttata_490 | Scymnus_suturalis_845 | 0.187 |
| Myrrha_octodecimguttata_494 | Scymnus_suturalis_845 | 0.187 |
| Myrrha_octodecimguttata_496 | Scymnus_suturalis_845 | 0.187 |
| Myrrha_octodecimguttata_497 | Scymnus_suturalis_845 | 0.187 |
| Myrrha_octodecimguttata_501 | Scymnus_suturalis_845 | 0.187 |
| Myrrha_octodecimguttata_502 | Scymnus_suturalis_845 | 0.187 |
| Nephus_redtenbacheri_678 | Scymnus_suturalis_845 | 0.187 |
| Chilocorus_stigma_44 | Scymnus_suturalis_846 | 0.187 |
| Chilocorus_stigma_46 | Scymnus_suturalis_846 | 0.187 |
| Chilocorus_stigma_44 | Scymnus_suturalis_847 | 0.187 |
| Chilocorus_stigma_46 | Scymnus_suturalis_847 | 0.187 |
| Chilocorus_stigma_44 | Scymnus_suturalis_848 | 0.187 |
| Chilocorus_stigma_46 | Scymnus_suturalis_848 | 0.187 |
| Hyperaspis_oregona_658 | Stethorus_punctillum_849 | 0.187 |
| Hyperaspis_oregona_659 | Stethorus_punctillum_849 | 0.187 |
| Hyperaspis_oregona_658 | Stethorus_punctillum_854 | 0.187 |
| Hyperaspis_oregona_659 | Stethorus_punctillum_854 | 0.187 |
| Hyperaspis_oregona_658 | Stethorus_punctillum_856 | 0.187 |
| Hyperaspis_oregona_659 | Stethorus_punctillum_856 | 0.187 |
| Scymnus_ningshanensis_789 | Tytthaspis_sedecimpunctata_580 | 0.187 |
| Scymnus_ningshanensis_790 | Tytthaspis_sedecimpunctata_580 | 0.187 |
| Scymnus_loewii_773 | Tytthaspis_sedecimpunctata_584 | 0.187 |
| Didion_punctatum_645 | Tytthaspis_sedecimpunctata_585 | 0.187 |
| Cheilomenes_sexmaculata_224 | Tytthaspis_sedecimpunctata_586 | 0.187 |
| Myzia_oblongoguttata_503 | Tytthaspis_sedecimpunctata_587 | 0.187 |
| Myzia_oblongoguttata_515 | Tytthaspis_sedecimpunctata_587 | 0.187 |
| Scymnus_nebulosus_782 | Tytthaspis_sedecimpunctata_587 | 0.187 |
| Cheilomenes_sexmaculata_224 | Tytthaspis_sedecimpunctata_588 | 0.187 |
| Myzia_oblongoguttata_506 | Tytthaspis_sedecimpunctata_591 | 0.187 |
| Chilocorus_stigma_44 | Scymnus_suturalis_832 | 0.187 |
| Chilocorus_stigma_46 | Scymnus_suturalis_832 | 0.187 |
| Propylaea_quatuordecimpunctata_539 | Scymnus_suturalis_833 | 0.187 |
| Propylaea_quatuordecimpunctata_545 | Scymnus_suturalis_833 | 0.187 |
| Propylaea_quatuordecimpunctata_547 | Scymnus_suturalis_833 | 0.187 |
| Chilocorus_stigma_47 | Scymnus_suturalis_834 | 0.187 |
| Coccidula_scutellata_83 | Scymnus_suturalis_834 | 0.187 |
| Nephus_redtenbacheri_678 | Scymnus_suturalis_834 | 0.187 |
| Chilocorus_stigma_44 | Scymnus_suturalis_835 | 0.187 |
| Chilocorus_stigma_46 | Scymnus_suturalis_835 | 0.187 |
| Chilocorus_stigma_44 | Scymnus_suturalis_836 | 0.187 |
| Chilocorus_stigma_46 | Scymnus_suturalis_836 | 0.187 |
| Chilocorus_stigma_44 | Scymnus_suturalis_837 | 0.187 |
| Chilocorus_stigma_46 | Scymnus_suturalis_837 | 0.187 |
| Myrrha_octodecimguttata_489 | Scymnus_suturalis_838 | 0.187 |
| Myrrha_octodecimguttata_500 | Scymnus_suturalis_838 | 0.187 |
| Chilocorus_stigma_44 | Scymnus_suturalis_839 | 0.187 |
| Chilocorus_stigma_46 | Scymnus_suturalis_839 | 0.187 |
| Myrrha_octodecimguttata_488 | Scymnus_suturalis_840 | 0.187 |
| Myrrha_octodecimguttata_491 | Scymnus_suturalis_840 | 0.187 |
| Myrrha_octodecimguttata_492 | Scymnus_suturalis_840 | 0.187 |
| Myrrha_octodecimguttata_489 | Scymnus_suturalis_841 | 0.188 |
| Myrrha_octodecimguttata_493 | Scymnus_suturalis_841 | 0.188 |
| Myrrha_octodecimguttata_495 | Scymnus_suturalis_841 | 0.188 |
| Myrrha_octodecimguttata_498 | Scymnus_suturalis_841 | 0.188 |
| Myrrha_octodecimguttata_500 | Scymnus_suturalis_841 | 0.188 |

| | | |
|---|---|---|
| Myrrha_octodecimguttata_489 | Scymnus_suturalis_842 | 0.188 |
| Myrrha_octodecimguttata_493 | Scymnus_suturalis_842 | 0.188 |
| Myrrha_octodecimguttata_495 | Scymnus_suturalis_842 | 0.188 |
| Myrrha_octodecimguttata_498 | Scymnus_suturalis_842 | 0.188 |
| Myrrha_octodecimguttata_500 | Scymnus_suturalis_842 | 0.188 |
| Myrrha_octodecimguttata_489 | Scymnus_suturalis_844 | 0.188 |
| Myrrha_octodecimguttata_493 | Scymnus_suturalis_844 | 0.188 |
| Myrrha_octodecimguttata_495 | Scymnus_suturalis_844 | 0.188 |
| Myrrha_octodecimguttata_498 | Scymnus_suturalis_844 | 0.188 |
| Myrrha_octodecimguttata_500 | Scymnus_suturalis_844 | 0.188 |
| Nephus_redtenbacheri_679 | Scymnus_suturalis_845 | 0.188 |
| Myrrha_octodecimguttata_489 | Scymnus_suturalis_846 | 0.188 |
| Myrrha_octodecimguttata_493 | Scymnus_suturalis_846 | 0.188 |
| Myrrha_octodecimguttata_495 | Scymnus_suturalis_846 | 0.188 |
| Myrrha_octodecimguttata_498 | Scymnus_suturalis_846 | 0.188 |
| Myrrha_octodecimguttata_500 | Scymnus_suturalis_846 | 0.188 |
| Myrrha_octodecimguttata_489 | Scymnus_suturalis_847 | 0.188 |
| Myrrha_octodecimguttata_493 | Scymnus_suturalis_847 | 0.188 |
| Myrrha_octodecimguttata_495 | Scymnus_suturalis_847 | 0.188 |
| Myrrha_octodecimguttata_498 | Scymnus_suturalis_847 | 0.188 |
| Myrrha_octodecimguttata_500 | Scymnus_suturalis_847 | 0.188 |
| Myrrha_octodecimguttata_489 | Scymnus_suturalis_848 | 0.188 |
| Myrrha_octodecimguttata_493 | Scymnus_suturalis_848 | 0.188 |
| Myrrha_octodecimguttata_495 | Scymnus_suturalis_848 | 0.188 |
| Myrrha_octodecimguttata_498 | Scymnus_suturalis_848 | 0.188 |
| Myrrha_octodecimguttata_500 | Scymnus_suturalis_848 | 0.188 |
| Hyperaspis_lateralis_655 | Subcoccinella_vigintiquatuorpunctata_620 | 0.188 |
| Hyperaspis_lateralis_655 | Subcoccinella_vigintiquatuorpunctata_621 | 0.188 |
| Hyperaspis_lateralis_655 | Subcoccinella_vigintiquatuorpunctata_628 | 0.188 |
| Hyperaspis_lateralis_655 | Subcoccinella_vigintiquatuorpunctata_629 | 0.188 |
| Hyperaspis_lateralis_655 | Subcoccinella_vigintiquatuorpunctata_631 | 0.188 |
| Cheilomenes_sexmaculata_224 | Tytthaspis_sedecimpunctata_577 | 0.188 |
| Cheilomenes_sexmaculata_224 | Tytthaspis_sedecimpunctata_578 | 0.188 |
| Cheilomenes_sexmaculata_224 | Tytthaspis_sedecimpunctata_579 | 0.188 |
| Cheilomenes_sexmaculata_224 | Tytthaspis_sedecimpunctata_581 | 0.188 |
| Cheilomenes_sexmaculata_224 | Tytthaspis_sedecimpunctata_582 | 0.188 |
| Didion_punctatum_646 | Tytthaspis_sedecimpunctata_585 | 0.188 |
| Coccinella_septempunctata_242 | Tytthaspis_sedecimpunctata_586 | 0.188 |
| Coccinella_septempunctata_244 | Tytthaspis_sedecimpunctata_586 | 0.188 |
| Coccinella_septempunctata_245 | Tytthaspis_sedecimpunctata_586 | 0.188 |
| Coccinella_septempunctata_247 | Tytthaspis_sedecimpunctata_586 | 0.188 |
| Coccinella_septempunctata_248 | Tytthaspis_sedecimpunctata_586 | 0.188 |
| Coccinella_septempunctata_249 | Tytthaspis_sedecimpunctata_586 | 0.188 |
| Coccinella_septempunctata_252 | Tytthaspis_sedecimpunctata_586 | 0.188 |
| Coccinella_septempunctata_255 | Tytthaspis_sedecimpunctata_586 | 0.188 |
| Coccinella_septempunctata_256 | Tytthaspis_sedecimpunctata_586 | 0.188 |
| Aphidecta_obliterata_183 | Tytthaspis_sedecimpunctata_587 | 0.188 |
| Coccinella_septempunctata_242 | Tytthaspis_sedecimpunctata_588 | 0.188 |
| Coccinella_septempunctata_244 | Tytthaspis_sedecimpunctata_588 | 0.188 |
| Coccinella_septempunctata_245 | Tytthaspis_sedecimpunctata_588 | 0.188 |
| Coccinella_septempunctata_247 | Tytthaspis_sedecimpunctata_588 | 0.188 |
| Coccinella_septempunctata_248 | Tytthaspis_sedecimpunctata_588 | 0.188 |
| Coccinella_septempunctata_249 | Tytthaspis_sedecimpunctata_588 | 0.188 |
| Coccinella_septempunctata_252 | Tytthaspis_sedecimpunctata_588 | 0.188 |
| Coccinella_septempunctata_255 | Tytthaspis_sedecimpunctata_588 | 0.188 |
| Coccinella_septempunctata_256 | Tytthaspis_sedecimpunctata_588 | 0.188 |

| | | |
|---|---|---|
| Coccinella_septempunctata_254 | Tytthaspis_sedecimpunctata_589 | 0.188 |
| Cheilomenes_sexmaculata_224 | Tytthaspis_sedecimpunctata_590 | 0.188 |
| Scymnus_camptodromus_704 | Tytthaspis_sedecimpunctata_591 | 0.188 |
| Cheilomenes_sexmaculata_224 | Tytthaspis_sedecimpunctata_592 | 0.188 |
| Cheilomenes_sexmaculata_224 | Tytthaspis_sedecimpunctata_594 | 0.188 |
| Cheilomenes_sexmaculata_224 | Tytthaspis_sedecimpunctata_595 | 0.188 |
| Coccidula_scutellata_78 | Scymnus_sinuanodulus_830 | 0.188 |
| Coccidula_scutellata_79 | Scymnus_sinuanodulus_830 | 0.188 |
| Myrrha_octodecimguttata_489 | Scymnus_suturalis_832 | 0.188 |
| Myrrha_octodecimguttata_493 | Scymnus_suturalis_832 | 0.188 |
| Myrrha_octodecimguttata_495 | Scymnus_suturalis_832 | 0.188 |
| Myrrha_octodecimguttata_498 | Scymnus_suturalis_832 | 0.188 |
| Myrrha_octodecimguttata_500 | Scymnus_suturalis_832 | 0.188 |
| Myrrha_octodecimguttata_488 | Scymnus_suturalis_834 | 0.188 |
| Myrrha_octodecimguttata_491 | Scymnus_suturalis_834 | 0.188 |
| Myrrha_octodecimguttata_492 | Scymnus_suturalis_834 | 0.188 |
| Nephus_redtenbacheri_679 | Scymnus_suturalis_834 | 0.188 |
| Myrrha_octodecimguttata_489 | Scymnus_suturalis_835 | 0.188 |
| Myrrha_octodecimguttata_493 | Scymnus_suturalis_835 | 0.188 |
| Myrrha_octodecimguttata_495 | Scymnus_suturalis_835 | 0.188 |
| Myrrha_octodecimguttata_498 | Scymnus_suturalis_835 | 0.188 |
| Myrrha_octodecimguttata_500 | Scymnus_suturalis_835 | 0.188 |
| Myrrha_octodecimguttata_489 | Scymnus_suturalis_836 | 0.188 |
| Myrrha_octodecimguttata_493 | Scymnus_suturalis_836 | 0.188 |
| Myrrha_octodecimguttata_495 | Scymnus_suturalis_836 | 0.188 |
| Myrrha_octodecimguttata_498 | Scymnus_suturalis_836 | 0.188 |
| Myrrha_octodecimguttata_500 | Scymnus_suturalis_836 | 0.188 |
| Myrrha_octodecimguttata_488 | Scymnus_suturalis_837 | 0.188 |
| Myrrha_octodecimguttata_491 | Scymnus_suturalis_837 | 0.188 |
| Myrrha_octodecimguttata_492 | Scymnus_suturalis_837 | 0.188 |
| Anatis_labiculata_123 | Scymnus_suturalis_838 | 0.188 |
| Anatis_labiculata_124 | Scymnus_suturalis_838 | 0.188 |
| Anatis_labiculata_125 | Scymnus_suturalis_838 | 0.188 |
| Anatis_labiculata_127 | Scymnus_suturalis_838 | 0.188 |
| Myrrha_octodecimguttata_489 | Scymnus_suturalis_839 | 0.188 |
| Myrrha_octodecimguttata_493 | Scymnus_suturalis_839 | 0.188 |
| Myrrha_octodecimguttata_495 | Scymnus_suturalis_839 | 0.188 |
| Myrrha_octodecimguttata_498 | Scymnus_suturalis_839 | 0.188 |
| Myrrha_octodecimguttata_500 | Scymnus_suturalis_839 | 0.188 |
| Anatis_labiculata_126 | Scymnus_suturalis_840 | 0.188 |
| Anatis_labiculata_126 | Scymnus_suturalis_842 | 0.188 |
| Anatis_labiculata_126 | Scymnus_suturalis_844 | 0.188 |
| Anatis_labiculata_126 | Scymnus_suturalis_846 | 0.188 |
| Anatis_labiculata_126 | Scymnus_suturalis_847 | 0.188 |
| Anatis_labiculata_126 | Scymnus_suturalis_848 | 0.188 |
| Scymnus_nebulosus_782 | Subcoccinella_vigintiquatuorpunctata_623 | 0.188 |
| Scymnus_nebulosus_781 | Subcoccinella_vigintiquatuorpunctata_630 | 0.188 |
| Coccinella_septempunctata_246 | Tytthaspis_sedecimpunctata_577 | 0.188 |
| Scymnus_caurinus_720 | Tytthaspis_sedecimpunctata_577 | 0.188 |
| Scymnus_caurinus_721 | Tytthaspis_sedecimpunctata_577 | 0.188 |
| Scymnus_caurinus_722 | Tytthaspis_sedecimpunctata_577 | 0.188 |
| Coccinella_septempunctata_246 | Tytthaspis_sedecimpunctata_578 | 0.188 |
| Scymnus_caurinus_720 | Tytthaspis_sedecimpunctata_578 | 0.188 |
| Scymnus_caurinus_721 | Tytthaspis_sedecimpunctata_578 | 0.188 |
| Scymnus_caurinus_722 | Tytthaspis_sedecimpunctata_578 | 0.188 |
| Coccinella_septempunctata_246 | Tytthaspis_sedecimpunctata_579 | 0.188 |

| | | |
|---|---|---|
| Scymnus_caurinus_720 | Tytthaspis_sedecimpunctata_579 | 0.188 |
| Scymnus_caurinus_721 | Tytthaspis_sedecimpunctata_579 | 0.188 |
| Scymnus_caurinus_722 | Tytthaspis_sedecimpunctata_579 | 0.188 |
| Coccinella_septempunctata_246 | Tytthaspis_sedecimpunctata_581 | 0.188 |
| Scymnus_caurinus_720 | Tytthaspis_sedecimpunctata_581 | 0.188 |
| Scymnus_caurinus_721 | Tytthaspis_sedecimpunctata_581 | 0.188 |
| Scymnus_caurinus_722 | Tytthaspis_sedecimpunctata_581 | 0.188 |
| Coccinella_septempunctata_246 | Tytthaspis_sedecimpunctata_582 | 0.188 |
| Scymnus_caurinus_720 | Tytthaspis_sedecimpunctata_582 | 0.188 |
| Scymnus_caurinus_721 | Tytthaspis_sedecimpunctata_582 | 0.188 |
| Scymnus_caurinus_722 | Tytthaspis_sedecimpunctata_582 | 0.188 |
| Coccinella_septempunctata_241 | Tytthaspis_sedecimpunctata_583 | 0.188 |
| Coccinella_septempunctata_243 | Tytthaspis_sedecimpunctata_583 | 0.188 |
| Coccinella_septempunctata_253 | Tytthaspis_sedecimpunctata_583 | 0.188 |
| Cheilomenes_sexmaculata_224 | Tytthaspis_sedecimpunctata_584 | 0.188 |
| Scymnus_interruptus_760 | Tytthaspis_sedecimpunctata_584 | 0.188 |
| Scymnus_interruptus_765 | Tytthaspis_sedecimpunctata_584 | 0.188 |
| Scymnus_interruptus_768 | Tytthaspis_sedecimpunctata_584 | 0.188 |
| Coccinella_septempunctata_246 | Tytthaspis_sedecimpunctata_585 | 0.188 |
| Didion_punctatum_644 | Tytthaspis_sedecimpunctata_585 | 0.188 |
| Coccinella_septempunctata_250 | Tytthaspis_sedecimpunctata_586 | 0.188 |
| Coccinella_septempunctata_251 | Tytthaspis_sedecimpunctata_586 | 0.188 |
| Aphidecta_obliterata_176 | Tytthaspis_sedecimpunctata_587 | 0.188 |
| Coccinella_septempunctata_250 | Tytthaspis_sedecimpunctata_588 | 0.188 |
| Coccinella_septempunctata_251 | Tytthaspis_sedecimpunctata_588 | 0.188 |
| Coccinella_septempunctata_246 | Tytthaspis_sedecimpunctata_590 | 0.188 |
| Scymnus_caurinus_720 | Tytthaspis_sedecimpunctata_590 | 0.188 |
| Scymnus_caurinus_721 | Tytthaspis_sedecimpunctata_590 | 0.188 |
| Scymnus_caurinus_722 | Tytthaspis_sedecimpunctata_590 | 0.188 |
| Coccinella_septempunctata_246 | Tytthaspis_sedecimpunctata_592 | 0.188 |
| Coccinella_septempunctata_246 | Tytthaspis_sedecimpunctata_593 | 0.188 |
| Scymnus_caurinus_720 | Tytthaspis_sedecimpunctata_593 | 0.188 |
| Scymnus_caurinus_721 | Tytthaspis_sedecimpunctata_593 | 0.188 |
| Scymnus_caurinus_722 | Tytthaspis_sedecimpunctata_593 | 0.188 |
| Coccinella_septempunctata_246 | Tytthaspis_sedecimpunctata_594 | 0.188 |
| Scymnus_caurinus_720 | Tytthaspis_sedecimpunctata_594 | 0.188 |
| Scymnus_caurinus_721 | Tytthaspis_sedecimpunctata_594 | 0.188 |
| Scymnus_caurinus_722 | Tytthaspis_sedecimpunctata_594 | 0.188 |
| Coccinella_septempunctata_246 | Tytthaspis_sedecimpunctata_595 | 0.188 |
| Scymnus_caurinus_720 | Tytthaspis_sedecimpunctata_595 | 0.188 |
| Scymnus_caurinus_721 | Tytthaspis_sedecimpunctata_595 | 0.188 |
| Scymnus_caurinus_722 | Tytthaspis_sedecimpunctata_595 | 0.188 |
| Chilocorus_bipustulatus_18 | Scymnus_sinuanodulus_830 | 0.188 |
| Chilocorus_bipustulatus_19 | Scymnus_sinuanodulus_830 | 0.188 |
| Chilocorus_bipustulatus_20 | Scymnus_sinuanodulus_830 | 0.188 |
| Chilocorus_bipustulatus_22 | Scymnus_sinuanodulus_830 | 0.188 |
| Chilocorus_bipustulatus_24 | Scymnus_sinuanodulus_830 | 0.188 |
| Anatis_labiculata_126 | Scymnus_suturalis_832 | 0.188 |
| Anatis_mali_128 | Scymnus_suturalis_833 | 0.188 |
| Anatis_mali_129 | Scymnus_suturalis_833 | 0.188 |
| Anatis_mali_130 | Scymnus_suturalis_833 | 0.188 |
| Anatis_mali_131 | Scymnus_suturalis_833 | 0.188 |
| Anatis_labiculata_126 | Scymnus_suturalis_835 | 0.188 |
| Anatis_labiculata_126 | Scymnus_suturalis_836 | 0.188 |
| Anatis_labiculata_123 | Scymnus_suturalis_837 | 0.188 |
| Anatis_labiculata_124 | Scymnus_suturalis_837 | 0.188 |

| | | |
|---|---|---|
| Anatis_labiculata_125 | Scymnus_suturalis_837 | 0.188 |
| Anatis_labiculata_127 | Scymnus_suturalis_837 | 0.188 |
| Anatis_labiculata_126 | Scymnus_suturalis_839 | 0.188 |
| Anatis_labiculata_126 | Scymnus_suturalis_841 | 0.188 |
| Platynaspis_luteorubra_50 | Scymnus_sinuanodulus_831 | 0.188 |
| Anatis_mali_128 | Scymnus_suturalis_842 | 0.188 |
| Anatis_mali_129 | Scymnus_suturalis_842 | 0.188 |
| Anatis_mali_130 | Scymnus_suturalis_842 | 0.188 |
| Anatis_mali_131 | Scymnus_suturalis_842 | 0.188 |
| Anatis_mali_128 | Scymnus_suturalis_844 | 0.188 |
| Anatis_mali_129 | Scymnus_suturalis_844 | 0.188 |
| Anatis_mali_130 | Scymnus_suturalis_844 | 0.188 |
| Anatis_mali_131 | Scymnus_suturalis_844 | 0.188 |
| Anatis_mali_128 | Scymnus_suturalis_846 | 0.188 |
| Anatis_mali_129 | Scymnus_suturalis_846 | 0.188 |
| Anatis_mali_130 | Scymnus_suturalis_846 | 0.188 |
| Anatis_mali_131 | Scymnus_suturalis_846 | 0.188 |
| Anatis_mali_128 | Scymnus_suturalis_847 | 0.188 |
| Anatis_mali_129 | Scymnus_suturalis_847 | 0.188 |
| Anatis_mali_130 | Scymnus_suturalis_847 | 0.188 |
| Anatis_mali_131 | Scymnus_suturalis_847 | 0.188 |
| Anatis_mali_128 | Scymnus_suturalis_848 | 0.188 |
| Anatis_mali_129 | Scymnus_suturalis_848 | 0.188 |
| Anatis_mali_130 | Scymnus_suturalis_848 | 0.188 |
| Anatis_mali_131 | Scymnus_suturalis_848 | 0.188 |
| Hyperaspis_proba_661 | Stethorus_punctillum_853 | 0.188 |
| Scymnus_nebulosus_782 | Subcoccinella_vigintiquatuorpunctata_622 | 0.188 |
| Scymnus_nebulosus_782 | Subcoccinella_vigintiquatuorpunctata_626 | 0.188 |
| Brachiacantha_rotunda_632 | Tytthaspis_sedecimpunctata_577 | 0.188 |
| Brachiacantha_rotunda_633 | Tytthaspis_sedecimpunctata_577 | 0.188 |
| Brachiacantha_rotunda_635 | Tytthaspis_sedecimpunctata_577 | 0.188 |
| Brachiacantha_rotunda_636 | Tytthaspis_sedecimpunctata_577 | 0.188 |
| Scymnus_interruptus_760 | Tytthaspis_sedecimpunctata_577 | 0.188 |
| Scymnus_interruptus_765 | Tytthaspis_sedecimpunctata_577 | 0.188 |
| Scymnus_interruptus_768 | Tytthaspis_sedecimpunctata_577 | 0.188 |
| Brachiacantha_rotunda_632 | Tytthaspis_sedecimpunctata_578 | 0.188 |
| Brachiacantha_rotunda_633 | Tytthaspis_sedecimpunctata_578 | 0.188 |
| Brachiacantha_rotunda_635 | Tytthaspis_sedecimpunctata_578 | 0.188 |
| Brachiacantha_rotunda_636 | Tytthaspis_sedecimpunctata_578 | 0.188 |
| Scymnus_interruptus_760 | Tytthaspis_sedecimpunctata_578 | 0.188 |
| Scymnus_interruptus_765 | Tytthaspis_sedecimpunctata_578 | 0.188 |
| Scymnus_interruptus_768 | Tytthaspis_sedecimpunctata_578 | 0.188 |
| Brachiacantha_rotunda_632 | Tytthaspis_sedecimpunctata_579 | 0.188 |
| Brachiacantha_rotunda_633 | Tytthaspis_sedecimpunctata_579 | 0.188 |
| Brachiacantha_rotunda_635 | Tytthaspis_sedecimpunctata_579 | 0.188 |
| Brachiacantha_rotunda_636 | Tytthaspis_sedecimpunctata_579 | 0.188 |
| Scymnus_interruptus_760 | Tytthaspis_sedecimpunctata_579 | 0.188 |
| Scymnus_interruptus_765 | Tytthaspis_sedecimpunctata_579 | 0.188 |
| Scymnus_interruptus_768 | Tytthaspis_sedecimpunctata_579 | 0.188 |
| Coccinella_septempunctata_246 | Tytthaspis_sedecimpunctata_580 | 0.188 |
| Brachiacantha_rotunda_632 | Tytthaspis_sedecimpunctata_581 | 0.188 |
| Brachiacantha_rotunda_633 | Tytthaspis_sedecimpunctata_581 | 0.188 |
| Brachiacantha_rotunda_635 | Tytthaspis_sedecimpunctata_581 | 0.188 |
| Brachiacantha_rotunda_636 | Tytthaspis_sedecimpunctata_581 | 0.188 |
| Scymnus_interruptus_760 | Tytthaspis_sedecimpunctata_581 | 0.188 |
| Scymnus_interruptus_765 | Tytthaspis_sedecimpunctata_581 | 0.188 |

| | | |
|---|---|---|
| Scymnus_interruptus_768 | Tytthaspis_sedecimpunctata_581 | 0.188 |
| Brachiacantha_rotunda_632 | Tytthaspis_sedecimpunctata_582 | 0.188 |
| Brachiacantha_rotunda_633 | Tytthaspis_sedecimpunctata_582 | 0.188 |
| Brachiacantha_rotunda_635 | Tytthaspis_sedecimpunctata_582 | 0.188 |
| Brachiacantha_rotunda_636 | Tytthaspis_sedecimpunctata_582 | 0.188 |
| Scymnus_interruptus_760 | Tytthaspis_sedecimpunctata_582 | 0.188 |
| Scymnus_interruptus_765 | Tytthaspis_sedecimpunctata_582 | 0.188 |
| Scymnus_interruptus_768 | Tytthaspis_sedecimpunctata_582 | 0.188 |
| Scymnus_camptodromus_705 | Tytthaspis_sedecimpunctata_583 | 0.188 |
| Scymnus_camptodromus_713 | Tytthaspis_sedecimpunctata_583 | 0.188 |
| Scymnus_camptodromus_716 | Tytthaspis_sedecimpunctata_583 | 0.188 |
| Scymnus_interruptus_761 | Tytthaspis_sedecimpunctata_583 | 0.188 |
| Scymnus_interruptus_763 | Tytthaspis_sedecimpunctata_583 | 0.188 |
| Scymnus_interruptus_764 | Tytthaspis_sedecimpunctata_583 | 0.188 |
| Scymnus_interruptus_766 | Tytthaspis_sedecimpunctata_583 | 0.188 |
| Aphidecta_obliterata_183 | Tytthaspis_sedecimpunctata_584 | 0.188 |
| Coccinella_septempunctata_246 | Tytthaspis_sedecimpunctata_584 | 0.188 |
| Scymnus_nigrinus_785 | Tytthaspis_sedecimpunctata_586 | 0.188 |
| Scymnus_nigrinus_786 | Tytthaspis_sedecimpunctata_586 | 0.188 |
| Scymnus_nigrinus_784 | Tytthaspis_sedecimpunctata_588 | 0.188 |
| Scymnus_interruptus_760 | Tytthaspis_sedecimpunctata_589 | 0.188 |
| Scymnus_interruptus_765 | Tytthaspis_sedecimpunctata_589 | 0.188 |
| Scymnus_interruptus_768 | Tytthaspis_sedecimpunctata_589 | 0.188 |
| Brachiacantha_rotunda_632 | Tytthaspis_sedecimpunctata_590 | 0.188 |
| Brachiacantha_rotunda_633 | Tytthaspis_sedecimpunctata_590 | 0.188 |
| Brachiacantha_rotunda_635 | Tytthaspis_sedecimpunctata_590 | 0.188 |
| Brachiacantha_rotunda_636 | Tytthaspis_sedecimpunctata_590 | 0.188 |
| Scymnus_interruptus_760 | Tytthaspis_sedecimpunctata_590 | 0.188 |
| Scymnus_interruptus_765 | Tytthaspis_sedecimpunctata_590 | 0.188 |
| Scymnus_interruptus_768 | Tytthaspis_sedecimpunctata_590 | 0.188 |
| Scymnus_camptodromus_712 | Tytthaspis_sedecimpunctata_591 | 0.188 |
| Brachiacantha_rotunda_632 | Tytthaspis_sedecimpunctata_592 | 0.188 |
| Brachiacantha_rotunda_633 | Tytthaspis_sedecimpunctata_592 | 0.188 |
| Brachiacantha_rotunda_635 | Tytthaspis_sedecimpunctata_592 | 0.188 |
| Brachiacantha_rotunda_636 | Tytthaspis_sedecimpunctata_592 | 0.188 |
| Scymnus_interruptus_760 | Tytthaspis_sedecimpunctata_593 | 0.188 |
| Scymnus_interruptus_765 | Tytthaspis_sedecimpunctata_593 | 0.188 |
| Scymnus_interruptus_768 | Tytthaspis_sedecimpunctata_593 | 0.188 |
| Brachiacantha_rotunda_632 | Tytthaspis_sedecimpunctata_594 | 0.188 |
| Brachiacantha_rotunda_633 | Tytthaspis_sedecimpunctata_594 | 0.188 |
| Brachiacantha_rotunda_635 | Tytthaspis_sedecimpunctata_594 | 0.188 |
| Brachiacantha_rotunda_636 | Tytthaspis_sedecimpunctata_594 | 0.188 |
| Scymnus_interruptus_760 | Tytthaspis_sedecimpunctata_594 | 0.188 |
| Scymnus_interruptus_765 | Tytthaspis_sedecimpunctata_594 | 0.188 |
| Scymnus_interruptus_768 | Tytthaspis_sedecimpunctata_594 | 0.188 |
| Brachiacantha_rotunda_632 | Tytthaspis_sedecimpunctata_595 | 0.188 |
| Brachiacantha_rotunda_633 | Tytthaspis_sedecimpunctata_595 | 0.188 |
| Brachiacantha_rotunda_635 | Tytthaspis_sedecimpunctata_595 | 0.188 |
| Brachiacantha_rotunda_636 | Tytthaspis_sedecimpunctata_595 | 0.188 |
| Scymnus_interruptus_760 | Tytthaspis_sedecimpunctata_595 | 0.188 |
| Scymnus_interruptus_765 | Tytthaspis_sedecimpunctata_595 | 0.188 |
| Scymnus_interruptus_768 | Tytthaspis_sedecimpunctata_595 | 0.188 |
| Chilocorus_bipustulatus_18 | Scymnus_sinuanodulus_828 | 0.188 |
| Chilocorus_bipustulatus_19 | Scymnus_sinuanodulus_828 | 0.188 |
| Chilocorus_bipustulatus_20 | Scymnus_sinuanodulus_828 | 0.188 |
| Chilocorus_bipustulatus_22 | Scymnus_sinuanodulus_828 | 0.188 |

| | | |
|---|---|---|
| Chilocorus_bipustulatus_24 | Scymnus_sinuanodulus_828 | 0.188 |
| Anatis_mali_128 | Scymnus_suturalis_832 | 0.188 |
| Anatis_mali_129 | Scymnus_suturalis_832 | 0.188 |
| Anatis_mali_130 | Scymnus_suturalis_832 | 0.188 |
| Anatis_mali_131 | Scymnus_suturalis_832 | 0.188 |
| Anatis_mali_128 | Scymnus_suturalis_835 | 0.188 |
| Anatis_mali_129 | Scymnus_suturalis_835 | 0.188 |
| Anatis_mali_130 | Scymnus_suturalis_835 | 0.188 |
| Anatis_mali_131 | Scymnus_suturalis_835 | 0.188 |
| Anatis_mali_128 | Scymnus_suturalis_836 | 0.188 |
| Anatis_mali_129 | Scymnus_suturalis_836 | 0.188 |
| Anatis_mali_130 | Scymnus_suturalis_836 | 0.188 |
| Anatis_mali_131 | Scymnus_suturalis_836 | 0.188 |
| Hyperaspis_binotata_649 | Scymnus_suturalis_838 | 0.188 |
| Anatis_mali_128 | Scymnus_suturalis_839 | 0.188 |
| Anatis_mali_129 | Scymnus_suturalis_839 | 0.188 |
| Anatis_mali_130 | Scymnus_suturalis_839 | 0.188 |
| Anatis_mali_131 | Scymnus_suturalis_839 | 0.188 |
| Hyperaspis_binotata_649 | Scymnus_suturalis_840 | 0.188 |
| Anatis_mali_128 | Scymnus_suturalis_841 | 0.188 |
| Anatis_mali_129 | Scymnus_suturalis_841 | 0.188 |
| Anatis_mali_130 | Scymnus_suturalis_841 | 0.188 |
| Anatis_mali_131 | Scymnus_suturalis_841 | 0.188 |
| Hyperaspis_binotata_649 | Subcoccinella_vigintiquatuorpunctata_626 | 0.188 |
| Scymnus_camptodromus_718 | Tytthaspis_sedecimpunctata_577 | 0.188 |
| Scymnus_camptodromus_718 | Tytthaspis_sedecimpunctata_578 | 0.188 |
| Scymnus_camptodromus_718 | Tytthaspis_sedecimpunctata_579 | 0.188 |
| Scymnus_camptodromus_705 | Tytthaspis_sedecimpunctata_580 | 0.188 |
| Scymnus_camptodromus_713 | Tytthaspis_sedecimpunctata_580 | 0.188 |
| Scymnus_camptodromus_716 | Tytthaspis_sedecimpunctata_580 | 0.188 |
| Scymnus_camptodromus_718 | Tytthaspis_sedecimpunctata_581 | 0.188 |
| Scymnus_camptodromus_718 | Tytthaspis_sedecimpunctata_582 | 0.188 |
| Brachiacantha_rotunda_632 | Tytthaspis_sedecimpunctata_586 | 0.188 |
| Brachiacantha_rotunda_633 | Tytthaspis_sedecimpunctata_586 | 0.188 |
| Brachiacantha_rotunda_635 | Tytthaspis_sedecimpunctata_586 | 0.188 |
| Brachiacantha_rotunda_636 | Tytthaspis_sedecimpunctata_586 | 0.188 |
| Psyllobora_renifer_550 | Tytthaspis_sedecimpunctata_586 | 0.188 |
| Scymnus_interruptus_760 | Tytthaspis_sedecimpunctata_586 | 0.188 |
| Scymnus_interruptus_765 | Tytthaspis_sedecimpunctata_586 | 0.188 |
| Scymnus_interruptus_768 | Tytthaspis_sedecimpunctata_586 | 0.188 |
| Scymnus_ferrugatus_724 | Tytthaspis_sedecimpunctata_588 | 0.188 |
| Scymnus_ferrugatus_729 | Tytthaspis_sedecimpunctata_588 | 0.188 |
| Scymnus_camptodromus_718 | Tytthaspis_sedecimpunctata_590 | 0.188 |
| Adalia_bipunctata_98 | Tytthaspis_sedecimpunctata_591 | 0.188 |
| Scymnus_camptodromus_718 | Tytthaspis_sedecimpunctata_592 | 0.188 |
| Scymnus_camptodromus_718 | Tytthaspis_sedecimpunctata_593 | 0.188 |
| Scymnus_camptodromus_718 | Tytthaspis_sedecimpunctata_594 | 0.188 |
| Scymnus_camptodromus_718 | Tytthaspis_sedecimpunctata_595 | 0.188 |
| Chilocorus_stigma_43 | Scymnus_sinuanodulus_828 | 0.188 |
| Hyperaspis_binotata_649 | Scymnus_suturalis_837 | 0.188 |
| Scymnus_frontalis_734 | Scymnus_suturalis_838 | 0.188 |
| Scymnus_frontalis_745 | Scymnus_suturalis_838 | 0.188 |
| Scymnus_frontalis_736 | Scymnus_suturalis_840 | 0.188 |
| Scymnus_frontalis_737 | Scymnus_suturalis_840 | 0.188 |
| Scymnus_frontalis_738 | Scymnus_suturalis_840 | 0.188 |
| Scymnus_frontalis_739 | Scymnus_suturalis_840 | 0.188 |

| | | |
|---|---|---|
| Scymnus_frontalis_740 | Scymnus_suturalis_840 | 0.188 |
| Scymnus_frontalis_741 | Scymnus_suturalis_840 | 0.188 |
| Scymnus_frontalis_743 | Scymnus_suturalis_840 | 0.188 |
| Scymnus_frontalis_744 | Scymnus_suturalis_840 | 0.188 |
| Brumus_quadripustulatus_11 | Scymnus_sinuanodulus_831 | 0.188 |
| Brumus_quadripustulatus_13 | Scymnus_sinuanodulus_831 | 0.188 |
| Brumus_quadripustulatus_14 | Scymnus_sinuanodulus_831 | 0.188 |
| Brumus_quadripustulatus_17 | Scymnus_sinuanodulus_831 | 0.188 |
| Brumus_quadripustulatus_4 | Scymnus_sinuanodulus_831 | 0.188 |
| Brumus_quadripustulatus_7 | Scymnus_sinuanodulus_831 | 0.188 |
| Macronaemia_episcopalis_478 | Scymnus_suturalis_841 | 0.188 |
| Macronaemia_episcopalis_478 | Scymnus_suturalis_842 | 0.188 |
| Macronaemia_episcopalis_478 | Scymnus_suturalis_844 | 0.188 |
| Macronaemia_episcopalis_478 | Scymnus_suturalis_846 | 0.188 |
| Macronaemia_episcopalis_478 | Scymnus_suturalis_847 | 0.188 |
| Macronaemia_episcopalis_478 | Scymnus_suturalis_848 | 0.188 |
| Brumus_quadripustulatus_15 | Scymnus_sinuanodulus_831 | 0.188 |
| Brumus_quadripustulatus_16 | Scymnus_sinuanodulus_831 | 0.188 |
| Brumus_quadripustulatus_6 | Scymnus_sinuanodulus_831 | 0.188 |
| Brumus_quadripustulatus_8 | Scymnus_sinuanodulus_831 | 0.188 |
| Macronaemia_episcopalis_478 | Scymnus_suturalis_832 | 0.188 |
| Macronaemia_episcopalis_478 | Scymnus_suturalis_835 | 0.188 |
| Macronaemia_episcopalis_478 | Scymnus_suturalis_836 | 0.188 |
| Macronaemia_episcopalis_478 | Scymnus_suturalis_839 | 0.188 |
| Scymnus_frontalis_736 | Scymnus_suturalis_841 | 0.188 |
| Scymnus_frontalis_737 | Scymnus_suturalis_841 | 0.188 |
| Scymnus_frontalis_738 | Scymnus_suturalis_841 | 0.188 |
| Scymnus_frontalis_739 | Scymnus_suturalis_841 | 0.188 |
| Scymnus_frontalis_740 | Scymnus_suturalis_841 | 0.188 |
| Scymnus_frontalis_741 | Scymnus_suturalis_841 | 0.188 |
| Scymnus_frontalis_743 | Scymnus_suturalis_841 | 0.188 |
| Scymnus_frontalis_744 | Scymnus_suturalis_841 | 0.188 |
| Scymnus_frontalis_736 | Scymnus_suturalis_842 | 0.188 |
| Scymnus_frontalis_737 | Scymnus_suturalis_842 | 0.188 |
| Scymnus_frontalis_738 | Scymnus_suturalis_842 | 0.188 |
| Scymnus_frontalis_739 | Scymnus_suturalis_842 | 0.188 |
| Scymnus_frontalis_740 | Scymnus_suturalis_842 | 0.188 |
| Scymnus_frontalis_741 | Scymnus_suturalis_842 | 0.188 |
| Scymnus_frontalis_743 | Scymnus_suturalis_842 | 0.188 |
| Scymnus_frontalis_744 | Scymnus_suturalis_842 | 0.188 |
| Scymnus_frontalis_742 | Scymnus_suturalis_843 | 0.188 |
| Scymnus_frontalis_736 | Scymnus_suturalis_844 | 0.188 |
| Scymnus_frontalis_737 | Scymnus_suturalis_844 | 0.188 |
| Scymnus_frontalis_738 | Scymnus_suturalis_844 | 0.188 |
| Scymnus_frontalis_739 | Scymnus_suturalis_844 | 0.188 |
| Scymnus_frontalis_740 | Scymnus_suturalis_844 | 0.188 |
| Scymnus_frontalis_741 | Scymnus_suturalis_844 | 0.188 |
| Scymnus_frontalis_743 | Scymnus_suturalis_844 | 0.188 |
| Scymnus_frontalis_744 | Scymnus_suturalis_844 | 0.188 |
| Scymnus_frontalis_742 | Scymnus_suturalis_845 | 0.188 |
| Scymnus_frontalis_736 | Scymnus_suturalis_846 | 0.188 |
| Scymnus_frontalis_737 | Scymnus_suturalis_846 | 0.188 |
| Scymnus_frontalis_738 | Scymnus_suturalis_846 | 0.188 |
| Scymnus_frontalis_739 | Scymnus_suturalis_846 | 0.188 |
| Scymnus_frontalis_740 | Scymnus_suturalis_846 | 0.188 |
| Scymnus_frontalis_741 | Scymnus_suturalis_846 | 0.188 |

| | | |
|---|---|---|
| Scymnus_frontalis_743 | Scymnus_suturalis_846 | 0.188 |
| Scymnus_frontalis_744 | Scymnus_suturalis_846 | 0.188 |
| Scymnus_frontalis_736 | Scymnus_suturalis_847 | 0.188 |
| Scymnus_frontalis_737 | Scymnus_suturalis_847 | 0.188 |
| Scymnus_frontalis_738 | Scymnus_suturalis_847 | 0.188 |
| Scymnus_frontalis_739 | Scymnus_suturalis_847 | 0.188 |
| Scymnus_frontalis_740 | Scymnus_suturalis_847 | 0.188 |
| Scymnus_frontalis_741 | Scymnus_suturalis_847 | 0.188 |
| Scymnus_frontalis_743 | Scymnus_suturalis_847 | 0.188 |
| Scymnus_frontalis_744 | Scymnus_suturalis_847 | 0.188 |
| Scymnus_frontalis_736 | Scymnus_suturalis_848 | 0.188 |
| Scymnus_frontalis_737 | Scymnus_suturalis_848 | 0.188 |
| Scymnus_frontalis_738 | Scymnus_suturalis_848 | 0.188 |
| Scymnus_frontalis_739 | Scymnus_suturalis_848 | 0.188 |
| Scymnus_frontalis_740 | Scymnus_suturalis_848 | 0.188 |
| Scymnus_frontalis_741 | Scymnus_suturalis_848 | 0.188 |
| Scymnus_frontalis_743 | Scymnus_suturalis_848 | 0.188 |
| Scymnus_frontalis_744 | Scymnus_suturalis_848 | 0.188 |
| Hyperaspis_lateralis_655 | Stethorus_punctillum_852 | 0.188 |
| Olla_vnigrum_527 | Tytthaspis_sedecimpunctata_580 | 0.188 |
| Olla_vnigrum_528 | Tytthaspis_sedecimpunctata_580 | 0.188 |
| Scymnus_camptodromus_719 | Tytthaspis_sedecimpunctata_583 | 0.188 |
| Olla_vnigrum_527 | Tytthaspis_sedecimpunctata_591 | 0.188 |
| Olla_vnigrum_528 | Tytthaspis_sedecimpunctata_591 | 0.188 |
| Coccinella_undecimpunctata_285 | Tytthaspis_sedecimpunctata_593 | 0.188 |
| Coccinella_undecimpunctata_286 | Tytthaspis_sedecimpunctata_593 | 0.188 |
| Coccinella_undecimpunctata_288 | Tytthaspis_sedecimpunctata_593 | 0.188 |
| Chilocorus_nigritus_27 | Scymnus_sinuanodulus_828 | 0.188 |
| Scymnus_frontalis_736 | Scymnus_suturalis_832 | 0.188 |
| Scymnus_frontalis_737 | Scymnus_suturalis_832 | 0.188 |
| Scymnus_frontalis_738 | Scymnus_suturalis_832 | 0.188 |
| Scymnus_frontalis_739 | Scymnus_suturalis_832 | 0.188 |
| Scymnus_frontalis_740 | Scymnus_suturalis_832 | 0.188 |
| Scymnus_frontalis_741 | Scymnus_suturalis_832 | 0.188 |
| Scymnus_frontalis_743 | Scymnus_suturalis_832 | 0.188 |
| Scymnus_frontalis_744 | Scymnus_suturalis_832 | 0.188 |
| Scymnus_frontalis_734 | Scymnus_suturalis_834 | 0.188 |
| Scymnus_frontalis_745 | Scymnus_suturalis_834 | 0.188 |
| Scymnus_frontalis_736 | Scymnus_suturalis_835 | 0.188 |
| Scymnus_frontalis_737 | Scymnus_suturalis_835 | 0.188 |
| Scymnus_frontalis_738 | Scymnus_suturalis_835 | 0.188 |
| Scymnus_frontalis_739 | Scymnus_suturalis_835 | 0.188 |
| Scymnus_frontalis_740 | Scymnus_suturalis_835 | 0.188 |
| Scymnus_frontalis_741 | Scymnus_suturalis_835 | 0.188 |
| Scymnus_frontalis_743 | Scymnus_suturalis_835 | 0.188 |
| Scymnus_frontalis_744 | Scymnus_suturalis_835 | 0.188 |
| Scymnus_frontalis_736 | Scymnus_suturalis_836 | 0.188 |
| Scymnus_frontalis_737 | Scymnus_suturalis_836 | 0.188 |
| Scymnus_frontalis_738 | Scymnus_suturalis_836 | 0.188 |
| Scymnus_frontalis_739 | Scymnus_suturalis_836 | 0.188 |
| Scymnus_frontalis_740 | Scymnus_suturalis_836 | 0.188 |
| Scymnus_frontalis_741 | Scymnus_suturalis_836 | 0.188 |
| Scymnus_frontalis_743 | Scymnus_suturalis_836 | 0.188 |
| Scymnus_frontalis_744 | Scymnus_suturalis_836 | 0.188 |
| Scymnus_frontalis_742 | Scymnus_suturalis_837 | 0.188 |
| Hyperaspis_binotata_650 | Scymnus_suturalis_838 | 0.188 |

| | | |
|---|---|---|
| Scymnus_frontalis_736 | Scymnus_suturalis_839 | 0.188 |
| Scymnus_frontalis_737 | Scymnus_suturalis_839 | 0.188 |
| Scymnus_frontalis_738 | Scymnus_suturalis_839 | 0.188 |
| Scymnus_frontalis_739 | Scymnus_suturalis_839 | 0.188 |
| Scymnus_frontalis_740 | Scymnus_suturalis_839 | 0.188 |
| Scymnus_frontalis_741 | Scymnus_suturalis_839 | 0.188 |
| Scymnus_frontalis_743 | Scymnus_suturalis_839 | 0.188 |
| Scymnus_frontalis_744 | Scymnus_suturalis_839 | 0.188 |
| Hyperaspis_binotata_650 | Scymnus_suturalis_840 | 0.188 |
| Hyperaspis_proba_660 | Stethorus_punctillum_850 | 0.188 |
| Hyperaspis_proba_660 | Stethorus_punctillum_854 | 0.188 |
| Hyperaspis_proba_660 | Stethorus_punctillum_856 | 0.188 |
| Hippodamia_parenthesis_432 | Scymnus_suturalis_843 | 0.188 |
| Hippodamia_parenthesis_434 | Scymnus_suturalis_843 | 0.188 |
| Hippodamia_parenthesis_435 | Scymnus_suturalis_843 | 0.188 |
| Hippodamia_parenthesis_437 | Scymnus_suturalis_843 | 0.188 |
| Hippodamia_parenthesis_438 | Scymnus_suturalis_843 | 0.188 |
| Hippodamia_parenthesis_441 | Scymnus_suturalis_843 | 0.188 |
| Hippodamia_parenthesis_442 | Scymnus_suturalis_843 | 0.188 |
| Hyperaspis_lateralis_656 | Stethorus_punctillum_855 | 0.188 |
| Scymnus_nubilus_796 | Subcoccinella_vigintiquatuorpunctata_622 | 0.188 |
| Scymnus_nubilus_803 | Subcoccinella_vigintiquatuorpunctata_622 | 0.188 |
| Scymnus_nubilus_804 | Subcoccinella_vigintiquatuorpunctata_622 | 0.188 |
| Scymnus_nubilus_809 | Subcoccinella_vigintiquatuorpunctata_622 | 0.188 |
| Coccidula_scutellata_77 | Subcoccinella_vigintiquatuorpunctata_623 | 0.188 |
| Scymnus_nubilus_796 | Subcoccinella_vigintiquatuorpunctata_624 | 0.188 |
| Scymnus_nubilus_796 | Subcoccinella_vigintiquatuorpunctata_625 | 0.188 |
| Scymnus_nubilus_803 | Subcoccinella_vigintiquatuorpunctata_625 | 0.188 |
| Scymnus_nubilus_804 | Subcoccinella_vigintiquatuorpunctata_625 | 0.188 |
| Scymnus_nubilus_809 | Subcoccinella_vigintiquatuorpunctata_625 | 0.188 |
| Hyperaspis_binotata_650 | Subcoccinella_vigintiquatuorpunctata_626 | 0.188 |
| Scymnus_nubilus_800 | Subcoccinella_vigintiquatuorpunctata_627 | 0.188 |
| Scymnus_nubilus_801 | Subcoccinella_vigintiquatuorpunctata_627 | 0.188 |
| Scymnus_nubilus_802 | Subcoccinella_vigintiquatuorpunctata_627 | 0.188 |
| Scymnus_nubilus_805 | Subcoccinella_vigintiquatuorpunctata_627 | 0.188 |
| Scymnus_nubilus_806 | Subcoccinella_vigintiquatuorpunctata_627 | 0.188 |
| Scymnus_nubilus_807 | Subcoccinella_vigintiquatuorpunctata_627 | 0.188 |
| Scymnus_nubilus_810 | Subcoccinella_vigintiquatuorpunctata_627 | 0.188 |
| Scymnus_nubilus_796 | Subcoccinella_vigintiquatuorpunctata_630 | 0.188 |
| Scymnus_nubilus_803 | Subcoccinella_vigintiquatuorpunctata_630 | 0.188 |
| Scymnus_nubilus_804 | Subcoccinella_vigintiquatuorpunctata_630 | 0.188 |
| Scymnus_nubilus_809 | Subcoccinella_vigintiquatuorpunctata_630 | 0.188 |
| Scymnus_auritus_701 | Tytthaspis_sedecimpunctata_577 | 0.188 |
| Scymnus_auritus_701 | Tytthaspis_sedecimpunctata_578 | 0.188 |
| Scymnus_auritus_701 | Tytthaspis_sedecimpunctata_579 | 0.188 |
| Scymnus_camptodromus_719 | Tytthaspis_sedecimpunctata_580 | 0.188 |
| Scymnus_auritus_701 | Tytthaspis_sedecimpunctata_581 | 0.188 |
| Scymnus_auritus_701 | Tytthaspis_sedecimpunctata_582 | 0.188 |
| Scymnus_camptodromus_707 | Tytthaspis_sedecimpunctata_584 | 0.188 |
| Scymnus_camptodromus_708 | Tytthaspis_sedecimpunctata_584 | 0.188 |
| Scymnus_camptodromus_709 | Tytthaspis_sedecimpunctata_584 | 0.188 |
| Scymnus_camptodromus_710 | Tytthaspis_sedecimpunctata_584 | 0.188 |
| Olla_vnigrum_527 | Tytthaspis_sedecimpunctata_585 | 0.188 |
| Olla_vnigrum_528 | Tytthaspis_sedecimpunctata_585 | 0.188 |
| Scymnus_camptodromus_719 | Tytthaspis_sedecimpunctata_587 | 0.188 |
| Scymnus_auritus_701 | Tytthaspis_sedecimpunctata_590 | 0.188 |

| | | |
|---|---|---|
| Olla_vnigrum_527 | Tytthaspis_sedecimpunctata_593 | 0.188 |
| Olla_vnigrum_528 | Tytthaspis_sedecimpunctata_593 | 0.188 |
| Scymnus_auritus_701 | Tytthaspis_sedecimpunctata_593 | 0.188 |
| Scymnus_auritus_701 | Tytthaspis_sedecimpunctata_594 | 0.188 |
| Scymnus_auritus_701 | Tytthaspis_sedecimpunctata_595 | 0.188 |
| Scymnus_auritus_701 | Scymnus_rubromaculatus_822 | 0.188 |
| Scymnus_auritus_701 | Scymnus_rubromaculatus_823 | 0.188 |
| Scymnus_auritus_701 | Scymnus_rubromaculatus_824 | 0.188 |
| Scymnus_auritus_701 | Scymnus_rubromaculatus_825 | 0.188 |
| Scymnus_auritus_701 | Scymnus_rubromaculatus_826 | 0.188 |
| Scymnus_auritus_701 | Scymnus_rubromaculatus_827 | 0.188 |
| Hippodamia_parenthesis_432 | Scymnus_suturalis_834 | 0.188 |
| Hippodamia_parenthesis_434 | Scymnus_suturalis_834 | 0.188 |
| Hippodamia_parenthesis_435 | Scymnus_suturalis_834 | 0.188 |
| Hippodamia_parenthesis_437 | Scymnus_suturalis_834 | 0.188 |
| Hippodamia_parenthesis_438 | Scymnus_suturalis_834 | 0.188 |
| Hippodamia_parenthesis_441 | Scymnus_suturalis_834 | 0.188 |
| Hippodamia_parenthesis_442 | Scymnus_suturalis_834 | 0.188 |
| Hyperaspis_binotata_650 | Scymnus_suturalis_837 | 0.188 |
| Exochomus_nigromaculatus_48 | Scymnus_suturalis_838 | 0.188 |
| Exochomus_nigromaculatus_48 | Scymnus_suturalis_840 | 0.188 |
| Scymnus_apetzi_692 | Subcoccinella_vigintiquatuorpunctata_620 | 0.188 |
| Scymnus_apetzi_692 | Subcoccinella_vigintiquatuorpunctata_621 | 0.188 |
| Scymnus_apetzi_692 | Subcoccinella_vigintiquatuorpunctata_628 | 0.188 |
| Scymnus_apetzi_692 | Subcoccinella_vigintiquatuorpunctata_629 | 0.188 |
| Scymnus_apetzi_692 | Subcoccinella_vigintiquatuorpunctata_631 | 0.188 |
| Hyperaspis_proba_660 | Stethorus_punctillum_855 | 0.188 |
| Scymnus_nigrinus_787 | Tytthaspis_sedecimpunctata_577 | 0.188 |
| Scymnus_nigrinus_787 | Tytthaspis_sedecimpunctata_578 | 0.188 |
| Scymnus_nigrinus_787 | Tytthaspis_sedecimpunctata_579 | 0.188 |
| Scymnus_nigrinus_787 | Tytthaspis_sedecimpunctata_581 | 0.188 |
| Scymnus_nigrinus_787 | Tytthaspis_sedecimpunctata_582 | 0.188 |
| Scymnus_nigrinus_787 | Tytthaspis_sedecimpunctata_590 | 0.188 |
| Scymnus_nigrinus_787 | Tytthaspis_sedecimpunctata_593 | 0.188 |
| Scymnus_nigrinus_787 | Tytthaspis_sedecimpunctata_594 | 0.188 |
| Scymnus_nigrinus_787 | Tytthaspis_sedecimpunctata_595 | 0.188 |
| Hippodamia_parenthesis_439 | Scymnus_suturalis_841 | 0.188 |
| Hippodamia_parenthesis_439 | Scymnus_suturalis_842 | 0.188 |
| Hippodamia_parenthesis_439 | Scymnus_suturalis_844 | 0.188 |
| Hippodamia_parenthesis_439 | Scymnus_suturalis_846 | 0.188 |
| Hippodamia_parenthesis_439 | Scymnus_suturalis_847 | 0.188 |
| Hippodamia_parenthesis_439 | Scymnus_suturalis_848 | 0.188 |
| Hyperaspis_lateralis_655 | Stethorus_punctillum_855 | 0.188 |
| Scymnus_nubilus_799 | Subcoccinella_vigintiquatuorpunctata_620 | 0.188 |
| Scymnus_nubilus_799 | Subcoccinella_vigintiquatuorpunctata_621 | 0.188 |
| Coccidula_scutellata_81 | Subcoccinella_vigintiquatuorpunctata_623 | 0.188 |
| Coccidula_scutellata_82 | Subcoccinella_vigintiquatuorpunctata_623 | 0.188 |
| Coccidula_scutellata_77 | Subcoccinella_vigintiquatuorpunctata_626 | 0.188 |
| Scymnus_nubilus_799 | Subcoccinella_vigintiquatuorpunctata_628 | 0.188 |
| Scymnus_nubilus_799 | Subcoccinella_vigintiquatuorpunctata_629 | 0.188 |
| Scymnus_nubilus_799 | Subcoccinella_vigintiquatuorpunctata_631 | 0.188 |
| Calvia_quatuordecimguttata_211 | Scymnus_sinuanodulus_828 | 0.188 |
| Calvia_quatuordecimguttata_212 | Scymnus_sinuanodulus_828 | 0.188 |
| Calvia_quatuordecimguttata_213 | Scymnus_sinuanodulus_828 | 0.188 |
| Calvia_quatuordecimguttata_214 | Scymnus_sinuanodulus_828 | 0.188 |
| Calvia_quatuordecimguttata_215 | Scymnus_sinuanodulus_828 | 0.188 |

| | | |
|---|---|---|
| Hippodamia_parenthesis_439 | Scymnus_suturalis_832 | 0.188 |
| Henosepilachna_argus_616 | Scymnus_suturalis_833 | 0.188 |
| Exochomus_nigromaculatus_48 | Scymnus_suturalis_834 | 0.188 |
| Hippodamia_parenthesis_439 | Scymnus_suturalis_835 | 0.188 |
| Hippodamia_parenthesis_439 | Scymnus_suturalis_836 | 0.188 |
| Exochomus_nigromaculatus_48 | Scymnus_suturalis_837 | 0.188 |
| Hippodamia_parenthesis_439 | Scymnus_suturalis_837 | 0.188 |
| Henosepilachna_argus_616 | Scymnus_suturalis_838 | 0.188 |
| Hippodamia_parenthesis_439 | Scymnus_suturalis_839 | 0.188 |
| Henosepilachna_argus_616 | Scymnus_suturalis_840 | 0.188 |
| Scymnus_nigrinus_787 | Tytthaspis_sedecimpunctata_584 | 0.188 |
| Chilocorus_nigritus_29 | Scymnus_sinuanodulus_828 | 0.188 |
| Scymnus_frontalis_735 | Scymnus_suturalis_838 | 0.188 |
| Henosepilachna_argus_615 | Scymnus_suturalis_841 | 0.188 |
| Henosepilachna_argus_617 | Scymnus_suturalis_841 | 0.188 |
| Henosepilachna_argus_618 | Scymnus_suturalis_841 | 0.188 |
| Henosepilachna_argus_615 | Scymnus_suturalis_842 | 0.188 |
| Henosepilachna_argus_617 | Scymnus_suturalis_842 | 0.188 |
| Henosepilachna_argus_618 | Scymnus_suturalis_842 | 0.188 |
| Henosepilachna_argus_615 | Scymnus_suturalis_844 | 0.188 |
| Henosepilachna_argus_617 | Scymnus_suturalis_844 | 0.188 |
| Henosepilachna_argus_618 | Scymnus_suturalis_844 | 0.188 |
| Henosepilachna_argus_615 | Scymnus_suturalis_846 | 0.188 |
| Henosepilachna_argus_617 | Scymnus_suturalis_846 | 0.188 |
| Henosepilachna_argus_618 | Scymnus_suturalis_846 | 0.188 |
| Henosepilachna_argus_615 | Scymnus_suturalis_847 | 0.188 |
| Henosepilachna_argus_617 | Scymnus_suturalis_847 | 0.188 |
| Henosepilachna_argus_618 | Scymnus_suturalis_847 | 0.188 |
| Henosepilachna_argus_615 | Scymnus_suturalis_848 | 0.188 |
| Henosepilachna_argus_617 | Scymnus_suturalis_848 | 0.188 |
| Henosepilachna_argus_618 | Scymnus_suturalis_848 | 0.188 |
| Rhyzobius_litura_96 | Subcoccinella_vigintiquatuorpunctata_622 | 0.188 |
| Scymnus_haemorrhoidalis_755 | Subcoccinella_vigintiquatuorpunctata_623 | 0.188 |
| Scymnus_haemorrhoidalis_756 | Subcoccinella_vigintiquatuorpunctata_623 | 0.188 |
| Coccidula_scutellata_81 | Subcoccinella_vigintiquatuorpunctata_626 | 0.188 |
| Coccidula_scutellata_82 | Subcoccinella_vigintiquatuorpunctata_626 | 0.188 |
| Coccidula_scutellata_80 | Subcoccinella_vigintiquatuorpunctata_627 | 0.188 |
| Coccidula_scutellata_80 | Subcoccinella_vigintiquatuorpunctata_630 | 0.188 |
| Hippodamia_undecimnotata_460 | Tytthaspis_sedecimpunctata_583 | 0.188 |
| Hippodamia_undecimnotata_461 | Tytthaspis_sedecimpunctata_583 | 0.188 |
| Hippodamia_undecimnotata_462 | Tytthaspis_sedecimpunctata_583 | 0.188 |
| Hippodamia_undecimnotata_463 | Tytthaspis_sedecimpunctata_583 | 0.188 |
| Hippodamia_undecimnotata_464 | Tytthaspis_sedecimpunctata_583 | 0.188 |
| Hippodamia_undecimnotata_465 | Tytthaspis_sedecimpunctata_583 | 0.188 |
| Hippodamia_undecimnotata_466 | Tytthaspis_sedecimpunctata_583 | 0.188 |
| Coccinella_transversoguttata_267 | Vibidia_duodecimguttata_597 | 0.188 |
| Coccinella_transversoguttata_268 | Vibidia_duodecimguttata_597 | 0.188 |
| Coccinella_transversoguttata_269 | Vibidia_duodecimguttata_597 | 0.188 |
| Coccinella_transversoguttata_270 | Vibidia_duodecimguttata_597 | 0.188 |
| Coccinella_transversoguttata_271 | Vibidia_duodecimguttata_597 | 0.188 |
| Coccinella_transversoguttata_267 | Vibidia_duodecimguttata_600 | 0.188 |
| Coccinella_transversoguttata_268 | Vibidia_duodecimguttata_600 | 0.188 |
| Coccinella_transversoguttata_269 | Vibidia_duodecimguttata_600 | 0.188 |
| Coccinella_transversoguttata_270 | Vibidia_duodecimguttata_600 | 0.188 |
| Coccinella_transversoguttata_271 | Vibidia_duodecimguttata_600 | 0.188 |
| Calvia_quatuordecimguttata_207 | Scymnus_sinuanodulus_828 | 0.188 |

| | | |
|---|---|---|
| Calvia_quatuordecimguttata_201 | Scymnus_sinuanodulus_830 | 0.188 |
| Henosepilachna_argus_615 | Scymnus_suturalis_832 | 0.188 |
| Henosepilachna_argus_617 | Scymnus_suturalis_832 | 0.188 |
| Henosepilachna_argus_618 | Scymnus_suturalis_832 | 0.188 |
| Brumoides_suturalis_2 | Scymnus_suturalis_833 | 0.188 |
| Henosepilachna_argus_616 | Scymnus_suturalis_834 | 0.188 |
| Henosepilachna_argus_615 | Scymnus_suturalis_835 | 0.188 |
| Henosepilachna_argus_617 | Scymnus_suturalis_835 | 0.188 |
| Henosepilachna_argus_618 | Scymnus_suturalis_835 | 0.188 |
| Henosepilachna_argus_615 | Scymnus_suturalis_836 | 0.188 |
| Henosepilachna_argus_617 | Scymnus_suturalis_836 | 0.188 |
| Henosepilachna_argus_618 | Scymnus_suturalis_836 | 0.188 |
| Henosepilachna_argus_616 | Scymnus_suturalis_837 | 0.188 |
| Henosepilachna_argus_615 | Scymnus_suturalis_839 | 0.188 |
| Henosepilachna_argus_617 | Scymnus_suturalis_839 | 0.188 |
| Henosepilachna_argus_618 | Scymnus_suturalis_839 | 0.188 |
| Coelophora_inaequalis_319 | Vibidia_duodecimguttata_597 | 0.188 |
| Coelophora_inaequalis_319 | Vibidia_duodecimguttata_600 | 0.188 |
| Chilocorus_nigritus_28 | Scymnus_sinuanodulus_828 | 0.188 |
| Scymnus_frontalis_735 | Scymnus_suturalis_834 | 0.188 |
| Hippodamia_convergens_400 | Scymnus_suturalis_843 | 0.188 |
| Hippodamia_convergens_400 | Scymnus_suturalis_845 | 0.188 |
| Scymnus_haemorrhoidalis_747 | Subcoccinella_vigintiquatuorpunctata_622 | 0.188 |
| Scymnus_haemorrhoidalis_748 | Subcoccinella_vigintiquatuorpunctata_622 | 0.188 |
| Scymnus_haemorrhoidalis_755 | Subcoccinella_vigintiquatuorpunctata_624 | 0.188 |
| Scymnus_haemorrhoidalis_756 | Subcoccinella_vigintiquatuorpunctata_624 | 0.188 |
| Scymnus_haemorrhoidalis_755 | Subcoccinella_vigintiquatuorpunctata_626 | 0.188 |
| Scymnus_haemorrhoidalis_756 | Subcoccinella_vigintiquatuorpunctata_626 | 0.188 |
| Hippodamia_undecimnotata_460 | Tytthaspis_sedecimpunctata_591 | 0.188 |
| Hippodamia_undecimnotata_461 | Tytthaspis_sedecimpunctata_591 | 0.188 |
| Hippodamia_undecimnotata_462 | Tytthaspis_sedecimpunctata_591 | 0.188 |
| Hippodamia_undecimnotata_463 | Tytthaspis_sedecimpunctata_591 | 0.188 |
| Hippodamia_undecimnotata_464 | Tytthaspis_sedecimpunctata_591 | 0.188 |
| Hippodamia_undecimnotata_465 | Tytthaspis_sedecimpunctata_591 | 0.188 |
| Hippodamia_undecimnotata_466 | Tytthaspis_sedecimpunctata_591 | 0.188 |
| Aphidecta_obliterata_183 | Vibidia_duodecimguttata_597 | 0.188 |
| Coccinella_transversoguttata_267 | Vibidia_duodecimguttata_598 | 0.188 |
| Coccinella_transversoguttata_268 | Vibidia_duodecimguttata_598 | 0.188 |
| Coccinella_transversoguttata_269 | Vibidia_duodecimguttata_598 | 0.188 |
| Coccinella_transversoguttata_270 | Vibidia_duodecimguttata_598 | 0.188 |
| Coccinella_transversoguttata_271 | Vibidia_duodecimguttata_598 | 0.188 |
| Aphidecta_obliterata_183 | Vibidia_duodecimguttata_600 | 0.188 |
| Hyperaspis_binotata_650 | Scymnus_sinuanodulus_830 | 0.188 |
| Hippodamia_parenthesis_436 | Scymnus_suturalis_841 | 0.188 |
| Hippodamia_parenthesis_436 | Scymnus_suturalis_842 | 0.188 |
| Hippodamia_parenthesis_436 | Scymnus_suturalis_844 | 0.188 |
| Hippodamia_parenthesis_436 | Scymnus_suturalis_846 | 0.188 |
| Hippodamia_parenthesis_436 | Scymnus_suturalis_847 | 0.188 |
| Hippodamia_parenthesis_436 | Scymnus_suturalis_848 | 0.188 |
| Coccinella_undecimpunctata_285 | Scymnus_sinuanodulus_831 | 0.188 |
| Coccinella_undecimpunctata_286 | Scymnus_sinuanodulus_831 | 0.188 |
| Coccinella_undecimpunctata_288 | Scymnus_sinuanodulus_831 | 0.188 |
| Hippodamia_parenthesis_436 | Scymnus_suturalis_832 | 0.188 |
| Hippodamia_parenthesis_436 | Scymnus_suturalis_834 | 0.188 |
| Hippodamia_parenthesis_436 | Scymnus_suturalis_835 | 0.188 |
| Hippodamia_parenthesis_436 | Scymnus_suturalis_836 | 0.188 |

Supplementary Table S4 (continued)

| | | |
|---|---|---|
| Hippodamia_parenthesis_436 | Scymnus_suturalis_837 | 0.188 |
| Hippodamia_parenthesis_436 | Scymnus_suturalis_839 | 0.188 |
| Psyllobora_vigintimaculata_573 | Vibidia_duodecimguttata_599 | 0.188 |
| Psyllobora_vigintimaculata_573 | Vibidia_duodecimguttata_601 | 0.188 |
| Psyllobora_vigintimaculata_573 | Vibidia_duodecimguttata_602 | 0.188 |
| Coccinella_undecimpunctata_287 | Vibidia_duodecimguttata_596 | 0.188 |
| Coccinella_undecimpunctata_287 | Vibidia_duodecimguttata_603 | 0.188 |
| Scymnus_nubilus_796 | Scymnus_suturalis_841 | 0.188 |
| Scymnus_nubilus_798 | Scymnus_suturalis_841 | 0.188 |
| Scymnus_nubilus_800 | Scymnus_suturalis_841 | 0.188 |
| Scymnus_nubilus_801 | Scymnus_suturalis_841 | 0.188 |
| Scymnus_nubilus_802 | Scymnus_suturalis_841 | 0.188 |
| Scymnus_nubilus_805 | Scymnus_suturalis_841 | 0.188 |
| Scymnus_nubilus_806 | Scymnus_suturalis_841 | 0.188 |
| Scymnus_nubilus_807 | Scymnus_suturalis_841 | 0.188 |
| Scymnus_nubilus_810 | Scymnus_suturalis_841 | 0.188 |
| Scymnus_nubilus_796 | Scymnus_suturalis_842 | 0.188 |
| Scymnus_nubilus_798 | Scymnus_suturalis_842 | 0.188 |
| Scymnus_nubilus_800 | Scymnus_suturalis_842 | 0.188 |
| Scymnus_nubilus_801 | Scymnus_suturalis_842 | 0.188 |
| Scymnus_nubilus_802 | Scymnus_suturalis_842 | 0.188 |
| Scymnus_nubilus_805 | Scymnus_suturalis_842 | 0.188 |
| Scymnus_nubilus_806 | Scymnus_suturalis_842 | 0.188 |
| Scymnus_nubilus_807 | Scymnus_suturalis_842 | 0.188 |
| Scymnus_nubilus_810 | Scymnus_suturalis_842 | 0.188 |
| Scymnus_nubilus_803 | Scymnus_suturalis_843 | 0.188 |
| Scymnus_nubilus_804 | Scymnus_suturalis_843 | 0.188 |
| Scymnus_nubilus_808 | Scymnus_suturalis_843 | 0.188 |
| Scymnus_nubilus_809 | Scymnus_suturalis_843 | 0.188 |
| Scymnus_nubilus_796 | Scymnus_suturalis_844 | 0.188 |
| Scymnus_nubilus_798 | Scymnus_suturalis_844 | 0.188 |
| Scymnus_nubilus_800 | Scymnus_suturalis_844 | 0.188 |
| Scymnus_nubilus_801 | Scymnus_suturalis_844 | 0.188 |
| Scymnus_nubilus_802 | Scymnus_suturalis_844 | 0.188 |
| Scymnus_nubilus_805 | Scymnus_suturalis_844 | 0.188 |
| Scymnus_nubilus_806 | Scymnus_suturalis_844 | 0.188 |
| Scymnus_nubilus_807 | Scymnus_suturalis_844 | 0.188 |
| Scymnus_nubilus_810 | Scymnus_suturalis_844 | 0.188 |
| Scymnus_nubilus_803 | Scymnus_suturalis_845 | 0.188 |
| Scymnus_nubilus_804 | Scymnus_suturalis_845 | 0.188 |
| Scymnus_nubilus_808 | Scymnus_suturalis_845 | 0.188 |
| Scymnus_nubilus_809 | Scymnus_suturalis_845 | 0.188 |
| Scymnus_nubilus_796 | Scymnus_suturalis_846 | 0.188 |
| Scymnus_nubilus_798 | Scymnus_suturalis_846 | 0.188 |
| Scymnus_nubilus_800 | Scymnus_suturalis_846 | 0.188 |
| Scymnus_nubilus_801 | Scymnus_suturalis_846 | 0.188 |
| Scymnus_nubilus_802 | Scymnus_suturalis_846 | 0.188 |
| Scymnus_nubilus_805 | Scymnus_suturalis_846 | 0.188 |
| Scymnus_nubilus_806 | Scymnus_suturalis_846 | 0.188 |
| Scymnus_nubilus_807 | Scymnus_suturalis_846 | 0.188 |
| Scymnus_nubilus_810 | Scymnus_suturalis_846 | 0.188 |
| Scymnus_nubilus_796 | Scymnus_suturalis_847 | 0.188 |
| Scymnus_nubilus_798 | Scymnus_suturalis_847 | 0.188 |
| Scymnus_nubilus_800 | Scymnus_suturalis_847 | 0.188 |
| Scymnus_nubilus_801 | Scymnus_suturalis_847 | 0.188 |
| Scymnus_nubilus_802 | Scymnus_suturalis_847 | 0.188 |

| | | |
|---|---|---|
| Scymnus_nubilus_805 | Scymnus_suturalis_847 | 0.188 |
| Scymnus_nubilus_806 | Scymnus_suturalis_847 | 0.188 |
| Scymnus_nubilus_807 | Scymnus_suturalis_847 | 0.188 |
| Scymnus_nubilus_810 | Scymnus_suturalis_847 | 0.188 |
| Scymnus_nubilus_796 | Scymnus_suturalis_848 | 0.188 |
| Scymnus_nubilus_798 | Scymnus_suturalis_848 | 0.188 |
| Scymnus_nubilus_800 | Scymnus_suturalis_848 | 0.188 |
| Scymnus_nubilus_801 | Scymnus_suturalis_848 | 0.188 |
| Scymnus_nubilus_802 | Scymnus_suturalis_848 | 0.188 |
| Scymnus_nubilus_805 | Scymnus_suturalis_848 | 0.188 |
| Scymnus_nubilus_806 | Scymnus_suturalis_848 | 0.188 |
| Scymnus_nubilus_807 | Scymnus_suturalis_848 | 0.188 |
| Scymnus_nubilus_810 | Scymnus_suturalis_848 | 0.188 |
| Scymnus_haemorrhoidalis_749 | Subcoccinella_vigintiquatuorpunctata_622 | 0.188 |
| Scymnus_haemorrhoidalis_750 | Subcoccinella_vigintiquatuorpunctata_622 | 0.188 |
| Scymnus_haemorrhoidalis_751 | Subcoccinella_vigintiquatuorpunctata_624 | 0.188 |
| Scymnus_haemorrhoidalis_754 | Subcoccinella_vigintiquatuorpunctata_624 | 0.188 |
| Scymnus_haemorrhoidalis_757 | Subcoccinella_vigintiquatuorpunctata_624 | 0.188 |
| Scymnus_haemorrhoidalis_758 | Subcoccinella_vigintiquatuorpunctata_624 | 0.188 |
| Aphidecta_obliterata_185 | Vibidia_duodecimguttata_596 | 0.188 |
| Aphidecta_obliterata_186 | Vibidia_duodecimguttata_596 | 0.188 |
| Coccinella_magnifica_228 | Vibidia_duodecimguttata_597 | 0.188 |
| Coccinella_magnifica_229 | Vibidia_duodecimguttata_597 | 0.188 |
| Coccinella_magnifica_230 | Vibidia_duodecimguttata_597 | 0.188 |
| Coccinella_magnifica_231 | Vibidia_duodecimguttata_597 | 0.188 |
| Aphidecta_obliterata_185 | Vibidia_duodecimguttata_598 | 0.188 |
| Aphidecta_obliterata_186 | Vibidia_duodecimguttata_598 | 0.188 |
| Coccinella_magnifica_228 | Vibidia_duodecimguttata_600 | 0.188 |
| Coccinella_magnifica_229 | Vibidia_duodecimguttata_600 | 0.188 |
| Coccinella_magnifica_230 | Vibidia_duodecimguttata_600 | 0.188 |
| Coccinella_magnifica_231 | Vibidia_duodecimguttata_600 | 0.188 |
| Aphidecta_obliterata_185 | Vibidia_duodecimguttata_603 | 0.188 |
| Aphidecta_obliterata_186 | Vibidia_duodecimguttata_603 | 0.188 |
| Coccinella_transversoguttata_265 | Vibidia_duodecimguttata_603 | 0.188 |
| Coccinella_transversoguttata_266 | Vibidia_duodecimguttata_603 | 0.188 |
| Hyperaspis_binotata_650 | Scymnus_sinuanodulus_828 | 0.188 |
| Scymnus_nubilus_796 | Scymnus_suturalis_832 | 0.188 |
| Scymnus_nubilus_798 | Scymnus_suturalis_832 | 0.188 |
| Scymnus_nubilus_800 | Scymnus_suturalis_832 | 0.188 |
| Scymnus_nubilus_801 | Scymnus_suturalis_832 | 0.188 |
| Scymnus_nubilus_802 | Scymnus_suturalis_832 | 0.188 |
| Scymnus_nubilus_805 | Scymnus_suturalis_832 | 0.188 |
| Scymnus_nubilus_806 | Scymnus_suturalis_832 | 0.188 |
| Scymnus_nubilus_807 | Scymnus_suturalis_832 | 0.188 |
| Scymnus_nubilus_810 | Scymnus_suturalis_832 | 0.188 |
| Scymnus_nubilus_803 | Scymnus_suturalis_834 | 0.188 |
| Scymnus_nubilus_804 | Scymnus_suturalis_834 | 0.188 |
| Scymnus_nubilus_808 | Scymnus_suturalis_834 | 0.188 |
| Scymnus_nubilus_809 | Scymnus_suturalis_834 | 0.188 |
| Scymnus_nubilus_796 | Scymnus_suturalis_835 | 0.188 |
| Scymnus_nubilus_798 | Scymnus_suturalis_835 | 0.188 |
| Scymnus_nubilus_800 | Scymnus_suturalis_835 | 0.188 |
| Scymnus_nubilus_801 | Scymnus_suturalis_835 | 0.188 |
| Scymnus_nubilus_802 | Scymnus_suturalis_835 | 0.188 |
| Scymnus_nubilus_805 | Scymnus_suturalis_835 | 0.188 |
| Scymnus_nubilus_806 | Scymnus_suturalis_835 | 0.188 |

| | | |
|---|---|---|
| Scymnus_nubilus_807 | Scymnus_suturalis_835 | 0.188 |
| Scymnus_nubilus_810 | Scymnus_suturalis_835 | 0.188 |
| Scymnus_nubilus_796 | Scymnus_suturalis_836 | 0.188 |
| Scymnus_nubilus_798 | Scymnus_suturalis_836 | 0.188 |
| Scymnus_nubilus_800 | Scymnus_suturalis_836 | 0.188 |
| Scymnus_nubilus_801 | Scymnus_suturalis_836 | 0.188 |
| Scymnus_nubilus_802 | Scymnus_suturalis_836 | 0.188 |
| Scymnus_nubilus_805 | Scymnus_suturalis_836 | 0.188 |
| Scymnus_nubilus_806 | Scymnus_suturalis_836 | 0.188 |
| Scymnus_nubilus_807 | Scymnus_suturalis_836 | 0.188 |
| Scymnus_nubilus_810 | Scymnus_suturalis_836 | 0.188 |
| Scymnus_nubilus_803 | Scymnus_suturalis_837 | 0.188 |
| Scymnus_nubilus_804 | Scymnus_suturalis_837 | 0.188 |
| Scymnus_nubilus_808 | Scymnus_suturalis_837 | 0.188 |
| Scymnus_nubilus_809 | Scymnus_suturalis_837 | 0.188 |
| Scymnus_nubilus_797 | Scymnus_suturalis_838 | 0.188 |
| Scymnus_nubilus_796 | Scymnus_suturalis_839 | 0.188 |
| Scymnus_nubilus_798 | Scymnus_suturalis_839 | 0.188 |
| Scymnus_nubilus_800 | Scymnus_suturalis_839 | 0.188 |
| Scymnus_nubilus_801 | Scymnus_suturalis_839 | 0.188 |
| Scymnus_nubilus_802 | Scymnus_suturalis_839 | 0.188 |
| Scymnus_nubilus_805 | Scymnus_suturalis_839 | 0.188 |
| Scymnus_nubilus_806 | Scymnus_suturalis_839 | 0.188 |
| Scymnus_nubilus_807 | Scymnus_suturalis_839 | 0.188 |
| Scymnus_nubilus_810 | Scymnus_suturalis_839 | 0.188 |
| Brumus_quadripustulatus_11 | Scymnus_sinuanodulus_829 | 0.188 |
| Brumus_quadripustulatus_13 | Scymnus_sinuanodulus_829 | 0.188 |
| Brumus_quadripustulatus_14 | Scymnus_sinuanodulus_829 | 0.188 |
| Brumus_quadripustulatus_17 | Scymnus_sinuanodulus_829 | 0.188 |
| Brumus_quadripustulatus_4 | Scymnus_sinuanodulus_829 | 0.188 |
| Brumus_quadripustulatus_7 | Scymnus_sinuanodulus_829 | 0.188 |
| Scymnus_haemorrhoidalis_752 | Subcoccinella_vigintiquatuorpunctata_624 | 0.188 |
| Scymnus_haemorrhoidalis_753 | Subcoccinella_vigintiquatuorpunctata_624 | 0.188 |
| Aphidecta_obliterata_176 | Vibidia_duodecimguttata_599 | 0.188 |
| Aphidecta_obliterata_176 | Vibidia_duodecimguttata_601 | 0.188 |
| Aphidecta_obliterata_176 | Vibidia_duodecimguttata_602 | 0.188 |
| Rhyzobius_chrysomeloides_85 | Scymnus_rubromaculatus_822 | 0.188 |
| Rhyzobius_chrysomeloides_86 | Scymnus_rubromaculatus_822 | 0.188 |
| Rhyzobius_chrysomeloides_87 | Scymnus_rubromaculatus_822 | 0.188 |
| Rhyzobius_chrysomeloides_88 | Scymnus_rubromaculatus_822 | 0.188 |
| Rhyzobius_chrysomeloides_89 | Scymnus_rubromaculatus_822 | 0.188 |
| Rhyzobius_chrysomeloides_90 | Scymnus_rubromaculatus_822 | 0.188 |
| Rhyzobius_chrysomeloides_91 | Scymnus_rubromaculatus_822 | 0.188 |
| Rhyzobius_chrysomeloides_92 | Scymnus_rubromaculatus_822 | 0.188 |
| Rhyzobius_chrysomeloides_93 | Scymnus_rubromaculatus_822 | 0.188 |
| Rhyzobius_chrysomeloides_94 | Scymnus_rubromaculatus_822 | 0.188 |
| Rhyzobius_chrysomeloides_95 | Scymnus_rubromaculatus_822 | 0.188 |
| Rhyzobius_chrysomeloides_85 | Scymnus_rubromaculatus_823 | 0.188 |
| Rhyzobius_chrysomeloides_86 | Scymnus_rubromaculatus_823 | 0.188 |
| Rhyzobius_chrysomeloides_87 | Scymnus_rubromaculatus_823 | 0.188 |
| Rhyzobius_chrysomeloides_88 | Scymnus_rubromaculatus_823 | 0.188 |
| Rhyzobius_chrysomeloides_89 | Scymnus_rubromaculatus_823 | 0.188 |
| Rhyzobius_chrysomeloides_90 | Scymnus_rubromaculatus_823 | 0.188 |
| Rhyzobius_chrysomeloides_91 | Scymnus_rubromaculatus_823 | 0.188 |
| Rhyzobius_chrysomeloides_92 | Scymnus_rubromaculatus_823 | 0.188 |
| Rhyzobius_chrysomeloides_93 | Scymnus_rubromaculatus_823 | 0.188 |

Supplementary Table S4 (continued)

| | | |
|---|---|---|
| Rhyzobius_chrysomeloides_94 | Scymnus_rubromaculatus_823 | 0.188 |
| Rhyzobius_chrysomeloides_95 | Scymnus_rubromaculatus_823 | 0.188 |
| Rhyzobius_chrysomeloides_85 | Scymnus_rubromaculatus_824 | 0.188 |
| Rhyzobius_chrysomeloides_86 | Scymnus_rubromaculatus_824 | 0.188 |
| Rhyzobius_chrysomeloides_87 | Scymnus_rubromaculatus_824 | 0.188 |
| Rhyzobius_chrysomeloides_88 | Scymnus_rubromaculatus_824 | 0.188 |
| Rhyzobius_chrysomeloides_89 | Scymnus_rubromaculatus_824 | 0.188 |
| Rhyzobius_chrysomeloides_90 | Scymnus_rubromaculatus_824 | 0.188 |
| Rhyzobius_chrysomeloides_91 | Scymnus_rubromaculatus_824 | 0.188 |
| Rhyzobius_chrysomeloides_92 | Scymnus_rubromaculatus_824 | 0.188 |
| Rhyzobius_chrysomeloides_93 | Scymnus_rubromaculatus_824 | 0.188 |
| Rhyzobius_chrysomeloides_94 | Scymnus_rubromaculatus_824 | 0.188 |
| Rhyzobius_chrysomeloides_95 | Scymnus_rubromaculatus_824 | 0.188 |
| Rhyzobius_chrysomeloides_85 | Scymnus_rubromaculatus_825 | 0.188 |
| Rhyzobius_chrysomeloides_86 | Scymnus_rubromaculatus_825 | 0.188 |
| Rhyzobius_chrysomeloides_87 | Scymnus_rubromaculatus_825 | 0.188 |
| Rhyzobius_chrysomeloides_88 | Scymnus_rubromaculatus_825 | 0.188 |
| Rhyzobius_chrysomeloides_89 | Scymnus_rubromaculatus_825 | 0.188 |
| Rhyzobius_chrysomeloides_90 | Scymnus_rubromaculatus_825 | 0.188 |
| Rhyzobius_chrysomeloides_91 | Scymnus_rubromaculatus_825 | 0.188 |
| Rhyzobius_chrysomeloides_92 | Scymnus_rubromaculatus_825 | 0.188 |
| Rhyzobius_chrysomeloides_93 | Scymnus_rubromaculatus_825 | 0.188 |
| Rhyzobius_chrysomeloides_94 | Scymnus_rubromaculatus_825 | 0.188 |
| Rhyzobius_chrysomeloides_95 | Scymnus_rubromaculatus_825 | 0.188 |
| Rhyzobius_chrysomeloides_85 | Scymnus_rubromaculatus_826 | 0.188 |
| Rhyzobius_chrysomeloides_86 | Scymnus_rubromaculatus_826 | 0.188 |
| Rhyzobius_chrysomeloides_87 | Scymnus_rubromaculatus_826 | 0.188 |
| Rhyzobius_chrysomeloides_88 | Scymnus_rubromaculatus_826 | 0.188 |
| Rhyzobius_chrysomeloides_89 | Scymnus_rubromaculatus_826 | 0.188 |
| Rhyzobius_chrysomeloides_90 | Scymnus_rubromaculatus_826 | 0.188 |
| Rhyzobius_chrysomeloides_91 | Scymnus_rubromaculatus_826 | 0.188 |
| Rhyzobius_chrysomeloides_92 | Scymnus_rubromaculatus_826 | 0.188 |
| Rhyzobius_chrysomeloides_93 | Scymnus_rubromaculatus_826 | 0.188 |
| Rhyzobius_chrysomeloides_94 | Scymnus_rubromaculatus_826 | 0.188 |
| Rhyzobius_chrysomeloides_95 | Scymnus_rubromaculatus_826 | 0.188 |
| Rhyzobius_chrysomeloides_85 | Scymnus_rubromaculatus_827 | 0.188 |
| Rhyzobius_chrysomeloides_86 | Scymnus_rubromaculatus_827 | 0.188 |
| Rhyzobius_chrysomeloides_87 | Scymnus_rubromaculatus_827 | 0.188 |
| Rhyzobius_chrysomeloides_88 | Scymnus_rubromaculatus_827 | 0.188 |
| Rhyzobius_chrysomeloides_89 | Scymnus_rubromaculatus_827 | 0.188 |
| Rhyzobius_chrysomeloides_90 | Scymnus_rubromaculatus_827 | 0.188 |
| Rhyzobius_chrysomeloides_91 | Scymnus_rubromaculatus_827 | 0.188 |
| Rhyzobius_chrysomeloides_92 | Scymnus_rubromaculatus_827 | 0.188 |
| Rhyzobius_chrysomeloides_93 | Scymnus_rubromaculatus_827 | 0.188 |
| Rhyzobius_chrysomeloides_94 | Scymnus_rubromaculatus_827 | 0.188 |
| Rhyzobius_chrysomeloides_95 | Scymnus_rubromaculatus_827 | 0.188 |
| Scymnus_nubilus_803 | Scymnus_suturalis_840 | 0.188 |
| Scymnus_nubilus_804 | Scymnus_suturalis_840 | 0.188 |
| Scymnus_nubilus_808 | Scymnus_suturalis_840 | 0.188 |
| Scymnus_nubilus_809 | Scymnus_suturalis_840 | 0.188 |
| Brumus_quadripustulatus_15 | Scymnus_sinuanodulus_829 | 0.188 |
| Brumus_quadripustulatus_16 | Scymnus_sinuanodulus_829 | 0.188 |
| Brumus_quadripustulatus_6 | Scymnus_sinuanodulus_829 | 0.188 |
| Brumus_quadripustulatus_8 | Scymnus_sinuanodulus_829 | 0.188 |
| Coccidula_scutellata_78 | Subcoccinella_vigintiquatuorpunctata_619 | 0.188 |
| Coccidula_scutellata_79 | Subcoccinella_vigintiquatuorpunctata_619 | 0.188 |

| | | |
|---|---|---|
| Scymnus_frontalis_746 | Scymnus_suturalis_841 | 0.188 |
| Scymnus_frontalis_746 | Scymnus_suturalis_842 | 0.188 |
| Scymnus_frontalis_746 | Scymnus_suturalis_844 | 0.188 |
| Scymnus_frontalis_746 | Scymnus_suturalis_846 | 0.188 |
| Scymnus_frontalis_746 | Scymnus_suturalis_847 | 0.188 |
| Scymnus_frontalis_746 | Scymnus_suturalis_848 | 0.188 |
| Scymnus_frontalis_746 | Scymnus_suturalis_832 | 0.188 |
| Scymnus_frontalis_746 | Scymnus_suturalis_835 | 0.188 |
| Scymnus_frontalis_746 | Scymnus_suturalis_836 | 0.188 |
| Scymnus_frontalis_746 | Scymnus_suturalis_839 | 0.188 |
| Scymnus_frontalis_746 | Scymnus_suturalis_840 | 0.188 |
| Brumus_quadripustulatus_10 | Scymnus_sinuanodulus_831 | 0.188 |
| Calvia_quatuordecimguttata_209 | Scymnus_sinuanodulus_831 | 0.188 |
| Diomus_terminatus_647 | Scymnus_sinuanodulus_829 | 0.189 |
| Diomus_terminatus_648 | Scymnus_sinuanodulus_829 | 0.189 |
| Hippodamia_glacialis_408 | Scymnus_suturalis_843 | 0.189 |
| Hippodamia_glacialis_409 | Scymnus_suturalis_843 | 0.189 |
| Hippodamia_glacialis_408 | Scymnus_suturalis_845 | 0.189 |
| Hippodamia_glacialis_409 | Scymnus_suturalis_845 | 0.189 |
| Brachiacantha_rotunda_632 | Subcoccinella_vigintiquatuorpunctata_630 | 0.189 |
| Brachiacantha_rotunda_633 | Subcoccinella_vigintiquatuorpunctata_630 | 0.189 |
| Brachiacantha_rotunda_635 | Subcoccinella_vigintiquatuorpunctata_630 | 0.189 |
| Psyllobora_vigintiduopunctata_571 | Vibidia_duodecimguttata_597 | 0.189 |
| Psyllobora_vigintiduopunctata_571 | Vibidia_duodecimguttata_600 | 0.189 |
| Scymnus_haemorrhoidalis_750 | Scymnus_sinuanodulus_830 | 0.189 |
| Scymnus_apetzi_694 | Subcoccinella_vigintiquatuorpunctata_627 | 0.189 |
| Psyllobora_vigintiduopunctata_571 | Vibidia_duodecimguttata_598 | 0.189 |
| Scymnus_haemorrhoidalis_750 | Scymnus_sinuanodulus_828 | 0.189 |
| Chilocorus_nigritus_29 | Vibidia_duodecimguttata_596 | 0.189 |
| Chilocorus_nigritus_29 | Vibidia_duodecimguttata_599 | 0.189 |
| Chilocorus_nigritus_29 | Vibidia_duodecimguttata_602 | 0.189 |
| Chilocorus_nigritus_29 | Vibidia_duodecimguttata_603 | 0.189 |
| Coelophora_inaequalis_315 | Vibidia_duodecimguttata_597 | 0.189 |
| Coelophora_inaequalis_315 | Vibidia_duodecimguttata_600 | 0.189 |
| Scymnus_apetzi_695 | Subcoccinella_vigintiquatuorpunctata_620 | 0.189 |
| Scymnus_apetzi_695 | Subcoccinella_vigintiquatuorpunctata_621 | 0.189 |
| Scymnus_apetzi_695 | Subcoccinella_vigintiquatuorpunctata_628 | 0.189 |
| Scymnus_apetzi_695 | Subcoccinella_vigintiquatuorpunctata_629 | 0.189 |
| Scymnus_apetzi_695 | Subcoccinella_vigintiquatuorpunctata_631 | 0.189 |
| Chilocorus_nigritus_29 | Vibidia_duodecimguttata_597 | 0.189 |
| Chilocorus_nigritus_29 | Vibidia_duodecimguttata_600 | 0.189 |
| Scymnus_apetzi_693 | Subcoccinella_vigintiquatuorpunctata_623 | 0.189 |
| Coccinella_hieroglyphica_226 | Vibidia_duodecimguttata_597 | 0.189 |
| Coccinella_quinquepunctata_236 | Vibidia_duodecimguttata_597 | 0.189 |
| Coccinella_hieroglyphica_226 | Vibidia_duodecimguttata_600 | 0.189 |
| Coccinella_quinquepunctata_236 | Vibidia_duodecimguttata_600 | 0.189 |
| Scymnus_apetzi_693 | Subcoccinella_vigintiquatuorpunctata_622 | 0.189 |
| Scymnus_apetzi_693 | Subcoccinella_vigintiquatuorpunctata_626 | 0.189 |
| Calvia_quatuordecimguttata_209 | Scymnus_sinuanodulus_829 | 0.189 |
| Brumus_quadripustulatus_10 | Scymnus_sinuanodulus_829 | 0.189 |
| Hyperaspis_reppensis_662 | Tytthaspis_sedecimpunctata_585 | 0.189 |
| Hyperaspis_reppensis_663 | Tytthaspis_sedecimpunctata_585 | 0.189 |
| Coelophora_inaequalis_312 | Vibidia_duodecimguttata_596 | 0.189 |
| Coelophora_inaequalis_314 | Vibidia_duodecimguttata_596 | 0.189 |
| Coelophora_inaequalis_317 | Vibidia_duodecimguttata_596 | 0.189 |
| Coelophora_inaequalis_325 | Vibidia_duodecimguttata_596 | 0.189 |

| | | |
|---|---|---|
| Psyllobora_renifer_550 | Vibidia_duodecimguttata_596 | 0.189 |
| Coccinella_hieroglyphica_226 | Vibidia_duodecimguttata_598 | 0.189 |
| Coccinella_quinquepunctata_236 | Vibidia_duodecimguttata_598 | 0.189 |
| Coccinella_undecimpunctata_282 | Vibidia_duodecimguttata_598 | 0.189 |
| Coccinella_undecimpunctata_283 | Vibidia_duodecimguttata_598 | 0.189 |
| Coccinella_undecimpunctata_284 | Vibidia_duodecimguttata_598 | 0.189 |
| Coelophora_inaequalis_312 | Vibidia_duodecimguttata_598 | 0.189 |
| Coelophora_inaequalis_314 | Vibidia_duodecimguttata_598 | 0.189 |
| Coelophora_inaequalis_317 | Vibidia_duodecimguttata_598 | 0.189 |
| Coelophora_inaequalis_325 | Vibidia_duodecimguttata_598 | 0.189 |
| Coccinella_hieroglyphica_226 | Vibidia_duodecimguttata_599 | 0.189 |
| Coccinella_quinquepunctata_236 | Vibidia_duodecimguttata_599 | 0.189 |
| Coccinella_undecimpunctata_282 | Vibidia_duodecimguttata_599 | 0.189 |
| Coccinella_undecimpunctata_283 | Vibidia_duodecimguttata_599 | 0.189 |
| Coccinella_undecimpunctata_284 | Vibidia_duodecimguttata_599 | 0.189 |
| Myzia_oblongoguttata_503 | Vibidia_duodecimguttata_599 | 0.189 |
| Myzia_oblongoguttata_504 | Vibidia_duodecimguttata_599 | 0.189 |
| Myzia_oblongoguttata_505 | Vibidia_duodecimguttata_599 | 0.189 |
| Myzia_oblongoguttata_507 | Vibidia_duodecimguttata_599 | 0.189 |
| Myzia_oblongoguttata_508 | Vibidia_duodecimguttata_599 | 0.189 |
| Myzia_oblongoguttata_509 | Vibidia_duodecimguttata_599 | 0.189 |
| Myzia_oblongoguttata_510 | Vibidia_duodecimguttata_599 | 0.189 |
| Myzia_oblongoguttata_511 | Vibidia_duodecimguttata_599 | 0.189 |
| Myzia_oblongoguttata_512 | Vibidia_duodecimguttata_599 | 0.189 |
| Myzia_oblongoguttata_513 | Vibidia_duodecimguttata_599 | 0.189 |
| Myzia_oblongoguttata_514 | Vibidia_duodecimguttata_599 | 0.189 |
| Myzia_oblongoguttata_516 | Vibidia_duodecimguttata_599 | 0.189 |
| Psyllobora_renifer_550 | Vibidia_duodecimguttata_599 | 0.189 |
| Coccinella_hieroglyphica_226 | Vibidia_duodecimguttata_601 | 0.189 |
| Coccinella_quinquepunctata_236 | Vibidia_duodecimguttata_601 | 0.189 |
| Myzia_oblongoguttata_506 | Vibidia_duodecimguttata_601 | 0.189 |
| Coccinella_hieroglyphica_226 | Vibidia_duodecimguttata_602 | 0.189 |
| Coccinella_quinquepunctata_236 | Vibidia_duodecimguttata_602 | 0.189 |
| Coccinella_undecimpunctata_282 | Vibidia_duodecimguttata_602 | 0.189 |
| Coccinella_undecimpunctata_283 | Vibidia_duodecimguttata_602 | 0.189 |
| Coccinella_undecimpunctata_284 | Vibidia_duodecimguttata_602 | 0.189 |
| Myzia_oblongoguttata_503 | Vibidia_duodecimguttata_602 | 0.189 |
| Myzia_oblongoguttata_504 | Vibidia_duodecimguttata_602 | 0.189 |
| Myzia_oblongoguttata_505 | Vibidia_duodecimguttata_602 | 0.189 |
| Myzia_oblongoguttata_507 | Vibidia_duodecimguttata_602 | 0.189 |
| Myzia_oblongoguttata_508 | Vibidia_duodecimguttata_602 | 0.189 |
| Myzia_oblongoguttata_509 | Vibidia_duodecimguttata_602 | 0.189 |
| Myzia_oblongoguttata_510 | Vibidia_duodecimguttata_602 | 0.189 |
| Myzia_oblongoguttata_511 | Vibidia_duodecimguttata_602 | 0.189 |
| Myzia_oblongoguttata_512 | Vibidia_duodecimguttata_602 | 0.189 |
| Myzia_oblongoguttata_513 | Vibidia_duodecimguttata_602 | 0.189 |
| Myzia_oblongoguttata_514 | Vibidia_duodecimguttata_602 | 0.189 |
| Myzia_oblongoguttata_516 | Vibidia_duodecimguttata_602 | 0.189 |
| Psyllobora_renifer_550 | Vibidia_duodecimguttata_602 | 0.189 |
| Coccinella_quinquepunctata_235 | Vibidia_duodecimguttata_603 | 0.189 |
| Coccinella_quinquepunctata_237 | Vibidia_duodecimguttata_603 | 0.189 |
| Coccinella_quinquepunctata_238 | Vibidia_duodecimguttata_603 | 0.189 |
| Coccinella_quinquepunctata_239 | Vibidia_duodecimguttata_603 | 0.189 |
| Coccinella_quinquepunctata_240 | Vibidia_duodecimguttata_603 | 0.189 |
| Myzia_oblongoguttata_503 | Vibidia_duodecimguttata_603 | 0.189 |
| Myzia_oblongoguttata_504 | Vibidia_duodecimguttata_603 | 0.189 |

| | | |
|---|---|---|
| Myzia_oblongoguttata_505 | Vibidia_duodecimguttata_603 | 0.189 |
| Myzia_oblongoguttata_507 | Vibidia_duodecimguttata_603 | 0.189 |
| Myzia_oblongoguttata_508 | Vibidia_duodecimguttata_603 | 0.189 |
| Myzia_oblongoguttata_509 | Vibidia_duodecimguttata_603 | 0.189 |
| Myzia_oblongoguttata_510 | Vibidia_duodecimguttata_603 | 0.189 |
| Myzia_oblongoguttata_511 | Vibidia_duodecimguttata_603 | 0.189 |
| Myzia_oblongoguttata_512 | Vibidia_duodecimguttata_603 | 0.189 |
| Myzia_oblongoguttata_513 | Vibidia_duodecimguttata_603 | 0.189 |
| Myzia_oblongoguttata_514 | Vibidia_duodecimguttata_603 | 0.189 |
| Myzia_oblongoguttata_516 | Vibidia_duodecimguttata_603 | 0.189 |
| Psyllobora_renifer_550 | Vibidia_duodecimguttata_603 | 0.189 |
| Hyperaspis_campestris_651 | Tytthaspis_sedecimpunctata_577 | 0.189 |
| Hyperaspis_campestris_652 | Tytthaspis_sedecimpunctata_577 | 0.189 |
| Hyperaspis_campestris_653 | Tytthaspis_sedecimpunctata_577 | 0.189 |
| Hyperaspis_campestris_651 | Tytthaspis_sedecimpunctata_578 | 0.189 |
| Hyperaspis_campestris_652 | Tytthaspis_sedecimpunctata_578 | 0.189 |
| Hyperaspis_campestris_653 | Tytthaspis_sedecimpunctata_578 | 0.189 |
| Hyperaspis_campestris_651 | Tytthaspis_sedecimpunctata_579 | 0.189 |
| Hyperaspis_campestris_652 | Tytthaspis_sedecimpunctata_579 | 0.189 |
| Hyperaspis_campestris_653 | Tytthaspis_sedecimpunctata_579 | 0.189 |
| Hyperaspis_campestris_651 | Tytthaspis_sedecimpunctata_581 | 0.189 |
| Hyperaspis_campestris_652 | Tytthaspis_sedecimpunctata_581 | 0.189 |
| Hyperaspis_campestris_653 | Tytthaspis_sedecimpunctata_581 | 0.189 |
| Hyperaspis_campestris_651 | Tytthaspis_sedecimpunctata_582 | 0.189 |
| Hyperaspis_campestris_652 | Tytthaspis_sedecimpunctata_582 | 0.189 |
| Hyperaspis_campestris_653 | Tytthaspis_sedecimpunctata_582 | 0.189 |
| Hyperaspis_campestris_651 | Tytthaspis_sedecimpunctata_590 | 0.189 |
| Hyperaspis_campestris_652 | Tytthaspis_sedecimpunctata_590 | 0.189 |
| Hyperaspis_campestris_653 | Tytthaspis_sedecimpunctata_590 | 0.189 |
| Hyperaspis_campestris_651 | Tytthaspis_sedecimpunctata_593 | 0.189 |
| Hyperaspis_campestris_652 | Tytthaspis_sedecimpunctata_593 | 0.189 |
| Hyperaspis_campestris_653 | Tytthaspis_sedecimpunctata_593 | 0.189 |
| Hyperaspis_campestris_651 | Tytthaspis_sedecimpunctata_594 | 0.189 |
| Hyperaspis_campestris_652 | Tytthaspis_sedecimpunctata_594 | 0.189 |
| Hyperaspis_campestris_653 | Tytthaspis_sedecimpunctata_594 | 0.189 |
| Hyperaspis_campestris_651 | Tytthaspis_sedecimpunctata_595 | 0.189 |
| Hyperaspis_campestris_652 | Tytthaspis_sedecimpunctata_595 | 0.189 |
| Hyperaspis_campestris_653 | Tytthaspis_sedecimpunctata_595 | 0.189 |
| Platynaspis_luteorubra_52 | Tytthaspis_sedecimpunctata_577 | 0.189 |
| Platynaspis_luteorubra_56 | Tytthaspis_sedecimpunctata_577 | 0.189 |
| Platynaspis_luteorubra_60 | Tytthaspis_sedecimpunctata_577 | 0.189 |
| Platynaspis_luteorubra_52 | Tytthaspis_sedecimpunctata_578 | 0.189 |
| Platynaspis_luteorubra_56 | Tytthaspis_sedecimpunctata_578 | 0.189 |
| Platynaspis_luteorubra_60 | Tytthaspis_sedecimpunctata_578 | 0.189 |
| Platynaspis_luteorubra_52 | Tytthaspis_sedecimpunctata_579 | 0.189 |
| Platynaspis_luteorubra_56 | Tytthaspis_sedecimpunctata_579 | 0.189 |
| Platynaspis_luteorubra_60 | Tytthaspis_sedecimpunctata_579 | 0.189 |
| Platynaspis_luteorubra_61 | Tytthaspis_sedecimpunctata_580 | 0.189 |
| Platynaspis_luteorubra_52 | Tytthaspis_sedecimpunctata_581 | 0.189 |
| Platynaspis_luteorubra_56 | Tytthaspis_sedecimpunctata_581 | 0.189 |
| Platynaspis_luteorubra_60 | Tytthaspis_sedecimpunctata_581 | 0.189 |
| Platynaspis_luteorubra_52 | Tytthaspis_sedecimpunctata_582 | 0.189 |
| Platynaspis_luteorubra_56 | Tytthaspis_sedecimpunctata_582 | 0.189 |
| Platynaspis_luteorubra_60 | Tytthaspis_sedecimpunctata_582 | 0.189 |
| Hyperaspis_oregona_658 | Tytthaspis_sedecimpunctata_583 | 0.189 |
| Hyperaspis_campestris_651 | Tytthaspis_sedecimpunctata_586 | 0.189 |

| | | |
|---|---|---|
| Hyperaspis_campestris_652 | Tytthaspis_sedecimpunctata_586 | 0.189 |
| Hyperaspis_campestris_653 | Tytthaspis_sedecimpunctata_586 | 0.189 |
| Hyperaspis_campestris_651 | Tytthaspis_sedecimpunctata_588 | 0.189 |
| Hyperaspis_campestris_652 | Tytthaspis_sedecimpunctata_588 | 0.189 |
| Hyperaspis_campestris_653 | Tytthaspis_sedecimpunctata_588 | 0.189 |
| Hyperaspis_oregona_659 | Tytthaspis_sedecimpunctata_589 | 0.189 |
| Platynaspis_luteorubra_52 | Tytthaspis_sedecimpunctata_589 | 0.189 |
| Platynaspis_luteorubra_56 | Tytthaspis_sedecimpunctata_589 | 0.189 |
| Platynaspis_luteorubra_60 | Tytthaspis_sedecimpunctata_589 | 0.189 |
| Platynaspis_luteorubra_52 | Tytthaspis_sedecimpunctata_590 | 0.189 |
| Platynaspis_luteorubra_56 | Tytthaspis_sedecimpunctata_590 | 0.189 |
| Platynaspis_luteorubra_60 | Tytthaspis_sedecimpunctata_590 | 0.189 |
| Platynaspis_luteorubra_55 | Tytthaspis_sedecimpunctata_591 | 0.189 |
| Hyperaspis_oregona_659 | Tytthaspis_sedecimpunctata_592 | 0.189 |
| Platynaspis_luteorubra_52 | Tytthaspis_sedecimpunctata_592 | 0.189 |
| Platynaspis_luteorubra_56 | Tytthaspis_sedecimpunctata_592 | 0.189 |
| Platynaspis_luteorubra_60 | Tytthaspis_sedecimpunctata_592 | 0.189 |
| Platynaspis_luteorubra_52 | Tytthaspis_sedecimpunctata_594 | 0.189 |
| Platynaspis_luteorubra_56 | Tytthaspis_sedecimpunctata_594 | 0.189 |
| Platynaspis_luteorubra_60 | Tytthaspis_sedecimpunctata_594 | 0.189 |
| Platynaspis_luteorubra_52 | Tytthaspis_sedecimpunctata_595 | 0.189 |
| Platynaspis_luteorubra_56 | Tytthaspis_sedecimpunctata_595 | 0.189 |
| Platynaspis_luteorubra_60 | Tytthaspis_sedecimpunctata_595 | 0.189 |
| Hyperaspis_lateralis_656 | Tytthaspis_sedecimpunctata_580 | 0.189 |
| Hyperaspis_lateralis_656 | Tytthaspis_sedecimpunctata_584 | 0.189 |
| Platynaspis_luteorubra_54 | Tytthaspis_sedecimpunctata_577 | 0.189 |
| Platynaspis_luteorubra_57 | Tytthaspis_sedecimpunctata_577 | 0.189 |
| Platynaspis_luteorubra_59 | Tytthaspis_sedecimpunctata_577 | 0.189 |
| Platynaspis_luteorubra_54 | Tytthaspis_sedecimpunctata_578 | 0.189 |
| Platynaspis_luteorubra_57 | Tytthaspis_sedecimpunctata_578 | 0.189 |
| Platynaspis_luteorubra_59 | Tytthaspis_sedecimpunctata_578 | 0.189 |
| Platynaspis_luteorubra_54 | Tytthaspis_sedecimpunctata_579 | 0.189 |
| Platynaspis_luteorubra_57 | Tytthaspis_sedecimpunctata_579 | 0.189 |
| Platynaspis_luteorubra_59 | Tytthaspis_sedecimpunctata_579 | 0.189 |
| Hyperaspis_oregona_659 | Tytthaspis_sedecimpunctata_580 | 0.189 |
| Platynaspis_luteorubra_54 | Tytthaspis_sedecimpunctata_581 | 0.189 |
| Platynaspis_luteorubra_57 | Tytthaspis_sedecimpunctata_581 | 0.189 |
| Platynaspis_luteorubra_59 | Tytthaspis_sedecimpunctata_581 | 0.189 |
| Platynaspis_luteorubra_54 | Tytthaspis_sedecimpunctata_582 | 0.189 |
| Platynaspis_luteorubra_57 | Tytthaspis_sedecimpunctata_582 | 0.189 |
| Platynaspis_luteorubra_59 | Tytthaspis_sedecimpunctata_582 | 0.189 |
| Hyperaspis_oregona_658 | Tytthaspis_sedecimpunctata_584 | 0.189 |
| Platynaspis_luteorubra_52 | Tytthaspis_sedecimpunctata_587 | 0.189 |
| Platynaspis_luteorubra_56 | Tytthaspis_sedecimpunctata_587 | 0.189 |
| Platynaspis_luteorubra_60 | Tytthaspis_sedecimpunctata_587 | 0.189 |
| Platynaspis_luteorubra_54 | Tytthaspis_sedecimpunctata_589 | 0.189 |
| Platynaspis_luteorubra_57 | Tytthaspis_sedecimpunctata_589 | 0.189 |
| Platynaspis_luteorubra_59 | Tytthaspis_sedecimpunctata_589 | 0.189 |
| Platynaspis_luteorubra_54 | Tytthaspis_sedecimpunctata_590 | 0.189 |
| Platynaspis_luteorubra_57 | Tytthaspis_sedecimpunctata_590 | 0.189 |
| Platynaspis_luteorubra_59 | Tytthaspis_sedecimpunctata_590 | 0.189 |
| Platynaspis_luteorubra_54 | Tytthaspis_sedecimpunctata_592 | 0.189 |
| Platynaspis_luteorubra_57 | Tytthaspis_sedecimpunctata_592 | 0.189 |
| Platynaspis_luteorubra_59 | Tytthaspis_sedecimpunctata_592 | 0.189 |
| Platynaspis_luteorubra_54 | Tytthaspis_sedecimpunctata_594 | 0.189 |
| Platynaspis_luteorubra_57 | Tytthaspis_sedecimpunctata_594 | 0.189 |

| | | |
|---|---|---|
| Platynaspis_luteorubra_59 | Tytthaspis_sedecimpunctata_594 | 0.189 |
| Platynaspis_luteorubra_54 | Tytthaspis_sedecimpunctata_595 | 0.189 |
| Platynaspis_luteorubra_57 | Tytthaspis_sedecimpunctata_595 | 0.189 |
| Platynaspis_luteorubra_59 | Tytthaspis_sedecimpunctata_595 | 0.189 |
| Hyperaspis_lateralis_656 | Tytthaspis_sedecimpunctata_583 | 0.189 |
| Hyperaspis_reppensis_662 | Stethorus_punctillum_850 | 0.189 |
| Hyperaspis_reppensis_663 | Stethorus_punctillum_850 | 0.189 |
| Chilocorus_renipustulatus_33 | Tytthaspis_sedecimpunctata_577 | 0.189 |
| Chilocorus_renipustulatus_33 | Tytthaspis_sedecimpunctata_578 | 0.189 |
| Chilocorus_renipustulatus_33 | Tytthaspis_sedecimpunctata_579 | 0.189 |
| Chilocorus_renipustulatus_33 | Tytthaspis_sedecimpunctata_581 | 0.189 |
| Chilocorus_renipustulatus_33 | Tytthaspis_sedecimpunctata_582 | 0.189 |
| Chilocorus_renipustulatus_34 | Tytthaspis_sedecimpunctata_583 | 0.189 |
| Chilocorus_renipustulatus_33 | Tytthaspis_sedecimpunctata_585 | 0.189 |
| Chilocorus_renipustulatus_39 | Tytthaspis_sedecimpunctata_586 | 0.189 |
| Scymnus_mitior_779 | Tytthaspis_sedecimpunctata_586 | 0.189 |
| Platynaspis_luteorubra_54 | Tytthaspis_sedecimpunctata_587 | 0.189 |
| Platynaspis_luteorubra_57 | Tytthaspis_sedecimpunctata_587 | 0.189 |
| Platynaspis_luteorubra_59 | Tytthaspis_sedecimpunctata_587 | 0.189 |
| Chilocorus_renipustulatus_39 | Tytthaspis_sedecimpunctata_588 | 0.189 |
| Scymnus_mitior_780 | Tytthaspis_sedecimpunctata_588 | 0.189 |
| Chilocorus_renipustulatus_33 | Tytthaspis_sedecimpunctata_590 | 0.189 |
| Chilocorus_renipustulatus_31 | Tytthaspis_sedecimpunctata_591 | 0.189 |
| Chilocorus_renipustulatus_32 | Tytthaspis_sedecimpunctata_591 | 0.189 |
| Chilocorus_renipustulatus_35 | Tytthaspis_sedecimpunctata_591 | 0.189 |
| Chilocorus_renipustulatus_36 | Tytthaspis_sedecimpunctata_591 | 0.189 |
| Chilocorus_renipustulatus_37 | Tytthaspis_sedecimpunctata_591 | 0.189 |
| Chilocorus_renipustulatus_38 | Tytthaspis_sedecimpunctata_591 | 0.189 |
| Chilocorus_renipustulatus_40 | Tytthaspis_sedecimpunctata_591 | 0.189 |
| Chilocorus_renipustulatus_41 | Tytthaspis_sedecimpunctata_591 | 0.189 |
| Chilocorus_renipustulatus_42 | Tytthaspis_sedecimpunctata_591 | 0.189 |
| Chilocorus_renipustulatus_33 | Tytthaspis_sedecimpunctata_592 | 0.189 |
| Chilocorus_renipustulatus_33 | Tytthaspis_sedecimpunctata_594 | 0.189 |
| Chilocorus_renipustulatus_33 | Tytthaspis_sedecimpunctata_595 | 0.189 |
| Hyperaspis_reppensis_665 | Tytthaspis_sedecimpunctata_577 | 0.189 |
| Hyperaspis_reppensis_665 | Tytthaspis_sedecimpunctata_578 | 0.189 |
| Hyperaspis_reppensis_665 | Tytthaspis_sedecimpunctata_579 | 0.189 |
| Chilocorus_renipustulatus_33 | Tytthaspis_sedecimpunctata_580 | 0.189 |
| Scymnus_loewii_774 | Tytthaspis_sedecimpunctata_580 | 0.189 |
| Hyperaspis_reppensis_665 | Tytthaspis_sedecimpunctata_581 | 0.189 |
| Hyperaspis_reppensis_665 | Tytthaspis_sedecimpunctata_582 | 0.189 |
| Hyperaspis_reppensis_666 | Tytthaspis_sedecimpunctata_583 | 0.189 |
| Scymnus_mitior_780 | Tytthaspis_sedecimpunctata_583 | 0.189 |
| Chilocorus_renipustulatus_34 | Tytthaspis_sedecimpunctata_584 | 0.189 |
| Hyperaspis_reppensis_665 | Tytthaspis_sedecimpunctata_590 | 0.189 |
| Hyperaspis_reppensis_665 | Tytthaspis_sedecimpunctata_593 | 0.189 |
| Hyperaspis_reppensis_665 | Tytthaspis_sedecimpunctata_594 | 0.189 |
| Hyperaspis_reppensis_665 | Tytthaspis_sedecimpunctata_595 | 0.189 |
| Coccidula_scutellata_77 | Scymnus_rubromaculatus_822 | 0.189 |
| Coccidula_scutellata_77 | Scymnus_rubromaculatus_823 | 0.189 |
| Coccidula_scutellata_77 | Scymnus_rubromaculatus_824 | 0.189 |
| Coccidula_scutellata_77 | Scymnus_rubromaculatus_825 | 0.189 |
| Coccidula_scutellata_77 | Scymnus_rubromaculatus_826 | 0.189 |
| Coccidula_scutellata_77 | Scymnus_rubromaculatus_827 | 0.189 |
| Coelophora_inaequalis_320 | Vibidia_duodecimguttata_596 | 0.189 |
| Coelophora_inaequalis_321 | Vibidia_duodecimguttata_596 | 0.189 |

| | | |
|---|---|---|
| Coelophora_inaequalis_322 | Vibidia_duodecimguttata_596 | 0.189 |
| Coelophora_inaequalis_320 | Vibidia_duodecimguttata_598 | 0.189 |
| Coelophora_inaequalis_321 | Vibidia_duodecimguttata_598 | 0.189 |
| Coelophora_inaequalis_322 | Vibidia_duodecimguttata_598 | 0.189 |
| Chilocorus_stigma_45 | Scymnus_suturalis_845 | 0.189 |
| Scymnus_loewii_772 | Tytthaspis_sedecimpunctata_580 | 0.189 |
| Coccinella_transversalis_258 | Tytthaspis_sedecimpunctata_583 | 0.189 |
| Hyperaspis_reppensis_665 | Tytthaspis_sedecimpunctata_584 | 0.189 |
| Myzia_oblongoguttata_506 | Tytthaspis_sedecimpunctata_584 | 0.189 |
| Scymnus_mitior_780 | Tytthaspis_sedecimpunctata_584 | 0.189 |
| Coccinella_transversalis_258 | Tytthaspis_sedecimpunctata_585 | 0.189 |
| Rhyzobius_chrysomeloides_85 | Tytthaspis_sedecimpunctata_587 | 0.189 |
| Rhyzobius_chrysomeloides_86 | Tytthaspis_sedecimpunctata_587 | 0.189 |
| Rhyzobius_chrysomeloides_87 | Tytthaspis_sedecimpunctata_587 | 0.189 |
| Rhyzobius_chrysomeloides_88 | Tytthaspis_sedecimpunctata_587 | 0.189 |
| Rhyzobius_chrysomeloides_89 | Tytthaspis_sedecimpunctata_587 | 0.189 |
| Rhyzobius_chrysomeloides_90 | Tytthaspis_sedecimpunctata_587 | 0.189 |
| Rhyzobius_chrysomeloides_91 | Tytthaspis_sedecimpunctata_587 | 0.189 |
| Rhyzobius_chrysomeloides_92 | Tytthaspis_sedecimpunctata_587 | 0.189 |
| Rhyzobius_chrysomeloides_93 | Tytthaspis_sedecimpunctata_587 | 0.189 |
| Rhyzobius_chrysomeloides_94 | Tytthaspis_sedecimpunctata_587 | 0.189 |
| Rhyzobius_chrysomeloides_95 | Tytthaspis_sedecimpunctata_587 | 0.189 |
| Scymnus_loewii_770 | Tytthaspis_sedecimpunctata_591 | 0.189 |
| Scymnus_loewii_773 | Tytthaspis_sedecimpunctata_591 | 0.189 |
| Scymnus_mitior_780 | Tytthaspis_sedecimpunctata_591 | 0.189 |
| Scymnus_nebulosus_782 | Tytthaspis_sedecimpunctata_591 | 0.189 |
| Coccidula_scutellata_78 | Scymnus_rubromaculatus_822 | 0.189 |
| Coccidula_scutellata_79 | Scymnus_rubromaculatus_822 | 0.189 |
| Coccidula_scutellata_78 | Scymnus_rubromaculatus_823 | 0.189 |
| Coccidula_scutellata_79 | Scymnus_rubromaculatus_823 | 0.189 |
| Coccidula_scutellata_78 | Scymnus_rubromaculatus_824 | 0.189 |
| Coccidula_scutellata_79 | Scymnus_rubromaculatus_824 | 0.189 |
| Coccidula_scutellata_78 | Scymnus_rubromaculatus_825 | 0.189 |
| Coccidula_scutellata_79 | Scymnus_rubromaculatus_825 | 0.189 |
| Coccidula_scutellata_78 | Scymnus_rubromaculatus_826 | 0.189 |
| Coccidula_scutellata_79 | Scymnus_rubromaculatus_826 | 0.189 |
| Coccidula_scutellata_78 | Scymnus_rubromaculatus_827 | 0.189 |
| Coccidula_scutellata_79 | Scymnus_rubromaculatus_827 | 0.189 |
| Macronaemia_episcopalis_477 | Scymnus_suturalis_833 | 0.189 |
| Propylaea_quatuordecimpunctata_532 | Scymnus_suturalis_838 | 0.189 |
| Propylaea_quatuordecimpunctata_533 | Scymnus_suturalis_838 | 0.189 |
| Propylaea_quatuordecimpunctata_535 | Scymnus_suturalis_838 | 0.189 |
| Propylaea_quatuordecimpunctata_536 | Scymnus_suturalis_838 | 0.189 |
| Propylaea_quatuordecimpunctata_537 | Scymnus_suturalis_838 | 0.189 |
| Propylaea_quatuordecimpunctata_542 | Scymnus_suturalis_838 | 0.189 |
| Propylaea_quatuordecimpunctata_546 | Scymnus_suturalis_838 | 0.189 |
| Propylaea_quatuordecimpunctata_532 | Scymnus_suturalis_840 | 0.189 |
| Propylaea_quatuordecimpunctata_533 | Scymnus_suturalis_840 | 0.189 |
| Propylaea_quatuordecimpunctata_535 | Scymnus_suturalis_840 | 0.189 |
| Propylaea_quatuordecimpunctata_536 | Scymnus_suturalis_840 | 0.189 |
| Propylaea_quatuordecimpunctata_537 | Scymnus_suturalis_840 | 0.189 |
| Propylaea_quatuordecimpunctata_542 | Scymnus_suturalis_840 | 0.189 |
| Propylaea_quatuordecimpunctata_546 | Scymnus_suturalis_840 | 0.189 |
| Coelophora_inaequalis_318 | Vibidia_duodecimguttata_597 | 0.189 |
| Coelophora_inaequalis_318 | Vibidia_duodecimguttata_600 | 0.189 |
| Scymnus_apetzi_693 | Subcoccinella_vigintiquatuorpunctata_619 | 0.189 |

| | | |
|---|---|---|
| Propylaea_quatuordecimpunctata_541 | Scymnus_suturalis_841 | 0.189 |
| Propylaea_quatuordecimpunctata_541 | Scymnus_suturalis_842 | 0.189 |
| Myrrha_octodecimguttata_499 | Scymnus_suturalis_843 | 0.189 |
| Propylaea_quatuordecimpunctata_541 | Scymnus_suturalis_844 | 0.189 |
| Myrrha_octodecimguttata_499 | Scymnus_suturalis_845 | 0.189 |
| Propylaea_quatuordecimpunctata_541 | Scymnus_suturalis_846 | 0.189 |
| Propylaea_quatuordecimpunctata_541 | Scymnus_suturalis_847 | 0.189 |
| Propylaea_quatuordecimpunctata_541 | Scymnus_suturalis_848 | 0.189 |
| Coccinella_transversalis_258 | Tytthaspis_sedecimpunctata_580 | 0.189 |
| Scymnus_ningshanensis_789 | Tytthaspis_sedecimpunctata_583 | 0.189 |
| Scymnus_ningshanensis_790 | Tytthaspis_sedecimpunctata_583 | 0.189 |
| Scymnus_loewii_774 | Tytthaspis_sedecimpunctata_584 | 0.189 |
| Didion_punctatum_645 | Tytthaspis_sedecimpunctata_587 | 0.189 |
| Scymnus_loewii_774 | Tytthaspis_sedecimpunctata_587 | 0.189 |
| Cheilomenes_sexmaculata_224 | Tytthaspis_sedecimpunctata_589 | 0.189 |
| Propylaea_quatuordecimpunctata_541 | Scymnus_suturalis_832 | 0.189 |
| Nephus_redtenbacheri_678 | Scymnus_suturalis_833 | 0.189 |
| Propylaea_quatuordecimpunctata_532 | Scymnus_suturalis_834 | 0.189 |
| Propylaea_quatuordecimpunctata_533 | Scymnus_suturalis_834 | 0.189 |
| Propylaea_quatuordecimpunctata_535 | Scymnus_suturalis_834 | 0.189 |
| Propylaea_quatuordecimpunctata_536 | Scymnus_suturalis_834 | 0.189 |
| Propylaea_quatuordecimpunctata_537 | Scymnus_suturalis_834 | 0.189 |
| Propylaea_quatuordecimpunctata_542 | Scymnus_suturalis_834 | 0.189 |
| Propylaea_quatuordecimpunctata_546 | Scymnus_suturalis_834 | 0.189 |
| Propylaea_quatuordecimpunctata_541 | Scymnus_suturalis_835 | 0.189 |
| Propylaea_quatuordecimpunctata_541 | Scymnus_suturalis_836 | 0.189 |
| Propylaea_quatuordecimpunctata_532 | Scymnus_suturalis_837 | 0.189 |
| Propylaea_quatuordecimpunctata_533 | Scymnus_suturalis_837 | 0.189 |
| Propylaea_quatuordecimpunctata_535 | Scymnus_suturalis_837 | 0.189 |
| Propylaea_quatuordecimpunctata_536 | Scymnus_suturalis_837 | 0.189 |
| Propylaea_quatuordecimpunctata_537 | Scymnus_suturalis_837 | 0.189 |
| Propylaea_quatuordecimpunctata_542 | Scymnus_suturalis_837 | 0.189 |
| Propylaea_quatuordecimpunctata_546 | Scymnus_suturalis_837 | 0.189 |
| Chilocorus_stigma_44 | Scymnus_suturalis_838 | 0.189 |
| Chilocorus_stigma_46 | Scymnus_suturalis_838 | 0.189 |
| Propylaea_quatuordecimpunctata_541 | Scymnus_suturalis_839 | 0.189 |
| Chilocorus_stigma_44 | Scymnus_suturalis_840 | 0.189 |
| Chilocorus_stigma_46 | Scymnus_suturalis_840 | 0.189 |
| Myrrha_octodecimguttata_485 | Scymnus_suturalis_841 | 0.189 |
| Myrrha_octodecimguttata_486 | Scymnus_suturalis_841 | 0.189 |
| Myrrha_octodecimguttata_487 | Scymnus_suturalis_841 | 0.189 |
| Myrrha_octodecimguttata_490 | Scymnus_suturalis_841 | 0.189 |
| Myrrha_octodecimguttata_494 | Scymnus_suturalis_841 | 0.189 |
| Myrrha_octodecimguttata_496 | Scymnus_suturalis_841 | 0.189 |
| Myrrha_octodecimguttata_497 | Scymnus_suturalis_841 | 0.189 |
| Myrrha_octodecimguttata_501 | Scymnus_suturalis_841 | 0.189 |
| Myrrha_octodecimguttata_502 | Scymnus_suturalis_841 | 0.189 |
| Nephus_redtenbacheri_678 | Scymnus_suturalis_841 | 0.189 |
| Myrrha_octodecimguttata_485 | Scymnus_suturalis_842 | 0.189 |
| Myrrha_octodecimguttata_486 | Scymnus_suturalis_842 | 0.189 |
| Myrrha_octodecimguttata_487 | Scymnus_suturalis_842 | 0.189 |
| Myrrha_octodecimguttata_490 | Scymnus_suturalis_842 | 0.189 |
| Myrrha_octodecimguttata_494 | Scymnus_suturalis_842 | 0.189 |
| Myrrha_octodecimguttata_496 | Scymnus_suturalis_842 | 0.189 |
| Myrrha_octodecimguttata_497 | Scymnus_suturalis_842 | 0.189 |
| Myrrha_octodecimguttata_501 | Scymnus_suturalis_842 | 0.189 |

| | | |
|---|---|---|
| Myrrha_octodecimguttata_502 | Scymnus_suturalis_842 | 0.189 |
| Nephus_redtenbacheri_678 | Scymnus_suturalis_842 | 0.189 |
| Myrrha_octodecimguttata_485 | Scymnus_suturalis_844 | 0.189 |
| Myrrha_octodecimguttata_486 | Scymnus_suturalis_844 | 0.189 |
| Myrrha_octodecimguttata_487 | Scymnus_suturalis_844 | 0.189 |
| Myrrha_octodecimguttata_490 | Scymnus_suturalis_844 | 0.189 |
| Myrrha_octodecimguttata_494 | Scymnus_suturalis_844 | 0.189 |
| Myrrha_octodecimguttata_496 | Scymnus_suturalis_844 | 0.189 |
| Myrrha_octodecimguttata_497 | Scymnus_suturalis_844 | 0.189 |
| Myrrha_octodecimguttata_501 | Scymnus_suturalis_844 | 0.189 |
| Myrrha_octodecimguttata_502 | Scymnus_suturalis_844 | 0.189 |
| Nephus_redtenbacheri_678 | Scymnus_suturalis_844 | 0.189 |
| Myrrha_octodecimguttata_485 | Scymnus_suturalis_846 | 0.189 |
| Myrrha_octodecimguttata_486 | Scymnus_suturalis_846 | 0.189 |
| Myrrha_octodecimguttata_487 | Scymnus_suturalis_846 | 0.189 |
| Myrrha_octodecimguttata_490 | Scymnus_suturalis_846 | 0.189 |
| Myrrha_octodecimguttata_494 | Scymnus_suturalis_846 | 0.189 |
| Myrrha_octodecimguttata_496 | Scymnus_suturalis_846 | 0.189 |
| Myrrha_octodecimguttata_497 | Scymnus_suturalis_846 | 0.189 |
| Myrrha_octodecimguttata_501 | Scymnus_suturalis_846 | 0.189 |
| Myrrha_octodecimguttata_502 | Scymnus_suturalis_846 | 0.189 |
| Nephus_redtenbacheri_678 | Scymnus_suturalis_846 | 0.189 |
| Myrrha_octodecimguttata_485 | Scymnus_suturalis_847 | 0.189 |
| Myrrha_octodecimguttata_486 | Scymnus_suturalis_847 | 0.189 |
| Myrrha_octodecimguttata_487 | Scymnus_suturalis_847 | 0.189 |
| Myrrha_octodecimguttata_490 | Scymnus_suturalis_847 | 0.189 |
| Myrrha_octodecimguttata_494 | Scymnus_suturalis_847 | 0.189 |
| Myrrha_octodecimguttata_496 | Scymnus_suturalis_847 | 0.189 |
| Myrrha_octodecimguttata_497 | Scymnus_suturalis_847 | 0.189 |
| Myrrha_octodecimguttata_501 | Scymnus_suturalis_847 | 0.189 |
| Myrrha_octodecimguttata_502 | Scymnus_suturalis_847 | 0.189 |
| Nephus_redtenbacheri_678 | Scymnus_suturalis_847 | 0.189 |
| Myrrha_octodecimguttata_485 | Scymnus_suturalis_848 | 0.189 |
| Myrrha_octodecimguttata_486 | Scymnus_suturalis_848 | 0.189 |
| Myrrha_octodecimguttata_487 | Scymnus_suturalis_848 | 0.189 |
| Myrrha_octodecimguttata_490 | Scymnus_suturalis_848 | 0.189 |
| Myrrha_octodecimguttata_494 | Scymnus_suturalis_848 | 0.189 |
| Myrrha_octodecimguttata_496 | Scymnus_suturalis_848 | 0.189 |
| Myrrha_octodecimguttata_497 | Scymnus_suturalis_848 | 0.189 |
| Myrrha_octodecimguttata_501 | Scymnus_suturalis_848 | 0.189 |
| Myrrha_octodecimguttata_502 | Scymnus_suturalis_848 | 0.189 |
| Nephus_redtenbacheri_678 | Scymnus_suturalis_848 | 0.189 |
| Hyperaspis_oregona_659 | Stethorus_punctillum_850 | 0.189 |
| Hyperaspis_oregona_658 | Stethorus_punctillum_852 | 0.189 |
| Hyperaspis_oregona_659 | Stethorus_punctillum_852 | 0.189 |
| Hyperaspis_oregona_658 | Stethorus_punctillum_860 | 0.189 |
| Hyperaspis_oregona_659 | Stethorus_punctillum_860 | 0.189 |
| Hyperaspis_lateralis_655 | Subcoccinella_vigintiquatuorpunctata_623 | 0.189 |
| Hyperaspis_lateralis_654 | Subcoccinella_vigintiquatuorpunctata_627 | 0.189 |
| Hyperaspis_lateralis_654 | Subcoccinella_vigintiquatuorpunctata_630 | 0.189 |
| Hyperaspis_reppensis_662 | Subcoccinella_vigintiquatuorpunctata_630 | 0.189 |
| Hyperaspis_reppensis_663 | Subcoccinella_vigintiquatuorpunctata_630 | 0.189 |
| Scymnus_ningshanensis_789 | Tytthaspis_sedecimpunctata_584 | 0.189 |
| Scymnus_ningshanensis_790 | Tytthaspis_sedecimpunctata_584 | 0.189 |
| Scymnus_ningshanensis_789 | Tytthaspis_sedecimpunctata_586 | 0.189 |
| Scymnus_ningshanensis_790 | Tytthaspis_sedecimpunctata_586 | 0.189 |

| | | |
|---|---|---|
| Didion_punctatum_646 | Tytthaspis_sedecimpunctata_587 | 0.189 |
| Scymnus_loewii_772 | Tytthaspis_sedecimpunctata_587 | 0.189 |
| Scymnus_ningshanensis_789 | Tytthaspis_sedecimpunctata_588 | 0.189 |
| Scymnus_ningshanensis_790 | Tytthaspis_sedecimpunctata_588 | 0.189 |
| Coccinella_septempunctata_242 | Tytthaspis_sedecimpunctata_589 | 0.189 |
| Coccinella_septempunctata_244 | Tytthaspis_sedecimpunctata_589 | 0.189 |
| Coccinella_septempunctata_245 | Tytthaspis_sedecimpunctata_589 | 0.189 |
| Coccinella_septempunctata_247 | Tytthaspis_sedecimpunctata_589 | 0.189 |
| Coccinella_septempunctata_248 | Tytthaspis_sedecimpunctata_589 | 0.189 |
| Coccinella_septempunctata_249 | Tytthaspis_sedecimpunctata_589 | 0.189 |
| Coccinella_septempunctata_252 | Tytthaspis_sedecimpunctata_589 | 0.189 |
| Coccinella_septempunctata_255 | Tytthaspis_sedecimpunctata_589 | 0.189 |
| Coccinella_septempunctata_256 | Tytthaspis_sedecimpunctata_589 | 0.189 |
| Myzia_oblongoguttata_503 | Tytthaspis_sedecimpunctata_591 | 0.189 |
| Myzia_oblongoguttata_515 | Tytthaspis_sedecimpunctata_591 | 0.189 |
| Scymnus_ningshanensis_789 | Tytthaspis_sedecimpunctata_591 | 0.189 |
| Scymnus_ningshanensis_790 | Tytthaspis_sedecimpunctata_591 | 0.189 |
| Myrrha_octodecimguttata_485 | Scymnus_suturalis_832 | 0.189 |
| Myrrha_octodecimguttata_486 | Scymnus_suturalis_832 | 0.189 |
| Myrrha_octodecimguttata_487 | Scymnus_suturalis_832 | 0.189 |
| Myrrha_octodecimguttata_490 | Scymnus_suturalis_832 | 0.189 |
| Myrrha_octodecimguttata_494 | Scymnus_suturalis_832 | 0.189 |
| Myrrha_octodecimguttata_496 | Scymnus_suturalis_832 | 0.189 |
| Myrrha_octodecimguttata_497 | Scymnus_suturalis_832 | 0.189 |
| Myrrha_octodecimguttata_501 | Scymnus_suturalis_832 | 0.189 |
| Myrrha_octodecimguttata_502 | Scymnus_suturalis_832 | 0.189 |
| Nephus_redtenbacheri_678 | Scymnus_suturalis_832 | 0.189 |
| Nephus_redtenbacheri_679 | Scymnus_suturalis_833 | 0.189 |
| Propylaea_quatuordecimpunctata_532 | Scymnus_suturalis_833 | 0.189 |
| Propylaea_quatuordecimpunctata_533 | Scymnus_suturalis_833 | 0.189 |
| Propylaea_quatuordecimpunctata_535 | Scymnus_suturalis_833 | 0.189 |
| Propylaea_quatuordecimpunctata_536 | Scymnus_suturalis_833 | 0.189 |
| Propylaea_quatuordecimpunctata_537 | Scymnus_suturalis_833 | 0.189 |
| Propylaea_quatuordecimpunctata_542 | Scymnus_suturalis_833 | 0.189 |
| Propylaea_quatuordecimpunctata_546 | Scymnus_suturalis_833 | 0.189 |
| Chilocorus_stigma_44 | Scymnus_suturalis_834 | 0.189 |
| Chilocorus_stigma_46 | Scymnus_suturalis_834 | 0.189 |
| Myrrha_octodecimguttata_485 | Scymnus_suturalis_835 | 0.189 |
| Myrrha_octodecimguttata_486 | Scymnus_suturalis_835 | 0.189 |
| Myrrha_octodecimguttata_487 | Scymnus_suturalis_835 | 0.189 |
| Myrrha_octodecimguttata_490 | Scymnus_suturalis_835 | 0.189 |
| Myrrha_octodecimguttata_494 | Scymnus_suturalis_835 | 0.189 |
| Myrrha_octodecimguttata_496 | Scymnus_suturalis_835 | 0.189 |
| Myrrha_octodecimguttata_497 | Scymnus_suturalis_835 | 0.189 |
| Myrrha_octodecimguttata_501 | Scymnus_suturalis_835 | 0.189 |
| Myrrha_octodecimguttata_502 | Scymnus_suturalis_835 | 0.189 |
| Nephus_redtenbacheri_678 | Scymnus_suturalis_835 | 0.189 |
| Myrrha_octodecimguttata_485 | Scymnus_suturalis_836 | 0.189 |
| Myrrha_octodecimguttata_486 | Scymnus_suturalis_836 | 0.189 |
| Myrrha_octodecimguttata_487 | Scymnus_suturalis_836 | 0.189 |
| Myrrha_octodecimguttata_490 | Scymnus_suturalis_836 | 0.189 |
| Myrrha_octodecimguttata_494 | Scymnus_suturalis_836 | 0.189 |
| Myrrha_octodecimguttata_496 | Scymnus_suturalis_836 | 0.189 |
| Myrrha_octodecimguttata_497 | Scymnus_suturalis_836 | 0.189 |
| Myrrha_octodecimguttata_501 | Scymnus_suturalis_836 | 0.189 |
| Myrrha_octodecimguttata_502 | Scymnus_suturalis_836 | 0.189 |

| | | |
|---|---|---|
| Nephus_redtenbacheri_678 | Scymnus_suturalis_836 | 0.189 |
| Nephus_redtenbacheri_678 | Scymnus_suturalis_837 | 0.189 |
| Myrrha_octodecimguttata_493 | Scymnus_suturalis_838 | 0.189 |
| Myrrha_octodecimguttata_495 | Scymnus_suturalis_838 | 0.189 |
| Myrrha_octodecimguttata_498 | Scymnus_suturalis_838 | 0.189 |
| Myrrha_octodecimguttata_485 | Scymnus_suturalis_839 | 0.189 |
| Myrrha_octodecimguttata_486 | Scymnus_suturalis_839 | 0.189 |
| Myrrha_octodecimguttata_487 | Scymnus_suturalis_839 | 0.189 |
| Myrrha_octodecimguttata_490 | Scymnus_suturalis_839 | 0.189 |
| Myrrha_octodecimguttata_494 | Scymnus_suturalis_839 | 0.189 |
| Myrrha_octodecimguttata_496 | Scymnus_suturalis_839 | 0.189 |
| Myrrha_octodecimguttata_497 | Scymnus_suturalis_839 | 0.189 |
| Myrrha_octodecimguttata_501 | Scymnus_suturalis_839 | 0.189 |
| Myrrha_octodecimguttata_502 | Scymnus_suturalis_839 | 0.189 |
| Nephus_redtenbacheri_678 | Scymnus_suturalis_839 | 0.189 |
| Myrrha_octodecimguttata_489 | Scymnus_suturalis_840 | 0.189 |
| Myrrha_octodecimguttata_493 | Scymnus_suturalis_840 | 0.189 |
| Myrrha_octodecimguttata_495 | Scymnus_suturalis_840 | 0.189 |
| Myrrha_octodecimguttata_498 | Scymnus_suturalis_840 | 0.189 |
| Myrrha_octodecimguttata_500 | Scymnus_suturalis_840 | 0.189 |
| Nephus_redtenbacheri_679 | Scymnus_suturalis_841 | 0.190 |
| Nephus_redtenbacheri_679 | Scymnus_suturalis_842 | 0.190 |
| Nephus_redtenbacheri_679 | Scymnus_suturalis_844 | 0.190 |
| Nephus_redtenbacheri_676 | Scymnus_suturalis_845 | 0.190 |
| Nephus_redtenbacheri_677 | Scymnus_suturalis_845 | 0.190 |
| Nephus_redtenbacheri_680 | Scymnus_suturalis_845 | 0.190 |
| Nephus_redtenbacheri_681 | Scymnus_suturalis_845 | 0.190 |
| Nephus_redtenbacheri_679 | Scymnus_suturalis_846 | 0.190 |
| Nephus_redtenbacheri_679 | Scymnus_suturalis_847 | 0.190 |
| Nephus_redtenbacheri_679 | Scymnus_suturalis_848 | 0.190 |
| Hyperaspis_proba_661 | Stethorus_punctillum_850 | 0.190 |
| Hyperaspis_proba_661 | Stethorus_punctillum_854 | 0.190 |
| Hyperaspis_proba_661 | Stethorus_punctillum_856 | 0.190 |
| Hyperaspis_lateralis_655 | Subcoccinella_vigintiquatuorpunctata_622 | 0.190 |
| Hyperaspis_lateralis_655 | Subcoccinella_vigintiquatuorpunctata_626 | 0.190 |
| Coccinella_septempunctata_241 | Tytthaspis_sedecimpunctata_586 | 0.190 |
| Coccinella_septempunctata_243 | Tytthaspis_sedecimpunctata_586 | 0.190 |
| Coccinella_septempunctata_253 | Tytthaspis_sedecimpunctata_586 | 0.190 |
| Didion_punctatum_644 | Tytthaspis_sedecimpunctata_587 | 0.190 |
| Coccinella_septempunctata_241 | Tytthaspis_sedecimpunctata_588 | 0.190 |
| Coccinella_septempunctata_243 | Tytthaspis_sedecimpunctata_588 | 0.190 |
| Coccinella_septempunctata_253 | Tytthaspis_sedecimpunctata_588 | 0.190 |
| Coccinella_septempunctata_250 | Tytthaspis_sedecimpunctata_589 | 0.190 |
| Coccinella_septempunctata_251 | Tytthaspis_sedecimpunctata_589 | 0.190 |
| Scymnus_camptodromus_714 | Tytthaspis_sedecimpunctata_591 | 0.190 |
| Chilocorus_bipustulatus_23 | Scymnus_sinuanodulus_830 | 0.190 |
| Nephus_redtenbacheri_679 | Scymnus_suturalis_832 | 0.190 |
| Myrrha_octodecimguttata_489 | Scymnus_suturalis_834 | 0.190 |
| Myrrha_octodecimguttata_493 | Scymnus_suturalis_834 | 0.190 |
| Myrrha_octodecimguttata_495 | Scymnus_suturalis_834 | 0.190 |
| Myrrha_octodecimguttata_498 | Scymnus_suturalis_834 | 0.190 |
| Myrrha_octodecimguttata_500 | Scymnus_suturalis_834 | 0.190 |
| Nephus_redtenbacheri_676 | Scymnus_suturalis_834 | 0.190 |
| Nephus_redtenbacheri_677 | Scymnus_suturalis_834 | 0.190 |
| Nephus_redtenbacheri_680 | Scymnus_suturalis_834 | 0.190 |
| Nephus_redtenbacheri_681 | Scymnus_suturalis_834 | 0.190 |

| | | |
|---|---|---|
| Nephus_redtenbacheri_679 | Scymnus_suturalis_835 | 0.190 |
| Nephus_redtenbacheri_679 | Scymnus_suturalis_836 | 0.190 |
| Myrrha_octodecimguttata_489 | Scymnus_suturalis_837 | 0.190 |
| Myrrha_octodecimguttata_493 | Scymnus_suturalis_837 | 0.190 |
| Myrrha_octodecimguttata_495 | Scymnus_suturalis_837 | 0.190 |
| Myrrha_octodecimguttata_498 | Scymnus_suturalis_837 | 0.190 |
| Myrrha_octodecimguttata_500 | Scymnus_suturalis_837 | 0.190 |
| Nephus_redtenbacheri_679 | Scymnus_suturalis_837 | 0.190 |
| Anatis_labiculata_126 | Scymnus_suturalis_838 | 0.190 |
| Nephus_redtenbacheri_679 | Scymnus_suturalis_839 | 0.190 |
| Scymnus_nebulosus_781 | Subcoccinella_vigintiquatuorpunctata_627 | 0.190 |
| Scymnus_apetzi_691 | Subcoccinella_vigintiquatuorpunctata_630 | 0.190 |
| Brachiacantha_rotunda_632 | Tytthaspis_sedecimpunctata_580 | 0.190 |
| Brachiacantha_rotunda_633 | Tytthaspis_sedecimpunctata_580 | 0.190 |
| Brachiacantha_rotunda_635 | Tytthaspis_sedecimpunctata_580 | 0.190 |
| Brachiacantha_rotunda_636 | Tytthaspis_sedecimpunctata_580 | 0.190 |
| Coccinella_septempunctata_246 | Tytthaspis_sedecimpunctata_583 | 0.190 |
| Scymnus_interruptus_762 | Tytthaspis_sedecimpunctata_583 | 0.190 |
| Scymnus_nigrinus_785 | Tytthaspis_sedecimpunctata_583 | 0.190 |
| Scymnus_nigrinus_786 | Tytthaspis_sedecimpunctata_583 | 0.190 |
| Scymnus_nigrinus_785 | Tytthaspis_sedecimpunctata_585 | 0.190 |
| Scymnus_nigrinus_786 | Tytthaspis_sedecimpunctata_585 | 0.190 |
| Coccinella_septempunctata_254 | Tytthaspis_sedecimpunctata_587 | 0.190 |
| Scymnus_nigrinus_785 | Tytthaspis_sedecimpunctata_589 | 0.190 |
| Scymnus_nigrinus_786 | Tytthaspis_sedecimpunctata_589 | 0.190 |
| Scymnus_nigrinus_785 | Tytthaspis_sedecimpunctata_592 | 0.190 |
| Scymnus_nigrinus_786 | Tytthaspis_sedecimpunctata_592 | 0.190 |
| Chilocorus_bipustulatus_23 | Scymnus_sinuanodulus_828 | 0.190 |
| Anatis_labiculata_126 | Scymnus_suturalis_837 | 0.190 |
| Anatis_mali_128 | Scymnus_suturalis_838 | 0.190 |
| Anatis_mali_129 | Scymnus_suturalis_838 | 0.190 |
| Anatis_mali_130 | Scymnus_suturalis_838 | 0.190 |
| Anatis_mali_131 | Scymnus_suturalis_838 | 0.190 |
| Anatis_mali_128 | Scymnus_suturalis_840 | 0.190 |
| Anatis_mali_129 | Scymnus_suturalis_840 | 0.190 |
| Anatis_mali_130 | Scymnus_suturalis_840 | 0.190 |
| Anatis_mali_131 | Scymnus_suturalis_840 | 0.190 |
| Cynegetis_impunctata_614 | Subcoccinella_vigintiquatuorpunctata_619 | 0.190 |
| Calvia_quatuordecimguttata_211 | Scymnus_suturalis_843 | 0.190 |
| Calvia_quatuordecimguttata_212 | Scymnus_suturalis_843 | 0.190 |
| Calvia_quatuordecimguttata_213 | Scymnus_suturalis_843 | 0.190 |
| Calvia_quatuordecimguttata_214 | Scymnus_suturalis_843 | 0.190 |
| Calvia_quatuordecimguttata_215 | Scymnus_suturalis_843 | 0.190 |
| Calvia_quatuordecimguttata_211 | Scymnus_suturalis_845 | 0.190 |
| Calvia_quatuordecimguttata_212 | Scymnus_suturalis_845 | 0.190 |
| Calvia_quatuordecimguttata_213 | Scymnus_suturalis_845 | 0.190 |
| Calvia_quatuordecimguttata_214 | Scymnus_suturalis_845 | 0.190 |
| Calvia_quatuordecimguttata_215 | Scymnus_suturalis_845 | 0.190 |
| Hyperaspis_proba_661 | Stethorus_punctillum_855 | 0.190 |
| Scymnus_camptodromus_705 | Tytthaspis_sedecimpunctata_577 | 0.190 |
| Scymnus_camptodromus_713 | Tytthaspis_sedecimpunctata_577 | 0.190 |
| Scymnus_camptodromus_716 | Tytthaspis_sedecimpunctata_577 | 0.190 |
| Scymnus_camptodromus_705 | Tytthaspis_sedecimpunctata_578 | 0.190 |
| Scymnus_camptodromus_713 | Tytthaspis_sedecimpunctata_578 | 0.190 |
| Scymnus_camptodromus_716 | Tytthaspis_sedecimpunctata_578 | 0.190 |
| Scymnus_camptodromus_705 | Tytthaspis_sedecimpunctata_579 | 0.190 |

| | | |
|---|---|---|
| Scymnus_camptodromus_713 | Tytthaspis_sedecimpunctata_579 | 0.190 |
| Scymnus_camptodromus_716 | Tytthaspis_sedecimpunctata_579 | 0.190 |
| Scymnus_camptodromus_705 | Tytthaspis_sedecimpunctata_581 | 0.190 |
| Scymnus_camptodromus_713 | Tytthaspis_sedecimpunctata_581 | 0.190 |
| Scymnus_camptodromus_716 | Tytthaspis_sedecimpunctata_581 | 0.190 |
| Scymnus_camptodromus_705 | Tytthaspis_sedecimpunctata_582 | 0.190 |
| Scymnus_camptodromus_713 | Tytthaspis_sedecimpunctata_582 | 0.190 |
| Scymnus_camptodromus_716 | Tytthaspis_sedecimpunctata_582 | 0.190 |
| Brachiacantha_rotunda_634 | Tytthaspis_sedecimpunctata_583 | 0.190 |
| Scymnus_interruptus_759 | Tytthaspis_sedecimpunctata_583 | 0.190 |
| Scymnus_interruptus_769 | Tytthaspis_sedecimpunctata_583 | 0.190 |
| Brachiacantha_rotunda_634 | Tytthaspis_sedecimpunctata_585 | 0.190 |
| Scymnus_interruptus_760 | Tytthaspis_sedecimpunctata_585 | 0.190 |
| Scymnus_interruptus_765 | Tytthaspis_sedecimpunctata_585 | 0.190 |
| Scymnus_interruptus_768 | Tytthaspis_sedecimpunctata_585 | 0.190 |
| Scymnus_nigrinus_784 | Tytthaspis_sedecimpunctata_586 | 0.190 |
| Scymnus_nigrinus_785 | Tytthaspis_sedecimpunctata_587 | 0.190 |
| Scymnus_nigrinus_786 | Tytthaspis_sedecimpunctata_587 | 0.190 |
| Scymnus_ferrugatus_723 | Tytthaspis_sedecimpunctata_588 | 0.190 |
| Scymnus_ferrugatus_725 | Tytthaspis_sedecimpunctata_588 | 0.190 |
| Scymnus_ferrugatus_726 | Tytthaspis_sedecimpunctata_588 | 0.190 |
| Scymnus_ferrugatus_727 | Tytthaspis_sedecimpunctata_588 | 0.190 |
| Scymnus_ferrugatus_728 | Tytthaspis_sedecimpunctata_588 | 0.190 |
| Scymnus_ferrugatus_730 | Tytthaspis_sedecimpunctata_588 | 0.190 |
| Scymnus_ferrugatus_731 | Tytthaspis_sedecimpunctata_588 | 0.190 |
| Scymnus_ferrugatus_732 | Tytthaspis_sedecimpunctata_588 | 0.190 |
| Scymnus_ferrugatus_733 | Tytthaspis_sedecimpunctata_588 | 0.190 |
| Brachiacantha_rotunda_632 | Tytthaspis_sedecimpunctata_589 | 0.190 |
| Brachiacantha_rotunda_633 | Tytthaspis_sedecimpunctata_589 | 0.190 |
| Brachiacantha_rotunda_635 | Tytthaspis_sedecimpunctata_589 | 0.190 |
| Brachiacantha_rotunda_636 | Tytthaspis_sedecimpunctata_589 | 0.190 |
| Scymnus_camptodromus_705 | Tytthaspis_sedecimpunctata_590 | 0.190 |
| Scymnus_camptodromus_713 | Tytthaspis_sedecimpunctata_590 | 0.190 |
| Scymnus_camptodromus_716 | Tytthaspis_sedecimpunctata_590 | 0.190 |
| Scymnus_camptodromus_705 | Tytthaspis_sedecimpunctata_592 | 0.190 |
| Scymnus_camptodromus_713 | Tytthaspis_sedecimpunctata_592 | 0.190 |
| Scymnus_camptodromus_716 | Tytthaspis_sedecimpunctata_592 | 0.190 |
| Scymnus_interruptus_767 | Tytthaspis_sedecimpunctata_592 | 0.190 |
| Brachiacantha_rotunda_634 | Tytthaspis_sedecimpunctata_593 | 0.190 |
| Scymnus_camptodromus_705 | Tytthaspis_sedecimpunctata_593 | 0.190 |
| Scymnus_camptodromus_713 | Tytthaspis_sedecimpunctata_593 | 0.190 |
| Scymnus_camptodromus_716 | Tytthaspis_sedecimpunctata_593 | 0.190 |
| Scymnus_camptodromus_705 | Tytthaspis_sedecimpunctata_594 | 0.190 |
| Scymnus_camptodromus_713 | Tytthaspis_sedecimpunctata_594 | 0.190 |
| Scymnus_camptodromus_716 | Tytthaspis_sedecimpunctata_594 | 0.190 |
| Scymnus_camptodromus_705 | Tytthaspis_sedecimpunctata_595 | 0.190 |
| Scymnus_camptodromus_713 | Tytthaspis_sedecimpunctata_595 | 0.190 |
| Scymnus_camptodromus_716 | Tytthaspis_sedecimpunctata_595 | 0.190 |
| Brumoides_suturalis_2 | Scymnus_rubromaculatus_822 | 0.190 |
| Platynaspis_luteorubra_61 | Scymnus_rubromaculatus_822 | 0.190 |
| Brumoides_suturalis_2 | Scymnus_rubromaculatus_823 | 0.190 |
| Platynaspis_luteorubra_61 | Scymnus_rubromaculatus_823 | 0.190 |
| Brumoides_suturalis_2 | Scymnus_rubromaculatus_824 | 0.190 |
| Platynaspis_luteorubra_61 | Scymnus_rubromaculatus_824 | 0.190 |
| Brumoides_suturalis_2 | Scymnus_rubromaculatus_825 | 0.190 |
| Platynaspis_luteorubra_61 | Scymnus_rubromaculatus_825 | 0.190 |

| | | |
|---|---|---|
| Brumoides_suturalis_2 | Scymnus_rubromaculatus_826 | 0.190 |
| Platynaspis_luteorubra_61 | Scymnus_rubromaculatus_826 | 0.190 |
| Brumoides_suturalis_2 | Scymnus_rubromaculatus_827 | 0.190 |
| Platynaspis_luteorubra_61 | Scymnus_rubromaculatus_827 | 0.190 |
| Chilocorus_stigma_43 | Scymnus_sinuanodulus_830 | 0.190 |
| Anatis_labiculata_123 | Scymnus_suturalis_833 | 0.190 |
| Anatis_labiculata_124 | Scymnus_suturalis_833 | 0.190 |
| Anatis_labiculata_125 | Scymnus_suturalis_833 | 0.190 |
| Anatis_labiculata_127 | Scymnus_suturalis_833 | 0.190 |
| Anatis_mali_128 | Scymnus_suturalis_834 | 0.190 |
| Anatis_mali_129 | Scymnus_suturalis_834 | 0.190 |
| Anatis_mali_130 | Scymnus_suturalis_834 | 0.190 |
| Anatis_mali_131 | Scymnus_suturalis_834 | 0.190 |
| Anatis_mali_128 | Scymnus_suturalis_837 | 0.190 |
| Anatis_mali_129 | Scymnus_suturalis_837 | 0.190 |
| Anatis_mali_130 | Scymnus_suturalis_837 | 0.190 |
| Anatis_mali_131 | Scymnus_suturalis_837 | 0.190 |
| Macronaemia_episcopalis_478 | Scymnus_suturalis_838 | 0.190 |
| Macronaemia_episcopalis_478 | Scymnus_suturalis_840 | 0.190 |
| Hyperaspis_lateralis_654 | Stethorus_punctillum_849 | 0.190 |
| Hyperaspis_lateralis_654 | Stethorus_punctillum_850 | 0.190 |
| Hyperaspis_lateralis_654 | Stethorus_punctillum_854 | 0.190 |
| Hyperaspis_lateralis_654 | Stethorus_punctillum_856 | 0.190 |
| Hyperaspis_binotata_649 | Subcoccinella_vigintiquatuorpunctata_622 | 0.190 |
| Scymnus_interruptus_767 | Tytthaspis_sedecimpunctata_580 | 0.190 |
| Scymnus_ferrugatus_724 | Tytthaspis_sedecimpunctata_586 | 0.190 |
| Scymnus_ferrugatus_729 | Tytthaspis_sedecimpunctata_586 | 0.190 |
| Brachiacantha_rotunda_632 | Tytthaspis_sedecimpunctata_587 | 0.190 |
| Brachiacantha_rotunda_633 | Tytthaspis_sedecimpunctata_587 | 0.190 |
| Brachiacantha_rotunda_635 | Tytthaspis_sedecimpunctata_587 | 0.190 |
| Brachiacantha_rotunda_636 | Tytthaspis_sedecimpunctata_587 | 0.190 |
| Psyllobora_renifer_550 | Tytthaspis_sedecimpunctata_587 | 0.190 |
| Brachiacantha_rotunda_634 | Tytthaspis_sedecimpunctata_588 | 0.190 |
| Scymnus_interruptus_767 | Tytthaspis_sedecimpunctata_588 | 0.190 |
| Olla_vnigrum_525 | Tytthaspis_sedecimpunctata_589 | 0.190 |
| Olla_vnigrum_529 | Tytthaspis_sedecimpunctata_589 | 0.190 |
| Olla_vnigrum_531 | Tytthaspis_sedecimpunctata_589 | 0.190 |
| Scymnus_camptodromus_707 | Tytthaspis_sedecimpunctata_591 | 0.190 |
| Scymnus_camptodromus_708 | Tytthaspis_sedecimpunctata_591 | 0.190 |
| Scymnus_camptodromus_709 | Tytthaspis_sedecimpunctata_591 | 0.190 |
| Scymnus_camptodromus_710 | Tytthaspis_sedecimpunctata_591 | 0.190 |
| Chilocorus_stigma_44 | Scymnus_sinuanodulus_828 | 0.190 |
| Chilocorus_stigma_45 | Scymnus_sinuanodulus_828 | 0.190 |
| Chilocorus_stigma_46 | Scymnus_sinuanodulus_828 | 0.190 |
| Coccinella_undecimpunctata_282 | Scymnus_sinuanodulus_830 | 0.190 |
| Coccinella_undecimpunctata_283 | Scymnus_sinuanodulus_830 | 0.190 |
| Coccinella_undecimpunctata_284 | Scymnus_sinuanodulus_830 | 0.190 |
| Scymnus_frontalis_736 | Scymnus_suturalis_838 | 0.190 |
| Scymnus_frontalis_737 | Scymnus_suturalis_838 | 0.190 |
| Scymnus_frontalis_738 | Scymnus_suturalis_838 | 0.190 |
| Scymnus_frontalis_739 | Scymnus_suturalis_838 | 0.190 |
| Scymnus_frontalis_740 | Scymnus_suturalis_838 | 0.190 |
| Scymnus_frontalis_741 | Scymnus_suturalis_838 | 0.190 |
| Scymnus_frontalis_743 | Scymnus_suturalis_838 | 0.190 |
| Scymnus_frontalis_744 | Scymnus_suturalis_838 | 0.190 |
| Scymnus_frontalis_742 | Scymnus_suturalis_840 | 0.190 |

| | | |
|---|---|---|
| Hyperaspis_lateralis_654 | Subcoccinella_vigintiquatuorpunctata_619 | 0.190 |
| Brumus_quadripustulatus_12 | Scymnus_sinuanodulus_831 | 0.190 |
| Macronaemia_episcopalis_478 | Scymnus_suturalis_834 | 0.190 |
| Macronaemia_episcopalis_478 | Scymnus_suturalis_837 | 0.190 |
| Illeis_galbula_474 | Vibidia_duodecimguttata_599 | 0.190 |
| Illeis_galbula_475 | Vibidia_duodecimguttata_599 | 0.190 |
| Illeis_galbula_476 | Vibidia_duodecimguttata_599 | 0.190 |
| Illeis_galbula_474 | Vibidia_duodecimguttata_602 | 0.190 |
| Illeis_galbula_475 | Vibidia_duodecimguttata_602 | 0.190 |
| Illeis_galbula_476 | Vibidia_duodecimguttata_602 | 0.190 |
| Illeis_galbula_474 | Vibidia_duodecimguttata_603 | 0.190 |
| Illeis_galbula_475 | Vibidia_duodecimguttata_603 | 0.190 |
| Illeis_galbula_476 | Vibidia_duodecimguttata_603 | 0.190 |
| Scymnus_frontalis_742 | Scymnus_suturalis_841 | 0.190 |
| Scymnus_frontalis_742 | Scymnus_suturalis_842 | 0.190 |
| Harmonia_axyridis_354 | Scymnus_suturalis_843 | 0.190 |
| Harmonia_axyridis_356 | Scymnus_suturalis_843 | 0.190 |
| Harmonia_axyridis_357 | Scymnus_suturalis_843 | 0.190 |
| Harmonia_axyridis_358 | Scymnus_suturalis_843 | 0.190 |
| Harmonia_axyridis_359 | Scymnus_suturalis_843 | 0.190 |
| Harmonia_axyridis_360 | Scymnus_suturalis_843 | 0.190 |
| Harmonia_axyridis_361 | Scymnus_suturalis_843 | 0.190 |
| Harmonia_axyridis_362 | Scymnus_suturalis_843 | 0.190 |
| Harmonia_axyridis_363 | Scymnus_suturalis_843 | 0.190 |
| Harmonia_axyridis_364 | Scymnus_suturalis_843 | 0.190 |
| Scymnus_frontalis_742 | Scymnus_suturalis_844 | 0.190 |
| Harmonia_axyridis_354 | Scymnus_suturalis_845 | 0.190 |
| Harmonia_axyridis_356 | Scymnus_suturalis_845 | 0.190 |
| Harmonia_axyridis_357 | Scymnus_suturalis_845 | 0.190 |
| Harmonia_axyridis_358 | Scymnus_suturalis_845 | 0.190 |
| Harmonia_axyridis_359 | Scymnus_suturalis_845 | 0.190 |
| Harmonia_axyridis_360 | Scymnus_suturalis_845 | 0.190 |
| Harmonia_axyridis_361 | Scymnus_suturalis_845 | 0.190 |
| Harmonia_axyridis_362 | Scymnus_suturalis_845 | 0.190 |
| Harmonia_axyridis_363 | Scymnus_suturalis_845 | 0.190 |
| Harmonia_axyridis_364 | Scymnus_suturalis_845 | 0.190 |
| Scymnus_frontalis_742 | Scymnus_suturalis_846 | 0.190 |
| Scymnus_frontalis_742 | Scymnus_suturalis_847 | 0.190 |
| Scymnus_frontalis_742 | Scymnus_suturalis_848 | 0.190 |
| Hyperaspis_lateralis_656 | Stethorus_punctillum_851 | 0.190 |
| Hyperaspis_lateralis_656 | Stethorus_punctillum_858 | 0.190 |
| Hyperaspis_lateralis_655 | Stethorus_punctillum_860 | 0.190 |
| Scymnus_camptodromus_719 | Tytthaspis_sedecimpunctata_577 | 0.190 |
| Scymnus_camptodromus_719 | Tytthaspis_sedecimpunctata_578 | 0.190 |
| Scymnus_camptodromus_719 | Tytthaspis_sedecimpunctata_579 | 0.190 |
| Scymnus_camptodromus_719 | Tytthaspis_sedecimpunctata_581 | 0.190 |
| Scymnus_camptodromus_719 | Tytthaspis_sedecimpunctata_582 | 0.190 |
| Scymnus_camptodromus_718 | Tytthaspis_sedecimpunctata_584 | 0.190 |
| Scymnus_camptodromus_719 | Tytthaspis_sedecimpunctata_585 | 0.190 |
| Scymnus_camptodromus_718 | Tytthaspis_sedecimpunctata_587 | 0.190 |
| Olla_vnigrum_525 | Tytthaspis_sedecimpunctata_588 | 0.190 |
| Olla_vnigrum_529 | Tytthaspis_sedecimpunctata_588 | 0.190 |
| Olla_vnigrum_531 | Tytthaspis_sedecimpunctata_588 | 0.190 |
| Scymnus_ferrugatus_724 | Tytthaspis_sedecimpunctata_589 | 0.190 |
| Scymnus_ferrugatus_729 | Tytthaspis_sedecimpunctata_589 | 0.190 |
| Scymnus_camptodromus_719 | Tytthaspis_sedecimpunctata_590 | 0.190 |

| | | |
|---|---|---|
| Scymnus_camptodromus_719 | Tytthaspis_sedecimpunctata_592 | 0.190 |
| Scymnus_camptodromus_719 | Tytthaspis_sedecimpunctata_593 | 0.190 |
| Scymnus_camptodromus_719 | Tytthaspis_sedecimpunctata_594 | 0.190 |
| Scymnus_camptodromus_719 | Tytthaspis_sedecimpunctata_595 | 0.190 |
| Coccinella_undecimpunctata_282 | Scymnus_sinuanodulus_828 | 0.190 |
| Coccinella_undecimpunctata_283 | Scymnus_sinuanodulus_828 | 0.190 |
| Coccinella_undecimpunctata_284 | Scymnus_sinuanodulus_828 | 0.190 |
| Hyperaspis_reppensis_662 | Scymnus_sinuanodulus_830 | 0.190 |
| Hyperaspis_reppensis_663 | Scymnus_sinuanodulus_830 | 0.190 |
| Scymnus_frontalis_742 | Scymnus_suturalis_832 | 0.190 |
| Harmonia_axyridis_354 | Scymnus_suturalis_834 | 0.190 |
| Harmonia_axyridis_356 | Scymnus_suturalis_834 | 0.190 |
| Harmonia_axyridis_357 | Scymnus_suturalis_834 | 0.190 |
| Harmonia_axyridis_358 | Scymnus_suturalis_834 | 0.190 |
| Harmonia_axyridis_359 | Scymnus_suturalis_834 | 0.190 |
| Harmonia_axyridis_360 | Scymnus_suturalis_834 | 0.190 |
| Harmonia_axyridis_361 | Scymnus_suturalis_834 | 0.190 |
| Harmonia_axyridis_362 | Scymnus_suturalis_834 | 0.190 |
| Harmonia_axyridis_363 | Scymnus_suturalis_834 | 0.190 |
| Harmonia_axyridis_364 | Scymnus_suturalis_834 | 0.190 |
| Scymnus_frontalis_736 | Scymnus_suturalis_834 | 0.190 |
| Scymnus_frontalis_737 | Scymnus_suturalis_834 | 0.190 |
| Scymnus_frontalis_738 | Scymnus_suturalis_834 | 0.190 |
| Scymnus_frontalis_739 | Scymnus_suturalis_834 | 0.190 |
| Scymnus_frontalis_740 | Scymnus_suturalis_834 | 0.190 |
| Scymnus_frontalis_741 | Scymnus_suturalis_834 | 0.190 |
| Scymnus_frontalis_743 | Scymnus_suturalis_834 | 0.190 |
| Scymnus_frontalis_744 | Scymnus_suturalis_834 | 0.190 |
| Scymnus_frontalis_742 | Scymnus_suturalis_835 | 0.190 |
| Scymnus_frontalis_742 | Scymnus_suturalis_836 | 0.190 |
| Scymnus_frontalis_742 | Scymnus_suturalis_839 | 0.190 |
| Hyperaspis_proba_660 | Stethorus_punctillum_849 | 0.190 |
| Hyperaspis_proba_660 | Stethorus_punctillum_857 | 0.190 |
| Hyperaspis_proba_660 | Stethorus_punctillum_860 | 0.190 |
| Scymnus_ningshanensis_788 | Subcoccinella_vigintiquatuorpunctata_619 | 0.190 |
| Scymnus_ningshanensis_791 | Subcoccinella_vigintiquatuorpunctata_619 | 0.190 |
| Scymnus_ningshanensis_792 | Subcoccinella_vigintiquatuorpunctata_619 | 0.190 |
| Scymnus_ningshanensis_793 | Subcoccinella_vigintiquatuorpunctata_619 | 0.190 |
| Scymnus_ningshanensis_794 | Subcoccinella_vigintiquatuorpunctata_619 | 0.190 |
| Scymnus_ningshanensis_795 | Subcoccinella_vigintiquatuorpunctata_619 | 0.190 |
| Scymnus_apetzi_692 | Subcoccinella_vigintiquatuorpunctata_623 | 0.190 |
| Coccinella_quinquepunctata_234 | Tytthaspis_sedecimpunctata_588 | 0.190 |
| Platynaspis_luteorubra_50 | Scymnus_sinuanodulus_829 | 0.190 |
| Hippodamia_parenthesis_432 | Scymnus_suturalis_841 | 0.190 |
| Hippodamia_parenthesis_434 | Scymnus_suturalis_841 | 0.190 |
| Hippodamia_parenthesis_435 | Scymnus_suturalis_841 | 0.190 |
| Hippodamia_parenthesis_437 | Scymnus_suturalis_841 | 0.190 |
| Hippodamia_parenthesis_438 | Scymnus_suturalis_841 | 0.190 |
| Hippodamia_parenthesis_441 | Scymnus_suturalis_841 | 0.190 |
| Hippodamia_parenthesis_442 | Scymnus_suturalis_841 | 0.190 |
| Hippodamia_parenthesis_432 | Scymnus_suturalis_842 | 0.190 |
| Hippodamia_parenthesis_434 | Scymnus_suturalis_842 | 0.190 |
| Hippodamia_parenthesis_435 | Scymnus_suturalis_842 | 0.190 |
| Hippodamia_parenthesis_437 | Scymnus_suturalis_842 | 0.190 |
| Hippodamia_parenthesis_438 | Scymnus_suturalis_842 | 0.190 |
| Hippodamia_parenthesis_441 | Scymnus_suturalis_842 | 0.190 |

Supplementary Table S4 (continued)

| | | |
|---|---|---|
| Hippodamia_parenthesis_442 | Scymnus_suturalis_842 | 0.190 |
| Hippodamia_parenthesis_433 | Scymnus_suturalis_843 | 0.190 |
| Hippodamia_parenthesis_440 | Scymnus_suturalis_843 | 0.190 |
| Hippodamia_parenthesis_432 | Scymnus_suturalis_844 | 0.190 |
| Hippodamia_parenthesis_434 | Scymnus_suturalis_844 | 0.190 |
| Hippodamia_parenthesis_435 | Scymnus_suturalis_844 | 0.190 |
| Hippodamia_parenthesis_437 | Scymnus_suturalis_844 | 0.190 |
| Hippodamia_parenthesis_438 | Scymnus_suturalis_844 | 0.190 |
| Hippodamia_parenthesis_441 | Scymnus_suturalis_844 | 0.190 |
| Hippodamia_parenthesis_442 | Scymnus_suturalis_844 | 0.190 |
| Hippodamia_parenthesis_432 | Scymnus_suturalis_846 | 0.190 |
| Hippodamia_parenthesis_434 | Scymnus_suturalis_846 | 0.190 |
| Hippodamia_parenthesis_435 | Scymnus_suturalis_846 | 0.190 |
| Hippodamia_parenthesis_437 | Scymnus_suturalis_846 | 0.190 |
| Hippodamia_parenthesis_438 | Scymnus_suturalis_846 | 0.190 |
| Hippodamia_parenthesis_441 | Scymnus_suturalis_846 | 0.190 |
| Hippodamia_parenthesis_442 | Scymnus_suturalis_846 | 0.190 |
| Hippodamia_parenthesis_432 | Scymnus_suturalis_847 | 0.190 |
| Hippodamia_parenthesis_434 | Scymnus_suturalis_847 | 0.190 |
| Hippodamia_parenthesis_435 | Scymnus_suturalis_847 | 0.190 |
| Hippodamia_parenthesis_437 | Scymnus_suturalis_847 | 0.190 |
| Hippodamia_parenthesis_438 | Scymnus_suturalis_847 | 0.190 |
| Hippodamia_parenthesis_441 | Scymnus_suturalis_847 | 0.190 |
| Hippodamia_parenthesis_442 | Scymnus_suturalis_847 | 0.190 |
| Hippodamia_parenthesis_432 | Scymnus_suturalis_848 | 0.190 |
| Hippodamia_parenthesis_434 | Scymnus_suturalis_848 | 0.190 |
| Hippodamia_parenthesis_435 | Scymnus_suturalis_848 | 0.190 |
| Hippodamia_parenthesis_437 | Scymnus_suturalis_848 | 0.190 |
| Hippodamia_parenthesis_438 | Scymnus_suturalis_848 | 0.190 |
| Hippodamia_parenthesis_441 | Scymnus_suturalis_848 | 0.190 |
| Hippodamia_parenthesis_442 | Scymnus_suturalis_848 | 0.190 |
| Hyperaspis_lateralis_655 | Stethorus_punctillum_851 | 0.190 |
| Hyperaspis_lateralis_654 | Stethorus_punctillum_853 | 0.190 |
| Hyperaspis_lateralis_655 | Stethorus_punctillum_858 | 0.190 |
| Scymnus_nubilus_800 | Subcoccinella_vigintiquatuorpunctata_620 | 0.190 |
| Scymnus_nubilus_801 | Subcoccinella_vigintiquatuorpunctata_620 | 0.190 |
| Scymnus_nubilus_802 | Subcoccinella_vigintiquatuorpunctata_620 | 0.190 |
| Scymnus_nubilus_805 | Subcoccinella_vigintiquatuorpunctata_620 | 0.190 |
| Scymnus_nubilus_806 | Subcoccinella_vigintiquatuorpunctata_620 | 0.190 |
| Scymnus_nubilus_807 | Subcoccinella_vigintiquatuorpunctata_620 | 0.190 |
| Scymnus_nubilus_810 | Subcoccinella_vigintiquatuorpunctata_620 | 0.190 |
| Scymnus_nubilus_800 | Subcoccinella_vigintiquatuorpunctata_621 | 0.190 |
| Scymnus_nubilus_801 | Subcoccinella_vigintiquatuorpunctata_621 | 0.190 |
| Scymnus_nubilus_802 | Subcoccinella_vigintiquatuorpunctata_621 | 0.190 |
| Scymnus_nubilus_805 | Subcoccinella_vigintiquatuorpunctata_621 | 0.190 |
| Scymnus_nubilus_806 | Subcoccinella_vigintiquatuorpunctata_621 | 0.190 |
| Scymnus_nubilus_807 | Subcoccinella_vigintiquatuorpunctata_621 | 0.190 |
| Scymnus_nubilus_810 | Subcoccinella_vigintiquatuorpunctata_621 | 0.190 |
| Hyperaspis_binotata_650 | Subcoccinella_vigintiquatuorpunctata_622 | 0.190 |
| Scymnus_nubilus_798 | Subcoccinella_vigintiquatuorpunctata_622 | 0.190 |
| Scymnus_nubilus_808 | Subcoccinella_vigintiquatuorpunctata_622 | 0.190 |
| Scymnus_nubilus_799 | Subcoccinella_vigintiquatuorpunctata_623 | 0.190 |
| Scymnus_nubilus_798 | Subcoccinella_vigintiquatuorpunctata_625 | 0.190 |
| Scymnus_nubilus_808 | Subcoccinella_vigintiquatuorpunctata_625 | 0.190 |
| Scymnus_nubilus_796 | Subcoccinella_vigintiquatuorpunctata_627 | 0.190 |
| Scymnus_nubilus_803 | Subcoccinella_vigintiquatuorpunctata_627 | 0.190 |

| | | |
|---|---|---|
| Scymnus_nubilus_804 | Subcoccinella_vigintiquatuorpunctata_627 | 0.190 |
| Scymnus_nubilus_809 | Subcoccinella_vigintiquatuorpunctata_627 | 0.190 |
| Scymnus_nubilus_800 | Subcoccinella_vigintiquatuorpunctata_628 | 0.190 |
| Scymnus_nubilus_801 | Subcoccinella_vigintiquatuorpunctata_628 | 0.190 |
| Scymnus_nubilus_802 | Subcoccinella_vigintiquatuorpunctata_628 | 0.190 |
| Scymnus_nubilus_805 | Subcoccinella_vigintiquatuorpunctata_628 | 0.190 |
| Scymnus_nubilus_806 | Subcoccinella_vigintiquatuorpunctata_628 | 0.190 |
| Scymnus_nubilus_807 | Subcoccinella_vigintiquatuorpunctata_628 | 0.190 |
| Scymnus_nubilus_810 | Subcoccinella_vigintiquatuorpunctata_628 | 0.190 |
| Scymnus_nubilus_800 | Subcoccinella_vigintiquatuorpunctata_629 | 0.190 |
| Scymnus_nubilus_801 | Subcoccinella_vigintiquatuorpunctata_629 | 0.190 |
| Scymnus_nubilus_802 | Subcoccinella_vigintiquatuorpunctata_629 | 0.190 |
| Scymnus_nubilus_805 | Subcoccinella_vigintiquatuorpunctata_629 | 0.190 |
| Scymnus_nubilus_806 | Subcoccinella_vigintiquatuorpunctata_629 | 0.190 |
| Scymnus_nubilus_807 | Subcoccinella_vigintiquatuorpunctata_629 | 0.190 |
| Scymnus_nubilus_810 | Subcoccinella_vigintiquatuorpunctata_629 | 0.190 |
| Scymnus_nubilus_798 | Subcoccinella_vigintiquatuorpunctata_630 | 0.190 |
| Scymnus_nubilus_808 | Subcoccinella_vigintiquatuorpunctata_630 | 0.190 |
| Scymnus_nubilus_800 | Subcoccinella_vigintiquatuorpunctata_631 | 0.190 |
| Scymnus_nubilus_801 | Subcoccinella_vigintiquatuorpunctata_631 | 0.190 |
| Scymnus_nubilus_802 | Subcoccinella_vigintiquatuorpunctata_631 | 0.190 |
| Scymnus_nubilus_805 | Subcoccinella_vigintiquatuorpunctata_631 | 0.190 |
| Scymnus_nubilus_806 | Subcoccinella_vigintiquatuorpunctata_631 | 0.190 |
| Scymnus_nubilus_807 | Subcoccinella_vigintiquatuorpunctata_631 | 0.190 |
| Scymnus_nubilus_810 | Subcoccinella_vigintiquatuorpunctata_631 | 0.190 |
| Hippodamia_undecimnotata_460 | Tytthaspis_sedecimpunctata_589 | 0.190 |
| Hippodamia_undecimnotata_461 | Tytthaspis_sedecimpunctata_589 | 0.190 |
| Hippodamia_undecimnotata_462 | Tytthaspis_sedecimpunctata_589 | 0.190 |
| Hippodamia_undecimnotata_463 | Tytthaspis_sedecimpunctata_589 | 0.190 |
| Hippodamia_undecimnotata_464 | Tytthaspis_sedecimpunctata_589 | 0.190 |
| Hippodamia_undecimnotata_465 | Tytthaspis_sedecimpunctata_589 | 0.190 |
| Hippodamia_undecimnotata_466 | Tytthaspis_sedecimpunctata_589 | 0.190 |
| Hippodamia_parenthesis_432 | Scymnus_suturalis_832 | 0.190 |
| Hippodamia_parenthesis_434 | Scymnus_suturalis_832 | 0.190 |
| Hippodamia_parenthesis_435 | Scymnus_suturalis_832 | 0.190 |
| Hippodamia_parenthesis_437 | Scymnus_suturalis_832 | 0.190 |
| Hippodamia_parenthesis_438 | Scymnus_suturalis_832 | 0.190 |
| Hippodamia_parenthesis_441 | Scymnus_suturalis_832 | 0.190 |
| Hippodamia_parenthesis_442 | Scymnus_suturalis_832 | 0.190 |
| Hippodamia_parenthesis_439 | Scymnus_suturalis_833 | 0.190 |
| Hippodamia_parenthesis_433 | Scymnus_suturalis_834 | 0.190 |
| Hippodamia_parenthesis_440 | Scymnus_suturalis_834 | 0.190 |
| Hippodamia_parenthesis_432 | Scymnus_suturalis_835 | 0.190 |
| Hippodamia_parenthesis_434 | Scymnus_suturalis_835 | 0.190 |
| Hippodamia_parenthesis_435 | Scymnus_suturalis_835 | 0.190 |
| Hippodamia_parenthesis_437 | Scymnus_suturalis_835 | 0.190 |
| Hippodamia_parenthesis_438 | Scymnus_suturalis_835 | 0.190 |
| Hippodamia_parenthesis_441 | Scymnus_suturalis_835 | 0.190 |
| Hippodamia_parenthesis_442 | Scymnus_suturalis_835 | 0.190 |
| Hippodamia_parenthesis_432 | Scymnus_suturalis_836 | 0.190 |
| Hippodamia_parenthesis_434 | Scymnus_suturalis_836 | 0.190 |
| Hippodamia_parenthesis_435 | Scymnus_suturalis_836 | 0.190 |
| Hippodamia_parenthesis_437 | Scymnus_suturalis_836 | 0.190 |
| Hippodamia_parenthesis_438 | Scymnus_suturalis_836 | 0.190 |
| Hippodamia_parenthesis_441 | Scymnus_suturalis_836 | 0.190 |
| Hippodamia_parenthesis_442 | Scymnus_suturalis_836 | 0.190 |

Supplementary Table S4 (continued)

| | | |
|---|---|---|
| Hippodamia_parenthesis_432 | Scymnus_suturalis_837 | 0.190 |
| Hippodamia_parenthesis_434 | Scymnus_suturalis_837 | 0.190 |
| Hippodamia_parenthesis_435 | Scymnus_suturalis_837 | 0.190 |
| Hippodamia_parenthesis_437 | Scymnus_suturalis_837 | 0.190 |
| Hippodamia_parenthesis_438 | Scymnus_suturalis_837 | 0.190 |
| Hippodamia_parenthesis_441 | Scymnus_suturalis_837 | 0.190 |
| Hippodamia_parenthesis_442 | Scymnus_suturalis_837 | 0.190 |
| Hippodamia_parenthesis_439 | Scymnus_suturalis_838 | 0.190 |
| Hippodamia_parenthesis_432 | Scymnus_suturalis_839 | 0.190 |
| Hippodamia_parenthesis_434 | Scymnus_suturalis_839 | 0.190 |
| Hippodamia_parenthesis_435 | Scymnus_suturalis_839 | 0.190 |
| Hippodamia_parenthesis_437 | Scymnus_suturalis_839 | 0.190 |
| Hippodamia_parenthesis_438 | Scymnus_suturalis_839 | 0.190 |
| Hippodamia_parenthesis_441 | Scymnus_suturalis_839 | 0.190 |
| Hippodamia_parenthesis_442 | Scymnus_suturalis_839 | 0.190 |
| Hippodamia_parenthesis_439 | Scymnus_suturalis_840 | 0.190 |
| Scymnus_apetzi_692 | Subcoccinella_vigintiquatuorpunctata_622 | 0.190 |
| Scymnus_apetzi_692 | Subcoccinella_vigintiquatuorpunctata_626 | 0.190 |
| Scymnus_nigrinus_787 | Tytthaspis_sedecimpunctata_580 | 0.190 |
| Diomus_terminatus_647 | Scymnus_sinuanodulus_831 | 0.190 |
| Diomus_terminatus_648 | Scymnus_sinuanodulus_831 | 0.190 |
| Scymnus_camptodromus_715 | Subcoccinella_vigintiquatuorpunctata_620 | 0.190 |
| Scymnus_camptodromus_715 | Subcoccinella_vigintiquatuorpunctata_621 | 0.190 |
| Scymnus_camptodromus_715 | Subcoccinella_vigintiquatuorpunctata_627 | 0.190 |
| Scymnus_camptodromus_715 | Subcoccinella_vigintiquatuorpunctata_628 | 0.190 |
| Scymnus_camptodromus_715 | Subcoccinella_vigintiquatuorpunctata_629 | 0.190 |
| Scymnus_camptodromus_715 | Subcoccinella_vigintiquatuorpunctata_630 | 0.190 |
| Scymnus_camptodromus_715 | Subcoccinella_vigintiquatuorpunctata_631 | 0.190 |
| Hippodamia_parenthesis_439 | Scymnus_suturalis_845 | 0.190 |
| Scymnus_nubilus_797 | Subcoccinella_vigintiquatuorpunctata_622 | 0.190 |
| Scymnus_nubilus_797 | Subcoccinella_vigintiquatuorpunctata_624 | 0.190 |
| Scymnus_nubilus_797 | Subcoccinella_vigintiquatuorpunctata_625 | 0.190 |
| Scymnus_nubilus_799 | Subcoccinella_vigintiquatuorpunctata_626 | 0.190 |
| Scymnus_nubilus_797 | Subcoccinella_vigintiquatuorpunctata_630 | 0.190 |
| Scymnus_auritus_701 | Tytthaspis_sedecimpunctata_584 | 0.190 |
| Hippodamia_undecimnotata_460 | Tytthaspis_sedecimpunctata_588 | 0.190 |
| Hippodamia_undecimnotata_461 | Tytthaspis_sedecimpunctata_588 | 0.190 |
| Hippodamia_undecimnotata_462 | Tytthaspis_sedecimpunctata_588 | 0.190 |
| Hippodamia_undecimnotata_463 | Tytthaspis_sedecimpunctata_588 | 0.190 |
| Hippodamia_undecimnotata_464 | Tytthaspis_sedecimpunctata_588 | 0.190 |
| Hippodamia_undecimnotata_465 | Tytthaspis_sedecimpunctata_588 | 0.190 |
| Hippodamia_undecimnotata_466 | Tytthaspis_sedecimpunctata_588 | 0.190 |
| Scymnus_auritus_701 | Tytthaspis_sedecimpunctata_591 | 0.190 |
| Calvia_quatuordecimguttata_201 | Scymnus_sinuanodulus_828 | 0.190 |
| Exochomus_nigromaculatus_49 | Scymnus_suturalis_833 | 0.190 |
| Henosepilachna_argus_615 | Scymnus_suturalis_833 | 0.190 |
| Henosepilachna_argus_617 | Scymnus_suturalis_833 | 0.190 |
| Henosepilachna_argus_618 | Scymnus_suturalis_833 | 0.190 |
| Henosepilachna_argus_615 | Scymnus_suturalis_838 | 0.190 |
| Henosepilachna_argus_617 | Scymnus_suturalis_838 | 0.190 |
| Henosepilachna_argus_618 | Scymnus_suturalis_838 | 0.190 |
| Henosepilachna_argus_615 | Scymnus_suturalis_840 | 0.190 |
| Henosepilachna_argus_617 | Scymnus_suturalis_840 | 0.190 |
| Henosepilachna_argus_618 | Scymnus_suturalis_840 | 0.190 |
| Brumus_quadripustulatus_10 | Scymnus_sinuanodulus_830 | 0.190 |
| Chilocorus_nigritus_30 | Scymnus_sinuanodulus_828 | 0.190 |

| | | |
|---|---|---|
| Coccidula_scutellata_80 | Subcoccinella_vigintiquatuorpunctata_620 | 0.190 |
| Coccidula_scutellata_80 | Subcoccinella_vigintiquatuorpunctata_621 | 0.190 |
| Coccidula_scutellata_80 | Subcoccinella_vigintiquatuorpunctata_622 | 0.190 |
| Rhyzobius_litura_97 | Subcoccinella_vigintiquatuorpunctata_625 | 0.190 |
| Coccidula_scutellata_78 | Subcoccinella_vigintiquatuorpunctata_627 | 0.190 |
| Coccidula_scutellata_79 | Subcoccinella_vigintiquatuorpunctata_627 | 0.190 |
| Coccidula_scutellata_80 | Subcoccinella_vigintiquatuorpunctata_628 | 0.190 |
| Coccidula_scutellata_80 | Subcoccinella_vigintiquatuorpunctata_629 | 0.190 |
| Coccidula_scutellata_78 | Subcoccinella_vigintiquatuorpunctata_630 | 0.190 |
| Coccidula_scutellata_79 | Subcoccinella_vigintiquatuorpunctata_630 | 0.190 |
| Coccidula_scutellata_80 | Subcoccinella_vigintiquatuorpunctata_631 | 0.190 |
| Henosepilachna_argus_615 | Scymnus_suturalis_834 | 0.190 |
| Henosepilachna_argus_617 | Scymnus_suturalis_834 | 0.190 |
| Henosepilachna_argus_618 | Scymnus_suturalis_834 | 0.190 |
| Henosepilachna_argus_615 | Scymnus_suturalis_837 | 0.190 |
| Henosepilachna_argus_617 | Scymnus_suturalis_837 | 0.190 |
| Henosepilachna_argus_618 | Scymnus_suturalis_837 | 0.190 |
| Psyllobora_vigintimaculata_573 | Vibidia_duodecimguttata_597 | 0.190 |
| Psyllobora_vigintimaculata_573 | Vibidia_duodecimguttata_600 | 0.190 |
| Hyperaspis_reppensis_662 | Scymnus_sinuanodulus_831 | 0.190 |
| Hyperaspis_reppensis_663 | Scymnus_sinuanodulus_831 | 0.190 |
| Hippodamia_parenthesis_436 | Scymnus_suturalis_833 | 0.190 |
| Hippodamia_parenthesis_436 | Scymnus_suturalis_838 | 0.190 |
| Hippodamia_parenthesis_436 | Scymnus_suturalis_840 | 0.190 |
| Hippodamia_convergens_400 | Scymnus_suturalis_841 | 0.190 |
| Hippodamia_convergens_400 | Scymnus_suturalis_842 | 0.190 |
| Hippodamia_convergens_400 | Scymnus_suturalis_844 | 0.190 |
| Hippodamia_convergens_400 | Scymnus_suturalis_846 | 0.190 |
| Hippodamia_convergens_400 | Scymnus_suturalis_847 | 0.190 |
| Hippodamia_convergens_400 | Scymnus_suturalis_848 | 0.190 |
| Scymnus_haemorrhoidalis_755 | Subcoccinella_vigintiquatuorpunctata_622 | 0.190 |
| Scymnus_haemorrhoidalis_756 | Subcoccinella_vigintiquatuorpunctata_622 | 0.190 |
| Scymnus_mitior_779 | Subcoccinella_vigintiquatuorpunctata_630 | 0.190 |
| Scymnus_mitior_780 | Subcoccinella_vigintiquatuorpunctata_630 | 0.190 |
| Hippodamia_parenthesis_439 | Vibidia_duodecimguttata_596 | 0.190 |
| Coccinella_transversoguttata_267 | Vibidia_duodecimguttata_603 | 0.190 |
| Coccinella_transversoguttata_268 | Vibidia_duodecimguttata_603 | 0.190 |
| Coccinella_transversoguttata_269 | Vibidia_duodecimguttata_603 | 0.190 |
| Coccinella_transversoguttata_270 | Vibidia_duodecimguttata_603 | 0.190 |
| Coccinella_transversoguttata_271 | Vibidia_duodecimguttata_603 | 0.190 |
| Calvia_quatuordecimguttata_209 | Scymnus_sinuanodulus_830 | 0.190 |
| Hippodamia_convergens_400 | Scymnus_suturalis_832 | 0.190 |
| Hippodamia_convergens_400 | Scymnus_suturalis_835 | 0.190 |
| Hippodamia_convergens_400 | Scymnus_suturalis_836 | 0.190 |
| Scymnus_nubilus_796 | Scymnus_suturalis_838 | 0.190 |
| Scymnus_nubilus_798 | Scymnus_suturalis_838 | 0.190 |
| Scymnus_nubilus_800 | Scymnus_suturalis_838 | 0.190 |
| Scymnus_nubilus_801 | Scymnus_suturalis_838 | 0.190 |
| Scymnus_nubilus_802 | Scymnus_suturalis_838 | 0.190 |
| Scymnus_nubilus_805 | Scymnus_suturalis_838 | 0.190 |
| Scymnus_nubilus_806 | Scymnus_suturalis_838 | 0.190 |
| Scymnus_nubilus_807 | Scymnus_suturalis_838 | 0.190 |
| Scymnus_nubilus_810 | Scymnus_suturalis_838 | 0.190 |
| Hippodamia_convergens_400 | Scymnus_suturalis_839 | 0.190 |
| Scymnus_nubilus_798 | Scymnus_suturalis_840 | 0.190 |
| Hippodamia_parenthesis_436 | Scymnus_suturalis_845 | 0.190 |

| | | |
|---|---|---|
| Scymnus_apetzi_692 | Subcoccinella_vigintiquatuorpunctata_619 | 0.190 |
| Scymnus_nubilus_803 | Scymnus_suturalis_841 | 0.190 |
| Scymnus_nubilus_804 | Scymnus_suturalis_841 | 0.190 |
| Scymnus_nubilus_808 | Scymnus_suturalis_841 | 0.190 |
| Scymnus_nubilus_809 | Scymnus_suturalis_841 | 0.190 |
| Scymnus_nubilus_803 | Scymnus_suturalis_842 | 0.190 |
| Scymnus_nubilus_804 | Scymnus_suturalis_842 | 0.190 |
| Scymnus_nubilus_808 | Scymnus_suturalis_842 | 0.190 |
| Scymnus_nubilus_809 | Scymnus_suturalis_842 | 0.190 |
| Scymnus_nubilus_803 | Scymnus_suturalis_844 | 0.190 |
| Scymnus_nubilus_804 | Scymnus_suturalis_844 | 0.190 |
| Scymnus_nubilus_808 | Scymnus_suturalis_844 | 0.190 |
| Scymnus_nubilus_809 | Scymnus_suturalis_844 | 0.190 |
| Scymnus_nubilus_803 | Scymnus_suturalis_846 | 0.190 |
| Scymnus_nubilus_804 | Scymnus_suturalis_846 | 0.190 |
| Scymnus_nubilus_808 | Scymnus_suturalis_846 | 0.190 |
| Scymnus_nubilus_809 | Scymnus_suturalis_846 | 0.190 |
| Scymnus_nubilus_803 | Scymnus_suturalis_847 | 0.190 |
| Scymnus_nubilus_804 | Scymnus_suturalis_847 | 0.190 |
| Scymnus_nubilus_808 | Scymnus_suturalis_847 | 0.190 |
| Scymnus_nubilus_809 | Scymnus_suturalis_847 | 0.190 |
| Scymnus_nubilus_803 | Scymnus_suturalis_848 | 0.190 |
| Scymnus_nubilus_804 | Scymnus_suturalis_848 | 0.190 |
| Scymnus_nubilus_808 | Scymnus_suturalis_848 | 0.190 |
| Scymnus_nubilus_809 | Scymnus_suturalis_848 | 0.190 |
| Scymnus_haemorrhoidalis_751 | Subcoccinella_vigintiquatuorpunctata_622 | 0.190 |
| Scymnus_haemorrhoidalis_754 | Subcoccinella_vigintiquatuorpunctata_622 | 0.190 |
| Scymnus_haemorrhoidalis_757 | Subcoccinella_vigintiquatuorpunctata_622 | 0.190 |
| Scymnus_haemorrhoidalis_758 | Subcoccinella_vigintiquatuorpunctata_622 | 0.190 |
| Aphidecta_obliterata_183 | Vibidia_duodecimguttata_596 | 0.190 |
| Adalia_bipunctata_98 | Vibidia_duodecimguttata_597 | 0.190 |
| Aphidecta_obliterata_183 | Vibidia_duodecimguttata_598 | 0.190 |
| Aphidecta_obliterata_185 | Vibidia_duodecimguttata_599 | 0.190 |
| Aphidecta_obliterata_186 | Vibidia_duodecimguttata_599 | 0.190 |
| Adalia_bipunctata_98 | Vibidia_duodecimguttata_600 | 0.190 |
| Aphidecta_obliterata_185 | Vibidia_duodecimguttata_601 | 0.190 |
| Aphidecta_obliterata_186 | Vibidia_duodecimguttata_601 | 0.190 |
| Aphidecta_obliterata_185 | Vibidia_duodecimguttata_602 | 0.190 |
| Aphidecta_obliterata_186 | Vibidia_duodecimguttata_602 | 0.190 |
| Aphidecta_obliterata_183 | Vibidia_duodecimguttata_603 | 0.190 |
| Scymnus_nubilus_803 | Scymnus_suturalis_832 | 0.190 |
| Scymnus_nubilus_804 | Scymnus_suturalis_832 | 0.190 |
| Scymnus_nubilus_808 | Scymnus_suturalis_832 | 0.190 |
| Scymnus_nubilus_809 | Scymnus_suturalis_832 | 0.190 |
| Scymnus_nubilus_797 | Scymnus_suturalis_833 | 0.190 |
| Scymnus_nubilus_803 | Scymnus_suturalis_835 | 0.190 |
| Scymnus_nubilus_804 | Scymnus_suturalis_835 | 0.190 |
| Scymnus_nubilus_808 | Scymnus_suturalis_835 | 0.190 |
| Scymnus_nubilus_809 | Scymnus_suturalis_835 | 0.190 |
| Scymnus_nubilus_803 | Scymnus_suturalis_836 | 0.190 |
| Scymnus_nubilus_804 | Scymnus_suturalis_836 | 0.190 |
| Scymnus_nubilus_808 | Scymnus_suturalis_836 | 0.190 |
| Scymnus_nubilus_809 | Scymnus_suturalis_836 | 0.190 |
| Scymnus_nubilus_803 | Scymnus_suturalis_839 | 0.190 |
| Scymnus_nubilus_804 | Scymnus_suturalis_839 | 0.190 |
| Scymnus_nubilus_808 | Scymnus_suturalis_839 | 0.190 |

| | | |
|---|---|---|
| Scymnus_nubilus_809 | Scymnus_suturalis_839 | 0.190 |
| Coelophora_inaequalis_319 | Vibidia_duodecimguttata_596 | 0.190 |
| Coccinella_undecimpunctata_287 | Vibidia_duodecimguttata_598 | 0.190 |
| Coelophora_inaequalis_319 | Vibidia_duodecimguttata_598 | 0.190 |
| Coccinella_undecimpunctata_287 | Vibidia_duodecimguttata_599 | 0.190 |
| Coccinella_undecimpunctata_287 | Vibidia_duodecimguttata_601 | 0.190 |
| Coccinella_undecimpunctata_287 | Vibidia_duodecimguttata_602 | 0.190 |
| Scymnus_nubilus_799 | Subcoccinella_vigintiquatuorpunctata_619 | 0.190 |
| Brumus_quadripustulatus_12 | Scymnus_sinuanodulus_829 | 0.190 |
| Scymnus_frontalis_746 | Scymnus_suturalis_838 | 0.190 |
| Scymnus_haemorrhoidalis_752 | Subcoccinella_vigintiquatuorpunctata_622 | 0.190 |
| Scymnus_haemorrhoidalis_753 | Subcoccinella_vigintiquatuorpunctata_622 | 0.190 |
| Coccinella_magnifica_228 | Vibidia_duodecimguttata_596 | 0.190 |
| Coccinella_magnifica_229 | Vibidia_duodecimguttata_596 | 0.190 |
| Coccinella_magnifica_230 | Vibidia_duodecimguttata_596 | 0.190 |
| Coccinella_magnifica_231 | Vibidia_duodecimguttata_596 | 0.190 |
| Coccinella_magnifica_228 | Vibidia_duodecimguttata_598 | 0.190 |
| Coccinella_magnifica_229 | Vibidia_duodecimguttata_598 | 0.190 |
| Coccinella_magnifica_230 | Vibidia_duodecimguttata_598 | 0.190 |
| Coccinella_magnifica_231 | Vibidia_duodecimguttata_598 | 0.190 |
| Harmonia_quadripunctata_376 | Vibidia_duodecimguttata_603 | 0.190 |
| Harmonia_quadripunctata_378 | Vibidia_duodecimguttata_603 | 0.190 |
| Scymnus_frontalis_746 | Scymnus_suturalis_834 | 0.190 |
| Scymnus_mitior_779 | Subcoccinella_vigintiquatuorpunctata_619 | 0.191 |
| Scymnus_mitior_780 | Subcoccinella_vigintiquatuorpunctata_619 | 0.191 |
| Calvia_quatuordecimguttata_218 | Scymnus_sinuanodulus_831 | 0.191 |
| Calvia_quatuordecimguttata_204 | Scymnus_sinuanodulus_831 | 0.191 |
| Calvia_quatuordecimguttata_208 | Scymnus_sinuanodulus_831 | 0.191 |
| Calvia_quatuordecimguttata_217 | Scymnus_sinuanodulus_831 | 0.191 |
| Scymnus_nubilus_799 | Scymnus_sinuanodulus_831 | 0.191 |
| Hippodamia_glacialis_408 | Scymnus_suturalis_841 | 0.191 |
| Hippodamia_glacialis_409 | Scymnus_suturalis_841 | 0.191 |
| Hippodamia_glacialis_408 | Scymnus_suturalis_842 | 0.191 |
| Hippodamia_glacialis_409 | Scymnus_suturalis_842 | 0.191 |
| Hippodamia_glacialis_407 | Scymnus_suturalis_843 | 0.191 |
| Hippodamia_glacialis_408 | Scymnus_suturalis_844 | 0.191 |
| Hippodamia_glacialis_409 | Scymnus_suturalis_844 | 0.191 |
| Hippodamia_glacialis_407 | Scymnus_suturalis_845 | 0.191 |
| Hippodamia_glacialis_408 | Scymnus_suturalis_846 | 0.191 |
| Hippodamia_glacialis_409 | Scymnus_suturalis_846 | 0.191 |
| Hippodamia_glacialis_408 | Scymnus_suturalis_847 | 0.191 |
| Hippodamia_glacialis_409 | Scymnus_suturalis_847 | 0.191 |
| Hippodamia_glacialis_408 | Scymnus_suturalis_848 | 0.191 |
| Hippodamia_glacialis_409 | Scymnus_suturalis_848 | 0.191 |
| Brachiacantha_rotunda_632 | Subcoccinella_vigintiquatuorpunctata_627 | 0.191 |
| Brachiacantha_rotunda_633 | Subcoccinella_vigintiquatuorpunctata_627 | 0.191 |
| Brachiacantha_rotunda_635 | Subcoccinella_vigintiquatuorpunctata_627 | 0.191 |
| Hippodamia_glacialis_408 | Scymnus_suturalis_832 | 0.191 |
| Hippodamia_glacialis_409 | Scymnus_suturalis_832 | 0.191 |
| Hippodamia_glacialis_408 | Scymnus_suturalis_835 | 0.191 |
| Hippodamia_glacialis_409 | Scymnus_suturalis_835 | 0.191 |
| Hippodamia_glacialis_408 | Scymnus_suturalis_836 | 0.191 |
| Hippodamia_glacialis_409 | Scymnus_suturalis_836 | 0.191 |
| Hippodamia_glacialis_408 | Scymnus_suturalis_839 | 0.191 |
| Hippodamia_glacialis_409 | Scymnus_suturalis_839 | 0.191 |
| Scymnus_apetzi_694 | Subcoccinella_vigintiquatuorpunctata_620 | 0.191 |

| | | |
|---|---|---|
| Scymnus_apetzi_694 | Subcoccinella_vigintiquatuorpunctata_621 | 0.191 |
| Scymnus_apetzi_694 | Subcoccinella_vigintiquatuorpunctata_624 | 0.191 |
| Scymnus_apetzi_694 | Subcoccinella_vigintiquatuorpunctata_628 | 0.191 |
| Scymnus_apetzi_694 | Subcoccinella_vigintiquatuorpunctata_629 | 0.191 |
| Scymnus_apetzi_694 | Subcoccinella_vigintiquatuorpunctata_631 | 0.191 |
| Hyperaspis_reppensis_662 | Scymnus_sinuanodulus_829 | 0.191 |
| Hyperaspis_reppensis_663 | Scymnus_sinuanodulus_829 | 0.191 |
| Calvia_quatuordecimguttata_205 | Scymnus_sinuanodulus_831 | 0.191 |
| Coccinella_trifasciata_273 | Vibidia_duodecimguttata_598 | 0.191 |
| Scymnus_apetzi_695 | Subcoccinella_vigintiquatuorpunctata_623 | 0.191 |
| Coccidula_scutellata_84 | Subcoccinella_vigintiquatuorpunctata_619 | 0.191 |
| Scymnus_camptodromus_717 | Subcoccinella_vigintiquatuorpunctata_620 | 0.191 |
| Scymnus_camptodromus_717 | Subcoccinella_vigintiquatuorpunctata_621 | 0.191 |
| Scymnus_camptodromus_717 | Subcoccinella_vigintiquatuorpunctata_627 | 0.191 |
| Scymnus_camptodromus_717 | Subcoccinella_vigintiquatuorpunctata_628 | 0.191 |
| Scymnus_camptodromus_717 | Subcoccinella_vigintiquatuorpunctata_629 | 0.191 |
| Scymnus_camptodromus_717 | Subcoccinella_vigintiquatuorpunctata_630 | 0.191 |
| Scymnus_camptodromus_717 | Subcoccinella_vigintiquatuorpunctata_631 | 0.191 |
| Chilocorus_nigritus_30 | Vibidia_duodecimguttata_596 | 0.191 |
| Chilocorus_nigritus_29 | Vibidia_duodecimguttata_598 | 0.191 |
| Chilocorus_nigritus_30 | Vibidia_duodecimguttata_599 | 0.191 |
| Chilocorus_nigritus_29 | Vibidia_duodecimguttata_601 | 0.191 |
| Chilocorus_nigritus_30 | Vibidia_duodecimguttata_602 | 0.191 |
| Chilocorus_nigritus_30 | Vibidia_duodecimguttata_603 | 0.191 |
| Scymnus_apetzi_695 | Subcoccinella_vigintiquatuorpunctata_622 | 0.191 |
| Scymnus_apetzi_695 | Subcoccinella_vigintiquatuorpunctata_626 | 0.191 |
| Brachiacantha_rotunda_632 | Subcoccinella_vigintiquatuorpunctata_619 | 0.191 |
| Brachiacantha_rotunda_633 | Subcoccinella_vigintiquatuorpunctata_619 | 0.191 |
| Brachiacantha_rotunda_635 | Subcoccinella_vigintiquatuorpunctata_619 | 0.191 |
| Chilocorus_nigritus_30 | Vibidia_duodecimguttata_597 | 0.191 |
| Chilocorus_nigritus_30 | Vibidia_duodecimguttata_600 | 0.191 |
| Scymnus_apetzi_693 | Subcoccinella_vigintiquatuorpunctata_624 | 0.191 |
| Coelophora_inaequalis_315 | Vibidia_duodecimguttata_596 | 0.191 |
| Coccinella_quinquepunctata_235 | Vibidia_duodecimguttata_597 | 0.191 |
| Coccinella_quinquepunctata_238 | Vibidia_duodecimguttata_597 | 0.191 |
| Coccinella_quinquepunctata_239 | Vibidia_duodecimguttata_597 | 0.191 |
| Coccinella_quinquepunctata_240 | Vibidia_duodecimguttata_597 | 0.191 |
| Coelophora_inaequalis_310 | Vibidia_duodecimguttata_597 | 0.191 |
| Coelophora_inaequalis_311 | Vibidia_duodecimguttata_597 | 0.191 |
| Coelophora_inaequalis_316 | Vibidia_duodecimguttata_597 | 0.191 |
| Coelophora_inaequalis_323 | Vibidia_duodecimguttata_597 | 0.191 |
| Coelophora_inaequalis_324 | Vibidia_duodecimguttata_597 | 0.191 |
| Coelophora_inaequalis_315 | Vibidia_duodecimguttata_598 | 0.191 |
| Coccinella_quinquepunctata_235 | Vibidia_duodecimguttata_600 | 0.191 |
| Coccinella_quinquepunctata_238 | Vibidia_duodecimguttata_600 | 0.191 |
| Coccinella_quinquepunctata_239 | Vibidia_duodecimguttata_600 | 0.191 |
| Coccinella_quinquepunctata_240 | Vibidia_duodecimguttata_600 | 0.191 |
| Coelophora_inaequalis_310 | Vibidia_duodecimguttata_600 | 0.191 |
| Coelophora_inaequalis_311 | Vibidia_duodecimguttata_600 | 0.191 |
| Coelophora_inaequalis_316 | Vibidia_duodecimguttata_600 | 0.191 |
| Coelophora_inaequalis_323 | Vibidia_duodecimguttata_600 | 0.191 |
| Coelophora_inaequalis_324 | Vibidia_duodecimguttata_600 | 0.191 |
| Calvia_quatuordecimguttata_218 | Scymnus_sinuanodulus_829 | 0.191 |
| Hyperaspis_reppensis_662 | Tytthaspis_sedecimpunctata_583 | 0.191 |
| Hyperaspis_reppensis_663 | Tytthaspis_sedecimpunctata_583 | 0.191 |
| Hyperaspis_reppensis_662 | Tytthaspis_sedecimpunctata_592 | 0.191 |

| | | |
|---|---|---|
| Hyperaspis_reppensis_663 | Tytthaspis_sedecimpunctata_592 | 0.191 |
| Hyperaspis_reppensis_662 | Tytthaspis_sedecimpunctata_580 | 0.191 |
| Hyperaspis_reppensis_663 | Tytthaspis_sedecimpunctata_580 | 0.191 |
| Hyperaspis_reppensis_662 | Tytthaspis_sedecimpunctata_586 | 0.191 |
| Hyperaspis_reppensis_663 | Tytthaspis_sedecimpunctata_586 | 0.191 |
| Hyperaspis_reppensis_662 | Tytthaspis_sedecimpunctata_588 | 0.191 |
| Hyperaspis_reppensis_663 | Tytthaspis_sedecimpunctata_588 | 0.191 |
| Platynaspis_luteorubra_52 | Tytthaspis_sedecimpunctata_580 | 0.191 |
| Platynaspis_luteorubra_56 | Tytthaspis_sedecimpunctata_580 | 0.191 |
| Platynaspis_luteorubra_60 | Tytthaspis_sedecimpunctata_580 | 0.191 |
| Hyperaspis_campestris_651 | Tytthaspis_sedecimpunctata_583 | 0.191 |
| Hyperaspis_campestris_652 | Tytthaspis_sedecimpunctata_583 | 0.191 |
| Hyperaspis_campestris_653 | Tytthaspis_sedecimpunctata_583 | 0.191 |
| Hyperaspis_oregona_659 | Tytthaspis_sedecimpunctata_577 | 0.191 |
| Hyperaspis_oregona_659 | Tytthaspis_sedecimpunctata_578 | 0.191 |
| Hyperaspis_oregona_659 | Tytthaspis_sedecimpunctata_579 | 0.191 |
| Platynaspis_luteorubra_54 | Tytthaspis_sedecimpunctata_580 | 0.191 |
| Platynaspis_luteorubra_57 | Tytthaspis_sedecimpunctata_580 | 0.191 |
| Platynaspis_luteorubra_59 | Tytthaspis_sedecimpunctata_580 | 0.191 |
| Hyperaspis_oregona_659 | Tytthaspis_sedecimpunctata_581 | 0.191 |
| Hyperaspis_oregona_659 | Tytthaspis_sedecimpunctata_582 | 0.191 |
| Chilocorus_renipustulatus_33 | Tytthaspis_sedecimpunctata_589 | 0.191 |
| Hyperaspis_oregona_659 | Tytthaspis_sedecimpunctata_590 | 0.191 |
| Hyperaspis_campestris_651 | Tytthaspis_sedecimpunctata_591 | 0.191 |
| Hyperaspis_campestris_652 | Tytthaspis_sedecimpunctata_591 | 0.191 |
| Hyperaspis_campestris_653 | Tytthaspis_sedecimpunctata_591 | 0.191 |
| Hyperaspis_oregona_659 | Tytthaspis_sedecimpunctata_593 | 0.191 |
| Hyperaspis_oregona_659 | Tytthaspis_sedecimpunctata_594 | 0.191 |
| Hyperaspis_oregona_659 | Tytthaspis_sedecimpunctata_595 | 0.191 |
| Hyperaspis_lateralis_656 | Tytthaspis_sedecimpunctata_577 | 0.191 |
| Hyperaspis_lateralis_656 | Tytthaspis_sedecimpunctata_578 | 0.191 |
| Hyperaspis_lateralis_656 | Tytthaspis_sedecimpunctata_579 | 0.191 |
| Hyperaspis_lateralis_656 | Tytthaspis_sedecimpunctata_581 | 0.191 |
| Hyperaspis_lateralis_656 | Tytthaspis_sedecimpunctata_582 | 0.191 |
| Hyperaspis_lateralis_656 | Tytthaspis_sedecimpunctata_590 | 0.191 |
| Hyperaspis_lateralis_656 | Tytthaspis_sedecimpunctata_592 | 0.191 |
| Hyperaspis_lateralis_656 | Tytthaspis_sedecimpunctata_594 | 0.191 |
| Hyperaspis_lateralis_656 | Tytthaspis_sedecimpunctata_595 | 0.191 |
| Calvia_quatuordecimguttata_204 | Scymnus_sinuanodulus_829 | 0.191 |
| Calvia_quatuordecimguttata_208 | Scymnus_sinuanodulus_829 | 0.191 |
| Calvia_quatuordecimguttata_217 | Scymnus_sinuanodulus_829 | 0.191 |
| Scymnus_nubilus_799 | Scymnus_sinuanodulus_829 | 0.191 |
| Chilocorus_renipustulatus_33 | Tytthaspis_sedecimpunctata_587 | 0.191 |
| Hyperaspis_oregona_659 | Tytthaspis_sedecimpunctata_587 | 0.191 |
| Chilocorus_renipustulatus_31 | Tytthaspis_sedecimpunctata_589 | 0.191 |
| Chilocorus_renipustulatus_32 | Tytthaspis_sedecimpunctata_589 | 0.191 |
| Chilocorus_renipustulatus_35 | Tytthaspis_sedecimpunctata_589 | 0.191 |
| Chilocorus_renipustulatus_36 | Tytthaspis_sedecimpunctata_589 | 0.191 |
| Chilocorus_renipustulatus_37 | Tytthaspis_sedecimpunctata_589 | 0.191 |
| Chilocorus_renipustulatus_38 | Tytthaspis_sedecimpunctata_589 | 0.191 |
| Chilocorus_renipustulatus_40 | Tytthaspis_sedecimpunctata_589 | 0.191 |
| Chilocorus_renipustulatus_41 | Tytthaspis_sedecimpunctata_589 | 0.191 |
| Chilocorus_renipustulatus_42 | Tytthaspis_sedecimpunctata_589 | 0.191 |
| Scymnus_mitior_779 | Tytthaspis_sedecimpunctata_589 | 0.191 |
| Coccinella_undecimpunctata_285 | Vibidia_duodecimguttata_596 | 0.191 |
| Coccinella_undecimpunctata_286 | Vibidia_duodecimguttata_596 | 0.191 |

| | | |
|---|---|---|
| Coccinella_undecimpunctata_288 | Vibidia_duodecimguttata_596 | 0.191 |
| Coccinella_undecimpunctata_291 | Vibidia_duodecimguttata_596 | 0.191 |
| Coccinella_quinquepunctata_235 | Vibidia_duodecimguttata_598 | 0.191 |
| Coccinella_quinquepunctata_238 | Vibidia_duodecimguttata_598 | 0.191 |
| Coccinella_quinquepunctata_239 | Vibidia_duodecimguttata_598 | 0.191 |
| Coccinella_quinquepunctata_240 | Vibidia_duodecimguttata_598 | 0.191 |
| Coccinella_quinquepunctata_235 | Vibidia_duodecimguttata_599 | 0.191 |
| Coccinella_quinquepunctata_237 | Vibidia_duodecimguttata_599 | 0.191 |
| Coccinella_quinquepunctata_238 | Vibidia_duodecimguttata_599 | 0.191 |
| Coccinella_quinquepunctata_239 | Vibidia_duodecimguttata_599 | 0.191 |
| Coccinella_quinquepunctata_240 | Vibidia_duodecimguttata_599 | 0.191 |
| Myzia_oblongoguttata_515 | Vibidia_duodecimguttata_599 | 0.191 |
| Coccinella_quinquepunctata_235 | Vibidia_duodecimguttata_601 | 0.191 |
| Coccinella_quinquepunctata_237 | Vibidia_duodecimguttata_601 | 0.191 |
| Coccinella_quinquepunctata_238 | Vibidia_duodecimguttata_601 | 0.191 |
| Coccinella_quinquepunctata_239 | Vibidia_duodecimguttata_601 | 0.191 |
| Coccinella_quinquepunctata_240 | Vibidia_duodecimguttata_601 | 0.191 |
| Coccinella_undecimpunctata_282 | Vibidia_duodecimguttata_601 | 0.191 |
| Coccinella_undecimpunctata_283 | Vibidia_duodecimguttata_601 | 0.191 |
| Coccinella_undecimpunctata_284 | Vibidia_duodecimguttata_601 | 0.191 |
| Myzia_oblongoguttata_503 | Vibidia_duodecimguttata_601 | 0.191 |
| Myzia_oblongoguttata_504 | Vibidia_duodecimguttata_601 | 0.191 |
| Myzia_oblongoguttata_505 | Vibidia_duodecimguttata_601 | 0.191 |
| Myzia_oblongoguttata_507 | Vibidia_duodecimguttata_601 | 0.191 |
| Myzia_oblongoguttata_508 | Vibidia_duodecimguttata_601 | 0.191 |
| Myzia_oblongoguttata_509 | Vibidia_duodecimguttata_601 | 0.191 |
| Myzia_oblongoguttata_510 | Vibidia_duodecimguttata_601 | 0.191 |
| Myzia_oblongoguttata_511 | Vibidia_duodecimguttata_601 | 0.191 |
| Myzia_oblongoguttata_512 | Vibidia_duodecimguttata_601 | 0.191 |
| Myzia_oblongoguttata_513 | Vibidia_duodecimguttata_601 | 0.191 |
| Myzia_oblongoguttata_514 | Vibidia_duodecimguttata_601 | 0.191 |
| Myzia_oblongoguttata_516 | Vibidia_duodecimguttata_601 | 0.191 |
| Coccinella_quinquepunctata_235 | Vibidia_duodecimguttata_602 | 0.191 |
| Coccinella_quinquepunctata_237 | Vibidia_duodecimguttata_602 | 0.191 |
| Coccinella_quinquepunctata_238 | Vibidia_duodecimguttata_602 | 0.191 |
| Coccinella_quinquepunctata_239 | Vibidia_duodecimguttata_602 | 0.191 |
| Coccinella_quinquepunctata_240 | Vibidia_duodecimguttata_602 | 0.191 |
| Myzia_oblongoguttata_515 | Vibidia_duodecimguttata_602 | 0.191 |
| Coccinella_undecimpunctata_285 | Vibidia_duodecimguttata_603 | 0.191 |
| Coccinella_undecimpunctata_286 | Vibidia_duodecimguttata_603 | 0.191 |
| Coccinella_undecimpunctata_288 | Vibidia_duodecimguttata_603 | 0.191 |
| Coelophora_inaequalis_312 | Vibidia_duodecimguttata_603 | 0.191 |
| Coelophora_inaequalis_314 | Vibidia_duodecimguttata_603 | 0.191 |
| Coelophora_inaequalis_317 | Vibidia_duodecimguttata_603 | 0.191 |
| Coelophora_inaequalis_325 | Vibidia_duodecimguttata_603 | 0.191 |
| Myzia_oblongoguttata_515 | Vibidia_duodecimguttata_603 | 0.191 |
| Scymnus_apetzi_695 | Subcoccinella_vigintiquatuorpunctata_619 | 0.191 |
| Hyperaspis_reppensis_662 | Stethorus_punctillum_849 | 0.191 |
| Hyperaspis_reppensis_663 | Stethorus_punctillum_849 | 0.191 |
| Hyperaspis_reppensis_662 | Stethorus_punctillum_854 | 0.191 |
| Hyperaspis_reppensis_663 | Stethorus_punctillum_854 | 0.191 |
| Hyperaspis_reppensis_662 | Stethorus_punctillum_856 | 0.191 |
| Hyperaspis_reppensis_663 | Stethorus_punctillum_856 | 0.191 |
| Chilocorus_renipustulatus_33 | Tytthaspis_sedecimpunctata_583 | 0.191 |
| Platynaspis_luteorubra_61 | Tytthaspis_sedecimpunctata_584 | 0.191 |
| Scymnus_mitior_780 | Tytthaspis_sedecimpunctata_586 | 0.191 |

Supplementary Table S4 (continued)

| | | |
|---|---|---|
| Scymnus_mitior_779 | Tytthaspis_sedecimpunctata_587 | 0.191 |
| Chilocorus_renipustulatus_39 | Tytthaspis_sedecimpunctata_591 | 0.191 |
| Hyperaspis_reppensis_664 | Tytthaspis_sedecimpunctata_591 | 0.191 |
| Myzia_oblongoguttata_506 | Scymnus_suturalis_843 | 0.191 |
| Myzia_oblongoguttata_506 | Scymnus_suturalis_845 | 0.191 |
| Scymnus_loewii_771 | Subcoccinella_vigintiquatuorpunctata_630 | 0.191 |
| Hyperaspis_reppensis_665 | Tytthaspis_sedecimpunctata_583 | 0.191 |
| Chilocorus_renipustulatus_33 | Tytthaspis_sedecimpunctata_584 | 0.191 |
| Brumus_quadripustulatus_3 | Scymnus_sinuanodulus_830 | 0.191 |
| Calvia_quatuordecimguttata_205 | Scymnus_sinuanodulus_829 | 0.191 |
| Chilocorus_stigma_45 | Scymnus_suturalis_841 | 0.191 |
| Chilocorus_stigma_45 | Scymnus_suturalis_842 | 0.191 |
| Chilocorus_stigma_45 | Scymnus_suturalis_844 | 0.191 |
| Chilocorus_stigma_45 | Scymnus_suturalis_846 | 0.191 |
| Chilocorus_stigma_45 | Scymnus_suturalis_847 | 0.191 |
| Chilocorus_stigma_45 | Scymnus_suturalis_848 | 0.191 |
| Hyperaspis_reppensis_662 | Stethorus_punctillum_853 | 0.191 |
| Hyperaspis_reppensis_663 | Stethorus_punctillum_853 | 0.191 |
| Coccinella_transversalis_258 | Tytthaspis_sedecimpunctata_577 | 0.191 |
| Coccinella_transversalis_258 | Tytthaspis_sedecimpunctata_578 | 0.191 |
| Coccinella_transversalis_258 | Tytthaspis_sedecimpunctata_579 | 0.191 |
| Coccinella_transversalis_258 | Tytthaspis_sedecimpunctata_581 | 0.191 |
| Coccinella_transversalis_258 | Tytthaspis_sedecimpunctata_582 | 0.191 |
| Myzia_oblongoguttata_503 | Tytthaspis_sedecimpunctata_584 | 0.191 |
| Myzia_oblongoguttata_515 | Tytthaspis_sedecimpunctata_584 | 0.191 |
| Coccinella_transversalis_258 | Tytthaspis_sedecimpunctata_590 | 0.191 |
| Coccinella_transversalis_258 | Tytthaspis_sedecimpunctata_592 | 0.191 |
| Coccinella_transversalis_258 | Tytthaspis_sedecimpunctata_594 | 0.191 |
| Coccinella_transversalis_258 | Tytthaspis_sedecimpunctata_595 | 0.191 |
| Brumus_quadripustulatus_3 | Scymnus_sinuanodulus_828 | 0.191 |
| Chilocorus_stigma_45 | Scymnus_suturalis_832 | 0.191 |
| Chilocorus_stigma_45 | Scymnus_suturalis_835 | 0.191 |
| Chilocorus_stigma_45 | Scymnus_suturalis_836 | 0.191 |
| Chilocorus_stigma_45 | Scymnus_suturalis_837 | 0.191 |
| Propylaea_quatuordecimpunctata_541 | Scymnus_suturalis_838 | 0.191 |
| Chilocorus_stigma_45 | Scymnus_suturalis_839 | 0.191 |
| Propylaea_quatuordecimpunctata_541 | Scymnus_suturalis_840 | 0.191 |
| Coelophora_inaequalis_320 | Vibidia_duodecimguttata_603 | 0.191 |
| Coelophora_inaequalis_321 | Vibidia_duodecimguttata_603 | 0.191 |
| Coelophora_inaequalis_322 | Vibidia_duodecimguttata_603 | 0.191 |
| Myrrha_octodecimguttata_499 | Scymnus_suturalis_841 | 0.191 |
| Myrrha_octodecimguttata_499 | Scymnus_suturalis_842 | 0.191 |
| Myrrha_octodecimguttata_499 | Scymnus_suturalis_844 | 0.191 |
| Myrrha_octodecimguttata_499 | Scymnus_suturalis_846 | 0.191 |
| Myrrha_octodecimguttata_499 | Scymnus_suturalis_847 | 0.191 |
| Myrrha_octodecimguttata_499 | Scymnus_suturalis_848 | 0.191 |
| Scymnus_ningshanensis_789 | Tytthaspis_sedecimpunctata_589 | 0.191 |
| Scymnus_ningshanensis_790 | Tytthaspis_sedecimpunctata_589 | 0.191 |
| Coccinella_transversalis_258 | Tytthaspis_sedecimpunctata_591 | 0.191 |
| Myrrha_octodecimguttata_499 | Scymnus_suturalis_832 | 0.191 |
| Propylaea_quatuordecimpunctata_541 | Scymnus_suturalis_834 | 0.191 |
| Myrrha_octodecimguttata_499 | Scymnus_suturalis_835 | 0.191 |
| Myrrha_octodecimguttata_499 | Scymnus_suturalis_836 | 0.191 |
| Propylaea_quatuordecimpunctata_541 | Scymnus_suturalis_837 | 0.191 |
| Myrrha_octodecimguttata_485 | Scymnus_suturalis_838 | 0.191 |
| Myrrha_octodecimguttata_486 | Scymnus_suturalis_838 | 0.191 |

| | | |
|---|---|---|
| Myrrha_octodecimguttata_487 | Scymnus_suturalis_838 | 0.191 |
| Myrrha_octodecimguttata_490 | Scymnus_suturalis_838 | 0.191 |
| Myrrha_octodecimguttata_494 | Scymnus_suturalis_838 | 0.191 |
| Myrrha_octodecimguttata_496 | Scymnus_suturalis_838 | 0.191 |
| Myrrha_octodecimguttata_497 | Scymnus_suturalis_838 | 0.191 |
| Myrrha_octodecimguttata_501 | Scymnus_suturalis_838 | 0.191 |
| Myrrha_octodecimguttata_502 | Scymnus_suturalis_838 | 0.191 |
| Nephus_redtenbacheri_678 | Scymnus_suturalis_838 | 0.191 |
| Myrrha_octodecimguttata_499 | Scymnus_suturalis_839 | 0.191 |
| Myrrha_octodecimguttata_485 | Scymnus_suturalis_840 | 0.191 |
| Myrrha_octodecimguttata_486 | Scymnus_suturalis_840 | 0.191 |
| Myrrha_octodecimguttata_487 | Scymnus_suturalis_840 | 0.191 |
| Myrrha_octodecimguttata_490 | Scymnus_suturalis_840 | 0.191 |
| Myrrha_octodecimguttata_494 | Scymnus_suturalis_840 | 0.191 |
| Myrrha_octodecimguttata_496 | Scymnus_suturalis_840 | 0.191 |
| Myrrha_octodecimguttata_497 | Scymnus_suturalis_840 | 0.191 |
| Myrrha_octodecimguttata_501 | Scymnus_suturalis_840 | 0.191 |
| Myrrha_octodecimguttata_502 | Scymnus_suturalis_840 | 0.191 |
| Nephus_redtenbacheri_678 | Scymnus_suturalis_840 | 0.191 |
| Nephus_redtenbacheri_678 | Scymnus_suturalis_843 | 0.191 |
| Hyperaspis_lateralis_654 | Subcoccinella_vigintiquatuorpunctata_620 | 0.191 |
| Hyperaspis_lateralis_654 | Subcoccinella_vigintiquatuorpunctata_621 | 0.191 |
| Hyperaspis_lateralis_656 | Subcoccinella_vigintiquatuorpunctata_627 | 0.191 |
| Hyperaspis_lateralis_657 | Subcoccinella_vigintiquatuorpunctata_627 | 0.191 |
| Hyperaspis_reppensis_662 | Subcoccinella_vigintiquatuorpunctata_627 | 0.191 |
| Hyperaspis_reppensis_663 | Subcoccinella_vigintiquatuorpunctata_627 | 0.191 |
| Hyperaspis_lateralis_654 | Subcoccinella_vigintiquatuorpunctata_628 | 0.191 |
| Hyperaspis_lateralis_654 | Subcoccinella_vigintiquatuorpunctata_629 | 0.191 |
| Hyperaspis_lateralis_656 | Subcoccinella_vigintiquatuorpunctata_630 | 0.191 |
| Hyperaspis_lateralis_657 | Subcoccinella_vigintiquatuorpunctata_630 | 0.191 |
| Hyperaspis_lateralis_654 | Subcoccinella_vigintiquatuorpunctata_631 | 0.191 |
| Scymnus_loewii_772 | Tytthaspis_sedecimpunctata_584 | 0.191 |
| Scymnus_ningshanensis_788 | Tytthaspis_sedecimpunctata_587 | 0.191 |
| Scymnus_ningshanensis_791 | Tytthaspis_sedecimpunctata_587 | 0.191 |
| Scymnus_ningshanensis_792 | Tytthaspis_sedecimpunctata_587 | 0.191 |
| Scymnus_ningshanensis_793 | Tytthaspis_sedecimpunctata_587 | 0.191 |
| Scymnus_ningshanensis_794 | Tytthaspis_sedecimpunctata_587 | 0.191 |
| Scymnus_ningshanensis_795 | Tytthaspis_sedecimpunctata_587 | 0.191 |
| Coccinella_septempunctata_241 | Tytthaspis_sedecimpunctata_589 | 0.191 |
| Coccinella_septempunctata_243 | Tytthaspis_sedecimpunctata_589 | 0.191 |
| Coccinella_septempunctata_253 | Tytthaspis_sedecimpunctata_589 | 0.191 |
| Myzia_pullata_519 | Tytthaspis_sedecimpunctata_589 | 0.191 |
| Myzia_pullata_519 | Tytthaspis_sedecimpunctata_593 | 0.191 |
| Scymnus_nebulosus_781 | Tytthaspis_sedecimpunctata_593 | 0.191 |
| Diomus_terminatus_647 | Scymnus_sinuanodulus_830 | 0.191 |
| Diomus_terminatus_648 | Scymnus_sinuanodulus_830 | 0.191 |
| Myrrha_octodecimguttata_488 | Scymnus_suturalis_833 | 0.191 |
| Myrrha_octodecimguttata_491 | Scymnus_suturalis_833 | 0.191 |
| Myrrha_octodecimguttata_492 | Scymnus_suturalis_833 | 0.191 |
| Nephus_redtenbacheri_676 | Scymnus_suturalis_833 | 0.191 |
| Nephus_redtenbacheri_677 | Scymnus_suturalis_833 | 0.191 |
| Nephus_redtenbacheri_680 | Scymnus_suturalis_833 | 0.191 |
| Nephus_redtenbacheri_681 | Scymnus_suturalis_833 | 0.191 |
| Propylaea_quatuordecimpunctata_541 | Scymnus_suturalis_833 | 0.191 |
| Myrrha_octodecimguttata_485 | Scymnus_suturalis_834 | 0.191 |
| Myrrha_octodecimguttata_486 | Scymnus_suturalis_834 | 0.191 |

Supplementary Table S4 (continued)

| | | |
|---|---|---|
| Myrrha_octodecimguttata_487 | Scymnus_suturalis_834 | 0.191 |
| Myrrha_octodecimguttata_490 | Scymnus_suturalis_834 | 0.191 |
| Myrrha_octodecimguttata_494 | Scymnus_suturalis_834 | 0.191 |
| Myrrha_octodecimguttata_496 | Scymnus_suturalis_834 | 0.191 |
| Myrrha_octodecimguttata_497 | Scymnus_suturalis_834 | 0.191 |
| Myrrha_octodecimguttata_501 | Scymnus_suturalis_834 | 0.191 |
| Myrrha_octodecimguttata_502 | Scymnus_suturalis_834 | 0.191 |
| Myrrha_octodecimguttata_485 | Scymnus_suturalis_837 | 0.191 |
| Myrrha_octodecimguttata_486 | Scymnus_suturalis_837 | 0.191 |
| Myrrha_octodecimguttata_487 | Scymnus_suturalis_837 | 0.191 |
| Myrrha_octodecimguttata_490 | Scymnus_suturalis_837 | 0.191 |
| Myrrha_octodecimguttata_494 | Scymnus_suturalis_837 | 0.191 |
| Myrrha_octodecimguttata_496 | Scymnus_suturalis_837 | 0.191 |
| Myrrha_octodecimguttata_497 | Scymnus_suturalis_837 | 0.191 |
| Myrrha_octodecimguttata_501 | Scymnus_suturalis_837 | 0.191 |
| Myrrha_octodecimguttata_502 | Scymnus_suturalis_837 | 0.191 |
| Nephus_redtenbacheri_679 | Scymnus_suturalis_838 | 0.191 |
| Nephus_redtenbacheri_679 | Scymnus_suturalis_840 | 0.191 |
| Nephus_redtenbacheri_676 | Scymnus_suturalis_841 | 0.192 |
| Nephus_redtenbacheri_677 | Scymnus_suturalis_841 | 0.192 |
| Nephus_redtenbacheri_680 | Scymnus_suturalis_841 | 0.192 |
| Nephus_redtenbacheri_681 | Scymnus_suturalis_841 | 0.192 |
| Nephus_redtenbacheri_676 | Scymnus_suturalis_842 | 0.192 |
| Nephus_redtenbacheri_677 | Scymnus_suturalis_842 | 0.192 |
| Nephus_redtenbacheri_680 | Scymnus_suturalis_842 | 0.192 |
| Nephus_redtenbacheri_681 | Scymnus_suturalis_842 | 0.192 |
| Nephus_redtenbacheri_679 | Scymnus_suturalis_843 | 0.192 |
| Nephus_redtenbacheri_676 | Scymnus_suturalis_844 | 0.192 |
| Nephus_redtenbacheri_677 | Scymnus_suturalis_844 | 0.192 |
| Nephus_redtenbacheri_680 | Scymnus_suturalis_844 | 0.192 |
| Nephus_redtenbacheri_681 | Scymnus_suturalis_844 | 0.192 |
| Nephus_redtenbacheri_674 | Scymnus_suturalis_845 | 0.192 |
| Nephus_redtenbacheri_675 | Scymnus_suturalis_845 | 0.192 |
| Nephus_redtenbacheri_676 | Scymnus_suturalis_846 | 0.192 |
| Nephus_redtenbacheri_677 | Scymnus_suturalis_846 | 0.192 |
| Nephus_redtenbacheri_680 | Scymnus_suturalis_846 | 0.192 |
| Nephus_redtenbacheri_681 | Scymnus_suturalis_846 | 0.192 |
| Nephus_redtenbacheri_676 | Scymnus_suturalis_847 | 0.192 |
| Nephus_redtenbacheri_677 | Scymnus_suturalis_847 | 0.192 |
| Nephus_redtenbacheri_680 | Scymnus_suturalis_847 | 0.192 |
| Nephus_redtenbacheri_681 | Scymnus_suturalis_847 | 0.192 |
| Nephus_redtenbacheri_676 | Scymnus_suturalis_848 | 0.192 |
| Nephus_redtenbacheri_677 | Scymnus_suturalis_848 | 0.192 |
| Nephus_redtenbacheri_680 | Scymnus_suturalis_848 | 0.192 |
| Nephus_redtenbacheri_681 | Scymnus_suturalis_848 | 0.192 |
| Hyperaspis_proba_661 | Stethorus_punctillum_849 | 0.192 |
| Hyperaspis_proba_661 | Stethorus_punctillum_857 | 0.192 |
| Hyperaspis_oregona_658 | Stethorus_punctillum_859 | 0.192 |
| Hyperaspis_oregona_659 | Stethorus_punctillum_859 | 0.192 |
| Hyperaspis_proba_661 | Stethorus_punctillum_860 | 0.192 |
| Scymnus_ningshanensis_788 | Subcoccinella_vigintiquatuorpunctata_620 | 0.192 |
| Scymnus_ningshanensis_791 | Subcoccinella_vigintiquatuorpunctata_620 | 0.192 |
| Scymnus_ningshanensis_792 | Subcoccinella_vigintiquatuorpunctata_620 | 0.192 |
| Scymnus_ningshanensis_793 | Subcoccinella_vigintiquatuorpunctata_620 | 0.192 |
| Scymnus_ningshanensis_794 | Subcoccinella_vigintiquatuorpunctata_620 | 0.192 |
| Scymnus_ningshanensis_795 | Subcoccinella_vigintiquatuorpunctata_620 | 0.192 |

| | | |
|---|---|---|
| Scymnus_ningshanensis_788 | Subcoccinella_vigintiquatuorpunctata_621 | 0.192 |
| Scymnus_ningshanensis_791 | Subcoccinella_vigintiquatuorpunctata_621 | 0.192 |
| Scymnus_ningshanensis_792 | Subcoccinella_vigintiquatuorpunctata_621 | 0.192 |
| Scymnus_ningshanensis_793 | Subcoccinella_vigintiquatuorpunctata_621 | 0.192 |
| Scymnus_ningshanensis_794 | Subcoccinella_vigintiquatuorpunctata_621 | 0.192 |
| Scymnus_ningshanensis_795 | Subcoccinella_vigintiquatuorpunctata_621 | 0.192 |
| Hyperaspis_lateralis_655 | Subcoccinella_vigintiquatuorpunctata_624 | 0.192 |
| Scymnus_ningshanensis_788 | Subcoccinella_vigintiquatuorpunctata_627 | 0.192 |
| Scymnus_ningshanensis_791 | Subcoccinella_vigintiquatuorpunctata_627 | 0.192 |
| Scymnus_ningshanensis_792 | Subcoccinella_vigintiquatuorpunctata_627 | 0.192 |
| Scymnus_ningshanensis_793 | Subcoccinella_vigintiquatuorpunctata_627 | 0.192 |
| Scymnus_ningshanensis_794 | Subcoccinella_vigintiquatuorpunctata_627 | 0.192 |
| Scymnus_ningshanensis_795 | Subcoccinella_vigintiquatuorpunctata_627 | 0.192 |
| Scymnus_ningshanensis_788 | Subcoccinella_vigintiquatuorpunctata_628 | 0.192 |
| Scymnus_ningshanensis_791 | Subcoccinella_vigintiquatuorpunctata_628 | 0.192 |
| Scymnus_ningshanensis_792 | Subcoccinella_vigintiquatuorpunctata_628 | 0.192 |
| Scymnus_ningshanensis_793 | Subcoccinella_vigintiquatuorpunctata_628 | 0.192 |
| Scymnus_ningshanensis_794 | Subcoccinella_vigintiquatuorpunctata_628 | 0.192 |
| Scymnus_ningshanensis_795 | Subcoccinella_vigintiquatuorpunctata_628 | 0.192 |
| Scymnus_ningshanensis_788 | Subcoccinella_vigintiquatuorpunctata_629 | 0.192 |
| Scymnus_ningshanensis_791 | Subcoccinella_vigintiquatuorpunctata_629 | 0.192 |
| Scymnus_ningshanensis_792 | Subcoccinella_vigintiquatuorpunctata_629 | 0.192 |
| Scymnus_ningshanensis_793 | Subcoccinella_vigintiquatuorpunctata_629 | 0.192 |
| Scymnus_ningshanensis_794 | Subcoccinella_vigintiquatuorpunctata_629 | 0.192 |
| Scymnus_ningshanensis_795 | Subcoccinella_vigintiquatuorpunctata_629 | 0.192 |
| Scymnus_ningshanensis_788 | Subcoccinella_vigintiquatuorpunctata_630 | 0.192 |
| Scymnus_ningshanensis_791 | Subcoccinella_vigintiquatuorpunctata_630 | 0.192 |
| Scymnus_ningshanensis_792 | Subcoccinella_vigintiquatuorpunctata_630 | 0.192 |
| Scymnus_ningshanensis_793 | Subcoccinella_vigintiquatuorpunctata_630 | 0.192 |
| Scymnus_ningshanensis_794 | Subcoccinella_vigintiquatuorpunctata_630 | 0.192 |
| Scymnus_ningshanensis_795 | Subcoccinella_vigintiquatuorpunctata_630 | 0.192 |
| Scymnus_ningshanensis_788 | Subcoccinella_vigintiquatuorpunctata_631 | 0.192 |
| Scymnus_ningshanensis_791 | Subcoccinella_vigintiquatuorpunctata_631 | 0.192 |
| Scymnus_ningshanensis_792 | Subcoccinella_vigintiquatuorpunctata_631 | 0.192 |
| Scymnus_ningshanensis_793 | Subcoccinella_vigintiquatuorpunctata_631 | 0.192 |
| Scymnus_ningshanensis_794 | Subcoccinella_vigintiquatuorpunctata_631 | 0.192 |
| Scymnus_ningshanensis_795 | Subcoccinella_vigintiquatuorpunctata_631 | 0.192 |
| Coccinella_septempunctata_246 | Tytthaspis_sedecimpunctata_586 | 0.192 |
| Coccinella_septempunctata_242 | Tytthaspis_sedecimpunctata_587 | 0.192 |
| Coccinella_septempunctata_244 | Tytthaspis_sedecimpunctata_587 | 0.192 |
| Coccinella_septempunctata_245 | Tytthaspis_sedecimpunctata_587 | 0.192 |
| Coccinella_septempunctata_247 | Tytthaspis_sedecimpunctata_587 | 0.192 |
| Coccinella_septempunctata_248 | Tytthaspis_sedecimpunctata_587 | 0.192 |
| Coccinella_septempunctata_249 | Tytthaspis_sedecimpunctata_587 | 0.192 |
| Coccinella_septempunctata_252 | Tytthaspis_sedecimpunctata_587 | 0.192 |
| Coccinella_septempunctata_255 | Tytthaspis_sedecimpunctata_587 | 0.192 |
| Coccinella_septempunctata_256 | Tytthaspis_sedecimpunctata_587 | 0.192 |
| Myzia_pullata_519 | Tytthaspis_sedecimpunctata_587 | 0.192 |
| Coccinella_septempunctata_246 | Tytthaspis_sedecimpunctata_588 | 0.192 |
| Brachiacantha_rotunda_636 | Scymnus_rubromaculatus_822 | 0.192 |
| Brachiacantha_rotunda_636 | Scymnus_rubromaculatus_823 | 0.192 |
| Brachiacantha_rotunda_636 | Scymnus_rubromaculatus_824 | 0.192 |
| Brachiacantha_rotunda_636 | Scymnus_rubromaculatus_825 | 0.192 |
| Brachiacantha_rotunda_636 | Scymnus_rubromaculatus_826 | 0.192 |
| Brachiacantha_rotunda_636 | Scymnus_rubromaculatus_827 | 0.192 |
| Coccidula_scutellata_83 | Scymnus_sinuanodulus_830 | 0.192 |

| | | |
|---|---|---|
| Nephus_redtenbacheri_676 | Scymnus_suturalis_832 | 0.192 |
| Nephus_redtenbacheri_677 | Scymnus_suturalis_832 | 0.192 |
| Nephus_redtenbacheri_680 | Scymnus_suturalis_832 | 0.192 |
| Nephus_redtenbacheri_681 | Scymnus_suturalis_832 | 0.192 |
| Nephus_redtenbacheri_674 | Scymnus_suturalis_834 | 0.192 |
| Nephus_redtenbacheri_675 | Scymnus_suturalis_834 | 0.192 |
| Nephus_redtenbacheri_676 | Scymnus_suturalis_835 | 0.192 |
| Nephus_redtenbacheri_677 | Scymnus_suturalis_835 | 0.192 |
| Nephus_redtenbacheri_680 | Scymnus_suturalis_835 | 0.192 |
| Nephus_redtenbacheri_681 | Scymnus_suturalis_835 | 0.192 |
| Nephus_redtenbacheri_676 | Scymnus_suturalis_836 | 0.192 |
| Nephus_redtenbacheri_677 | Scymnus_suturalis_836 | 0.192 |
| Nephus_redtenbacheri_680 | Scymnus_suturalis_836 | 0.192 |
| Nephus_redtenbacheri_681 | Scymnus_suturalis_836 | 0.192 |
| Nephus_redtenbacheri_676 | Scymnus_suturalis_837 | 0.192 |
| Nephus_redtenbacheri_677 | Scymnus_suturalis_837 | 0.192 |
| Nephus_redtenbacheri_680 | Scymnus_suturalis_837 | 0.192 |
| Nephus_redtenbacheri_681 | Scymnus_suturalis_837 | 0.192 |
| Nephus_redtenbacheri_676 | Scymnus_suturalis_839 | 0.192 |
| Nephus_redtenbacheri_677 | Scymnus_suturalis_839 | 0.192 |
| Nephus_redtenbacheri_680 | Scymnus_suturalis_839 | 0.192 |
| Nephus_redtenbacheri_681 | Scymnus_suturalis_839 | 0.192 |
| Coccinella_undecimpunctata_289 | Vibidia_duodecimguttata_596 | 0.192 |
| Coccinella_undecimpunctata_290 | Vibidia_duodecimguttata_596 | 0.192 |
| Coelophora_inaequalis_318 | Vibidia_duodecimguttata_596 | 0.192 |
| Coelophora_inaequalis_318 | Vibidia_duodecimguttata_598 | 0.192 |
| Hyperaspis_oregona_658 | Stethorus_punctillum_853 | 0.192 |
| Hyperaspis_oregona_659 | Stethorus_punctillum_853 | 0.192 |
| Scymnus_nebulosus_781 | Subcoccinella_vigintiquatuorpunctata_620 | 0.192 |
| Scymnus_nebulosus_781 | Subcoccinella_vigintiquatuorpunctata_621 | 0.192 |
| Scymnus_nebulosus_781 | Subcoccinella_vigintiquatuorpunctata_624 | 0.192 |
| Scymnus_apetzi_691 | Subcoccinella_vigintiquatuorpunctata_627 | 0.192 |
| Scymnus_nebulosus_781 | Subcoccinella_vigintiquatuorpunctata_628 | 0.192 |
| Scymnus_nebulosus_781 | Subcoccinella_vigintiquatuorpunctata_629 | 0.192 |
| Scymnus_apetzi_690 | Subcoccinella_vigintiquatuorpunctata_630 | 0.192 |
| Scymnus_apetzi_697 | Subcoccinella_vigintiquatuorpunctata_630 | 0.192 |
| Scymnus_pallipediformis_811 | Subcoccinella_vigintiquatuorpunctata_630 | 0.192 |
| Scymnus_nebulosus_781 | Subcoccinella_vigintiquatuorpunctata_631 | 0.192 |
| Scymnus_nigrinus_785 | Tytthaspis_sedecimpunctata_577 | 0.192 |
| Scymnus_nigrinus_786 | Tytthaspis_sedecimpunctata_577 | 0.192 |
| Scymnus_nigrinus_785 | Tytthaspis_sedecimpunctata_578 | 0.192 |
| Scymnus_nigrinus_786 | Tytthaspis_sedecimpunctata_578 | 0.192 |
| Scymnus_nigrinus_785 | Tytthaspis_sedecimpunctata_579 | 0.192 |
| Scymnus_nigrinus_786 | Tytthaspis_sedecimpunctata_579 | 0.192 |
| Scymnus_nigrinus_785 | Tytthaspis_sedecimpunctata_581 | 0.192 |
| Scymnus_nigrinus_786 | Tytthaspis_sedecimpunctata_581 | 0.192 |
| Scymnus_nigrinus_785 | Tytthaspis_sedecimpunctata_582 | 0.192 |
| Scymnus_nigrinus_786 | Tytthaspis_sedecimpunctata_582 | 0.192 |
| Scymnus_nigrinus_784 | Tytthaspis_sedecimpunctata_583 | 0.192 |
| Brachiacantha_rotunda_632 | Tytthaspis_sedecimpunctata_584 | 0.192 |
| Brachiacantha_rotunda_633 | Tytthaspis_sedecimpunctata_584 | 0.192 |
| Brachiacantha_rotunda_635 | Tytthaspis_sedecimpunctata_584 | 0.192 |
| Brachiacantha_rotunda_636 | Tytthaspis_sedecimpunctata_584 | 0.192 |
| Scymnus_interruptus_767 | Tytthaspis_sedecimpunctata_584 | 0.192 |
| Scymnus_nigrinus_784 | Tytthaspis_sedecimpunctata_585 | 0.192 |
| Coccinella_septempunctata_250 | Tytthaspis_sedecimpunctata_587 | 0.192 |

| | | |
|---|---|---|
| Coccinella_septempunctata_251 | Tytthaspis_sedecimpunctata_587 | 0.192 |
| Scymnus_nigrinus_784 | Tytthaspis_sedecimpunctata_589 | 0.192 |
| Scymnus_nigrinus_785 | Tytthaspis_sedecimpunctata_590 | 0.192 |
| Scymnus_nigrinus_786 | Tytthaspis_sedecimpunctata_590 | 0.192 |
| Cheilomenes_sexmaculata_224 | Tytthaspis_sedecimpunctata_591 | 0.192 |
| Scymnus_nigrinus_784 | Tytthaspis_sedecimpunctata_592 | 0.192 |
| Scymnus_nigrinus_785 | Tytthaspis_sedecimpunctata_593 | 0.192 |
| Scymnus_nigrinus_786 | Tytthaspis_sedecimpunctata_593 | 0.192 |
| Scymnus_nigrinus_785 | Tytthaspis_sedecimpunctata_594 | 0.192 |
| Scymnus_nigrinus_786 | Tytthaspis_sedecimpunctata_594 | 0.192 |
| Scymnus_nigrinus_785 | Tytthaspis_sedecimpunctata_595 | 0.192 |
| Scymnus_nigrinus_786 | Tytthaspis_sedecimpunctata_595 | 0.192 |
| Platynaspis_luteorubra_52 | Scymnus_rubromaculatus_822 | 0.192 |
| Platynaspis_luteorubra_56 | Scymnus_rubromaculatus_822 | 0.192 |
| Platynaspis_luteorubra_60 | Scymnus_rubromaculatus_822 | 0.192 |
| Platynaspis_luteorubra_52 | Scymnus_rubromaculatus_823 | 0.192 |
| Platynaspis_luteorubra_56 | Scymnus_rubromaculatus_823 | 0.192 |
| Platynaspis_luteorubra_60 | Scymnus_rubromaculatus_823 | 0.192 |
| Platynaspis_luteorubra_52 | Scymnus_rubromaculatus_824 | 0.192 |
| Platynaspis_luteorubra_56 | Scymnus_rubromaculatus_824 | 0.192 |
| Platynaspis_luteorubra_60 | Scymnus_rubromaculatus_824 | 0.192 |
| Platynaspis_luteorubra_52 | Scymnus_rubromaculatus_825 | 0.192 |
| Platynaspis_luteorubra_56 | Scymnus_rubromaculatus_825 | 0.192 |
| Platynaspis_luteorubra_60 | Scymnus_rubromaculatus_825 | 0.192 |
| Platynaspis_luteorubra_52 | Scymnus_rubromaculatus_826 | 0.192 |
| Platynaspis_luteorubra_56 | Scymnus_rubromaculatus_826 | 0.192 |
| Platynaspis_luteorubra_60 | Scymnus_rubromaculatus_826 | 0.192 |
| Platynaspis_luteorubra_52 | Scymnus_rubromaculatus_827 | 0.192 |
| Platynaspis_luteorubra_56 | Scymnus_rubromaculatus_827 | 0.192 |
| Platynaspis_luteorubra_60 | Scymnus_rubromaculatus_827 | 0.192 |
| Calvia_quatuordecimguttata_211 | Scymnus_suturalis_841 | 0.192 |
| Calvia_quatuordecimguttata_212 | Scymnus_suturalis_841 | 0.192 |
| Calvia_quatuordecimguttata_213 | Scymnus_suturalis_841 | 0.192 |
| Calvia_quatuordecimguttata_214 | Scymnus_suturalis_841 | 0.192 |
| Calvia_quatuordecimguttata_215 | Scymnus_suturalis_841 | 0.192 |
| Calvia_quatuordecimguttata_211 | Scymnus_suturalis_842 | 0.192 |
| Calvia_quatuordecimguttata_212 | Scymnus_suturalis_842 | 0.192 |
| Calvia_quatuordecimguttata_213 | Scymnus_suturalis_842 | 0.192 |
| Calvia_quatuordecimguttata_214 | Scymnus_suturalis_842 | 0.192 |
| Calvia_quatuordecimguttata_215 | Scymnus_suturalis_842 | 0.192 |
| Calvia_quatuordecimguttata_201 | Scymnus_suturalis_843 | 0.192 |
| Calvia_quatuordecimguttata_202 | Scymnus_suturalis_843 | 0.192 |
| Calvia_quatuordecimguttata_210 | Scymnus_suturalis_843 | 0.192 |
| Calvia_quatuordecimguttata_211 | Scymnus_suturalis_844 | 0.192 |
| Calvia_quatuordecimguttata_212 | Scymnus_suturalis_844 | 0.192 |
| Calvia_quatuordecimguttata_213 | Scymnus_suturalis_844 | 0.192 |
| Calvia_quatuordecimguttata_214 | Scymnus_suturalis_844 | 0.192 |
| Calvia_quatuordecimguttata_215 | Scymnus_suturalis_844 | 0.192 |
| Calvia_quatuordecimguttata_201 | Scymnus_suturalis_845 | 0.192 |
| Calvia_quatuordecimguttata_202 | Scymnus_suturalis_845 | 0.192 |
| Calvia_quatuordecimguttata_210 | Scymnus_suturalis_845 | 0.192 |
| Calvia_quatuordecimguttata_211 | Scymnus_suturalis_846 | 0.192 |
| Calvia_quatuordecimguttata_212 | Scymnus_suturalis_846 | 0.192 |
| Calvia_quatuordecimguttata_213 | Scymnus_suturalis_846 | 0.192 |
| Calvia_quatuordecimguttata_214 | Scymnus_suturalis_846 | 0.192 |
| Calvia_quatuordecimguttata_215 | Scymnus_suturalis_846 | 0.192 |

Supplementary Table S4 (continued)

| | | |
|---|---|---|
| Calvia_quatuordecimguttata_211 | Scymnus_suturalis_847 | 0.192 |
| Calvia_quatuordecimguttata_212 | Scymnus_suturalis_847 | 0.192 |
| Calvia_quatuordecimguttata_213 | Scymnus_suturalis_847 | 0.192 |
| Calvia_quatuordecimguttata_214 | Scymnus_suturalis_847 | 0.192 |
| Calvia_quatuordecimguttata_215 | Scymnus_suturalis_847 | 0.192 |
| Calvia_quatuordecimguttata_211 | Scymnus_suturalis_848 | 0.192 |
| Calvia_quatuordecimguttata_212 | Scymnus_suturalis_848 | 0.192 |
| Calvia_quatuordecimguttata_213 | Scymnus_suturalis_848 | 0.192 |
| Calvia_quatuordecimguttata_214 | Scymnus_suturalis_848 | 0.192 |
| Calvia_quatuordecimguttata_215 | Scymnus_suturalis_848 | 0.192 |
| Scymnus_nebulosus_782 | Subcoccinella_vigintiquatuorpunctata_625 | 0.192 |
| Brachiacantha_rotunda_634 | Tytthaspis_sedecimpunctata_577 | 0.192 |
| Scymnus_interruptus_767 | Tytthaspis_sedecimpunctata_577 | 0.192 |
| Brachiacantha_rotunda_634 | Tytthaspis_sedecimpunctata_578 | 0.192 |
| Scymnus_interruptus_767 | Tytthaspis_sedecimpunctata_578 | 0.192 |
| Brachiacantha_rotunda_634 | Tytthaspis_sedecimpunctata_579 | 0.192 |
| Scymnus_interruptus_767 | Tytthaspis_sedecimpunctata_579 | 0.192 |
| Brachiacantha_rotunda_634 | Tytthaspis_sedecimpunctata_581 | 0.192 |
| Scymnus_interruptus_767 | Tytthaspis_sedecimpunctata_581 | 0.192 |
| Brachiacantha_rotunda_634 | Tytthaspis_sedecimpunctata_582 | 0.192 |
| Scymnus_interruptus_767 | Tytthaspis_sedecimpunctata_582 | 0.192 |
| Scymnus_nigrinus_785 | Tytthaspis_sedecimpunctata_584 | 0.192 |
| Scymnus_nigrinus_786 | Tytthaspis_sedecimpunctata_584 | 0.192 |
| Scymnus_ferrugatus_723 | Tytthaspis_sedecimpunctata_586 | 0.192 |
| Scymnus_ferrugatus_725 | Tytthaspis_sedecimpunctata_586 | 0.192 |
| Scymnus_ferrugatus_726 | Tytthaspis_sedecimpunctata_586 | 0.192 |
| Scymnus_ferrugatus_727 | Tytthaspis_sedecimpunctata_586 | 0.192 |
| Scymnus_ferrugatus_728 | Tytthaspis_sedecimpunctata_586 | 0.192 |
| Scymnus_ferrugatus_730 | Tytthaspis_sedecimpunctata_586 | 0.192 |
| Scymnus_ferrugatus_731 | Tytthaspis_sedecimpunctata_586 | 0.192 |
| Scymnus_ferrugatus_732 | Tytthaspis_sedecimpunctata_586 | 0.192 |
| Scymnus_ferrugatus_733 | Tytthaspis_sedecimpunctata_586 | 0.192 |
| Scymnus_nigrinus_784 | Tytthaspis_sedecimpunctata_587 | 0.192 |
| Olla_vnigrum_530 | Tytthaspis_sedecimpunctata_589 | 0.192 |
| Scymnus_interruptus_767 | Tytthaspis_sedecimpunctata_589 | 0.192 |
| Brachiacantha_rotunda_634 | Tytthaspis_sedecimpunctata_590 | 0.192 |
| Scymnus_interruptus_767 | Tytthaspis_sedecimpunctata_590 | 0.192 |
| Brachiacantha_rotunda_634 | Tytthaspis_sedecimpunctata_592 | 0.192 |
| Scymnus_interruptus_761 | Tytthaspis_sedecimpunctata_592 | 0.192 |
| Scymnus_interruptus_763 | Tytthaspis_sedecimpunctata_592 | 0.192 |
| Scymnus_interruptus_764 | Tytthaspis_sedecimpunctata_592 | 0.192 |
| Scymnus_interruptus_766 | Tytthaspis_sedecimpunctata_592 | 0.192 |
| Scymnus_interruptus_767 | Tytthaspis_sedecimpunctata_593 | 0.192 |
| Brachiacantha_rotunda_634 | Tytthaspis_sedecimpunctata_594 | 0.192 |
| Scymnus_interruptus_767 | Tytthaspis_sedecimpunctata_594 | 0.192 |
| Brachiacantha_rotunda_634 | Tytthaspis_sedecimpunctata_595 | 0.192 |
| Scymnus_interruptus_767 | Tytthaspis_sedecimpunctata_595 | 0.192 |
| Platynaspis_luteorubra_54 | Scymnus_rubromaculatus_822 | 0.192 |
| Platynaspis_luteorubra_55 | Scymnus_rubromaculatus_822 | 0.192 |
| Platynaspis_luteorubra_57 | Scymnus_rubromaculatus_822 | 0.192 |
| Platynaspis_luteorubra_59 | Scymnus_rubromaculatus_822 | 0.192 |
| Platynaspis_luteorubra_54 | Scymnus_rubromaculatus_823 | 0.192 |
| Platynaspis_luteorubra_55 | Scymnus_rubromaculatus_823 | 0.192 |
| Platynaspis_luteorubra_57 | Scymnus_rubromaculatus_823 | 0.192 |
| Platynaspis_luteorubra_59 | Scymnus_rubromaculatus_823 | 0.192 |
| Platynaspis_luteorubra_54 | Scymnus_rubromaculatus_824 | 0.192 |

| | | |
|---|---|---|
| Platynaspis_luteorubra_55 | Scymnus_rubromaculatus_824 | 0.192 |
| Platynaspis_luteorubra_57 | Scymnus_rubromaculatus_824 | 0.192 |
| Platynaspis_luteorubra_59 | Scymnus_rubromaculatus_824 | 0.192 |
| Platynaspis_luteorubra_54 | Scymnus_rubromaculatus_825 | 0.192 |
| Platynaspis_luteorubra_55 | Scymnus_rubromaculatus_825 | 0.192 |
| Platynaspis_luteorubra_57 | Scymnus_rubromaculatus_825 | 0.192 |
| Platynaspis_luteorubra_59 | Scymnus_rubromaculatus_825 | 0.192 |
| Platynaspis_luteorubra_54 | Scymnus_rubromaculatus_826 | 0.192 |
| Platynaspis_luteorubra_55 | Scymnus_rubromaculatus_826 | 0.192 |
| Platynaspis_luteorubra_57 | Scymnus_rubromaculatus_826 | 0.192 |
| Platynaspis_luteorubra_59 | Scymnus_rubromaculatus_826 | 0.192 |
| Platynaspis_luteorubra_54 | Scymnus_rubromaculatus_827 | 0.192 |
| Platynaspis_luteorubra_55 | Scymnus_rubromaculatus_827 | 0.192 |
| Platynaspis_luteorubra_57 | Scymnus_rubromaculatus_827 | 0.192 |
| Platynaspis_luteorubra_59 | Scymnus_rubromaculatus_827 | 0.192 |
| Chilocorus_stigma_44 | Scymnus_sinuanodulus_830 | 0.192 |
| Chilocorus_stigma_45 | Scymnus_sinuanodulus_830 | 0.192 |
| Chilocorus_stigma_46 | Scymnus_sinuanodulus_830 | 0.192 |
| Calvia_quatuordecimguttata_211 | Scymnus_suturalis_832 | 0.192 |
| Calvia_quatuordecimguttata_212 | Scymnus_suturalis_832 | 0.192 |
| Calvia_quatuordecimguttata_213 | Scymnus_suturalis_832 | 0.192 |
| Calvia_quatuordecimguttata_214 | Scymnus_suturalis_832 | 0.192 |
| Calvia_quatuordecimguttata_215 | Scymnus_suturalis_832 | 0.192 |
| Anatis_labiculata_126 | Scymnus_suturalis_833 | 0.192 |
| Hyperaspis_binotata_649 | Scymnus_suturalis_833 | 0.192 |
| Calvia_quatuordecimguttata_211 | Scymnus_suturalis_835 | 0.192 |
| Calvia_quatuordecimguttata_212 | Scymnus_suturalis_835 | 0.192 |
| Calvia_quatuordecimguttata_213 | Scymnus_suturalis_835 | 0.192 |
| Calvia_quatuordecimguttata_214 | Scymnus_suturalis_835 | 0.192 |
| Calvia_quatuordecimguttata_215 | Scymnus_suturalis_835 | 0.192 |
| Calvia_quatuordecimguttata_211 | Scymnus_suturalis_836 | 0.192 |
| Calvia_quatuordecimguttata_212 | Scymnus_suturalis_836 | 0.192 |
| Calvia_quatuordecimguttata_213 | Scymnus_suturalis_836 | 0.192 |
| Calvia_quatuordecimguttata_214 | Scymnus_suturalis_836 | 0.192 |
| Calvia_quatuordecimguttata_215 | Scymnus_suturalis_836 | 0.192 |
| Calvia_quatuordecimguttata_211 | Scymnus_suturalis_839 | 0.192 |
| Calvia_quatuordecimguttata_212 | Scymnus_suturalis_839 | 0.192 |
| Calvia_quatuordecimguttata_213 | Scymnus_suturalis_839 | 0.192 |
| Calvia_quatuordecimguttata_214 | Scymnus_suturalis_839 | 0.192 |
| Calvia_quatuordecimguttata_215 | Scymnus_suturalis_839 | 0.192 |
| Cynegetis_impunctata_604 | Subcoccinella_vigintiquatuorpunctata_619 | 0.192 |
| Cynegetis_impunctata_605 | Subcoccinella_vigintiquatuorpunctata_619 | 0.192 |
| Cynegetis_impunctata_606 | Subcoccinella_vigintiquatuorpunctata_619 | 0.192 |
| Cynegetis_impunctata_607 | Subcoccinella_vigintiquatuorpunctata_619 | 0.192 |
| Cynegetis_impunctata_608 | Subcoccinella_vigintiquatuorpunctata_619 | 0.192 |
| Cynegetis_impunctata_609 | Subcoccinella_vigintiquatuorpunctata_619 | 0.192 |
| Cynegetis_impunctata_610 | Subcoccinella_vigintiquatuorpunctata_619 | 0.192 |
| Cynegetis_impunctata_611 | Subcoccinella_vigintiquatuorpunctata_619 | 0.192 |
| Cynegetis_impunctata_612 | Subcoccinella_vigintiquatuorpunctata_619 | 0.192 |
| Cynegetis_impunctata_613 | Subcoccinella_vigintiquatuorpunctata_619 | 0.192 |
| Platynaspis_luteorubra_51 | Scymnus_sinuanodulus_831 | 0.192 |
| Platynaspis_luteorubra_53 | Scymnus_sinuanodulus_831 | 0.192 |
| Platynaspis_luteorubra_58 | Scymnus_sinuanodulus_831 | 0.192 |
| Clitostethus_arcuatus_642 | Scymnus_suturalis_843 | 0.192 |
| Harmonia_axyridis_355 | Scymnus_suturalis_843 | 0.192 |
| Harmonia_axyridis_355 | Scymnus_suturalis_845 | 0.192 |

| | | |
|---|---|---|
| Hyperaspis_lateralis_654 | Stethorus_punctillum_852 | 0.192 |
| Hyperaspis_binotata_649 | Subcoccinella_vigintiquatuorpunctata_624 | 0.192 |
| Scymnus_interruptus_761 | Tytthaspis_sedecimpunctata_580 | 0.192 |
| Scymnus_interruptus_763 | Tytthaspis_sedecimpunctata_580 | 0.192 |
| Scymnus_interruptus_764 | Tytthaspis_sedecimpunctata_580 | 0.192 |
| Scymnus_interruptus_766 | Tytthaspis_sedecimpunctata_580 | 0.192 |
| Scymnus_camptodromus_705 | Tytthaspis_sedecimpunctata_584 | 0.192 |
| Scymnus_camptodromus_713 | Tytthaspis_sedecimpunctata_584 | 0.192 |
| Scymnus_camptodromus_716 | Tytthaspis_sedecimpunctata_584 | 0.192 |
| Scymnus_camptodromus_718 | Tytthaspis_sedecimpunctata_585 | 0.192 |
| Brachiacantha_rotunda_634 | Tytthaspis_sedecimpunctata_586 | 0.192 |
| Scymnus_interruptus_767 | Tytthaspis_sedecimpunctata_586 | 0.192 |
| Scymnus_camptodromus_705 | Tytthaspis_sedecimpunctata_587 | 0.192 |
| Scymnus_camptodromus_713 | Tytthaspis_sedecimpunctata_587 | 0.192 |
| Scymnus_camptodromus_716 | Tytthaspis_sedecimpunctata_587 | 0.192 |
| Scymnus_ferrugatus_724 | Tytthaspis_sedecimpunctata_587 | 0.192 |
| Scymnus_ferrugatus_729 | Tytthaspis_sedecimpunctata_587 | 0.192 |
| Scymnus_interruptus_760 | Tytthaspis_sedecimpunctata_587 | 0.192 |
| Scymnus_interruptus_765 | Tytthaspis_sedecimpunctata_587 | 0.192 |
| Scymnus_interruptus_768 | Tytthaspis_sedecimpunctata_587 | 0.192 |
| Olla_vnigrum_530 | Tytthaspis_sedecimpunctata_588 | 0.192 |
| Scymnus_ferrugatus_723 | Tytthaspis_sedecimpunctata_589 | 0.192 |
| Scymnus_ferrugatus_725 | Tytthaspis_sedecimpunctata_589 | 0.192 |
| Scymnus_ferrugatus_726 | Tytthaspis_sedecimpunctata_589 | 0.192 |
| Scymnus_ferrugatus_727 | Tytthaspis_sedecimpunctata_589 | 0.192 |
| Scymnus_ferrugatus_728 | Tytthaspis_sedecimpunctata_589 | 0.192 |
| Scymnus_ferrugatus_730 | Tytthaspis_sedecimpunctata_589 | 0.192 |
| Scymnus_ferrugatus_731 | Tytthaspis_sedecimpunctata_589 | 0.192 |
| Scymnus_ferrugatus_732 | Tytthaspis_sedecimpunctata_589 | 0.192 |
| Scymnus_ferrugatus_733 | Tytthaspis_sedecimpunctata_589 | 0.192 |
| Brumoides_suturalis_1 | Scymnus_rubromaculatus_822 | 0.192 |
| Brumoides_suturalis_1 | Scymnus_rubromaculatus_823 | 0.192 |
| Brumoides_suturalis_1 | Scymnus_rubromaculatus_824 | 0.192 |
| Brumoides_suturalis_1 | Scymnus_rubromaculatus_825 | 0.192 |
| Brumoides_suturalis_1 | Scymnus_rubromaculatus_826 | 0.192 |
| Brumoides_suturalis_1 | Scymnus_rubromaculatus_827 | 0.192 |
| Chilocorus_stigma_47 | Scymnus_sinuanodulus_828 | 0.192 |
| Coccinella_undecimpunctata_285 | Scymnus_sinuanodulus_830 | 0.192 |
| Coccinella_undecimpunctata_286 | Scymnus_sinuanodulus_830 | 0.192 |
| Coccinella_undecimpunctata_288 | Scymnus_sinuanodulus_830 | 0.192 |
| Scymnus_frontalis_734 | Scymnus_suturalis_833 | 0.192 |
| Scymnus_frontalis_745 | Scymnus_suturalis_833 | 0.192 |
| Clitostethus_arcuatus_642 | Scymnus_suturalis_834 | 0.192 |
| Harmonia_axyridis_355 | Scymnus_suturalis_834 | 0.192 |
| Scymnus_frontalis_742 | Scymnus_suturalis_838 | 0.192 |
| Hyperaspis_lateralis_656 | Subcoccinella_vigintiquatuorpunctata_619 | 0.192 |
| Hyperaspis_lateralis_657 | Subcoccinella_vigintiquatuorpunctata_619 | 0.192 |
| Hyperaspis_reppensis_662 | Subcoccinella_vigintiquatuorpunctata_619 | 0.192 |
| Hyperaspis_reppensis_663 | Subcoccinella_vigintiquatuorpunctata_619 | 0.192 |
| Brumus_quadripustulatus_9 | Scymnus_sinuanodulus_831 | 0.192 |
| Harmonia_axyridis_354 | Scymnus_suturalis_841 | 0.192 |
| Harmonia_axyridis_356 | Scymnus_suturalis_841 | 0.192 |
| Harmonia_axyridis_357 | Scymnus_suturalis_841 | 0.192 |
| Harmonia_axyridis_358 | Scymnus_suturalis_841 | 0.192 |
| Harmonia_axyridis_359 | Scymnus_suturalis_841 | 0.192 |
| Harmonia_axyridis_360 | Scymnus_suturalis_841 | 0.192 |

| | | |
|---|---|---|
| Harmonia_axyridis_361 | Scymnus_suturalis_841 | 0.192 |
| Harmonia_axyridis_362 | Scymnus_suturalis_841 | 0.192 |
| Harmonia_axyridis_363 | Scymnus_suturalis_841 | 0.192 |
| Harmonia_axyridis_364 | Scymnus_suturalis_841 | 0.192 |
| Harmonia_axyridis_354 | Scymnus_suturalis_842 | 0.192 |
| Harmonia_axyridis_356 | Scymnus_suturalis_842 | 0.192 |
| Harmonia_axyridis_357 | Scymnus_suturalis_842 | 0.192 |
| Harmonia_axyridis_358 | Scymnus_suturalis_842 | 0.192 |
| Harmonia_axyridis_359 | Scymnus_suturalis_842 | 0.192 |
| Harmonia_axyridis_360 | Scymnus_suturalis_842 | 0.192 |
| Harmonia_axyridis_361 | Scymnus_suturalis_842 | 0.192 |
| Harmonia_axyridis_362 | Scymnus_suturalis_842 | 0.192 |
| Harmonia_axyridis_363 | Scymnus_suturalis_842 | 0.192 |
| Harmonia_axyridis_364 | Scymnus_suturalis_842 | 0.192 |
| Harmonia_axyridis_354 | Scymnus_suturalis_844 | 0.192 |
| Harmonia_axyridis_356 | Scymnus_suturalis_844 | 0.192 |
| Harmonia_axyridis_357 | Scymnus_suturalis_844 | 0.192 |
| Harmonia_axyridis_358 | Scymnus_suturalis_844 | 0.192 |
| Harmonia_axyridis_359 | Scymnus_suturalis_844 | 0.192 |
| Harmonia_axyridis_360 | Scymnus_suturalis_844 | 0.192 |
| Harmonia_axyridis_361 | Scymnus_suturalis_844 | 0.192 |
| Harmonia_axyridis_362 | Scymnus_suturalis_844 | 0.192 |
| Harmonia_axyridis_363 | Scymnus_suturalis_844 | 0.192 |
| Harmonia_axyridis_364 | Scymnus_suturalis_844 | 0.192 |
| Harmonia_axyridis_354 | Scymnus_suturalis_846 | 0.192 |
| Harmonia_axyridis_356 | Scymnus_suturalis_846 | 0.192 |
| Harmonia_axyridis_357 | Scymnus_suturalis_846 | 0.192 |
| Harmonia_axyridis_358 | Scymnus_suturalis_846 | 0.192 |
| Harmonia_axyridis_359 | Scymnus_suturalis_846 | 0.192 |
| Harmonia_axyridis_360 | Scymnus_suturalis_846 | 0.192 |
| Harmonia_axyridis_361 | Scymnus_suturalis_846 | 0.192 |
| Harmonia_axyridis_362 | Scymnus_suturalis_846 | 0.192 |
| Harmonia_axyridis_363 | Scymnus_suturalis_846 | 0.192 |
| Harmonia_axyridis_364 | Scymnus_suturalis_846 | 0.192 |
| Harmonia_axyridis_354 | Scymnus_suturalis_847 | 0.192 |
| Harmonia_axyridis_356 | Scymnus_suturalis_847 | 0.192 |
| Harmonia_axyridis_357 | Scymnus_suturalis_847 | 0.192 |
| Harmonia_axyridis_358 | Scymnus_suturalis_847 | 0.192 |
| Harmonia_axyridis_359 | Scymnus_suturalis_847 | 0.192 |
| Harmonia_axyridis_360 | Scymnus_suturalis_847 | 0.192 |
| Harmonia_axyridis_361 | Scymnus_suturalis_847 | 0.192 |
| Harmonia_axyridis_362 | Scymnus_suturalis_847 | 0.192 |
| Harmonia_axyridis_363 | Scymnus_suturalis_847 | 0.192 |
| Harmonia_axyridis_364 | Scymnus_suturalis_847 | 0.192 |
| Harmonia_axyridis_354 | Scymnus_suturalis_848 | 0.192 |
| Harmonia_axyridis_356 | Scymnus_suturalis_848 | 0.192 |
| Harmonia_axyridis_357 | Scymnus_suturalis_848 | 0.192 |
| Harmonia_axyridis_358 | Scymnus_suturalis_848 | 0.192 |
| Harmonia_axyridis_359 | Scymnus_suturalis_848 | 0.192 |
| Harmonia_axyridis_360 | Scymnus_suturalis_848 | 0.192 |
| Harmonia_axyridis_361 | Scymnus_suturalis_848 | 0.192 |
| Harmonia_axyridis_362 | Scymnus_suturalis_848 | 0.192 |
| Harmonia_axyridis_363 | Scymnus_suturalis_848 | 0.192 |
| Harmonia_axyridis_364 | Scymnus_suturalis_848 | 0.192 |
| Hyperaspis_lateralis_654 | Stethorus_punctillum_859 | 0.192 |
| Scymnus_nubilus_800 | Subcoccinella_vigintiquatuorpunctata_623 | 0.192 |

| | | |
|---|---|---|
| Scymnus_nubilus_801 | Subcoccinella_vigintiquatuorpunctata_623 | 0.192 |
| Scymnus_nubilus_802 | Subcoccinella_vigintiquatuorpunctata_623 | 0.192 |
| Scymnus_nubilus_805 | Subcoccinella_vigintiquatuorpunctata_623 | 0.192 |
| Scymnus_nubilus_806 | Subcoccinella_vigintiquatuorpunctata_623 | 0.192 |
| Scymnus_nubilus_807 | Subcoccinella_vigintiquatuorpunctata_623 | 0.192 |
| Scymnus_nubilus_810 | Subcoccinella_vigintiquatuorpunctata_623 | 0.192 |
| Olla_vnigrum_525 | Tytthaspis_sedecimpunctata_586 | 0.192 |
| Olla_vnigrum_529 | Tytthaspis_sedecimpunctata_586 | 0.192 |
| Olla_vnigrum_531 | Tytthaspis_sedecimpunctata_586 | 0.192 |
| Coccinella_undecimpunctata_285 | Scymnus_sinuanodulus_828 | 0.192 |
| Coccinella_undecimpunctata_286 | Scymnus_sinuanodulus_828 | 0.192 |
| Coccinella_undecimpunctata_288 | Scymnus_sinuanodulus_828 | 0.192 |
| Harmonia_axyridis_354 | Scymnus_suturalis_832 | 0.192 |
| Harmonia_axyridis_356 | Scymnus_suturalis_832 | 0.192 |
| Harmonia_axyridis_357 | Scymnus_suturalis_832 | 0.192 |
| Harmonia_axyridis_358 | Scymnus_suturalis_832 | 0.192 |
| Harmonia_axyridis_359 | Scymnus_suturalis_832 | 0.192 |
| Harmonia_axyridis_360 | Scymnus_suturalis_832 | 0.192 |
| Harmonia_axyridis_361 | Scymnus_suturalis_832 | 0.192 |
| Harmonia_axyridis_362 | Scymnus_suturalis_832 | 0.192 |
| Harmonia_axyridis_363 | Scymnus_suturalis_832 | 0.192 |
| Harmonia_axyridis_364 | Scymnus_suturalis_832 | 0.192 |
| Hippodamia_parenthesis_432 | Scymnus_suturalis_833 | 0.192 |
| Hippodamia_parenthesis_434 | Scymnus_suturalis_833 | 0.192 |
| Hippodamia_parenthesis_435 | Scymnus_suturalis_833 | 0.192 |
| Hippodamia_parenthesis_437 | Scymnus_suturalis_833 | 0.192 |
| Hippodamia_parenthesis_438 | Scymnus_suturalis_833 | 0.192 |
| Hippodamia_parenthesis_441 | Scymnus_suturalis_833 | 0.192 |
| Hippodamia_parenthesis_442 | Scymnus_suturalis_833 | 0.192 |
| Hyperaspis_binotata_650 | Scymnus_suturalis_833 | 0.192 |
| Scymnus_frontalis_742 | Scymnus_suturalis_834 | 0.192 |
| Harmonia_axyridis_354 | Scymnus_suturalis_835 | 0.192 |
| Harmonia_axyridis_356 | Scymnus_suturalis_835 | 0.192 |
| Harmonia_axyridis_357 | Scymnus_suturalis_835 | 0.192 |
| Harmonia_axyridis_358 | Scymnus_suturalis_835 | 0.192 |
| Harmonia_axyridis_359 | Scymnus_suturalis_835 | 0.192 |
| Harmonia_axyridis_360 | Scymnus_suturalis_835 | 0.192 |
| Harmonia_axyridis_361 | Scymnus_suturalis_835 | 0.192 |
| Harmonia_axyridis_362 | Scymnus_suturalis_835 | 0.192 |
| Harmonia_axyridis_363 | Scymnus_suturalis_835 | 0.192 |
| Harmonia_axyridis_364 | Scymnus_suturalis_835 | 0.192 |
| Harmonia_axyridis_354 | Scymnus_suturalis_836 | 0.192 |
| Harmonia_axyridis_356 | Scymnus_suturalis_836 | 0.192 |
| Harmonia_axyridis_357 | Scymnus_suturalis_836 | 0.192 |
| Harmonia_axyridis_358 | Scymnus_suturalis_836 | 0.192 |
| Harmonia_axyridis_359 | Scymnus_suturalis_836 | 0.192 |
| Harmonia_axyridis_360 | Scymnus_suturalis_836 | 0.192 |
| Harmonia_axyridis_361 | Scymnus_suturalis_836 | 0.192 |
| Harmonia_axyridis_362 | Scymnus_suturalis_836 | 0.192 |
| Harmonia_axyridis_363 | Scymnus_suturalis_836 | 0.192 |
| Harmonia_axyridis_364 | Scymnus_suturalis_836 | 0.192 |
| Hippodamia_parenthesis_432 | Scymnus_suturalis_838 | 0.192 |
| Hippodamia_parenthesis_434 | Scymnus_suturalis_838 | 0.192 |
| Hippodamia_parenthesis_435 | Scymnus_suturalis_838 | 0.192 |
| Hippodamia_parenthesis_437 | Scymnus_suturalis_838 | 0.192 |
| Hippodamia_parenthesis_438 | Scymnus_suturalis_838 | 0.192 |

Supplementary Table S4 (continued)

| | | |
|---|---|---|
| Hippodamia_parenthesis_441 | Scymnus_suturalis_838 | 0.192 |
| Hippodamia_parenthesis_442 | Scymnus_suturalis_838 | 0.192 |
| Harmonia_axyridis_354 | Scymnus_suturalis_839 | 0.192 |
| Harmonia_axyridis_356 | Scymnus_suturalis_839 | 0.192 |
| Harmonia_axyridis_357 | Scymnus_suturalis_839 | 0.192 |
| Harmonia_axyridis_358 | Scymnus_suturalis_839 | 0.192 |
| Harmonia_axyridis_359 | Scymnus_suturalis_839 | 0.192 |
| Harmonia_axyridis_360 | Scymnus_suturalis_839 | 0.192 |
| Harmonia_axyridis_361 | Scymnus_suturalis_839 | 0.192 |
| Harmonia_axyridis_362 | Scymnus_suturalis_839 | 0.192 |
| Harmonia_axyridis_363 | Scymnus_suturalis_839 | 0.192 |
| Harmonia_axyridis_364 | Scymnus_suturalis_839 | 0.192 |
| Hippodamia_parenthesis_432 | Scymnus_suturalis_840 | 0.192 |
| Hippodamia_parenthesis_434 | Scymnus_suturalis_840 | 0.192 |
| Hippodamia_parenthesis_435 | Scymnus_suturalis_840 | 0.192 |
| Hippodamia_parenthesis_437 | Scymnus_suturalis_840 | 0.192 |
| Hippodamia_parenthesis_438 | Scymnus_suturalis_840 | 0.192 |
| Hippodamia_parenthesis_441 | Scymnus_suturalis_840 | 0.192 |
| Hippodamia_parenthesis_442 | Scymnus_suturalis_840 | 0.192 |
| Scymnus_ningshanensis_789 | Subcoccinella_vigintiquatuorpunctata_619 | 0.192 |
| Scymnus_ningshanensis_790 | Subcoccinella_vigintiquatuorpunctata_619 | 0.192 |
| Illeis_galbula_474 | Vibidia_duodecimguttata_601 | 0.192 |
| Illeis_galbula_475 | Vibidia_duodecimguttata_601 | 0.192 |
| Illeis_galbula_476 | Vibidia_duodecimguttata_601 | 0.192 |
| Scymnus_camptodromus_715 | Subcoccinella_vigintiquatuorpunctata_623 | 0.192 |
| Coccinella_quinquepunctata_234 | Tytthaspis_sedecimpunctata_586 | 0.192 |
| Hyperaspis_proba_660 | Stethorus_punctillum_859 | 0.192 |
| Scymnus_apetzi_692 | Subcoccinella_vigintiquatuorpunctata_624 | 0.192 |
| Hippodamia_parenthesis_433 | Scymnus_suturalis_841 | 0.192 |
| Hippodamia_parenthesis_440 | Scymnus_suturalis_841 | 0.192 |
| Hippodamia_parenthesis_433 | Scymnus_suturalis_842 | 0.192 |
| Hippodamia_parenthesis_440 | Scymnus_suturalis_842 | 0.192 |
| Hippodamia_parenthesis_433 | Scymnus_suturalis_844 | 0.192 |
| Hippodamia_parenthesis_440 | Scymnus_suturalis_844 | 0.192 |
| Hippodamia_parenthesis_432 | Scymnus_suturalis_845 | 0.192 |
| Hippodamia_parenthesis_434 | Scymnus_suturalis_845 | 0.192 |
| Hippodamia_parenthesis_435 | Scymnus_suturalis_845 | 0.192 |
| Hippodamia_parenthesis_437 | Scymnus_suturalis_845 | 0.192 |
| Hippodamia_parenthesis_438 | Scymnus_suturalis_845 | 0.192 |
| Hippodamia_parenthesis_441 | Scymnus_suturalis_845 | 0.192 |
| Hippodamia_parenthesis_442 | Scymnus_suturalis_845 | 0.192 |
| Hippodamia_parenthesis_433 | Scymnus_suturalis_846 | 0.192 |
| Hippodamia_parenthesis_440 | Scymnus_suturalis_846 | 0.192 |
| Hippodamia_parenthesis_433 | Scymnus_suturalis_847 | 0.192 |
| Hippodamia_parenthesis_440 | Scymnus_suturalis_847 | 0.192 |
| Hippodamia_parenthesis_433 | Scymnus_suturalis_848 | 0.192 |
| Hippodamia_parenthesis_440 | Scymnus_suturalis_848 | 0.192 |
| Hyperaspis_lateralis_654 | Stethorus_punctillum_855 | 0.192 |
| Scymnus_nubilus_796 | Subcoccinella_vigintiquatuorpunctata_620 | 0.192 |
| Scymnus_nubilus_803 | Subcoccinella_vigintiquatuorpunctata_620 | 0.192 |
| Scymnus_nubilus_804 | Subcoccinella_vigintiquatuorpunctata_620 | 0.192 |
| Scymnus_nubilus_809 | Subcoccinella_vigintiquatuorpunctata_620 | 0.192 |
| Scymnus_nubilus_796 | Subcoccinella_vigintiquatuorpunctata_621 | 0.192 |
| Scymnus_nubilus_803 | Subcoccinella_vigintiquatuorpunctata_621 | 0.192 |
| Scymnus_nubilus_804 | Subcoccinella_vigintiquatuorpunctata_621 | 0.192 |
| Scymnus_nubilus_809 | Subcoccinella_vigintiquatuorpunctata_621 | 0.192 |

| | | |
|---|---|---|
| Hyperaspis_binotata_650 | Subcoccinella_vigintiquatuorpunctata_624 | 0.192 |
| Scymnus_nubilus_800 | Subcoccinella_vigintiquatuorpunctata_626 | 0.192 |
| Scymnus_nubilus_801 | Subcoccinella_vigintiquatuorpunctata_626 | 0.192 |
| Scymnus_nubilus_802 | Subcoccinella_vigintiquatuorpunctata_626 | 0.192 |
| Scymnus_nubilus_805 | Subcoccinella_vigintiquatuorpunctata_626 | 0.192 |
| Scymnus_nubilus_806 | Subcoccinella_vigintiquatuorpunctata_626 | 0.192 |
| Scymnus_nubilus_807 | Subcoccinella_vigintiquatuorpunctata_626 | 0.192 |
| Scymnus_nubilus_810 | Subcoccinella_vigintiquatuorpunctata_626 | 0.192 |
| Scymnus_nubilus_798 | Subcoccinella_vigintiquatuorpunctata_627 | 0.192 |
| Scymnus_nubilus_808 | Subcoccinella_vigintiquatuorpunctata_627 | 0.192 |
| Scymnus_nubilus_796 | Subcoccinella_vigintiquatuorpunctata_628 | 0.192 |
| Scymnus_nubilus_803 | Subcoccinella_vigintiquatuorpunctata_628 | 0.192 |
| Scymnus_nubilus_804 | Subcoccinella_vigintiquatuorpunctata_628 | 0.192 |
| Scymnus_nubilus_809 | Subcoccinella_vigintiquatuorpunctata_628 | 0.192 |
| Scymnus_nubilus_796 | Subcoccinella_vigintiquatuorpunctata_629 | 0.192 |
| Scymnus_nubilus_803 | Subcoccinella_vigintiquatuorpunctata_629 | 0.192 |
| Scymnus_nubilus_804 | Subcoccinella_vigintiquatuorpunctata_629 | 0.192 |
| Scymnus_nubilus_809 | Subcoccinella_vigintiquatuorpunctata_629 | 0.192 |
| Scymnus_nubilus_796 | Subcoccinella_vigintiquatuorpunctata_631 | 0.192 |
| Scymnus_nubilus_803 | Subcoccinella_vigintiquatuorpunctata_631 | 0.192 |
| Scymnus_nubilus_804 | Subcoccinella_vigintiquatuorpunctata_631 | 0.192 |
| Scymnus_nubilus_809 | Subcoccinella_vigintiquatuorpunctata_631 | 0.192 |
| Scymnus_ferrugatus_724 | Tytthaspis_sedecimpunctata_580 | 0.192 |
| Scymnus_camptodromus_719 | Tytthaspis_sedecimpunctata_584 | 0.192 |
| Hippodamia_parenthesis_433 | Scymnus_suturalis_832 | 0.192 |
| Hippodamia_parenthesis_440 | Scymnus_suturalis_832 | 0.192 |
| Exochomus_nigromaculatus_48 | Scymnus_suturalis_833 | 0.192 |
| Hippodamia_parenthesis_433 | Scymnus_suturalis_835 | 0.192 |
| Hippodamia_parenthesis_440 | Scymnus_suturalis_835 | 0.192 |
| Hippodamia_parenthesis_433 | Scymnus_suturalis_836 | 0.192 |
| Hippodamia_parenthesis_440 | Scymnus_suturalis_836 | 0.192 |
| Hippodamia_parenthesis_433 | Scymnus_suturalis_837 | 0.192 |
| Hippodamia_parenthesis_440 | Scymnus_suturalis_837 | 0.192 |
| Hippodamia_parenthesis_433 | Scymnus_suturalis_839 | 0.192 |
| Hippodamia_parenthesis_440 | Scymnus_suturalis_839 | 0.192 |
| Scymnus_camptodromus_715 | Subcoccinella_vigintiquatuorpunctata_622 | 0.192 |
| Scymnus_camptodromus_715 | Subcoccinella_vigintiquatuorpunctata_626 | 0.192 |
| Hyperaspis_lateralis_654 | Scymnus_sinuanodulus_831 | 0.192 |
| Hyperaspis_lateralis_656 | Scymnus_sinuanodulus_831 | 0.192 |
| Coccidula_scutellata_80 | Subcoccinella_vigintiquatuorpunctata_623 | 0.192 |
| Coccidula_scutellata_77 | Subcoccinella_vigintiquatuorpunctata_624 | 0.192 |
| Scymnus_nubilus_797 | Subcoccinella_vigintiquatuorpunctata_627 | 0.192 |
| Hippodamia_undecimnotata_460 | Tytthaspis_sedecimpunctata_586 | 0.192 |
| Hippodamia_undecimnotata_461 | Tytthaspis_sedecimpunctata_586 | 0.192 |
| Hippodamia_undecimnotata_462 | Tytthaspis_sedecimpunctata_586 | 0.192 |
| Hippodamia_undecimnotata_463 | Tytthaspis_sedecimpunctata_586 | 0.192 |
| Hippodamia_undecimnotata_464 | Tytthaspis_sedecimpunctata_586 | 0.192 |
| Hippodamia_undecimnotata_465 | Tytthaspis_sedecimpunctata_586 | 0.192 |
| Hippodamia_undecimnotata_466 | Tytthaspis_sedecimpunctata_586 | 0.192 |
| Hippodamia_undecimnotata_460 | Tytthaspis_sedecimpunctata_587 | 0.192 |
| Hippodamia_undecimnotata_461 | Tytthaspis_sedecimpunctata_587 | 0.192 |
| Hippodamia_undecimnotata_462 | Tytthaspis_sedecimpunctata_587 | 0.192 |
| Hippodamia_undecimnotata_463 | Tytthaspis_sedecimpunctata_587 | 0.192 |
| Hippodamia_undecimnotata_464 | Tytthaspis_sedecimpunctata_587 | 0.192 |
| Hippodamia_undecimnotata_465 | Tytthaspis_sedecimpunctata_587 | 0.192 |
| Hippodamia_undecimnotata_466 | Tytthaspis_sedecimpunctata_587 | 0.192 |

Supplementary Table S4 (continued)

| | | |
|---|---|---|
| Scymnus_frontalis_735 | Scymnus_suturalis_833 | 0.192 |
| Coccidula_scutellata_78 | Subcoccinella_vigintiquatuorpunctata_620 | 0.192 |
| Coccidula_scutellata_79 | Subcoccinella_vigintiquatuorpunctata_620 | 0.192 |
| Coccidula_scutellata_78 | Subcoccinella_vigintiquatuorpunctata_621 | 0.192 |
| Coccidula_scutellata_79 | Subcoccinella_vigintiquatuorpunctata_621 | 0.192 |
| Coccidula_scutellata_78 | Subcoccinella_vigintiquatuorpunctata_622 | 0.192 |
| Coccidula_scutellata_79 | Subcoccinella_vigintiquatuorpunctata_622 | 0.192 |
| Coccidula_scutellata_81 | Subcoccinella_vigintiquatuorpunctata_624 | 0.192 |
| Coccidula_scutellata_82 | Subcoccinella_vigintiquatuorpunctata_624 | 0.192 |
| Rhyzobius_litura_96 | Subcoccinella_vigintiquatuorpunctata_625 | 0.192 |
| Coccidula_scutellata_80 | Subcoccinella_vigintiquatuorpunctata_626 | 0.192 |
| Coccidula_scutellata_78 | Subcoccinella_vigintiquatuorpunctata_628 | 0.192 |
| Coccidula_scutellata_79 | Subcoccinella_vigintiquatuorpunctata_628 | 0.192 |
| Coccidula_scutellata_78 | Subcoccinella_vigintiquatuorpunctata_629 | 0.192 |
| Coccidula_scutellata_79 | Subcoccinella_vigintiquatuorpunctata_629 | 0.192 |
| Coccidula_scutellata_78 | Subcoccinella_vigintiquatuorpunctata_631 | 0.192 |
| Coccidula_scutellata_79 | Subcoccinella_vigintiquatuorpunctata_631 | 0.192 |
| Hippodamia_parenthesis_432 | Vibidia_duodecimguttata_596 | 0.192 |
| Hippodamia_parenthesis_434 | Vibidia_duodecimguttata_596 | 0.192 |
| Hippodamia_parenthesis_435 | Vibidia_duodecimguttata_596 | 0.192 |
| Hippodamia_parenthesis_437 | Vibidia_duodecimguttata_596 | 0.192 |
| Hippodamia_parenthesis_438 | Vibidia_duodecimguttata_596 | 0.192 |
| Hippodamia_parenthesis_440 | Vibidia_duodecimguttata_596 | 0.192 |
| Hippodamia_parenthesis_441 | Vibidia_duodecimguttata_596 | 0.192 |
| Hippodamia_parenthesis_442 | Vibidia_duodecimguttata_596 | 0.192 |
| Hippodamia_parenthesis_439 | Vibidia_duodecimguttata_597 | 0.192 |
| Hippodamia_parenthesis_439 | Vibidia_duodecimguttata_600 | 0.192 |
| Calvia_quatuordecimguttata_209 | Scymnus_sinuanodulus_828 | 0.192 |
| Hippodamia_convergens_400 | Scymnus_suturalis_838 | 0.192 |
| Hippodamia_convergens_400 | Scymnus_suturalis_840 | 0.192 |
| Hippodamia_convergens_395 | Scymnus_suturalis_843 | 0.192 |
| Hippodamia_convergens_396 | Scymnus_suturalis_843 | 0.192 |
| Hippodamia_convergens_403 | Scymnus_suturalis_843 | 0.192 |
| Hippodamia_moesta_410 | Scymnus_suturalis_843 | 0.192 |
| Hippodamia_convergens_395 | Scymnus_suturalis_845 | 0.192 |
| Hippodamia_convergens_396 | Scymnus_suturalis_845 | 0.192 |
| Hippodamia_convergens_403 | Scymnus_suturalis_845 | 0.192 |
| Hippodamia_moesta_410 | Scymnus_suturalis_845 | 0.192 |
| Scymnus_mitior_780 | Subcoccinella_vigintiquatuorpunctata_622 | 0.192 |
| Scymnus_haemorrhoidalis_747 | Subcoccinella_vigintiquatuorpunctata_625 | 0.192 |
| Scymnus_haemorrhoidalis_748 | Subcoccinella_vigintiquatuorpunctata_625 | 0.192 |
| Aphidecta_obliterata_178 | Vibidia_duodecimguttata_597 | 0.192 |
| Coccinella_transversoguttata_267 | Vibidia_duodecimguttata_599 | 0.192 |
| Coccinella_transversoguttata_268 | Vibidia_duodecimguttata_599 | 0.192 |
| Coccinella_transversoguttata_269 | Vibidia_duodecimguttata_599 | 0.192 |
| Coccinella_transversoguttata_270 | Vibidia_duodecimguttata_599 | 0.192 |
| Coccinella_transversoguttata_271 | Vibidia_duodecimguttata_599 | 0.192 |
| Aphidecta_obliterata_178 | Vibidia_duodecimguttata_600 | 0.192 |
| Coccinella_transversoguttata_267 | Vibidia_duodecimguttata_601 | 0.192 |
| Coccinella_transversoguttata_268 | Vibidia_duodecimguttata_601 | 0.192 |
| Coccinella_transversoguttata_269 | Vibidia_duodecimguttata_601 | 0.192 |
| Coccinella_transversoguttata_270 | Vibidia_duodecimguttata_601 | 0.192 |
| Coccinella_transversoguttata_271 | Vibidia_duodecimguttata_601 | 0.192 |
| Coccinella_transversoguttata_267 | Vibidia_duodecimguttata_602 | 0.192 |
| Coccinella_transversoguttata_268 | Vibidia_duodecimguttata_602 | 0.192 |
| Coccinella_transversoguttata_269 | Vibidia_duodecimguttata_602 | 0.192 |

Supplementary Table S4 (continued)

| | | |
|---|---|---|
| Coccinella_transversoguttata_270 | Vibidia_duodecimguttata_602 | 0.192 |
| Coccinella_transversoguttata_271 | Vibidia_duodecimguttata_602 | 0.192 |
| Calvia_quatuordecimguttata_218 | Scymnus_sinuanodulus_830 | 0.192 |
| Scymnus_nubilus_796 | Scymnus_suturalis_833 | 0.192 |
| Scymnus_nubilus_798 | Scymnus_suturalis_833 | 0.192 |
| Scymnus_nubilus_800 | Scymnus_suturalis_833 | 0.192 |
| Scymnus_nubilus_801 | Scymnus_suturalis_833 | 0.192 |
| Scymnus_nubilus_802 | Scymnus_suturalis_833 | 0.192 |
| Scymnus_nubilus_805 | Scymnus_suturalis_833 | 0.192 |
| Scymnus_nubilus_806 | Scymnus_suturalis_833 | 0.192 |
| Scymnus_nubilus_807 | Scymnus_suturalis_833 | 0.192 |
| Scymnus_nubilus_810 | Scymnus_suturalis_833 | 0.192 |
| Hippodamia_convergens_400 | Scymnus_suturalis_834 | 0.192 |
| Hippodamia_convergens_400 | Scymnus_suturalis_837 | 0.192 |
| Scymnus_nubilus_803 | Scymnus_suturalis_838 | 0.192 |
| Scymnus_nubilus_804 | Scymnus_suturalis_838 | 0.192 |
| Scymnus_nubilus_808 | Scymnus_suturalis_838 | 0.192 |
| Scymnus_nubilus_809 | Scymnus_suturalis_838 | 0.192 |
| Scymnus_nubilus_800 | Subcoccinella_vigintiquatuorpunctata_619 | 0.192 |
| Scymnus_nubilus_801 | Subcoccinella_vigintiquatuorpunctata_619 | 0.192 |
| Scymnus_nubilus_802 | Subcoccinella_vigintiquatuorpunctata_619 | 0.192 |
| Scymnus_nubilus_805 | Subcoccinella_vigintiquatuorpunctata_619 | 0.192 |
| Scymnus_nubilus_806 | Subcoccinella_vigintiquatuorpunctata_619 | 0.192 |
| Scymnus_nubilus_807 | Subcoccinella_vigintiquatuorpunctata_619 | 0.192 |
| Scymnus_nubilus_810 | Subcoccinella_vigintiquatuorpunctata_619 | 0.192 |
| Scymnus_camptodromus_715 | Subcoccinella_vigintiquatuorpunctata_619 | 0.192 |
| Scymnus_haemorrhoidalis_749 | Subcoccinella_vigintiquatuorpunctata_625 | 0.192 |
| Scymnus_haemorrhoidalis_750 | Subcoccinella_vigintiquatuorpunctata_625 | 0.192 |
| Aphidecta_obliterata_183 | Vibidia_duodecimguttata_599 | 0.192 |
| Aphidecta_obliterata_183 | Vibidia_duodecimguttata_601 | 0.192 |
| Aphidecta_obliterata_183 | Vibidia_duodecimguttata_602 | 0.192 |
| Calvia_quatuordecimguttata_204 | Scymnus_sinuanodulus_830 | 0.192 |
| Calvia_quatuordecimguttata_208 | Scymnus_sinuanodulus_830 | 0.192 |
| Calvia_quatuordecimguttata_217 | Scymnus_sinuanodulus_830 | 0.192 |
| Coelophora_inaequalis_319 | Vibidia_duodecimguttata_603 | 0.192 |
| Brumus_quadripustulatus_9 | Scymnus_sinuanodulus_829 | 0.192 |
| Platynaspis_luteorubra_51 | Scymnus_sinuanodulus_829 | 0.192 |
| Platynaspis_luteorubra_53 | Scymnus_sinuanodulus_829 | 0.192 |
| Platynaspis_luteorubra_58 | Scymnus_sinuanodulus_829 | 0.192 |
| Psyllobora_vigintimaculata_573 | Vibidia_duodecimguttata_596 | 0.192 |
| Psyllobora_vigintimaculata_573 | Vibidia_duodecimguttata_598 | 0.192 |
| Coccidula_scutellata_83 | Subcoccinella_vigintiquatuorpunctata_619 | 0.192 |
| Hippodamia_parenthesis_436 | Vibidia_duodecimguttata_596 | 0.192 |
| Harmonia_quadripunctata_376 | Vibidia_duodecimguttata_599 | 0.192 |
| Harmonia_quadripunctata_378 | Vibidia_duodecimguttata_599 | 0.192 |
| Harmonia_quadripunctata_376 | Vibidia_duodecimguttata_601 | 0.192 |
| Harmonia_quadripunctata_378 | Vibidia_duodecimguttata_601 | 0.192 |
| Harmonia_quadripunctata_376 | Vibidia_duodecimguttata_602 | 0.192 |
| Harmonia_quadripunctata_378 | Vibidia_duodecimguttata_602 | 0.192 |
| Coccinella_magnifica_228 | Vibidia_duodecimguttata_603 | 0.192 |
| Coccinella_magnifica_229 | Vibidia_duodecimguttata_603 | 0.192 |
| Coccinella_magnifica_230 | Vibidia_duodecimguttata_603 | 0.192 |
| Coccinella_magnifica_231 | Vibidia_duodecimguttata_603 | 0.192 |
| Harmonia_quadripunctata_382 | Vibidia_duodecimguttata_603 | 0.192 |
| Calvia_quatuordecimguttata_205 | Scymnus_sinuanodulus_830 | 0.192 |
| Coccidula_scutellata_84 | Subcoccinella_vigintiquatuorpunctata_627 | 0.193 |

| | | |
|---|---|---|
| Coccidula_scutellata_84 | Subcoccinella_vigintiquatuorpunctata_630 | 0.193 |
| Oenopia_conglobata_522 | Scymnus_suturalis_843 | 0.193 |
| Harmonia_quadripunctata_376 | Vibidia_duodecimguttata_597 | 0.193 |
| Harmonia_quadripunctata_378 | Vibidia_duodecimguttata_597 | 0.193 |
| Harmonia_quadripunctata_376 | Vibidia_duodecimguttata_600 | 0.193 |
| Harmonia_quadripunctata_378 | Vibidia_duodecimguttata_600 | 0.193 |
| Hippodamia_glacialis_408 | Scymnus_suturalis_833 | 0.193 |
| Hippodamia_glacialis_409 | Scymnus_suturalis_833 | 0.193 |
| Hippodamia_glacialis_408 | Scymnus_suturalis_838 | 0.193 |
| Hippodamia_glacialis_409 | Scymnus_suturalis_838 | 0.193 |
| Hippodamia_glacialis_408 | Scymnus_suturalis_840 | 0.193 |
| Hippodamia_glacialis_409 | Scymnus_suturalis_840 | 0.193 |
| Scymnus_apetzi_694 | Subcoccinella_vigintiquatuorpunctata_623 | 0.193 |
| Hyperaspis_lateralis_654 | Scymnus_sinuanodulus_829 | 0.193 |
| Hyperaspis_lateralis_656 | Scymnus_sinuanodulus_829 | 0.193 |
| Calvia_quatuordecimguttata_203 | Scymnus_sinuanodulus_831 | 0.193 |
| Hippodamia_glacialis_407 | Scymnus_suturalis_841 | 0.193 |
| Hippodamia_glacialis_407 | Scymnus_suturalis_842 | 0.193 |
| Hippodamia_glacialis_407 | Scymnus_suturalis_844 | 0.193 |
| Hippodamia_glacialis_407 | Scymnus_suturalis_846 | 0.193 |
| Hippodamia_glacialis_407 | Scymnus_suturalis_847 | 0.193 |
| Hippodamia_glacialis_407 | Scymnus_suturalis_848 | 0.193 |
| Brachiacantha_rotunda_632 | Subcoccinella_vigintiquatuorpunctata_620 | 0.193 |
| Brachiacantha_rotunda_633 | Subcoccinella_vigintiquatuorpunctata_620 | 0.193 |
| Brachiacantha_rotunda_635 | Subcoccinella_vigintiquatuorpunctata_620 | 0.193 |
| Brachiacantha_rotunda_632 | Subcoccinella_vigintiquatuorpunctata_621 | 0.193 |
| Brachiacantha_rotunda_633 | Subcoccinella_vigintiquatuorpunctata_621 | 0.193 |
| Brachiacantha_rotunda_635 | Subcoccinella_vigintiquatuorpunctata_621 | 0.193 |
| Brachiacantha_rotunda_632 | Subcoccinella_vigintiquatuorpunctata_628 | 0.193 |
| Brachiacantha_rotunda_633 | Subcoccinella_vigintiquatuorpunctata_628 | 0.193 |
| Brachiacantha_rotunda_635 | Subcoccinella_vigintiquatuorpunctata_628 | 0.193 |
| Brachiacantha_rotunda_632 | Subcoccinella_vigintiquatuorpunctata_629 | 0.193 |
| Brachiacantha_rotunda_633 | Subcoccinella_vigintiquatuorpunctata_629 | 0.193 |
| Brachiacantha_rotunda_635 | Subcoccinella_vigintiquatuorpunctata_629 | 0.193 |
| Brachiacantha_rotunda_636 | Subcoccinella_vigintiquatuorpunctata_630 | 0.193 |
| Brachiacantha_rotunda_632 | Subcoccinella_vigintiquatuorpunctata_631 | 0.193 |
| Brachiacantha_rotunda_633 | Subcoccinella_vigintiquatuorpunctata_631 | 0.193 |
| Brachiacantha_rotunda_635 | Subcoccinella_vigintiquatuorpunctata_631 | 0.193 |
| Coccinella_trifasciata_273 | Vibidia_duodecimguttata_597 | 0.193 |
| Coccinella_trifasciata_273 | Vibidia_duodecimguttata_600 | 0.193 |
| Hippodamia_glacialis_407 | Scymnus_suturalis_832 | 0.193 |
| Hippodamia_glacialis_408 | Scymnus_suturalis_834 | 0.193 |
| Hippodamia_glacialis_409 | Scymnus_suturalis_834 | 0.193 |
| Hippodamia_glacialis_407 | Scymnus_suturalis_835 | 0.193 |
| Hippodamia_glacialis_407 | Scymnus_suturalis_836 | 0.193 |
| Hippodamia_glacialis_408 | Scymnus_suturalis_837 | 0.193 |
| Hippodamia_glacialis_409 | Scymnus_suturalis_837 | 0.193 |
| Hippodamia_glacialis_407 | Scymnus_suturalis_839 | 0.193 |
| Oenopia_conglobata_522 | Scymnus_suturalis_840 | 0.193 |
| Scymnus_apetzi_694 | Subcoccinella_vigintiquatuorpunctata_622 | 0.193 |
| Scymnus_apetzi_694 | Subcoccinella_vigintiquatuorpunctata_626 | 0.193 |
| Scymnus_camptodromus_717 | Subcoccinella_vigintiquatuorpunctata_623 | 0.193 |
| Brachiacantha_rotunda_632 | Subcoccinella_vigintiquatuorpunctata_623 | 0.193 |
| Brachiacantha_rotunda_633 | Subcoccinella_vigintiquatuorpunctata_623 | 0.193 |
| Brachiacantha_rotunda_635 | Subcoccinella_vigintiquatuorpunctata_623 | 0.193 |
| Coccinella_trifasciata_272 | Vibidia_duodecimguttata_596 | 0.193 |

| | | |
|---|---|---|
| Coccinella_trifasciata_276 | Vibidia_duodecimguttata_596 | 0.193 |
| Coccinella_trifasciata_278 | Vibidia_duodecimguttata_596 | 0.193 |
| Coccinella_trifasciata_279 | Vibidia_duodecimguttata_596 | 0.193 |
| Coccinella_trifasciata_272 | Vibidia_duodecimguttata_598 | 0.193 |
| Coccinella_trifasciata_276 | Vibidia_duodecimguttata_598 | 0.193 |
| Coccinella_trifasciata_278 | Vibidia_duodecimguttata_598 | 0.193 |
| Coccinella_trifasciata_279 | Vibidia_duodecimguttata_598 | 0.193 |
| Coccinella_trifasciata_273 | Vibidia_duodecimguttata_603 | 0.193 |
| Scymnus_camptodromus_717 | Subcoccinella_vigintiquatuorpunctata_622 | 0.193 |
| Scymnus_camptodromus_717 | Subcoccinella_vigintiquatuorpunctata_626 | 0.193 |
| Scymnus_apetzi_695 | Subcoccinella_vigintiquatuorpunctata_624 | 0.193 |
| Chilocorus_nigritus_30 | Vibidia_duodecimguttata_598 | 0.193 |
| Chilocorus_nigritus_30 | Vibidia_duodecimguttata_601 | 0.193 |
| Hyperaspis_reppensis_662 | Tytthaspis_sedecimpunctata_577 | 0.193 |
| Hyperaspis_reppensis_663 | Tytthaspis_sedecimpunctata_577 | 0.193 |
| Hyperaspis_reppensis_662 | Tytthaspis_sedecimpunctata_578 | 0.193 |
| Hyperaspis_reppensis_663 | Tytthaspis_sedecimpunctata_578 | 0.193 |
| Hyperaspis_reppensis_662 | Tytthaspis_sedecimpunctata_579 | 0.193 |
| Hyperaspis_reppensis_663 | Tytthaspis_sedecimpunctata_579 | 0.193 |
| Hyperaspis_reppensis_662 | Tytthaspis_sedecimpunctata_581 | 0.193 |
| Hyperaspis_reppensis_663 | Tytthaspis_sedecimpunctata_581 | 0.193 |
| Hyperaspis_reppensis_662 | Tytthaspis_sedecimpunctata_582 | 0.193 |
| Hyperaspis_reppensis_663 | Tytthaspis_sedecimpunctata_582 | 0.193 |
| Hyperaspis_reppensis_662 | Tytthaspis_sedecimpunctata_590 | 0.193 |
| Hyperaspis_reppensis_663 | Tytthaspis_sedecimpunctata_590 | 0.193 |
| Hyperaspis_reppensis_662 | Tytthaspis_sedecimpunctata_594 | 0.193 |
| Hyperaspis_reppensis_663 | Tytthaspis_sedecimpunctata_594 | 0.193 |
| Hyperaspis_reppensis_662 | Tytthaspis_sedecimpunctata_595 | 0.193 |
| Hyperaspis_reppensis_663 | Tytthaspis_sedecimpunctata_595 | 0.193 |
| Hyperaspis_lateralis_655 | Tytthaspis_sedecimpunctata_580 | 0.193 |
| Hyperaspis_lateralis_655 | Tytthaspis_sedecimpunctata_584 | 0.193 |
| Scymnus_apetzi_694 | Subcoccinella_vigintiquatuorpunctata_619 | 0.193 |
| Scymnus_apetzi_693 | Subcoccinella_vigintiquatuorpunctata_625 | 0.193 |
| Chilocorus_bipustulatus_18 | Scymnus_suturalis_843 | 0.193 |
| Chilocorus_bipustulatus_20 | Scymnus_suturalis_843 | 0.193 |
| Chilocorus_bipustulatus_24 | Scymnus_suturalis_843 | 0.193 |
| Chilocorus_bipustulatus_18 | Scymnus_suturalis_845 | 0.193 |
| Chilocorus_bipustulatus_20 | Scymnus_suturalis_845 | 0.193 |
| Chilocorus_bipustulatus_24 | Scymnus_suturalis_845 | 0.193 |
| Hyperaspis_campestris_651 | Tytthaspis_sedecimpunctata_584 | 0.193 |
| Hyperaspis_campestris_652 | Tytthaspis_sedecimpunctata_584 | 0.193 |
| Hyperaspis_campestris_653 | Tytthaspis_sedecimpunctata_584 | 0.193 |
| Chilocorus_bipustulatus_18 | Scymnus_suturalis_833 | 0.193 |
| Chilocorus_bipustulatus_20 | Scymnus_suturalis_833 | 0.193 |
| Chilocorus_bipustulatus_24 | Scymnus_suturalis_833 | 0.193 |
| Hyperaspis_lateralis_656 | Tytthaspis_sedecimpunctata_585 | 0.193 |
| Platynaspis_luteorubra_50 | Scymnus_sinuanodulus_830 | 0.193 |
| Hyperaspis_campestris_651 | Tytthaspis_sedecimpunctata_589 | 0.193 |
| Hyperaspis_campestris_652 | Tytthaspis_sedecimpunctata_589 | 0.193 |
| Hyperaspis_campestris_653 | Tytthaspis_sedecimpunctata_589 | 0.193 |
| Hyperaspis_oregona_658 | Tytthaspis_sedecimpunctata_591 | 0.193 |
| Coccinella_undecimpunctata_291 | Scymnus_sinuanodulus_829 | 0.193 |
| Nephus_quadrimaculatus_668 | Scymnus_sinuanodulus_829 | 0.193 |
| Coccinella_undecimpunctata_282 | Vibidia_duodecimguttata_597 | 0.193 |
| Coccinella_undecimpunctata_283 | Vibidia_duodecimguttata_597 | 0.193 |
| Coccinella_undecimpunctata_284 | Vibidia_duodecimguttata_597 | 0.193 |

| | | |
|---|---|---|
| Coelophora_inaequalis_313 | Vibidia_duodecimguttata_597 | 0.193 |
| Coelophora_inaequalis_326 | Vibidia_duodecimguttata_597 | 0.193 |
| Coccinella_undecimpunctata_282 | Vibidia_duodecimguttata_600 | 0.193 |
| Coccinella_undecimpunctata_283 | Vibidia_duodecimguttata_600 | 0.193 |
| Coccinella_undecimpunctata_284 | Vibidia_duodecimguttata_600 | 0.193 |
| Coelophora_inaequalis_313 | Vibidia_duodecimguttata_600 | 0.193 |
| Coelophora_inaequalis_326 | Vibidia_duodecimguttata_600 | 0.193 |
| Coelophora_inaequalis_315 | Vibidia_duodecimguttata_603 | 0.193 |
| Hyperaspis_oregona_659 | Tytthaspis_sedecimpunctata_583 | 0.193 |
| Hyperaspis_campestris_651 | Tytthaspis_sedecimpunctata_587 | 0.193 |
| Hyperaspis_campestris_652 | Tytthaspis_sedecimpunctata_587 | 0.193 |
| Hyperaspis_campestris_653 | Tytthaspis_sedecimpunctata_587 | 0.193 |
| Chilocorus_renipustulatus_34 | Tytthaspis_sedecimpunctata_589 | 0.193 |
| Platynaspis_luteorubra_61 | Tytthaspis_sedecimpunctata_591 | 0.193 |
| Platynaspis_luteorubra_50 | Scymnus_sinuanodulus_828 | 0.193 |
| Hyperaspis_oregona_659 | Tytthaspis_sedecimpunctata_584 | 0.193 |
| Platynaspis_luteorubra_52 | Tytthaspis_sedecimpunctata_584 | 0.193 |
| Platynaspis_luteorubra_56 | Tytthaspis_sedecimpunctata_584 | 0.193 |
| Platynaspis_luteorubra_60 | Tytthaspis_sedecimpunctata_584 | 0.193 |
| Chilocorus_renipustulatus_34 | Tytthaspis_sedecimpunctata_587 | 0.193 |
| Chilocorus_renipustulatus_39 | Tytthaspis_sedecimpunctata_589 | 0.193 |
| Scymnus_mitior_780 | Tytthaspis_sedecimpunctata_589 | 0.193 |
| Chilocorus_renipustulatus_34 | Tytthaspis_sedecimpunctata_591 | 0.193 |
| Hyperaspis_reppensis_662 | Stethorus_punctillum_852 | 0.193 |
| Hyperaspis_reppensis_663 | Stethorus_punctillum_852 | 0.193 |
| Platynaspis_luteorubra_54 | Tytthaspis_sedecimpunctata_584 | 0.193 |
| Platynaspis_luteorubra_57 | Tytthaspis_sedecimpunctata_584 | 0.193 |
| Platynaspis_luteorubra_59 | Tytthaspis_sedecimpunctata_584 | 0.193 |
| Chilocorus_renipustulatus_31 | Tytthaspis_sedecimpunctata_587 | 0.193 |
| Chilocorus_renipustulatus_32 | Tytthaspis_sedecimpunctata_587 | 0.193 |
| Chilocorus_renipustulatus_35 | Tytthaspis_sedecimpunctata_587 | 0.193 |
| Chilocorus_renipustulatus_36 | Tytthaspis_sedecimpunctata_587 | 0.193 |
| Chilocorus_renipustulatus_37 | Tytthaspis_sedecimpunctata_587 | 0.193 |
| Chilocorus_renipustulatus_38 | Tytthaspis_sedecimpunctata_587 | 0.193 |
| Chilocorus_renipustulatus_40 | Tytthaspis_sedecimpunctata_587 | 0.193 |
| Chilocorus_renipustulatus_41 | Tytthaspis_sedecimpunctata_587 | 0.193 |
| Chilocorus_renipustulatus_42 | Tytthaspis_sedecimpunctata_587 | 0.193 |
| Scymnus_mitior_780 | Tytthaspis_sedecimpunctata_587 | 0.193 |
| Hyperaspis_reppensis_666 | Tytthaspis_sedecimpunctata_591 | 0.193 |
| Myzia_oblongoguttata_506 | Scymnus_suturalis_833 | 0.193 |
| Scymnus_camptodromus_717 | Subcoccinella_vigintiquatuorpunctata_619 | 0.193 |
| Calvia_quatuordecimguttata_203 | Scymnus_sinuanodulus_829 | 0.193 |
| Myzia_oblongoguttata_506 | Scymnus_suturalis_841 | 0.193 |
| Myzia_oblongoguttata_506 | Scymnus_suturalis_842 | 0.193 |
| Chilocorus_renipustulatus_39 | Scymnus_suturalis_843 | 0.193 |
| Myzia_oblongoguttata_516 | Scymnus_suturalis_843 | 0.193 |
| Myzia_oblongoguttata_506 | Scymnus_suturalis_844 | 0.193 |
| Chilocorus_renipustulatus_39 | Scymnus_suturalis_845 | 0.193 |
| Myzia_oblongoguttata_516 | Scymnus_suturalis_845 | 0.193 |
| Myzia_oblongoguttata_506 | Scymnus_suturalis_846 | 0.193 |
| Myzia_oblongoguttata_506 | Scymnus_suturalis_847 | 0.193 |
| Myzia_oblongoguttata_506 | Scymnus_suturalis_848 | 0.193 |
| Scymnus_loewii_771 | Subcoccinella_vigintiquatuorpunctata_627 | 0.193 |
| Scymnus_loewii_770 | Subcoccinella_vigintiquatuorpunctata_630 | 0.193 |
| Coccinella_transversalis_258 | Tytthaspis_sedecimpunctata_584 | 0.193 |
| Scymnus_loewii_774 | Tytthaspis_sedecimpunctata_591 | 0.193 |

| | | |
|---|---|---|
| Brumus_quadripustulatus_11 | Scymnus_sinuanodulus_830 | 0.193 |
| Brumus_quadripustulatus_13 | Scymnus_sinuanodulus_830 | 0.193 |
| Brumus_quadripustulatus_14 | Scymnus_sinuanodulus_830 | 0.193 |
| Brumus_quadripustulatus_17 | Scymnus_sinuanodulus_830 | 0.193 |
| Brumus_quadripustulatus_4 | Scymnus_sinuanodulus_830 | 0.193 |
| Brumus_quadripustulatus_7 | Scymnus_sinuanodulus_830 | 0.193 |
| Myzia_oblongoguttata_506 | Scymnus_suturalis_832 | 0.193 |
| Chilocorus_renipustulatus_39 | Scymnus_suturalis_834 | 0.193 |
| Myzia_oblongoguttata_506 | Scymnus_suturalis_835 | 0.193 |
| Myzia_oblongoguttata_506 | Scymnus_suturalis_836 | 0.193 |
| Myzia_oblongoguttata_506 | Scymnus_suturalis_837 | 0.193 |
| Chilocorus_stigma_45 | Scymnus_suturalis_838 | 0.193 |
| Myzia_oblongoguttata_506 | Scymnus_suturalis_839 | 0.193 |
| Chilocorus_stigma_45 | Scymnus_suturalis_840 | 0.193 |
| Coelophora_inaequalis_310 | Vibidia_duodecimguttata_596 | 0.193 |
| Coelophora_inaequalis_311 | Vibidia_duodecimguttata_596 | 0.193 |
| Coelophora_inaequalis_316 | Vibidia_duodecimguttata_596 | 0.193 |
| Coelophora_inaequalis_323 | Vibidia_duodecimguttata_596 | 0.193 |
| Coelophora_inaequalis_324 | Vibidia_duodecimguttata_596 | 0.193 |
| Coccinella_undecimpunctata_285 | Vibidia_duodecimguttata_598 | 0.193 |
| Coccinella_undecimpunctata_286 | Vibidia_duodecimguttata_598 | 0.193 |
| Coccinella_undecimpunctata_288 | Vibidia_duodecimguttata_598 | 0.193 |
| Coccinella_undecimpunctata_291 | Vibidia_duodecimguttata_598 | 0.193 |
| Coelophora_inaequalis_310 | Vibidia_duodecimguttata_598 | 0.193 |
| Coelophora_inaequalis_311 | Vibidia_duodecimguttata_598 | 0.193 |
| Coelophora_inaequalis_316 | Vibidia_duodecimguttata_598 | 0.193 |
| Coelophora_inaequalis_323 | Vibidia_duodecimguttata_598 | 0.193 |
| Coelophora_inaequalis_324 | Vibidia_duodecimguttata_598 | 0.193 |
| Coccinella_undecimpunctata_285 | Vibidia_duodecimguttata_599 | 0.193 |
| Coccinella_undecimpunctata_286 | Vibidia_duodecimguttata_599 | 0.193 |
| Coccinella_undecimpunctata_288 | Vibidia_duodecimguttata_599 | 0.193 |
| Coelophora_inaequalis_312 | Vibidia_duodecimguttata_599 | 0.193 |
| Coelophora_inaequalis_314 | Vibidia_duodecimguttata_599 | 0.193 |
| Coelophora_inaequalis_317 | Vibidia_duodecimguttata_599 | 0.193 |
| Coelophora_inaequalis_325 | Vibidia_duodecimguttata_599 | 0.193 |
| Coelophora_inaequalis_312 | Vibidia_duodecimguttata_601 | 0.193 |
| Coelophora_inaequalis_314 | Vibidia_duodecimguttata_601 | 0.193 |
| Coelophora_inaequalis_317 | Vibidia_duodecimguttata_601 | 0.193 |
| Coelophora_inaequalis_325 | Vibidia_duodecimguttata_601 | 0.193 |
| Myzia_oblongoguttata_515 | Vibidia_duodecimguttata_601 | 0.193 |
| Coccinella_undecimpunctata_285 | Vibidia_duodecimguttata_602 | 0.193 |
| Coccinella_undecimpunctata_286 | Vibidia_duodecimguttata_602 | 0.193 |
| Coccinella_undecimpunctata_288 | Vibidia_duodecimguttata_602 | 0.193 |
| Coelophora_inaequalis_312 | Vibidia_duodecimguttata_602 | 0.193 |
| Coelophora_inaequalis_314 | Vibidia_duodecimguttata_602 | 0.193 |
| Coelophora_inaequalis_317 | Vibidia_duodecimguttata_602 | 0.193 |
| Coelophora_inaequalis_325 | Vibidia_duodecimguttata_602 | 0.193 |
| Chilocorus_stigma_45 | Scymnus_suturalis_843 | 0.193 |
| Hyperaspis_reppensis_662 | Stethorus_punctillum_855 | 0.193 |
| Hyperaspis_reppensis_663 | Stethorus_punctillum_855 | 0.193 |
| Coccinella_transversalis_257 | Tytthaspis_sedecimpunctata_587 | 0.193 |
| Coccinella_transversalis_259 | Tytthaspis_sedecimpunctata_587 | 0.193 |
| Coccinella_transversalis_260 | Tytthaspis_sedecimpunctata_587 | 0.193 |
| Coccinella_transversalis_261 | Tytthaspis_sedecimpunctata_587 | 0.193 |
| Coccinella_transversalis_262 | Tytthaspis_sedecimpunctata_587 | 0.193 |
| Coccinella_transversalis_263 | Tytthaspis_sedecimpunctata_587 | 0.193 |

| | | |
|---|---|---|
| Coccinella_transversalis_264 | Tytthaspis_sedecimpunctata_587 | 0.193 |
| Coccinella_transversalis_257 | Tytthaspis_sedecimpunctata_588 | 0.193 |
| Coccinella_transversalis_259 | Tytthaspis_sedecimpunctata_588 | 0.193 |
| Coccinella_transversalis_260 | Tytthaspis_sedecimpunctata_588 | 0.193 |
| Coccinella_transversalis_261 | Tytthaspis_sedecimpunctata_588 | 0.193 |
| Coccinella_transversalis_262 | Tytthaspis_sedecimpunctata_588 | 0.193 |
| Coccinella_transversalis_263 | Tytthaspis_sedecimpunctata_588 | 0.193 |
| Coccinella_transversalis_264 | Tytthaspis_sedecimpunctata_588 | 0.193 |
| Rhyzobius_chrysomeloides_85 | Tytthaspis_sedecimpunctata_588 | 0.193 |
| Rhyzobius_chrysomeloides_86 | Tytthaspis_sedecimpunctata_588 | 0.193 |
| Rhyzobius_chrysomeloides_87 | Tytthaspis_sedecimpunctata_588 | 0.193 |
| Rhyzobius_chrysomeloides_88 | Tytthaspis_sedecimpunctata_588 | 0.193 |
| Rhyzobius_chrysomeloides_89 | Tytthaspis_sedecimpunctata_588 | 0.193 |
| Rhyzobius_chrysomeloides_90 | Tytthaspis_sedecimpunctata_588 | 0.193 |
| Rhyzobius_chrysomeloides_91 | Tytthaspis_sedecimpunctata_588 | 0.193 |
| Rhyzobius_chrysomeloides_92 | Tytthaspis_sedecimpunctata_588 | 0.193 |
| Rhyzobius_chrysomeloides_93 | Tytthaspis_sedecimpunctata_588 | 0.193 |
| Rhyzobius_chrysomeloides_94 | Tytthaspis_sedecimpunctata_588 | 0.193 |
| Rhyzobius_chrysomeloides_95 | Tytthaspis_sedecimpunctata_588 | 0.193 |
| Scymnus_loewii_772 | Tytthaspis_sedecimpunctata_591 | 0.193 |
| Brumus_quadripustulatus_11 | Scymnus_sinuanodulus_828 | 0.193 |
| Brumus_quadripustulatus_13 | Scymnus_sinuanodulus_828 | 0.193 |
| Brumus_quadripustulatus_14 | Scymnus_sinuanodulus_828 | 0.193 |
| Brumus_quadripustulatus_17 | Scymnus_sinuanodulus_828 | 0.193 |
| Brumus_quadripustulatus_4 | Scymnus_sinuanodulus_828 | 0.193 |
| Brumus_quadripustulatus_7 | Scymnus_sinuanodulus_828 | 0.193 |
| Brumus_quadripustulatus_15 | Scymnus_sinuanodulus_830 | 0.193 |
| Brumus_quadripustulatus_16 | Scymnus_sinuanodulus_830 | 0.193 |
| Brumus_quadripustulatus_6 | Scymnus_sinuanodulus_830 | 0.193 |
| Brumus_quadripustulatus_8 | Scymnus_sinuanodulus_830 | 0.193 |
| Chilocorus_stigma_45 | Scymnus_suturalis_834 | 0.193 |
| Myrrha_octodecimguttata_499 | Scymnus_suturalis_838 | 0.193 |
| Myrrha_octodecimguttata_499 | Scymnus_suturalis_840 | 0.193 |
| Macronaemia_episcopalis_477 | Scymnus_suturalis_843 | 0.193 |
| Macronaemia_episcopalis_477 | Scymnus_suturalis_845 | 0.193 |
| Hyperaspis_lateralis_654 | Subcoccinella_vigintiquatuorpunctata_623 | 0.193 |
| Brumus_quadripustulatus_15 | Scymnus_sinuanodulus_828 | 0.193 |
| Brumus_quadripustulatus_16 | Scymnus_sinuanodulus_828 | 0.193 |
| Brumus_quadripustulatus_6 | Scymnus_sinuanodulus_828 | 0.193 |
| Brumus_quadripustulatus_8 | Scymnus_sinuanodulus_828 | 0.193 |
| Chilocorus_stigma_45 | Scymnus_suturalis_833 | 0.193 |
| Myrrha_octodecimguttata_499 | Scymnus_suturalis_834 | 0.193 |
| Myrrha_octodecimguttata_499 | Scymnus_suturalis_837 | 0.193 |
| Cynegetis_impunctata_614 | Subcoccinella_vigintiquatuorpunctata_620 | 0.193 |
| Cynegetis_impunctata_614 | Subcoccinella_vigintiquatuorpunctata_621 | 0.193 |
| Cynegetis_impunctata_614 | Subcoccinella_vigintiquatuorpunctata_627 | 0.193 |
| Cynegetis_impunctata_614 | Subcoccinella_vigintiquatuorpunctata_628 | 0.193 |
| Cynegetis_impunctata_614 | Subcoccinella_vigintiquatuorpunctata_629 | 0.193 |
| Cynegetis_impunctata_614 | Subcoccinella_vigintiquatuorpunctata_630 | 0.193 |
| Cynegetis_impunctata_614 | Subcoccinella_vigintiquatuorpunctata_631 | 0.193 |
| Coelophora_inaequalis_320 | Vibidia_duodecimguttata_599 | 0.193 |
| Coelophora_inaequalis_321 | Vibidia_duodecimguttata_599 | 0.193 |
| Coelophora_inaequalis_322 | Vibidia_duodecimguttata_599 | 0.193 |
| Coelophora_inaequalis_320 | Vibidia_duodecimguttata_601 | 0.193 |
| Coelophora_inaequalis_321 | Vibidia_duodecimguttata_601 | 0.193 |
| Coelophora_inaequalis_322 | Vibidia_duodecimguttata_601 | 0.193 |

| | | |
|---|---|---|
| Coelophora_inaequalis_320 | Vibidia_duodecimguttata_602 | 0.193 |
| Coelophora_inaequalis_321 | Vibidia_duodecimguttata_602 | 0.193 |
| Coelophora_inaequalis_322 | Vibidia_duodecimguttata_602 | 0.193 |
| Hyperaspis_lateralis_656 | Subcoccinella_vigintiquatuorpunctata_620 | 0.193 |
| Hyperaspis_lateralis_657 | Subcoccinella_vigintiquatuorpunctata_620 | 0.193 |
| Hyperaspis_reppensis_662 | Subcoccinella_vigintiquatuorpunctata_620 | 0.193 |
| Hyperaspis_reppensis_663 | Subcoccinella_vigintiquatuorpunctata_620 | 0.193 |
| Hyperaspis_lateralis_656 | Subcoccinella_vigintiquatuorpunctata_621 | 0.193 |
| Hyperaspis_lateralis_657 | Subcoccinella_vigintiquatuorpunctata_621 | 0.193 |
| Hyperaspis_reppensis_662 | Subcoccinella_vigintiquatuorpunctata_621 | 0.193 |
| Hyperaspis_reppensis_663 | Subcoccinella_vigintiquatuorpunctata_621 | 0.193 |
| Hyperaspis_lateralis_654 | Subcoccinella_vigintiquatuorpunctata_622 | 0.193 |
| Scymnus_ningshanensis_788 | Subcoccinella_vigintiquatuorpunctata_623 | 0.193 |
| Scymnus_ningshanensis_791 | Subcoccinella_vigintiquatuorpunctata_623 | 0.193 |
| Scymnus_ningshanensis_792 | Subcoccinella_vigintiquatuorpunctata_623 | 0.193 |
| Scymnus_ningshanensis_793 | Subcoccinella_vigintiquatuorpunctata_623 | 0.193 |
| Scymnus_ningshanensis_794 | Subcoccinella_vigintiquatuorpunctata_623 | 0.193 |
| Scymnus_ningshanensis_795 | Subcoccinella_vigintiquatuorpunctata_623 | 0.193 |
| Hyperaspis_reppensis_662 | Subcoccinella_vigintiquatuorpunctata_624 | 0.193 |
| Hyperaspis_reppensis_663 | Subcoccinella_vigintiquatuorpunctata_624 | 0.193 |
| Hyperaspis_lateralis_654 | Subcoccinella_vigintiquatuorpunctata_626 | 0.193 |
| Hyperaspis_lateralis_656 | Subcoccinella_vigintiquatuorpunctata_628 | 0.193 |
| Hyperaspis_lateralis_657 | Subcoccinella_vigintiquatuorpunctata_628 | 0.193 |
| Hyperaspis_reppensis_662 | Subcoccinella_vigintiquatuorpunctata_628 | 0.193 |
| Hyperaspis_reppensis_663 | Subcoccinella_vigintiquatuorpunctata_628 | 0.193 |
| Hyperaspis_lateralis_656 | Subcoccinella_vigintiquatuorpunctata_629 | 0.193 |
| Hyperaspis_lateralis_657 | Subcoccinella_vigintiquatuorpunctata_629 | 0.193 |
| Hyperaspis_reppensis_662 | Subcoccinella_vigintiquatuorpunctata_629 | 0.193 |
| Hyperaspis_reppensis_663 | Subcoccinella_vigintiquatuorpunctata_629 | 0.193 |
| Hyperaspis_lateralis_656 | Subcoccinella_vigintiquatuorpunctata_631 | 0.193 |
| Hyperaspis_lateralis_657 | Subcoccinella_vigintiquatuorpunctata_631 | 0.193 |
| Hyperaspis_reppensis_662 | Subcoccinella_vigintiquatuorpunctata_631 | 0.193 |
| Hyperaspis_reppensis_663 | Subcoccinella_vigintiquatuorpunctata_631 | 0.193 |
| Myzia_pullata_519 | Tytthaspis_sedecimpunctata_583 | 0.193 |
| Brachiacantha_ursina_637 | Tytthaspis_sedecimpunctata_585 | 0.193 |
| Cheilomenes_sexmaculata_224 | Tytthaspis_sedecimpunctata_587 | 0.193 |
| Coccinella_septempunctata_246 | Tytthaspis_sedecimpunctata_589 | 0.193 |
| Brachiacantha_ursina_637 | Tytthaspis_sedecimpunctata_593 | 0.193 |
| Scymnus_nebulosus_783 | Tytthaspis_sedecimpunctata_593 | 0.193 |
| Myrrha_octodecimguttata_489 | Scymnus_suturalis_833 | 0.193 |
| Myrrha_octodecimguttata_493 | Scymnus_suturalis_833 | 0.193 |
| Myrrha_octodecimguttata_495 | Scymnus_suturalis_833 | 0.193 |
| Myrrha_octodecimguttata_498 | Scymnus_suturalis_833 | 0.193 |
| Myrrha_octodecimguttata_500 | Scymnus_suturalis_833 | 0.193 |
| Nephus_redtenbacheri_674 | Scymnus_suturalis_833 | 0.193 |
| Nephus_redtenbacheri_675 | Scymnus_suturalis_833 | 0.193 |
| Nephus_redtenbacheri_676 | Scymnus_suturalis_838 | 0.193 |
| Nephus_redtenbacheri_677 | Scymnus_suturalis_838 | 0.193 |
| Nephus_redtenbacheri_680 | Scymnus_suturalis_838 | 0.193 |
| Nephus_redtenbacheri_681 | Scymnus_suturalis_838 | 0.193 |
| Nephus_redtenbacheri_676 | Scymnus_suturalis_840 | 0.193 |
| Nephus_redtenbacheri_677 | Scymnus_suturalis_840 | 0.193 |
| Nephus_redtenbacheri_680 | Scymnus_suturalis_840 | 0.193 |
| Nephus_redtenbacheri_681 | Scymnus_suturalis_840 | 0.193 |
| Coccinella_undecimpunctata_289 | Scymnus_sinuanodulus_829 | 0.193 |
| Coccinella_undecimpunctata_290 | Scymnus_sinuanodulus_829 | 0.193 |

Supplementary Table S4 (continued)

| | | |
|---|---|---|
| Nephus_redtenbacheri_674 | Scymnus_suturalis_841 | 0.194 |
| Nephus_redtenbacheri_675 | Scymnus_suturalis_841 | 0.194 |
| Nephus_redtenbacheri_674 | Scymnus_suturalis_842 | 0.194 |
| Nephus_redtenbacheri_675 | Scymnus_suturalis_842 | 0.194 |
| Nephus_redtenbacheri_676 | Scymnus_suturalis_843 | 0.194 |
| Nephus_redtenbacheri_677 | Scymnus_suturalis_843 | 0.194 |
| Nephus_redtenbacheri_680 | Scymnus_suturalis_843 | 0.194 |
| Nephus_redtenbacheri_681 | Scymnus_suturalis_843 | 0.194 |
| Nephus_redtenbacheri_674 | Scymnus_suturalis_844 | 0.194 |
| Nephus_redtenbacheri_675 | Scymnus_suturalis_844 | 0.194 |
| Nephus_redtenbacheri_674 | Scymnus_suturalis_846 | 0.194 |
| Nephus_redtenbacheri_675 | Scymnus_suturalis_846 | 0.194 |
| Nephus_redtenbacheri_674 | Scymnus_suturalis_847 | 0.194 |
| Nephus_redtenbacheri_675 | Scymnus_suturalis_847 | 0.194 |
| Nephus_redtenbacheri_674 | Scymnus_suturalis_848 | 0.194 |
| Nephus_redtenbacheri_675 | Scymnus_suturalis_848 | 0.194 |
| Hyperaspis_proba_661 | Stethorus_punctillum_852 | 0.194 |
| Scymnus_ningshanensis_789 | Subcoccinella_vigintiquatuorpunctata_620 | 0.194 |
| Scymnus_ningshanensis_790 | Subcoccinella_vigintiquatuorpunctata_620 | 0.194 |
| Scymnus_ningshanensis_789 | Subcoccinella_vigintiquatuorpunctata_621 | 0.194 |
| Scymnus_ningshanensis_790 | Subcoccinella_vigintiquatuorpunctata_621 | 0.194 |
| Hyperaspis_reppensis_662 | Subcoccinella_vigintiquatuorpunctata_623 | 0.194 |
| Hyperaspis_reppensis_663 | Subcoccinella_vigintiquatuorpunctata_623 | 0.194 |
| Scymnus_nebulosus_781 | Subcoccinella_vigintiquatuorpunctata_623 | 0.194 |
| Hyperaspis_lateralis_655 | Subcoccinella_vigintiquatuorpunctata_625 | 0.194 |
| Scymnus_ningshanensis_788 | Subcoccinella_vigintiquatuorpunctata_626 | 0.194 |
| Scymnus_ningshanensis_791 | Subcoccinella_vigintiquatuorpunctata_626 | 0.194 |
| Scymnus_ningshanensis_792 | Subcoccinella_vigintiquatuorpunctata_626 | 0.194 |
| Scymnus_ningshanensis_793 | Subcoccinella_vigintiquatuorpunctata_626 | 0.194 |
| Scymnus_ningshanensis_794 | Subcoccinella_vigintiquatuorpunctata_626 | 0.194 |
| Scymnus_ningshanensis_795 | Subcoccinella_vigintiquatuorpunctata_626 | 0.194 |
| Scymnus_ningshanensis_789 | Subcoccinella_vigintiquatuorpunctata_627 | 0.194 |
| Scymnus_ningshanensis_790 | Subcoccinella_vigintiquatuorpunctata_627 | 0.194 |
| Scymnus_ningshanensis_789 | Subcoccinella_vigintiquatuorpunctata_628 | 0.194 |
| Scymnus_ningshanensis_790 | Subcoccinella_vigintiquatuorpunctata_628 | 0.194 |
| Scymnus_ningshanensis_789 | Subcoccinella_vigintiquatuorpunctata_629 | 0.194 |
| Scymnus_ningshanensis_790 | Subcoccinella_vigintiquatuorpunctata_629 | 0.194 |
| Scymnus_ningshanensis_789 | Subcoccinella_vigintiquatuorpunctata_630 | 0.194 |
| Scymnus_ningshanensis_790 | Subcoccinella_vigintiquatuorpunctata_630 | 0.194 |
| Scymnus_ningshanensis_789 | Subcoccinella_vigintiquatuorpunctata_631 | 0.194 |
| Scymnus_ningshanensis_790 | Subcoccinella_vigintiquatuorpunctata_631 | 0.194 |
| Scymnus_nebulosus_781 | Tytthaspis_sedecimpunctata_580 | 0.194 |
| Scymnus_nigrinus_785 | Tytthaspis_sedecimpunctata_580 | 0.194 |
| Scymnus_nigrinus_786 | Tytthaspis_sedecimpunctata_580 | 0.194 |
| Coccinella_septempunctata_241 | Tytthaspis_sedecimpunctata_587 | 0.194 |
| Coccinella_septempunctata_243 | Tytthaspis_sedecimpunctata_587 | 0.194 |
| Coccinella_septempunctata_253 | Tytthaspis_sedecimpunctata_587 | 0.194 |
| Brachiacantha_ursina_637 | Tytthaspis_sedecimpunctata_588 | 0.194 |
| Scymnus_caurinus_720 | Tytthaspis_sedecimpunctata_591 | 0.194 |
| Scymnus_caurinus_721 | Tytthaspis_sedecimpunctata_591 | 0.194 |
| Scymnus_caurinus_722 | Tytthaspis_sedecimpunctata_591 | 0.194 |
| Nephus_redtenbacheri_674 | Scymnus_suturalis_832 | 0.194 |
| Nephus_redtenbacheri_675 | Scymnus_suturalis_832 | 0.194 |
| Nephus_redtenbacheri_674 | Scymnus_suturalis_835 | 0.194 |
| Nephus_redtenbacheri_675 | Scymnus_suturalis_835 | 0.194 |
| Nephus_redtenbacheri_674 | Scymnus_suturalis_836 | 0.194 |

Supplementary Table S4 (continued)

| | | |
|---|---|---|
| Nephus_redtenbacheri_675 | Scymnus_suturalis_836 | 0.194 |
| Nephus_redtenbacheri_674 | Scymnus_suturalis_837 | 0.194 |
| Nephus_redtenbacheri_675 | Scymnus_suturalis_837 | 0.194 |
| Nephus_redtenbacheri_674 | Scymnus_suturalis_839 | 0.194 |
| Nephus_redtenbacheri_675 | Scymnus_suturalis_839 | 0.194 |
| Myrrha_octodecimguttata_499 | Scymnus_sinuanodulus_831 | 0.194 |
| Rhyzobius_litura_96 | Scymnus_suturalis_843 | 0.194 |
| Rhyzobius_litura_96 | Scymnus_suturalis_845 | 0.194 |
| Hyperaspis_oregona_658 | Stethorus_punctillum_855 | 0.194 |
| Hyperaspis_oregona_659 | Stethorus_punctillum_855 | 0.194 |
| Hyperaspis_proba_661 | Stethorus_punctillum_859 | 0.194 |
| Scymnus_apetzi_691 | Subcoccinella_vigintiquatuorpunctata_620 | 0.194 |
| Scymnus_apetzi_691 | Subcoccinella_vigintiquatuorpunctata_621 | 0.194 |
| Scymnus_nebulosus_781 | Subcoccinella_vigintiquatuorpunctata_622 | 0.194 |
| Scymnus_nebulosus_781 | Subcoccinella_vigintiquatuorpunctata_626 | 0.194 |
| Scymnus_apetzi_690 | Subcoccinella_vigintiquatuorpunctata_627 | 0.194 |
| Scymnus_apetzi_697 | Subcoccinella_vigintiquatuorpunctata_627 | 0.194 |
| Scymnus_pallipediformis_811 | Subcoccinella_vigintiquatuorpunctata_627 | 0.194 |
| Scymnus_apetzi_691 | Subcoccinella_vigintiquatuorpunctata_628 | 0.194 |
| Scymnus_apetzi_691 | Subcoccinella_vigintiquatuorpunctata_629 | 0.194 |
| Scymnus_apetzi_696 | Subcoccinella_vigintiquatuorpunctata_630 | 0.194 |
| Scymnus_nebulosus_783 | Subcoccinella_vigintiquatuorpunctata_630 | 0.194 |
| Scymnus_apetzi_691 | Subcoccinella_vigintiquatuorpunctata_631 | 0.194 |
| Scymnus_nigrinus_784 | Tytthaspis_sedecimpunctata_577 | 0.194 |
| Scymnus_nigrinus_784 | Tytthaspis_sedecimpunctata_578 | 0.194 |
| Scymnus_nigrinus_784 | Tytthaspis_sedecimpunctata_579 | 0.194 |
| Brachiacantha_rotunda_634 | Tytthaspis_sedecimpunctata_580 | 0.194 |
| Scymnus_nigrinus_784 | Tytthaspis_sedecimpunctata_581 | 0.194 |
| Scymnus_nigrinus_784 | Tytthaspis_sedecimpunctata_582 | 0.194 |
| Scymnus_interruptus_761 | Tytthaspis_sedecimpunctata_584 | 0.194 |
| Scymnus_interruptus_763 | Tytthaspis_sedecimpunctata_584 | 0.194 |
| Scymnus_interruptus_764 | Tytthaspis_sedecimpunctata_584 | 0.194 |
| Scymnus_interruptus_766 | Tytthaspis_sedecimpunctata_584 | 0.194 |
| Scymnus_nigrinus_784 | Tytthaspis_sedecimpunctata_590 | 0.194 |
| Brachiacantha_rotunda_632 | Tytthaspis_sedecimpunctata_591 | 0.194 |
| Brachiacantha_rotunda_633 | Tytthaspis_sedecimpunctata_591 | 0.194 |
| Brachiacantha_rotunda_634 | Tytthaspis_sedecimpunctata_591 | 0.194 |
| Brachiacantha_rotunda_635 | Tytthaspis_sedecimpunctata_591 | 0.194 |
| Brachiacantha_rotunda_636 | Tytthaspis_sedecimpunctata_591 | 0.194 |
| Scymnus_interruptus_760 | Tytthaspis_sedecimpunctata_591 | 0.194 |
| Scymnus_interruptus_765 | Tytthaspis_sedecimpunctata_591 | 0.194 |
| Scymnus_interruptus_768 | Tytthaspis_sedecimpunctata_591 | 0.194 |
| Scymnus_interruptus_762 | Tytthaspis_sedecimpunctata_592 | 0.194 |
| Scymnus_nigrinus_784 | Tytthaspis_sedecimpunctata_593 | 0.194 |
| Scymnus_nigrinus_784 | Tytthaspis_sedecimpunctata_594 | 0.194 |
| Scymnus_nigrinus_784 | Tytthaspis_sedecimpunctata_595 | 0.194 |
| Calvia_quatuordecimguttata_202 | Scymnus_suturalis_833 | 0.194 |
| Calvia_quatuordecimguttata_210 | Scymnus_suturalis_833 | 0.194 |
| Calvia_quatuordecimguttata_211 | Scymnus_suturalis_838 | 0.194 |
| Calvia_quatuordecimguttata_212 | Scymnus_suturalis_838 | 0.194 |
| Calvia_quatuordecimguttata_213 | Scymnus_suturalis_838 | 0.194 |
| Calvia_quatuordecimguttata_214 | Scymnus_suturalis_838 | 0.194 |
| Calvia_quatuordecimguttata_215 | Scymnus_suturalis_838 | 0.194 |
| Calvia_quatuordecimguttata_211 | Scymnus_suturalis_840 | 0.194 |
| Calvia_quatuordecimguttata_212 | Scymnus_suturalis_840 | 0.194 |
| Calvia_quatuordecimguttata_213 | Scymnus_suturalis_840 | 0.194 |

| | | |
|---|---|---|
| Calvia_quatuordecimguttata_214 | Scymnus_suturalis_840 | 0.194 |
| Calvia_quatuordecimguttata_215 | Scymnus_suturalis_840 | 0.194 |
| Coccinella_undecimpunctata_289 | Vibidia_duodecimguttata_598 | 0.194 |
| Coccinella_undecimpunctata_290 | Vibidia_duodecimguttata_598 | 0.194 |
| Coelophora_inaequalis_318 | Vibidia_duodecimguttata_603 | 0.194 |
| Myrrha_octodecimguttata_495 | Scymnus_sinuanodulus_831 | 0.194 |
| Calvia_quatuordecimguttata_201 | Scymnus_suturalis_841 | 0.194 |
| Calvia_quatuordecimguttata_202 | Scymnus_suturalis_841 | 0.194 |
| Calvia_quatuordecimguttata_210 | Scymnus_suturalis_841 | 0.194 |
| Calvia_quatuordecimguttata_201 | Scymnus_suturalis_842 | 0.194 |
| Calvia_quatuordecimguttata_202 | Scymnus_suturalis_842 | 0.194 |
| Calvia_quatuordecimguttata_210 | Scymnus_suturalis_842 | 0.194 |
| Calvia_quatuordecimguttata_201 | Scymnus_suturalis_844 | 0.194 |
| Calvia_quatuordecimguttata_202 | Scymnus_suturalis_844 | 0.194 |
| Calvia_quatuordecimguttata_210 | Scymnus_suturalis_844 | 0.194 |
| Calvia_quatuordecimguttata_201 | Scymnus_suturalis_846 | 0.194 |
| Calvia_quatuordecimguttata_202 | Scymnus_suturalis_846 | 0.194 |
| Calvia_quatuordecimguttata_210 | Scymnus_suturalis_846 | 0.194 |
| Calvia_quatuordecimguttata_201 | Scymnus_suturalis_847 | 0.194 |
| Calvia_quatuordecimguttata_202 | Scymnus_suturalis_847 | 0.194 |
| Calvia_quatuordecimguttata_210 | Scymnus_suturalis_847 | 0.194 |
| Calvia_quatuordecimguttata_201 | Scymnus_suturalis_848 | 0.194 |
| Calvia_quatuordecimguttata_202 | Scymnus_suturalis_848 | 0.194 |
| Calvia_quatuordecimguttata_210 | Scymnus_suturalis_848 | 0.194 |
| Hyperaspis_reppensis_667 | Stethorus_punctillum_850 | 0.194 |
| Scymnus_interruptus_761 | Tytthaspis_sedecimpunctata_577 | 0.194 |
| Scymnus_interruptus_763 | Tytthaspis_sedecimpunctata_577 | 0.194 |
| Scymnus_interruptus_764 | Tytthaspis_sedecimpunctata_577 | 0.194 |
| Scymnus_interruptus_766 | Tytthaspis_sedecimpunctata_577 | 0.194 |
| Scymnus_interruptus_761 | Tytthaspis_sedecimpunctata_578 | 0.194 |
| Scymnus_interruptus_763 | Tytthaspis_sedecimpunctata_578 | 0.194 |
| Scymnus_interruptus_764 | Tytthaspis_sedecimpunctata_578 | 0.194 |
| Scymnus_interruptus_766 | Tytthaspis_sedecimpunctata_578 | 0.194 |
| Scymnus_interruptus_761 | Tytthaspis_sedecimpunctata_579 | 0.194 |
| Scymnus_interruptus_763 | Tytthaspis_sedecimpunctata_579 | 0.194 |
| Scymnus_interruptus_764 | Tytthaspis_sedecimpunctata_579 | 0.194 |
| Scymnus_interruptus_766 | Tytthaspis_sedecimpunctata_579 | 0.194 |
| Scymnus_interruptus_762 | Tytthaspis_sedecimpunctata_580 | 0.194 |
| Scymnus_interruptus_761 | Tytthaspis_sedecimpunctata_581 | 0.194 |
| Scymnus_interruptus_763 | Tytthaspis_sedecimpunctata_581 | 0.194 |
| Scymnus_interruptus_764 | Tytthaspis_sedecimpunctata_581 | 0.194 |
| Scymnus_interruptus_766 | Tytthaspis_sedecimpunctata_581 | 0.194 |
| Scymnus_interruptus_761 | Tytthaspis_sedecimpunctata_582 | 0.194 |
| Scymnus_interruptus_763 | Tytthaspis_sedecimpunctata_582 | 0.194 |
| Scymnus_interruptus_764 | Tytthaspis_sedecimpunctata_582 | 0.194 |
| Scymnus_interruptus_766 | Tytthaspis_sedecimpunctata_582 | 0.194 |
| Scymnus_nigrinus_784 | Tytthaspis_sedecimpunctata_584 | 0.194 |
| Scymnus_camptodromus_705 | Tytthaspis_sedecimpunctata_585 | 0.194 |
| Scymnus_camptodromus_713 | Tytthaspis_sedecimpunctata_585 | 0.194 |
| Scymnus_camptodromus_716 | Tytthaspis_sedecimpunctata_585 | 0.194 |
| Scymnus_interruptus_767 | Tytthaspis_sedecimpunctata_585 | 0.194 |
| Scymnus_ferrugatus_723 | Tytthaspis_sedecimpunctata_587 | 0.194 |
| Scymnus_ferrugatus_725 | Tytthaspis_sedecimpunctata_587 | 0.194 |
| Scymnus_ferrugatus_726 | Tytthaspis_sedecimpunctata_587 | 0.194 |
| Scymnus_ferrugatus_727 | Tytthaspis_sedecimpunctata_587 | 0.194 |
| Scymnus_ferrugatus_728 | Tytthaspis_sedecimpunctata_587 | 0.194 |

| | | |
|---|---|---|
| Scymnus_ferrugatus_730 | Tytthaspis_sedecimpunctata_587 | 0.194 |
| Scymnus_ferrugatus_731 | Tytthaspis_sedecimpunctata_587 | 0.194 |
| Scymnus_ferrugatus_732 | Tytthaspis_sedecimpunctata_587 | 0.194 |
| Scymnus_ferrugatus_733 | Tytthaspis_sedecimpunctata_587 | 0.194 |
| Scymnus_interruptus_762 | Tytthaspis_sedecimpunctata_588 | 0.194 |
| Brachiacantha_rotunda_634 | Tytthaspis_sedecimpunctata_589 | 0.194 |
| Scymnus_interruptus_761 | Tytthaspis_sedecimpunctata_590 | 0.194 |
| Scymnus_interruptus_763 | Tytthaspis_sedecimpunctata_590 | 0.194 |
| Scymnus_interruptus_764 | Tytthaspis_sedecimpunctata_590 | 0.194 |
| Scymnus_interruptus_766 | Tytthaspis_sedecimpunctata_590 | 0.194 |
| Scymnus_camptodromus_718 | Tytthaspis_sedecimpunctata_591 | 0.194 |
| Scymnus_interruptus_759 | Tytthaspis_sedecimpunctata_592 | 0.194 |
| Scymnus_interruptus_769 | Tytthaspis_sedecimpunctata_592 | 0.194 |
| Scymnus_interruptus_761 | Tytthaspis_sedecimpunctata_593 | 0.194 |
| Scymnus_interruptus_763 | Tytthaspis_sedecimpunctata_593 | 0.194 |
| Scymnus_interruptus_764 | Tytthaspis_sedecimpunctata_593 | 0.194 |
| Scymnus_interruptus_766 | Tytthaspis_sedecimpunctata_593 | 0.194 |
| Scymnus_interruptus_761 | Tytthaspis_sedecimpunctata_594 | 0.194 |
| Scymnus_interruptus_763 | Tytthaspis_sedecimpunctata_594 | 0.194 |
| Scymnus_interruptus_764 | Tytthaspis_sedecimpunctata_594 | 0.194 |
| Scymnus_interruptus_766 | Tytthaspis_sedecimpunctata_594 | 0.194 |
| Scymnus_interruptus_761 | Tytthaspis_sedecimpunctata_595 | 0.194 |
| Scymnus_interruptus_763 | Tytthaspis_sedecimpunctata_595 | 0.194 |
| Scymnus_interruptus_764 | Tytthaspis_sedecimpunctata_595 | 0.194 |
| Scymnus_interruptus_766 | Tytthaspis_sedecimpunctata_595 | 0.194 |
| Platynaspis_luteorubra_51 | Scymnus_rubromaculatus_822 | 0.194 |
| Platynaspis_luteorubra_53 | Scymnus_rubromaculatus_822 | 0.194 |
| Platynaspis_luteorubra_58 | Scymnus_rubromaculatus_822 | 0.194 |
| Platynaspis_luteorubra_51 | Scymnus_rubromaculatus_823 | 0.194 |
| Platynaspis_luteorubra_53 | Scymnus_rubromaculatus_823 | 0.194 |
| Platynaspis_luteorubra_58 | Scymnus_rubromaculatus_823 | 0.194 |
| Platynaspis_luteorubra_51 | Scymnus_rubromaculatus_824 | 0.194 |
| Platynaspis_luteorubra_53 | Scymnus_rubromaculatus_824 | 0.194 |
| Platynaspis_luteorubra_58 | Scymnus_rubromaculatus_824 | 0.194 |
| Platynaspis_luteorubra_51 | Scymnus_rubromaculatus_825 | 0.194 |
| Platynaspis_luteorubra_53 | Scymnus_rubromaculatus_825 | 0.194 |
| Platynaspis_luteorubra_58 | Scymnus_rubromaculatus_825 | 0.194 |
| Platynaspis_luteorubra_51 | Scymnus_rubromaculatus_826 | 0.194 |
| Platynaspis_luteorubra_53 | Scymnus_rubromaculatus_826 | 0.194 |
| Platynaspis_luteorubra_58 | Scymnus_rubromaculatus_826 | 0.194 |
| Platynaspis_luteorubra_51 | Scymnus_rubromaculatus_827 | 0.194 |
| Platynaspis_luteorubra_53 | Scymnus_rubromaculatus_827 | 0.194 |
| Platynaspis_luteorubra_58 | Scymnus_rubromaculatus_827 | 0.194 |
| Chilocorus_stigma_47 | Scymnus_sinuanodulus_830 | 0.194 |
| Calvia_quatuordecimguttata_201 | Scymnus_suturalis_832 | 0.194 |
| Calvia_quatuordecimguttata_202 | Scymnus_suturalis_832 | 0.194 |
| Calvia_quatuordecimguttata_210 | Scymnus_suturalis_832 | 0.194 |
| Myzia_pullata_519 | Scymnus_suturalis_833 | 0.194 |
| Calvia_quatuordecimguttata_211 | Scymnus_suturalis_834 | 0.194 |
| Calvia_quatuordecimguttata_212 | Scymnus_suturalis_834 | 0.194 |
| Calvia_quatuordecimguttata_213 | Scymnus_suturalis_834 | 0.194 |
| Calvia_quatuordecimguttata_214 | Scymnus_suturalis_834 | 0.194 |
| Calvia_quatuordecimguttata_215 | Scymnus_suturalis_834 | 0.194 |
| Calvia_quatuordecimguttata_201 | Scymnus_suturalis_835 | 0.194 |
| Calvia_quatuordecimguttata_202 | Scymnus_suturalis_835 | 0.194 |
| Calvia_quatuordecimguttata_210 | Scymnus_suturalis_835 | 0.194 |

| | | |
|---|---|---|
| Calvia_quatuordecimguttata_201 | Scymnus_suturalis_836 | 0.194 |
| Calvia_quatuordecimguttata_202 | Scymnus_suturalis_836 | 0.194 |
| Calvia_quatuordecimguttata_210 | Scymnus_suturalis_836 | 0.194 |
| Calvia_quatuordecimguttata_211 | Scymnus_suturalis_837 | 0.194 |
| Calvia_quatuordecimguttata_212 | Scymnus_suturalis_837 | 0.194 |
| Calvia_quatuordecimguttata_213 | Scymnus_suturalis_837 | 0.194 |
| Calvia_quatuordecimguttata_214 | Scymnus_suturalis_837 | 0.194 |
| Calvia_quatuordecimguttata_215 | Scymnus_suturalis_837 | 0.194 |
| Calvia_quatuordecimguttata_201 | Scymnus_suturalis_839 | 0.194 |
| Calvia_quatuordecimguttata_202 | Scymnus_suturalis_839 | 0.194 |
| Calvia_quatuordecimguttata_210 | Scymnus_suturalis_839 | 0.194 |
| Coccidula_scutellata_84 | Scymnus_rubromaculatus_822 | 0.194 |
| Coccidula_scutellata_84 | Scymnus_rubromaculatus_823 | 0.194 |
| Coccidula_scutellata_84 | Scymnus_rubromaculatus_824 | 0.194 |
| Coccidula_scutellata_84 | Scymnus_rubromaculatus_825 | 0.194 |
| Coccidula_scutellata_84 | Scymnus_rubromaculatus_826 | 0.194 |
| Coccidula_scutellata_84 | Scymnus_rubromaculatus_827 | 0.194 |
| Clitostethus_arcuatus_642 | Scymnus_suturalis_841 | 0.194 |
| Harmonia_axyridis_355 | Scymnus_suturalis_841 | 0.194 |
| Clitostethus_arcuatus_642 | Scymnus_suturalis_842 | 0.194 |
| Harmonia_axyridis_355 | Scymnus_suturalis_842 | 0.194 |
| Calvia_quatuordecimguttata_218 | Scymnus_suturalis_843 | 0.194 |
| Clitostethus_arcuatus_639 | Scymnus_suturalis_843 | 0.194 |
| Clitostethus_arcuatus_640 | Scymnus_suturalis_843 | 0.194 |
| Clitostethus_arcuatus_641 | Scymnus_suturalis_843 | 0.194 |
| Clitostethus_arcuatus_643 | Scymnus_suturalis_843 | 0.194 |
| Clitostethus_arcuatus_642 | Scymnus_suturalis_844 | 0.194 |
| Harmonia_axyridis_355 | Scymnus_suturalis_844 | 0.194 |
| Calvia_quatuordecimguttata_218 | Scymnus_suturalis_845 | 0.194 |
| Clitostethus_arcuatus_642 | Scymnus_suturalis_846 | 0.194 |
| Harmonia_axyridis_355 | Scymnus_suturalis_846 | 0.194 |
| Clitostethus_arcuatus_642 | Scymnus_suturalis_847 | 0.194 |
| Harmonia_axyridis_355 | Scymnus_suturalis_847 | 0.194 |
| Clitostethus_arcuatus_642 | Scymnus_suturalis_848 | 0.194 |
| Harmonia_axyridis_355 | Scymnus_suturalis_848 | 0.194 |
| Hyperaspis_lateralis_657 | Stethorus_punctillum_849 | 0.194 |
| Hyperaspis_lateralis_657 | Stethorus_punctillum_850 | 0.194 |
| Hyperaspis_lateralis_657 | Stethorus_punctillum_854 | 0.194 |
| Hyperaspis_lateralis_657 | Stethorus_punctillum_856 | 0.194 |
| Hyperaspis_lateralis_654 | Stethorus_punctillum_857 | 0.194 |
| Hyperaspis_lateralis_654 | Stethorus_punctillum_860 | 0.194 |
| Psyllobora_vigintiduopunctata_561 | Subcoccinella_vigintiquatuorpunctata_624 | 0.194 |
| Psyllobora_vigintiduopunctata_562 | Subcoccinella_vigintiquatuorpunctata_624 | 0.194 |
| Psyllobora_vigintiduopunctata_565 | Subcoccinella_vigintiquatuorpunctata_624 | 0.194 |
| Psyllobora_vigintiduopunctata_567 | Subcoccinella_vigintiquatuorpunctata_624 | 0.194 |
| Hyperaspis_binotata_649 | Subcoccinella_vigintiquatuorpunctata_625 | 0.194 |
| Psyllobora_vigintiduopunctata_561 | Subcoccinella_vigintiquatuorpunctata_630 | 0.194 |
| Psyllobora_vigintiduopunctata_562 | Subcoccinella_vigintiquatuorpunctata_630 | 0.194 |
| Psyllobora_vigintiduopunctata_565 | Subcoccinella_vigintiquatuorpunctata_630 | 0.194 |
| Psyllobora_vigintiduopunctata_567 | Subcoccinella_vigintiquatuorpunctata_630 | 0.194 |
| Scymnus_interruptus_759 | Tytthaspis_sedecimpunctata_580 | 0.194 |
| Scymnus_interruptus_769 | Tytthaspis_sedecimpunctata_580 | 0.194 |
| Olla_vnigrum_530 | Tytthaspis_sedecimpunctata_586 | 0.194 |
| Brachiacantha_rotunda_634 | Tytthaspis_sedecimpunctata_587 | 0.194 |
| Scymnus_interruptus_761 | Tytthaspis_sedecimpunctata_588 | 0.194 |
| Scymnus_interruptus_763 | Tytthaspis_sedecimpunctata_588 | 0.194 |

Supplementary Table S4 (continued)

| | | |
|---|---|---|
| Scymnus_interruptus_764 | Tytthaspis_sedecimpunctata_588 | 0.194 |
| Scymnus_interruptus_766 | Tytthaspis_sedecimpunctata_588 | 0.194 |
| Olla_vnigrum_526 | Tytthaspis_sedecimpunctata_589 | 0.194 |
| Scymnus_camptodromus_719 | Tytthaspis_sedecimpunctata_591 | 0.194 |
| Clitostethus_arcuatus_642 | Scymnus_suturalis_832 | 0.194 |
| Harmonia_axyridis_355 | Scymnus_suturalis_832 | 0.194 |
| Harmonia_axyridis_354 | Scymnus_suturalis_833 | 0.194 |
| Harmonia_axyridis_356 | Scymnus_suturalis_833 | 0.194 |
| Harmonia_axyridis_357 | Scymnus_suturalis_833 | 0.194 |
| Harmonia_axyridis_358 | Scymnus_suturalis_833 | 0.194 |
| Harmonia_axyridis_359 | Scymnus_suturalis_833 | 0.194 |
| Harmonia_axyridis_360 | Scymnus_suturalis_833 | 0.194 |
| Harmonia_axyridis_361 | Scymnus_suturalis_833 | 0.194 |
| Harmonia_axyridis_362 | Scymnus_suturalis_833 | 0.194 |
| Harmonia_axyridis_363 | Scymnus_suturalis_833 | 0.194 |
| Harmonia_axyridis_364 | Scymnus_suturalis_833 | 0.194 |
| Scymnus_frontalis_736 | Scymnus_suturalis_833 | 0.194 |
| Scymnus_frontalis_737 | Scymnus_suturalis_833 | 0.194 |
| Scymnus_frontalis_738 | Scymnus_suturalis_833 | 0.194 |
| Scymnus_frontalis_739 | Scymnus_suturalis_833 | 0.194 |
| Scymnus_frontalis_740 | Scymnus_suturalis_833 | 0.194 |
| Scymnus_frontalis_741 | Scymnus_suturalis_833 | 0.194 |
| Scymnus_frontalis_743 | Scymnus_suturalis_833 | 0.194 |
| Scymnus_frontalis_744 | Scymnus_suturalis_833 | 0.194 |
| Calvia_quatuordecimguttata_218 | Scymnus_suturalis_834 | 0.194 |
| Clitostethus_arcuatus_639 | Scymnus_suturalis_834 | 0.194 |
| Clitostethus_arcuatus_640 | Scymnus_suturalis_834 | 0.194 |
| Clitostethus_arcuatus_641 | Scymnus_suturalis_834 | 0.194 |
| Clitostethus_arcuatus_643 | Scymnus_suturalis_834 | 0.194 |
| Clitostethus_arcuatus_642 | Scymnus_suturalis_835 | 0.194 |
| Harmonia_axyridis_355 | Scymnus_suturalis_835 | 0.194 |
| Clitostethus_arcuatus_642 | Scymnus_suturalis_836 | 0.194 |
| Harmonia_axyridis_355 | Scymnus_suturalis_836 | 0.194 |
| Harmonia_axyridis_354 | Scymnus_suturalis_838 | 0.194 |
| Harmonia_axyridis_356 | Scymnus_suturalis_838 | 0.194 |
| Harmonia_axyridis_357 | Scymnus_suturalis_838 | 0.194 |
| Harmonia_axyridis_358 | Scymnus_suturalis_838 | 0.194 |
| Harmonia_axyridis_359 | Scymnus_suturalis_838 | 0.194 |
| Harmonia_axyridis_360 | Scymnus_suturalis_838 | 0.194 |
| Harmonia_axyridis_361 | Scymnus_suturalis_838 | 0.194 |
| Harmonia_axyridis_362 | Scymnus_suturalis_838 | 0.194 |
| Harmonia_axyridis_363 | Scymnus_suturalis_838 | 0.194 |
| Harmonia_axyridis_364 | Scymnus_suturalis_838 | 0.194 |
| Clitostethus_arcuatus_642 | Scymnus_suturalis_839 | 0.194 |
| Harmonia_axyridis_355 | Scymnus_suturalis_839 | 0.194 |
| Harmonia_axyridis_354 | Scymnus_suturalis_840 | 0.194 |
| Harmonia_axyridis_356 | Scymnus_suturalis_840 | 0.194 |
| Harmonia_axyridis_357 | Scymnus_suturalis_840 | 0.194 |
| Harmonia_axyridis_358 | Scymnus_suturalis_840 | 0.194 |
| Harmonia_axyridis_359 | Scymnus_suturalis_840 | 0.194 |
| Harmonia_axyridis_360 | Scymnus_suturalis_840 | 0.194 |
| Harmonia_axyridis_361 | Scymnus_suturalis_840 | 0.194 |
| Harmonia_axyridis_362 | Scymnus_suturalis_840 | 0.194 |
| Harmonia_axyridis_363 | Scymnus_suturalis_840 | 0.194 |
| Harmonia_axyridis_364 | Scymnus_suturalis_840 | 0.194 |
| Coccinella_quinquepunctata_234 | Tytthaspis_sedecimpunctata_589 | 0.194 |

| | | |
|---|---|---|
| Calvia_quatuordecimguttata_204 | Scymnus_suturalis_843 | 0.194 |
| Calvia_quatuordecimguttata_208 | Scymnus_suturalis_843 | 0.194 |
| Calvia_quatuordecimguttata_217 | Scymnus_suturalis_843 | 0.194 |
| Calvia_quatuordecimguttata_204 | Scymnus_suturalis_845 | 0.194 |
| Calvia_quatuordecimguttata_208 | Scymnus_suturalis_845 | 0.194 |
| Calvia_quatuordecimguttata_217 | Scymnus_suturalis_845 | 0.194 |
| Scymnus_nubilus_796 | Subcoccinella_vigintiquatuorpunctata_623 | 0.194 |
| Scymnus_nubilus_803 | Subcoccinella_vigintiquatuorpunctata_623 | 0.194 |
| Scymnus_nubilus_804 | Subcoccinella_vigintiquatuorpunctata_623 | 0.194 |
| Scymnus_nubilus_809 | Subcoccinella_vigintiquatuorpunctata_623 | 0.194 |
| Scymnus_ferrugatus_724 | Tytthaspis_sedecimpunctata_577 | 0.194 |
| Scymnus_ferrugatus_724 | Tytthaspis_sedecimpunctata_578 | 0.194 |
| Scymnus_ferrugatus_724 | Tytthaspis_sedecimpunctata_579 | 0.194 |
| Scymnus_ferrugatus_723 | Tytthaspis_sedecimpunctata_580 | 0.194 |
| Scymnus_ferrugatus_725 | Tytthaspis_sedecimpunctata_580 | 0.194 |
| Scymnus_ferrugatus_726 | Tytthaspis_sedecimpunctata_580 | 0.194 |
| Scymnus_ferrugatus_727 | Tytthaspis_sedecimpunctata_580 | 0.194 |
| Scymnus_ferrugatus_728 | Tytthaspis_sedecimpunctata_580 | 0.194 |
| Scymnus_ferrugatus_730 | Tytthaspis_sedecimpunctata_580 | 0.194 |
| Scymnus_ferrugatus_731 | Tytthaspis_sedecimpunctata_580 | 0.194 |
| Scymnus_ferrugatus_732 | Tytthaspis_sedecimpunctata_580 | 0.194 |
| Scymnus_ferrugatus_733 | Tytthaspis_sedecimpunctata_580 | 0.194 |
| Scymnus_ferrugatus_724 | Tytthaspis_sedecimpunctata_581 | 0.194 |
| Scymnus_ferrugatus_724 | Tytthaspis_sedecimpunctata_582 | 0.194 |
| Scymnus_ferrugatus_724 | Tytthaspis_sedecimpunctata_585 | 0.194 |
| Scymnus_ferrugatus_729 | Tytthaspis_sedecimpunctata_585 | 0.194 |
| Olla_vnigrum_525 | Tytthaspis_sedecimpunctata_587 | 0.194 |
| Olla_vnigrum_529 | Tytthaspis_sedecimpunctata_587 | 0.194 |
| Olla_vnigrum_531 | Tytthaspis_sedecimpunctata_587 | 0.194 |
| Olla_vnigrum_526 | Tytthaspis_sedecimpunctata_588 | 0.194 |
| Scymnus_ferrugatus_724 | Tytthaspis_sedecimpunctata_590 | 0.194 |
| Scymnus_ferrugatus_724 | Tytthaspis_sedecimpunctata_592 | 0.194 |
| Scymnus_ferrugatus_729 | Tytthaspis_sedecimpunctata_592 | 0.194 |
| Scymnus_ferrugatus_724 | Tytthaspis_sedecimpunctata_594 | 0.194 |
| Scymnus_ferrugatus_724 | Tytthaspis_sedecimpunctata_595 | 0.194 |
| Hippodamia_parenthesis_433 | Scymnus_suturalis_833 | 0.194 |
| Hippodamia_parenthesis_440 | Scymnus_suturalis_833 | 0.194 |
| Calvia_quatuordecimguttata_204 | Scymnus_suturalis_834 | 0.194 |
| Calvia_quatuordecimguttata_208 | Scymnus_suturalis_834 | 0.194 |
| Calvia_quatuordecimguttata_217 | Scymnus_suturalis_834 | 0.194 |
| Harmonia_axyridis_354 | Scymnus_suturalis_837 | 0.194 |
| Harmonia_axyridis_356 | Scymnus_suturalis_837 | 0.194 |
| Harmonia_axyridis_357 | Scymnus_suturalis_837 | 0.194 |
| Harmonia_axyridis_358 | Scymnus_suturalis_837 | 0.194 |
| Harmonia_axyridis_359 | Scymnus_suturalis_837 | 0.194 |
| Harmonia_axyridis_360 | Scymnus_suturalis_837 | 0.194 |
| Harmonia_axyridis_361 | Scymnus_suturalis_837 | 0.194 |
| Harmonia_axyridis_362 | Scymnus_suturalis_837 | 0.194 |
| Harmonia_axyridis_363 | Scymnus_suturalis_837 | 0.194 |
| Harmonia_axyridis_364 | Scymnus_suturalis_837 | 0.194 |
| Clitostethus_arcuatus_642 | Scymnus_suturalis_838 | 0.194 |
| Hippodamia_parenthesis_433 | Scymnus_suturalis_838 | 0.194 |
| Hippodamia_parenthesis_440 | Scymnus_suturalis_838 | 0.194 |
| Hippodamia_parenthesis_433 | Scymnus_suturalis_840 | 0.194 |
| Hippodamia_parenthesis_440 | Scymnus_suturalis_840 | 0.194 |
| Hyperaspis_proba_660 | Stethorus_punctillum_851 | 0.194 |

Supplementary Table S4 (continued)

| | | |
|---|---|---|
| Hyperaspis_proba_660 | Stethorus_punctillum_858 | 0.194 |
| Scymnus_nebulosus_781 | Subcoccinella_vigintiquatuorpunctata_619 | 0.194 |
| Scymnus_apetzi_692 | Subcoccinella_vigintiquatuorpunctata_625 | 0.194 |
| Coccinella_quinquepunctata_234 | Tytthaspis_sedecimpunctata_587 | 0.194 |
| Hippodamia_parenthesis_433 | Scymnus_suturalis_845 | 0.194 |
| Hippodamia_parenthesis_440 | Scymnus_suturalis_845 | 0.194 |
| Hyperaspis_lateralis_657 | Stethorus_punctillum_853 | 0.194 |
| Scymnus_nubilus_798 | Subcoccinella_vigintiquatuorpunctata_620 | 0.194 |
| Scymnus_nubilus_808 | Subcoccinella_vigintiquatuorpunctata_620 | 0.194 |
| Scymnus_nubilus_798 | Subcoccinella_vigintiquatuorpunctata_621 | 0.194 |
| Scymnus_nubilus_808 | Subcoccinella_vigintiquatuorpunctata_621 | 0.194 |
| Hyperaspis_binotata_650 | Subcoccinella_vigintiquatuorpunctata_625 | 0.194 |
| Scymnus_nubilus_796 | Subcoccinella_vigintiquatuorpunctata_626 | 0.194 |
| Scymnus_nubilus_803 | Subcoccinella_vigintiquatuorpunctata_626 | 0.194 |
| Scymnus_nubilus_804 | Subcoccinella_vigintiquatuorpunctata_626 | 0.194 |
| Scymnus_nubilus_809 | Subcoccinella_vigintiquatuorpunctata_626 | 0.194 |
| Scymnus_nubilus_798 | Subcoccinella_vigintiquatuorpunctata_628 | 0.194 |
| Scymnus_nubilus_808 | Subcoccinella_vigintiquatuorpunctata_628 | 0.194 |
| Scymnus_nubilus_798 | Subcoccinella_vigintiquatuorpunctata_629 | 0.194 |
| Scymnus_nubilus_808 | Subcoccinella_vigintiquatuorpunctata_629 | 0.194 |
| Scymnus_nubilus_798 | Subcoccinella_vigintiquatuorpunctata_631 | 0.194 |
| Scymnus_nubilus_808 | Subcoccinella_vigintiquatuorpunctata_631 | 0.194 |
| Scymnus_ferrugatus_729 | Tytthaspis_sedecimpunctata_580 | 0.194 |
| Hyperaspis_lateralis_657 | Scymnus_sinuanodulus_831 | 0.194 |
| Scymnus_camptodromus_715 | Subcoccinella_vigintiquatuorpunctata_624 | 0.194 |
| Calvia_quatuordecimguttata_203 | Scymnus_suturalis_843 | 0.194 |
| Calvia_quatuordecimguttata_203 | Scymnus_suturalis_845 | 0.194 |
| Scymnus_nubilus_797 | Subcoccinella_vigintiquatuorpunctata_620 | 0.194 |
| Scymnus_nubilus_797 | Subcoccinella_vigintiquatuorpunctata_621 | 0.194 |
| Coccidula_scutellata_78 | Subcoccinella_vigintiquatuorpunctata_623 | 0.194 |
| Coccidula_scutellata_79 | Subcoccinella_vigintiquatuorpunctata_623 | 0.194 |
| Coccidula_scutellata_77 | Subcoccinella_vigintiquatuorpunctata_625 | 0.194 |
| Scymnus_nubilus_797 | Subcoccinella_vigintiquatuorpunctata_628 | 0.194 |
| Scymnus_nubilus_797 | Subcoccinella_vigintiquatuorpunctata_629 | 0.194 |
| Scymnus_interruptus_767 | Subcoccinella_vigintiquatuorpunctata_630 | 0.194 |
| Scymnus_nubilus_797 | Subcoccinella_vigintiquatuorpunctata_631 | 0.194 |
| Scymnus_abietis_682 | Tytthaspis_sedecimpunctata_583 | 0.194 |
| Scymnus_abietis_683 | Tytthaspis_sedecimpunctata_583 | 0.194 |
| Scymnus_abietis_685 | Tytthaspis_sedecimpunctata_583 | 0.194 |
| Hippodamia_parenthesis_432 | Vibidia_duodecimguttata_597 | 0.194 |
| Hippodamia_parenthesis_434 | Vibidia_duodecimguttata_597 | 0.194 |
| Hippodamia_parenthesis_435 | Vibidia_duodecimguttata_597 | 0.194 |
| Hippodamia_parenthesis_437 | Vibidia_duodecimguttata_597 | 0.194 |
| Hippodamia_parenthesis_438 | Vibidia_duodecimguttata_597 | 0.194 |
| Hippodamia_parenthesis_440 | Vibidia_duodecimguttata_597 | 0.194 |
| Hippodamia_parenthesis_441 | Vibidia_duodecimguttata_597 | 0.194 |
| Hippodamia_parenthesis_442 | Vibidia_duodecimguttata_597 | 0.194 |
| Hippodamia_parenthesis_432 | Vibidia_duodecimguttata_600 | 0.194 |
| Hippodamia_parenthesis_434 | Vibidia_duodecimguttata_600 | 0.194 |
| Hippodamia_parenthesis_435 | Vibidia_duodecimguttata_600 | 0.194 |
| Hippodamia_parenthesis_437 | Vibidia_duodecimguttata_600 | 0.194 |
| Hippodamia_parenthesis_438 | Vibidia_duodecimguttata_600 | 0.194 |
| Hippodamia_parenthesis_440 | Vibidia_duodecimguttata_600 | 0.194 |
| Hippodamia_parenthesis_441 | Vibidia_duodecimguttata_600 | 0.194 |
| Hippodamia_parenthesis_442 | Vibidia_duodecimguttata_600 | 0.194 |
| Calvia_quatuordecimguttata_202 | Scymnus_rubromaculatus_822 | 0.194 |

| | | |
|---|---|---|
| Calvia_quatuordecimguttata_210 | Scymnus_rubromaculatus_822 | 0.194 |
| Calvia_quatuordecimguttata_202 | Scymnus_rubromaculatus_823 | 0.194 |
| Calvia_quatuordecimguttata_210 | Scymnus_rubromaculatus_823 | 0.194 |
| Calvia_quatuordecimguttata_202 | Scymnus_rubromaculatus_824 | 0.194 |
| Calvia_quatuordecimguttata_210 | Scymnus_rubromaculatus_824 | 0.194 |
| Calvia_quatuordecimguttata_202 | Scymnus_rubromaculatus_825 | 0.194 |
| Calvia_quatuordecimguttata_210 | Scymnus_rubromaculatus_825 | 0.194 |
| Calvia_quatuordecimguttata_202 | Scymnus_rubromaculatus_826 | 0.194 |
| Calvia_quatuordecimguttata_210 | Scymnus_rubromaculatus_826 | 0.194 |
| Calvia_quatuordecimguttata_202 | Scymnus_rubromaculatus_827 | 0.194 |
| Calvia_quatuordecimguttata_210 | Scymnus_rubromaculatus_827 | 0.194 |
| Calvia_quatuordecimguttata_203 | Scymnus_suturalis_834 | 0.194 |
| Brumus_quadripustulatus_10 | Scymnus_sinuanodulus_828 | 0.194 |
| Coccidula_scutellata_81 | Subcoccinella_vigintiquatuorpunctata_625 | 0.194 |
| Coccidula_scutellata_82 | Subcoccinella_vigintiquatuorpunctata_625 | 0.194 |
| Coccidula_scutellata_78 | Subcoccinella_vigintiquatuorpunctata_626 | 0.194 |
| Coccidula_scutellata_79 | Subcoccinella_vigintiquatuorpunctata_626 | 0.194 |
| Coccidula_scutellata_83 | Subcoccinella_vigintiquatuorpunctata_627 | 0.194 |
| Coccidula_scutellata_83 | Subcoccinella_vigintiquatuorpunctata_630 | 0.194 |
| Scymnus_abietis_682 | Tytthaspis_sedecimpunctata_586 | 0.194 |
| Scymnus_abietis_683 | Tytthaspis_sedecimpunctata_586 | 0.194 |
| Scymnus_abietis_685 | Tytthaspis_sedecimpunctata_586 | 0.194 |
| Scymnus_abietis_682 | Tytthaspis_sedecimpunctata_587 | 0.194 |
| Scymnus_abietis_683 | Tytthaspis_sedecimpunctata_587 | 0.194 |
| Scymnus_abietis_682 | Tytthaspis_sedecimpunctata_588 | 0.194 |
| Scymnus_abietis_683 | Tytthaspis_sedecimpunctata_588 | 0.194 |
| Scymnus_abietis_685 | Tytthaspis_sedecimpunctata_588 | 0.194 |
| Scymnus_abietis_682 | Tytthaspis_sedecimpunctata_591 | 0.194 |
| Scymnus_abietis_683 | Tytthaspis_sedecimpunctata_591 | 0.194 |
| Scymnus_abietis_685 | Tytthaspis_sedecimpunctata_591 | 0.194 |
| Hippodamia_parenthesis_433 | Vibidia_duodecimguttata_596 | 0.194 |
| Calvia_quatuordecimguttata_218 | Scymnus_sinuanodulus_828 | 0.194 |
| Scymnus_nubilus_799 | Scymnus_sinuanodulus_830 | 0.194 |
| Hippodamia_convergens_400 | Scymnus_suturalis_833 | 0.194 |
| Myrrha_octodecimguttata_499 | Scymnus_sinuanodulus_829 | 0.194 |
| Hippodamia_convergens_395 | Scymnus_suturalis_841 | 0.194 |
| Hippodamia_convergens_396 | Scymnus_suturalis_841 | 0.194 |
| Hippodamia_convergens_403 | Scymnus_suturalis_841 | 0.194 |
| Hippodamia_moesta_410 | Scymnus_suturalis_841 | 0.194 |
| Hippodamia_convergens_395 | Scymnus_suturalis_842 | 0.194 |
| Hippodamia_convergens_396 | Scymnus_suturalis_842 | 0.194 |
| Hippodamia_convergens_403 | Scymnus_suturalis_842 | 0.194 |
| Hippodamia_moesta_410 | Scymnus_suturalis_842 | 0.194 |
| Hippodamia_convergens_393 | Scymnus_suturalis_843 | 0.194 |
| Hippodamia_convergens_394 | Scymnus_suturalis_843 | 0.194 |
| Hippodamia_convergens_397 | Scymnus_suturalis_843 | 0.194 |
| Hippodamia_convergens_398 | Scymnus_suturalis_843 | 0.194 |
| Hippodamia_convergens_401 | Scymnus_suturalis_843 | 0.194 |
| Hippodamia_convergens_402 | Scymnus_suturalis_843 | 0.194 |
| Hippodamia_convergens_395 | Scymnus_suturalis_844 | 0.194 |
| Hippodamia_convergens_396 | Scymnus_suturalis_844 | 0.194 |
| Hippodamia_convergens_403 | Scymnus_suturalis_844 | 0.194 |
| Hippodamia_moesta_410 | Scymnus_suturalis_844 | 0.194 |
| Hippodamia_convergens_393 | Scymnus_suturalis_845 | 0.194 |
| Hippodamia_convergens_394 | Scymnus_suturalis_845 | 0.194 |
| Hippodamia_convergens_397 | Scymnus_suturalis_845 | 0.194 |

| | | |
|---|---|---|
| Hippodamia_convergens_398 | Scymnus_suturalis_845 | 0.194 |
| Hippodamia_convergens_401 | Scymnus_suturalis_845 | 0.194 |
| Hippodamia_convergens_402 | Scymnus_suturalis_845 | 0.194 |
| Hippodamia_convergens_395 | Scymnus_suturalis_846 | 0.194 |
| Hippodamia_convergens_396 | Scymnus_suturalis_846 | 0.194 |
| Hippodamia_convergens_403 | Scymnus_suturalis_846 | 0.194 |
| Hippodamia_moesta_410 | Scymnus_suturalis_846 | 0.194 |
| Hippodamia_convergens_395 | Scymnus_suturalis_847 | 0.194 |
| Hippodamia_convergens_396 | Scymnus_suturalis_847 | 0.194 |
| Hippodamia_convergens_403 | Scymnus_suturalis_847 | 0.194 |
| Hippodamia_moesta_410 | Scymnus_suturalis_847 | 0.194 |
| Hippodamia_convergens_395 | Scymnus_suturalis_848 | 0.194 |
| Hippodamia_convergens_396 | Scymnus_suturalis_848 | 0.194 |
| Hippodamia_convergens_403 | Scymnus_suturalis_848 | 0.194 |
| Hippodamia_moesta_410 | Scymnus_suturalis_848 | 0.194 |
| Scymnus_mitior_779 | Subcoccinella_vigintiquatuorpunctata_620 | 0.194 |
| Scymnus_mitior_780 | Subcoccinella_vigintiquatuorpunctata_620 | 0.194 |
| Scymnus_mitior_779 | Subcoccinella_vigintiquatuorpunctata_621 | 0.194 |
| Scymnus_mitior_780 | Subcoccinella_vigintiquatuorpunctata_621 | 0.194 |
| Scymnus_haemorrhoidalis_755 | Subcoccinella_vigintiquatuorpunctata_625 | 0.194 |
| Scymnus_haemorrhoidalis_756 | Subcoccinella_vigintiquatuorpunctata_625 | 0.194 |
| Scymnus_mitior_779 | Subcoccinella_vigintiquatuorpunctata_627 | 0.194 |
| Scymnus_mitior_780 | Subcoccinella_vigintiquatuorpunctata_627 | 0.194 |
| Scymnus_mitior_779 | Subcoccinella_vigintiquatuorpunctata_628 | 0.194 |
| Scymnus_mitior_780 | Subcoccinella_vigintiquatuorpunctata_628 | 0.194 |
| Scymnus_mitior_779 | Subcoccinella_vigintiquatuorpunctata_629 | 0.194 |
| Scymnus_mitior_780 | Subcoccinella_vigintiquatuorpunctata_629 | 0.194 |
| Scymnus_mitior_779 | Subcoccinella_vigintiquatuorpunctata_631 | 0.194 |
| Scymnus_mitior_780 | Subcoccinella_vigintiquatuorpunctata_631 | 0.194 |
| Hippodamia_parenthesis_439 | Vibidia_duodecimguttata_598 | 0.194 |
| Hippodamia_parenthesis_439 | Vibidia_duodecimguttata_599 | 0.194 |
| Hippodamia_parenthesis_439 | Vibidia_duodecimguttata_601 | 0.194 |
| Hippodamia_parenthesis_439 | Vibidia_duodecimguttata_602 | 0.194 |
| Calvia_quatuordecimguttata_204 | Scymnus_sinuanodulus_828 | 0.194 |
| Calvia_quatuordecimguttata_208 | Scymnus_sinuanodulus_828 | 0.194 |
| Calvia_quatuordecimguttata_217 | Scymnus_sinuanodulus_828 | 0.194 |
| Scymnus_nubilus_799 | Scymnus_sinuanodulus_828 | 0.194 |
| Hippodamia_convergens_395 | Scymnus_suturalis_832 | 0.194 |
| Hippodamia_convergens_396 | Scymnus_suturalis_832 | 0.194 |
| Hippodamia_convergens_403 | Scymnus_suturalis_832 | 0.194 |
| Hippodamia_moesta_410 | Scymnus_suturalis_832 | 0.194 |
| Scymnus_nubilus_803 | Scymnus_suturalis_833 | 0.194 |
| Scymnus_nubilus_804 | Scymnus_suturalis_833 | 0.194 |
| Scymnus_nubilus_808 | Scymnus_suturalis_833 | 0.194 |
| Scymnus_nubilus_809 | Scymnus_suturalis_833 | 0.194 |
| Hippodamia_convergens_395 | Scymnus_suturalis_835 | 0.194 |
| Hippodamia_convergens_396 | Scymnus_suturalis_835 | 0.194 |
| Hippodamia_convergens_403 | Scymnus_suturalis_835 | 0.194 |
| Hippodamia_moesta_410 | Scymnus_suturalis_835 | 0.194 |
| Hippodamia_convergens_395 | Scymnus_suturalis_836 | 0.194 |
| Hippodamia_convergens_396 | Scymnus_suturalis_836 | 0.194 |
| Hippodamia_convergens_403 | Scymnus_suturalis_836 | 0.194 |
| Hippodamia_moesta_410 | Scymnus_suturalis_836 | 0.194 |
| Hippodamia_convergens_395 | Scymnus_suturalis_839 | 0.194 |
| Hippodamia_convergens_396 | Scymnus_suturalis_839 | 0.194 |
| Hippodamia_convergens_403 | Scymnus_suturalis_839 | 0.194 |

Supplementary Table S4 (continued)

| | | |
|---|---|---|
| Hippodamia_moesta_410 | Scymnus_suturalis_839 | 0.194 |
| Scymnus_nubilus_796 | Subcoccinella_vigintiquatuorpunctata_619 | 0.194 |
| Scymnus_nubilus_803 | Subcoccinella_vigintiquatuorpunctata_619 | 0.194 |
| Scymnus_nubilus_804 | Subcoccinella_vigintiquatuorpunctata_619 | 0.194 |
| Scymnus_nubilus_809 | Subcoccinella_vigintiquatuorpunctata_619 | 0.194 |
| Hyperaspis_campestris_651 | Scymnus_sinuanodulus_831 | 0.194 |
| Hyperaspis_campestris_652 | Scymnus_sinuanodulus_831 | 0.194 |
| Hyperaspis_campestris_653 | Scymnus_sinuanodulus_831 | 0.194 |
| Coccinella_undecimpunctata_287 | Vibidia_duodecimguttata_597 | 0.194 |
| Coccinella_undecimpunctata_287 | Vibidia_duodecimguttata_600 | 0.194 |
| Myrrha_octodecimguttata_495 | Scymnus_sinuanodulus_829 | 0.194 |
| Scymnus_mitior_779 | Subcoccinella_vigintiquatuorpunctata_623 | 0.194 |
| Scymnus_mitior_780 | Subcoccinella_vigintiquatuorpunctata_623 | 0.194 |
| Scymnus_haemorrhoidalis_751 | Subcoccinella_vigintiquatuorpunctata_625 | 0.194 |
| Scymnus_haemorrhoidalis_754 | Subcoccinella_vigintiquatuorpunctata_625 | 0.194 |
| Scymnus_haemorrhoidalis_757 | Subcoccinella_vigintiquatuorpunctata_625 | 0.194 |
| Scymnus_haemorrhoidalis_758 | Subcoccinella_vigintiquatuorpunctata_625 | 0.194 |
| Aphidecta_obliterata_178 | Vibidia_duodecimguttata_596 | 0.194 |
| Aphidecta_obliterata_178 | Vibidia_duodecimguttata_598 | 0.194 |
| Aphidecta_obliterata_178 | Vibidia_duodecimguttata_603 | 0.194 |
| Calvia_quatuordecimguttata_205 | Scymnus_sinuanodulus_828 | 0.194 |
| Calvia_quatuordecimguttata_203 | Scymnus_sinuanodulus_830 | 0.194 |
| Hippodamia_parenthesis_436 | Vibidia_duodecimguttata_597 | 0.194 |
| Hippodamia_parenthesis_436 | Vibidia_duodecimguttata_600 | 0.194 |
| Nephus_quadrimaculatus_668 | Scymnus_sinuanodulus_831 | 0.194 |
| Scymnus_frontalis_746 | Scymnus_suturalis_833 | 0.194 |
| Coelophora_inaequalis_319 | Vibidia_duodecimguttata_599 | 0.194 |
| Coelophora_inaequalis_319 | Vibidia_duodecimguttata_601 | 0.194 |
| Coelophora_inaequalis_319 | Vibidia_duodecimguttata_602 | 0.194 |
| Scymnus_abietis_689 | Tytthaspis_sedecimpunctata_583 | 0.194 |
| Coccinella_undecimpunctata_291 | Scymnus_sinuanodulus_831 | 0.194 |
| Scymnus_haemorrhoidalis_752 | Subcoccinella_vigintiquatuorpunctata_625 | 0.195 |
| Scymnus_haemorrhoidalis_753 | Subcoccinella_vigintiquatuorpunctata_625 | 0.195 |
| Harmonia_quadripunctata_376 | Vibidia_duodecimguttata_596 | 0.195 |
| Harmonia_quadripunctata_378 | Vibidia_duodecimguttata_596 | 0.195 |
| Adalia_bipunctata_105 | Vibidia_duodecimguttata_597 | 0.195 |
| Adalia_bipunctata_99 | Vibidia_duodecimguttata_597 | 0.195 |
| Harmonia_quadripunctata_376 | Vibidia_duodecimguttata_598 | 0.195 |
| Harmonia_quadripunctata_378 | Vibidia_duodecimguttata_598 | 0.195 |
| Coccinella_magnifica_228 | Vibidia_duodecimguttata_599 | 0.195 |
| Coccinella_magnifica_229 | Vibidia_duodecimguttata_599 | 0.195 |
| Coccinella_magnifica_230 | Vibidia_duodecimguttata_599 | 0.195 |
| Coccinella_magnifica_231 | Vibidia_duodecimguttata_599 | 0.195 |
| Harmonia_quadripunctata_382 | Vibidia_duodecimguttata_599 | 0.195 |
| Adalia_bipunctata_105 | Vibidia_duodecimguttata_600 | 0.195 |
| Adalia_bipunctata_99 | Vibidia_duodecimguttata_600 | 0.195 |
| Coccinella_magnifica_228 | Vibidia_duodecimguttata_601 | 0.195 |
| Coccinella_magnifica_229 | Vibidia_duodecimguttata_601 | 0.195 |
| Coccinella_magnifica_230 | Vibidia_duodecimguttata_601 | 0.195 |
| Coccinella_magnifica_231 | Vibidia_duodecimguttata_601 | 0.195 |
| Harmonia_quadripunctata_382 | Vibidia_duodecimguttata_601 | 0.195 |
| Coccinella_magnifica_228 | Vibidia_duodecimguttata_602 | 0.195 |
| Coccinella_magnifica_229 | Vibidia_duodecimguttata_602 | 0.195 |
| Coccinella_magnifica_230 | Vibidia_duodecimguttata_602 | 0.195 |
| Coccinella_magnifica_231 | Vibidia_duodecimguttata_602 | 0.195 |
| Harmonia_quadripunctata_382 | Vibidia_duodecimguttata_602 | 0.195 |

| | | |
|---|---|---|
| Harmonia_quadripunctata_377 | Vibidia_duodecimguttata_603 | 0.195 |
| Harmonia_quadripunctata_379 | Vibidia_duodecimguttata_603 | 0.195 |
| Harmonia_quadripunctata_381 | Vibidia_duodecimguttata_603 | 0.195 |
| Oenopia_conglobata_522 | Scymnus_suturalis_833 | 0.195 |
| Psyllobora_vigintiduopunctata_568 | Subcoccinella_vigintiquatuorpunctata_624 | 0.195 |
| Psyllobora_vigintiduopunctata_568 | Subcoccinella_vigintiquatuorpunctata_630 | 0.195 |
| Coccidula_scutellata_84 | Subcoccinella_vigintiquatuorpunctata_620 | 0.195 |
| Coccidula_scutellata_84 | Subcoccinella_vigintiquatuorpunctata_621 | 0.195 |
| Coccidula_scutellata_84 | Subcoccinella_vigintiquatuorpunctata_622 | 0.195 |
| Coccidula_scutellata_84 | Subcoccinella_vigintiquatuorpunctata_628 | 0.195 |
| Coccidula_scutellata_84 | Subcoccinella_vigintiquatuorpunctata_629 | 0.195 |
| Coccidula_scutellata_84 | Subcoccinella_vigintiquatuorpunctata_631 | 0.195 |
| Scymnus_abietis_689 | Tytthaspis_sedecimpunctata_580 | 0.195 |
| Scymnus_abietis_689 | Tytthaspis_sedecimpunctata_586 | 0.195 |
| Scymnus_abietis_689 | Tytthaspis_sedecimpunctata_587 | 0.195 |
| Scymnus_abietis_689 | Tytthaspis_sedecimpunctata_588 | 0.195 |
| Scymnus_abietis_689 | Tytthaspis_sedecimpunctata_591 | 0.195 |
| Oenopia_conglobata_522 | Scymnus_suturalis_841 | 0.195 |
| Oenopia_conglobata_522 | Scymnus_suturalis_842 | 0.195 |
| Oenopia_conglobata_522 | Scymnus_suturalis_844 | 0.195 |
| Oenopia_conglobata_522 | Scymnus_suturalis_846 | 0.195 |
| Oenopia_conglobata_522 | Scymnus_suturalis_847 | 0.195 |
| Oenopia_conglobata_522 | Scymnus_suturalis_848 | 0.195 |
| Harmonia_quadripunctata_382 | Vibidia_duodecimguttata_597 | 0.195 |
| Harmonia_quadripunctata_382 | Vibidia_duodecimguttata_600 | 0.195 |
| Oenopia_conglobata_522 | Scymnus_suturalis_832 | 0.195 |
| Hippodamia_glacialis_407 | Scymnus_suturalis_833 | 0.195 |
| Oenopia_conglobata_522 | Scymnus_suturalis_835 | 0.195 |
| Oenopia_conglobata_522 | Scymnus_suturalis_836 | 0.195 |
| Oenopia_conglobata_522 | Scymnus_suturalis_837 | 0.195 |
| Hippodamia_glacialis_407 | Scymnus_suturalis_838 | 0.195 |
| Oenopia_conglobata_522 | Scymnus_suturalis_839 | 0.195 |
| Hippodamia_glacialis_407 | Scymnus_suturalis_840 | 0.195 |
| Brachiacantha_rotunda_632 | Scymnus_sinuanodulus_829 | 0.195 |
| Brachiacantha_rotunda_633 | Scymnus_sinuanodulus_829 | 0.195 |
| Brachiacantha_rotunda_635 | Scymnus_sinuanodulus_829 | 0.195 |
| Hyperaspis_lateralis_657 | Scymnus_sinuanodulus_829 | 0.195 |
| Scymnus_camptodromus_711 | Subcoccinella_vigintiquatuorpunctata_620 | 0.195 |
| Scymnus_camptodromus_711 | Subcoccinella_vigintiquatuorpunctata_621 | 0.195 |
| Scymnus_camptodromus_711 | Subcoccinella_vigintiquatuorpunctata_627 | 0.195 |
| Scymnus_camptodromus_711 | Subcoccinella_vigintiquatuorpunctata_628 | 0.195 |
| Scymnus_camptodromus_711 | Subcoccinella_vigintiquatuorpunctata_629 | 0.195 |
| Scymnus_camptodromus_711 | Subcoccinella_vigintiquatuorpunctata_630 | 0.195 |
| Scymnus_camptodromus_711 | Subcoccinella_vigintiquatuorpunctata_631 | 0.195 |
| Brachiacantha_rotunda_632 | Subcoccinella_vigintiquatuorpunctata_622 | 0.195 |
| Brachiacantha_rotunda_633 | Subcoccinella_vigintiquatuorpunctata_622 | 0.195 |
| Brachiacantha_rotunda_635 | Subcoccinella_vigintiquatuorpunctata_622 | 0.195 |
| Brachiacantha_rotunda_632 | Subcoccinella_vigintiquatuorpunctata_624 | 0.195 |
| Brachiacantha_rotunda_633 | Subcoccinella_vigintiquatuorpunctata_624 | 0.195 |
| Brachiacantha_rotunda_635 | Subcoccinella_vigintiquatuorpunctata_624 | 0.195 |
| Brachiacantha_rotunda_632 | Subcoccinella_vigintiquatuorpunctata_626 | 0.195 |
| Brachiacantha_rotunda_633 | Subcoccinella_vigintiquatuorpunctata_626 | 0.195 |
| Brachiacantha_rotunda_635 | Subcoccinella_vigintiquatuorpunctata_626 | 0.195 |
| Brachiacantha_rotunda_636 | Subcoccinella_vigintiquatuorpunctata_627 | 0.195 |
| Brachiacantha_rotunda_634 | Subcoccinella_vigintiquatuorpunctata_630 | 0.195 |
| Neoharmonia_venusta_520 | Vibidia_duodecimguttata_597 | 0.195 |

| | | |
|---|---|---|
| Neoharmonia_venusta_520 | Vibidia_duodecimguttata_600 | 0.195 |
| Hippodamia_glacialis_407 | Scymnus_suturalis_834 | 0.195 |
| Hippodamia_glacialis_407 | Scymnus_suturalis_837 | 0.195 |
| Coccinella_undecimpunctata_289 | Scymnus_sinuanodulus_831 | 0.195 |
| Coccinella_undecimpunctata_290 | Scymnus_sinuanodulus_831 | 0.195 |
| Hyperaspis_campestris_651 | Scymnus_sinuanodulus_829 | 0.195 |
| Hyperaspis_campestris_652 | Scymnus_sinuanodulus_829 | 0.195 |
| Hyperaspis_campestris_653 | Scymnus_sinuanodulus_829 | 0.195 |
| Coccinella_trifasciata_275 | Vibidia_duodecimguttata_596 | 0.195 |
| Coccinella_trifasciata_277 | Vibidia_duodecimguttata_596 | 0.195 |
| Coccinella_trifasciata_280 | Vibidia_duodecimguttata_596 | 0.195 |
| Coccinella_trifasciata_281 | Vibidia_duodecimguttata_596 | 0.195 |
| Coccinella_trifasciata_275 | Vibidia_duodecimguttata_598 | 0.195 |
| Coccinella_trifasciata_277 | Vibidia_duodecimguttata_598 | 0.195 |
| Coccinella_trifasciata_280 | Vibidia_duodecimguttata_598 | 0.195 |
| Coccinella_trifasciata_281 | Vibidia_duodecimguttata_598 | 0.195 |
| Coccinella_trifasciata_273 | Vibidia_duodecimguttata_599 | 0.195 |
| Coccinella_trifasciata_273 | Vibidia_duodecimguttata_601 | 0.195 |
| Coccinella_trifasciata_273 | Vibidia_duodecimguttata_602 | 0.195 |
| Coccinella_trifasciata_272 | Vibidia_duodecimguttata_603 | 0.195 |
| Coccinella_trifasciata_276 | Vibidia_duodecimguttata_603 | 0.195 |
| Coccinella_trifasciata_278 | Vibidia_duodecimguttata_603 | 0.195 |
| Coccinella_trifasciata_279 | Vibidia_duodecimguttata_603 | 0.195 |
| Neoharmonia_venusta_520 | Vibidia_duodecimguttata_603 | 0.195 |
| Scymnus_camptodromus_717 | Subcoccinella_vigintiquatuorpunctata_624 | 0.195 |
| Scymnus_apetzi_695 | Subcoccinella_vigintiquatuorpunctata_625 | 0.195 |
| Oenopia_conglobata_524 | Scymnus_suturalis_843 | 0.195 |
| Coccidophilus_marginatus_869 | Tytthaspis_sedecimpunctata_585 | 0.195 |
| Chilocorus_bipustulatus_23 | Scymnus_suturalis_843 | 0.195 |
| Chilocorus_bipustulatus_23 | Scymnus_suturalis_845 | 0.195 |
| Hyperaspis_reppensis_662 | Tytthaspis_sedecimpunctata_589 | 0.195 |
| Hyperaspis_reppensis_663 | Tytthaspis_sedecimpunctata_589 | 0.195 |
| Hyperaspis_lateralis_655 | Tytthaspis_sedecimpunctata_577 | 0.195 |
| Hyperaspis_lateralis_655 | Tytthaspis_sedecimpunctata_578 | 0.195 |
| Hyperaspis_lateralis_655 | Tytthaspis_sedecimpunctata_579 | 0.195 |
| Hyperaspis_lateralis_654 | Tytthaspis_sedecimpunctata_580 | 0.195 |
| Hyperaspis_lateralis_657 | Tytthaspis_sedecimpunctata_580 | 0.195 |
| Hyperaspis_lateralis_655 | Tytthaspis_sedecimpunctata_581 | 0.195 |
| Hyperaspis_lateralis_655 | Tytthaspis_sedecimpunctata_582 | 0.195 |
| Hyperaspis_lateralis_655 | Tytthaspis_sedecimpunctata_590 | 0.195 |
| Hyperaspis_lateralis_655 | Tytthaspis_sedecimpunctata_592 | 0.195 |
| Hyperaspis_lateralis_655 | Tytthaspis_sedecimpunctata_593 | 0.195 |
| Hyperaspis_lateralis_655 | Tytthaspis_sedecimpunctata_594 | 0.195 |
| Hyperaspis_lateralis_655 | Tytthaspis_sedecimpunctata_595 | 0.195 |
| Coccinella_trifasciata_274 | Vibidia_duodecimguttata_596 | 0.195 |
| Coccinella_trifasciata_274 | Vibidia_duodecimguttata_598 | 0.195 |
| Chilocorus_bipustulatus_18 | Scymnus_suturalis_841 | 0.195 |
| Chilocorus_bipustulatus_20 | Scymnus_suturalis_841 | 0.195 |
| Chilocorus_bipustulatus_24 | Scymnus_suturalis_841 | 0.195 |
| Chilocorus_bipustulatus_18 | Scymnus_suturalis_842 | 0.195 |
| Chilocorus_bipustulatus_20 | Scymnus_suturalis_842 | 0.195 |
| Chilocorus_bipustulatus_24 | Scymnus_suturalis_842 | 0.195 |
| Chilocorus_bipustulatus_18 | Scymnus_suturalis_844 | 0.195 |
| Chilocorus_bipustulatus_20 | Scymnus_suturalis_844 | 0.195 |
| Chilocorus_bipustulatus_24 | Scymnus_suturalis_844 | 0.195 |
| Chilocorus_bipustulatus_18 | Scymnus_suturalis_846 | 0.195 |

| | | |
|---|---|---|
| Chilocorus_bipustulatus_20 | Scymnus_suturalis_846 | 0.195 |
| Chilocorus_bipustulatus_24 | Scymnus_suturalis_846 | 0.195 |
| Chilocorus_bipustulatus_18 | Scymnus_suturalis_847 | 0.195 |
| Chilocorus_bipustulatus_20 | Scymnus_suturalis_847 | 0.195 |
| Chilocorus_bipustulatus_24 | Scymnus_suturalis_847 | 0.195 |
| Chilocorus_bipustulatus_18 | Scymnus_suturalis_848 | 0.195 |
| Chilocorus_bipustulatus_20 | Scymnus_suturalis_848 | 0.195 |
| Chilocorus_bipustulatus_24 | Scymnus_suturalis_848 | 0.195 |
| Chilocorus_bipustulatus_18 | Scymnus_suturalis_832 | 0.195 |
| Chilocorus_bipustulatus_20 | Scymnus_suturalis_832 | 0.195 |
| Chilocorus_bipustulatus_24 | Scymnus_suturalis_832 | 0.195 |
| Chilocorus_bipustulatus_23 | Scymnus_suturalis_833 | 0.195 |
| Chilocorus_bipustulatus_18 | Scymnus_suturalis_835 | 0.195 |
| Chilocorus_bipustulatus_20 | Scymnus_suturalis_835 | 0.195 |
| Chilocorus_bipustulatus_24 | Scymnus_suturalis_835 | 0.195 |
| Chilocorus_bipustulatus_18 | Scymnus_suturalis_836 | 0.195 |
| Chilocorus_bipustulatus_20 | Scymnus_suturalis_836 | 0.195 |
| Chilocorus_bipustulatus_24 | Scymnus_suturalis_836 | 0.195 |
| Chilocorus_bipustulatus_18 | Scymnus_suturalis_837 | 0.195 |
| Chilocorus_bipustulatus_20 | Scymnus_suturalis_837 | 0.195 |
| Chilocorus_bipustulatus_24 | Scymnus_suturalis_837 | 0.195 |
| Chilocorus_bipustulatus_18 | Scymnus_suturalis_839 | 0.195 |
| Chilocorus_bipustulatus_20 | Scymnus_suturalis_839 | 0.195 |
| Chilocorus_bipustulatus_24 | Scymnus_suturalis_839 | 0.195 |
| Hyperaspis_lateralis_656 | Tytthaspis_sedecimpunctata_593 | 0.195 |
| Hyperaspis_lateralis_656 | Tytthaspis_sedecimpunctata_588 | 0.195 |
| Platynaspis_luteorubra_52 | Tytthaspis_sedecimpunctata_591 | 0.195 |
| Platynaspis_luteorubra_56 | Tytthaspis_sedecimpunctata_591 | 0.195 |
| Platynaspis_luteorubra_60 | Tytthaspis_sedecimpunctata_591 | 0.195 |
| Brachiacantha_rotunda_636 | Subcoccinella_vigintiquatuorpunctata_619 | 0.195 |
| Oenopia_conglobata_524 | Scymnus_suturalis_840 | 0.195 |
| Platynaspis_luteorubra_54 | Tytthaspis_sedecimpunctata_591 | 0.195 |
| Platynaspis_luteorubra_57 | Tytthaspis_sedecimpunctata_591 | 0.195 |
| Platynaspis_luteorubra_59 | Tytthaspis_sedecimpunctata_591 | 0.195 |
| Nephus_quadrimaculatus_669 | Scymnus_sinuanodulus_829 | 0.195 |
| Nephus_quadrimaculatus_670 | Scymnus_sinuanodulus_829 | 0.195 |
| Nephus_quadrimaculatus_671 | Scymnus_sinuanodulus_829 | 0.195 |
| Nephus_quadrimaculatus_672 | Scymnus_sinuanodulus_829 | 0.195 |
| Nephus_quadrimaculatus_673 | Scymnus_sinuanodulus_829 | 0.195 |
| Platynaspis_luteorubra_51 | Stethorus_punctillum_855 | 0.195 |
| Platynaspis_luteorubra_53 | Stethorus_punctillum_855 | 0.195 |
| Platynaspis_luteorubra_58 | Stethorus_punctillum_855 | 0.195 |
| Scymnus_mimulus_778 | Tytthaspis_sedecimpunctata_588 | 0.195 |
| Chilocorus_renipustulatus_33 | Tytthaspis_sedecimpunctata_591 | 0.195 |
| Coelophora_inaequalis_315 | Vibidia_duodecimguttata_599 | 0.195 |
| Coelophora_inaequalis_315 | Vibidia_duodecimguttata_601 | 0.195 |
| Coelophora_inaequalis_315 | Vibidia_duodecimguttata_602 | 0.195 |
| Chilocorus_renipustulatus_34 | Scymnus_suturalis_843 | 0.195 |
| Hyperaspis_reppensis_662 | Stethorus_punctillum_857 | 0.195 |
| Hyperaspis_reppensis_663 | Stethorus_punctillum_857 | 0.195 |
| Hyperaspis_reppensis_662 | Stethorus_punctillum_860 | 0.195 |
| Hyperaspis_reppensis_663 | Stethorus_punctillum_860 | 0.195 |
| Chilocorus_renipustulatus_39 | Tytthaspis_sedecimpunctata_587 | 0.195 |
| Hyperaspis_reppensis_665 | Tytthaspis_sedecimpunctata_591 | 0.195 |
| Myzia_oblongoguttata_516 | Scymnus_suturalis_833 | 0.195 |
| Chilocorus_renipustulatus_34 | Scymnus_suturalis_834 | 0.195 |

Supplementary Table S4 (continued)

| | | |
|---|---|---|
| Myzia_oblongoguttata_506 | Scymnus_suturalis_838 | 0.195 |
| Myzia_oblongoguttata_506 | Scymnus_suturalis_840 | 0.195 |
| Chilocorus_renipustulatus_39 | Scymnus_suturalis_841 | 0.195 |
| Myzia_oblongoguttata_516 | Scymnus_suturalis_841 | 0.195 |
| Chilocorus_renipustulatus_39 | Scymnus_suturalis_842 | 0.195 |
| Myzia_oblongoguttata_516 | Scymnus_suturalis_842 | 0.195 |
| Chilocorus_renipustulatus_31 | Scymnus_suturalis_843 | 0.195 |
| Chilocorus_renipustulatus_32 | Scymnus_suturalis_843 | 0.195 |
| Chilocorus_renipustulatus_35 | Scymnus_suturalis_843 | 0.195 |
| Chilocorus_renipustulatus_36 | Scymnus_suturalis_843 | 0.195 |
| Chilocorus_renipustulatus_37 | Scymnus_suturalis_843 | 0.195 |
| Chilocorus_renipustulatus_38 | Scymnus_suturalis_843 | 0.195 |
| Chilocorus_renipustulatus_40 | Scymnus_suturalis_843 | 0.195 |
| Chilocorus_renipustulatus_41 | Scymnus_suturalis_843 | 0.195 |
| Chilocorus_renipustulatus_42 | Scymnus_suturalis_843 | 0.195 |
| Myzia_oblongoguttata_504 | Scymnus_suturalis_843 | 0.195 |
| Myzia_oblongoguttata_505 | Scymnus_suturalis_843 | 0.195 |
| Myzia_oblongoguttata_507 | Scymnus_suturalis_843 | 0.195 |
| Myzia_oblongoguttata_508 | Scymnus_suturalis_843 | 0.195 |
| Myzia_oblongoguttata_509 | Scymnus_suturalis_843 | 0.195 |
| Myzia_oblongoguttata_510 | Scymnus_suturalis_843 | 0.195 |
| Myzia_oblongoguttata_511 | Scymnus_suturalis_843 | 0.195 |
| Myzia_oblongoguttata_512 | Scymnus_suturalis_843 | 0.195 |
| Myzia_oblongoguttata_513 | Scymnus_suturalis_843 | 0.195 |
| Myzia_oblongoguttata_514 | Scymnus_suturalis_843 | 0.195 |
| Myzia_oblongoguttata_515 | Scymnus_suturalis_843 | 0.195 |
| Chilocorus_renipustulatus_39 | Scymnus_suturalis_844 | 0.195 |
| Myzia_oblongoguttata_516 | Scymnus_suturalis_844 | 0.195 |
| Chilocorus_renipustulatus_31 | Scymnus_suturalis_845 | 0.195 |
| Chilocorus_renipustulatus_32 | Scymnus_suturalis_845 | 0.195 |
| Chilocorus_renipustulatus_35 | Scymnus_suturalis_845 | 0.195 |
| Chilocorus_renipustulatus_36 | Scymnus_suturalis_845 | 0.195 |
| Chilocorus_renipustulatus_37 | Scymnus_suturalis_845 | 0.195 |
| Chilocorus_renipustulatus_38 | Scymnus_suturalis_845 | 0.195 |
| Chilocorus_renipustulatus_40 | Scymnus_suturalis_845 | 0.195 |
| Chilocorus_renipustulatus_41 | Scymnus_suturalis_845 | 0.195 |
| Chilocorus_renipustulatus_42 | Scymnus_suturalis_845 | 0.195 |
| Myzia_oblongoguttata_504 | Scymnus_suturalis_845 | 0.195 |
| Myzia_oblongoguttata_505 | Scymnus_suturalis_845 | 0.195 |
| Myzia_oblongoguttata_507 | Scymnus_suturalis_845 | 0.195 |
| Myzia_oblongoguttata_508 | Scymnus_suturalis_845 | 0.195 |
| Myzia_oblongoguttata_509 | Scymnus_suturalis_845 | 0.195 |
| Myzia_oblongoguttata_510 | Scymnus_suturalis_845 | 0.195 |
| Myzia_oblongoguttata_511 | Scymnus_suturalis_845 | 0.195 |
| Myzia_oblongoguttata_512 | Scymnus_suturalis_845 | 0.195 |
| Myzia_oblongoguttata_513 | Scymnus_suturalis_845 | 0.195 |
| Myzia_oblongoguttata_514 | Scymnus_suturalis_845 | 0.195 |
| Myzia_oblongoguttata_515 | Scymnus_suturalis_845 | 0.195 |
| Chilocorus_renipustulatus_39 | Scymnus_suturalis_846 | 0.195 |
| Myzia_oblongoguttata_516 | Scymnus_suturalis_846 | 0.195 |
| Chilocorus_renipustulatus_39 | Scymnus_suturalis_847 | 0.195 |
| Myzia_oblongoguttata_516 | Scymnus_suturalis_847 | 0.195 |
| Chilocorus_renipustulatus_39 | Scymnus_suturalis_848 | 0.195 |
| Myzia_oblongoguttata_516 | Scymnus_suturalis_848 | 0.195 |
| Hyperaspis_reppensis_662 | Stethorus_punctillum_859 | 0.195 |
| Hyperaspis_reppensis_663 | Stethorus_punctillum_859 | 0.195 |

Supplementary Table S4 (continued)

| | | |
|---|---|---|
| Scymnus_loewii_771 | Subcoccinella_vigintiquatuorpunctata_620 | 0.195 |
| Scymnus_loewii_771 | Subcoccinella_vigintiquatuorpunctata_621 | 0.195 |
| Scymnus_loewii_771 | Subcoccinella_vigintiquatuorpunctata_624 | 0.195 |
| Scymnus_loewii_770 | Subcoccinella_vigintiquatuorpunctata_627 | 0.195 |
| Scymnus_loewii_771 | Subcoccinella_vigintiquatuorpunctata_628 | 0.195 |
| Scymnus_loewii_771 | Subcoccinella_vigintiquatuorpunctata_629 | 0.195 |
| Scymnus_loewii_771 | Subcoccinella_vigintiquatuorpunctata_631 | 0.195 |
| Chilocorus_renipustulatus_39 | Scymnus_suturalis_832 | 0.195 |
| Myzia_oblongoguttata_516 | Scymnus_suturalis_832 | 0.195 |
| Chilocorus_renipustulatus_31 | Scymnus_suturalis_834 | 0.195 |
| Chilocorus_renipustulatus_32 | Scymnus_suturalis_834 | 0.195 |
| Chilocorus_renipustulatus_35 | Scymnus_suturalis_834 | 0.195 |
| Chilocorus_renipustulatus_36 | Scymnus_suturalis_834 | 0.195 |
| Chilocorus_renipustulatus_37 | Scymnus_suturalis_834 | 0.195 |
| Chilocorus_renipustulatus_38 | Scymnus_suturalis_834 | 0.195 |
| Chilocorus_renipustulatus_40 | Scymnus_suturalis_834 | 0.195 |
| Chilocorus_renipustulatus_41 | Scymnus_suturalis_834 | 0.195 |
| Chilocorus_renipustulatus_42 | Scymnus_suturalis_834 | 0.195 |
| Myzia_oblongoguttata_506 | Scymnus_suturalis_834 | 0.195 |
| Chilocorus_renipustulatus_39 | Scymnus_suturalis_835 | 0.195 |
| Myzia_oblongoguttata_516 | Scymnus_suturalis_835 | 0.195 |
| Chilocorus_renipustulatus_39 | Scymnus_suturalis_836 | 0.195 |
| Myzia_oblongoguttata_516 | Scymnus_suturalis_836 | 0.195 |
| Myzia_oblongoguttata_516 | Scymnus_suturalis_837 | 0.195 |
| Chilocorus_renipustulatus_39 | Scymnus_suturalis_839 | 0.195 |
| Myzia_oblongoguttata_516 | Scymnus_suturalis_839 | 0.195 |
| Coccinella_transversalis_257 | Tytthaspis_sedecimpunctata_586 | 0.195 |
| Coccinella_transversalis_259 | Tytthaspis_sedecimpunctata_586 | 0.195 |
| Coccinella_transversalis_260 | Tytthaspis_sedecimpunctata_586 | 0.195 |
| Coccinella_transversalis_261 | Tytthaspis_sedecimpunctata_586 | 0.195 |
| Coccinella_transversalis_262 | Tytthaspis_sedecimpunctata_586 | 0.195 |
| Coccinella_transversalis_263 | Tytthaspis_sedecimpunctata_586 | 0.195 |
| Coccinella_transversalis_264 | Tytthaspis_sedecimpunctata_586 | 0.195 |
| Rhyzobius_chrysomeloides_85 | Tytthaspis_sedecimpunctata_586 | 0.195 |
| Rhyzobius_chrysomeloides_86 | Tytthaspis_sedecimpunctata_586 | 0.195 |
| Rhyzobius_chrysomeloides_87 | Tytthaspis_sedecimpunctata_586 | 0.195 |
| Rhyzobius_chrysomeloides_88 | Tytthaspis_sedecimpunctata_586 | 0.195 |
| Rhyzobius_chrysomeloides_89 | Tytthaspis_sedecimpunctata_586 | 0.195 |
| Rhyzobius_chrysomeloides_90 | Tytthaspis_sedecimpunctata_586 | 0.195 |
| Rhyzobius_chrysomeloides_91 | Tytthaspis_sedecimpunctata_586 | 0.195 |
| Rhyzobius_chrysomeloides_92 | Tytthaspis_sedecimpunctata_586 | 0.195 |
| Rhyzobius_chrysomeloides_93 | Tytthaspis_sedecimpunctata_586 | 0.195 |
| Rhyzobius_chrysomeloides_94 | Tytthaspis_sedecimpunctata_586 | 0.195 |
| Rhyzobius_chrysomeloides_95 | Tytthaspis_sedecimpunctata_586 | 0.195 |
| Coccidula_scutellata_83 | Scymnus_rubromaculatus_822 | 0.195 |
| Hyperaspis_reppensis_662 | Scymnus_rubromaculatus_822 | 0.195 |
| Hyperaspis_reppensis_663 | Scymnus_rubromaculatus_822 | 0.195 |
| Coccidula_scutellata_83 | Scymnus_rubromaculatus_823 | 0.195 |
| Hyperaspis_reppensis_662 | Scymnus_rubromaculatus_823 | 0.195 |
| Hyperaspis_reppensis_663 | Scymnus_rubromaculatus_823 | 0.195 |
| Coccidula_scutellata_83 | Scymnus_rubromaculatus_824 | 0.195 |
| Hyperaspis_reppensis_662 | Scymnus_rubromaculatus_824 | 0.195 |
| Hyperaspis_reppensis_663 | Scymnus_rubromaculatus_824 | 0.195 |
| Coccidula_scutellata_83 | Scymnus_rubromaculatus_825 | 0.195 |
| Hyperaspis_reppensis_662 | Scymnus_rubromaculatus_825 | 0.195 |
| Hyperaspis_reppensis_663 | Scymnus_rubromaculatus_825 | 0.195 |

| | | |
|---|---|---|
| Coccidula_scutellata_83 | Scymnus_rubromaculatus_826 | 0.195 |
| Hyperaspis_reppensis_662 | Scymnus_rubromaculatus_826 | 0.195 |
| Hyperaspis_reppensis_663 | Scymnus_rubromaculatus_826 | 0.195 |
| Coccidula_scutellata_83 | Scymnus_rubromaculatus_827 | 0.195 |
| Hyperaspis_reppensis_662 | Scymnus_rubromaculatus_827 | 0.195 |
| Hyperaspis_reppensis_663 | Scymnus_rubromaculatus_827 | 0.195 |
| Brumus_quadripustulatus_12 | Scymnus_sinuanodulus_830 | 0.195 |
| Coccinella_quinquepunctata_233 | Vibidia_duodecimguttata_596 | 0.195 |
| Coccinella_quinquepunctata_234 | Vibidia_duodecimguttata_596 | 0.195 |
| Cynegetis_impunctata_614 | Subcoccinella_vigintiquatuorpunctata_623 | 0.195 |
| Coelophora_inaequalis_313 | Vibidia_duodecimguttata_596 | 0.195 |
| Coelophora_inaequalis_326 | Vibidia_duodecimguttata_596 | 0.195 |
| Coelophora_inaequalis_313 | Vibidia_duodecimguttata_598 | 0.195 |
| Coelophora_inaequalis_326 | Vibidia_duodecimguttata_598 | 0.195 |
| Coccinella_undecimpunctata_285 | Vibidia_duodecimguttata_601 | 0.195 |
| Coccinella_undecimpunctata_286 | Vibidia_duodecimguttata_601 | 0.195 |
| Coccinella_undecimpunctata_288 | Vibidia_duodecimguttata_601 | 0.195 |
| Coccinella_undecimpunctata_291 | Vibidia_duodecimguttata_603 | 0.195 |
| Coelophora_inaequalis_310 | Vibidia_duodecimguttata_603 | 0.195 |
| Coelophora_inaequalis_311 | Vibidia_duodecimguttata_603 | 0.195 |
| Coelophora_inaequalis_316 | Vibidia_duodecimguttata_603 | 0.195 |
| Coelophora_inaequalis_323 | Vibidia_duodecimguttata_603 | 0.195 |
| Coelophora_inaequalis_324 | Vibidia_duodecimguttata_603 | 0.195 |
| Macronaemia_episcopalis_477 | Scymnus_suturalis_841 | 0.195 |
| Macronaemia_episcopalis_477 | Scymnus_suturalis_842 | 0.195 |
| Macronaemia_episcopalis_477 | Scymnus_suturalis_844 | 0.195 |
| Macronaemia_episcopalis_477 | Scymnus_suturalis_846 | 0.195 |
| Macronaemia_episcopalis_477 | Scymnus_suturalis_847 | 0.195 |
| Macronaemia_episcopalis_477 | Scymnus_suturalis_848 | 0.195 |
| Hyperaspis_lateralis_656 | Subcoccinella_vigintiquatuorpunctata_623 | 0.195 |
| Hyperaspis_lateralis_657 | Subcoccinella_vigintiquatuorpunctata_623 | 0.195 |
| Brumus_quadripustulatus_12 | Scymnus_sinuanodulus_828 | 0.195 |
| Macronaemia_episcopalis_477 | Scymnus_suturalis_832 | 0.195 |
| Myrrha_octodecimguttata_485 | Scymnus_suturalis_833 | 0.195 |
| Myrrha_octodecimguttata_486 | Scymnus_suturalis_833 | 0.195 |
| Myrrha_octodecimguttata_487 | Scymnus_suturalis_833 | 0.195 |
| Myrrha_octodecimguttata_490 | Scymnus_suturalis_833 | 0.195 |
| Myrrha_octodecimguttata_494 | Scymnus_suturalis_833 | 0.195 |
| Myrrha_octodecimguttata_496 | Scymnus_suturalis_833 | 0.195 |
| Myrrha_octodecimguttata_497 | Scymnus_suturalis_833 | 0.195 |
| Myrrha_octodecimguttata_501 | Scymnus_suturalis_833 | 0.195 |
| Myrrha_octodecimguttata_502 | Scymnus_suturalis_833 | 0.195 |
| Macronaemia_episcopalis_477 | Scymnus_suturalis_835 | 0.195 |
| Macronaemia_episcopalis_477 | Scymnus_suturalis_836 | 0.195 |
| Macronaemia_episcopalis_477 | Scymnus_suturalis_839 | 0.195 |
| Clitostethus_arcuatus_642 | Scymnus_sinuanodulus_829 | 0.195 |
| Hyperaspis_oregona_658 | Stethorus_punctillum_857 | 0.195 |
| Hyperaspis_oregona_659 | Stethorus_punctillum_857 | 0.195 |
| Hyperaspis_lateralis_656 | Subcoccinella_vigintiquatuorpunctata_622 | 0.195 |
| Hyperaspis_lateralis_657 | Subcoccinella_vigintiquatuorpunctata_622 | 0.195 |
| Hyperaspis_reppensis_662 | Subcoccinella_vigintiquatuorpunctata_622 | 0.195 |
| Hyperaspis_reppensis_663 | Subcoccinella_vigintiquatuorpunctata_622 | 0.195 |
| Scymnus_ningshanensis_789 | Subcoccinella_vigintiquatuorpunctata_623 | 0.195 |
| Scymnus_ningshanensis_790 | Subcoccinella_vigintiquatuorpunctata_623 | 0.195 |
| Hyperaspis_lateralis_654 | Subcoccinella_vigintiquatuorpunctata_624 | 0.195 |
| Hyperaspis_lateralis_656 | Subcoccinella_vigintiquatuorpunctata_626 | 0.195 |

Supplementary Table S4 (continued)

| | | |
|---|---|---|
| Hyperaspis_lateralis_657 | Subcoccinella_vigintiquatuorpunctata_626 | 0.195 |
| Hyperaspis_reppensis_662 | Subcoccinella_vigintiquatuorpunctata_626 | 0.195 |
| Hyperaspis_reppensis_663 | Subcoccinella_vigintiquatuorpunctata_626 | 0.195 |
| Brachiacantha_ursina_637 | Tytthaspis_sedecimpunctata_577 | 0.195 |
| Myzia_pullata_519 | Tytthaspis_sedecimpunctata_577 | 0.195 |
| Scymnus_nebulosus_781 | Tytthaspis_sedecimpunctata_577 | 0.195 |
| Brachiacantha_ursina_637 | Tytthaspis_sedecimpunctata_578 | 0.195 |
| Myzia_pullata_519 | Tytthaspis_sedecimpunctata_578 | 0.195 |
| Scymnus_nebulosus_781 | Tytthaspis_sedecimpunctata_578 | 0.195 |
| Brachiacantha_ursina_637 | Tytthaspis_sedecimpunctata_579 | 0.195 |
| Myzia_pullata_519 | Tytthaspis_sedecimpunctata_579 | 0.195 |
| Scymnus_nebulosus_781 | Tytthaspis_sedecimpunctata_579 | 0.195 |
| Brachiacantha_ursina_637 | Tytthaspis_sedecimpunctata_581 | 0.195 |
| Myzia_pullata_519 | Tytthaspis_sedecimpunctata_581 | 0.195 |
| Scymnus_nebulosus_781 | Tytthaspis_sedecimpunctata_581 | 0.195 |
| Brachiacantha_ursina_637 | Tytthaspis_sedecimpunctata_582 | 0.195 |
| Myzia_pullata_519 | Tytthaspis_sedecimpunctata_582 | 0.195 |
| Scymnus_nebulosus_781 | Tytthaspis_sedecimpunctata_582 | 0.195 |
| Scymnus_nebulosus_783 | Tytthaspis_sedecimpunctata_583 | 0.195 |
| Brachiacantha_ursina_638 | Tytthaspis_sedecimpunctata_585 | 0.195 |
| Myzia_pullata_519 | Tytthaspis_sedecimpunctata_585 | 0.195 |
| Scymnus_nebulosus_781 | Tytthaspis_sedecimpunctata_585 | 0.195 |
| Scymnus_ningshanensis_789 | Tytthaspis_sedecimpunctata_587 | 0.195 |
| Scymnus_ningshanensis_790 | Tytthaspis_sedecimpunctata_587 | 0.195 |
| Brachiacantha_ursina_637 | Tytthaspis_sedecimpunctata_590 | 0.195 |
| Myzia_pullata_519 | Tytthaspis_sedecimpunctata_590 | 0.195 |
| Scymnus_nebulosus_781 | Tytthaspis_sedecimpunctata_590 | 0.195 |
| Brachiacantha_ursina_637 | Tytthaspis_sedecimpunctata_592 | 0.195 |
| Myzia_pullata_519 | Tytthaspis_sedecimpunctata_592 | 0.195 |
| Scymnus_nebulosus_781 | Tytthaspis_sedecimpunctata_592 | 0.195 |
| Brachiacantha_ursina_638 | Tytthaspis_sedecimpunctata_593 | 0.195 |
| Brachiacantha_ursina_637 | Tytthaspis_sedecimpunctata_594 | 0.195 |
| Myzia_pullata_519 | Tytthaspis_sedecimpunctata_594 | 0.195 |
| Scymnus_nebulosus_781 | Tytthaspis_sedecimpunctata_594 | 0.195 |
| Brachiacantha_ursina_637 | Tytthaspis_sedecimpunctata_595 | 0.195 |
| Myzia_pullata_519 | Tytthaspis_sedecimpunctata_595 | 0.195 |
| Scymnus_nebulosus_781 | Tytthaspis_sedecimpunctata_595 | 0.195 |
| Nephus_redtenbacheri_674 | Scymnus_suturalis_838 | 0.195 |
| Nephus_redtenbacheri_675 | Scymnus_suturalis_838 | 0.195 |
| Nephus_redtenbacheri_674 | Scymnus_suturalis_840 | 0.195 |
| Nephus_redtenbacheri_675 | Scymnus_suturalis_840 | 0.195 |
| Cynegetis_impunctata_604 | Subcoccinella_vigintiquatuorpunctata_620 | 0.196 |
| Cynegetis_impunctata_605 | Subcoccinella_vigintiquatuorpunctata_620 | 0.196 |
| Cynegetis_impunctata_606 | Subcoccinella_vigintiquatuorpunctata_620 | 0.196 |
| Cynegetis_impunctata_607 | Subcoccinella_vigintiquatuorpunctata_620 | 0.196 |
| Cynegetis_impunctata_608 | Subcoccinella_vigintiquatuorpunctata_620 | 0.196 |
| Cynegetis_impunctata_609 | Subcoccinella_vigintiquatuorpunctata_620 | 0.196 |
| Cynegetis_impunctata_610 | Subcoccinella_vigintiquatuorpunctata_620 | 0.196 |
| Cynegetis_impunctata_611 | Subcoccinella_vigintiquatuorpunctata_620 | 0.196 |
| Cynegetis_impunctata_612 | Subcoccinella_vigintiquatuorpunctata_620 | 0.196 |
| Cynegetis_impunctata_613 | Subcoccinella_vigintiquatuorpunctata_620 | 0.196 |
| Cynegetis_impunctata_604 | Subcoccinella_vigintiquatuorpunctata_621 | 0.196 |
| Cynegetis_impunctata_605 | Subcoccinella_vigintiquatuorpunctata_621 | 0.196 |
| Cynegetis_impunctata_606 | Subcoccinella_vigintiquatuorpunctata_621 | 0.196 |
| Cynegetis_impunctata_607 | Subcoccinella_vigintiquatuorpunctata_621 | 0.196 |
| Cynegetis_impunctata_608 | Subcoccinella_vigintiquatuorpunctata_621 | 0.196 |

| | | |
|---|---|---|
| Cynegetis_impunctata_609 | Subcoccinella_vigintiquatuorpunctata_621 | 0.196 |
| Cynegetis_impunctata_610 | Subcoccinella_vigintiquatuorpunctata_621 | 0.196 |
| Cynegetis_impunctata_611 | Subcoccinella_vigintiquatuorpunctata_621 | 0.196 |
| Cynegetis_impunctata_612 | Subcoccinella_vigintiquatuorpunctata_621 | 0.196 |
| Cynegetis_impunctata_613 | Subcoccinella_vigintiquatuorpunctata_621 | 0.196 |
| Cynegetis_impunctata_614 | Subcoccinella_vigintiquatuorpunctata_626 | 0.196 |
| Cynegetis_impunctata_604 | Subcoccinella_vigintiquatuorpunctata_627 | 0.196 |
| Cynegetis_impunctata_605 | Subcoccinella_vigintiquatuorpunctata_627 | 0.196 |
| Cynegetis_impunctata_606 | Subcoccinella_vigintiquatuorpunctata_627 | 0.196 |
| Cynegetis_impunctata_607 | Subcoccinella_vigintiquatuorpunctata_627 | 0.196 |
| Cynegetis_impunctata_608 | Subcoccinella_vigintiquatuorpunctata_627 | 0.196 |
| Cynegetis_impunctata_609 | Subcoccinella_vigintiquatuorpunctata_627 | 0.196 |
| Cynegetis_impunctata_610 | Subcoccinella_vigintiquatuorpunctata_627 | 0.196 |
| Cynegetis_impunctata_611 | Subcoccinella_vigintiquatuorpunctata_627 | 0.196 |
| Cynegetis_impunctata_612 | Subcoccinella_vigintiquatuorpunctata_627 | 0.196 |
| Cynegetis_impunctata_613 | Subcoccinella_vigintiquatuorpunctata_627 | 0.196 |
| Cynegetis_impunctata_604 | Subcoccinella_vigintiquatuorpunctata_628 | 0.196 |
| Cynegetis_impunctata_605 | Subcoccinella_vigintiquatuorpunctata_628 | 0.196 |
| Cynegetis_impunctata_606 | Subcoccinella_vigintiquatuorpunctata_628 | 0.196 |
| Cynegetis_impunctata_607 | Subcoccinella_vigintiquatuorpunctata_628 | 0.196 |
| Cynegetis_impunctata_608 | Subcoccinella_vigintiquatuorpunctata_628 | 0.196 |
| Cynegetis_impunctata_609 | Subcoccinella_vigintiquatuorpunctata_628 | 0.196 |
| Cynegetis_impunctata_610 | Subcoccinella_vigintiquatuorpunctata_628 | 0.196 |
| Cynegetis_impunctata_611 | Subcoccinella_vigintiquatuorpunctata_628 | 0.196 |
| Cynegetis_impunctata_612 | Subcoccinella_vigintiquatuorpunctata_628 | 0.196 |
| Cynegetis_impunctata_613 | Subcoccinella_vigintiquatuorpunctata_628 | 0.196 |
| Cynegetis_impunctata_604 | Subcoccinella_vigintiquatuorpunctata_629 | 0.196 |
| Cynegetis_impunctata_605 | Subcoccinella_vigintiquatuorpunctata_629 | 0.196 |
| Cynegetis_impunctata_606 | Subcoccinella_vigintiquatuorpunctata_629 | 0.196 |
| Cynegetis_impunctata_607 | Subcoccinella_vigintiquatuorpunctata_629 | 0.196 |
| Cynegetis_impunctata_608 | Subcoccinella_vigintiquatuorpunctata_629 | 0.196 |
| Cynegetis_impunctata_609 | Subcoccinella_vigintiquatuorpunctata_629 | 0.196 |
| Cynegetis_impunctata_610 | Subcoccinella_vigintiquatuorpunctata_629 | 0.196 |
| Cynegetis_impunctata_611 | Subcoccinella_vigintiquatuorpunctata_629 | 0.196 |
| Cynegetis_impunctata_612 | Subcoccinella_vigintiquatuorpunctata_629 | 0.196 |
| Cynegetis_impunctata_613 | Subcoccinella_vigintiquatuorpunctata_629 | 0.196 |
| Cynegetis_impunctata_604 | Subcoccinella_vigintiquatuorpunctata_630 | 0.196 |
| Cynegetis_impunctata_605 | Subcoccinella_vigintiquatuorpunctata_630 | 0.196 |
| Cynegetis_impunctata_606 | Subcoccinella_vigintiquatuorpunctata_630 | 0.196 |
| Cynegetis_impunctata_607 | Subcoccinella_vigintiquatuorpunctata_630 | 0.196 |
| Cynegetis_impunctata_608 | Subcoccinella_vigintiquatuorpunctata_630 | 0.196 |
| Cynegetis_impunctata_609 | Subcoccinella_vigintiquatuorpunctata_630 | 0.196 |
| Cynegetis_impunctata_610 | Subcoccinella_vigintiquatuorpunctata_630 | 0.196 |
| Cynegetis_impunctata_611 | Subcoccinella_vigintiquatuorpunctata_630 | 0.196 |
| Cynegetis_impunctata_612 | Subcoccinella_vigintiquatuorpunctata_630 | 0.196 |
| Cynegetis_impunctata_613 | Subcoccinella_vigintiquatuorpunctata_630 | 0.196 |
| Cynegetis_impunctata_604 | Subcoccinella_vigintiquatuorpunctata_631 | 0.196 |
| Cynegetis_impunctata_605 | Subcoccinella_vigintiquatuorpunctata_631 | 0.196 |
| Cynegetis_impunctata_606 | Subcoccinella_vigintiquatuorpunctata_631 | 0.196 |
| Cynegetis_impunctata_607 | Subcoccinella_vigintiquatuorpunctata_631 | 0.196 |
| Cynegetis_impunctata_608 | Subcoccinella_vigintiquatuorpunctata_631 | 0.196 |
| Cynegetis_impunctata_609 | Subcoccinella_vigintiquatuorpunctata_631 | 0.196 |
| Cynegetis_impunctata_610 | Subcoccinella_vigintiquatuorpunctata_631 | 0.196 |
| Cynegetis_impunctata_611 | Subcoccinella_vigintiquatuorpunctata_631 | 0.196 |
| Cynegetis_impunctata_612 | Subcoccinella_vigintiquatuorpunctata_631 | 0.196 |
| Cynegetis_impunctata_613 | Subcoccinella_vigintiquatuorpunctata_631 | 0.196 |

Supplementary Table S4 (continued)

| | | |
|---|---|---|
| Nephus_redtenbacheri_674 | Scymnus_suturalis_843 | 0.196 |
| Nephus_redtenbacheri_675 | Scymnus_suturalis_843 | 0.196 |
| Hyperaspis_campestris_651 | Stethorus_punctillum_850 | 0.196 |
| Hyperaspis_campestris_652 | Stethorus_punctillum_850 | 0.196 |
| Hyperaspis_campestris_653 | Stethorus_punctillum_850 | 0.196 |
| Scymnus_apetzi_691 | Subcoccinella_vigintiquatuorpunctata_623 | 0.196 |
| Scymnus_ningshanensis_789 | Subcoccinella_vigintiquatuorpunctata_626 | 0.196 |
| Scymnus_ningshanensis_790 | Subcoccinella_vigintiquatuorpunctata_626 | 0.196 |
| Myzia_pullata_519 | Tytthaspis_sedecimpunctata_580 | 0.196 |
| Scymnus_nebulosus_783 | Tytthaspis_sedecimpunctata_580 | 0.196 |
| Scymnus_nigrinus_784 | Tytthaspis_sedecimpunctata_580 | 0.196 |
| Scymnus_interruptus_762 | Tytthaspis_sedecimpunctata_584 | 0.196 |
| Brachiacantha_ursina_637 | Tytthaspis_sedecimpunctata_586 | 0.196 |
| Coccinella_septempunctata_246 | Tytthaspis_sedecimpunctata_587 | 0.196 |
| Brachiacantha_ursina_638 | Tytthaspis_sedecimpunctata_588 | 0.196 |
| Brachiacantha_rotunda_632 | Scymnus_rubromaculatus_822 | 0.196 |
| Brachiacantha_rotunda_633 | Scymnus_rubromaculatus_822 | 0.196 |
| Brachiacantha_rotunda_634 | Scymnus_rubromaculatus_822 | 0.196 |
| Brachiacantha_rotunda_635 | Scymnus_rubromaculatus_822 | 0.196 |
| Nephus_redtenbacheri_680 | Scymnus_rubromaculatus_822 | 0.196 |
| Nephus_redtenbacheri_681 | Scymnus_rubromaculatus_822 | 0.196 |
| Brachiacantha_rotunda_632 | Scymnus_rubromaculatus_823 | 0.196 |
| Brachiacantha_rotunda_633 | Scymnus_rubromaculatus_823 | 0.196 |
| Brachiacantha_rotunda_634 | Scymnus_rubromaculatus_823 | 0.196 |
| Brachiacantha_rotunda_635 | Scymnus_rubromaculatus_823 | 0.196 |
| Nephus_redtenbacheri_680 | Scymnus_rubromaculatus_823 | 0.196 |
| Nephus_redtenbacheri_681 | Scymnus_rubromaculatus_823 | 0.196 |
| Brachiacantha_rotunda_632 | Scymnus_rubromaculatus_824 | 0.196 |
| Brachiacantha_rotunda_633 | Scymnus_rubromaculatus_824 | 0.196 |
| Brachiacantha_rotunda_634 | Scymnus_rubromaculatus_824 | 0.196 |
| Brachiacantha_rotunda_635 | Scymnus_rubromaculatus_824 | 0.196 |
| Nephus_redtenbacheri_680 | Scymnus_rubromaculatus_824 | 0.196 |
| Nephus_redtenbacheri_681 | Scymnus_rubromaculatus_824 | 0.196 |
| Brachiacantha_rotunda_632 | Scymnus_rubromaculatus_825 | 0.196 |
| Brachiacantha_rotunda_633 | Scymnus_rubromaculatus_825 | 0.196 |
| Brachiacantha_rotunda_634 | Scymnus_rubromaculatus_825 | 0.196 |
| Brachiacantha_rotunda_635 | Scymnus_rubromaculatus_825 | 0.196 |
| Nephus_redtenbacheri_680 | Scymnus_rubromaculatus_825 | 0.196 |
| Nephus_redtenbacheri_681 | Scymnus_rubromaculatus_825 | 0.196 |
| Brachiacantha_rotunda_632 | Scymnus_rubromaculatus_826 | 0.196 |
| Brachiacantha_rotunda_633 | Scymnus_rubromaculatus_826 | 0.196 |
| Brachiacantha_rotunda_634 | Scymnus_rubromaculatus_826 | 0.196 |
| Brachiacantha_rotunda_635 | Scymnus_rubromaculatus_826 | 0.196 |
| Nephus_redtenbacheri_680 | Scymnus_rubromaculatus_826 | 0.196 |
| Nephus_redtenbacheri_681 | Scymnus_rubromaculatus_826 | 0.196 |
| Brachiacantha_rotunda_632 | Scymnus_rubromaculatus_827 | 0.196 |
| Brachiacantha_rotunda_633 | Scymnus_rubromaculatus_827 | 0.196 |
| Brachiacantha_rotunda_634 | Scymnus_rubromaculatus_827 | 0.196 |
| Brachiacantha_rotunda_635 | Scymnus_rubromaculatus_827 | 0.196 |
| Nephus_redtenbacheri_680 | Scymnus_rubromaculatus_827 | 0.196 |
| Nephus_redtenbacheri_681 | Scymnus_rubromaculatus_827 | 0.196 |
| Diomus_terminatus_647 | Scymnus_sinuanodulus_828 | 0.196 |
| Diomus_terminatus_648 | Scymnus_sinuanodulus_828 | 0.196 |
| Rhyzobius_litura_96 | Scymnus_suturalis_841 | 0.196 |
| Rhyzobius_litura_96 | Scymnus_suturalis_842 | 0.196 |
| Rhyzobius_litura_97 | Scymnus_suturalis_843 | 0.196 |

| | | |
|---|---|---|
| Rhyzobius_litura_96 | Scymnus_suturalis_844 | 0.196 |
| Rhyzobius_litura_97 | Scymnus_suturalis_845 | 0.196 |
| Rhyzobius_litura_96 | Scymnus_suturalis_846 | 0.196 |
| Rhyzobius_litura_96 | Scymnus_suturalis_847 | 0.196 |
| Rhyzobius_litura_96 | Scymnus_suturalis_848 | 0.196 |
| Hyperaspis_proba_661 | Stethorus_punctillum_851 | 0.196 |
| Hyperaspis_proba_661 | Stethorus_punctillum_858 | 0.196 |
| Scymnus_apetzi_690 | Subcoccinella_vigintiquatuorpunctata_620 | 0.196 |
| Scymnus_apetzi_697 | Subcoccinella_vigintiquatuorpunctata_620 | 0.196 |
| Scymnus_pallipediformis_811 | Subcoccinella_vigintiquatuorpunctata_620 | 0.196 |
| Scymnus_apetzi_690 | Subcoccinella_vigintiquatuorpunctata_621 | 0.196 |
| Scymnus_apetzi_697 | Subcoccinella_vigintiquatuorpunctata_621 | 0.196 |
| Scymnus_pallipediformis_811 | Subcoccinella_vigintiquatuorpunctata_621 | 0.196 |
| Scymnus_apetzi_691 | Subcoccinella_vigintiquatuorpunctata_622 | 0.196 |
| Scymnus_apetzi_691 | Subcoccinella_vigintiquatuorpunctata_626 | 0.196 |
| Scymnus_apetzi_696 | Subcoccinella_vigintiquatuorpunctata_627 | 0.196 |
| Scymnus_nebulosus_783 | Subcoccinella_vigintiquatuorpunctata_627 | 0.196 |
| Scymnus_apetzi_690 | Subcoccinella_vigintiquatuorpunctata_628 | 0.196 |
| Scymnus_apetzi_697 | Subcoccinella_vigintiquatuorpunctata_628 | 0.196 |
| Scymnus_pallipediformis_811 | Subcoccinella_vigintiquatuorpunctata_628 | 0.196 |
| Scymnus_apetzi_690 | Subcoccinella_vigintiquatuorpunctata_629 | 0.196 |
| Scymnus_apetzi_697 | Subcoccinella_vigintiquatuorpunctata_629 | 0.196 |
| Scymnus_pallipediformis_811 | Subcoccinella_vigintiquatuorpunctata_629 | 0.196 |
| Scymnus_apetzi_690 | Subcoccinella_vigintiquatuorpunctata_631 | 0.196 |
| Scymnus_apetzi_697 | Subcoccinella_vigintiquatuorpunctata_631 | 0.196 |
| Scymnus_pallipediformis_811 | Subcoccinella_vigintiquatuorpunctata_631 | 0.196 |
| Scymnus_interruptus_762 | Tytthaspis_sedecimpunctata_577 | 0.196 |
| Scymnus_interruptus_762 | Tytthaspis_sedecimpunctata_578 | 0.196 |
| Scymnus_interruptus_762 | Tytthaspis_sedecimpunctata_579 | 0.196 |
| Scymnus_interruptus_762 | Tytthaspis_sedecimpunctata_581 | 0.196 |
| Scymnus_interruptus_762 | Tytthaspis_sedecimpunctata_582 | 0.196 |
| Brachiacantha_rotunda_634 | Tytthaspis_sedecimpunctata_584 | 0.196 |
| Scymnus_interruptus_759 | Tytthaspis_sedecimpunctata_584 | 0.196 |
| Scymnus_interruptus_769 | Tytthaspis_sedecimpunctata_584 | 0.196 |
| Scymnus_interruptus_762 | Tytthaspis_sedecimpunctata_589 | 0.196 |
| Scymnus_interruptus_762 | Tytthaspis_sedecimpunctata_590 | 0.196 |
| Scymnus_camptodromus_705 | Tytthaspis_sedecimpunctata_591 | 0.196 |
| Scymnus_camptodromus_713 | Tytthaspis_sedecimpunctata_591 | 0.196 |
| Scymnus_camptodromus_716 | Tytthaspis_sedecimpunctata_591 | 0.196 |
| Scymnus_interruptus_762 | Tytthaspis_sedecimpunctata_593 | 0.196 |
| Scymnus_interruptus_762 | Tytthaspis_sedecimpunctata_594 | 0.196 |
| Scymnus_interruptus_762 | Tytthaspis_sedecimpunctata_595 | 0.196 |
| Rhyzobius_litura_96 | Scymnus_suturalis_832 | 0.196 |
| Calvia_quatuordecimguttata_211 | Scymnus_suturalis_833 | 0.196 |
| Calvia_quatuordecimguttata_212 | Scymnus_suturalis_833 | 0.196 |
| Calvia_quatuordecimguttata_213 | Scymnus_suturalis_833 | 0.196 |
| Calvia_quatuordecimguttata_214 | Scymnus_suturalis_833 | 0.196 |
| Calvia_quatuordecimguttata_215 | Scymnus_suturalis_833 | 0.196 |
| Rhyzobius_litura_96 | Scymnus_suturalis_835 | 0.196 |
| Rhyzobius_litura_96 | Scymnus_suturalis_836 | 0.196 |
| Calvia_quatuordecimguttata_201 | Scymnus_suturalis_838 | 0.196 |
| Calvia_quatuordecimguttata_202 | Scymnus_suturalis_838 | 0.196 |
| Calvia_quatuordecimguttata_210 | Scymnus_suturalis_838 | 0.196 |
| Rhyzobius_litura_96 | Scymnus_suturalis_839 | 0.196 |
| Calvia_quatuordecimguttata_201 | Scymnus_suturalis_840 | 0.196 |
| Calvia_quatuordecimguttata_202 | Scymnus_suturalis_840 | 0.196 |

| | | |
|---|---|---|
| Calvia_quatuordecimguttata_210 | Scymnus_suturalis_840 | 0.196 |
| Myrrha_octodecimguttata_485 | Scymnus_sinuanodulus_831 | 0.196 |
| Myrrha_octodecimguttata_486 | Scymnus_sinuanodulus_831 | 0.196 |
| Myrrha_octodecimguttata_487 | Scymnus_sinuanodulus_831 | 0.196 |
| Myrrha_octodecimguttata_490 | Scymnus_sinuanodulus_831 | 0.196 |
| Myrrha_octodecimguttata_494 | Scymnus_sinuanodulus_831 | 0.196 |
| Myrrha_octodecimguttata_496 | Scymnus_sinuanodulus_831 | 0.196 |
| Myrrha_octodecimguttata_497 | Scymnus_sinuanodulus_831 | 0.196 |
| Myrrha_octodecimguttata_501 | Scymnus_sinuanodulus_831 | 0.196 |
| Myrrha_octodecimguttata_502 | Scymnus_sinuanodulus_831 | 0.196 |
| Hyperaspis_reppensis_667 | Stethorus_punctillum_849 | 0.196 |
| Hyperaspis_reppensis_664 | Stethorus_punctillum_850 | 0.196 |
| Hyperaspis_reppensis_665 | Stethorus_punctillum_850 | 0.196 |
| Hyperaspis_reppensis_666 | Stethorus_punctillum_850 | 0.196 |
| Hyperaspis_reppensis_667 | Stethorus_punctillum_854 | 0.196 |
| Hyperaspis_reppensis_667 | Stethorus_punctillum_856 | 0.196 |
| Scymnus_interruptus_759 | Tytthaspis_sedecimpunctata_577 | 0.196 |
| Scymnus_interruptus_769 | Tytthaspis_sedecimpunctata_577 | 0.196 |
| Scymnus_interruptus_759 | Tytthaspis_sedecimpunctata_578 | 0.196 |
| Scymnus_interruptus_769 | Tytthaspis_sedecimpunctata_578 | 0.196 |
| Scymnus_interruptus_759 | Tytthaspis_sedecimpunctata_579 | 0.196 |
| Scymnus_interruptus_769 | Tytthaspis_sedecimpunctata_579 | 0.196 |
| Scymnus_interruptus_759 | Tytthaspis_sedecimpunctata_581 | 0.196 |
| Scymnus_interruptus_769 | Tytthaspis_sedecimpunctata_581 | 0.196 |
| Scymnus_interruptus_759 | Tytthaspis_sedecimpunctata_582 | 0.196 |
| Scymnus_interruptus_769 | Tytthaspis_sedecimpunctata_582 | 0.196 |
| Scymnus_interruptus_761 | Tytthaspis_sedecimpunctata_585 | 0.196 |
| Scymnus_interruptus_763 | Tytthaspis_sedecimpunctata_585 | 0.196 |
| Scymnus_interruptus_764 | Tytthaspis_sedecimpunctata_585 | 0.196 |
| Scymnus_interruptus_766 | Tytthaspis_sedecimpunctata_585 | 0.196 |
| Scymnus_interruptus_762 | Tytthaspis_sedecimpunctata_586 | 0.196 |
| Scymnus_interruptus_761 | Tytthaspis_sedecimpunctata_589 | 0.196 |
| Scymnus_interruptus_763 | Tytthaspis_sedecimpunctata_589 | 0.196 |
| Scymnus_interruptus_764 | Tytthaspis_sedecimpunctata_589 | 0.196 |
| Scymnus_interruptus_766 | Tytthaspis_sedecimpunctata_589 | 0.196 |
| Scymnus_interruptus_759 | Tytthaspis_sedecimpunctata_590 | 0.196 |
| Scymnus_interruptus_769 | Tytthaspis_sedecimpunctata_590 | 0.196 |
| Scymnus_interruptus_759 | Tytthaspis_sedecimpunctata_593 | 0.196 |
| Scymnus_interruptus_769 | Tytthaspis_sedecimpunctata_593 | 0.196 |
| Scymnus_interruptus_759 | Tytthaspis_sedecimpunctata_594 | 0.196 |
| Scymnus_interruptus_769 | Tytthaspis_sedecimpunctata_594 | 0.196 |
| Scymnus_interruptus_759 | Tytthaspis_sedecimpunctata_595 | 0.196 |
| Scymnus_interruptus_769 | Tytthaspis_sedecimpunctata_595 | 0.196 |
| Harmonia_axyridis_355 | Scymnus_suturalis_833 | 0.196 |
| Calvia_quatuordecimguttata_201 | Scymnus_suturalis_834 | 0.196 |
| Calvia_quatuordecimguttata_202 | Scymnus_suturalis_834 | 0.196 |
| Calvia_quatuordecimguttata_210 | Scymnus_suturalis_834 | 0.196 |
| Calvia_quatuordecimguttata_201 | Scymnus_suturalis_837 | 0.196 |
| Calvia_quatuordecimguttata_202 | Scymnus_suturalis_837 | 0.196 |
| Calvia_quatuordecimguttata_210 | Scymnus_suturalis_837 | 0.196 |
| Harmonia_axyridis_355 | Scymnus_suturalis_838 | 0.196 |
| Rhyzobius_litura_96 | Scymnus_suturalis_838 | 0.196 |
| Clitostethus_arcuatus_642 | Scymnus_suturalis_840 | 0.196 |
| Harmonia_axyridis_355 | Scymnus_suturalis_840 | 0.196 |
| Coelophora_inaequalis_318 | Vibidia_duodecimguttata_599 | 0.196 |
| Coelophora_inaequalis_318 | Vibidia_duodecimguttata_601 | 0.196 |

| | | |
|---|---|---|
| Coelophora_inaequalis_318 | Vibidia_duodecimguttata_602 | 0.196 |
| Coccinella_undecimpunctata_289 | Vibidia_duodecimguttata_603 | 0.196 |
| Coccinella_undecimpunctata_290 | Vibidia_duodecimguttata_603 | 0.196 |
| Platynaspis_luteorubra_50 | Subcoccinella_vigintiquatuorpunctata_627 | 0.196 |
| Platynaspis_luteorubra_50 | Subcoccinella_vigintiquatuorpunctata_630 | 0.196 |
| Calvia_quatuordecimguttata_218 | Scymnus_suturalis_841 | 0.196 |
| Clitostethus_arcuatus_639 | Scymnus_suturalis_841 | 0.196 |
| Clitostethus_arcuatus_640 | Scymnus_suturalis_841 | 0.196 |
| Clitostethus_arcuatus_641 | Scymnus_suturalis_841 | 0.196 |
| Clitostethus_arcuatus_643 | Scymnus_suturalis_841 | 0.196 |
| Calvia_quatuordecimguttata_218 | Scymnus_suturalis_842 | 0.196 |
| Clitostethus_arcuatus_639 | Scymnus_suturalis_842 | 0.196 |
| Clitostethus_arcuatus_640 | Scymnus_suturalis_842 | 0.196 |
| Clitostethus_arcuatus_641 | Scymnus_suturalis_842 | 0.196 |
| Clitostethus_arcuatus_643 | Scymnus_suturalis_842 | 0.196 |
| Calvia_quatuordecimguttata_218 | Scymnus_suturalis_844 | 0.196 |
| Clitostethus_arcuatus_639 | Scymnus_suturalis_844 | 0.196 |
| Clitostethus_arcuatus_640 | Scymnus_suturalis_844 | 0.196 |
| Clitostethus_arcuatus_641 | Scymnus_suturalis_844 | 0.196 |
| Clitostethus_arcuatus_643 | Scymnus_suturalis_844 | 0.196 |
| Clitostethus_arcuatus_642 | Scymnus_suturalis_845 | 0.196 |
| Calvia_quatuordecimguttata_218 | Scymnus_suturalis_846 | 0.196 |
| Clitostethus_arcuatus_639 | Scymnus_suturalis_846 | 0.196 |
| Clitostethus_arcuatus_640 | Scymnus_suturalis_846 | 0.196 |
| Clitostethus_arcuatus_641 | Scymnus_suturalis_846 | 0.196 |
| Clitostethus_arcuatus_643 | Scymnus_suturalis_846 | 0.196 |
| Calvia_quatuordecimguttata_218 | Scymnus_suturalis_847 | 0.196 |
| Clitostethus_arcuatus_639 | Scymnus_suturalis_847 | 0.196 |
| Clitostethus_arcuatus_640 | Scymnus_suturalis_847 | 0.196 |
| Clitostethus_arcuatus_641 | Scymnus_suturalis_847 | 0.196 |
| Clitostethus_arcuatus_643 | Scymnus_suturalis_847 | 0.196 |
| Calvia_quatuordecimguttata_218 | Scymnus_suturalis_848 | 0.196 |
| Clitostethus_arcuatus_639 | Scymnus_suturalis_848 | 0.196 |
| Clitostethus_arcuatus_640 | Scymnus_suturalis_848 | 0.196 |
| Clitostethus_arcuatus_641 | Scymnus_suturalis_848 | 0.196 |
| Clitostethus_arcuatus_643 | Scymnus_suturalis_848 | 0.196 |
| Hyperaspis_lateralis_657 | Stethorus_punctillum_852 | 0.196 |
| Psyllobora_vigintiduopunctata_570 | Subcoccinella_vigintiquatuorpunctata_624 | 0.196 |
| Psyllobora_vigintiduopunctata_572 | Subcoccinella_vigintiquatuorpunctata_624 | 0.196 |
| Psyllobora_vigintiduopunctata_570 | Subcoccinella_vigintiquatuorpunctata_630 | 0.196 |
| Psyllobora_vigintiduopunctata_572 | Subcoccinella_vigintiquatuorpunctata_630 | 0.196 |
| Scymnus_ferrugatus_723 | Tytthaspis_sedecimpunctata_577 | 0.196 |
| Scymnus_ferrugatus_725 | Tytthaspis_sedecimpunctata_577 | 0.196 |
| Scymnus_ferrugatus_726 | Tytthaspis_sedecimpunctata_577 | 0.196 |
| Scymnus_ferrugatus_727 | Tytthaspis_sedecimpunctata_577 | 0.196 |
| Scymnus_ferrugatus_728 | Tytthaspis_sedecimpunctata_577 | 0.196 |
| Scymnus_ferrugatus_730 | Tytthaspis_sedecimpunctata_577 | 0.196 |
| Scymnus_ferrugatus_731 | Tytthaspis_sedecimpunctata_577 | 0.196 |
| Scymnus_ferrugatus_732 | Tytthaspis_sedecimpunctata_577 | 0.196 |
| Scymnus_ferrugatus_733 | Tytthaspis_sedecimpunctata_577 | 0.196 |
| Scymnus_ferrugatus_723 | Tytthaspis_sedecimpunctata_578 | 0.196 |
| Scymnus_ferrugatus_725 | Tytthaspis_sedecimpunctata_578 | 0.196 |
| Scymnus_ferrugatus_726 | Tytthaspis_sedecimpunctata_578 | 0.196 |
| Scymnus_ferrugatus_727 | Tytthaspis_sedecimpunctata_578 | 0.196 |
| Scymnus_ferrugatus_728 | Tytthaspis_sedecimpunctata_578 | 0.196 |
| Scymnus_ferrugatus_730 | Tytthaspis_sedecimpunctata_578 | 0.196 |

| | | |
|---|---|---|
| Scymnus_ferrugatus_731 | Tytthaspis_sedecimpunctata_578 | 0.196 |
| Scymnus_ferrugatus_732 | Tytthaspis_sedecimpunctata_578 | 0.196 |
| Scymnus_ferrugatus_733 | Tytthaspis_sedecimpunctata_578 | 0.196 |
| Scymnus_ferrugatus_723 | Tytthaspis_sedecimpunctata_579 | 0.196 |
| Scymnus_ferrugatus_725 | Tytthaspis_sedecimpunctata_579 | 0.196 |
| Scymnus_ferrugatus_726 | Tytthaspis_sedecimpunctata_579 | 0.196 |
| Scymnus_ferrugatus_727 | Tytthaspis_sedecimpunctata_579 | 0.196 |
| Scymnus_ferrugatus_728 | Tytthaspis_sedecimpunctata_579 | 0.196 |
| Scymnus_ferrugatus_730 | Tytthaspis_sedecimpunctata_579 | 0.196 |
| Scymnus_ferrugatus_731 | Tytthaspis_sedecimpunctata_579 | 0.196 |
| Scymnus_ferrugatus_732 | Tytthaspis_sedecimpunctata_579 | 0.196 |
| Scymnus_ferrugatus_733 | Tytthaspis_sedecimpunctata_579 | 0.196 |
| Scymnus_ferrugatus_723 | Tytthaspis_sedecimpunctata_581 | 0.196 |
| Scymnus_ferrugatus_725 | Tytthaspis_sedecimpunctata_581 | 0.196 |
| Scymnus_ferrugatus_726 | Tytthaspis_sedecimpunctata_581 | 0.196 |
| Scymnus_ferrugatus_727 | Tytthaspis_sedecimpunctata_581 | 0.196 |
| Scymnus_ferrugatus_728 | Tytthaspis_sedecimpunctata_581 | 0.196 |
| Scymnus_ferrugatus_730 | Tytthaspis_sedecimpunctata_581 | 0.196 |
| Scymnus_ferrugatus_731 | Tytthaspis_sedecimpunctata_581 | 0.196 |
| Scymnus_ferrugatus_732 | Tytthaspis_sedecimpunctata_581 | 0.196 |
| Scymnus_ferrugatus_733 | Tytthaspis_sedecimpunctata_581 | 0.196 |
| Scymnus_ferrugatus_723 | Tytthaspis_sedecimpunctata_582 | 0.196 |
| Scymnus_ferrugatus_725 | Tytthaspis_sedecimpunctata_582 | 0.196 |
| Scymnus_ferrugatus_726 | Tytthaspis_sedecimpunctata_582 | 0.196 |
| Scymnus_ferrugatus_727 | Tytthaspis_sedecimpunctata_582 | 0.196 |
| Scymnus_ferrugatus_728 | Tytthaspis_sedecimpunctata_582 | 0.196 |
| Scymnus_ferrugatus_730 | Tytthaspis_sedecimpunctata_582 | 0.196 |
| Scymnus_ferrugatus_731 | Tytthaspis_sedecimpunctata_582 | 0.196 |
| Scymnus_ferrugatus_732 | Tytthaspis_sedecimpunctata_582 | 0.196 |
| Scymnus_ferrugatus_733 | Tytthaspis_sedecimpunctata_582 | 0.196 |
| Scymnus_ferrugatus_723 | Tytthaspis_sedecimpunctata_585 | 0.196 |
| Scymnus_ferrugatus_725 | Tytthaspis_sedecimpunctata_585 | 0.196 |
| Scymnus_ferrugatus_726 | Tytthaspis_sedecimpunctata_585 | 0.196 |
| Scymnus_ferrugatus_727 | Tytthaspis_sedecimpunctata_585 | 0.196 |
| Scymnus_ferrugatus_728 | Tytthaspis_sedecimpunctata_585 | 0.196 |
| Scymnus_ferrugatus_730 | Tytthaspis_sedecimpunctata_585 | 0.196 |
| Scymnus_ferrugatus_731 | Tytthaspis_sedecimpunctata_585 | 0.196 |
| Scymnus_ferrugatus_732 | Tytthaspis_sedecimpunctata_585 | 0.196 |
| Scymnus_ferrugatus_733 | Tytthaspis_sedecimpunctata_585 | 0.196 |
| Scymnus_interruptus_761 | Tytthaspis_sedecimpunctata_586 | 0.196 |
| Scymnus_interruptus_763 | Tytthaspis_sedecimpunctata_586 | 0.196 |
| Scymnus_interruptus_764 | Tytthaspis_sedecimpunctata_586 | 0.196 |
| Scymnus_interruptus_766 | Tytthaspis_sedecimpunctata_586 | 0.196 |
| Olla_vnigrum_530 | Tytthaspis_sedecimpunctata_587 | 0.196 |
| Scymnus_interruptus_767 | Tytthaspis_sedecimpunctata_587 | 0.196 |
| Scymnus_interruptus_759 | Tytthaspis_sedecimpunctata_588 | 0.196 |
| Scymnus_interruptus_769 | Tytthaspis_sedecimpunctata_588 | 0.196 |
| Olla_vnigrum_527 | Tytthaspis_sedecimpunctata_589 | 0.196 |
| Olla_vnigrum_528 | Tytthaspis_sedecimpunctata_589 | 0.196 |
| Scymnus_ferrugatus_723 | Tytthaspis_sedecimpunctata_590 | 0.196 |
| Scymnus_ferrugatus_725 | Tytthaspis_sedecimpunctata_590 | 0.196 |
| Scymnus_ferrugatus_726 | Tytthaspis_sedecimpunctata_590 | 0.196 |
| Scymnus_ferrugatus_727 | Tytthaspis_sedecimpunctata_590 | 0.196 |
| Scymnus_ferrugatus_728 | Tytthaspis_sedecimpunctata_590 | 0.196 |
| Scymnus_ferrugatus_730 | Tytthaspis_sedecimpunctata_590 | 0.196 |
| Scymnus_ferrugatus_731 | Tytthaspis_sedecimpunctata_590 | 0.196 |

Supplementary Table S4 (continued)

| | | |
|---|---|---|
| Scymnus_ferrugatus_732 | Tytthaspis_sedecimpunctata_590 | 0.196 |
| Scymnus_ferrugatus_733 | Tytthaspis_sedecimpunctata_590 | 0.196 |
| Scymnus_ferrugatus_723 | Tytthaspis_sedecimpunctata_592 | 0.196 |
| Scymnus_ferrugatus_725 | Tytthaspis_sedecimpunctata_592 | 0.196 |
| Scymnus_ferrugatus_726 | Tytthaspis_sedecimpunctata_592 | 0.196 |
| Scymnus_ferrugatus_727 | Tytthaspis_sedecimpunctata_592 | 0.196 |
| Scymnus_ferrugatus_728 | Tytthaspis_sedecimpunctata_592 | 0.196 |
| Scymnus_ferrugatus_730 | Tytthaspis_sedecimpunctata_592 | 0.196 |
| Scymnus_ferrugatus_731 | Tytthaspis_sedecimpunctata_592 | 0.196 |
| Scymnus_ferrugatus_732 | Tytthaspis_sedecimpunctata_592 | 0.196 |
| Scymnus_ferrugatus_733 | Tytthaspis_sedecimpunctata_592 | 0.196 |
| Scymnus_ferrugatus_723 | Tytthaspis_sedecimpunctata_594 | 0.196 |
| Scymnus_ferrugatus_725 | Tytthaspis_sedecimpunctata_594 | 0.196 |
| Scymnus_ferrugatus_726 | Tytthaspis_sedecimpunctata_594 | 0.196 |
| Scymnus_ferrugatus_727 | Tytthaspis_sedecimpunctata_594 | 0.196 |
| Scymnus_ferrugatus_728 | Tytthaspis_sedecimpunctata_594 | 0.196 |
| Scymnus_ferrugatus_730 | Tytthaspis_sedecimpunctata_594 | 0.196 |
| Scymnus_ferrugatus_731 | Tytthaspis_sedecimpunctata_594 | 0.196 |
| Scymnus_ferrugatus_732 | Tytthaspis_sedecimpunctata_594 | 0.196 |
| Scymnus_ferrugatus_733 | Tytthaspis_sedecimpunctata_594 | 0.196 |
| Scymnus_ferrugatus_723 | Tytthaspis_sedecimpunctata_595 | 0.196 |
| Scymnus_ferrugatus_725 | Tytthaspis_sedecimpunctata_595 | 0.196 |
| Scymnus_ferrugatus_726 | Tytthaspis_sedecimpunctata_595 | 0.196 |
| Scymnus_ferrugatus_727 | Tytthaspis_sedecimpunctata_595 | 0.196 |
| Scymnus_ferrugatus_728 | Tytthaspis_sedecimpunctata_595 | 0.196 |
| Scymnus_ferrugatus_730 | Tytthaspis_sedecimpunctata_595 | 0.196 |
| Scymnus_ferrugatus_731 | Tytthaspis_sedecimpunctata_595 | 0.196 |
| Scymnus_ferrugatus_732 | Tytthaspis_sedecimpunctata_595 | 0.196 |
| Scymnus_ferrugatus_733 | Tytthaspis_sedecimpunctata_595 | 0.196 |
| Hyperaspis_reppensis_662 | Scymnus_sinuanodulus_828 | 0.196 |
| Hyperaspis_reppensis_663 | Scymnus_sinuanodulus_828 | 0.196 |
| Calvia_quatuordecimguttata_218 | Scymnus_suturalis_832 | 0.196 |
| Clitostethus_arcuatus_639 | Scymnus_suturalis_832 | 0.196 |
| Clitostethus_arcuatus_640 | Scymnus_suturalis_832 | 0.196 |
| Clitostethus_arcuatus_641 | Scymnus_suturalis_832 | 0.196 |
| Clitostethus_arcuatus_643 | Scymnus_suturalis_832 | 0.196 |
| Scymnus_frontalis_742 | Scymnus_suturalis_833 | 0.196 |
| Calvia_quatuordecimguttata_218 | Scymnus_suturalis_835 | 0.196 |
| Clitostethus_arcuatus_639 | Scymnus_suturalis_835 | 0.196 |
| Clitostethus_arcuatus_640 | Scymnus_suturalis_835 | 0.196 |
| Clitostethus_arcuatus_641 | Scymnus_suturalis_835 | 0.196 |
| Clitostethus_arcuatus_643 | Scymnus_suturalis_835 | 0.196 |
| Calvia_quatuordecimguttata_218 | Scymnus_suturalis_836 | 0.196 |
| Clitostethus_arcuatus_639 | Scymnus_suturalis_836 | 0.196 |
| Clitostethus_arcuatus_640 | Scymnus_suturalis_836 | 0.196 |
| Clitostethus_arcuatus_641 | Scymnus_suturalis_836 | 0.196 |
| Clitostethus_arcuatus_643 | Scymnus_suturalis_836 | 0.196 |
| Clitostethus_arcuatus_642 | Scymnus_suturalis_837 | 0.196 |
| Harmonia_axyridis_355 | Scymnus_suturalis_837 | 0.196 |
| Calvia_quatuordecimguttata_218 | Scymnus_suturalis_839 | 0.196 |
| Clitostethus_arcuatus_639 | Scymnus_suturalis_839 | 0.196 |
| Clitostethus_arcuatus_640 | Scymnus_suturalis_839 | 0.196 |
| Clitostethus_arcuatus_641 | Scymnus_suturalis_839 | 0.196 |
| Clitostethus_arcuatus_643 | Scymnus_suturalis_839 | 0.196 |
| Hyperaspis_proba_660 | Stethorus_punctillum_852 | 0.196 |
| Platynaspis_luteorubra_55 | Scymnus_sinuanodulus_831 | 0.196 |

| | | |
|---|---|---|
| Platynaspis_luteorubra_61 | Scymnus_sinuanodulus_831 | 0.196 |
| Calvia_quatuordecimguttata_204 | Scymnus_suturalis_841 | 0.196 |
| Calvia_quatuordecimguttata_208 | Scymnus_suturalis_841 | 0.196 |
| Calvia_quatuordecimguttata_217 | Scymnus_suturalis_841 | 0.196 |
| Calvia_quatuordecimguttata_204 | Scymnus_suturalis_842 | 0.196 |
| Calvia_quatuordecimguttata_208 | Scymnus_suturalis_842 | 0.196 |
| Calvia_quatuordecimguttata_217 | Scymnus_suturalis_842 | 0.196 |
| Calvia_quatuordecimguttata_204 | Scymnus_suturalis_844 | 0.196 |
| Calvia_quatuordecimguttata_208 | Scymnus_suturalis_844 | 0.196 |
| Calvia_quatuordecimguttata_217 | Scymnus_suturalis_844 | 0.196 |
| Calvia_quatuordecimguttata_204 | Scymnus_suturalis_846 | 0.196 |
| Calvia_quatuordecimguttata_208 | Scymnus_suturalis_846 | 0.196 |
| Calvia_quatuordecimguttata_217 | Scymnus_suturalis_846 | 0.196 |
| Calvia_quatuordecimguttata_204 | Scymnus_suturalis_847 | 0.196 |
| Calvia_quatuordecimguttata_208 | Scymnus_suturalis_847 | 0.196 |
| Calvia_quatuordecimguttata_217 | Scymnus_suturalis_847 | 0.196 |
| Calvia_quatuordecimguttata_204 | Scymnus_suturalis_848 | 0.196 |
| Calvia_quatuordecimguttata_208 | Scymnus_suturalis_848 | 0.196 |
| Calvia_quatuordecimguttata_217 | Scymnus_suturalis_848 | 0.196 |
| Hyperaspis_lateralis_657 | Stethorus_punctillum_859 | 0.196 |
| Scymnus_nubilus_798 | Subcoccinella_vigintiquatuorpunctata_623 | 0.196 |
| Scymnus_nubilus_808 | Subcoccinella_vigintiquatuorpunctata_623 | 0.196 |
| Scymnus_ferrugatus_729 | Tytthaspis_sedecimpunctata_577 | 0.196 |
| Scymnus_ferrugatus_729 | Tytthaspis_sedecimpunctata_578 | 0.196 |
| Scymnus_ferrugatus_729 | Tytthaspis_sedecimpunctata_579 | 0.196 |
| Scymnus_ferrugatus_729 | Tytthaspis_sedecimpunctata_581 | 0.196 |
| Scymnus_ferrugatus_729 | Tytthaspis_sedecimpunctata_582 | 0.196 |
| Scymnus_ferrugatus_724 | Tytthaspis_sedecimpunctata_583 | 0.196 |
| Olla_vnigrum_526 | Tytthaspis_sedecimpunctata_586 | 0.196 |
| Olla_vnigrum_527 | Tytthaspis_sedecimpunctata_588 | 0.196 |
| Olla_vnigrum_528 | Tytthaspis_sedecimpunctata_588 | 0.196 |
| Scymnus_ferrugatus_729 | Tytthaspis_sedecimpunctata_590 | 0.196 |
| Scymnus_ferrugatus_729 | Tytthaspis_sedecimpunctata_594 | 0.196 |
| Scymnus_ferrugatus_729 | Tytthaspis_sedecimpunctata_595 | 0.196 |
| Calvia_quatuordecimguttata_204 | Scymnus_suturalis_832 | 0.196 |
| Calvia_quatuordecimguttata_208 | Scymnus_suturalis_832 | 0.196 |
| Calvia_quatuordecimguttata_217 | Scymnus_suturalis_832 | 0.196 |
| Calvia_quatuordecimguttata_204 | Scymnus_suturalis_835 | 0.196 |
| Calvia_quatuordecimguttata_208 | Scymnus_suturalis_835 | 0.196 |
| Calvia_quatuordecimguttata_217 | Scymnus_suturalis_835 | 0.196 |
| Calvia_quatuordecimguttata_204 | Scymnus_suturalis_836 | 0.196 |
| Calvia_quatuordecimguttata_208 | Scymnus_suturalis_836 | 0.196 |
| Calvia_quatuordecimguttata_217 | Scymnus_suturalis_836 | 0.196 |
| Clitostethus_arcuatus_639 | Scymnus_suturalis_838 | 0.196 |
| Clitostethus_arcuatus_640 | Scymnus_suturalis_838 | 0.196 |
| Clitostethus_arcuatus_641 | Scymnus_suturalis_838 | 0.196 |
| Clitostethus_arcuatus_643 | Scymnus_suturalis_838 | 0.196 |
| Calvia_quatuordecimguttata_204 | Scymnus_suturalis_839 | 0.196 |
| Calvia_quatuordecimguttata_208 | Scymnus_suturalis_839 | 0.196 |
| Calvia_quatuordecimguttata_217 | Scymnus_suturalis_839 | 0.196 |
| Scymnus_apetzi_691 | Subcoccinella_vigintiquatuorpunctata_619 | 0.196 |
| Scymnus_nigrinus_787 | Tytthaspis_sedecimpunctata_591 | 0.196 |
| Calvia_quatuordecimguttata_205 | Scymnus_suturalis_843 | 0.196 |
| Calvia_quatuordecimguttata_205 | Scymnus_suturalis_845 | 0.196 |
| Hyperaspis_lateralis_657 | Stethorus_punctillum_855 | 0.196 |
| Scymnus_nubilus_797 | Subcoccinella_vigintiquatuorpunctata_623 | 0.196 |

| | | |
|---|---|---|
| Scymnus_nubilus_798 | Subcoccinella_vigintiquatuorpunctata_626 | 0.196 |
| Scymnus_nubilus_808 | Subcoccinella_vigintiquatuorpunctata_626 | 0.196 |
| Calvia_quatuordecimguttata_209 | Scymnus_rubromaculatus_822 | 0.196 |
| Calvia_quatuordecimguttata_209 | Scymnus_rubromaculatus_823 | 0.196 |
| Calvia_quatuordecimguttata_209 | Scymnus_rubromaculatus_824 | 0.196 |
| Calvia_quatuordecimguttata_209 | Scymnus_rubromaculatus_825 | 0.196 |
| Calvia_quatuordecimguttata_209 | Scymnus_rubromaculatus_826 | 0.196 |
| Calvia_quatuordecimguttata_209 | Scymnus_rubromaculatus_827 | 0.196 |
| Calvia_quatuordecimguttata_205 | Scymnus_suturalis_834 | 0.196 |
| Calvia_quatuordecimguttata_203 | Scymnus_suturalis_841 | 0.196 |
| Calvia_quatuordecimguttata_203 | Scymnus_suturalis_842 | 0.196 |
| Calvia_quatuordecimguttata_203 | Scymnus_suturalis_844 | 0.196 |
| Calvia_quatuordecimguttata_203 | Scymnus_suturalis_846 | 0.196 |
| Calvia_quatuordecimguttata_203 | Scymnus_suturalis_847 | 0.196 |
| Calvia_quatuordecimguttata_203 | Scymnus_suturalis_848 | 0.196 |
| Scymnus_interruptus_767 | Subcoccinella_vigintiquatuorpunctata_624 | 0.196 |
| Scymnus_nubilus_797 | Subcoccinella_vigintiquatuorpunctata_626 | 0.196 |
| Myzia_pullata_517 | Subcoccinella_vigintiquatuorpunctata_627 | 0.196 |
| Scymnus_interruptus_767 | Subcoccinella_vigintiquatuorpunctata_627 | 0.196 |
| Myzia_pullata_517 | Subcoccinella_vigintiquatuorpunctata_630 | 0.196 |
| Scymnus_interruptus_760 | Subcoccinella_vigintiquatuorpunctata_630 | 0.196 |
| Scymnus_interruptus_765 | Subcoccinella_vigintiquatuorpunctata_630 | 0.196 |
| Scymnus_interruptus_768 | Subcoccinella_vigintiquatuorpunctata_630 | 0.196 |
| Scymnus_abietis_682 | Tytthaspis_sedecimpunctata_577 | 0.196 |
| Scymnus_abietis_683 | Tytthaspis_sedecimpunctata_577 | 0.196 |
| Scymnus_abietis_685 | Tytthaspis_sedecimpunctata_577 | 0.196 |
| Scymnus_abietis_682 | Tytthaspis_sedecimpunctata_578 | 0.196 |
| Scymnus_abietis_683 | Tytthaspis_sedecimpunctata_578 | 0.196 |
| Scymnus_abietis_685 | Tytthaspis_sedecimpunctata_578 | 0.196 |
| Scymnus_abietis_682 | Tytthaspis_sedecimpunctata_579 | 0.196 |
| Scymnus_abietis_683 | Tytthaspis_sedecimpunctata_579 | 0.196 |
| Scymnus_abietis_685 | Tytthaspis_sedecimpunctata_579 | 0.196 |
| Scymnus_abietis_682 | Tytthaspis_sedecimpunctata_581 | 0.196 |
| Scymnus_abietis_683 | Tytthaspis_sedecimpunctata_581 | 0.196 |
| Scymnus_abietis_685 | Tytthaspis_sedecimpunctata_581 | 0.196 |
| Scymnus_abietis_682 | Tytthaspis_sedecimpunctata_582 | 0.196 |
| Scymnus_abietis_683 | Tytthaspis_sedecimpunctata_582 | 0.196 |
| Scymnus_abietis_685 | Tytthaspis_sedecimpunctata_582 | 0.196 |
| Scymnus_abietis_684 | Tytthaspis_sedecimpunctata_583 | 0.196 |
| Scymnus_abietis_686 | Tytthaspis_sedecimpunctata_583 | 0.196 |
| Scymnus_abietis_687 | Tytthaspis_sedecimpunctata_583 | 0.196 |
| Scymnus_abietis_688 | Tytthaspis_sedecimpunctata_583 | 0.196 |
| Scymnus_abietis_682 | Tytthaspis_sedecimpunctata_590 | 0.196 |
| Scymnus_abietis_683 | Tytthaspis_sedecimpunctata_590 | 0.196 |
| Scymnus_abietis_685 | Tytthaspis_sedecimpunctata_590 | 0.196 |
| Scymnus_abietis_682 | Tytthaspis_sedecimpunctata_592 | 0.196 |
| Scymnus_abietis_683 | Tytthaspis_sedecimpunctata_592 | 0.196 |
| Scymnus_abietis_685 | Tytthaspis_sedecimpunctata_592 | 0.196 |
| Scymnus_abietis_682 | Tytthaspis_sedecimpunctata_593 | 0.196 |
| Scymnus_abietis_683 | Tytthaspis_sedecimpunctata_593 | 0.196 |
| Scymnus_abietis_685 | Tytthaspis_sedecimpunctata_593 | 0.196 |
| Scymnus_abietis_682 | Tytthaspis_sedecimpunctata_594 | 0.196 |
| Scymnus_abietis_683 | Tytthaspis_sedecimpunctata_594 | 0.196 |
| Scymnus_abietis_685 | Tytthaspis_sedecimpunctata_594 | 0.196 |
| Scymnus_abietis_682 | Tytthaspis_sedecimpunctata_595 | 0.196 |
| Scymnus_abietis_683 | Tytthaspis_sedecimpunctata_595 | 0.196 |

| | | |
|---|---|---|
| Scymnus_abietis_685 | Tytthaspis_sedecimpunctata_595 | 0.196 |
| Hippodamia_parenthesis_433 | Vibidia_duodecimguttata_597 | 0.196 |
| Hippodamia_parenthesis_433 | Vibidia_duodecimguttata_600 | 0.196 |
| Calvia_quatuordecimguttata_211 | Scymnus_rubromaculatus_822 | 0.196 |
| Calvia_quatuordecimguttata_212 | Scymnus_rubromaculatus_822 | 0.196 |
| Calvia_quatuordecimguttata_213 | Scymnus_rubromaculatus_822 | 0.196 |
| Calvia_quatuordecimguttata_214 | Scymnus_rubromaculatus_822 | 0.196 |
| Calvia_quatuordecimguttata_215 | Scymnus_rubromaculatus_822 | 0.196 |
| Calvia_quatuordecimguttata_211 | Scymnus_rubromaculatus_823 | 0.196 |
| Calvia_quatuordecimguttata_212 | Scymnus_rubromaculatus_823 | 0.196 |
| Calvia_quatuordecimguttata_213 | Scymnus_rubromaculatus_823 | 0.196 |
| Calvia_quatuordecimguttata_214 | Scymnus_rubromaculatus_823 | 0.196 |
| Calvia_quatuordecimguttata_215 | Scymnus_rubromaculatus_823 | 0.196 |
| Calvia_quatuordecimguttata_211 | Scymnus_rubromaculatus_824 | 0.196 |
| Calvia_quatuordecimguttata_212 | Scymnus_rubromaculatus_824 | 0.196 |
| Calvia_quatuordecimguttata_213 | Scymnus_rubromaculatus_824 | 0.196 |
| Calvia_quatuordecimguttata_214 | Scymnus_rubromaculatus_824 | 0.196 |
| Calvia_quatuordecimguttata_215 | Scymnus_rubromaculatus_824 | 0.196 |
| Calvia_quatuordecimguttata_211 | Scymnus_rubromaculatus_825 | 0.196 |
| Calvia_quatuordecimguttata_212 | Scymnus_rubromaculatus_825 | 0.196 |
| Calvia_quatuordecimguttata_213 | Scymnus_rubromaculatus_825 | 0.196 |
| Calvia_quatuordecimguttata_214 | Scymnus_rubromaculatus_825 | 0.196 |
| Calvia_quatuordecimguttata_215 | Scymnus_rubromaculatus_825 | 0.196 |
| Calvia_quatuordecimguttata_211 | Scymnus_rubromaculatus_826 | 0.196 |
| Calvia_quatuordecimguttata_212 | Scymnus_rubromaculatus_826 | 0.196 |
| Calvia_quatuordecimguttata_213 | Scymnus_rubromaculatus_826 | 0.196 |
| Calvia_quatuordecimguttata_214 | Scymnus_rubromaculatus_826 | 0.196 |
| Calvia_quatuordecimguttata_215 | Scymnus_rubromaculatus_826 | 0.196 |
| Calvia_quatuordecimguttata_211 | Scymnus_rubromaculatus_827 | 0.196 |
| Calvia_quatuordecimguttata_212 | Scymnus_rubromaculatus_827 | 0.196 |
| Calvia_quatuordecimguttata_213 | Scymnus_rubromaculatus_827 | 0.196 |
| Calvia_quatuordecimguttata_214 | Scymnus_rubromaculatus_827 | 0.196 |
| Calvia_quatuordecimguttata_215 | Scymnus_rubromaculatus_827 | 0.196 |
| Calvia_quatuordecimguttata_203 | Scymnus_suturalis_832 | 0.196 |
| Calvia_quatuordecimguttata_203 | Scymnus_suturalis_835 | 0.196 |
| Calvia_quatuordecimguttata_203 | Scymnus_suturalis_836 | 0.196 |
| Calvia_quatuordecimguttata_203 | Scymnus_suturalis_839 | 0.196 |
| Scymnus_camptodromus_715 | Subcoccinella_vigintiquatuorpunctata_625 | 0.196 |
| Brachiacantha_rotunda_632 | Scymnus_sinuanodulus_831 | 0.196 |
| Brachiacantha_rotunda_633 | Scymnus_sinuanodulus_831 | 0.196 |
| Brachiacantha_rotunda_635 | Scymnus_sinuanodulus_831 | 0.196 |
| Coccidula_scutellata_83 | Subcoccinella_vigintiquatuorpunctata_620 | 0.196 |
| Scymnus_camptodromus_718 | Subcoccinella_vigintiquatuorpunctata_620 | 0.196 |
| Coccidula_scutellata_83 | Subcoccinella_vigintiquatuorpunctata_621 | 0.196 |
| Scymnus_camptodromus_718 | Subcoccinella_vigintiquatuorpunctata_621 | 0.196 |
| Coccidula_scutellata_83 | Subcoccinella_vigintiquatuorpunctata_622 | 0.196 |
| Coccidula_scutellata_80 | Subcoccinella_vigintiquatuorpunctata_624 | 0.196 |
| Scymnus_camptodromus_718 | Subcoccinella_vigintiquatuorpunctata_627 | 0.196 |
| Coccidula_scutellata_83 | Subcoccinella_vigintiquatuorpunctata_628 | 0.196 |
| Scymnus_camptodromus_718 | Subcoccinella_vigintiquatuorpunctata_628 | 0.196 |
| Coccidula_scutellata_83 | Subcoccinella_vigintiquatuorpunctata_629 | 0.196 |
| Scymnus_camptodromus_718 | Subcoccinella_vigintiquatuorpunctata_629 | 0.196 |
| Scymnus_camptodromus_718 | Subcoccinella_vigintiquatuorpunctata_630 | 0.196 |
| Coccidula_scutellata_83 | Subcoccinella_vigintiquatuorpunctata_631 | 0.196 |
| Scymnus_camptodromus_718 | Subcoccinella_vigintiquatuorpunctata_631 | 0.196 |
| Scymnus_abietis_682 | Tytthaspis_sedecimpunctata_580 | 0.196 |

| | | |
|---|---|---|
| Scymnus_abietis_683 | Tytthaspis_sedecimpunctata_580 | 0.196 |
| Scymnus_abietis_685 | Tytthaspis_sedecimpunctata_580 | 0.196 |
| Scymnus_abietis_684 | Tytthaspis_sedecimpunctata_586 | 0.196 |
| Scymnus_abietis_686 | Tytthaspis_sedecimpunctata_586 | 0.196 |
| Scymnus_abietis_687 | Tytthaspis_sedecimpunctata_586 | 0.196 |
| Scymnus_abietis_688 | Tytthaspis_sedecimpunctata_586 | 0.196 |
| Scymnus_abietis_684 | Tytthaspis_sedecimpunctata_587 | 0.196 |
| Scymnus_abietis_686 | Tytthaspis_sedecimpunctata_587 | 0.196 |
| Scymnus_abietis_687 | Tytthaspis_sedecimpunctata_587 | 0.196 |
| Scymnus_abietis_688 | Tytthaspis_sedecimpunctata_587 | 0.196 |
| Scymnus_abietis_684 | Tytthaspis_sedecimpunctata_588 | 0.196 |
| Scymnus_abietis_686 | Tytthaspis_sedecimpunctata_588 | 0.196 |
| Scymnus_abietis_687 | Tytthaspis_sedecimpunctata_588 | 0.196 |
| Scymnus_abietis_688 | Tytthaspis_sedecimpunctata_588 | 0.196 |
| Scymnus_abietis_684 | Tytthaspis_sedecimpunctata_591 | 0.196 |
| Scymnus_abietis_686 | Tytthaspis_sedecimpunctata_591 | 0.196 |
| Scymnus_abietis_687 | Tytthaspis_sedecimpunctata_591 | 0.196 |
| Scymnus_abietis_688 | Tytthaspis_sedecimpunctata_591 | 0.196 |
| Hippodamia_parenthesis_432 | Vibidia_duodecimguttata_598 | 0.196 |
| Hippodamia_parenthesis_434 | Vibidia_duodecimguttata_598 | 0.196 |
| Hippodamia_parenthesis_435 | Vibidia_duodecimguttata_598 | 0.196 |
| Hippodamia_parenthesis_437 | Vibidia_duodecimguttata_598 | 0.196 |
| Hippodamia_parenthesis_438 | Vibidia_duodecimguttata_598 | 0.196 |
| Hippodamia_parenthesis_440 | Vibidia_duodecimguttata_598 | 0.196 |
| Hippodamia_parenthesis_441 | Vibidia_duodecimguttata_598 | 0.196 |
| Hippodamia_parenthesis_442 | Vibidia_duodecimguttata_598 | 0.196 |
| Hippodamia_undecimnotata_460 | Vibidia_duodecimguttata_598 | 0.196 |
| Hippodamia_undecimnotata_461 | Vibidia_duodecimguttata_598 | 0.196 |
| Hippodamia_undecimnotata_462 | Vibidia_duodecimguttata_598 | 0.196 |
| Hippodamia_undecimnotata_463 | Vibidia_duodecimguttata_598 | 0.196 |
| Hippodamia_undecimnotata_464 | Vibidia_duodecimguttata_598 | 0.196 |
| Hippodamia_undecimnotata_465 | Vibidia_duodecimguttata_598 | 0.196 |
| Hippodamia_undecimnotata_466 | Vibidia_duodecimguttata_598 | 0.196 |
| Hippodamia_parenthesis_432 | Vibidia_duodecimguttata_599 | 0.196 |
| Hippodamia_parenthesis_434 | Vibidia_duodecimguttata_599 | 0.196 |
| Hippodamia_parenthesis_435 | Vibidia_duodecimguttata_599 | 0.196 |
| Hippodamia_parenthesis_437 | Vibidia_duodecimguttata_599 | 0.196 |
| Hippodamia_parenthesis_438 | Vibidia_duodecimguttata_599 | 0.196 |
| Hippodamia_parenthesis_440 | Vibidia_duodecimguttata_599 | 0.196 |
| Hippodamia_parenthesis_441 | Vibidia_duodecimguttata_599 | 0.196 |
| Hippodamia_parenthesis_442 | Vibidia_duodecimguttata_599 | 0.196 |
| Hippodamia_parenthesis_432 | Vibidia_duodecimguttata_601 | 0.196 |
| Hippodamia_parenthesis_434 | Vibidia_duodecimguttata_601 | 0.196 |
| Hippodamia_parenthesis_435 | Vibidia_duodecimguttata_601 | 0.196 |
| Hippodamia_parenthesis_437 | Vibidia_duodecimguttata_601 | 0.196 |
| Hippodamia_parenthesis_438 | Vibidia_duodecimguttata_601 | 0.196 |
| Hippodamia_parenthesis_440 | Vibidia_duodecimguttata_601 | 0.196 |
| Hippodamia_parenthesis_441 | Vibidia_duodecimguttata_601 | 0.196 |
| Hippodamia_parenthesis_442 | Vibidia_duodecimguttata_601 | 0.196 |
| Hippodamia_parenthesis_432 | Vibidia_duodecimguttata_602 | 0.196 |
| Hippodamia_parenthesis_434 | Vibidia_duodecimguttata_602 | 0.196 |
| Hippodamia_parenthesis_435 | Vibidia_duodecimguttata_602 | 0.196 |
| Hippodamia_parenthesis_437 | Vibidia_duodecimguttata_602 | 0.196 |
| Hippodamia_parenthesis_438 | Vibidia_duodecimguttata_602 | 0.196 |
| Hippodamia_parenthesis_440 | Vibidia_duodecimguttata_602 | 0.196 |
| Hippodamia_parenthesis_441 | Vibidia_duodecimguttata_602 | 0.196 |

Supplementary Table S4 (continued)

| | | |
|---|---|---|
| Hippodamia_parenthesis_442 | Vibidia_duodecimguttata_602 | 0.196 |
| Hippodamia_undecimnotata_460 | Vibidia_duodecimguttata_603 | 0.196 |
| Hippodamia_undecimnotata_461 | Vibidia_duodecimguttata_603 | 0.196 |
| Hippodamia_undecimnotata_462 | Vibidia_duodecimguttata_603 | 0.196 |
| Hippodamia_undecimnotata_463 | Vibidia_duodecimguttata_603 | 0.196 |
| Hippodamia_undecimnotata_464 | Vibidia_duodecimguttata_603 | 0.196 |
| Hippodamia_undecimnotata_465 | Vibidia_duodecimguttata_603 | 0.196 |
| Hippodamia_undecimnotata_466 | Vibidia_duodecimguttata_603 | 0.196 |
| Coccidula_rufa_62 | Scymnus_rubromaculatus_822 | 0.196 |
| Coccidula_rufa_62 | Scymnus_rubromaculatus_823 | 0.196 |
| Coccidula_rufa_62 | Scymnus_rubromaculatus_824 | 0.196 |
| Coccidula_rufa_62 | Scymnus_rubromaculatus_825 | 0.196 |
| Coccidula_rufa_62 | Scymnus_rubromaculatus_826 | 0.196 |
| Coccidula_rufa_62 | Scymnus_rubromaculatus_827 | 0.196 |
| Hippodamia_convergens_395 | Scymnus_suturalis_838 | 0.196 |
| Hippodamia_convergens_396 | Scymnus_suturalis_838 | 0.196 |
| Hippodamia_convergens_403 | Scymnus_suturalis_838 | 0.196 |
| Hippodamia_moesta_410 | Scymnus_suturalis_838 | 0.196 |
| Hippodamia_convergens_395 | Scymnus_suturalis_840 | 0.196 |
| Hippodamia_convergens_396 | Scymnus_suturalis_840 | 0.196 |
| Hippodamia_convergens_403 | Scymnus_suturalis_840 | 0.196 |
| Hippodamia_moesta_410 | Scymnus_suturalis_840 | 0.196 |
| Myrrha_octodecimguttata_485 | Scymnus_sinuanodulus_829 | 0.196 |
| Myrrha_octodecimguttata_486 | Scymnus_sinuanodulus_829 | 0.196 |
| Myrrha_octodecimguttata_487 | Scymnus_sinuanodulus_829 | 0.196 |
| Myrrha_octodecimguttata_490 | Scymnus_sinuanodulus_829 | 0.196 |
| Myrrha_octodecimguttata_494 | Scymnus_sinuanodulus_829 | 0.196 |
| Myrrha_octodecimguttata_496 | Scymnus_sinuanodulus_829 | 0.196 |
| Myrrha_octodecimguttata_497 | Scymnus_sinuanodulus_829 | 0.196 |
| Myrrha_octodecimguttata_501 | Scymnus_sinuanodulus_829 | 0.196 |
| Myrrha_octodecimguttata_502 | Scymnus_sinuanodulus_829 | 0.196 |
| Hippodamia_convergens_393 | Scymnus_suturalis_841 | 0.196 |
| Hippodamia_convergens_394 | Scymnus_suturalis_841 | 0.196 |
| Hippodamia_convergens_397 | Scymnus_suturalis_841 | 0.196 |
| Hippodamia_convergens_398 | Scymnus_suturalis_841 | 0.196 |
| Hippodamia_convergens_401 | Scymnus_suturalis_841 | 0.196 |
| Hippodamia_convergens_402 | Scymnus_suturalis_841 | 0.196 |
| Hippodamia_convergens_393 | Scymnus_suturalis_842 | 0.196 |
| Hippodamia_convergens_394 | Scymnus_suturalis_842 | 0.196 |
| Hippodamia_convergens_397 | Scymnus_suturalis_842 | 0.196 |
| Hippodamia_convergens_398 | Scymnus_suturalis_842 | 0.196 |
| Hippodamia_convergens_401 | Scymnus_suturalis_842 | 0.196 |
| Hippodamia_convergens_402 | Scymnus_suturalis_842 | 0.196 |
| Hippodamia_convergens_399 | Scymnus_suturalis_843 | 0.196 |
| Hippodamia_convergens_404 | Scymnus_suturalis_843 | 0.196 |
| Hippodamia_convergens_405 | Scymnus_suturalis_843 | 0.196 |
| Hippodamia_convergens_406 | Scymnus_suturalis_843 | 0.196 |
| Hippodamia_quinquesignata_443 | Scymnus_suturalis_843 | 0.196 |
| Hippodamia_quinquesignata_444 | Scymnus_suturalis_843 | 0.196 |
| Hippodamia_quinquesignata_445 | Scymnus_suturalis_843 | 0.196 |
| Hippodamia_convergens_393 | Scymnus_suturalis_844 | 0.196 |
| Hippodamia_convergens_394 | Scymnus_suturalis_844 | 0.196 |
| Hippodamia_convergens_397 | Scymnus_suturalis_844 | 0.196 |
| Hippodamia_convergens_398 | Scymnus_suturalis_844 | 0.196 |
| Hippodamia_convergens_401 | Scymnus_suturalis_844 | 0.196 |
| Hippodamia_convergens_402 | Scymnus_suturalis_844 | 0.196 |

| | | |
|---|---|---|
| Hippodamia_convergens_399 | Scymnus_suturalis_845 | 0.196 |
| Hippodamia_convergens_404 | Scymnus_suturalis_845 | 0.196 |
| Hippodamia_convergens_405 | Scymnus_suturalis_845 | 0.196 |
| Hippodamia_convergens_406 | Scymnus_suturalis_845 | 0.196 |
| Hippodamia_quinquesignata_443 | Scymnus_suturalis_845 | 0.196 |
| Hippodamia_quinquesignata_444 | Scymnus_suturalis_845 | 0.196 |
| Hippodamia_quinquesignata_445 | Scymnus_suturalis_845 | 0.196 |
| Hippodamia_convergens_393 | Scymnus_suturalis_846 | 0.196 |
| Hippodamia_convergens_394 | Scymnus_suturalis_846 | 0.196 |
| Hippodamia_convergens_397 | Scymnus_suturalis_846 | 0.196 |
| Hippodamia_convergens_398 | Scymnus_suturalis_846 | 0.196 |
| Hippodamia_convergens_401 | Scymnus_suturalis_846 | 0.196 |
| Hippodamia_convergens_402 | Scymnus_suturalis_846 | 0.196 |
| Hippodamia_convergens_393 | Scymnus_suturalis_847 | 0.196 |
| Hippodamia_convergens_394 | Scymnus_suturalis_847 | 0.196 |
| Hippodamia_convergens_397 | Scymnus_suturalis_847 | 0.196 |
| Hippodamia_convergens_398 | Scymnus_suturalis_847 | 0.196 |
| Hippodamia_convergens_401 | Scymnus_suturalis_847 | 0.196 |
| Hippodamia_convergens_402 | Scymnus_suturalis_847 | 0.196 |
| Hippodamia_convergens_393 | Scymnus_suturalis_848 | 0.196 |
| Hippodamia_convergens_394 | Scymnus_suturalis_848 | 0.196 |
| Hippodamia_convergens_397 | Scymnus_suturalis_848 | 0.196 |
| Hippodamia_convergens_398 | Scymnus_suturalis_848 | 0.196 |
| Hippodamia_convergens_401 | Scymnus_suturalis_848 | 0.196 |
| Hippodamia_convergens_402 | Scymnus_suturalis_848 | 0.196 |
| Scymnus_mitior_779 | Subcoccinella_vigintiquatuorpunctata_622 | 0.196 |
| Scymnus_mitior_779 | Subcoccinella_vigintiquatuorpunctata_624 | 0.196 |
| Scymnus_mitior_780 | Subcoccinella_vigintiquatuorpunctata_624 | 0.196 |
| Scymnus_mitior_779 | Subcoccinella_vigintiquatuorpunctata_626 | 0.196 |
| Scymnus_mitior_780 | Subcoccinella_vigintiquatuorpunctata_626 | 0.196 |
| Adalia_bipunctata_98 | Vibidia_duodecimguttata_596 | 0.196 |
| Adalia_bipunctata_98 | Vibidia_duodecimguttata_598 | 0.196 |
| Adalia_bipunctata_98 | Vibidia_duodecimguttata_603 | 0.196 |
| Hippodamia_parenthesis_439 | Vibidia_duodecimguttata_603 | 0.196 |
| Calvia_quatuordecimguttata_203 | Scymnus_sinuanodulus_828 | 0.196 |
| Hippodamia_convergens_393 | Scymnus_suturalis_832 | 0.196 |
| Hippodamia_convergens_394 | Scymnus_suturalis_832 | 0.196 |
| Hippodamia_convergens_397 | Scymnus_suturalis_832 | 0.196 |
| Hippodamia_convergens_398 | Scymnus_suturalis_832 | 0.196 |
| Hippodamia_convergens_401 | Scymnus_suturalis_832 | 0.196 |
| Hippodamia_convergens_402 | Scymnus_suturalis_832 | 0.196 |
| Hippodamia_convergens_395 | Scymnus_suturalis_834 | 0.196 |
| Hippodamia_convergens_396 | Scymnus_suturalis_834 | 0.196 |
| Hippodamia_convergens_403 | Scymnus_suturalis_834 | 0.196 |
| Hippodamia_moesta_410 | Scymnus_suturalis_834 | 0.196 |
| Hippodamia_convergens_393 | Scymnus_suturalis_835 | 0.196 |
| Hippodamia_convergens_394 | Scymnus_suturalis_835 | 0.196 |
| Hippodamia_convergens_397 | Scymnus_suturalis_835 | 0.196 |
| Hippodamia_convergens_398 | Scymnus_suturalis_835 | 0.196 |
| Hippodamia_convergens_401 | Scymnus_suturalis_835 | 0.196 |
| Hippodamia_convergens_402 | Scymnus_suturalis_835 | 0.196 |
| Hippodamia_convergens_393 | Scymnus_suturalis_836 | 0.196 |
| Hippodamia_convergens_394 | Scymnus_suturalis_836 | 0.196 |
| Hippodamia_convergens_397 | Scymnus_suturalis_836 | 0.196 |
| Hippodamia_convergens_398 | Scymnus_suturalis_836 | 0.196 |
| Hippodamia_convergens_401 | Scymnus_suturalis_836 | 0.196 |

| | | |
|---|---|---|
| Hippodamia_convergens_402 | Scymnus_suturalis_836 | 0.196 |
| Hippodamia_convergens_395 | Scymnus_suturalis_837 | 0.196 |
| Hippodamia_convergens_396 | Scymnus_suturalis_837 | 0.196 |
| Hippodamia_convergens_403 | Scymnus_suturalis_837 | 0.196 |
| Hippodamia_moesta_410 | Scymnus_suturalis_837 | 0.196 |
| Hippodamia_convergens_393 | Scymnus_suturalis_839 | 0.196 |
| Hippodamia_convergens_394 | Scymnus_suturalis_839 | 0.196 |
| Hippodamia_convergens_397 | Scymnus_suturalis_839 | 0.196 |
| Hippodamia_convergens_398 | Scymnus_suturalis_839 | 0.196 |
| Hippodamia_convergens_401 | Scymnus_suturalis_839 | 0.196 |
| Hippodamia_convergens_402 | Scymnus_suturalis_839 | 0.196 |
| Scymnus_nubilus_798 | Subcoccinella_vigintiquatuorpunctata_619 | 0.196 |
| Scymnus_nubilus_808 | Subcoccinella_vigintiquatuorpunctata_619 | 0.196 |
| Chilocorus_renipustulatus_39 | Scymnus_sinuanodulus_831 | 0.196 |
| Myrrha_octodecimguttata_488 | Scymnus_sinuanodulus_829 | 0.196 |
| Myrrha_octodecimguttata_491 | Scymnus_sinuanodulus_829 | 0.196 |
| Myrrha_octodecimguttata_492 | Scymnus_sinuanodulus_829 | 0.196 |
| Psyllobora_renifer_555 | Vibidia_duodecimguttata_597 | 0.196 |
| Psyllobora_renifer_555 | Vibidia_duodecimguttata_600 | 0.196 |
| Scymnus_nubilus_797 | Subcoccinella_vigintiquatuorpunctata_619 | 0.196 |
| Nephus_quadrimaculatus_669 | Scymnus_sinuanodulus_831 | 0.196 |
| Nephus_quadrimaculatus_670 | Scymnus_sinuanodulus_831 | 0.196 |
| Nephus_quadrimaculatus_671 | Scymnus_sinuanodulus_831 | 0.196 |
| Nephus_quadrimaculatus_672 | Scymnus_sinuanodulus_831 | 0.196 |
| Nephus_quadrimaculatus_673 | Scymnus_sinuanodulus_831 | 0.196 |
| Aphidecta_obliterata_178 | Vibidia_duodecimguttata_599 | 0.196 |
| Aphidecta_obliterata_178 | Vibidia_duodecimguttata_601 | 0.196 |
| Aphidecta_obliterata_178 | Vibidia_duodecimguttata_602 | 0.196 |
| Adalia_bipunctata_105 | Vibidia_duodecimguttata_603 | 0.196 |
| Adalia_bipunctata_99 | Vibidia_duodecimguttata_603 | 0.196 |
| Mulsantina_hudsonica_482 | Vibidia_duodecimguttata_603 | 0.196 |
| Coccidula_scutellata_84 | Subcoccinella_vigintiquatuorpunctata_623 | 0.197 |
| Scymnus_abietis_689 | Tytthaspis_sedecimpunctata_577 | 0.197 |
| Scymnus_abietis_689 | Tytthaspis_sedecimpunctata_578 | 0.197 |
| Scymnus_abietis_689 | Tytthaspis_sedecimpunctata_579 | 0.197 |
| Scymnus_abietis_689 | Tytthaspis_sedecimpunctata_581 | 0.197 |
| Scymnus_abietis_689 | Tytthaspis_sedecimpunctata_582 | 0.197 |
| Scymnus_abietis_689 | Tytthaspis_sedecimpunctata_590 | 0.197 |
| Scymnus_abietis_689 | Tytthaspis_sedecimpunctata_592 | 0.197 |
| Scymnus_abietis_689 | Tytthaspis_sedecimpunctata_593 | 0.197 |
| Scymnus_abietis_689 | Tytthaspis_sedecimpunctata_594 | 0.197 |
| Scymnus_abietis_689 | Tytthaspis_sedecimpunctata_595 | 0.197 |
| Hippodamia_parenthesis_436 | Vibidia_duodecimguttata_598 | 0.197 |
| Hippodamia_parenthesis_436 | Vibidia_duodecimguttata_599 | 0.197 |
| Hippodamia_parenthesis_436 | Vibidia_duodecimguttata_601 | 0.197 |
| Hippodamia_parenthesis_436 | Vibidia_duodecimguttata_602 | 0.197 |
| Platynaspis_luteorubra_55 | Scymnus_sinuanodulus_829 | 0.197 |
| Platynaspis_luteorubra_61 | Scymnus_sinuanodulus_829 | 0.197 |
| Scymnus_abietis_685 | Subcoccinella_vigintiquatuorpunctata_622 | 0.197 |
| Chilocorus_nigritus_27 | Vibidia_duodecimguttata_596 | 0.197 |
| Harmonia_quadripunctata_382 | Vibidia_duodecimguttata_596 | 0.197 |
| Harmonia_quadripunctata_382 | Vibidia_duodecimguttata_598 | 0.197 |
| Chilocorus_nigritus_27 | Vibidia_duodecimguttata_599 | 0.197 |
| Harmonia_quadripunctata_377 | Vibidia_duodecimguttata_599 | 0.197 |
| Harmonia_quadripunctata_379 | Vibidia_duodecimguttata_599 | 0.197 |
| Harmonia_quadripunctata_381 | Vibidia_duodecimguttata_599 | 0.197 |

| | | |
|---|---|---|
| Harmonia_quadripunctata_377 | Vibidia_duodecimguttata_601 | 0.197 |
| Harmonia_quadripunctata_379 | Vibidia_duodecimguttata_601 | 0.197 |
| Harmonia_quadripunctata_381 | Vibidia_duodecimguttata_601 | 0.197 |
| Chilocorus_nigritus_27 | Vibidia_duodecimguttata_602 | 0.197 |
| Harmonia_quadripunctata_377 | Vibidia_duodecimguttata_602 | 0.197 |
| Harmonia_quadripunctata_379 | Vibidia_duodecimguttata_602 | 0.197 |
| Harmonia_quadripunctata_381 | Vibidia_duodecimguttata_602 | 0.197 |
| Chilocorus_nigritus_27 | Vibidia_duodecimguttata_603 | 0.197 |
| Oenopia_conglobata_522 | Scymnus_suturalis_838 | 0.197 |
| Psyllobora_renifer_555 | Vibidia_duodecimguttata_603 | 0.197 |
| Coccidula_scutellata_84 | Subcoccinella_vigintiquatuorpunctata_626 | 0.197 |
| Clitostethus_arcuatus_642 | Scymnus_sinuanodulus_831 | 0.197 |
| Scymnus_camptodromus_711 | Subcoccinella_vigintiquatuorpunctata_623 | 0.197 |
| Oenopia_conglobata_523 | Scymnus_suturalis_843 | 0.197 |
| Oenopia_conglobata_522 | Scymnus_suturalis_845 | 0.197 |
| Chilocorus_nigritus_27 | Vibidia_duodecimguttata_597 | 0.197 |
| Harmonia_quadripunctata_377 | Vibidia_duodecimguttata_597 | 0.197 |
| Harmonia_quadripunctata_379 | Vibidia_duodecimguttata_597 | 0.197 |
| Harmonia_quadripunctata_381 | Vibidia_duodecimguttata_597 | 0.197 |
| Chilocorus_nigritus_27 | Vibidia_duodecimguttata_600 | 0.197 |
| Harmonia_quadripunctata_377 | Vibidia_duodecimguttata_600 | 0.197 |
| Harmonia_quadripunctata_379 | Vibidia_duodecimguttata_600 | 0.197 |
| Harmonia_quadripunctata_381 | Vibidia_duodecimguttata_600 | 0.197 |
| Oenopia_conglobata_522 | Scymnus_suturalis_834 | 0.197 |
| Anisosticta_bitriangularis_147 | Scymnus_sinuanodulus_830 | 0.197 |
| Scymnus_camptodromus_711 | Subcoccinella_vigintiquatuorpunctata_622 | 0.197 |
| Scymnus_camptodromus_711 | Subcoccinella_vigintiquatuorpunctata_626 | 0.197 |
| Scymnus_apetzi_694 | Subcoccinella_vigintiquatuorpunctata_625 | 0.197 |
| Brachiacantha_rotunda_636 | Subcoccinella_vigintiquatuorpunctata_620 | 0.197 |
| Brachiacantha_rotunda_636 | Subcoccinella_vigintiquatuorpunctata_621 | 0.197 |
| Brachiacantha_rotunda_634 | Subcoccinella_vigintiquatuorpunctata_627 | 0.197 |
| Brachiacantha_rotunda_636 | Subcoccinella_vigintiquatuorpunctata_628 | 0.197 |
| Brachiacantha_rotunda_636 | Subcoccinella_vigintiquatuorpunctata_629 | 0.197 |
| Brachiacantha_rotunda_636 | Subcoccinella_vigintiquatuorpunctata_631 | 0.197 |
| Propylaea_quatuordecimpunctata_533 | Vibidia_duodecimguttata_596 | 0.197 |
| Propylaea_quatuordecimpunctata_534 | Vibidia_duodecimguttata_596 | 0.197 |
| Propylaea_quatuordecimpunctata_535 | Vibidia_duodecimguttata_596 | 0.197 |
| Propylaea_quatuordecimpunctata_536 | Vibidia_duodecimguttata_596 | 0.197 |
| Propylaea_quatuordecimpunctata_538 | Vibidia_duodecimguttata_596 | 0.197 |
| Propylaea_quatuordecimpunctata_541 | Vibidia_duodecimguttata_596 | 0.197 |
| Propylaea_quatuordecimpunctata_544 | Vibidia_duodecimguttata_596 | 0.197 |
| Propylaea_quatuordecimpunctata_549 | Vibidia_duodecimguttata_596 | 0.197 |
| Coccinella_trifasciata_272 | Vibidia_duodecimguttata_597 | 0.197 |
| Coccinella_trifasciata_276 | Vibidia_duodecimguttata_597 | 0.197 |
| Coccinella_trifasciata_278 | Vibidia_duodecimguttata_597 | 0.197 |
| Coccinella_trifasciata_279 | Vibidia_duodecimguttata_597 | 0.197 |
| Coccinella_trifasciata_272 | Vibidia_duodecimguttata_600 | 0.197 |
| Coccinella_trifasciata_276 | Vibidia_duodecimguttata_600 | 0.197 |
| Coccinella_trifasciata_278 | Vibidia_duodecimguttata_600 | 0.197 |
| Coccinella_trifasciata_279 | Vibidia_duodecimguttata_600 | 0.197 |
| Oenopia_conglobata_523 | Scymnus_suturalis_840 | 0.197 |
| Chilocorus_nigritus_28 | Vibidia_duodecimguttata_596 | 0.197 |
| Chilocorus_nigritus_28 | Vibidia_duodecimguttata_599 | 0.197 |
| Chilocorus_nigritus_28 | Vibidia_duodecimguttata_602 | 0.197 |
| Chilocorus_nigritus_28 | Vibidia_duodecimguttata_603 | 0.197 |
| Scymnus_nubilus_798 | Scymnus_sinuanodulus_831 | 0.197 |

| | | |
|---|---|---|
| Scymnus_nubilus_800 | Scymnus_sinuanodulus_831 | 0.197 |
| Scymnus_nubilus_801 | Scymnus_sinuanodulus_831 | 0.197 |
| Scymnus_nubilus_802 | Scymnus_sinuanodulus_831 | 0.197 |
| Scymnus_nubilus_805 | Scymnus_sinuanodulus_831 | 0.197 |
| Scymnus_nubilus_806 | Scymnus_sinuanodulus_831 | 0.197 |
| Scymnus_nubilus_807 | Scymnus_sinuanodulus_831 | 0.197 |
| Scymnus_nubilus_810 | Scymnus_sinuanodulus_831 | 0.197 |
| Oenopia_conglobata_524 | Scymnus_suturalis_833 | 0.197 |
| Chilocorus_renipustulatus_39 | Scymnus_sinuanodulus_829 | 0.197 |
| Brachiacantha_rotunda_636 | Subcoccinella_vigintiquatuorpunctata_623 | 0.197 |
| Coccinella_hieroglyphica_227 | Vibidia_duodecimguttata_596 | 0.197 |
| Neoharmonia_venusta_520 | Vibidia_duodecimguttata_596 | 0.197 |
| Neoharmonia_venusta_520 | Vibidia_duodecimguttata_598 | 0.197 |
| Coccinella_trifasciata_272 | Vibidia_duodecimguttata_599 | 0.197 |
| Coccinella_trifasciata_276 | Vibidia_duodecimguttata_599 | 0.197 |
| Coccinella_trifasciata_278 | Vibidia_duodecimguttata_599 | 0.197 |
| Coccinella_trifasciata_279 | Vibidia_duodecimguttata_599 | 0.197 |
| Neoharmonia_venusta_520 | Vibidia_duodecimguttata_599 | 0.197 |
| Coccinella_trifasciata_272 | Vibidia_duodecimguttata_601 | 0.197 |
| Coccinella_trifasciata_276 | Vibidia_duodecimguttata_601 | 0.197 |
| Coccinella_trifasciata_278 | Vibidia_duodecimguttata_601 | 0.197 |
| Coccinella_trifasciata_279 | Vibidia_duodecimguttata_601 | 0.197 |
| Coccinella_trifasciata_272 | Vibidia_duodecimguttata_602 | 0.197 |
| Coccinella_trifasciata_276 | Vibidia_duodecimguttata_602 | 0.197 |
| Coccinella_trifasciata_278 | Vibidia_duodecimguttata_602 | 0.197 |
| Coccinella_trifasciata_279 | Vibidia_duodecimguttata_602 | 0.197 |
| Neoharmonia_venusta_520 | Vibidia_duodecimguttata_602 | 0.197 |
| Coccinella_trifasciata_275 | Vibidia_duodecimguttata_603 | 0.197 |
| Coccinella_trifasciata_277 | Vibidia_duodecimguttata_603 | 0.197 |
| Coccinella_trifasciata_280 | Vibidia_duodecimguttata_603 | 0.197 |
| Coccinella_trifasciata_281 | Vibidia_duodecimguttata_603 | 0.197 |
| Platynaspis_luteorubra_50 | Stethorus_punctillum_855 | 0.197 |
| Scymnus_camptodromus_717 | Subcoccinella_vigintiquatuorpunctata_625 | 0.197 |
| Hyperaspis_reppensis_662 | Tytthaspis_sedecimpunctata_584 | 0.197 |
| Hyperaspis_reppensis_663 | Tytthaspis_sedecimpunctata_584 | 0.197 |
| Hyperaspis_lateralis_655 | Tytthaspis_sedecimpunctata_583 | 0.197 |
| Hyperaspis_lateralis_655 | Tytthaspis_sedecimpunctata_585 | 0.197 |
| Chilocorus_bipustulatus_23 | Scymnus_suturalis_841 | 0.197 |
| Chilocorus_bipustulatus_23 | Scymnus_suturalis_842 | 0.197 |
| Chilocorus_bipustulatus_23 | Scymnus_suturalis_844 | 0.197 |
| Chilocorus_bipustulatus_23 | Scymnus_suturalis_846 | 0.197 |
| Chilocorus_bipustulatus_23 | Scymnus_suturalis_847 | 0.197 |
| Chilocorus_bipustulatus_23 | Scymnus_suturalis_848 | 0.197 |
| Hyperaspis_reppensis_662 | Tytthaspis_sedecimpunctata_593 | 0.197 |
| Hyperaspis_reppensis_663 | Tytthaspis_sedecimpunctata_593 | 0.197 |
| Chilocorus_bipustulatus_23 | Scymnus_suturalis_832 | 0.197 |
| Chilocorus_bipustulatus_23 | Scymnus_suturalis_835 | 0.197 |
| Chilocorus_bipustulatus_23 | Scymnus_suturalis_836 | 0.197 |
| Chilocorus_bipustulatus_23 | Scymnus_suturalis_837 | 0.197 |
| Chilocorus_bipustulatus_18 | Scymnus_suturalis_838 | 0.197 |
| Chilocorus_bipustulatus_20 | Scymnus_suturalis_838 | 0.197 |
| Chilocorus_bipustulatus_24 | Scymnus_suturalis_838 | 0.197 |
| Chilocorus_bipustulatus_23 | Scymnus_suturalis_839 | 0.197 |
| Chilocorus_bipustulatus_18 | Scymnus_suturalis_840 | 0.197 |
| Chilocorus_bipustulatus_20 | Scymnus_suturalis_840 | 0.197 |
| Chilocorus_bipustulatus_24 | Scymnus_suturalis_840 | 0.197 |

| | | |
|---|---|---|
| Hyperaspis_lateralis_654 | Tytthaspis_sedecimpunctata_577 | 0.197 |
| Hyperaspis_lateralis_657 | Tytthaspis_sedecimpunctata_577 | 0.197 |
| Hyperaspis_lateralis_654 | Tytthaspis_sedecimpunctata_578 | 0.197 |
| Hyperaspis_lateralis_657 | Tytthaspis_sedecimpunctata_578 | 0.197 |
| Hyperaspis_lateralis_654 | Tytthaspis_sedecimpunctata_579 | 0.197 |
| Hyperaspis_lateralis_657 | Tytthaspis_sedecimpunctata_579 | 0.197 |
| Hyperaspis_lateralis_654 | Tytthaspis_sedecimpunctata_581 | 0.197 |
| Hyperaspis_lateralis_657 | Tytthaspis_sedecimpunctata_581 | 0.197 |
| Hyperaspis_lateralis_654 | Tytthaspis_sedecimpunctata_582 | 0.197 |
| Hyperaspis_lateralis_657 | Tytthaspis_sedecimpunctata_582 | 0.197 |
| Hyperaspis_lateralis_654 | Tytthaspis_sedecimpunctata_590 | 0.197 |
| Hyperaspis_lateralis_657 | Tytthaspis_sedecimpunctata_590 | 0.197 |
| Hyperaspis_lateralis_654 | Tytthaspis_sedecimpunctata_592 | 0.197 |
| Hyperaspis_lateralis_657 | Tytthaspis_sedecimpunctata_592 | 0.197 |
| Hyperaspis_lateralis_654 | Tytthaspis_sedecimpunctata_593 | 0.197 |
| Hyperaspis_lateralis_657 | Tytthaspis_sedecimpunctata_593 | 0.197 |
| Hyperaspis_lateralis_654 | Tytthaspis_sedecimpunctata_594 | 0.197 |
| Hyperaspis_lateralis_657 | Tytthaspis_sedecimpunctata_594 | 0.197 |
| Hyperaspis_lateralis_654 | Tytthaspis_sedecimpunctata_595 | 0.197 |
| Hyperaspis_lateralis_657 | Tytthaspis_sedecimpunctata_595 | 0.197 |
| Chilocorus_bipustulatus_19 | Scymnus_suturalis_843 | 0.197 |
| Chilocorus_bipustulatus_22 | Scymnus_suturalis_843 | 0.197 |
| Chilocorus_bipustulatus_19 | Scymnus_suturalis_845 | 0.197 |
| Chilocorus_bipustulatus_22 | Scymnus_suturalis_845 | 0.197 |
| Hyperaspis_reppensis_662 | Tytthaspis_sedecimpunctata_587 | 0.197 |
| Hyperaspis_reppensis_663 | Tytthaspis_sedecimpunctata_587 | 0.197 |
| Chilocorus_bipustulatus_18 | Scymnus_suturalis_834 | 0.197 |
| Chilocorus_bipustulatus_20 | Scymnus_suturalis_834 | 0.197 |
| Chilocorus_bipustulatus_24 | Scymnus_suturalis_834 | 0.197 |
| Hyperaspis_lateralis_656 | Tytthaspis_sedecimpunctata_586 | 0.197 |
| Hyperaspis_lateralis_656 | Tytthaspis_sedecimpunctata_591 | 0.197 |
| Oenopia_conglobata_524 | Scymnus_suturalis_841 | 0.197 |
| Oenopia_conglobata_524 | Scymnus_suturalis_842 | 0.197 |
| Oenopia_conglobata_524 | Scymnus_suturalis_844 | 0.197 |
| Oenopia_conglobata_524 | Scymnus_suturalis_846 | 0.197 |
| Oenopia_conglobata_524 | Scymnus_suturalis_847 | 0.197 |
| Oenopia_conglobata_524 | Scymnus_suturalis_848 | 0.197 |
| Chilocorus_nigritus_28 | Vibidia_duodecimguttata_597 | 0.197 |
| Chilocorus_nigritus_28 | Vibidia_duodecimguttata_600 | 0.197 |
| Oenopia_conglobata_524 | Scymnus_suturalis_832 | 0.197 |
| Oenopia_conglobata_524 | Scymnus_suturalis_835 | 0.197 |
| Oenopia_conglobata_524 | Scymnus_suturalis_836 | 0.197 |
| Oenopia_conglobata_524 | Scymnus_suturalis_837 | 0.197 |
| Oenopia_conglobata_524 | Scymnus_suturalis_839 | 0.197 |
| Chilocorus_bipustulatus_19 | Scymnus_suturalis_833 | 0.197 |
| Chilocorus_bipustulatus_22 | Scymnus_suturalis_833 | 0.197 |
| Platynaspis_luteorubra_51 | Stethorus_punctillum_857 | 0.197 |
| Platynaspis_luteorubra_53 | Stethorus_punctillum_857 | 0.197 |
| Platynaspis_luteorubra_58 | Stethorus_punctillum_857 | 0.197 |
| Hyperaspis_oregona_659 | Tytthaspis_sedecimpunctata_591 | 0.197 |
| Scymnus_camptodromus_711 | Subcoccinella_vigintiquatuorpunctata_619 | 0.197 |
| Coccinella_quinquepunctata_232 | Vibidia_duodecimguttata_596 | 0.197 |
| Coccinella_trifasciata_274 | Vibidia_duodecimguttata_603 | 0.197 |
| Platynaspis_luteorubra_51 | Stethorus_punctillum_859 | 0.197 |
| Platynaspis_luteorubra_53 | Stethorus_punctillum_859 | 0.197 |
| Platynaspis_luteorubra_58 | Stethorus_punctillum_859 | 0.197 |

| | | |
|---|---|---|
| Scymnus_mimulus_776 | Tytthaspis_sedecimpunctata_588 | 0.197 |
| Platynaspis_luteorubra_51 | Stethorus_punctillum_853 | 0.197 |
| Platynaspis_luteorubra_53 | Stethorus_punctillum_853 | 0.197 |
| Platynaspis_luteorubra_58 | Stethorus_punctillum_853 | 0.197 |
| Scymnus_mimulus_778 | Tytthaspis_sedecimpunctata_586 | 0.197 |
| Scymnus_mimulus_776 | Tytthaspis_sedecimpunctata_589 | 0.197 |
| Myrrha_octodecimguttata_499 | Scymnus_sinuanodulus_828 | 0.197 |
| Cynegetis_impunctata_604 | Scymnus_suturalis_833 | 0.197 |
| Cynegetis_impunctata_608 | Scymnus_suturalis_833 | 0.197 |
| Cynegetis_impunctata_609 | Scymnus_suturalis_833 | 0.197 |
| Cynegetis_impunctata_610 | Scymnus_suturalis_833 | 0.197 |
| Cynegetis_impunctata_611 | Scymnus_suturalis_833 | 0.197 |
| Cynegetis_impunctata_612 | Scymnus_suturalis_833 | 0.197 |
| Brachiacantha_rotunda_634 | Subcoccinella_vigintiquatuorpunctata_619 | 0.197 |
| Chilocorus_renipustulatus_34 | Scymnus_suturalis_841 | 0.197 |
| Chilocorus_renipustulatus_34 | Scymnus_suturalis_842 | 0.197 |
| Chilocorus_renipustulatus_34 | Scymnus_suturalis_844 | 0.197 |
| Cynegetis_impunctata_604 | Scymnus_suturalis_845 | 0.197 |
| Cynegetis_impunctata_608 | Scymnus_suturalis_845 | 0.197 |
| Cynegetis_impunctata_609 | Scymnus_suturalis_845 | 0.197 |
| Cynegetis_impunctata_610 | Scymnus_suturalis_845 | 0.197 |
| Cynegetis_impunctata_611 | Scymnus_suturalis_845 | 0.197 |
| Cynegetis_impunctata_612 | Scymnus_suturalis_845 | 0.197 |
| Chilocorus_renipustulatus_34 | Scymnus_suturalis_846 | 0.197 |
| Chilocorus_renipustulatus_34 | Scymnus_suturalis_847 | 0.197 |
| Chilocorus_renipustulatus_34 | Scymnus_suturalis_848 | 0.197 |
| Scymnus_loewii_771 | Subcoccinella_vigintiquatuorpunctata_623 | 0.197 |
| Scymnus_loewii_774 | Subcoccinella_vigintiquatuorpunctata_630 | 0.197 |
| Scymnus_mimulus_778 | Tytthaspis_sedecimpunctata_589 | 0.197 |
| Platynaspis_luteorubra_51 | Scymnus_sinuanodulus_830 | 0.197 |
| Platynaspis_luteorubra_53 | Scymnus_sinuanodulus_830 | 0.197 |
| Platynaspis_luteorubra_58 | Scymnus_sinuanodulus_830 | 0.197 |
| Chilocorus_renipustulatus_34 | Scymnus_suturalis_832 | 0.197 |
| Myzia_oblongoguttata_504 | Scymnus_suturalis_833 | 0.197 |
| Myzia_oblongoguttata_505 | Scymnus_suturalis_833 | 0.197 |
| Myzia_oblongoguttata_507 | Scymnus_suturalis_833 | 0.197 |
| Myzia_oblongoguttata_508 | Scymnus_suturalis_833 | 0.197 |
| Myzia_oblongoguttata_509 | Scymnus_suturalis_833 | 0.197 |
| Myzia_oblongoguttata_510 | Scymnus_suturalis_833 | 0.197 |
| Myzia_oblongoguttata_511 | Scymnus_suturalis_833 | 0.197 |
| Myzia_oblongoguttata_512 | Scymnus_suturalis_833 | 0.197 |
| Myzia_oblongoguttata_513 | Scymnus_suturalis_833 | 0.197 |
| Myzia_oblongoguttata_514 | Scymnus_suturalis_833 | 0.197 |
| Myzia_oblongoguttata_515 | Scymnus_suturalis_833 | 0.197 |
| Chilocorus_renipustulatus_34 | Scymnus_suturalis_835 | 0.197 |
| Chilocorus_renipustulatus_34 | Scymnus_suturalis_836 | 0.197 |
| Chilocorus_renipustulatus_39 | Scymnus_suturalis_838 | 0.197 |
| Myzia_oblongoguttata_515 | Scymnus_suturalis_838 | 0.197 |
| Myzia_oblongoguttata_516 | Scymnus_suturalis_838 | 0.197 |
| Chilocorus_renipustulatus_34 | Scymnus_suturalis_839 | 0.197 |
| Chilocorus_renipustulatus_39 | Scymnus_suturalis_840 | 0.197 |
| Myzia_oblongoguttata_516 | Scymnus_suturalis_840 | 0.197 |
| Anisosticta_bitriangularis_150 | Scymnus_sinuanodulus_830 | 0.197 |
| Coccinella_undecimpunctata_285 | Vibidia_duodecimguttata_597 | 0.197 |
| Coccinella_undecimpunctata_286 | Vibidia_duodecimguttata_597 | 0.197 |
| Coccinella_undecimpunctata_288 | Vibidia_duodecimguttata_597 | 0.197 |

| | | |
|---|---|---|
| Coccinella_undecimpunctata_291 | Vibidia_duodecimguttata_597 | 0.197 |
| Coccinella_undecimpunctata_285 | Vibidia_duodecimguttata_600 | 0.197 |
| Coccinella_undecimpunctata_286 | Vibidia_duodecimguttata_600 | 0.197 |
| Coccinella_undecimpunctata_288 | Vibidia_duodecimguttata_600 | 0.197 |
| Coccinella_undecimpunctata_291 | Vibidia_duodecimguttata_600 | 0.197 |
| Chilocorus_renipustulatus_31 | Scymnus_suturalis_841 | 0.197 |
| Chilocorus_renipustulatus_32 | Scymnus_suturalis_841 | 0.197 |
| Chilocorus_renipustulatus_35 | Scymnus_suturalis_841 | 0.197 |
| Chilocorus_renipustulatus_36 | Scymnus_suturalis_841 | 0.197 |
| Chilocorus_renipustulatus_37 | Scymnus_suturalis_841 | 0.197 |
| Chilocorus_renipustulatus_38 | Scymnus_suturalis_841 | 0.197 |
| Chilocorus_renipustulatus_40 | Scymnus_suturalis_841 | 0.197 |
| Chilocorus_renipustulatus_41 | Scymnus_suturalis_841 | 0.197 |
| Chilocorus_renipustulatus_42 | Scymnus_suturalis_841 | 0.197 |
| Myzia_oblongoguttata_504 | Scymnus_suturalis_841 | 0.197 |
| Myzia_oblongoguttata_505 | Scymnus_suturalis_841 | 0.197 |
| Myzia_oblongoguttata_507 | Scymnus_suturalis_841 | 0.197 |
| Myzia_oblongoguttata_508 | Scymnus_suturalis_841 | 0.197 |
| Myzia_oblongoguttata_509 | Scymnus_suturalis_841 | 0.197 |
| Myzia_oblongoguttata_510 | Scymnus_suturalis_841 | 0.197 |
| Myzia_oblongoguttata_511 | Scymnus_suturalis_841 | 0.197 |
| Myzia_oblongoguttata_512 | Scymnus_suturalis_841 | 0.197 |
| Myzia_oblongoguttata_513 | Scymnus_suturalis_841 | 0.197 |
| Myzia_oblongoguttata_514 | Scymnus_suturalis_841 | 0.197 |
| Myzia_oblongoguttata_515 | Scymnus_suturalis_841 | 0.197 |
| Chilocorus_renipustulatus_31 | Scymnus_suturalis_842 | 0.197 |
| Chilocorus_renipustulatus_32 | Scymnus_suturalis_842 | 0.197 |
| Chilocorus_renipustulatus_35 | Scymnus_suturalis_842 | 0.197 |
| Chilocorus_renipustulatus_36 | Scymnus_suturalis_842 | 0.197 |
| Chilocorus_renipustulatus_37 | Scymnus_suturalis_842 | 0.197 |
| Chilocorus_renipustulatus_38 | Scymnus_suturalis_842 | 0.197 |
| Chilocorus_renipustulatus_40 | Scymnus_suturalis_842 | 0.197 |
| Chilocorus_renipustulatus_41 | Scymnus_suturalis_842 | 0.197 |
| Chilocorus_renipustulatus_42 | Scymnus_suturalis_842 | 0.197 |
| Myzia_oblongoguttata_504 | Scymnus_suturalis_842 | 0.197 |
| Myzia_oblongoguttata_505 | Scymnus_suturalis_842 | 0.197 |
| Myzia_oblongoguttata_507 | Scymnus_suturalis_842 | 0.197 |
| Myzia_oblongoguttata_508 | Scymnus_suturalis_842 | 0.197 |
| Myzia_oblongoguttata_509 | Scymnus_suturalis_842 | 0.197 |
| Myzia_oblongoguttata_510 | Scymnus_suturalis_842 | 0.197 |
| Myzia_oblongoguttata_511 | Scymnus_suturalis_842 | 0.197 |
| Myzia_oblongoguttata_512 | Scymnus_suturalis_842 | 0.197 |
| Myzia_oblongoguttata_513 | Scymnus_suturalis_842 | 0.197 |
| Myzia_oblongoguttata_514 | Scymnus_suturalis_842 | 0.197 |
| Myzia_oblongoguttata_515 | Scymnus_suturalis_842 | 0.197 |
| Myzia_oblongoguttata_503 | Scymnus_suturalis_843 | 0.197 |
| Chilocorus_renipustulatus_31 | Scymnus_suturalis_844 | 0.197 |
| Chilocorus_renipustulatus_32 | Scymnus_suturalis_844 | 0.197 |
| Chilocorus_renipustulatus_35 | Scymnus_suturalis_844 | 0.197 |
| Chilocorus_renipustulatus_36 | Scymnus_suturalis_844 | 0.197 |
| Chilocorus_renipustulatus_37 | Scymnus_suturalis_844 | 0.197 |
| Chilocorus_renipustulatus_38 | Scymnus_suturalis_844 | 0.197 |
| Chilocorus_renipustulatus_40 | Scymnus_suturalis_844 | 0.197 |
| Chilocorus_renipustulatus_41 | Scymnus_suturalis_844 | 0.197 |
| Chilocorus_renipustulatus_42 | Scymnus_suturalis_844 | 0.197 |
| Myzia_oblongoguttata_504 | Scymnus_suturalis_844 | 0.197 |

| | | |
|---|---|---|
| Myzia_oblongoguttata_505 | Scymnus_suturalis_844 | 0.197 |
| Myzia_oblongoguttata_507 | Scymnus_suturalis_844 | 0.197 |
| Myzia_oblongoguttata_508 | Scymnus_suturalis_844 | 0.197 |
| Myzia_oblongoguttata_509 | Scymnus_suturalis_844 | 0.197 |
| Myzia_oblongoguttata_510 | Scymnus_suturalis_844 | 0.197 |
| Myzia_oblongoguttata_511 | Scymnus_suturalis_844 | 0.197 |
| Myzia_oblongoguttata_512 | Scymnus_suturalis_844 | 0.197 |
| Myzia_oblongoguttata_513 | Scymnus_suturalis_844 | 0.197 |
| Myzia_oblongoguttata_514 | Scymnus_suturalis_844 | 0.197 |
| Myzia_oblongoguttata_515 | Scymnus_suturalis_844 | 0.197 |
| Myzia_oblongoguttata_503 | Scymnus_suturalis_845 | 0.197 |
| Chilocorus_renipustulatus_31 | Scymnus_suturalis_846 | 0.197 |
| Chilocorus_renipustulatus_32 | Scymnus_suturalis_846 | 0.197 |
| Chilocorus_renipustulatus_35 | Scymnus_suturalis_846 | 0.197 |
| Chilocorus_renipustulatus_36 | Scymnus_suturalis_846 | 0.197 |
| Chilocorus_renipustulatus_37 | Scymnus_suturalis_846 | 0.197 |
| Chilocorus_renipustulatus_38 | Scymnus_suturalis_846 | 0.197 |
| Chilocorus_renipustulatus_40 | Scymnus_suturalis_846 | 0.197 |
| Chilocorus_renipustulatus_41 | Scymnus_suturalis_846 | 0.197 |
| Chilocorus_renipustulatus_42 | Scymnus_suturalis_846 | 0.197 |
| Myzia_oblongoguttata_504 | Scymnus_suturalis_846 | 0.197 |
| Myzia_oblongoguttata_505 | Scymnus_suturalis_846 | 0.197 |
| Myzia_oblongoguttata_507 | Scymnus_suturalis_846 | 0.197 |
| Myzia_oblongoguttata_508 | Scymnus_suturalis_846 | 0.197 |
| Myzia_oblongoguttata_509 | Scymnus_suturalis_846 | 0.197 |
| Myzia_oblongoguttata_510 | Scymnus_suturalis_846 | 0.197 |
| Myzia_oblongoguttata_511 | Scymnus_suturalis_846 | 0.197 |
| Myzia_oblongoguttata_512 | Scymnus_suturalis_846 | 0.197 |
| Myzia_oblongoguttata_513 | Scymnus_suturalis_846 | 0.197 |
| Myzia_oblongoguttata_514 | Scymnus_suturalis_846 | 0.197 |
| Myzia_oblongoguttata_515 | Scymnus_suturalis_846 | 0.197 |
| Chilocorus_renipustulatus_31 | Scymnus_suturalis_847 | 0.197 |
| Chilocorus_renipustulatus_32 | Scymnus_suturalis_847 | 0.197 |
| Chilocorus_renipustulatus_35 | Scymnus_suturalis_847 | 0.197 |
| Chilocorus_renipustulatus_36 | Scymnus_suturalis_847 | 0.197 |
| Chilocorus_renipustulatus_37 | Scymnus_suturalis_847 | 0.197 |
| Chilocorus_renipustulatus_38 | Scymnus_suturalis_847 | 0.197 |
| Chilocorus_renipustulatus_40 | Scymnus_suturalis_847 | 0.197 |
| Chilocorus_renipustulatus_41 | Scymnus_suturalis_847 | 0.197 |
| Chilocorus_renipustulatus_42 | Scymnus_suturalis_847 | 0.197 |
| Myzia_oblongoguttata_504 | Scymnus_suturalis_847 | 0.197 |
| Myzia_oblongoguttata_505 | Scymnus_suturalis_847 | 0.197 |
| Myzia_oblongoguttata_507 | Scymnus_suturalis_847 | 0.197 |
| Myzia_oblongoguttata_508 | Scymnus_suturalis_847 | 0.197 |
| Myzia_oblongoguttata_509 | Scymnus_suturalis_847 | 0.197 |
| Myzia_oblongoguttata_510 | Scymnus_suturalis_847 | 0.197 |
| Myzia_oblongoguttata_511 | Scymnus_suturalis_847 | 0.197 |
| Myzia_oblongoguttata_512 | Scymnus_suturalis_847 | 0.197 |
| Myzia_oblongoguttata_513 | Scymnus_suturalis_847 | 0.197 |
| Myzia_oblongoguttata_514 | Scymnus_suturalis_847 | 0.197 |
| Myzia_oblongoguttata_515 | Scymnus_suturalis_847 | 0.197 |
| Chilocorus_renipustulatus_31 | Scymnus_suturalis_848 | 0.197 |
| Chilocorus_renipustulatus_32 | Scymnus_suturalis_848 | 0.197 |
| Chilocorus_renipustulatus_35 | Scymnus_suturalis_848 | 0.197 |
| Chilocorus_renipustulatus_36 | Scymnus_suturalis_848 | 0.197 |
| Chilocorus_renipustulatus_37 | Scymnus_suturalis_848 | 0.197 |

| | | |
|---|---|---|
| Chilocorus_renipustulatus_38 | Scymnus_suturalis_848 | 0.197 |
| Chilocorus_renipustulatus_40 | Scymnus_suturalis_848 | 0.197 |
| Chilocorus_renipustulatus_41 | Scymnus_suturalis_848 | 0.197 |
| Chilocorus_renipustulatus_42 | Scymnus_suturalis_848 | 0.197 |
| Myzia_oblongoguttata_504 | Scymnus_suturalis_848 | 0.197 |
| Myzia_oblongoguttata_505 | Scymnus_suturalis_848 | 0.197 |
| Myzia_oblongoguttata_507 | Scymnus_suturalis_848 | 0.197 |
| Myzia_oblongoguttata_508 | Scymnus_suturalis_848 | 0.197 |
| Myzia_oblongoguttata_509 | Scymnus_suturalis_848 | 0.197 |
| Myzia_oblongoguttata_510 | Scymnus_suturalis_848 | 0.197 |
| Myzia_oblongoguttata_511 | Scymnus_suturalis_848 | 0.197 |
| Myzia_oblongoguttata_512 | Scymnus_suturalis_848 | 0.197 |
| Myzia_oblongoguttata_513 | Scymnus_suturalis_848 | 0.197 |
| Myzia_oblongoguttata_514 | Scymnus_suturalis_848 | 0.197 |
| Myzia_oblongoguttata_515 | Scymnus_suturalis_848 | 0.197 |
| Hyperaspis_reppensis_662 | Stethorus_punctillum_851 | 0.197 |
| Hyperaspis_reppensis_663 | Stethorus_punctillum_851 | 0.197 |
| Hyperaspis_reppensis_662 | Stethorus_punctillum_858 | 0.197 |
| Hyperaspis_reppensis_663 | Stethorus_punctillum_858 | 0.197 |
| Scymnus_loewii_770 | Subcoccinella_vigintiquatuorpunctata_620 | 0.197 |
| Scymnus_loewii_770 | Subcoccinella_vigintiquatuorpunctata_621 | 0.197 |
| Scymnus_loewii_771 | Subcoccinella_vigintiquatuorpunctata_622 | 0.197 |
| Scymnus_loewii_770 | Subcoccinella_vigintiquatuorpunctata_624 | 0.197 |
| Scymnus_loewii_771 | Subcoccinella_vigintiquatuorpunctata_626 | 0.197 |
| Scymnus_loewii_770 | Subcoccinella_vigintiquatuorpunctata_628 | 0.197 |
| Scymnus_loewii_770 | Subcoccinella_vigintiquatuorpunctata_629 | 0.197 |
| Scymnus_loewii_772 | Subcoccinella_vigintiquatuorpunctata_630 | 0.197 |
| Scymnus_loewii_773 | Subcoccinella_vigintiquatuorpunctata_630 | 0.197 |
| Scymnus_loewii_770 | Subcoccinella_vigintiquatuorpunctata_631 | 0.197 |
| Coccinella_transversalis_257 | Tytthaspis_sedecimpunctata_589 | 0.197 |
| Coccinella_transversalis_259 | Tytthaspis_sedecimpunctata_589 | 0.197 |
| Coccinella_transversalis_260 | Tytthaspis_sedecimpunctata_589 | 0.197 |
| Coccinella_transversalis_261 | Tytthaspis_sedecimpunctata_589 | 0.197 |
| Coccinella_transversalis_262 | Tytthaspis_sedecimpunctata_589 | 0.197 |
| Coccinella_transversalis_263 | Tytthaspis_sedecimpunctata_589 | 0.197 |
| Coccinella_transversalis_264 | Tytthaspis_sedecimpunctata_589 | 0.197 |
| Rhyzobius_chrysomeloides_85 | Tytthaspis_sedecimpunctata_589 | 0.197 |
| Rhyzobius_chrysomeloides_86 | Tytthaspis_sedecimpunctata_589 | 0.197 |
| Rhyzobius_chrysomeloides_87 | Tytthaspis_sedecimpunctata_589 | 0.197 |
| Rhyzobius_chrysomeloides_88 | Tytthaspis_sedecimpunctata_589 | 0.197 |
| Rhyzobius_chrysomeloides_89 | Tytthaspis_sedecimpunctata_589 | 0.197 |
| Rhyzobius_chrysomeloides_90 | Tytthaspis_sedecimpunctata_589 | 0.197 |
| Rhyzobius_chrysomeloides_91 | Tytthaspis_sedecimpunctata_589 | 0.197 |
| Rhyzobius_chrysomeloides_92 | Tytthaspis_sedecimpunctata_589 | 0.197 |
| Rhyzobius_chrysomeloides_93 | Tytthaspis_sedecimpunctata_589 | 0.197 |
| Rhyzobius_chrysomeloides_94 | Tytthaspis_sedecimpunctata_589 | 0.197 |
| Rhyzobius_chrysomeloides_95 | Tytthaspis_sedecimpunctata_589 | 0.197 |
| Myrrha_octodecimguttata_495 | Scymnus_sinuanodulus_828 | 0.197 |
| Platynaspis_luteorubra_51 | Scymnus_sinuanodulus_828 | 0.197 |
| Platynaspis_luteorubra_53 | Scymnus_sinuanodulus_828 | 0.197 |
| Platynaspis_luteorubra_58 | Scymnus_sinuanodulus_828 | 0.197 |
| Chilocorus_renipustulatus_31 | Scymnus_suturalis_832 | 0.197 |
| Chilocorus_renipustulatus_32 | Scymnus_suturalis_832 | 0.197 |
| Chilocorus_renipustulatus_35 | Scymnus_suturalis_832 | 0.197 |
| Chilocorus_renipustulatus_36 | Scymnus_suturalis_832 | 0.197 |
| Chilocorus_renipustulatus_37 | Scymnus_suturalis_832 | 0.197 |

Supplementary Table S4 (continued)

| | | |
|---|---|---|
| Chilocorus_renipustulatus_38 | Scymnus_suturalis_832 | 0.197 |
| Chilocorus_renipustulatus_40 | Scymnus_suturalis_832 | 0.197 |
| Chilocorus_renipustulatus_41 | Scymnus_suturalis_832 | 0.197 |
| Chilocorus_renipustulatus_42 | Scymnus_suturalis_832 | 0.197 |
| Myzia_oblongoguttata_504 | Scymnus_suturalis_832 | 0.197 |
| Myzia_oblongoguttata_505 | Scymnus_suturalis_832 | 0.197 |
| Myzia_oblongoguttata_507 | Scymnus_suturalis_832 | 0.197 |
| Myzia_oblongoguttata_508 | Scymnus_suturalis_832 | 0.197 |
| Myzia_oblongoguttata_509 | Scymnus_suturalis_832 | 0.197 |
| Myzia_oblongoguttata_510 | Scymnus_suturalis_832 | 0.197 |
| Myzia_oblongoguttata_511 | Scymnus_suturalis_832 | 0.197 |
| Myzia_oblongoguttata_512 | Scymnus_suturalis_832 | 0.197 |
| Myzia_oblongoguttata_513 | Scymnus_suturalis_832 | 0.197 |
| Myzia_oblongoguttata_514 | Scymnus_suturalis_832 | 0.197 |
| Myzia_oblongoguttata_515 | Scymnus_suturalis_832 | 0.197 |
| Myzia_oblongoguttata_516 | Scymnus_suturalis_834 | 0.197 |
| Chilocorus_renipustulatus_31 | Scymnus_suturalis_835 | 0.197 |
| Chilocorus_renipustulatus_32 | Scymnus_suturalis_835 | 0.197 |
| Chilocorus_renipustulatus_35 | Scymnus_suturalis_835 | 0.197 |
| Chilocorus_renipustulatus_36 | Scymnus_suturalis_835 | 0.197 |
| Chilocorus_renipustulatus_37 | Scymnus_suturalis_835 | 0.197 |
| Chilocorus_renipustulatus_38 | Scymnus_suturalis_835 | 0.197 |
| Chilocorus_renipustulatus_40 | Scymnus_suturalis_835 | 0.197 |
| Chilocorus_renipustulatus_41 | Scymnus_suturalis_835 | 0.197 |
| Chilocorus_renipustulatus_42 | Scymnus_suturalis_835 | 0.197 |
| Myzia_oblongoguttata_504 | Scymnus_suturalis_835 | 0.197 |
| Myzia_oblongoguttata_505 | Scymnus_suturalis_835 | 0.197 |
| Myzia_oblongoguttata_507 | Scymnus_suturalis_835 | 0.197 |
| Myzia_oblongoguttata_508 | Scymnus_suturalis_835 | 0.197 |
| Myzia_oblongoguttata_509 | Scymnus_suturalis_835 | 0.197 |
| Myzia_oblongoguttata_510 | Scymnus_suturalis_835 | 0.197 |
| Myzia_oblongoguttata_511 | Scymnus_suturalis_835 | 0.197 |
| Myzia_oblongoguttata_512 | Scymnus_suturalis_835 | 0.197 |
| Myzia_oblongoguttata_513 | Scymnus_suturalis_835 | 0.197 |
| Myzia_oblongoguttata_514 | Scymnus_suturalis_835 | 0.197 |
| Myzia_oblongoguttata_515 | Scymnus_suturalis_835 | 0.197 |
| Chilocorus_renipustulatus_31 | Scymnus_suturalis_836 | 0.197 |
| Chilocorus_renipustulatus_32 | Scymnus_suturalis_836 | 0.197 |
| Chilocorus_renipustulatus_35 | Scymnus_suturalis_836 | 0.197 |
| Chilocorus_renipustulatus_36 | Scymnus_suturalis_836 | 0.197 |
| Chilocorus_renipustulatus_37 | Scymnus_suturalis_836 | 0.197 |
| Chilocorus_renipustulatus_38 | Scymnus_suturalis_836 | 0.197 |
| Chilocorus_renipustulatus_40 | Scymnus_suturalis_836 | 0.197 |
| Chilocorus_renipustulatus_41 | Scymnus_suturalis_836 | 0.197 |
| Chilocorus_renipustulatus_42 | Scymnus_suturalis_836 | 0.197 |
| Myzia_oblongoguttata_504 | Scymnus_suturalis_836 | 0.197 |
| Myzia_oblongoguttata_505 | Scymnus_suturalis_836 | 0.197 |
| Myzia_oblongoguttata_507 | Scymnus_suturalis_836 | 0.197 |
| Myzia_oblongoguttata_508 | Scymnus_suturalis_836 | 0.197 |
| Myzia_oblongoguttata_509 | Scymnus_suturalis_836 | 0.197 |
| Myzia_oblongoguttata_510 | Scymnus_suturalis_836 | 0.197 |
| Myzia_oblongoguttata_511 | Scymnus_suturalis_836 | 0.197 |
| Myzia_oblongoguttata_512 | Scymnus_suturalis_836 | 0.197 |
| Myzia_oblongoguttata_513 | Scymnus_suturalis_836 | 0.197 |
| Myzia_oblongoguttata_514 | Scymnus_suturalis_836 | 0.197 |
| Myzia_oblongoguttata_515 | Scymnus_suturalis_836 | 0.197 |

| | | |
|---|---|---|
| Chilocorus_renipustulatus_39 | Scymnus_suturalis_837 | 0.197 |
| Myzia_oblongoguttata_504 | Scymnus_suturalis_837 | 0.197 |
| Myzia_oblongoguttata_505 | Scymnus_suturalis_837 | 0.197 |
| Myzia_oblongoguttata_507 | Scymnus_suturalis_837 | 0.197 |
| Myzia_oblongoguttata_508 | Scymnus_suturalis_837 | 0.197 |
| Myzia_oblongoguttata_509 | Scymnus_suturalis_837 | 0.197 |
| Myzia_oblongoguttata_510 | Scymnus_suturalis_837 | 0.197 |
| Myzia_oblongoguttata_511 | Scymnus_suturalis_837 | 0.197 |
| Myzia_oblongoguttata_512 | Scymnus_suturalis_837 | 0.197 |
| Myzia_oblongoguttata_513 | Scymnus_suturalis_837 | 0.197 |
| Myzia_oblongoguttata_514 | Scymnus_suturalis_837 | 0.197 |
| Myzia_oblongoguttata_515 | Scymnus_suturalis_837 | 0.197 |
| Cynegetis_impunctata_604 | Scymnus_suturalis_838 | 0.197 |
| Cynegetis_impunctata_608 | Scymnus_suturalis_838 | 0.197 |
| Cynegetis_impunctata_609 | Scymnus_suturalis_838 | 0.197 |
| Cynegetis_impunctata_610 | Scymnus_suturalis_838 | 0.197 |
| Cynegetis_impunctata_611 | Scymnus_suturalis_838 | 0.197 |
| Cynegetis_impunctata_612 | Scymnus_suturalis_838 | 0.197 |
| Chilocorus_renipustulatus_31 | Scymnus_suturalis_839 | 0.197 |
| Chilocorus_renipustulatus_32 | Scymnus_suturalis_839 | 0.197 |
| Chilocorus_renipustulatus_35 | Scymnus_suturalis_839 | 0.197 |
| Chilocorus_renipustulatus_36 | Scymnus_suturalis_839 | 0.197 |
| Chilocorus_renipustulatus_37 | Scymnus_suturalis_839 | 0.197 |
| Chilocorus_renipustulatus_38 | Scymnus_suturalis_839 | 0.197 |
| Chilocorus_renipustulatus_40 | Scymnus_suturalis_839 | 0.197 |
| Chilocorus_renipustulatus_41 | Scymnus_suturalis_839 | 0.197 |
| Chilocorus_renipustulatus_42 | Scymnus_suturalis_839 | 0.197 |
| Myzia_oblongoguttata_504 | Scymnus_suturalis_839 | 0.197 |
| Myzia_oblongoguttata_505 | Scymnus_suturalis_839 | 0.197 |
| Myzia_oblongoguttata_507 | Scymnus_suturalis_839 | 0.197 |
| Myzia_oblongoguttata_508 | Scymnus_suturalis_839 | 0.197 |
| Myzia_oblongoguttata_509 | Scymnus_suturalis_839 | 0.197 |
| Myzia_oblongoguttata_510 | Scymnus_suturalis_839 | 0.197 |
| Myzia_oblongoguttata_511 | Scymnus_suturalis_839 | 0.197 |
| Myzia_oblongoguttata_512 | Scymnus_suturalis_839 | 0.197 |
| Myzia_oblongoguttata_513 | Scymnus_suturalis_839 | 0.197 |
| Myzia_oblongoguttata_514 | Scymnus_suturalis_839 | 0.197 |
| Myzia_oblongoguttata_515 | Scymnus_suturalis_839 | 0.197 |
| Brumus_quadripustulatus_9 | Scymnus_sinuanodulus_830 | 0.197 |
| Myrrha_octodecimguttata_499 | Scymnus_suturalis_833 | 0.197 |
| Macronaemia_episcopalis_477 | Scymnus_suturalis_838 | 0.197 |
| Macronaemia_episcopalis_477 | Scymnus_suturalis_840 | 0.197 |
| Chilocorus_bipustulatus_21 | Scymnus_suturalis_843 | 0.197 |
| Chilocorus_bipustulatus_21 | Scymnus_suturalis_845 | 0.197 |
| Harmonia_quadripunctata_376 | Tytthaspis_sedecimpunctata_577 | 0.197 |
| Harmonia_quadripunctata_378 | Tytthaspis_sedecimpunctata_577 | 0.197 |
| Harmonia_quadripunctata_382 | Tytthaspis_sedecimpunctata_577 | 0.197 |
| Harmonia_quadripunctata_376 | Tytthaspis_sedecimpunctata_578 | 0.197 |
| Harmonia_quadripunctata_378 | Tytthaspis_sedecimpunctata_578 | 0.197 |
| Harmonia_quadripunctata_382 | Tytthaspis_sedecimpunctata_578 | 0.197 |
| Harmonia_quadripunctata_376 | Tytthaspis_sedecimpunctata_579 | 0.197 |
| Harmonia_quadripunctata_378 | Tytthaspis_sedecimpunctata_579 | 0.197 |
| Harmonia_quadripunctata_382 | Tytthaspis_sedecimpunctata_579 | 0.197 |
| Brachiacantha_ursina_637 | Tytthaspis_sedecimpunctata_580 | 0.197 |
| Harmonia_quadripunctata_376 | Tytthaspis_sedecimpunctata_581 | 0.197 |
| Harmonia_quadripunctata_378 | Tytthaspis_sedecimpunctata_581 | 0.197 |

| | | |
|---|---|---|
| Harmonia_quadripunctata_382 | Tytthaspis_sedecimpunctata_581 | 0.197 |
| Harmonia_quadripunctata_376 | Tytthaspis_sedecimpunctata_582 | 0.197 |
| Harmonia_quadripunctata_378 | Tytthaspis_sedecimpunctata_582 | 0.197 |
| Harmonia_quadripunctata_382 | Tytthaspis_sedecimpunctata_582 | 0.197 |
| Diomus_terminatus_647 | Tytthaspis_sedecimpunctata_583 | 0.197 |
| Diomus_terminatus_648 | Tytthaspis_sedecimpunctata_583 | 0.197 |
| Myzia_pullata_519 | Tytthaspis_sedecimpunctata_584 | 0.197 |
| Adalia_decempunctata_118 | Tytthaspis_sedecimpunctata_585 | 0.197 |
| Adalia_decempunctata_119 | Tytthaspis_sedecimpunctata_585 | 0.197 |
| Adalia_decempunctata_120 | Tytthaspis_sedecimpunctata_585 | 0.197 |
| Adalia_decempunctata_121 | Tytthaspis_sedecimpunctata_585 | 0.197 |
| Harmonia_quadripunctata_376 | Tytthaspis_sedecimpunctata_590 | 0.197 |
| Harmonia_quadripunctata_378 | Tytthaspis_sedecimpunctata_590 | 0.197 |
| Harmonia_quadripunctata_382 | Tytthaspis_sedecimpunctata_590 | 0.197 |
| Harmonia_quadripunctata_376 | Tytthaspis_sedecimpunctata_592 | 0.197 |
| Harmonia_quadripunctata_378 | Tytthaspis_sedecimpunctata_592 | 0.197 |
| Harmonia_quadripunctata_382 | Tytthaspis_sedecimpunctata_592 | 0.197 |
| Harmonia_quadripunctata_376 | Tytthaspis_sedecimpunctata_593 | 0.197 |
| Harmonia_quadripunctata_378 | Tytthaspis_sedecimpunctata_593 | 0.197 |
| Harmonia_quadripunctata_382 | Tytthaspis_sedecimpunctata_593 | 0.197 |
| Harmonia_quadripunctata_376 | Tytthaspis_sedecimpunctata_594 | 0.197 |
| Harmonia_quadripunctata_378 | Tytthaspis_sedecimpunctata_594 | 0.197 |
| Harmonia_quadripunctata_382 | Tytthaspis_sedecimpunctata_594 | 0.197 |
| Harmonia_quadripunctata_376 | Tytthaspis_sedecimpunctata_595 | 0.197 |
| Harmonia_quadripunctata_378 | Tytthaspis_sedecimpunctata_595 | 0.197 |
| Harmonia_quadripunctata_382 | Tytthaspis_sedecimpunctata_595 | 0.197 |
| Hyperaspis_campestris_651 | Scymnus_rubromaculatus_822 | 0.197 |
| Hyperaspis_campestris_652 | Scymnus_rubromaculatus_822 | 0.197 |
| Hyperaspis_campestris_653 | Scymnus_rubromaculatus_822 | 0.197 |
| Hyperaspis_campestris_651 | Scymnus_rubromaculatus_823 | 0.197 |
| Hyperaspis_campestris_652 | Scymnus_rubromaculatus_823 | 0.197 |
| Hyperaspis_campestris_653 | Scymnus_rubromaculatus_823 | 0.197 |
| Hyperaspis_campestris_651 | Scymnus_rubromaculatus_824 | 0.197 |
| Hyperaspis_campestris_652 | Scymnus_rubromaculatus_824 | 0.197 |
| Hyperaspis_campestris_653 | Scymnus_rubromaculatus_824 | 0.197 |
| Hyperaspis_campestris_651 | Scymnus_rubromaculatus_825 | 0.197 |
| Hyperaspis_campestris_652 | Scymnus_rubromaculatus_825 | 0.197 |
| Hyperaspis_campestris_653 | Scymnus_rubromaculatus_825 | 0.197 |
| Hyperaspis_campestris_651 | Scymnus_rubromaculatus_826 | 0.197 |
| Hyperaspis_campestris_652 | Scymnus_rubromaculatus_826 | 0.197 |
| Hyperaspis_campestris_653 | Scymnus_rubromaculatus_826 | 0.197 |
| Hyperaspis_campestris_651 | Scymnus_rubromaculatus_827 | 0.197 |
| Hyperaspis_campestris_652 | Scymnus_rubromaculatus_827 | 0.197 |
| Hyperaspis_campestris_653 | Scymnus_rubromaculatus_827 | 0.197 |
| Brumus_quadripustulatus_9 | Scymnus_sinuanodulus_828 | 0.197 |
| Anisosticta_bitriangularis_151 | Scymnus_sinuanodulus_830 | 0.197 |
| Anisosticta_bitriangularis_153 | Scymnus_sinuanodulus_830 | 0.197 |
| Anisosticta_bitriangularis_154 | Scymnus_sinuanodulus_830 | 0.197 |
| Anisosticta_bitriangularis_156 | Scymnus_sinuanodulus_830 | 0.197 |
| Anisosticta_bitriangularis_157 | Scymnus_sinuanodulus_830 | 0.197 |
| Macronaemia_episcopalis_477 | Scymnus_suturalis_834 | 0.197 |
| Macronaemia_episcopalis_477 | Scymnus_suturalis_837 | 0.197 |
| Scymnus_apetzi_692 | Tytthaspis_sedecimpunctata_589 | 0.197 |
| Chilocorus_bipustulatus_21 | Scymnus_suturalis_833 | 0.197 |
| Scymnus_apetzi_692 | Tytthaspis_sedecimpunctata_588 | 0.197 |
| Cynegetis_impunctata_604 | Subcoccinella_vigintiquatuorpunctata_623 | 0.197 |

| | | |
|---|---|---|
| Cynegetis_impunctata_605 | Subcoccinella_vigintiquatuorpunctata_623 | 0.197 |
| Cynegetis_impunctata_606 | Subcoccinella_vigintiquatuorpunctata_623 | 0.197 |
| Cynegetis_impunctata_607 | Subcoccinella_vigintiquatuorpunctata_623 | 0.197 |
| Cynegetis_impunctata_608 | Subcoccinella_vigintiquatuorpunctata_623 | 0.197 |
| Cynegetis_impunctata_609 | Subcoccinella_vigintiquatuorpunctata_623 | 0.197 |
| Cynegetis_impunctata_610 | Subcoccinella_vigintiquatuorpunctata_623 | 0.197 |
| Cynegetis_impunctata_611 | Subcoccinella_vigintiquatuorpunctata_623 | 0.197 |
| Cynegetis_impunctata_612 | Subcoccinella_vigintiquatuorpunctata_623 | 0.197 |
| Cynegetis_impunctata_613 | Subcoccinella_vigintiquatuorpunctata_623 | 0.197 |
| Coccinella_undecimpunctata_291 | Vibidia_duodecimguttata_599 | 0.197 |
| Coelophora_inaequalis_310 | Vibidia_duodecimguttata_599 | 0.197 |
| Coelophora_inaequalis_311 | Vibidia_duodecimguttata_599 | 0.197 |
| Coelophora_inaequalis_316 | Vibidia_duodecimguttata_599 | 0.197 |
| Coelophora_inaequalis_323 | Vibidia_duodecimguttata_599 | 0.197 |
| Coelophora_inaequalis_324 | Vibidia_duodecimguttata_599 | 0.197 |
| Coccinella_undecimpunctata_291 | Vibidia_duodecimguttata_601 | 0.197 |
| Coelophora_inaequalis_310 | Vibidia_duodecimguttata_601 | 0.197 |
| Coelophora_inaequalis_311 | Vibidia_duodecimguttata_601 | 0.197 |
| Coelophora_inaequalis_316 | Vibidia_duodecimguttata_601 | 0.197 |
| Coelophora_inaequalis_323 | Vibidia_duodecimguttata_601 | 0.197 |
| Coelophora_inaequalis_324 | Vibidia_duodecimguttata_601 | 0.197 |
| Coccinella_undecimpunctata_291 | Vibidia_duodecimguttata_602 | 0.197 |
| Coelophora_inaequalis_310 | Vibidia_duodecimguttata_602 | 0.197 |
| Coelophora_inaequalis_311 | Vibidia_duodecimguttata_602 | 0.197 |
| Coelophora_inaequalis_316 | Vibidia_duodecimguttata_602 | 0.197 |
| Coelophora_inaequalis_323 | Vibidia_duodecimguttata_602 | 0.197 |
| Coelophora_inaequalis_324 | Vibidia_duodecimguttata_602 | 0.197 |
| Coelophora_inaequalis_313 | Vibidia_duodecimguttata_603 | 0.197 |
| Coelophora_inaequalis_326 | Vibidia_duodecimguttata_603 | 0.197 |
| Clitostethus_arcuatus_639 | Scymnus_sinuanodulus_829 | 0.197 |
| Clitostethus_arcuatus_640 | Scymnus_sinuanodulus_829 | 0.197 |
| Clitostethus_arcuatus_641 | Scymnus_sinuanodulus_829 | 0.197 |
| Clitostethus_arcuatus_643 | Scymnus_sinuanodulus_829 | 0.197 |
| Scymnus_nubilus_798 | Scymnus_sinuanodulus_829 | 0.197 |
| Scymnus_nubilus_800 | Scymnus_sinuanodulus_829 | 0.197 |
| Scymnus_nubilus_801 | Scymnus_sinuanodulus_829 | 0.197 |
| Scymnus_nubilus_802 | Scymnus_sinuanodulus_829 | 0.197 |
| Scymnus_nubilus_805 | Scymnus_sinuanodulus_829 | 0.197 |
| Scymnus_nubilus_806 | Scymnus_sinuanodulus_829 | 0.197 |
| Scymnus_nubilus_807 | Scymnus_sinuanodulus_829 | 0.197 |
| Scymnus_nubilus_810 | Scymnus_sinuanodulus_829 | 0.197 |
| Chilocorus_nigritus_29 | Stethorus_punctillum_850 | 0.197 |
| Chilocorus_nigritus_29 | Stethorus_punctillum_854 | 0.197 |
| Chilocorus_nigritus_29 | Stethorus_punctillum_856 | 0.197 |
| Hyperaspis_lateralis_656 | Subcoccinella_vigintiquatuorpunctata_624 | 0.197 |
| Hyperaspis_lateralis_657 | Subcoccinella_vigintiquatuorpunctata_624 | 0.197 |
| Hyperaspis_lateralis_654 | Subcoccinella_vigintiquatuorpunctata_625 | 0.197 |
| Brachiacantha_ursina_638 | Tytthaspis_sedecimpunctata_577 | 0.197 |
| Scymnus_nebulosus_783 | Tytthaspis_sedecimpunctata_577 | 0.197 |
| Brachiacantha_ursina_638 | Tytthaspis_sedecimpunctata_578 | 0.197 |
| Scymnus_nebulosus_783 | Tytthaspis_sedecimpunctata_578 | 0.197 |
| Brachiacantha_ursina_638 | Tytthaspis_sedecimpunctata_579 | 0.197 |
| Scymnus_nebulosus_783 | Tytthaspis_sedecimpunctata_579 | 0.197 |
| Harmonia_quadripunctata_376 | Tytthaspis_sedecimpunctata_580 | 0.197 |
| Harmonia_quadripunctata_378 | Tytthaspis_sedecimpunctata_580 | 0.197 |
| Harmonia_quadripunctata_382 | Tytthaspis_sedecimpunctata_580 | 0.197 |

| | | |
|---|---|---|
| Brachiacantha_ursina_638 | Tytthaspis_sedecimpunctata_581 | 0.197 |
| Scymnus_nebulosus_783 | Tytthaspis_sedecimpunctata_581 | 0.197 |
| Brachiacantha_ursina_638 | Tytthaspis_sedecimpunctata_582 | 0.197 |
| Scymnus_nebulosus_783 | Tytthaspis_sedecimpunctata_582 | 0.197 |
| Brachiacantha_ursina_637 | Tytthaspis_sedecimpunctata_583 | 0.197 |
| Scymnus_nebulosus_781 | Tytthaspis_sedecimpunctata_583 | 0.197 |
| Scymnus_nebulosus_783 | Tytthaspis_sedecimpunctata_585 | 0.197 |
| Brachiacantha_ursina_637 | Tytthaspis_sedecimpunctata_589 | 0.197 |
| Brachiacantha_ursina_638 | Tytthaspis_sedecimpunctata_590 | 0.197 |
| Scymnus_nebulosus_783 | Tytthaspis_sedecimpunctata_590 | 0.197 |
| Brachiacantha_ursina_638 | Tytthaspis_sedecimpunctata_592 | 0.197 |
| Scymnus_nebulosus_783 | Tytthaspis_sedecimpunctata_592 | 0.197 |
| Brachiacantha_ursina_638 | Tytthaspis_sedecimpunctata_594 | 0.197 |
| Scymnus_nebulosus_783 | Tytthaspis_sedecimpunctata_594 | 0.197 |
| Brachiacantha_ursina_638 | Tytthaspis_sedecimpunctata_595 | 0.197 |
| Scymnus_nebulosus_783 | Tytthaspis_sedecimpunctata_595 | 0.197 |
| Nephus_redtenbacheri_678 | Scymnus_rubromaculatus_822 | 0.197 |
| Nephus_redtenbacheri_678 | Scymnus_rubromaculatus_823 | 0.197 |
| Nephus_redtenbacheri_678 | Scymnus_rubromaculatus_824 | 0.197 |
| Nephus_redtenbacheri_678 | Scymnus_rubromaculatus_825 | 0.197 |
| Nephus_redtenbacheri_678 | Scymnus_rubromaculatus_826 | 0.197 |
| Nephus_redtenbacheri_678 | Scymnus_rubromaculatus_827 | 0.197 |
| Hyperaspis_campestris_651 | Stethorus_punctillum_849 | 0.198 |
| Hyperaspis_campestris_652 | Stethorus_punctillum_849 | 0.198 |
| Hyperaspis_campestris_653 | Stethorus_punctillum_849 | 0.198 |
| Hyperaspis_oregona_658 | Stethorus_punctillum_851 | 0.198 |
| Hyperaspis_oregona_659 | Stethorus_punctillum_851 | 0.198 |
| Hyperaspis_campestris_651 | Stethorus_punctillum_854 | 0.198 |
| Hyperaspis_campestris_652 | Stethorus_punctillum_854 | 0.198 |
| Hyperaspis_campestris_653 | Stethorus_punctillum_854 | 0.198 |
| Hyperaspis_campestris_651 | Stethorus_punctillum_856 | 0.198 |
| Hyperaspis_campestris_652 | Stethorus_punctillum_856 | 0.198 |
| Hyperaspis_campestris_653 | Stethorus_punctillum_856 | 0.198 |
| Hyperaspis_oregona_658 | Stethorus_punctillum_858 | 0.198 |
| Hyperaspis_oregona_659 | Stethorus_punctillum_858 | 0.198 |
| Scymnus_ningshanensis_788 | Subcoccinella_vigintiquatuorpunctata_622 | 0.198 |
| Scymnus_ningshanensis_791 | Subcoccinella_vigintiquatuorpunctata_622 | 0.198 |
| Scymnus_ningshanensis_792 | Subcoccinella_vigintiquatuorpunctata_622 | 0.198 |
| Scymnus_ningshanensis_793 | Subcoccinella_vigintiquatuorpunctata_622 | 0.198 |
| Scymnus_ningshanensis_794 | Subcoccinella_vigintiquatuorpunctata_622 | 0.198 |
| Scymnus_ningshanensis_795 | Subcoccinella_vigintiquatuorpunctata_622 | 0.198 |
| Scymnus_apetzi_690 | Subcoccinella_vigintiquatuorpunctata_623 | 0.198 |
| Scymnus_apetzi_697 | Subcoccinella_vigintiquatuorpunctata_623 | 0.198 |
| Scymnus_pallipediformis_811 | Subcoccinella_vigintiquatuorpunctata_623 | 0.198 |
| Scymnus_nebulosus_781 | Tytthaspis_sedecimpunctata_584 | 0.198 |
| Brachiacantha_ursina_638 | Tytthaspis_sedecimpunctata_586 | 0.198 |
| Scymnus_nebulosus_783 | Tytthaspis_sedecimpunctata_586 | 0.198 |
| Brachiacantha_ursina_637 | Tytthaspis_sedecimpunctata_587 | 0.198 |
| Scymnus_nebulosus_783 | Tytthaspis_sedecimpunctata_588 | 0.198 |
| Myzia_pullata_519 | Tytthaspis_sedecimpunctata_591 | 0.198 |
| Adalia_bipunctata_100 | Scymnus_rubromaculatus_823 | 0.198 |
| Adalia_bipunctata_100 | Scymnus_rubromaculatus_824 | 0.198 |
| Adalia_bipunctata_100 | Scymnus_rubromaculatus_825 | 0.198 |
| Adalia_bipunctata_100 | Scymnus_rubromaculatus_826 | 0.198 |
| Adalia_bipunctata_100 | Scymnus_rubromaculatus_827 | 0.198 |
| Rhyzobius_litura_96 | Scymnus_suturalis_840 | 0.198 |

| | | |
|---|---|---|
| Cynegetis_impunctata_614 | Subcoccinella_vigintiquatuorpunctata_622 | 0.198 |
| Cynegetis_impunctata_614 | Subcoccinella_vigintiquatuorpunctata_625 | 0.198 |
| Cynegetis_impunctata_604 | Subcoccinella_vigintiquatuorpunctata_626 | 0.198 |
| Cynegetis_impunctata_605 | Subcoccinella_vigintiquatuorpunctata_626 | 0.198 |
| Cynegetis_impunctata_606 | Subcoccinella_vigintiquatuorpunctata_626 | 0.198 |
| Cynegetis_impunctata_607 | Subcoccinella_vigintiquatuorpunctata_626 | 0.198 |
| Cynegetis_impunctata_608 | Subcoccinella_vigintiquatuorpunctata_626 | 0.198 |
| Cynegetis_impunctata_609 | Subcoccinella_vigintiquatuorpunctata_626 | 0.198 |
| Cynegetis_impunctata_610 | Subcoccinella_vigintiquatuorpunctata_626 | 0.198 |
| Cynegetis_impunctata_611 | Subcoccinella_vigintiquatuorpunctata_626 | 0.198 |
| Cynegetis_impunctata_612 | Subcoccinella_vigintiquatuorpunctata_626 | 0.198 |
| Cynegetis_impunctata_613 | Subcoccinella_vigintiquatuorpunctata_626 | 0.198 |
| Scymnus_loewii_771 | Subcoccinella_vigintiquatuorpunctata_619 | 0.198 |
| Coccinella_undecimpunctata_289 | Vibidia_duodecimguttata_597 | 0.198 |
| Coccinella_undecimpunctata_290 | Vibidia_duodecimguttata_597 | 0.198 |
| Coccinella_undecimpunctata_289 | Vibidia_duodecimguttata_600 | 0.198 |
| Coccinella_undecimpunctata_290 | Vibidia_duodecimguttata_600 | 0.198 |
| Rhyzobius_litura_97 | Scymnus_suturalis_841 | 0.198 |
| Rhyzobius_litura_97 | Scymnus_suturalis_842 | 0.198 |
| Myzia_pullata_519 | Scymnus_suturalis_843 | 0.198 |
| Rhyzobius_litura_97 | Scymnus_suturalis_844 | 0.198 |
| Myzia_pullata_519 | Scymnus_suturalis_845 | 0.198 |
| Rhyzobius_litura_97 | Scymnus_suturalis_846 | 0.198 |
| Rhyzobius_litura_97 | Scymnus_suturalis_847 | 0.198 |
| Rhyzobius_litura_97 | Scymnus_suturalis_848 | 0.198 |
| Scymnus_apetzi_696 | Subcoccinella_vigintiquatuorpunctata_620 | 0.198 |
| Scymnus_nebulosus_783 | Subcoccinella_vigintiquatuorpunctata_620 | 0.198 |
| Scymnus_apetzi_696 | Subcoccinella_vigintiquatuorpunctata_621 | 0.198 |
| Scymnus_nebulosus_783 | Subcoccinella_vigintiquatuorpunctata_621 | 0.198 |
| Scymnus_apetzi_690 | Subcoccinella_vigintiquatuorpunctata_622 | 0.198 |
| Scymnus_apetzi_697 | Subcoccinella_vigintiquatuorpunctata_622 | 0.198 |
| Scymnus_pallipediformis_811 | Subcoccinella_vigintiquatuorpunctata_622 | 0.198 |
| Scymnus_apetzi_691 | Subcoccinella_vigintiquatuorpunctata_624 | 0.198 |
| Scymnus_apetzi_696 | Subcoccinella_vigintiquatuorpunctata_624 | 0.198 |
| Scymnus_nebulosus_783 | Subcoccinella_vigintiquatuorpunctata_624 | 0.198 |
| Scymnus_nebulosus_781 | Subcoccinella_vigintiquatuorpunctata_625 | 0.198 |
| Scymnus_apetzi_690 | Subcoccinella_vigintiquatuorpunctata_626 | 0.198 |
| Scymnus_apetzi_697 | Subcoccinella_vigintiquatuorpunctata_626 | 0.198 |
| Scymnus_pallipediformis_811 | Subcoccinella_vigintiquatuorpunctata_626 | 0.198 |
| Scymnus_apetzi_696 | Subcoccinella_vigintiquatuorpunctata_628 | 0.198 |
| Scymnus_nebulosus_783 | Subcoccinella_vigintiquatuorpunctata_628 | 0.198 |
| Scymnus_apetzi_696 | Subcoccinella_vigintiquatuorpunctata_629 | 0.198 |
| Scymnus_nebulosus_783 | Subcoccinella_vigintiquatuorpunctata_629 | 0.198 |
| Scymnus_apetzi_696 | Subcoccinella_vigintiquatuorpunctata_631 | 0.198 |
| Scymnus_nebulosus_783 | Subcoccinella_vigintiquatuorpunctata_631 | 0.198 |
| Scymnus_interruptus_762 | Tytthaspis_sedecimpunctata_585 | 0.198 |
| Scymnus_interruptus_767 | Tytthaspis_sedecimpunctata_591 | 0.198 |
| Hyperaspis_lateralis_654 | Scymnus_sinuanodulus_828 | 0.198 |
| Hyperaspis_lateralis_656 | Scymnus_sinuanodulus_828 | 0.198 |
| Rhyzobius_litura_97 | Scymnus_suturalis_832 | 0.198 |
| Calvia_quatuordecimguttata_201 | Scymnus_suturalis_833 | 0.198 |
| Myzia_pullata_519 | Scymnus_suturalis_834 | 0.198 |
| Rhyzobius_litura_96 | Scymnus_suturalis_834 | 0.198 |
| Rhyzobius_litura_97 | Scymnus_suturalis_835 | 0.198 |
| Rhyzobius_litura_97 | Scymnus_suturalis_836 | 0.198 |
| Rhyzobius_litura_96 | Scymnus_suturalis_837 | 0.198 |

| | | |
|---|---|---|
| Rhyzobius_litura_97 | Scymnus_suturalis_839 | 0.198 |
| Myrrha_octodecimguttata_488 | Scymnus_sinuanodulus_831 | 0.198 |
| Myrrha_octodecimguttata_491 | Scymnus_sinuanodulus_831 | 0.198 |
| Myrrha_octodecimguttata_492 | Scymnus_sinuanodulus_831 | 0.198 |
| Myrrha_octodecimguttata_493 | Scymnus_sinuanodulus_831 | 0.198 |
| Myrrha_octodecimguttata_498 | Scymnus_sinuanodulus_831 | 0.198 |
| Hyperaspis_reppensis_664 | Stethorus_punctillum_849 | 0.198 |
| Hyperaspis_reppensis_665 | Stethorus_punctillum_849 | 0.198 |
| Hyperaspis_reppensis_666 | Stethorus_punctillum_849 | 0.198 |
| Hyperaspis_reppensis_667 | Stethorus_punctillum_852 | 0.198 |
| Hyperaspis_reppensis_664 | Stethorus_punctillum_854 | 0.198 |
| Hyperaspis_reppensis_665 | Stethorus_punctillum_854 | 0.198 |
| Hyperaspis_reppensis_666 | Stethorus_punctillum_854 | 0.198 |
| Hyperaspis_reppensis_664 | Stethorus_punctillum_856 | 0.198 |
| Hyperaspis_reppensis_665 | Stethorus_punctillum_856 | 0.198 |
| Hyperaspis_reppensis_666 | Stethorus_punctillum_856 | 0.198 |
| Hyperaspis_reppensis_667 | Stethorus_punctillum_860 | 0.198 |
| Scymnus_interruptus_759 | Tytthaspis_sedecimpunctata_585 | 0.198 |
| Scymnus_interruptus_769 | Tytthaspis_sedecimpunctata_585 | 0.198 |
| Scymnus_interruptus_759 | Tytthaspis_sedecimpunctata_589 | 0.198 |
| Scymnus_interruptus_769 | Tytthaspis_sedecimpunctata_589 | 0.198 |
| Hyperaspis_lateralis_654 | Scymnus_sinuanodulus_830 | 0.198 |
| Hyperaspis_lateralis_656 | Scymnus_sinuanodulus_830 | 0.198 |
| Clitostethus_arcuatus_639 | Scymnus_suturalis_833 | 0.198 |
| Clitostethus_arcuatus_640 | Scymnus_suturalis_833 | 0.198 |
| Clitostethus_arcuatus_642 | Scymnus_suturalis_833 | 0.198 |
| Clitostethus_arcuatus_643 | Scymnus_suturalis_833 | 0.198 |
| Rhyzobius_litura_96 | Scymnus_suturalis_833 | 0.198 |
| Calvia_quatuordecimguttata_218 | Scymnus_suturalis_838 | 0.198 |
| Rhyzobius_litura_97 | Scymnus_suturalis_838 | 0.198 |
| Calvia_quatuordecimguttata_218 | Scymnus_suturalis_840 | 0.198 |
| Clitostethus_arcuatus_639 | Scymnus_suturalis_840 | 0.198 |
| Clitostethus_arcuatus_640 | Scymnus_suturalis_840 | 0.198 |
| Clitostethus_arcuatus_641 | Scymnus_suturalis_840 | 0.198 |
| Clitostethus_arcuatus_643 | Scymnus_suturalis_840 | 0.198 |
| Anisosticta_bitriangularis_149 | Scymnus_sinuanodulus_830 | 0.198 |
| Coccinella_undecimpunctata_289 | Vibidia_duodecimguttata_599 | 0.198 |
| Coccinella_undecimpunctata_290 | Vibidia_duodecimguttata_599 | 0.198 |
| Coccinella_undecimpunctata_289 | Vibidia_duodecimguttata_601 | 0.198 |
| Coccinella_undecimpunctata_290 | Vibidia_duodecimguttata_601 | 0.198 |
| Coccinella_undecimpunctata_289 | Vibidia_duodecimguttata_602 | 0.198 |
| Coccinella_undecimpunctata_290 | Vibidia_duodecimguttata_602 | 0.198 |
| Clitostethus_arcuatus_639 | Scymnus_suturalis_845 | 0.198 |
| Clitostethus_arcuatus_640 | Scymnus_suturalis_845 | 0.198 |
| Clitostethus_arcuatus_641 | Scymnus_suturalis_845 | 0.198 |
| Clitostethus_arcuatus_643 | Scymnus_suturalis_845 | 0.198 |
| Hyperaspis_lateralis_657 | Stethorus_punctillum_857 | 0.198 |
| Hyperaspis_lateralis_657 | Stethorus_punctillum_860 | 0.198 |
| Psyllobora_vigintiduopunctata_561 | Subcoccinella_vigintiquatuorpunctata_620 | 0.198 |
| Psyllobora_vigintiduopunctata_562 | Subcoccinella_vigintiquatuorpunctata_620 | 0.198 |
| Psyllobora_vigintiduopunctata_565 | Subcoccinella_vigintiquatuorpunctata_620 | 0.198 |
| Psyllobora_vigintiduopunctata_567 | Subcoccinella_vigintiquatuorpunctata_620 | 0.198 |
| Psyllobora_vigintiduopunctata_561 | Subcoccinella_vigintiquatuorpunctata_621 | 0.198 |
| Psyllobora_vigintiduopunctata_562 | Subcoccinella_vigintiquatuorpunctata_621 | 0.198 |
| Psyllobora_vigintiduopunctata_565 | Subcoccinella_vigintiquatuorpunctata_621 | 0.198 |
| Psyllobora_vigintiduopunctata_567 | Subcoccinella_vigintiquatuorpunctata_621 | 0.198 |

| | | |
|---|---|---|
| Psyllobora_vigintiduopunctata_561 | Subcoccinella_vigintiquatuorpunctata_627 | 0.198 |
| Psyllobora_vigintiduopunctata_562 | Subcoccinella_vigintiquatuorpunctata_627 | 0.198 |
| Psyllobora_vigintiduopunctata_565 | Subcoccinella_vigintiquatuorpunctata_627 | 0.198 |
| Psyllobora_vigintiduopunctata_567 | Subcoccinella_vigintiquatuorpunctata_627 | 0.198 |
| Psyllobora_vigintiduopunctata_561 | Subcoccinella_vigintiquatuorpunctata_628 | 0.198 |
| Psyllobora_vigintiduopunctata_562 | Subcoccinella_vigintiquatuorpunctata_628 | 0.198 |
| Psyllobora_vigintiduopunctata_565 | Subcoccinella_vigintiquatuorpunctata_628 | 0.198 |
| Psyllobora_vigintiduopunctata_567 | Subcoccinella_vigintiquatuorpunctata_628 | 0.198 |
| Psyllobora_vigintiduopunctata_561 | Subcoccinella_vigintiquatuorpunctata_629 | 0.198 |
| Psyllobora_vigintiduopunctata_562 | Subcoccinella_vigintiquatuorpunctata_629 | 0.198 |
| Psyllobora_vigintiduopunctata_565 | Subcoccinella_vigintiquatuorpunctata_629 | 0.198 |
| Psyllobora_vigintiduopunctata_567 | Subcoccinella_vigintiquatuorpunctata_629 | 0.198 |
| Psyllobora_vigintiduopunctata_561 | Subcoccinella_vigintiquatuorpunctata_631 | 0.198 |
| Psyllobora_vigintiduopunctata_562 | Subcoccinella_vigintiquatuorpunctata_631 | 0.198 |
| Psyllobora_vigintiduopunctata_565 | Subcoccinella_vigintiquatuorpunctata_631 | 0.198 |
| Psyllobora_vigintiduopunctata_567 | Subcoccinella_vigintiquatuorpunctata_631 | 0.198 |
| Scymnus_ferrugatus_723 | Tytthaspis_sedecimpunctata_583 | 0.198 |
| Scymnus_ferrugatus_725 | Tytthaspis_sedecimpunctata_583 | 0.198 |
| Scymnus_ferrugatus_726 | Tytthaspis_sedecimpunctata_583 | 0.198 |
| Scymnus_ferrugatus_727 | Tytthaspis_sedecimpunctata_583 | 0.198 |
| Scymnus_ferrugatus_728 | Tytthaspis_sedecimpunctata_583 | 0.198 |
| Scymnus_ferrugatus_730 | Tytthaspis_sedecimpunctata_583 | 0.198 |
| Scymnus_ferrugatus_731 | Tytthaspis_sedecimpunctata_583 | 0.198 |
| Scymnus_ferrugatus_732 | Tytthaspis_sedecimpunctata_583 | 0.198 |
| Scymnus_ferrugatus_733 | Tytthaspis_sedecimpunctata_583 | 0.198 |
| Scymnus_interruptus_759 | Tytthaspis_sedecimpunctata_586 | 0.198 |
| Scymnus_interruptus_769 | Tytthaspis_sedecimpunctata_586 | 0.198 |
| Calvia_quatuordecimguttata_218 | Scymnus_suturalis_837 | 0.198 |
| Clitostethus_arcuatus_639 | Scymnus_suturalis_837 | 0.198 |
| Clitostethus_arcuatus_640 | Scymnus_suturalis_837 | 0.198 |
| Clitostethus_arcuatus_641 | Scymnus_suturalis_837 | 0.198 |
| Clitostethus_arcuatus_643 | Scymnus_suturalis_837 | 0.198 |
| Calvia_quatuordecimguttata_204 | Scymnus_suturalis_838 | 0.198 |
| Calvia_quatuordecimguttata_208 | Scymnus_suturalis_838 | 0.198 |
| Calvia_quatuordecimguttata_217 | Scymnus_suturalis_838 | 0.198 |
| Calvia_quatuordecimguttata_204 | Scymnus_suturalis_840 | 0.198 |
| Calvia_quatuordecimguttata_208 | Scymnus_suturalis_840 | 0.198 |
| Calvia_quatuordecimguttata_217 | Scymnus_suturalis_840 | 0.198 |
| Platynaspis_luteorubra_52 | Scymnus_sinuanodulus_831 | 0.198 |
| Platynaspis_luteorubra_56 | Scymnus_sinuanodulus_831 | 0.198 |
| Platynaspis_luteorubra_60 | Scymnus_sinuanodulus_831 | 0.198 |
| Platynaspis_luteorubra_54 | Scymnus_sinuanodulus_831 | 0.198 |
| Platynaspis_luteorubra_57 | Scymnus_sinuanodulus_831 | 0.198 |
| Platynaspis_luteorubra_59 | Scymnus_sinuanodulus_831 | 0.198 |
| Hyperaspis_lateralis_654 | Stethorus_punctillum_851 | 0.198 |
| Hyperaspis_reppensis_667 | Stethorus_punctillum_853 | 0.198 |
| Hyperaspis_lateralis_654 | Stethorus_punctillum_858 | 0.198 |
| Platynaspis_luteorubra_51 | Subcoccinella_vigintiquatuorpunctata_627 | 0.198 |
| Platynaspis_luteorubra_53 | Subcoccinella_vigintiquatuorpunctata_627 | 0.198 |
| Platynaspis_luteorubra_58 | Subcoccinella_vigintiquatuorpunctata_627 | 0.198 |
| Platynaspis_luteorubra_51 | Subcoccinella_vigintiquatuorpunctata_630 | 0.198 |
| Platynaspis_luteorubra_53 | Subcoccinella_vigintiquatuorpunctata_630 | 0.198 |
| Platynaspis_luteorubra_58 | Subcoccinella_vigintiquatuorpunctata_630 | 0.198 |
| Scymnus_ferrugatus_729 | Tytthaspis_sedecimpunctata_583 | 0.198 |
| Olla_vnigrum_527 | Tytthaspis_sedecimpunctata_586 | 0.198 |
| Olla_vnigrum_528 | Tytthaspis_sedecimpunctata_586 | 0.198 |

| | | |
|---|---|---|
| Olla_vnigrum_526 | Tytthaspis_sedecimpunctata_587 | 0.198 |
| Scymnus_ferrugatus_724 | Tytthaspis_sedecimpunctata_593 | 0.198 |
| Calvia_quatuordecimguttata_204 | Scymnus_suturalis_837 | 0.198 |
| Calvia_quatuordecimguttata_208 | Scymnus_suturalis_837 | 0.198 |
| Calvia_quatuordecimguttata_217 | Scymnus_suturalis_837 | 0.198 |
| Scymnus_apetzi_690 | Subcoccinella_vigintiquatuorpunctata_619 | 0.198 |
| Scymnus_apetzi_696 | Subcoccinella_vigintiquatuorpunctata_619 | 0.198 |
| Scymnus_apetzi_697 | Subcoccinella_vigintiquatuorpunctata_619 | 0.198 |
| Scymnus_nebulosus_783 | Subcoccinella_vigintiquatuorpunctata_619 | 0.198 |
| Scymnus_pallipediformis_811 | Subcoccinella_vigintiquatuorpunctata_619 | 0.198 |
| Calvia_quatuordecimguttata_205 | Scymnus_suturalis_841 | 0.198 |
| Calvia_quatuordecimguttata_205 | Scymnus_suturalis_842 | 0.198 |
| Calvia_quatuordecimguttata_205 | Scymnus_suturalis_844 | 0.198 |
| Calvia_quatuordecimguttata_205 | Scymnus_suturalis_846 | 0.198 |
| Calvia_quatuordecimguttata_205 | Scymnus_suturalis_847 | 0.198 |
| Calvia_quatuordecimguttata_205 | Scymnus_suturalis_848 | 0.198 |
| Scymnus_ferrugatus_724 | Tytthaspis_sedecimpunctata_584 | 0.198 |
| Chilocorus_renipustulatus_39 | Scymnus_sinuanodulus_830 | 0.198 |
| Coccinella_undecimpunctata_291 | Scymnus_sinuanodulus_830 | 0.198 |
| Calvia_quatuordecimguttata_205 | Scymnus_suturalis_832 | 0.198 |
| Calvia_quatuordecimguttata_205 | Scymnus_suturalis_835 | 0.198 |
| Calvia_quatuordecimguttata_205 | Scymnus_suturalis_836 | 0.198 |
| Calvia_quatuordecimguttata_203 | Scymnus_suturalis_838 | 0.198 |
| Calvia_quatuordecimguttata_205 | Scymnus_suturalis_839 | 0.198 |
| Calvia_quatuordecimguttata_203 | Scymnus_suturalis_840 | 0.198 |
| Scymnus_interruptus_767 | Subcoccinella_vigintiquatuorpunctata_620 | 0.198 |
| Scymnus_interruptus_767 | Subcoccinella_vigintiquatuorpunctata_621 | 0.198 |
| Coccidula_scutellata_83 | Subcoccinella_vigintiquatuorpunctata_623 | 0.198 |
| Scymnus_camptodromus_718 | Subcoccinella_vigintiquatuorpunctata_623 | 0.198 |
| Scymnus_interruptus_760 | Subcoccinella_vigintiquatuorpunctata_624 | 0.198 |
| Scymnus_interruptus_765 | Subcoccinella_vigintiquatuorpunctata_624 | 0.198 |
| Scymnus_interruptus_768 | Subcoccinella_vigintiquatuorpunctata_624 | 0.198 |
| Myzia_pullata_518 | Subcoccinella_vigintiquatuorpunctata_627 | 0.198 |
| Scymnus_interruptus_760 | Subcoccinella_vigintiquatuorpunctata_627 | 0.198 |
| Scymnus_interruptus_765 | Subcoccinella_vigintiquatuorpunctata_627 | 0.198 |
| Scymnus_interruptus_768 | Subcoccinella_vigintiquatuorpunctata_627 | 0.198 |
| Scymnus_interruptus_767 | Subcoccinella_vigintiquatuorpunctata_628 | 0.198 |
| Scymnus_interruptus_767 | Subcoccinella_vigintiquatuorpunctata_629 | 0.198 |
| Myzia_pullata_518 | Subcoccinella_vigintiquatuorpunctata_630 | 0.198 |
| Scymnus_interruptus_767 | Subcoccinella_vigintiquatuorpunctata_631 | 0.198 |
| Scymnus_abietis_684 | Tytthaspis_sedecimpunctata_577 | 0.198 |
| Scymnus_abietis_686 | Tytthaspis_sedecimpunctata_577 | 0.198 |
| Scymnus_abietis_687 | Tytthaspis_sedecimpunctata_577 | 0.198 |
| Scymnus_abietis_688 | Tytthaspis_sedecimpunctata_577 | 0.198 |
| Scymnus_abietis_684 | Tytthaspis_sedecimpunctata_578 | 0.198 |
| Scymnus_abietis_686 | Tytthaspis_sedecimpunctata_578 | 0.198 |
| Scymnus_abietis_687 | Tytthaspis_sedecimpunctata_578 | 0.198 |
| Scymnus_abietis_688 | Tytthaspis_sedecimpunctata_578 | 0.198 |
| Scymnus_abietis_684 | Tytthaspis_sedecimpunctata_579 | 0.198 |
| Scymnus_abietis_686 | Tytthaspis_sedecimpunctata_579 | 0.198 |
| Scymnus_abietis_687 | Tytthaspis_sedecimpunctata_579 | 0.198 |
| Scymnus_abietis_688 | Tytthaspis_sedecimpunctata_579 | 0.198 |
| Scymnus_abietis_684 | Tytthaspis_sedecimpunctata_581 | 0.198 |
| Scymnus_abietis_686 | Tytthaspis_sedecimpunctata_581 | 0.198 |
| Scymnus_abietis_687 | Tytthaspis_sedecimpunctata_581 | 0.198 |
| Scymnus_abietis_688 | Tytthaspis_sedecimpunctata_581 | 0.198 |

| | | |
|---|---|---|
| Scymnus_abietis_684 | Tytthaspis_sedecimpunctata_582 | 0.198 |
| Scymnus_abietis_686 | Tytthaspis_sedecimpunctata_582 | 0.198 |
| Scymnus_abietis_687 | Tytthaspis_sedecimpunctata_582 | 0.198 |
| Scymnus_abietis_688 | Tytthaspis_sedecimpunctata_582 | 0.198 |
| Scymnus_abietis_682 | Tytthaspis_sedecimpunctata_589 | 0.198 |
| Scymnus_abietis_683 | Tytthaspis_sedecimpunctata_589 | 0.198 |
| Scymnus_abietis_685 | Tytthaspis_sedecimpunctata_589 | 0.198 |
| Scymnus_abietis_684 | Tytthaspis_sedecimpunctata_590 | 0.198 |
| Scymnus_abietis_686 | Tytthaspis_sedecimpunctata_590 | 0.198 |
| Scymnus_abietis_687 | Tytthaspis_sedecimpunctata_590 | 0.198 |
| Scymnus_abietis_688 | Tytthaspis_sedecimpunctata_590 | 0.198 |
| Scymnus_abietis_684 | Tytthaspis_sedecimpunctata_592 | 0.198 |
| Scymnus_abietis_686 | Tytthaspis_sedecimpunctata_592 | 0.198 |
| Scymnus_abietis_687 | Tytthaspis_sedecimpunctata_592 | 0.198 |
| Scymnus_abietis_688 | Tytthaspis_sedecimpunctata_592 | 0.198 |
| Scymnus_abietis_684 | Tytthaspis_sedecimpunctata_593 | 0.198 |
| Scymnus_abietis_686 | Tytthaspis_sedecimpunctata_593 | 0.198 |
| Scymnus_abietis_687 | Tytthaspis_sedecimpunctata_593 | 0.198 |
| Scymnus_abietis_688 | Tytthaspis_sedecimpunctata_593 | 0.198 |
| Scymnus_abietis_684 | Tytthaspis_sedecimpunctata_594 | 0.198 |
| Scymnus_abietis_686 | Tytthaspis_sedecimpunctata_594 | 0.198 |
| Scymnus_abietis_687 | Tytthaspis_sedecimpunctata_594 | 0.198 |
| Scymnus_abietis_688 | Tytthaspis_sedecimpunctata_594 | 0.198 |
| Scymnus_abietis_684 | Tytthaspis_sedecimpunctata_595 | 0.198 |
| Scymnus_abietis_686 | Tytthaspis_sedecimpunctata_595 | 0.198 |
| Scymnus_abietis_687 | Tytthaspis_sedecimpunctata_595 | 0.198 |
| Scymnus_abietis_688 | Tytthaspis_sedecimpunctata_595 | 0.198 |
| Hippodamia_undecimnotata_460 | Vibidia_duodecimguttata_597 | 0.198 |
| Hippodamia_undecimnotata_461 | Vibidia_duodecimguttata_597 | 0.198 |
| Hippodamia_undecimnotata_462 | Vibidia_duodecimguttata_597 | 0.198 |
| Hippodamia_undecimnotata_463 | Vibidia_duodecimguttata_597 | 0.198 |
| Hippodamia_undecimnotata_464 | Vibidia_duodecimguttata_597 | 0.198 |
| Hippodamia_undecimnotata_465 | Vibidia_duodecimguttata_597 | 0.198 |
| Hippodamia_undecimnotata_466 | Vibidia_duodecimguttata_597 | 0.198 |
| Hippodamia_undecimnotata_460 | Vibidia_duodecimguttata_600 | 0.198 |
| Hippodamia_undecimnotata_461 | Vibidia_duodecimguttata_600 | 0.198 |
| Hippodamia_undecimnotata_462 | Vibidia_duodecimguttata_600 | 0.198 |
| Hippodamia_undecimnotata_463 | Vibidia_duodecimguttata_600 | 0.198 |
| Hippodamia_undecimnotata_464 | Vibidia_duodecimguttata_600 | 0.198 |
| Hippodamia_undecimnotata_465 | Vibidia_duodecimguttata_600 | 0.198 |
| Hippodamia_undecimnotata_466 | Vibidia_duodecimguttata_600 | 0.198 |
| Calvia_quatuordecimguttata_201 | Scymnus_rubromaculatus_822 | 0.198 |
| Calvia_quatuordecimguttata_201 | Scymnus_rubromaculatus_823 | 0.198 |
| Calvia_quatuordecimguttata_201 | Scymnus_rubromaculatus_824 | 0.198 |
| Calvia_quatuordecimguttata_201 | Scymnus_rubromaculatus_825 | 0.198 |
| Calvia_quatuordecimguttata_201 | Scymnus_rubromaculatus_826 | 0.198 |
| Calvia_quatuordecimguttata_201 | Scymnus_rubromaculatus_827 | 0.198 |
| Coccinella_undecimpunctata_291 | Scymnus_sinuanodulus_828 | 0.198 |
| Calvia_quatuordecimguttata_203 | Scymnus_suturalis_837 | 0.198 |
| Platynaspis_luteorubra_50 | Subcoccinella_vigintiquatuorpunctata_619 | 0.198 |
| Hyperaspis_lateralis_655 | Scymnus_sinuanodulus_831 | 0.198 |
| Scymnus_camptodromus_712 | Subcoccinella_vigintiquatuorpunctata_620 | 0.198 |
| Scymnus_camptodromus_712 | Subcoccinella_vigintiquatuorpunctata_621 | 0.198 |
| Scymnus_camptodromus_718 | Subcoccinella_vigintiquatuorpunctata_622 | 0.198 |
| Scymnus_interruptus_767 | Subcoccinella_vigintiquatuorpunctata_623 | 0.198 |
| Coccidula_scutellata_78 | Subcoccinella_vigintiquatuorpunctata_624 | 0.198 |

| | | |
|---|---|---|
| Coccidula_scutellata_79 | Subcoccinella_vigintiquatuorpunctata_624 | 0.198 |
| Coccidula_scutellata_83 | Subcoccinella_vigintiquatuorpunctata_624 | 0.198 |
| Coccidula_scutellata_80 | Subcoccinella_vigintiquatuorpunctata_625 | 0.198 |
| Coccidula_scutellata_83 | Subcoccinella_vigintiquatuorpunctata_626 | 0.198 |
| Scymnus_camptodromus_718 | Subcoccinella_vigintiquatuorpunctata_626 | 0.198 |
| Scymnus_camptodromus_712 | Subcoccinella_vigintiquatuorpunctata_627 | 0.198 |
| Scymnus_camptodromus_712 | Subcoccinella_vigintiquatuorpunctata_628 | 0.198 |
| Scymnus_camptodromus_712 | Subcoccinella_vigintiquatuorpunctata_629 | 0.198 |
| Scymnus_camptodromus_712 | Subcoccinella_vigintiquatuorpunctata_630 | 0.198 |
| Scymnus_camptodromus_712 | Subcoccinella_vigintiquatuorpunctata_631 | 0.198 |
| Scymnus_abietis_684 | Tytthaspis_sedecimpunctata_580 | 0.198 |
| Scymnus_abietis_686 | Tytthaspis_sedecimpunctata_580 | 0.198 |
| Scymnus_abietis_687 | Tytthaspis_sedecimpunctata_580 | 0.198 |
| Scymnus_abietis_688 | Tytthaspis_sedecimpunctata_580 | 0.198 |
| Scymnus_abietis_682 | Tytthaspis_sedecimpunctata_584 | 0.198 |
| Scymnus_abietis_683 | Tytthaspis_sedecimpunctata_584 | 0.198 |
| Scymnus_abietis_685 | Tytthaspis_sedecimpunctata_584 | 0.198 |
| Scymnus_abietis_685 | Tytthaspis_sedecimpunctata_587 | 0.198 |
| Hippodamia_parenthesis_433 | Vibidia_duodecimguttata_598 | 0.198 |
| Hippodamia_parenthesis_433 | Vibidia_duodecimguttata_599 | 0.198 |
| Hippodamia_undecimnotata_460 | Vibidia_duodecimguttata_599 | 0.198 |
| Hippodamia_undecimnotata_461 | Vibidia_duodecimguttata_599 | 0.198 |
| Hippodamia_undecimnotata_462 | Vibidia_duodecimguttata_599 | 0.198 |
| Hippodamia_undecimnotata_463 | Vibidia_duodecimguttata_599 | 0.198 |
| Hippodamia_undecimnotata_464 | Vibidia_duodecimguttata_599 | 0.198 |
| Hippodamia_undecimnotata_465 | Vibidia_duodecimguttata_599 | 0.198 |
| Hippodamia_undecimnotata_466 | Vibidia_duodecimguttata_599 | 0.198 |
| Hippodamia_parenthesis_433 | Vibidia_duodecimguttata_601 | 0.198 |
| Hippodamia_undecimnotata_460 | Vibidia_duodecimguttata_601 | 0.198 |
| Hippodamia_undecimnotata_461 | Vibidia_duodecimguttata_601 | 0.198 |
| Hippodamia_undecimnotata_462 | Vibidia_duodecimguttata_601 | 0.198 |
| Hippodamia_undecimnotata_463 | Vibidia_duodecimguttata_601 | 0.198 |
| Hippodamia_undecimnotata_464 | Vibidia_duodecimguttata_601 | 0.198 |
| Hippodamia_undecimnotata_465 | Vibidia_duodecimguttata_601 | 0.198 |
| Hippodamia_undecimnotata_466 | Vibidia_duodecimguttata_601 | 0.198 |
| Hippodamia_parenthesis_433 | Vibidia_duodecimguttata_602 | 0.198 |
| Hippodamia_undecimnotata_460 | Vibidia_duodecimguttata_602 | 0.198 |
| Hippodamia_undecimnotata_461 | Vibidia_duodecimguttata_602 | 0.198 |
| Hippodamia_undecimnotata_462 | Vibidia_duodecimguttata_602 | 0.198 |
| Hippodamia_undecimnotata_463 | Vibidia_duodecimguttata_602 | 0.198 |
| Hippodamia_undecimnotata_464 | Vibidia_duodecimguttata_602 | 0.198 |
| Hippodamia_undecimnotata_465 | Vibidia_duodecimguttata_602 | 0.198 |
| Hippodamia_undecimnotata_466 | Vibidia_duodecimguttata_602 | 0.198 |
| Hippodamia_parenthesis_432 | Vibidia_duodecimguttata_603 | 0.198 |
| Hippodamia_parenthesis_434 | Vibidia_duodecimguttata_603 | 0.198 |
| Hippodamia_parenthesis_435 | Vibidia_duodecimguttata_603 | 0.198 |
| Hippodamia_parenthesis_437 | Vibidia_duodecimguttata_603 | 0.198 |
| Hippodamia_parenthesis_438 | Vibidia_duodecimguttata_603 | 0.198 |
| Hippodamia_parenthesis_440 | Vibidia_duodecimguttata_603 | 0.198 |
| Hippodamia_parenthesis_441 | Vibidia_duodecimguttata_603 | 0.198 |
| Hippodamia_parenthesis_442 | Vibidia_duodecimguttata_603 | 0.198 |
| Hippodamia_convergens_395 | Scymnus_suturalis_833 | 0.198 |
| Hippodamia_convergens_396 | Scymnus_suturalis_833 | 0.198 |
| Hippodamia_convergens_403 | Scymnus_suturalis_833 | 0.198 |
| Hippodamia_moesta_410 | Scymnus_suturalis_833 | 0.198 |
| Hippodamia_convergens_393 | Scymnus_suturalis_838 | 0.198 |

| | | |
|---|---|---|
| Hippodamia_convergens_394 | Scymnus_suturalis_838 | 0.198 |
| Hippodamia_convergens_397 | Scymnus_suturalis_838 | 0.198 |
| Hippodamia_convergens_398 | Scymnus_suturalis_838 | 0.198 |
| Hippodamia_convergens_401 | Scymnus_suturalis_838 | 0.198 |
| Hippodamia_convergens_402 | Scymnus_suturalis_838 | 0.198 |
| Hippodamia_convergens_393 | Scymnus_suturalis_840 | 0.198 |
| Hippodamia_convergens_394 | Scymnus_suturalis_840 | 0.198 |
| Hippodamia_convergens_397 | Scymnus_suturalis_840 | 0.198 |
| Hippodamia_convergens_398 | Scymnus_suturalis_840 | 0.198 |
| Hippodamia_convergens_401 | Scymnus_suturalis_840 | 0.198 |
| Hippodamia_convergens_402 | Scymnus_suturalis_840 | 0.198 |
| Chilocorus_renipustulatus_34 | Scymnus_sinuanodulus_831 | 0.198 |
| Psyllobora_renifer_551 | Vibidia_duodecimguttata_597 | 0.198 |
| Psyllobora_renifer_552 | Vibidia_duodecimguttata_597 | 0.198 |
| Psyllobora_renifer_553 | Vibidia_duodecimguttata_597 | 0.198 |
| Psyllobora_renifer_554 | Vibidia_duodecimguttata_597 | 0.198 |
| Psyllobora_renifer_551 | Vibidia_duodecimguttata_600 | 0.198 |
| Psyllobora_renifer_552 | Vibidia_duodecimguttata_600 | 0.198 |
| Psyllobora_renifer_553 | Vibidia_duodecimguttata_600 | 0.198 |
| Psyllobora_renifer_554 | Vibidia_duodecimguttata_600 | 0.198 |
| Coccinella_undecimpunctata_289 | Scymnus_sinuanodulus_830 | 0.198 |
| Coccinella_undecimpunctata_290 | Scymnus_sinuanodulus_830 | 0.198 |
| Chilocorus_renipustulatus_31 | Scymnus_sinuanodulus_831 | 0.198 |
| Chilocorus_renipustulatus_32 | Scymnus_sinuanodulus_831 | 0.198 |
| Chilocorus_renipustulatus_35 | Scymnus_sinuanodulus_831 | 0.198 |
| Chilocorus_renipustulatus_36 | Scymnus_sinuanodulus_831 | 0.198 |
| Chilocorus_renipustulatus_37 | Scymnus_sinuanodulus_831 | 0.198 |
| Chilocorus_renipustulatus_38 | Scymnus_sinuanodulus_831 | 0.198 |
| Chilocorus_renipustulatus_40 | Scymnus_sinuanodulus_831 | 0.198 |
| Chilocorus_renipustulatus_41 | Scymnus_sinuanodulus_831 | 0.198 |
| Chilocorus_renipustulatus_42 | Scymnus_sinuanodulus_831 | 0.198 |
| Hippodamia_convergens_399 | Scymnus_suturalis_841 | 0.198 |
| Hippodamia_convergens_404 | Scymnus_suturalis_841 | 0.198 |
| Hippodamia_convergens_405 | Scymnus_suturalis_841 | 0.198 |
| Hippodamia_convergens_406 | Scymnus_suturalis_841 | 0.198 |
| Hippodamia_quinquesignata_443 | Scymnus_suturalis_841 | 0.198 |
| Hippodamia_quinquesignata_444 | Scymnus_suturalis_841 | 0.198 |
| Hippodamia_quinquesignata_445 | Scymnus_suturalis_841 | 0.198 |
| Hippodamia_convergens_399 | Scymnus_suturalis_842 | 0.198 |
| Hippodamia_convergens_404 | Scymnus_suturalis_842 | 0.198 |
| Hippodamia_convergens_405 | Scymnus_suturalis_842 | 0.198 |
| Hippodamia_convergens_406 | Scymnus_suturalis_842 | 0.198 |
| Hippodamia_quinquesignata_443 | Scymnus_suturalis_842 | 0.198 |
| Hippodamia_quinquesignata_444 | Scymnus_suturalis_842 | 0.198 |
| Hippodamia_quinquesignata_445 | Scymnus_suturalis_842 | 0.198 |
| Hippodamia_caseyi_391 | Scymnus_suturalis_843 | 0.198 |
| Hippodamia_moesta_411 | Scymnus_suturalis_843 | 0.198 |
| Hippodamia_convergens_399 | Scymnus_suturalis_844 | 0.198 |
| Hippodamia_convergens_404 | Scymnus_suturalis_844 | 0.198 |
| Hippodamia_convergens_405 | Scymnus_suturalis_844 | 0.198 |
| Hippodamia_convergens_406 | Scymnus_suturalis_844 | 0.198 |
| Hippodamia_quinquesignata_443 | Scymnus_suturalis_844 | 0.198 |
| Hippodamia_quinquesignata_444 | Scymnus_suturalis_844 | 0.198 |
| Hippodamia_quinquesignata_445 | Scymnus_suturalis_844 | 0.198 |
| Hippodamia_caseyi_391 | Scymnus_suturalis_845 | 0.198 |
| Hippodamia_moesta_411 | Scymnus_suturalis_845 | 0.198 |

Supplementary Table S4 (continued)

| | | |
|---|---|---|
| Hippodamia_convergens_399 | Scymnus_suturalis_846 | 0.198 |
| Hippodamia_convergens_404 | Scymnus_suturalis_846 | 0.198 |
| Hippodamia_convergens_405 | Scymnus_suturalis_846 | 0.198 |
| Hippodamia_convergens_406 | Scymnus_suturalis_846 | 0.198 |
| Hippodamia_quinquesignata_443 | Scymnus_suturalis_846 | 0.198 |
| Hippodamia_quinquesignata_444 | Scymnus_suturalis_846 | 0.198 |
| Hippodamia_quinquesignata_445 | Scymnus_suturalis_846 | 0.198 |
| Hippodamia_convergens_399 | Scymnus_suturalis_847 | 0.198 |
| Hippodamia_convergens_404 | Scymnus_suturalis_847 | 0.198 |
| Hippodamia_convergens_405 | Scymnus_suturalis_847 | 0.198 |
| Hippodamia_convergens_406 | Scymnus_suturalis_847 | 0.198 |
| Hippodamia_quinquesignata_443 | Scymnus_suturalis_847 | 0.198 |
| Hippodamia_quinquesignata_444 | Scymnus_suturalis_847 | 0.198 |
| Hippodamia_quinquesignata_445 | Scymnus_suturalis_847 | 0.198 |
| Hippodamia_convergens_399 | Scymnus_suturalis_848 | 0.198 |
| Hippodamia_convergens_404 | Scymnus_suturalis_848 | 0.198 |
| Hippodamia_convergens_405 | Scymnus_suturalis_848 | 0.198 |
| Hippodamia_convergens_406 | Scymnus_suturalis_848 | 0.198 |
| Hippodamia_quinquesignata_443 | Scymnus_suturalis_848 | 0.198 |
| Hippodamia_quinquesignata_444 | Scymnus_suturalis_848 | 0.198 |
| Hippodamia_quinquesignata_445 | Scymnus_suturalis_848 | 0.198 |
| Scymnus_ferrugatus_729 | Subcoccinella_vigintiquatuorpunctata_620 | 0.198 |
| Scymnus_ferrugatus_729 | Subcoccinella_vigintiquatuorpunctata_621 | 0.198 |
| Scymnus_camptodromus_705 | Subcoccinella_vigintiquatuorpunctata_627 | 0.198 |
| Scymnus_ferrugatus_729 | Subcoccinella_vigintiquatuorpunctata_628 | 0.198 |
| Scymnus_ferrugatus_729 | Subcoccinella_vigintiquatuorpunctata_629 | 0.198 |
| Scymnus_camptodromus_705 | Subcoccinella_vigintiquatuorpunctata_630 | 0.198 |
| Scymnus_ferrugatus_729 | Subcoccinella_vigintiquatuorpunctata_631 | 0.198 |
| Chilocorus_nigritus_25 | Vibidia_duodecimguttata_596 | 0.198 |
| Mulsantina_hudsonica_481 | Vibidia_duodecimguttata_597 | 0.198 |
| Adalia_bipunctata_98 | Vibidia_duodecimguttata_599 | 0.198 |
| Chilocorus_nigritus_25 | Vibidia_duodecimguttata_599 | 0.198 |
| Mulsantina_hudsonica_481 | Vibidia_duodecimguttata_600 | 0.198 |
| Adalia_bipunctata_98 | Vibidia_duodecimguttata_601 | 0.198 |
| Adalia_bipunctata_98 | Vibidia_duodecimguttata_602 | 0.198 |
| Chilocorus_nigritus_25 | Vibidia_duodecimguttata_602 | 0.198 |
| Chilocorus_nigritus_25 | Vibidia_duodecimguttata_603 | 0.198 |
| Coccidula_rufa_63 | Scymnus_rubromaculatus_822 | 0.198 |
| Coccidula_rufa_63 | Scymnus_rubromaculatus_823 | 0.198 |
| Coccidula_rufa_63 | Scymnus_rubromaculatus_824 | 0.198 |
| Coccidula_rufa_63 | Scymnus_rubromaculatus_825 | 0.198 |
| Coccidula_rufa_63 | Scymnus_rubromaculatus_826 | 0.198 |
| Coccidula_rufa_63 | Scymnus_rubromaculatus_827 | 0.198 |
| Clitostethus_arcuatus_642 | Scymnus_sinuanodulus_830 | 0.198 |
| Hippodamia_convergens_399 | Scymnus_suturalis_832 | 0.198 |
| Hippodamia_convergens_404 | Scymnus_suturalis_832 | 0.198 |
| Hippodamia_convergens_405 | Scymnus_suturalis_832 | 0.198 |
| Hippodamia_convergens_406 | Scymnus_suturalis_832 | 0.198 |
| Hippodamia_quinquesignata_443 | Scymnus_suturalis_832 | 0.198 |
| Hippodamia_quinquesignata_444 | Scymnus_suturalis_832 | 0.198 |
| Hippodamia_quinquesignata_445 | Scymnus_suturalis_832 | 0.198 |
| Hippodamia_convergens_393 | Scymnus_suturalis_834 | 0.198 |
| Hippodamia_convergens_394 | Scymnus_suturalis_834 | 0.198 |
| Hippodamia_convergens_397 | Scymnus_suturalis_834 | 0.198 |
| Hippodamia_convergens_398 | Scymnus_suturalis_834 | 0.198 |
| Hippodamia_convergens_401 | Scymnus_suturalis_834 | 0.198 |

| | | |
|---|---|---|
| Hippodamia_convergens_402 | Scymnus_suturalis_834 | 0.198 |
| Hippodamia_convergens_399 | Scymnus_suturalis_835 | 0.198 |
| Hippodamia_convergens_404 | Scymnus_suturalis_835 | 0.198 |
| Hippodamia_convergens_405 | Scymnus_suturalis_835 | 0.198 |
| Hippodamia_convergens_406 | Scymnus_suturalis_835 | 0.198 |
| Hippodamia_quinquesignata_443 | Scymnus_suturalis_835 | 0.198 |
| Hippodamia_quinquesignata_444 | Scymnus_suturalis_835 | 0.198 |
| Hippodamia_quinquesignata_445 | Scymnus_suturalis_835 | 0.198 |
| Hippodamia_convergens_399 | Scymnus_suturalis_836 | 0.198 |
| Hippodamia_convergens_404 | Scymnus_suturalis_836 | 0.198 |
| Hippodamia_convergens_405 | Scymnus_suturalis_836 | 0.198 |
| Hippodamia_convergens_406 | Scymnus_suturalis_836 | 0.198 |
| Hippodamia_quinquesignata_443 | Scymnus_suturalis_836 | 0.198 |
| Hippodamia_quinquesignata_444 | Scymnus_suturalis_836 | 0.198 |
| Hippodamia_quinquesignata_445 | Scymnus_suturalis_836 | 0.198 |
| Hippodamia_convergens_393 | Scymnus_suturalis_837 | 0.198 |
| Hippodamia_convergens_394 | Scymnus_suturalis_837 | 0.198 |
| Hippodamia_convergens_397 | Scymnus_suturalis_837 | 0.198 |
| Hippodamia_convergens_398 | Scymnus_suturalis_837 | 0.198 |
| Hippodamia_convergens_401 | Scymnus_suturalis_837 | 0.198 |
| Hippodamia_convergens_402 | Scymnus_suturalis_837 | 0.198 |
| Hippodamia_convergens_399 | Scymnus_suturalis_839 | 0.198 |
| Hippodamia_convergens_404 | Scymnus_suturalis_839 | 0.198 |
| Hippodamia_convergens_405 | Scymnus_suturalis_839 | 0.198 |
| Hippodamia_convergens_406 | Scymnus_suturalis_839 | 0.198 |
| Hippodamia_quinquesignata_443 | Scymnus_suturalis_839 | 0.198 |
| Hippodamia_quinquesignata_444 | Scymnus_suturalis_839 | 0.198 |
| Hippodamia_quinquesignata_445 | Scymnus_suturalis_839 | 0.198 |
| Myrrha_octodecimguttata_489 | Scymnus_sinuanodulus_829 | 0.198 |
| Myrrha_octodecimguttata_493 | Scymnus_sinuanodulus_829 | 0.198 |
| Myrrha_octodecimguttata_498 | Scymnus_sinuanodulus_829 | 0.198 |
| Myrrha_octodecimguttata_500 | Scymnus_sinuanodulus_829 | 0.198 |
| Scymnus_apetzi_695 | Tytthaspis_sedecimpunctata_583 | 0.198 |
| Scymnus_apetzi_695 | Tytthaspis_sedecimpunctata_589 | 0.198 |
| Scymnus_apetzi_695 | Tytthaspis_sedecimpunctata_592 | 0.198 |
| Coccinella_undecimpunctata_289 | Scymnus_sinuanodulus_828 | 0.198 |
| Coccinella_undecimpunctata_290 | Scymnus_sinuanodulus_828 | 0.198 |
| Scymnus_ferrugatus_723 | Subcoccinella_vigintiquatuorpunctata_620 | 0.199 |
| Scymnus_ferrugatus_725 | Subcoccinella_vigintiquatuorpunctata_620 | 0.199 |
| Scymnus_ferrugatus_726 | Subcoccinella_vigintiquatuorpunctata_620 | 0.199 |
| Scymnus_ferrugatus_727 | Subcoccinella_vigintiquatuorpunctata_620 | 0.199 |
| Scymnus_ferrugatus_728 | Subcoccinella_vigintiquatuorpunctata_620 | 0.199 |
| Scymnus_ferrugatus_730 | Subcoccinella_vigintiquatuorpunctata_620 | 0.199 |
| Scymnus_ferrugatus_732 | Subcoccinella_vigintiquatuorpunctata_620 | 0.199 |
| Scymnus_ferrugatus_733 | Subcoccinella_vigintiquatuorpunctata_620 | 0.199 |
| Scymnus_ferrugatus_723 | Subcoccinella_vigintiquatuorpunctata_621 | 0.199 |
| Scymnus_ferrugatus_725 | Subcoccinella_vigintiquatuorpunctata_621 | 0.199 |
| Scymnus_ferrugatus_726 | Subcoccinella_vigintiquatuorpunctata_621 | 0.199 |
| Scymnus_ferrugatus_727 | Subcoccinella_vigintiquatuorpunctata_621 | 0.199 |
| Scymnus_ferrugatus_728 | Subcoccinella_vigintiquatuorpunctata_621 | 0.199 |
| Scymnus_ferrugatus_730 | Subcoccinella_vigintiquatuorpunctata_621 | 0.199 |
| Scymnus_ferrugatus_732 | Subcoccinella_vigintiquatuorpunctata_621 | 0.199 |
| Scymnus_ferrugatus_733 | Subcoccinella_vigintiquatuorpunctata_621 | 0.199 |
| Scymnus_ferrugatus_729 | Subcoccinella_vigintiquatuorpunctata_623 | 0.199 |
| Scymnus_ferrugatus_723 | Subcoccinella_vigintiquatuorpunctata_628 | 0.199 |
| Scymnus_ferrugatus_725 | Subcoccinella_vigintiquatuorpunctata_628 | 0.199 |

Supplementary Table S4 (continued)

| | | |
|---|---|---|
| Scymnus_ferrugatus_726 | Subcoccinella_vigintiquatuorpunctata_628 | 0.199 |
| Scymnus_ferrugatus_727 | Subcoccinella_vigintiquatuorpunctata_628 | 0.199 |
| Scymnus_ferrugatus_728 | Subcoccinella_vigintiquatuorpunctata_628 | 0.199 |
| Scymnus_ferrugatus_730 | Subcoccinella_vigintiquatuorpunctata_628 | 0.199 |
| Scymnus_ferrugatus_732 | Subcoccinella_vigintiquatuorpunctata_628 | 0.199 |
| Scymnus_ferrugatus_733 | Subcoccinella_vigintiquatuorpunctata_628 | 0.199 |
| Scymnus_ferrugatus_723 | Subcoccinella_vigintiquatuorpunctata_629 | 0.199 |
| Scymnus_ferrugatus_725 | Subcoccinella_vigintiquatuorpunctata_629 | 0.199 |
| Scymnus_ferrugatus_726 | Subcoccinella_vigintiquatuorpunctata_629 | 0.199 |
| Scymnus_ferrugatus_727 | Subcoccinella_vigintiquatuorpunctata_629 | 0.199 |
| Scymnus_ferrugatus_728 | Subcoccinella_vigintiquatuorpunctata_629 | 0.199 |
| Scymnus_ferrugatus_730 | Subcoccinella_vigintiquatuorpunctata_629 | 0.199 |
| Scymnus_ferrugatus_732 | Subcoccinella_vigintiquatuorpunctata_629 | 0.199 |
| Scymnus_ferrugatus_733 | Subcoccinella_vigintiquatuorpunctata_629 | 0.199 |
| Brachiacantha_ursina_637 | Subcoccinella_vigintiquatuorpunctata_630 | 0.199 |
| Scymnus_ferrugatus_723 | Subcoccinella_vigintiquatuorpunctata_631 | 0.199 |
| Scymnus_ferrugatus_725 | Subcoccinella_vigintiquatuorpunctata_631 | 0.199 |
| Scymnus_ferrugatus_726 | Subcoccinella_vigintiquatuorpunctata_631 | 0.199 |
| Scymnus_ferrugatus_727 | Subcoccinella_vigintiquatuorpunctata_631 | 0.199 |
| Scymnus_ferrugatus_728 | Subcoccinella_vigintiquatuorpunctata_631 | 0.199 |
| Scymnus_ferrugatus_730 | Subcoccinella_vigintiquatuorpunctata_631 | 0.199 |
| Scymnus_ferrugatus_732 | Subcoccinella_vigintiquatuorpunctata_631 | 0.199 |
| Scymnus_ferrugatus_733 | Subcoccinella_vigintiquatuorpunctata_631 | 0.199 |
| Adalia_bipunctata_101 | Vibidia_duodecimguttata_597 | 0.199 |
| Adalia_bipunctata_104 | Vibidia_duodecimguttata_597 | 0.199 |
| Chilocorus_nigritus_25 | Vibidia_duodecimguttata_597 | 0.199 |
| Adalia_bipunctata_105 | Vibidia_duodecimguttata_599 | 0.199 |
| Adalia_bipunctata_99 | Vibidia_duodecimguttata_599 | 0.199 |
| Mulsantina_hudsonica_482 | Vibidia_duodecimguttata_599 | 0.199 |
| Adalia_bipunctata_101 | Vibidia_duodecimguttata_600 | 0.199 |
| Adalia_bipunctata_104 | Vibidia_duodecimguttata_600 | 0.199 |
| Chilocorus_nigritus_25 | Vibidia_duodecimguttata_600 | 0.199 |
| Adalia_bipunctata_105 | Vibidia_duodecimguttata_601 | 0.199 |
| Adalia_bipunctata_99 | Vibidia_duodecimguttata_601 | 0.199 |
| Adalia_bipunctata_105 | Vibidia_duodecimguttata_602 | 0.199 |
| Adalia_bipunctata_99 | Vibidia_duodecimguttata_602 | 0.199 |
| Mulsantina_hudsonica_482 | Vibidia_duodecimguttata_602 | 0.199 |
| Mulsantina_hudsonica_479 | Vibidia_duodecimguttata_603 | 0.199 |
| Psyllobora_renifer_551 | Vibidia_duodecimguttata_603 | 0.199 |
| Psyllobora_renifer_552 | Vibidia_duodecimguttata_603 | 0.199 |
| Psyllobora_renifer_553 | Vibidia_duodecimguttata_603 | 0.199 |
| Psyllobora_renifer_554 | Vibidia_duodecimguttata_603 | 0.199 |
| Psyllobora_vigintiduopunctata_558 | Subcoccinella_vigintiquatuorpunctata_624 | 0.199 |
| Psyllobora_vigintiduopunctata_558 | Subcoccinella_vigintiquatuorpunctata_630 | 0.199 |
| Scymnus_apetzi_695 | Tytthaspis_sedecimpunctata_580 | 0.199 |
| Scymnus_apetzi_693 | Tytthaspis_sedecimpunctata_583 | 0.199 |
| Scymnus_apetzi_693 | Tytthaspis_sedecimpunctata_585 | 0.199 |
| Scymnus_apetzi_695 | Tytthaspis_sedecimpunctata_588 | 0.199 |
| Scymnus_apetzi_693 | Tytthaspis_sedecimpunctata_592 | 0.199 |
| Platynaspis_luteorubra_52 | Scymnus_sinuanodulus_829 | 0.199 |
| Platynaspis_luteorubra_56 | Scymnus_sinuanodulus_829 | 0.199 |
| Platynaspis_luteorubra_60 | Scymnus_sinuanodulus_829 | 0.199 |
| Scymnus_apetzi_693 | Tytthaspis_sedecimpunctata_580 | 0.199 |
| Platynaspis_luteorubra_54 | Scymnus_sinuanodulus_829 | 0.199 |
| Platynaspis_luteorubra_57 | Scymnus_sinuanodulus_829 | 0.199 |
| Platynaspis_luteorubra_59 | Scymnus_sinuanodulus_829 | 0.199 |

| | | |
|---|---|---|
| Scymnus_camptodromus_718 | Subcoccinella_vigintiquatuorpunctata_619 | 0.199 |
| Scymnus_abietis_689 | Tytthaspis_sedecimpunctata_589 | 0.199 |
| Hippodamia_parenthesis_436 | Vibidia_duodecimguttata_603 | 0.199 |
| Psyllobora_vigintiduopunctata_568 | Subcoccinella_vigintiquatuorpunctata_620 | 0.199 |
| Psyllobora_vigintiduopunctata_568 | Subcoccinella_vigintiquatuorpunctata_621 | 0.199 |
| Psyllobora_vigintiduopunctata_568 | Subcoccinella_vigintiquatuorpunctata_627 | 0.199 |
| Psyllobora_vigintiduopunctata_568 | Subcoccinella_vigintiquatuorpunctata_628 | 0.199 |
| Psyllobora_vigintiduopunctata_568 | Subcoccinella_vigintiquatuorpunctata_629 | 0.199 |
| Psyllobora_vigintiduopunctata_568 | Subcoccinella_vigintiquatuorpunctata_631 | 0.199 |
| Scymnus_abietis_682 | Subcoccinella_vigintiquatuorpunctata_620 | 0.199 |
| Scymnus_abietis_685 | Subcoccinella_vigintiquatuorpunctata_620 | 0.199 |
| Scymnus_abietis_682 | Subcoccinella_vigintiquatuorpunctata_621 | 0.199 |
| Scymnus_abietis_685 | Subcoccinella_vigintiquatuorpunctata_621 | 0.199 |
| Scymnus_abietis_683 | Subcoccinella_vigintiquatuorpunctata_622 | 0.199 |
| Scymnus_abietis_684 | Subcoccinella_vigintiquatuorpunctata_622 | 0.199 |
| Scymnus_abietis_686 | Subcoccinella_vigintiquatuorpunctata_622 | 0.199 |
| Scymnus_abietis_687 | Subcoccinella_vigintiquatuorpunctata_622 | 0.199 |
| Scymnus_abietis_688 | Subcoccinella_vigintiquatuorpunctata_622 | 0.199 |
| Scymnus_ferrugatus_723 | Subcoccinella_vigintiquatuorpunctata_623 | 0.199 |
| Scymnus_ferrugatus_725 | Subcoccinella_vigintiquatuorpunctata_623 | 0.199 |
| Scymnus_ferrugatus_726 | Subcoccinella_vigintiquatuorpunctata_623 | 0.199 |
| Scymnus_ferrugatus_727 | Subcoccinella_vigintiquatuorpunctata_623 | 0.199 |
| Scymnus_ferrugatus_728 | Subcoccinella_vigintiquatuorpunctata_623 | 0.199 |
| Scymnus_ferrugatus_730 | Subcoccinella_vigintiquatuorpunctata_623 | 0.199 |
| Scymnus_ferrugatus_732 | Subcoccinella_vigintiquatuorpunctata_623 | 0.199 |
| Scymnus_ferrugatus_733 | Subcoccinella_vigintiquatuorpunctata_623 | 0.199 |
| Calvia_quatuordecimguttata_211 | Subcoccinella_vigintiquatuorpunctata_627 | 0.199 |
| Calvia_quatuordecimguttata_212 | Subcoccinella_vigintiquatuorpunctata_627 | 0.199 |
| Calvia_quatuordecimguttata_213 | Subcoccinella_vigintiquatuorpunctata_627 | 0.199 |
| Calvia_quatuordecimguttata_214 | Subcoccinella_vigintiquatuorpunctata_627 | 0.199 |
| Calvia_quatuordecimguttata_215 | Subcoccinella_vigintiquatuorpunctata_627 | 0.199 |
| Scymnus_abietis_682 | Subcoccinella_vigintiquatuorpunctata_627 | 0.199 |
| Scymnus_abietis_685 | Subcoccinella_vigintiquatuorpunctata_627 | 0.199 |
| Scymnus_abietis_682 | Subcoccinella_vigintiquatuorpunctata_628 | 0.199 |
| Scymnus_abietis_685 | Subcoccinella_vigintiquatuorpunctata_628 | 0.199 |
| Scymnus_abietis_682 | Subcoccinella_vigintiquatuorpunctata_629 | 0.199 |
| Scymnus_abietis_685 | Subcoccinella_vigintiquatuorpunctata_629 | 0.199 |
| Calvia_quatuordecimguttata_211 | Subcoccinella_vigintiquatuorpunctata_630 | 0.199 |
| Calvia_quatuordecimguttata_212 | Subcoccinella_vigintiquatuorpunctata_630 | 0.199 |
| Calvia_quatuordecimguttata_213 | Subcoccinella_vigintiquatuorpunctata_630 | 0.199 |
| Calvia_quatuordecimguttata_214 | Subcoccinella_vigintiquatuorpunctata_630 | 0.199 |
| Calvia_quatuordecimguttata_215 | Subcoccinella_vigintiquatuorpunctata_630 | 0.199 |
| Scymnus_abietis_682 | Subcoccinella_vigintiquatuorpunctata_630 | 0.199 |
| Scymnus_abietis_685 | Subcoccinella_vigintiquatuorpunctata_630 | 0.199 |
| Scymnus_abietis_682 | Subcoccinella_vigintiquatuorpunctata_631 | 0.199 |
| Scymnus_abietis_685 | Subcoccinella_vigintiquatuorpunctata_631 | 0.199 |
| Harmonia_quadripunctata_377 | Vibidia_duodecimguttata_596 | 0.199 |
| Harmonia_quadripunctata_379 | Vibidia_duodecimguttata_596 | 0.199 |
| Harmonia_quadripunctata_381 | Vibidia_duodecimguttata_596 | 0.199 |
| Chilocorus_nigritus_27 | Vibidia_duodecimguttata_598 | 0.199 |
| Harmonia_quadripunctata_377 | Vibidia_duodecimguttata_598 | 0.199 |
| Harmonia_quadripunctata_379 | Vibidia_duodecimguttata_598 | 0.199 |
| Harmonia_quadripunctata_381 | Vibidia_duodecimguttata_598 | 0.199 |
| Chilocorus_nigritus_27 | Vibidia_duodecimguttata_601 | 0.199 |
| Harmonia_quadripunctata_380 | Vibidia_duodecimguttata_603 | 0.199 |
| Oenopia_conglobata_523 | Scymnus_suturalis_833 | 0.199 |

| | | |
|---|---|---|
| Psyllobora_renifer_555 | Vibidia_duodecimguttata_596 | 0.199 |
| Psyllobora_renifer_555 | Vibidia_duodecimguttata_598 | 0.199 |
| Psyllobora_renifer_555 | Vibidia_duodecimguttata_599 | 0.199 |
| Psyllobora_renifer_555 | Vibidia_duodecimguttata_601 | 0.199 |
| Psyllobora_renifer_555 | Vibidia_duodecimguttata_602 | 0.199 |
| Scymnus_abietis_689 | Tytthaspis_sedecimpunctata_584 | 0.199 |
| Clitostethus_arcuatus_639 | Scymnus_sinuanodulus_831 | 0.199 |
| Clitostethus_arcuatus_640 | Scymnus_sinuanodulus_831 | 0.199 |
| Clitostethus_arcuatus_641 | Scymnus_sinuanodulus_831 | 0.199 |
| Clitostethus_arcuatus_643 | Scymnus_sinuanodulus_831 | 0.199 |
| Oenopia_conglobata_523 | Scymnus_suturalis_841 | 0.199 |
| Oenopia_conglobata_523 | Scymnus_suturalis_842 | 0.199 |
| Oenopia_conglobata_523 | Scymnus_suturalis_844 | 0.199 |
| Oenopia_conglobata_523 | Scymnus_suturalis_846 | 0.199 |
| Oenopia_conglobata_523 | Scymnus_suturalis_847 | 0.199 |
| Oenopia_conglobata_523 | Scymnus_suturalis_848 | 0.199 |
| Neoharmonia_venusta_521 | Vibidia_duodecimguttata_597 | 0.199 |
| Neoharmonia_venusta_521 | Vibidia_duodecimguttata_600 | 0.199 |
| Oenopia_conglobata_523 | Scymnus_suturalis_832 | 0.199 |
| Oenopia_conglobata_523 | Scymnus_suturalis_835 | 0.199 |
| Oenopia_conglobata_523 | Scymnus_suturalis_836 | 0.199 |
| Oenopia_conglobata_523 | Scymnus_suturalis_837 | 0.199 |
| Oenopia_conglobata_523 | Scymnus_suturalis_839 | 0.199 |
| Scymnus_camptodromus_711 | Subcoccinella_vigintiquatuorpunctata_624 | 0.199 |
| Chilocorus_renipustulatus_34 | Scymnus_sinuanodulus_829 | 0.199 |
| Hyperaspis_lateralis_655 | Scymnus_sinuanodulus_829 | 0.199 |
| Platynaspis_luteorubra_50 | Stethorus_punctillum_857 | 0.199 |
| Brachiacantha_rotunda_634 | Subcoccinella_vigintiquatuorpunctata_620 | 0.199 |
| Brachiacantha_rotunda_634 | Subcoccinella_vigintiquatuorpunctata_621 | 0.199 |
| Brachiacantha_rotunda_636 | Subcoccinella_vigintiquatuorpunctata_622 | 0.199 |
| Brachiacantha_rotunda_636 | Subcoccinella_vigintiquatuorpunctata_624 | 0.199 |
| Brachiacantha_rotunda_632 | Subcoccinella_vigintiquatuorpunctata_625 | 0.199 |
| Brachiacantha_rotunda_633 | Subcoccinella_vigintiquatuorpunctata_625 | 0.199 |
| Brachiacantha_rotunda_635 | Subcoccinella_vigintiquatuorpunctata_625 | 0.199 |
| Brachiacantha_rotunda_636 | Subcoccinella_vigintiquatuorpunctata_626 | 0.199 |
| Brachiacantha_rotunda_634 | Subcoccinella_vigintiquatuorpunctata_628 | 0.199 |
| Brachiacantha_rotunda_634 | Subcoccinella_vigintiquatuorpunctata_629 | 0.199 |
| Brachiacantha_rotunda_634 | Subcoccinella_vigintiquatuorpunctata_631 | 0.199 |
| Propylaea_quatuordecimpunctata_532 | Vibidia_duodecimguttata_596 | 0.199 |
| Propylaea_quatuordecimpunctata_537 | Vibidia_duodecimguttata_596 | 0.199 |
| Propylaea_quatuordecimpunctata_539 | Vibidia_duodecimguttata_596 | 0.199 |
| Coccinella_trifasciata_275 | Vibidia_duodecimguttata_597 | 0.199 |
| Coccinella_trifasciata_277 | Vibidia_duodecimguttata_597 | 0.199 |
| Coccinella_trifasciata_280 | Vibidia_duodecimguttata_597 | 0.199 |
| Coccinella_trifasciata_281 | Vibidia_duodecimguttata_597 | 0.199 |
| Coccinella_trifasciata_275 | Vibidia_duodecimguttata_600 | 0.199 |
| Coccinella_trifasciata_277 | Vibidia_duodecimguttata_600 | 0.199 |
| Coccinella_trifasciata_280 | Vibidia_duodecimguttata_600 | 0.199 |
| Coccinella_trifasciata_281 | Vibidia_duodecimguttata_600 | 0.199 |
| Neoharmonia_venusta_521 | Vibidia_duodecimguttata_603 | 0.199 |
| Propylaea_quatuordecimpunctata_533 | Vibidia_duodecimguttata_603 | 0.199 |
| Propylaea_quatuordecimpunctata_535 | Vibidia_duodecimguttata_603 | 0.199 |
| Propylaea_quatuordecimpunctata_536 | Vibidia_duodecimguttata_603 | 0.199 |
| Propylaea_quatuordecimpunctata_541 | Vibidia_duodecimguttata_603 | 0.199 |
| Platynaspis_luteorubra_50 | Stethorus_punctillum_859 | 0.199 |
| Chilocorus_renipustulatus_31 | Scymnus_sinuanodulus_829 | 0.199 |

| | | |
|---|---|---|
| Chilocorus_renipustulatus_32 | Scymnus_sinuanodulus_829 | 0.199 |
| Chilocorus_renipustulatus_35 | Scymnus_sinuanodulus_829 | 0.199 |
| Chilocorus_renipustulatus_36 | Scymnus_sinuanodulus_829 | 0.199 |
| Chilocorus_renipustulatus_37 | Scymnus_sinuanodulus_829 | 0.199 |
| Chilocorus_renipustulatus_38 | Scymnus_sinuanodulus_829 | 0.199 |
| Chilocorus_renipustulatus_40 | Scymnus_sinuanodulus_829 | 0.199 |
| Chilocorus_renipustulatus_41 | Scymnus_sinuanodulus_829 | 0.199 |
| Chilocorus_renipustulatus_42 | Scymnus_sinuanodulus_829 | 0.199 |
| Hyperaspis_lateralis_654 | Tytthaspis_sedecimpunctata_584 | 0.199 |
| Hyperaspis_lateralis_657 | Tytthaspis_sedecimpunctata_584 | 0.199 |
| Coccidophilus_marginatus_868 | Tytthaspis_sedecimpunctata_585 | 0.199 |
| Chilocorus_nigritus_28 | Vibidia_duodecimguttata_598 | 0.199 |
| Chilocorus_nigritus_28 | Vibidia_duodecimguttata_601 | 0.199 |
| Scymnus_nubilus_803 | Scymnus_sinuanodulus_831 | 0.199 |
| Scymnus_nubilus_804 | Scymnus_sinuanodulus_831 | 0.199 |
| Scymnus_nubilus_808 | Scymnus_sinuanodulus_831 | 0.199 |
| Scymnus_nubilus_809 | Scymnus_sinuanodulus_831 | 0.199 |
| Oenopia_conglobata_524 | Scymnus_suturalis_838 | 0.199 |
| Hyperaspis_lateralis_654 | Tytthaspis_sedecimpunctata_583 | 0.199 |
| Hyperaspis_lateralis_657 | Tytthaspis_sedecimpunctata_583 | 0.199 |
| Hyperaspis_lateralis_654 | Tytthaspis_sedecimpunctata_585 | 0.199 |
| Hyperaspis_lateralis_657 | Tytthaspis_sedecimpunctata_585 | 0.199 |
| Hyperaspis_lateralis_655 | Tytthaspis_sedecimpunctata_588 | 0.199 |
| Coccidophilus_marginatus_869 | Tytthaspis_sedecimpunctata_583 | 0.199 |
| Hyperaspis_reppensis_662 | Tytthaspis_sedecimpunctata_591 | 0.199 |
| Hyperaspis_reppensis_663 | Tytthaspis_sedecimpunctata_591 | 0.199 |
| Coccidophilus_marginatus_869 | Tytthaspis_sedecimpunctata_592 | 0.199 |
| Chilocorus_bipustulatus_23 | Scymnus_suturalis_838 | 0.199 |
| Chilocorus_bipustulatus_23 | Scymnus_suturalis_840 | 0.199 |
| Hyperaspis_lateralis_656 | Tytthaspis_sedecimpunctata_587 | 0.199 |
| Chilocorus_bipustulatus_23 | Scymnus_suturalis_834 | 0.199 |
| Platynaspis_luteorubra_50 | Stethorus_punctillum_853 | 0.199 |
| Hyperaspis_lateralis_656 | Tytthaspis_sedecimpunctata_589 | 0.199 |
| Chilocorus_bipustulatus_19 | Scymnus_suturalis_841 | 0.199 |
| Chilocorus_bipustulatus_22 | Scymnus_suturalis_841 | 0.199 |
| Chilocorus_bipustulatus_19 | Scymnus_suturalis_842 | 0.199 |
| Chilocorus_bipustulatus_22 | Scymnus_suturalis_842 | 0.199 |
| Chilocorus_bipustulatus_19 | Scymnus_suturalis_844 | 0.199 |
| Chilocorus_bipustulatus_22 | Scymnus_suturalis_844 | 0.199 |
| Chilocorus_bipustulatus_19 | Scymnus_suturalis_846 | 0.199 |
| Chilocorus_bipustulatus_22 | Scymnus_suturalis_846 | 0.199 |
| Chilocorus_bipustulatus_19 | Scymnus_suturalis_847 | 0.199 |
| Chilocorus_bipustulatus_22 | Scymnus_suturalis_847 | 0.199 |
| Chilocorus_bipustulatus_19 | Scymnus_suturalis_848 | 0.199 |
| Chilocorus_bipustulatus_22 | Scymnus_suturalis_848 | 0.199 |
| Chilocorus_bipustulatus_19 | Scymnus_suturalis_832 | 0.199 |
| Chilocorus_bipustulatus_22 | Scymnus_suturalis_832 | 0.199 |
| Chilocorus_bipustulatus_19 | Scymnus_suturalis_835 | 0.199 |
| Chilocorus_bipustulatus_22 | Scymnus_suturalis_835 | 0.199 |
| Chilocorus_bipustulatus_19 | Scymnus_suturalis_836 | 0.199 |
| Chilocorus_bipustulatus_22 | Scymnus_suturalis_836 | 0.199 |
| Chilocorus_bipustulatus_19 | Scymnus_suturalis_837 | 0.199 |
| Chilocorus_bipustulatus_22 | Scymnus_suturalis_837 | 0.199 |
| Chilocorus_bipustulatus_19 | Scymnus_suturalis_839 | 0.199 |
| Chilocorus_bipustulatus_22 | Scymnus_suturalis_839 | 0.199 |
| Brachiacantha_rotunda_634 | Subcoccinella_vigintiquatuorpunctata_623 | 0.199 |

| | | |
|---|---|---|
| Coccinella_trifasciata_274 | Vibidia_duodecimguttata_597 | 0.199 |
| Coccinella_trifasciata_275 | Vibidia_duodecimguttata_599 | 0.199 |
| Coccinella_trifasciata_277 | Vibidia_duodecimguttata_599 | 0.199 |
| Coccinella_trifasciata_280 | Vibidia_duodecimguttata_599 | 0.199 |
| Coccinella_trifasciata_281 | Vibidia_duodecimguttata_599 | 0.199 |
| Coccinella_trifasciata_274 | Vibidia_duodecimguttata_600 | 0.199 |
| Coccinella_trifasciata_275 | Vibidia_duodecimguttata_601 | 0.199 |
| Coccinella_trifasciata_277 | Vibidia_duodecimguttata_601 | 0.199 |
| Coccinella_trifasciata_280 | Vibidia_duodecimguttata_601 | 0.199 |
| Coccinella_trifasciata_281 | Vibidia_duodecimguttata_601 | 0.199 |
| Neoharmonia_venusta_520 | Vibidia_duodecimguttata_601 | 0.199 |
| Coccinella_trifasciata_275 | Vibidia_duodecimguttata_602 | 0.199 |
| Coccinella_trifasciata_277 | Vibidia_duodecimguttata_602 | 0.199 |
| Coccinella_trifasciata_280 | Vibidia_duodecimguttata_602 | 0.199 |
| Coccinella_trifasciata_281 | Vibidia_duodecimguttata_602 | 0.199 |
| Coccinella_hieroglyphica_227 | Vibidia_duodecimguttata_603 | 0.199 |
| Platynaspis_luteorubra_51 | Stethorus_punctillum_849 | 0.199 |
| Platynaspis_luteorubra_53 | Stethorus_punctillum_849 | 0.199 |
| Platynaspis_luteorubra_58 | Stethorus_punctillum_849 | 0.199 |
| Platynaspis_luteorubra_51 | Stethorus_punctillum_850 | 0.199 |
| Platynaspis_luteorubra_53 | Stethorus_punctillum_850 | 0.199 |
| Platynaspis_luteorubra_58 | Stethorus_punctillum_850 | 0.199 |
| Platynaspis_luteorubra_51 | Stethorus_punctillum_852 | 0.199 |
| Platynaspis_luteorubra_53 | Stethorus_punctillum_852 | 0.199 |
| Platynaspis_luteorubra_58 | Stethorus_punctillum_852 | 0.199 |
| Platynaspis_luteorubra_51 | Stethorus_punctillum_854 | 0.199 |
| Platynaspis_luteorubra_53 | Stethorus_punctillum_854 | 0.199 |
| Platynaspis_luteorubra_58 | Stethorus_punctillum_854 | 0.199 |
| Platynaspis_luteorubra_51 | Stethorus_punctillum_856 | 0.199 |
| Platynaspis_luteorubra_53 | Stethorus_punctillum_856 | 0.199 |
| Platynaspis_luteorubra_58 | Stethorus_punctillum_856 | 0.199 |
| Scymnus_mimulus_778 | Tytthaspis_sedecimpunctata_584 | 0.199 |
| Oenopia_conglobata_524 | Scymnus_suturalis_845 | 0.199 |
| Scymnus_abietis_689 | Subcoccinella_vigintiquatuorpunctata_622 | 0.199 |
| Oenopia_conglobata_524 | Scymnus_suturalis_834 | 0.199 |
| Scymnus_mimulus_778 | Tytthaspis_sedecimpunctata_577 | 0.199 |
| Scymnus_mimulus_778 | Tytthaspis_sedecimpunctata_578 | 0.199 |
| Scymnus_mimulus_778 | Tytthaspis_sedecimpunctata_579 | 0.199 |
| Scymnus_mimulus_778 | Tytthaspis_sedecimpunctata_581 | 0.199 |
| Scymnus_mimulus_778 | Tytthaspis_sedecimpunctata_582 | 0.199 |
| Coccidophilus_marginatus_870 | Tytthaspis_sedecimpunctata_583 | 0.199 |
| Coccidophilus_marginatus_870 | Tytthaspis_sedecimpunctata_585 | 0.199 |
| Scymnus_mimulus_778 | Tytthaspis_sedecimpunctata_585 | 0.199 |
| Scymnus_mimulus_776 | Tytthaspis_sedecimpunctata_586 | 0.199 |
| Scymnus_mimulus_775 | Tytthaspis_sedecimpunctata_588 | 0.199 |
| Scymnus_mimulus_777 | Tytthaspis_sedecimpunctata_588 | 0.199 |
| Scymnus_mimulus_778 | Tytthaspis_sedecimpunctata_590 | 0.199 |
| Scymnus_mimulus_778 | Tytthaspis_sedecimpunctata_592 | 0.199 |
| Scymnus_mimulus_778 | Tytthaspis_sedecimpunctata_594 | 0.199 |
| Scymnus_mimulus_778 | Tytthaspis_sedecimpunctata_595 | 0.199 |
| Chilocorus_nigritus_27 | Stethorus_punctillum_850 | 0.199 |
| Chilocorus_nigritus_27 | Stethorus_punctillum_854 | 0.199 |
| Chilocorus_nigritus_27 | Stethorus_punctillum_856 | 0.199 |
| Scymnus_mimulus_778 | Tytthaspis_sedecimpunctata_580 | 0.199 |
| Scymnus_mimulus_775 | Tytthaspis_sedecimpunctata_589 | 0.199 |
| Scymnus_mimulus_777 | Tytthaspis_sedecimpunctata_589 | 0.199 |

| | | |
|---|---|---|
| Chilocorus_renipustulatus_34 | Scymnus_suturalis_838 | 0.199 |
| Chilocorus_renipustulatus_34 | Scymnus_suturalis_840 | 0.199 |
| Cynegetis_impunctata_604 | Scymnus_suturalis_840 | 0.199 |
| Cynegetis_impunctata_608 | Scymnus_suturalis_840 | 0.199 |
| Cynegetis_impunctata_609 | Scymnus_suturalis_840 | 0.199 |
| Cynegetis_impunctata_610 | Scymnus_suturalis_840 | 0.199 |
| Cynegetis_impunctata_611 | Scymnus_suturalis_840 | 0.199 |
| Cynegetis_impunctata_612 | Scymnus_suturalis_840 | 0.199 |
| Coccinella_trifasciata_274 | Vibidia_duodecimguttata_599 | 0.199 |
| Coccinella_trifasciata_274 | Vibidia_duodecimguttata_601 | 0.199 |
| Coccinella_trifasciata_274 | Vibidia_duodecimguttata_602 | 0.199 |
| Coccinella_quinquepunctata_232 | Vibidia_duodecimguttata_603 | 0.199 |
| Cynegetis_impunctata_604 | Scymnus_suturalis_841 | 0.199 |
| Cynegetis_impunctata_608 | Scymnus_suturalis_841 | 0.199 |
| Cynegetis_impunctata_609 | Scymnus_suturalis_841 | 0.199 |
| Cynegetis_impunctata_610 | Scymnus_suturalis_841 | 0.199 |
| Cynegetis_impunctata_611 | Scymnus_suturalis_841 | 0.199 |
| Cynegetis_impunctata_612 | Scymnus_suturalis_841 | 0.199 |
| Cynegetis_impunctata_604 | Scymnus_suturalis_842 | 0.199 |
| Cynegetis_impunctata_608 | Scymnus_suturalis_842 | 0.199 |
| Cynegetis_impunctata_609 | Scymnus_suturalis_842 | 0.199 |
| Cynegetis_impunctata_610 | Scymnus_suturalis_842 | 0.199 |
| Cynegetis_impunctata_611 | Scymnus_suturalis_842 | 0.199 |
| Cynegetis_impunctata_612 | Scymnus_suturalis_842 | 0.199 |
| Cynegetis_impunctata_604 | Scymnus_suturalis_844 | 0.199 |
| Cynegetis_impunctata_608 | Scymnus_suturalis_844 | 0.199 |
| Cynegetis_impunctata_609 | Scymnus_suturalis_844 | 0.199 |
| Cynegetis_impunctata_610 | Scymnus_suturalis_844 | 0.199 |
| Cynegetis_impunctata_611 | Scymnus_suturalis_844 | 0.199 |
| Cynegetis_impunctata_612 | Scymnus_suturalis_844 | 0.199 |
| Chilocorus_renipustulatus_34 | Scymnus_suturalis_845 | 0.199 |
| Cynegetis_impunctata_604 | Scymnus_suturalis_846 | 0.199 |
| Cynegetis_impunctata_608 | Scymnus_suturalis_846 | 0.199 |
| Cynegetis_impunctata_609 | Scymnus_suturalis_846 | 0.199 |
| Cynegetis_impunctata_610 | Scymnus_suturalis_846 | 0.199 |
| Cynegetis_impunctata_611 | Scymnus_suturalis_846 | 0.199 |
| Cynegetis_impunctata_612 | Scymnus_suturalis_846 | 0.199 |
| Cynegetis_impunctata_604 | Scymnus_suturalis_847 | 0.199 |
| Cynegetis_impunctata_608 | Scymnus_suturalis_847 | 0.199 |
| Cynegetis_impunctata_609 | Scymnus_suturalis_847 | 0.199 |
| Cynegetis_impunctata_610 | Scymnus_suturalis_847 | 0.199 |
| Cynegetis_impunctata_611 | Scymnus_suturalis_847 | 0.199 |
| Cynegetis_impunctata_612 | Scymnus_suturalis_847 | 0.199 |
| Cynegetis_impunctata_604 | Scymnus_suturalis_848 | 0.199 |
| Cynegetis_impunctata_608 | Scymnus_suturalis_848 | 0.199 |
| Cynegetis_impunctata_609 | Scymnus_suturalis_848 | 0.199 |
| Cynegetis_impunctata_610 | Scymnus_suturalis_848 | 0.199 |
| Cynegetis_impunctata_611 | Scymnus_suturalis_848 | 0.199 |
| Cynegetis_impunctata_612 | Scymnus_suturalis_848 | 0.199 |
| Scymnus_loewii_770 | Subcoccinella_vigintiquatuorpunctata_623 | 0.199 |
| Scymnus_loewii_774 | Subcoccinella_vigintiquatuorpunctata_627 | 0.199 |
| Coccinella_transversalis_258 | Tytthaspis_sedecimpunctata_589 | 0.199 |
| Myrrha_octodecimguttata_485 | Scymnus_sinuanodulus_828 | 0.199 |
| Myrrha_octodecimguttata_486 | Scymnus_sinuanodulus_828 | 0.199 |
| Myrrha_octodecimguttata_487 | Scymnus_sinuanodulus_828 | 0.199 |
| Myrrha_octodecimguttata_490 | Scymnus_sinuanodulus_828 | 0.199 |

| | | |
|---|---|---|
| Myrrha_octodecimguttata_494 | Scymnus_sinuanodulus_828 | 0.199 |
| Myrrha_octodecimguttata_496 | Scymnus_sinuanodulus_828 | 0.199 |
| Myrrha_octodecimguttata_497 | Scymnus_sinuanodulus_828 | 0.199 |
| Myrrha_octodecimguttata_501 | Scymnus_sinuanodulus_828 | 0.199 |
| Myrrha_octodecimguttata_502 | Scymnus_sinuanodulus_828 | 0.199 |
| Myrrha_octodecimguttata_499 | Scymnus_sinuanodulus_830 | 0.199 |
| Cynegetis_impunctata_604 | Scymnus_suturalis_832 | 0.199 |
| Cynegetis_impunctata_608 | Scymnus_suturalis_832 | 0.199 |
| Cynegetis_impunctata_609 | Scymnus_suturalis_832 | 0.199 |
| Cynegetis_impunctata_610 | Scymnus_suturalis_832 | 0.199 |
| Cynegetis_impunctata_611 | Scymnus_suturalis_832 | 0.199 |
| Cynegetis_impunctata_612 | Scymnus_suturalis_832 | 0.199 |
| Myzia_oblongoguttata_503 | Scymnus_suturalis_833 | 0.199 |
| Cynegetis_impunctata_604 | Scymnus_suturalis_835 | 0.199 |
| Cynegetis_impunctata_608 | Scymnus_suturalis_835 | 0.199 |
| Cynegetis_impunctata_609 | Scymnus_suturalis_835 | 0.199 |
| Cynegetis_impunctata_610 | Scymnus_suturalis_835 | 0.199 |
| Cynegetis_impunctata_611 | Scymnus_suturalis_835 | 0.199 |
| Cynegetis_impunctata_612 | Scymnus_suturalis_835 | 0.199 |
| Cynegetis_impunctata_604 | Scymnus_suturalis_836 | 0.199 |
| Cynegetis_impunctata_608 | Scymnus_suturalis_836 | 0.199 |
| Cynegetis_impunctata_609 | Scymnus_suturalis_836 | 0.199 |
| Cynegetis_impunctata_610 | Scymnus_suturalis_836 | 0.199 |
| Cynegetis_impunctata_611 | Scymnus_suturalis_836 | 0.199 |
| Cynegetis_impunctata_612 | Scymnus_suturalis_836 | 0.199 |
| Chilocorus_renipustulatus_34 | Scymnus_suturalis_837 | 0.199 |
| Cynegetis_impunctata_604 | Scymnus_suturalis_837 | 0.199 |
| Cynegetis_impunctata_608 | Scymnus_suturalis_837 | 0.199 |
| Cynegetis_impunctata_609 | Scymnus_suturalis_837 | 0.199 |
| Cynegetis_impunctata_610 | Scymnus_suturalis_837 | 0.199 |
| Cynegetis_impunctata_611 | Scymnus_suturalis_837 | 0.199 |
| Cynegetis_impunctata_612 | Scymnus_suturalis_837 | 0.199 |
| Chilocorus_renipustulatus_31 | Scymnus_suturalis_838 | 0.199 |
| Chilocorus_renipustulatus_32 | Scymnus_suturalis_838 | 0.199 |
| Chilocorus_renipustulatus_35 | Scymnus_suturalis_838 | 0.199 |
| Chilocorus_renipustulatus_36 | Scymnus_suturalis_838 | 0.199 |
| Chilocorus_renipustulatus_37 | Scymnus_suturalis_838 | 0.199 |
| Chilocorus_renipustulatus_38 | Scymnus_suturalis_838 | 0.199 |
| Chilocorus_renipustulatus_40 | Scymnus_suturalis_838 | 0.199 |
| Chilocorus_renipustulatus_41 | Scymnus_suturalis_838 | 0.199 |
| Chilocorus_renipustulatus_42 | Scymnus_suturalis_838 | 0.199 |
| Myzia_oblongoguttata_503 | Scymnus_suturalis_838 | 0.199 |
| Myzia_oblongoguttata_504 | Scymnus_suturalis_838 | 0.199 |
| Myzia_oblongoguttata_505 | Scymnus_suturalis_838 | 0.199 |
| Myzia_oblongoguttata_507 | Scymnus_suturalis_838 | 0.199 |
| Myzia_oblongoguttata_508 | Scymnus_suturalis_838 | 0.199 |
| Myzia_oblongoguttata_509 | Scymnus_suturalis_838 | 0.199 |
| Myzia_oblongoguttata_510 | Scymnus_suturalis_838 | 0.199 |
| Myzia_oblongoguttata_511 | Scymnus_suturalis_838 | 0.199 |
| Myzia_oblongoguttata_512 | Scymnus_suturalis_838 | 0.199 |
| Myzia_oblongoguttata_513 | Scymnus_suturalis_838 | 0.199 |
| Myzia_oblongoguttata_514 | Scymnus_suturalis_838 | 0.199 |
| Cynegetis_impunctata_604 | Scymnus_suturalis_839 | 0.199 |
| Cynegetis_impunctata_608 | Scymnus_suturalis_839 | 0.199 |
| Cynegetis_impunctata_609 | Scymnus_suturalis_839 | 0.199 |
| Cynegetis_impunctata_610 | Scymnus_suturalis_839 | 0.199 |

| | | |
|---|---|---|
| Cynegetis_impunctata_611 | Scymnus_suturalis_839 | 0.199 |
| Cynegetis_impunctata_612 | Scymnus_suturalis_839 | 0.199 |
| Chilocorus_renipustulatus_31 | Scymnus_suturalis_840 | 0.199 |
| Chilocorus_renipustulatus_32 | Scymnus_suturalis_840 | 0.199 |
| Chilocorus_renipustulatus_35 | Scymnus_suturalis_840 | 0.199 |
| Chilocorus_renipustulatus_36 | Scymnus_suturalis_840 | 0.199 |
| Chilocorus_renipustulatus_37 | Scymnus_suturalis_840 | 0.199 |
| Chilocorus_renipustulatus_38 | Scymnus_suturalis_840 | 0.199 |
| Chilocorus_renipustulatus_40 | Scymnus_suturalis_840 | 0.199 |
| Chilocorus_renipustulatus_41 | Scymnus_suturalis_840 | 0.199 |
| Chilocorus_renipustulatus_42 | Scymnus_suturalis_840 | 0.199 |
| Myzia_oblongoguttata_504 | Scymnus_suturalis_840 | 0.199 |
| Myzia_oblongoguttata_505 | Scymnus_suturalis_840 | 0.199 |
| Myzia_oblongoguttata_507 | Scymnus_suturalis_840 | 0.199 |
| Myzia_oblongoguttata_508 | Scymnus_suturalis_840 | 0.199 |
| Myzia_oblongoguttata_509 | Scymnus_suturalis_840 | 0.199 |
| Myzia_oblongoguttata_510 | Scymnus_suturalis_840 | 0.199 |
| Myzia_oblongoguttata_511 | Scymnus_suturalis_840 | 0.199 |
| Myzia_oblongoguttata_512 | Scymnus_suturalis_840 | 0.199 |
| Myzia_oblongoguttata_513 | Scymnus_suturalis_840 | 0.199 |
| Myzia_oblongoguttata_514 | Scymnus_suturalis_840 | 0.199 |
| Myzia_oblongoguttata_515 | Scymnus_suturalis_840 | 0.199 |
| Anisosticta_bitriangularis_147 | Scymnus_sinuanodulus_831 | 0.199 |
| Myzia_oblongoguttata_503 | Scymnus_suturalis_841 | 0.199 |
| Myzia_oblongoguttata_503 | Scymnus_suturalis_842 | 0.199 |
| Myzia_oblongoguttata_503 | Scymnus_suturalis_844 | 0.199 |
| Myzia_oblongoguttata_503 | Scymnus_suturalis_846 | 0.199 |
| Myzia_oblongoguttata_503 | Scymnus_suturalis_847 | 0.199 |
| Myzia_oblongoguttata_503 | Scymnus_suturalis_848 | 0.199 |
| Chilocorus_nigritus_26 | Stethorus_punctillum_850 | 0.199 |
| Chilocorus_nigritus_26 | Stethorus_punctillum_854 | 0.199 |
| Chilocorus_nigritus_26 | Stethorus_punctillum_856 | 0.199 |
| Scymnus_loewii_770 | Subcoccinella_vigintiquatuorpunctata_622 | 0.199 |
| Scymnus_loewii_770 | Subcoccinella_vigintiquatuorpunctata_626 | 0.199 |
| Scymnus_loewii_772 | Subcoccinella_vigintiquatuorpunctata_627 | 0.199 |
| Scymnus_loewii_773 | Subcoccinella_vigintiquatuorpunctata_627 | 0.199 |
| Coccinella_transversalis_258 | Tytthaspis_sedecimpunctata_587 | 0.199 |
| Coccinella_transversalis_258 | Tytthaspis_sedecimpunctata_588 | 0.199 |
| Myrrha_octodecimguttata_488 | Scymnus_sinuanodulus_828 | 0.199 |
| Myrrha_octodecimguttata_491 | Scymnus_sinuanodulus_828 | 0.199 |
| Myrrha_octodecimguttata_492 | Scymnus_sinuanodulus_828 | 0.199 |
| Myzia_oblongoguttata_503 | Scymnus_suturalis_832 | 0.199 |
| Myzia_oblongoguttata_504 | Scymnus_suturalis_834 | 0.199 |
| Myzia_oblongoguttata_505 | Scymnus_suturalis_834 | 0.199 |
| Myzia_oblongoguttata_507 | Scymnus_suturalis_834 | 0.199 |
| Myzia_oblongoguttata_508 | Scymnus_suturalis_834 | 0.199 |
| Myzia_oblongoguttata_509 | Scymnus_suturalis_834 | 0.199 |
| Myzia_oblongoguttata_510 | Scymnus_suturalis_834 | 0.199 |
| Myzia_oblongoguttata_511 | Scymnus_suturalis_834 | 0.199 |
| Myzia_oblongoguttata_512 | Scymnus_suturalis_834 | 0.199 |
| Myzia_oblongoguttata_513 | Scymnus_suturalis_834 | 0.199 |
| Myzia_oblongoguttata_514 | Scymnus_suturalis_834 | 0.199 |
| Myzia_oblongoguttata_515 | Scymnus_suturalis_834 | 0.199 |
| Myzia_oblongoguttata_503 | Scymnus_suturalis_835 | 0.199 |
| Myzia_oblongoguttata_503 | Scymnus_suturalis_836 | 0.199 |
| Chilocorus_renipustulatus_31 | Scymnus_suturalis_837 | 0.199 |

| | | |
|---|---|---|
| Chilocorus_renipustulatus_32 | Scymnus_suturalis_837 | 0.199 |
| Chilocorus_renipustulatus_35 | Scymnus_suturalis_837 | 0.199 |
| Chilocorus_renipustulatus_36 | Scymnus_suturalis_837 | 0.199 |
| Chilocorus_renipustulatus_37 | Scymnus_suturalis_837 | 0.199 |
| Chilocorus_renipustulatus_38 | Scymnus_suturalis_837 | 0.199 |
| Chilocorus_renipustulatus_40 | Scymnus_suturalis_837 | 0.199 |
| Chilocorus_renipustulatus_41 | Scymnus_suturalis_837 | 0.199 |
| Chilocorus_renipustulatus_42 | Scymnus_suturalis_837 | 0.199 |
| Myzia_oblongoguttata_503 | Scymnus_suturalis_837 | 0.199 |
| Myzia_oblongoguttata_503 | Scymnus_suturalis_839 | 0.199 |
| Rhyzobius_chrysomeloides_85 | Tytthaspis_sedecimpunctata_580 | 0.199 |
| Rhyzobius_chrysomeloides_86 | Tytthaspis_sedecimpunctata_580 | 0.199 |
| Rhyzobius_chrysomeloides_87 | Tytthaspis_sedecimpunctata_580 | 0.199 |
| Rhyzobius_chrysomeloides_88 | Tytthaspis_sedecimpunctata_580 | 0.199 |
| Rhyzobius_chrysomeloides_89 | Tytthaspis_sedecimpunctata_580 | 0.199 |
| Rhyzobius_chrysomeloides_90 | Tytthaspis_sedecimpunctata_580 | 0.199 |
| Rhyzobius_chrysomeloides_91 | Tytthaspis_sedecimpunctata_580 | 0.199 |
| Rhyzobius_chrysomeloides_92 | Tytthaspis_sedecimpunctata_580 | 0.199 |
| Rhyzobius_chrysomeloides_93 | Tytthaspis_sedecimpunctata_580 | 0.199 |
| Rhyzobius_chrysomeloides_94 | Tytthaspis_sedecimpunctata_580 | 0.199 |
| Rhyzobius_chrysomeloides_95 | Tytthaspis_sedecimpunctata_580 | 0.199 |
| Rhyzobius_chrysomeloides_85 | Tytthaspis_sedecimpunctata_584 | 0.199 |
| Rhyzobius_chrysomeloides_86 | Tytthaspis_sedecimpunctata_584 | 0.199 |
| Rhyzobius_chrysomeloides_87 | Tytthaspis_sedecimpunctata_584 | 0.199 |
| Rhyzobius_chrysomeloides_88 | Tytthaspis_sedecimpunctata_584 | 0.199 |
| Rhyzobius_chrysomeloides_89 | Tytthaspis_sedecimpunctata_584 | 0.199 |
| Rhyzobius_chrysomeloides_90 | Tytthaspis_sedecimpunctata_584 | 0.199 |
| Rhyzobius_chrysomeloides_91 | Tytthaspis_sedecimpunctata_584 | 0.199 |
| Rhyzobius_chrysomeloides_92 | Tytthaspis_sedecimpunctata_584 | 0.199 |
| Rhyzobius_chrysomeloides_93 | Tytthaspis_sedecimpunctata_584 | 0.199 |
| Rhyzobius_chrysomeloides_94 | Tytthaspis_sedecimpunctata_584 | 0.199 |
| Rhyzobius_chrysomeloides_95 | Tytthaspis_sedecimpunctata_584 | 0.199 |
| Harmonia_quadripunctata_376 | Tytthaspis_sedecimpunctata_591 | 0.199 |
| Harmonia_quadripunctata_378 | Tytthaspis_sedecimpunctata_591 | 0.199 |
| Harmonia_quadripunctata_382 | Tytthaspis_sedecimpunctata_591 | 0.199 |
| Myrrha_octodecimguttata_495 | Scymnus_sinuanodulus_830 | 0.199 |
| Chilocorus_renipustulatus_39 | Scymnus_suturalis_833 | 0.199 |
| Chilocorus_bipustulatus_21 | Scymnus_suturalis_841 | 0.199 |
| Chilocorus_bipustulatus_21 | Scymnus_suturalis_842 | 0.199 |
| Chilocorus_bipustulatus_21 | Scymnus_suturalis_844 | 0.199 |
| Chilocorus_bipustulatus_21 | Scymnus_suturalis_846 | 0.199 |
| Chilocorus_bipustulatus_21 | Scymnus_suturalis_847 | 0.199 |
| Chilocorus_bipustulatus_21 | Scymnus_suturalis_848 | 0.199 |
| Chilocorus_bipustulatus_21 | Scymnus_suturalis_832 | 0.199 |
| Chilocorus_bipustulatus_21 | Scymnus_suturalis_835 | 0.199 |
| Chilocorus_bipustulatus_21 | Scymnus_suturalis_836 | 0.199 |
| Chilocorus_bipustulatus_21 | Scymnus_suturalis_837 | 0.199 |
| Chilocorus_bipustulatus_21 | Scymnus_suturalis_839 | 0.199 |
| Coccinella_quinquepunctata_233 | Vibidia_duodecimguttata_597 | 0.199 |
| Coccinella_quinquepunctata_234 | Vibidia_duodecimguttata_597 | 0.199 |
| Coccinella_quinquepunctata_233 | Vibidia_duodecimguttata_600 | 0.199 |
| Coccinella_quinquepunctata_234 | Vibidia_duodecimguttata_600 | 0.199 |
| Scymnus_apetzi_692 | Tytthaspis_sedecimpunctata_583 | 0.199 |
| Scymnus_apetzi_692 | Tytthaspis_sedecimpunctata_585 | 0.199 |
| Scymnus_apetzi_692 | Tytthaspis_sedecimpunctata_592 | 0.199 |
| Brachiacantha_ursina_638 | Tytthaspis_sedecimpunctata_580 | 0.199 |

Supplementary Table S4 (continued)

| | | |
|---|---|---|
| Adalia_decempunctata_118 | Tytthaspis_sedecimpunctata_583 | 0.199 |
| Adalia_decempunctata_119 | Tytthaspis_sedecimpunctata_583 | 0.199 |
| Adalia_decempunctata_120 | Tytthaspis_sedecimpunctata_583 | 0.199 |
| Adalia_decempunctata_121 | Tytthaspis_sedecimpunctata_583 | 0.199 |
| Brachiacantha_ursina_637 | Tytthaspis_sedecimpunctata_584 | 0.199 |
| Harmonia_quadripunctata_376 | Tytthaspis_sedecimpunctata_585 | 0.199 |
| Harmonia_quadripunctata_378 | Tytthaspis_sedecimpunctata_585 | 0.199 |
| Harmonia_quadripunctata_382 | Tytthaspis_sedecimpunctata_585 | 0.199 |
| Adalia_decempunctata_118 | Tytthaspis_sedecimpunctata_592 | 0.199 |
| Adalia_decempunctata_119 | Tytthaspis_sedecimpunctata_592 | 0.199 |
| Adalia_decempunctata_120 | Tytthaspis_sedecimpunctata_592 | 0.199 |
| Adalia_decempunctata_121 | Tytthaspis_sedecimpunctata_592 | 0.199 |
| Anisosticta_bitriangularis_155 | Scymnus_sinuanodulus_830 | 0.199 |
| Anisosticta_bitriangularis_158 | Scymnus_sinuanodulus_830 | 0.199 |
| Scymnus_apetzi_692 | Tytthaspis_sedecimpunctata_580 | 0.199 |
| Scymnus_apetzi_692 | Tytthaspis_sedecimpunctata_586 | 0.199 |
| Chilocorus_nigritus_29 | Stethorus_punctillum_849 | 0.199 |
| Chilocorus_nigritus_30 | Stethorus_punctillum_850 | 0.199 |
| Chilocorus_nigritus_30 | Stethorus_punctillum_854 | 0.199 |
| Chilocorus_nigritus_30 | Stethorus_punctillum_856 | 0.199 |
| Chilocorus_nigritus_29 | Stethorus_punctillum_860 | 0.199 |
| Hyperaspis_lateralis_656 | Subcoccinella_vigintiquatuorpunctata_625 | 0.200 |
| Hyperaspis_lateralis_657 | Subcoccinella_vigintiquatuorpunctata_625 | 0.200 |
| Hyperaspis_reppensis_662 | Subcoccinella_vigintiquatuorpunctata_625 | 0.200 |
| Hyperaspis_reppensis_663 | Subcoccinella_vigintiquatuorpunctata_625 | 0.200 |
| Calvia_decemguttata_195 | Subcoccinella_vigintiquatuorpunctata_627 | 0.200 |
| Brachiacantha_ursina_638 | Tytthaspis_sedecimpunctata_583 | 0.200 |
| Harmonia_quadripunctata_376 | Tytthaspis_sedecimpunctata_584 | 0.200 |
| Harmonia_quadripunctata_378 | Tytthaspis_sedecimpunctata_584 | 0.200 |
| Harmonia_quadripunctata_382 | Tytthaspis_sedecimpunctata_584 | 0.200 |
| Diomus_terminatus_647 | Tytthaspis_sedecimpunctata_586 | 0.200 |
| Diomus_terminatus_648 | Tytthaspis_sedecimpunctata_586 | 0.200 |
| Diomus_terminatus_647 | Tytthaspis_sedecimpunctata_588 | 0.200 |
| Diomus_terminatus_648 | Tytthaspis_sedecimpunctata_588 | 0.200 |
| Brachiacantha_ursina_638 | Tytthaspis_sedecimpunctata_589 | 0.200 |
| Scymnus_nebulosus_781 | Tytthaspis_sedecimpunctata_589 | 0.200 |
| Adalia_bipunctata_99 | Scymnus_rubromaculatus_823 | 0.200 |
| Adalia_bipunctata_99 | Scymnus_rubromaculatus_824 | 0.200 |
| Adalia_bipunctata_99 | Scymnus_rubromaculatus_825 | 0.200 |
| Adalia_bipunctata_99 | Scymnus_rubromaculatus_826 | 0.200 |
| Adalia_bipunctata_99 | Scymnus_rubromaculatus_827 | 0.200 |
| Coelophora_inaequalis_313 | Vibidia_duodecimguttata_599 | 0.200 |
| Coelophora_inaequalis_326 | Vibidia_duodecimguttata_599 | 0.200 |
| Coelophora_inaequalis_313 | Vibidia_duodecimguttata_601 | 0.200 |
| Coelophora_inaequalis_326 | Vibidia_duodecimguttata_601 | 0.200 |
| Coelophora_inaequalis_313 | Vibidia_duodecimguttata_602 | 0.200 |
| Coelophora_inaequalis_326 | Vibidia_duodecimguttata_602 | 0.200 |
| Chilocorus_nigritus_28 | Stethorus_punctillum_850 | 0.200 |
| Chilocorus_nigritus_28 | Stethorus_punctillum_854 | 0.200 |
| Chilocorus_nigritus_28 | Stethorus_punctillum_856 | 0.200 |
| Scymnus_nubilus_803 | Scymnus_sinuanodulus_829 | 0.200 |
| Scymnus_nubilus_804 | Scymnus_sinuanodulus_829 | 0.200 |
| Scymnus_nubilus_808 | Scymnus_sinuanodulus_829 | 0.200 |
| Scymnus_nubilus_809 | Scymnus_sinuanodulus_829 | 0.200 |
| Coccinella_quinquepunctata_233 | Vibidia_duodecimguttata_598 | 0.200 |
| Coccinella_quinquepunctata_234 | Vibidia_duodecimguttata_598 | 0.200 |

| | | |
|---|---|---|
| Coccinella_quinquepunctata_233 | Vibidia_duodecimguttata_599 | 0.200 |
| Coccinella_quinquepunctata_234 | Vibidia_duodecimguttata_599 | 0.200 |
| Coccinella_quinquepunctata_233 | Vibidia_duodecimguttata_601 | 0.200 |
| Coccinella_quinquepunctata_234 | Vibidia_duodecimguttata_601 | 0.200 |
| Coccinella_quinquepunctata_233 | Vibidia_duodecimguttata_602 | 0.200 |
| Coccinella_quinquepunctata_234 | Vibidia_duodecimguttata_602 | 0.200 |
| Hyperaspis_campestris_651 | Stethorus_punctillum_852 | 0.200 |
| Hyperaspis_campestris_652 | Stethorus_punctillum_852 | 0.200 |
| Hyperaspis_campestris_653 | Stethorus_punctillum_852 | 0.200 |
| Hyperaspis_campestris_651 | Stethorus_punctillum_860 | 0.200 |
| Hyperaspis_campestris_652 | Stethorus_punctillum_860 | 0.200 |
| Hyperaspis_campestris_653 | Stethorus_punctillum_860 | 0.200 |
| Scymnus_ningshanensis_789 | Subcoccinella_vigintiquatuorpunctata_622 | 0.200 |
| Scymnus_ningshanensis_790 | Subcoccinella_vigintiquatuorpunctata_622 | 0.200 |
| Scymnus_apetzi_696 | Subcoccinella_vigintiquatuorpunctata_623 | 0.200 |
| Scymnus_nebulosus_783 | Subcoccinella_vigintiquatuorpunctata_623 | 0.200 |
| Scymnus_ningshanensis_788 | Subcoccinella_vigintiquatuorpunctata_624 | 0.200 |
| Scymnus_ningshanensis_791 | Subcoccinella_vigintiquatuorpunctata_624 | 0.200 |
| Scymnus_ningshanensis_792 | Subcoccinella_vigintiquatuorpunctata_624 | 0.200 |
| Scymnus_ningshanensis_793 | Subcoccinella_vigintiquatuorpunctata_624 | 0.200 |
| Scymnus_ningshanensis_794 | Subcoccinella_vigintiquatuorpunctata_624 | 0.200 |
| Scymnus_ningshanensis_795 | Subcoccinella_vigintiquatuorpunctata_624 | 0.200 |
| Scymnus_nebulosus_783 | Tytthaspis_sedecimpunctata_584 | 0.200 |
| Scymnus_nebulosus_781 | Tytthaspis_sedecimpunctata_586 | 0.200 |
| Brachiacantha_ursina_638 | Tytthaspis_sedecimpunctata_587 | 0.200 |
| Scymnus_nebulosus_781 | Tytthaspis_sedecimpunctata_588 | 0.200 |
| Scymnus_nigrinus_785 | Tytthaspis_sedecimpunctata_591 | 0.200 |
| Scymnus_nigrinus_786 | Tytthaspis_sedecimpunctata_591 | 0.200 |
| Nephus_redtenbacheri_675 | Scymnus_rubromaculatus_822 | 0.200 |
| Nephus_redtenbacheri_676 | Scymnus_rubromaculatus_822 | 0.200 |
| Nephus_redtenbacheri_677 | Scymnus_rubromaculatus_822 | 0.200 |
| Adalia_bipunctata_102 | Scymnus_rubromaculatus_823 | 0.200 |
| Adalia_bipunctata_103 | Scymnus_rubromaculatus_823 | 0.200 |
| Adalia_bipunctata_106 | Scymnus_rubromaculatus_823 | 0.200 |
| Adalia_bipunctata_107 | Scymnus_rubromaculatus_823 | 0.200 |
| Adalia_bipunctata_108 | Scymnus_rubromaculatus_823 | 0.200 |
| Adalia_bipunctata_109 | Scymnus_rubromaculatus_823 | 0.200 |
| Nephus_redtenbacheri_675 | Scymnus_rubromaculatus_823 | 0.200 |
| Nephus_redtenbacheri_676 | Scymnus_rubromaculatus_823 | 0.200 |
| Nephus_redtenbacheri_677 | Scymnus_rubromaculatus_823 | 0.200 |
| Adalia_bipunctata_102 | Scymnus_rubromaculatus_824 | 0.200 |
| Adalia_bipunctata_103 | Scymnus_rubromaculatus_824 | 0.200 |
| Adalia_bipunctata_106 | Scymnus_rubromaculatus_824 | 0.200 |
| Adalia_bipunctata_107 | Scymnus_rubromaculatus_824 | 0.200 |
| Adalia_bipunctata_108 | Scymnus_rubromaculatus_824 | 0.200 |
| Adalia_bipunctata_109 | Scymnus_rubromaculatus_824 | 0.200 |
| Nephus_redtenbacheri_675 | Scymnus_rubromaculatus_824 | 0.200 |
| Nephus_redtenbacheri_676 | Scymnus_rubromaculatus_824 | 0.200 |
| Nephus_redtenbacheri_677 | Scymnus_rubromaculatus_824 | 0.200 |
| Adalia_bipunctata_102 | Scymnus_rubromaculatus_825 | 0.200 |
| Adalia_bipunctata_103 | Scymnus_rubromaculatus_825 | 0.200 |
| Adalia_bipunctata_106 | Scymnus_rubromaculatus_825 | 0.200 |
| Adalia_bipunctata_107 | Scymnus_rubromaculatus_825 | 0.200 |
| Adalia_bipunctata_108 | Scymnus_rubromaculatus_825 | 0.200 |
| Adalia_bipunctata_109 | Scymnus_rubromaculatus_825 | 0.200 |
| Nephus_redtenbacheri_675 | Scymnus_rubromaculatus_825 | 0.200 |

| | | |
|---|---|---|
| Nephus_redtenbacheri_676 | Scymnus_rubromaculatus_825 | 0.200 |
| Nephus_redtenbacheri_677 | Scymnus_rubromaculatus_825 | 0.200 |
| Adalia_bipunctata_102 | Scymnus_rubromaculatus_826 | 0.200 |
| Adalia_bipunctata_103 | Scymnus_rubromaculatus_826 | 0.200 |
| Adalia_bipunctata_106 | Scymnus_rubromaculatus_826 | 0.200 |
| Adalia_bipunctata_107 | Scymnus_rubromaculatus_826 | 0.200 |
| Adalia_bipunctata_108 | Scymnus_rubromaculatus_826 | 0.200 |
| Adalia_bipunctata_109 | Scymnus_rubromaculatus_826 | 0.200 |
| Nephus_redtenbacheri_675 | Scymnus_rubromaculatus_826 | 0.200 |
| Nephus_redtenbacheri_676 | Scymnus_rubromaculatus_826 | 0.200 |
| Nephus_redtenbacheri_677 | Scymnus_rubromaculatus_826 | 0.200 |
| Adalia_bipunctata_102 | Scymnus_rubromaculatus_827 | 0.200 |
| Adalia_bipunctata_103 | Scymnus_rubromaculatus_827 | 0.200 |
| Adalia_bipunctata_106 | Scymnus_rubromaculatus_827 | 0.200 |
| Adalia_bipunctata_107 | Scymnus_rubromaculatus_827 | 0.200 |
| Adalia_bipunctata_108 | Scymnus_rubromaculatus_827 | 0.200 |
| Adalia_bipunctata_109 | Scymnus_rubromaculatus_827 | 0.200 |
| Nephus_redtenbacheri_675 | Scymnus_rubromaculatus_827 | 0.200 |
| Nephus_redtenbacheri_676 | Scymnus_rubromaculatus_827 | 0.200 |
| Nephus_redtenbacheri_677 | Scymnus_rubromaculatus_827 | 0.200 |
| Rhyzobius_litura_97 | Scymnus_suturalis_840 | 0.200 |
| Scymnus_loewii_770 | Subcoccinella_vigintiquatuorpunctata_619 | 0.200 |
| Scymnus_loewii_772 | Subcoccinella_vigintiquatuorpunctata_619 | 0.200 |
| Scymnus_loewii_773 | Subcoccinella_vigintiquatuorpunctata_619 | 0.200 |
| Myzia_pullata_519 | Scymnus_suturalis_841 | 0.200 |
| Myzia_pullata_519 | Scymnus_suturalis_842 | 0.200 |
| Myzia_pullata_519 | Scymnus_suturalis_844 | 0.200 |
| Myzia_pullata_519 | Scymnus_suturalis_846 | 0.200 |
| Myzia_pullata_519 | Scymnus_suturalis_847 | 0.200 |
| Myzia_pullata_519 | Scymnus_suturalis_848 | 0.200 |
| Scymnus_apetzi_696 | Subcoccinella_vigintiquatuorpunctata_622 | 0.200 |
| Scymnus_nebulosus_783 | Subcoccinella_vigintiquatuorpunctata_622 | 0.200 |
| Scymnus_apetzi_690 | Subcoccinella_vigintiquatuorpunctata_624 | 0.200 |
| Scymnus_apetzi_697 | Subcoccinella_vigintiquatuorpunctata_624 | 0.200 |
| Scymnus_apetzi_691 | Subcoccinella_vigintiquatuorpunctata_625 | 0.200 |
| Scymnus_apetzi_696 | Subcoccinella_vigintiquatuorpunctata_626 | 0.200 |
| Scymnus_nebulosus_783 | Subcoccinella_vigintiquatuorpunctata_626 | 0.200 |
| Scymnus_interruptus_761 | Tytthaspis_sedecimpunctata_591 | 0.200 |
| Scymnus_interruptus_763 | Tytthaspis_sedecimpunctata_591 | 0.200 |
| Scymnus_interruptus_764 | Tytthaspis_sedecimpunctata_591 | 0.200 |
| Scymnus_interruptus_766 | Tytthaspis_sedecimpunctata_591 | 0.200 |
| Hyperaspis_binotata_649 | Scymnus_rubromaculatus_822 | 0.200 |
| Hyperaspis_binotata_649 | Scymnus_rubromaculatus_823 | 0.200 |
| Hyperaspis_binotata_649 | Scymnus_rubromaculatus_824 | 0.200 |
| Hyperaspis_binotata_649 | Scymnus_rubromaculatus_825 | 0.200 |
| Hyperaspis_binotata_649 | Scymnus_rubromaculatus_826 | 0.200 |
| Hyperaspis_binotata_649 | Scymnus_rubromaculatus_827 | 0.200 |
| Hyperaspis_lateralis_657 | Scymnus_sinuanodulus_828 | 0.200 |
| Brachiacantha_rotunda_632 | Scymnus_sinuanodulus_830 | 0.200 |
| Brachiacantha_rotunda_633 | Scymnus_sinuanodulus_830 | 0.200 |
| Brachiacantha_rotunda_635 | Scymnus_sinuanodulus_830 | 0.200 |
| Myzia_pullata_519 | Scymnus_suturalis_832 | 0.200 |
| Rhyzobius_litura_97 | Scymnus_suturalis_834 | 0.200 |
| Myzia_pullata_519 | Scymnus_suturalis_835 | 0.200 |
| Myzia_pullata_519 | Scymnus_suturalis_836 | 0.200 |
| Myzia_pullata_519 | Scymnus_suturalis_837 | 0.200 |

| | | |
|---|---|---|
| Rhyzobius_litura_97 | Scymnus_suturalis_837 | 0.200 |
| Myzia_pullata_519 | Scymnus_suturalis_839 | 0.200 |
| Cynegetis_impunctata_604 | Subcoccinella_vigintiquatuorpunctata_622 | 0.200 |
| Cynegetis_impunctata_605 | Subcoccinella_vigintiquatuorpunctata_622 | 0.200 |
| Cynegetis_impunctata_606 | Subcoccinella_vigintiquatuorpunctata_622 | 0.200 |
| Cynegetis_impunctata_607 | Subcoccinella_vigintiquatuorpunctata_622 | 0.200 |
| Cynegetis_impunctata_608 | Subcoccinella_vigintiquatuorpunctata_622 | 0.200 |
| Cynegetis_impunctata_609 | Subcoccinella_vigintiquatuorpunctata_622 | 0.200 |
| Cynegetis_impunctata_610 | Subcoccinella_vigintiquatuorpunctata_622 | 0.200 |
| Cynegetis_impunctata_611 | Subcoccinella_vigintiquatuorpunctata_622 | 0.200 |
| Cynegetis_impunctata_612 | Subcoccinella_vigintiquatuorpunctata_622 | 0.200 |
| Cynegetis_impunctata_613 | Subcoccinella_vigintiquatuorpunctata_622 | 0.200 |
| Cynegetis_impunctata_614 | Subcoccinella_vigintiquatuorpunctata_624 | 0.200 |
| Cynegetis_impunctata_604 | Subcoccinella_vigintiquatuorpunctata_625 | 0.200 |
| Cynegetis_impunctata_605 | Subcoccinella_vigintiquatuorpunctata_625 | 0.200 |
| Cynegetis_impunctata_606 | Subcoccinella_vigintiquatuorpunctata_625 | 0.200 |
| Cynegetis_impunctata_607 | Subcoccinella_vigintiquatuorpunctata_625 | 0.200 |
| Cynegetis_impunctata_608 | Subcoccinella_vigintiquatuorpunctata_625 | 0.200 |
| Cynegetis_impunctata_609 | Subcoccinella_vigintiquatuorpunctata_625 | 0.200 |
| Cynegetis_impunctata_610 | Subcoccinella_vigintiquatuorpunctata_625 | 0.200 |
| Cynegetis_impunctata_611 | Subcoccinella_vigintiquatuorpunctata_625 | 0.200 |
| Cynegetis_impunctata_612 | Subcoccinella_vigintiquatuorpunctata_625 | 0.200 |
| Cynegetis_impunctata_613 | Subcoccinella_vigintiquatuorpunctata_625 | 0.200 |
| Myrrha_octodecimguttata_489 | Scymnus_sinuanodulus_831 | 0.200 |
| Myrrha_octodecimguttata_500 | Scymnus_sinuanodulus_831 | 0.200 |
| Myzia_oblongoguttata_504 | Scymnus_sinuanodulus_831 | 0.200 |
| Myzia_oblongoguttata_505 | Scymnus_sinuanodulus_831 | 0.200 |
| Myzia_oblongoguttata_507 | Scymnus_sinuanodulus_831 | 0.200 |
| Myzia_oblongoguttata_508 | Scymnus_sinuanodulus_831 | 0.200 |
| Myzia_oblongoguttata_509 | Scymnus_sinuanodulus_831 | 0.200 |
| Myzia_oblongoguttata_510 | Scymnus_sinuanodulus_831 | 0.200 |
| Myzia_oblongoguttata_511 | Scymnus_sinuanodulus_831 | 0.200 |
| Myzia_oblongoguttata_512 | Scymnus_sinuanodulus_831 | 0.200 |
| Myzia_oblongoguttata_513 | Scymnus_sinuanodulus_831 | 0.200 |
| Myzia_oblongoguttata_514 | Scymnus_sinuanodulus_831 | 0.200 |
| Myzia_oblongoguttata_516 | Scymnus_sinuanodulus_831 | 0.200 |
| Hyperaspis_reppensis_664 | Stethorus_punctillum_852 | 0.200 |
| Hyperaspis_reppensis_665 | Stethorus_punctillum_852 | 0.200 |
| Hyperaspis_reppensis_666 | Stethorus_punctillum_852 | 0.200 |
| Hyperaspis_campestris_651 | Stethorus_punctillum_853 | 0.200 |
| Hyperaspis_campestris_652 | Stethorus_punctillum_853 | 0.200 |
| Hyperaspis_campestris_653 | Stethorus_punctillum_853 | 0.200 |
| Hyperaspis_reppensis_667 | Stethorus_punctillum_857 | 0.200 |
| Hyperaspis_reppensis_664 | Stethorus_punctillum_860 | 0.200 |
| Hyperaspis_reppensis_665 | Stethorus_punctillum_860 | 0.200 |
| Hyperaspis_reppensis_666 | Stethorus_punctillum_860 | 0.200 |
| Psyllobora_vigintiduopunctata_561 | Subcoccinella_vigintiquatuorpunctata_623 | 0.200 |
| Psyllobora_vigintiduopunctata_562 | Subcoccinella_vigintiquatuorpunctata_623 | 0.200 |
| Psyllobora_vigintiduopunctata_565 | Subcoccinella_vigintiquatuorpunctata_623 | 0.200 |
| Psyllobora_vigintiduopunctata_567 | Subcoccinella_vigintiquatuorpunctata_623 | 0.200 |
| Platynaspis_luteorubra_55 | Subcoccinella_vigintiquatuorpunctata_627 | 0.200 |
| Platynaspis_luteorubra_55 | Subcoccinella_vigintiquatuorpunctata_630 | 0.200 |
| Scymnus_interruptus_762 | Tytthaspis_sedecimpunctata_587 | 0.200 |
| Brachiacantha_rotunda_632 | Scymnus_sinuanodulus_828 | 0.200 |
| Brachiacantha_rotunda_633 | Scymnus_sinuanodulus_828 | 0.200 |
| Brachiacantha_rotunda_635 | Scymnus_sinuanodulus_828 | 0.200 |

| | | |
|---|---|---|
| Hyperaspis_lateralis_657 | Scymnus_sinuanodulus_830 | 0.200 |
| Calvia_quatuordecimguttata_218 | Scymnus_suturalis_833 | 0.200 |
| Clitostethus_arcuatus_641 | Scymnus_suturalis_833 | 0.200 |
| Rhyzobius_litura_97 | Scymnus_suturalis_833 | 0.200 |
| Calvia_quatuordecimguttata_209 | Scymnus_suturalis_843 | 0.200 |
| Calvia_quatuordecimguttata_209 | Scymnus_suturalis_845 | 0.200 |
| Hyperaspis_reppensis_667 | Stethorus_punctillum_859 | 0.200 |
| Psyllobora_vigintiduopunctata_570 | Subcoccinella_vigintiquatuorpunctata_620 | 0.200 |
| Psyllobora_vigintiduopunctata_572 | Subcoccinella_vigintiquatuorpunctata_620 | 0.200 |
| Psyllobora_vigintiduopunctata_570 | Subcoccinella_vigintiquatuorpunctata_621 | 0.200 |
| Psyllobora_vigintiduopunctata_572 | Subcoccinella_vigintiquatuorpunctata_621 | 0.200 |
| Psyllobora_vigintiduopunctata_561 | Subcoccinella_vigintiquatuorpunctata_622 | 0.200 |
| Psyllobora_vigintiduopunctata_562 | Subcoccinella_vigintiquatuorpunctata_622 | 0.200 |
| Psyllobora_vigintiduopunctata_565 | Subcoccinella_vigintiquatuorpunctata_622 | 0.200 |
| Psyllobora_vigintiduopunctata_567 | Subcoccinella_vigintiquatuorpunctata_622 | 0.200 |
| Psyllobora_vigintiduopunctata_557 | Subcoccinella_vigintiquatuorpunctata_624 | 0.200 |
| Psyllobora_vigintiduopunctata_563 | Subcoccinella_vigintiquatuorpunctata_624 | 0.200 |
| Psyllobora_vigintiduopunctata_564 | Subcoccinella_vigintiquatuorpunctata_624 | 0.200 |
| Psyllobora_vigintiduopunctata_561 | Subcoccinella_vigintiquatuorpunctata_625 | 0.200 |
| Psyllobora_vigintiduopunctata_562 | Subcoccinella_vigintiquatuorpunctata_625 | 0.200 |
| Psyllobora_vigintiduopunctata_565 | Subcoccinella_vigintiquatuorpunctata_625 | 0.200 |
| Psyllobora_vigintiduopunctata_567 | Subcoccinella_vigintiquatuorpunctata_625 | 0.200 |
| Psyllobora_vigintiduopunctata_561 | Subcoccinella_vigintiquatuorpunctata_626 | 0.200 |
| Psyllobora_vigintiduopunctata_562 | Subcoccinella_vigintiquatuorpunctata_626 | 0.200 |
| Psyllobora_vigintiduopunctata_565 | Subcoccinella_vigintiquatuorpunctata_626 | 0.200 |
| Psyllobora_vigintiduopunctata_567 | Subcoccinella_vigintiquatuorpunctata_626 | 0.200 |
| Chilocorus_stigma_43 | Subcoccinella_vigintiquatuorpunctata_627 | 0.200 |
| Chilocorus_stigma_45 | Subcoccinella_vigintiquatuorpunctata_627 | 0.200 |
| Psyllobora_vigintiduopunctata_570 | Subcoccinella_vigintiquatuorpunctata_627 | 0.200 |
| Psyllobora_vigintiduopunctata_572 | Subcoccinella_vigintiquatuorpunctata_627 | 0.200 |
| Psyllobora_vigintiduopunctata_570 | Subcoccinella_vigintiquatuorpunctata_628 | 0.200 |
| Psyllobora_vigintiduopunctata_572 | Subcoccinella_vigintiquatuorpunctata_628 | 0.200 |
| Psyllobora_vigintiduopunctata_570 | Subcoccinella_vigintiquatuorpunctata_629 | 0.200 |
| Psyllobora_vigintiduopunctata_572 | Subcoccinella_vigintiquatuorpunctata_629 | 0.200 |
| Chilocorus_stigma_43 | Subcoccinella_vigintiquatuorpunctata_630 | 0.200 |
| Chilocorus_stigma_45 | Subcoccinella_vigintiquatuorpunctata_630 | 0.200 |
| Psyllobora_vigintiduopunctata_557 | Subcoccinella_vigintiquatuorpunctata_630 | 0.200 |
| Psyllobora_vigintiduopunctata_563 | Subcoccinella_vigintiquatuorpunctata_630 | 0.200 |
| Psyllobora_vigintiduopunctata_564 | Subcoccinella_vigintiquatuorpunctata_630 | 0.200 |
| Psyllobora_vigintiduopunctata_570 | Subcoccinella_vigintiquatuorpunctata_631 | 0.200 |
| Psyllobora_vigintiduopunctata_572 | Subcoccinella_vigintiquatuorpunctata_631 | 0.200 |
| Scymnus_interruptus_761 | Tytthaspis_sedecimpunctata_587 | 0.200 |
| Scymnus_interruptus_763 | Tytthaspis_sedecimpunctata_587 | 0.200 |
| Scymnus_interruptus_764 | Tytthaspis_sedecimpunctata_587 | 0.200 |
| Scymnus_interruptus_766 | Tytthaspis_sedecimpunctata_587 | 0.200 |
| Scymnus_ferrugatus_724 | Tytthaspis_sedecimpunctata_591 | 0.200 |
| Scymnus_ferrugatus_723 | Tytthaspis_sedecimpunctata_593 | 0.200 |
| Scymnus_ferrugatus_725 | Tytthaspis_sedecimpunctata_593 | 0.200 |
| Scymnus_ferrugatus_726 | Tytthaspis_sedecimpunctata_593 | 0.200 |
| Scymnus_ferrugatus_727 | Tytthaspis_sedecimpunctata_593 | 0.200 |
| Scymnus_ferrugatus_728 | Tytthaspis_sedecimpunctata_593 | 0.200 |
| Scymnus_ferrugatus_730 | Tytthaspis_sedecimpunctata_593 | 0.200 |
| Scymnus_ferrugatus_731 | Tytthaspis_sedecimpunctata_593 | 0.200 |
| Scymnus_ferrugatus_732 | Tytthaspis_sedecimpunctata_593 | 0.200 |
| Scymnus_ferrugatus_733 | Tytthaspis_sedecimpunctata_593 | 0.200 |
| Hyperaspis_binotata_650 | Scymnus_rubromaculatus_822 | 0.200 |

| | | |
|---|---|---|
| Hyperaspis_binotata_650 | Scymnus_rubromaculatus_823 | 0.200 |
| Hyperaspis_binotata_650 | Scymnus_rubromaculatus_824 | 0.200 |
| Hyperaspis_binotata_650 | Scymnus_rubromaculatus_825 | 0.200 |
| Hyperaspis_binotata_650 | Scymnus_rubromaculatus_826 | 0.200 |
| Hyperaspis_binotata_650 | Scymnus_rubromaculatus_827 | 0.200 |
| Hyperaspis_campestris_651 | Scymnus_sinuanodulus_830 | 0.200 |
| Hyperaspis_campestris_652 | Scymnus_sinuanodulus_830 | 0.200 |
| Hyperaspis_campestris_653 | Scymnus_sinuanodulus_830 | 0.200 |
| Calvia_quatuordecimguttata_204 | Scymnus_suturalis_833 | 0.200 |
| Calvia_quatuordecimguttata_208 | Scymnus_suturalis_833 | 0.200 |
| Calvia_quatuordecimguttata_217 | Scymnus_suturalis_833 | 0.200 |
| Calvia_quatuordecimguttata_209 | Scymnus_suturalis_834 | 0.200 |
| Platynaspis_luteorubra_50 | Subcoccinella_vigintiquatuorpunctata_620 | 0.200 |
| Platynaspis_luteorubra_50 | Subcoccinella_vigintiquatuorpunctata_621 | 0.200 |
| Platynaspis_luteorubra_50 | Subcoccinella_vigintiquatuorpunctata_624 | 0.200 |
| Platynaspis_luteorubra_50 | Subcoccinella_vigintiquatuorpunctata_628 | 0.200 |
| Platynaspis_luteorubra_50 | Subcoccinella_vigintiquatuorpunctata_629 | 0.200 |
| Platynaspis_luteorubra_50 | Subcoccinella_vigintiquatuorpunctata_631 | 0.200 |
| Scymnus_camptodromus_715 | Vibidia_duodecimguttata_596 | 0.200 |
| Anisosticta_bitriangularis_147 | Scymnus_sinuanodulus_829 | 0.200 |
| Anisosticta_bitriangularis_151 | Scymnus_sinuanodulus_831 | 0.200 |
| Anisosticta_bitriangularis_153 | Scymnus_sinuanodulus_831 | 0.200 |
| Anisosticta_bitriangularis_154 | Scymnus_sinuanodulus_831 | 0.200 |
| Anisosticta_bitriangularis_156 | Scymnus_sinuanodulus_831 | 0.200 |
| Anisosticta_bitriangularis_157 | Scymnus_sinuanodulus_831 | 0.200 |
| Hyperaspis_reppensis_664 | Stethorus_punctillum_853 | 0.200 |
| Hyperaspis_reppensis_665 | Stethorus_punctillum_853 | 0.200 |
| Hyperaspis_reppensis_666 | Stethorus_punctillum_853 | 0.200 |
| Hyperaspis_reppensis_667 | Stethorus_punctillum_855 | 0.200 |
| Scymnus_ferrugatus_723 | Tytthaspis_sedecimpunctata_584 | 0.200 |
| Scymnus_ferrugatus_725 | Tytthaspis_sedecimpunctata_584 | 0.200 |
| Scymnus_ferrugatus_726 | Tytthaspis_sedecimpunctata_584 | 0.200 |
| Scymnus_ferrugatus_727 | Tytthaspis_sedecimpunctata_584 | 0.200 |
| Scymnus_ferrugatus_728 | Tytthaspis_sedecimpunctata_584 | 0.200 |
| Scymnus_ferrugatus_730 | Tytthaspis_sedecimpunctata_584 | 0.200 |
| Scymnus_ferrugatus_731 | Tytthaspis_sedecimpunctata_584 | 0.200 |
| Scymnus_ferrugatus_732 | Tytthaspis_sedecimpunctata_584 | 0.200 |
| Scymnus_ferrugatus_733 | Tytthaspis_sedecimpunctata_584 | 0.200 |
| Olla_vnigrum_527 | Tytthaspis_sedecimpunctata_587 | 0.200 |
| Olla_vnigrum_528 | Tytthaspis_sedecimpunctata_587 | 0.200 |
| Scymnus_ferrugatus_729 | Tytthaspis_sedecimpunctata_593 | 0.200 |
| Hyperaspis_campestris_651 | Scymnus_sinuanodulus_828 | 0.200 |
| Hyperaspis_campestris_652 | Scymnus_sinuanodulus_828 | 0.200 |
| Hyperaspis_campestris_653 | Scymnus_sinuanodulus_828 | 0.200 |
| Calvia_quatuordecimguttata_205 | Scymnus_suturalis_838 | 0.200 |
| Calvia_quatuordecimguttata_205 | Scymnus_suturalis_840 | 0.200 |
| Scymnus_ferrugatus_729 | Tytthaspis_sedecimpunctata_584 | 0.200 |
| Nephus_quadrimaculatus_668 | Scymnus_sinuanodulus_828 | 0.200 |
| Chilocorus_renipustulatus_31 | Scymnus_sinuanodulus_830 | 0.200 |
| Chilocorus_renipustulatus_32 | Scymnus_sinuanodulus_830 | 0.200 |
| Chilocorus_renipustulatus_35 | Scymnus_sinuanodulus_830 | 0.200 |
| Chilocorus_renipustulatus_36 | Scymnus_sinuanodulus_830 | 0.200 |
| Chilocorus_renipustulatus_37 | Scymnus_sinuanodulus_830 | 0.200 |
| Chilocorus_renipustulatus_38 | Scymnus_sinuanodulus_830 | 0.200 |
| Chilocorus_renipustulatus_40 | Scymnus_sinuanodulus_830 | 0.200 |
| Chilocorus_renipustulatus_41 | Scymnus_sinuanodulus_830 | 0.200 |

| | | |
|---|---|---|
| Chilocorus_renipustulatus_42 | Scymnus_sinuanodulus_830 | 0.200 |
| Calvia_quatuordecimguttata_203 | Scymnus_suturalis_833 | 0.200 |
| Calvia_quatuordecimguttata_205 | Scymnus_suturalis_837 | 0.200 |
| Myzia_pullata_517 | Subcoccinella_vigintiquatuorpunctata_620 | 0.200 |
| Scymnus_interruptus_760 | Subcoccinella_vigintiquatuorpunctata_620 | 0.200 |
| Scymnus_interruptus_765 | Subcoccinella_vigintiquatuorpunctata_620 | 0.200 |
| Scymnus_interruptus_768 | Subcoccinella_vigintiquatuorpunctata_620 | 0.200 |
| Myzia_pullata_517 | Subcoccinella_vigintiquatuorpunctata_621 | 0.200 |
| Scymnus_interruptus_760 | Subcoccinella_vigintiquatuorpunctata_621 | 0.200 |
| Scymnus_interruptus_765 | Subcoccinella_vigintiquatuorpunctata_621 | 0.200 |
| Scymnus_interruptus_768 | Subcoccinella_vigintiquatuorpunctata_621 | 0.200 |
| Scymnus_interruptus_767 | Subcoccinella_vigintiquatuorpunctata_622 | 0.200 |
| Scymnus_camptodromus_712 | Subcoccinella_vigintiquatuorpunctata_623 | 0.200 |
| Scymnus_interruptus_767 | Subcoccinella_vigintiquatuorpunctata_625 | 0.200 |
| Scymnus_interruptus_767 | Subcoccinella_vigintiquatuorpunctata_626 | 0.200 |
| Myzia_pullata_517 | Subcoccinella_vigintiquatuorpunctata_628 | 0.200 |
| Scymnus_interruptus_760 | Subcoccinella_vigintiquatuorpunctata_628 | 0.200 |
| Scymnus_interruptus_765 | Subcoccinella_vigintiquatuorpunctata_628 | 0.200 |
| Scymnus_interruptus_768 | Subcoccinella_vigintiquatuorpunctata_628 | 0.200 |
| Myzia_pullata_517 | Subcoccinella_vigintiquatuorpunctata_629 | 0.200 |
| Scymnus_interruptus_760 | Subcoccinella_vigintiquatuorpunctata_629 | 0.200 |
| Scymnus_interruptus_765 | Subcoccinella_vigintiquatuorpunctata_629 | 0.200 |
| Scymnus_interruptus_768 | Subcoccinella_vigintiquatuorpunctata_629 | 0.200 |
| Myzia_pullata_517 | Subcoccinella_vigintiquatuorpunctata_631 | 0.200 |
| Scymnus_interruptus_760 | Subcoccinella_vigintiquatuorpunctata_631 | 0.200 |
| Scymnus_interruptus_765 | Subcoccinella_vigintiquatuorpunctata_631 | 0.200 |
| Scymnus_interruptus_768 | Subcoccinella_vigintiquatuorpunctata_631 | 0.200 |
| Scymnus_abietis_682 | Tytthaspis_sedecimpunctata_585 | 0.200 |
| Scymnus_abietis_683 | Tytthaspis_sedecimpunctata_585 | 0.200 |
| Scymnus_abietis_685 | Tytthaspis_sedecimpunctata_585 | 0.200 |
| Scymnus_abietis_684 | Tytthaspis_sedecimpunctata_589 | 0.200 |
| Scymnus_abietis_686 | Tytthaspis_sedecimpunctata_589 | 0.200 |
| Scymnus_abietis_687 | Tytthaspis_sedecimpunctata_589 | 0.200 |
| Scymnus_abietis_688 | Tytthaspis_sedecimpunctata_589 | 0.200 |
| Coccidula_rufa_69 | Scymnus_rubromaculatus_822 | 0.200 |
| Coccidula_rufa_69 | Scymnus_rubromaculatus_823 | 0.200 |
| Coccidula_rufa_69 | Scymnus_rubromaculatus_824 | 0.200 |
| Coccidula_rufa_69 | Scymnus_rubromaculatus_825 | 0.200 |
| Coccidula_rufa_69 | Scymnus_rubromaculatus_826 | 0.200 |
| Coccidula_rufa_69 | Scymnus_rubromaculatus_827 | 0.200 |
| Chilocorus_stigma_46 | Subcoccinella_vigintiquatuorpunctata_619 | 0.200 |
| Psyllobora_vigintiduopunctata_561 | Subcoccinella_vigintiquatuorpunctata_619 | 0.200 |
| Psyllobora_vigintiduopunctata_562 | Subcoccinella_vigintiquatuorpunctata_619 | 0.200 |
| Psyllobora_vigintiduopunctata_565 | Subcoccinella_vigintiquatuorpunctata_619 | 0.200 |
| Psyllobora_vigintiduopunctata_567 | Subcoccinella_vigintiquatuorpunctata_619 | 0.200 |
| Psyllobora_vigintiduopunctata_569 | Subcoccinella_vigintiquatuorpunctata_624 | 0.200 |
| Psyllobora_vigintiduopunctata_569 | Subcoccinella_vigintiquatuorpunctata_630 | 0.200 |
| Anisosticta_bitriangularis_149 | Scymnus_sinuanodulus_831 | 0.200 |
| Scymnus_camptodromus_712 | Subcoccinella_vigintiquatuorpunctata_622 | 0.200 |
| Scymnus_interruptus_760 | Subcoccinella_vigintiquatuorpunctata_623 | 0.200 |
| Scymnus_interruptus_765 | Subcoccinella_vigintiquatuorpunctata_623 | 0.200 |
| Scymnus_interruptus_768 | Subcoccinella_vigintiquatuorpunctata_623 | 0.200 |
| Scymnus_camptodromus_718 | Subcoccinella_vigintiquatuorpunctata_624 | 0.200 |
| Coccidula_scutellata_78 | Subcoccinella_vigintiquatuorpunctata_625 | 0.200 |
| Coccidula_scutellata_79 | Subcoccinella_vigintiquatuorpunctata_625 | 0.200 |
| Coccidula_scutellata_83 | Subcoccinella_vigintiquatuorpunctata_625 | 0.200 |

| | | |
|---|---|---|
| Scymnus_camptodromus_712 | Subcoccinella_vigintiquatuorpunctata_626 | 0.200 |
| Scymnus_abietis_684 | Tytthaspis_sedecimpunctata_584 | 0.200 |
| Scymnus_abietis_686 | Tytthaspis_sedecimpunctata_584 | 0.200 |
| Scymnus_abietis_687 | Tytthaspis_sedecimpunctata_584 | 0.200 |
| Scymnus_abietis_688 | Tytthaspis_sedecimpunctata_584 | 0.200 |
| Hippodamia_undecimnotata_460 | Vibidia_duodecimguttata_596 | 0.200 |
| Hippodamia_undecimnotata_461 | Vibidia_duodecimguttata_596 | 0.200 |
| Hippodamia_undecimnotata_462 | Vibidia_duodecimguttata_596 | 0.200 |
| Hippodamia_undecimnotata_463 | Vibidia_duodecimguttata_596 | 0.200 |
| Hippodamia_undecimnotata_464 | Vibidia_duodecimguttata_596 | 0.200 |
| Hippodamia_undecimnotata_465 | Vibidia_duodecimguttata_596 | 0.200 |
| Hippodamia_undecimnotata_466 | Vibidia_duodecimguttata_596 | 0.200 |
| Hippodamia_parenthesis_433 | Vibidia_duodecimguttata_603 | 0.200 |
| Hippodamia_convergens_393 | Scymnus_suturalis_833 | 0.200 |
| Hippodamia_convergens_394 | Scymnus_suturalis_833 | 0.200 |
| Hippodamia_convergens_397 | Scymnus_suturalis_833 | 0.200 |
| Hippodamia_convergens_398 | Scymnus_suturalis_833 | 0.200 |
| Hippodamia_convergens_401 | Scymnus_suturalis_833 | 0.200 |
| Hippodamia_convergens_402 | Scymnus_suturalis_833 | 0.200 |
| Hippodamia_convergens_399 | Scymnus_suturalis_838 | 0.200 |
| Hippodamia_convergens_404 | Scymnus_suturalis_838 | 0.200 |
| Hippodamia_convergens_405 | Scymnus_suturalis_838 | 0.200 |
| Hippodamia_convergens_406 | Scymnus_suturalis_838 | 0.200 |
| Hippodamia_quinquesignata_443 | Scymnus_suturalis_838 | 0.200 |
| Hippodamia_quinquesignata_444 | Scymnus_suturalis_838 | 0.200 |
| Hippodamia_quinquesignata_445 | Scymnus_suturalis_838 | 0.200 |
| Hippodamia_convergens_399 | Scymnus_suturalis_840 | 0.200 |
| Hippodamia_convergens_404 | Scymnus_suturalis_840 | 0.200 |
| Hippodamia_convergens_405 | Scymnus_suturalis_840 | 0.200 |
| Hippodamia_convergens_406 | Scymnus_suturalis_840 | 0.200 |
| Hippodamia_quinquesignata_443 | Scymnus_suturalis_840 | 0.200 |
| Hippodamia_quinquesignata_444 | Scymnus_suturalis_840 | 0.200 |
| Hippodamia_quinquesignata_445 | Scymnus_suturalis_840 | 0.200 |
| Platynaspis_luteorubra_51 | Subcoccinella_vigintiquatuorpunctata_619 | 0.200 |
| Platynaspis_luteorubra_53 | Subcoccinella_vigintiquatuorpunctata_619 | 0.200 |
| Platynaspis_luteorubra_58 | Subcoccinella_vigintiquatuorpunctata_619 | 0.200 |
| Psyllobora_vigintiduopunctata_566 | Subcoccinella_vigintiquatuorpunctata_630 | 0.200 |
| Chilocorus_renipustulatus_33 | Scymnus_sinuanodulus_831 | 0.200 |
| Myzia_oblongoguttata_504 | Scymnus_sinuanodulus_829 | 0.200 |
| Myzia_oblongoguttata_505 | Scymnus_sinuanodulus_829 | 0.200 |
| Myzia_oblongoguttata_507 | Scymnus_sinuanodulus_829 | 0.200 |
| Myzia_oblongoguttata_508 | Scymnus_sinuanodulus_829 | 0.200 |
| Myzia_oblongoguttata_509 | Scymnus_sinuanodulus_829 | 0.200 |
| Myzia_oblongoguttata_510 | Scymnus_sinuanodulus_829 | 0.200 |
| Myzia_oblongoguttata_511 | Scymnus_sinuanodulus_829 | 0.200 |
| Myzia_oblongoguttata_512 | Scymnus_sinuanodulus_829 | 0.200 |
| Myzia_oblongoguttata_513 | Scymnus_sinuanodulus_829 | 0.200 |
| Myzia_oblongoguttata_514 | Scymnus_sinuanodulus_829 | 0.200 |
| Myzia_oblongoguttata_516 | Scymnus_sinuanodulus_829 | 0.200 |
| Anisosticta_bitriangularis_150 | Scymnus_sinuanodulus_829 | 0.200 |
| Hippodamia_caseyi_391 | Scymnus_suturalis_841 | 0.200 |
| Hippodamia_moesta_411 | Scymnus_suturalis_841 | 0.200 |
| Hippodamia_caseyi_391 | Scymnus_suturalis_842 | 0.200 |
| Hippodamia_moesta_411 | Scymnus_suturalis_842 | 0.200 |
| Hippodamia_caseyi_392 | Scymnus_suturalis_843 | 0.200 |
| Hippodamia_caseyi_391 | Scymnus_suturalis_844 | 0.200 |

| | | |
|---|---|---|
| Hippodamia_moesta_411 | Scymnus_suturalis_844 | 0.200 |
| Hippodamia_caseyi_392 | Scymnus_suturalis_845 | 0.200 |
| Hippodamia_caseyi_391 | Scymnus_suturalis_846 | 0.200 |
| Hippodamia_moesta_411 | Scymnus_suturalis_846 | 0.200 |
| Hippodamia_caseyi_391 | Scymnus_suturalis_847 | 0.200 |
| Hippodamia_moesta_411 | Scymnus_suturalis_847 | 0.200 |
| Hippodamia_caseyi_391 | Scymnus_suturalis_848 | 0.200 |
| Hippodamia_moesta_411 | Scymnus_suturalis_848 | 0.200 |
| Scymnus_camptodromus_713 | Subcoccinella_vigintiquatuorpunctata_620 | 0.200 |
| Scymnus_camptodromus_716 | Subcoccinella_vigintiquatuorpunctata_620 | 0.200 |
| Scymnus_ferrugatus_724 | Subcoccinella_vigintiquatuorpunctata_620 | 0.200 |
| Scymnus_camptodromus_713 | Subcoccinella_vigintiquatuorpunctata_621 | 0.200 |
| Scymnus_camptodromus_716 | Subcoccinella_vigintiquatuorpunctata_621 | 0.200 |
| Scymnus_ferrugatus_724 | Subcoccinella_vigintiquatuorpunctata_621 | 0.200 |
| Scymnus_camptodromus_705 | Subcoccinella_vigintiquatuorpunctata_622 | 0.200 |
| Scymnus_ferrugatus_729 | Subcoccinella_vigintiquatuorpunctata_622 | 0.200 |
| Scymnus_mitior_779 | Subcoccinella_vigintiquatuorpunctata_625 | 0.200 |
| Scymnus_mitior_780 | Subcoccinella_vigintiquatuorpunctata_625 | 0.200 |
| Scymnus_ferrugatus_729 | Subcoccinella_vigintiquatuorpunctata_626 | 0.200 |
| Scymnus_camptodromus_713 | Subcoccinella_vigintiquatuorpunctata_627 | 0.200 |
| Scymnus_camptodromus_716 | Subcoccinella_vigintiquatuorpunctata_627 | 0.200 |
| Scymnus_ferrugatus_729 | Subcoccinella_vigintiquatuorpunctata_627 | 0.200 |
| Scymnus_camptodromus_713 | Subcoccinella_vigintiquatuorpunctata_628 | 0.200 |
| Scymnus_camptodromus_716 | Subcoccinella_vigintiquatuorpunctata_628 | 0.200 |
| Scymnus_ferrugatus_724 | Subcoccinella_vigintiquatuorpunctata_628 | 0.200 |
| Scymnus_camptodromus_713 | Subcoccinella_vigintiquatuorpunctata_629 | 0.200 |
| Scymnus_camptodromus_716 | Subcoccinella_vigintiquatuorpunctata_629 | 0.200 |
| Scymnus_ferrugatus_724 | Subcoccinella_vigintiquatuorpunctata_629 | 0.200 |
| Scymnus_camptodromus_713 | Subcoccinella_vigintiquatuorpunctata_630 | 0.200 |
| Scymnus_camptodromus_716 | Subcoccinella_vigintiquatuorpunctata_630 | 0.200 |
| Scymnus_ferrugatus_729 | Subcoccinella_vigintiquatuorpunctata_630 | 0.200 |
| Scymnus_camptodromus_713 | Subcoccinella_vigintiquatuorpunctata_631 | 0.200 |
| Scymnus_camptodromus_716 | Subcoccinella_vigintiquatuorpunctata_631 | 0.200 |
| Scymnus_ferrugatus_724 | Subcoccinella_vigintiquatuorpunctata_631 | 0.200 |
| Mulsantina_hudsonica_479 | Vibidia_duodecimguttata_597 | 0.200 |
| Mulsantina_hudsonica_480 | Vibidia_duodecimguttata_597 | 0.200 |
| Mulsantina_hudsonica_483 | Vibidia_duodecimguttata_597 | 0.200 |
| Chilocorus_nigritus_25 | Vibidia_duodecimguttata_598 | 0.200 |
| Mulsantina_hudsonica_479 | Vibidia_duodecimguttata_600 | 0.200 |
| Mulsantina_hudsonica_480 | Vibidia_duodecimguttata_600 | 0.200 |
| Mulsantina_hudsonica_483 | Vibidia_duodecimguttata_600 | 0.200 |
| Chilocorus_nigritus_25 | Vibidia_duodecimguttata_601 | 0.200 |
| Adalia_bipunctata_101 | Vibidia_duodecimguttata_603 | 0.200 |
| Adalia_bipunctata_104 | Vibidia_duodecimguttata_603 | 0.200 |
| Clitostethus_arcuatus_639 | Scymnus_sinuanodulus_830 | 0.200 |
| Clitostethus_arcuatus_640 | Scymnus_sinuanodulus_830 | 0.200 |
| Clitostethus_arcuatus_641 | Scymnus_sinuanodulus_830 | 0.200 |
| Clitostethus_arcuatus_643 | Scymnus_sinuanodulus_830 | 0.200 |
| Scymnus_nubilus_798 | Scymnus_sinuanodulus_830 | 0.200 |
| Scymnus_nubilus_800 | Scymnus_sinuanodulus_830 | 0.200 |
| Scymnus_nubilus_801 | Scymnus_sinuanodulus_830 | 0.200 |
| Scymnus_nubilus_802 | Scymnus_sinuanodulus_830 | 0.200 |
| Scymnus_nubilus_805 | Scymnus_sinuanodulus_830 | 0.200 |
| Scymnus_nubilus_806 | Scymnus_sinuanodulus_830 | 0.200 |
| Scymnus_nubilus_807 | Scymnus_sinuanodulus_830 | 0.200 |
| Scymnus_nubilus_810 | Scymnus_sinuanodulus_830 | 0.200 |

| | | |
|---|---|---|
| Hippodamia_caseyi_391 | Scymnus_suturalis_832 | 0.200 |
| Hippodamia_moesta_411 | Scymnus_suturalis_832 | 0.200 |
| Hippodamia_convergens_399 | Scymnus_suturalis_834 | 0.200 |
| Hippodamia_convergens_404 | Scymnus_suturalis_834 | 0.200 |
| Hippodamia_convergens_405 | Scymnus_suturalis_834 | 0.200 |
| Hippodamia_convergens_406 | Scymnus_suturalis_834 | 0.200 |
| Hippodamia_quinquesignata_443 | Scymnus_suturalis_834 | 0.200 |
| Hippodamia_quinquesignata_444 | Scymnus_suturalis_834 | 0.200 |
| Hippodamia_quinquesignata_445 | Scymnus_suturalis_834 | 0.200 |
| Hippodamia_caseyi_391 | Scymnus_suturalis_835 | 0.200 |
| Hippodamia_moesta_411 | Scymnus_suturalis_835 | 0.200 |
| Hippodamia_caseyi_391 | Scymnus_suturalis_836 | 0.200 |
| Hippodamia_moesta_411 | Scymnus_suturalis_836 | 0.200 |
| Hippodamia_convergens_399 | Scymnus_suturalis_837 | 0.200 |
| Hippodamia_convergens_404 | Scymnus_suturalis_837 | 0.200 |
| Hippodamia_convergens_405 | Scymnus_suturalis_837 | 0.200 |
| Hippodamia_convergens_406 | Scymnus_suturalis_837 | 0.200 |
| Hippodamia_quinquesignata_443 | Scymnus_suturalis_837 | 0.200 |
| Hippodamia_quinquesignata_444 | Scymnus_suturalis_837 | 0.200 |
| Hippodamia_quinquesignata_445 | Scymnus_suturalis_837 | 0.200 |
| Hippodamia_caseyi_391 | Scymnus_suturalis_839 | 0.200 |
| Hippodamia_moesta_411 | Scymnus_suturalis_839 | 0.200 |
| Scymnus_apetzi_695 | Tytthaspis_sedecimpunctata_577 | 0.201 |
| Scymnus_apetzi_695 | Tytthaspis_sedecimpunctata_578 | 0.201 |
| Scymnus_apetzi_695 | Tytthaspis_sedecimpunctata_579 | 0.201 |
| Scymnus_apetzi_695 | Tytthaspis_sedecimpunctata_581 | 0.201 |
| Scymnus_apetzi_695 | Tytthaspis_sedecimpunctata_582 | 0.201 |
| Scymnus_apetzi_695 | Tytthaspis_sedecimpunctata_585 | 0.201 |
| Scymnus_apetzi_695 | Tytthaspis_sedecimpunctata_590 | 0.201 |
| Scymnus_apetzi_695 | Tytthaspis_sedecimpunctata_594 | 0.201 |
| Scymnus_apetzi_695 | Tytthaspis_sedecimpunctata_595 | 0.201 |
| Scymnus_apetzi_693 | Tytthaspis_sedecimpunctata_577 | 0.201 |
| Scymnus_apetzi_693 | Tytthaspis_sedecimpunctata_578 | 0.201 |
| Scymnus_apetzi_693 | Tytthaspis_sedecimpunctata_579 | 0.201 |
| Scymnus_apetzi_693 | Tytthaspis_sedecimpunctata_581 | 0.201 |
| Scymnus_apetzi_693 | Tytthaspis_sedecimpunctata_582 | 0.201 |
| Scymnus_apetzi_695 | Tytthaspis_sedecimpunctata_584 | 0.201 |
| Scymnus_apetzi_695 | Tytthaspis_sedecimpunctata_586 | 0.201 |
| Scymnus_apetzi_693 | Tytthaspis_sedecimpunctata_589 | 0.201 |
| Scymnus_apetzi_693 | Tytthaspis_sedecimpunctata_590 | 0.201 |
| Scymnus_apetzi_693 | Tytthaspis_sedecimpunctata_594 | 0.201 |
| Scymnus_apetzi_693 | Tytthaspis_sedecimpunctata_595 | 0.201 |
| Scymnus_interruptus_767 | Subcoccinella_vigintiquatuorpunctata_619 | 0.201 |
| Anisosticta_bitriangularis_151 | Scymnus_sinuanodulus_829 | 0.201 |
| Anisosticta_bitriangularis_153 | Scymnus_sinuanodulus_829 | 0.201 |
| Anisosticta_bitriangularis_154 | Scymnus_sinuanodulus_829 | 0.201 |
| Anisosticta_bitriangularis_156 | Scymnus_sinuanodulus_829 | 0.201 |
| Anisosticta_bitriangularis_157 | Scymnus_sinuanodulus_829 | 0.201 |
| Psyllobora_vigintiduopunctata_568 | Subcoccinella_vigintiquatuorpunctata_623 | 0.201 |
| Scymnus_ferrugatus_723 | Subcoccinella_vigintiquatuorpunctata_622 | 0.201 |
| Scymnus_ferrugatus_725 | Subcoccinella_vigintiquatuorpunctata_622 | 0.201 |
| Scymnus_ferrugatus_726 | Subcoccinella_vigintiquatuorpunctata_622 | 0.201 |
| Scymnus_ferrugatus_727 | Subcoccinella_vigintiquatuorpunctata_622 | 0.201 |
| Scymnus_ferrugatus_728 | Subcoccinella_vigintiquatuorpunctata_622 | 0.201 |
| Scymnus_ferrugatus_730 | Subcoccinella_vigintiquatuorpunctata_622 | 0.201 |
| Scymnus_ferrugatus_732 | Subcoccinella_vigintiquatuorpunctata_622 | 0.201 |

| | | |
|---|---|---|
| Scymnus_ferrugatus_733 | Subcoccinella_vigintiquatuorpunctata_622 | 0.201 |
| Scymnus_abietis_682 | Subcoccinella_vigintiquatuorpunctata_623 | 0.201 |
| Scymnus_abietis_685 | Subcoccinella_vigintiquatuorpunctata_623 | 0.201 |
| Scymnus_ferrugatus_724 | Subcoccinella_vigintiquatuorpunctata_623 | 0.201 |
| Scymnus_ferrugatus_723 | Subcoccinella_vigintiquatuorpunctata_626 | 0.201 |
| Scymnus_ferrugatus_725 | Subcoccinella_vigintiquatuorpunctata_626 | 0.201 |
| Scymnus_ferrugatus_726 | Subcoccinella_vigintiquatuorpunctata_626 | 0.201 |
| Scymnus_ferrugatus_727 | Subcoccinella_vigintiquatuorpunctata_626 | 0.201 |
| Scymnus_ferrugatus_728 | Subcoccinella_vigintiquatuorpunctata_626 | 0.201 |
| Scymnus_ferrugatus_730 | Subcoccinella_vigintiquatuorpunctata_626 | 0.201 |
| Scymnus_ferrugatus_732 | Subcoccinella_vigintiquatuorpunctata_626 | 0.201 |
| Scymnus_ferrugatus_733 | Subcoccinella_vigintiquatuorpunctata_626 | 0.201 |
| Brachiacantha_ursina_637 | Subcoccinella_vigintiquatuorpunctata_627 | 0.201 |
| Calvia_quatuordecimguttata_209 | Subcoccinella_vigintiquatuorpunctata_627 | 0.201 |
| Scymnus_ferrugatus_723 | Subcoccinella_vigintiquatuorpunctata_627 | 0.201 |
| Scymnus_ferrugatus_725 | Subcoccinella_vigintiquatuorpunctata_627 | 0.201 |
| Scymnus_ferrugatus_726 | Subcoccinella_vigintiquatuorpunctata_627 | 0.201 |
| Scymnus_ferrugatus_727 | Subcoccinella_vigintiquatuorpunctata_627 | 0.201 |
| Scymnus_ferrugatus_728 | Subcoccinella_vigintiquatuorpunctata_627 | 0.201 |
| Scymnus_ferrugatus_730 | Subcoccinella_vigintiquatuorpunctata_627 | 0.201 |
| Scymnus_ferrugatus_732 | Subcoccinella_vigintiquatuorpunctata_627 | 0.201 |
| Scymnus_ferrugatus_733 | Subcoccinella_vigintiquatuorpunctata_627 | 0.201 |
| Calvia_quatuordecimguttata_209 | Subcoccinella_vigintiquatuorpunctata_630 | 0.201 |
| Scymnus_ferrugatus_723 | Subcoccinella_vigintiquatuorpunctata_630 | 0.201 |
| Scymnus_ferrugatus_725 | Subcoccinella_vigintiquatuorpunctata_630 | 0.201 |
| Scymnus_ferrugatus_726 | Subcoccinella_vigintiquatuorpunctata_630 | 0.201 |
| Scymnus_ferrugatus_727 | Subcoccinella_vigintiquatuorpunctata_630 | 0.201 |
| Scymnus_ferrugatus_728 | Subcoccinella_vigintiquatuorpunctata_630 | 0.201 |
| Scymnus_ferrugatus_730 | Subcoccinella_vigintiquatuorpunctata_630 | 0.201 |
| Scymnus_ferrugatus_732 | Subcoccinella_vigintiquatuorpunctata_630 | 0.201 |
| Scymnus_ferrugatus_733 | Subcoccinella_vigintiquatuorpunctata_630 | 0.201 |
| Adalia_bipunctata_105 | Vibidia_duodecimguttata_596 | 0.201 |
| Adalia_bipunctata_99 | Vibidia_duodecimguttata_596 | 0.201 |
| Mulsantina_hudsonica_481 | Vibidia_duodecimguttata_596 | 0.201 |
| Adalia_bipunctata_100 | Vibidia_duodecimguttata_597 | 0.201 |
| Adalia_bipunctata_102 | Vibidia_duodecimguttata_597 | 0.201 |
| Adalia_bipunctata_103 | Vibidia_duodecimguttata_597 | 0.201 |
| Adalia_bipunctata_106 | Vibidia_duodecimguttata_597 | 0.201 |
| Adalia_bipunctata_107 | Vibidia_duodecimguttata_597 | 0.201 |
| Adalia_bipunctata_108 | Vibidia_duodecimguttata_597 | 0.201 |
| Adalia_bipunctata_109 | Vibidia_duodecimguttata_597 | 0.201 |
| Adalia_bipunctata_105 | Vibidia_duodecimguttata_598 | 0.201 |
| Adalia_bipunctata_99 | Vibidia_duodecimguttata_598 | 0.201 |
| Mulsantina_hudsonica_481 | Vibidia_duodecimguttata_598 | 0.201 |
| Mulsantina_hudsonica_482 | Vibidia_duodecimguttata_598 | 0.201 |
| Mulsantina_hudsonica_479 | Vibidia_duodecimguttata_599 | 0.201 |
| Adalia_bipunctata_100 | Vibidia_duodecimguttata_600 | 0.201 |
| Adalia_bipunctata_102 | Vibidia_duodecimguttata_600 | 0.201 |
| Adalia_bipunctata_103 | Vibidia_duodecimguttata_600 | 0.201 |
| Adalia_bipunctata_106 | Vibidia_duodecimguttata_600 | 0.201 |
| Adalia_bipunctata_107 | Vibidia_duodecimguttata_600 | 0.201 |
| Adalia_bipunctata_108 | Vibidia_duodecimguttata_600 | 0.201 |
| Adalia_bipunctata_109 | Vibidia_duodecimguttata_600 | 0.201 |
| Mulsantina_hudsonica_482 | Vibidia_duodecimguttata_601 | 0.201 |
| Mulsantina_hudsonica_479 | Vibidia_duodecimguttata_602 | 0.201 |
| Mulsantina_hudsonica_481 | Vibidia_duodecimguttata_603 | 0.201 |

| | | |
|---|---|---|
| Clitostethus_arcuatus_642 | Scymnus_sinuanodulus_828 | 0.201 |
| Scymnus_nubilus_798 | Scymnus_sinuanodulus_828 | 0.201 |
| Scymnus_nubilus_800 | Scymnus_sinuanodulus_828 | 0.201 |
| Scymnus_nubilus_801 | Scymnus_sinuanodulus_828 | 0.201 |
| Scymnus_nubilus_802 | Scymnus_sinuanodulus_828 | 0.201 |
| Scymnus_nubilus_805 | Scymnus_sinuanodulus_828 | 0.201 |
| Scymnus_nubilus_806 | Scymnus_sinuanodulus_828 | 0.201 |
| Scymnus_nubilus_807 | Scymnus_sinuanodulus_828 | 0.201 |
| Scymnus_nubilus_810 | Scymnus_sinuanodulus_828 | 0.201 |
| Scymnus_apetzi_693 | Tytthaspis_sedecimpunctata_584 | 0.201 |
| Scymnus_apetzi_693 | Tytthaspis_sedecimpunctata_588 | 0.201 |
| Psyllobora_renifer_551 | Vibidia_duodecimguttata_596 | 0.201 |
| Psyllobora_renifer_552 | Vibidia_duodecimguttata_596 | 0.201 |
| Psyllobora_renifer_553 | Vibidia_duodecimguttata_596 | 0.201 |
| Psyllobora_renifer_554 | Vibidia_duodecimguttata_596 | 0.201 |
| Psyllobora_renifer_551 | Vibidia_duodecimguttata_598 | 0.201 |
| Psyllobora_renifer_552 | Vibidia_duodecimguttata_598 | 0.201 |
| Psyllobora_renifer_553 | Vibidia_duodecimguttata_598 | 0.201 |
| Psyllobora_renifer_554 | Vibidia_duodecimguttata_598 | 0.201 |
| Psyllobora_renifer_551 | Vibidia_duodecimguttata_599 | 0.201 |
| Psyllobora_renifer_552 | Vibidia_duodecimguttata_599 | 0.201 |
| Psyllobora_renifer_553 | Vibidia_duodecimguttata_599 | 0.201 |
| Psyllobora_renifer_554 | Vibidia_duodecimguttata_599 | 0.201 |
| Psyllobora_renifer_551 | Vibidia_duodecimguttata_601 | 0.201 |
| Psyllobora_renifer_552 | Vibidia_duodecimguttata_601 | 0.201 |
| Psyllobora_renifer_553 | Vibidia_duodecimguttata_601 | 0.201 |
| Psyllobora_renifer_554 | Vibidia_duodecimguttata_601 | 0.201 |
| Psyllobora_renifer_551 | Vibidia_duodecimguttata_602 | 0.201 |
| Psyllobora_renifer_552 | Vibidia_duodecimguttata_602 | 0.201 |
| Psyllobora_renifer_553 | Vibidia_duodecimguttata_602 | 0.201 |
| Psyllobora_renifer_554 | Vibidia_duodecimguttata_602 | 0.201 |
| Scymnus_camptodromus_712 | Subcoccinella_vigintiquatuorpunctata_619 | 0.201 |
| Scymnus_abietis_689 | Tytthaspis_sedecimpunctata_585 | 0.201 |
| Psyllobora_vigintiduopunctata_568 | Subcoccinella_vigintiquatuorpunctata_622 | 0.201 |
| Psyllobora_vigintiduopunctata_568 | Subcoccinella_vigintiquatuorpunctata_625 | 0.201 |
| Psyllobora_vigintiduopunctata_568 | Subcoccinella_vigintiquatuorpunctata_626 | 0.201 |
| Scymnus_abietis_683 | Subcoccinella_vigintiquatuorpunctata_620 | 0.201 |
| Scymnus_abietis_684 | Subcoccinella_vigintiquatuorpunctata_620 | 0.201 |
| Scymnus_abietis_686 | Subcoccinella_vigintiquatuorpunctata_620 | 0.201 |
| Scymnus_abietis_687 | Subcoccinella_vigintiquatuorpunctata_620 | 0.201 |
| Scymnus_abietis_688 | Subcoccinella_vigintiquatuorpunctata_620 | 0.201 |
| Scymnus_abietis_683 | Subcoccinella_vigintiquatuorpunctata_621 | 0.201 |
| Scymnus_abietis_684 | Subcoccinella_vigintiquatuorpunctata_621 | 0.201 |
| Scymnus_abietis_686 | Subcoccinella_vigintiquatuorpunctata_621 | 0.201 |
| Scymnus_abietis_687 | Subcoccinella_vigintiquatuorpunctata_621 | 0.201 |
| Scymnus_abietis_688 | Subcoccinella_vigintiquatuorpunctata_621 | 0.201 |
| Scymnus_abietis_682 | Subcoccinella_vigintiquatuorpunctata_622 | 0.201 |
| Scymnus_abietis_682 | Subcoccinella_vigintiquatuorpunctata_626 | 0.201 |
| Scymnus_abietis_685 | Subcoccinella_vigintiquatuorpunctata_626 | 0.201 |
| Calvia_quatuordecimguttata_201 | Subcoccinella_vigintiquatuorpunctata_627 | 0.201 |
| Calvia_quatuordecimguttata_202 | Subcoccinella_vigintiquatuorpunctata_627 | 0.201 |
| Calvia_quatuordecimguttata_210 | Subcoccinella_vigintiquatuorpunctata_627 | 0.201 |
| Scymnus_abietis_683 | Subcoccinella_vigintiquatuorpunctata_627 | 0.201 |
| Scymnus_abietis_684 | Subcoccinella_vigintiquatuorpunctata_627 | 0.201 |
| Scymnus_abietis_686 | Subcoccinella_vigintiquatuorpunctata_627 | 0.201 |
| Scymnus_abietis_687 | Subcoccinella_vigintiquatuorpunctata_627 | 0.201 |

Supplementary Table S4 (continued)

| | | |
|---|---|---|
| Scymnus_abietis_688 | Subcoccinella_vigintiquatuorpunctata_627 | 0.201 |
| Scymnus_abietis_683 | Subcoccinella_vigintiquatuorpunctata_628 | 0.201 |
| Scymnus_abietis_684 | Subcoccinella_vigintiquatuorpunctata_628 | 0.201 |
| Scymnus_abietis_686 | Subcoccinella_vigintiquatuorpunctata_628 | 0.201 |
| Scymnus_abietis_687 | Subcoccinella_vigintiquatuorpunctata_628 | 0.201 |
| Scymnus_abietis_688 | Subcoccinella_vigintiquatuorpunctata_628 | 0.201 |
| Scymnus_abietis_683 | Subcoccinella_vigintiquatuorpunctata_629 | 0.201 |
| Scymnus_abietis_684 | Subcoccinella_vigintiquatuorpunctata_629 | 0.201 |
| Scymnus_abietis_686 | Subcoccinella_vigintiquatuorpunctata_629 | 0.201 |
| Scymnus_abietis_687 | Subcoccinella_vigintiquatuorpunctata_629 | 0.201 |
| Scymnus_abietis_688 | Subcoccinella_vigintiquatuorpunctata_629 | 0.201 |
| Calvia_quatuordecimguttata_201 | Subcoccinella_vigintiquatuorpunctata_630 | 0.201 |
| Calvia_quatuordecimguttata_202 | Subcoccinella_vigintiquatuorpunctata_630 | 0.201 |
| Calvia_quatuordecimguttata_210 | Subcoccinella_vigintiquatuorpunctata_630 | 0.201 |
| Rhyzobius_chrysomeloides_85 | Subcoccinella_vigintiquatuorpunctata_630 | 0.201 |
| Rhyzobius_chrysomeloides_86 | Subcoccinella_vigintiquatuorpunctata_630 | 0.201 |
| Rhyzobius_chrysomeloides_87 | Subcoccinella_vigintiquatuorpunctata_630 | 0.201 |
| Rhyzobius_chrysomeloides_88 | Subcoccinella_vigintiquatuorpunctata_630 | 0.201 |
| Rhyzobius_chrysomeloides_89 | Subcoccinella_vigintiquatuorpunctata_630 | 0.201 |
| Rhyzobius_chrysomeloides_90 | Subcoccinella_vigintiquatuorpunctata_630 | 0.201 |
| Rhyzobius_chrysomeloides_91 | Subcoccinella_vigintiquatuorpunctata_630 | 0.201 |
| Rhyzobius_chrysomeloides_92 | Subcoccinella_vigintiquatuorpunctata_630 | 0.201 |
| Rhyzobius_chrysomeloides_93 | Subcoccinella_vigintiquatuorpunctata_630 | 0.201 |
| Rhyzobius_chrysomeloides_94 | Subcoccinella_vigintiquatuorpunctata_630 | 0.201 |
| Rhyzobius_chrysomeloides_95 | Subcoccinella_vigintiquatuorpunctata_630 | 0.201 |
| Scymnus_abietis_683 | Subcoccinella_vigintiquatuorpunctata_630 | 0.201 |
| Scymnus_abietis_684 | Subcoccinella_vigintiquatuorpunctata_630 | 0.201 |
| Scymnus_abietis_686 | Subcoccinella_vigintiquatuorpunctata_630 | 0.201 |
| Scymnus_abietis_687 | Subcoccinella_vigintiquatuorpunctata_630 | 0.201 |
| Scymnus_abietis_688 | Subcoccinella_vigintiquatuorpunctata_630 | 0.201 |
| Scymnus_abietis_683 | Subcoccinella_vigintiquatuorpunctata_631 | 0.201 |
| Scymnus_abietis_684 | Subcoccinella_vigintiquatuorpunctata_631 | 0.201 |
| Scymnus_abietis_686 | Subcoccinella_vigintiquatuorpunctata_631 | 0.201 |
| Scymnus_abietis_687 | Subcoccinella_vigintiquatuorpunctata_631 | 0.201 |
| Scymnus_abietis_688 | Subcoccinella_vigintiquatuorpunctata_631 | 0.201 |
| Harmonia_quadripunctata_380 | Vibidia_duodecimguttata_599 | 0.201 |
| Harmonia_quadripunctata_380 | Vibidia_duodecimguttata_601 | 0.201 |
| Harmonia_quadripunctata_380 | Vibidia_duodecimguttata_602 | 0.201 |
| Oenopia_conglobata_523 | Scymnus_suturalis_838 | 0.201 |
| Scymnus_ferrugatus_729 | Subcoccinella_vigintiquatuorpunctata_619 | 0.201 |
| Coccidula_scutellata_84 | Subcoccinella_vigintiquatuorpunctata_624 | 0.201 |
| Oenopia_conglobata_523 | Scymnus_suturalis_845 | 0.201 |
| Harmonia_quadripunctata_380 | Vibidia_duodecimguttata_597 | 0.201 |
| Propylaea_quatuordecimpunctata_533 | Vibidia_duodecimguttata_597 | 0.201 |
| Propylaea_quatuordecimpunctata_534 | Vibidia_duodecimguttata_597 | 0.201 |
| Propylaea_quatuordecimpunctata_535 | Vibidia_duodecimguttata_597 | 0.201 |
| Propylaea_quatuordecimpunctata_536 | Vibidia_duodecimguttata_597 | 0.201 |
| Propylaea_quatuordecimpunctata_538 | Vibidia_duodecimguttata_597 | 0.201 |
| Propylaea_quatuordecimpunctata_541 | Vibidia_duodecimguttata_597 | 0.201 |
| Propylaea_quatuordecimpunctata_544 | Vibidia_duodecimguttata_597 | 0.201 |
| Propylaea_quatuordecimpunctata_549 | Vibidia_duodecimguttata_597 | 0.201 |
| Harmonia_quadripunctata_380 | Vibidia_duodecimguttata_600 | 0.201 |
| Propylaea_quatuordecimpunctata_533 | Vibidia_duodecimguttata_600 | 0.201 |
| Propylaea_quatuordecimpunctata_534 | Vibidia_duodecimguttata_600 | 0.201 |
| Propylaea_quatuordecimpunctata_535 | Vibidia_duodecimguttata_600 | 0.201 |
| Propylaea_quatuordecimpunctata_536 | Vibidia_duodecimguttata_600 | 0.201 |

| | | |
|---|---|---|
| Propylaea_quatuordecimpunctata_538 | Vibidia_duodecimguttata_600 | 0.201 |
| Propylaea_quatuordecimpunctata_541 | Vibidia_duodecimguttata_600 | 0.201 |
| Propylaea_quatuordecimpunctata_544 | Vibidia_duodecimguttata_600 | 0.201 |
| Propylaea_quatuordecimpunctata_549 | Vibidia_duodecimguttata_600 | 0.201 |
| Oenopia_conglobata_523 | Scymnus_suturalis_834 | 0.201 |
| Scymnus_camptodromus_711 | Subcoccinella_vigintiquatuorpunctata_625 | 0.201 |
| Scymnus_camptodromus_717 | Vibidia_duodecimguttata_596 | 0.201 |
| Scymnus_camptodromus_711 | Vibidia_duodecimguttata_603 | 0.201 |
| Scymnus_camptodromus_717 | Vibidia_duodecimguttata_603 | 0.201 |
| Anisosticta_bitriangularis_149 | Scymnus_sinuanodulus_829 | 0.201 |
| Chilocorus_renipustulatus_33 | Scymnus_sinuanodulus_829 | 0.201 |
| Brachiacantha_ursina_637 | Subcoccinella_vigintiquatuorpunctata_619 | 0.201 |
| Scymnus_ferrugatus_723 | Subcoccinella_vigintiquatuorpunctata_619 | 0.201 |
| Scymnus_ferrugatus_725 | Subcoccinella_vigintiquatuorpunctata_619 | 0.201 |
| Scymnus_ferrugatus_726 | Subcoccinella_vigintiquatuorpunctata_619 | 0.201 |
| Scymnus_ferrugatus_727 | Subcoccinella_vigintiquatuorpunctata_619 | 0.201 |
| Scymnus_ferrugatus_728 | Subcoccinella_vigintiquatuorpunctata_619 | 0.201 |
| Scymnus_ferrugatus_730 | Subcoccinella_vigintiquatuorpunctata_619 | 0.201 |
| Scymnus_ferrugatus_732 | Subcoccinella_vigintiquatuorpunctata_619 | 0.201 |
| Scymnus_ferrugatus_733 | Subcoccinella_vigintiquatuorpunctata_619 | 0.201 |
| Platynaspis_luteorubra_50 | Stethorus_punctillum_849 | 0.201 |
| Platynaspis_luteorubra_50 | Stethorus_punctillum_850 | 0.201 |
| Platynaspis_luteorubra_50 | Stethorus_punctillum_852 | 0.201 |
| Platynaspis_luteorubra_50 | Stethorus_punctillum_854 | 0.201 |
| Platynaspis_luteorubra_50 | Stethorus_punctillum_856 | 0.201 |
| Hyperaspis_lateralis_655 | Tytthaspis_sedecimpunctata_586 | 0.201 |
| Hyperaspis_lateralis_655 | Tytthaspis_sedecimpunctata_591 | 0.201 |
| Hyperaspis_lateralis_654 | Tytthaspis_sedecimpunctata_588 | 0.201 |
| Hyperaspis_lateralis_657 | Tytthaspis_sedecimpunctata_588 | 0.201 |
| Coccidophilus_marginatus_869 | Tytthaspis_sedecimpunctata_577 | 0.201 |
| Coccidophilus_marginatus_869 | Tytthaspis_sedecimpunctata_578 | 0.201 |
| Coccidophilus_marginatus_869 | Tytthaspis_sedecimpunctata_579 | 0.201 |
| Coccidophilus_marginatus_869 | Tytthaspis_sedecimpunctata_581 | 0.201 |
| Coccidophilus_marginatus_869 | Tytthaspis_sedecimpunctata_582 | 0.201 |
| Coccidophilus_marginatus_869 | Tytthaspis_sedecimpunctata_590 | 0.201 |
| Coccidophilus_marginatus_869 | Tytthaspis_sedecimpunctata_594 | 0.201 |
| Coccidophilus_marginatus_869 | Tytthaspis_sedecimpunctata_595 | 0.201 |
| Hyperaspis_lateralis_654 | Tytthaspis_sedecimpunctata_589 | 0.201 |
| Brachiacantha_rotunda_634 | Subcoccinella_vigintiquatuorpunctata_622 | 0.201 |
| Brachiacantha_rotunda_634 | Subcoccinella_vigintiquatuorpunctata_624 | 0.201 |
| Brachiacantha_rotunda_634 | Subcoccinella_vigintiquatuorpunctata_626 | 0.201 |
| Neoharmonia_venusta_521 | Vibidia_duodecimguttata_596 | 0.201 |
| Propylaea_quatuordecimpunctata_542 | Vibidia_duodecimguttata_596 | 0.201 |
| Propylaea_quatuordecimpunctata_545 | Vibidia_duodecimguttata_596 | 0.201 |
| Propylaea_quatuordecimpunctata_546 | Vibidia_duodecimguttata_596 | 0.201 |
| Coccinella_hieroglyphica_227 | Vibidia_duodecimguttata_597 | 0.201 |
| Neoharmonia_venusta_521 | Vibidia_duodecimguttata_598 | 0.201 |
| Propylaea_quatuordecimpunctata_533 | Vibidia_duodecimguttata_598 | 0.201 |
| Propylaea_quatuordecimpunctata_534 | Vibidia_duodecimguttata_598 | 0.201 |
| Propylaea_quatuordecimpunctata_535 | Vibidia_duodecimguttata_598 | 0.201 |
| Propylaea_quatuordecimpunctata_536 | Vibidia_duodecimguttata_598 | 0.201 |
| Propylaea_quatuordecimpunctata_538 | Vibidia_duodecimguttata_598 | 0.201 |
| Propylaea_quatuordecimpunctata_541 | Vibidia_duodecimguttata_598 | 0.201 |
| Propylaea_quatuordecimpunctata_542 | Vibidia_duodecimguttata_598 | 0.201 |
| Propylaea_quatuordecimpunctata_544 | Vibidia_duodecimguttata_598 | 0.201 |
| Propylaea_quatuordecimpunctata_546 | Vibidia_duodecimguttata_598 | 0.201 |

| | | |
|---|---|---|
| Propylaea_quatuordecimpunctata_549 | Vibidia_duodecimguttata_598 | 0.201 |
| Neoharmonia_venusta_521 | Vibidia_duodecimguttata_599 | 0.201 |
| Coccinella_hieroglyphica_227 | Vibidia_duodecimguttata_600 | 0.201 |
| Neoharmonia_venusta_521 | Vibidia_duodecimguttata_602 | 0.201 |
| Propylaea_quatuordecimpunctata_532 | Vibidia_duodecimguttata_603 | 0.201 |
| Propylaea_quatuordecimpunctata_537 | Vibidia_duodecimguttata_603 | 0.201 |
| Coccidophilus_marginatus_869 | Tytthaspis_sedecimpunctata_591 | 0.201 |
| Chilocorus_bipustulatus_19 | Scymnus_suturalis_838 | 0.201 |
| Chilocorus_bipustulatus_22 | Scymnus_suturalis_838 | 0.201 |
| Chilocorus_bipustulatus_19 | Scymnus_suturalis_840 | 0.201 |
| Chilocorus_bipustulatus_22 | Scymnus_suturalis_840 | 0.201 |
| Psyllobora_vigintiduopunctata_568 | Subcoccinella_vigintiquatuorpunctata_619 | 0.201 |
| Chilocorus_bipustulatus_19 | Scymnus_suturalis_834 | 0.201 |
| Chilocorus_bipustulatus_22 | Scymnus_suturalis_834 | 0.201 |
| Scymnus_mimulus_776 | Tytthaspis_sedecimpunctata_584 | 0.201 |
| Scymnus_interruptus_767 | Stethorus_punctillum_850 | 0.201 |
| Scymnus_interruptus_767 | Stethorus_punctillum_854 | 0.201 |
| Scymnus_interruptus_767 | Stethorus_punctillum_856 | 0.201 |
| Scymnus_interruptus_767 | Stethorus_punctillum_860 | 0.201 |
| Scymnus_mimulus_776 | Tytthaspis_sedecimpunctata_577 | 0.201 |
| Scymnus_mimulus_776 | Tytthaspis_sedecimpunctata_578 | 0.201 |
| Scymnus_mimulus_776 | Tytthaspis_sedecimpunctata_579 | 0.201 |
| Scymnus_mimulus_776 | Tytthaspis_sedecimpunctata_581 | 0.201 |
| Scymnus_mimulus_776 | Tytthaspis_sedecimpunctata_582 | 0.201 |
| Scymnus_mimulus_776 | Tytthaspis_sedecimpunctata_585 | 0.201 |
| Scymnus_mimulus_776 | Tytthaspis_sedecimpunctata_590 | 0.201 |
| Scymnus_mimulus_776 | Tytthaspis_sedecimpunctata_592 | 0.201 |
| Scymnus_mimulus_776 | Tytthaspis_sedecimpunctata_594 | 0.201 |
| Scymnus_mimulus_776 | Tytthaspis_sedecimpunctata_595 | 0.201 |
| Coccinella_hieroglyphica_225 | Vibidia_duodecimguttata_596 | 0.201 |
| Coccinella_quinquepunctata_232 | Vibidia_duodecimguttata_597 | 0.201 |
| Coccinella_hieroglyphica_227 | Vibidia_duodecimguttata_598 | 0.201 |
| Coccinella_hieroglyphica_227 | Vibidia_duodecimguttata_599 | 0.201 |
| Coccinella_quinquepunctata_232 | Vibidia_duodecimguttata_600 | 0.201 |
| Coccinella_hieroglyphica_227 | Vibidia_duodecimguttata_601 | 0.201 |
| Coccinella_hieroglyphica_227 | Vibidia_duodecimguttata_602 | 0.201 |
| Platynaspis_luteorubra_51 | Stethorus_punctillum_851 | 0.201 |
| Platynaspis_luteorubra_53 | Stethorus_punctillum_851 | 0.201 |
| Platynaspis_luteorubra_58 | Stethorus_punctillum_851 | 0.201 |
| Platynaspis_luteorubra_51 | Stethorus_punctillum_858 | 0.201 |
| Platynaspis_luteorubra_53 | Stethorus_punctillum_858 | 0.201 |
| Platynaspis_luteorubra_58 | Stethorus_punctillum_858 | 0.201 |
| Scymnus_mimulus_776 | Tytthaspis_sedecimpunctata_580 | 0.201 |
| Scymnus_mimulus_778 | Tytthaspis_sedecimpunctata_583 | 0.201 |
| Scymnus_mimulus_775 | Tytthaspis_sedecimpunctata_586 | 0.201 |
| Scymnus_mimulus_777 | Tytthaspis_sedecimpunctata_586 | 0.201 |
| Scymnus_abietis_689 | Subcoccinella_vigintiquatuorpunctata_620 | 0.201 |
| Scymnus_abietis_689 | Subcoccinella_vigintiquatuorpunctata_621 | 0.201 |
| Scymnus_abietis_689 | Subcoccinella_vigintiquatuorpunctata_627 | 0.201 |
| Scymnus_abietis_689 | Subcoccinella_vigintiquatuorpunctata_628 | 0.201 |
| Scymnus_abietis_689 | Subcoccinella_vigintiquatuorpunctata_629 | 0.201 |
| Scymnus_abietis_689 | Subcoccinella_vigintiquatuorpunctata_630 | 0.201 |
| Scymnus_abietis_689 | Subcoccinella_vigintiquatuorpunctata_631 | 0.201 |
| Scymnus_nubilus_797 | Scymnus_sinuanodulus_831 | 0.201 |
| Chilocorus_nigritus_27 | Stethorus_punctillum_849 | 0.201 |
| Brachiacantha_rotunda_634 | Stethorus_punctillum_850 | 0.201 |

| | | |
|---|---|---|
| Brachiacantha_rotunda_634 | Stethorus_punctillum_854 | 0.201 |
| Brachiacantha_rotunda_634 | Stethorus_punctillum_856 | 0.201 |
| Chilocorus_nigritus_27 | Stethorus_punctillum_860 | 0.201 |
| Scymnus_mimulus_778 | Tytthaspis_sedecimpunctata_587 | 0.201 |
| Cynegetis_impunctata_605 | Scymnus_suturalis_833 | 0.201 |
| Cynegetis_impunctata_606 | Scymnus_suturalis_833 | 0.201 |
| Cynegetis_impunctata_607 | Scymnus_suturalis_833 | 0.201 |
| Cynegetis_impunctata_613 | Scymnus_suturalis_833 | 0.201 |
| Scymnus_camptodromus_706 | Subcoccinella_vigintiquatuorpunctata_627 | 0.201 |
| Scymnus_camptodromus_706 | Subcoccinella_vigintiquatuorpunctata_630 | 0.201 |
| Chilocorus_renipustulatus_33 | Scymnus_suturalis_843 | 0.201 |
| Cynegetis_impunctata_604 | Scymnus_suturalis_843 | 0.201 |
| Cynegetis_impunctata_605 | Scymnus_suturalis_843 | 0.201 |
| Cynegetis_impunctata_606 | Scymnus_suturalis_843 | 0.201 |
| Cynegetis_impunctata_607 | Scymnus_suturalis_843 | 0.201 |
| Cynegetis_impunctata_608 | Scymnus_suturalis_843 | 0.201 |
| Cynegetis_impunctata_609 | Scymnus_suturalis_843 | 0.201 |
| Cynegetis_impunctata_610 | Scymnus_suturalis_843 | 0.201 |
| Cynegetis_impunctata_611 | Scymnus_suturalis_843 | 0.201 |
| Cynegetis_impunctata_612 | Scymnus_suturalis_843 | 0.201 |
| Cynegetis_impunctata_613 | Scymnus_suturalis_843 | 0.201 |
| Chilocorus_renipustulatus_33 | Scymnus_suturalis_845 | 0.201 |
| Cynegetis_impunctata_605 | Scymnus_suturalis_845 | 0.201 |
| Cynegetis_impunctata_606 | Scymnus_suturalis_845 | 0.201 |
| Cynegetis_impunctata_607 | Scymnus_suturalis_845 | 0.201 |
| Cynegetis_impunctata_613 | Scymnus_suturalis_845 | 0.201 |
| Scymnus_loewii_774 | Subcoccinella_vigintiquatuorpunctata_620 | 0.201 |
| Scymnus_loewii_774 | Subcoccinella_vigintiquatuorpunctata_621 | 0.201 |
| Scymnus_loewii_774 | Subcoccinella_vigintiquatuorpunctata_624 | 0.201 |
| Scymnus_loewii_774 | Subcoccinella_vigintiquatuorpunctata_628 | 0.201 |
| Scymnus_loewii_774 | Subcoccinella_vigintiquatuorpunctata_629 | 0.201 |
| Scymnus_loewii_774 | Subcoccinella_vigintiquatuorpunctata_631 | 0.201 |
| Rhyzobius_litura_97 | Tytthaspis_sedecimpunctata_589 | 0.201 |
| Chilocorus_renipustulatus_33 | Scymnus_suturalis_834 | 0.201 |
| Cynegetis_impunctata_604 | Scymnus_suturalis_834 | 0.201 |
| Cynegetis_impunctata_608 | Scymnus_suturalis_834 | 0.201 |
| Cynegetis_impunctata_609 | Scymnus_suturalis_834 | 0.201 |
| Cynegetis_impunctata_610 | Scymnus_suturalis_834 | 0.201 |
| Cynegetis_impunctata_611 | Scymnus_suturalis_834 | 0.201 |
| Cynegetis_impunctata_612 | Scymnus_suturalis_834 | 0.201 |
| Myzia_oblongoguttata_503 | Scymnus_suturalis_840 | 0.201 |
| Coccinella_quinquepunctata_232 | Vibidia_duodecimguttata_598 | 0.201 |
| Coccinella_quinquepunctata_232 | Vibidia_duodecimguttata_599 | 0.201 |
| Coccinella_quinquepunctata_232 | Vibidia_duodecimguttata_601 | 0.201 |
| Coccinella_quinquepunctata_232 | Vibidia_duodecimguttata_602 | 0.201 |
| Chilocorus_nigritus_26 | Stethorus_punctillum_849 | 0.201 |
| Chilocorus_nigritus_26 | Stethorus_punctillum_860 | 0.201 |
| Scymnus_loewii_772 | Subcoccinella_vigintiquatuorpunctata_620 | 0.201 |
| Scymnus_loewii_773 | Subcoccinella_vigintiquatuorpunctata_620 | 0.201 |
| Scymnus_loewii_772 | Subcoccinella_vigintiquatuorpunctata_621 | 0.201 |
| Scymnus_loewii_773 | Subcoccinella_vigintiquatuorpunctata_621 | 0.201 |
| Scymnus_loewii_772 | Subcoccinella_vigintiquatuorpunctata_624 | 0.201 |
| Scymnus_loewii_773 | Subcoccinella_vigintiquatuorpunctata_624 | 0.201 |
| Scymnus_loewii_771 | Subcoccinella_vigintiquatuorpunctata_625 | 0.201 |
| Scymnus_loewii_772 | Subcoccinella_vigintiquatuorpunctata_628 | 0.201 |
| Scymnus_loewii_773 | Subcoccinella_vigintiquatuorpunctata_628 | 0.201 |

| | | |
|---|---|---|
| Scymnus_loewii_772 | Subcoccinella_vigintiquatuorpunctata_629 | 0.201 |
| Scymnus_loewii_773 | Subcoccinella_vigintiquatuorpunctata_629 | 0.201 |
| Scymnus_loewii_772 | Subcoccinella_vigintiquatuorpunctata_631 | 0.201 |
| Scymnus_loewii_773 | Subcoccinella_vigintiquatuorpunctata_631 | 0.201 |
| Rhyzobius_chrysomeloides_85 | Tytthaspis_sedecimpunctata_577 | 0.201 |
| Rhyzobius_chrysomeloides_86 | Tytthaspis_sedecimpunctata_577 | 0.201 |
| Rhyzobius_chrysomeloides_87 | Tytthaspis_sedecimpunctata_577 | 0.201 |
| Rhyzobius_chrysomeloides_88 | Tytthaspis_sedecimpunctata_577 | 0.201 |
| Rhyzobius_chrysomeloides_89 | Tytthaspis_sedecimpunctata_577 | 0.201 |
| Rhyzobius_chrysomeloides_90 | Tytthaspis_sedecimpunctata_577 | 0.201 |
| Rhyzobius_chrysomeloides_91 | Tytthaspis_sedecimpunctata_577 | 0.201 |
| Rhyzobius_chrysomeloides_92 | Tytthaspis_sedecimpunctata_577 | 0.201 |
| Rhyzobius_chrysomeloides_93 | Tytthaspis_sedecimpunctata_577 | 0.201 |
| Rhyzobius_chrysomeloides_94 | Tytthaspis_sedecimpunctata_577 | 0.201 |
| Rhyzobius_chrysomeloides_95 | Tytthaspis_sedecimpunctata_577 | 0.201 |
| Rhyzobius_chrysomeloides_85 | Tytthaspis_sedecimpunctata_578 | 0.201 |
| Rhyzobius_chrysomeloides_86 | Tytthaspis_sedecimpunctata_578 | 0.201 |
| Rhyzobius_chrysomeloides_87 | Tytthaspis_sedecimpunctata_578 | 0.201 |
| Rhyzobius_chrysomeloides_88 | Tytthaspis_sedecimpunctata_578 | 0.201 |
| Rhyzobius_chrysomeloides_89 | Tytthaspis_sedecimpunctata_578 | 0.201 |
| Rhyzobius_chrysomeloides_90 | Tytthaspis_sedecimpunctata_578 | 0.201 |
| Rhyzobius_chrysomeloides_91 | Tytthaspis_sedecimpunctata_578 | 0.201 |
| Rhyzobius_chrysomeloides_92 | Tytthaspis_sedecimpunctata_578 | 0.201 |
| Rhyzobius_chrysomeloides_93 | Tytthaspis_sedecimpunctata_578 | 0.201 |
| Rhyzobius_chrysomeloides_94 | Tytthaspis_sedecimpunctata_578 | 0.201 |
| Rhyzobius_chrysomeloides_95 | Tytthaspis_sedecimpunctata_578 | 0.201 |
| Rhyzobius_chrysomeloides_85 | Tytthaspis_sedecimpunctata_579 | 0.201 |
| Rhyzobius_chrysomeloides_86 | Tytthaspis_sedecimpunctata_579 | 0.201 |
| Rhyzobius_chrysomeloides_87 | Tytthaspis_sedecimpunctata_579 | 0.201 |
| Rhyzobius_chrysomeloides_88 | Tytthaspis_sedecimpunctata_579 | 0.201 |
| Rhyzobius_chrysomeloides_89 | Tytthaspis_sedecimpunctata_579 | 0.201 |
| Rhyzobius_chrysomeloides_90 | Tytthaspis_sedecimpunctata_579 | 0.201 |
| Rhyzobius_chrysomeloides_91 | Tytthaspis_sedecimpunctata_579 | 0.201 |
| Rhyzobius_chrysomeloides_92 | Tytthaspis_sedecimpunctata_579 | 0.201 |
| Rhyzobius_chrysomeloides_93 | Tytthaspis_sedecimpunctata_579 | 0.201 |
| Rhyzobius_chrysomeloides_94 | Tytthaspis_sedecimpunctata_579 | 0.201 |
| Rhyzobius_chrysomeloides_95 | Tytthaspis_sedecimpunctata_579 | 0.201 |
| Rhyzobius_chrysomeloides_85 | Tytthaspis_sedecimpunctata_581 | 0.201 |
| Rhyzobius_chrysomeloides_86 | Tytthaspis_sedecimpunctata_581 | 0.201 |
| Rhyzobius_chrysomeloides_87 | Tytthaspis_sedecimpunctata_581 | 0.201 |
| Rhyzobius_chrysomeloides_88 | Tytthaspis_sedecimpunctata_581 | 0.201 |
| Rhyzobius_chrysomeloides_89 | Tytthaspis_sedecimpunctata_581 | 0.201 |
| Rhyzobius_chrysomeloides_90 | Tytthaspis_sedecimpunctata_581 | 0.201 |
| Rhyzobius_chrysomeloides_91 | Tytthaspis_sedecimpunctata_581 | 0.201 |
| Rhyzobius_chrysomeloides_92 | Tytthaspis_sedecimpunctata_581 | 0.201 |
| Rhyzobius_chrysomeloides_93 | Tytthaspis_sedecimpunctata_581 | 0.201 |
| Rhyzobius_chrysomeloides_94 | Tytthaspis_sedecimpunctata_581 | 0.201 |
| Rhyzobius_chrysomeloides_95 | Tytthaspis_sedecimpunctata_581 | 0.201 |
| Rhyzobius_chrysomeloides_85 | Tytthaspis_sedecimpunctata_582 | 0.201 |
| Rhyzobius_chrysomeloides_86 | Tytthaspis_sedecimpunctata_582 | 0.201 |
| Rhyzobius_chrysomeloides_87 | Tytthaspis_sedecimpunctata_582 | 0.201 |
| Rhyzobius_chrysomeloides_88 | Tytthaspis_sedecimpunctata_582 | 0.201 |
| Rhyzobius_chrysomeloides_89 | Tytthaspis_sedecimpunctata_582 | 0.201 |
| Rhyzobius_chrysomeloides_90 | Tytthaspis_sedecimpunctata_582 | 0.201 |
| Rhyzobius_chrysomeloides_91 | Tytthaspis_sedecimpunctata_582 | 0.201 |
| Rhyzobius_chrysomeloides_92 | Tytthaspis_sedecimpunctata_582 | 0.201 |

| | | |
|---|---|---|
| Rhyzobius_chrysomeloides_93 | Tytthaspis_sedecimpunctata_582 | 0.201 |
| Rhyzobius_chrysomeloides_94 | Tytthaspis_sedecimpunctata_582 | 0.201 |
| Rhyzobius_chrysomeloides_95 | Tytthaspis_sedecimpunctata_582 | 0.201 |
| Rhyzobius_chrysomeloides_85 | Tytthaspis_sedecimpunctata_585 | 0.201 |
| Rhyzobius_chrysomeloides_86 | Tytthaspis_sedecimpunctata_585 | 0.201 |
| Rhyzobius_chrysomeloides_87 | Tytthaspis_sedecimpunctata_585 | 0.201 |
| Rhyzobius_chrysomeloides_88 | Tytthaspis_sedecimpunctata_585 | 0.201 |
| Rhyzobius_chrysomeloides_89 | Tytthaspis_sedecimpunctata_585 | 0.201 |
| Rhyzobius_chrysomeloides_90 | Tytthaspis_sedecimpunctata_585 | 0.201 |
| Rhyzobius_chrysomeloides_91 | Tytthaspis_sedecimpunctata_585 | 0.201 |
| Rhyzobius_chrysomeloides_92 | Tytthaspis_sedecimpunctata_585 | 0.201 |
| Rhyzobius_chrysomeloides_93 | Tytthaspis_sedecimpunctata_585 | 0.201 |
| Rhyzobius_chrysomeloides_94 | Tytthaspis_sedecimpunctata_585 | 0.201 |
| Rhyzobius_chrysomeloides_95 | Tytthaspis_sedecimpunctata_585 | 0.201 |
| Coccinella_transversalis_258 | Tytthaspis_sedecimpunctata_586 | 0.201 |
| Rhyzobius_litura_97 | Tytthaspis_sedecimpunctata_588 | 0.201 |
| Rhyzobius_chrysomeloides_85 | Tytthaspis_sedecimpunctata_590 | 0.201 |
| Rhyzobius_chrysomeloides_86 | Tytthaspis_sedecimpunctata_590 | 0.201 |
| Rhyzobius_chrysomeloides_87 | Tytthaspis_sedecimpunctata_590 | 0.201 |
| Rhyzobius_chrysomeloides_88 | Tytthaspis_sedecimpunctata_590 | 0.201 |
| Rhyzobius_chrysomeloides_89 | Tytthaspis_sedecimpunctata_590 | 0.201 |
| Rhyzobius_chrysomeloides_90 | Tytthaspis_sedecimpunctata_590 | 0.201 |
| Rhyzobius_chrysomeloides_91 | Tytthaspis_sedecimpunctata_590 | 0.201 |
| Rhyzobius_chrysomeloides_92 | Tytthaspis_sedecimpunctata_590 | 0.201 |
| Rhyzobius_chrysomeloides_93 | Tytthaspis_sedecimpunctata_590 | 0.201 |
| Rhyzobius_chrysomeloides_94 | Tytthaspis_sedecimpunctata_590 | 0.201 |
| Rhyzobius_chrysomeloides_95 | Tytthaspis_sedecimpunctata_590 | 0.201 |
| Rhyzobius_chrysomeloides_85 | Tytthaspis_sedecimpunctata_592 | 0.201 |
| Rhyzobius_chrysomeloides_86 | Tytthaspis_sedecimpunctata_592 | 0.201 |
| Rhyzobius_chrysomeloides_87 | Tytthaspis_sedecimpunctata_592 | 0.201 |
| Rhyzobius_chrysomeloides_88 | Tytthaspis_sedecimpunctata_592 | 0.201 |
| Rhyzobius_chrysomeloides_89 | Tytthaspis_sedecimpunctata_592 | 0.201 |
| Rhyzobius_chrysomeloides_90 | Tytthaspis_sedecimpunctata_592 | 0.201 |
| Rhyzobius_chrysomeloides_91 | Tytthaspis_sedecimpunctata_592 | 0.201 |
| Rhyzobius_chrysomeloides_92 | Tytthaspis_sedecimpunctata_592 | 0.201 |
| Rhyzobius_chrysomeloides_93 | Tytthaspis_sedecimpunctata_592 | 0.201 |
| Rhyzobius_chrysomeloides_94 | Tytthaspis_sedecimpunctata_592 | 0.201 |
| Rhyzobius_chrysomeloides_95 | Tytthaspis_sedecimpunctata_592 | 0.201 |
| Rhyzobius_chrysomeloides_85 | Tytthaspis_sedecimpunctata_593 | 0.201 |
| Rhyzobius_chrysomeloides_86 | Tytthaspis_sedecimpunctata_593 | 0.201 |
| Rhyzobius_chrysomeloides_87 | Tytthaspis_sedecimpunctata_593 | 0.201 |
| Rhyzobius_chrysomeloides_88 | Tytthaspis_sedecimpunctata_593 | 0.201 |
| Rhyzobius_chrysomeloides_89 | Tytthaspis_sedecimpunctata_593 | 0.201 |
| Rhyzobius_chrysomeloides_90 | Tytthaspis_sedecimpunctata_593 | 0.201 |
| Rhyzobius_chrysomeloides_91 | Tytthaspis_sedecimpunctata_593 | 0.201 |
| Rhyzobius_chrysomeloides_92 | Tytthaspis_sedecimpunctata_593 | 0.201 |
| Rhyzobius_chrysomeloides_93 | Tytthaspis_sedecimpunctata_593 | 0.201 |
| Rhyzobius_chrysomeloides_94 | Tytthaspis_sedecimpunctata_593 | 0.201 |
| Rhyzobius_chrysomeloides_95 | Tytthaspis_sedecimpunctata_593 | 0.201 |
| Rhyzobius_chrysomeloides_85 | Tytthaspis_sedecimpunctata_594 | 0.201 |
| Rhyzobius_chrysomeloides_86 | Tytthaspis_sedecimpunctata_594 | 0.201 |
| Rhyzobius_chrysomeloides_87 | Tytthaspis_sedecimpunctata_594 | 0.201 |
| Rhyzobius_chrysomeloides_88 | Tytthaspis_sedecimpunctata_594 | 0.201 |
| Rhyzobius_chrysomeloides_89 | Tytthaspis_sedecimpunctata_594 | 0.201 |
| Rhyzobius_chrysomeloides_90 | Tytthaspis_sedecimpunctata_594 | 0.201 |
| Rhyzobius_chrysomeloides_91 | Tytthaspis_sedecimpunctata_594 | 0.201 |

| | | |
|---|---|---|
| Rhyzobius_chrysomeloides_92 | Tytthaspis_sedecimpunctata_594 | 0.201 |
| Rhyzobius_chrysomeloides_93 | Tytthaspis_sedecimpunctata_594 | 0.201 |
| Rhyzobius_chrysomeloides_94 | Tytthaspis_sedecimpunctata_594 | 0.201 |
| Rhyzobius_chrysomeloides_95 | Tytthaspis_sedecimpunctata_594 | 0.201 |
| Rhyzobius_chrysomeloides_85 | Tytthaspis_sedecimpunctata_595 | 0.201 |
| Rhyzobius_chrysomeloides_86 | Tytthaspis_sedecimpunctata_595 | 0.201 |
| Rhyzobius_chrysomeloides_87 | Tytthaspis_sedecimpunctata_595 | 0.201 |
| Rhyzobius_chrysomeloides_88 | Tytthaspis_sedecimpunctata_595 | 0.201 |
| Rhyzobius_chrysomeloides_89 | Tytthaspis_sedecimpunctata_595 | 0.201 |
| Rhyzobius_chrysomeloides_90 | Tytthaspis_sedecimpunctata_595 | 0.201 |
| Rhyzobius_chrysomeloides_91 | Tytthaspis_sedecimpunctata_595 | 0.201 |
| Rhyzobius_chrysomeloides_92 | Tytthaspis_sedecimpunctata_595 | 0.201 |
| Rhyzobius_chrysomeloides_93 | Tytthaspis_sedecimpunctata_595 | 0.201 |
| Rhyzobius_chrysomeloides_94 | Tytthaspis_sedecimpunctata_595 | 0.201 |
| Rhyzobius_chrysomeloides_95 | Tytthaspis_sedecimpunctata_595 | 0.201 |
| Myrrha_octodecimguttata_489 | Scymnus_sinuanodulus_828 | 0.201 |
| Myrrha_octodecimguttata_493 | Scymnus_sinuanodulus_828 | 0.201 |
| Myrrha_octodecimguttata_498 | Scymnus_sinuanodulus_828 | 0.201 |
| Myrrha_octodecimguttata_500 | Scymnus_sinuanodulus_828 | 0.201 |
| Myrrha_octodecimguttata_485 | Scymnus_sinuanodulus_830 | 0.201 |
| Myrrha_octodecimguttata_486 | Scymnus_sinuanodulus_830 | 0.201 |
| Myrrha_octodecimguttata_487 | Scymnus_sinuanodulus_830 | 0.201 |
| Myrrha_octodecimguttata_490 | Scymnus_sinuanodulus_830 | 0.201 |
| Myrrha_octodecimguttata_494 | Scymnus_sinuanodulus_830 | 0.201 |
| Myrrha_octodecimguttata_496 | Scymnus_sinuanodulus_830 | 0.201 |
| Myrrha_octodecimguttata_497 | Scymnus_sinuanodulus_830 | 0.201 |
| Myrrha_octodecimguttata_501 | Scymnus_sinuanodulus_830 | 0.201 |
| Myrrha_octodecimguttata_502 | Scymnus_sinuanodulus_830 | 0.201 |
| Chilocorus_renipustulatus_34 | Scymnus_suturalis_833 | 0.201 |
| Myzia_oblongoguttata_503 | Scymnus_suturalis_834 | 0.201 |
| Cynegetis_impunctata_605 | Scymnus_suturalis_838 | 0.201 |
| Cynegetis_impunctata_606 | Scymnus_suturalis_838 | 0.201 |
| Cynegetis_impunctata_607 | Scymnus_suturalis_838 | 0.201 |
| Cynegetis_impunctata_613 | Scymnus_suturalis_838 | 0.201 |
| Chilocorus_bipustulatus_21 | Scymnus_suturalis_838 | 0.201 |
| Chilocorus_bipustulatus_21 | Scymnus_suturalis_840 | 0.201 |
| Chilocorus_bipustulatus_21 | Scymnus_suturalis_834 | 0.201 |
| Diomus_terminatus_647 | Tytthaspis_sedecimpunctata_584 | 0.201 |
| Diomus_terminatus_648 | Tytthaspis_sedecimpunctata_584 | 0.201 |
| Myrrha_octodecimguttata_488 | Scymnus_sinuanodulus_830 | 0.201 |
| Myrrha_octodecimguttata_491 | Scymnus_sinuanodulus_830 | 0.201 |
| Myrrha_octodecimguttata_492 | Scymnus_sinuanodulus_830 | 0.201 |
| Platynaspis_luteorubra_55 | Scymnus_sinuanodulus_830 | 0.201 |
| Platynaspis_luteorubra_61 | Scymnus_sinuanodulus_830 | 0.201 |
| Adalia_bipunctata_100 | Scymnus_suturalis_833 | 0.201 |
| Chilocorus_renipustulatus_31 | Scymnus_suturalis_833 | 0.201 |
| Chilocorus_renipustulatus_32 | Scymnus_suturalis_833 | 0.201 |
| Chilocorus_renipustulatus_35 | Scymnus_suturalis_833 | 0.201 |
| Chilocorus_renipustulatus_36 | Scymnus_suturalis_833 | 0.201 |
| Chilocorus_renipustulatus_37 | Scymnus_suturalis_833 | 0.201 |
| Chilocorus_renipustulatus_38 | Scymnus_suturalis_833 | 0.201 |
| Chilocorus_renipustulatus_40 | Scymnus_suturalis_833 | 0.201 |
| Chilocorus_renipustulatus_41 | Scymnus_suturalis_833 | 0.201 |
| Chilocorus_renipustulatus_42 | Scymnus_suturalis_833 | 0.201 |
| Scymnus_apetzi_692 | Tytthaspis_sedecimpunctata_577 | 0.201 |
| Scymnus_apetzi_692 | Tytthaspis_sedecimpunctata_578 | 0.201 |

| | | |
|---|---|---|
| Scymnus_apetzi_692 | Tytthaspis_sedecimpunctata_579 | 0.201 |
| Scymnus_apetzi_692 | Tytthaspis_sedecimpunctata_581 | 0.201 |
| Scymnus_apetzi_692 | Tytthaspis_sedecimpunctata_582 | 0.201 |
| Scymnus_apetzi_692 | Tytthaspis_sedecimpunctata_590 | 0.201 |
| Scymnus_apetzi_692 | Tytthaspis_sedecimpunctata_594 | 0.201 |
| Scymnus_apetzi_692 | Tytthaspis_sedecimpunctata_595 | 0.201 |
| Scymnus_apetzi_692 | Tytthaspis_sedecimpunctata_584 | 0.201 |
| Adalia_decempunctata_118 | Tytthaspis_sedecimpunctata_577 | 0.201 |
| Adalia_decempunctata_119 | Tytthaspis_sedecimpunctata_577 | 0.201 |
| Adalia_decempunctata_120 | Tytthaspis_sedecimpunctata_577 | 0.201 |
| Adalia_decempunctata_121 | Tytthaspis_sedecimpunctata_577 | 0.201 |
| Harmonia_quadripunctata_377 | Tytthaspis_sedecimpunctata_577 | 0.201 |
| Harmonia_quadripunctata_379 | Tytthaspis_sedecimpunctata_577 | 0.201 |
| Harmonia_quadripunctata_380 | Tytthaspis_sedecimpunctata_577 | 0.201 |
| Harmonia_quadripunctata_381 | Tytthaspis_sedecimpunctata_577 | 0.201 |
| Adalia_decempunctata_118 | Tytthaspis_sedecimpunctata_578 | 0.201 |
| Adalia_decempunctata_119 | Tytthaspis_sedecimpunctata_578 | 0.201 |
| Adalia_decempunctata_120 | Tytthaspis_sedecimpunctata_578 | 0.201 |
| Adalia_decempunctata_121 | Tytthaspis_sedecimpunctata_578 | 0.201 |
| Harmonia_quadripunctata_377 | Tytthaspis_sedecimpunctata_578 | 0.201 |
| Harmonia_quadripunctata_379 | Tytthaspis_sedecimpunctata_578 | 0.201 |
| Harmonia_quadripunctata_380 | Tytthaspis_sedecimpunctata_578 | 0.201 |
| Harmonia_quadripunctata_381 | Tytthaspis_sedecimpunctata_578 | 0.201 |
| Adalia_decempunctata_118 | Tytthaspis_sedecimpunctata_579 | 0.201 |
| Adalia_decempunctata_119 | Tytthaspis_sedecimpunctata_579 | 0.201 |
| Adalia_decempunctata_120 | Tytthaspis_sedecimpunctata_579 | 0.201 |
| Adalia_decempunctata_121 | Tytthaspis_sedecimpunctata_579 | 0.201 |
| Harmonia_quadripunctata_377 | Tytthaspis_sedecimpunctata_579 | 0.201 |
| Harmonia_quadripunctata_379 | Tytthaspis_sedecimpunctata_579 | 0.201 |
| Harmonia_quadripunctata_380 | Tytthaspis_sedecimpunctata_579 | 0.201 |
| Harmonia_quadripunctata_381 | Tytthaspis_sedecimpunctata_579 | 0.201 |
| Adalia_decempunctata_118 | Tytthaspis_sedecimpunctata_581 | 0.201 |
| Adalia_decempunctata_119 | Tytthaspis_sedecimpunctata_581 | 0.201 |
| Adalia_decempunctata_120 | Tytthaspis_sedecimpunctata_581 | 0.201 |
| Adalia_decempunctata_121 | Tytthaspis_sedecimpunctata_581 | 0.201 |
| Harmonia_quadripunctata_377 | Tytthaspis_sedecimpunctata_581 | 0.201 |
| Harmonia_quadripunctata_379 | Tytthaspis_sedecimpunctata_581 | 0.201 |
| Harmonia_quadripunctata_380 | Tytthaspis_sedecimpunctata_581 | 0.201 |
| Harmonia_quadripunctata_381 | Tytthaspis_sedecimpunctata_581 | 0.201 |
| Adalia_decempunctata_118 | Tytthaspis_sedecimpunctata_582 | 0.201 |
| Adalia_decempunctata_119 | Tytthaspis_sedecimpunctata_582 | 0.201 |
| Adalia_decempunctata_120 | Tytthaspis_sedecimpunctata_582 | 0.201 |
| Adalia_decempunctata_121 | Tytthaspis_sedecimpunctata_582 | 0.201 |
| Harmonia_quadripunctata_377 | Tytthaspis_sedecimpunctata_582 | 0.201 |
| Harmonia_quadripunctata_379 | Tytthaspis_sedecimpunctata_582 | 0.201 |
| Harmonia_quadripunctata_380 | Tytthaspis_sedecimpunctata_582 | 0.201 |
| Harmonia_quadripunctata_381 | Tytthaspis_sedecimpunctata_582 | 0.201 |
| Brachiacantha_ursina_638 | Tytthaspis_sedecimpunctata_584 | 0.201 |
| Cynegetis_impunctata_614 | Tytthaspis_sedecimpunctata_584 | 0.201 |
| Diomus_terminatus_647 | Tytthaspis_sedecimpunctata_589 | 0.201 |
| Diomus_terminatus_648 | Tytthaspis_sedecimpunctata_589 | 0.201 |
| Adalia_decempunctata_118 | Tytthaspis_sedecimpunctata_590 | 0.201 |
| Adalia_decempunctata_119 | Tytthaspis_sedecimpunctata_590 | 0.201 |
| Adalia_decempunctata_120 | Tytthaspis_sedecimpunctata_590 | 0.201 |
| Adalia_decempunctata_121 | Tytthaspis_sedecimpunctata_590 | 0.201 |
| Harmonia_quadripunctata_377 | Tytthaspis_sedecimpunctata_590 | 0.201 |

Supplementary Table S4 (continued)

| | | |
|---|---|---|
| Harmonia_quadripunctata_379 | Tytthaspis_sedecimpunctata_590 | 0.201 |
| Harmonia_quadripunctata_380 | Tytthaspis_sedecimpunctata_590 | 0.201 |
| Harmonia_quadripunctata_381 | Tytthaspis_sedecimpunctata_590 | 0.201 |
| Brachiacantha_ursina_637 | Tytthaspis_sedecimpunctata_591 | 0.201 |
| Scymnus_nebulosus_781 | Tytthaspis_sedecimpunctata_591 | 0.201 |
| Harmonia_quadripunctata_377 | Tytthaspis_sedecimpunctata_592 | 0.201 |
| Harmonia_quadripunctata_379 | Tytthaspis_sedecimpunctata_592 | 0.201 |
| Harmonia_quadripunctata_380 | Tytthaspis_sedecimpunctata_592 | 0.201 |
| Harmonia_quadripunctata_381 | Tytthaspis_sedecimpunctata_592 | 0.201 |
| Harmonia_quadripunctata_377 | Tytthaspis_sedecimpunctata_593 | 0.201 |
| Harmonia_quadripunctata_379 | Tytthaspis_sedecimpunctata_593 | 0.201 |
| Harmonia_quadripunctata_381 | Tytthaspis_sedecimpunctata_593 | 0.201 |
| Adalia_decempunctata_118 | Tytthaspis_sedecimpunctata_594 | 0.201 |
| Adalia_decempunctata_119 | Tytthaspis_sedecimpunctata_594 | 0.201 |
| Adalia_decempunctata_120 | Tytthaspis_sedecimpunctata_594 | 0.201 |
| Adalia_decempunctata_121 | Tytthaspis_sedecimpunctata_594 | 0.201 |
| Harmonia_quadripunctata_377 | Tytthaspis_sedecimpunctata_594 | 0.201 |
| Harmonia_quadripunctata_379 | Tytthaspis_sedecimpunctata_594 | 0.201 |
| Harmonia_quadripunctata_380 | Tytthaspis_sedecimpunctata_594 | 0.201 |
| Harmonia_quadripunctata_381 | Tytthaspis_sedecimpunctata_594 | 0.201 |
| Adalia_decempunctata_118 | Tytthaspis_sedecimpunctata_595 | 0.201 |
| Adalia_decempunctata_119 | Tytthaspis_sedecimpunctata_595 | 0.201 |
| Adalia_decempunctata_120 | Tytthaspis_sedecimpunctata_595 | 0.201 |
| Adalia_decempunctata_121 | Tytthaspis_sedecimpunctata_595 | 0.201 |
| Harmonia_quadripunctata_377 | Tytthaspis_sedecimpunctata_595 | 0.201 |
| Harmonia_quadripunctata_379 | Tytthaspis_sedecimpunctata_595 | 0.201 |
| Harmonia_quadripunctata_380 | Tytthaspis_sedecimpunctata_595 | 0.201 |
| Harmonia_quadripunctata_381 | Tytthaspis_sedecimpunctata_595 | 0.201 |
| Nephus_redtenbacheri_679 | Scymnus_rubromaculatus_822 | 0.201 |
| Nephus_redtenbacheri_679 | Scymnus_rubromaculatus_823 | 0.201 |
| Nephus_redtenbacheri_679 | Scymnus_rubromaculatus_824 | 0.201 |
| Nephus_redtenbacheri_679 | Scymnus_rubromaculatus_825 | 0.201 |
| Nephus_redtenbacheri_679 | Scymnus_rubromaculatus_826 | 0.201 |
| Nephus_redtenbacheri_679 | Scymnus_rubromaculatus_827 | 0.201 |
| Platynaspis_luteorubra_55 | Scymnus_sinuanodulus_828 | 0.201 |
| Platynaspis_luteorubra_61 | Scymnus_sinuanodulus_828 | 0.201 |
| Adalia_bipunctata_100 | Scymnus_suturalis_837 | 0.201 |
| Chilocorus_nigritus_30 | Stethorus_punctillum_849 | 0.202 |
| Chilocorus_nigritus_29 | Stethorus_punctillum_852 | 0.202 |
| Chilocorus_nigritus_30 | Stethorus_punctillum_860 | 0.202 |
| Calvia_decemguttata_195 | Subcoccinella_vigintiquatuorpunctata_622 | 0.202 |
| Calvia_decemguttata_195 | Subcoccinella_vigintiquatuorpunctata_624 | 0.202 |
| Calvia_decemguttata_199 | Subcoccinella_vigintiquatuorpunctata_627 | 0.202 |
| Calvia_decemguttata_200 | Subcoccinella_vigintiquatuorpunctata_627 | 0.202 |
| Calvia_decemguttata_195 | Subcoccinella_vigintiquatuorpunctata_630 | 0.202 |
| Adalia_decempunctata_118 | Tytthaspis_sedecimpunctata_580 | 0.202 |
| Adalia_decempunctata_119 | Tytthaspis_sedecimpunctata_580 | 0.202 |
| Adalia_decempunctata_120 | Tytthaspis_sedecimpunctata_580 | 0.202 |
| Adalia_decempunctata_121 | Tytthaspis_sedecimpunctata_580 | 0.202 |
| Harmonia_quadripunctata_377 | Tytthaspis_sedecimpunctata_580 | 0.202 |
| Harmonia_quadripunctata_379 | Tytthaspis_sedecimpunctata_580 | 0.202 |
| Harmonia_quadripunctata_380 | Tytthaspis_sedecimpunctata_580 | 0.202 |
| Harmonia_quadripunctata_381 | Tytthaspis_sedecimpunctata_580 | 0.202 |
| Diomus_terminatus_647 | Tytthaspis_sedecimpunctata_587 | 0.202 |
| Diomus_terminatus_648 | Tytthaspis_sedecimpunctata_587 | 0.202 |
| Scymnus_nebulosus_783 | Tytthaspis_sedecimpunctata_589 | 0.202 |

| | | |
|---|---|---|
| Adalia_decempunctata_118 | Tytthaspis_sedecimpunctata_591 | 0.202 |
| Adalia_decempunctata_119 | Tytthaspis_sedecimpunctata_591 | 0.202 |
| Adalia_decempunctata_120 | Tytthaspis_sedecimpunctata_591 | 0.202 |
| Adalia_decempunctata_121 | Tytthaspis_sedecimpunctata_591 | 0.202 |
| Adalia_bipunctata_105 | Scymnus_rubromaculatus_823 | 0.202 |
| Adalia_bipunctata_105 | Scymnus_rubromaculatus_824 | 0.202 |
| Adalia_bipunctata_105 | Scymnus_rubromaculatus_825 | 0.202 |
| Adalia_bipunctata_105 | Scymnus_rubromaculatus_826 | 0.202 |
| Adalia_bipunctata_105 | Scymnus_rubromaculatus_827 | 0.202 |
| Chilocorus_nigritus_28 | Stethorus_punctillum_849 | 0.202 |
| Chilocorus_nigritus_28 | Stethorus_punctillum_860 | 0.202 |
| Scymnus_ningshanensis_789 | Subcoccinella_vigintiquatuorpunctata_624 | 0.202 |
| Scymnus_ningshanensis_790 | Subcoccinella_vigintiquatuorpunctata_624 | 0.202 |
| Scymnus_ningshanensis_788 | Subcoccinella_vigintiquatuorpunctata_625 | 0.202 |
| Scymnus_ningshanensis_791 | Subcoccinella_vigintiquatuorpunctata_625 | 0.202 |
| Scymnus_ningshanensis_792 | Subcoccinella_vigintiquatuorpunctata_625 | 0.202 |
| Scymnus_ningshanensis_793 | Subcoccinella_vigintiquatuorpunctata_625 | 0.202 |
| Scymnus_ningshanensis_794 | Subcoccinella_vigintiquatuorpunctata_625 | 0.202 |
| Scymnus_ningshanensis_795 | Subcoccinella_vigintiquatuorpunctata_625 | 0.202 |
| Scymnus_nebulosus_783 | Tytthaspis_sedecimpunctata_587 | 0.202 |
| Scymnus_interruptus_762 | Tytthaspis_sedecimpunctata_591 | 0.202 |
| Scymnus_nigrinus_784 | Tytthaspis_sedecimpunctata_591 | 0.202 |
| Nephus_redtenbacheri_674 | Scymnus_rubromaculatus_822 | 0.202 |
| Adalia_bipunctata_101 | Scymnus_rubromaculatus_823 | 0.202 |
| Adalia_bipunctata_104 | Scymnus_rubromaculatus_823 | 0.202 |
| Nephus_redtenbacheri_674 | Scymnus_rubromaculatus_823 | 0.202 |
| Adalia_bipunctata_101 | Scymnus_rubromaculatus_824 | 0.202 |
| Adalia_bipunctata_104 | Scymnus_rubromaculatus_824 | 0.202 |
| Nephus_redtenbacheri_674 | Scymnus_rubromaculatus_824 | 0.202 |
| Adalia_bipunctata_101 | Scymnus_rubromaculatus_825 | 0.202 |
| Adalia_bipunctata_104 | Scymnus_rubromaculatus_825 | 0.202 |
| Nephus_redtenbacheri_674 | Scymnus_rubromaculatus_825 | 0.202 |
| Adalia_bipunctata_101 | Scymnus_rubromaculatus_826 | 0.202 |
| Adalia_bipunctata_104 | Scymnus_rubromaculatus_826 | 0.202 |
| Nephus_redtenbacheri_674 | Scymnus_rubromaculatus_826 | 0.202 |
| Adalia_bipunctata_101 | Scymnus_rubromaculatus_827 | 0.202 |
| Adalia_bipunctata_104 | Scymnus_rubromaculatus_827 | 0.202 |
| Nephus_redtenbacheri_674 | Scymnus_rubromaculatus_827 | 0.202 |
| Myzia_pullata_519 | Scymnus_suturalis_838 | 0.202 |
| Myzia_pullata_519 | Scymnus_suturalis_840 | 0.202 |
| Coccinella_quinquepunctata_233 | Vibidia_duodecimguttata_603 | 0.202 |
| Coccinella_quinquepunctata_234 | Vibidia_duodecimguttata_603 | 0.202 |
| Scymnus_pallipediformis_811 | Subcoccinella_vigintiquatuorpunctata_624 | 0.202 |
| Scymnus_apetzi_690 | Subcoccinella_vigintiquatuorpunctata_625 | 0.202 |
| Scymnus_apetzi_697 | Subcoccinella_vigintiquatuorpunctata_625 | 0.202 |
| Scymnus_pallipediformis_811 | Subcoccinella_vigintiquatuorpunctata_625 | 0.202 |
| Didion_punctatum_644 | Subcoccinella_vigintiquatuorpunctata_630 | 0.202 |
| Scymnus_interruptus_759 | Tytthaspis_sedecimpunctata_591 | 0.202 |
| Scymnus_interruptus_769 | Tytthaspis_sedecimpunctata_591 | 0.202 |
| Anisosticta_bitriangularis_148 | Scymnus_sinuanodulus_830 | 0.202 |
| Myzia_oblongoguttata_506 | Scymnus_sinuanodulus_831 | 0.202 |
| Scymnus_nubilus_797 | Scymnus_sinuanodulus_829 | 0.202 |
| Cynegetis_impunctata_604 | Subcoccinella_vigintiquatuorpunctata_624 | 0.202 |
| Cynegetis_impunctata_605 | Subcoccinella_vigintiquatuorpunctata_624 | 0.202 |
| Cynegetis_impunctata_606 | Subcoccinella_vigintiquatuorpunctata_624 | 0.202 |
| Cynegetis_impunctata_607 | Subcoccinella_vigintiquatuorpunctata_624 | 0.202 |

| | | |
|---|---|---|
| Cynegetis_impunctata_608 | Subcoccinella_vigintiquatuorpunctata_624 | 0.202 |
| Cynegetis_impunctata_609 | Subcoccinella_vigintiquatuorpunctata_624 | 0.202 |
| Cynegetis_impunctata_610 | Subcoccinella_vigintiquatuorpunctata_624 | 0.202 |
| Cynegetis_impunctata_611 | Subcoccinella_vigintiquatuorpunctata_624 | 0.202 |
| Cynegetis_impunctata_612 | Subcoccinella_vigintiquatuorpunctata_624 | 0.202 |
| Cynegetis_impunctata_613 | Subcoccinella_vigintiquatuorpunctata_624 | 0.202 |
| Brachiacantha_rotunda_632 | Scymnus_suturalis_843 | 0.202 |
| Brachiacantha_rotunda_633 | Scymnus_suturalis_843 | 0.202 |
| Brachiacantha_rotunda_635 | Scymnus_suturalis_843 | 0.202 |
| Brachiacantha_rotunda_632 | Scymnus_suturalis_845 | 0.202 |
| Brachiacantha_rotunda_633 | Scymnus_suturalis_845 | 0.202 |
| Brachiacantha_rotunda_635 | Scymnus_suturalis_845 | 0.202 |
| Hyperaspis_campestris_651 | Stethorus_punctillum_855 | 0.202 |
| Hyperaspis_campestris_652 | Stethorus_punctillum_855 | 0.202 |
| Hyperaspis_campestris_653 | Stethorus_punctillum_855 | 0.202 |
| Hyperaspis_reppensis_664 | Stethorus_punctillum_857 | 0.202 |
| Hyperaspis_reppensis_666 | Stethorus_punctillum_857 | 0.202 |
| Psyllobora_vigintiduopunctata_570 | Subcoccinella_vigintiquatuorpunctata_623 | 0.202 |
| Psyllobora_vigintiduopunctata_572 | Subcoccinella_vigintiquatuorpunctata_623 | 0.202 |
| Scymnus_ferrugatus_723 | Tytthaspis_sedecimpunctata_591 | 0.202 |
| Scymnus_ferrugatus_725 | Tytthaspis_sedecimpunctata_591 | 0.202 |
| Scymnus_ferrugatus_726 | Tytthaspis_sedecimpunctata_591 | 0.202 |
| Scymnus_ferrugatus_727 | Tytthaspis_sedecimpunctata_591 | 0.202 |
| Scymnus_ferrugatus_728 | Tytthaspis_sedecimpunctata_591 | 0.202 |
| Scymnus_ferrugatus_730 | Tytthaspis_sedecimpunctata_591 | 0.202 |
| Scymnus_ferrugatus_731 | Tytthaspis_sedecimpunctata_591 | 0.202 |
| Scymnus_ferrugatus_732 | Tytthaspis_sedecimpunctata_591 | 0.202 |
| Scymnus_ferrugatus_733 | Tytthaspis_sedecimpunctata_591 | 0.202 |
| Hippodamia_convergens_399 | Vibidia_duodecimguttata_596 | 0.202 |
| Hippodamia_convergens_404 | Vibidia_duodecimguttata_596 | 0.202 |
| Hippodamia_convergens_405 | Vibidia_duodecimguttata_596 | 0.202 |
| Hippodamia_convergens_406 | Vibidia_duodecimguttata_596 | 0.202 |
| Hippodamia_quinquesignata_443 | Vibidia_duodecimguttata_596 | 0.202 |
| Hippodamia_quinquesignata_444 | Vibidia_duodecimguttata_596 | 0.202 |
| Hippodamia_quinquesignata_445 | Vibidia_duodecimguttata_596 | 0.202 |
| Calvia_quatuordecimguttata_209 | Scymnus_suturalis_833 | 0.202 |
| Brachiacantha_rotunda_632 | Scymnus_suturalis_834 | 0.202 |
| Brachiacantha_rotunda_633 | Scymnus_suturalis_834 | 0.202 |
| Brachiacantha_rotunda_635 | Scymnus_suturalis_834 | 0.202 |
| Platynaspis_luteorubra_50 | Subcoccinella_vigintiquatuorpunctata_623 | 0.202 |
| Scymnus_camptodromus_715 | Vibidia_duodecimguttata_597 | 0.202 |
| Scymnus_camptodromus_715 | Vibidia_duodecimguttata_600 | 0.202 |
| Calvia_quatuordecimguttata_209 | Scymnus_suturalis_841 | 0.202 |
| Calvia_quatuordecimguttata_209 | Scymnus_suturalis_842 | 0.202 |
| Calvia_quatuordecimguttata_206 | Scymnus_suturalis_843 | 0.202 |
| Calvia_quatuordecimguttata_216 | Scymnus_suturalis_843 | 0.202 |
| Calvia_quatuordecimguttata_209 | Scymnus_suturalis_844 | 0.202 |
| Calvia_quatuordecimguttata_206 | Scymnus_suturalis_845 | 0.202 |
| Calvia_quatuordecimguttata_216 | Scymnus_suturalis_845 | 0.202 |
| Calvia_quatuordecimguttata_209 | Scymnus_suturalis_846 | 0.202 |
| Calvia_quatuordecimguttata_209 | Scymnus_suturalis_847 | 0.202 |
| Calvia_quatuordecimguttata_209 | Scymnus_suturalis_848 | 0.202 |
| Hyperaspis_reppensis_667 | Stethorus_punctillum_851 | 0.202 |
| Hyperaspis_reppensis_667 | Stethorus_punctillum_858 | 0.202 |
| Hyperaspis_reppensis_664 | Stethorus_punctillum_859 | 0.202 |
| Hyperaspis_reppensis_665 | Stethorus_punctillum_859 | 0.202 |

| | | |
|---|---|---|
| Hyperaspis_reppensis_666 | Stethorus_punctillum_859 | 0.202 |
| Chilocorus_stigma_46 | Subcoccinella_vigintiquatuorpunctata_620 | 0.202 |
| Chilocorus_stigma_46 | Subcoccinella_vigintiquatuorpunctata_621 | 0.202 |
| Psyllobora_vigintiduopunctata_570 | Subcoccinella_vigintiquatuorpunctata_622 | 0.202 |
| Psyllobora_vigintiduopunctata_572 | Subcoccinella_vigintiquatuorpunctata_622 | 0.202 |
| Psyllobora_vigintiduopunctata_570 | Subcoccinella_vigintiquatuorpunctata_625 | 0.202 |
| Psyllobora_vigintiduopunctata_572 | Subcoccinella_vigintiquatuorpunctata_625 | 0.202 |
| Psyllobora_vigintiduopunctata_570 | Subcoccinella_vigintiquatuorpunctata_626 | 0.202 |
| Psyllobora_vigintiduopunctata_572 | Subcoccinella_vigintiquatuorpunctata_626 | 0.202 |
| Chilocorus_stigma_46 | Subcoccinella_vigintiquatuorpunctata_627 | 0.202 |
| Chilocorus_stigma_46 | Subcoccinella_vigintiquatuorpunctata_628 | 0.202 |
| Chilocorus_stigma_46 | Subcoccinella_vigintiquatuorpunctata_629 | 0.202 |
| Chilocorus_stigma_46 | Subcoccinella_vigintiquatuorpunctata_630 | 0.202 |
| Chilocorus_stigma_46 | Subcoccinella_vigintiquatuorpunctata_631 | 0.202 |
| Scymnus_interruptus_759 | Tytthaspis_sedecimpunctata_587 | 0.202 |
| Scymnus_interruptus_769 | Tytthaspis_sedecimpunctata_587 | 0.202 |
| Scymnus_ferrugatus_729 | Tytthaspis_sedecimpunctata_591 | 0.202 |
| Calvia_quatuordecimguttata_209 | Scymnus_suturalis_832 | 0.202 |
| Calvia_quatuordecimguttata_206 | Scymnus_suturalis_834 | 0.202 |
| Calvia_quatuordecimguttata_216 | Scymnus_suturalis_834 | 0.202 |
| Calvia_quatuordecimguttata_209 | Scymnus_suturalis_835 | 0.202 |
| Calvia_quatuordecimguttata_209 | Scymnus_suturalis_836 | 0.202 |
| Calvia_quatuordecimguttata_209 | Scymnus_suturalis_839 | 0.202 |
| Platynaspis_luteorubra_50 | Subcoccinella_vigintiquatuorpunctata_622 | 0.202 |
| Platynaspis_luteorubra_50 | Subcoccinella_vigintiquatuorpunctata_626 | 0.202 |
| Scymnus_camptodromus_715 | Vibidia_duodecimguttata_598 | 0.202 |
| Scymnus_camptodromus_715 | Vibidia_duodecimguttata_603 | 0.202 |
| Anisosticta_bitriangularis_155 | Scymnus_sinuanodulus_831 | 0.202 |
| Anisosticta_bitriangularis_158 | Scymnus_sinuanodulus_831 | 0.202 |
| Hyperaspis_lateralis_657 | Stethorus_punctillum_851 | 0.202 |
| Hyperaspis_reppensis_664 | Stethorus_punctillum_855 | 0.202 |
| Hyperaspis_reppensis_665 | Stethorus_punctillum_855 | 0.202 |
| Hyperaspis_reppensis_666 | Stethorus_punctillum_855 | 0.202 |
| Hyperaspis_lateralis_657 | Stethorus_punctillum_858 | 0.202 |
| Platynaspis_luteorubra_51 | Subcoccinella_vigintiquatuorpunctata_620 | 0.202 |
| Platynaspis_luteorubra_53 | Subcoccinella_vigintiquatuorpunctata_620 | 0.202 |
| Platynaspis_luteorubra_58 | Subcoccinella_vigintiquatuorpunctata_620 | 0.202 |
| Platynaspis_luteorubra_51 | Subcoccinella_vigintiquatuorpunctata_621 | 0.202 |
| Platynaspis_luteorubra_53 | Subcoccinella_vigintiquatuorpunctata_621 | 0.202 |
| Platynaspis_luteorubra_58 | Subcoccinella_vigintiquatuorpunctata_621 | 0.202 |
| Chilocorus_stigma_46 | Subcoccinella_vigintiquatuorpunctata_623 | 0.202 |
| Platynaspis_luteorubra_51 | Subcoccinella_vigintiquatuorpunctata_624 | 0.202 |
| Platynaspis_luteorubra_53 | Subcoccinella_vigintiquatuorpunctata_624 | 0.202 |
| Platynaspis_luteorubra_58 | Subcoccinella_vigintiquatuorpunctata_624 | 0.202 |
| Platynaspis_luteorubra_51 | Subcoccinella_vigintiquatuorpunctata_628 | 0.202 |
| Platynaspis_luteorubra_53 | Subcoccinella_vigintiquatuorpunctata_628 | 0.202 |
| Platynaspis_luteorubra_58 | Subcoccinella_vigintiquatuorpunctata_628 | 0.202 |
| Platynaspis_luteorubra_51 | Subcoccinella_vigintiquatuorpunctata_629 | 0.202 |
| Platynaspis_luteorubra_53 | Subcoccinella_vigintiquatuorpunctata_629 | 0.202 |
| Platynaspis_luteorubra_58 | Subcoccinella_vigintiquatuorpunctata_629 | 0.202 |
| Platynaspis_luteorubra_51 | Subcoccinella_vigintiquatuorpunctata_631 | 0.202 |
| Platynaspis_luteorubra_53 | Subcoccinella_vigintiquatuorpunctata_631 | 0.202 |
| Platynaspis_luteorubra_58 | Subcoccinella_vigintiquatuorpunctata_631 | 0.202 |
| Chilocorus_renipustulatus_39 | Scymnus_sinuanodulus_828 | 0.202 |
| Chilocorus_renipustulatus_34 | Scymnus_sinuanodulus_830 | 0.202 |
| Nephus_quadrimaculatus_668 | Scymnus_sinuanodulus_830 | 0.202 |

Supplementary Table S4 (continued)

| | | |
|---|---|---|
| Calvia_quatuordecimguttata_205 | Scymnus_suturalis_833 | 0.202 |
| Nephus_redtenbacheri_674 | Scymnus_sinuanodulus_831 | 0.202 |
| Myzia_pullata_517 | Subcoccinella_vigintiquatuorpunctata_623 | 0.202 |
| Scymnus_interruptus_762 | Subcoccinella_vigintiquatuorpunctata_630 | 0.202 |
| Brumoides_suturalis_1 | Vibidia_duodecimguttata_596 | 0.202 |
| Calvia_quatuordecimguttata_206 | Scymnus_rubromaculatus_822 | 0.202 |
| Calvia_quatuordecimguttata_216 | Scymnus_rubromaculatus_822 | 0.202 |
| Coccidula_rufa_67 | Scymnus_rubromaculatus_822 | 0.202 |
| Coccidula_rufa_74 | Scymnus_rubromaculatus_822 | 0.202 |
| Henosepilachna_argus_615 | Scymnus_rubromaculatus_822 | 0.202 |
| Henosepilachna_argus_616 | Scymnus_rubromaculatus_822 | 0.202 |
| Henosepilachna_argus_617 | Scymnus_rubromaculatus_822 | 0.202 |
| Henosepilachna_argus_618 | Scymnus_rubromaculatus_822 | 0.202 |
| Calvia_quatuordecimguttata_206 | Scymnus_rubromaculatus_823 | 0.202 |
| Calvia_quatuordecimguttata_216 | Scymnus_rubromaculatus_823 | 0.202 |
| Coccidula_rufa_67 | Scymnus_rubromaculatus_823 | 0.202 |
| Coccidula_rufa_74 | Scymnus_rubromaculatus_823 | 0.202 |
| Henosepilachna_argus_615 | Scymnus_rubromaculatus_823 | 0.202 |
| Henosepilachna_argus_616 | Scymnus_rubromaculatus_823 | 0.202 |
| Henosepilachna_argus_617 | Scymnus_rubromaculatus_823 | 0.202 |
| Henosepilachna_argus_618 | Scymnus_rubromaculatus_823 | 0.202 |
| Calvia_quatuordecimguttata_206 | Scymnus_rubromaculatus_824 | 0.202 |
| Calvia_quatuordecimguttata_216 | Scymnus_rubromaculatus_824 | 0.202 |
| Coccidula_rufa_67 | Scymnus_rubromaculatus_824 | 0.202 |
| Coccidula_rufa_74 | Scymnus_rubromaculatus_824 | 0.202 |
| Henosepilachna_argus_615 | Scymnus_rubromaculatus_824 | 0.202 |
| Henosepilachna_argus_616 | Scymnus_rubromaculatus_824 | 0.202 |
| Henosepilachna_argus_617 | Scymnus_rubromaculatus_824 | 0.202 |
| Henosepilachna_argus_618 | Scymnus_rubromaculatus_824 | 0.202 |
| Calvia_quatuordecimguttata_206 | Scymnus_rubromaculatus_825 | 0.202 |
| Calvia_quatuordecimguttata_216 | Scymnus_rubromaculatus_825 | 0.202 |
| Coccidula_rufa_67 | Scymnus_rubromaculatus_825 | 0.202 |
| Coccidula_rufa_74 | Scymnus_rubromaculatus_825 | 0.202 |
| Henosepilachna_argus_615 | Scymnus_rubromaculatus_825 | 0.202 |
| Henosepilachna_argus_616 | Scymnus_rubromaculatus_825 | 0.202 |
| Henosepilachna_argus_617 | Scymnus_rubromaculatus_825 | 0.202 |
| Henosepilachna_argus_618 | Scymnus_rubromaculatus_825 | 0.202 |
| Calvia_quatuordecimguttata_206 | Scymnus_rubromaculatus_826 | 0.202 |
| Calvia_quatuordecimguttata_216 | Scymnus_rubromaculatus_826 | 0.202 |
| Coccidula_rufa_67 | Scymnus_rubromaculatus_826 | 0.202 |
| Coccidula_rufa_74 | Scymnus_rubromaculatus_826 | 0.202 |
| Henosepilachna_argus_615 | Scymnus_rubromaculatus_826 | 0.202 |
| Henosepilachna_argus_616 | Scymnus_rubromaculatus_826 | 0.202 |
| Henosepilachna_argus_617 | Scymnus_rubromaculatus_826 | 0.202 |
| Henosepilachna_argus_618 | Scymnus_rubromaculatus_826 | 0.202 |
| Calvia_quatuordecimguttata_206 | Scymnus_rubromaculatus_827 | 0.202 |
| Calvia_quatuordecimguttata_216 | Scymnus_rubromaculatus_827 | 0.202 |
| Coccidula_rufa_67 | Scymnus_rubromaculatus_827 | 0.202 |
| Coccidula_rufa_74 | Scymnus_rubromaculatus_827 | 0.202 |
| Henosepilachna_argus_615 | Scymnus_rubromaculatus_827 | 0.202 |
| Henosepilachna_argus_616 | Scymnus_rubromaculatus_827 | 0.202 |
| Henosepilachna_argus_617 | Scymnus_rubromaculatus_827 | 0.202 |
| Henosepilachna_argus_618 | Scymnus_rubromaculatus_827 | 0.202 |
| Nephus_quadrimaculatus_669 | Scymnus_sinuanodulus_828 | 0.202 |
| Nephus_quadrimaculatus_670 | Scymnus_sinuanodulus_828 | 0.202 |
| Nephus_quadrimaculatus_671 | Scymnus_sinuanodulus_828 | 0.202 |

| | | |
|---|---|---|
| Nephus_quadrimaculatus_672 | Scymnus_sinuanodulus_828 | 0.202 |
| Nephus_quadrimaculatus_673 | Scymnus_sinuanodulus_828 | 0.202 |
| Platynaspis_luteorubra_55 | Subcoccinella_vigintiquatuorpunctata_619 | 0.202 |
| Psyllobora_vigintiduopunctata_570 | Scymnus_sinuanodulus_831 | 0.202 |
| Psyllobora_vigintiduopunctata_572 | Scymnus_sinuanodulus_831 | 0.202 |
| Myzia_pullata_518 | Subcoccinella_vigintiquatuorpunctata_620 | 0.202 |
| Myzia_pullata_518 | Subcoccinella_vigintiquatuorpunctata_621 | 0.202 |
| Myzia_pullata_517 | Subcoccinella_vigintiquatuorpunctata_622 | 0.202 |
| Scymnus_interruptus_760 | Subcoccinella_vigintiquatuorpunctata_622 | 0.202 |
| Scymnus_interruptus_765 | Subcoccinella_vigintiquatuorpunctata_622 | 0.202 |
| Scymnus_interruptus_768 | Subcoccinella_vigintiquatuorpunctata_622 | 0.202 |
| Myzia_pullata_517 | Subcoccinella_vigintiquatuorpunctata_624 | 0.202 |
| Scymnus_interruptus_760 | Subcoccinella_vigintiquatuorpunctata_625 | 0.202 |
| Scymnus_interruptus_765 | Subcoccinella_vigintiquatuorpunctata_625 | 0.202 |
| Scymnus_interruptus_768 | Subcoccinella_vigintiquatuorpunctata_625 | 0.202 |
| Myzia_pullata_517 | Subcoccinella_vigintiquatuorpunctata_626 | 0.202 |
| Scymnus_interruptus_760 | Subcoccinella_vigintiquatuorpunctata_626 | 0.202 |
| Scymnus_interruptus_765 | Subcoccinella_vigintiquatuorpunctata_626 | 0.202 |
| Scymnus_interruptus_768 | Subcoccinella_vigintiquatuorpunctata_626 | 0.202 |
| Scymnus_camptodromus_707 | Subcoccinella_vigintiquatuorpunctata_627 | 0.202 |
| Scymnus_camptodromus_708 | Subcoccinella_vigintiquatuorpunctata_627 | 0.202 |
| Scymnus_camptodromus_709 | Subcoccinella_vigintiquatuorpunctata_627 | 0.202 |
| Scymnus_camptodromus_710 | Subcoccinella_vigintiquatuorpunctata_627 | 0.202 |
| Scymnus_camptodromus_719 | Subcoccinella_vigintiquatuorpunctata_627 | 0.202 |
| Myzia_pullata_518 | Subcoccinella_vigintiquatuorpunctata_628 | 0.202 |
| Myzia_pullata_518 | Subcoccinella_vigintiquatuorpunctata_629 | 0.202 |
| Scymnus_camptodromus_707 | Subcoccinella_vigintiquatuorpunctata_630 | 0.202 |
| Scymnus_camptodromus_708 | Subcoccinella_vigintiquatuorpunctata_630 | 0.202 |
| Scymnus_camptodromus_709 | Subcoccinella_vigintiquatuorpunctata_630 | 0.202 |
| Scymnus_camptodromus_710 | Subcoccinella_vigintiquatuorpunctata_630 | 0.202 |
| Scymnus_camptodromus_719 | Subcoccinella_vigintiquatuorpunctata_630 | 0.202 |
| Myzia_pullata_518 | Subcoccinella_vigintiquatuorpunctata_631 | 0.202 |
| Scymnus_abietis_684 | Tytthaspis_sedecimpunctata_585 | 0.202 |
| Scymnus_abietis_686 | Tytthaspis_sedecimpunctata_585 | 0.202 |
| Scymnus_abietis_687 | Tytthaspis_sedecimpunctata_585 | 0.202 |
| Scymnus_abietis_688 | Tytthaspis_sedecimpunctata_585 | 0.202 |
| Coccidula_rufa_70 | Scymnus_rubromaculatus_822 | 0.202 |
| Coccidula_rufa_70 | Scymnus_rubromaculatus_823 | 0.202 |
| Coccidula_rufa_70 | Scymnus_rubromaculatus_824 | 0.202 |
| Coccidula_rufa_70 | Scymnus_rubromaculatus_825 | 0.202 |
| Coccidula_rufa_70 | Scymnus_rubromaculatus_826 | 0.202 |
| Coccidula_rufa_70 | Scymnus_rubromaculatus_827 | 0.202 |
| Chilocorus_stigma_43 | Subcoccinella_vigintiquatuorpunctata_619 | 0.202 |
| Chilocorus_stigma_47 | Subcoccinella_vigintiquatuorpunctata_619 | 0.202 |
| Psyllobora_vigintiduopunctata_570 | Subcoccinella_vigintiquatuorpunctata_619 | 0.202 |
| Psyllobora_vigintiduopunctata_572 | Subcoccinella_vigintiquatuorpunctata_619 | 0.202 |
| Psyllobora_vigintiduopunctata_559 | Subcoccinella_vigintiquatuorpunctata_624 | 0.202 |
| Psyllobora_vigintiduopunctata_559 | Subcoccinella_vigintiquatuorpunctata_630 | 0.202 |
| Myzia_oblongoguttata_503 | Scymnus_sinuanodulus_829 | 0.202 |
| Myzia_oblongoguttata_506 | Scymnus_sinuanodulus_829 | 0.202 |
| Myzia_oblongoguttata_515 | Scymnus_sinuanodulus_829 | 0.202 |
| Scymnus_camptodromus_713 | Subcoccinella_vigintiquatuorpunctata_623 | 0.202 |
| Scymnus_camptodromus_716 | Subcoccinella_vigintiquatuorpunctata_623 | 0.202 |
| Scymnus_camptodromus_712 | Subcoccinella_vigintiquatuorpunctata_624 | 0.202 |
| Hippodamia_convergens_399 | Scymnus_suturalis_833 | 0.202 |
| Hippodamia_convergens_404 | Scymnus_suturalis_833 | 0.202 |

| | | |
|---|---|---|
| Hippodamia_convergens_405 | Scymnus_suturalis_833 | 0.202 |
| Hippodamia_convergens_406 | Scymnus_suturalis_833 | 0.202 |
| Hippodamia_quinquesignata_443 | Scymnus_suturalis_833 | 0.202 |
| Hippodamia_quinquesignata_444 | Scymnus_suturalis_833 | 0.202 |
| Hippodamia_quinquesignata_445 | Scymnus_suturalis_833 | 0.202 |
| Hippodamia_caseyi_391 | Scymnus_suturalis_838 | 0.202 |
| Hippodamia_moesta_411 | Scymnus_suturalis_838 | 0.202 |
| Hippodamia_caseyi_391 | Scymnus_suturalis_840 | 0.202 |
| Hippodamia_moesta_411 | Scymnus_suturalis_840 | 0.202 |
| Scymnus_apetzi_695 | Tytthaspis_sedecimpunctata_591 | 0.202 |
| Hippodamia_caseyi_392 | Scymnus_suturalis_841 | 0.203 |
| Hippodamia_caseyi_392 | Scymnus_suturalis_842 | 0.203 |
| Hippodamia_caseyi_392 | Scymnus_suturalis_844 | 0.203 |
| Hippodamia_caseyi_392 | Scymnus_suturalis_846 | 0.203 |
| Hippodamia_caseyi_392 | Scymnus_suturalis_847 | 0.203 |
| Hippodamia_caseyi_392 | Scymnus_suturalis_848 | 0.203 |
| Scymnus_camptodromus_705 | Subcoccinella_vigintiquatuorpunctata_620 | 0.203 |
| Scymnus_camptodromus_705 | Subcoccinella_vigintiquatuorpunctata_621 | 0.203 |
| Scymnus_camptodromus_713 | Subcoccinella_vigintiquatuorpunctata_622 | 0.203 |
| Scymnus_camptodromus_716 | Subcoccinella_vigintiquatuorpunctata_622 | 0.203 |
| Scymnus_ferrugatus_724 | Subcoccinella_vigintiquatuorpunctata_622 | 0.203 |
| Scymnus_camptodromus_705 | Subcoccinella_vigintiquatuorpunctata_624 | 0.203 |
| Scymnus_camptodromus_713 | Subcoccinella_vigintiquatuorpunctata_626 | 0.203 |
| Scymnus_camptodromus_716 | Subcoccinella_vigintiquatuorpunctata_626 | 0.203 |
| Scymnus_ferrugatus_724 | Subcoccinella_vigintiquatuorpunctata_626 | 0.203 |
| Scymnus_ferrugatus_724 | Subcoccinella_vigintiquatuorpunctata_627 | 0.203 |
| Scymnus_camptodromus_705 | Subcoccinella_vigintiquatuorpunctata_628 | 0.203 |
| Scymnus_camptodromus_705 | Subcoccinella_vigintiquatuorpunctata_629 | 0.203 |
| Scymnus_ferrugatus_724 | Subcoccinella_vigintiquatuorpunctata_630 | 0.203 |
| Scymnus_camptodromus_705 | Subcoccinella_vigintiquatuorpunctata_631 | 0.203 |
| Chilocorus_nigritus_26 | Vibidia_duodecimguttata_596 | 0.203 |
| Mulsantina_hudsonica_482 | Vibidia_duodecimguttata_597 | 0.203 |
| Adalia_bipunctata_101 | Vibidia_duodecimguttata_599 | 0.203 |
| Adalia_bipunctata_104 | Vibidia_duodecimguttata_599 | 0.203 |
| Chilocorus_nigritus_26 | Vibidia_duodecimguttata_599 | 0.203 |
| Mulsantina_hudsonica_482 | Vibidia_duodecimguttata_600 | 0.203 |
| Adalia_bipunctata_101 | Vibidia_duodecimguttata_601 | 0.203 |
| Adalia_bipunctata_104 | Vibidia_duodecimguttata_601 | 0.203 |
| Adalia_bipunctata_101 | Vibidia_duodecimguttata_602 | 0.203 |
| Adalia_bipunctata_104 | Vibidia_duodecimguttata_602 | 0.203 |
| Chilocorus_nigritus_26 | Vibidia_duodecimguttata_602 | 0.203 |
| Adalia_bipunctata_100 | Vibidia_duodecimguttata_603 | 0.203 |
| Adalia_bipunctata_102 | Vibidia_duodecimguttata_603 | 0.203 |
| Adalia_bipunctata_103 | Vibidia_duodecimguttata_603 | 0.203 |
| Adalia_bipunctata_106 | Vibidia_duodecimguttata_603 | 0.203 |
| Adalia_bipunctata_107 | Vibidia_duodecimguttata_603 | 0.203 |
| Adalia_bipunctata_108 | Vibidia_duodecimguttata_603 | 0.203 |
| Adalia_bipunctata_109 | Vibidia_duodecimguttata_603 | 0.203 |
| Chilocorus_nigritus_26 | Vibidia_duodecimguttata_603 | 0.203 |
| Scymnus_nubilus_803 | Scymnus_sinuanodulus_830 | 0.203 |
| Scymnus_nubilus_804 | Scymnus_sinuanodulus_830 | 0.203 |
| Scymnus_nubilus_808 | Scymnus_sinuanodulus_830 | 0.203 |
| Scymnus_nubilus_809 | Scymnus_sinuanodulus_830 | 0.203 |
| Hippodamia_caseyi_392 | Scymnus_suturalis_832 | 0.203 |
| Hippodamia_caseyi_391 | Scymnus_suturalis_834 | 0.203 |
| Hippodamia_moesta_411 | Scymnus_suturalis_834 | 0.203 |

Supplementary Table S4 (continued)

| | | |
|---|---|---|
| Hippodamia_caseyi_392 | Scymnus_suturalis_835 | 0.203 |
| Hippodamia_caseyi_392 | Scymnus_suturalis_836 | 0.203 |
| Hippodamia_caseyi_391 | Scymnus_suturalis_837 | 0.203 |
| Hippodamia_moesta_411 | Scymnus_suturalis_837 | 0.203 |
| Hippodamia_caseyi_392 | Scymnus_suturalis_839 | 0.203 |
| Scymnus_apetzi_695 | Stethorus_punctillum_850 | 0.203 |
| Scymnus_apetzi_695 | Stethorus_punctillum_854 | 0.203 |
| Scymnus_apetzi_695 | Stethorus_punctillum_856 | 0.203 |
| Anisosticta_bitriangularis_155 | Scymnus_sinuanodulus_829 | 0.203 |
| Anisosticta_bitriangularis_158 | Scymnus_sinuanodulus_829 | 0.203 |
| Scymnus_interruptus_760 | Subcoccinella_vigintiquatuorpunctata_619 | 0.203 |
| Scymnus_interruptus_765 | Subcoccinella_vigintiquatuorpunctata_619 | 0.203 |
| Scymnus_interruptus_768 | Subcoccinella_vigintiquatuorpunctata_619 | 0.203 |
| Scymnus_apetzi_693 | Tytthaspis_sedecimpunctata_586 | 0.203 |
| Psyllobora_vigintiduopunctata_558 | Subcoccinella_vigintiquatuorpunctata_620 | 0.203 |
| Psyllobora_vigintiduopunctata_558 | Subcoccinella_vigintiquatuorpunctata_621 | 0.203 |
| Psyllobora_vigintiduopunctata_558 | Subcoccinella_vigintiquatuorpunctata_627 | 0.203 |
| Psyllobora_vigintiduopunctata_558 | Subcoccinella_vigintiquatuorpunctata_628 | 0.203 |
| Psyllobora_vigintiduopunctata_558 | Subcoccinella_vigintiquatuorpunctata_629 | 0.203 |
| Psyllobora_vigintiduopunctata_558 | Subcoccinella_vigintiquatuorpunctata_631 | 0.203 |
| Brachiacantha_ursina_637 | Subcoccinella_vigintiquatuorpunctata_620 | 0.203 |
| Scymnus_ferrugatus_731 | Subcoccinella_vigintiquatuorpunctata_620 | 0.203 |
| Brachiacantha_ursina_637 | Subcoccinella_vigintiquatuorpunctata_621 | 0.203 |
| Scymnus_ferrugatus_731 | Subcoccinella_vigintiquatuorpunctata_621 | 0.203 |
| Scymnus_abietis_683 | Subcoccinella_vigintiquatuorpunctata_623 | 0.203 |
| Scymnus_abietis_684 | Subcoccinella_vigintiquatuorpunctata_623 | 0.203 |
| Scymnus_abietis_686 | Subcoccinella_vigintiquatuorpunctata_623 | 0.203 |
| Scymnus_abietis_687 | Subcoccinella_vigintiquatuorpunctata_623 | 0.203 |
| Scymnus_abietis_688 | Subcoccinella_vigintiquatuorpunctata_623 | 0.203 |
| Calvia_quatuordecimguttata_206 | Subcoccinella_vigintiquatuorpunctata_627 | 0.203 |
| Calvia_quatuordecimguttata_207 | Subcoccinella_vigintiquatuorpunctata_627 | 0.203 |
| Calvia_quatuordecimguttata_216 | Subcoccinella_vigintiquatuorpunctata_627 | 0.203 |
| Brachiacantha_ursina_637 | Subcoccinella_vigintiquatuorpunctata_628 | 0.203 |
| Scymnus_ferrugatus_731 | Subcoccinella_vigintiquatuorpunctata_628 | 0.203 |
| Brachiacantha_ursina_637 | Subcoccinella_vigintiquatuorpunctata_629 | 0.203 |
| Scymnus_ferrugatus_731 | Subcoccinella_vigintiquatuorpunctata_629 | 0.203 |
| Brachiacantha_ursina_638 | Subcoccinella_vigintiquatuorpunctata_630 | 0.203 |
| Calvia_quatuordecimguttata_206 | Subcoccinella_vigintiquatuorpunctata_630 | 0.203 |
| Calvia_quatuordecimguttata_207 | Subcoccinella_vigintiquatuorpunctata_630 | 0.203 |
| Calvia_quatuordecimguttata_216 | Subcoccinella_vigintiquatuorpunctata_630 | 0.203 |
| Brachiacantha_ursina_637 | Subcoccinella_vigintiquatuorpunctata_631 | 0.203 |
| Scymnus_ferrugatus_731 | Subcoccinella_vigintiquatuorpunctata_631 | 0.203 |
| Mulsantina_hudsonica_479 | Vibidia_duodecimguttata_596 | 0.203 |
| Mulsantina_hudsonica_480 | Vibidia_duodecimguttata_596 | 0.203 |
| Mulsantina_hudsonica_483 | Vibidia_duodecimguttata_596 | 0.203 |
| Chilocorus_nigritus_26 | Vibidia_duodecimguttata_597 | 0.203 |
| Mulsantina_hudsonica_479 | Vibidia_duodecimguttata_598 | 0.203 |
| Mulsantina_hudsonica_480 | Vibidia_duodecimguttata_598 | 0.203 |
| Mulsantina_hudsonica_483 | Vibidia_duodecimguttata_598 | 0.203 |
| Mulsantina_hudsonica_481 | Vibidia_duodecimguttata_599 | 0.203 |
| Chilocorus_nigritus_26 | Vibidia_duodecimguttata_600 | 0.203 |
| Mulsantina_hudsonica_479 | Vibidia_duodecimguttata_601 | 0.203 |
| Mulsantina_hudsonica_481 | Vibidia_duodecimguttata_602 | 0.203 |
| Mulsantina_hudsonica_480 | Vibidia_duodecimguttata_603 | 0.203 |
| Mulsantina_hudsonica_483 | Vibidia_duodecimguttata_603 | 0.203 |
| Clitostethus_arcuatus_639 | Scymnus_sinuanodulus_828 | 0.203 |

Supplementary Table S4 (continued)

| | | |
|---|---|---|
| Clitostethus_arcuatus_640 | Scymnus_sinuanodulus_828 | 0.203 |
| Clitostethus_arcuatus_641 | Scymnus_sinuanodulus_828 | 0.203 |
| Clitostethus_arcuatus_643 | Scymnus_sinuanodulus_828 | 0.203 |
| Scymnus_nubilus_803 | Scymnus_sinuanodulus_828 | 0.203 |
| Scymnus_nubilus_804 | Scymnus_sinuanodulus_828 | 0.203 |
| Scymnus_nubilus_808 | Scymnus_sinuanodulus_828 | 0.203 |
| Scymnus_nubilus_809 | Scymnus_sinuanodulus_828 | 0.203 |
| Scymnus_camptodromus_704 | Subcoccinella_vigintiquatuorpunctata_619 | 0.203 |
| Nephus_redtenbacheri_674 | Scymnus_sinuanodulus_829 | 0.203 |
| Calvia_quatuordecimguttata_211 | Subcoccinella_vigintiquatuorpunctata_620 | 0.203 |
| Calvia_quatuordecimguttata_212 | Subcoccinella_vigintiquatuorpunctata_620 | 0.203 |
| Calvia_quatuordecimguttata_213 | Subcoccinella_vigintiquatuorpunctata_620 | 0.203 |
| Calvia_quatuordecimguttata_214 | Subcoccinella_vigintiquatuorpunctata_620 | 0.203 |
| Calvia_quatuordecimguttata_215 | Subcoccinella_vigintiquatuorpunctata_620 | 0.203 |
| Calvia_quatuordecimguttata_211 | Subcoccinella_vigintiquatuorpunctata_621 | 0.203 |
| Calvia_quatuordecimguttata_212 | Subcoccinella_vigintiquatuorpunctata_621 | 0.203 |
| Calvia_quatuordecimguttata_213 | Subcoccinella_vigintiquatuorpunctata_621 | 0.203 |
| Calvia_quatuordecimguttata_214 | Subcoccinella_vigintiquatuorpunctata_621 | 0.203 |
| Calvia_quatuordecimguttata_215 | Subcoccinella_vigintiquatuorpunctata_621 | 0.203 |
| Brachiacantha_ursina_637 | Subcoccinella_vigintiquatuorpunctata_623 | 0.203 |
| Scymnus_ferrugatus_731 | Subcoccinella_vigintiquatuorpunctata_623 | 0.203 |
| Scymnus_abietis_683 | Subcoccinella_vigintiquatuorpunctata_626 | 0.203 |
| Scymnus_abietis_684 | Subcoccinella_vigintiquatuorpunctata_626 | 0.203 |
| Scymnus_abietis_686 | Subcoccinella_vigintiquatuorpunctata_626 | 0.203 |
| Scymnus_abietis_687 | Subcoccinella_vigintiquatuorpunctata_626 | 0.203 |
| Scymnus_abietis_688 | Subcoccinella_vigintiquatuorpunctata_626 | 0.203 |
| Rhyzobius_chrysomeloides_85 | Subcoccinella_vigintiquatuorpunctata_627 | 0.203 |
| Rhyzobius_chrysomeloides_86 | Subcoccinella_vigintiquatuorpunctata_627 | 0.203 |
| Rhyzobius_chrysomeloides_87 | Subcoccinella_vigintiquatuorpunctata_627 | 0.203 |
| Rhyzobius_chrysomeloides_88 | Subcoccinella_vigintiquatuorpunctata_627 | 0.203 |
| Rhyzobius_chrysomeloides_89 | Subcoccinella_vigintiquatuorpunctata_627 | 0.203 |
| Rhyzobius_chrysomeloides_90 | Subcoccinella_vigintiquatuorpunctata_627 | 0.203 |
| Rhyzobius_chrysomeloides_91 | Subcoccinella_vigintiquatuorpunctata_627 | 0.203 |
| Rhyzobius_chrysomeloides_92 | Subcoccinella_vigintiquatuorpunctata_627 | 0.203 |
| Rhyzobius_chrysomeloides_93 | Subcoccinella_vigintiquatuorpunctata_627 | 0.203 |
| Rhyzobius_chrysomeloides_94 | Subcoccinella_vigintiquatuorpunctata_627 | 0.203 |
| Rhyzobius_chrysomeloides_95 | Subcoccinella_vigintiquatuorpunctata_627 | 0.203 |
| Calvia_quatuordecimguttata_211 | Subcoccinella_vigintiquatuorpunctata_628 | 0.203 |
| Calvia_quatuordecimguttata_212 | Subcoccinella_vigintiquatuorpunctata_628 | 0.203 |
| Calvia_quatuordecimguttata_213 | Subcoccinella_vigintiquatuorpunctata_628 | 0.203 |
| Calvia_quatuordecimguttata_214 | Subcoccinella_vigintiquatuorpunctata_628 | 0.203 |
| Calvia_quatuordecimguttata_215 | Subcoccinella_vigintiquatuorpunctata_628 | 0.203 |
| Calvia_quatuordecimguttata_211 | Subcoccinella_vigintiquatuorpunctata_629 | 0.203 |
| Calvia_quatuordecimguttata_212 | Subcoccinella_vigintiquatuorpunctata_629 | 0.203 |
| Calvia_quatuordecimguttata_213 | Subcoccinella_vigintiquatuorpunctata_629 | 0.203 |
| Calvia_quatuordecimguttata_214 | Subcoccinella_vigintiquatuorpunctata_629 | 0.203 |
| Calvia_quatuordecimguttata_215 | Subcoccinella_vigintiquatuorpunctata_629 | 0.203 |
| Calvia_quatuordecimguttata_211 | Subcoccinella_vigintiquatuorpunctata_631 | 0.203 |
| Calvia_quatuordecimguttata_212 | Subcoccinella_vigintiquatuorpunctata_631 | 0.203 |
| Calvia_quatuordecimguttata_213 | Subcoccinella_vigintiquatuorpunctata_631 | 0.203 |
| Calvia_quatuordecimguttata_214 | Subcoccinella_vigintiquatuorpunctata_631 | 0.203 |
| Calvia_quatuordecimguttata_215 | Subcoccinella_vigintiquatuorpunctata_631 | 0.203 |
| Harmonia_quadripunctata_380 | Vibidia_duodecimguttata_596 | 0.203 |
| Harmonia_quadripunctata_380 | Vibidia_duodecimguttata_598 | 0.203 |
| Scymnus_camptodromus_717 | Vibidia_duodecimguttata_597 | 0.203 |
| Scymnus_camptodromus_717 | Vibidia_duodecimguttata_600 | 0.203 |

| | | |
|---|---|---|
| Scymnus_camptodromus_713 | Subcoccinella_vigintiquatuorpunctata_619 | 0.203 |
| Scymnus_camptodromus_716 | Subcoccinella_vigintiquatuorpunctata_619 | 0.203 |
| Scymnus_ferrugatus_724 | Subcoccinella_vigintiquatuorpunctata_619 | 0.203 |
| Coccidula_scutellata_84 | Subcoccinella_vigintiquatuorpunctata_625 | 0.203 |
| Brachiacantha_rotunda_636 | Scymnus_sinuanodulus_829 | 0.203 |
| Psyllobora_vigintiduopunctata_570 | Scymnus_sinuanodulus_829 | 0.203 |
| Psyllobora_vigintiduopunctata_572 | Scymnus_sinuanodulus_829 | 0.203 |
| Psyllobora_vigintiduopunctata_568 | Scymnus_sinuanodulus_831 | 0.203 |
| Propylaea_quatuordecimpunctata_532 | Vibidia_duodecimguttata_597 | 0.203 |
| Propylaea_quatuordecimpunctata_537 | Vibidia_duodecimguttata_597 | 0.203 |
| Propylaea_quatuordecimpunctata_539 | Vibidia_duodecimguttata_597 | 0.203 |
| Propylaea_quatuordecimpunctata_532 | Vibidia_duodecimguttata_600 | 0.203 |
| Propylaea_quatuordecimpunctata_537 | Vibidia_duodecimguttata_600 | 0.203 |
| Propylaea_quatuordecimpunctata_539 | Vibidia_duodecimguttata_600 | 0.203 |
| Hyperaspis_lateralis_655 | Tytthaspis_sedecimpunctata_587 | 0.203 |
| Hyperaspis_lateralis_654 | Tytthaspis_sedecimpunctata_586 | 0.203 |
| Hyperaspis_lateralis_657 | Tytthaspis_sedecimpunctata_586 | 0.203 |
| Hyperaspis_lateralis_655 | Tytthaspis_sedecimpunctata_589 | 0.203 |
| Hyperaspis_lateralis_654 | Tytthaspis_sedecimpunctata_591 | 0.203 |
| Hyperaspis_lateralis_657 | Tytthaspis_sedecimpunctata_591 | 0.203 |
| Coccidophilus_marginatus_869 | Tytthaspis_sedecimpunctata_580 | 0.203 |
| Coccidophilus_marginatus_868 | Tytthaspis_sedecimpunctata_583 | 0.203 |
| Coccidophilus_marginatus_868 | Tytthaspis_sedecimpunctata_592 | 0.203 |
| Scymnus_camptodromus_711 | Vibidia_duodecimguttata_596 | 0.203 |
| Scymnus_camptodromus_711 | Vibidia_duodecimguttata_598 | 0.203 |
| Scymnus_camptodromus_717 | Vibidia_duodecimguttata_598 | 0.203 |
| Scymnus_camptodromus_711 | Vibidia_duodecimguttata_599 | 0.203 |
| Scymnus_camptodromus_717 | Vibidia_duodecimguttata_599 | 0.203 |
| Scymnus_camptodromus_711 | Vibidia_duodecimguttata_601 | 0.203 |
| Scymnus_camptodromus_717 | Vibidia_duodecimguttata_601 | 0.203 |
| Scymnus_camptodromus_711 | Vibidia_duodecimguttata_602 | 0.203 |
| Scymnus_camptodromus_717 | Vibidia_duodecimguttata_602 | 0.203 |
| Platynaspis_luteorubra_50 | Stethorus_punctillum_860 | 0.203 |
| Coccidophilus_marginatus_869 | Tytthaspis_sedecimpunctata_584 | 0.203 |
| Platynaspis_luteorubra_50 | Stethorus_punctillum_851 | 0.203 |
| Platynaspis_luteorubra_50 | Stethorus_punctillum_858 | 0.203 |
| Scymnus_mimulus_775 | Tytthaspis_sedecimpunctata_584 | 0.203 |
| Scymnus_mimulus_777 | Tytthaspis_sedecimpunctata_584 | 0.203 |
| Brachiacantha_rotunda_636 | Subcoccinella_vigintiquatuorpunctata_625 | 0.203 |
| Propylaea_quatuordecimpunctata_540 | Vibidia_duodecimguttata_596 | 0.203 |
| Propylaea_quatuordecimpunctata_543 | Vibidia_duodecimguttata_596 | 0.203 |
| Propylaea_quatuordecimpunctata_548 | Vibidia_duodecimguttata_596 | 0.203 |
| Propylaea_quatuordecimpunctata_532 | Vibidia_duodecimguttata_598 | 0.203 |
| Propylaea_quatuordecimpunctata_537 | Vibidia_duodecimguttata_598 | 0.203 |
| Propylaea_quatuordecimpunctata_539 | Vibidia_duodecimguttata_598 | 0.203 |
| Propylaea_quatuordecimpunctata_533 | Vibidia_duodecimguttata_599 | 0.203 |
| Propylaea_quatuordecimpunctata_534 | Vibidia_duodecimguttata_599 | 0.203 |
| Propylaea_quatuordecimpunctata_535 | Vibidia_duodecimguttata_599 | 0.203 |
| Propylaea_quatuordecimpunctata_536 | Vibidia_duodecimguttata_599 | 0.203 |
| Propylaea_quatuordecimpunctata_538 | Vibidia_duodecimguttata_599 | 0.203 |
| Propylaea_quatuordecimpunctata_541 | Vibidia_duodecimguttata_599 | 0.203 |
| Propylaea_quatuordecimpunctata_542 | Vibidia_duodecimguttata_599 | 0.203 |
| Propylaea_quatuordecimpunctata_544 | Vibidia_duodecimguttata_599 | 0.203 |
| Propylaea_quatuordecimpunctata_546 | Vibidia_duodecimguttata_599 | 0.203 |
| Propylaea_quatuordecimpunctata_549 | Vibidia_duodecimguttata_599 | 0.203 |
| Neoharmonia_venusta_521 | Vibidia_duodecimguttata_601 | 0.203 |

| | | |
|---|---|---|
| Propylaea_quatuordecimpunctata_533 | Vibidia_duodecimguttata_602 | 0.203 |
| Propylaea_quatuordecimpunctata_534 | Vibidia_duodecimguttata_602 | 0.203 |
| Propylaea_quatuordecimpunctata_535 | Vibidia_duodecimguttata_602 | 0.203 |
| Propylaea_quatuordecimpunctata_536 | Vibidia_duodecimguttata_602 | 0.203 |
| Propylaea_quatuordecimpunctata_538 | Vibidia_duodecimguttata_602 | 0.203 |
| Propylaea_quatuordecimpunctata_541 | Vibidia_duodecimguttata_602 | 0.203 |
| Propylaea_quatuordecimpunctata_542 | Vibidia_duodecimguttata_602 | 0.203 |
| Propylaea_quatuordecimpunctata_544 | Vibidia_duodecimguttata_602 | 0.203 |
| Propylaea_quatuordecimpunctata_546 | Vibidia_duodecimguttata_602 | 0.203 |
| Propylaea_quatuordecimpunctata_549 | Vibidia_duodecimguttata_602 | 0.203 |
| Propylaea_quatuordecimpunctata_534 | Vibidia_duodecimguttata_603 | 0.203 |
| Propylaea_quatuordecimpunctata_538 | Vibidia_duodecimguttata_603 | 0.203 |
| Propylaea_quatuordecimpunctata_542 | Vibidia_duodecimguttata_603 | 0.203 |
| Propylaea_quatuordecimpunctata_544 | Vibidia_duodecimguttata_603 | 0.203 |
| Propylaea_quatuordecimpunctata_545 | Vibidia_duodecimguttata_603 | 0.203 |
| Propylaea_quatuordecimpunctata_546 | Vibidia_duodecimguttata_603 | 0.203 |
| Propylaea_quatuordecimpunctata_549 | Vibidia_duodecimguttata_603 | 0.203 |
| Scymnus_interruptus_760 | Stethorus_punctillum_850 | 0.203 |
| Scymnus_interruptus_765 | Stethorus_punctillum_850 | 0.203 |
| Scymnus_interruptus_768 | Stethorus_punctillum_850 | 0.203 |
| Scymnus_interruptus_767 | Stethorus_punctillum_852 | 0.203 |
| Scymnus_interruptus_760 | Stethorus_punctillum_854 | 0.203 |
| Scymnus_interruptus_765 | Stethorus_punctillum_854 | 0.203 |
| Scymnus_interruptus_768 | Stethorus_punctillum_854 | 0.203 |
| Scymnus_interruptus_760 | Stethorus_punctillum_856 | 0.203 |
| Scymnus_interruptus_765 | Stethorus_punctillum_856 | 0.203 |
| Scymnus_interruptus_768 | Stethorus_punctillum_856 | 0.203 |
| Platynaspis_luteorubra_51 | Stethorus_punctillum_860 | 0.203 |
| Platynaspis_luteorubra_53 | Stethorus_punctillum_860 | 0.203 |
| Platynaspis_luteorubra_58 | Stethorus_punctillum_860 | 0.203 |
| Scymnus_interruptus_760 | Stethorus_punctillum_860 | 0.203 |
| Scymnus_interruptus_765 | Stethorus_punctillum_860 | 0.203 |
| Scymnus_interruptus_768 | Stethorus_punctillum_860 | 0.203 |
| Scymnus_mimulus_775 | Tytthaspis_sedecimpunctata_577 | 0.203 |
| Scymnus_mimulus_777 | Tytthaspis_sedecimpunctata_577 | 0.203 |
| Scymnus_mimulus_775 | Tytthaspis_sedecimpunctata_578 | 0.203 |
| Scymnus_mimulus_777 | Tytthaspis_sedecimpunctata_578 | 0.203 |
| Scymnus_mimulus_775 | Tytthaspis_sedecimpunctata_579 | 0.203 |
| Scymnus_mimulus_777 | Tytthaspis_sedecimpunctata_579 | 0.203 |
| Scymnus_mimulus_775 | Tytthaspis_sedecimpunctata_581 | 0.203 |
| Scymnus_mimulus_777 | Tytthaspis_sedecimpunctata_581 | 0.203 |
| Scymnus_mimulus_775 | Tytthaspis_sedecimpunctata_582 | 0.203 |
| Scymnus_mimulus_777 | Tytthaspis_sedecimpunctata_582 | 0.203 |
| Scymnus_mimulus_776 | Tytthaspis_sedecimpunctata_583 | 0.203 |
| Scymnus_mimulus_775 | Tytthaspis_sedecimpunctata_585 | 0.203 |
| Scymnus_mimulus_777 | Tytthaspis_sedecimpunctata_585 | 0.203 |
| Coccidophilus_marginatus_870 | Tytthaspis_sedecimpunctata_586 | 0.203 |
| Coccidophilus_marginatus_870 | Tytthaspis_sedecimpunctata_588 | 0.203 |
| Scymnus_mimulus_775 | Tytthaspis_sedecimpunctata_590 | 0.203 |
| Scymnus_mimulus_777 | Tytthaspis_sedecimpunctata_590 | 0.203 |
| Scymnus_mimulus_775 | Tytthaspis_sedecimpunctata_592 | 0.203 |
| Scymnus_mimulus_777 | Tytthaspis_sedecimpunctata_592 | 0.203 |
| Scymnus_mimulus_775 | Tytthaspis_sedecimpunctata_594 | 0.203 |
| Scymnus_mimulus_777 | Tytthaspis_sedecimpunctata_594 | 0.203 |
| Scymnus_mimulus_775 | Tytthaspis_sedecimpunctata_595 | 0.203 |
| Scymnus_mimulus_777 | Tytthaspis_sedecimpunctata_595 | 0.203 |

Supplementary Table S4 (continued)

| | | |
|---|---|---|
| Scymnus_abietis_689 | Subcoccinella_vigintiquatuorpunctata_623 | 0.203 |
| Scymnus_interruptus_762 | Stethorus_punctillum_850 | 0.203 |
| Scymnus_interruptus_762 | Stethorus_punctillum_854 | 0.203 |
| Scymnus_interruptus_762 | Stethorus_punctillum_856 | 0.203 |
| Scymnus_interruptus_762 | Stethorus_punctillum_860 | 0.203 |
| Scymnus_mimulus_775 | Tytthaspis_sedecimpunctata_580 | 0.203 |
| Scymnus_mimulus_777 | Tytthaspis_sedecimpunctata_580 | 0.203 |
| Scymnus_mimulus_776 | Tytthaspis_sedecimpunctata_587 | 0.203 |
| Coccidophilus_marginatus_870 | Tytthaspis_sedecimpunctata_592 | 0.203 |
| Scymnus_mimulus_778 | Tytthaspis_sedecimpunctata_593 | 0.203 |
| Chilocorus_nigritus_27 | Stethorus_punctillum_852 | 0.203 |
| Brachiacantha_rotunda_634 | Stethorus_punctillum_857 | 0.203 |
| Brachiacantha_rotunda_634 | Stethorus_punctillum_860 | 0.203 |
| Scymnus_loewii_774 | Subcoccinella_vigintiquatuorpunctata_623 | 0.203 |
| Cynegetis_impunctata_614 | Scymnus_suturalis_833 | 0.203 |
| Cynegetis_impunctata_605 | Scymnus_suturalis_840 | 0.203 |
| Cynegetis_impunctata_606 | Scymnus_suturalis_840 | 0.203 |
| Cynegetis_impunctata_607 | Scymnus_suturalis_840 | 0.203 |
| Cynegetis_impunctata_613 | Scymnus_suturalis_840 | 0.203 |
| Coccinella_hieroglyphica_225 | Vibidia_duodecimguttata_603 | 0.203 |
| Chilocorus_renipustulatus_33 | Scymnus_suturalis_841 | 0.203 |
| Cynegetis_impunctata_605 | Scymnus_suturalis_841 | 0.203 |
| Cynegetis_impunctata_606 | Scymnus_suturalis_841 | 0.203 |
| Cynegetis_impunctata_607 | Scymnus_suturalis_841 | 0.203 |
| Cynegetis_impunctata_613 | Scymnus_suturalis_841 | 0.203 |
| Chilocorus_renipustulatus_33 | Scymnus_suturalis_842 | 0.203 |
| Cynegetis_impunctata_605 | Scymnus_suturalis_842 | 0.203 |
| Cynegetis_impunctata_606 | Scymnus_suturalis_842 | 0.203 |
| Cynegetis_impunctata_607 | Scymnus_suturalis_842 | 0.203 |
| Cynegetis_impunctata_613 | Scymnus_suturalis_842 | 0.203 |
| Cynegetis_impunctata_614 | Scymnus_suturalis_843 | 0.203 |
| Chilocorus_renipustulatus_33 | Scymnus_suturalis_844 | 0.203 |
| Cynegetis_impunctata_605 | Scymnus_suturalis_844 | 0.203 |
| Cynegetis_impunctata_606 | Scymnus_suturalis_844 | 0.203 |
| Cynegetis_impunctata_607 | Scymnus_suturalis_844 | 0.203 |
| Cynegetis_impunctata_613 | Scymnus_suturalis_844 | 0.203 |
| Cynegetis_impunctata_614 | Scymnus_suturalis_845 | 0.203 |
| Chilocorus_renipustulatus_33 | Scymnus_suturalis_846 | 0.203 |
| Cynegetis_impunctata_605 | Scymnus_suturalis_846 | 0.203 |
| Cynegetis_impunctata_606 | Scymnus_suturalis_846 | 0.203 |
| Cynegetis_impunctata_607 | Scymnus_suturalis_846 | 0.203 |
| Cynegetis_impunctata_613 | Scymnus_suturalis_846 | 0.203 |
| Chilocorus_renipustulatus_33 | Scymnus_suturalis_847 | 0.203 |
| Cynegetis_impunctata_605 | Scymnus_suturalis_847 | 0.203 |
| Cynegetis_impunctata_606 | Scymnus_suturalis_847 | 0.203 |
| Cynegetis_impunctata_607 | Scymnus_suturalis_847 | 0.203 |
| Cynegetis_impunctata_613 | Scymnus_suturalis_847 | 0.203 |
| Chilocorus_renipustulatus_33 | Scymnus_suturalis_848 | 0.203 |
| Cynegetis_impunctata_605 | Scymnus_suturalis_848 | 0.203 |
| Cynegetis_impunctata_606 | Scymnus_suturalis_848 | 0.203 |
| Cynegetis_impunctata_607 | Scymnus_suturalis_848 | 0.203 |
| Cynegetis_impunctata_613 | Scymnus_suturalis_848 | 0.203 |
| Brachiacantha_rotunda_634 | Stethorus_punctillum_859 | 0.203 |
| Scymnus_loewii_774 | Subcoccinella_vigintiquatuorpunctata_622 | 0.203 |
| Scymnus_loewii_772 | Subcoccinella_vigintiquatuorpunctata_623 | 0.203 |
| Scymnus_loewii_773 | Subcoccinella_vigintiquatuorpunctata_623 | 0.203 |

| | | |
|---|---|---|
| Scymnus_loewii_774 | Subcoccinella_vigintiquatuorpunctata_626 | 0.203 |
| Rhyzobius_litura_97 | Tytthaspis_sedecimpunctata_583 | 0.203 |
| Rhyzobius_litura_96 | Tytthaspis_sedecimpunctata_589 | 0.203 |
| Rhyzobius_litura_97 | Tytthaspis_sedecimpunctata_593 | 0.203 |
| Chilocorus_renipustulatus_33 | Scymnus_suturalis_832 | 0.203 |
| Cynegetis_impunctata_605 | Scymnus_suturalis_832 | 0.203 |
[truncated: 507,128 more chars]
